# Phaeochromocytomas and paragangliomas harbour tumour-initiating SOX2+ stem cells

**DOI:** 10.1530/ERC-25-0242

**Published:** 2026-05-12

**Authors:** Yasmine Kemkem, Mark Quinn, Bence Kövér, Alice Santambrogio, James Kaufman-Cook, Olivia Sherwin, Laura D Scriba, Dimitria Brempou, Miriam Vazquez Segoviano, Dylan Cameron, Ilona Berger, Wen Ng, Daisuke Nonaka, Marily Theodoropoulou, Christina Pamporaki, Paul V Carroll, Louise Izatt, J Paul Chapple, Stefan R Bornstein, Nicole Bechmann, Charlotte Steenblock, Rebecca J Oakey, Cynthia L Andoniadou

**Affiliations:** ^1^ Centre for Craniofacial and Regenerative Biology, King’s College London, London, United Kingdom; ^2^ Department of Medical and Molecular Genetics, King’s College London, London, United Kingdom; ^3^ Department of Internal Medicine III, University Hospital Carl Gustav Carus, Technische Universität Dresden, Dresden, Germany; ^4^ Guy’s & St. Thomas’ NHS Foundation Trust, London, United Kingdom; ^5^ School of Cancer & Pharmaceutical Sciences, King’s College London, London, United Kingdom; ^6^ Medizinische Klinik Und Poliklinik IV, LMU Klinikum, LMU München, Munich, Germany; ^7^ Department of Clinical Genetics, Guy’s and St Thomas’ NHS Foundation Trust, London, United Kingdom; ^8^ William Harvey Research Institute, Faculty of Medicine and Dentistry, Queen Mary University of London, London, United Kingdom; ^9^ Institute for Clinical Chemistry and Laboratory Medicine, University Hospital Carl Gustav Carus, Medical Faculty Carl Gustav Carus, Technische Universität Dresden, Dresden, Germany

**Keywords:** PPGL, PCC, PGL, stem cells, SOX2, sustentacular cells, tumour-initiating cells, cancer stem cells, phaeochromocytoma, paraganglioma

## Abstract

Phaeochromocytomas (PCCs) and paragangliomas (PGLs) are rare neuroendocrine tumours that arise in the neural crest (NC)-derived adrenal medulla and the paraganglia, respectively. Approximately 10–15% of patients with PCCs and 35–40% with PGLs go on to develop metastatic disease, leading to a reported median overall survival of 7 years. The development of prognostic markers and subsequent personal therapeutic strategies are hindered by a lack of understanding of tumourigenesis. In other organs, cells with stem-like properties are at the root of tumour initiation and maintenance, due to their ability to self-renew and give rise to differentiated cells. We have recently shown that, in the human adrenal, a subset of sustentacular cells, endowed with a support role, are in fact SOX2+ postnatal adrenomedullary stem cells that are specified along the NC migratory route. In this study, we intended to determine if SOX2+ cells in PCCs and PGLs can behave as tumour-initiating stem cells. Using expression and transcriptomic studies, we demonstrate the presence of SOX2/*SOX2*-expressing cells across a broad range of PCCs and PGLs, irrespective of tumour aggressiveness, location, and causative mutation. *In silico* analyses reveal the co-expression of SOX2 and chromaffin cell markers in the tumour and reveal the active proliferation of these double-positive cells. Isolation of these cells *in vitro* in stem cell-promoting media, and their xenotransplantation on chicken chorioallantoic membranes, demonstrates that they have the potential to expand and metastasise *in ovo*, supporting their potential as tumour-initiating cells.

## Introduction

Phaeochromocytomas (PCCs) and paragangliomas (PGLs), collectively known as PPGLs, are adrenal and extra-adrenal neuroendocrine tumours, respectively, composed of adrenaline- and noradrenaline-secreting chromaffin cells. They arise from neural crest-derived cells of the sympathetic and parasympathetic nervous system, and according to WHO 2022 5th edition, all PPGLs have metastatic potential (1). With scarce therapeutic approaches available, the median survival rate of patients with metastatic PPGLs is ∼7 years (2). Even PPGLs classified as benign are associated with high morbidity and mortality due to comorbidities related to excessive catecholamine production and secretion, which include hypertension, arrhythmia and stroke (3, 4).

PPGLs are associated with the highest degree of heritability in human neoplasms, with approximately 40% carrying germline mutations or deletions in at least one of several susceptibility genes that include *VHL*, *SDHA*, *SDHB*, *SDHC*, *SDHD*, *SDHAF2, RET*, *NF1*, *TMEM127*, *MAX*, *H3F3A* and *HIF2A* (5). The most frequent cause of inherited PPGLs is germline pathogenic variants in genes encoding for subunits of the mitochondrial succinate dehydrogenase (SDH) enzyme complex: *SDHA, SDHB*, *SDHC* and *SDHD* (6). Patients with *SDHB*-mutant PPGLs present with higher morbidity and mortality (7, 8, 9). Pathogenic germline *SDHB* variants have been detected in 42% of metastatic PPGLs (reaching 80% in paediatric metastatic PPGLs), but in a multivariate analysis on adult patients, they did not act as a prognostic factor of overall survival (2). In *SDHB*-mutant PPGLs, secondary driver events in *TERT* or *ATRX* have been associated with metastatic disease (10). Combined germline and somatic driver genetic events are known in over 70% of cases, enabling the classification of PPGLs into three clusters: cluster 1 (pseudohypoxia), cluster 2 (kinase signalling) and cluster 3 (WNT signalling) (11).

Despite our extensive knowledge of PPGL genetics, the exact cell-of-origin of PPGLs is currently unknown, and this together with their heterogeneity severely limits the generation of *in vivo* experimental models and the road to targeted therapies. The possibility of a cancer stem cell (CSC) or tumour-initiating cell (TIC) has been proposed but not demonstrated for these tumours (reviewed in (12)). A previous study reported the expression of SOX2, a transcription factor associated with stem cells of multiple tissues, in 12% of PPGLs analysed by immunostaining on tumour microarrays (13). The study did not provide data to support stem cell function in these tumours. We have recently identified a postnatal population of SOX2+ stem cells in the normal mouse adrenal medulla, which derives from the migratory neural crest population during embryonic development and which colonises the paraganglia and the adrenal medulla (14). That work demonstrated that SOX2+ adrenomedullary stem cells contribute to the generation of new chromaffin cells throughout life in mice and are specified from neural crest-derived progenitor cells as a distinct population. Confirmation of their presence in normal human adrenals, coupled with the location of PPGLs along sites populated by the neural crest, raises the possibility that neural crest-derived SOX2+ cells might act as stem cells in PPGLs. In this study, we set out to determine if SOX2+ cells of PPGLs can behave as CSCs and candidate cells-of-origin of these tumours.

## Materials and methods

### Ethical approval

All animal studies were performed under compliance with the Animals (Scientific Procedures) Act 1986, Home Office License P8D5E2773 (chicken), and KCL Biological Safety approval for project ‘Function and Regulation of Adrenal Stem Cells in Mammals’. Fetal adrenals were received from the Human Development Biological Resource (HDBR)/project ID 200587. PPGL tumour studies were conducted under King’s College London and were approved by the Cornwall and Plymouth Health Research Authority, study title: Investigation of genetic and epigenetic marks in cancer, IRAS project ID: 216133, REC reference: 17/SW/0018. Formalin-fixed, paraffin-embedded (FFPE) tumour and adrenal tissue samples with pathologically confirmed PPGLs were prepared by the Tumour and Normal Tissue Bank (TNTB) of the BioBank Dresden, originated from the archive of the Institute of Pathology (EK 59032007) of the University Hospital Dresden. Patients were included from the Registry and Repository of biological samples of the European Network for the Study of Adrenal Tumours (ENS@T, EK 407122010), the Prospective Monoamine-producing Tumour study (PMT study; EK 189062010) and/or PROSPHEO (NCT03344016; EK 210052017), all of which had ethical approval at the University Hospital Carl Gustav Carus Dresden. Informed consent was obtained from all patients. Additional FFPE tumour samples were obtained from Queen Mary under the following study title: Genetics of Endocrine Tumours (Cambridgeshire Research Ethics Committee Reference MREC 06/Q0104/133. Normal adrenal human tissue samples were obtained from the University Hospital Würzburg (Germany). Normal adrenal glands were collected as part of tumour nephrectomy and proven to be histologically normal. Informed consent was obtained for all samples; patients were not compensated for participation.

### Chorioallantoic membrane (CAM) assays

Fertilised Shaver Brown eggs were purchased from Medeggs Ltd developmental. Day 0 corresponds to the day eggs were placed in an egg incubator set at 37.8°C/60% humidity. Four days later, eggs were windowed using curved spring scissors, exposing the CAM. Windows were sealed and eggs were placed in the incubator until day 10, when isolated stem cells were seeded onto the CAM. 25 μL of a single-cell suspension (100,000 cells) were pipetted onto the CAM, within a 6 mm diameter silicone ring. Eggs were sealed and placed back in the incubator until collection at 14 days post-fertilisation. Chicks were killed by schedule 1 method. Resected CAM and dissected chick’s lungs were washed in PBS and fixed accordingly.

### Primary culture of adrenomedullary and PPGL-derived stem cells

PPGL tumours and adrenal glands were dissociated using an enzymatic digestion solution of 50 μg/mL DNAse I (CAT#D5025, Merck, UK), 10 mg/mL Collagenase II (CAT#LS004177, Worthington, USA), 2.5 μg/mL Fungizone (CAT#15290026, Gibco, UK) and 0.1X trypsin–EDTA (CAT#59418C, Merck, UK) in 1X Hank’s Balanced Salt Solution (HBSS) (CAT#14025050, Gibco, UK). Samples were sequentially incubated at 37°C and manually triturated, until reaching a single-cell suspension. Dissociation enzymes were deactivated with serum-rich base medium (DMEM/F-12 (CAT#31330-038, Gibco, UK) + 5% FBS (CAT#F0804, Merck, UK) + 50 μL/mL penicillin–streptomycin (CAT#15070063, Gibco, UK). The cell suspension was centrifuged, and the pellet was resuspended and plated in stem cell-promoting media: base media + 20 ng/mL bFGF (CAT#234-FSE, R&D Systems, UK) + 50 μg/mL cholera toxin (CAT#C8052, Merck, UK). For immunostaining, cells were plated on glass coverslips coated with 0.1% gelatine diluted in PBS. For CAM assays, stem cell colonies were expanded in stem cell-promoting media. On transplantation day, cells were trypsinised, labelled with a green fluorescent chloromethyl derivative of fluorescein diacetate (CMFDA) intracellularly activated CellTracker dye (CAT#C7025, Thermo Fisher Scientific, USA) as per the manufacturer’s instructions. Labelled cells were resuspended in stem cell-promoting media as a single-cell suspension for seeding onto CAM.

### Tissue processing for immunofluorescence and immunohistochemistry

For paraffin-embedding, resected grafts/CAMs were fixed in 10% neutral buffered formalin (NBF) (CAT#HT501128, Merck, UK) overnight at room temperature, on a roller. The following day, samples were washed and gradually dehydrated using ethanol series. Paraffin-embedded samples were sectioned at 5 μm thickness. Chicks’ lungs were cryo-embedded. For this, they were fixed in 4% PFA at 4°C overnight. Samples were washed and placed in a cryoprotective 30% sucrose/PBS solution overnight at 4°C. Samples were embedded in optical cutting temperature compound (CAT#361603E, VWR, USA), flash-frozen and cryo-sectioned at 15 μm thickness.

### Immunofluorescence and immunohistochemistry

Paraffin sections were de-paraffinised with Neoclear and were gradually rehydrated in decreasing concentrations of ethanol. Antigen retrieval was performed in a decloaking chamber NXGEN (CAT#DC2012-220V, Menarini Diagnostics, Italy) at 110°C for 3 min using Dako Target Retrieval Solution, pH 9.0 (CAT#S236784-2, Agilent Technologies, USA), as per the supplier’s instructions.

Immunohistochemistry was performed using the ImmPRESS Excel Amplified HRP Polymer Staining Kit Anti-Rabbit IgG (CAT#MP-7601-50, Vector Laboratories, USA) as per the supplier’s instructions, using a rabbit anti-SOX2 antibody (CAT#ab92494, Abcam, UK, 1:500). Nuclei were counterstained with Vector Haematoxylin QS (CAT#H-3404-100, Vector Laboratories, USA), and slides were mounted in VectaMount Permanent Mounting Medium (CAT#H-5000-60, Vector Laboratories, USA).

Immunofluorescence staining was performed as follows: sections were blocked at room temperature for 1 h in a 10% donkey serum/blocking buffer (0.15% glycine, 2 mg/mL BSA, 0.1% Triton X-100 in PBS). Sections were then incubated overnight at 4°C with primary antibody diluted in 1% donkey serum/blocking buffer (mouse anti-HNA (CAT#MAB4383, Merck, UK, 1:300)). Following three five-minute washes in PBS + 0.1% Triton X-100 (PBST), sections were incubated in secondary fluorophore-conjugated antibodies (Goat anti-mouse594 (CAT#ab150116, Abcam, UK, 1:500) and DAPI (CAT# ab228549, Abcam, UK, 1:5,000) in 1% donkey serum/blocking buffer.

Cryosections were air-dried and washed in PBS. Fixed cells were washed in PBS. In both cases, samples were blocked at room temperature for 1 h in blocking buffer (1% BSA, 0.1% Triton X-100, 5% goat serum), followed by a 1 h incubation in primary antibodies diluted in 1% goat serum/blocking buffer (mouse anti-tyrosine hydroxylase (CAT# 612300, BD Biosciences, USA, 1:500 – goat anti-SOX2 (CAT# AF2018, R&D Systems, USA, 1:500)) and a 1 h incubation in fluorophore-conjugated secondary antibodies (Biotinylated Goat anti-mouse (CAT# ab6788, Abcam, UK, 1:500), donkey anti-goat647 (CAT# ab150131, Abcam, UK, 1:500). For tyrosine hydroxylase staining, a biotin–streptavidin amplification was performed. Following secondary biotinylated antibody incubation, sections were washed in PBST and incubated at room temperature for 1 h with fluorescent-labelled streptavidin (CAT#S32355, Life Technologies, USA, 1:500). DAPI (CAT# ab228549, Abcam, UK, 1:5,000) was added in the last incubation. Samples were washed and mounted in Vectashield Antifade Mounting Medium (CAT#H- 1000-10, Vector Laboratories, USA).

### RNAscope mRNA *in situ* hybridisation

RNAscope was carried out on paraffin-embedded tissue sections with the RNAscope™ 2.5 HD Detection Reagents-RED kit (CAT#322360, ACD Bio, USA) and the Hs-*MKI67* detection probe (CAT#591771, ACD Bio, USA) following the manufacturer’s instructions. Sections were counterstained with Hematoxylin QS (CAT#H-3404-100, Vector Laboratories, USA) and mounted using VectaMount Permanent Mounting Medium (CAT#H-5000-60, Vector Laboratories, USA).

### Imaging

Stained sections following immunohistochemistry or RNAscope mRNA *in situ* hybridisation were scanned using a Nanozoomer-XR Digital Slide Scanner (Hamamatsu). High-magnification images were acquired with an Olympus BX34F Brightfield microscope. Cell culture images were obtained with an Olympus Phase Contrast microscope. Immunofluorescence imaging was performed using a Zeiss LSM980 confocal microscope (Zeiss Plan – Apochromat 20×/0.8 dry objective). Z-stacks were acquired with a 0.75 μm step. Imaging files were processed with Fiji and Nanozoomer Digital Pathology view. Figures were created in Adobe Photoshop, version 26.6.0.

### Computational studies

#### scRNA-seq and snRNA-seq dataset acquisition

PPGL tumour samples were obtained directly from the Guy’s and St Thomas’ NHS Foundation Trust (GSTFT) operating theatres. Patients were enrolled to this study through the departments of endocrinology and clinical genetics in GSTFT. All patients enrolled provided written informed consent, which granted access to tumour tissues and clinical data. Following resection, tumour samples were immediately reviewed and dissected by an expert clinical pathologist. Confirmed PPGL tumour samples were then transferred to our laboratory where they were either snap-frozen and stored at −80°C or underwent dissociation in a single-cell suspension in expectation of immediate scRNA-sequencing. Single-cell dissociation was performed using an enzymatic digestion of 10 μL/mL DNAse I (CAT#D5025, Merck, UK), 20 μL/mL FBS (CAT #10270106, Thermo Fisher Scientific, USA), 10 mg/mL Collagenase II (CAT#LS004177, Worthington, USA), 1 mg/mL Dispase-II (CAT#SCM133, Merck, UK) and 40 U/mL RNaseOUT (CAT#10777019, Thermo Fisher Scientific, USA) in 1 mL of 1X Hank’s Balanced Salt Solution (HBSS) (CAT#14025050, Gibco, UK). Samples were sequentially incubated at 37°C and intermittently mechanically dissociated and vortexed throughout. Samples were then passed through a 40 μm filter (CAT#UY-06336-63, Cole-Parmer, USA) and centrifuged. The enzyme dissociation mix was removed, and the pellet was washed with and eventually resuspended in HBSS and 5% FBS (CAT#10270106, Thermo Fisher Scientific, USA). The single-cell suspension was then inspected under the microscope using Trypan Blue Solution, 0.4% (CAT#15250061, Thermo Fisher Scientific, USA), for cellular number and viability. Frozen samples underwent nuclei extraction as per 10X protocols. Samples were kept at 4°C throughout. 3–50 mg of frozen PPGL tumour tissue were transferred to a pre-chilled sample dissociation tube (CAT#2000564, 10X Genomics, USA) with 200 μL of lysis buffer (Lysis Reagent (CAT#200558, 10X Genomics, USA), Reducing Agent B (CAT#200087, 10X Genomics, USA) and Surfactant A (CAT#2000559, 10X Genomics, USA). Samples were dissociated mechanically, and 300 μL of lysis buffer were added. The samples were incubated on ice for 8 min. Dissociated tissues were then centrifuged through a pre-chilled Nuclei Isolation Column (CAT#2000562, 10X Genomics, USA). Flowthrough was vortexed and centrifuged, and the pellet was resuspended with 500 μL of Debris Removal Buffer (CAT#200560, 10X Genomics, USA). Samples were centrifuged and supernatant was removed before the pellet was resuspended in 1 mL of Wash and Resuspension Buffer (1X PBS, 10% BSA and RNase inhibitor (CAT#2000565, 10X Genomics, USA). This was repeated depending on debris, with the final pellet resuspended in 50–500 μL Wash and Resuspension Buffer. The suspension was then inspected for nuclei number, viability, debris and clumping using Trypan Blue Solution, 0.4%. Single cells/nuclei were processed on the Chromium iX instrument using 3′ v3.1 gene expression profiling reagents (10X Genomics, USA) to generate GEMs (gel bead-in-emulsion) for cell barcoding. Cells were loaded on the chip with an aim to capture 8,000–10,000 cells per sample. Full-length cDNA was generated from poly-adenylated mRNA and amplified. Dual indexed sequencing libraries were prepared from the amplified cDNA, and final libraries were evaluated on the Agilent TapeStation 4200 using a High Sensitivity D5000 ScreenTape (Agilent Technologies, USA). The libraries were then pooled and sequenced on NextSeq 2000 (Illumina, USA) at a depth of approximately 20,000 read pairs per cell.

#### Pre-processing for samples collected in this study

Samples were aligned to the GRCh38 reference genome using CellRanger 9.0.1 (10X Genomics, USA) with default settings, meaning that intronic reads were also included for both single-cell and single-nucleus samples.

#### Additional publicly available samples

*SDHB* PPGL single-nucleus RNA-seq samples were accessed from the corresponding Figshare of Flynn *et al.* (10). The Figshare folder contained the CellRanger output matrices, from which the raw_feature_barcode matrix was used as input. Patient metadata for the corresponding samples was also accessed from the supplementary material of Flynn *et al.* Additional datasets for healthy adrenal medulla samples were accessed from the Figshare of Flynn *et al.* as well, although these are originally from Zethoven *et al.* (15). Dataset identifiers for samples accessed from other publications were kept as original throughout.

#### Quality control (QC) and further processing

To separate true cells from cell-free droplets, we first filtered out barcodes with less than 800 UMIs. Percentages of ribosomal and mitochondrial counts were determined using the Scanpy (16) calculate_qc_metrics function with ‘RPS’, ‘RPL’, or ‘MT-’ flags.

QC was then performed within Scanpy using median +/− X * median absolute deviation filters, with x ranging from 4 to 5 for different metrics. Specifically, these included percentage of mitochondrial counts (max = 25%, *X* = 5), percentage of ribosomal counts (max = 30%, *X* = 5), percentage of counts in top 20 genes (*X* = 5), log1p total counts (*X* = 4) and log1p genes detected (*X* = 4). Following this, doublets were identified and removed using the scrublet algorithm implemented in Scanpy with default settings (16).

#### Dataset integration

To jointly analyse single cells/nuclei from all transcriptomic datasets, we used the scVI variational auto-encoder framework (17). Specifically, we set each patient ID (one per dataset) as the batch_key and set the percentage of mitochondrial (pct_mito) and ribosomal (pct_ribo) counts as continuous covariates. The model was set up using two hidden layers and a latent space with 30 dimensions. The generative end of the model was specified to be negative binomial. The 30-dimensional latent space was then used for nearest-neighbour identification, Leiden clustering (resolution = 0.4, flavour = ‘igraph’) and UMAP visualisations, all with Scanpy implementations (16).

#### Cell type annotation

Leiden clusters of cells were annotated using established markers from previous work on PPGLs (10, 15) as follows: Chromaffin_cells: HAND2, SLC18A1, TH, PHOX2A; Cortex_cells: CYP11B1, STAR; Sustentacular_cells: SOX10, CDH19, S100B, SOX2; T_cells: CD4, TRBC1, TRBC2; Macrophages: CSF1R, CD163, C1QA; Mast_cells: TPSAB1, TPSB2; B_cells: JCHAIN, CD79A, CD79B; Endothelial_cells: ROBO4, FLT1; and Fibroblasts: COL1A1, COL1A2, PDGFRB. Following initial overclustering, clusters with the same broad identities were merged.

#### Pseudobulk differential expression analysis

To perform differential expression analysis, pseudobulk samples were generated from each cell type in each sample (where the cell type exceeded 30 cells) using the decoupleR package (18). In the DE analysis, only cell types with at least 3 pseudobulk samples were kept. We then used the Limma-voom workflow (19) to find cell type-specific markers, with the model: ‘∼ cell_type’, and contrasts between all cell type pairs. Markers were assigned if they were significant in all comparisons. We applied a similar workflow to find differentially expressed genes in metastasis and *SOX2* status, with the models ‘∼ 0 + cell_type + cell_type: Metastasis’ and ‘∼ 0 + cell_type + cell_type: Chrom_SOX2_status’.

#### Ligand–receptor interaction predictions

Ligand–receptor interaction prediction was performed using LIANA+ (20). This approach was chosen as it allows the use of an ensemble of ligand–receptor algorithms, as well as an integrated ligand–receptor interaction database. Here, five algorithms (CellPhoneDB with 1000 permutations, Connectome, log2FC, NATMI and SingleCellSignalR) were used as implemented in the aggregate_rank function using a merged version of databases (called ‘consensus’ in LIANA+).

#### LIANA+ signalling dot plots

Top ligand–receptor interactions were visualised using the native plotting functions within the LIANA+ package, using the commands li.pl.dotplot with the following arguments: colour = ‘magnitude_rank’, size = ‘specificity_rank’, inverse_size = true, inverse_colour = true, top_*n* = 20, orderby_ascending = true. We have plotted interactions with chromaffin cells as a source, ranking by specificity. These were controlled through the ‘orderby’, ‘target_labels’ and ‘source_labels’ arguments.

#### Heatmap plotting

Heatmaps were generated following pseudobulking of datasets with decoupleR and counts per million and log1p normalising the resulting anndata objects. Heatmaps were then generated with the Scanpy sc.pl.heatmap function with row-wise (e.g. per gene) min–max normalised values, with the arguments log = false, standard_scale = ‘var’.

#### UMAP plotting

UMAPs were plotted using the Scanpy function sc.pl.umap with the sort_order = true argument for feature plots to prevent the few expressing cells from being buried under the large number of non-expressing cells.

#### Annotation of top TFs and signalling genes

A list of all human TFs was accessed from ‘https://humantfs.ccbr.utoronto.ca/download/v_1.01/TF_names_v_1.01.txt’, and the LIANA ‘consensus’ database was used to retrieve gene names relevant to cell–cell signalling (either ligands or receptors in the database).

#### Code and data accessibility

Our code and processed data are available on https://github.com/Andoniadou-Lab/SOX2_PPGL. We provide a Jupyter Notebook of all Python code used for processing the datasets from CellRanger outputs. In addition, we provide an R script that was used for differential gene expression analysis with Limma-voom. Finally, we provide the cell type annotated AnnData object as a resource, allowing others to reproduce our work. Cell Ranger output files (raw and filtered count matrices) have been deposited on Figshare with the following DOI: 10.6084/m9.figshare.29447750.

### Generation of a doxycycline-inducible SOX2-overexpressing stable PC12 cell line

#### Cloning

The (PB)TetO-SOX2_2xNLS-eGFP vector was generated using the PB-TO-hNGN2 plasmid (CAT#172115, Addgene, USA) as the backbone. The hNGN2 cassette was replaced with mouse *Sox2* (#20326, Addgene, USA) and the mTagBFP2 with the 2xNLS-eGFP reporter (kindly donated by F. Riccio).

*Sox2* and 2xNLS-eGFP were amplified using Q5® High-Fidelity 2X Master Mix (New England Biolabs, M0492S, USA) as per manufacturer’s instructions using the following primers:

*Sox2* forward: CGT​ACC​ACT​TCC​TAC​CCT​CGT​AAA​GGA​TCC​GCC​GCC​ACC​ATG​TAT​AAC​ATG​ATG​GAG​ACG​GAG.

*Sox2* reverse: GGG​TGG​GCG​ATC​GAT​TCG​CGA​GTT​TAA​ACT​CAC​ATG​TGC​GAC​AGG​GG.

*2xNLS-eGFP* forward: GTC​AAA​CTC​GAC​GGC​GGT​TCT​GGA​GGT​CCT​GCT​GCA​AAG​CGC​GTT.

*2xNLS-eGFP* reverse: AAC​TAG​AAG​GCA​CAG​AAT​TCT​TAG​CGG​CCG​CTT​ACT​TGT​ACA​GCT​CGT​CCA​TGC.

The PB-TO-hNGN2 backbone was digested with *BamHI*, *PmeI*, *NotI* and *PpuMI* (New England Biolabs, USA) to enable insertion of the Sox2 and 2xNLS-eGFP fragments. PCR products and the digested backbone were resolved by agarose gel electrophoresis, and the appropriate bands were excised and purified using the Monarch DNA Gel Extraction Kit (New England Biolabs, T1120S). The four fragments were assembled using HiFi DNA Assembly Master Mix (New England Biolabs, E2621S) according to the manufacturer’s instructions. The assembled plasmid was transformed into NEB® 10-beta Competent *E. coli* (High Efficiency; New England Biolabs, C3019H). Positive clones were screened, and the final construct was verified by Sanger sequencing.

#### Transfection and cell line generation

To generate a stable PC12 cell line in which *Sox2* is overexpressed, PC12 cells (CAT# CRL-1721, ATCC, USA) were co-transfected with the (PB)TetO-SOX2_2xNLS-eGFP and a PiggyBac transposase plasmid (Addgene, USA) using Lipofectamine 3000 (CAT# L3000001, Thermo Fisher Scientific, USA), following the manufacturer’s protocol. Forty-eight hours post-transfection, cells were selected with puromycin (3 μg/mL) for 3 weeks. Stable integration was confirmed by induction with doxycycline (2 μg/mL) 48 h prior to downstream assays.

### Flow cytometry cell cycle analysis

For cell cycle analysis, cells were dissociated into single cells by trituration in PBS, pelleted and fixed in 4% PFA on ice for 15 min. Following 2 PBS washes, pellets were resuspended in 1 mg/mL of DAPI diluted in PBS (CAT# D9542, Merck, UK), for 30 min. Cells were resuspended in 5 mM EDTA, 5% FBS in 1x PBS prior to cell cycle analysis. Data were collected on BD LSRFortessa™ Cell Analyzer and analysed using FlowJo. Gating was based on the corresponding untreated cell controls.

### RNA extraction and qPCR

Total RNA was extracted from harvested cells using the Monarch® Total RNA Miniprep kit (CAT# T2010S, New England Biolabs, USA) as per the manufacturer’s protocol, including DNase I treatment. RNA concentrations and quality were determined using the NanoDrop™ One instrument (Thermo Fisher Scientific, USA). RNA was converted to cDNA using the QuantiTect Reverse Transcription kit (CAT#205314, Qiagen, Germany) following manufacturer’s recommendations. Then, RT-qPCR was performed using the iTaq Universal SYBR Green Supermix (CAT#1725121, Bio-Rad, USA) on the CFX Connect platform (Bio-Rad, USA) in technical triplicates.

Mouse *Sox2* primers: forward: AAC​GGC​AGC​TAC​AGC​ATG​ATG​C; reverse: CGA​GCT​GGT​CAT​GGA​GTT​GTA​C.

## Results

### SOX2+ cells are found in both phaeochromocytomas and paragangliomas, irrespective of mutation and tumour site

**Figure 1:**
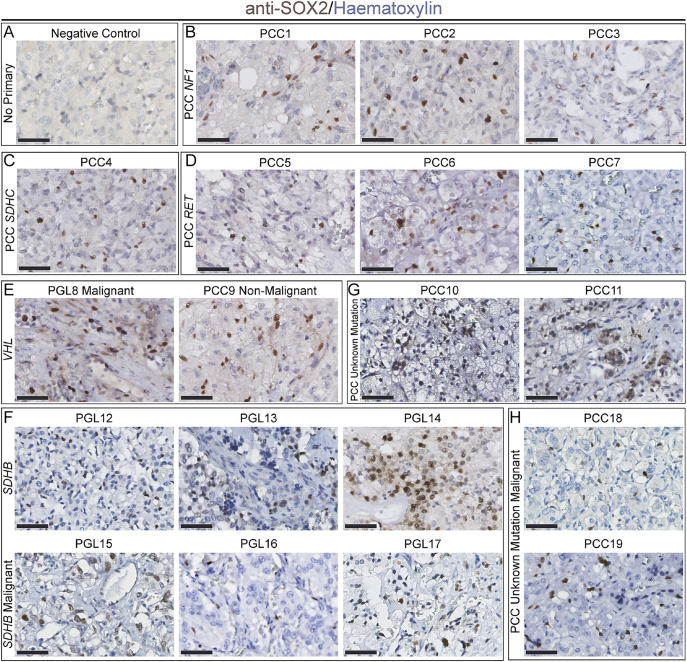
*Immunohistochemistry using antibodies against SOX2 on FFPE sections of phaeochromocytomas and paragangliomas (PPGLs).* In all panels, cells immunopositive for SOX2 are in brown, cells counterstained with haematoxylin. (A) Negative control staining without primary antibody. (B) Non-metastatic phaeochromocytomas PCC1 to PCC3 with mutations in NF1. (C) Non-metastatic phaeochromocytoma PCC4 with mutation in *SDHC*. (D) Non-metastatic phaeochromocytomas PCC5 to PCC7 with mutations in *RET*. (E) Malignant paraganglioma PGL8 and non-malignant phaeochromocytoma PCC9 with mutations in *VHL*. (F) Non-malignant paragangliomas PGL12 to PGL14 and malignant paragangliomas PGL15 to PGL17 with mutations in *SDHB*. (G) Non-malignant phaeochromocytomas PCC10 and PCC11 with no known mutations. (H) Malignant phaeochromocytomas PCC18 and PCC19 with no known mutations. Scale bars: 50 μm.

To determine the presence of putative stem cells in PPGL tumours, we focused on SOX2, which we previously established as a marker of progenitor/stem cells of the adrenomedullary lineage (14). Extending the previous report of stem cell markers in PPGLs (13), we tested for SOX2 expression on FFPE sections from a cohort of 19 PPGLs across different tumour sites, mutations and malignancy status. This cohort was composed of 12 adrenal PCCs and seven extra-adrenal PGLs, of which two were located in the carotid body, three abdominal, and two thoracic. Germline mutations in known genes were present in 15 patients, spanning *NF1*, *SDHC*, *RET* and *SDHB*, whilst three patients had somatic mutations in *RET* and *VHL*. Five of the tumours were metastatic (i.e. primary tumours that subsequently gave rise to metastases), three of which harboured mutations in *SDHB*. Tumour data are given in Table 1. Immunostaining using antibodies against SOX2 identified positive cells in all tumours tested (Fig. 1B, C, D, E, F, G, H, negative control in A). Heterogeneity was seen across different tumour locations. SOX2-positive cells ranged from 0.93 to 8.31% with an average of 4.54% SOX2+ of all nuclei (Supplementary Table 1 (see section on Supplementary materials given at the end of the article)). These data confirm that SOX2+ cells can be present across PPGLs irrespective of tumour type, metastatic status and mutation status, as observed in this small tumour cohort.

**Table 1:** SOX2 expression analysis tumour cohort information.

Tumour ID	Tumour type	Tumour status	Metastasis	Variant identified	
				Confirmed germline mutation	Confirmed somatic mutation
PCC1	Phaeochromocytoma	Primary	No	*NF1* – no variant stated	
PCC2	Phaeochromocytoma	Primary	No	*NF1* – no variant stated	
PCC3	Phaeochromocytoma	Primary	No	*NF1* – no variant stated	
PCC4	Phaeochromocytoma	Primary	No	*SDHC* – no variant stated	
PCC5	Phaeochromocytoma	Primary	No	None	*RET* – no variant stated
PCC6	Phaeochromocytoma	Primary	No	*RET* – no variant stated	
PCC7	Phaeochromocytoma	Primary	Unknown	*RET* – no variant stated	
PGL8	Carotid body paraganglioma	Primary	No	None	*VHL* – no variant stated
PCC9	Phaeochromocytoma	Primary	No	None	*VHL* – no variant stated
PCC10	Phaeochromocytoma	Primary	Unknown	None	None
PCC11	Phaeochromocytoma	Primary	Unknown	None	None
PGL12	Mediastinal paraganglioma	Primary	No	*SDHB* – c.136C>T	
PGL13	Retroperitoneal paraganglioma	Primary	Unknown	*SDHB* – deletion exon 1	
PGL14	Carotid body paraganglioma	Primary	Yes	*SDHB* – no variant stated	
PGL15	Abdominal para-aortic paraganglioma	Primary	Yes	*SDHB* – c.268C>T, p.Arg90*	
PGL16	Abdominal para-aortic paraganglioma	Primary	Yes	*SDHB* – c.338G>A, p.Cys113Tyr	
PGL17	Mediastinal paraganglioma	Primary	No	*SDHB* – c.79C>T, p.Arg27*	
PCC18	Phaeochromocytoma	Primary	Yes	None	None
PCC19	Phaeochromocytoma	Primary	Yes	None	None

### Single-cell analysis of PPGLs identifies a second *SOX2*-expressing population of chromaffin cells in tumours

To examine the molecular profile of tumour cell populations, we took advantage of published single-nucleus datasets (10) including nine PGLs (PGL20, PGL21 and PGL23 to PGL29) and one PCC (PCC22). Additionally, we carried out single-cell analysis on three fresh tumour samples (PCC30, PGL31 and PGL32) and single-nucleus analysis on an additional four frozen samples (PGL33 to PGL36). Of the seven new samples, two were metastatic (PGL32 and PGL33) and three had germline mutations in *SDHB* and two in *SDHD* (one metastatic). All tumour data used for transcriptomic analysis are shown in Table 2. For comparisons, we included two published single-nucleus datasets of normal adrenal tissue (Healthy1 and Healthy2) (15).

**Table 2:** Metadata of tumours used in the transcriptomic analyses.

Source	Tumour ID in original publication	Tumour ID in this publication	Tumour type	Single cell or single nucleus	Tumour status	Metastasis	Metastasis site	Variant identified	*SOX2+* cells
Flynn *et al. *(10)	E019-P1	PGL20	Abdominal paraganglioma	SN	Primary	No	NA	*SDHB* c.418G>T	Yes
Flynn *et al. *(10)	E123-M1	PGL21	Abdominal paraganglioma	SN	Metastasis	Yes	Bone	*SDHB* c.380T>G	No
Flynn *et al.* (10)	E140-P1	PCC22	Phaeochromocytoma	SN	Primary	No	NA	*SDHB* c.380T>G	Yes
Flynn *et al.* (10)	E143-M1	PGL23	Abdominal paraganglioma	SN	Metastasis	Yes	Abdominal	*SDHB* c.268C>T	Yes
Flynn *et al.* (10)	E146-P1	PGL24	Abdominal paraganglioma	SN	Primary	No	NA	*SDHB* c.136C>T	Yes
Flynn *et al.*	E156-P1	PGL25	Bladder paraganglioma	SN	Primary	No	NA	*SDHB* c.526G>T	Yes
Flynn *et al.*	E166-M1	PGL26	Abdominal paraganglioma	SN	Metastasis	Yes	Abdominal	*SDHB* c.72+1G>T	Yes
Flynn *et al. *(10)	E171-M1	PGL27	Abdominal paraganglioma	SN	Metastasis	Yes	Chest wall – left rib 5–9	*SDHB* c.736A>T	No
Flynn *et al. *(10)	E197-M1	PGL28	Abdominal paraganglioma	SN	Metastasis	Yes	Thoracic mediastinum lymph node	*SDHB* c.1_72del	Yes
Flynn *et al. *(10)	E225-P1	PGL29	Abdominal paraganglioma	SN	Primary	No	NA	*SDHB* c.190delG	Yes
This publication	NA	PCC30	Phaeochromocytoma	SC	Primary	No	NA	None	Yes
This publication	NA	PGL31	Abdominal paraganglioma	SC	Primary	No	NA	*SDHB* c.268C>T p(Arg90Ter)	Yes
This publication	NA	PGL32	Abdominal paraganglioma	SC	Primary	Yes	Skeletal	None	Yes
This publication	NA	PGL33	Carotid body paraganglioma	SN	Primary	Yes	Pulmonary	*SDHD* c.337_340del p.(Asp113 fs)	Yes
This publication	NA	PGL34	Abdominal paraganglioma	SN	Primary	No	NA	*SDHB* c.268C>T p.(Arg90*)	Yes
This publication	NA	PGL35	Pelvic paraganglioma	SN	Primary	No	NA	*SDHB* exon 1 deletion	Yes
This publication	NA	PGL36	Carotid body paraganglioma	SN	Primary	No	NA	*SDHD* c.242C>T, p.(P81L)	Yes
Zethoven *et al.* (15)	E240	Healthy 1	NA	SN	NA	No	NA	NA	Yes
Zethoven *et al.* (15)	E243	Healthy 2	NA	SN	NA	No	NA	NA	Yes

**Figure 2:**
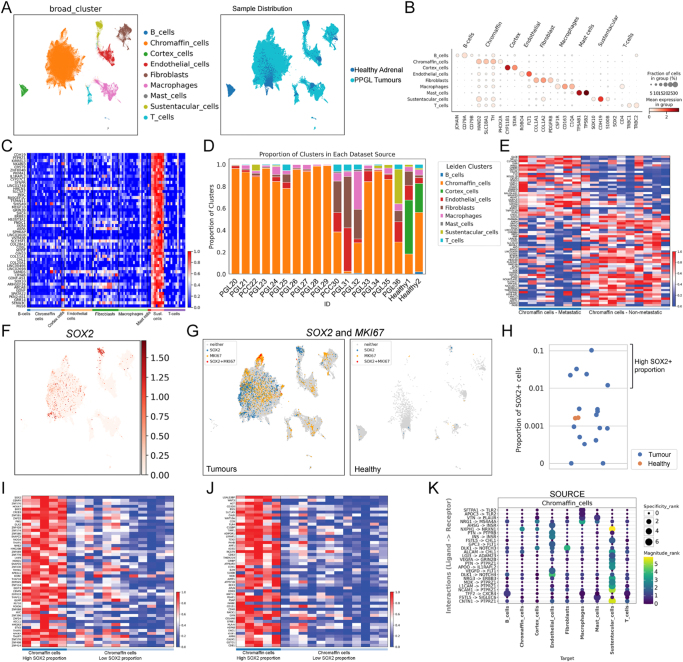
*Single-cell analysis of PPGLs identifies expression of SOX2 in tumour chromaffin cells.* (A) UMAP plot of the integrated dataset with each cell type (left) and patient group (right – healthy vs tumour) coloured separately. (B) Dot plot of key markers associated with the identified cell populations. (C) Heatmap of the top marker genes in sustentacular cells ordered by statistical significance from top to bottom. Colours correspond to gene-wise min–max normalised values. (D) Stacked bar plot of cell type proportions in each sequenced sample. (E) Heatmap of top marker genes in chromaffin tumour cells comparing metastatic (left) to non-metastatic (right) samples. Genes were ranked by statistical significance from top to bottom for upregulated genes (for metastatic) and bottom to top for downregulated genes. Colours correspond to gene-wise min–max normalised values. (F) UMAP plot of normalised *SOX2* expression across all tumour cells, with the dots ordered by their expression values. (G) UMAP showing *SOX2* (blue), *MKI67* (cycling cells, yellow) and double-positive cells (red). Cells were classified as positive if they had at least a single transcript. (H) Strip plot of the proportion of *SOX2*+ cells in each sample. Distances on the Y-axis are on a log10 scale, and for visualisation purposes, datasets with 0% SOX2+ were plotted at *Y* = 0.0001. Jitter has been added to the X-axis to reduce overlapping observations. (I) Heatmap of top transcription factor-encoding genes distinguishing chromaffin tumour cells in tumours with high SOX2+ cell proportion, from chromaffin tumour cells in tumours with low SOX2+ cell proportion, as determined with a 1% cut-off (see H). Genes are ordered by statistical significance from top to bottom. Colours correspond to gene-wise min–max normalised values. (J) Heatmap of genes associated with signalling, distinguishing chromaffin tumour cells in tumours with high SOX2+ cell proportion, from chromaffin tumour cells in tumours with low SOX2+ cell proportion, as determined with a 1% cut-off (see H). Genes are ordered by statistical significance from top to bottom. Colours correspond to gene-wise min–max normalised values. (K) LIANA+ dot plot of the top 20 interactions where chromaffin cells are the source and ordered by specificity rank. Colours correspond to magnitude, whilst dot size increases with specificity rank, as calculated by LIANA+.

Our analysis identified nine clusters of cells, including B-cells, chromaffin cells, cortex, endothelial cells, fibroblasts, macrophages, mast cells, sustentacular cells and T-cells (Fig. 2A). Sample distribution split between the two healthy samples and 17 tumours revealed the cortex cell cluster derives almost entirely from the healthy adrenal controls, with representation from both groups in all other clusters. Key markers of cell populations known from mouse data are plotted in Fig. 2B; for chromaffin cells *HAND2*, *SLC18A1*, *TH* and *PHOX2A* and for the sustentacular population *SOX10*, *CDH19*, *S100B* and *SOX2*. The markers defining each cell population are listed in Supplementary Table 2. Top sustentacular markers included previously undescribed *PTPRZ1*, *KIRREL3*, *NKAIN3* and *CHST9* (Fig. 2C). Proportions of all clusters in each of the datasets reveal variation in the sustentacular cells, with an over-representation of this cluster in PGL36 (31%) (Fig. 2D). Comparison of the chromaffin cell cluster of tumours from patients with metastatic disease (7 tumours) to those from tumours that had not metastasised at the time of analysis (10 tumours) identified factors potentially associated with metastasis. The top candidate metastasis genes were *GLCE* and *NELL1*, both involved in the tumour matrisome (21); *C17orf97* (LIAT1); *GHR* encoding the growth hormone receptor, known to play a role in cancer metastasis and chemoresistance (22, 23); *MND1*, involved in cell cycle regulation and DNA repair and a prognostic marker for several cancers (24, 25, 26); and *CREB5*, proposed to promote invasion and metastasis in colorectal cancer and glioma (27, 28). *EZH2*, a histone methyltransferase that promotes cancer progression (29, 30), and *PIMREG*, promoting aggressiveness through NF-κΒ and prognostic for several tumour types (31, 32, 33), are also in this group (Fig. 2E). *EZH2*, *MND1* and *PIMREG* have been independently identified as associated with metastasis in *SDHx* tumours using bulk RNA-seq (15).

Gene expression analysis of only the tumour samples confirms highest *SOX2* expression in the sustentacular cluster (Fig. 2F). Unexpectedly, there was also widespread *SOX2* expression across the chromaffin cell cluster. Projection of *SOX2* expression (blue) combined with *MKI67* (yellow) marking cycling cells demonstrated that double-positive cells (red) are located in the chromaffin cell cluster (Fig. 2G, left). This confirms a population of cycling tumour cells that express *SOX2* but have chromaffin characteristics, unlike any population in healthy controls (Fig. 2G, right). *SOX2*+ cells were detected in healthy controls and 15 out of 17 tumours (Supplementary Fig. 1). We expect *SOX2* transcript detection to be sparser than protein detected by immunostaining (34), so the absence of *SOX2* expression in two tumour samples in these assays is not confirmation of the lack of SOX2^+^ cells in the tumour. Overall, 77% of *SOX2*-expressing cells in tumours had detectable *TH* transcripts. The proportion of *SOX2*-expressing cells varied across tumours, with a group of five tumours having a substantially higher proportion of *SOX2*+ cells than healthy controls (Fig. 2H). Given the highly variable number of *SOX2*-expressing cells across samples, we decided to compare chromaffin cells with higher proportion of *SOX2*+ cells to those with lower proportions or absence of *SOX2* (cut-off of 1%). Reassuringly, this analysis found the most differentially expressed transcription factor-encoding gene to be *SOX2*. Other highly ranked genes include *HOXA7*, whose protein product can function as an oncogene and contribute to malignancy (35, 36, 37), and the proto-oncogene *BRF2* (38, 39) (Fig. 2I and Supplementary Table 3 for a full list of significant factors). Our previous findings in mice demonstrated that SOX2+ cells have a major role in regulating cell proliferation of neighbouring cells through secretion of WNT6 (14). Comparative analysis of chromaffin cells from tumours with a high SOX2 proportion versus those with lower/absent *SOX2*+ cell proportions revealed differential expression of genes related to cell signalling. These include *LGALS3BP*, encoding a secreted factor involved in cancer progression (40), as well as the WNT protein-encoding genes *WNT3*, *WNT10A*, *WNT11* and *WNT6* (41, 42, 43) (Fig. 2J and Supplementary Table 3). To further investigate the relationships of chromaffin cells with the other tumourigenic populations, we carried out ligand–receptor analysis framework (LIANA+) analysis (20). This determines the intercellular communication inference between chromaffin cells and the other populations. Interestingly, the strongest, in magnitude, predicted signalling from chromaffin cells is to sustentacular cells, primarily through adhesion molecules ASHG, CNTN1, NCAM1, L1CAM, ALCAM, as well as through NRG3, PTN and APOO. Signalling to endothelial cells and fibroblasts is predicted through DLK1 and to macrophages by NRG1 (Fig. 2K). Taken together, these *in silico* data identify a malignant signature for PPGLs and support that tumour chromaffin cells aberrantly expressing SOX2 and have distinct oncogenic gene expression and a secretory signature consistent with cancer promotion.

In several cancers, including glioblastoma, small cell lung cancer and squamous cell carcinoma, *SOX2* amplification has been shown to grant a proliferative advantage to tumour cells (44, 45, 46, 47, 48). To determine whether SOX2 confers a proliferative advantage in PPGL cells, we generated a vector enabling doxycycline-inducible overexpression of mouse *Sox2* in a reversible manner ((PB)TetO-SOX2-2xNLS-eGFP) (Supplementary Fig. 2A and B). This allowed the generation of a stable PC12 rat phaeochromocytoma cell line in which cells with successful integration (GFP^+^) overexpress *Sox2* upon doxycycline administration (Supplementary Fig. 2C).

Quantitative PCR confirmed *Sox2* expression in doxycycline-treated cells compared with vehicle-treated or untransfected PC12 cells (Supplementary Fig. 2D). No gross differences in cell morphology or cell density were observed (Supplementary Fig. 2C). DNA content analysis by flow cytometry showed that doxycycline-induced, transfected cells (+DOX/GFP^+^) and non-induced, transfected cells (−DOX/GFP^+^) had similar proportions of cells in G2/M (3.2 and 3.6%, respectively). Likewise, doxycycline-treated and untreated GFP-negative, non-transfected control cells (+DOX/GFP^−^ and −DOX/GFP^−^) showed identical proportions of cells in G2/M (1.3%) (Supplementary Fig. 2E). Together, these data indicate that *Sox2* overexpression alone is not sufficient to promote cell cycle progression in PPGL cells, supporting its interpretation as a stem cell-associated feature, rather than a proliferative driver.

### PPGLs harbour cells that can be isolated in stem cell-promoting media and express SOX2 *in vitro*

**Figure 3:**
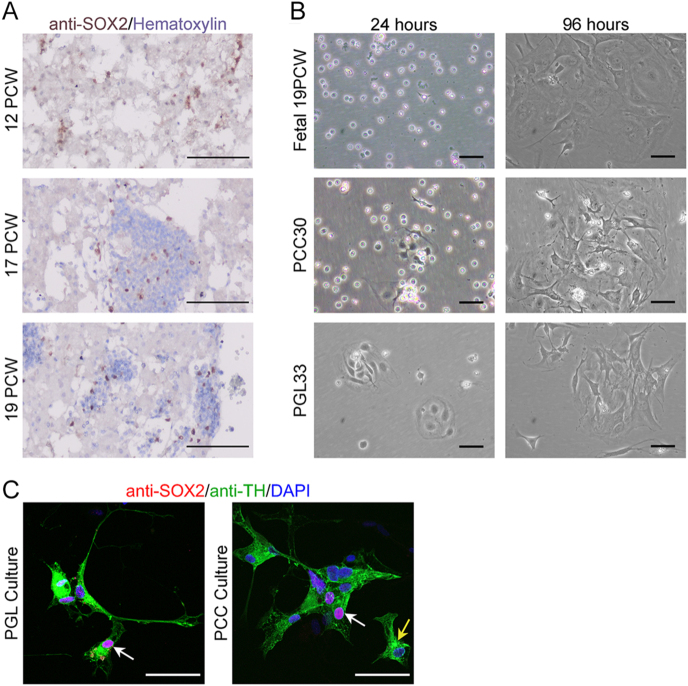
*In vitro isolation and characterisation of PPGL-derived stem cells.* (A) Immunohistochemistry confirming the presence of SOX2+ cells in fetal adrenal medullae at 12 post-conception weeks (PCW), 17 PCW and 19 PCW. SOX2-positive cells in brown, tissues counterstained with haematoxylin. Scale bars: 100 μm. (B) *In vitro* isolation of fetal adrenomedullary cells at 19 PCW leads to the generation of adherent colonies from 3 days under defined stem cell-promoting media. Scale bars: 50 μm. *In vitro* isolation of adult adrenomedullary cells from PCC30 benign phaeochromocytoma with no known mutations and PGL33, malignant paraganglioma with a germline mutation in *SDHD*, under defined stem cell-promoting media. Adherent cells and colonies seen at 24 and 96 h. Scale bars: 50 μm. (C) Double immunofluorescence staining using antibodies against SOX2 and TH confirms cells co-expressing both (white arrows) in PCC and PGL adherent colonies after ten days in culture in stem cell-promoting media, as well as cells only expressing TH (yellow arrow). Scale bars: 50 μm.

We previously established a protocol for the isolation of stem cells from the mouse adrenal medulla (14). We sought to determine if these culture conditions can be used to isolate stem cells from human tissues. Fetal adrenal medullae at 12, 17 and 19 post-conception weeks (PCW) were immunostained using antibodies against SOX2. Positive cells were identified in all conditions (Fig. 3A). Cells from an adrenal medulla at 19 PCW were dissociated into a single-cell suspension and plated in defined stem cell-promoting media (14). Cells adhered after 24 h and colonies were visible at 96 h (distinct by 72 h), which could be expanded and passaged (Fig. 3B). This stem cell isolation protocol was also applied to two fresh PPGL samples, a benign PCC with no known mutations (PCC30) and a metastatic PGL with a mutation in *SDHD* (PGL33); transcriptomic analyses confirmed *SOX2*-expressing cells in both tumours (Supplementary Fig. 1). Colonies were obtained from both tumours *in vitro* (Fig. 3B). Immunofluorescence against TH did not detect chromaffin cells in the control fetal sample (not shown) but did in two independent PCC and PGL cultures, where some TH-positive cells also expressed SOX2 (Fig. 3C). Isolated cells were capable of at least three passages and recovered one freeze–thaw cycle. These findings confirm the presence of a PPGL cell population with *in vitro* clonogenic properties, consistent with stem cell potential.

### SOX2+ PPGL cells have tumour-initiating capacity *in ovo*

**Figure 4:**
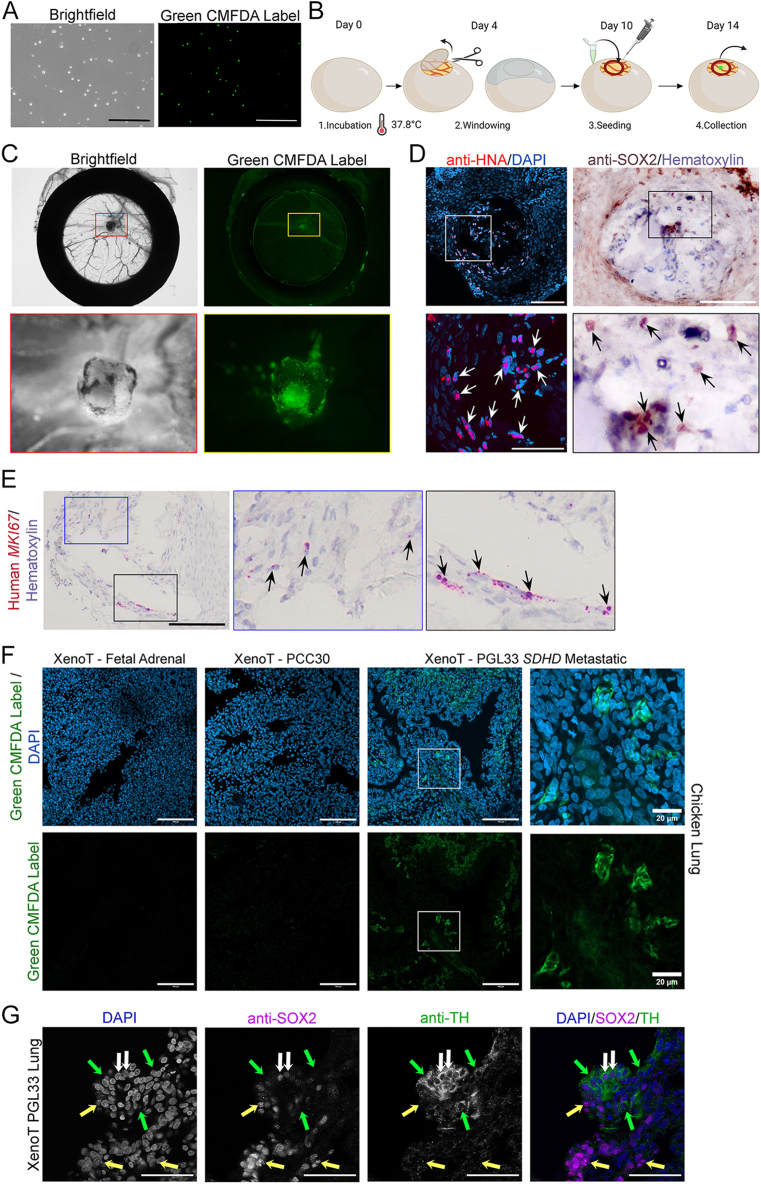
*SOX2+ PPGL stem cells have tumour-inducing and metastatic capacity in ovo.* (A) Single-cell suspension and labelling of fetal 19 PCW cells with CellTracker green CMFDA label. Scale bars: 500 μm. (B) Pipeline for the *in vitro* isolation and *in ovo* transplantation of human SOX2+ cells (schematic created in BioRender.com). (C) Chick chorioallantoic membrane (CAM) xenograft of PGL33 after 4 days of incubation. Bright-field and UV fluorescence images of the dissected CAM, with a visible cell mass established on the vascularised CAM. The silicon O-ring is visible. The red and yellow boxes indicate the magnified regions shown below, depicting the mass. (D) Immunostaining on formalin-fixed, paraffin sections of the xenograft in C. Immunofluorescence using antibodies against human nuclear antigen (HNA, red)) confirming the presence of human cells in the mass. Nuclei counterstained with DAPI. Immunohistochemistry with antibodies against SOX2 (brown), confirming the presence of SOX2-expressing cells. Nuclei counterstained with haematoxylin. Scale bars: 100 μm. (E) RNAscope mRNA *in situ* hybridisation using a human-specific *MKI67* probe (red) on CAM graft. Nuclei counterstained with haematoxylin. Scale bars: 100 μm. The arrows indicate detection of *MKI67* mRNA transcripts in insets. (F) Cryosections through the lungs of chicks at 14 days post-fertilisation, where CAMs were successfully grafted with either fetal adrenal stem cell cultures, isolated stem cell cultures from PCC30 or isolated stem cell cultures from PGL33. The green fluorescent cells are detected in PGL33 cultures confirming metastasis. Nuclei counterstained with DAPI. Scale bars: 100 μm, except on right-hand panels where scale bars are 20 μm. (G) Immunofluorescence staining on cryosections through chick lungs at 14 days post-fertilisation, including a PGL33 xenograft with metastasis, using antibodies against SOX2 (magenta) and TH (green). Nuclei counterstained with DAPI (blue). The greyscale images of single channels are shown for signal clarity. The yellow arrows indicate SOX2-positive cells, the green arrows indicate TH-positive cells, and the white arrows indicate SOX2; TH double-positive cells. Nuclei counterstained with DAPI. Scale bars: 50 μm. XenoT = xenotransplanted.

We next sought to determine if *in vitro* isolated SOX2+ PPGL cells can regenerate and metastasise in a xenograft assay. Isolated and expanded human SOX2+ PPGL cells PCC30 and PGL33, as well as control fetal adrenal cultures, were dissociated into a single-cell suspension and labelled with CellTracker CMFDA label, which converts into a green, fluorescent dye intracellularly, allowing cell tracing without dye transfer (Fig. 4A). Purified and labelled *in vitr*o isolated stem cells were seeded onto fertilised chick chorioallantoic membranes (CAMs) as a single-cell suspension (100,000 cells per CAM) and incubated for four days (see Fig. 4B for schematic). CAM assays are established *in vivo* models for the study of tumour propagation, angiogenesis and metastasis (49, 50, 51, 52, 53, 54). For each culture, grafting was carried out in a total of 10 eggs, over two independent experiments. Two eggs for PCC30, four eggs for PGL33 and four eggs for fetal controls survived and were analysed further. For PGL33, this led to the formation of tissue masses, presenting with green CMFDA label fluorescence (*n* = 4) (Fig. 4C). This was not observed in CAMs seeded with PCC30 (*n* = 2 surviving eggs), or control fetal adrenal-derived stem cells (*n* = 4 surviving eggs). Immunofluorescence using an antibody against human nuclear antigen (HNA) and immunohistochemistry using antibodies against SOX2 confirmed cells within the *de novo* formed masses are derived from seeded human PGL33 stem cells and continue to express SOX2 protein (Fig. 4D). A proportion of these cells or their descendants remain proliferative, as evidenced by human-specific *MKI67 in situ* in CAM grafts (Fig. 4E), marking cycling cells. The lungs of each chicken embryo were harvested at 14 days post-fertilisation, fixed and processed for cryosectioning to enable analysis of possible metastasis through visualisation of green fluorescence. Detection of green CMFDA fluorescence in host chickens’ lungs revealed the presence of xenotransplanted, *SDHD*-mutant, PGL33-derived stem cells or their derivatives, confirming their potential to invade and metastasise (Fig. 4F). Immunofluorescence staining using antibodies against SOX2 and TH on chicken lungs from PGL33 xenotransplantations demonstrated the presence of SOX2+ cells, TH+ chromaffin cells and infrequent double-positive cells (Fig. 4G). In summary, *in vitro* isolated PPGL stem cells have tumour-initiating and metastatic capacity in a xenograft assay.

## Discussion

Identifying the cell-of-origin in tumours and cancers is critical for developing accurate experimental models of tumourigenesis, discovering prognostic markers and the design of targeted therapies. The paradigm of CSCs or TICs contributing directly to tumour formation and maintenance has been established in various solid tumours (55, 56, 57, 58, 59, 60, 61). We have recently identified a neural crest-derived postnatal stem cell population expressing *SOX2*. Here, we demonstrate that *SOX2+* cells are consistently present in PPGLs, regardless of tumour location, malignancy status or underlying genetic alterations. *In silico* analysis of 17 PPGL single-cell RNA sequencing datasets corroborates widespread *SOX2* expression in all but two tumours, highlights an enrichment of *SOX2*-expressing cells in a subset (5 out of 17) and defines a robust molecular signature linked to metastatic potential. In addition to *EZH2*, *MND1* and *PIMREG*, which are known promoters of cancer progression and have previously emerged as candidate metastatic markers in PPGL (15), we report additional novel candidates associated with metastatic behaviour. One particularly promising candidate is cyclic adenosine monophosphate (cAMP)-response element-binding protein 5 (*CREB5*). In colorectal cancer, *CREB5* can promote invasion and metastasis through activation of the receptor tyrosine kinase *MET* and is associated with an unfavourable prognosis in multiple cancers, including hepatocellular carcinoma, glioma, breast, prostate, and epithelial ovarian cancer (27, 28). These candidates require functional validation to determine the true drivers of metastasis, which will subsequently guide the identification of actionable therapeutic targets. Future work encompassing larger tumour cohorts will better resolve these drivers and aid in prognostic predictions.

Stem cells have been implicated in cancer relapse and metastasis (62, 63) and can regenerate tumours when grafted (64). This study provides compelling evidence that PPGL-derived *SOX2*+ cells possess tumour-initiating capacity, with the ability to self-renew *in vitro*, regenerate and metastasise in xenotransplantation assays – key hallmarks of CSCs (56). We identified two distinct *SOX2*+ populations within PPGLs: a sustentacular population, also present in healthy controls, and a tumour-specific subpopulation of chromaffin cells, echoing earlier histological observations (13). The presence of double-positive cells (expressing both *SOX2* and chromaffin markers) exclusively in tumours is particularly intriguing; these cells may be a unique stem cell of the tumours, since these are absent in normal adrenal tissue. Such cells may arise either from re-activation of a stem-like transcriptional programme in chromaffin cells or from aberrant differentiation of sustentacular cells whilst they retain *SOX2* expression instead of downregulating this gene as in normal differentiation. Currently, it remains unclear whether metastasis in xenografts originates from *SOX2*+ tumour chromaffin cells or the *SOX2*+ sustentacular stem cell population, but the expression of *SOX2* itself does not appear to be implicated in this process. It is unlikely that *SOX2+* sustentacular cells are the cell-of-origin of these tumours, given their overtly quiescent nature based on transcriptomic studies. However, our data raise the possibility that PPGLs may contain both normal sustentacular cells recruited from surrounding tissue and a neoplastic sustentacular-like population derived from tumour cells, which may be indistinguishable by conventional markers. These populations may still contribute to tumourigenesis in a cell non-autonomous manner.

Supporting a direct role in tumour growth, *SOX2*+ tumour chromaffin cells actively proliferate, unlike the more quiescent *SOX2*+ sustentacular cells. Moreover, they display a distinct transcriptomic signature that is enriched in genes promoting tumourigenesis and cancer progression. Our prior work demonstrated that WNT6 secretion by adrenomedullary stem cells regulates chromaffin cell proliferation (14). In the present study, the expression of several *WNT* genes, including *WNT6*, and additional pro-tumourigenic factors by *SOX2*+ tumour chromaffin cells suggests a possible paracrine contribution to tumourigenesis, mirroring mechanisms observed in other cancers (65, 66, 67, 68, 69, 70, 71).

Overall, our findings establish that PPGLs contain cells with stem-like properties that can be isolated, expanded and transplanted and have the capacity to propagate tumours. These results provide the first functional evidence suggesting that *SOX2*+ cells in PPGLs possess the potential to serve as tumour cells of origin.

## Supplementary materials



## Declaration of interest

AS is currently an employee of Relation Therapeutics. IB is currently an employee of Novartis. The remaining authors declare no competing interests.

## Funding

This work was funded by the Medical Research Council (grant APP40962, to CLA); the Paradifference Foundation (to CLA and RO); the Deutsche Forschungsgemeinschaft (DFG, German Research Foundation) (Project no. 314061271, TRR 205: ‘The Adrenal: Central Relay in Health and Disease’ to CLA, SRB, CS, NB and MT; Project no. 288034826, IRTG 2251: ‘Immunological and Cellular Strategies in Metabolic Disease’ to CLA, SRB and CS); the Bernice Bibby Research Trust (to RJO and LI); the NIHR Biomedical Research Centre at Guy’s and St. Thomas’ NHSFT (to MQ and GSK); and the NIHR Biomedical Research Centre at Guy’s and St. Thomas’ NHSFT through the DRIVE-Health CDT (to DB). BK and MVS were funded by the Wellcome Trust as part of the Advanced Therapies for Regenerative Medicine Wellcome Trust PhD Programme (218461/Z/19/Z).
