# Supplementary material for: Phaeochromocytomas and paragangliomas harbour tumour-initiating SOX2+ stem cells
Source: Endocr Relat Cancer. 2026 May 12;33(5):e250242. doi: 10.1530/ERC-25-0242 (PMC13188188; doi:10.1530/ERC-25-0242)
Supplement: Supplementary file 1 [file supplementary_materials.pdf]

Supplementary Material

SOX2+ cells in phaeochromocytoma and paraganglioma are candidate tumour-initiating stem cells

Kemkem et al.

Supplementary Figure 1 *Single-cell analysis of PPGLs identifies SOX2 expression in healthy controls and 15 out of 17 tumours.* FeaturePlots of *SOX2* expression in PPGLs and control adrenals. Colour scale represents Log-normalised expression level, with 0 (grey) representing no expression, and 1.8 (red) representing highest expression.

Supplementary Figure 2 *Sox2 overexpression in PC12 rat pheochromocytoma cells does not confer a proliferative advantage.* A) Map of the (PB)TetO-SOX2_2xNLS-eGFP vector. B) Experimental workflow: PC12 cells were co-transfected with the (PB)TetO-SOX2_2xNLS-eGFP and PiggyBac Transposase Expression vectors. Cells with successful integration were selected through puromycin administration. *Sox2* overexpression was induced through doxycycline administration. Cell cycle analysis was performed by flow cytometry. Made using BioRender. C) Brightfield and GFP images of doxycycline-induced and non-induced PC12 cells in culture following 48 hours of doxycycline treatment. Scale bar = 20µm D) Quantitative PCR analysis of *Sox2* expression relative to *Hprt* in doxycycline-induced, transfected cells (+DOX), non-induced, transfected cells (-DOX), and doxycycline-treated, non-transfected cells (CTRL), confirming *Sox2* overexpression in this experimental model. Error bars represent ±SEM, ****p<0.0001, one-way ANOVA, *n*=3 independent samples. E) Flow cytometric analysis of DAPI stained cells was used to determine cell cycle distribution (G1, S, and G2/M), based on DNA content, analysis using FlowJo, sorting on 300,000 cells per sample. Representative histograms for doxycycline-induced, transfected PC12 cells (+DOX/GFP^{+}), non-induced, transfected cells (-DOX/GFP^{+}), doxycycline-treated non-transfected cells (+DOX/GFP^{-}) and non-treated, non-transfected cells (-DOX/GFP^{-}). Cells in G2/M expressed as percent of the total number of cells for each sample.

Supplementary Table 1 Quantification of SOX2 positive cells by immunohistochemistry for PPGL tumours 1-19, representative images of which are shown in Figure 1.

Supplementary Table 2 Genes defining each cell population in transcriptomic analysis.

Supplementary Table 3 Significant genes that are differentially expressed between chromaffin cells from tumours with a high SOX2+ cell proportion compared to chromaffin cells from tumours with a lower/absent SOX2+ cell proportion.

Supplementary Figure 1

SOX2 expression

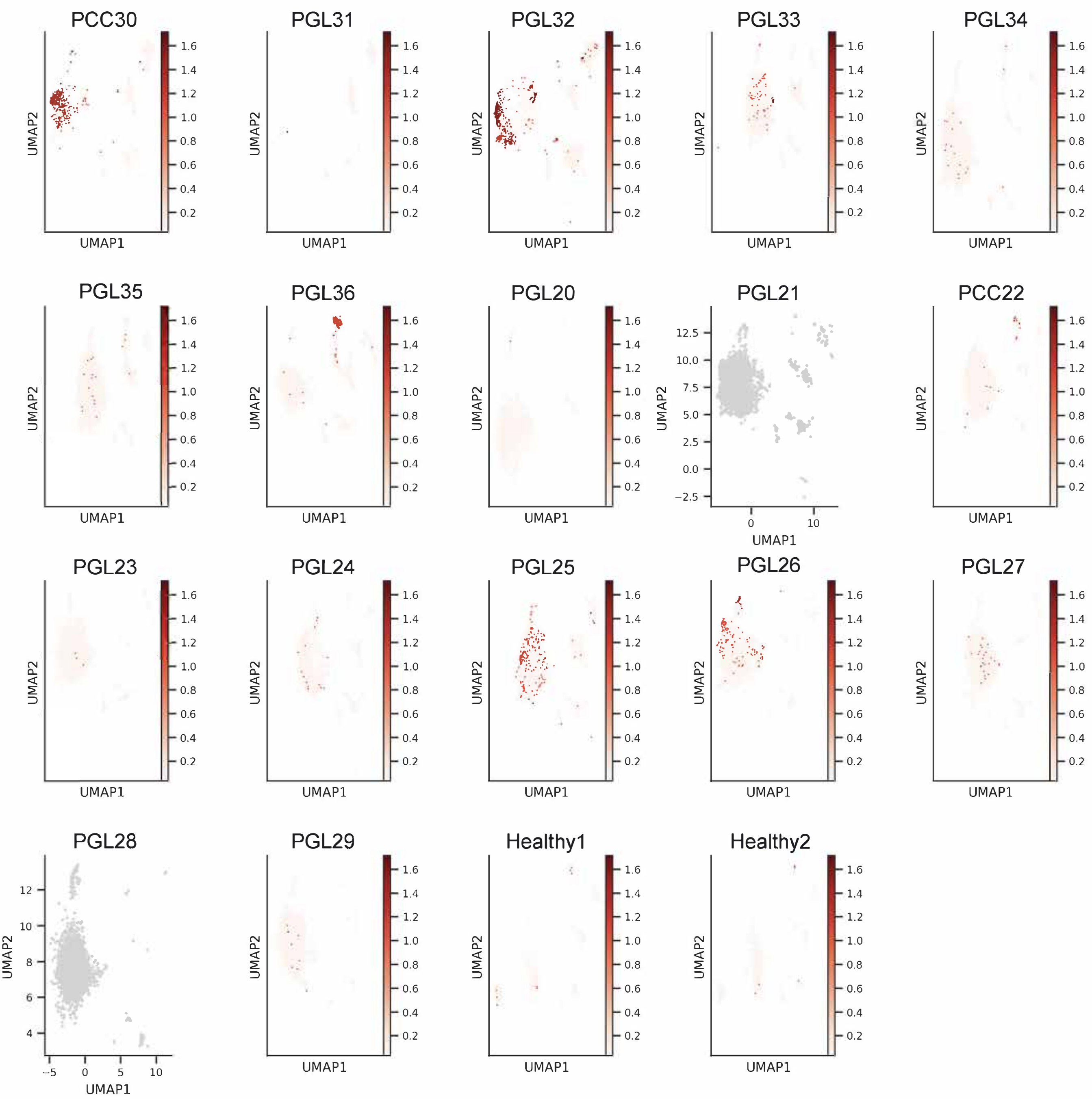

Supplementary Figure 2

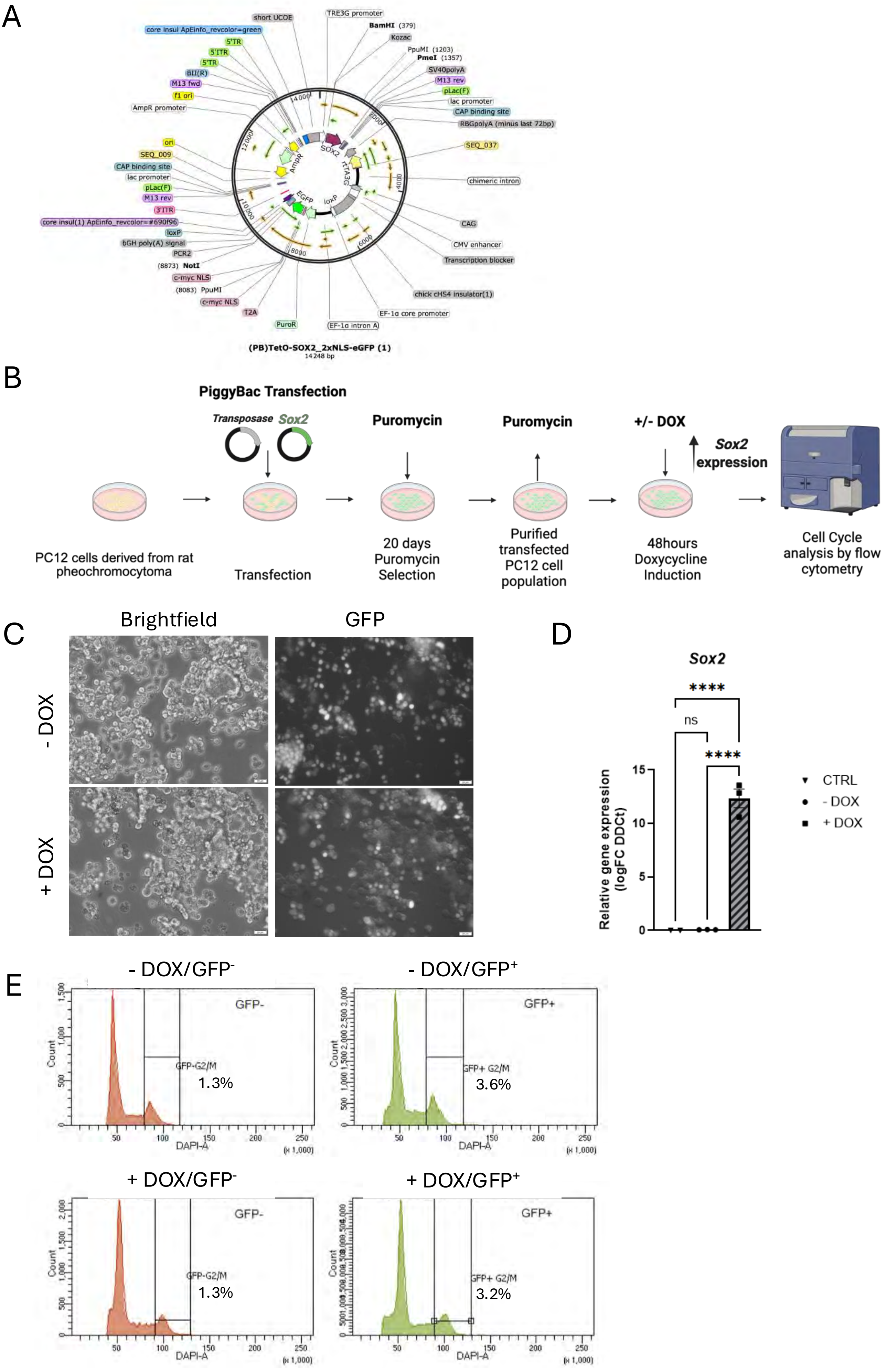

Supplementary Table 1 *Quantification of SOX2 positive cells by immunohistochemistry for PPGL tumours 1-19, representative images of which are shown in Figure 1.*

Tumour ID	Tumour type	Tumour status	%SOX2+ cells/nuclei
PCC1	Phaeochromocytoma	Primary	8.31
PCC2	Phaeochromocytoma	Primary	7.66
PCC3	Phaeochromocytoma	Primary	0.93
PCC4	Phaeochromocytoma	Primary	1.38
PCC5	Phaeochromocytoma	Primary	2.65
PCC6	Phaeochromocytoma	Primary	5.37
PCC7	Phaeochromocytoma	Primary	1.65
PGL8	Carotid body Paraganglioma	Primary	8.02
PCC9	Phaeochromocytoma	Primary	4.91
PCC10	Phaeochromocytoma	Primary	3.05
PCC11	Phaeochromocytoma	Primary	8.08
PGL12	Mediastinal Paraganglioma	Primary	4.18
PGL13	Retroperitoneal Paraganglioma	Primary	4.51
PGL14	Carotid body Paraganglioma	Primary	8.36
PGL15	Abdominal para-aortic Paraganglioma	Primary	3.92
PGL16	Abdominal para-aortic Paraganglioma	Primary	1.22
PGL17	Mediastinal Paraganglioma	Primary	5.79
PCC18	Phaeochromocytoma	Primary	3.51
PCC19	Phaeochromocytoma	Primary	2.79

Supplementary Table 2 *Genes defining each cell population in transcriptomic analysis.*

Gene	Cluster
GGT7	Chromaffin_cells
TOM1L1	Chromaffin_cells
SHTN1	Chromaffin_cells
DTX3	Chromaffin_cells
CCP110	Chromaffin_cells
ORC4	Chromaffin_cells
ZNF382	Chromaffin_cells
FASTKD1	Chromaffin_cells
NNT	Chromaffin_cells
CLDN12	Chromaffin_cells
GTF2H3	Chromaffin_cells
TMEM175	Chromaffin_cells
RSBN1L	Chromaffin_cells
GAS8	Chromaffin_cells
ARL3	Chromaffin_cells
MAPT	Chromaffin_cells
FKBP14	Chromaffin_cells
MAP3K21	Chromaffin_cells
WDR17	Chromaffin_cells
HAND2-AS1	Chromaffin_cells
SV2A	Chromaffin_cells
SFXN4	Chromaffin_cells
SARM1	Chromaffin_cells
RUFY3	Chromaffin_cells
ATP6V0A1	Chromaffin_cells
PAQR6	Chromaffin_cells
TLX2	Chromaffin_cells
DZIP3	Chromaffin_cells
ABHD18	Chromaffin_cells
MAST1	Chromaffin_cells
ZNF780B	Chromaffin_cells
FN3K	Chromaffin_cells
STRADB	Chromaffin_cells
PSD	Chromaffin_cells
COPS4	Chromaffin_cells
ZNF529	Chromaffin_cells
TTC3	Chromaffin_cells
UQCRC2	Chromaffin_cells
CEP290	Chromaffin_cells
IPO9	Chromaffin_cells
ASPHD1	Chromaffin_cells
ABCB7	Chromaffin_cells
TBL1X	Chromaffin_cells
L2HGDH	Chromaffin_cells
NUP58	Chromaffin_cells
ACBD6	Chromaffin_cells
TIMM44	Chromaffin_cells
CEP164	Chromaffin_cells
MICU2	Chromaffin_cells
SPEG	Chromaffin_cells
POGZ	Chromaffin_cells
FBXO41	Chromaffin_cells
ACTL6B	Chromaffin_cells
CEP295	Chromaffin_cells
CRYZL1	Chromaffin_cells
SSBP1	Chromaffin_cells
SLC25A12	Chromaffin_cells
CA11	Chromaffin_cells
SCRN1	Chromaffin_cells
TRIM37	Chromaffin_cells
KCNH2	Chromaffin_cells
SYT14	Chromaffin_cells
CEP44	Chromaffin_cells
ZNF540	Chromaffin_cells
SCN8A	Chromaffin_cells
REEP2	Chromaffin_cells
COPG2	Chromaffin_cells
DICER1-AS1	Chromaffin_cells
SMG5	Chromaffin_cells
TMEM38B	Chromaffin_cells
ZNF248	Chromaffin_cells
SCN3B	Chromaffin_cells
RUNDC3A	Chromaffin_cells
NALCN	Chromaffin_cells
PDZD7	Chromaffin_cells
ANKMY2	Chromaffin_cells
WHRN	Chromaffin_cells
MAP3K9	Chromaffin_cells
PCBP3	Chromaffin_cells
APC2	Chromaffin_cells
KIF3A	Chromaffin_cells
ADAM22	Chromaffin_cells
PPIP5K1	Chromaffin_cells
RBM28	Chromaffin_cells
NDUFV3	Chromaffin_cells
HCN3	Chromaffin_cells
TMEM51-AS1	Chromaffin_cells
ECSIT	Chromaffin_cells
MED6	Chromaffin_cells
HOMER2	Chromaffin_cells
RIC3	Chromaffin_cells
FBXO16	Chromaffin_cells
UCHL3	Chromaffin_cells
AKT2	Chromaffin_cells
PEX5L	Chromaffin_cells
TCAF1	Chromaffin_cells
SOGA3	Chromaffin_cells
MYH15	Chromaffin_cells
ANKS3	Chromaffin_cells
GNAO1	Chromaffin_cells
NME7	Chromaffin_cells
PREPL	Chromaffin_cells
KIAA1958	Chromaffin_cells
PLXNA3	Chromaffin_cells
TTC39A	Chromaffin_cells
TTC8	Chromaffin_cells
TAF15	Chromaffin_cells
MTMR4	Chromaffin_cells
LNPK	Chromaffin_cells
TBCEL	Chromaffin_cells
HIBADH	Chromaffin_cells
OBSL1	Chromaffin_cells
ONECUT2	Chromaffin_cells
KIAA0408	Chromaffin_cells
ACTR3B	Chromaffin_cells
GSTO2	Chromaffin_cells
ACACA	Chromaffin_cells
NOL4	Chromaffin_cells
FAXC	Chromaffin_cells
NECAB3	Chromaffin_cells
NOL3	Chromaffin_cells
NPEPPS	Chromaffin_cells
CELF3	Chromaffin_cells
SLC22A17	Chromaffin_cells
IDS	Chromaffin_cells
GNG4	Chromaffin_cells
C3orf14	Chromaffin_cells
CNTN1	Chromaffin_cells
KIFC2	Chromaffin_cells
RIMBP2	Chromaffin_cells
JPH4	Chromaffin_cells
UROS	Chromaffin_cells
OCIAD1	Chromaffin_cells
FUT9	Chromaffin_cells
CACNG2	Chromaffin_cells
LHFPL4	Chromaffin_cells
ZNF667	Chromaffin_cells
CUL7	Chromaffin_cells
SOBP	Chromaffin_cells
ATXN2L	Chromaffin_cells
OGDHL	Chromaffin_cells
ZNF229	Chromaffin_cells
CDON	Chromaffin_cells
ADNP2	Chromaffin_cells
TBX20	Chromaffin_cells
ISL1	Chromaffin_cells
GREB1L	Chromaffin_cells
KIF5A	Chromaffin_cells
PURA	Chromaffin_cells
SUPT20H	Chromaffin_cells
DCAF8	Chromaffin_cells
FOXRED1	Chromaffin_cells
TIAL1	Chromaffin_cells
RC3H1	Chromaffin_cells
ENO2	Chromaffin_cells
CAMK2B	Chromaffin_cells
SNAP91	Chromaffin_cells
BCAT2	Chromaffin_cells
PARP2	Chromaffin_cells
PPFIA3	Chromaffin_cells
RTN3	Chromaffin_cells
GAD1	Chromaffin_cells
ZMYM3	Chromaffin_cells
STXBP5L	Chromaffin_cells
DNM1L	Chromaffin_cells
MED24	Chromaffin_cells
LRRC4B	Chromaffin_cells
PPM1E	Chromaffin_cells
WASF1	Chromaffin_cells
GATA3-AS1	Chromaffin_cells
PLAG1	Chromaffin_cells
MPP2	Chromaffin_cells
MAB21L1	Chromaffin_cells
FARSB	Chromaffin_cells
DET1	Chromaffin_cells
SYP	Chromaffin_cells
SLC36A4	Chromaffin_cells
AP3B2	Chromaffin_cells
NUDCD3	Chromaffin_cells
ADAMTS13	Chromaffin_cells
CXADR	Chromaffin_cells
NDUFA5	Chromaffin_cells
ANKRD31	Chromaffin_cells
ASNS	Chromaffin_cells
RGS4	Chromaffin_cells
ZNF213-AS1	Chromaffin_cells
MYT1L	Chromaffin_cells
EML6	Chromaffin_cells
FAM185A	Chromaffin_cells
FMN2	Chromaffin_cells
KDM5B	Chromaffin_cells
STK33	Chromaffin_cells
FAM162B	Chromaffin_cells
TP53BP1	Chromaffin_cells
NUP133	Chromaffin_cells
FBXL16	Chromaffin_cells
STEAP3	Chromaffin_cells
NUDT7	Chromaffin_cells
HSPA4L	Chromaffin_cells
RIMS2	Chromaffin_cells
MSI2	Chromaffin_cells
ST7	Chromaffin_cells
TIA1	Chromaffin_cells
METTL2B	Chromaffin_cells
SGPP2	Chromaffin_cells
SCML2	Chromaffin_cells
ZNF821	Chromaffin_cells
MYL6B	Chromaffin_cells
SRSF1	Chromaffin_cells
PLCD4	Chromaffin_cells
CELSR3	Chromaffin_cells
DOCK3	Chromaffin_cells
VLDLR-AS1	Chromaffin_cells
PLA2G12A	Chromaffin_cells
DLD	Chromaffin_cells
SRRM4	Chromaffin_cells
SLC26A11	Chromaffin_cells
UNC80	Chromaffin_cells
ZNF891	Chromaffin_cells
UNC79	Chromaffin_cells
LUC7L3	Chromaffin_cells
TUSC3	Chromaffin_cells
BSN	Chromaffin_cells
TRIM46	Chromaffin_cells
FLVCR1	Chromaffin_cells
REEP1	Chromaffin_cells
ABHD8	Chromaffin_cells
LRPPRC	Chromaffin_cells
SYT1	Chromaffin_cells
SYN2	Chromaffin_cells
KBTBD3	Chromaffin_cells
FAM161A	Chromaffin_cells
CCSER1	Chromaffin_cells
SUN1	Chromaffin_cells
P2RX6	Chromaffin_cells
NOVA1	Chromaffin_cells
DDX1	Chromaffin_cells
FAM133A	Chromaffin_cells
BTBD8	Chromaffin_cells
SCAMP5	Chromaffin_cells
ELOVL7	Chromaffin_cells
ATCAY	Chromaffin_cells
RGS7	Chromaffin_cells
PHKA1	Chromaffin_cells
ACAD10	Chromaffin_cells
RAB3C	Chromaffin_cells
DPYSL5	Chromaffin_cells
ZFP30	Chromaffin_cells
ZNF692	Chromaffin_cells
CHRNA7	Chromaffin_cells
LURAP1L-AS1	Chromaffin_cells
PAN2	Chromaffin_cells
CACNA1D	Chromaffin_cells
METTL2A	Chromaffin_cells
SPIRE2	Chromaffin_cells
MDH1	Chromaffin_cells
TMEM145	Chromaffin_cells
MSANTD4	Chromaffin_cells
EIF2AK1	Chromaffin_cells
CHRNA3	Chromaffin_cells
C7orf50	Chromaffin_cells
SRRM3	Chromaffin_cells
SNAP25-AS1	Chromaffin_cells
TIGD7	Chromaffin_cells
PSMC2	Chromaffin_cells
AMH	Chromaffin_cells
TRAP1	Chromaffin_cells
TMX2	Chromaffin_cells
BEGAIN	Chromaffin_cells
ZNF337	Chromaffin_cells
NMNAT2	Chromaffin_cells
AKAP1	Chromaffin_cells
ZDHHC2	Chromaffin_cells
MAPK8IP2	Chromaffin_cells
POLL	Chromaffin_cells
CATSPER2	Chromaffin_cells
RNPS1	Chromaffin_cells
CFAP36	Chromaffin_cells
PI4KB	Chromaffin_cells
FAM66D	Chromaffin_cells
SND1-IT1	Chromaffin_cells
UCHL1	Chromaffin_cells
IGFBPL1	Chromaffin_cells
STXBP1	Chromaffin_cells
PRPF3	Chromaffin_cells
ZGRF1	Chromaffin_cells
SLC8A3	Chromaffin_cells
AVL9	Chromaffin_cells
LCORL	Chromaffin_cells
PTPRN2	Chromaffin_cells
SYNGAP1	Chromaffin_cells
SCN2A	Chromaffin_cells
THOP1	Chromaffin_cells
ALG9	Chromaffin_cells
GTF2I	Chromaffin_cells
UBE2G2	Chromaffin_cells
CACNG7	Chromaffin_cells
PROX1-AS1	Chromaffin_cells
RNFT2	Chromaffin_cells
KHDC4	Chromaffin_cells
GALNT13	Chromaffin_cells
TNPO1	Chromaffin_cells
MCCC2	Chromaffin_cells
TNPO2	Chromaffin_cells
CIART	Chromaffin_cells
INA	Chromaffin_cells
AARSD1	Chromaffin_cells
KCNQ2	Chromaffin_cells
EIF2S3	Chromaffin_cells
POLR1A	Chromaffin_cells
LINC01535	Chromaffin_cells
MAPK8IP1	Chromaffin_cells
ABCB9	Chromaffin_cells
NOS1AP	Chromaffin_cells
PTGES2	Chromaffin_cells
NR2C1	Chromaffin_cells
RPS6KL1	Chromaffin_cells
MMAB	Chromaffin_cells
ATP1B1	Chromaffin_cells
GOT1	Chromaffin_cells
SLC9A7	Chromaffin_cells
NTPCR	Chromaffin_cells
ENSA	Chromaffin_cells
LRP11	Chromaffin_cells
PAICS	Chromaffin_cells
ZNF502	Chromaffin_cells
ZNF772	Chromaffin_cells
DGUOK	Chromaffin_cells
TRIM2	Chromaffin_cells
DCUN1D5	Chromaffin_cells
PCLO	Chromaffin_cells
TMEM63B	Chromaffin_cells
PDHA1	Chromaffin_cells
PTRH2	Chromaffin_cells
NAA25	Chromaffin_cells
KCTD16	Chromaffin_cells
INTS3	Chromaffin_cells
KLC2	Chromaffin_cells
SPTB	Chromaffin_cells
CDS1	Chromaffin_cells
ATG4B	Chromaffin_cells
FAIM2	Chromaffin_cells
XPOT	Chromaffin_cells
NT5DC2	Chromaffin_cells
KIAA0319	Chromaffin_cells
SNAP25	Chromaffin_cells
C1orf226	Chromaffin_cells
SEZ6L2	Chromaffin_cells
SESN3	Chromaffin_cells
SCRT1	Chromaffin_cells
DHX40	Chromaffin_cells
SLC9B2	Chromaffin_cells
DPP6	Chromaffin_cells
CAMSAP3	Chromaffin_cells
MC1R	Chromaffin_cells
CCPG1	Chromaffin_cells
VPS13A	Chromaffin_cells
DUSP12	Chromaffin_cells
TCF25	Chromaffin_cells
GVQW3	Chromaffin_cells
ADD2	Chromaffin_cells
AKAP8L	Chromaffin_cells
PTPRN	Chromaffin_cells
SEZ6L	Chromaffin_cells
TBC1D4	Chromaffin_cells
PRH1	Chromaffin_cells
CDC5L	Chromaffin_cells
CISD1	Chromaffin_cells
IGSF9B	Chromaffin_cells
TSNAX	Chromaffin_cells
TBC1D24	Chromaffin_cells
STT3A	Chromaffin_cells
CRNDE	Chromaffin_cells
FAF2	Chromaffin_cells
ZNF316	Chromaffin_cells
PROX1	Chromaffin_cells
CRMP1	Chromaffin_cells
RPRD1B	Chromaffin_cells
ZCRB1	Chromaffin_cells
SLC25A14	Chromaffin_cells
IARS2	Chromaffin_cells
LTA4H	Chromaffin_cells
COQ9	Chromaffin_cells
RNF40	Chromaffin_cells
AK4	Chromaffin_cells
ASL	Chromaffin_cells
RALGAPB	Chromaffin_cells
PIGQ	Chromaffin_cells
BSCL2	Chromaffin_cells
AGAP4	Chromaffin_cells
GRIK4	Chromaffin_cells
FAM171B	Chromaffin_cells
TSC2	Chromaffin_cells
DPH7	Chromaffin_cells
NUMA1	Chromaffin_cells
GOSR2	Chromaffin_cells
SPRYD7	Chromaffin_cells
ASPSCR1	Chromaffin_cells
NBPF15	Chromaffin_cells
RNF165	Chromaffin_cells
CXXC4	Chromaffin_cells
RYR2	Chromaffin_cells
ZDHHC16	Chromaffin_cells
HID1	Chromaffin_cells
CYP46A1	Chromaffin_cells
VEZT	Chromaffin_cells
COX7A2L	Chromaffin_cells
MRRF	Chromaffin_cells
GOT2	Chromaffin_cells

NOSTRIN	Endothelial_cells	RP1	Endothelial_cells	RAMP2	Endothelial_cells
AFAP1L1	Endothelial_cells	PABPC4L	Endothelial_cells	GABRD	Endothelial_cells
NOTCH4	Endothelial_cells	RAPGEF3	Endothelial_cells	RAPGEF5	Endothelial_cells
CDH5	Endothelial_cells	JCAD	Endothelial_cells	IGFBP3	Endothelial_cells
EXOC3L2	Endothelial_cells	PLA1A	Endothelial_cells	GRB10	Endothelial_cells
BTNL9	Endothelial_cells	ASB9	Endothelial_cells	ACKR3	Endothelial_cells
ROBO4	Endothelial_cells	GIMAP8	Endothelial_cells	LRRC8A	Endothelial_cells
DIPK2B	Endothelial_cells	ELK3	Endothelial_cells	HEY1	Endothelial_cells
KDR	Endothelial_cells	PALMD	Endothelial_cells	HYAL2	Endothelial_cells
MYCT1	Endothelial_cells	GPR4	Endothelial_cells	TMEM44	Endothelial_cells
CLEC1A	Endothelial_cells	FCN3	Endothelial_cells	MAGI1	Endothelial_cells
ADGRL4	Endothelial_cells	SPTBN5	Endothelial_cells	DENND11	Endothelial_cells
EMCN	Endothelial_cells	STC2	Endothelial_cells	TMOD3	Endothelial_cells
HSPG2	Endothelial_cells	CDH13	Endothelial_cells	ADA	Endothelial_cells
PCAT19	Endothelial_cells	CCM2L	Endothelial_cells	SHROOM2	Endothelial_cells
PECAM1	Endothelial_cells	SOX17	Endothelial_cells	EML1	Endothelial_cells
FLT4	Endothelial_cells	KCNQ1	Endothelial_cells	CCDC50	Endothelial_cells
ERG	Endothelial_cells	ITGA8	Endothelial_cells	SOX7	Endothelial_cells
NOS3	Endothelial_cells	VEGFC	Endothelial_cells	SDCBP2	Endothelial_cells
GJA1	Endothelial_cells	MMRN1	Endothelial_cells	RFLNB	Endothelial_cells
MPZL2	Endothelial_cells	CDA	Endothelial_cells	JAG2	Endothelial_cells
TIE1	Endothelial_cells	CX3CL1	Endothelial_cells	UPP1	Endothelial_cells
KCNE3	Endothelial_cells	TGM2	Endothelial_cells	PRKD2	Endothelial_cells
ADCY4	Endothelial_cells	ITGA6	Endothelial_cells	PDE2A	Endothelial_cells
TAL1	Endothelial_cells	CPAMD8	Endothelial_cells	PINK1	Endothelial_cells
SLCO2A1	Endothelial_cells	LINC01235	Endothelial_cells	RNF220	Endothelial_cells
ARHGEF15	Endothelial_cells	SMAD6	Endothelial_cells	CYYR1	Endothelial_cells
BCL6B	Endothelial_cells	DUSP6	Endothelial_cells	SLC5A4	Endothelial_cells
PLVAP	Endothelial_cells	CA2	Endothelial_cells	RAMP2-AS1	Endothelial_cells
KANK3	Endothelial_cells	SPNS2	Endothelial_cells	MCF2L	Endothelial_cells
ARHGAP29	Endothelial_cells	BTNL8	Endothelial_cells	BDKRB2	Endothelial_cells
FLT1	Endothelial_cells	PLK2	Endothelial_cells	CDK17	Endothelial_cells
LDB2	Endothelial_cells	LINC02582	Endothelial_cells	GRAMD1A	Endothelial_cells
DLL4	Endothelial_cells	ITIH5	Endothelial_cells	PIP5K1C	Endothelial_cells
ANO2	Endothelial_cells	PKN3	Endothelial_cells	TMEM150C	Endothelial_cells
PLPP3	Endothelial_cells	PKD1L1	Endothelial_cells	NETO1	Endothelial_cells
VWF	Endothelial_cells	DCHS1	Endothelial_cells	BCL10	Endothelial_cells
ESAM	Endothelial_cells	PREX2	Endothelial_cells	ARL15	Endothelial_cells
RASGRF2	Endothelial_cells	TP53I11	Endothelial_cells	STC1	Endothelial_cells
S1PR1	Endothelial_cells	EDNRB	Endothelial_cells	BMX	Endothelial_cells
CD34	Endothelial_cells	LHX6	Endothelial_cells	PCDH17	Endothelial_cells
SPRY1	Endothelial_cells	FGD5	Endothelial_cells	EPAS1	Endothelial_cells
ACVRL1	Endothelial_cells	LOXHD1	Endothelial_cells	SLC35E4	Endothelial_cells
PTPRB	Endothelial_cells	CLEC14A	Endothelial_cells	SMAD1	Endothelial_cells
TEK	Endothelial_cells	SHANK3	Endothelial_cells	TMC7	Endothelial_cells
RASIP1	Endothelial_cells	FAM107A	Endothelial_cells	LINC01320	Endothelial_cells
EPHB4	Endothelial_cells	FRMD8	Endothelial_cells	FGD6	Endothelial_cells
WWTR1	Endothelial_cells	VWA1	Endothelial_cells	USHBP1	Endothelial_cells
CPNE5	Endothelial_cells	GALNT15	Endothelial_cells	NOVA2	Endothelial_cells
ECSCR	Endothelial_cells	ENG	Endothelial_cells	RGS3	Endothelial_cells
FZD4	Endothelial_cells	ITGA10	Endothelial_cells	CNST	Endothelial_cells
GRAPL	Endothelial_cells	PIK3R3	Endothelial_cells	MOV10L1	Endothelial_cells
RASGRP3	Endothelial_cells	PCDH12	Endothelial_cells	TBCD	Endothelial_cells
PDLIM1	Endothelial_cells	MYOM3	Endothelial_cells	ZHX3	Endothelial_cells
RHOJ	Endothelial_cells	FAM110D	Endothelial_cells	USP13	Endothelial_cells
SEMA3F	Endothelial_cells	COL13A1	Endothelial_cells	THSD1	Endothelial_cells
CALCRL	Endothelial_cells	ELFN1	Endothelial_cells	LRATD1	Endothelial_cells
EFCC1	Endothelial_cells	LMO2	Endothelial_cells	PIK3C2B	Endothelial_cells
MMRN2	Endothelial_cells	IQCA1	Endothelial_cells	PNP	Endothelial_cells
DYSF	Endothelial_cells	YES1	Endothelial_cells	PTPRM	Endothelial_cells
EHD4	Endothelial_cells	VIP	Endothelial_cells	GFOD2	Endothelial_cells
TMEM233	Endothelial_cells	CCDC60	Endothelial_cells	TNFAIP1	Endothelial_cells
CAVIN2	Endothelial_cells	TSPAN18	Endothelial_cells	YWHAH	Endothelial_cells
FBXO39	Endothelial_cells	CLDN5	Endothelial_cells	RALGAPA2	Endothelial_cells
TM4SF1	Endothelial_cells	SEMA3F-AS1	Endothelial_cells	GALNT1	Endothelial_cells
EFNB2	Endothelial_cells	FSCN1	Endothelial_cells	ARL4A	Endothelial_cells
HSPA12B	Endothelial_cells	EXOC3L1	Endothelial_cells	EOGT	Endothelial_cells
KCNK5	Endothelial_cells	FKBP1A	Endothelial_cells	MYO6	Endothelial_cells
SHE	Endothelial_cells	LINC01483	Endothelial_cells	TAOK2	Endothelial_cells
GRAP	Endothelial_cells	RBP7	Endothelial_cells	SLC35B4	Endothelial_cells
EPB41L4A	Endothelial_cells	CTNNB1	Endothelial_cells	SIN3B	Endothelial_cells
INHBB	Endothelial_cells	PTPN5	Endothelial_cells	MRTFB	Endothelial_cells
MECOM	Endothelial_cells	KCNJ2	Endothelial_cells	HECW2	Endothelial_cells
F2RL3	Endothelial_cells	TJP2	Endothelial_cells	KLHL5	Endothelial_cells
FOLH1	Endothelial_cells	ST6GALNAC3	Endothelial_cells	CDKN2B	Endothelial_cells
ARAP3	Endothelial_cells	DLL1	Endothelial_cells	MPP3	Endothelial_cells
SH2D4B	Endothelial_cells	SNRK	Endothelial_cells	APP	Endothelial_cells
PODXL	Endothelial_cells	GRPEL2	Endothelial_cells	FNBP1L	Endothelial_cells
RAMP3	Endothelial_cells	CFAP74	Endothelial_cells	PDCL	Endothelial_cells
EGFL7	Endothelial_cells	F8	Endothelial_cells	RAPGEF4	Endothelial_cells
GIPC3	Endothelial_cells	APLNR	Endothelial_cells	PPP1R13B	Endothelial_cells
TM4SF18	Endothelial_cells	SOX18	Endothelial_cells	MFAP3	Endothelial_cells
EFNA1	Endothelial_cells	APOLD1	Endothelial_cells	RIMKLB	Endothelial_cells
TMEM255B	Endothelial_cells	ESM1	Endothelial_cells	MAOA	Endothelial_cells
TCIM	Endothelial_cells	AQP1	Endothelial_cells	TCF4	Endothelial_cells
LNX1	Endothelial_cells	HIP1R	Endothelial_cells	OPA3	Endothelial_cells
VSIG2	Endothelial_cells	INSR	Endothelial_cells	GBA2	Endothelial_cells
GABRE	Endothelial_cells	CASKIN2	Endothelial_cells	TCEA2	Endothelial_cells
NR5A2	Endothelial_cells	CFAP161	Endothelial_cells	TSPAN13	Endothelial_cells
IVNS1ABP	Endothelial_cells	JUP	Endothelial_cells	PPM1D	Endothelial_cells
SCARF1	Endothelial_cells	DOCK9	Endothelial_cells	LMBR1	Endothelial_cells
BMP6	Endothelial_cells	EDN1	Endothelial_cells	CEACAM19	Endothelial_cells
ADAMTS5	Endothelial_cells	TNFAIP8L1	Endothelial_cells	NPDC1	Endothelial_cells
MANCR	Endothelial_cells	SPINK5	Endothelial_cells	AFDN	Endothelial_cells
ZNF366	Endothelial_cells	CDC42EP2	Endothelial_cells		
MALL	Endothelial_cells	SLC10A6	Endothelial_cells		
SPAAR	Endothelial_cells	MAGI1-IT1	Endothelial_cells		
PROSER2	Endothelial_cells	ADAMTS18	Endothelial_cells		
PDGFD	Endothelial_cells	PLXNA2	Endothelial_cells		

CSPG4	Fibroblasts	C7	Fibroblasts	C11orf96	Fibroblasts
RBPMS2	Fibroblasts	LHFPL6	Fibroblasts	LINC00607	Fibroblasts
ANO1	Fibroblasts	PHLDA1	Fibroblasts	SLC38A2	Fibroblasts
LINC02664	Fibroblasts	LAMA2	Fibroblasts	FAM13C	Fibroblasts
LINC02204	Fibroblasts	COL3A1	Fibroblasts	VASN	Fibroblasts
COL6A2	Fibroblasts	MT1E	Fibroblasts	SETBP1	Fibroblasts
TINAGL1	Fibroblasts	FBLN1	Fibroblasts	ARHGAP10	Fibroblasts
GGT5	Fibroblasts	FHL1	Fibroblasts	C2orf27A	Fibroblasts
LINC00702	Fibroblasts	THBS2	Fibroblasts		
FN1	Fibroblasts	CHRD	Fibroblasts		
MEOX2	Fibroblasts	PTH1R	Fibroblasts		
TRPC4	Fibroblasts	TBX15	Fibroblasts		
PLCL1	Fibroblasts	MYH11	Fibroblasts		
LINC02202	Fibroblasts	LINC01091	Fibroblasts		
SELENBP1	Fibroblasts	PAMR1	Fibroblasts		
PDGFRB	Fibroblasts	SLC38A11	Fibroblasts		
ENPEP	Fibroblasts	KLHL23	Fibroblasts		
REM1	Fibroblasts	SLC6A1	Fibroblasts		
NGF-AS1	Fibroblasts	PDZRN4	Fibroblasts		
POSTN	Fibroblasts	LDB3	Fibroblasts		
CD248	Fibroblasts	PRDM6	Fibroblasts		
LOX	Fibroblasts	DUXAP8	Fibroblasts		
PDE1A	Fibroblasts	SMOC2	Fibroblasts		
EBF2	Fibroblasts	CNN1	Fibroblasts		
CFH	Fibroblasts	GFPT2	Fibroblasts		
CRISPLD2	Fibroblasts	DCN	Fibroblasts		
PTGIR	Fibroblasts	C1R	Fibroblasts		
VCAN	Fibroblasts	KCNE4	Fibroblasts		
SULF1	Fibroblasts	IL1RL2	Fibroblasts		
LINC00924	Fibroblasts	PDGFRA	Fibroblasts		
S1PR3	Fibroblasts	COL1A1	Fibroblasts		
ADIRF	Fibroblasts	TARID	Fibroblasts		
UACA	Fibroblasts	CCDC102B	Fibroblasts		
PRKG1	Fibroblasts	RGS6	Fibroblasts		
AVPR1A	Fibroblasts	ADAMTSL3	Fibroblasts		
PCDH18	Fibroblasts	CCDC80	Fibroblasts		
ACAN	Fibroblasts	TGFB3	Fibroblasts		
MYL9	Fibroblasts	STK38L	Fibroblasts		
NOTCH3	Fibroblasts	CLMN	Fibroblasts		
FHL2	Fibroblasts	GUCY1B1	Fibroblasts		
ZBTB7C	Fibroblasts	ENAH	Fibroblasts		
FHL5	Fibroblasts	TAGLN	Fibroblasts		
GJA4	Fibroblasts	SLC24A3	Fibroblasts		
GJC1	Fibroblasts	ITGA7	Fibroblasts		
CCN4	Fibroblasts	SVEP1	Fibroblasts		
C1QTNF1	Fibroblasts	EDNRA	Fibroblasts		
LINC01426	Fibroblasts	NEXN	Fibroblasts		
NR2F2	Fibroblasts	MOCS1	Fibroblasts		
FGF1	Fibroblasts	MYOM1	Fibroblasts		
EPS8	Fibroblasts	ADCY3	Fibroblasts		
LINC02469	Fibroblasts	THSD7B	Fibroblasts		
TBX18	Fibroblasts	ACTA2	Fibroblasts		
INHBA	Fibroblasts	BMP8A	Fibroblasts		
PLAC9	Fibroblasts	PTCH2	Fibroblasts		
ADAMTS14	Fibroblasts	FGF7	Fibroblasts		
CYP1B1-AS1	Fibroblasts	ITGBL1	Fibroblasts		
LINC00989	Fibroblasts	PLEKHH2	Fibroblasts		
CASQ2	Fibroblasts	ADAMTS2	Fibroblasts		
CDH11	Fibroblasts	ABCC9	Fibroblasts		
EFEMP1	Fibroblasts	GUCY1A2	Fibroblasts		
COL6A3	Fibroblasts	CARMN	Fibroblasts		
TNC	Fibroblasts	RNF24	Fibroblasts		
ADRA1B	Fibroblasts	NBL1	Fibroblasts		
TPPP3	Fibroblasts	LINC00578	Fibroblasts		
TNFAIP6	Fibroblasts	CSRP2	Fibroblasts		
PRRX2	Fibroblasts	GLI2	Fibroblasts		
COL5A3	Fibroblasts	ART4	Fibroblasts		
TPM2	Fibroblasts	PDE4C	Fibroblasts		
CALD1	Fibroblasts	IL34	Fibroblasts		
DACT1	Fibroblasts	TBX2	Fibroblasts		
BGN	Fibroblasts	ANO3	Fibroblasts		
DCDC2C	Fibroblasts	PCOLCE	Fibroblasts		
ADAMTS12	Fibroblasts	KCNAB1	Fibroblasts		
NGF	Fibroblasts	SEPTIN11	Fibroblasts		
MMP11	Fibroblasts	FAM9B	Fibroblasts		
STEAP4	Fibroblasts	ANGPT1	Fibroblasts		
PPP1R14A	Fibroblasts	ZFPM2-AS1	Fibroblasts		
NPY1R	Fibroblasts	MAP3K20	Fibroblasts		
CACNA1H	Fibroblasts	NMD3	Fibroblasts		
FAT3	Fibroblasts	LRRC17	Fibroblasts		
LMOD1	Fibroblasts	SLCO3A1	Fibroblasts		
CYP4X1	Fibroblasts	CLTCL1	Fibroblasts		
HEYL	Fibroblasts	RNF152	Fibroblasts		
MYOCD	Fibroblasts	SPECC1	Fibroblasts		

HMCN1	Sustentacular_cells
KLHDC8A	Sustentacular_cells
LINC01474	Sustentacular_cells
ANXA1	Sustentacular_cells
GPR17	Sustentacular_cells
SOX8	Sustentacular_cells
CASC15	Sustentacular_cells
SAMD5	Sustentacular_cells
PLAT	Sustentacular_cells
SH3TC2-DT	Sustentacular_cells
C5orf67	Sustentacular_cells
AQP7	Sustentacular_cells
ADGRG6	Sustentacular_cells
WDR86	Sustentacular_cells
CLDN11	Sustentacular_cells
NBAT1	Sustentacular_cells
LINC00354	Sustentacular_cells
ITGB4	Sustentacular_cells
GLIS3	Sustentacular_cells
SLITRK2	Sustentacular_cells
LINC02751	Sustentacular_cells
RGS8	Sustentacular_cells
LINC01622	Sustentacular_cells
LINC02251	Sustentacular_cells
GJA5	Sustentacular_cells
GPR149	Sustentacular_cells
FOXD3	Sustentacular_cells
ARSI	Sustentacular_cells
LINC01117	Sustentacular_cells
HEPACAM	Sustentacular_cells
FOXD3-AS1	Sustentacular_cells
ARNT2	Sustentacular_cells
TRIM63	Sustentacular_cells
RCAN1	Sustentacular_cells
GRIN2B	Sustentacular_cells
MEGF6	Sustentacular_cells
TGFB2	Sustentacular_cells
GALNT5	Sustentacular_cells
CTXND1	Sustentacular_cells
LINC00598	Sustentacular_cells
GDNF	Sustentacular_cells
ART3	Sustentacular_cells
MEGF10	Sustentacular_cells
LRRC8C-DT	Sustentacular_cells
ANXA3	Sustentacular_cells
RXRG	Sustentacular_cells
EGFL8	Sustentacular_cells
TMEM132B	Sustentacular_cells
SPATA22	Sustentacular_cells
DCSTAMP	Sustentacular_cells
MFSD2A	Sustentacular_cells
ADAMTS8	Sustentacular_cells
KREMEN1	Sustentacular_cells
MIR3659HG	Sustentacular_cells
LINC00407	Sustentacular_cells
MYOT	Sustentacular_cells
FBN3	Sustentacular_cells
CYP27C1	Sustentacular_cells
MAL	Sustentacular_cells
LINC01198	Sustentacular_cells
MACROD2	Sustentacular_cells
VSTM2B	Sustentacular_cells
LINC01748	Sustentacular_cells
TTLL7	Sustentacular_cells
LINC00466	Sustentacular_cells
ALDH1A1	Sustentacular_cells
SSUH2	Sustentacular_cells
CCN3	Sustentacular_cells
NAMA	Sustentacular_cells
FNDC1	Sustentacular_cells
KCNS3	Sustentacular_cells
SH3PXD2A	Sustentacular_cells
SPATA6	Sustentacular_cells
LINC02698	Sustentacular_cells
SFRP1	Sustentacular_cells
FHDC1	Sustentacular_cells
ASPA	Sustentacular_cells
PLEKHB1	Sustentacular_cells
ERBIN	Sustentacular_cells
TIMP4	Sustentacular_cells
TSPAN11	Sustentacular_cells
GDNF-AS1	Sustentacular_cells
NRXN1	Sustentacular_cells
LGI4	Sustentacular_cells
ABCA8	Sustentacular_cells
ILDR2	Sustentacular_cells
CDH19	Sustentacular_cells
VWC2	Sustentacular_cells
DTNA	Sustentacular_cells
TTPA	Sustentacular_cells
FRMD5	Sustentacular_cells
C4orf19	Sustentacular_cells
COL9A3	Sustentacular_cells
WNT4	Sustentacular_cells
TSBP1-AS1	Sustentacular_cells
DST	Sustentacular_cells
ADAM23	Sustentacular_cells
TIGD4	Sustentacular_cells
SERPINA3	Sustentacular_cells
CAMK2A	Sustentacular_cells
LINC00327	Sustentacular_cells
TRDN-AS1	Sustentacular_cells
GRAMD2A	Sustentacular_cells
SLC5A7	Sustentacular_cells
LINC01608	Sustentacular_cells
SPHKAP	Sustentacular_cells
STK32A-AS1	Sustentacular_cells
ADK	Sustentacular_cells
SYNPR-AS1	Sustentacular_cells
COL11A1	Sustentacular_cells
LINC01505	Sustentacular_cells
COBL	Sustentacular_cells
KANK1	Sustentacular_cells
SH3BGR	Sustentacular_cells
ADGRB3	Sustentacular_cells
DOCK7	Sustentacular_cells
PLEKHH1	Sustentacular_cells
RASGEF1C	Sustentacular_cells
TENM3	Sustentacular_cells
KLHL3	Sustentacular_cells
FIGN	Sustentacular_cells
PLP1	Sustentacular_cells
LINC00393	Sustentacular_cells
P3H2-AS1	Sustentacular_cells
KDSR	Sustentacular_cells
SHC4	Sustentacular_cells
TMEM72-AS1	Sustentacular_cells
RSPO3	Sustentacular_cells
CA1	Sustentacular_cells
SLC47A1	Sustentacular_cells
CAB39L	Sustentacular_cells
PKN2-AS1	Sustentacular_cells
TRDN	Sustentacular_cells
RTKN	Sustentacular_cells
B4GALT6	Sustentacular_cells
NKAIN3	Sustentacular_cells
CA3	Sustentacular_cells
ABHD2	Sustentacular_cells
ARHGEF26	Sustentacular_cells
ZNF397	Sustentacular_cells
KCTD1	Sustentacular_cells
ARHGEF11	Sustentacular_cells
CRYAB	Sustentacular_cells
CMYA5	Sustentacular_cells
COL20A1	Sustentacular_cells
PTPRZ1	Sustentacular_cells
CADM4	Sustentacular_cells
GAB1	Sustentacular_cells
ERBB2	Sustentacular_cells
NEGR1	Sustentacular_cells
CA13	Sustentacular_cells
BBS2	Sustentacular_cells
MAL2-AS1	Sustentacular_cells
NLGN4X	Sustentacular_cells
EHBP1	Sustentacular_cells
ST3GAL6	Sustentacular_cells
NAALADL2	Sustentacular_cells
SLC35F1	Sustentacular_cells
LINC02343	Sustentacular_cells
CTNNA3	Sustentacular_cells
SOX6	Sustentacular_cells
DYRK2	Sustentacular_cells
SOX9-AS1	Sustentacular_cells
GRB14	Sustentacular_cells
GPM6B	Sustentacular_cells
ANKRD10	Sustentacular_cells
HS3ST3A1	Sustentacular_cells
SEMA3C	Sustentacular_cells
ST3GAL6-AS1	Sustentacular_cells
CEP350	Sustentacular_cells
TFAP2A	Sustentacular_cells
ARRDC4	Sustentacular_cells
CIT	Sustentacular_cells
SORCS1	Sustentacular_cells
LINC00511	Sustentacular_cells
MICAL3	Sustentacular_cells
CADM2	Sustentacular_cells
GRIK2	Sustentacular_cells
MOSPD2	Sustentacular_cells
POLR3G	Sustentacular_cells
TRNP1	Sustentacular_cells
OTUD7B	Sustentacular_cells
ST6GALNAC2	Sustentacular_cells
HSPA12A	Sustentacular_cells
GULP1	Sustentacular_cells
TMEM178A	Sustentacular_cells
TMEM67	Sustentacular_cells
PANX1	Sustentacular_cells
PTPDC1	Sustentacular_cells
ITGB1BP1	Sustentacular_cells
FGF2	Sustentacular_cells
MFAP3L	Sustentacular_cells
DIP2C	Sustentacular_cells
AP1S2	Sustentacular_cells
KCTD3	Sustentacular_cells
CRADD	Sustentacular_cells
P2RY12	Sustentacular_cells
LRRC4C	Sustentacular_cells
BCHE	Sustentacular_cells
CRISPLD1	Sustentacular_cells
IGSF3	Sustentacular_cells
MAP4	Sustentacular_cells
SMC5	Sustentacular_cells
AGAP1	Sustentacular_cells
LMCD1-AS1	Sustentacular_cells
SOCS2-AS1	Sustentacular_cells
SNX22	Sustentacular_cells
WWC1	Sustentacular_cells
JHY	Sustentacular_cells
COL7A1	Sustentacular_cells
POLR2F	Sustentacular_cells
DPY19L3	Sustentacular_cells
DAG1	Sustentacular_cells
CHST9	Sustentacular_cells
SEMA3B	Sustentacular_cells
GINS3	Sustentacular_cells
ERBB3	Sustentacular_cells
ACSBG1	Sustentacular_cells
IL1RAPL2	Sustentacular_cells
PRIMA1	Sustentacular_cells
ZNF804B	Sustentacular_cells
COL28A1	Sustentacular_cells
SYNPR	Sustentacular_cells
KIRREL3	Sustentacular_cells
PLCH1	Sustentacular_cells
SOX10	Sustentacular_cells
INSC	Sustentacular_cells
NKAIN4	Sustentacular_cells
MYO16	Sustentacular_cells
SHISA9	Sustentacular_cells
INPP5F	Sustentacular_cells
ATP10B	Sustentacular_cells
LINC01116	Sustentacular_cells
GFRA1	Sustentacular_cells
SYT10	Sustentacular_cells
IGSF11	Sustentacular_cells
CHL1	Sustentacular_cells
ARHGAP39	Sustentacular_cells
LINC01239	Sustentacular_cells
PSD2	Sustentacular_cells
KCNMB4	Sustentacular_cells
GRIA1	Sustentacular_cells
IP6K3	Sustentacular_cells
WDR64	Sustentacular_cells
ASPH	Sustentacular_cells
MED14	Sustentacular_cells
CEP152	Sustentacular_cells
NPSR1-AS1	Sustentacular_cells
MMP17	Sustentacular_cells
XKR4	Sustentacular_cells
ST6GALNAC5	Sustentacular_cells
LRRC7	Sustentacular_cells
ALDH3A2	Sustentacular_cells
HDAC2-AS2	Sustentacular_cells
IMMP2L	Sustentacular_cells
ARHGEF26-AS1	Sustentacular_cells
SECISBP2L	Sustentacular_cells
CSMD2	Sustentacular_cells
MAPRE2	Sustentacular_cells
STS	Sustentacular_cells
TTLL4	Sustentacular_cells
AQP4-AS1	Sustentacular_cells
SLC7A14-AS1	Sustentacular_cells
LINC02018	Sustentacular_cells
MEGF9	Sustentacular_cells
LYST	Sustentacular_cells
ABCD3	Sustentacular_cells
TYRO3	Sustentacular_cells
PRRG4	Sustentacular_cells
SCN7A	Sustentacular_cells
FAM184B	Sustentacular_cells
LINC02240	Sustentacular_cells
DAGLA	Sustentacular_cells
CALN1	Sustentacular_cells
ALK	Sustentacular_cells
RTCA-AS1	Sustentacular_cells
SLC35D2	Sustentacular_cells
TRPM3	Sustentacular_cells
OLMALINC	Sustentacular_cells
TMCC2	Sustentacular_cells
USP54	Sustentacular_cells
CBFB	Sustentacular_cells
EHHADH	Sustentacular_cells
CCDC126	Sustentacular_cells
NMT1	Sustentacular_cells
FAM13B	Sustentacular_cells
ARFIP1	Sustentacular_cells
POU2F1	Sustentacular_cells

LINC00299	T_cells
RASAL3	T_cells
ARHGDIB	T_cells
CCDC88C	T_cells
LY75	T_cells
ERP27	T_cells
TMEM154	T_cells
EOMES	T_cells
EPHA1	T_cells
CTLA4	T_cells
TRGV10	T_cells
FCMR	T_cells
CLIC3	T_cells
NCR1	T_cells
TRAV3	T_cells
VSIG1	T_cells
SUSD3	T_cells
TRAT1	T_cells
CD40LG	T_cells
PDCD1	T_cells
CD244	T_cells
FCRL3	T_cells
LINC00944	T_cells
FASLG	T_cells
KCNA3	T_cells
UBASH3A	T_cells
ICOS	T_cells
FCRL6	T_cells
GPR174	T_cells
TRG-AS1	T_cells
LCK	T_cells
IL2RB	T_cells
KLRC3	T_cells
SH2D3A	T_cells
KLRC1	T_cells
SHISAL2A	T_cells
LINC02325	T_cells
RUNX3	T_cells
TRBV5-1	T_cells
P2RY10	T_cells
PCED1B-AS1	T_cells
LINC01871	T_cells
DPP4	T_cells
NLRC3	T_cells
IL32	T_cells
S1PR4	T_cells
LGR6	T_cells
TNIP3	T_cells
ZBP1	T_cells
RNF125	T_cells
SLAMF1	T_cells
IL18RAP	T_cells
ZNF683	T_cells
SH2D1A	T_cells
GML	T_cells
ICAM3	T_cells
KLRC4	T_cells
SELL	T_cells
TESPA1	T_cells
PTGDR	T_cells
SIRPG	T_cells
TBX21	T_cells
CXCR3	T_cells
ZAP70	T_cells
SIT1	T_cells
CCND3	T_cells
ANKRD55	T_cells
GZMK	T_cells
STAP1	T_cells
TRGC2	T_cells
KLRC2	T_cells
SLAMF7	T_cells
PTPN7	T_cells
APOBEC3H	T_cells
TRABD2A	T_cells
LAX1	T_cells
IFNG-AS1	T_cells
LINC00649	T_cells
CD70	T_cells
SLAMF6	T_cells
CD7	T_cells
CD6	T_cells
TNFRSF9	T_cells
DTHD1	T_cells
GZMB	T_cells
FGFBP2	T_cells
ITGB2-AS1	T_cells
CD48	T_cells
IL2RG	T_cells
ADGRG5	T_cells
PRF1	T_cells
SH2D1B	T_cells
ALOX5AP	T_cells
GBP5	T_cells
LINC00426	T_cells
SP140	T_cells
PTPRC	T_cells
LINC02446	T_cells
TRAC	T_cells
CX3CR1	T_cells
GZMH	T_cells
GCHFR	T_cells
TRBC2	T_cells
TRBC1	T_cells
RAC2	T_cells
CD69	T_cells
SLFN12L	T_cells
RHOH	T_cells
PTPRCAP	T_cells
CYTIP	T_cells
CD3E	T_cells
CTSW	T_cells
BTLA	T_cells
LINC00402	T_cells
CYFIP2	T_cells
RGS14	T_cells
CD3D	T_cells
TTC16	T_cells
TBC1D10C	T_cells
CXCR6	T_cells
LY9	T_cells
SH2D2A	T_cells
IKZF1	T_cells
KLRF1	T_cells
IL21R	T_cells
ANXA2R	T_cells
MZB1	T_cells
KLRK1	T_cells
NUP210	T_cells
GZMA	T_cells
CD8B	T_cells
GPA33	T_cells
ARHGEF1	T_cells
TIGIT	T_cells
DENND2D	T_cells
GRAP2	T_cells
GFI1	T_cells
CNOT6L	T_cells
ITK	T_cells
SELPLG	T_cells
BIN2	T_cells
CDC42SE2	T_cells
ITGB7	T_cells
PLAAT4	T_cells
IKZF3	T_cells
LINC02100	T_cells
MAP4K1	T_cells
SLC14A1	T_cells
ZNF101	T_cells
CASP8	T_cells
LTB	T_cells
PARP8	T_cells
ITGAL	T_cells
THEMIS	T_cells
CCR7	T_cells
PTPN22	T_cells
ANO9	T_cells
SPN	T_cells
MCOLN2	T_cells
CD247	T_cells
CD2	T_cells
TMIGD2	T_cells
SKAP1-AS1	T_cells
SKAP1	T_cells
LINC00243	T_cells
A2M-AS1	T_cells
PYHIN1	T_cells
CST7	T_cells
TC2N	T_cells
NKG7	T_cells
SOCS1	T_cells
IL7R	T_cells
STK26	T_cells
SYTL3	T_cells
APOBEC3D	T_cells
IRF1	T_cells
DUSP2	T_cells
TENT5C	T_cells
LINC02694	T_cells
CD96	T_cells
SEMA4D	T_cells
CRYBG2	T_cells
CD52	T_cells
AQP3	T_cells
BLK	T_cells
CARD11	T_cells
HSH2D	T_cells
SLA2	T_cells
TRAF3IP3	T_cells
CCL5	T_cells
HCST	T_cells
ZNF831	T_cells
CORO1A	T_cells
GNLY	T_cells
LSP1	T_cells
KLRG1	T_cells
PRKCQ-AS1	T_cells
CCR6	T_cells
SAMD3	T_cells
PDCD4	T_cells
CD27	T_cells
PBX4	T_cells
GPR171	T_cells
CRYBG1	T_cells
TNFAIP8	T_cells
CLEC2D	T_cells
PLAC8	T_cells
CD226	T_cells
TSPAN32	T_cells
IL12RB2	T_cells
PDE7A	T_cells
CD5	T_cells
ABCD2	T_cells
SEPTIN1	T_cells
MYBL1	T_cells
LINC00861	T_cells
LINC01934	T_cells
ACAP1	T_cells
RASGRP1	T_cells
CD8A	T_cells
CD3G	T_cells
PRKCQ	T_cells
OXNAD1	T_cells
BTN3A1	T_cells
KLRB1	T_cells
SCML4	T_cells
LINC02320	T_cells
GZMM	T_cells
RBL2	T_cells
APOBEC3G	T_cells
BCL11B	T_cells
P2RX5	T_cells
LEPROTL1	T_cells
LAT	T_cells
SUN2	T_cells
ITGAE	T_cells
HELB	T_cells
SMCHD1	T_cells
PLCXD2	T_cells
TBC1D31	T_cells
SYTL1	T_cells
NCAPH	T_cells
SFI1	T_cells
ERN1	T_cells
PPP2R5C	T_cells
EML4	T_cells
GPD1L	T_cells
MPP7	T_cells
RIPOR2	T_cells
LIME1	T_cells
PIM2	T_cells
TSEN54	T_cells
MEI1	T_cells
DBF4	T_cells

LCP2	Macrophages	CLEC7A	Macrophages	MB21D2	Macrophages
TNFAIP2	Macrophages	SLCO2B1	Macrophages	HPSE	Macrophages
DAB2	Macrophages	ADGRE2	Macrophages	ABCC3	Macrophages
ADPRH	Macrophages	CIITA	Macrophages	CASS4	Macrophages
PLEKHO2	Macrophages	MRC1	Macrophages	SRGAP2C	Macrophages
CMTM7	Macrophages	FCHO2	Macrophages	CD163	Macrophages
STAB1	Macrophages	KCNJ5	Macrophages	ALOX5	Macrophages
SRGN	Macrophages	METRNL	Macrophages	DAPK1-IT1	Macrophages
CSF2RB	Macrophages	CD300A	Macrophages	SAMSN1	Macrophages
RHBDF2	Macrophages	LRRC25	Macrophages	MYO9B	Macrophages
RNF144B	Macrophages	RAB7B	Macrophages	TFCP2L1	Macrophages
KLHL6	Macrophages	PDK4	Macrophages	MAFB	Macrophages
SH2B3	Macrophages	FGD2	Macrophages	SLC39A12	Macrophages
HCLS1	Macrophages	WNT5A	Macrophages	MS4A6E	Macrophages
TNFRSF1B	Macrophages	FCGR2A	Macrophages	HOMER3	Macrophages
PIK3R6	Macrophages	IL10	Macrophages	FPR1	Macrophages
TMEM37	Macrophages	LILRB4	Macrophages	TMEM170B	Macrophages
LILRB1	Macrophages	CD300E	Macrophages	ST14	Macrophages
SGK1	Macrophages	TLR4	Macrophages	RNF19B	Macrophages
STX11	Macrophages	CLDN7	Macrophages	OLR1	Macrophages
SIRPB2	Macrophages	TFEC	Macrophages	CD74	Macrophages
SAT1	Macrophages	RBM47	Macrophages	IRF8	Macrophages
SECTM1	Macrophages	OSM	Macrophages	CCDC170	Macrophages
LINC02642	Macrophages	HLA-DMB	Macrophages	TBXAS1	Macrophages
LGALS9	Macrophages	CSF3R	Macrophages	CLN8	Macrophages
C9	Macrophages	FGL2	Macrophages	BTK	Macrophages
BNC2	Macrophages	LINC01094	Macrophages	ZMYND15	Macrophages
PLIN2	Macrophages	HLA-DOA	Macrophages	DTNB	Macrophages
SH3BP5	Macrophages	EMILIN2	Macrophages	P2RY13	Macrophages
PLAU	Macrophages	LNCAROD	Macrophages	MPEG1	Macrophages
GADD45G	Macrophages	SYK	Macrophages	FAM20A	Macrophages
RAB20	Macrophages	NCF4	Macrophages	GABRB2	Macrophages
SLC9A9	Macrophages	FPR3	Macrophages	RYR1	Macrophages
GPR85	Macrophages	FCGR2B	Macrophages	CMTM6	Macrophages
GNA15	Macrophages	FCGR1A	Macrophages	CCL3	Macrophages
HBEGF	Macrophages	CSF1R	Macrophages	GPR183	Macrophages
MAP3K8	Macrophages	RGS1	Macrophages	CD83	Macrophages
TLR5	Macrophages	RNASE6	Macrophages	CR1	Macrophages
SH3TC1	Macrophages	TPK1	Macrophages	PID1	Macrophages
PARVG	Macrophages	PFKFB3	Macrophages	HLA-DRA	Macrophages
MSR1	Macrophages	TREM2	Macrophages	C3	Macrophages
LINC01480	Macrophages	LAIR1	Macrophages	CAPZB	Macrophages
SLC17A9	Macrophages	CD80	Macrophages	GSAP	Macrophages
KYNU	Macrophages	DIRC3	Macrophages	PLSCR1	Macrophages
ARL11	Macrophages	SPATC1	Macrophages	NFKBIE	Macrophages
SHB	Macrophages	CD209	Macrophages	SLC38A6	Macrophages
HMGA1P4	Macrophages	VSIG4	Macrophages	GK	Macrophages
B4GALT1	Macrophages	SRGAP2	Macrophages	HLA-DQA1	Macrophages
ZEB2-AS1	Macrophages	HCK	Macrophages	LILRB5	Macrophages
SIGLEC7	Macrophages	LILRB2	Macrophages	GPR137B	Macrophages
PLAUR	Macrophages	LY86	Macrophages	ME2	Macrophages
THEMIS2	Macrophages	RASL10A	Macrophages	FCGRT	Macrophages
TMEM144	Macrophages	SLC29A3	Macrophages	DPYD	Macrophages
LINC01678	Macrophages	EYA2	Macrophages	IL1RN	Macrophages
TRPM2	Macrophages	TLR2	Macrophages	CHIT1	Macrophages
SLC24A4	Macrophages	RUBCNL	Macrophages	F13A1	Macrophages
LAPTM5	Macrophages	PTAFR	Macrophages	CCL4L2	Macrophages
CPVL	Macrophages	GAB2	Macrophages	C9orf72	Macrophages
SIGLEC9	Macrophages	SLAMF8	Macrophages	IFNGR1	Macrophages
ETV6	Macrophages	RHOT1	Macrophages	FBP1	Macrophages
CD86	Macrophages	ITGAX	Macrophages	SLC37A2	Macrophages
VENTX	Macrophages	IL2RA	Macrophages	LINC00877	Macrophages
ZNF710	Macrophages	CD300LF	Macrophages	SLC7A7	Macrophages
LY96	Macrophages	LILRB3	Macrophages	PLTP	Macrophages
LINC02391	Macrophages	GNB4	Macrophages	DAPK1	Macrophages
SYT6	Macrophages	P2RY6	Macrophages	MGAT1	Macrophages
CMKLR1	Macrophages	FCER1G	Macrophages	HLA-DRB1	Macrophages
PLEK	Macrophages	UBXN2B	Macrophages	TMEM268	Macrophages
CD300LB	Macrophages	FCHSD2	Macrophages	M1AP	Macrophages
RGS18	Macrophages	LST1	Macrophages	VMO1	Macrophages
EBI3	Macrophages	SLC8B1	Macrophages	CTSB	Macrophages
RILPL2	Macrophages	FOLR2	Macrophages	NFAM1	Macrophages
LPAR6	Macrophages	ITGAM	Macrophages	GRK3	Macrophages
TM6SF1	Macrophages	DMXL2	Macrophages	TRPS1	Macrophages
TGFBI	Macrophages	ARHGAP8	Macrophages	TYROBP	Macrophages
ADGRE1	Macrophages	RREB1	Macrophages	NCF2	Macrophages
CYTH4	Macrophages	CALHM6	Macrophages	FHIT	Macrophages
PADI2	Macrophages	LINC00996	Macrophages	IFITM10	Macrophages
LILRA5	Macrophages	S100Z	Macrophages	DAGLB	Macrophages
MNDA	Macrophages	TGFA	Macrophages	BMP2K	Macrophages
TMEM26	Macrophages	MS4A4A	Macrophages	ATP8B4	Macrophages
CDCP1	Macrophages	CTSZ	Macrophages	BCAT1	Macrophages
IER3	Macrophages	KMO	Macrophages	PLA2G7	Macrophages
CASP5	Macrophages	FCN1	Macrophages	NEU3	Macrophages
MILR1	Macrophages	PRSS36	Macrophages	IL6R	Macrophages
WDFY4	Macrophages	C3AR1	Macrophages	PDK3	Macrophages
ZNF267	Macrophages	ADAMTSL4	Macrophages	PRKCD	Macrophages
MS4A7	Macrophages	PLBD1	Macrophages	TLR6	Macrophages
SIGLEC1	Macrophages	IL1R2	Macrophages	SNX29	Macrophages
NABP1	Macrophages	HLA-DPB1	Macrophages	CABLES1	Macrophages
UNC93B1	Macrophages	FGD4	Macrophages	DOK3	Macrophages
SERPINA1	Macrophages	CXCL3	Macrophages	DHX34	Macrophages
LILRA6	Macrophages	AMPD3	Macrophages	MS4A4E	Macrophages
RAB31	Macrophages	MEF2A	Macrophages	IL18	Macrophages
MS4A14	Macrophages	SLC11A1	Macrophages	HNMT	Macrophages
CLEC4E	Macrophages	ATG16L2	Macrophages	LGMN	Macrophages
MMP2-AS1	Macrophages	SLC11A2	Macrophages	TOM1	Macrophages
TREM1	Macrophages	KCNE1	Macrophages	CD33	Macrophages
CSF2RA	Macrophages	MS4A6A	Macrophages	RELT	Macrophages
LINC02712	Macrophages	MANBA	Macrophages	USP4	Macrophages
SIGLEC10	Macrophages	LINC01645	Macrophages	PDGFC	Macrophages
LINC02207	Macrophages	CRHBP	Macrophages	TMEM150B	Macrophages
MEFV	Macrophages	NLRP3	Macrophages	PLBD2	Macrophages
IL1B	Macrophages	SFMBT2	Macrophages	MKNK1	Macrophages

CD14	Macrophag	SNX6	Macrophages	PSAP	Macrophages
SPTLC3	Macrophag	SIRPA	Macrophages	UBE2W	Macrophages
HTR7	Macrophag	CCDC26	Macrophages	PPT1	Macrophages
HLA-DPA1	Macrophag	SLC18B1	Macrophages	SRBD1	Macrophages
GAA	Macrophag	RAP2B	Macrophages	COP1	Macrophages
TYMP	Macrophag	HLA-DQB1	Macrophages	RCOR1	Macrophages
LINC01482	Macrophag	STX4	Macrophages	COQ2	Macrophages
GPR34	Macrophag	SIPA1L1	Macrophages	PIAS1	Macrophages
TNFSF13	Macrophag	DSE	Macrophages	SGK3	Macrophages
CXCL16	Macrophag	C1QA	Macrophages	ZNF277	Macrophages
C5AR1	Macrophag	MYO5A	Macrophages	SLC25A19	Macrophages
RNF114	Macrophag	CTSL	Macrophages	YTHDF3	Macrophages
IGSF21	Macrophag	SLC40A1	Macrophages	MAX	Macrophages
VPS35	Macrophag	MACC1	Macrophages	CHKA	Macrophages
TMEM106A	Macrophag	RNASET2	Macrophages	PACC1	Macrophages
IFI30	Macrophag	TBC1D22A	Macrophages	VAC14	Macrophages
FKBP15	Macrophag	APAF1	Macrophages	ABHD12	Macrophages
NCEH1	Macrophag	HK3	Macrophages	NT5C2	Macrophages
CCDC88A	Macrophag	MPHOSPH6	Macrophages	IRAK1	Macrophages
FTCDNL1	Macrophag	OTUD1	Macrophages	TENT2	Macrophages
SRGAP1	Macrophag	RNF13	Macrophages	ME1	Macrophages
HEXA	Macrophag	INSIG1	Macrophages	APPL2	Macrophages
PLD2	Macrophag	LACC1	Macrophages	BRCA2	Macrophages
GRB2	Macrophag	DSC2	Macrophages	VPS8	Macrophages
ADAP2	Macrophag	SNX2	Macrophages	DENND1A	Macrophages
LYN	Macrophag	DOP1B	Macrophages	ZDHHC7	Macrophages
LITAF	Macrophag	MEIKIN	Macrophages	BNIP3L	Macrophages
GNS	Macrophag	SLC1A3	Macrophages	SCLT1	Macrophages
LINC02245	Macrophag	CNDP2	Macrophages	TMEM51	Macrophages
ELL	Macrophag	NINJ1	Macrophages	VPS26A	Macrophages
AIF1	Macrophag	HERPUD1	Macrophages	TIGAR	Macrophages
STX6	Macrophag	FMNL2	Macrophages	STON2	Macrophages
GCA	Macrophag	CREG1	Macrophages	GLB1	Macrophages
RASGEF1B	Macrophag	PLEKHO1	Macrophages	SPG21	Macrophages
MITF	Macrophag	HPS5	Macrophages	ARID3A	Macrophages
IDH1	Macrophag	SDCBP	Macrophages	SFXN2	Macrophages
TBC1D12	Macrophag	CEP170	Macrophages	PGD	Macrophages
TTYH2	Macrophag	DRAM2	Macrophages	CTTNBP2	Macrophages
DENND4B	Macrophag	MCOLN1	Macrophages	ANKH	Macrophages
ABCA1	Macrophag	OTULINL	Macrophages	ZCCHC2	Macrophages
SLC16A10	Macrophag	HPS3	Macrophages	LDLRAD4	Macrophages
LAP3	Macrophag	WBP1L	Macrophages	NCOA4	Macrophages
SDCCAG8	Macrophag	LINC02196	Macrophages	SNX24	Macrophages
CARD9	Macrophag	DISP1	Macrophages	TMEM70	Macrophages
NFKBID	Macrophag	ACER3	Macrophages	PLA2G15	Macrophages
AP1B1	Macrophag	SPIDR	Macrophages	ATF6	Macrophages
TBC1D14	Macrophag	MAN2B1	Macrophages		
NANS	Macrophag	DNM2	Macrophages		
HAUS8	Macrophag	AP2A2	Macrophages		
SLC38A7	Macrophag	ABR	Macrophages		
BLNK	Macrophag	MXD1	Macrophages		
CRYL1	Macrophag	SEMA4A	Macrophages		
LINC01637	Macrophag	MIOS	Macrophages		
SCO2	Macrophag	HSPBAP1	Macrophages		
NAMPT	Macrophag	NAGK	Macrophages		
SPI1	Macrophag	SLC31A2	Macrophages		
ACSL1	Macrophag	PI4K2A	Macrophages		
C1orf162	Macrophag	IRF5	Macrophages		
RNF149	Macrophag	SCIMP	Macrophages		
MFSD1	Macrophag	FTH1	Macrophages		
NPL	Macrophag	CNEP1R1	Macrophages		
GLUL	Macrophag	TANGO2	Macrophages		
MERTK	Macrophag	SLC49A4	Macrophages		
TLR1	Macrophag	OSBPL11	Macrophages		
HAVCR2	Macrophag	SNX8	Macrophages		
HMOX1	Macrophag	PILRA	Macrophages		
CD72	Macrophag	PTPN18	Macrophages		
ADAM28	Macrophag	TPCN1	Macrophages		
SELENOP	Macrophag	TNFRSF21	Macrophages		
LPAR5	Macrophag	TAOK3	Macrophages		
PIK3AP1	Macrophag	GNA13	Macrophages		
FUCA1	Macrophag	PIGX	Macrophages		
C1QB	Macrophag	DCP2	Macrophages		
HEATR3	Macrophag	CREM	Macrophages		
SLC43A2	Macrophag	CEPT1	Macrophages		
STX7	Macrophag	ATP6V1C1	Macrophages		
TFRC	Macrophag	PAPOLG	Macrophages		
PPARD	Macrophag	ASAH1	Macrophages		
SDS	Macrophag	MICOS10	Macrophages		
FMN1	Macrophag	CEBPD	Macrophages		
ARRB2	Macrophag	RBPJ	Macrophages		
CYBB	Macrophag	HSPA6	Macrophages		
GRAMD4	Macrophag	SNX30	Macrophages		
RNF130	Macrophag	SLC8A1	Macrophages		
REL	Macrophag	OSCAR	Macrophages		
MPP1	Macrophag	GNAI3	Macrophages		
TTYH3	Macrophag	DNAJC5	Macrophages		
MALT1	Macrophag	SOD2	Macrophages		
EAF2	Macrophag	NAIP	Macrophages		
CD163L1	Macrophag	SDSL	Macrophages		
ITSN1	Macrophag	RGS2	Macrophages		
LINC01374	Macrophag	MOB1B	Macrophages		
WDR91	Macrophag	TET3	Macrophages		
PLB1	Macrophag	CHMP1B	Macrophages		
TBC1D2	Macrophag	SMS	Macrophages		
NLRC4	Macrophag	XRCC4	Macrophages		
SLC7A8	Macrophag	SRGAP2B	Macrophages		
ATG7	Macrophag	ARAP1	Macrophages		
ADPGK	Macrophag	KCNK13	Macrophages		
HLA-DMA	Macrophag	GGA1	Macrophages		
LAT2	Macrophag	AKR1A1	Macrophages		
SOAT1	Macrophag	MFSD12	Macrophages		
C1QC	Macrophag	HIF1A	Macrophages		
SLC15A2	Macrophag	HEXB	Macrophages		

Supplementary Table 3 *Significant genes that are differentially expressed between chromaffin cells from tumours with a high SOX2+ cell proportion compared to chromaffin cells from tumours with a lower/absent SOX2+ cell proportion.*

	logFC	AveExpr	t	P.Value	adj.P.Val	B	genes	comparison
assignmentsChromaffin_cells.Chrom_SOX2_statusHigh.PIGT	2.46814495	4.55217712	12.2437418	1.82E-19	2.92E-15	33.7794796	PIGT	assignmentsChromaffin_cells.Chrom_SOX2_statusHigh
assignmentsChromaffin_cells.Chrom_SOX2_statusHigh.IMP3	3.04395708	3.51075852	10.5409144	2.19E-16	1.20E-12	26.8406933	IMP3	assignmentsChromaffin_cells.Chrom_SOX2_statusHigh
assignmentsChromaffin_cells.Chrom_SOX2_statusHigh.PHACTR1	-5.3216607	7.27848729	-10.473303	2.99E-16	1.20E-12	26.5311139	PHACTR1	assignmentsChromaffin_cells.Chrom_SOX2_statusHigh
assignmentsChromaffin_cells.Chrom_SOX2_statusHigh.LINC00923	5.73549873	1.80205695	10.5068317	2.55E-16	1.20E-12	26.3499162	LINC00923	assignmentsChromaffin_cells.Chrom_SOX2_statusHigh
assignmentsChromaffin_cells.Chrom_SOX2_statusHigh.LGALS3BP	4.75362962	4.16012162	10.1645258	1.12E-15	3.34E-12	25.3077669	LGALS3BP	assignmentsChromaffin_cells.Chrom_SOX2_statusHigh
assignmentsChromaffin_cells.Chrom_SOX2_statusHigh.SPR	3.7804454	1.26125923	10.132374	1.25E-15	3.34E-12	25.0380881	SPR	assignmentsChromaffin_cells.Chrom_SOX2_statusHigh
assignmentsChromaffin_cells.Chrom_SOX2_statusHigh.STMN1	4.80190878	3.74725907	10.0634034	1.70E-15	3.90E-12	24.8353281	STMN1	assignmentsChromaffin_cells.Chrom_SOX2_statusHigh
assignmentsChromaffin_cells.Chrom_SOX2_statusHigh.SOX2	5.18070135	-0.0219306	9.88831039	3.56E-15	7.15E-12	23.5987293	SOX2	assignmentsChromaffin_cells.Chrom_SOX2_statusHigh
assignmentsChromaffin_cells.Chrom_SOX2_statusHigh.TM9SF1	2.00643564	3.70249828	9.63186908	1.08E-14	1.69E-11	23.0797897	TM9SF1	assignmentsChromaffin_cells.Chrom_SOX2_statusHigh
assignmentsChromaffin_cells.Chrom_SOX2_statusHigh.HINT2	2.93576429	2.81311436	9.61467789	1.16E-14	1.69E-11	22.993489	HINT2	assignmentsChromaffin_cells.Chrom_SOX2_statusHigh
assignmentsChromaffin_cells.Chrom_SOX2_statusHigh.CFAP58-DT	5.85943194	-0.093026	9.75287316	6.39E-15	1.14E-11	22.8519941	CFAP58-DT	assignmentsChromaffin_cells.Chrom_SOX2_statusHigh
assignmentsChromaffin_cells.Chrom_SOX2_statusHigh.PPDPF	3.9193404	4.86667386	9.51547319	1.80E-14	2.41E-11	22.6028013	PPDPF	assignmentsChromaffin_cells.Chrom_SOX2_statusHigh
assignmentsChromaffin_cells.Chrom_SOX2_statusHigh.CHMP5	2.30623284	5.0161659	9.4737838	2.13E-14	2.63E-11	22.43633	CHMP5	assignmentsChromaffin_cells.Chrom_SOX2_statusHigh
assignmentsChromaffin_cells.Chrom_SOX2_statusHigh.EMC7	2.23308193	4.79333498	9.41181209	2.79E-14	3.19E-11	22.1740895	EMC7	assignmentsChromaffin_cells.Chrom_SOX2_statusHigh
assignmentsChromaffin_cells.Chrom_SOX2_statusHigh.CDK4	2.29343292	4.00591962	9.37598839	3.25E-14	3.48E-11	22.0174888	CDK4	assignmentsChromaffin_cells.Chrom_SOX2_statusHigh
assignmentsChromaffin_cells.Chrom_SOX2_statusHigh.BAG1	2.83780013	4.5453358	9.34288617	3.76E-14	3.77E-11	21.8815418	BAG1	assignmentsChromaffin_cells.Chrom_SOX2_statusHigh
assignmentsChromaffin_cells.Chrom_SOX2_statusHigh.LMAN2	2.14966327	5.1059706	9.31732963	4.20E-14	3.96E-11	21.7736293	LMAN2	assignmentsChromaffin_cells.Chrom_SOX2_statusHigh
assignmentsChromaffin_cells.Chrom_SOX2_statusHigh.SDHAF1	3.09764959	2.09352506	9.19740695	7.06E-14	6.11E-11	21.2219804	SDHAF1	assignmentsChromaffin_cells.Chrom_SOX2_statusHigh
assignmentsChromaffin_cells.Chrom_SOX2_statusHigh.RNF6	1.80641043	3.83951747	9.17975888	7.62E-14	6.11E-11	21.1760146	RNF6	assignmentsChromaffin_cells.Chrom_SOX2_statusHigh
assignmentsChromaffin_cells.Chrom_SOX2_statusHigh.ABCB4	4.57906868	1.87090299	9.18533725	7.44E-14	6.11E-11	21.0174624	ABCB4	assignmentsChromaffin_cells.Chrom_SOX2_statusHigh
assignmentsChromaffin_cells.Chrom_SOX2_statusHigh.CISD3	2.4028073	3.3874973	9.11511196	1.01E-13	7.71E-11	20.9012207	CISD3	assignmentsChromaffin_cells.Chrom_SOX2_statusHigh
assignmentsChromaffin_cells.Chrom_SOX2_statusHigh.SDF2	1.81466677	4.54297192	9.08366918	1.16E-13	7.80E-11	20.7829458	SDF2	assignmentsChromaffin_cells.Chrom_SOX2_statusHigh
assignmentsChromaffin_cells.Chrom_SOX2_statusHigh.ERP29	2.5312584	5.48226516	9.08042954	1.17E-13	7.80E-11	20.7694934	ERP29	assignmentsChromaffin_cells.Chrom_SOX2_statusHigh
assignmentsChromaffin_cells.Chrom_SOX2_statusHigh.PLP2	2.96060441	3.92419925	9.07210397	1.22E-13	7.80E-11	20.7318418	PLP2	assignmentsChromaffin_cells.Chrom_SOX2_statusHigh
assignmentsChromaffin_cells.Chrom_SOX2_statusHigh.PTGER1	4.56269047	0.1792403	9.08861057	1.13E-13	7.80E-11	20.5424323	PTGER1	assignmentsChromaffin_cells.Chrom_SOX2_statusHigh
assignmentsChromaffin_cells.Chrom_SOX2_statusHigh.BCAP31	2.7348513	5.08367207	9.02632025	1.48E-13	9.16E-11	20.5415504	BCAP31	assignmentsChromaffin_cells.Chrom_SOX2_statusHigh
assignmentsChromaffin_cells.Chrom_SOX2_statusHigh.NDUFA2	3.31468159	4.04488963	9.0051103	1.63E-13	9.37E-11	20.4486516	NDUFA2	assignmentsChromaffin_cells.Chrom_SOX2_statusHigh
assignmentsChromaffin_cells.Chrom_SOX2_statusHigh.SMIM15	2.4687685	3.4416465	9.00402305	1.64E-13	9.37E-11	20.4376112	SMIM15	assignmentsChromaffin_cells.Chrom_SOX2_statusHigh
assignmentsChromaffin_cells.Chrom_SOX2_statusHigh.NDUFB9	2.61947299	5.28334518	8.97810178	1.83E-13	1.01E-10	20.3367536	NDUFB9	assignmentsChromaffin_cells.Chrom_SOX2_statusHigh
assignmentsChromaffin_cells.Chrom_SOX2_statusHigh.COA3	3.03819465	2.99472069	8.93619598	2.20E-13	1.17E-10	20.145623	COA3	assignmentsChromaffin_cells.Chrom_SOX2_statusHigh
assignmentsChromaffin_cells.Chrom_SOX2_statusHigh.ANAPC13	2.76562274	3.34203308	8.90617362	2.50E-13	1.29E-10	20.0243403	ANAPC13	assignmentsChromaffin_cells.Chrom_SOX2_statusHigh
assignmentsChromaffin_cells.Chrom_SOX2_statusHigh.GET3	2.57528482	3.43077144	8.85113412	3.18E-13	1.59E-10	19.7886467	GET3	assignmentsChromaffin_cells.Chrom_SOX2_statusHigh
assignmentsChromaffin_cells.Chrom_SOX2_statusHigh.ATP6V1G1	2.86143703	5.94653598	8.84054766	3.33E-13	1.62E-10	19.7463509	ATP6V1G1	assignmentsChromaffin_cells.Chrom_SOX2_statusHigh
assignmentsChromaffin_cells.Chrom_SOX2_statusHigh.DYM	-1.2976458	7.99596239	-8.8120737	3.77E-13	1.78E-10	19.6228743	DYM	assignmentsChromaffin_cells.Chrom_SOX2_statusHigh
assignmentsChromaffin_cells.Chrom_SOX2_statusHigh.MRPL34	3.18445497	4.03435529	8.79819735	4.00E-13	1.83E-10	19.5731231	MRPL34	assignmentsChromaffin_cells.Chrom_SOX2_statusHigh
assignmentsChromaffin_cells.Chrom_SOX2_statusHigh.ANAPC11	3.1330984	4.31625826	8.77943045	4.34E-13	1.94E-10	19.4936353	ANAPC11	assignmentsChromaffin_cells.Chrom_SOX2_statusHigh
assignmentsChromaffin_cells.Chrom_SOX2_statusHigh.SUPT4H1	3.00835239	3.20848129	8.7675482	4.58E-13	1.98E-10	19.4343979	SUPT4H1	assignmentsChromaffin_cells.Chrom_SOX2_statusHigh
assignmentsChromaffin_cells.Chrom_SOX2_statusHigh.CDK5	3.19472331	1.46129909	8.75819901	4.77E-13	2.01E-10	19.3468495	CDK5	assignmentsChromaffin_cells.Chrom_SOX2_statusHigh
assignmentsChromaffin_cells.Chrom_SOX2_statusHigh.MRPL58	1.55238562	3.43147121	8.72951994	5.40E-13	2.22E-10	19.2752258	MRPL58	assignmentsChromaffin_cells.Chrom_SOX2_statusHigh
assignmentsChromaffin_cells.Chrom_SOX2_statusHigh.G6PC3	2.13338177	3.2773393	8.71720211	5.70E-13	2.23E-10	19.2229602	G6PC3	assignmentsChromaffin_cells.Chrom_SOX2_statusHigh
assignmentsChromaffin_cells.Chrom_SOX2_statusHigh.AURKAIP1	3.17117781	4.05443094	8.69855142	6.18E-13	2.33E-10	19.1495741	AURKAIP1	assignmentsChromaffin_cells.Chrom_SOX2_statusHigh
assignmentsChromaffin_cells.Chrom_SOX2_statusHigh.DUT	1.92203509	5.72663024	8.69181985	6.36E-13	2.33E-10	19.1150534	DUT	assignmentsChromaffin_cells.Chrom_SOX2_statusHigh
assignmentsChromaffin_cells.Chrom_SOX2_statusHigh.RAD23A	2.17188534	5.36117339	8.69024422	6.41E-13	2.33E-10	19.1124092	RAD23A	assignmentsChromaffin_cells.Chrom_SOX2_statusHigh
assignmentsChromaffin_cells.Chrom_SOX2_statusHigh.AK1	2.52344816	3.23810131	8.66854536	7.04E-13	2.51E-10	19.0189388	AK1	assignmentsChromaffin_cells.Chrom_SOX2_statusHigh
assignmentsChromaffin_cells.Chrom_SOX2_statusHigh.EMC4	2.70064662	4.12336474	8.65886417	7.34E-13	2.56E-10	18.9820275	EMC4	assignmentsChromaffin_cells.Chrom_SOX2_statusHigh
assignmentsChromaffin_cells.Chrom_SOX2_statusHigh.WNT3	4.81321921	0.44615482	8.71672481	5.71E-13	2.23E-10	18.9593237	WNT3	assignmentsChromaffin_cells.Chrom_SOX2_statusHigh
assignmentsChromaffin_cells.Chrom_SOX2_statusHigh.TMEM9	2.26237145	3.63457736	8.64507493	7.80E-13	2.66E-10	18.9234813	TMEM9	assignmentsChromaffin_cells.Chrom_SOX2_statusHigh
assignmentsChromaffin_cells.Chrom_SOX2_statusHigh.NDUFA7	2.88448296	4.55076127	8.6205248	8.68E-13	2.90E-10	18.8175115	NDUFA7	assignmentsChromaffin_cells.Chrom_SOX2_statusHigh
assignmentsChromaffin_cells.Chrom_SOX2_statusHigh.NGRN	2.37690541	5.0828287	8.61360927	8.94E-13	2.93E-10	18.7849991	NGRN	assignmentsChromaffin_cells.Chrom_SOX2_statusHigh
assignmentsChromaffin_cells.Chrom_SOX2_statusHigh.TMED4	2.28540633	5.65741142	8.58053319	1.03E-12	3.31E-10	18.6386088	TMED4	assignmentsChromaffin_cells.Chrom_SOX2_statusHigh
assignmentsChromaffin_cells.Chrom_SOX2_statusHigh.LENG1	2.27403293	2.90416655	8.54392644	1.21E-12	3.73E-10	18.4755659	LENG1	assignmentsChromaffin_cells.Chrom_SOX2_statusHigh
assignmentsChromaffin_cells.Chrom_SOX2_statusHigh.PSENEN	3.69438487	3.10564728	8.54135304	1.23E-12	3.73E-10	18.4740207	PSENEN	assignmentsChromaffin_cells.Chrom_SOX2_statusHigh
assignmentsChromaffin_cells.Chrom_SOX2_statusHigh.STBD1	3.21579136	1.62604362	8.52148554	1.34E-12	3.97E-10	18.3779455	STBD1	assignmentsChromaffin_cells.Chrom_SOX2_statusHigh
assignmentsChromaffin_cells.Chrom_SOX2_statusHigh.CENPX	2.69661645	2.93792247	8.49998242	1.47E-12	4.28E-10	18.2992045	CENPX	assignmentsChromaffin_cells.Chrom_SOX2_statusHigh
assignmentsChromaffin_cells.Chrom_SOX2_statusHigh.GNG11	4.75358142	4.39102494	8.49302867	1.54E-12	4.39E-10	18.2578443	GNG11	assignmentsChromaffin_cells.Chrom_SOX2_statusHigh
assignmentsChromaffin_cells.Chrom_SOX2_statusHigh.TMED1	3.297538	2.30015124	8.47931399	1.61E-12	4.39E-10	18.2043305	TMED1	assignmentsChromaffin_cells.Chrom_SOX2_statusHigh
assignmentsChromaffin_cells.Chrom_SOX2_statusHigh.MIEN1	3.25386217	3.34557392	8.47435252	1.64E-12	4.39E-10	18.1975313	MIEN1	assignmentsChromaffin_cells.Chrom_SOX2_statusHigh
assignmentsChromaffin_cells.Chrom_SOX2_statusHigh.PEBP1	3.21291957	6.02479523	8.47471262	1.65E-12	4.39E-10	18.1739351	PEBP1	assignmentsChromaffin_cells.Chrom_SOX2_statusHigh
assignmentsChromaffin_cells.Chrom_SOX2_statusHigh.KDELR1	3.04983946	5.41397461	8.47051623	1.68E-12	4.39E-10	18.1701727	KDELR1	assignmentsChromaffin_cells.Chrom_SOX2_statusHigh
assignmentsChromaffin_cells.Chrom_SOX2_statusHigh.AGT	4.90307203	0.92316892	8.5447558	1.21E-12	3.73E-10	18.1669933	AGT	assignmentsChromaffin_cells.Chrom_SOX2_statusHigh
assignmentsChromaffin_cells.Chrom_SOX2_statusHigh.PUF60	2.45083365	3.93311302	8.46665677	1.70E-12	4.39E-10	18.1663554	PUF60	assignmentsChromaffin_cells.Chrom_SOX2_statusHigh
assignmentsChromaffin_cells.Chrom_SOX2_statusHigh.TMEM97	2.64303237	1.39750036	8.47380744	1.64E-12	4.39E-10	18.1414354	TMEM97	assignmentsChromaffin_cells.Chrom_SOX2_statusHigh
assignmentsChromaffin_cells.Chrom_SOX2_statusHigh.DDOST	2.42889398	4.27316653	8.43855325	1.92E-12	4.88E-10	18.0472585	DDOST	assignmentsChromaffin_cells.Chrom_SOX2_statusHigh

assignmentsChromaffin_cells.Chrom_SOX2_statusHigh.CD320	2.6550909	3.40476275	8.43416246	1.95E-12	4.90E-10	18.0288521	CD320	assignmentsChromaffin_cells.Chrom_SOX2_statusHigh
assignmentsChromaffin_cells.Chrom_SOX2_statusHigh.BSG	3.10597862	5.79615044	8.42244713	2.07E-12	5.11E-10	17.9575241	BSG	assignmentsChromaffin_cells.Chrom_SOX2_statusHigh
assignmentsChromaffin_cells.Chrom_SOX2_statusHigh.EXOSC4	3.29920088	2.32759419	8.39423741	2.33E-12	5.65E-10	17.8384088	EXOSC4	assignmentsChromaffin_cells.Chrom_SOX2_statusHigh
assignmentsChromaffin_cells.Chrom_SOX2_statusHigh.GGH	2.67941063	2.01752588	8.3855605	2.42E-12	5.78E-10	17.7975115	GGH	assignmentsChromaffin_cells.Chrom_SOX2_statusHigh
assignmentsChromaffin_cells.Chrom_SOX2_statusHigh.SYS1	1.79942674	3.49189926	8.3746882	2.53E-12	5.97E-10	17.7740704	SYS1	assignmentsChromaffin_cells.Chrom_SOX2_statusHigh
assignmentsChromaffin_cells.Chrom_SOX2_statusHigh.PSMC5	2.30091663	4.60570386	8.35518033	2.76E-12	6.41E-10	17.6917216	PSMC5	assignmentsChromaffin_cells.Chrom_SOX2_statusHigh
assignmentsChromaffin_cells.Chrom_SOX2_statusHigh.GADD45GIP	3.6296623	3.99489675	8.35171167	2.82E-12	6.47E-10	17.6713314	GADD45GIP1	assignmentsChromaffin_cells.Chrom_SOX2_statusHigh
assignmentsChromaffin_cells.Chrom_SOX2_statusHigh.ZNF576	2.24029365	2.55330253	8.34578179	2.87E-12	6.49E-10	17.6490315	ZNF576	assignmentsChromaffin_cells.Chrom_SOX2_statusHigh
assignmentsChromaffin_cells.Chrom_SOX2_statusHigh.RPL26L1	2.57391725	3.21259328	8.33958739	2.95E-12	6.58E-10	17.6262646	RPL26L1	assignmentsChromaffin_cells.Chrom_SOX2_statusHigh
assignmentsChromaffin_cells.Chrom_SOX2_statusHigh.TAGLN2	4.63774696	5.14046178	8.33563432	3.06E-12	6.72E-10	17.5934912	TAGLN2	assignmentsChromaffin_cells.Chrom_SOX2_statusHigh
assignmentsChromaffin_cells.Chrom_SOX2_statusHigh.CNPY2	1.90738568	5.92263027	8.31144667	3.34E-12	7.23E-10	17.4906035	CNPY2	assignmentsChromaffin_cells.Chrom_SOX2_statusHigh
assignmentsChromaffin_cells.Chrom_SOX2_statusHigh.COMMD5	2.05906327	3.12364679	8.3062794	3.41E-12	7.30E-10	17.4808642	COMMD5	assignmentsChromaffin_cells.Chrom_SOX2_statusHigh
assignmentsChromaffin_cells.Chrom_SOX2_statusHigh.CCDC184	2.82913957	2.49341586	8.30097484	3.49E-12	7.37E-10	17.4638744	CCDC184	assignmentsChromaffin_cells.Chrom_SOX2_statusHigh
assignmentsChromaffin_cells.Chrom_SOX2_statusHigh.FUNDC1	2.37285403	2.56317491	8.28458951	3.75E-12	7.81E-10	17.3918714	FUNDC1	assignmentsChromaffin_cells.Chrom_SOX2_statusHigh
assignmentsChromaffin_cells.Chrom_SOX2_statusHigh.NDUFA3	3.03046016	4.50113506	8.24238382	4.53E-12	9.31E-10	17.2071824	NDUFA3	assignmentsChromaffin_cells.Chrom_SOX2_statusHigh
assignmentsChromaffin_cells.Chrom_SOX2_statusHigh.ATRAID	2.97482814	4.23567022	8.23822156	4.59E-12	9.32E-10	17.1955604	ATRAID	assignmentsChromaffin_cells.Chrom_SOX2_statusHigh
assignmentsChromaffin_cells.Chrom_SOX2_statusHigh.PRDX4	3.38788873	4.28281857	8.22681436	4.86E-12	9.75E-10	17.1379852	PRDX4	assignmentsChromaffin_cells.Chrom_SOX2_statusHigh
assignmentsChromaffin_cells.Chrom_SOX2_statusHigh.TMCO1	1.56458583	5.77648436	8.18170192	5.87E-12	1.16E-09	16.9414244	TMCO1	assignmentsChromaffin_cells.Chrom_SOX2_statusHigh
assignmentsChromaffin_cells.Chrom_SOX2_statusHigh.PSMB5	2.13207298	4.89084572	8.1722618	6.12E-12	1.20E-09	16.9107984	PSMB5	assignmentsChromaffin_cells.Chrom_SOX2_statusHigh
assignmentsChromaffin_cells.Chrom_SOX2_statusHigh.PTRH1	1.97128325	2.8943586	8.16434312	6.33E-12	1.21E-09	16.8839842	PTRH1	assignmentsChromaffin_cells.Chrom_SOX2_statusHigh
assignmentsChromaffin_cells.Chrom_SOX2_statusHigh.MRPS12	3.01670447	2.69594561	8.16444589	6.33E-12	1.21E-09	16.8821694	MRPS12	assignmentsChromaffin_cells.Chrom_SOX2_statusHigh
assignmentsChromaffin_cells.Chrom_SOX2_statusHigh.EBAG9	1.56207015	4.56566555	8.15844004	6.50E-12	1.23E-09	16.8560612	EBAG9	assignmentsChromaffin_cells.Chrom_SOX2_statusHigh
assignmentsChromaffin_cells.Chrom_SOX2_statusHigh.CUEDC2	1.97168414	4.12829975	8.14044586	7.03E-12	1.31E-09	16.7827121	CUEDC2	assignmentsChromaffin_cells.Chrom_SOX2_statusHigh
assignmentsChromaffin_cells.Chrom_SOX2_statusHigh.DCTN3	3.21181391	3.84965802	8.11860266	7.77E-12	1.40E-09	16.6840789	DCTN3	assignmentsChromaffin_cells.Chrom_SOX2_statusHigh
assignmentsChromaffin_cells.Chrom_SOX2_statusHigh.PRDX1	3.49828643	5.0051173	8.11915955	7.77E-12	1.40E-09	16.6789097	PRDX1	assignmentsChromaffin_cells.Chrom_SOX2_statusHigh
assignmentsChromaffin_cells.Chrom_SOX2_statusHigh.PSMB3	2.45409159	4.8088188	8.10475089	8.21E-12	1.46E-09	16.6275816	PSMB3	assignmentsChromaffin_cells.Chrom_SOX2_statusHigh
assignmentsChromaffin_cells.Chrom_SOX2_statusHigh.TMEM205	2.44320041	4.3559178	8.1029089	8.28E-12	1.46E-09	16.6171269	TMEM205	assignmentsChromaffin_cells.Chrom_SOX2_statusHigh
assignmentsChromaffin_cells.Chrom_SOX2_statusHigh.TAF7	1.65914334	5.92435756	8.10000707	8.38E-12	1.46E-09	16.5903551	TAF7	assignmentsChromaffin_cells.Chrom_SOX2_statusHigh
assignmentsChromaffin_cells.Chrom_SOX2_statusHigh.POLR3K	2.65823271	2.5180722	8.09542736	8.55E-12	1.47E-09	16.5849895	POLR3K	assignmentsChromaffin_cells.Chrom_SOX2_statusHigh
assignmentsChromaffin_cells.Chrom_SOX2_statusHigh.SNX17	2.11191908	3.8847422	8.08344292	9.01E-12	1.52E-09	16.5426835	SNX17	assignmentsChromaffin_cells.Chrom_SOX2_statusHigh
assignmentsChromaffin_cells.Chrom_SOX2_statusHigh.CYC1	2.74248185	4.07760435	8.08413065	8.98E-12	1.52E-09	16.5416823	CYC1	assignmentsChromaffin_cells.Chrom_SOX2_statusHigh
assignmentsChromaffin_cells.Chrom_SOX2_statusHigh.DNASE2	3.2108646	2.72505855	8.07842537	9.21E-12	1.54E-09	16.5167059	DNASE2	assignmentsChromaffin_cells.Chrom_SOX2_statusHigh
assignmentsChromaffin_cells.Chrom_SOX2_statusHigh.COX14	3.25218286	3.82955456	8.07482752	9.40E-12	1.55E-09	16.5000933	COX14	assignmentsChromaffin_cells.Chrom_SOX2_statusHigh
assignmentsChromaffin_cells.Chrom_SOX2_statusHigh.NHP2	2.85555317	3.59545615	8.04941386	1.04E-11	1.69E-09	16.3996721	NHP2	assignmentsChromaffin_cells.Chrom_SOX2_statusHigh
assignmentsChromaffin_cells.Chrom_SOX2_statusHigh.DBNDD2	3.40361859	3.18726542	8.04587314	1.07E-11	1.69E-09	16.3804407	DBNDD2	assignmentsChromaffin_cells.Chrom_SOX2_statusHigh
assignmentsChromaffin_cells.Chrom_SOX2_statusHigh.TSR2	2.34864961	3.89451506	8.04414195	1.07E-11	1.69E-09	16.376097	TSR2	assignmentsChromaffin_cells.Chrom_SOX2_statusHigh
assignmentsChromaffin_cells.Chrom_SOX2_statusHigh.IFI6	3.96234535	4.14380599	8.04678688	1.07E-11	1.69E-09	16.3740217	IFI6	assignmentsChromaffin_cells.Chrom_SOX2_statusHigh
assignmentsChromaffin_cells.Chrom_SOX2_statusHigh.ATOX1	2.52791554	4.88591112	8.04371751	1.07E-11	1.69E-09	16.3688791	ATOX1	assignmentsChromaffin_cells.Chrom_SOX2_statusHigh
assignmentsChromaffin_cells.Chrom_SOX2_statusHigh.ETFB	2.41937081	4.39796414	8.03677877	1.10E-11	1.72E-09	16.3410425	ETFB	assignmentsChromaffin_cells.Chrom_SOX2_statusHigh
assignmentsChromaffin_cells.Chrom_SOX2_statusHigh.SLC52A2	2.29725821	2.99551851	8.02334508	1.17E-11	1.79E-09	16.289117	SLC52A2	assignmentsChromaffin_cells.Chrom_SOX2_statusHigh
assignmentsChromaffin_cells.Chrom_SOX2_statusHigh.TM2D2	2.60210946	3.49331356	8.02180469	1.18E-11	1.79E-09	16.283261	TM2D2	assignmentsChromaffin_cells.Chrom_SOX2_statusHigh
assignmentsChromaffin_cells.Chrom_SOX2_statusHigh.PPIB	3.02085985	6.07530931	8.00520199	1.28E-11	1.91E-09	16.1791764	PPIB	assignmentsChromaffin_cells.Chrom_SOX2_statusHigh
assignmentsChromaffin_cells.Chrom_SOX2_statusHigh.MT1G	6.00531906	-0.2416547	8.13266142	7.27E-12	1.34E-09	16.1605833	MT1G	assignmentsChromaffin_cells.Chrom_SOX2_statusHigh
assignmentsChromaffin_cells.Chrom_SOX2_statusHigh.SCAND1	2.57917555	4.29007434	7.98774599	1.37E-11	2.03E-09	16.1328273	SCAND1	assignmentsChromaffin_cells.Chrom_SOX2_statusHigh
assignmentsChromaffin_cells.Chrom_SOX2_statusHigh.MRPS34	2.55628523	4.11231487	7.98540671	1.38E-11	2.03E-09	16.1265249	MRPS34	assignmentsChromaffin_cells.Chrom_SOX2_statusHigh
assignmentsChromaffin_cells.Chrom_SOX2_statusHigh.SLC1A5	4.4908915	2.10924281	8.02057956	1.18E-11	1.79E-09	16.0267391	SLC1A5	assignmentsChromaffin_cells.Chrom_SOX2_statusHigh
assignmentsChromaffin_cells.Chrom_SOX2_statusHigh.GABARAPL2	2.71623004	5.60068664	7.95966725	1.55E-11	2.26E-09	15.9910622	GABARAPL2	assignmentsChromaffin_cells.Chrom_SOX2_statusHigh
assignmentsChromaffin_cells.Chrom_SOX2_statusHigh.UQCRQ	3.34233972	4.90343373	7.95502794	1.59E-11	2.29E-09	15.977769	UQCRQ	assignmentsChromaffin_cells.Chrom_SOX2_statusHigh
assignmentsChromaffin_cells.Chrom_SOX2_statusHigh.AHCY	1.86535306	3.41895499	7.94879402	1.62E-11	2.32E-09	15.9750106	AHCY	assignmentsChromaffin_cells.Chrom_SOX2_statusHigh
assignmentsChromaffin_cells.Chrom_SOX2_statusHigh.NCBP2AS2	2.90406866	2.8255571	7.93880305	1.69E-11	2.39E-09	15.9330168	NCBP2AS2	assignmentsChromaffin_cells.Chrom_SOX2_statusHigh
assignmentsChromaffin_cells.Chrom_SOX2_statusHigh.NUDCD2	1.83560974	3.79022777	7.93803367	1.70E-11	2.39E-09	15.928877	NUDCD2	assignmentsChromaffin_cells.Chrom_SOX2_statusHigh
assignmentsChromaffin_cells.Chrom_SOX2_statusHigh.C6orf120	2.26840281	3.15009579	7.93107535	1.75E-11	2.42E-09	15.8998568	C6orf120	assignmentsChromaffin_cells.Chrom_SOX2_statusHigh
assignmentsChromaffin_cells.Chrom_SOX2_statusHigh.DAD1	2.27506834	5.50488345	7.93237914	1.74E-11	2.42E-09	15.8858284	DAD1	assignmentsChromaffin_cells.Chrom_SOX2_statusHigh
assignmentsChromaffin_cells.Chrom_SOX2_statusHigh.CCT3	1.84189211	6.01839183	7.91461931	1.88E-11	2.58E-09	15.8000207	CCT3	assignmentsChromaffin_cells.Chrom_SOX2_statusHigh
assignmentsChromaffin_cells.Chrom_SOX2_statusHigh.MDH2	2.26828715	4.77613916	7.90344873	1.97E-11	2.66E-09	15.7674837	MDH2	assignmentsChromaffin_cells.Chrom_SOX2_statusHigh
assignmentsChromaffin_cells.Chrom_SOX2_statusHigh.MAD2L1	2.91807981	0.894852	7.9069204	1.94E-11	2.64E-09	15.75708	MAD2L1	assignmentsChromaffin_cells.Chrom_SOX2_statusHigh
assignmentsChromaffin_cells.Chrom_SOX2_statusHigh.CLASP1	-1.3947245	8.21633751	-7.9000164	2.00E-11	2.68E-09	15.7248749	CLASP1	assignmentsChromaffin_cells.Chrom_SOX2_statusHigh
assignmentsChromaffin_cells.Chrom_SOX2_statusHigh.CHMP2A	3.16743445	4.32120742	7.89224216	2.08E-11	2.76E-09	15.723893	CHMP2A	assignmentsChromaffin_cells.Chrom_SOX2_statusHigh
assignmentsChromaffin_cells.Chrom_SOX2_statusHigh.PTRHD1	3.8058081	2.03108447	7.88853624	2.11E-11	2.77E-09	15.7062921	PTRHD1	assignmentsChromaffin_cells.Chrom_SOX2_statusHigh
assignmentsChromaffin_cells.Chrom_SOX2_statusHigh.SPCS1	3.24601106	4.86448079	7.88798237	2.12E-11	2.77E-09	15.6931952	SPCS1	assignmentsChromaffin_cells.Chrom_SOX2_statusHigh
assignmentsChromaffin_cells.Chrom_SOX2_statusHigh.MRPL57	2.43443052	4.73360142	7.86174129	2.36E-11	3.05E-09	15.5914889	MRPL57	assignmentsChromaffin_cells.Chrom_SOX2_statusHigh
assignmentsChromaffin_cells.Chrom_SOX2_statusHigh.HIGD1A	3.27072966	3.66870814	7.86064797	2.39E-11	3.05E-09	15.5896287	HIGD1A	assignmentsChromaffin_cells.Chrom_SOX2_statusHigh
assignmentsChromaffin_cells.Chrom_SOX2_statusHigh.CALR	3.00414658	6.03512281	7.86015837	2.40E-11	3.05E-09	15.5660085	CALR	assignmentsChromaffin_cells.Chrom_SOX2_statusHigh
assignmentsChromaffin_cells.Chrom_SOX2_statusHigh.SEPHS2	2.60603003	3.11233196	7.84074117	2.59E-11	3.25E-09	15.5192907	SEPHS2	assignmentsChromaffin_cells.Chrom_SOX2_statusHigh

assignmentsChromaffin_cells.Chrom_SOX2_statusHigh.NENF	2.56745904	4.97295141	7.84350161	2.56E-11	3.23E-09	15.5083424	NENF	assignmentsChromaffin_cells.Chrom_SOX2_statusHigh
assignmentsChromaffin_cells.Chrom_SOX2_statusHigh.ECH1	2.61655775	4.33685066	7.83221198	2.69E-11	3.34E-09	15.4778258	ECH1	assignmentsChromaffin_cells.Chrom_SOX2_statusHigh
assignmentsChromaffin_cells.Chrom_SOX2_statusHigh.MYDGF	2.72738226	5.14870812	7.83074004	2.71E-11	3.34E-09	15.4550357	MYDGF	assignmentsChromaffin_cells.Chrom_SOX2_statusHigh
assignmentsChromaffin_cells.Chrom_SOX2_statusHigh.NDUFAF8	2.13070609	4.53607803	7.8192464	2.84E-11	3.48E-09	15.4106631	NDUFAF8	assignmentsChromaffin_cells.Chrom_SOX2_statusHigh
assignmentsChromaffin_cells.Chrom_SOX2_statusHigh.TOMM5	2.43364383	4.32707566	7.81577573	2.89E-11	3.51E-09	15.4049551	TOMM5	assignmentsChromaffin_cells.Chrom_SOX2_statusHigh
assignmentsChromaffin_cells.Chrom_SOX2_statusHigh.MEA1	2.84212958	3.20388441	7.80722399	3.00E-11	3.59E-09	15.3782035	MEA1	assignmentsChromaffin_cells.Chrom_SOX2_statusHigh
assignmentsChromaffin_cells.Chrom_SOX2_statusHigh.REEP5	1.61152414	6.12458143	7.81402876	2.91E-11	3.51E-09	15.3767797	REEP5	assignmentsChromaffin_cells.Chrom_SOX2_statusHigh
assignmentsChromaffin_cells.Chrom_SOX2_statusHigh.GHITM	2.28138982	5.34701562	7.80548752	3.02E-11	3.59E-09	15.3450709	GHITM	assignmentsChromaffin_cells.Chrom_SOX2_statusHigh
assignmentsChromaffin_cells.Chrom_SOX2_statusHigh.ERH	2.05964185	5.26333624	7.78921902	3.24E-11	3.82E-09	15.2782423	ERH	assignmentsChromaffin_cells.Chrom_SOX2_statusHigh
assignmentsChromaffin_cells.Chrom_SOX2_statusHigh.MRPL12	2.38333664	3.37229039	7.76753033	3.56E-11	4.17E-09	15.2088086	MRPL12	assignmentsChromaffin_cells.Chrom_SOX2_statusHigh
assignmentsChromaffin_cells.Chrom_SOX2_statusHigh.NAXE	2.43143525	3.83942884	7.75486226	3.76E-11	4.31E-09	15.1523927	NAXE	assignmentsChromaffin_cells.Chrom_SOX2_statusHigh
assignmentsChromaffin_cells.Chrom_SOX2_statusHigh.S100A11	3.86852267	5.67124433	7.76031242	3.73E-11	4.31E-09	15.145236	S100A11	assignmentsChromaffin_cells.Chrom_SOX2_statusHigh
assignmentsChromaffin_cells.Chrom_SOX2_statusHigh.GIHCG	1.89153527	3.15368754	7.75060816	3.83E-11	4.36E-09	15.1362954	GIHCG	assignmentsChromaffin_cells.Chrom_SOX2_statusHigh
assignmentsChromaffin_cells.Chrom_SOX2_statusHigh.ZNF593	3.0036516	2.31620389	7.7491761	3.86E-11	4.36E-09	15.1297534	ZNF593	assignmentsChromaffin_cells.Chrom_SOX2_statusHigh
assignmentsChromaffin_cells.Chrom_SOX2_statusHigh.NUDT18	2.60363838	1.1804502	7.75723635	3.72E-11	4.31E-09	15.1287048	NUDT18	assignmentsChromaffin_cells.Chrom_SOX2_statusHigh
assignmentsChromaffin_cells.Chrom_SOX2_statusHigh.MRPS23	1.79669762	3.80833664	7.73473175	4.11E-11	4.61E-09	15.067179	MRPS23	assignmentsChromaffin_cells.Chrom_SOX2_statusHigh
assignmentsChromaffin_cells.Chrom_SOX2_statusHigh.TMEM115	2.63138354	2.34663307	7.72944374	4.20E-11	4.65E-09	15.0490065	TMEM115	assignmentsChromaffin_cells.Chrom_SOX2_statusHigh
assignmentsChromaffin_cells.Chrom_SOX2_statusHigh.POLR2K	3.19870932	3.8609345	7.73285764	4.17E-11	4.64E-09	15.0478443	POLR2K	assignmentsChromaffin_cells.Chrom_SOX2_statusHigh
assignmentsChromaffin_cells.Chrom_SOX2_statusHigh.EMC6	3.02006192	3.1262771	7.71856916	4.41E-11	4.81E-09	15.0039695	EMC6	assignmentsChromaffin_cells.Chrom_SOX2_statusHigh
assignmentsChromaffin_cells.Chrom_SOX2_statusHigh.PSME1	3.07196221	4.68740944	7.72069892	4.39E-11	4.81E-09	14.9981389	PSME1	assignmentsChromaffin_cells.Chrom_SOX2_statusHigh
assignmentsChromaffin_cells.Chrom_SOX2_statusHigh.C19orf53	3.85023025	4.28333065	7.70906581	4.66E-11	5.05E-09	14.935503	C19orf53	assignmentsChromaffin_cells.Chrom_SOX2_statusHigh
assignmentsChromaffin_cells.Chrom_SOX2_statusHigh.LSM4	2.69328409	4.13985708	7.70394733	4.70E-11	5.05E-09	14.9321356	LSM4	assignmentsChromaffin_cells.Chrom_SOX2_statusHigh
assignmentsChromaffin_cells.Chrom_SOX2_statusHigh.PDZD11	2.23098127	2.95202196	7.69786708	4.82E-11	5.09E-09	14.9174803	PDZD11	assignmentsChromaffin_cells.Chrom_SOX2_statusHigh
assignmentsChromaffin_cells.Chrom_SOX2_statusHigh.KEAP1	1.50200766	3.45901105	7.69548128	4.87E-11	5.11E-09	14.9073487	KEAP1	assignmentsChromaffin_cells.Chrom_SOX2_statusHigh
assignmentsChromaffin_cells.Chrom_SOX2_statusHigh.NDUFB10	3.22998478	4.21233765	7.6946641	4.92E-11	5.11E-09	14.8869507	NDUFB10	assignmentsChromaffin_cells.Chrom_SOX2_statusHigh
assignmentsChromaffin_cells.Chrom_SOX2_statusHigh.PRR13	2.24297203	4.53713657	7.69224717	4.94E-11	5.11E-09	14.8860914	PRR13	assignmentsChromaffin_cells.Chrom_SOX2_statusHigh
assignmentsChromaffin_cells.Chrom_SOX2_statusHigh.RNASEK	3.26422302	6.19099179	7.70098903	4.81E-11	5.09E-09	14.878405	RNASEK	assignmentsChromaffin_cells.Chrom_SOX2_statusHigh
assignmentsChromaffin_cells.Chrom_SOX2_statusHigh.BRWD1	-1.1589394	7.79162752	-7.6986595	4.80E-11	5.09E-09	14.8750599	BRWD1	assignmentsChromaffin_cells.Chrom_SOX2_statusHigh
assignmentsChromaffin_cells.Chrom_SOX2_statusHigh.DMAC1	2.25390598	3.68371876	7.68863492	5.02E-11	5.16E-09	14.8742611	DMAC1	assignmentsChromaffin_cells.Chrom_SOX2_statusHigh
assignmentsChromaffin_cells.Chrom_SOX2_statusHigh.MAGEF1	2.28971119	3.28791411	7.68352427	5.13E-11	5.21E-09	14.8513203	MAGEF1	assignmentsChromaffin_cells.Chrom_SOX2_statusHigh
assignmentsChromaffin_cells.Chrom_SOX2_statusHigh.SNRNP25	2.70104403	2.41602478	7.6796029	5.22E-11	5.26E-09	14.8415772	SNRNP25	assignmentsChromaffin_cells.Chrom_SOX2_statusHigh
assignmentsChromaffin_cells.Chrom_SOX2_statusHigh.KRTCAP2	2.88163681	5.2978122	7.6866522	5.09E-11	5.19E-09	14.8353982	KRTCAP2	assignmentsChromaffin_cells.Chrom_SOX2_statusHigh
assignmentsChromaffin_cells.Chrom_SOX2_statusHigh.RTL8A	2.61157379	3.55809927	7.66919228	5.46E-11	5.47E-09	14.7909385	RTL8A	assignmentsChromaffin_cells.Chrom_SOX2_statusHigh
assignmentsChromaffin_cells.Chrom_SOX2_statusHigh.ELOF1	2.19924373	3.18046656	7.66524264	5.55E-11	5.53E-09	14.7795671	ELOF1	assignmentsChromaffin_cells.Chrom_SOX2_statusHigh
assignmentsChromaffin_cells.Chrom_SOX2_statusHigh.CCDC106	2.34061154	2.14414049	7.64739057	6.00E-11	5.84E-09	14.7062144	CCDC106	assignmentsChromaffin_cells.Chrom_SOX2_statusHigh
assignmentsChromaffin_cells.Chrom_SOX2_statusHigh.PSMB1	2.22018613	5.9345369	7.65316325	5.85E-11	5.79E-09	14.6946747	PSMB1	assignmentsChromaffin_cells.Chrom_SOX2_statusHigh
assignmentsChromaffin_cells.Chrom_SOX2_statusHigh.UQCR11	3.21039277	5.66892071	7.6543458	5.89E-11	5.80E-09	14.6830645	UQCR11	assignmentsChromaffin_cells.Chrom_SOX2_statusHigh
assignmentsChromaffin_cells.Chrom_SOX2_statusHigh.SUMO2	2.6562103	6.09835065	7.64797208	6.01E-11	5.84E-09	14.661498	SUMO2	assignmentsChromaffin_cells.Chrom_SOX2_statusHigh
assignmentsChromaffin_cells.Chrom_SOX2_statusHigh.MEAF6	1.51267412	5.10075798	7.63854074	6.24E-11	6.03E-09	14.6507357	MEAF6	assignmentsChromaffin_cells.Chrom_SOX2_statusHigh
assignmentsChromaffin_cells.Chrom_SOX2_statusHigh.SRP9	1.68630785	5.80457117	7.63689175	6.28E-11	6.03E-09	14.621369	SRP9	assignmentsChromaffin_cells.Chrom_SOX2_statusHigh
assignmentsChromaffin_cells.Chrom_SOX2_statusHigh.RRAGA	2.71544101	3.83493366	7.62192644	6.70E-11	6.32E-09	14.588873	RRAGA	assignmentsChromaffin_cells.Chrom_SOX2_statusHigh
assignmentsChromaffin_cells.Chrom_SOX2_statusHigh.SAP18	2.81338291	5.31956034	7.62791507	6.56E-11	6.26E-09	14.5866802	SAP18	assignmentsChromaffin_cells.Chrom_SOX2_statusHigh
assignmentsChromaffin_cells.Chrom_SOX2_statusHigh.SELENOW	2.90244173	6.12089193	7.62338034	6.70E-11	6.32E-09	14.5542638	SELENOW	assignmentsChromaffin_cells.Chrom_SOX2_statusHigh
assignmentsChromaffin_cells.Chrom_SOX2_statusHigh.ALG3	2.09004332	2.6808564	7.60694086	7.15E-11	6.59E-09	14.5365979	ALG3	assignmentsChromaffin_cells.Chrom_SOX2_statusHigh
assignmentsChromaffin_cells.Chrom_SOX2_statusHigh.MRPS7	2.05767719	4.11387585	7.61024891	7.05E-11	6.58E-09	14.5357107	MRPS7	assignmentsChromaffin_cells.Chrom_SOX2_statusHigh
assignmentsChromaffin_cells.Chrom_SOX2_statusHigh.CEP192	-1.4901618	6.73982861	-7.6108804	7.03E-11	6.58E-09	14.5271573	CEP192	assignmentsChromaffin_cells.Chrom_SOX2_statusHigh
assignmentsChromaffin_cells.Chrom_SOX2_statusHigh.P4HTM	2.01198903	4.7867923	7.60776363	7.13E-11	6.59E-09	14.5066327	P4HTM	assignmentsChromaffin_cells.Chrom_SOX2_statusHigh
assignmentsChromaffin_cells.Chrom_SOX2_statusHigh.HOXA7	5.0298976	1.12646864	7.60650015	7.28E-11	6.63E-09	14.491093	HOXA7	assignmentsChromaffin_cells.Chrom_SOX2_statusHigh
assignmentsChromaffin_cells.Chrom_SOX2_statusHigh.PRADC1	2.35243928	2.16138796	7.59473115	7.54E-11	6.83E-09	14.4838029	PRADC1	assignmentsChromaffin_cells.Chrom_SOX2_statusHigh
assignmentsChromaffin_cells.Chrom_SOX2_statusHigh.LAPTM4A	2.76012244	6.34992414	7.60580398	7.23E-11	6.62E-09	14.4762528	LAPTM4A	assignmentsChromaffin_cells.Chrom_SOX2_statusHigh
assignmentsChromaffin_cells.Chrom_SOX2_statusHigh.BRF2	1.99390182	1.73938212	7.58797217	7.77E-11	6.96E-09	14.4486699	BRF2	assignmentsChromaffin_cells.Chrom_SOX2_statusHigh
assignmentsChromaffin_cells.Chrom_SOX2_statusHigh.ZMAT2	2.10660673	4.02696474	7.57973332	8.05E-11	7.08E-09	14.4124962	ZMAT2	assignmentsChromaffin_cells.Chrom_SOX2_statusHigh
assignmentsChromaffin_cells.Chrom_SOX2_statusHigh.DYNLRB1	2.31410087	4.99928576	7.58227131	7.96E-11	7.08E-09	14.4081101	DYNLRB1	assignmentsChromaffin_cells.Chrom_SOX2_statusHigh
assignmentsChromaffin_cells.Chrom_SOX2_statusHigh.ARPC5L	1.91302422	4.45834753	7.57732256	8.13E-11	7.08E-09	14.4035905	ARPC5L	assignmentsChromaffin_cells.Chrom_SOX2_statusHigh
assignmentsChromaffin_cells.Chrom_SOX2_statusHigh.JTB	2.69001863	4.87837395	7.5799029	8.06E-11	7.08E-09	14.3950387	JTB	assignmentsChromaffin_cells.Chrom_SOX2_statusHigh
assignmentsChromaffin_cells.Chrom_SOX2_statusHigh.LAMTOR2	2.8588211	3.75635427	7.57425766	8.24E-11	7.08E-09	14.3931856	LAMTOR2	assignmentsChromaffin_cells.Chrom_SOX2_statusHigh
assignmentsChromaffin_cells.Chrom_SOX2_statusHigh.CKS1B	2.65482615	2.93387173	7.5726983	8.30E-11	7.08E-09	14.3905665	CKS1B	assignmentsChromaffin_cells.Chrom_SOX2_statusHigh
assignmentsChromaffin_cells.Chrom_SOX2_statusHigh.GTF2A2	2.23600316	4.68875128	7.57409752	8.25E-11	7.08E-09	14.3762959	GTF2A2	assignmentsChromaffin_cells.Chrom_SOX2_statusHigh
assignmentsChromaffin_cells.Chrom_SOX2_statusHigh.HIGD2A	3.29010685	4.28206596	7.57428009	8.29E-11	7.08E-09	14.3757255	HIGD2A	assignmentsChromaffin_cells.Chrom_SOX2_statusHigh
assignmentsChromaffin_cells.Chrom_SOX2_statusHigh.SLC39A6	2.24559609	4.78400639	7.57375568	8.26E-11	7.08E-09	14.3653594	SLC39A6	assignmentsChromaffin_cells.Chrom_SOX2_statusHigh
assignmentsChromaffin_cells.Chrom_SOX2_statusHigh.SIVA1	2.30547602	4.43710049	7.56821849	8.46E-11	7.14E-09	14.3627247	SIVA1	assignmentsChromaffin_cells.Chrom_SOX2_statusHigh
assignmentsChromaffin_cells.Chrom_SOX2_statusHigh.PRELID1	3.36306493	4.12302525	7.56414366	8.67E-11	7.28E-09	14.3397009	PRELID1	assignmentsChromaffin_cells.Chrom_SOX2_statusHigh
assignmentsChromaffin_cells.Chrom_SOX2_statusHigh.ACTG1	4.31327415	7.12849611	7.57178815	8.46E-11	7.14E-09	14.3207123	ACTG1	assignmentsChromaffin_cells.Chrom_SOX2_statusHigh
assignmentsChromaffin_cells.Chrom_SOX2_statusHigh.SYT7	5.16185953	0.92050202	7.56361822	8.76E-11	7.32E-09	14.3166075	SYT7	assignmentsChromaffin_cells.Chrom_SOX2_statusHigh

assignmentsChromaffin_cells.Chrom_SOX2_statusHigh.TUSC2	2.69175282	3.12996108	7.55469545	8.97E-11	7.46E-09	14.3146704	TUSC2	assignmentsChromaffin_cells.Chrom_SOX2_statusHigh
assignmentsChromaffin_cells.Chrom_SOX2_statusHigh.STOML2	2.48487603	3.40076569	7.55203953	9.08E-11	7.50E-09	14.2998275	STOML2	assignmentsChromaffin_cells.Chrom_SOX2_statusHigh
assignmentsChromaffin_cells.Chrom_SOX2_statusHigh.CERS2	1.83668724	4.19802076	7.54969795	9.17E-11	7.54E-09	14.2844878	CERS2	assignmentsChromaffin_cells.Chrom_SOX2_statusHigh
assignmentsChromaffin_cells.Chrom_SOX2_statusHigh.SLC39A3	2.49149054	3.1995342	7.54813027	9.23E-11	7.55E-09	14.2842722	SLC39A3	assignmentsChromaffin_cells.Chrom_SOX2_statusHigh
assignmentsChromaffin_cells.Chrom_SOX2_statusHigh.NDUFAF3	3.67069997	3.89517113	7.54969085	9.28E-11	7.55E-09	14.2727321	NDUFAF3	assignmentsChromaffin_cells.Chrom_SOX2_statusHigh
assignmentsChromaffin_cells.Chrom_SOX2_statusHigh.HNRNPH2	2.02214497	4.67173041	7.54220309	9.47E-11	7.67E-09	14.243358	HNRNPH2	assignmentsChromaffin_cells.Chrom_SOX2_statusHigh
assignmentsChromaffin_cells.Chrom_SOX2_statusHigh.TRAPPC1	3.35801127	3.72981699	7.53820777	9.70E-11	7.78E-09	14.2314941	TRAPPC1	assignmentsChromaffin_cells.Chrom_SOX2_statusHigh
assignmentsChromaffin_cells.Chrom_SOX2_statusHigh.HINT1	2.91577629	6.20886205	7.54208825	9.54E-11	7.68E-09	14.2027249	HINT1	assignmentsChromaffin_cells.Chrom_SOX2_statusHigh
assignmentsChromaffin_cells.Chrom_SOX2_statusHigh.WDR83OS	3.51584374	4.74487817	7.53585811	9.88E-11	7.88E-09	14.1926579	WDR83OS	assignmentsChromaffin_cells.Chrom_SOX2_statusHigh
assignmentsChromaffin_cells.Chrom_SOX2_statusHigh.HSD17B10	2.80579194	3.12618287	7.50547075	1.11E-10	8.73E-09	14.1070217	HSD17B10	assignmentsChromaffin_cells.Chrom_SOX2_statusHigh
assignmentsChromaffin_cells.Chrom_SOX2_statusHigh.SLCO4A1	5.16485977	0.562433	7.59275284	7.61E-11	6.85E-09	14.1042363	SLCO4A1	assignmentsChromaffin_cells.Chrom_SOX2_statusHigh
assignmentsChromaffin_cells.Chrom_SOX2_statusHigh.PUM2	-1.1084589	8.03002499	-7.5114301	1.08E-10	8.59E-09	14.0748889	PUM2	assignmentsChromaffin_cells.Chrom_SOX2_statusHigh
assignmentsChromaffin_cells.Chrom_SOX2_statusHigh.PTTG1	2.73533001	2.08867641	7.49772495	1.15E-10	8.92E-09	14.0728433	PTTG1	assignmentsChromaffin_cells.Chrom_SOX2_statusHigh
assignmentsChromaffin_cells.Chrom_SOX2_statusHigh.NDUFA8	1.79183438	3.83588177	7.49710541	1.15E-10	8.92E-09	14.0622379	NDUFA8	assignmentsChromaffin_cells.Chrom_SOX2_statusHigh
assignmentsChromaffin_cells.Chrom_SOX2_statusHigh.COPRS	2.69861615	3.12391932	7.49504677	1.16E-10	8.96E-09	14.0618437	COPRS	assignmentsChromaffin_cells.Chrom_SOX2_statusHigh
assignmentsChromaffin_cells.Chrom_SOX2_statusHigh.REV1	-1.2403614	7.05799067	-7.5014047	1.13E-10	8.84E-09	14.0511257	REV1	assignmentsChromaffin_cells.Chrom_SOX2_statusHigh
assignmentsChromaffin_cells.Chrom_SOX2_statusHigh.MANF	2.81922701	3.78290434	7.49112494	1.18E-10	9.05E-09	14.0420354	MANF	assignmentsChromaffin_cells.Chrom_SOX2_statusHigh
assignmentsChromaffin_cells.Chrom_SOX2_statusHigh.MRPL36	2.93706173	2.89465269	7.4869531	1.20E-10	9.05E-09	14.030927	MRPL36	assignmentsChromaffin_cells.Chrom_SOX2_statusHigh
assignmentsChromaffin_cells.Chrom_SOX2_statusHigh.SF3B5	3.44297527	3.65942953	7.48754629	1.21E-10	9.05E-09	14.0216651	SF3B5	assignmentsChromaffin_cells.Chrom_SOX2_statusHigh
assignmentsChromaffin_cells.Chrom_SOX2_statusHigh.WNT10A	5.4454229	0.22951737	7.51173672	1.09E-10	8.63E-09	14.0186128	WNT10A	assignmentsChromaffin_cells.Chrom_SOX2_statusHigh
assignmentsChromaffin_cells.Chrom_SOX2_statusHigh.FAM168A	-1.6342522	7.32235588	-7.4881901	1.20E-10	9.05E-09	13.9930865	FAM168A	assignmentsChromaffin_cells.Chrom_SOX2_statusHigh
assignmentsChromaffin_cells.Chrom_SOX2_statusHigh.MRPL41	2.33008859	4.33512927	7.47823946	1.25E-10	9.32E-09	13.9810832	MRPL41	assignmentsChromaffin_cells.Chrom_SOX2_statusHigh
assignmentsChromaffin_cells.Chrom_SOX2_statusHigh.RTBDN	4.29487161	0.46661022	7.46978333	1.30E-10	9.68E-09	13.9360514	RTBDN	assignmentsChromaffin_cells.Chrom_SOX2_statusHigh
assignmentsChromaffin_cells.Chrom_SOX2_statusHigh.CD9	5.20959734	5.50390925	7.46803818	1.32E-10	9.79E-09	13.9336255	CD9	assignmentsChromaffin_cells.Chrom_SOX2_statusHigh
assignmentsChromaffin_cells.Chrom_SOX2_statusHigh.TIMM10	3.60410034	2.45472198	7.44928411	1.42E-10	1.03E-08	13.8717624	TIMM10	assignmentsChromaffin_cells.Chrom_SOX2_statusHigh
assignmentsChromaffin_cells.Chrom_SOX2_statusHigh.TSPAN3	1.29320322	6.13877199	7.45795318	1.36E-10	1.00E-08	13.8568912	TSPAN3	assignmentsChromaffin_cells.Chrom_SOX2_statusHigh
assignmentsChromaffin_cells.Chrom_SOX2_statusHigh.TLR4	4.37879791	3.11448019	7.453603	1.40E-10	1.02E-08	13.8519078	TLR4	assignmentsChromaffin_cells.Chrom_SOX2_statusHigh
assignmentsChromaffin_cells.Chrom_SOX2_statusHigh.MLLT10	-1.2190873	7.79669766	-7.4532533	1.39E-10	1.02E-08	13.8337437	MLLT10	assignmentsChromaffin_cells.Chrom_SOX2_statusHigh
assignmentsChromaffin_cells.Chrom_SOX2_statusHigh.TEX264	2.00124186	3.54764029	7.43768855	1.49E-10	1.08E-08	13.8246112	TEX264	assignmentsChromaffin_cells.Chrom_SOX2_statusHigh
assignmentsChromaffin_cells.Chrom_SOX2_statusHigh.ABHD14A	2.70566259	2.79009076	7.43475644	1.51E-10	1.08E-08	13.8110833	ABHD14A	assignmentsChromaffin_cells.Chrom_SOX2_statusHigh
assignmentsChromaffin_cells.Chrom_SOX2_statusHigh.RRS1	2.90783405	1.71517803	7.43386254	1.51E-10	1.08E-08	13.8020013	RRS1	assignmentsChromaffin_cells.Chrom_SOX2_statusHigh
assignmentsChromaffin_cells.Chrom_SOX2_statusHigh.PRR34-AS1	4.83891023	1.28120528	7.43783996	1.50E-10	1.08E-08	13.77874	PRR34-AS1	assignmentsChromaffin_cells.Chrom_SOX2_statusHigh
assignmentsChromaffin_cells.Chrom_SOX2_statusHigh.PPCS	2.2147351	3.67273816	7.42560045	1.57E-10	1.11E-08	13.7753648	PPCS	assignmentsChromaffin_cells.Chrom_SOX2_statusHigh
assignmentsChromaffin_cells.Chrom_SOX2_statusHigh.NAT1	2.91453021	0.87525054	7.48938175	1.19E-10	9.05E-09	13.7631777	NAT1	assignmentsChromaffin_cells.Chrom_SOX2_statusHigh
assignmentsChromaffin_cells.Chrom_SOX2_statusHigh.NDUFB7	3.23331818	4.79723634	7.42671405	1.58E-10	1.11E-08	13.7405586	NDUFB7	assignmentsChromaffin_cells.Chrom_SOX2_statusHigh
assignmentsChromaffin_cells.Chrom_SOX2_statusHigh.ECHS1	2.28085726	4.09400229	7.41718118	1.63E-10	1.15E-08	13.7212717	ECHS1	assignmentsChromaffin_cells.Chrom_SOX2_statusHigh
assignmentsChromaffin_cells.Chrom_SOX2_statusHigh.TXNDC17	3.46979914	3.06297151	7.41200195	1.67E-10	1.16E-08	13.7143063	TXNDC17	assignmentsChromaffin_cells.Chrom_SOX2_statusHigh
assignmentsChromaffin_cells.Chrom_SOX2_statusHigh.MRPL54	3.26690011	3.22784743	7.41138416	1.68E-10	1.16E-08	13.7062908	MRPL54	assignmentsChromaffin_cells.Chrom_SOX2_statusHigh
assignmentsChromaffin_cells.Chrom_SOX2_statusHigh.TIMM17B	2.48964954	3.49652487	7.41089583	1.67E-10	1.16E-08	13.7037345	TIMM17B	assignmentsChromaffin_cells.Chrom_SOX2_statusHigh
assignmentsChromaffin_cells.Chrom_SOX2_statusHigh.SHISA4	3.12507741	1.350235	7.40669385	1.70E-10	1.17E-08	13.6872232	SHISA4	assignmentsChromaffin_cells.Chrom_SOX2_statusHigh
assignmentsChromaffin_cells.Chrom_SOX2_statusHigh.TM6SF2	4.69076821	-0.2013056	7.48821094	1.20E-10	9.05E-09	13.6806532	TM6SF2	assignmentsChromaffin_cells.Chrom_SOX2_statusHigh
assignmentsChromaffin_cells.Chrom_SOX2_statusHigh.NME1	2.654302	3.83603322	7.40385584	1.72E-10	1.18E-08	13.6684983	NME1	assignmentsChromaffin_cells.Chrom_SOX2_statusHigh
assignmentsChromaffin_cells.Chrom_SOX2_statusHigh.SKP1	1.99706619	6.82571689	7.41114017	1.67E-10	1.16E-08	13.6465376	SKP1	assignmentsChromaffin_cells.Chrom_SOX2_statusHigh
assignmentsChromaffin_cells.Chrom_SOX2_statusHigh.TRMT10C	2.36011503	3.66137572	7.39294466	1.81E-10	1.23E-08	13.6333511	TRMT10C	assignmentsChromaffin_cells.Chrom_SOX2_statusHigh
assignmentsChromaffin_cells.Chrom_SOX2_statusHigh.ATAD2B	-1.3064608	7.3746423	-7.3871222	1.85E-10	1.26E-08	13.5688919	ATAD2B	assignmentsChromaffin_cells.Chrom_SOX2_statusHigh
assignmentsChromaffin_cells.Chrom_SOX2_statusHigh.RAB13	2.89349859	4.09956973	7.37733954	1.94E-10	1.30E-08	13.5627002	RAB13	assignmentsChromaffin_cells.Chrom_SOX2_statusHigh
assignmentsChromaffin_cells.Chrom_SOX2_statusHigh.DHPS	1.95654508	4.42178975	7.37814654	1.93E-10	1.30E-08	13.5538545	DHPS	assignmentsChromaffin_cells.Chrom_SOX2_statusHigh
assignmentsChromaffin_cells.Chrom_SOX2_statusHigh.CDKAL1	-1.2233453	8.16522771	-7.3868701	1.86E-10	1.26E-08	13.5505461	CDKAL1	assignmentsChromaffin_cells.Chrom_SOX2_statusHigh
assignmentsChromaffin_cells.Chrom_SOX2_statusHigh.ORMDL2	2.37304572	3.20426267	7.36894182	2.01E-10	1.33E-08	13.5366715	ORMDL2	assignmentsChromaffin_cells.Chrom_SOX2_statusHigh
assignmentsChromaffin_cells.Chrom_SOX2_statusHigh.C4orf3	2.71105451	4.38371635	7.36777952	2.02E-10	1.33E-08	13.5166645	C4orf3	assignmentsChromaffin_cells.Chrom_SOX2_statusHigh
assignmentsChromaffin_cells.Chrom_SOX2_statusHigh.ZNF407	-1.3484468	7.5709371	-7.3747735	1.96E-10	1.31E-08	13.5142732	ZNF407	assignmentsChromaffin_cells.Chrom_SOX2_statusHigh
assignmentsChromaffin_cells.Chrom_SOX2_statusHigh.ATP6AP1	2.38427845	5.10454992	7.36574015	2.03E-10	1.34E-08	13.4859724	ATP6AP1	assignmentsChromaffin_cells.Chrom_SOX2_statusHigh
assignmentsChromaffin_cells.Chrom_SOX2_statusHigh.COX6B1	3.17774649	5.3944733	7.36744463	2.04E-10	1.34E-08	13.4772506	COX6B1	assignmentsChromaffin_cells.Chrom_SOX2_statusHigh
assignmentsChromaffin_cells.Chrom_SOX2_statusHigh.C1GALT1C1	2.36719015	3.13205023	7.35115079	2.17E-10	1.41E-08	13.4627683	C1GALT1C1	assignmentsChromaffin_cells.Chrom_SOX2_statusHigh
assignmentsChromaffin_cells.Chrom_SOX2_statusHigh.GGACT	3.42805042	0.57799454	7.3735375	1.97E-10	1.31E-08	13.4239355	GGACT	assignmentsChromaffin_cells.Chrom_SOX2_statusHigh
assignmentsChromaffin_cells.Chrom_SOX2_statusHigh.ADRM1	2.56973234	3.75466341	7.33158224	2.36E-10	1.52E-08	13.3757833	ADRM1	assignmentsChromaffin_cells.Chrom_SOX2_statusHigh
assignmentsChromaffin_cells.Chrom_SOX2_statusHigh.SNRPD2	2.99489796	4.78602403	7.33437373	2.34E-10	1.52E-08	13.3569797	SNRPD2	assignmentsChromaffin_cells.Chrom_SOX2_statusHigh
assignmentsChromaffin_cells.Chrom_SOX2_statusHigh.CIB1	2.85219749	4.03137172	7.32853705	2.39E-10	1.54E-08	13.3558859	CIB1	assignmentsChromaffin_cells.Chrom_SOX2_statusHigh
assignmentsChromaffin_cells.Chrom_SOX2_statusHigh.TRIR	2.23238807	5.65162837	7.3326076	2.35E-10	1.52E-08	13.3434226	TRIR	assignmentsChromaffin_cells.Chrom_SOX2_statusHigh
assignmentsChromaffin_cells.Chrom_SOX2_statusHigh.CCNQ	2.72404054	2.23803191	7.3142914	2.54E-10	1.62E-08	13.3124855	CCNQ	assignmentsChromaffin_cells.Chrom_SOX2_statusHigh
assignmentsChromaffin_cells.Chrom_SOX2_statusHigh.RNF187	2.30103077	4.40568799	7.31852914	2.49E-10	1.59E-08	13.2986439	RNF187	assignmentsChromaffin_cells.Chrom_SOX2_statusHigh
assignmentsChromaffin_cells.Chrom_SOX2_statusHigh.EIF3G	2.72923045	4.59241302	7.31010408	2.59E-10	1.63E-08	13.2653382	EIF3G	assignmentsChromaffin_cells.Chrom_SOX2_statusHigh
assignmentsChromaffin_cells.Chrom_SOX2_statusHigh.SERPINB1	3.55456949	4.4830543	7.30125591	2.70E-10	1.67E-08	13.2478588	SERPINB1	assignmentsChromaffin_cells.Chrom_SOX2_statusHigh
assignmentsChromaffin_cells.Chrom_SOX2_statusHigh.FAM174C	2.85207398	3.04353154	7.29914654	2.71E-10	1.67E-08	13.2442531	FAM174C	assignmentsChromaffin_cells.Chrom_SOX2_statusHigh

assignmentsChromaffin_cells.Chrom_SOX2_statusHigh.ILF2	1.94822096	4.90300324	7.30280545	2.67E-10	1.67E-08	13.2292138	ILF2	assignmentsChromaffin_cells.Chrom_SOX2_statusHigh
assignmentsChromaffin_cells.Chrom_SOX2_statusHigh.SSR4	4.26470498	5.78415355	7.31314339	2.59E-10	1.63E-08	13.2260625	SSR4	assignmentsChromaffin_cells.Chrom_SOX2_statusHigh
assignmentsChromaffin_cells.Chrom_SOX2_statusHigh.COX5A	2.30829595	4.8938351	7.30156977	2.68E-10	1.67E-08	13.222961	COX5A	assignmentsChromaffin_cells.Chrom_SOX2_statusHigh
assignmentsChromaffin_cells.Chrom_SOX2_statusHigh.FARS2	-1.3938017	7.61114787	-7.3022472	2.68E-10	1.67E-08	13.2027282	FARS2	assignmentsChromaffin_cells.Chrom_SOX2_statusHigh
assignmentsChromaffin_cells.Chrom_SOX2_statusHigh.RNF181	3.13407052	3.44433157	7.2908572	2.82E-10	1.73E-08	13.2026657	RNF181	assignmentsChromaffin_cells.Chrom_SOX2_statusHigh
assignmentsChromaffin_cells.Chrom_SOX2_statusHigh.PLD3	2.14750004	7.22143178	7.31106567	2.58E-10	1.63E-08	13.2000756	PLD3	assignmentsChromaffin_cells.Chrom_SOX2_statusHigh
assignmentsChromaffin_cells.Chrom_SOX2_statusHigh.GPN3	2.22544097	3.1053154	7.28463043	2.89E-10	1.76E-08	13.1833666	GPN3	assignmentsChromaffin_cells.Chrom_SOX2_statusHigh
assignmentsChromaffin_cells.Chrom_SOX2_statusHigh.STUB1	1.99643418	4.78813288	7.28636449	2.87E-10	1.75E-08	13.159079	STUB1	assignmentsChromaffin_cells.Chrom_SOX2_statusHigh
assignmentsChromaffin_cells.Chrom_SOX2_statusHigh.TPGS1	2.16112503	3.55329069	7.2804808	2.94E-10	1.78E-08	13.1581944	TPGS1	assignmentsChromaffin_cells.Chrom_SOX2_statusHigh
assignmentsChromaffin_cells.Chrom_SOX2_statusHigh.RPS19BP1	2.57505385	3.67880063	7.27851201	2.96E-10	1.79E-08	13.1518902	RPS19BP1	assignmentsChromaffin_cells.Chrom_SOX2_statusHigh
assignmentsChromaffin_cells.Chrom_SOX2_statusHigh.C1QBP	2.71183535	3.98188444	7.27613137	2.99E-10	1.80E-08	13.1332836	C1QBP	assignmentsChromaffin_cells.Chrom_SOX2_statusHigh
assignmentsChromaffin_cells.Chrom_SOX2_statusHigh.MPPED2	-5.7937761	5.27187304	-7.2777962	3.01E-10	1.80E-08	13.130543	MPPED2	assignmentsChromaffin_cells.Chrom_SOX2_statusHigh
assignmentsChromaffin_cells.Chrom_SOX2_statusHigh.TIMP1	4.34353275	6.02941989	7.28461054	2.93E-10	1.78E-08	13.1260842	TIMP1	assignmentsChromaffin_cells.Chrom_SOX2_statusHigh
assignmentsChromaffin_cells.Chrom_SOX2_statusHigh.UBL5	4.01429137	5.15777934	7.27834326	3.01E-10	1.80E-08	13.0995931	UBL5	assignmentsChromaffin_cells.Chrom_SOX2_statusHigh
assignmentsChromaffin_cells.Chrom_SOX2_statusHigh.RABAC1	3.41592781	5.45219023	7.27463593	3.05E-10	1.81E-08	13.0778943	RABAC1	assignmentsChromaffin_cells.Chrom_SOX2_statusHigh
assignmentsChromaffin_cells.Chrom_SOX2_statusHigh.PPP4R1	-1.1877519	6.33758604	-7.2608906	3.20E-10	1.89E-08	13.0600259	PPP4R1	assignmentsChromaffin_cells.Chrom_SOX2_statusHigh
assignmentsChromaffin_cells.Chrom_SOX2_statusHigh.MRPS26	2.16424936	3.84131999	7.25610196	3.27E-10	1.92E-08	13.0503305	MRPS26	assignmentsChromaffin_cells.Chrom_SOX2_statusHigh
assignmentsChromaffin_cells.Chrom_SOX2_statusHigh.ADIPOR1	1.53244718	4.86966116	7.25887685	3.23E-10	1.90E-08	13.0464306	ADIPOR1	assignmentsChromaffin_cells.Chrom_SOX2_statusHigh
assignmentsChromaffin_cells.Chrom_SOX2_statusHigh.RTL8C	2.42106153	4.63613922	7.25554199	3.27E-10	1.92E-08	13.029538	RTL8C	assignmentsChromaffin_cells.Chrom_SOX2_statusHigh
assignmentsChromaffin_cells.Chrom_SOX2_statusHigh.ROMO1	2.96933737	4.00023581	7.24913761	3.38E-10	1.96E-08	13.0147759	ROMO1	assignmentsChromaffin_cells.Chrom_SOX2_statusHigh
assignmentsChromaffin_cells.Chrom_SOX2_statusHigh.FIS1	2.75137901	4.64925532	7.25037529	3.36E-10	1.96E-08	13.0063994	FIS1	assignmentsChromaffin_cells.Chrom_SOX2_statusHigh
assignmentsChromaffin_cells.Chrom_SOX2_statusHigh.POLR2I	3.8292204	3.14784361	7.241346	3.53E-10	2.04E-08	12.9849587	POLR2I	assignmentsChromaffin_cells.Chrom_SOX2_statusHigh
assignmentsChromaffin_cells.Chrom_SOX2_statusHigh.TECR	2.1799902	4.87320191	7.23699331	3.55E-10	2.05E-08	12.9554111	TECR	assignmentsChromaffin_cells.Chrom_SOX2_statusHigh
assignmentsChromaffin_cells.Chrom_SOX2_statusHigh.TFPI2	8.23492518	1.75333101	7.23490738	3.62E-10	2.08E-08	12.9322696	TFPI2	assignmentsChromaffin_cells.Chrom_SOX2_statusHigh
assignmentsChromaffin_cells.Chrom_SOX2_statusHigh.MPV17L2	2.81225651	1.63842528	7.22488077	3.74E-10	2.13E-08	12.9280419	MPV17L2	assignmentsChromaffin_cells.Chrom_SOX2_statusHigh
assignmentsChromaffin_cells.Chrom_SOX2_statusHigh.NEDD8	2.11080971	5.30941094	7.23164908	3.63E-10	2.08E-08	12.9219172	NEDD8	assignmentsChromaffin_cells.Chrom_SOX2_statusHigh
assignmentsChromaffin_cells.Chrom_SOX2_statusHigh.ACVR2A	-1.5153227	5.82113329	-7.2213394	3.79E-10	2.16E-08	12.900736	ACVR2A	assignmentsChromaffin_cells.Chrom_SOX2_statusHigh
assignmentsChromaffin_cells.Chrom_SOX2_statusHigh.TUSC1	3.41173632	1.31159196	7.21356219	3.92E-10	2.18E-08	12.884366	TUSC1	assignmentsChromaffin_cells.Chrom_SOX2_statusHigh
assignmentsChromaffin_cells.Chrom_SOX2_statusHigh.BUD31	2.41446738	3.80058485	7.21578954	3.89E-10	2.18E-08	12.8808884	BUD31	assignmentsChromaffin_cells.Chrom_SOX2_statusHigh
assignmentsChromaffin_cells.Chrom_SOX2_statusHigh.SHISA5	1.80234859	4.80501746	7.21485116	3.90E-10	2.18E-08	12.8799939	SHISA5	assignmentsChromaffin_cells.Chrom_SOX2_statusHigh
assignmentsChromaffin_cells.Chrom_SOX2_statusHigh.SHARPIN	1.63473339	4.14995735	7.21508173	3.90E-10	2.18E-08	12.8797961	SHARPIN	assignmentsChromaffin_cells.Chrom_SOX2_statusHigh
assignmentsChromaffin_cells.Chrom_SOX2_statusHigh.DIS3L2	-1.3826438	6.88394539	-7.2202256	3.81E-10	2.16E-08	12.8700702	DIS3L2	assignmentsChromaffin_cells.Chrom_SOX2_statusHigh
assignmentsChromaffin_cells.Chrom_SOX2_statusHigh.GEMIN7	2.07046951	1.92268676	7.2052744	4.07E-10	2.23E-08	12.8595607	GEMIN7	assignmentsChromaffin_cells.Chrom_SOX2_statusHigh
assignmentsChromaffin_cells.Chrom_SOX2_statusHigh.CREB3	1.94462699	3.48475168	7.20819042	4.01E-10	2.21E-08	12.8590934	CREB3	assignmentsChromaffin_cells.Chrom_SOX2_statusHigh
assignmentsChromaffin_cells.Chrom_SOX2_statusHigh.NUCB1	1.90080194	5.45903406	7.2125765	3.94E-10	2.19E-08	12.8435716	NUCB1	assignmentsChromaffin_cells.Chrom_SOX2_statusHigh
assignmentsChromaffin_cells.Chrom_SOX2_statusHigh.OTUD6B-AS	2.40901374	5.01566547	7.21114887	3.96E-10	2.19E-08	12.8343914	OTUD6B-AS1	assignmentsChromaffin_cells.Chrom_SOX2_statusHigh
assignmentsChromaffin_cells.Chrom_SOX2_statusHigh.TMEM141	2.38101695	3.27119428	7.19589525	4.23E-10	2.31E-08	12.8066749	TMEM141	assignmentsChromaffin_cells.Chrom_SOX2_statusHigh
assignmentsChromaffin_cells.Chrom_SOX2_statusHigh.DCXR	2.71370756	3.51455581	7.1930745	4.29E-10	2.33E-08	12.7881444	DCXR	assignmentsChromaffin_cells.Chrom_SOX2_statusHigh
assignmentsChromaffin_cells.Chrom_SOX2_statusHigh.CARD19	2.16200356	3.51992507	7.18613728	4.42E-10	2.38E-08	12.7794766	CARD19	assignmentsChromaffin_cells.Chrom_SOX2_statusHigh
assignmentsChromaffin_cells.Chrom_SOX2_statusHigh.SPOCK2	4.64700459	3.22285069	7.18786017	4.44E-10	2.39E-08	12.7680438	SPOCK2	assignmentsChromaffin_cells.Chrom_SOX2_statusHigh
assignmentsChromaffin_cells.Chrom_SOX2_statusHigh.GABARAP	3.28937751	5.79697298	7.20043973	4.21E-10	2.30E-08	12.7619313	GABARAP	assignmentsChromaffin_cells.Chrom_SOX2_statusHigh
assignmentsChromaffin_cells.Chrom_SOX2_statusHigh.MRPS33	2.05633024	3.40900371	7.18154837	4.50E-10	2.42E-08	12.7415761	MRPS33	assignmentsChromaffin_cells.Chrom_SOX2_statusHigh
assignmentsChromaffin_cells.Chrom_SOX2_statusHigh.PRDX2	3.38007962	5.15754633	7.19256368	4.35E-10	2.36E-08	12.732696	PRDX2	assignmentsChromaffin_cells.Chrom_SOX2_statusHigh
assignmentsChromaffin_cells.Chrom_SOX2_statusHigh.HDDC2	2.27179956	3.91282403	7.17798596	4.57E-10	2.44E-08	12.7253555	HDDC2	assignmentsChromaffin_cells.Chrom_SOX2_statusHigh
assignmentsChromaffin_cells.Chrom_SOX2_statusHigh.S100A16	3.89207542	2.14585417	7.17421243	4.67E-10	2.49E-08	12.7138846	S100A16	assignmentsChromaffin_cells.Chrom_SOX2_statusHigh
assignmentsChromaffin_cells.Chrom_SOX2_statusHigh.ZNF581	2.51293158	1.81424363	7.16696527	4.80E-10	2.51E-08	12.7000009	ZNF581	assignmentsChromaffin_cells.Chrom_SOX2_statusHigh
assignmentsChromaffin_cells.Chrom_SOX2_statusHigh.TSEN34	2.57815835	3.70796172	7.17034917	4.73E-10	2.51E-08	12.6955776	TSEN34	assignmentsChromaffin_cells.Chrom_SOX2_statusHigh
assignmentsChromaffin_cells.Chrom_SOX2_statusHigh.MRPL9	1.58053384	3.48124261	7.16764541	4.78E-10	2.51E-08	12.693794	MRPL9	assignmentsChromaffin_cells.Chrom_SOX2_statusHigh
assignmentsChromaffin_cells.Chrom_SOX2_statusHigh.C11orf87	4.57977268	-0.6865366	7.21652996	3.87E-10	2.18E-08	12.6934225	C11orf87	assignmentsChromaffin_cells.Chrom_SOX2_statusHigh
assignmentsChromaffin_cells.Chrom_SOX2_statusHigh.CHCHD4	1.73544714	1.91492362	7.16529964	4.83E-10	2.51E-08	12.692408	CHCHD4	assignmentsChromaffin_cells.Chrom_SOX2_statusHigh
assignmentsChromaffin_cells.Chrom_SOX2_statusHigh.ISG15	3.47335759	3.54182717	7.16647503	4.82E-10	2.51E-08	12.6922744	ISG15	assignmentsChromaffin_cells.Chrom_SOX2_statusHigh
assignmentsChromaffin_cells.Chrom_SOX2_statusHigh.PELO	2.29095771	3.90650139	7.16502928	4.84E-10	2.51E-08	12.6750325	PELO	assignmentsChromaffin_cells.Chrom_SOX2_statusHigh
assignmentsChromaffin_cells.Chrom_SOX2_statusHigh.ZNF22	2.14108241	3.74096807	7.16416641	4.85E-10	2.51E-08	12.6733081	ZNF22	assignmentsChromaffin_cells.Chrom_SOX2_statusHigh
assignmentsChromaffin_cells.Chrom_SOX2_statusHigh.ATP5PD	2.60505153	4.92994441	7.1704966	4.74E-10	2.51E-08	12.6662533	ATP5PD	assignmentsChromaffin_cells.Chrom_SOX2_statusHigh
assignmentsChromaffin_cells.Chrom_SOX2_statusHigh.SOD1	2.6544147	5.79085834	7.16873929	4.78E-10	2.51E-08	12.6405953	SOD1	assignmentsChromaffin_cells.Chrom_SOX2_statusHigh
assignmentsChromaffin_cells.Chrom_SOX2_statusHigh.DNAJC19	1.91761196	4.29170174	7.15891781	4.96E-10	2.54E-08	12.6382667	DNAJC19	assignmentsChromaffin_cells.Chrom_SOX2_statusHigh
assignmentsChromaffin_cells.Chrom_SOX2_statusHigh.AKR7A2	2.40272365	3.00705955	7.15166457	5.12E-10	2.62E-08	12.630037	AKR7A2	assignmentsChromaffin_cells.Chrom_SOX2_statusHigh
assignmentsChromaffin_cells.Chrom_SOX2_statusHigh.ARL6IP1	2.9334492	5.44723276	7.16240144	4.92E-10	2.54E-08	12.6227628	ARL6IP1	assignmentsChromaffin_cells.Chrom_SOX2_statusHigh
assignmentsChromaffin_cells.Chrom_SOX2_statusHigh.RAN	2.92568907	5.16422767	7.16028284	4.96E-10	2.54E-08	12.6208262	RAN	assignmentsChromaffin_cells.Chrom_SOX2_statusHigh
assignmentsChromaffin_cells.Chrom_SOX2_statusHigh.CHCHD1	2.72297172	3.27487217	7.14848072	5.19E-10	2.63E-08	12.6125188	CHCHD1	assignmentsChromaffin_cells.Chrom_SOX2_statusHigh
assignmentsChromaffin_cells.Chrom_SOX2_statusHigh.POLR2E	2.10675814	4.10070177	7.14856438	5.19E-10	2.63E-08	12.5968868	POLR2E	assignmentsChromaffin_cells.Chrom_SOX2_statusHigh
assignmentsChromaffin_cells.Chrom_SOX2_statusHigh.WDSUB1	-1.4462753	5.39635249	-7.147896	5.21E-10	2.63E-08	12.5927538	WDSUB1	assignmentsChromaffin_cells.Chrom_SOX2_statusHigh
assignmentsChromaffin_cells.Chrom_SOX2_statusHigh.LRPAP1	2.19740671	5.3611491	7.14794139	5.20E-10	2.63E-08	12.565908	LRPAP1	assignmentsChromaffin_cells.Chrom_SOX2_statusHigh
assignmentsChromaffin_cells.Chrom_SOX2_statusHigh.LLPH	1.79641539	3.75563337	7.13664095	5.46E-10	2.74E-08	12.5603147	LLPH	assignmentsChromaffin_cells.Chrom_SOX2_statusHigh

assignmentsChromaffin_cells.Chrom_SOX2_statusHigh.THEM6	2.21440445	2.07738799	7.13116171	5.59E-10	2.77E-08	12.5514745	THEM6	assignmentsChromaffin_cells.Chrom_SOX2_statusHigh
assignmentsChromaffin_cells.Chrom_SOX2_statusHigh.EXOSC5	2.74278796	1.76401394	7.13107159	5.60E-10	2.77E-08	12.5508236	EXOSC5	assignmentsChromaffin_cells.Chrom_SOX2_statusHigh
assignmentsChromaffin_cells.Chrom_SOX2_statusHigh.RNF113A	2.40360171	2.33154963	7.12659007	5.71E-10	2.81E-08	12.5325682	RNF113A	assignmentsChromaffin_cells.Chrom_SOX2_statusHigh
assignmentsChromaffin_cells.Chrom_SOX2_statusHigh.SLC7A4	3.10597938	0.13073725	7.13197278	5.58E-10	2.77E-08	12.5319963	SLC7A4	assignmentsChromaffin_cells.Chrom_SOX2_statusHigh
assignmentsChromaffin_cells.Chrom_SOX2_statusHigh.SMG1	-1.0822056	7.18347273	-7.1353107	5.50E-10	2.75E-08	12.5133549	SMG1	assignmentsChromaffin_cells.Chrom_SOX2_statusHigh
assignmentsChromaffin_cells.Chrom_SOX2_statusHigh.MRPL17	2.75835611	3.02674297	7.12292818	5.80E-10	2.84E-08	12.5111228	MRPL17	assignmentsChromaffin_cells.Chrom_SOX2_statusHigh
assignmentsChromaffin_cells.Chrom_SOX2_statusHigh.PDIA6	2.0571221	5.97560506	7.12680196	5.70E-10	2.81E-08	12.4695764	PDIA6	assignmentsChromaffin_cells.Chrom_SOX2_statusHigh
assignmentsChromaffin_cells.Chrom_SOX2_statusHigh.IFT22	2.23766722	2.36266549	7.11027458	6.12E-10	2.97E-08	12.4589397	IFT22	assignmentsChromaffin_cells.Chrom_SOX2_statusHigh
assignmentsChromaffin_cells.Chrom_SOX2_statusHigh.EIF1	3.32848588	7.87597092	7.14063172	5.44E-10	2.73E-08	12.4578632	EIF1	assignmentsChromaffin_cells.Chrom_SOX2_statusHigh
assignmentsChromaffin_cells.Chrom_SOX2_statusHigh.UBQLN2	1.67924757	3.91035385	7.11380565	6.03E-10	2.94E-08	12.4560987	UBQLN2	assignmentsChromaffin_cells.Chrom_SOX2_statusHigh
assignmentsChromaffin_cells.Chrom_SOX2_statusHigh.SIGMAR1	1.96683727	2.96912849	7.10825736	6.17E-10	2.98E-08	12.4522239	SIGMAR1	assignmentsChromaffin_cells.Chrom_SOX2_statusHigh
assignmentsChromaffin_cells.Chrom_SOX2_statusHigh.HAX1	1.95180655	4.7938839	7.11485472	6.00E-10	2.93E-08	12.438517	HAX1	assignmentsChromaffin_cells.Chrom_SOX2_statusHigh
assignmentsChromaffin_cells.Chrom_SOX2_statusHigh.NAV3	-4.7044128	7.06623574	-7.1155586	6.06E-10	2.94E-08	12.4357477	NAV3	assignmentsChromaffin_cells.Chrom_SOX2_statusHigh
assignmentsChromaffin_cells.Chrom_SOX2_statusHigh.COA8	1.52458721	3.70912862	7.10483546	6.27E-10	3.02E-08	12.4246153	COA8	assignmentsChromaffin_cells.Chrom_SOX2_statusHigh
assignmentsChromaffin_cells.Chrom_SOX2_statusHigh.SLC10A3	2.66786475	1.91262798	7.09568189	6.52E-10	3.10E-08	12.4024486	SLC10A3	assignmentsChromaffin_cells.Chrom_SOX2_statusHigh
assignmentsChromaffin_cells.Chrom_SOX2_statusHigh.POP7	3.12995074	2.19340293	7.0961798	6.52E-10	3.10E-08	12.4018718	POP7	assignmentsChromaffin_cells.Chrom_SOX2_statusHigh
assignmentsChromaffin_cells.Chrom_SOX2_statusHigh.POLR2L	3.41030242	4.08194356	7.10202258	6.40E-10	3.07E-08	12.395703	POLR2L	assignmentsChromaffin_cells.Chrom_SOX2_statusHigh
assignmentsChromaffin_cells.Chrom_SOX2_statusHigh.DCTPP1	2.91849964	1.91477938	7.08750197	6.75E-10	3.18E-08	12.3700836	DCTPP1	assignmentsChromaffin_cells.Chrom_SOX2_statusHigh
assignmentsChromaffin_cells.Chrom_SOX2_statusHigh.TMBIM6	2.11114507	6.54645531	7.10226035	6.34E-10	3.04E-08	12.3564536	TMBIM6	assignmentsChromaffin_cells.Chrom_SOX2_statusHigh
assignmentsChromaffin_cells.Chrom_SOX2_statusHigh.UFC1	2.60504898	4.48652229	7.08777261	6.75E-10	3.18E-08	12.3350226	UFC1	assignmentsChromaffin_cells.Chrom_SOX2_statusHigh
assignmentsChromaffin_cells.Chrom_SOX2_statusHigh.TCN2	3.7501292	1.80848117	7.09335699	6.58E-10	3.12E-08	12.3304085	TCN2	assignmentsChromaffin_cells.Chrom_SOX2_statusHigh
assignmentsChromaffin_cells.Chrom_SOX2_statusHigh.ZNHIT2	2.90410888	1.24440242	7.07653196	7.08E-10	3.29E-08	12.3190953	ZNHIT2	assignmentsChromaffin_cells.Chrom_SOX2_statusHigh
assignmentsChromaffin_cells.Chrom_SOX2_statusHigh.MYD88	2.31478699	2.24200282	7.0764278	7.08E-10	3.29E-08	12.3142444	MYD88	assignmentsChromaffin_cells.Chrom_SOX2_statusHigh
assignmentsChromaffin_cells.Chrom_SOX2_statusHigh.BAD	1.73643557	3.49652263	7.07639419	7.08E-10	3.29E-08	12.3082313	BAD	assignmentsChromaffin_cells.Chrom_SOX2_statusHigh
assignmentsChromaffin_cells.Chrom_SOX2_statusHigh.NDN	3.58362677	2.61592928	7.07639739	7.15E-10	3.31E-08	12.3041673	NDN	assignmentsChromaffin_cells.Chrom_SOX2_statusHigh
assignmentsChromaffin_cells.Chrom_SOX2_statusHigh.ATP6V0B	3.13831727	5.02517852	7.08387859	6.92E-10	3.25E-08	12.3001438	ATP6V0B	assignmentsChromaffin_cells.Chrom_SOX2_statusHigh
assignmentsChromaffin_cells.Chrom_SOX2_statusHigh.ATP5MF	2.75302528	4.47220522	7.07485325	7.15E-10	3.31E-08	12.2761371	ATP5MF	assignmentsChromaffin_cells.Chrom_SOX2_statusHigh
assignmentsChromaffin_cells.Chrom_SOX2_statusHigh.FAM32A	2.13672186	3.39248441	7.06685286	7.38E-10	3.40E-08	12.2737797	FAM32A	assignmentsChromaffin_cells.Chrom_SOX2_statusHigh
assignmentsChromaffin_cells.Chrom_SOX2_statusHigh.SPECC1L	-1.3004125	7.51725991	-7.077737	7.04E-10	3.29E-08	12.2558899	SPECC1L	assignmentsChromaffin_cells.Chrom_SOX2_statusHigh
assignmentsChromaffin_cells.Chrom_SOX2_statusHigh.SMG8	1.81893638	1.87981954	7.0595347	7.61E-10	3.46E-08	12.2557115	SMG8	assignmentsChromaffin_cells.Chrom_SOX2_statusHigh
assignmentsChromaffin_cells.Chrom_SOX2_statusHigh.PPP1R3D	2.21936087	1.82145723	7.05904142	7.63E-10	3.46E-08	12.2455623	PPP1R3D	assignmentsChromaffin_cells.Chrom_SOX2_statusHigh
assignmentsChromaffin_cells.Chrom_SOX2_statusHigh.BANF1	2.60770553	4.35833894	7.06468188	7.45E-10	3.42E-08	12.2442226	BANF1	assignmentsChromaffin_cells.Chrom_SOX2_statusHigh
assignmentsChromaffin_cells.Chrom_SOX2_statusHigh.SNHG7	2.55633335	4.94828045	7.06514219	7.45E-10	3.42E-08	12.2258122	SNHG7	assignmentsChromaffin_cells.Chrom_SOX2_statusHigh
assignmentsChromaffin_cells.Chrom_SOX2_statusHigh.MICOS13	2.80925003	4.54386676	7.06287983	7.54E-10	3.44E-08	12.2199399	MICOS13	assignmentsChromaffin_cells.Chrom_SOX2_statusHigh
assignmentsChromaffin_cells.Chrom_SOX2_statusHigh.HDDC3	1.94488271	2.65962558	7.05268798	7.84E-10	3.52E-08	12.2181067	HDDC3	assignmentsChromaffin_cells.Chrom_SOX2_statusHigh
assignmentsChromaffin_cells.Chrom_SOX2_statusHigh.SMPD1	1.97639296	2.97606711	7.05232512	7.85E-10	3.52E-08	12.2175628	SMPD1	assignmentsChromaffin_cells.Chrom_SOX2_statusHigh
assignmentsChromaffin_cells.Chrom_SOX2_statusHigh.TBCB	2.47630073	4.67957409	7.0560224	7.73E-10	3.48E-08	12.1950387	TBCB	assignmentsChromaffin_cells.Chrom_SOX2_statusHigh
assignmentsChromaffin_cells.Chrom_SOX2_statusHigh.ERLEC1	1.65305468	6.01253507	7.05933453	7.62E-10	3.46E-08	12.1804127	ERLEC1	assignmentsChromaffin_cells.Chrom_SOX2_statusHigh
assignmentsChromaffin_cells.Chrom_SOX2_statusHigh.P4HB	1.76796706	6.06959667	7.05714637	7.69E-10	3.48E-08	12.1740166	P4HB	assignmentsChromaffin_cells.Chrom_SOX2_statusHigh
assignmentsChromaffin_cells.Chrom_SOX2_statusHigh.RDH14	1.5762132	3.83086292	7.04447303	8.12E-10	3.61E-08	12.1690094	RDH14	assignmentsChromaffin_cells.Chrom_SOX2_statusHigh
assignmentsChromaffin_cells.Chrom_SOX2_statusHigh.HSPB6	4.73330912	1.02258979	7.04647389	8.11E-10	3.61E-08	12.161163	HSPB6	assignmentsChromaffin_cells.Chrom_SOX2_statusHigh
assignmentsChromaffin_cells.Chrom_SOX2_statusHigh.PIN1	1.86330693	4.5067639	7.0465023	8.05E-10	3.60E-08	12.1589302	PIN1	assignmentsChromaffin_cells.Chrom_SOX2_statusHigh
assignmentsChromaffin_cells.Chrom_SOX2_statusHigh.JAGN1	2.50718369	2.87274536	7.03237662	8.56E-10	3.77E-08	12.1331435	JAGN1	assignmentsChromaffin_cells.Chrom_SOX2_statusHigh
assignmentsChromaffin_cells.Chrom_SOX2_statusHigh.SCOC	2.34304538	4.87888613	7.04079427	8.25E-10	3.66E-08	12.1154804	SCOC	assignmentsChromaffin_cells.Chrom_SOX2_statusHigh
assignmentsChromaffin_cells.Chrom_SOX2_statusHigh.COPE	2.14115039	5.50781732	7.03894091	8.32E-10	3.68E-08	12.1059633	COPE	assignmentsChromaffin_cells.Chrom_SOX2_statusHigh
assignmentsChromaffin_cells.Chrom_SOX2_statusHigh.POLR2J	2.84521669	3.8077553	7.02972355	8.68E-10	3.82E-08	12.0952545	POLR2J	assignmentsChromaffin_cells.Chrom_SOX2_statusHigh
assignmentsChromaffin_cells.Chrom_SOX2_statusHigh.PCGF6	-1.2559406	4.74146258	-7.0235422	8.89E-10	3.88E-08	12.0902614	PCGF6	assignmentsChromaffin_cells.Chrom_SOX2_statusHigh
assignmentsChromaffin_cells.Chrom_SOX2_statusHigh.RAB3A	2.77386484	2.18241601	7.01961756	9.04E-10	3.93E-08	12.0825545	RAB3A	assignmentsChromaffin_cells.Chrom_SOX2_statusHigh
assignmentsChromaffin_cells.Chrom_SOX2_statusHigh.CNIH4	1.20557107	5.05384991	7.02347178	8.89E-10	3.88E-08	12.0591847	CNIH4	assignmentsChromaffin_cells.Chrom_SOX2_statusHigh
assignmentsChromaffin_cells.Chrom_SOX2_statusHigh.MTX1	2.01590423	3.01995781	7.01302433	9.30E-10	4.03E-08	12.0519686	MTX1	assignmentsChromaffin_cells.Chrom_SOX2_statusHigh
assignmentsChromaffin_cells.Chrom_SOX2_statusHigh.TBC1D22B	-1.4333847	5.03447152	-7.012123	9.33E-10	4.03E-08	12.0430618	TBC1D22B	assignmentsChromaffin_cells.Chrom_SOX2_statusHigh
assignmentsChromaffin_cells.Chrom_SOX2_statusHigh.PARK7	2.77143074	5.28620548	7.02072932	9.04E-10	3.93E-08	12.0377534	PARK7	assignmentsChromaffin_cells.Chrom_SOX2_statusHigh
assignmentsChromaffin_cells.Chrom_SOX2_statusHigh.IGFBP2	4.65900082	2.24446375	7.00207788	9.86E-10	4.23E-08	12.0040673	IGFBP2	assignmentsChromaffin_cells.Chrom_SOX2_statusHigh
assignmentsChromaffin_cells.Chrom_SOX2_statusHigh.PRKCSH	1.7846207	4.520953	7.00361584	9.68E-10	4.17E-08	11.989398	PRKCSH	assignmentsChromaffin_cells.Chrom_SOX2_statusHigh
assignmentsChromaffin_cells.Chrom_SOX2_statusHigh.LAMTOR5	2.8137911	4.25960735	7.00166226	9.80E-10	4.21E-08	11.9786884	LAMTOR5	assignmentsChromaffin_cells.Chrom_SOX2_statusHigh
assignmentsChromaffin_cells.Chrom_SOX2_statusHigh.TWF2	2.42596086	3.38319975	6.99173099	1.02E-09	4.35E-08	11.9677014	TWF2	assignmentsChromaffin_cells.Chrom_SOX2_statusHigh
assignmentsChromaffin_cells.Chrom_SOX2_statusHigh.SRI	1.83005121	5.40011844	6.99502063	1.00E-09	4.30E-08	11.9265444	SRI	assignmentsChromaffin_cells.Chrom_SOX2_statusHigh
assignmentsChromaffin_cells.Chrom_SOX2_statusHigh.BAG5	1.29402584	4.32575476	6.97808904	1.08E-09	4.56E-08	11.891624	BAG5	assignmentsChromaffin_cells.Chrom_SOX2_statusHigh
assignmentsChromaffin_cells.Chrom_SOX2_statusHigh.ZNF236	-1.3332637	5.65888535	-6.9784895	1.08E-09	4.56E-08	11.8914809	ZNF236	assignmentsChromaffin_cells.Chrom_SOX2_statusHigh
assignmentsChromaffin_cells.Chrom_SOX2_statusHigh.PAK5	-5.2068952	2.31537825	-6.9833322	1.07E-09	4.54E-08	11.8827506	PAK5	assignmentsChromaffin_cells.Chrom_SOX2_statusHigh
assignmentsChromaffin_cells.Chrom_SOX2_statusHigh.DLX2	2.98234782	1.30744266	6.96757666	1.13E-09	4.71E-08	11.8757674	DLX2	assignmentsChromaffin_cells.Chrom_SOX2_statusHigh
assignmentsChromaffin_cells.Chrom_SOX2_statusHigh.TOMM22	3.07350038	2.91840355	6.97026591	1.12E-09	4.68E-08	11.8708032	TOMM22	assignmentsChromaffin_cells.Chrom_SOX2_statusHigh
assignmentsChromaffin_cells.Chrom_SOX2_statusHigh.NRF1	-1.4332565	6.68934527	-6.976064	1.09E-09	4.59E-08	11.8556898	NRF1	assignmentsChromaffin_cells.Chrom_SOX2_statusHigh
assignmentsChromaffin_cells.Chrom_SOX2_statusHigh.ZNF428	2.16972851	4.29430046	6.97281038	1.10E-09	4.63E-08	11.8521201	ZNF428	assignmentsChromaffin_cells.Chrom_SOX2_statusHigh

assignmentsChromaffin_cells.Chrom_SOX2_statusHigh.SELENOH	2.09144486	5.2991648	6.97546804	1.09E-09	4.59E-08	11.8505447	SELENOH	assignmentsChromaffin_cells.Chrom_SOX2_statusHigh
assignmentsChromaffin_cells.Chrom_SOX2_statusHigh.PBDC1	2.29492299	3.24687961	6.95919207	1.17E-09	4.83E-08	11.8334235	PBDC1	assignmentsChromaffin_cells.Chrom_SOX2_statusHigh
assignmentsChromaffin_cells.Chrom_SOX2_statusHigh.MZT1	1.71507697	3.6941781	6.95840872	1.18E-09	4.83E-08	11.8105208	MZT1	assignmentsChromaffin_cells.Chrom_SOX2_statusHigh
assignmentsChromaffin_cells.Chrom_SOX2_statusHigh.RPL19	3.95206219	7.31492275	6.98154947	1.08E-09	4.56E-08	11.809349	RPL19	assignmentsChromaffin_cells.Chrom_SOX2_statusHigh
assignmentsChromaffin_cells.Chrom_SOX2_statusHigh.MESD	1.37122923	5.52474789	6.96549882	1.14E-09	4.74E-08	11.8088577	MESD	assignmentsChromaffin_cells.Chrom_SOX2_statusHigh
assignmentsChromaffin_cells.Chrom_SOX2_statusHigh.PCBD1	2.95153118	4.18021552	6.96554808	1.15E-09	4.75E-08	11.8068287	PCBD1	assignmentsChromaffin_cells.Chrom_SOX2_statusHigh
assignmentsChromaffin_cells.Chrom_SOX2_statusHigh.RIIAD1	2.7992713	1.18610152	6.95064655	1.22E-09	4.95E-08	11.8052905	RIIAD1	assignmentsChromaffin_cells.Chrom_SOX2_statusHigh
assignmentsChromaffin_cells.Chrom_SOX2_statusHigh.NOP10	3.17179107	3.84848722	6.953724	1.21E-09	4.92E-08	11.7832059	NOP10	assignmentsChromaffin_cells.Chrom_SOX2_statusHigh
assignmentsChromaffin_cells.Chrom_SOX2_statusHigh.TMEM256	2.96194388	3.17906465	6.94842852	1.23E-09	4.97E-08	11.7784387	TMEM256	assignmentsChromaffin_cells.Chrom_SOX2_statusHigh
assignmentsChromaffin_cells.Chrom_SOX2_statusHigh.TRMT112	3.4979791	4.8919493	6.95963395	1.18E-09	4.85E-08	11.7742023	TRMT112	assignmentsChromaffin_cells.Chrom_SOX2_statusHigh
assignmentsChromaffin_cells.Chrom_SOX2_statusHigh.MLEC	1.65862813	5.78474452	6.95875964	1.17E-09	4.83E-08	11.7722893	MLEC	assignmentsChromaffin_cells.Chrom_SOX2_statusHigh
assignmentsChromaffin_cells.Chrom_SOX2_statusHigh.ZNF574	1.44130483	2.39673276	6.94070689	1.27E-09	5.08E-08	11.7592689	ZNF574	assignmentsChromaffin_cells.Chrom_SOX2_statusHigh
assignmentsChromaffin_cells.Chrom_SOX2_statusHigh.MRPL43	2.33077069	4.00117975	6.94792049	1.23E-09	4.97E-08	11.7586339	MRPL43	assignmentsChromaffin_cells.Chrom_SOX2_statusHigh
assignmentsChromaffin_cells.Chrom_SOX2_statusHigh.TMEM59	2.49175713	6.17748134	6.95571915	1.19E-09	4.88E-08	11.7402883	TMEM59	assignmentsChromaffin_cells.Chrom_SOX2_statusHigh
assignmentsChromaffin_cells.Chrom_SOX2_statusHigh.MRPS24	3.34891052	3.49951663	6.94319635	1.27E-09	5.08E-08	11.7394132	MRPS24	assignmentsChromaffin_cells.Chrom_SOX2_statusHigh
assignmentsChromaffin_cells.Chrom_SOX2_statusHigh.DDA1	1.93095546	3.73211186	6.94128231	1.26E-09	5.08E-08	11.7387032	DDA1	assignmentsChromaffin_cells.Chrom_SOX2_statusHigh
assignmentsChromaffin_cells.Chrom_SOX2_statusHigh.TCEA2	1.80370797	4.10235424	6.93783497	1.28E-09	5.13E-08	11.7159869	TCEA2	assignmentsChromaffin_cells.Chrom_SOX2_statusHigh
assignmentsChromaffin_cells.Chrom_SOX2_statusHigh.BLOC1S1	2.82751771	4.18548137	6.93503991	1.30E-09	5.18E-08	11.7047666	BLOC1S1	assignmentsChromaffin_cells.Chrom_SOX2_statusHigh
assignmentsChromaffin_cells.Chrom_SOX2_statusHigh.UQCRFS1	2.22973484	4.85914325	6.93235372	1.31E-09	5.20E-08	11.6715958	UQCRFS1	assignmentsChromaffin_cells.Chrom_SOX2_statusHigh
assignmentsChromaffin_cells.Chrom_SOX2_statusHigh.ZDHHC17	-0.9616364	6.49170458	-6.9338402	1.31E-09	5.18E-08	11.6714312	ZDHHC17	assignmentsChromaffin_cells.Chrom_SOX2_statusHigh
assignmentsChromaffin_cells.Chrom_SOX2_statusHigh.OCEL1	2.14788031	2.48441427	6.91669271	1.41E-09	5.54E-08	11.6634063	OCEL1	assignmentsChromaffin_cells.Chrom_SOX2_statusHigh
assignmentsChromaffin_cells.Chrom_SOX2_statusHigh.SERINC1	1.85217242	6.58831062	6.93695708	1.29E-09	5.14E-08	11.6585209	SERINC1	assignmentsChromaffin_cells.Chrom_SOX2_statusHigh
assignmentsChromaffin_cells.Chrom_SOX2_statusHigh.TMEM258	3.05254883	4.61048386	6.92743932	1.35E-09	5.34E-08	11.6515244	TMEM258	assignmentsChromaffin_cells.Chrom_SOX2_statusHigh
assignmentsChromaffin_cells.Chrom_SOX2_statusHigh.SAP130	-1.2280892	5.4416081	-6.9156325	1.41E-09	5.55E-08	11.6275888	SAP130	assignmentsChromaffin_cells.Chrom_SOX2_statusHigh
assignmentsChromaffin_cells.Chrom_SOX2_statusHigh.EBNA1BP2	2.32185691	3.11157928	6.90748083	1.46E-09	5.68E-08	11.6164901	EBNA1BP2	assignmentsChromaffin_cells.Chrom_SOX2_statusHigh
assignmentsChromaffin_cells.Chrom_SOX2_statusHigh.HNRNPA0	2.34564607	5.03955204	6.91225243	1.43E-09	5.62E-08	11.593907	HNRNPA0	assignmentsChromaffin_cells.Chrom_SOX2_statusHigh
assignmentsChromaffin_cells.Chrom_SOX2_statusHigh.CIAO2B	2.41913923	4.90054534	6.9106258	1.44E-09	5.64E-08	11.5860903	CIAO2B	assignmentsChromaffin_cells.Chrom_SOX2_statusHigh
assignmentsChromaffin_cells.Chrom_SOX2_statusHigh.DPM2	1.57458451	3.67700478	6.89822712	1.52E-09	5.82E-08	11.5710831	DPM2	assignmentsChromaffin_cells.Chrom_SOX2_statusHigh
assignmentsChromaffin_cells.Chrom_SOX2_statusHigh.EXOC2	-1.4078409	6.70587057	-6.9074053	1.46E-09	5.68E-08	11.568753	EXOC2	assignmentsChromaffin_cells.Chrom_SOX2_statusHigh
assignmentsChromaffin_cells.Chrom_SOX2_statusHigh.BRIX1	1.65711946	3.99916137	6.89986937	1.51E-09	5.79E-08	11.5668413	BRIX1	assignmentsChromaffin_cells.Chrom_SOX2_statusHigh
assignmentsChromaffin_cells.Chrom_SOX2_statusHigh.TUBA4A	3.37815616	2.86984008	6.89115499	1.58E-09	6.00E-08	11.5461967	TUBA4A	assignmentsChromaffin_cells.Chrom_SOX2_statusHigh
assignmentsChromaffin_cells.Chrom_SOX2_statusHigh.MAF1	2.33866191	3.48002277	6.89125058	1.57E-09	5.98E-08	11.5443392	MAF1	assignmentsChromaffin_cells.Chrom_SOX2_statusHigh
assignmentsChromaffin_cells.Chrom_SOX2_statusHigh.UBE2L6	2.12657485	3.46088464	6.88928733	1.58E-09	6.01E-08	11.5432493	UBE2L6	assignmentsChromaffin_cells.Chrom_SOX2_statusHigh
assignmentsChromaffin_cells.Chrom_SOX2_statusHigh.NDUFA13	2.64314202	6.31367581	6.9110768	1.45E-09	5.64E-08	11.5428033	NDUFA13	assignmentsChromaffin_cells.Chrom_SOX2_statusHigh
assignmentsChromaffin_cells.Chrom_SOX2_statusHigh.UROD	2.2384128	3.0872398	6.88797839	1.59E-09	6.03E-08	11.5385193	UROD	assignmentsChromaffin_cells.Chrom_SOX2_statusHigh
assignmentsChromaffin_cells.Chrom_SOX2_statusHigh.TOMM20	2.0844569	5.8785075	6.90567202	1.47E-09	5.69E-08	11.5379455	TOMM20	assignmentsChromaffin_cells.Chrom_SOX2_statusHigh
assignmentsChromaffin_cells.Chrom_SOX2_statusHigh.DYNLL1	3.2705847	5.76433164	6.9060939	1.49E-09	5.73E-08	11.5332006	DYNLL1	assignmentsChromaffin_cells.Chrom_SOX2_statusHigh
assignmentsChromaffin_cells.Chrom_SOX2_statusHigh.ARF1	1.8913517	6.63381396	6.90606134	1.47E-09	5.69E-08	11.5302056	ARF1	assignmentsChromaffin_cells.Chrom_SOX2_statusHigh
assignmentsChromaffin_cells.Chrom_SOX2_statusHigh.COX6A1	3.50245866	5.59628261	6.90515962	1.49E-09	5.74E-08	11.5266165	COX6A1	assignmentsChromaffin_cells.Chrom_SOX2_statusHigh
assignmentsChromaffin_cells.Chrom_SOX2_statusHigh.YRDC	2.10807118	2.25113927	6.88190061	1.63E-09	6.13E-08	11.523002	YRDC	assignmentsChromaffin_cells.Chrom_SOX2_statusHigh
assignmentsChromaffin_cells.Chrom_SOX2_statusHigh.PYCR2	1.80252947	3.73654647	6.88725936	1.59E-09	6.03E-08	11.5164806	PYCR2	assignmentsChromaffin_cells.Chrom_SOX2_statusHigh
assignmentsChromaffin_cells.Chrom_SOX2_statusHigh.COPZ1	1.86580797	4.11409309	6.88588984	1.60E-09	6.04E-08	11.4994328	COPZ1	assignmentsChromaffin_cells.Chrom_SOX2_statusHigh
assignmentsChromaffin_cells.Chrom_SOX2_statusHigh.VDAC2	1.840751	5.70412433	6.88613906	1.60E-09	6.04E-08	11.4630388	VDAC2	assignmentsChromaffin_cells.Chrom_SOX2_statusHigh
assignmentsChromaffin_cells.Chrom_SOX2_statusHigh.METTL26	2.26132421	4.11577881	6.87642188	1.67E-09	6.24E-08	11.4587154	METTL26	assignmentsChromaffin_cells.Chrom_SOX2_statusHigh
assignmentsChromaffin_cells.Chrom_SOX2_statusHigh.VKORC1	2.88066791	4.58893919	6.87968115	1.65E-09	6.20E-08	11.4531113	VKORC1	assignmentsChromaffin_cells.Chrom_SOX2_statusHigh
assignmentsChromaffin_cells.Chrom_SOX2_statusHigh.CCDC25	1.37767848	4.52873367	6.87354501	1.69E-09	6.30E-08	11.4417032	CCDC25	assignmentsChromaffin_cells.Chrom_SOX2_statusHigh
assignmentsChromaffin_cells.Chrom_SOX2_statusHigh.CHAT	4.73048909	-1.0332327	6.94921671	1.22E-09	4.96E-08	11.438095	CHAT	assignmentsChromaffin_cells.Chrom_SOX2_statusHigh
assignmentsChromaffin_cells.Chrom_SOX2_statusHigh.EXOC3-AS1	1.82152469	1.65859365	6.85487156	1.83E-09	6.74E-08	11.4106838	EXOC3-AS1	assignmentsChromaffin_cells.Chrom_SOX2_statusHigh
assignmentsChromaffin_cells.Chrom_SOX2_statusHigh.UXT	2.38501513	4.65163012	6.86341717	1.77E-09	6.54E-08	11.3958601	UXT	assignmentsChromaffin_cells.Chrom_SOX2_statusHigh
assignmentsChromaffin_cells.Chrom_SOX2_statusHigh.SDF4	1.70686589	4.87774958	6.85992553	1.79E-09	6.62E-08	11.3913638	SDF4	assignmentsChromaffin_cells.Chrom_SOX2_statusHigh
assignmentsChromaffin_cells.Chrom_SOX2_statusHigh.CHCHD5	2.40571784	3.1063601	6.84885413	1.88E-09	6.86E-08	11.3670933	CHCHD5	assignmentsChromaffin_cells.Chrom_SOX2_statusHigh
assignmentsChromaffin_cells.Chrom_SOX2_statusHigh.LSM1	1.71226912	3.81408803	6.8511745	1.86E-09	6.81E-08	11.3664513	LSM1	assignmentsChromaffin_cells.Chrom_SOX2_statusHigh
assignmentsChromaffin_cells.Chrom_SOX2_statusHigh.NUDT1	2.87086978	1.81885277	6.84086508	1.94E-09	7.08E-08	11.3548939	NUDT1	assignmentsChromaffin_cells.Chrom_SOX2_statusHigh
assignmentsChromaffin_cells.Chrom_SOX2_statusHigh.MRPL40	2.81306141	2.71289799	6.84087839	1.95E-09	7.08E-08	11.3355692	MRPL40	assignmentsChromaffin_cells.Chrom_SOX2_statusHigh
assignmentsChromaffin_cells.Chrom_SOX2_statusHigh.IFI30	3.66519531	2.99183063	6.83772096	1.98E-09	7.15E-08	11.3314111	IFI30	assignmentsChromaffin_cells.Chrom_SOX2_statusHigh
assignmentsChromaffin_cells.Chrom_SOX2_statusHigh.RABGAP1L	-1.1618116	9.06455467	-6.8655189	1.75E-09	6.50E-08	11.3279189	RABGAP1L	assignmentsChromaffin_cells.Chrom_SOX2_statusHigh
assignmentsChromaffin_cells.Chrom_SOX2_statusHigh.NDUFS3	2.0809192	3.83112973	6.84015481	1.95E-09	7.08E-08	11.3202725	NDUFS3	assignmentsChromaffin_cells.Chrom_SOX2_statusHigh
assignmentsChromaffin_cells.Chrom_SOX2_statusHigh.CCDC51	2.09840075	1.40883881	6.83024877	2.03E-09	7.31E-08	11.310994	CCDC51	assignmentsChromaffin_cells.Chrom_SOX2_statusHigh
assignmentsChromaffin_cells.Chrom_SOX2_statusHigh.MORN2	2.8945184	2.35370152	6.82727666	2.06E-09	7.40E-08	11.2867926	MORN2	assignmentsChromaffin_cells.Chrom_SOX2_statusHigh
assignmentsChromaffin_cells.Chrom_SOX2_statusHigh.MRPS2	1.80940314	2.72021948	6.82503399	2.08E-09	7.45E-08	11.2824093	MRPS2	assignmentsChromaffin_cells.Chrom_SOX2_statusHigh
assignmentsChromaffin_cells.Chrom_SOX2_statusHigh.DRICH1	-2.7353144	1.54647551	-6.8538556	1.84E-09	6.75E-08	11.2780857	DRICH1	assignmentsChromaffin_cells.Chrom_SOX2_statusHigh
assignmentsChromaffin_cells.Chrom_SOX2_statusHigh.PHLPP2	-1.20523	5.23748412	-6.8312374	2.03E-09	7.30E-08	11.2770365	PHLPP2	assignmentsChromaffin_cells.Chrom_SOX2_statusHigh
assignmentsChromaffin_cells.Chrom_SOX2_statusHigh.CSTB	2.73361911	5.03679442	6.83296091	2.02E-09	7.29E-08	11.2608285	CSTB	assignmentsChromaffin_cells.Chrom_SOX2_statusHigh
assignmentsChromaffin_cells.Chrom_SOX2_statusHigh.SLC39A1	2.34861672	3.28380943	6.82049239	2.12E-09	7.58E-08	11.2590656	SLC39A1	assignmentsChromaffin_cells.Chrom_SOX2_statusHigh

assignmentsChromaffin_cells.Chrom_SOX2_statusHigh.GMPR2	1.71846406	3.25624974	6.81514374	2.17E-09	7.70E-08	11.2372825	GMPR2	assignmentsChromaffin_cells.Chrom_SOX2_statusHigh
assignmentsChromaffin_cells.Chrom_SOX2_statusHigh.DPM3	2.71886276	3.71787476	6.81857343	2.15E-09	7.65E-08	11.2224589	DPM3	assignmentsChromaffin_cells.Chrom_SOX2_statusHigh
assignmentsChromaffin_cells.Chrom_SOX2_statusHigh.PIGBOS1	1.59560854	2.97592025	6.80917458	2.23E-09	7.82E-08	11.2087728	PIGBOS1	assignmentsChromaffin_cells.Chrom_SOX2_statusHigh
assignmentsChromaffin_cells.Chrom_SOX2_statusHigh.PSMD10	2.31175541	3.07297251	6.81050415	2.21E-09	7.82E-08	11.207911	PSMD10	assignmentsChromaffin_cells.Chrom_SOX2_statusHigh
assignmentsChromaffin_cells.Chrom_SOX2_statusHigh.LAMTOR3	1.19545629	4.19428722	6.81348234	2.19E-09	7.74E-08	11.2001924	LAMTOR3	assignmentsChromaffin_cells.Chrom_SOX2_statusHigh
assignmentsChromaffin_cells.Chrom_SOX2_statusHigh.PLCH2	4.69867554	-0.4094304	6.85728329	1.81E-09	6.68E-08	11.1835602	PLCH2	assignmentsChromaffin_cells.Chrom_SOX2_statusHigh
assignmentsChromaffin_cells.Chrom_SOX2_statusHigh.CNOT1	-0.9528729	6.83915556	-6.8172371	2.15E-09	7.65E-08	11.1832048	CNOT1	assignmentsChromaffin_cells.Chrom_SOX2_statusHigh
assignmentsChromaffin_cells.Chrom_SOX2_statusHigh.ZNF580	1.6560388	4.15093194	6.80819333	2.24E-09	7.82E-08	11.1793264	ZNF580	assignmentsChromaffin_cells.Chrom_SOX2_statusHigh
assignmentsChromaffin_cells.Chrom_SOX2_statusHigh.YIPF3	2.06006841	4.44493988	6.80974791	2.22E-09	7.82E-08	11.1738465	YIPF3	assignmentsChromaffin_cells.Chrom_SOX2_statusHigh
assignmentsChromaffin_cells.Chrom_SOX2_statusHigh.C14orf119	2.13880381	3.64730231	6.80092688	2.31E-09	8.02E-08	11.1700056	C14orf119	assignmentsChromaffin_cells.Chrom_SOX2_statusHigh
assignmentsChromaffin_cells.Chrom_SOX2_statusHigh.PSMB10	2.69339435	3.44803945	6.79843903	2.33E-09	8.07E-08	11.1661566	PSMB10	assignmentsChromaffin_cells.Chrom_SOX2_statusHigh
assignmentsChromaffin_cells.Chrom_SOX2_statusHigh.OST4	3.63914941	5.02066215	6.8109447	2.23E-09	7.82E-08	11.1469234	OST4	assignmentsChromaffin_cells.Chrom_SOX2_statusHigh
assignmentsChromaffin_cells.Chrom_SOX2_statusHigh.MDP1	2.20747633	3.28830613	6.79446644	2.37E-09	8.16E-08	11.1415153	MDP1	assignmentsChromaffin_cells.Chrom_SOX2_statusHigh
assignmentsChromaffin_cells.Chrom_SOX2_statusHigh.A1BG	1.74307258	3.98042613	6.79868255	2.33E-09	8.07E-08	11.1374	A1BG	assignmentsChromaffin_cells.Chrom_SOX2_statusHigh
assignmentsChromaffin_cells.Chrom_SOX2_statusHigh.LUC7L	-1.0261399	7.11973502	-6.8064383	2.25E-09	7.85E-08	11.1297163	LUC7L	assignmentsChromaffin_cells.Chrom_SOX2_statusHigh
assignmentsChromaffin_cells.Chrom_SOX2_statusHigh.TRAPPC5	2.80698885	4.2991376	6.79776999	2.35E-09	8.11E-08	11.1233583	TRAPPC5	assignmentsChromaffin_cells.Chrom_SOX2_statusHigh
assignmentsChromaffin_cells.Chrom_SOX2_statusHigh.PFDN5	3.00839723	6.41451419	6.81035448	2.24E-09	7.82E-08	11.1184606	PFDN5	assignmentsChromaffin_cells.Chrom_SOX2_statusHigh
assignmentsChromaffin_cells.Chrom_SOX2_statusHigh.KRT10	3.16788339	4.64805771	6.79827857	2.36E-09	8.13E-08	11.1009716	KRT10	assignmentsChromaffin_cells.Chrom_SOX2_statusHigh
assignmentsChromaffin_cells.Chrom_SOX2_statusHigh.HPN	3.72731556	0.25808768	6.86524867	1.75E-09	6.50E-08	11.0968704	HPN	assignmentsChromaffin_cells.Chrom_SOX2_statusHigh
assignmentsChromaffin_cells.Chrom_SOX2_statusHigh.POP4	2.06646969	2.80172542	6.7808919	2.51E-09	8.57E-08	11.0967789	POP4	assignmentsChromaffin_cells.Chrom_SOX2_statusHigh
assignmentsChromaffin_cells.Chrom_SOX2_statusHigh.EPB41L4A-A	2.16713351	3.7850306	6.786733	2.45E-09	8.41E-08	11.0922739	EPB41L4A-AS	assignmentsChromaffin_cells.Chrom_SOX2_statusHigh
assignmentsChromaffin_cells.Chrom_SOX2_statusHigh.VPS25	2.04431029	3.02886783	6.77893331	2.53E-09	8.62E-08	11.0855171	VPS25	assignmentsChromaffin_cells.Chrom_SOX2_statusHigh
assignmentsChromaffin_cells.Chrom_SOX2_statusHigh.RAB5IF	2.10790146	4.1990586	6.78384313	2.48E-09	8.49E-08	11.0831054	RAB5IF	assignmentsChromaffin_cells.Chrom_SOX2_statusHigh
assignmentsChromaffin_cells.Chrom_SOX2_statusHigh.ARL16	1.85539284	4.04496562	6.77607079	2.56E-09	8.67E-08	11.0501068	ARL16	assignmentsChromaffin_cells.Chrom_SOX2_statusHigh
assignmentsChromaffin_cells.Chrom_SOX2_statusHigh.PPP6R2	-1.2812962	7.13347806	-6.7835751	2.48E-09	8.49E-08	11.0316604	PPP6R2	assignmentsChromaffin_cells.Chrom_SOX2_statusHigh
assignmentsChromaffin_cells.Chrom_SOX2_statusHigh.THOC1	-0.9677127	6.33067032	-6.7766425	2.56E-09	8.67E-08	11.0216369	THOC1	assignmentsChromaffin_cells.Chrom_SOX2_statusHigh
assignmentsChromaffin_cells.Chrom_SOX2_statusHigh.RHBDD2	1.63442626	5.03671967	6.77625294	2.56E-09	8.67E-08	11.0196607	RHBDD2	assignmentsChromaffin_cells.Chrom_SOX2_statusHigh
assignmentsChromaffin_cells.Chrom_SOX2_statusHigh.GRHPR	1.34733831	4.42898424	6.76661309	2.67E-09	8.99E-08	11.0089254	GRHPR	assignmentsChromaffin_cells.Chrom_SOX2_statusHigh
assignmentsChromaffin_cells.Chrom_SOX2_statusHigh.NINJ1	2.51118423	3.51906754	6.75817603	2.77E-09	9.28E-08	11.0023985	NINJ1	assignmentsChromaffin_cells.Chrom_SOX2_statusHigh
assignmentsChromaffin_cells.Chrom_SOX2_statusHigh.LAMTOR4	2.2523887	4.97680115	6.76735842	2.66E-09	8.98E-08	10.988645	LAMTOR4	assignmentsChromaffin_cells.Chrom_SOX2_statusHigh
assignmentsChromaffin_cells.Chrom_SOX2_statusHigh.FMC1	2.59430717	3.05929538	6.75405873	2.82E-09	9.43E-08	10.9673358	FMC1	assignmentsChromaffin_cells.Chrom_SOX2_statusHigh
assignmentsChromaffin_cells.Chrom_SOX2_statusHigh.CLEC16A	-1.0596807	6.77124931	-6.762869	2.71E-09	9.12E-08	10.9605027	CLEC16A	assignmentsChromaffin_cells.Chrom_SOX2_statusHigh
assignmentsChromaffin_cells.Chrom_SOX2_statusHigh.MRPS15	2.17264651	3.36422677	6.74908215	2.88E-09	9.57E-08	10.959341	MRPS15	assignmentsChromaffin_cells.Chrom_SOX2_statusHigh
assignmentsChromaffin_cells.Chrom_SOX2_statusHigh.MRPL4	1.90258158	3.95774118	6.74873775	2.88E-09	9.57E-08	10.9302762	MRPL4	assignmentsChromaffin_cells.Chrom_SOX2_statusHigh
assignmentsChromaffin_cells.Chrom_SOX2_statusHigh.LSM10	2.46409176	2.69066966	6.73850927	3.01E-09	9.93E-08	10.9291767	LSM10	assignmentsChromaffin_cells.Chrom_SOX2_statusHigh
assignmentsChromaffin_cells.Chrom_SOX2_statusHigh.PSMB6	2.8440613	4.2709063	6.7480822	2.90E-09	9.61E-08	10.9282405	PSMB6	assignmentsChromaffin_cells.Chrom_SOX2_statusHigh
assignmentsChromaffin_cells.Chrom_SOX2_statusHigh.MSRB1	2.73725864	1.36419641	6.73361215	3.07E-09	1.01E-07	10.9135521	MSRB1	assignmentsChromaffin_cells.Chrom_SOX2_statusHigh
assignmentsChromaffin_cells.Chrom_SOX2_statusHigh.MED8	1.49357161	3.46061803	6.73655962	3.03E-09	9.99E-08	10.9075113	MED8	assignmentsChromaffin_cells.Chrom_SOX2_statusHigh
assignmentsChromaffin_cells.Chrom_SOX2_statusHigh.TUFM	2.22122042	4.47309856	6.74152654	2.97E-09	9.82E-08	10.8929087	TUFM	assignmentsChromaffin_cells.Chrom_SOX2_statusHigh
assignmentsChromaffin_cells.Chrom_SOX2_statusHigh.EDF1	2.26392435	5.70196563	6.74872117	2.88E-09	9.57E-08	10.8896472	EDF1	assignmentsChromaffin_cells.Chrom_SOX2_statusHigh
assignmentsChromaffin_cells.Chrom_SOX2_statusHigh.ZNF622	1.90014632	3.81980671	6.72906341	3.13E-09	1.02E-07	10.8639659	ZNF622	assignmentsChromaffin_cells.Chrom_SOX2_statusHigh
assignmentsChromaffin_cells.Chrom_SOX2_statusHigh.NUDC	1.84646707	5.25730079	6.73583674	3.04E-09	1.00E-07	10.8619947	NUDC	assignmentsChromaffin_cells.Chrom_SOX2_statusHigh
assignmentsChromaffin_cells.Chrom_SOX2_statusHigh.TMEM208	2.38416339	4.3194227	6.73026791	3.12E-09	1.02E-07	10.8416125	TMEM208	assignmentsChromaffin_cells.Chrom_SOX2_statusHigh
assignmentsChromaffin_cells.Chrom_SOX2_statusHigh.FBXW4	-1.2741646	6.52085533	-6.7316624	3.10E-09	1.01E-07	10.834715	FBXW4	assignmentsChromaffin_cells.Chrom_SOX2_statusHigh
assignmentsChromaffin_cells.Chrom_SOX2_statusHigh.NCSTN	1.42617942	4.32624971	6.72082274	3.24E-09	1.05E-07	10.816567	NCSTN	assignmentsChromaffin_cells.Chrom_SOX2_statusHigh
assignmentsChromaffin_cells.Chrom_SOX2_statusHigh.UQCC3	2.92778947	2.34990424	6.70869314	3.42E-09	1.09E-07	10.8089179	UQCC3	assignmentsChromaffin_cells.Chrom_SOX2_statusHigh
assignmentsChromaffin_cells.Chrom_SOX2_statusHigh.SH2D2A	4.49373272	0.68304375	6.7493357	2.87E-09	9.57E-08	10.8076498	SH2D2A	assignmentsChromaffin_cells.Chrom_SOX2_statusHigh
assignmentsChromaffin_cells.Chrom_SOX2_statusHigh.CHID1	1.67738976	4.20599094	6.71188435	3.37E-09	1.08E-07	10.7870834	CHID1	assignmentsChromaffin_cells.Chrom_SOX2_statusHigh
assignmentsChromaffin_cells.Chrom_SOX2_statusHigh.ATXN7L3B	1.59951257	5.10837678	6.71839494	3.28E-09	1.06E-07	10.7799689	ATXN7L3B	assignmentsChromaffin_cells.Chrom_SOX2_statusHigh
assignmentsChromaffin_cells.Chrom_SOX2_statusHigh.SEC24B	-1.2206419	7.20301486	-6.7199953	3.26E-09	1.05E-07	10.7739082	SEC24B	assignmentsChromaffin_cells.Chrom_SOX2_statusHigh
assignmentsChromaffin_cells.Chrom_SOX2_statusHigh.CPNE3	1.36788306	5.68186754	6.72035589	3.25E-09	1.05E-07	10.7720144	CPNE3	assignmentsChromaffin_cells.Chrom_SOX2_statusHigh
assignmentsChromaffin_cells.Chrom_SOX2_statusHigh.SNAPC2	2.61602562	1.83856494	6.69835456	3.57E-09	1.13E-07	10.7710878	SNAPC2	assignmentsChromaffin_cells.Chrom_SOX2_statusHigh
assignmentsChromaffin_cells.Chrom_SOX2_statusHigh.LYRM2	1.64030767	4.82423391	6.71413537	3.34E-09	1.08E-07	10.769291	LYRM2	assignmentsChromaffin_cells.Chrom_SOX2_statusHigh
assignmentsChromaffin_cells.Chrom_SOX2_statusHigh.TIMM13	2.75280931	3.79419223	6.7064899	3.46E-09	1.10E-07	10.7513662	TIMM13	assignmentsChromaffin_cells.Chrom_SOX2_statusHigh
assignmentsChromaffin_cells.Chrom_SOX2_statusHigh.LIN7A	-3.1015374	6.24330549	-6.707659	3.46E-09	1.10E-07	10.7353712	LIN7A	assignmentsChromaffin_cells.Chrom_SOX2_statusHigh
assignmentsChromaffin_cells.Chrom_SOX2_statusHigh.MBD5	-1.5470336	9.08627808	-6.724894	3.19E-09	1.04E-07	10.7332952	MBD5	assignmentsChromaffin_cells.Chrom_SOX2_statusHigh
assignmentsChromaffin_cells.Chrom_SOX2_statusHigh.COX6C	2.70103903	5.92999103	6.71401261	3.36E-09	1.08E-07	10.7263504	COX6C	assignmentsChromaffin_cells.Chrom_SOX2_statusHigh
assignmentsChromaffin_cells.Chrom_SOX2_statusHigh.NDUFA1	2.90217682	5.24899779	6.70565093	3.49E-09	1.11E-07	10.7081619	NDUFA1	assignmentsChromaffin_cells.Chrom_SOX2_statusHigh
assignmentsChromaffin_cells.Chrom_SOX2_statusHigh.IDH3G	1.73056546	3.98965669	6.69190932	3.67E-09	1.16E-07	10.7012189	IDH3G	assignmentsChromaffin_cells.Chrom_SOX2_statusHigh
assignmentsChromaffin_cells.Chrom_SOX2_statusHigh.SLC35F5	-1.0803085	6.44372363	-6.696978	3.59E-09	1.14E-07	10.6919914	SLC35F5	assignmentsChromaffin_cells.Chrom_SOX2_statusHigh
assignmentsChromaffin_cells.Chrom_SOX2_statusHigh.CYB5R1	1.92320568	4.29367866	6.68753462	3.74E-09	1.18E-07	10.6667274	CYB5R1	assignmentsChromaffin_cells.Chrom_SOX2_statusHigh
assignmentsChromaffin_cells.Chrom_SOX2_statusHigh.YIPF5	1.64222301	4.440919	6.68514108	3.78E-09	1.19E-07	10.6602577	YIPF5	assignmentsChromaffin_cells.Chrom_SOX2_statusHigh
assignmentsChromaffin_cells.Chrom_SOX2_statusHigh.CD63	3.58452428	6.80766459	6.700902	3.57E-09	1.13E-07	10.6522942	CD63	assignmentsChromaffin_cells.Chrom_SOX2_statusHigh
assignmentsChromaffin_cells.Chrom_SOX2_statusHigh.ATP6V1F	3.39469531	4.1989373	6.68464956	3.82E-09	1.20E-07	10.6485026	ATP6V1F	assignmentsChromaffin_cells.Chrom_SOX2_statusHigh

assignmentsChromaffin_cells.Chrom_SOX2_statusHigh.UQCR10	3.43274306	4.38495617	6.68604639	3.80E-09	1.19E-07	10.6450788	UQCR10	assignmentsChromaffin_cells.Chrom_SOX2_statusHigh
assignmentsChromaffin_cells.Chrom_SOX2_statusHigh.PGRMC1	2.67135784	4.16889009	6.68040881	3.87E-09	1.20E-07	10.6392919	PGRMC1	assignmentsChromaffin_cells.Chrom_SOX2_statusHigh
assignmentsChromaffin_cells.Chrom_SOX2_statusHigh.SNHG6	2.54051267	5.51051775	6.68823863	3.74E-09	1.18E-07	10.63444	SNHG6	assignmentsChromaffin_cells.Chrom_SOX2_statusHigh
assignmentsChromaffin_cells.Chrom_SOX2_statusHigh.GAMT	2.0334648	3.17073638	6.66737504	4.07E-09	1.25E-07	10.6078781	GAMT	assignmentsChromaffin_cells.Chrom_SOX2_statusHigh
assignmentsChromaffin_cells.Chrom_SOX2_statusHigh.ISCA2	2.02046709	3.02340272	6.66282396	4.15E-09	1.26E-07	10.602277	ISCA2	assignmentsChromaffin_cells.Chrom_SOX2_statusHigh
assignmentsChromaffin_cells.Chrom_SOX2_statusHigh.CLPP	1.95343433	3.75098433	6.66446908	4.12E-09	1.26E-07	10.5983221	CLPP	assignmentsChromaffin_cells.Chrom_SOX2_statusHigh
assignmentsChromaffin_cells.Chrom_SOX2_statusHigh.RAB3GAP1	-0.9913112	7.39853097	-6.6807607	3.85E-09	1.20E-07	10.5978566	RAB3GAP1	assignmentsChromaffin_cells.Chrom_SOX2_statusHigh
assignmentsChromaffin_cells.Chrom_SOX2_statusHigh.SNHG16	2.28503534	4.71294686	6.67115646	4.01E-09	1.24E-07	10.5938781	SNHG16	assignmentsChromaffin_cells.Chrom_SOX2_statusHigh
assignmentsChromaffin_cells.Chrom_SOX2_statusHigh.ELOB	3.2335696	5.29018257	6.67728956	3.95E-09	1.23E-07	10.5938278	ELOB	assignmentsChromaffin_cells.Chrom_SOX2_statusHigh
assignmentsChromaffin_cells.Chrom_SOX2_statusHigh.EIF6	2.22754765	3.87421076	6.66405626	4.13E-09	1.26E-07	10.5901321	EIF6	assignmentsChromaffin_cells.Chrom_SOX2_statusHigh
assignmentsChromaffin_cells.Chrom_SOX2_statusHigh.PDCD5	2.24036005	4.94806061	6.67240231	3.99E-09	1.24E-07	10.5850453	PDCD5	assignmentsChromaffin_cells.Chrom_SOX2_statusHigh
assignmentsChromaffin_cells.Chrom_SOX2_statusHigh.KLHL22	-1.4276669	5.50596675	-6.6635635	4.14E-09	1.26E-07	10.5755288	KLHL22	assignmentsChromaffin_cells.Chrom_SOX2_statusHigh
assignmentsChromaffin_cells.Chrom_SOX2_statusHigh.TMED9	2.72818096	4.74216543	6.66739191	4.09E-09	1.26E-07	10.5723423	TMED9	assignmentsChromaffin_cells.Chrom_SOX2_statusHigh
assignmentsChromaffin_cells.Chrom_SOX2_statusHigh.C1orf43	1.09507233	5.83745276	6.67045976	4.02E-09	1.24E-07	10.5676004	C1orf43	assignmentsChromaffin_cells.Chrom_SOX2_statusHigh
assignmentsChromaffin_cells.Chrom_SOX2_statusHigh.PIGP	1.68652047	3.74073949	6.65608962	4.27E-09	1.29E-07	10.5556545	PIGP	assignmentsChromaffin_cells.Chrom_SOX2_statusHigh
assignmentsChromaffin_cells.Chrom_SOX2_statusHigh.SSR3	2.2274441	5.17670371	6.66228228	4.16E-09	1.26E-07	10.5553309	SSR3	assignmentsChromaffin_cells.Chrom_SOX2_statusHigh
assignmentsChromaffin_cells.Chrom_SOX2_statusHigh.MRPL46	1.32397149	3.28412793	6.65232815	4.34E-09	1.31E-07	10.5535521	MRPL46	assignmentsChromaffin_cells.Chrom_SOX2_statusHigh
assignmentsChromaffin_cells.Chrom_SOX2_statusHigh.R3HCC1	2.06536734	3.94211698	6.65344187	4.32E-09	1.31E-07	10.5405568	R3HCC1	assignmentsChromaffin_cells.Chrom_SOX2_statusHigh
assignmentsChromaffin_cells.Chrom_SOX2_statusHigh.YWHAB	1.80362565	6.71964315	6.66617833	4.09E-09	1.26E-07	10.5384313	YWHAB	assignmentsChromaffin_cells.Chrom_SOX2_statusHigh
assignmentsChromaffin_cells.Chrom_SOX2_statusHigh.OAZ1	3.24378887	6.57268235	6.6727496	4.02E-09	1.24E-07	10.5355539	OAZ1	assignmentsChromaffin_cells.Chrom_SOX2_statusHigh
assignmentsChromaffin_cells.Chrom_SOX2_statusHigh.RUVBL2	2.02053101	3.9468611	6.65020019	4.38E-09	1.32E-07	10.5288455	RUVBL2	assignmentsChromaffin_cells.Chrom_SOX2_statusHigh
assignmentsChromaffin_cells.Chrom_SOX2_statusHigh.MRPL10	1.78714244	2.66031114	6.64052119	4.57E-09	1.37E-07	10.5275124	MRPL10	assignmentsChromaffin_cells.Chrom_SOX2_statusHigh
assignmentsChromaffin_cells.Chrom_SOX2_statusHigh.RBM42	2.32940861	3.34396459	6.64464289	4.49E-09	1.35E-07	10.5268884	RBM42	assignmentsChromaffin_cells.Chrom_SOX2_statusHigh
assignmentsChromaffin_cells.Chrom_SOX2_statusHigh.TMEM131	-1.1652899	7.47468613	-6.6619125	4.17E-09	1.26E-07	10.5240834	TMEM131	assignmentsChromaffin_cells.Chrom_SOX2_statusHigh
assignmentsChromaffin_cells.Chrom_SOX2_statusHigh.HSPA2	3.67101764	1.42653824	6.63722179	4.63E-09	1.38E-07	10.5007789	HSPA2	assignmentsChromaffin_cells.Chrom_SOX2_statusHigh
assignmentsChromaffin_cells.Chrom_SOX2_statusHigh.GRWD1	1.84885794	2.47393844	6.62569114	4.86E-09	1.43E-07	10.4681279	GRWD1	assignmentsChromaffin_cells.Chrom_SOX2_statusHigh
assignmentsChromaffin_cells.Chrom_SOX2_statusHigh.TRAPPC3	1.43643674	4.15141529	6.63009694	4.77E-09	1.40E-07	10.4577444	TRAPPC3	assignmentsChromaffin_cells.Chrom_SOX2_statusHigh
assignmentsChromaffin_cells.Chrom_SOX2_statusHigh.ELOVL1	1.93305912	2.62154233	6.62084771	4.96E-09	1.44E-07	10.4566036	ELOVL1	assignmentsChromaffin_cells.Chrom_SOX2_statusHigh
assignmentsChromaffin_cells.Chrom_SOX2_statusHigh.PCBP1	2.94230426	4.96403204	6.63910299	4.62E-09	1.38E-07	10.4555794	PCBP1	assignmentsChromaffin_cells.Chrom_SOX2_statusHigh
assignmentsChromaffin_cells.Chrom_SOX2_statusHigh.MICOS10	2.82169962	4.74943994	6.63568973	4.68E-09	1.39E-07	10.4544119	MICOS10	assignmentsChromaffin_cells.Chrom_SOX2_statusHigh
assignmentsChromaffin_cells.Chrom_SOX2_statusHigh.NTMT1	1.51297341	3.58454399	6.62517685	4.87E-09	1.43E-07	10.4503023	NTMT1	assignmentsChromaffin_cells.Chrom_SOX2_statusHigh
assignmentsChromaffin_cells.Chrom_SOX2_statusHigh.PA2G4	1.99210013	5.35490147	6.63762785	4.62E-09	1.38E-07	10.4472634	PA2G4	assignmentsChromaffin_cells.Chrom_SOX2_statusHigh
assignmentsChromaffin_cells.Chrom_SOX2_statusHigh.HSBP1	2.45006538	4.86104114	6.6342653	4.70E-09	1.39E-07	10.4389001	HSBP1	assignmentsChromaffin_cells.Chrom_SOX2_statusHigh
assignmentsChromaffin_cells.Chrom_SOX2_statusHigh.SERTAD3	2.6231947	2.92141703	6.62167025	4.95E-09	1.44E-07	10.4379501	SERTAD3	assignmentsChromaffin_cells.Chrom_SOX2_statusHigh
assignmentsChromaffin_cells.Chrom_SOX2_statusHigh.UGGT1	-1.0025539	5.82506289	-6.6310178	4.75E-09	1.40E-07	10.436285	UGGT1	assignmentsChromaffin_cells.Chrom_SOX2_statusHigh
assignmentsChromaffin_cells.Chrom_SOX2_statusHigh.SERF2	3.08436637	7.03450104	6.65009599	4.43E-09	1.33E-07	10.4345236	SERF2	assignmentsChromaffin_cells.Chrom_SOX2_statusHigh
assignmentsChromaffin_cells.Chrom_SOX2_statusHigh.TMEM147	3.4712429	4.14045746	6.6336828	4.75E-09	1.40E-07	10.4324981	TMEM147	assignmentsChromaffin_cells.Chrom_SOX2_statusHigh
assignmentsChromaffin_cells.Chrom_SOX2_statusHigh.TMEM35B	2.22927439	3.18829859	6.61442612	5.10E-09	1.47E-07	10.4206515	TMEM35B	assignmentsChromaffin_cells.Chrom_SOX2_statusHigh
assignmentsChromaffin_cells.Chrom_SOX2_statusHigh.ISCU	2.47949509	5.45504143	6.63440706	4.70E-09	1.39E-07	10.410688	ISCU	assignmentsChromaffin_cells.Chrom_SOX2_statusHigh
assignmentsChromaffin_cells.Chrom_SOX2_statusHigh.ARL2BP	1.75835178	4.16566151	6.62192311	4.94E-09	1.44E-07	10.4058406	ARL2BP	assignmentsChromaffin_cells.Chrom_SOX2_statusHigh
assignmentsChromaffin_cells.Chrom_SOX2_statusHigh.CLIC1	2.93884097	5.26759876	6.61841211	5.04E-09	1.46E-07	10.3866445	CLIC1	assignmentsChromaffin_cells.Chrom_SOX2_statusHigh
assignmentsChromaffin_cells.Chrom_SOX2_statusHigh.SUMF1	-1.3230547	7.56581211	-6.626842	4.84E-09	1.42E-07	10.3821575	SUMF1	assignmentsChromaffin_cells.Chrom_SOX2_statusHigh
assignmentsChromaffin_cells.Chrom_SOX2_statusHigh.SLC35B1	1.9457573	4.23443078	6.6164697	5.06E-09	1.46E-07	10.3725589	SLC35B1	assignmentsChromaffin_cells.Chrom_SOX2_statusHigh
assignmentsChromaffin_cells.Chrom_SOX2_statusHigh.NDUFB11	3.392073	4.6338158	6.62037207	5.02E-09	1.46E-07	10.3696589	NDUFB11	assignmentsChromaffin_cells.Chrom_SOX2_statusHigh
assignmentsChromaffin_cells.Chrom_SOX2_statusHigh.MRPL55	2.22760747	3.74196164	6.60839133	5.23E-09	1.50E-07	10.3546637	MRPL55	assignmentsChromaffin_cells.Chrom_SOX2_statusHigh
assignmentsChromaffin_cells.Chrom_SOX2_statusHigh.CCDC124	2.18826065	3.38396752	6.60179624	5.38E-09	1.53E-07	10.3502749	CCDC124	assignmentsChromaffin_cells.Chrom_SOX2_statusHigh
assignmentsChromaffin_cells.Chrom_SOX2_statusHigh.COX7C	3.30856127	6.06235188	6.61873534	5.06E-09	1.46E-07	10.3237479	COX7C	assignmentsChromaffin_cells.Chrom_SOX2_statusHigh
assignmentsChromaffin_cells.Chrom_SOX2_statusHigh.UBR3	-1.3232732	7.9298531	-6.6172494	5.04E-09	1.46E-07	10.322858	UBR3	assignmentsChromaffin_cells.Chrom_SOX2_statusHigh
assignmentsChromaffin_cells.Chrom_SOX2_statusHigh.UBL4A	2.38969156	1.98334021	6.58801124	5.71E-09	1.61E-07	10.3220813	UBL4A	assignmentsChromaffin_cells.Chrom_SOX2_statusHigh
assignmentsChromaffin_cells.Chrom_SOX2_statusHigh.CDKN2C	2.75497474	2.43113556	6.59230791	5.61E-09	1.59E-07	10.3194771	CDKN2C	assignmentsChromaffin_cells.Chrom_SOX2_statusHigh
assignmentsChromaffin_cells.Chrom_SOX2_statusHigh.ZDHHC12	2.63304851	2.1712111	6.58682624	5.74E-09	1.61E-07	10.3141175	ZDHHC12	assignmentsChromaffin_cells.Chrom_SOX2_statusHigh
assignmentsChromaffin_cells.Chrom_SOX2_statusHigh.ARL6IP4	1.67141083	5.84382113	6.60748614	5.25E-09	1.51E-07	10.3055297	ARL6IP4	assignmentsChromaffin_cells.Chrom_SOX2_statusHigh
assignmentsChromaffin_cells.Chrom_SOX2_statusHigh.TMEM219	2.25868271	4.63137777	6.5949334	5.54E-09	1.57E-07	10.2915197	TMEM219	assignmentsChromaffin_cells.Chrom_SOX2_statusHigh
assignmentsChromaffin_cells.Chrom_SOX2_statusHigh.CHCHD2	3.16808139	5.98223257	6.60796707	5.30E-09	1.51E-07	10.2811155	CHCHD2	assignmentsChromaffin_cells.Chrom_SOX2_statusHigh
assignmentsChromaffin_cells.Chrom_SOX2_statusHigh.DYRK1A	-1.4748835	8.26278824	-6.6059601	5.29E-09	1.51E-07	10.2733261	DYRK1A	assignmentsChromaffin_cells.Chrom_SOX2_statusHigh
assignmentsChromaffin_cells.Chrom_SOX2_statusHigh.GPX3	5.82575188	2.71511556	6.57743334	6.03E-09	1.68E-07	10.271344	GPX3	assignmentsChromaffin_cells.Chrom_SOX2_statusHigh
assignmentsChromaffin_cells.Chrom_SOX2_statusHigh.SMPD4	-1.318583	5.24329296	-6.584645	5.79E-09	1.62E-07	10.2541284	SMPD4	assignmentsChromaffin_cells.Chrom_SOX2_statusHigh
assignmentsChromaffin_cells.Chrom_SOX2_statusHigh.VDAC1	1.6605599	5.66985526	6.59379259	5.57E-09	1.58E-07	10.2525647	VDAC1	assignmentsChromaffin_cells.Chrom_SOX2_statusHigh
assignmentsChromaffin_cells.Chrom_SOX2_statusHigh.FTO	-1.1086626	8.17828311	-6.6015626	5.39E-09	1.53E-07	10.2463558	FTO	assignmentsChromaffin_cells.Chrom_SOX2_statusHigh
assignmentsChromaffin_cells.Chrom_SOX2_statusHigh.CIRBP	1.54763812	7.5791416	6.60441438	5.32E-09	1.52E-07	10.2395476	CIRBP	assignmentsChromaffin_cells.Chrom_SOX2_statusHigh
assignmentsChromaffin_cells.Chrom_SOX2_statusHigh.CANT1	1.45273507	3.34496077	6.57215508	6.10E-09	1.69E-07	10.235575	CANT1	assignmentsChromaffin_cells.Chrom_SOX2_statusHigh
assignmentsChromaffin_cells.Chrom_SOX2_statusHigh.RWDD1	1.80701921	5.52970684	6.58571289	5.76E-09	1.62E-07	10.2242114	RWDD1	assignmentsChromaffin_cells.Chrom_SOX2_statusHigh
assignmentsChromaffin_cells.Chrom_SOX2_statusHigh.EID1	2.56251496	6.10358403	6.58911094	5.70E-09	1.61E-07	10.2054268	EID1	assignmentsChromaffin_cells.Chrom_SOX2_statusHigh
assignmentsChromaffin_cells.Chrom_SOX2_statusHigh.ATP6AP2	2.01670753	5.62294231	6.58194606	5.86E-09	1.64E-07	10.2052195	ATP6AP2	assignmentsChromaffin_cells.Chrom_SOX2_statusHigh

assignmentsChromaffin_cells.Chrom_SOX2_statusHigh.PCDHGC3	2.26272661	1.72549969	6.55894718	6.45E-09	1.76E-07	10.2047625	PCDHGC3	assignmentsChromaffin_cells.Chrom_SOX2_statusHigh
assignmentsChromaffin_cells.Chrom_SOX2_statusHigh.ATP2B3	4.98070791	0.78347344	6.56368814	6.39E-09	1.75E-07	10.2041187	ATP2B3	assignmentsChromaffin_cells.Chrom_SOX2_statusHigh
assignmentsChromaffin_cells.Chrom_SOX2_statusHigh.RPL22L1	2.63802789	3.95802273	6.57130518	6.14E-09	1.70E-07	10.2028484	RPL22L1	assignmentsChromaffin_cells.Chrom_SOX2_statusHigh
assignmentsChromaffin_cells.Chrom_SOX2_statusHigh.SRP14	3.26686168	6.2033631	6.58803423	5.76E-09	1.62E-07	10.1986721	SRP14	assignmentsChromaffin_cells.Chrom_SOX2_statusHigh
assignmentsChromaffin_cells.Chrom_SOX2_statusHigh.BRK1	2.26497879	4.99570926	6.5753154	6.02E-09	1.68E-07	10.1942145	BRK1	assignmentsChromaffin_cells.Chrom_SOX2_statusHigh
assignmentsChromaffin_cells.Chrom_SOX2_statusHigh.ICMT	1.77995501	3.07765862	6.55764457	6.49E-09	1.77E-07	10.1827171	ICMT	assignmentsChromaffin_cells.Chrom_SOX2_statusHigh
assignmentsChromaffin_cells.Chrom_SOX2_statusHigh.RTL8B	3.41406706	1.83632782	6.55494808	6.59E-09	1.78E-07	10.1786445	RTL8B	assignmentsChromaffin_cells.Chrom_SOX2_statusHigh
assignmentsChromaffin_cells.Chrom_SOX2_statusHigh.UNC119	1.84002135	3.07576783	6.55982314	6.43E-09	1.76E-07	10.1785263	UNC119	assignmentsChromaffin_cells.Chrom_SOX2_statusHigh
assignmentsChromaffin_cells.Chrom_SOX2_statusHigh.ZPR1	1.64100156	3.02872261	6.55413314	6.59E-09	1.78E-07	10.1730272	ZPR1	assignmentsChromaffin_cells.Chrom_SOX2_statusHigh
assignmentsChromaffin_cells.Chrom_SOX2_statusHigh.PNPLA4	1.50841765	2.58587674	6.55684543	6.51E-09	1.77E-07	10.1728378	PNPLA4	assignmentsChromaffin_cells.Chrom_SOX2_statusHigh
assignmentsChromaffin_cells.Chrom_SOX2_statusHigh.ATP5PF	2.52630976	5.2323418	6.57396076	6.08E-09	1.69E-07	10.1719108	ATP5PF	assignmentsChromaffin_cells.Chrom_SOX2_statusHigh
assignmentsChromaffin_cells.Chrom_SOX2_statusHigh.AP1S1	2.26031394	3.51697926	6.56142225	6.39E-09	1.75E-07	10.1585637	AP1S1	assignmentsChromaffin_cells.Chrom_SOX2_statusHigh
assignmentsChromaffin_cells.Chrom_SOX2_statusHigh.CDKN2AIPN	1.66476932	2.91679776	6.55377929	6.60E-09	1.78E-07	10.1578151	CDKN2AIPNL	assignmentsChromaffin_cells.Chrom_SOX2_statusHigh
assignmentsChromaffin_cells.Chrom_SOX2_statusHigh.SEC61B	1.98923979	6.12937166	6.57432657	6.05E-09	1.68E-07	10.1560039	SEC61B	assignmentsChromaffin_cells.Chrom_SOX2_statusHigh
assignmentsChromaffin_cells.Chrom_SOX2_statusHigh.RBM8A	2.10054215	5.05940668	6.56287575	6.35E-09	1.75E-07	10.1487691	RBM8A	assignmentsChromaffin_cells.Chrom_SOX2_statusHigh
assignmentsChromaffin_cells.Chrom_SOX2_statusHigh.NOP16	2.20376823	2.5766315	6.54633732	6.81E-09	1.83E-07	10.1421725	NOP16	assignmentsChromaffin_cells.Chrom_SOX2_statusHigh
assignmentsChromaffin_cells.Chrom_SOX2_statusHigh.IK	1.77604046	5.18353412	6.56200476	6.37E-09	1.75E-07	10.1405846	IK	assignmentsChromaffin_cells.Chrom_SOX2_statusHigh
assignmentsChromaffin_cells.Chrom_SOX2_statusHigh.RPL36AL	3.42441893	5.45273482	6.56573677	6.33E-09	1.75E-07	10.1288249	RPL36AL	assignmentsChromaffin_cells.Chrom_SOX2_statusHigh
assignmentsChromaffin_cells.Chrom_SOX2_statusHigh.RPS5	3.94462257	6.30304099	6.57264926	6.15E-09	1.70E-07	10.1286715	RPS5	assignmentsChromaffin_cells.Chrom_SOX2_statusHigh
assignmentsChromaffin_cells.Chrom_SOX2_statusHigh.ECM1	3.45219968	2.36290775	6.53949196	7.04E-09	1.88E-07	10.1190984	ECM1	assignmentsChromaffin_cells.Chrom_SOX2_statusHigh
assignmentsChromaffin_cells.Chrom_SOX2_statusHigh.WDR59	-1.1621101	6.0965846	-6.5553103	6.55E-09	1.78E-07	10.109512	WDR59	assignmentsChromaffin_cells.Chrom_SOX2_statusHigh
assignmentsChromaffin_cells.Chrom_SOX2_statusHigh.ATP5PB	1.68203485	5.06293355	6.54978756	6.71E-09	1.81E-07	10.0922748	ATP5PB	assignmentsChromaffin_cells.Chrom_SOX2_statusHigh
assignmentsChromaffin_cells.Chrom_SOX2_statusHigh.ATP6V0C	3.05351707	5.83693552	6.5577232	6.55E-09	1.78E-07	10.0776159	ATP6V0C	assignmentsChromaffin_cells.Chrom_SOX2_statusHigh
assignmentsChromaffin_cells.Chrom_SOX2_statusHigh.MPG	1.6478037	4.44438929	6.53432457	7.16E-09	1.91E-07	10.0508492	MPG	assignmentsChromaffin_cells.Chrom_SOX2_statusHigh
assignmentsChromaffin_cells.Chrom_SOX2_statusHigh.NDUFAB1	2.03043037	5.11995219	6.54289955	6.91E-09	1.85E-07	10.0508335	NDUFAB1	assignmentsChromaffin_cells.Chrom_SOX2_statusHigh
assignmentsChromaffin_cells.Chrom_SOX2_statusHigh.MAD2L2	2.17063786	2.30002746	6.52143695	7.57E-09	1.99E-07	10.0507258	MAD2L2	assignmentsChromaffin_cells.Chrom_SOX2_statusHigh
assignmentsChromaffin_cells.Chrom_SOX2_statusHigh.ANKRD9	2.62348772	2.26515801	6.52209623	7.54E-09	1.99E-07	10.049803	ANKRD9	assignmentsChromaffin_cells.Chrom_SOX2_statusHigh
assignmentsChromaffin_cells.Chrom_SOX2_statusHigh.NIT2	1.30058924	4.08733606	6.5311843	7.26E-09	1.93E-07	10.0471962	NIT2	assignmentsChromaffin_cells.Chrom_SOX2_statusHigh
assignmentsChromaffin_cells.Chrom_SOX2_statusHigh.CETN2	2.84954108	2.36249408	6.52495277	7.48E-09	1.97E-07	10.0397011	CETN2	assignmentsChromaffin_cells.Chrom_SOX2_statusHigh
assignmentsChromaffin_cells.Chrom_SOX2_statusHigh.C22orf39	1.8131454	3.96490442	6.53215514	7.23E-09	1.92E-07	10.0379706	C22orf39	assignmentsChromaffin_cells.Chrom_SOX2_statusHigh
assignmentsChromaffin_cells.Chrom_SOX2_statusHigh.TPBG	4.1684794	1.61805369	6.52091444	7.61E-09	2.00E-07	10.0336824	TPBG	assignmentsChromaffin_cells.Chrom_SOX2_statusHigh
assignmentsChromaffin_cells.Chrom_SOX2_statusHigh.PET100	2.63264173	4.35530704	6.53293497	7.23E-09	1.92E-07	10.0254427	PET100	assignmentsChromaffin_cells.Chrom_SOX2_statusHigh
assignmentsChromaffin_cells.Chrom_SOX2_statusHigh.UBB	4.04885633	5.86050928	6.53650998	7.17E-09	1.91E-07	9.99418061	UBB	assignmentsChromaffin_cells.Chrom_SOX2_statusHigh
assignmentsChromaffin_cells.Chrom_SOX2_statusHigh.NDUFA11	2.6540204	5.51999049	6.53136831	7.29E-09	1.93E-07	9.9848935	NDUFA11	assignmentsChromaffin_cells.Chrom_SOX2_statusHigh
assignmentsChromaffin_cells.Chrom_SOX2_statusHigh.MRPS17	2.28474725	2.35551757	6.50838259	8.00E-09	2.08E-07	9.98422558	MRPS17	assignmentsChromaffin_cells.Chrom_SOX2_statusHigh
assignmentsChromaffin_cells.Chrom_SOX2_statusHigh.DPH3	1.92872719	3.52630519	6.51347603	7.83E-09	2.05E-07	9.9832925	DPH3	assignmentsChromaffin_cells.Chrom_SOX2_statusHigh
assignmentsChromaffin_cells.Chrom_SOX2_statusHigh.R3HDM1	-1.4784413	7.4072019	-6.5311132	7.26E-09	1.93E-07	9.97819326	R3HDM1	assignmentsChromaffin_cells.Chrom_SOX2_statusHigh
assignmentsChromaffin_cells.Chrom_SOX2_statusHigh.TMEM203	2.19413354	2.15789673	6.50394668	8.15E-09	2.12E-07	9.97024588	TMEM203	assignmentsChromaffin_cells.Chrom_SOX2_statusHigh
assignmentsChromaffin_cells.Chrom_SOX2_statusHigh.NOP53	2.5103291	5.18052723	6.51954069	7.65E-09	2.00E-07	9.96099223	NOP53	assignmentsChromaffin_cells.Chrom_SOX2_statusHigh
assignmentsChromaffin_cells.Chrom_SOX2_statusHigh.EIF4A2	1.86585922	7.12749319	6.52821353	7.35E-09	1.94E-07	9.94984613	EIF4A2	assignmentsChromaffin_cells.Chrom_SOX2_statusHigh
assignmentsChromaffin_cells.Chrom_SOX2_statusHigh.GFER	1.78090319	2.33707019	6.49828616	8.34E-09	2.16E-07	9.94764101	GFER	assignmentsChromaffin_cells.Chrom_SOX2_statusHigh
assignmentsChromaffin_cells.Chrom_SOX2_statusHigh.KANSL3	-1.2420759	5.47688845	-6.5029994	8.18E-09	2.12E-07	9.93495139	KANSL3	assignmentsChromaffin_cells.Chrom_SOX2_statusHigh
assignmentsChromaffin_cells.Chrom_SOX2_statusHigh.TMEM11	1.41543301	3.68049538	6.4994177	8.30E-09	2.15E-07	9.92979072	TMEM11	assignmentsChromaffin_cells.Chrom_SOX2_statusHigh
assignmentsChromaffin_cells.Chrom_SOX2_statusHigh.EXOC8	1.56509177	3.13010901	6.4959299	8.43E-09	2.17E-07	9.92468068	EXOC8	assignmentsChromaffin_cells.Chrom_SOX2_statusHigh
assignmentsChromaffin_cells.Chrom_SOX2_statusHigh.THAP1	1.82398048	2.36430579	6.49230849	8.56E-09	2.20E-07	9.92396516	THAP1	assignmentsChromaffin_cells.Chrom_SOX2_statusHigh
assignmentsChromaffin_cells.Chrom_SOX2_statusHigh.PRMT6	1.75437483	1.48356382	6.48854206	8.70E-09	2.22E-07	9.92118338	PRMT6	assignmentsChromaffin_cells.Chrom_SOX2_statusHigh
assignmentsChromaffin_cells.Chrom_SOX2_statusHigh.LAGE3	3.21341377	2.62059702	6.49032425	8.66E-09	2.22E-07	9.90087156	LAGE3	assignmentsChromaffin_cells.Chrom_SOX2_statusHigh
assignmentsChromaffin_cells.Chrom_SOX2_statusHigh.VAMP2	2.06256655	6.17538093	6.5111826	7.90E-09	2.06E-07	9.89205547	VAMP2	assignmentsChromaffin_cells.Chrom_SOX2_statusHigh
assignmentsChromaffin_cells.Chrom_SOX2_statusHigh.TMED2	1.94619826	5.1103277	6.50171747	8.22E-09	2.13E-07	9.88551063	TMED2	assignmentsChromaffin_cells.Chrom_SOX2_statusHigh
assignmentsChromaffin_cells.Chrom_SOX2_statusHigh.EIF3I	2.39592154	4.17778811	6.48692654	8.76E-09	2.24E-07	9.87511448	EIF3I	assignmentsChromaffin_cells.Chrom_SOX2_statusHigh
assignmentsChromaffin_cells.Chrom_SOX2_statusHigh.RHOC	2.51562078	4.40312553	6.48603695	8.79E-09	2.24E-07	9.87384013	RHOC	assignmentsChromaffin_cells.Chrom_SOX2_statusHigh
assignmentsChromaffin_cells.Chrom_SOX2_statusHigh.ATP5MC1	2.47469218	4.68135025	6.49725977	8.40E-09	2.17E-07	9.86956308	ATP5MC1	assignmentsChromaffin_cells.Chrom_SOX2_statusHigh
assignmentsChromaffin_cells.Chrom_SOX2_statusHigh.TMEM70	2.37685444	3.3593318	6.48286028	8.91E-09	2.26E-07	9.86436367	TMEM70	assignmentsChromaffin_cells.Chrom_SOX2_statusHigh
assignmentsChromaffin_cells.Chrom_SOX2_statusHigh.BICRAL	-1.8846396	6.41862936	-6.492205	8.56E-09	2.20E-07	9.86405785	BICRAL	assignmentsChromaffin_cells.Chrom_SOX2_statusHigh
assignmentsChromaffin_cells.Chrom_SOX2_statusHigh.B2M	3.26036001	8.71900565	6.51477197	7.86E-09	2.05E-07	9.8578357	B2M	assignmentsChromaffin_cells.Chrom_SOX2_statusHigh
assignmentsChromaffin_cells.Chrom_SOX2_statusHigh.PHB2	2.10127801	4.35323244	6.48575129	8.80E-09	2.24E-07	9.84755082	PHB2	assignmentsChromaffin_cells.Chrom_SOX2_statusHigh
assignmentsChromaffin_cells.Chrom_SOX2_statusHigh.SNRPE	2.26764531	4.24346042	6.48438686	8.85E-09	2.25E-07	9.83356864	SNRPE	assignmentsChromaffin_cells.Chrom_SOX2_statusHigh
assignmentsChromaffin_cells.Chrom_SOX2_statusHigh.SLC35F6	1.64608177	2.99704261	6.47016492	9.40E-09	2.36E-07	9.82406431	SLC35F6	assignmentsChromaffin_cells.Chrom_SOX2_statusHigh
assignmentsChromaffin_cells.Chrom_SOX2_statusHigh.NME4	2.00973279	4.17978493	6.4828766	8.91E-09	2.26E-07	9.82172509	NME4	assignmentsChromaffin_cells.Chrom_SOX2_statusHigh
assignmentsChromaffin_cells.Chrom_SOX2_statusHigh.METTL23	1.35195701	4.07834311	6.47004616	9.40E-09	2.36E-07	9.79922912	METTL23	assignmentsChromaffin_cells.Chrom_SOX2_statusHigh
assignmentsChromaffin_cells.Chrom_SOX2_statusHigh.ATP5IF1	2.52300916	4.79327646	6.47682316	9.16E-09	2.32E-07	9.79436362	ATP5IF1	assignmentsChromaffin_cells.Chrom_SOX2_statusHigh
assignmentsChromaffin_cells.Chrom_SOX2_statusHigh.BOD1	1.24927853	4.05221346	6.46893435	9.45E-09	2.37E-07	9.7867192	BOD1	assignmentsChromaffin_cells.Chrom_SOX2_statusHigh
assignmentsChromaffin_cells.Chrom_SOX2_statusHigh.PFN1	3.28622489	5.83196514	6.47562878	9.27E-09	2.34E-07	9.7742176	PFN1	assignmentsChromaffin_cells.Chrom_SOX2_statusHigh
assignmentsChromaffin_cells.Chrom_SOX2_statusHigh.CLTB	1.64684494	4.95832872	6.47064251	9.38E-09	2.36E-07	9.76726707	CLTB	assignmentsChromaffin_cells.Chrom_SOX2_statusHigh

assignmentsChromaffin_cells.Chrom_SOX2_statusHigh.MINCR	2.34703765	1.52519826	6.4521582	1.01E-08	2.51E-07	9.76536161	MINCR	assignmentsChromaffin_cells.Chrom_SOX2_statusHigh
assignmentsChromaffin_cells.Chrom_SOX2_statusHigh.BUD23	1.43142964	5.06395739	6.46819965	9.48E-09	2.37E-07	9.74920254	BUD23	assignmentsChromaffin_cells.Chrom_SOX2_statusHigh
assignmentsChromaffin_cells.Chrom_SOX2_statusHigh.S100B	6.21536663	1.69247732	6.4487881	1.04E-08	2.56E-07	9.74729673	S100B	assignmentsChromaffin_cells.Chrom_SOX2_statusHigh
assignmentsChromaffin_cells.Chrom_SOX2_statusHigh.MDK	4.32478245	3.04183077	6.45478306	1.01E-08	2.51E-07	9.73799673	MDK	assignmentsChromaffin_cells.Chrom_SOX2_statusHigh
assignmentsChromaffin_cells.Chrom_SOX2_statusHigh.NDUFS7	1.98646248	4.86888712	6.46295854	9.69E-09	2.42E-07	9.72336325	NDUFS7	assignmentsChromaffin_cells.Chrom_SOX2_statusHigh
assignmentsChromaffin_cells.Chrom_SOX2_statusHigh.MARK3	-1.1247889	7.90812337	-6.4696531	9.42E-09	2.36E-07	9.71863789	MARK3	assignmentsChromaffin_cells.Chrom_SOX2_statusHigh
assignmentsChromaffin_cells.Chrom_SOX2_statusHigh.C2CD5	-1.3638046	6.69925843	-6.4624368	9.71E-09	2.42E-07	9.71483575	C2CD5	assignmentsChromaffin_cells.Chrom_SOX2_statusHigh
assignmentsChromaffin_cells.Chrom_SOX2_statusHigh.LAMTOR1	2.4003663	4.64708791	6.4534232	1.01E-08	2.51E-07	9.7019309	LAMTOR1	assignmentsChromaffin_cells.Chrom_SOX2_statusHigh
assignmentsChromaffin_cells.Chrom_SOX2_statusHigh.RNF7	2.42026543	4.85424836	6.45405684	1.01E-08	2.51E-07	9.70158223	RNF7	assignmentsChromaffin_cells.Chrom_SOX2_statusHigh
assignmentsChromaffin_cells.Chrom_SOX2_statusHigh.DDT	2.7608297	3.83364492	6.44634743	1.04E-08	2.57E-07	9.68634534	DDT	assignmentsChromaffin_cells.Chrom_SOX2_statusHigh
assignmentsChromaffin_cells.Chrom_SOX2_statusHigh.MAGEH1	3.75597076	2.57602383	6.4351546	1.10E-08	2.68E-07	9.67403125	MAGEH1	assignmentsChromaffin_cells.Chrom_SOX2_statusHigh
assignmentsChromaffin_cells.Chrom_SOX2_statusHigh.AIMP1	1.06548768	5.45240325	6.44830267	1.03E-08	2.55E-07	9.66654859	AIMP1	assignmentsChromaffin_cells.Chrom_SOX2_statusHigh
assignmentsChromaffin_cells.Chrom_SOX2_statusHigh.SPRYD3	1.79005508	3.39489326	6.43324823	1.10E-08	2.68E-07	9.65801609	SPRYD3	assignmentsChromaffin_cells.Chrom_SOX2_statusHigh
assignmentsChromaffin_cells.Chrom_SOX2_statusHigh.NME2	3.69329525	4.91927101	6.43754727	1.09E-08	2.67E-07	9.63164247	NME2	assignmentsChromaffin_cells.Chrom_SOX2_statusHigh
assignmentsChromaffin_cells.Chrom_SOX2_statusHigh.CDK8	-1.1906941	6.94505895	-6.4403972	1.07E-08	2.62E-07	9.62783187	CDK8	assignmentsChromaffin_cells.Chrom_SOX2_statusHigh
assignmentsChromaffin_cells.Chrom_SOX2_statusHigh.DOHH	1.87228779	2.23343711	6.41864431	1.17E-08	2.82E-07	9.62468046	DOHH	assignmentsChromaffin_cells.Chrom_SOX2_statusHigh
assignmentsChromaffin_cells.Chrom_SOX2_statusHigh.PTPMT1	2.16597539	2.56990707	6.41944187	1.16E-08	2.82E-07	9.62324886	PTPMT1	assignmentsChromaffin_cells.Chrom_SOX2_statusHigh
assignmentsChromaffin_cells.Chrom_SOX2_statusHigh.ARHGDIA	2.10835167	5.0636079	6.43480678	1.09E-08	2.67E-07	9.62212231	ARHGDIA	assignmentsChromaffin_cells.Chrom_SOX2_statusHigh
assignmentsChromaffin_cells.Chrom_SOX2_statusHigh.UQCC1	-1.4481042	5.97394109	-6.4347796	1.09E-08	2.67E-07	9.61956653	UQCC1	assignmentsChromaffin_cells.Chrom_SOX2_statusHigh
assignmentsChromaffin_cells.Chrom_SOX2_statusHigh.RBM43	2.17938018	2.55883561	6.41330188	1.19E-08	2.87E-07	9.6138413	RBM43	assignmentsChromaffin_cells.Chrom_SOX2_statusHigh
assignmentsChromaffin_cells.Chrom_SOX2_statusHigh.ATP5F1B	2.06491925	6.17913751	6.43492992	1.09E-08	2.67E-07	9.57716255	ATP5F1B	assignmentsChromaffin_cells.Chrom_SOX2_statusHigh
assignmentsChromaffin_cells.Chrom_SOX2_statusHigh.ATL2	-1.1951185	6.48823142	-6.4256252	1.13E-08	2.76E-07	9.56794718	ATL2	assignmentsChromaffin_cells.Chrom_SOX2_statusHigh
assignmentsChromaffin_cells.Chrom_SOX2_statusHigh.TCEAL8	2.64104236	3.47192033	6.41441951	1.19E-08	2.87E-07	9.56212249	TCEAL8	assignmentsChromaffin_cells.Chrom_SOX2_statusHigh
assignmentsChromaffin_cells.Chrom_SOX2_statusHigh.TACO1	1.53175769	2.67677265	6.40454677	1.24E-08	2.97E-07	9.55359898	TACO1	assignmentsChromaffin_cells.Chrom_SOX2_statusHigh
assignmentsChromaffin_cells.Chrom_SOX2_statusHigh.SBDS	2.47704261	5.45657111	6.42185628	1.16E-08	2.80E-07	9.54238672	SBDS	assignmentsChromaffin_cells.Chrom_SOX2_statusHigh
assignmentsChromaffin_cells.Chrom_SOX2_statusHigh.UBR2	-0.8668554	7.62127324	-6.4238797	1.14E-08	2.77E-07	9.53348914	UBR2	assignmentsChromaffin_cells.Chrom_SOX2_statusHigh
assignmentsChromaffin_cells.Chrom_SOX2_statusHigh.MED13L	-1.2385362	8.964327	-6.4275122	1.13E-08	2.74E-07	9.5271606	MED13L	assignmentsChromaffin_cells.Chrom_SOX2_statusHigh
assignmentsChromaffin_cells.Chrom_SOX2_statusHigh.SMIM26	2.92099464	3.71511851	6.40380027	1.25E-08	2.98E-07	9.51257604	SMIM26	assignmentsChromaffin_cells.Chrom_SOX2_statusHigh
assignmentsChromaffin_cells.Chrom_SOX2_statusHigh.NBAT1	3.32629418	0.34784789	6.46222675	9.72E-09	2.42E-07	9.50596908	NBAT1	assignmentsChromaffin_cells.Chrom_SOX2_statusHigh
assignmentsChromaffin_cells.Chrom_SOX2_statusHigh.BECN1	1.02078402	4.53439214	6.40056587	1.26E-08	3.00E-07	9.50370167	BECN1	assignmentsChromaffin_cells.Chrom_SOX2_statusHigh
assignmentsChromaffin_cells.Chrom_SOX2_statusHigh.BEX3	2.16180901	5.49994637	6.41495396	1.19E-08	2.86E-07	9.50274489	BEX3	assignmentsChromaffin_cells.Chrom_SOX2_statusHigh
assignmentsChromaffin_cells.Chrom_SOX2_statusHigh.RPN2	1.3458383	6.3426778	6.41406404	1.19E-08	2.87E-07	9.49610026	RPN2	assignmentsChromaffin_cells.Chrom_SOX2_statusHigh
assignmentsChromaffin_cells.Chrom_SOX2_statusHigh.FIBP	2.23561034	3.47155772	6.39402943	1.30E-08	3.06E-07	9.49207593	FIBP	assignmentsChromaffin_cells.Chrom_SOX2_statusHigh
assignmentsChromaffin_cells.Chrom_SOX2_statusHigh.APLN	4.62748912	0.41928188	6.39071069	1.32E-08	3.10E-07	9.49154817	APLN	assignmentsChromaffin_cells.Chrom_SOX2_statusHigh
assignmentsChromaffin_cells.Chrom_SOX2_statusHigh.TXN2	1.90875429	4.14376555	6.39785378	1.28E-08	3.02E-07	9.48934662	TXN2	assignmentsChromaffin_cells.Chrom_SOX2_statusHigh
assignmentsChromaffin_cells.Chrom_SOX2_statusHigh.HCCS	1.63128876	2.33941203	6.38341737	1.35E-08	3.17E-07	9.48501721	HCCS	assignmentsChromaffin_cells.Chrom_SOX2_statusHigh
assignmentsChromaffin_cells.Chrom_SOX2_statusHigh.ILF3-DT	1.5065318	4.43133454	6.39757459	1.28E-08	3.02E-07	9.48370741	ILF3-DT	assignmentsChromaffin_cells.Chrom_SOX2_statusHigh
assignmentsChromaffin_cells.Chrom_SOX2_statusHigh.HMG20B	1.66759438	4.01793128	6.38952163	1.32E-08	3.10E-07	9.48015994	HMG20B	assignmentsChromaffin_cells.Chrom_SOX2_statusHigh
assignmentsChromaffin_cells.Chrom_SOX2_statusHigh.PFN2	1.70488593	3.10901717	6.3926142	1.30E-08	3.07E-07	9.47911575	PFN2	assignmentsChromaffin_cells.Chrom_SOX2_statusHigh
assignmentsChromaffin_cells.Chrom_SOX2_statusHigh.C1D	1.26224373	5.19077348	6.3977789	1.28E-08	3.02E-07	9.4628633	C1D	assignmentsChromaffin_cells.Chrom_SOX2_statusHigh
assignmentsChromaffin_cells.Chrom_SOX2_statusHigh.ATF2	-0.8867459	6.83686151	-6.4017543	1.25E-08	2.99E-07	9.45825381	ATF2	assignmentsChromaffin_cells.Chrom_SOX2_statusHigh
assignmentsChromaffin_cells.Chrom_SOX2_statusHigh.GIGYF2	-1.0852605	7.46737822	-6.4050793	1.24E-08	2.96E-07	9.45682098	GIGYF2	assignmentsChromaffin_cells.Chrom_SOX2_statusHigh
assignmentsChromaffin_cells.Chrom_SOX2_statusHigh.GPX4	2.88864807	5.73493472	6.40125541	1.27E-08	3.02E-07	9.43826219	GPX4	assignmentsChromaffin_cells.Chrom_SOX2_statusHigh
assignmentsChromaffin_cells.Chrom_SOX2_statusHigh.PARP11	-1.3033302	5.26184749	-6.3799006	1.38E-08	3.20E-07	9.43821539	PARP11	assignmentsChromaffin_cells.Chrom_SOX2_statusHigh
assignmentsChromaffin_cells.Chrom_SOX2_statusHigh.PRDX5	3.26038451	4.98345598	6.39590533	1.30E-08	3.06E-07	9.43671382	PRDX5	assignmentsChromaffin_cells.Chrom_SOX2_statusHigh
assignmentsChromaffin_cells.Chrom_SOX2_statusHigh.PHAX	1.24251639	4.52463744	6.38249849	1.36E-08	3.17E-07	9.42347487	PHAX	assignmentsChromaffin_cells.Chrom_SOX2_statusHigh
assignmentsChromaffin_cells.Chrom_SOX2_statusHigh.FRMD4B	-5.120199	7.13517813	-6.3733483	1.43E-08	3.30E-07	9.42064165	FRMD4B	assignmentsChromaffin_cells.Chrom_SOX2_statusHigh
assignmentsChromaffin_cells.Chrom_SOX2_statusHigh.TMA7	3.05826763	5.55029782	6.39321162	1.31E-08	3.09E-07	9.417565	TMA7	assignmentsChromaffin_cells.Chrom_SOX2_statusHigh
assignmentsChromaffin_cells.Chrom_SOX2_statusHigh.SF3B6	2.64414421	4.30780751	6.38319267	1.36E-08	3.17E-07	9.41509926	SF3B6	assignmentsChromaffin_cells.Chrom_SOX2_statusHigh
assignmentsChromaffin_cells.Chrom_SOX2_statusHigh.C15orf61	1.87517507	3.56771664	6.37614766	1.40E-08	3.25E-07	9.40327549	C15orf61	assignmentsChromaffin_cells.Chrom_SOX2_statusHigh
assignmentsChromaffin_cells.Chrom_SOX2_statusHigh.GPAA1	2.02644288	4.17180486	6.37561213	1.40E-08	3.25E-07	9.39057618	GPAA1	assignmentsChromaffin_cells.Chrom_SOX2_statusHigh
assignmentsChromaffin_cells.Chrom_SOX2_statusHigh.CCT7	1.64970201	5.03790395	6.37570292	1.40E-08	3.25E-07	9.37144616	CCT7	assignmentsChromaffin_cells.Chrom_SOX2_statusHigh
assignmentsChromaffin_cells.Chrom_SOX2_statusHigh.CTSD	3.18304296	5.23718851	6.37284904	1.43E-08	3.30E-07	9.36857852	CTSD	assignmentsChromaffin_cells.Chrom_SOX2_statusHigh
assignmentsChromaffin_cells.Chrom_SOX2_statusHigh.ACP1	1.41545118	4.95728828	6.37397608	1.41E-08	3.26E-07	9.36799688	ACP1	assignmentsChromaffin_cells.Chrom_SOX2_statusHigh
assignmentsChromaffin_cells.Chrom_SOX2_statusHigh.PPIA	2.67232313	6.61008333	6.38465047	1.36E-08	3.17E-07	9.36117754	PPIA	assignmentsChromaffin_cells.Chrom_SOX2_statusHigh
assignmentsChromaffin_cells.Chrom_SOX2_statusHigh.ALKBH7	3.36023249	3.42979995	6.36556929	1.47E-08	3.38E-07	9.35848685	ALKBH7	assignmentsChromaffin_cells.Chrom_SOX2_statusHigh
assignmentsChromaffin_cells.Chrom_SOX2_statusHigh.BRD3OS	1.53746969	1.52581592	6.34882424	1.57E-08	3.56E-07	9.35412516	BRD3OS	assignmentsChromaffin_cells.Chrom_SOX2_statusHigh
assignmentsChromaffin_cells.Chrom_SOX2_statusHigh.SRSF8	1.93865411	3.88695798	6.36036854	1.49E-08	3.42E-07	9.34373588	SRSF8	assignmentsChromaffin_cells.Chrom_SOX2_statusHigh
assignmentsChromaffin_cells.Chrom_SOX2_statusHigh.NDUFC2	2.76994116	5.24859439	6.37186934	1.43E-08	3.30E-07	9.33873262	NDUFC2	assignmentsChromaffin_cells.Chrom_SOX2_statusHigh
assignmentsChromaffin_cells.Chrom_SOX2_statusHigh.BTF3	2.94066828	5.93715393	6.37101296	1.44E-08	3.31E-07	9.31924894	BTF3	assignmentsChromaffin_cells.Chrom_SOX2_statusHigh
assignmentsChromaffin_cells.Chrom_SOX2_statusHigh.UCP2	3.20841004	2.07350755	6.34608267	1.59E-08	3.59E-07	9.3164085	UCP2	assignmentsChromaffin_cells.Chrom_SOX2_statusHigh
assignmentsChromaffin_cells.Chrom_SOX2_statusHigh.NDUFA6	2.4944607	4.42238558	6.35906995	1.51E-08	3.44E-07	9.31476927	NDUFA6	assignmentsChromaffin_cells.Chrom_SOX2_statusHigh
assignmentsChromaffin_cells.Chrom_SOX2_statusHigh.EDN3	6.08002994	-0.6703588	6.38819308	1.34E-08	3.14E-07	9.30947391	EDN3	assignmentsChromaffin_cells.Chrom_SOX2_statusHigh
assignmentsChromaffin_cells.Chrom_SOX2_statusHigh.NDUFB2	2.3726236	5.56745982	6.36508343	1.47E-08	3.37E-07	9.30745036	NDUFB2	assignmentsChromaffin_cells.Chrom_SOX2_statusHigh

assignmentsChromaffin_cells.Chrom_SOX2_statusHigh.PSMB4	2.29540057	4.57529138	6.35492568	1.53E-08	3.48E-07	9.30082203	PSMB4	assignmentsChromaffin_cells.Chrom_SOX2_statusHigh
assignmentsChromaffin_cells.Chrom_SOX2_statusHigh.FRYL	-0.98827	8.07940687	-6.3666177	1.45E-08	3.34E-07	9.29571873	FRYL	assignmentsChromaffin_cells.Chrom_SOX2_statusHigh
assignmentsChromaffin_cells.Chrom_SOX2_statusHigh.C9orf78	1.97674003	4.30408633	6.34677781	1.58E-08	3.58E-07	9.28653117	C9orf78	assignmentsChromaffin_cells.Chrom_SOX2_statusHigh
assignmentsChromaffin_cells.Chrom_SOX2_statusHigh.SPAG7	1.91276925	4.19217179	6.34494736	1.59E-08	3.59E-07	9.28555501	SPAG7	assignmentsChromaffin_cells.Chrom_SOX2_statusHigh
assignmentsChromaffin_cells.Chrom_SOX2_statusHigh.HTRA1	3.33351299	4.78190026	6.3478584	1.59E-08	3.59E-07	9.27363227	HTRA1	assignmentsChromaffin_cells.Chrom_SOX2_statusHigh
assignmentsChromaffin_cells.Chrom_SOX2_statusHigh.AKR1E2	4.31946066	1.90565126	6.34396278	1.60E-08	3.61E-07	9.27124621	AKR1E2	assignmentsChromaffin_cells.Chrom_SOX2_statusHigh
assignmentsChromaffin_cells.Chrom_SOX2_statusHigh.ARAP3	3.83563866	2.74909955	6.32949274	1.70E-08	3.80E-07	9.26225252	ARAP3	assignmentsChromaffin_cells.Chrom_SOX2_statusHigh
assignmentsChromaffin_cells.Chrom_SOX2_statusHigh.SLC25A12	-1.3362835	6.56129721	-6.3531037	1.54E-08	3.50E-07	9.25396233	SLC25A12	assignmentsChromaffin_cells.Chrom_SOX2_statusHigh
assignmentsChromaffin_cells.Chrom_SOX2_statusHigh.HTT	-1.0615934	7.14113569	-6.3531746	1.54E-08	3.50E-07	9.25262347	HTT	assignmentsChromaffin_cells.Chrom_SOX2_statusHigh
assignmentsChromaffin_cells.Chrom_SOX2_statusHigh.MRPL20	1.91245206	4.92769856	6.34312509	1.61E-08	3.61E-07	9.25251266	MRPL20	assignmentsChromaffin_cells.Chrom_SOX2_statusHigh
assignmentsChromaffin_cells.Chrom_SOX2_statusHigh.USP40	-1.4437734	6.00563958	-6.342761	1.61E-08	3.61E-07	9.24866595	USP40	assignmentsChromaffin_cells.Chrom_SOX2_statusHigh
assignmentsChromaffin_cells.Chrom_SOX2_statusHigh.SPAG4	2.82332148	1.02931103	6.32098922	1.76E-08	3.90E-07	9.24751325	SPAG4	assignmentsChromaffin_cells.Chrom_SOX2_statusHigh
assignmentsChromaffin_cells.Chrom_SOX2_statusHigh.WDR7	-1.0813133	7.42300461	-6.350496	1.56E-08	3.54E-07	9.23875845	WDR7	assignmentsChromaffin_cells.Chrom_SOX2_statusHigh
assignmentsChromaffin_cells.Chrom_SOX2_statusHigh.GNG5	2.68701989	4.98422128	6.33329261	1.68E-08	3.77E-07	9.21872388	GNG5	assignmentsChromaffin_cells.Chrom_SOX2_statusHigh
assignmentsChromaffin_cells.Chrom_SOX2_statusHigh.GON7	1.70057818	2.58373007	6.31412311	1.81E-08	3.99E-07	9.19804175	GON7	assignmentsChromaffin_cells.Chrom_SOX2_statusHigh
assignmentsChromaffin_cells.Chrom_SOX2_statusHigh.TOMM6	2.79101149	4.3219496	6.32782035	1.72E-08	3.83E-07	9.1904642	TOMM6	assignmentsChromaffin_cells.Chrom_SOX2_statusHigh
assignmentsChromaffin_cells.Chrom_SOX2_statusHigh.OS9	1.34503497	5.71181385	6.33191472	1.68E-08	3.77E-07	9.18523724	OS9	assignmentsChromaffin_cells.Chrom_SOX2_statusHigh
assignmentsChromaffin_cells.Chrom_SOX2_statusHigh.QPRT	3.53629145	2.8023243	6.31086435	1.85E-08	4.06E-07	9.17276729	QPRT	assignmentsChromaffin_cells.Chrom_SOX2_statusHigh
assignmentsChromaffin_cells.Chrom_SOX2_statusHigh.PSMD8	2.2051014	5.36461049	6.32969002	1.70E-08	3.80E-07	9.16954174	PSMD8	assignmentsChromaffin_cells.Chrom_SOX2_statusHigh
assignmentsChromaffin_cells.Chrom_SOX2_statusHigh.SPTSSA	2.29893121	3.60160852	6.31354126	1.82E-08	3.99E-07	9.16632414	SPTSSA	assignmentsChromaffin_cells.Chrom_SOX2_statusHigh
assignmentsChromaffin_cells.Chrom_SOX2_statusHigh.TMEM60	1.94316506	3.3897434	6.31352732	1.82E-08	3.99E-07	9.15220539	TMEM60	assignmentsChromaffin_cells.Chrom_SOX2_statusHigh
assignmentsChromaffin_cells.Chrom_SOX2_statusHigh.HECTD4	-1.0826627	7.4539123	-6.3292967	1.70E-08	3.80E-07	9.14053531	HECTD4	assignmentsChromaffin_cells.Chrom_SOX2_statusHigh
assignmentsChromaffin_cells.Chrom_SOX2_statusHigh.COX5B	2.67530716	5.75287137	6.32630199	1.73E-08	3.85E-07	9.13825476	COX5B	assignmentsChromaffin_cells.Chrom_SOX2_statusHigh
assignmentsChromaffin_cells.Chrom_SOX2_statusHigh.GTF3C6	2.4091947	3.66368332	6.30787906	1.86E-08	4.06E-07	9.13787181	GTF3C6	assignmentsChromaffin_cells.Chrom_SOX2_statusHigh
assignmentsChromaffin_cells.Chrom_SOX2_statusHigh.TMEM167A	1.50102393	5.23802476	6.3177974	1.79E-08	3.93E-07	9.13513399	TMEM167A	assignmentsChromaffin_cells.Chrom_SOX2_statusHigh
assignmentsChromaffin_cells.Chrom_SOX2_statusHigh.TADA3	1.35768442	4.16705975	6.30817872	1.86E-08	4.06E-07	9.13312164	TADA3	assignmentsChromaffin_cells.Chrom_SOX2_statusHigh
assignmentsChromaffin_cells.Chrom_SOX2_statusHigh.COX4I1	2.88068059	6.22750927	6.32787339	1.73E-08	3.84E-07	9.12864242	COX4I1	assignmentsChromaffin_cells.Chrom_SOX2_statusHigh
assignmentsChromaffin_cells.Chrom_SOX2_statusHigh.TTC28	-1.7350236	8.27035702	-6.325336	1.73E-08	3.84E-07	9.12074439	TTC28	assignmentsChromaffin_cells.Chrom_SOX2_statusHigh
assignmentsChromaffin_cells.Chrom_SOX2_statusHigh.SUPT3H	-1.2092799	7.3837654	-6.3189423	1.78E-08	3.93E-07	9.11826829	SUPT3H	assignmentsChromaffin_cells.Chrom_SOX2_statusHigh
assignmentsChromaffin_cells.Chrom_SOX2_statusHigh.GTF2H5	1.98253295	3.82200137	6.30238568	1.90E-08	4.15E-07	9.11232559	GTF2H5	assignmentsChromaffin_cells.Chrom_SOX2_statusHigh
assignmentsChromaffin_cells.Chrom_SOX2_statusHigh.SDF2L1	3.0463744	3.37170421	6.30064896	1.93E-08	4.18E-07	9.1085168	SDF2L1	assignmentsChromaffin_cells.Chrom_SOX2_statusHigh
assignmentsChromaffin_cells.Chrom_SOX2_statusHigh.WDR45	1.54139445	3.88758957	6.29908338	1.93E-08	4.18E-07	9.10477629	WDR45	assignmentsChromaffin_cells.Chrom_SOX2_statusHigh
assignmentsChromaffin_cells.Chrom_SOX2_statusHigh.VILL	3.37555219	1.71569982	6.28934298	2.01E-08	4.33E-07	9.09895884	VILL	assignmentsChromaffin_cells.Chrom_SOX2_statusHigh
assignmentsChromaffin_cells.Chrom_SOX2_statusHigh.CNPY3	1.81441392	4.24218326	6.2977095	1.94E-08	4.19E-07	9.09775411	CNPY3	assignmentsChromaffin_cells.Chrom_SOX2_statusHigh
assignmentsChromaffin_cells.Chrom_SOX2_statusHigh.SNRPA	1.58707013	4.44274057	6.29951512	1.93E-08	4.18E-07	9.09553463	SNRPA	assignmentsChromaffin_cells.Chrom_SOX2_statusHigh
assignmentsChromaffin_cells.Chrom_SOX2_statusHigh.ATP6V0E1	1.88866561	6.6857471	6.31847171	1.78E-08	3.93E-07	9.09353136	ATP6V0E1	assignmentsChromaffin_cells.Chrom_SOX2_statusHigh
assignmentsChromaffin_cells.Chrom_SOX2_statusHigh.C1orf53	3.30446533	1.23514132	6.28829698	2.03E-08	4.35E-07	9.09033344	C1orf53	assignmentsChromaffin_cells.Chrom_SOX2_statusHigh
assignmentsChromaffin_cells.Chrom_SOX2_statusHigh.WNT11	3.87148617	0.04126991	6.31856854	1.78E-08	3.93E-07	9.08913982	WNT11	assignmentsChromaffin_cells.Chrom_SOX2_statusHigh
assignmentsChromaffin_cells.Chrom_SOX2_statusHigh.FAM131C	3.42277858	0.78261838	6.2831011	2.07E-08	4.40E-07	9.08737159	FAM131C	assignmentsChromaffin_cells.Chrom_SOX2_statusHigh
assignmentsChromaffin_cells.Chrom_SOX2_statusHigh.PIGM	1.29634554	3.41277958	6.28707573	2.03E-08	4.35E-07	9.06443959	PIGM	assignmentsChromaffin_cells.Chrom_SOX2_statusHigh
assignmentsChromaffin_cells.Chrom_SOX2_statusHigh.MTA3	-1.3010267	6.19567772	-6.3011515	1.91E-08	4.16E-07	9.060584	MTA3	assignmentsChromaffin_cells.Chrom_SOX2_statusHigh
assignmentsChromaffin_cells.Chrom_SOX2_statusHigh.DNAJC30	1.52054108	2.71417891	6.2847162	2.05E-08	4.37E-07	9.05873173	DNAJC30	assignmentsChromaffin_cells.Chrom_SOX2_statusHigh
assignmentsChromaffin_cells.Chrom_SOX2_statusHigh.ATP9B	-0.8755727	7.78561115	-6.3091503	1.85E-08	4.06E-07	9.05717446	ATP9B	assignmentsChromaffin_cells.Chrom_SOX2_statusHigh
assignmentsChromaffin_cells.Chrom_SOX2_statusHigh.PSMD2	1.61760424	5.00413275	6.29621229	1.95E-08	4.21E-07	9.05574057	PSMD2	assignmentsChromaffin_cells.Chrom_SOX2_statusHigh
assignmentsChromaffin_cells.Chrom_SOX2_statusHigh.EIF3K	2.0806889	6.28131378	6.30753112	1.87E-08	4.06E-07	9.05526262	EIF3K	assignmentsChromaffin_cells.Chrom_SOX2_statusHigh
assignmentsChromaffin_cells.Chrom_SOX2_statusHigh.SRM	2.34317072	3.34233554	6.28218382	2.07E-08	4.40E-07	9.05382683	SRM	assignmentsChromaffin_cells.Chrom_SOX2_statusHigh
assignmentsChromaffin_cells.Chrom_SOX2_statusHigh.PDAP1	1.40452115	5.15250188	6.29764916	1.94E-08	4.19E-07	9.05214402	PDAP1	assignmentsChromaffin_cells.Chrom_SOX2_statusHigh
assignmentsChromaffin_cells.Chrom_SOX2_statusHigh.MRPL24	1.87823202	3.60932991	6.28657812	2.04E-08	4.35E-07	9.0498749	MRPL24	assignmentsChromaffin_cells.Chrom_SOX2_statusHigh
assignmentsChromaffin_cells.Chrom_SOX2_statusHigh.ZNF669	1.41877477	2.26504796	6.2667496	2.21E-08	4.63E-07	9.01439347	ZNF669	assignmentsChromaffin_cells.Chrom_SOX2_statusHigh
assignmentsChromaffin_cells.Chrom_SOX2_statusHigh.CCT5	1.89217003	5.46598675	6.28995088	2.01E-08	4.32E-07	9.01397226	CCT5	assignmentsChromaffin_cells.Chrom_SOX2_statusHigh
assignmentsChromaffin_cells.Chrom_SOX2_statusHigh.NAT14	1.95531074	2.65942507	6.27460607	2.14E-08	4.51E-07	9.00873386	NAT14	assignmentsChromaffin_cells.Chrom_SOX2_statusHigh
assignmentsChromaffin_cells.Chrom_SOX2_statusHigh.TAF9	1.49935636	4.18180755	6.28073571	2.09E-08	4.41E-07	9.0067356	TAF9	assignmentsChromaffin_cells.Chrom_SOX2_statusHigh
assignmentsChromaffin_cells.Chrom_SOX2_statusHigh.ATP6V0E2	1.70688608	3.75507435	6.28177579	2.08E-08	4.40E-07	9.00352098	ATP6V0E2	assignmentsChromaffin_cells.Chrom_SOX2_statusHigh
assignmentsChromaffin_cells.Chrom_SOX2_statusHigh.APMAP	1.35095343	5.3666812	6.28128456	2.08E-08	4.40E-07	8.99480438	APMAP	assignmentsChromaffin_cells.Chrom_SOX2_statusHigh
assignmentsChromaffin_cells.Chrom_SOX2_statusHigh.MTMR3	-1.0300007	6.62945461	-6.2826932	2.07E-08	4.40E-07	8.99194604	MTMR3	assignmentsChromaffin_cells.Chrom_SOX2_statusHigh
assignmentsChromaffin_cells.Chrom_SOX2_statusHigh.HMBOX1	-0.9401805	7.45085386	-6.2864377	2.04E-08	4.35E-07	8.97693134	HMBOX1	assignmentsChromaffin_cells.Chrom_SOX2_statusHigh
assignmentsChromaffin_cells.Chrom_SOX2_statusHigh.JOSD2	2.39457786	2.98323293	6.26389573	2.24E-08	4.67E-07	8.9749011	JOSD2	assignmentsChromaffin_cells.Chrom_SOX2_statusHigh
assignmentsChromaffin_cells.Chrom_SOX2_statusHigh.RPP25L	2.46793935	1.62685524	6.25253845	2.35E-08	4.86E-07	8.97103289	RPP25L	assignmentsChromaffin_cells.Chrom_SOX2_statusHigh
assignmentsChromaffin_cells.Chrom_SOX2_statusHigh.FAM174A	1.56917351	4.82189728	6.27548883	2.13E-08	4.50E-07	8.96810937	FAM174A	assignmentsChromaffin_cells.Chrom_SOX2_statusHigh
assignmentsChromaffin_cells.Chrom_SOX2_statusHigh.RBM6	-1.2540288	8.31787002	-6.2886825	2.02E-08	4.33E-07	8.96524434	RBM6	assignmentsChromaffin_cells.Chrom_SOX2_statusHigh
assignmentsChromaffin_cells.Chrom_SOX2_statusHigh.JPT1	2.08500056	4.26514479	6.2662344	2.22E-08	4.64E-07	8.96461946	JPT1	assignmentsChromaffin_cells.Chrom_SOX2_statusHigh
assignmentsChromaffin_cells.Chrom_SOX2_statusHigh.GPS1	1.53416838	3.41209881	6.26305344	2.25E-08	4.68E-07	8.96302308	GPS1	assignmentsChromaffin_cells.Chrom_SOX2_statusHigh
assignmentsChromaffin_cells.Chrom_SOX2_statusHigh.PNMA1	2.06691792	3.50331273	6.26664903	2.21E-08	4.63E-07	8.96001268	PNMA1	assignmentsChromaffin_cells.Chrom_SOX2_statusHigh
assignmentsChromaffin_cells.Chrom_SOX2_statusHigh.CARMIL1	-4.1456829	6.0185026	-6.2581163	2.31E-08	4.80E-07	8.95155763	CARMIL1	assignmentsChromaffin_cells.Chrom_SOX2_statusHigh

assignmentsChromaffin_cells.Chrom_SOX2_statusHigh.ALDOC	3.33581568	2.02963907	6.25438249	2.34E-08	4.86E-07	8.94889266	ALDOC	assignmentsChromaffin_cells.Chrom_SOX2_statusHigh
assignmentsChromaffin_cells.Chrom_SOX2_statusHigh.SNRPG	1.8573689	5.17933047	6.27008858	2.18E-08	4.58E-07	8.94297326	SNRPG	assignmentsChromaffin_cells.Chrom_SOX2_statusHigh
assignmentsChromaffin_cells.Chrom_SOX2_statusHigh.PSAP	2.23889558	7.80607807	6.28696296	2.04E-08	4.35E-07	8.93157418	PSAP	assignmentsChromaffin_cells.Chrom_SOX2_statusHigh
assignmentsChromaffin_cells.Chrom_SOX2_statusHigh.PEX16	1.76435673	3.13097214	6.24970576	2.38E-08	4.90E-07	8.92925093	PEX16	assignmentsChromaffin_cells.Chrom_SOX2_statusHigh
assignmentsChromaffin_cells.Chrom_SOX2_statusHigh.NAP1L5	2.1242337	3.18243127	6.25599522	2.31E-08	4.80E-07	8.9240657	NAP1L5	assignmentsChromaffin_cells.Chrom_SOX2_statusHigh
assignmentsChromaffin_cells.Chrom_SOX2_statusHigh.BRWD3	-1.1607377	6.84795611	-6.2667148	2.21E-08	4.63E-07	8.90753701	BRWD3	assignmentsChromaffin_cells.Chrom_SOX2_statusHigh
assignmentsChromaffin_cells.Chrom_SOX2_statusHigh.ARMH3	-1.2489293	6.8185071	-6.2628683	2.25E-08	4.68E-07	8.9041692	ARMH3	assignmentsChromaffin_cells.Chrom_SOX2_statusHigh
assignmentsChromaffin_cells.Chrom_SOX2_statusHigh.NIP7	1.75814287	2.67821176	6.23988762	2.48E-08	5.09E-07	8.90370792	NIP7	assignmentsChromaffin_cells.Chrom_SOX2_statusHigh
assignmentsChromaffin_cells.Chrom_SOX2_statusHigh.RPL23A	3.5632328	6.7845066	6.27644395	2.14E-08	4.51E-07	8.89752667	RPL23A	assignmentsChromaffin_cells.Chrom_SOX2_statusHigh
assignmentsChromaffin_cells.Chrom_SOX2_statusHigh.ISOC2	2.27386581	3.00731582	6.24003309	2.47E-08	5.09E-07	8.87150975	ISOC2	assignmentsChromaffin_cells.Chrom_SOX2_statusHigh
assignmentsChromaffin_cells.Chrom_SOX2_statusHigh.PPP6R3	-1.1131795	8.19567011	-6.2654438	2.22E-08	4.64E-07	8.87114207	PPP6R3	assignmentsChromaffin_cells.Chrom_SOX2_statusHigh
assignmentsChromaffin_cells.Chrom_SOX2_statusHigh.COX17	2.6866294	5.34193359	6.25214624	2.37E-08	4.89E-07	8.84993986	COX17	assignmentsChromaffin_cells.Chrom_SOX2_statusHigh
assignmentsChromaffin_cells.Chrom_SOX2_statusHigh.NDUFC1	2.35909027	4.7437269	6.23833596	2.50E-08	5.13E-07	8.8157884	NDUFC1	assignmentsChromaffin_cells.Chrom_SOX2_statusHigh
assignmentsChromaffin_cells.Chrom_SOX2_statusHigh.FICD	1.83125481	2.53588579	6.22114515	2.68E-08	5.44E-07	8.80667365	FICD	assignmentsChromaffin_cells.Chrom_SOX2_statusHigh
assignmentsChromaffin_cells.Chrom_SOX2_statusHigh.NAT16	3.90621499	-0.4938897	6.22302182	2.66E-08	5.41E-07	8.80429271	NAT16	assignmentsChromaffin_cells.Chrom_SOX2_statusHigh
assignmentsChromaffin_cells.Chrom_SOX2_statusHigh.ZNF770	1.22604775	4.39140957	6.23016702	2.58E-08	5.26E-07	8.80292802	ZNF770	assignmentsChromaffin_cells.Chrom_SOX2_statusHigh
assignmentsChromaffin_cells.Chrom_SOX2_statusHigh.MRPL15	1.74120064	3.37447893	6.22384246	2.65E-08	5.39E-07	8.80036057	MRPL15	assignmentsChromaffin_cells.Chrom_SOX2_statusHigh
assignmentsChromaffin_cells.Chrom_SOX2_statusHigh.ARL2	2.46631797	4.35643249	6.23070373	2.58E-08	5.26E-07	8.79133657	ARL2	assignmentsChromaffin_cells.Chrom_SOX2_statusHigh
assignmentsChromaffin_cells.Chrom_SOX2_statusHigh.FABP6	5.36288592	-0.0105205	6.21995187	2.71E-08	5.51E-07	8.7858348	FABP6	assignmentsChromaffin_cells.Chrom_SOX2_statusHigh
assignmentsChromaffin_cells.Chrom_SOX2_statusHigh.UPF2	-0.8950958	6.88194475	-6.2355545	2.52E-08	5.17E-07	8.78468581	UPF2	assignmentsChromaffin_cells.Chrom_SOX2_statusHigh
assignmentsChromaffin_cells.Chrom_SOX2_statusHigh.CUTA	2.73168585	4.88957744	6.23471736	2.55E-08	5.21E-07	8.7803719	CUTA	assignmentsChromaffin_cells.Chrom_SOX2_statusHigh
assignmentsChromaffin_cells.Chrom_SOX2_statusHigh.KMT2C	-0.9569565	8.99318612	-6.2408974	2.46E-08	5.08E-07	8.73980637	KMT2C	assignmentsChromaffin_cells.Chrom_SOX2_statusHigh
assignmentsChromaffin_cells.Chrom_SOX2_statusHigh.LCORL	-1.0640631	7.80376799	-6.2300572	2.58E-08	5.26E-07	8.72041978	LCORL	assignmentsChromaffin_cells.Chrom_SOX2_statusHigh
assignmentsChromaffin_cells.Chrom_SOX2_statusHigh.SLC25A11	2.09355033	3.36388756	6.19854425	2.94E-08	5.94E-07	8.71156602	SLC25A11	assignmentsChromaffin_cells.Chrom_SOX2_statusHigh
assignmentsChromaffin_cells.Chrom_SOX2_statusHigh.LHX9	4.6725631	0.3651616	6.1883399	3.10E-08	6.22E-07	8.71108235	LHX9	assignmentsChromaffin_cells.Chrom_SOX2_statusHigh
assignmentsChromaffin_cells.Chrom_SOX2_statusHigh.AMPH	-4.8047798	3.07814177	-6.1854061	3.13E-08	6.28E-07	8.69738142	AMPH	assignmentsChromaffin_cells.Chrom_SOX2_statusHigh
assignmentsChromaffin_cells.Chrom_SOX2_statusHigh.SLC25A6	2.38882671	6.17992915	6.21616693	2.74E-08	5.56E-07	8.68230978	SLC25A6	assignmentsChromaffin_cells.Chrom_SOX2_statusHigh
assignmentsChromaffin_cells.Chrom_SOX2_statusHigh.PDLIM4	1.89906944	4.02584126	6.20136623	2.91E-08	5.88E-07	8.67296655	PDLIM4	assignmentsChromaffin_cells.Chrom_SOX2_statusHigh
assignmentsChromaffin_cells.Chrom_SOX2_statusHigh.MRPL11	2.0725828	2.87749073	6.18437057	3.12E-08	6.27E-07	8.66272988	MRPL11	assignmentsChromaffin_cells.Chrom_SOX2_statusHigh
assignmentsChromaffin_cells.Chrom_SOX2_statusHigh.SRSF9	1.93821959	5.27631675	6.20069644	2.92E-08	5.89E-07	8.65519411	SRSF9	assignmentsChromaffin_cells.Chrom_SOX2_statusHigh
assignmentsChromaffin_cells.Chrom_SOX2_statusHigh.METTL18	2.24694258	1.25623356	6.17232904	3.28E-08	6.48E-07	8.65076717	METTL18	assignmentsChromaffin_cells.Chrom_SOX2_statusHigh
assignmentsChromaffin_cells.Chrom_SOX2_statusHigh.SCN1B	2.56141907	2.21313775	6.16928249	3.33E-08	6.53E-07	8.63798829	SCN1B	assignmentsChromaffin_cells.Chrom_SOX2_statusHigh
assignmentsChromaffin_cells.Chrom_SOX2_statusHigh.NUP188	-1.3354282	4.74415899	-6.1787668	3.20E-08	6.35E-07	8.63297985	NUP188	assignmentsChromaffin_cells.Chrom_SOX2_statusHigh
assignmentsChromaffin_cells.Chrom_SOX2_statusHigh.HCFC1R1	2.01695254	4.32360446	6.18812481	3.07E-08	6.19E-07	8.61976914	HCFC1R1	assignmentsChromaffin_cells.Chrom_SOX2_statusHigh
assignmentsChromaffin_cells.Chrom_SOX2_statusHigh.COMMD6	2.69257461	5.00910658	6.19385225	3.02E-08	6.08E-07	8.61513453	COMMD6	assignmentsChromaffin_cells.Chrom_SOX2_statusHigh
assignmentsChromaffin_cells.Chrom_SOX2_statusHigh.SARAF	2.06212783	7.72693214	6.20957581	2.82E-08	5.70E-07	8.61210606	SARAF	assignmentsChromaffin_cells.Chrom_SOX2_statusHigh
assignmentsChromaffin_cells.Chrom_SOX2_statusHigh.PHC1	-1.2237492	4.81870459	-6.1778435	3.21E-08	6.36E-07	8.60928771	PHC1	assignmentsChromaffin_cells.Chrom_SOX2_statusHigh
assignmentsChromaffin_cells.Chrom_SOX2_statusHigh.C1orf122	2.8651842	3.48536941	6.1767761	3.23E-08	6.40E-07	8.59821908	C1orf122	assignmentsChromaffin_cells.Chrom_SOX2_statusHigh
assignmentsChromaffin_cells.Chrom_SOX2_statusHigh.PBXIP1	1.66809178	4.53126346	6.17607318	3.23E-08	6.40E-07	8.58930266	PBXIP1	assignmentsChromaffin_cells.Chrom_SOX2_statusHigh
assignmentsChromaffin_cells.Chrom_SOX2_statusHigh.JADE3	-1.6728829	4.56157524	-6.1695558	3.32E-08	6.53E-07	8.58890786	JADE3	assignmentsChromaffin_cells.Chrom_SOX2_statusHigh
assignmentsChromaffin_cells.Chrom_SOX2_statusHigh.INHBE	3.95271354	-0.2218255	6.16977069	3.32E-08	6.53E-07	8.58503038	INHBE	assignmentsChromaffin_cells.Chrom_SOX2_statusHigh
assignmentsChromaffin_cells.Chrom_SOX2_statusHigh.TMEM167B	1.55522347	3.57907759	6.16562029	3.38E-08	6.61E-07	8.58365438	TMEM167B	assignmentsChromaffin_cells.Chrom_SOX2_statusHigh
assignmentsChromaffin_cells.Chrom_SOX2_statusHigh.ZCRB1	1.60454168	4.57318829	6.18029807	3.18E-08	6.34E-07	8.5823171	ZCRB1	assignmentsChromaffin_cells.Chrom_SOX2_statusHigh
assignmentsChromaffin_cells.Chrom_SOX2_statusHigh.POLR3GL	1.8139357	4.14378141	6.17112925	3.30E-08	6.51E-07	8.57432081	POLR3GL	assignmentsChromaffin_cells.Chrom_SOX2_statusHigh
assignmentsChromaffin_cells.Chrom_SOX2_statusHigh.MRPL18	2.56860238	4.14957962	6.17093986	3.31E-08	6.52E-07	8.56658319	MRPL18	assignmentsChromaffin_cells.Chrom_SOX2_statusHigh
assignmentsChromaffin_cells.Chrom_SOX2_statusHigh.ASB8	1.24077166	4.16570438	6.16721677	3.35E-08	6.58E-07	8.54963725	ASB8	assignmentsChromaffin_cells.Chrom_SOX2_statusHigh
assignmentsChromaffin_cells.Chrom_SOX2_statusHigh.PSMA7	2.28342286	6.09556853	6.17948015	3.19E-08	6.35E-07	8.54878477	PSMA7	assignmentsChromaffin_cells.Chrom_SOX2_statusHigh
assignmentsChromaffin_cells.Chrom_SOX2_statusHigh.DUSP1	3.83798178	7.46979218	6.18353357	3.16E-08	6.32E-07	8.54697822	DUSP1	assignmentsChromaffin_cells.Chrom_SOX2_statusHigh
assignmentsChromaffin_cells.Chrom_SOX2_statusHigh.CRELD2	1.81172613	3.96300802	6.16426686	3.40E-08	6.63E-07	8.53777341	CRELD2	assignmentsChromaffin_cells.Chrom_SOX2_statusHigh
assignmentsChromaffin_cells.Chrom_SOX2_statusHigh.HPS6	2.42208543	1.06864164	6.14211134	3.72E-08	7.18E-07	8.53539432	HPS6	assignmentsChromaffin_cells.Chrom_SOX2_statusHigh
assignmentsChromaffin_cells.Chrom_SOX2_statusHigh.MRPL33	1.6695132	6.45917655	6.18347218	3.13E-08	6.28E-07	8.53248296	MRPL33	assignmentsChromaffin_cells.Chrom_SOX2_statusHigh
assignmentsChromaffin_cells.Chrom_SOX2_statusHigh.BOLA3	2.33224297	3.36937904	6.15854713	3.48E-08	6.76E-07	8.53027269	BOLA3	assignmentsChromaffin_cells.Chrom_SOX2_statusHigh
assignmentsChromaffin_cells.Chrom_SOX2_statusHigh.RC3H1	-0.8789639	7.64450676	-6.1795822	3.19E-08	6.35E-07	8.5278941	RC3H1	assignmentsChromaffin_cells.Chrom_SOX2_statusHigh
assignmentsChromaffin_cells.Chrom_SOX2_statusHigh.TNRC6A	-1.2105998	8.2674299	-6.1799422	3.18E-08	6.34E-07	8.50932782	TNRC6A	assignmentsChromaffin_cells.Chrom_SOX2_statusHigh
assignmentsChromaffin_cells.Chrom_SOX2_statusHigh.TCEAL4	1.62846444	5.63271523	6.16531733	3.38E-08	6.61E-07	8.49483113	TCEAL4	assignmentsChromaffin_cells.Chrom_SOX2_statusHigh
assignmentsChromaffin_cells.Chrom_SOX2_statusHigh.ANKRD17	-0.9239415	8.7443614	-6.1783484	3.20E-08	6.36E-07	8.49137202	ANKRD17	assignmentsChromaffin_cells.Chrom_SOX2_statusHigh
assignmentsChromaffin_cells.Chrom_SOX2_statusHigh.LRRC24	2.08880184	1.63592654	6.13996643	3.76E-08	7.23E-07	8.49101636	LRRC24	assignmentsChromaffin_cells.Chrom_SOX2_statusHigh
assignmentsChromaffin_cells.Chrom_SOX2_statusHigh.GTF2F1	1.53992456	4.49865685	6.15181951	3.58E-08	6.94E-07	8.48287896	GTF2F1	assignmentsChromaffin_cells.Chrom_SOX2_statusHigh
assignmentsChromaffin_cells.Chrom_SOX2_statusHigh.ZMYND11	-0.9570082	7.1268464	-6.1631504	3.41E-08	6.66E-07	8.48069016	ZMYND11	assignmentsChromaffin_cells.Chrom_SOX2_statusHigh
assignmentsChromaffin_cells.Chrom_SOX2_statusHigh.SNU13	2.09377604	5.69762119	6.15993345	3.46E-08	6.73E-07	8.47608632	SNU13	assignmentsChromaffin_cells.Chrom_SOX2_statusHigh
assignmentsChromaffin_cells.Chrom_SOX2_statusHigh.RBM33	-1.0185283	7.29522579	-6.1615866	3.43E-08	6.69E-07	8.47259286	RBM33	assignmentsChromaffin_cells.Chrom_SOX2_statusHigh
assignmentsChromaffin_cells.Chrom_SOX2_statusHigh.PRAF2	1.81908505	3.27005764	6.14028344	3.75E-08	7.23E-07	8.46853118	PRAF2	assignmentsChromaffin_cells.Chrom_SOX2_statusHigh
assignmentsChromaffin_cells.Chrom_SOX2_statusHigh.CD151	2.46520063	4.54786408	6.14622651	3.67E-08	7.09E-07	8.46335043	CD151	assignmentsChromaffin_cells.Chrom_SOX2_statusHigh
assignmentsChromaffin_cells.Chrom_SOX2_statusHigh.ARL1	1.81095061	4.78358591	6.14923446	3.62E-08	7.00E-07	8.4479645	ARL1	assignmentsChromaffin_cells.Chrom_SOX2_statusHigh

assignmentsChromaffin_cells.Chrom_SOX2_statusHigh.ZNHIT3	1.69955945	3.54798185	6.13017182	3.91E-08	7.48E-07	8.42417306	ZNHIT3	assignmentsChromaffin_cells.Chrom_SOX2_statusHigh
assignmentsChromaffin_cells.Chrom_SOX2_statusHigh.CD164	1.73801079	6.37771288	6.14804186	3.63E-08	7.03E-07	8.41706581	CD164	assignmentsChromaffin_cells.Chrom_SOX2_statusHigh
assignmentsChromaffin_cells.Chrom_SOX2_statusHigh.PRKRIP1	-1.1458999	6.69094735	-6.14344	3.70E-08	7.15E-07	8.40728866	PRKRIP1	assignmentsChromaffin_cells.Chrom_SOX2_statusHigh
assignmentsChromaffin_cells.Chrom_SOX2_statusHigh.VPS28	2.52944321	5.14133295	6.13917477	3.78E-08	7.25E-07	8.40551904	VPS28	assignmentsChromaffin_cells.Chrom_SOX2_statusHigh
assignmentsChromaffin_cells.Chrom_SOX2_statusHigh.CDYL	-1.7050303	7.01062095	-6.1388509	3.78E-08	7.24E-07	8.40426073	CDYL	assignmentsChromaffin_cells.Chrom_SOX2_statusHigh
assignmentsChromaffin_cells.Chrom_SOX2_statusHigh.ALDH9A1	1.26849906	4.42088731	6.12834888	3.94E-08	7.52E-07	8.40318942	ALDH9A1	assignmentsChromaffin_cells.Chrom_SOX2_statusHigh
assignmentsChromaffin_cells.Chrom_SOX2_statusHigh.PGP	1.1614257	4.57429931	6.12872526	3.94E-08	7.52E-07	8.39907808	PGP	assignmentsChromaffin_cells.Chrom_SOX2_statusHigh
assignmentsChromaffin_cells.Chrom_SOX2_statusHigh.PFDN4	1.43016525	3.74310627	6.12525611	4.00E-08	7.61E-07	8.39681966	PFDN4	assignmentsChromaffin_cells.Chrom_SOX2_statusHigh
assignmentsChromaffin_cells.Chrom_SOX2_statusHigh.LRRC14	1.5446731	2.72893523	6.11392353	4.19E-08	7.95E-07	8.39244217	LRRC14	assignmentsChromaffin_cells.Chrom_SOX2_statusHigh
assignmentsChromaffin_cells.Chrom_SOX2_statusHigh.BORCS6	2.46146659	1.51140947	6.10609244	4.33E-08	8.15E-07	8.39148881	BORCS6	assignmentsChromaffin_cells.Chrom_SOX2_statusHigh
assignmentsChromaffin_cells.Chrom_SOX2_statusHigh.RPL28	3.51218138	7.52600911	6.15344753	3.58E-08	6.95E-07	8.38014167	RPL28	assignmentsChromaffin_cells.Chrom_SOX2_statusHigh
assignmentsChromaffin_cells.Chrom_SOX2_statusHigh.RPS21	3.76451009	6.31028851	6.14161978	3.76E-08	7.23E-07	8.36615098	RPS21	assignmentsChromaffin_cells.Chrom_SOX2_statusHigh
assignmentsChromaffin_cells.Chrom_SOX2_statusHigh.YKT6	1.14802594	3.99085521	6.11619369	4.15E-08	7.89E-07	8.36204508	YKT6	assignmentsChromaffin_cells.Chrom_SOX2_statusHigh
assignmentsChromaffin_cells.Chrom_SOX2_statusHigh.TMEM107	1.84327756	3.17234319	6.10766685	4.30E-08	8.11E-07	8.36040355	TMEM107	assignmentsChromaffin_cells.Chrom_SOX2_statusHigh
assignmentsChromaffin_cells.Chrom_SOX2_statusHigh.MRPL52	1.82290282	4.17362934	6.1141645	4.18E-08	7.95E-07	8.35133584	MRPL52	assignmentsChromaffin_cells.Chrom_SOX2_statusHigh
assignmentsChromaffin_cells.Chrom_SOX2_statusHigh.ATP5MC2	2.11507338	6.38929008	6.13383741	3.86E-08	7.40E-07	8.34009579	ATP5MC2	assignmentsChromaffin_cells.Chrom_SOX2_statusHigh
assignmentsChromaffin_cells.Chrom_SOX2_statusHigh.CDKN2D	3.0817285	2.38940198	6.09755802	4.50E-08	8.44E-07	8.32872173	CDKN2D	assignmentsChromaffin_cells.Chrom_SOX2_statusHigh
assignmentsChromaffin_cells.Chrom_SOX2_statusHigh.PERP	2.39353623	3.63484059	6.11016301	4.26E-08	8.06E-07	8.3122373	PERP	assignmentsChromaffin_cells.Chrom_SOX2_statusHigh
assignmentsChromaffin_cells.Chrom_SOX2_statusHigh.PCYOX1	1.93813106	4.10293175	6.10528301	4.34E-08	8.17E-07	8.29180128	PCYOX1	assignmentsChromaffin_cells.Chrom_SOX2_statusHigh
assignmentsChromaffin_cells.Chrom_SOX2_statusHigh.TUBB3	1.96361614	4.78326587	6.11238935	4.22E-08	7.99E-07	8.28118198	TUBB3	assignmentsChromaffin_cells.Chrom_SOX2_statusHigh
assignmentsChromaffin_cells.Chrom_SOX2_statusHigh.DCTN6	1.86441394	3.98746136	6.09854936	4.46E-08	8.40E-07	8.27982541	DCTN6	assignmentsChromaffin_cells.Chrom_SOX2_statusHigh
assignmentsChromaffin_cells.Chrom_SOX2_statusHigh.SLAIN2	-1.0169711	6.74543521	-6.1098429	4.26E-08	8.06E-07	8.27897922	SLAIN2	assignmentsChromaffin_cells.Chrom_SOX2_statusHigh
assignmentsChromaffin_cells.Chrom_SOX2_statusHigh.POLE3	1.66230889	3.21239922	6.08315475	4.76E-08	8.86E-07	8.25898273	POLE3	assignmentsChromaffin_cells.Chrom_SOX2_statusHigh
assignmentsChromaffin_cells.Chrom_SOX2_statusHigh.NSMCE3	2.51021566	3.57618666	6.09377211	4.57E-08	8.52E-07	8.25559883	NSMCE3	assignmentsChromaffin_cells.Chrom_SOX2_statusHigh
assignmentsChromaffin_cells.Chrom_SOX2_statusHigh.MRPL13	1.28235517	4.79665261	6.09585209	4.52E-08	8.46E-07	8.24933364	MRPL13	assignmentsChromaffin_cells.Chrom_SOX2_statusHigh
assignmentsChromaffin_cells.Chrom_SOX2_statusHigh.CA5B	-1.4249496	5.50861894	-6.0878975	4.67E-08	8.69E-07	8.24266007	CA5B	assignmentsChromaffin_cells.Chrom_SOX2_statusHigh
assignmentsChromaffin_cells.Chrom_SOX2_statusHigh.EPG5	-0.9268377	6.16010132	-6.0935944	4.56E-08	8.52E-07	8.22658518	EPG5	assignmentsChromaffin_cells.Chrom_SOX2_statusHigh
assignmentsChromaffin_cells.Chrom_SOX2_statusHigh.PDSS2	-1.0745444	6.79002513	-6.0947731	4.54E-08	8.49E-07	8.22294523	PDSS2	assignmentsChromaffin_cells.Chrom_SOX2_statusHigh
assignmentsChromaffin_cells.Chrom_SOX2_statusHigh.RPS27	3.69461287	7.0778922	6.11118728	4.27E-08	8.07E-07	8.21883866	RPS27	assignmentsChromaffin_cells.Chrom_SOX2_statusHigh
assignmentsChromaffin_cells.Chrom_SOX2_statusHigh.HEIH	2.98831668	2.35447556	6.06426596	5.16E-08	9.49E-07	8.21799276	HEIH	assignmentsChromaffin_cells.Chrom_SOX2_statusHigh
assignmentsChromaffin_cells.Chrom_SOX2_statusHigh.CCDC115	2.16467016	2.71265958	6.07129757	5.00E-08	9.26E-07	8.21108574	CCDC115	assignmentsChromaffin_cells.Chrom_SOX2_statusHigh
assignmentsChromaffin_cells.Chrom_SOX2_statusHigh.SNX13	-0.9220656	7.29477669	-6.0966527	4.50E-08	8.44E-07	8.20265008	SNX13	assignmentsChromaffin_cells.Chrom_SOX2_statusHigh
assignmentsChromaffin_cells.Chrom_SOX2_statusHigh.NPC2	2.64594335	4.99816515	6.0824174	4.79E-08	8.90E-07	8.18990291	NPC2	assignmentsChromaffin_cells.Chrom_SOX2_statusHigh
assignmentsChromaffin_cells.Chrom_SOX2_statusHigh.SUMO3	2.39486945	3.28241243	6.06569608	5.12E-08	9.45E-07	8.18233324	SUMO3	assignmentsChromaffin_cells.Chrom_SOX2_statusHigh
assignmentsChromaffin_cells.Chrom_SOX2_statusHigh.FAAP20	1.84967519	4.02069667	6.07017327	5.02E-08	9.29E-07	8.17042362	FAAP20	assignmentsChromaffin_cells.Chrom_SOX2_statusHigh
assignmentsChromaffin_cells.Chrom_SOX2_statusHigh.MTFR2	2.49415732	0.52764386	6.05263871	5.40E-08	9.89E-07	8.16982477	MTFR2	assignmentsChromaffin_cells.Chrom_SOX2_statusHigh
assignmentsChromaffin_cells.Chrom_SOX2_statusHigh.BORCS8	1.55269188	3.46724197	6.06435092	5.15E-08	9.49E-07	8.1613193	BORCS8	assignmentsChromaffin_cells.Chrom_SOX2_statusHigh
assignmentsChromaffin_cells.Chrom_SOX2_statusHigh.CD40	3.8747191	2.32345922	6.06647883	5.10E-08	9.43E-07	8.14072647	CD40	assignmentsChromaffin_cells.Chrom_SOX2_statusHigh
assignmentsChromaffin_cells.Chrom_SOX2_statusHigh.AKT3	-1.1073825	9.33049425	-6.0927483	4.57E-08	8.53E-07	8.12159708	AKT3	assignmentsChromaffin_cells.Chrom_SOX2_statusHigh
assignmentsChromaffin_cells.Chrom_SOX2_statusHigh.PDIA3	1.8945116	7.17940865	6.07943395	4.83E-08	8.97E-07	8.10447364	PDIA3	assignmentsChromaffin_cells.Chrom_SOX2_statusHigh
assignmentsChromaffin_cells.Chrom_SOX2_statusHigh.ZNF638	-0.9832706	8.65304071	-6.0787987	4.85E-08	8.99E-07	8.09464775	ZNF638	assignmentsChromaffin_cells.Chrom_SOX2_statusHigh
assignmentsChromaffin_cells.Chrom_SOX2_statusHigh.LRRC59	1.63118049	3.82343757	6.04921165	5.48E-08	9.97E-07	8.09166282	LRRC59	assignmentsChromaffin_cells.Chrom_SOX2_statusHigh
assignmentsChromaffin_cells.Chrom_SOX2_statusHigh.SYF2	2.11994458	4.34145597	6.04910374	5.48E-08	9.97E-07	8.08429878	SYF2	assignmentsChromaffin_cells.Chrom_SOX2_statusHigh
assignmentsChromaffin_cells.Chrom_SOX2_statusHigh.TCEAL3	1.61698741	5.01108521	6.05989507	5.24E-08	9.62E-07	8.08200865	TCEAL3	assignmentsChromaffin_cells.Chrom_SOX2_statusHigh
assignmentsChromaffin_cells.Chrom_SOX2_statusHigh.MFSD5	2.31636894	1.90757665	6.03026134	5.93E-08	1.06E-06	8.07471755	MFSD5	assignmentsChromaffin_cells.Chrom_SOX2_statusHigh
assignmentsChromaffin_cells.Chrom_SOX2_statusHigh.COX7A2	2.86902159	5.72822781	6.06512549	5.17E-08	9.50E-07	8.07185885	COX7A2	assignmentsChromaffin_cells.Chrom_SOX2_statusHigh
assignmentsChromaffin_cells.Chrom_SOX2_statusHigh.SLC25A5	3.12792157	4.74993728	6.05565318	5.38E-08	9.86E-07	8.07047876	SLC25A5	assignmentsChromaffin_cells.Chrom_SOX2_statusHigh
assignmentsChromaffin_cells.Chrom_SOX2_statusHigh.ZNF827	-1.413188	5.80185041	-6.0514884	5.43E-08	9.92E-07	8.06531715	ZNF827	assignmentsChromaffin_cells.Chrom_SOX2_statusHigh
assignmentsChromaffin_cells.Chrom_SOX2_statusHigh.SHKBP1	1.83870146	3.4651301	6.03369974	5.85E-08	1.05E-06	8.06481007	SHKBP1	assignmentsChromaffin_cells.Chrom_SOX2_statusHigh
assignmentsChromaffin_cells.Chrom_SOX2_statusHigh.KXD1	1.33879381	4.12877663	6.04314583	5.62E-08	1.02E-06	8.06379451	KXD1	assignmentsChromaffin_cells.Chrom_SOX2_statusHigh
assignmentsChromaffin_cells.Chrom_SOX2_statusHigh.NDUFS5	2.70920963	5.17803711	6.05382199	5.41E-08	9.89E-07	8.06180684	NDUFS5	assignmentsChromaffin_cells.Chrom_SOX2_statusHigh
assignmentsChromaffin_cells.Chrom_SOX2_statusHigh.HOXD1	4.09045293	-0.1615169	6.03324927	5.87E-08	1.05E-06	8.06109925	HOXD1	assignmentsChromaffin_cells.Chrom_SOX2_statusHigh
assignmentsChromaffin_cells.Chrom_SOX2_statusHigh.CLU	4.29614808	5.5566908	6.06593122	5.15E-08	9.49E-07	8.05675203	CLU	assignmentsChromaffin_cells.Chrom_SOX2_statusHigh
assignmentsChromaffin_cells.Chrom_SOX2_statusHigh.DGCR6L	1.93870772	3.14307248	6.03385584	5.84E-08	1.05E-06	8.05054311	DGCR6L	assignmentsChromaffin_cells.Chrom_SOX2_statusHigh
assignmentsChromaffin_cells.Chrom_SOX2_statusHigh.ORAI3	1.72192088	2.84685194	6.0244198	6.07E-08	1.08E-06	8.0361096	ORAI3	assignmentsChromaffin_cells.Chrom_SOX2_statusHigh
assignmentsChromaffin_cells.Chrom_SOX2_statusHigh.MRPS21	1.38251266	5.88632128	6.05061098	5.45E-08	9.93E-07	8.02844688	MRPS21	assignmentsChromaffin_cells.Chrom_SOX2_statusHigh
assignmentsChromaffin_cells.Chrom_SOX2_statusHigh.SLC25A39	1.4486964	4.72358335	6.04067129	5.68E-08	1.03E-06	8.02103324	SLC25A39	assignmentsChromaffin_cells.Chrom_SOX2_statusHigh
assignmentsChromaffin_cells.Chrom_SOX2_statusHigh.SNAPC5	1.48063683	3.48916811	6.02583244	6.04E-08	1.08E-06	8.01041705	SNAPC5	assignmentsChromaffin_cells.Chrom_SOX2_statusHigh
assignmentsChromaffin_cells.Chrom_SOX2_statusHigh.COMMD8	1.97664612	2.80473312	6.02265295	6.12E-08	1.09E-06	8.00994455	COMMD8	assignmentsChromaffin_cells.Chrom_SOX2_statusHigh
assignmentsChromaffin_cells.Chrom_SOX2_statusHigh.PKD2	-1.3891873	6.61808338	-6.0371617	5.76E-08	1.04E-06	8.00984037	PKD2	assignmentsChromaffin_cells.Chrom_SOX2_statusHigh
assignmentsChromaffin_cells.Chrom_SOX2_statusHigh.AP2S1	2.39297913	4.56090981	6.03332139	5.87E-08	1.05E-06	8.00720966	AP2S1	assignmentsChromaffin_cells.Chrom_SOX2_statusHigh
assignmentsChromaffin_cells.Chrom_SOX2_statusHigh.NCOA6	-1.2677793	6.6306975	-6.0407036	5.68E-08	1.03E-06	8.00720324	NCOA6	assignmentsChromaffin_cells.Chrom_SOX2_statusHigh
assignmentsChromaffin_cells.Chrom_SOX2_statusHigh.LINC02636	-3.6608556	0.59709714	-6.0362122	5.78E-08	1.04E-06	8.00648213	LINC02636	assignmentsChromaffin_cells.Chrom_SOX2_statusHigh
assignmentsChromaffin_cells.Chrom_SOX2_statusHigh.DUSP23	4.11758499	1.92224988	6.00779685	6.53E-08	1.15E-06	7.99708739	DUSP23	assignmentsChromaffin_cells.Chrom_SOX2_statusHigh

assignmentsChromaffin_cells.Chrom_SOX2_statusHigh.NDUFB5	1.88664715	4.63687153	6.03196481	5.89E-08	1.06E-06	7.98856605	NDUFB5	assignmentsChromaffin_cells.Chrom_SOX2_statusHigh
assignmentsChromaffin_cells.Chrom_SOX2_statusHigh.NT5C3B	1.27716854	3.98659251	6.0262277	6.03E-08	1.08E-06	7.98001422	NT5C3B	assignmentsChromaffin_cells.Chrom_SOX2_statusHigh
assignmentsChromaffin_cells.Chrom_SOX2_statusHigh.ATP5F1D	2.58621646	5.74776598	6.04165267	5.69E-08	1.03E-06	7.97956669	ATP5F1D	assignmentsChromaffin_cells.Chrom_SOX2_statusHigh
assignmentsChromaffin_cells.Chrom_SOX2_statusHigh.RPS15	3.90658991	7.17482753	6.0525741	5.45E-08	9.93E-07	7.97644816	RPS15	assignmentsChromaffin_cells.Chrom_SOX2_statusHigh
assignmentsChromaffin_cells.Chrom_SOX2_statusHigh.XPO5	-1.1398857	5.1085511	-6.022141	6.13E-08	1.09E-06	7.97580922	XPO5	assignmentsChromaffin_cells.Chrom_SOX2_statusHigh
assignmentsChromaffin_cells.Chrom_SOX2_statusHigh.RACK1	2.75706059	7.00343742	6.0474811	5.56E-08	1.01E-06	7.9748342	RACK1	assignmentsChromaffin_cells.Chrom_SOX2_statusHigh
assignmentsChromaffin_cells.Chrom_SOX2_statusHigh.MYL6B	1.62123084	3.80915121	6.02089956	6.16E-08	1.09E-06	7.95792546	MYL6B	assignmentsChromaffin_cells.Chrom_SOX2_statusHigh
assignmentsChromaffin_cells.Chrom_SOX2_statusHigh.PHPT1	2.24857342	4.92348911	6.02777844	6.00E-08	1.07E-06	7.94825753	PHPT1	assignmentsChromaffin_cells.Chrom_SOX2_statusHigh
assignmentsChromaffin_cells.Chrom_SOX2_statusHigh.GRN	2.5101754	5.30638394	6.02421455	6.10E-08	1.09E-06	7.93195118	GRN	assignmentsChromaffin_cells.Chrom_SOX2_statusHigh
assignmentsChromaffin_cells.Chrom_SOX2_statusHigh.CSNK2B	1.98964276	5.04153414	6.0160764	6.29E-08	1.11E-06	7.92377599	CSNK2B	assignmentsChromaffin_cells.Chrom_SOX2_statusHigh
assignmentsChromaffin_cells.Chrom_SOX2_statusHigh.MRPL51	2.95713944	3.77109928	6.01165801	6.45E-08	1.14E-06	7.918925	MRPL51	assignmentsChromaffin_cells.Chrom_SOX2_statusHigh
assignmentsChromaffin_cells.Chrom_SOX2_statusHigh.PI4KA	-1.0312338	7.17654383	-6.0244941	6.07E-08	1.08E-06	7.91230063	PI4KA	assignmentsChromaffin_cells.Chrom_SOX2_statusHigh
assignmentsChromaffin_cells.Chrom_SOX2_statusHigh.OXLD1	1.95785117	3.37255555	5.99894563	6.75E-08	1.19E-06	7.90577423	OXLD1	assignmentsChromaffin_cells.Chrom_SOX2_statusHigh
assignmentsChromaffin_cells.Chrom_SOX2_statusHigh.COL4A6	4.39766833	1.23986669	5.98982284	7.06E-08	1.24E-06	7.90537896	COL4A6	assignmentsChromaffin_cells.Chrom_SOX2_statusHigh
assignmentsChromaffin_cells.Chrom_SOX2_statusHigh.STOX1	-2.2245807	3.13811884	-5.9891836	7.03E-08	1.23E-06	7.88995685	STOX1	assignmentsChromaffin_cells.Chrom_SOX2_statusHigh
assignmentsChromaffin_cells.Chrom_SOX2_statusHigh.PSMA2	1.83912713	5.07755927	6.00646967	6.54E-08	1.15E-06	7.87352121	PSMA2	assignmentsChromaffin_cells.Chrom_SOX2_statusHigh
assignmentsChromaffin_cells.Chrom_SOX2_statusHigh.CFL1	3.0135486	6.12531637	6.01314547	6.41E-08	1.14E-06	7.87084684	CFL1	assignmentsChromaffin_cells.Chrom_SOX2_statusHigh
assignmentsChromaffin_cells.Chrom_SOX2_statusHigh.CD2BP2	1.61522084	3.80119417	5.98762545	7.07E-08	1.24E-06	7.84957184	CD2BP2	assignmentsChromaffin_cells.Chrom_SOX2_statusHigh
assignmentsChromaffin_cells.Chrom_SOX2_statusHigh.URB1-AS1	2.07655763	1.3401702	5.96691001	7.71E-08	1.33E-06	7.8311823	URB1-AS1	assignmentsChromaffin_cells.Chrom_SOX2_statusHigh
assignmentsChromaffin_cells.Chrom_SOX2_statusHigh.TUBA1B	3.20678001	5.92786765	6.00297968	6.69E-08	1.18E-06	7.83057604	TUBA1B	assignmentsChromaffin_cells.Chrom_SOX2_statusHigh
assignmentsChromaffin_cells.Chrom_SOX2_statusHigh.POLR2G	2.13744611	3.4554615	5.97566011	7.43E-08	1.29E-06	7.82799136	POLR2G	assignmentsChromaffin_cells.Chrom_SOX2_statusHigh
assignmentsChromaffin_cells.Chrom_SOX2_statusHigh.PPP4C	1.40629539	4.70276111	5.9810939	7.27E-08	1.27E-06	7.80954801	PPP4C	assignmentsChromaffin_cells.Chrom_SOX2_statusHigh
assignmentsChromaffin_cells.Chrom_SOX2_statusHigh.SH3BGRL3	3.75380335	4.27756533	5.9752794	7.50E-08	1.30E-06	7.80893446	SH3BGRL3	assignmentsChromaffin_cells.Chrom_SOX2_statusHigh
assignmentsChromaffin_cells.Chrom_SOX2_statusHigh.FUCA1	1.9413677	3.25923176	5.96480096	7.77E-08	1.33E-06	7.79650249	FUCA1	assignmentsChromaffin_cells.Chrom_SOX2_statusHigh
assignmentsChromaffin_cells.Chrom_SOX2_statusHigh.CHFR	-1.7169771	6.05254755	-5.9787833	7.34E-08	1.28E-06	7.79410796	CHFR	assignmentsChromaffin_cells.Chrom_SOX2_statusHigh
assignmentsChromaffin_cells.Chrom_SOX2_statusHigh.EFNB1	2.46492056	1.63687969	5.95583231	8.07E-08	1.37E-06	7.78972097	EFNB1	assignmentsChromaffin_cells.Chrom_SOX2_statusHigh
assignmentsChromaffin_cells.Chrom_SOX2_statusHigh.ZNF830	1.62557127	3.66060744	5.97143095	7.56E-08	1.31E-06	7.78673938	ZNF830	assignmentsChromaffin_cells.Chrom_SOX2_statusHigh
assignmentsChromaffin_cells.Chrom_SOX2_statusHigh.ATXN3	-1.1158111	6.16191771	-5.9794643	7.32E-08	1.27E-06	7.78562831	ATXN3	assignmentsChromaffin_cells.Chrom_SOX2_statusHigh
assignmentsChromaffin_cells.Chrom_SOX2_statusHigh.SMIM20	2.5769351	3.09251382	5.95915901	7.96E-08	1.36E-06	7.78517377	SMIM20	assignmentsChromaffin_cells.Chrom_SOX2_statusHigh
assignmentsChromaffin_cells.Chrom_SOX2_statusHigh.MLLT11	1.76864663	4.65403956	5.98817381	7.06E-08	1.24E-06	7.78510254	MLLT11	assignmentsChromaffin_cells.Chrom_SOX2_statusHigh
assignmentsChromaffin_cells.Chrom_SOX2_statusHigh.ATP5PO	1.96751923	4.91057721	5.98198632	7.24E-08	1.27E-06	7.78208686	ATP5PO	assignmentsChromaffin_cells.Chrom_SOX2_statusHigh
assignmentsChromaffin_cells.Chrom_SOX2_statusHigh.CLTA	1.20866686	6.72109938	5.99230718	6.94E-08	1.22E-06	7.77697179	CLTA	assignmentsChromaffin_cells.Chrom_SOX2_statusHigh
assignmentsChromaffin_cells.Chrom_SOX2_statusHigh.RTRAF	1.67277721	5.11594764	5.97987226	7.31E-08	1.27E-06	7.77573723	RTRAF	assignmentsChromaffin_cells.Chrom_SOX2_statusHigh
assignmentsChromaffin_cells.Chrom_SOX2_statusHigh.PIH1D1	1.68041282	4.24938163	5.97158627	7.56E-08	1.31E-06	7.76203603	PIH1D1	assignmentsChromaffin_cells.Chrom_SOX2_statusHigh
assignmentsChromaffin_cells.Chrom_SOX2_statusHigh.AQP3	5.16560891	0.59250915	5.96376012	7.84E-08	1.34E-06	7.75411731	AQP3	assignmentsChromaffin_cells.Chrom_SOX2_statusHigh
assignmentsChromaffin_cells.Chrom_SOX2_statusHigh.ROM1	2.77421183	0.70782265	5.9416322	8.56E-08	1.44E-06	7.74658673	ROM1	assignmentsChromaffin_cells.Chrom_SOX2_statusHigh
assignmentsChromaffin_cells.Chrom_SOX2_statusHigh.LINC01315	3.67544067	0.73201881	5.94189913	8.57E-08	1.44E-06	7.74493022	LINC01315	assignmentsChromaffin_cells.Chrom_SOX2_statusHigh
assignmentsChromaffin_cells.Chrom_SOX2_statusHigh.HSPB1	6.00070779	5.17331051	5.95816496	8.05E-08	1.37E-06	7.73533879	HSPB1	assignmentsChromaffin_cells.Chrom_SOX2_statusHigh
assignmentsChromaffin_cells.Chrom_SOX2_statusHigh.PRDX3	1.70262394	4.54118633	5.96885231	7.65E-08	1.32E-06	7.73419957	PRDX3	assignmentsChromaffin_cells.Chrom_SOX2_statusHigh
assignmentsChromaffin_cells.Chrom_SOX2_statusHigh.YIF1B	2.16060868	3.466391	5.95520994	8.09E-08	1.37E-06	7.733007	YIF1B	assignmentsChromaffin_cells.Chrom_SOX2_statusHigh
assignmentsChromaffin_cells.Chrom_SOX2_statusHigh.PITPNA-AS1	2.3748021	1.67769277	5.94508731	8.43E-08	1.42E-06	7.73271383	PITPNA-AS1	assignmentsChromaffin_cells.Chrom_SOX2_statusHigh
assignmentsChromaffin_cells.Chrom_SOX2_statusHigh.EEF1AKMT2	-0.9865941	5.85490876	-5.9697677	7.62E-08	1.32E-06	7.7315734	EEF1AKMT2	assignmentsChromaffin_cells.Chrom_SOX2_statusHigh
assignmentsChromaffin_cells.Chrom_SOX2_statusHigh.MOCS2	1.69146583	4.08845064	5.96378587	7.81E-08	1.34E-06	7.73120266	MOCS2	assignmentsChromaffin_cells.Chrom_SOX2_statusHigh
assignmentsChromaffin_cells.Chrom_SOX2_statusHigh.PTPN4	-1.344674	6.81024573	-5.9739702	7.49E-08	1.30E-06	7.72602551	PTPN4	assignmentsChromaffin_cells.Chrom_SOX2_statusHigh
assignmentsChromaffin_cells.Chrom_SOX2_statusHigh.HSPBP1	1.35372576	3.13890301	5.95291595	8.17E-08	1.38E-06	7.72205466	HSPBP1	assignmentsChromaffin_cells.Chrom_SOX2_statusHigh
assignmentsChromaffin_cells.Chrom_SOX2_statusHigh.C17orf49	2.65228977	3.02407991	5.94401185	8.48E-08	1.43E-06	7.71921804	C17orf49	assignmentsChromaffin_cells.Chrom_SOX2_statusHigh
assignmentsChromaffin_cells.Chrom_SOX2_statusHigh.GASAL1	2.66827057	0.51787844	5.93866382	8.66E-08	1.45E-06	7.71504768	GASAL1	assignmentsChromaffin_cells.Chrom_SOX2_statusHigh
assignmentsChromaffin_cells.Chrom_SOX2_statusHigh.SLCO1C1	3.40764193	1.67004083	5.93316522	8.88E-08	1.48E-06	7.70993469	SLCO1C1	assignmentsChromaffin_cells.Chrom_SOX2_statusHigh
assignmentsChromaffin_cells.Chrom_SOX2_statusHigh.HSPA5	2.65788442	6.82363438	5.98159665	7.31E-08	1.27E-06	7.70852047	HSPA5	assignmentsChromaffin_cells.Chrom_SOX2_statusHigh
assignmentsChromaffin_cells.Chrom_SOX2_statusHigh.OGFOD3	1.1473618	4.07523738	5.95743567	8.02E-08	1.37E-06	7.70217127	OGFOD3	assignmentsChromaffin_cells.Chrom_SOX2_statusHigh
assignmentsChromaffin_cells.Chrom_SOX2_statusHigh.KIF22	1.40687583	3.49813219	5.9459793	8.40E-08	1.42E-06	7.69326904	KIF22	assignmentsChromaffin_cells.Chrom_SOX2_statusHigh
assignmentsChromaffin_cells.Chrom_SOX2_statusHigh.BET1	1.97088756	5.33245441	5.96779502	7.68E-08	1.32E-06	7.69044355	BET1	assignmentsChromaffin_cells.Chrom_SOX2_statusHigh
assignmentsChromaffin_cells.Chrom_SOX2_statusHigh.AGPS	-1.0828751	6.28149819	-5.9538315	8.14E-08	1.38E-06	7.67699869	AGPS	assignmentsChromaffin_cells.Chrom_SOX2_statusHigh
assignmentsChromaffin_cells.Chrom_SOX2_statusHigh.GLRX2	1.37551522	3.6549856	5.93909044	8.65E-08	1.45E-06	7.67334244	GLRX2	assignmentsChromaffin_cells.Chrom_SOX2_statusHigh
assignmentsChromaffin_cells.Chrom_SOX2_statusHigh.APH1A	1.89708194	4.09607882	5.94757644	8.35E-08	1.41E-06	7.66899173	APH1A	assignmentsChromaffin_cells.Chrom_SOX2_statusHigh
assignmentsChromaffin_cells.Chrom_SOX2_statusHigh.PCBP2	0.998132	7.07438142	5.96694645	7.71E-08	1.33E-06	7.66569288	PCBP2	assignmentsChromaffin_cells.Chrom_SOX2_statusHigh
assignmentsChromaffin_cells.Chrom_SOX2_statusHigh.RAD52	-1.2626255	4.62542495	-5.938366	8.67E-08	1.45E-06	7.65961685	RAD52	assignmentsChromaffin_cells.Chrom_SOX2_statusHigh
assignmentsChromaffin_cells.Chrom_SOX2_statusHigh.RPLP0	3.14408804	7.10507637	5.96790514	7.73E-08	1.33E-06	7.6412125	RPLP0	assignmentsChromaffin_cells.Chrom_SOX2_statusHigh
assignmentsChromaffin_cells.Chrom_SOX2_statusHigh.RPUSD2	1.78991414	1.83915981	5.9223371	9.26E-08	1.54E-06	7.63821322	RPUSD2	assignmentsChromaffin_cells.Chrom_SOX2_statusHigh
assignmentsChromaffin_cells.Chrom_SOX2_statusHigh.PGK1	1.56176938	6.69285284	5.9567088	8.04E-08	1.37E-06	7.63116992	PGK1	assignmentsChromaffin_cells.Chrom_SOX2_statusHigh
assignmentsChromaffin_cells.Chrom_SOX2_statusHigh.MED10	1.51555991	5.25148865	5.94396418	8.47E-08	1.43E-06	7.62548831	MED10	assignmentsChromaffin_cells.Chrom_SOX2_statusHigh
assignmentsChromaffin_cells.Chrom_SOX2_statusHigh.BCKDHA	1.44052621	3.42174216	5.92911962	9.01E-08	1.50E-06	7.62531278	BCKDHA	assignmentsChromaffin_cells.Chrom_SOX2_statusHigh
assignmentsChromaffin_cells.Chrom_SOX2_statusHigh.CCND1	2.04846129	4.47048956	5.93560838	8.77E-08	1.47E-06	7.61874265	CCND1	assignmentsChromaffin_cells.Chrom_SOX2_statusHigh
assignmentsChromaffin_cells.Chrom_SOX2_statusHigh.ITM2B	2.12957003	8.53967443	5.96763293	7.71E-08	1.33E-06	7.61362679	ITM2B	assignmentsChromaffin_cells.Chrom_SOX2_statusHigh

assignmentsChromaffin_cells.Chrom_SOX2_statusHigh.SMYD3	-1.2161599	8.93951419	-5.9583883	7.98E-08	1.36E-06	7.60754162	SMYD3	assignmentsChromaffin_cells.Chrom_SOX2_statusHigh
assignmentsChromaffin_cells.Chrom_SOX2_statusHigh.CZIB	1.65458605	3.51807127	5.91702805	9.47E-08	1.56E-06	7.58889208	CZIB	assignmentsChromaffin_cells.Chrom_SOX2_statusHigh
assignmentsChromaffin_cells.Chrom_SOX2_statusHigh.NUTF2	1.45438875	4.15742099	5.9243766	9.19E-08	1.53E-06	7.58139077	NUTF2	assignmentsChromaffin_cells.Chrom_SOX2_statusHigh
assignmentsChromaffin_cells.Chrom_SOX2_statusHigh.THYN1	1.83146511	3.56890324	5.92035527	9.34E-08	1.54E-06	7.57748934	THYN1	assignmentsChromaffin_cells.Chrom_SOX2_statusHigh
assignmentsChromaffin_cells.Chrom_SOX2_statusHigh.BAZ2B	-1.4296657	8.69698509	-5.9490497	8.30E-08	1.41E-06	7.57443647	BAZ2B	assignmentsChromaffin_cells.Chrom_SOX2_statusHigh
assignmentsChromaffin_cells.Chrom_SOX2_statusHigh.UTP3	1.65552169	3.43465673	5.91615379	9.50E-08	1.56E-06	7.57356439	UTP3	assignmentsChromaffin_cells.Chrom_SOX2_statusHigh
assignmentsChromaffin_cells.Chrom_SOX2_statusHigh.LIN37	1.32925591	3.17342905	5.91077007	9.72E-08	1.60E-06	7.55947458	LIN37	assignmentsChromaffin_cells.Chrom_SOX2_statusHigh
assignmentsChromaffin_cells.Chrom_SOX2_statusHigh.TPRG1L	2.09418109	2.67174395	5.90223543	1.01E-07	1.64E-06	7.55009242	TPRG1L	assignmentsChromaffin_cells.Chrom_SOX2_statusHigh
assignmentsChromaffin_cells.Chrom_SOX2_statusHigh.RPRD2	-1.0482728	7.18916045	-5.934791	8.80E-08	1.47E-06	7.54696914	RPRD2	assignmentsChromaffin_cells.Chrom_SOX2_statusHigh
assignmentsChromaffin_cells.Chrom_SOX2_statusHigh.REX1BD	2.17707298	5.35814401	5.92979702	9.00E-08	1.50E-06	7.54524733	REX1BD	assignmentsChromaffin_cells.Chrom_SOX2_statusHigh
assignmentsChromaffin_cells.Chrom_SOX2_statusHigh.D2HGDH	-3.0112353	5.15029809	-5.9041438	1.00E-07	1.64E-06	7.53862911	D2HGDH	assignmentsChromaffin_cells.Chrom_SOX2_statusHigh
assignmentsChromaffin_cells.Chrom_SOX2_statusHigh.SBF2	-1.4623178	9.00185616	-5.9419347	8.54E-08	1.44E-06	7.53742849	SBF2	assignmentsChromaffin_cells.Chrom_SOX2_statusHigh
assignmentsChromaffin_cells.Chrom_SOX2_statusHigh.TOMM7	3.14104295	5.99092383	5.93491487	8.86E-08	1.48E-06	7.53738641	TOMM7	assignmentsChromaffin_cells.Chrom_SOX2_statusHigh
assignmentsChromaffin_cells.Chrom_SOX2_statusHigh.PPM1G	1.17206242	5.65201927	5.92226327	9.27E-08	1.54E-06	7.53571772	PPM1G	assignmentsChromaffin_cells.Chrom_SOX2_statusHigh
assignmentsChromaffin_cells.Chrom_SOX2_statusHigh.MKLN1	-0.9778001	8.84168138	-5.9385607	8.66E-08	1.45E-06	7.52702684	MKLN1	assignmentsChromaffin_cells.Chrom_SOX2_statusHigh
assignmentsChromaffin_cells.Chrom_SOX2_statusHigh.LACTB2	1.46139596	3.44281331	5.90056785	1.01E-07	1.65E-06	7.52589698	LACTB2	assignmentsChromaffin_cells.Chrom_SOX2_statusHigh
assignmentsChromaffin_cells.Chrom_SOX2_statusHigh.MRPS30	1.37312441	4.27932675	5.90907729	9.79E-08	1.60E-06	7.51430761	MRPS30	assignmentsChromaffin_cells.Chrom_SOX2_statusHigh
assignmentsChromaffin_cells.Chrom_SOX2_statusHigh.RIDA	1.6746369	2.55978369	5.89336726	1.04E-07	1.70E-06	7.51298063	RIDA	assignmentsChromaffin_cells.Chrom_SOX2_statusHigh
assignmentsChromaffin_cells.Chrom_SOX2_statusHigh.CHRAC1	1.64654039	3.26378195	5.89149662	1.05E-07	1.70E-06	7.4887236	CHRAC1	assignmentsChromaffin_cells.Chrom_SOX2_statusHigh
assignmentsChromaffin_cells.Chrom_SOX2_statusHigh.GALK1	2.2688172	2.37794086	5.88170165	1.10E-07	1.76E-06	7.4885053	GALK1	assignmentsChromaffin_cells.Chrom_SOX2_statusHigh
assignmentsChromaffin_cells.Chrom_SOX2_statusHigh.NDUFB4	2.61003766	5.04085736	5.91280613	9.69E-08	1.59E-06	7.48628148	NDUFB4	assignmentsChromaffin_cells.Chrom_SOX2_statusHigh
assignmentsChromaffin_cells.Chrom_SOX2_statusHigh.IDE	-1.1789854	5.6712608	-5.9039031	1.00E-07	1.63E-06	7.48364384	IDE	assignmentsChromaffin_cells.Chrom_SOX2_statusHigh
assignmentsChromaffin_cells.Chrom_SOX2_statusHigh.PHLPP1	-2.1812793	6.40361457	-5.891906	1.05E-07	1.70E-06	7.47883594	PHLPP1	assignmentsChromaffin_cells.Chrom_SOX2_statusHigh
assignmentsChromaffin_cells.Chrom_SOX2_statusHigh.HLA-A	2.90661479	7.49887707	5.92227785	9.33E-08	1.54E-06	7.47693524	HLA-A	assignmentsChromaffin_cells.Chrom_SOX2_statusHigh
assignmentsChromaffin_cells.Chrom_SOX2_statusHigh.LRRN1	-2.349614	2.78903913	-5.878962	1.11E-07	1.78E-06	7.47418035	LRRN1	assignmentsChromaffin_cells.Chrom_SOX2_statusHigh
assignmentsChromaffin_cells.Chrom_SOX2_statusHigh.C8orf48	2.8924132	0.32713186	5.86945322	1.15E-07	1.83E-06	7.46516082	C8orf48	assignmentsChromaffin_cells.Chrom_SOX2_statusHigh
assignmentsChromaffin_cells.Chrom_SOX2_statusHigh.PAF1	1.63533491	3.68990562	5.89151215	1.05E-07	1.70E-06	7.46510803	PAF1	assignmentsChromaffin_cells.Chrom_SOX2_statusHigh
assignmentsChromaffin_cells.Chrom_SOX2_statusHigh.PSMD3	1.23268578	4.67411242	5.89871437	1.02E-07	1.66E-06	7.4615857	PSMD3	assignmentsChromaffin_cells.Chrom_SOX2_statusHigh
assignmentsChromaffin_cells.Chrom_SOX2_statusHigh.FTH1	3.60260737	8.20181238	5.92299502	9.31E-08	1.54E-06	7.45363923	FTH1	assignmentsChromaffin_cells.Chrom_SOX2_statusHigh
assignmentsChromaffin_cells.Chrom_SOX2_statusHigh.SLC35A2	1.74731311	2.41751163	5.87553332	1.12E-07	1.80E-06	7.44953785	SLC35A2	assignmentsChromaffin_cells.Chrom_SOX2_statusHigh
assignmentsChromaffin_cells.Chrom_SOX2_statusHigh.NACA	3.02637576	6.89437886	5.91801942	9.50E-08	1.56E-06	7.4483889	NACA	assignmentsChromaffin_cells.Chrom_SOX2_statusHigh
assignmentsChromaffin_cells.Chrom_SOX2_statusHigh.SQSTM1	2.0024509	7.29090877	5.91379042	9.62E-08	1.58E-06	7.44464932	SQSTM1	assignmentsChromaffin_cells.Chrom_SOX2_statusHigh
assignmentsChromaffin_cells.Chrom_SOX2_statusHigh.LSM3	2.39108065	3.59924821	5.88593462	1.08E-07	1.74E-06	7.44358891	LSM3	assignmentsChromaffin_cells.Chrom_SOX2_statusHigh
assignmentsChromaffin_cells.Chrom_SOX2_statusHigh.ABHD14B	2.07244374	3.11225132	5.87656481	1.12E-07	1.79E-06	7.43815496	ABHD14B	assignmentsChromaffin_cells.Chrom_SOX2_statusHigh
assignmentsChromaffin_cells.Chrom_SOX2_statusHigh.MRFAP1L1	1.62978823	3.99148368	5.88488024	1.08E-07	1.74E-06	7.42837044	MRFAP1L1	assignmentsChromaffin_cells.Chrom_SOX2_statusHigh
assignmentsChromaffin_cells.Chrom_SOX2_statusHigh.YIF1A	2.25202252	4.03685971	5.8879968	1.07E-07	1.73E-06	7.41636249	YIF1A	assignmentsChromaffin_cells.Chrom_SOX2_statusHigh
assignmentsChromaffin_cells.Chrom_SOX2_statusHigh.PLPBP	1.17525457	4.17960468	5.8805833	1.10E-07	1.77E-06	7.41447835	PLPBP	assignmentsChromaffin_cells.Chrom_SOX2_statusHigh
assignmentsChromaffin_cells.Chrom_SOX2_statusHigh.RPL8	3.56505138	7.2151379	5.91141919	9.76E-08	1.60E-06	7.41156149	RPL8	assignmentsChromaffin_cells.Chrom_SOX2_statusHigh
assignmentsChromaffin_cells.Chrom_SOX2_statusHigh.RPL7A	3.164339	7.33175922	5.91077408	9.79E-08	1.60E-06	7.40870531	RPL7A	assignmentsChromaffin_cells.Chrom_SOX2_statusHigh
assignmentsChromaffin_cells.Chrom_SOX2_statusHigh.SEC31B	-1.531585	4.70178922	-5.8767809	1.12E-07	1.79E-06	7.40112866	SEC31B	assignmentsChromaffin_cells.Chrom_SOX2_statusHigh
assignmentsChromaffin_cells.Chrom_SOX2_statusHigh.MTLN	2.39689747	3.56336302	5.87724982	1.12E-07	1.79E-06	7.39763232	MTLN	assignmentsChromaffin_cells.Chrom_SOX2_statusHigh
assignmentsChromaffin_cells.Chrom_SOX2_statusHigh.CCDC127	1.36427277	3.42447818	5.87347916	1.13E-07	1.81E-06	7.39571945	CCDC127	assignmentsChromaffin_cells.Chrom_SOX2_statusHigh
assignmentsChromaffin_cells.Chrom_SOX2_statusHigh.CFAP58	3.20682157	0.94262015	5.84946409	1.25E-07	1.96E-06	7.38494144	CFAP58	assignmentsChromaffin_cells.Chrom_SOX2_statusHigh
assignmentsChromaffin_cells.Chrom_SOX2_statusHigh.RGS19	3.27813353	2.22851753	5.85052775	1.25E-07	1.95E-06	7.38469096	RGS19	assignmentsChromaffin_cells.Chrom_SOX2_statusHigh
assignmentsChromaffin_cells.Chrom_SOX2_statusHigh.RPP21	2.02498643	3.60399758	5.86834518	1.16E-07	1.84E-06	7.3744779	RPP21	assignmentsChromaffin_cells.Chrom_SOX2_statusHigh
assignmentsChromaffin_cells.Chrom_SOX2_statusHigh.C18orf32	2.32462058	4.88432107	5.88357586	1.09E-07	1.76E-06	7.37373818	C18orf32	assignmentsChromaffin_cells.Chrom_SOX2_statusHigh
assignmentsChromaffin_cells.Chrom_SOX2_statusHigh.MTREX	-0.8628269	6.81528423	-5.8874695	1.07E-07	1.73E-06	7.3710983	MTREX	assignmentsChromaffin_cells.Chrom_SOX2_statusHigh
assignmentsChromaffin_cells.Chrom_SOX2_statusHigh.TRAPPC4	1.70953368	4.23649464	5.86715371	1.16E-07	1.84E-06	7.34738558	TRAPPC4	assignmentsChromaffin_cells.Chrom_SOX2_statusHigh
assignmentsChromaffin_cells.Chrom_SOX2_statusHigh.ZNHIT1	1.93233249	4.43191124	5.86737471	1.16E-07	1.84E-06	7.34362945	ZNHIT1	assignmentsChromaffin_cells.Chrom_SOX2_statusHigh
assignmentsChromaffin_cells.Chrom_SOX2_statusHigh.FTL	4.49411861	7.64061449	5.89488936	1.04E-07	1.70E-06	7.33786963	FTL	assignmentsChromaffin_cells.Chrom_SOX2_statusHigh
assignmentsChromaffin_cells.Chrom_SOX2_statusHigh.LINC00900	4.34069006	-0.2745513	5.86397415	1.18E-07	1.87E-06	7.33200528	LINC00900	assignmentsChromaffin_cells.Chrom_SOX2_statusHigh
assignmentsChromaffin_cells.Chrom_SOX2_statusHigh.EBLN3P	0.8821499	5.0919211	5.86963107	1.15E-07	1.83E-06	7.32921243	EBLN3P	assignmentsChromaffin_cells.Chrom_SOX2_statusHigh
assignmentsChromaffin_cells.Chrom_SOX2_statusHigh.USP49	-1.4437048	4.6593237	-5.8505063	1.25E-07	1.95E-06	7.31422935	USP49	assignmentsChromaffin_cells.Chrom_SOX2_statusHigh
assignmentsChromaffin_cells.Chrom_SOX2_statusHigh.ARL6IP5	2.30257735	5.61773173	5.86953718	1.15E-07	1.84E-06	7.30575668	ARL6IP5	assignmentsChromaffin_cells.Chrom_SOX2_statusHigh
assignmentsChromaffin_cells.Chrom_SOX2_statusHigh.NFE2L1	1.16045772	5.75052012	5.86805526	1.16E-07	1.84E-06	7.29506794	NFE2L1	assignmentsChromaffin_cells.Chrom_SOX2_statusHigh
assignmentsChromaffin_cells.Chrom_SOX2_statusHigh.POMP	1.8166897	5.81498626	5.86372743	1.18E-07	1.87E-06	7.29365921	POMP	assignmentsChromaffin_cells.Chrom_SOX2_statusHigh
assignmentsChromaffin_cells.Chrom_SOX2_statusHigh.DAND5	3.0655726	-0.1190175	5.832149	1.34E-07	2.08E-06	7.28944163	DAND5	assignmentsChromaffin_cells.Chrom_SOX2_statusHigh
assignmentsChromaffin_cells.Chrom_SOX2_statusHigh.MOAP1	2.08114473	2.94875363	5.84281901	1.29E-07	2.00E-06	7.28870284	MOAP1	assignmentsChromaffin_cells.Chrom_SOX2_statusHigh
assignmentsChromaffin_cells.Chrom_SOX2_statusHigh.APOL4	3.58393224	0.33632281	5.85466821	1.22E-07	1.93E-06	7.28408511	APOL4	assignmentsChromaffin_cells.Chrom_SOX2_statusHigh
assignmentsChromaffin_cells.Chrom_SOX2_statusHigh.COMMD4	1.65880685	3.7970717	5.84732125	1.26E-07	1.97E-06	7.28138093	COMMD4	assignmentsChromaffin_cells.Chrom_SOX2_statusHigh
assignmentsChromaffin_cells.Chrom_SOX2_statusHigh.GNAQ	-1.2861064	8.70169525	-5.8726792	1.14E-07	1.81E-06	7.27283653	GNAQ	assignmentsChromaffin_cells.Chrom_SOX2_statusHigh
assignmentsChromaffin_cells.Chrom_SOX2_statusHigh.HSPA8	2.95201615	7.15916899	5.87437444	1.14E-07	1.81E-06	7.2719913	HSPA8	assignmentsChromaffin_cells.Chrom_SOX2_statusHigh
assignmentsChromaffin_cells.Chrom_SOX2_statusHigh.MFSD9	-1.4246768	3.12466035	-5.8281407	1.36E-07	2.11E-06	7.25998383	MFSD9	assignmentsChromaffin_cells.Chrom_SOX2_statusHigh
assignmentsChromaffin_cells.Chrom_SOX2_statusHigh.PFDN2	1.75007748	5.41166366	5.8546551	1.22E-07	1.93E-06	7.25966237	PFDN2	assignmentsChromaffin_cells.Chrom_SOX2_statusHigh

assignmentsChromaffin_cells.Chrom_SOX2_statusHigh.PGLS	2.05399621	4.24670791	5.84334133	1.28E-07	2.00E-06	7.25184123	PGLS	assignmentsChromaffin_cells.Chrom_SOX2_statusHigh
assignmentsChromaffin_cells.Chrom_SOX2_statusHigh.SUGCT	-2.6580167	5.71880007	-5.839558	1.31E-07	2.03E-06	7.24703637	SUGCT	assignmentsChromaffin_cells.Chrom_SOX2_statusHigh
assignmentsChromaffin_cells.Chrom_SOX2_statusHigh.TMED7	1.73816066	5.06905824	5.84949679	1.25E-07	1.96E-06	7.24534934	TMED7	assignmentsChromaffin_cells.Chrom_SOX2_statusHigh
assignmentsChromaffin_cells.Chrom_SOX2_statusHigh.ITCH	-0.9941211	7.51829561	-5.8567781	1.21E-07	1.92E-06	7.24231019	ITCH	assignmentsChromaffin_cells.Chrom_SOX2_statusHigh
assignmentsChromaffin_cells.Chrom_SOX2_statusHigh.ERG28	1.38031712	3.2889676	5.83159471	1.35E-07	2.08E-06	7.22874425	ERG28	assignmentsChromaffin_cells.Chrom_SOX2_statusHigh
assignmentsChromaffin_cells.Chrom_SOX2_statusHigh.SPIN1	-1.1554662	6.73212372	-5.845998	1.27E-07	1.98E-06	7.22821592	SPIN1	assignmentsChromaffin_cells.Chrom_SOX2_statusHigh
assignmentsChromaffin_cells.Chrom_SOX2_statusHigh.RPL18A	3.92405841	6.74577805	5.86112924	1.20E-07	1.90E-06	7.22698028	RPL18A	assignmentsChromaffin_cells.Chrom_SOX2_statusHigh
assignmentsChromaffin_cells.Chrom_SOX2_statusHigh.NDUFB3	2.50058027	4.05920885	5.83795167	1.31E-07	2.04E-06	7.22232608	NDUFB3	assignmentsChromaffin_cells.Chrom_SOX2_statusHigh
assignmentsChromaffin_cells.Chrom_SOX2_statusHigh.ENO1	2.42799765	5.37538521	5.83840225	1.31E-07	2.04E-06	7.21138757	ENO1	assignmentsChromaffin_cells.Chrom_SOX2_statusHigh
assignmentsChromaffin_cells.Chrom_SOX2_statusHigh.RPL36	3.56484244	6.77175624	5.85532351	1.23E-07	1.93E-06	7.20027512	RPL36	assignmentsChromaffin_cells.Chrom_SOX2_statusHigh
assignmentsChromaffin_cells.Chrom_SOX2_statusHigh.CANX	1.89473261	7.11818383	5.85369627	1.23E-07	1.93E-06	7.19673766	CANX	assignmentsChromaffin_cells.Chrom_SOX2_statusHigh
assignmentsChromaffin_cells.Chrom_SOX2_statusHigh.OR2L5	-4.3202187	1.22401129	-5.811766	1.46E-07	2.22E-06	7.19664163	OR2L5	assignmentsChromaffin_cells.Chrom_SOX2_statusHigh
assignmentsChromaffin_cells.Chrom_SOX2_statusHigh.SLC25A3	2.03274801	6.44243692	5.84820489	1.26E-07	1.97E-06	7.19589664	SLC25A3	assignmentsChromaffin_cells.Chrom_SOX2_statusHigh
assignmentsChromaffin_cells.Chrom_SOX2_statusHigh.TMEM160	2.58513058	3.30704555	5.8208423	1.41E-07	2.16E-06	7.19074467	TMEM160	assignmentsChromaffin_cells.Chrom_SOX2_statusHigh
assignmentsChromaffin_cells.Chrom_SOX2_statusHigh.FEM1A	1.47875629	2.30499142	5.80618957	1.49E-07	2.27E-06	7.18172063	FEM1A	assignmentsChromaffin_cells.Chrom_SOX2_statusHigh
assignmentsChromaffin_cells.Chrom_SOX2_statusHigh.DYNLT3	1.9252279	4.90051319	5.8333174	1.34E-07	2.07E-06	7.18090496	DYNLT3	assignmentsChromaffin_cells.Chrom_SOX2_statusHigh
assignmentsChromaffin_cells.Chrom_SOX2_statusHigh.CHAC1	2.18469244	1.25396078	5.8025087	1.52E-07	2.29E-06	7.17876432	CHAC1	assignmentsChromaffin_cells.Chrom_SOX2_statusHigh
assignmentsChromaffin_cells.Chrom_SOX2_statusHigh.POLR2C	1.40873592	3.88211852	5.82182352	1.40E-07	2.16E-06	7.17722719	POLR2C	assignmentsChromaffin_cells.Chrom_SOX2_statusHigh
assignmentsChromaffin_cells.Chrom_SOX2_statusHigh.LSM5	1.91403475	4.11207798	5.82371653	1.39E-07	2.14E-06	7.16296883	LSM5	assignmentsChromaffin_cells.Chrom_SOX2_statusHigh
assignmentsChromaffin_cells.Chrom_SOX2_statusHigh.ACAA2	1.41776196	4.518694	5.82154732	1.40E-07	2.16E-06	7.15904854	ACAA2	assignmentsChromaffin_cells.Chrom_SOX2_statusHigh
assignmentsChromaffin_cells.Chrom_SOX2_statusHigh.RAB4B	1.43008471	4.45911836	5.8137727	1.45E-07	2.21E-06	7.15836163	RAB4B	assignmentsChromaffin_cells.Chrom_SOX2_statusHigh
assignmentsChromaffin_cells.Chrom_SOX2_statusHigh.IGSF1	4.18105583	0.50305182	5.79095783	1.60E-07	2.40E-06	7.15596839	IGSF1	assignmentsChromaffin_cells.Chrom_SOX2_statusHigh
assignmentsChromaffin_cells.Chrom_SOX2_statusHigh.VAT1	1.93386172	4.3906034	5.82147479	1.40E-07	2.16E-06	7.15043753	VAT1	assignmentsChromaffin_cells.Chrom_SOX2_statusHigh
assignmentsChromaffin_cells.Chrom_SOX2_statusHigh.FAM210B	2.07465674	3.93895074	5.81471589	1.44E-07	2.20E-06	7.14785562	FAM210B	assignmentsChromaffin_cells.Chrom_SOX2_statusHigh
assignmentsChromaffin_cells.Chrom_SOX2_statusHigh.USP1	1.36631675	4.19764428	5.8090459	1.48E-07	2.24E-06	7.13627957	USP1	assignmentsChromaffin_cells.Chrom_SOX2_statusHigh
assignmentsChromaffin_cells.Chrom_SOX2_statusHigh.HSP90B1	2.39163048	7.64843508	5.843806	1.29E-07	2.01E-06	7.13514665	HSP90B1	assignmentsChromaffin_cells.Chrom_SOX2_statusHigh
assignmentsChromaffin_cells.Chrom_SOX2_statusHigh.MDH1	1.43444161	5.59073448	5.82922402	1.36E-07	2.10E-06	7.13446394	MDH1	assignmentsChromaffin_cells.Chrom_SOX2_statusHigh
assignmentsChromaffin_cells.Chrom_SOX2_statusHigh.HEXB	0.97474791	5.58997587	5.82040049	1.41E-07	2.16E-06	7.13369191	HEXB	assignmentsChromaffin_cells.Chrom_SOX2_statusHigh
assignmentsChromaffin_cells.Chrom_SOX2_statusHigh.LCOR	-0.9950721	7.32890526	-5.8282179	1.36E-07	2.11E-06	7.13289717	LCOR	assignmentsChromaffin_cells.Chrom_SOX2_statusHigh
assignmentsChromaffin_cells.Chrom_SOX2_statusHigh.ANKAR	-1.6812914	5.88918563	-5.8123912	1.46E-07	2.22E-06	7.1275816	ANKAR	assignmentsChromaffin_cells.Chrom_SOX2_statusHigh
assignmentsChromaffin_cells.Chrom_SOX2_statusHigh.RORC	4.47108387	1.49699292	5.78897098	1.61E-07	2.42E-06	7.12429274	RORC	assignmentsChromaffin_cells.Chrom_SOX2_statusHigh
assignmentsChromaffin_cells.Chrom_SOX2_statusHigh.KIAA1143	1.47227261	3.76957537	5.80328862	1.51E-07	2.29E-06	7.11774666	KIAA1143	assignmentsChromaffin_cells.Chrom_SOX2_statusHigh
assignmentsChromaffin_cells.Chrom_SOX2_statusHigh.TAPT1	-1.0683373	5.88363556	-5.8118543	1.46E-07	2.22E-06	7.11591396	TAPT1	assignmentsChromaffin_cells.Chrom_SOX2_statusHigh
assignmentsChromaffin_cells.Chrom_SOX2_statusHigh.SSR2	1.95673137	5.43485539	5.81754839	1.43E-07	2.18E-06	7.11399387	SSR2	assignmentsChromaffin_cells.Chrom_SOX2_statusHigh
assignmentsChromaffin_cells.Chrom_SOX2_statusHigh.TLE6	2.9323523	0.90616425	5.778995	1.67E-07	2.49E-06	7.11295787	TLE6	assignmentsChromaffin_cells.Chrom_SOX2_statusHigh
assignmentsChromaffin_cells.Chrom_SOX2_statusHigh.NDUFS6	1.86039638	5.09984948	5.81522039	1.44E-07	2.20E-06	7.10906327	NDUFS6	assignmentsChromaffin_cells.Chrom_SOX2_statusHigh
assignmentsChromaffin_cells.Chrom_SOX2_statusHigh.CAMK2A	4.17254025	-0.015758	5.7926218	1.58E-07	2.38E-06	7.09912586	CAMK2A	assignmentsChromaffin_cells.Chrom_SOX2_statusHigh
assignmentsChromaffin_cells.Chrom_SOX2_statusHigh.ORMDL3	1.81108885	3.21668759	5.79410926	1.57E-07	2.37E-06	7.09844921	ORMDL3	assignmentsChromaffin_cells.Chrom_SOX2_statusHigh
assignmentsChromaffin_cells.Chrom_SOX2_statusHigh.FANCF	1.93426895	1.50455409	5.77846495	1.67E-07	2.49E-06	7.0950474	FANCF	assignmentsChromaffin_cells.Chrom_SOX2_statusHigh
assignmentsChromaffin_cells.Chrom_SOX2_statusHigh.TLK1	-1.0061882	7.49985221	-5.8196501	1.41E-07	2.17E-06	7.08045691	TLK1	assignmentsChromaffin_cells.Chrom_SOX2_statusHigh
assignmentsChromaffin_cells.Chrom_SOX2_statusHigh.MANBAL	1.11753977	3.97588767	5.79451466	1.57E-07	2.36E-06	7.07600903	MANBAL	assignmentsChromaffin_cells.Chrom_SOX2_statusHigh
assignmentsChromaffin_cells.Chrom_SOX2_statusHigh.HTATSF1	2.95446691	5.33889666	5.81619306	1.44E-07	2.20E-06	7.06556537	HTATSF1	assignmentsChromaffin_cells.Chrom_SOX2_statusHigh
assignmentsChromaffin_cells.Chrom_SOX2_statusHigh.NAA10	1.5074199	4.54737949	5.79562452	1.56E-07	2.36E-06	7.06149594	NAA10	assignmentsChromaffin_cells.Chrom_SOX2_statusHigh
assignmentsChromaffin_cells.Chrom_SOX2_statusHigh.RPL4	2.80033916	6.28722093	5.81337476	1.46E-07	2.22E-06	7.05975583	RPL4	assignmentsChromaffin_cells.Chrom_SOX2_statusHigh
assignmentsChromaffin_cells.Chrom_SOX2_statusHigh.FKBP8	1.83253671	5.23075605	5.80382936	1.51E-07	2.29E-06	7.05512593	FKBP8	assignmentsChromaffin_cells.Chrom_SOX2_statusHigh
assignmentsChromaffin_cells.Chrom_SOX2_statusHigh.DCK	1.6478458	3.30675475	5.77795332	1.68E-07	2.49E-06	7.04924839	DCK	assignmentsChromaffin_cells.Chrom_SOX2_statusHigh
assignmentsChromaffin_cells.Chrom_SOX2_statusHigh.YIPF2	1.62826063	3.97419283	5.79351805	1.57E-07	2.37E-06	7.04707744	YIPF2	assignmentsChromaffin_cells.Chrom_SOX2_statusHigh
assignmentsChromaffin_cells.Chrom_SOX2_statusHigh.ARF6	2.25452309	3.91471197	5.77978273	1.66E-07	2.49E-06	7.04307054	ARF6	assignmentsChromaffin_cells.Chrom_SOX2_statusHigh
assignmentsChromaffin_cells.Chrom_SOX2_statusHigh.NDUFB6	1.39741987	4.97530867	5.79780901	1.55E-07	2.34E-06	7.04238764	NDUFB6	assignmentsChromaffin_cells.Chrom_SOX2_statusHigh
assignmentsChromaffin_cells.Chrom_SOX2_statusHigh.GRPEL1	1.93546827	3.4879862	5.77697784	1.68E-07	2.50E-06	7.0263313	GRPEL1	assignmentsChromaffin_cells.Chrom_SOX2_statusHigh
assignmentsChromaffin_cells.Chrom_SOX2_statusHigh.SNX33	2.26829016	1.83569094	5.75691562	1.83E-07	2.68E-06	7.02242341	SNX33	assignmentsChromaffin_cells.Chrom_SOX2_statusHigh
assignmentsChromaffin_cells.Chrom_SOX2_statusHigh.NDUFS8	1.81429425	4.83354626	5.79175664	1.58E-07	2.38E-06	7.01911618	NDUFS8	assignmentsChromaffin_cells.Chrom_SOX2_statusHigh
assignmentsChromaffin_cells.Chrom_SOX2_statusHigh.USP54	-1.4047451	5.11144368	-5.7783605	1.67E-07	2.49E-06	7.01483609	USP54	assignmentsChromaffin_cells.Chrom_SOX2_statusHigh
assignmentsChromaffin_cells.Chrom_SOX2_statusHigh.STK38	-1.0946712	6.66333263	-5.7901577	1.59E-07	2.40E-06	7.01228638	STK38	assignmentsChromaffin_cells.Chrom_SOX2_statusHigh
assignmentsChromaffin_cells.Chrom_SOX2_statusHigh.TOR3A	1.35568742	3.32982939	5.76847589	1.74E-07	2.57E-06	7.0059866	TOR3A	assignmentsChromaffin_cells.Chrom_SOX2_statusHigh
assignmentsChromaffin_cells.Chrom_SOX2_statusHigh.THAP10	1.56614509	0.99282319	5.75428117	1.85E-07	2.70E-06	7.00508826	THAP10	assignmentsChromaffin_cells.Chrom_SOX2_statusHigh
assignmentsChromaffin_cells.Chrom_SOX2_statusHigh.SNRPB	1.79131858	4.93822839	5.78128995	1.65E-07	2.47E-06	6.99620397	SNRPB	assignmentsChromaffin_cells.Chrom_SOX2_statusHigh
assignmentsChromaffin_cells.Chrom_SOX2_statusHigh.ORC2	-1.0103416	6.04168955	-5.7847959	1.63E-07	2.44E-06	6.99552794	ORC2	assignmentsChromaffin_cells.Chrom_SOX2_statusHigh
assignmentsChromaffin_cells.Chrom_SOX2_statusHigh.SURF1	1.68385706	3.45363682	5.76819458	1.74E-07	2.57E-06	6.98833966	SURF1	assignmentsChromaffin_cells.Chrom_SOX2_statusHigh
assignmentsChromaffin_cells.Chrom_SOX2_statusHigh.DHRS7	1.78123712	5.10630102	5.78284248	1.64E-07	2.46E-06	6.98732944	DHRS7	assignmentsChromaffin_cells.Chrom_SOX2_statusHigh
assignmentsChromaffin_cells.Chrom_SOX2_statusHigh.CCNJL	3.49399909	1.79001663	5.74622708	1.91E-07	2.79E-06	6.9815617	CCNJL	assignmentsChromaffin_cells.Chrom_SOX2_statusHigh
assignmentsChromaffin_cells.Chrom_SOX2_statusHigh.RIMKLB	-1.4711189	5.81745294	-5.7693966	1.74E-07	2.56E-06	6.96311574	RIMKLB	assignmentsChromaffin_cells.Chrom_SOX2_statusHigh
assignmentsChromaffin_cells.Chrom_SOX2_statusHigh.RAB39B	1.5934024	2.1046998	5.75657955	1.83E-07	2.68E-06	6.96263368	RAB39B	assignmentsChromaffin_cells.Chrom_SOX2_statusHigh
assignmentsChromaffin_cells.Chrom_SOX2_statusHigh.PEX11G	2.29216362	1.28278448	5.74096654	1.95E-07	2.83E-06	6.96243443	PEX11G	assignmentsChromaffin_cells.Chrom_SOX2_statusHigh

assignmentsChromaffin_cells.Chrom_SOX2_statusHigh.TRMT44	-1.0429783	4.12523604	-5.7576245	1.82E-07	2.67E-06	6.95714463	TRMT44	assignmentsChromaffin_cells.Chrom_SOX2_statusHigh
assignmentsChromaffin_cells.Chrom_SOX2_statusHigh.SAMD9	2.53704422	3.81578629	5.74830255	1.89E-07	2.76E-06	6.95607629	SAMD9	assignmentsChromaffin_cells.Chrom_SOX2_statusHigh
assignmentsChromaffin_cells.Chrom_SOX2_statusHigh.L3MBTL4	-3.3253908	6.56615809	-5.763319	1.79E-07	2.64E-06	6.94377425	L3MBTL4	assignmentsChromaffin_cells.Chrom_SOX2_statusHigh
assignmentsChromaffin_cells.Chrom_SOX2_statusHigh.CENPH	1.66226789	1.62273412	5.73932865	1.96E-07	2.84E-06	6.93853343	CENPH	assignmentsChromaffin_cells.Chrom_SOX2_statusHigh
assignmentsChromaffin_cells.Chrom_SOX2_statusHigh.PNRC2	1.6712975	4.95654886	5.75989243	1.81E-07	2.65E-06	6.93639894	PNRC2	assignmentsChromaffin_cells.Chrom_SOX2_statusHigh
assignmentsChromaffin_cells.Chrom_SOX2_statusHigh.SYNJ1	-0.9013806	6.38519367	-5.7731442	1.71E-07	2.53E-06	6.93290421	SYNJ1	assignmentsChromaffin_cells.Chrom_SOX2_statusHigh
assignmentsChromaffin_cells.Chrom_SOX2_statusHigh.SNHG8	2.57107611	5.22891977	5.77825374	1.68E-07	2.50E-06	6.93225942	SNHG8	assignmentsChromaffin_cells.Chrom_SOX2_statusHigh
assignmentsChromaffin_cells.Chrom_SOX2_statusHigh.RBIS	1.63768094	5.1863193	5.77029925	1.73E-07	2.56E-06	6.93033681	RBIS	assignmentsChromaffin_cells.Chrom_SOX2_statusHigh
assignmentsChromaffin_cells.Chrom_SOX2_statusHigh.PDHB	1.34067836	4.0607697	5.7575454	1.82E-07	2.67E-06	6.92685949	PDHB	assignmentsChromaffin_cells.Chrom_SOX2_statusHigh
assignmentsChromaffin_cells.Chrom_SOX2_statusHigh.CCDC121	2.08840409	1.43857135	5.73446839	2.00E-07	2.90E-06	6.92326595	CCDC121	assignmentsChromaffin_cells.Chrom_SOX2_statusHigh
assignmentsChromaffin_cells.Chrom_SOX2_statusHigh.MGST3	1.95560449	6.04713453	5.7748895	1.70E-07	2.52E-06	6.91897796	MGST3	assignmentsChromaffin_cells.Chrom_SOX2_statusHigh
assignmentsChromaffin_cells.Chrom_SOX2_statusHigh.MGAT2	2.01625397	2.3145454	5.73727957	1.98E-07	2.87E-06	6.91182411	MGAT2	assignmentsChromaffin_cells.Chrom_SOX2_statusHigh
assignmentsChromaffin_cells.Chrom_SOX2_statusHigh.PPA1	1.66689706	5.81432404	5.77181634	1.72E-07	2.54E-06	6.90752747	PPA1	assignmentsChromaffin_cells.Chrom_SOX2_statusHigh
assignmentsChromaffin_cells.Chrom_SOX2_statusHigh.PDK2	2.03528766	3.37450244	5.75017006	1.88E-07	2.74E-06	6.90051872	PDK2	assignmentsChromaffin_cells.Chrom_SOX2_statusHigh
assignmentsChromaffin_cells.Chrom_SOX2_statusHigh.GNGT1	2.98293256	0.41990465	5.72298322	2.10E-07	3.01E-06	6.89871083	GNGT1	assignmentsChromaffin_cells.Chrom_SOX2_statusHigh
assignmentsChromaffin_cells.Chrom_SOX2_statusHigh.AKT1S1	1.77875391	2.66206936	5.74023924	1.96E-07	2.84E-06	6.89619352	AKT1S1	assignmentsChromaffin_cells.Chrom_SOX2_statusHigh
assignmentsChromaffin_cells.Chrom_SOX2_statusHigh.ZBTB45	1.50184896	1.87357902	5.72854609	2.05E-07	2.95E-06	6.89417672	ZBTB45	assignmentsChromaffin_cells.Chrom_SOX2_statusHigh
assignmentsChromaffin_cells.Chrom_SOX2_statusHigh.TRIP12	-3.3430259	7.81539883	-5.7542411	1.86E-07	2.72E-06	6.87171195	TRIP12	assignmentsChromaffin_cells.Chrom_SOX2_statusHigh
assignmentsChromaffin_cells.Chrom_SOX2_statusHigh.NUAK2	3.74172283	1.66420187	5.72898564	2.05E-07	2.95E-06	6.87038803	NUAK2	assignmentsChromaffin_cells.Chrom_SOX2_statusHigh
assignmentsChromaffin_cells.Chrom_SOX2_statusHigh.DNAL4	1.96293825	1.3950906	5.72007935	2.12E-07	3.04E-06	6.86395044	DNAL4	assignmentsChromaffin_cells.Chrom_SOX2_statusHigh
assignmentsChromaffin_cells.Chrom_SOX2_statusHigh.RPL30	3.41539604	7.18206718	5.77397816	1.72E-07	2.54E-06	6.86337894	RPL30	assignmentsChromaffin_cells.Chrom_SOX2_statusHigh
assignmentsChromaffin_cells.Chrom_SOX2_statusHigh.SDHD	1.33887298	5.11250086	5.74429803	1.92E-07	2.80E-06	6.83878068	SDHD	assignmentsChromaffin_cells.Chrom_SOX2_statusHigh
assignmentsChromaffin_cells.Chrom_SOX2_statusHigh.RPS12	4.09174184	7.3156132	5.76644871	1.77E-07	2.61E-06	6.82956096	RPS12	assignmentsChromaffin_cells.Chrom_SOX2_statusHigh
assignmentsChromaffin_cells.Chrom_SOX2_statusHigh.KLHL9	1.10411067	4.12294108	5.73138733	2.03E-07	2.93E-06	6.81776481	KLHL9	assignmentsChromaffin_cells.Chrom_SOX2_statusHigh
assignmentsChromaffin_cells.Chrom_SOX2_statusHigh.TCP1	2.01220905	5.52422012	5.7423637	1.94E-07	2.82E-06	6.81673716	TCP1	assignmentsChromaffin_cells.Chrom_SOX2_statusHigh
assignmentsChromaffin_cells.Chrom_SOX2_statusHigh.TMEM9B	1.40253779	4.39104113	5.72847868	2.05E-07	2.95E-06	6.81294921	TMEM9B	assignmentsChromaffin_cells.Chrom_SOX2_statusHigh
assignmentsChromaffin_cells.Chrom_SOX2_statusHigh.SURF2	1.65442259	2.99375721	5.71982651	2.13E-07	3.04E-06	6.81229247	SURF2	assignmentsChromaffin_cells.Chrom_SOX2_statusHigh
assignmentsChromaffin_cells.Chrom_SOX2_statusHigh.FAM229B	2.31169677	2.32772668	5.71547561	2.16E-07	3.08E-06	6.80222005	FAM229B	assignmentsChromaffin_cells.Chrom_SOX2_statusHigh
assignmentsChromaffin_cells.Chrom_SOX2_statusHigh.TALDO1	1.8979894	4.42825527	5.72394764	2.09E-07	3.00E-06	6.79911735	TALDO1	assignmentsChromaffin_cells.Chrom_SOX2_statusHigh
assignmentsChromaffin_cells.Chrom_SOX2_statusHigh.ZBTB11-AS	1.82655315	2.11258841	5.70173451	2.29E-07	3.22E-06	6.79145504	ZBTB11-AS1	assignmentsChromaffin_cells.Chrom_SOX2_statusHigh
assignmentsChromaffin_cells.Chrom_SOX2_statusHigh.TTLL4	-1.0281778	3.52501279	-5.7060394	2.25E-07	3.19E-06	6.78826514	TTLL4	assignmentsChromaffin_cells.Chrom_SOX2_statusHigh
assignmentsChromaffin_cells.Chrom_SOX2_statusHigh.STX18	-0.9462942	6.33215454	-5.7340203	2.01E-07	2.90E-06	6.78603913	STX18	assignmentsChromaffin_cells.Chrom_SOX2_statusHigh
assignmentsChromaffin_cells.Chrom_SOX2_statusHigh.ISOC1	1.37926079	2.47317777	5.70727921	2.24E-07	3.18E-06	6.78581274	ISOC1	assignmentsChromaffin_cells.Chrom_SOX2_statusHigh
assignmentsChromaffin_cells.Chrom_SOX2_statusHigh.UBL7	1.64280245	2.31586346	5.7040695	2.27E-07	3.21E-06	6.78515499	UBL7	assignmentsChromaffin_cells.Chrom_SOX2_statusHigh
assignmentsChromaffin_cells.Chrom_SOX2_statusHigh.MPC2	1.93553194	4.53757692	5.72694231	2.07E-07	2.97E-06	6.77040933	MPC2	assignmentsChromaffin_cells.Chrom_SOX2_statusHigh
assignmentsChromaffin_cells.Chrom_SOX2_statusHigh.CPEB3	-1.4522836	7.48665083	-5.7399219	1.96E-07	2.84E-06	6.76086239	CPEB3	assignmentsChromaffin_cells.Chrom_SOX2_statusHigh
assignmentsChromaffin_cells.Chrom_SOX2_statusHigh.CALM1	2.4566459	7.93982888	5.74914936	1.90E-07	2.77E-06	6.74820916	CALM1	assignmentsChromaffin_cells.Chrom_SOX2_statusHigh
assignmentsChromaffin_cells.Chrom_SOX2_statusHigh.MAPKAP1	-1.2731592	7.29477482	-5.726761	2.07E-07	2.97E-06	6.7399155	MAPKAP1	assignmentsChromaffin_cells.Chrom_SOX2_statusHigh
assignmentsChromaffin_cells.Chrom_SOX2_statusHigh.PGAM1	2.12580516	5.00026584	5.72276667	2.10E-07	3.01E-06	6.73796072	PGAM1	assignmentsChromaffin_cells.Chrom_SOX2_statusHigh
assignmentsChromaffin_cells.Chrom_SOX2_statusHigh.RANBP6	1.21680837	3.77237041	5.70628174	2.25E-07	3.19E-06	6.72977265	RANBP6	assignmentsChromaffin_cells.Chrom_SOX2_statusHigh
assignmentsChromaffin_cells.Chrom_SOX2_statusHigh.CDC34	1.71054202	3.54030004	5.70208259	2.29E-07	3.22E-06	6.72200461	CDC34	assignmentsChromaffin_cells.Chrom_SOX2_statusHigh
assignmentsChromaffin_cells.Chrom_SOX2_statusHigh.DANCR	1.98993738	4.33824419	5.7109859	2.20E-07	3.13E-06	6.71787854	DANCR	assignmentsChromaffin_cells.Chrom_SOX2_statusHigh
assignmentsChromaffin_cells.Chrom_SOX2_statusHigh.PMM1	1.82203294	3.05550105	5.69745308	2.33E-07	3.27E-06	6.71150425	PMM1	assignmentsChromaffin_cells.Chrom_SOX2_statusHigh
assignmentsChromaffin_cells.Chrom_SOX2_statusHigh.TANGO6	-1.5282671	5.87380335	-5.7032804	2.27E-07	3.21E-06	6.70115522	TANGO6	assignmentsChromaffin_cells.Chrom_SOX2_statusHigh
assignmentsChromaffin_cells.Chrom_SOX2_statusHigh.KANK1	-2.2979804	5.56978781	-5.7031901	2.28E-07	3.22E-06	6.70045308	KANK1	assignmentsChromaffin_cells.Chrom_SOX2_statusHigh
assignmentsChromaffin_cells.Chrom_SOX2_statusHigh.EHMT1	-1.4017907	7.45458935	-5.7153192	2.17E-07	3.08E-06	6.69328539	EHMT1	assignmentsChromaffin_cells.Chrom_SOX2_statusHigh
assignmentsChromaffin_cells.Chrom_SOX2_statusHigh.ABT1	1.86944189	2.79654087	5.68510783	2.45E-07	3.40E-06	6.68623991	ABT1	assignmentsChromaffin_cells.Chrom_SOX2_statusHigh
assignmentsChromaffin_cells.Chrom_SOX2_statusHigh.MAP1LC3A	3.70994158	3.48873688	5.68250148	2.49E-07	3.45E-06	6.68581312	MAP1LC3A	assignmentsChromaffin_cells.Chrom_SOX2_statusHigh
assignmentsChromaffin_cells.Chrom_SOX2_statusHigh.ZNF518A	-1.2851459	6.96128891	-5.7158504	2.16E-07	3.08E-06	6.68409597	ZNF518A	assignmentsChromaffin_cells.Chrom_SOX2_statusHigh
assignmentsChromaffin_cells.Chrom_SOX2_statusHigh.TPBGL	4.1867813	0.45027628	5.66823245	2.64E-07	3.63E-06	6.68408923	TPBGL	assignmentsChromaffin_cells.Chrom_SOX2_statusHigh
assignmentsChromaffin_cells.Chrom_SOX2_statusHigh.ATXN1	-1.1280296	9.07081881	-5.7278574	2.06E-07	2.96E-06	6.68333115	ATXN1	assignmentsChromaffin_cells.Chrom_SOX2_statusHigh
assignmentsChromaffin_cells.Chrom_SOX2_statusHigh.VTI1A	-1.015931	8.21264246	-5.7202719	2.12E-07	3.04E-06	6.67947839	VTI1A	assignmentsChromaffin_cells.Chrom_SOX2_statusHigh
assignmentsChromaffin_cells.Chrom_SOX2_statusHigh.ATP5F1E	3.41645238	6.42026527	5.7190008	2.15E-07	3.06E-06	6.66965578	ATP5F1E	assignmentsChromaffin_cells.Chrom_SOX2_statusHigh
assignmentsChromaffin_cells.Chrom_SOX2_statusHigh.PURG	-3.5604175	2.93297948	-5.664731	2.67E-07	3.66E-06	6.66541496	PURG	assignmentsChromaffin_cells.Chrom_SOX2_statusHigh
assignmentsChromaffin_cells.Chrom_SOX2_statusHigh.CFAP20	1.78222946	4.1161946	5.69646864	2.34E-07	3.28E-06	6.66483134	CFAP20	assignmentsChromaffin_cells.Chrom_SOX2_statusHigh
assignmentsChromaffin_cells.Chrom_SOX2_statusHigh.TMEM109	2.42012373	3.46499993	5.68063045	2.50E-07	3.45E-06	6.65719954	TMEM109	assignmentsChromaffin_cells.Chrom_SOX2_statusHigh
assignmentsChromaffin_cells.Chrom_SOX2_statusHigh.DYNLT1	1.69118129	5.68424317	5.69739244	2.33E-07	3.27E-06	6.65058712	DYNLT1	assignmentsChromaffin_cells.Chrom_SOX2_statusHigh
assignmentsChromaffin_cells.Chrom_SOX2_statusHigh.FBL	2.20751769	3.91714351	5.68552563	2.45E-07	3.40E-06	6.64983979	FBL	assignmentsChromaffin_cells.Chrom_SOX2_statusHigh
assignmentsChromaffin_cells.Chrom_SOX2_statusHigh.RAB4A	1.2328836	4.91122317	5.69262139	2.38E-07	3.32E-06	6.64861381	RAB4A	assignmentsChromaffin_cells.Chrom_SOX2_statusHigh
assignmentsChromaffin_cells.Chrom_SOX2_statusHigh.EMC10	1.57375965	5.09379656	5.69810599	2.32E-07	3.27E-06	6.64675302	EMC10	assignmentsChromaffin_cells.Chrom_SOX2_statusHigh
assignmentsChromaffin_cells.Chrom_SOX2_statusHigh.RNF167	1.62918482	3.93362073	5.6863426	2.44E-07	3.39E-06	6.64667003	RNF167	assignmentsChromaffin_cells.Chrom_SOX2_statusHigh
assignmentsChromaffin_cells.Chrom_SOX2_statusHigh.RPN1	1.51254641	5.31446349	5.69569565	2.35E-07	3.29E-06	6.64462874	RPN1	assignmentsChromaffin_cells.Chrom_SOX2_statusHigh
assignmentsChromaffin_cells.Chrom_SOX2_statusHigh.C1QL1	4.78740087	0.35601904	5.65864683	2.74E-07	3.73E-06	6.6383872	C1QL1	assignmentsChromaffin_cells.Chrom_SOX2_statusHigh
assignmentsChromaffin_cells.Chrom_SOX2_statusHigh.ZNF524	2.23571763	2.33648048	5.66693542	2.64E-07	3.63E-06	6.63770162	ZNF524	assignmentsChromaffin_cells.Chrom_SOX2_statusHigh

assignmentsChromaffin_cells.Chrom_SOX2_statusHigh.PRKAR1A	1.29203024	6.72121608	5.70659591	2.24E-07	3.18E-06	6.63609326	PRKAR1A	assignmentsChromaffin_cells.Chrom_SOX2_statusHigh
assignmentsChromaffin_cells.Chrom_SOX2_statusHigh.STRN3	-1.3224875	7.6017766	-5.7049154	2.26E-07	3.20E-06	6.63370979	STRN3	assignmentsChromaffin_cells.Chrom_SOX2_statusHigh
assignmentsChromaffin_cells.Chrom_SOX2_statusHigh.DENND1A	-1.3315441	7.75811464	-5.7029857	2.28E-07	3.21E-06	6.63171067	DENND1A	assignmentsChromaffin_cells.Chrom_SOX2_statusHigh
assignmentsChromaffin_cells.Chrom_SOX2_statusHigh.SNHG11	1.50849491	2.05539653	5.66351777	2.67E-07	3.66E-06	6.62469471	SNHG11	assignmentsChromaffin_cells.Chrom_SOX2_statusHigh
assignmentsChromaffin_cells.Chrom_SOX2_statusHigh.PSME2	1.80943679	4.74462645	5.68616498	2.44E-07	3.39E-06	6.61620188	PSME2	assignmentsChromaffin_cells.Chrom_SOX2_statusHigh
assignmentsChromaffin_cells.Chrom_SOX2_statusHigh.VBP1	1.48584903	4.11696239	5.68120494	2.49E-07	3.45E-06	6.61520435	VBP1	assignmentsChromaffin_cells.Chrom_SOX2_statusHigh
assignmentsChromaffin_cells.Chrom_SOX2_statusHigh.CCT6A	1.32126155	6.04803838	5.69619221	2.34E-07	3.28E-06	6.61070578	CCT6A	assignmentsChromaffin_cells.Chrom_SOX2_statusHigh
assignmentsChromaffin_cells.Chrom_SOX2_statusHigh.GSTO1	2.0405998	5.1244606	5.68259081	2.47E-07	3.43E-06	6.60803929	GSTO1	assignmentsChromaffin_cells.Chrom_SOX2_statusHigh
assignmentsChromaffin_cells.Chrom_SOX2_statusHigh.YWHAH	1.83667173	6.03216101	5.6938805	2.36E-07	3.31E-06	6.60584001	YWHAH	assignmentsChromaffin_cells.Chrom_SOX2_statusHigh
assignmentsChromaffin_cells.Chrom_SOX2_statusHigh.CD81	2.84460786	5.94038884	5.69164676	2.40E-07	3.35E-06	6.60487644	CD81	assignmentsChromaffin_cells.Chrom_SOX2_statusHigh
assignmentsChromaffin_cells.Chrom_SOX2_statusHigh.CYB561D2	1.51500752	3.33563694	5.66685327	2.64E-07	3.63E-06	6.60331387	CYB561D2	assignmentsChromaffin_cells.Chrom_SOX2_statusHigh
assignmentsChromaffin_cells.Chrom_SOX2_statusHigh.PRMT1	1.65259448	5.1106268	5.68877253	2.41E-07	3.36E-06	6.60272667	PRMT1	assignmentsChromaffin_cells.Chrom_SOX2_statusHigh
assignmentsChromaffin_cells.Chrom_SOX2_statusHigh.ECEL1	7.6294732	-0.6525318	5.67366729	2.58E-07	3.56E-06	6.59918226	ECEL1	assignmentsChromaffin_cells.Chrom_SOX2_statusHigh
assignmentsChromaffin_cells.Chrom_SOX2_statusHigh.CPS1	-2.8238769	3.24426799	-5.6554841	2.77E-07	3.76E-06	6.59596727	CPS1	assignmentsChromaffin_cells.Chrom_SOX2_statusHigh
assignmentsChromaffin_cells.Chrom_SOX2_statusHigh.ABI2	-0.982827	7.01861777	-5.6919914	2.38E-07	3.33E-06	6.58946272	ABI2	assignmentsChromaffin_cells.Chrom_SOX2_statusHigh
assignmentsChromaffin_cells.Chrom_SOX2_statusHigh.PDRG1	1.17091846	3.02350725	5.65976192	2.71E-07	3.70E-06	6.57625721	PDRG1	assignmentsChromaffin_cells.Chrom_SOX2_statusHigh
assignmentsChromaffin_cells.Chrom_SOX2_statusHigh.ATE1	-0.9996793	7.02319085	-5.6854481	2.45E-07	3.40E-06	6.56830163	ATE1	assignmentsChromaffin_cells.Chrom_SOX2_statusHigh
assignmentsChromaffin_cells.Chrom_SOX2_statusHigh.RPL27	3.2512498	5.86542695	5.68628697	2.45E-07	3.40E-06	6.56559078	RPL27	assignmentsChromaffin_cells.Chrom_SOX2_statusHigh
assignmentsChromaffin_cells.Chrom_SOX2_statusHigh.TP53TG1	2.07954941	2.85817216	5.65302031	2.79E-07	3.78E-06	6.5462542	TP53TG1	assignmentsChromaffin_cells.Chrom_SOX2_statusHigh
assignmentsChromaffin_cells.Chrom_SOX2_statusHigh.DBI	2.85364211	4.17746505	5.65850262	2.74E-07	3.73E-06	6.54306923	DBI	assignmentsChromaffin_cells.Chrom_SOX2_statusHigh
assignmentsChromaffin_cells.Chrom_SOX2_statusHigh.DDIT3	2.13443416	5.08787992	5.67682886	2.54E-07	3.51E-06	6.541443	DDIT3	assignmentsChromaffin_cells.Chrom_SOX2_statusHigh
assignmentsChromaffin_cells.Chrom_SOX2_statusHigh.SLC10A7	-1.1170573	6.45076726	-5.6682582	2.62E-07	3.61E-06	6.54056499	SLC10A7	assignmentsChromaffin_cells.Chrom_SOX2_statusHigh
assignmentsChromaffin_cells.Chrom_SOX2_statusHigh.TMEM18	1.64415028	3.88776661	5.66066768	2.70E-07	3.69E-06	6.53826329	TMEM18	assignmentsChromaffin_cells.Chrom_SOX2_statusHigh
assignmentsChromaffin_cells.Chrom_SOX2_statusHigh.DUSP3	1.14646079	4.60825119	5.66382999	2.67E-07	3.66E-06	6.53796639	DUSP3	assignmentsChromaffin_cells.Chrom_SOX2_statusHigh
assignmentsChromaffin_cells.Chrom_SOX2_statusHigh.B4GALT7	1.60960894	2.86081571	5.64597707	2.87E-07	3.89E-06	6.53776766	B4GALT7	assignmentsChromaffin_cells.Chrom_SOX2_statusHigh
assignmentsChromaffin_cells.Chrom_SOX2_statusHigh.AFG1L	-1.1079459	5.24200488	-5.6584768	2.73E-07	3.72E-06	6.53736635	AFG1L	assignmentsChromaffin_cells.Chrom_SOX2_statusHigh
assignmentsChromaffin_cells.Chrom_SOX2_statusHigh.WBP1	1.69758844	4.04111702	5.66098855	2.70E-07	3.69E-06	6.53315296	WBP1	assignmentsChromaffin_cells.Chrom_SOX2_statusHigh
assignmentsChromaffin_cells.Chrom_SOX2_statusHigh.CENPS	1.63566116	1.77515287	5.63620039	2.99E-07	4.03E-06	6.53288499	CENPS	assignmentsChromaffin_cells.Chrom_SOX2_statusHigh
assignmentsChromaffin_cells.Chrom_SOX2_statusHigh.RNF111	-0.9891425	6.98488688	-5.6715585	2.59E-07	3.57E-06	6.52285593	RNF111	assignmentsChromaffin_cells.Chrom_SOX2_statusHigh
assignmentsChromaffin_cells.Chrom_SOX2_statusHigh.ZBTB33	1.47187228	2.37977241	5.63589144	2.99E-07	4.03E-06	6.51801189	ZBTB33	assignmentsChromaffin_cells.Chrom_SOX2_statusHigh
assignmentsChromaffin_cells.Chrom_SOX2_statusHigh.ARNT	-0.9740683	6.33469755	-5.6640593	2.67E-07	3.66E-06	6.49866048	ARNT	assignmentsChromaffin_cells.Chrom_SOX2_statusHigh
assignmentsChromaffin_cells.Chrom_SOX2_statusHigh.TMEM102	3.55464582	-0.2909442	5.66601659	2.65E-07	3.63E-06	6.49529264	TMEM102	assignmentsChromaffin_cells.Chrom_SOX2_statusHigh
assignmentsChromaffin_cells.Chrom_SOX2_statusHigh.PNMT	5.64077727	-0.3424619	5.64378219	2.91E-07	3.94E-06	6.49499456	PNMT	assignmentsChromaffin_cells.Chrom_SOX2_statusHigh
assignmentsChromaffin_cells.Chrom_SOX2_statusHigh.GNB2	1.84379511	5.28833542	5.66002891	2.71E-07	3.70E-06	6.49128409	GNB2	assignmentsChromaffin_cells.Chrom_SOX2_statusHigh
assignmentsChromaffin_cells.Chrom_SOX2_statusHigh.TMEM199	1.25505257	3.40805529	5.63971129	2.95E-07	3.98E-06	6.48570111	TMEM199	assignmentsChromaffin_cells.Chrom_SOX2_statusHigh
assignmentsChromaffin_cells.Chrom_SOX2_statusHigh.IMP4	1.74509034	3.20661272	5.63510985	3.00E-07	4.04E-06	6.48219396	IMP4	assignmentsChromaffin_cells.Chrom_SOX2_statusHigh
assignmentsChromaffin_cells.Chrom_SOX2_statusHigh.LDOC1	2.31380164	2.85769059	5.64185738	2.92E-07	3.95E-06	6.48071622	LDOC1	assignmentsChromaffin_cells.Chrom_SOX2_statusHigh
assignmentsChromaffin_cells.Chrom_SOX2_statusHigh.PAFAH1B3	1.92028607	2.3965976	5.62892375	3.08E-07	4.13E-06	6.46519664	PAFAH1B3	assignmentsChromaffin_cells.Chrom_SOX2_statusHigh
assignmentsChromaffin_cells.Chrom_SOX2_statusHigh.RPL18	3.72922434	7.18683441	5.66256729	2.70E-07	3.69E-06	6.42183248	RPL18	assignmentsChromaffin_cells.Chrom_SOX2_statusHigh
assignmentsChromaffin_cells.Chrom_SOX2_statusHigh.EFHC2	3.25493401	0.54282883	5.60262627	3.42E-07	4.52E-06	6.41991273	EFHC2	assignmentsChromaffin_cells.Chrom_SOX2_statusHigh
assignmentsChromaffin_cells.Chrom_SOX2_statusHigh.NTHL1	2.18389158	2.15578106	5.60889052	3.34E-07	4.42E-06	6.41762556	NTHL1	assignmentsChromaffin_cells.Chrom_SOX2_statusHigh
assignmentsChromaffin_cells.Chrom_SOX2_statusHigh.MCM4	1.56494725	2.92840985	5.61875724	3.21E-07	4.29E-06	6.41308684	MCM4	assignmentsChromaffin_cells.Chrom_SOX2_statusHigh
assignmentsChromaffin_cells.Chrom_SOX2_statusHigh.PDS5B	-1.2222467	8.16281334	-5.6549191	2.77E-07	3.76E-06	6.41016188	PDS5B	assignmentsChromaffin_cells.Chrom_SOX2_statusHigh
assignmentsChromaffin_cells.Chrom_SOX2_statusHigh.PRIM2	-1.1536098	6.27975298	-5.631483	3.05E-07	4.09E-06	6.40948281	PRIM2	assignmentsChromaffin_cells.Chrom_SOX2_statusHigh
assignmentsChromaffin_cells.Chrom_SOX2_statusHigh.TIMM29	1.28882966	3.05150315	5.6161524	3.24E-07	4.32E-06	6.40692823	TIMM29	assignmentsChromaffin_cells.Chrom_SOX2_statusHigh
assignmentsChromaffin_cells.Chrom_SOX2_statusHigh.LINC01003	1.88201759	1.90712317	5.61036137	3.32E-07	4.39E-06	6.40689032	LINC01003	assignmentsChromaffin_cells.Chrom_SOX2_statusHigh
assignmentsChromaffin_cells.Chrom_SOX2_statusHigh.DMTF1	-1.0626539	6.97109719	-5.6416236	2.92E-07	3.95E-06	6.40655748	DMTF1	assignmentsChromaffin_cells.Chrom_SOX2_statusHigh
assignmentsChromaffin_cells.Chrom_SOX2_statusHigh.CYP2U1	1.16408269	3.67148364	5.62160895	3.17E-07	4.25E-06	6.39361175	CYP2U1	assignmentsChromaffin_cells.Chrom_SOX2_statusHigh
assignmentsChromaffin_cells.Chrom_SOX2_statusHigh.TCEAL5	5.56660292	0.39686707	5.59171933	3.60E-07	4.71E-06	6.38959428	TCEAL5	assignmentsChromaffin_cells.Chrom_SOX2_statusHigh
assignmentsChromaffin_cells.Chrom_SOX2_statusHigh.ZNF135	-2.6388786	4.24730211	-5.6024161	3.43E-07	4.53E-06	6.38060457	ZNF135	assignmentsChromaffin_cells.Chrom_SOX2_statusHigh
assignmentsChromaffin_cells.Chrom_SOX2_statusHigh.LARP4B	-0.9859612	7.2353838	-5.6368175	2.98E-07	4.02E-06	6.37877844	LARP4B	assignmentsChromaffin_cells.Chrom_SOX2_statusHigh
assignmentsChromaffin_cells.Chrom_SOX2_statusHigh.PRELID3B	4.17828612	2.39413564	5.59187172	3.60E-07	4.71E-06	6.37603601	PRELID3B	assignmentsChromaffin_cells.Chrom_SOX2_statusHigh
assignmentsChromaffin_cells.Chrom_SOX2_statusHigh.PTER	4.45384649	2.604673	5.58913903	3.62E-07	4.73E-06	6.36241929	PTER	assignmentsChromaffin_cells.Chrom_SOX2_statusHigh
assignmentsChromaffin_cells.Chrom_SOX2_statusHigh.RANBP1	1.96642172	4.51174485	5.62057514	3.18E-07	4.26E-06	6.35959237	RANBP1	assignmentsChromaffin_cells.Chrom_SOX2_statusHigh
assignmentsChromaffin_cells.Chrom_SOX2_statusHigh.CDON	-2.5034197	4.70138859	-5.6135922	3.28E-07	4.37E-06	6.35747758	CDON	assignmentsChromaffin_cells.Chrom_SOX2_statusHigh
assignmentsChromaffin_cells.Chrom_SOX2_statusHigh.TP53I13	1.85301674	3.82705586	5.61134565	3.30E-07	4.38E-06	6.35035409	TP53I13	assignmentsChromaffin_cells.Chrom_SOX2_statusHigh
assignmentsChromaffin_cells.Chrom_SOX2_statusHigh.ILKAP	-0.7958058	6.11359242	-5.6206429	3.18E-07	4.26E-06	6.34492171	ILKAP	assignmentsChromaffin_cells.Chrom_SOX2_statusHigh
assignmentsChromaffin_cells.Chrom_SOX2_statusHigh.CNBP	1.74116709	6.1846645	5.62620136	3.11E-07	4.18E-06	6.34419242	CNBP	assignmentsChromaffin_cells.Chrom_SOX2_statusHigh
assignmentsChromaffin_cells.Chrom_SOX2_statusHigh.PSMC3	1.83949875	3.79950684	5.60352534	3.41E-07	4.51E-06	6.34302559	PSMC3	assignmentsChromaffin_cells.Chrom_SOX2_statusHigh
assignmentsChromaffin_cells.Chrom_SOX2_statusHigh.SPINT2	4.51121297	2.47216483	5.5837213	3.72E-07	4.83E-06	6.33818873	SPINT2	assignmentsChromaffin_cells.Chrom_SOX2_statusHigh
assignmentsChromaffin_cells.Chrom_SOX2_statusHigh.PNPO	1.4018824	2.8374563	5.59428027	3.54E-07	4.66E-06	6.32786247	PNPO	assignmentsChromaffin_cells.Chrom_SOX2_statusHigh
assignmentsChromaffin_cells.Chrom_SOX2_statusHigh.PRDM10	-1.0541637	4.68093568	-5.5984494	3.48E-07	4.58E-06	6.32161207	PRDM10	assignmentsChromaffin_cells.Chrom_SOX2_statusHigh
assignmentsChromaffin_cells.Chrom_SOX2_statusHigh.FKBP11	1.82434173	3.91688481	5.60264069	3.42E-07	4.52E-06	6.31676994	FKBP11	assignmentsChromaffin_cells.Chrom_SOX2_statusHigh
assignmentsChromaffin_cells.Chrom_SOX2_statusHigh.TFCP2	-1.4549315	6.86303176	-5.6173665	3.22E-07	4.31E-06	6.31503552	TFCP2	assignmentsChromaffin_cells.Chrom_SOX2_statusHigh

assignmentsChromaffin_cells.Chrom_SOX2_statusHigh.LYPLA2	1.57872721	3.01987293	5.58424365	3.69E-07	4.80E-06	6.31190787	LYPLA2	assignmentsChromaffin_cells.Chrom_SOX2_statusHigh
assignmentsChromaffin_cells.Chrom_SOX2_statusHigh.NPDC1	1.3518251	5.54774698	5.61762296	3.22E-07	4.31E-06	6.29711389	NPDC1	assignmentsChromaffin_cells.Chrom_SOX2_statusHigh
assignmentsChromaffin_cells.Chrom_SOX2_statusHigh.GMDS	-1.2312289	7.72254347	-5.6159084	3.24E-07	4.32E-06	6.28792428	GMDS	assignmentsChromaffin_cells.Chrom_SOX2_statusHigh
assignmentsChromaffin_cells.Chrom_SOX2_statusHigh.SPIRE1	-1.3901084	7.04248486	-5.61191	3.30E-07	4.38E-06	6.28707075	SPIRE1	assignmentsChromaffin_cells.Chrom_SOX2_statusHigh
assignmentsChromaffin_cells.Chrom_SOX2_statusHigh.ARPC3	1.98820588	6.22437215	5.61219554	3.30E-07	4.38E-06	6.28504781	ARPC3	assignmentsChromaffin_cells.Chrom_SOX2_statusHigh
assignmentsChromaffin_cells.Chrom_SOX2_statusHigh.PEAR1	2.89064528	1.49024583	5.56858054	3.93E-07	5.08E-06	6.28441115	PEAR1	assignmentsChromaffin_cells.Chrom_SOX2_statusHigh
assignmentsChromaffin_cells.Chrom_SOX2_statusHigh.THAP11	2.17657471	1.93971211	5.57298701	3.86E-07	5.00E-06	6.28411866	THAP11	assignmentsChromaffin_cells.Chrom_SOX2_statusHigh
assignmentsChromaffin_cells.Chrom_SOX2_statusHigh.SLU7	1.58424941	4.35622378	5.59340355	3.55E-07	4.67E-06	6.2716843	SLU7	assignmentsChromaffin_cells.Chrom_SOX2_statusHigh
assignmentsChromaffin_cells.Chrom_SOX2_statusHigh.GLMP	1.80617298	3.3664021	5.58571293	3.67E-07	4.78E-06	6.26972371	GLMP	assignmentsChromaffin_cells.Chrom_SOX2_statusHigh
assignmentsChromaffin_cells.Chrom_SOX2_statusHigh.RPL38	3.09857448	6.19649406	5.61243	3.31E-07	4.39E-06	6.26144199	RPL38	assignmentsChromaffin_cells.Chrom_SOX2_statusHigh
assignmentsChromaffin_cells.Chrom_SOX2_statusHigh.TAF1A-AS1	2.07447002	0.50715178	5.55854671	4.09E-07	5.23E-06	6.26126002	TAF1A-AS1	assignmentsChromaffin_cells.Chrom_SOX2_statusHigh
assignmentsChromaffin_cells.Chrom_SOX2_statusHigh.ZNRD2	2.10936995	2.75365407	5.57312764	3.86E-07	5.00E-06	6.25630701	ZNRD2	assignmentsChromaffin_cells.Chrom_SOX2_statusHigh
assignmentsChromaffin_cells.Chrom_SOX2_statusHigh.NDUFA4L2	3.13959031	5.74943117	5.61403814	3.29E-07	4.38E-06	6.25408336	NDUFA4L2	assignmentsChromaffin_cells.Chrom_SOX2_statusHigh
assignmentsChromaffin_cells.Chrom_SOX2_statusHigh.FBXO5	1.39337405	2.29796955	5.56450567	3.99E-07	5.14E-06	6.24683288	FBXO5	assignmentsChromaffin_cells.Chrom_SOX2_statusHigh
assignmentsChromaffin_cells.Chrom_SOX2_statusHigh.SMDT1	2.46700599	4.33482038	5.59138647	3.59E-07	4.71E-06	6.23669492	SMDT1	assignmentsChromaffin_cells.Chrom_SOX2_statusHigh
assignmentsChromaffin_cells.Chrom_SOX2_statusHigh.RAMAC	2.64858595	1.70406274	5.56221396	4.04E-07	5.19E-06	6.23641184	RAMAC	assignmentsChromaffin_cells.Chrom_SOX2_statusHigh
assignmentsChromaffin_cells.Chrom_SOX2_statusHigh.DOLK	1.88622188	1.29701029	5.55500509	4.15E-07	5.30E-06	6.23532218	DOLK	assignmentsChromaffin_cells.Chrom_SOX2_statusHigh
assignmentsChromaffin_cells.Chrom_SOX2_statusHigh.OAS3	3.22030834	2.46846897	5.55041711	4.23E-07	5.39E-06	6.23455424	OAS3	assignmentsChromaffin_cells.Chrom_SOX2_statusHigh
assignmentsChromaffin_cells.Chrom_SOX2_statusHigh.SPACA9	1.58237731	1.62770502	5.561475	4.04E-07	5.20E-06	6.23388117	SPACA9	assignmentsChromaffin_cells.Chrom_SOX2_statusHigh
assignmentsChromaffin_cells.Chrom_SOX2_statusHigh.PPP1CA	1.95302903	3.96271039	5.57692014	3.80E-07	4.93E-06	6.23191804	PPP1CA	assignmentsChromaffin_cells.Chrom_SOX2_statusHigh
assignmentsChromaffin_cells.Chrom_SOX2_statusHigh.EXOC6B	-1.0522625	8.50435814	-5.6121612	3.29E-07	4.38E-06	6.22859803	EXOC6B	assignmentsChromaffin_cells.Chrom_SOX2_statusHigh
assignmentsChromaffin_cells.Chrom_SOX2_statusHigh.WNT6	3.27875975	0.50012996	5.5482203	4.27E-07	5.42E-06	6.22829062	WNT6	assignmentsChromaffin_cells.Chrom_SOX2_statusHigh
assignmentsChromaffin_cells.Chrom_SOX2_statusHigh.ATXN7L1	-1.6029913	6.91868445	-5.5900726	3.60E-07	4.71E-06	6.22029285	ATXN7L1	assignmentsChromaffin_cells.Chrom_SOX2_statusHigh
assignmentsChromaffin_cells.Chrom_SOX2_statusHigh.TRAPPC2L	2.18948378	3.20298257	5.57136801	3.88E-07	5.02E-06	6.21942698	TRAPPC2L	assignmentsChromaffin_cells.Chrom_SOX2_statusHigh
assignmentsChromaffin_cells.Chrom_SOX2_statusHigh.ZNF354C	-2.0081629	3.78481227	-5.5586594	4.09E-07	5.23E-06	6.21476493	ZNF354C	assignmentsChromaffin_cells.Chrom_SOX2_statusHigh
assignmentsChromaffin_cells.Chrom_SOX2_statusHigh.CHRNB1	1.98396787	1.34147328	5.54786494	4.27E-07	5.42E-06	6.2133719	CHRNB1	assignmentsChromaffin_cells.Chrom_SOX2_statusHigh
assignmentsChromaffin_cells.Chrom_SOX2_statusHigh.NXT1	2.37233011	3.16904042	5.56699123	3.96E-07	5.11E-06	6.21259704	NXT1	assignmentsChromaffin_cells.Chrom_SOX2_statusHigh
assignmentsChromaffin_cells.Chrom_SOX2_statusHigh.SMIM10L1	1.55202639	4.29550709	5.58099172	3.74E-07	4.85E-06	6.2079972	SMIM10L1	assignmentsChromaffin_cells.Chrom_SOX2_statusHigh
assignmentsChromaffin_cells.Chrom_SOX2_statusHigh.ORC4	-0.7643977	6.15580005	-5.588838	3.62E-07	4.73E-06	6.20324113	ORC4	assignmentsChromaffin_cells.Chrom_SOX2_statusHigh
assignmentsChromaffin_cells.Chrom_SOX2_statusHigh.PPP4R3A	-1.2466675	6.89632016	-5.5868579	3.65E-07	4.76E-06	6.20063823	PPP4R3A	assignmentsChromaffin_cells.Chrom_SOX2_statusHigh
assignmentsChromaffin_cells.Chrom_SOX2_statusHigh.RPS9	3.22595533	7.07712593	5.60179291	3.46E-07	4.55E-06	6.19911584	RPS9	assignmentsChromaffin_cells.Chrom_SOX2_statusHigh
assignmentsChromaffin_cells.Chrom_SOX2_statusHigh.NT5E	2.02592723	3.22599206	5.56706415	3.95E-07	5.10E-06	6.19910465	NT5E	assignmentsChromaffin_cells.Chrom_SOX2_statusHigh
assignmentsChromaffin_cells.Chrom_SOX2_statusHigh.NSD2	-1.1509314	6.66026301	-5.5876947	3.64E-07	4.75E-06	6.18752901	NSD2	assignmentsChromaffin_cells.Chrom_SOX2_statusHigh
assignmentsChromaffin_cells.Chrom_SOX2_statusHigh.NYNRIN	3.89142882	1.16404568	5.55027867	4.23E-07	5.39E-06	6.18563841	NYNRIN	assignmentsChromaffin_cells.Chrom_SOX2_statusHigh
assignmentsChromaffin_cells.Chrom_SOX2_statusHigh.AGTRAP	2.19354684	2.79135321	5.53932976	4.42E-07	5.57E-06	6.18270061	AGTRAP	assignmentsChromaffin_cells.Chrom_SOX2_statusHigh
assignmentsChromaffin_cells.Chrom_SOX2_statusHigh.SNHG29	2.69045771	6.37825049	5.59253167	3.59E-07	4.71E-06	6.17006838	SNHG29	assignmentsChromaffin_cells.Chrom_SOX2_statusHigh
assignmentsChromaffin_cells.Chrom_SOX2_statusHigh.CD68	3.29286234	2.22806049	5.5349852	4.51E-07	5.67E-06	6.16202396	CD68	assignmentsChromaffin_cells.Chrom_SOX2_statusHigh
assignmentsChromaffin_cells.Chrom_SOX2_statusHigh.FKBP1B	3.72956779	1.11273103	5.53196071	4.58E-07	5.74E-06	6.14647428	FKBP1B	assignmentsChromaffin_cells.Chrom_SOX2_statusHigh
assignmentsChromaffin_cells.Chrom_SOX2_statusHigh.ACTB	2.96683171	8.1223656	5.59357055	3.57E-07	4.69E-06	6.14273442	ACTB	assignmentsChromaffin_cells.Chrom_SOX2_statusHigh
assignmentsChromaffin_cells.Chrom_SOX2_statusHigh.SNHG22	-1.7152965	4.10438632	-5.5452332	4.32E-07	5.46E-06	6.14269824	SNHG22	assignmentsChromaffin_cells.Chrom_SOX2_statusHigh
assignmentsChromaffin_cells.Chrom_SOX2_statusHigh.BDKRB2	3.97082765	2.43157653	5.53586507	4.51E-07	5.67E-06	6.13820644	BDKRB2	assignmentsChromaffin_cells.Chrom_SOX2_statusHigh
assignmentsChromaffin_cells.Chrom_SOX2_statusHigh.NPM1	2.15514993	6.96864694	5.58540808	3.68E-07	4.79E-06	6.13545636	NPM1	assignmentsChromaffin_cells.Chrom_SOX2_statusHigh
assignmentsChromaffin_cells.Chrom_SOX2_statusHigh.RABGGTB	1.20191125	4.83272608	5.55890412	4.09E-07	5.23E-06	6.13280053	RABGGTB	assignmentsChromaffin_cells.Chrom_SOX2_statusHigh
assignmentsChromaffin_cells.Chrom_SOX2_statusHigh.LYRM4	-0.9848357	6.03780898	-5.5653682	3.98E-07	5.13E-06	6.13021884	LYRM4	assignmentsChromaffin_cells.Chrom_SOX2_statusHigh
assignmentsChromaffin_cells.Chrom_SOX2_statusHigh.ERGIC3	1.25403417	4.99851395	5.55989501	4.07E-07	5.22E-06	6.11425256	ERGIC3	assignmentsChromaffin_cells.Chrom_SOX2_statusHigh
assignmentsChromaffin_cells.Chrom_SOX2_statusHigh.CAB39	-1.1560936	6.97796004	-5.5606937	4.06E-07	5.20E-06	6.11424463	CAB39	assignmentsChromaffin_cells.Chrom_SOX2_statusHigh
assignmentsChromaffin_cells.Chrom_SOX2_statusHigh.CMTR1	-1.1312006	4.73281474	-5.5468316	4.29E-07	5.44E-06	6.11393877	CMTR1	assignmentsChromaffin_cells.Chrom_SOX2_statusHigh
assignmentsChromaffin_cells.Chrom_SOX2_statusHigh.YDJC	2.24060467	1.82695704	5.52821702	4.62E-07	5.78E-06	6.10781309	YDJC	assignmentsChromaffin_cells.Chrom_SOX2_statusHigh
assignmentsChromaffin_cells.Chrom_SOX2_statusHigh.KLF10	2.51877925	4.60767544	5.55032727	4.24E-07	5.40E-06	6.09932855	KLF10	assignmentsChromaffin_cells.Chrom_SOX2_statusHigh
assignmentsChromaffin_cells.Chrom_SOX2_statusHigh.KBTBD7	1.53762809	2.3235156	5.53218824	4.55E-07	5.71E-06	6.09696241	KBTBD7	assignmentsChromaffin_cells.Chrom_SOX2_statusHigh
assignmentsChromaffin_cells.Chrom_SOX2_statusHigh.HERC1	-1.0282455	8.4615292	-5.5731008	3.86E-07	5.00E-06	6.09220313	HERC1	assignmentsChromaffin_cells.Chrom_SOX2_statusHigh
assignmentsChromaffin_cells.Chrom_SOX2_statusHigh.TGOLN2	1.29151324	6.30237764	5.5612008	4.05E-07	5.20E-06	6.0891234	TGOLN2	assignmentsChromaffin_cells.Chrom_SOX2_statusHigh
assignmentsChromaffin_cells.Chrom_SOX2_statusHigh.ATP5MG	2.26656074	6.00849044	5.56388919	4.02E-07	5.17E-06	6.08044988	ATP5MG	assignmentsChromaffin_cells.Chrom_SOX2_statusHigh
assignmentsChromaffin_cells.Chrom_SOX2_statusHigh.FOXO6	4.15344419	0.38294249	5.51101618	4.97E-07	6.14E-06	6.07864153	FOXO6	assignmentsChromaffin_cells.Chrom_SOX2_statusHigh
assignmentsChromaffin_cells.Chrom_SOX2_statusHigh.ATP5MC3	2.28905283	5.43190337	5.56048865	4.07E-07	5.22E-06	6.07681044	ATP5MC3	assignmentsChromaffin_cells.Chrom_SOX2_statusHigh
assignmentsChromaffin_cells.Chrom_SOX2_statusHigh.C12orf57	3.07106635	4.57234697	5.54784732	4.30E-07	5.44E-06	6.07640125	C12orf57	assignmentsChromaffin_cells.Chrom_SOX2_statusHigh
assignmentsChromaffin_cells.Chrom_SOX2_statusHigh.ZNF688	1.57898893	2.65249937	5.52472802	4.69E-07	5.83E-06	6.07504464	ZNF688	assignmentsChromaffin_cells.Chrom_SOX2_statusHigh
assignmentsChromaffin_cells.Chrom_SOX2_statusHigh.MSRB2	1.47448949	4.5638033	5.54783229	4.27E-07	5.42E-06	6.07148906	MSRB2	assignmentsChromaffin_cells.Chrom_SOX2_statusHigh
assignmentsChromaffin_cells.Chrom_SOX2_statusHigh.FRMD4A	-3.1921888	7.65821674	-5.5295241	4.63E-07	5.78E-06	6.05979739	FRMD4A	assignmentsChromaffin_cells.Chrom_SOX2_statusHigh
assignmentsChromaffin_cells.Chrom_SOX2_statusHigh.MOSPD3	1.82309453	2.42264373	5.52362919	4.71E-07	5.86E-06	6.05579655	MOSPD3	assignmentsChromaffin_cells.Chrom_SOX2_statusHigh
assignmentsChromaffin_cells.Chrom_SOX2_statusHigh.ZNF587B	-1.4006828	4.79122032	-5.5290008	4.61E-07	5.77E-06	6.04967955	ZNF587B	assignmentsChromaffin_cells.Chrom_SOX2_statusHigh
assignmentsChromaffin_cells.Chrom_SOX2_statusHigh.FADD	1.79943475	1.84028957	5.50751921	5.03E-07	6.20E-06	6.04843829	FADD	assignmentsChromaffin_cells.Chrom_SOX2_statusHigh
assignmentsChromaffin_cells.Chrom_SOX2_statusHigh.RAB6A	-0.8800533	7.41576725	-5.5541607	4.16E-07	5.31E-06	6.0399629	RAB6A	assignmentsChromaffin_cells.Chrom_SOX2_statusHigh
assignmentsChromaffin_cells.Chrom_SOX2_statusHigh.HAGLROS	4.33383897	-0.3090138	5.51022386	4.98E-07	6.15E-06	6.03953918	HAGLROS	assignmentsChromaffin_cells.Chrom_SOX2_statusHigh

assignmentsChromaffin_cells.Chrom_SOX2_statusHigh.ZNF721	-1.4257543	7.08374283	-5.5480047	4.27E-07	5.42E-06	6.02803507	ZNF721	assignmentsChromaffin_cells.Chrom_SOX2_statusHigh
assignmentsChromaffin_cells.Chrom_SOX2_statusHigh.TPT1-AS1	-1.0276995	6.77301353	-5.5461921	4.30E-07	5.44E-06	6.02569839	TPT1-AS1	assignmentsChromaffin_cells.Chrom_SOX2_statusHigh
assignmentsChromaffin_cells.Chrom_SOX2_statusHigh.ATP5ME	2.36679083	5.0735634	5.54273278	4.38E-07	5.52E-06	6.02350384	ATP5ME	assignmentsChromaffin_cells.Chrom_SOX2_statusHigh
assignmentsChromaffin_cells.Chrom_SOX2_statusHigh.TMEM19	1.42685149	3.12861182	5.5157662	4.86E-07	6.02E-06	6.02113312	TMEM19	assignmentsChromaffin_cells.Chrom_SOX2_statusHigh
assignmentsChromaffin_cells.Chrom_SOX2_statusHigh.EIF1B	2.10081243	5.15601344	5.53800527	4.45E-07	5.61E-06	6.01118776	EIF1B	assignmentsChromaffin_cells.Chrom_SOX2_statusHigh
assignmentsChromaffin_cells.Chrom_SOX2_statusHigh.WDR20	-1.0609	6.4162982	-5.5370103	4.46E-07	5.62E-06	6.01079851	WDR20	assignmentsChromaffin_cells.Chrom_SOX2_statusHigh
assignmentsChromaffin_cells.Chrom_SOX2_statusHigh.AIP	2.08362543	3.69505002	5.51723568	4.83E-07	5.99E-06	6.0050374	AIP	assignmentsChromaffin_cells.Chrom_SOX2_statusHigh
assignmentsChromaffin_cells.Chrom_SOX2_statusHigh.ILRUN	-1.0414247	6.36759496	-5.5317172	4.56E-07	5.72E-06	6.00044538	ILRUN	assignmentsChromaffin_cells.Chrom_SOX2_statusHigh
assignmentsChromaffin_cells.Chrom_SOX2_statusHigh.IFIT5	1.62653903	3.13360879	5.50961335	4.98E-07	6.15E-06	5.99843187	IFIT5	assignmentsChromaffin_cells.Chrom_SOX2_statusHigh
assignmentsChromaffin_cells.Chrom_SOX2_statusHigh.TRIAP1	2.26240836	1.97890792	5.5021538	5.14E-07	6.30E-06	5.99683758	TRIAP1	assignmentsChromaffin_cells.Chrom_SOX2_statusHigh
assignmentsChromaffin_cells.Chrom_SOX2_statusHigh.ZCCHC14	-1.1281518	6.20007902	-5.5312627	4.57E-07	5.72E-06	5.99284701	ZCCHC14	assignmentsChromaffin_cells.Chrom_SOX2_statusHigh
assignmentsChromaffin_cells.Chrom_SOX2_statusHigh.APEX1	1.49386457	4.69975532	5.52515831	4.68E-07	5.83E-06	5.98929851	APEX1	assignmentsChromaffin_cells.Chrom_SOX2_statusHigh
assignmentsChromaffin_cells.Chrom_SOX2_statusHigh.RPL14	3.20122294	6.88468883	5.54553877	4.34E-07	5.48E-06	5.98245344	RPL14	assignmentsChromaffin_cells.Chrom_SOX2_statusHigh
assignmentsChromaffin_cells.Chrom_SOX2_statusHigh.NTRK1	2.96487581	1.99129632	5.49867345	5.23E-07	6.40E-06	5.9742059	NTRK1	assignmentsChromaffin_cells.Chrom_SOX2_statusHigh
assignmentsChromaffin_cells.Chrom_SOX2_statusHigh.PIM3	3.10819417	3.79364605	5.50411586	5.12E-07	6.30E-06	5.97095244	PIM3	assignmentsChromaffin_cells.Chrom_SOX2_statusHigh
assignmentsChromaffin_cells.Chrom_SOX2_statusHigh.MED19	1.6163292	3.76596194	5.51281005	4.92E-07	6.09E-06	5.96789823	MED19	assignmentsChromaffin_cells.Chrom_SOX2_statusHigh
assignmentsChromaffin_cells.Chrom_SOX2_statusHigh.ZNF655	-0.9099557	6.19791072	-5.5255791	4.67E-07	5.83E-06	5.96582828	ZNF655	assignmentsChromaffin_cells.Chrom_SOX2_statusHigh
assignmentsChromaffin_cells.Chrom_SOX2_statusHigh.FUOM	3.50413188	1.1937536	5.48599149	5.48E-07	6.67E-06	5.96278449	FUOM	assignmentsChromaffin_cells.Chrom_SOX2_statusHigh
assignmentsChromaffin_cells.Chrom_SOX2_statusHigh.TPI1	2.45934801	5.9650976	5.53363993	4.55E-07	5.71E-06	5.95777413	TPI1	assignmentsChromaffin_cells.Chrom_SOX2_statusHigh
assignmentsChromaffin_cells.Chrom_SOX2_statusHigh.EXOC4	-0.9696676	8.93064065	-5.5437468	4.34E-07	5.48E-06	5.95606003	EXOC4	assignmentsChromaffin_cells.Chrom_SOX2_statusHigh
assignmentsChromaffin_cells.Chrom_SOX2_statusHigh.SMAD4	-0.9439684	6.50833092	-5.5219216	4.74E-07	5.88E-06	5.94754616	SMAD4	assignmentsChromaffin_cells.Chrom_SOX2_statusHigh
assignmentsChromaffin_cells.Chrom_SOX2_statusHigh.TBCC	1.90264015	2.74777307	5.4936544	5.31E-07	6.48E-06	5.94101893	TBCC	assignmentsChromaffin_cells.Chrom_SOX2_statusHigh
assignmentsChromaffin_cells.Chrom_SOX2_statusHigh.NMB	3.69229299	1.12168581	5.47134589	5.83E-07	7.01E-06	5.93765058	NMB	assignmentsChromaffin_cells.Chrom_SOX2_statusHigh
assignmentsChromaffin_cells.Chrom_SOX2_statusHigh.ZDHHC4	1.34065858	3.48866739	5.50241861	5.13E-07	6.30E-06	5.93713311	ZDHHC4	assignmentsChromaffin_cells.Chrom_SOX2_statusHigh
assignmentsChromaffin_cells.Chrom_SOX2_statusHigh.MYO1D	-2.6559911	7.18397791	-5.5060632	5.08E-07	6.25E-06	5.93572997	MYO1D	assignmentsChromaffin_cells.Chrom_SOX2_statusHigh
assignmentsChromaffin_cells.Chrom_SOX2_statusHigh.SNAPIN	2.15318182	2.92830567	5.49566136	5.27E-07	6.43E-06	5.93272875	SNAPIN	assignmentsChromaffin_cells.Chrom_SOX2_statusHigh
assignmentsChromaffin_cells.Chrom_SOX2_statusHigh.B4GAT1	2.8119274	3.3055516	5.50863764	5.03E-07	6.20E-06	5.93267909	B4GAT1	assignmentsChromaffin_cells.Chrom_SOX2_statusHigh
assignmentsChromaffin_cells.Chrom_SOX2_statusHigh.CCDC43	1.18220211	3.27194576	5.49359558	5.32E-07	6.48E-06	5.93033533	CCDC43	assignmentsChromaffin_cells.Chrom_SOX2_statusHigh
assignmentsChromaffin_cells.Chrom_SOX2_statusHigh.TMEM222	1.13418447	3.76228229	5.49620124	5.26E-07	6.43E-06	5.92332832	TMEM222	assignmentsChromaffin_cells.Chrom_SOX2_statusHigh
assignmentsChromaffin_cells.Chrom_SOX2_statusHigh.ZNF185	2.0685141	1.46281314	5.47123989	5.81E-07	7.01E-06	5.92320529	ZNF185	assignmentsChromaffin_cells.Chrom_SOX2_statusHigh
assignmentsChromaffin_cells.Chrom_SOX2_statusHigh.RPL12	2.99533515	6.93266134	5.52761879	4.66E-07	5.82E-06	5.9093247	RPL12	assignmentsChromaffin_cells.Chrom_SOX2_statusHigh
assignmentsChromaffin_cells.Chrom_SOX2_statusHigh.FKBPL	2.32665438	0.69603686	5.46330725	6.00E-07	7.19E-06	5.90904727	FKBPL	assignmentsChromaffin_cells.Chrom_SOX2_statusHigh
assignmentsChromaffin_cells.Chrom_SOX2_statusHigh.PSMD7	1.17531171	5.56138683	5.51027392	4.97E-07	6.14E-06	5.90449149	PSMD7	assignmentsChromaffin_cells.Chrom_SOX2_statusHigh
assignmentsChromaffin_cells.Chrom_SOX2_statusHigh.QPCT	3.59222291	3.14682029	5.49761563	5.26E-07	6.43E-06	5.90263091	QPCT	assignmentsChromaffin_cells.Chrom_SOX2_statusHigh
assignmentsChromaffin_cells.Chrom_SOX2_statusHigh.WDFY1	-0.9000664	6.30922676	-5.5029437	5.12E-07	6.30E-06	5.8960973	WDFY1	assignmentsChromaffin_cells.Chrom_SOX2_statusHigh
assignmentsChromaffin_cells.Chrom_SOX2_statusHigh.NBAS	-1.0554268	7.90950428	-5.523421	4.71E-07	5.86E-06	5.89526051	NBAS	assignmentsChromaffin_cells.Chrom_SOX2_statusHigh
assignmentsChromaffin_cells.Chrom_SOX2_statusHigh.RPS25	3.12302149	6.89609681	5.52424457	4.73E-07	5.87E-06	5.89372492	RPS25	assignmentsChromaffin_cells.Chrom_SOX2_statusHigh
assignmentsChromaffin_cells.Chrom_SOX2_statusHigh.RPS4X	3.73074485	7.06334526	5.5265053	4.68E-07	5.83E-06	5.89172825	RPS4X	assignmentsChromaffin_cells.Chrom_SOX2_statusHigh
assignmentsChromaffin_cells.Chrom_SOX2_statusHigh.MYC	3.61021352	2.95840606	5.46355176	6.03E-07	7.22E-06	5.87712021	MYC	assignmentsChromaffin_cells.Chrom_SOX2_statusHigh
assignmentsChromaffin_cells.Chrom_SOX2_statusHigh.HIRIP3	1.58930719	2.50569335	5.47237551	5.79E-07	7.00E-06	5.87176974	HIRIP3	assignmentsChromaffin_cells.Chrom_SOX2_statusHigh
assignmentsChromaffin_cells.Chrom_SOX2_statusHigh.CADM4	1.53572499	2.37455893	5.47400796	5.75E-07	6.96E-06	5.86471475	CADM4	assignmentsChromaffin_cells.Chrom_SOX2_statusHigh
assignmentsChromaffin_cells.Chrom_SOX2_statusHigh.AGER	-1.8675418	2.52845168	-5.4521561	6.28E-07	7.47E-06	5.86226879	AGER	assignmentsChromaffin_cells.Chrom_SOX2_statusHigh
assignmentsChromaffin_cells.Chrom_SOX2_statusHigh.SCART1	-1.7837302	3.71940242	-5.4635049	6.00E-07	7.19E-06	5.8606672	SCART1	assignmentsChromaffin_cells.Chrom_SOX2_statusHigh
assignmentsChromaffin_cells.Chrom_SOX2_statusHigh.CNTNAP3	-2.6925327	2.78233331	-5.4637981	6.00E-07	7.19E-06	5.85059077	CNTNAP3	assignmentsChromaffin_cells.Chrom_SOX2_statusHigh
assignmentsChromaffin_cells.Chrom_SOX2_statusHigh.BMPR2	-1.2256923	7.80128694	-5.5009333	5.16E-07	6.33E-06	5.83774479	BMPR2	assignmentsChromaffin_cells.Chrom_SOX2_statusHigh
assignmentsChromaffin_cells.Chrom_SOX2_statusHigh.CHCHD7	1.46786976	4.01641609	5.47968933	5.62E-07	6.83E-06	5.82947289	CHCHD7	assignmentsChromaffin_cells.Chrom_SOX2_statusHigh
assignmentsChromaffin_cells.Chrom_SOX2_statusHigh.AP1G1	-0.8626935	6.62419905	-5.4904875	5.38E-07	6.55E-06	5.82768725	AP1G1	assignmentsChromaffin_cells.Chrom_SOX2_statusHigh
assignmentsChromaffin_cells.Chrom_SOX2_statusHigh.SMIM1	5.76043226	-0.4998205	5.46488607	6.00E-07	7.19E-06	5.81258782	SMIM1	assignmentsChromaffin_cells.Chrom_SOX2_statusHigh
assignmentsChromaffin_cells.Chrom_SOX2_statusHigh.POLR2M	1.37061026	3.42549311	5.46692045	5.92E-07	7.12E-06	5.81226066	POLR2M	assignmentsChromaffin_cells.Chrom_SOX2_statusHigh
assignmentsChromaffin_cells.Chrom_SOX2_statusHigh.MID1IP1	4.71991958	1.32283665	5.44122195	6.59E-07	7.78E-06	5.811231	MID1IP1	assignmentsChromaffin_cells.Chrom_SOX2_statusHigh
assignmentsChromaffin_cells.Chrom_SOX2_statusHigh.CDIPT	1.63244843	3.7739982	5.4715417	5.81E-07	7.01E-06	5.80884762	CDIPT	assignmentsChromaffin_cells.Chrom_SOX2_statusHigh
assignmentsChromaffin_cells.Chrom_SOX2_statusHigh.TCEAL7	4.28707268	3.07116711	5.47291381	5.81E-07	7.01E-06	5.79487844	TCEAL7	assignmentsChromaffin_cells.Chrom_SOX2_statusHigh
assignmentsChromaffin_cells.Chrom_SOX2_statusHigh.TTC21B	-0.94742	6.28673967	-5.4777601	5.66E-07	6.87E-06	5.78842589	TTC21B	assignmentsChromaffin_cells.Chrom_SOX2_statusHigh
assignmentsChromaffin_cells.Chrom_SOX2_statusHigh.RPS23	3.57990564	7.37130923	5.49907134	5.23E-07	6.40E-06	5.77758103	RPS23	assignmentsChromaffin_cells.Chrom_SOX2_statusHigh
assignmentsChromaffin_cells.Chrom_SOX2_statusHigh.ACTL6A	1.12719304	4.01384671	5.45875044	6.11E-07	7.30E-06	5.77342691	ACTL6A	assignmentsChromaffin_cells.Chrom_SOX2_statusHigh
assignmentsChromaffin_cells.Chrom_SOX2_statusHigh.C6orf226	1.92057916	2.4295723	5.44824713	6.38E-07	7.56E-06	5.76779696	C6orf226	assignmentsChromaffin_cells.Chrom_SOX2_statusHigh
assignmentsChromaffin_cells.Chrom_SOX2_statusHigh.NUDT16	1.84035823	3.36805159	5.44856957	6.37E-07	7.55E-06	5.76715559	NUDT16	assignmentsChromaffin_cells.Chrom_SOX2_statusHigh
assignmentsChromaffin_cells.Chrom_SOX2_statusHigh.GEMIN6	1.85158202	1.97672839	5.43844445	6.63E-07	7.82E-06	5.76358333	GEMIN6	assignmentsChromaffin_cells.Chrom_SOX2_statusHigh
assignmentsChromaffin_cells.Chrom_SOX2_statusHigh.COX8A	3.29487557	5.21804527	5.47845628	5.68E-07	6.89E-06	5.76346258	COX8A	assignmentsChromaffin_cells.Chrom_SOX2_statusHigh
assignmentsChromaffin_cells.Chrom_SOX2_statusHigh.CCNT2	-0.8556394	6.69611319	-5.4738021	5.76E-07	6.96E-06	5.75398053	CCNT2	assignmentsChromaffin_cells.Chrom_SOX2_statusHigh
assignmentsChromaffin_cells.Chrom_SOX2_statusHigh.RPLP1	3.66613867	8.10598504	5.49979207	5.21E-07	6.39E-06	5.75330396	RPLP1	assignmentsChromaffin_cells.Chrom_SOX2_statusHigh
assignmentsChromaffin_cells.Chrom_SOX2_statusHigh.AGAP2	-2.2239028	2.26408643	-5.4214291	7.10E-07	8.27E-06	5.74902616	AGAP2	assignmentsChromaffin_cells.Chrom_SOX2_statusHigh
assignmentsChromaffin_cells.Chrom_SOX2_statusHigh.TMSB10	3.81936312	6.43494652	5.46559923	5.98E-07	7.19E-06	5.74658868	TMSB10	assignmentsChromaffin_cells.Chrom_SOX2_statusHigh
assignmentsChromaffin_cells.Chrom_SOX2_statusHigh.SCO2	1.90965956	3.36021051	5.43514323	6.72E-07	7.88E-06	5.74404895	SCO2	assignmentsChromaffin_cells.Chrom_SOX2_statusHigh

assignmentsChromaffin_cells.Chrom_SOX2_statusHigh.PCNX1	-1.4224297	8.03978443	-5.4800043	5.61E-07	6.83E-06	5.74196625	PCNX1	assignmentsChromaffin_cells.Chrom_SOX2_statusHigh
assignmentsChromaffin_cells.Chrom_SOX2_statusHigh.MYRIP	-3.9523851	4.45080593	-5.435994	6.74E-07	7.89E-06	5.74022393	MYRIP	assignmentsChromaffin_cells.Chrom_SOX2_statusHigh
assignmentsChromaffin_cells.Chrom_SOX2_statusHigh.TMEM17	1.83614307	1.4082885	5.43107642	6.83E-07	7.99E-06	5.73402144	TMEM17	assignmentsChromaffin_cells.Chrom_SOX2_statusHigh
assignmentsChromaffin_cells.Chrom_SOX2_statusHigh.ABHD18	-0.9552337	6.56953427	-5.4708593	5.82E-07	7.01E-06	5.7282471	ABHD18	assignmentsChromaffin_cells.Chrom_SOX2_statusHigh
assignmentsChromaffin_cells.Chrom_SOX2_statusHigh.MEIS3	-2.6286041	4.11176376	-5.4378259	6.66E-07	7.84E-06	5.72236069	MEIS3	assignmentsChromaffin_cells.Chrom_SOX2_statusHigh
assignmentsChromaffin_cells.Chrom_SOX2_statusHigh.TRAPPC8	-0.8774377	6.53591156	-5.4644063	5.98E-07	7.18E-06	5.72174769	TRAPPC8	assignmentsChromaffin_cells.Chrom_SOX2_statusHigh
assignmentsChromaffin_cells.Chrom_SOX2_statusHigh.VAMP5	3.3644786	2.56827696	5.4132724	7.35E-07	8.51E-06	5.72151464	VAMP5	assignmentsChromaffin_cells.Chrom_SOX2_statusHigh
assignmentsChromaffin_cells.Chrom_SOX2_statusHigh.KLHDC9	2.05709659	1.65324433	5.43797313	6.64E-07	7.82E-06	5.72070399	KLHDC9	assignmentsChromaffin_cells.Chrom_SOX2_statusHigh
assignmentsChromaffin_cells.Chrom_SOX2_statusHigh.ATP8B2	-1.2511844	5.02479911	-5.4527566	6.26E-07	7.46E-06	5.71961194	ATP8B2	assignmentsChromaffin_cells.Chrom_SOX2_statusHigh
assignmentsChromaffin_cells.Chrom_SOX2_statusHigh.NDUFB8	1.68549164	6.35863381	5.47404141	5.75E-07	6.96E-06	5.71776218	NDUFB8	assignmentsChromaffin_cells.Chrom_SOX2_statusHigh
assignmentsChromaffin_cells.Chrom_SOX2_statusHigh.OSTC	1.72962307	5.36001752	5.46162237	6.04E-07	7.23E-06	5.71265007	OSTC	assignmentsChromaffin_cells.Chrom_SOX2_statusHigh
assignmentsChromaffin_cells.Chrom_SOX2_statusHigh.ZNF396	1.52981887	1.10234842	5.41566356	7.27E-07	8.43E-06	5.71222727	ZNF396	assignmentsChromaffin_cells.Chrom_SOX2_statusHigh
assignmentsChromaffin_cells.Chrom_SOX2_statusHigh.ELOC	1.78226782	5.36565132	5.45739	6.15E-07	7.34E-06	5.70119465	ELOC	assignmentsChromaffin_cells.Chrom_SOX2_statusHigh
assignmentsChromaffin_cells.Chrom_SOX2_statusHigh.OR2L2	-3.8266861	1.36879151	-5.4086194	7.48E-07	8.63E-06	5.69639057	OR2L2	assignmentsChromaffin_cells.Chrom_SOX2_statusHigh
assignmentsChromaffin_cells.Chrom_SOX2_statusHigh.RPS28	3.68957751	7.40484075	5.47932095	5.66E-07	6.87E-06	5.68948596	RPS28	assignmentsChromaffin_cells.Chrom_SOX2_statusHigh
assignmentsChromaffin_cells.Chrom_SOX2_statusHigh.ALS2	-1.1657211	5.86200319	-5.4488727	6.36E-07	7.55E-06	5.68885267	ALS2	assignmentsChromaffin_cells.Chrom_SOX2_statusHigh
assignmentsChromaffin_cells.Chrom_SOX2_statusHigh.CNOT10	-1.0522121	5.82391569	-5.4451048	6.46E-07	7.65E-06	5.68449718	CNOT10	assignmentsChromaffin_cells.Chrom_SOX2_statusHigh
assignmentsChromaffin_cells.Chrom_SOX2_statusHigh.FAM149B1	-1.1982177	5.06583321	-5.4387271	6.62E-07	7.81E-06	5.68210127	FAM149B1	assignmentsChromaffin_cells.Chrom_SOX2_statusHigh
assignmentsChromaffin_cells.Chrom_SOX2_statusHigh.OR2L3	-4.5497273	1.24111736	-5.4094088	7.47E-07	8.62E-06	5.68074124	OR2L3	assignmentsChromaffin_cells.Chrom_SOX2_statusHigh
assignmentsChromaffin_cells.Chrom_SOX2_statusHigh.MYL12B	2.38984775	5.96879745	5.45139187	6.32E-07	7.51E-06	5.67271916	MYL12B	assignmentsChromaffin_cells.Chrom_SOX2_statusHigh
assignmentsChromaffin_cells.Chrom_SOX2_statusHigh.PMVK	1.82176324	4.21865128	5.44166607	6.55E-07	7.74E-06	5.67034876	PMVK	assignmentsChromaffin_cells.Chrom_SOX2_statusHigh
assignmentsChromaffin_cells.Chrom_SOX2_statusHigh.IRF2BP1	2.10665209	1.77959359	5.41559257	7.27E-07	8.43E-06	5.6686467	IRF2BP1	assignmentsChromaffin_cells.Chrom_SOX2_statusHigh
assignmentsChromaffin_cells.Chrom_SOX2_statusHigh.ITPRIP	3.42339707	4.98493319	5.41867307	7.22E-07	8.40E-06	5.65527686	ITPRIP	assignmentsChromaffin_cells.Chrom_SOX2_statusHigh
assignmentsChromaffin_cells.Chrom_SOX2_statusHigh.CSTF2T	1.65343467	2.43052795	5.41157479	7.38E-07	8.54E-06	5.64913872	CSTF2T	assignmentsChromaffin_cells.Chrom_SOX2_statusHigh
assignmentsChromaffin_cells.Chrom_SOX2_statusHigh.HCG27	-2.2915247	2.39786091	-5.3936265	7.93E-07	9.06E-06	5.64902149	HCG27	assignmentsChromaffin_cells.Chrom_SOX2_statusHigh
assignmentsChromaffin_cells.Chrom_SOX2_statusHigh.SMIM10	5.26480248	0.3551767	5.41667292	7.26E-07	8.43E-06	5.64798458	SMIM10	assignmentsChromaffin_cells.Chrom_SOX2_statusHigh
assignmentsChromaffin_cells.Chrom_SOX2_statusHigh.RPL29	3.51493809	6.27278919	5.45329107	6.28E-07	7.48E-06	5.64787566	RPL29	assignmentsChromaffin_cells.Chrom_SOX2_statusHigh
assignmentsChromaffin_cells.Chrom_SOX2_statusHigh.NFXL1	-0.8995069	5.29071957	-5.4352967	6.72E-07	7.88E-06	5.64432128	NFXL1	assignmentsChromaffin_cells.Chrom_SOX2_statusHigh
assignmentsChromaffin_cells.Chrom_SOX2_statusHigh.TMEM50A	1.65359675	5.11211558	5.43142031	6.82E-07	7.99E-06	5.64426471	TMEM50A	assignmentsChromaffin_cells.Chrom_SOX2_statusHigh
assignmentsChromaffin_cells.Chrom_SOX2_statusHigh.GUCY1A2	2.48352614	4.33938655	5.43664379	6.71E-07	7.87E-06	5.64013656	GUCY1A2	assignmentsChromaffin_cells.Chrom_SOX2_statusHigh
assignmentsChromaffin_cells.Chrom_SOX2_statusHigh.UBA52	3.55823126	6.23419486	5.45250123	6.30E-07	7.50E-06	5.64000986	UBA52	assignmentsChromaffin_cells.Chrom_SOX2_statusHigh
assignmentsChromaffin_cells.Chrom_SOX2_statusHigh.NFAT5	-1.0977862	8.59880446	-5.4603592	6.07E-07	7.26E-06	5.63956299	NFAT5	assignmentsChromaffin_cells.Chrom_SOX2_statusHigh
assignmentsChromaffin_cells.Chrom_SOX2_statusHigh.ACLY	1.30407645	5.09998595	5.43801577	6.64E-07	7.82E-06	5.63905262	ACLY	assignmentsChromaffin_cells.Chrom_SOX2_statusHigh
assignmentsChromaffin_cells.Chrom_SOX2_statusHigh.RPS16	3.24700411	6.97249731	5.45771719	6.17E-07	7.36E-06	5.63813687	RPS16	assignmentsChromaffin_cells.Chrom_SOX2_statusHigh
assignmentsChromaffin_cells.Chrom_SOX2_statusHigh.TRAM1	1.35988843	6.07798384	5.44311346	6.51E-07	7.70E-06	5.63250303	TRAM1	assignmentsChromaffin_cells.Chrom_SOX2_statusHigh
assignmentsChromaffin_cells.Chrom_SOX2_statusHigh.ETFDH	-0.9731152	6.10678732	-5.4411133	6.56E-07	7.75E-06	5.63222445	ETFDH	assignmentsChromaffin_cells.Chrom_SOX2_statusHigh
assignmentsChromaffin_cells.Chrom_SOX2_statusHigh.COA4	1.42333205	4.21271485	5.42921097	6.88E-07	8.04E-06	5.63088782	COA4	assignmentsChromaffin_cells.Chrom_SOX2_statusHigh
assignmentsChromaffin_cells.Chrom_SOX2_statusHigh.MCFD2	1.3805967	5.25729604	5.43917736	6.61E-07	7.80E-06	5.62988373	MCFD2	assignmentsChromaffin_cells.Chrom_SOX2_statusHigh
assignmentsChromaffin_cells.Chrom_SOX2_statusHigh.MRPS14	1.16958414	4.13925088	5.42492445	7.00E-07	8.17E-06	5.61720609	MRPS14	assignmentsChromaffin_cells.Chrom_SOX2_statusHigh
assignmentsChromaffin_cells.Chrom_SOX2_statusHigh.TEN1	1.72798884	2.85704713	5.40236125	7.66E-07	8.81E-06	5.58853673	TEN1	assignmentsChromaffin_cells.Chrom_SOX2_statusHigh
assignmentsChromaffin_cells.Chrom_SOX2_statusHigh.GADD45B	4.98449495	5.07158652	5.3965851	7.88E-07	9.01E-06	5.58507195	GADD45B	assignmentsChromaffin_cells.Chrom_SOX2_statusHigh
assignmentsChromaffin_cells.Chrom_SOX2_statusHigh.RPS13	3.34388523	6.56069602	5.43845785	6.67E-07	7.84E-06	5.5821766	RPS13	assignmentsChromaffin_cells.Chrom_SOX2_statusHigh
assignmentsChromaffin_cells.Chrom_SOX2_statusHigh.CCNDBP1	1.48394037	4.21995073	5.41078438	7.41E-07	8.56E-06	5.57577379	CCNDBP1	assignmentsChromaffin_cells.Chrom_SOX2_statusHigh
assignmentsChromaffin_cells.Chrom_SOX2_statusHigh.TLCD1	2.37799689	0.19196371	5.37387917	8.58E-07	9.69E-06	5.57415599	TLCD1	assignmentsChromaffin_cells.Chrom_SOX2_statusHigh
assignmentsChromaffin_cells.Chrom_SOX2_statusHigh.TYW1	-0.8921624	6.70734536	-5.4265638	6.96E-07	8.12E-06	5.56290777	TYW1	assignmentsChromaffin_cells.Chrom_SOX2_statusHigh
assignmentsChromaffin_cells.Chrom_SOX2_statusHigh.SWSAP1	2.1585743	1.72997274	5.37780081	8.45E-07	9.57E-06	5.55537299	SWSAP1	assignmentsChromaffin_cells.Chrom_SOX2_statusHigh
assignmentsChromaffin_cells.Chrom_SOX2_statusHigh.CACYBP	2.22398731	5.31347108	5.42194537	7.10E-07	8.27E-06	5.55206699	CACYBP	assignmentsChromaffin_cells.Chrom_SOX2_statusHigh
assignmentsChromaffin_cells.Chrom_SOX2_statusHigh.ALDH1B1	2.33584156	0.45875476	5.36430573	8.92E-07	1.00E-05	5.53967069	ALDH1B1	assignmentsChromaffin_cells.Chrom_SOX2_statusHigh
assignmentsChromaffin_cells.Chrom_SOX2_statusHigh.CTBS	1.50660988	4.46343413	5.39652661	7.84E-07	8.98E-06	5.53835446	CTBS	assignmentsChromaffin_cells.Chrom_SOX2_statusHigh
assignmentsChromaffin_cells.Chrom_SOX2_statusHigh.PNKD	1.75195764	4.46848046	5.4042129	7.61E-07	8.76E-06	5.53573234	PNKD	assignmentsChromaffin_cells.Chrom_SOX2_statusHigh
assignmentsChromaffin_cells.Chrom_SOX2_statusHigh.TMEM45A	2.27369167	4.07242024	5.41350914	7.35E-07	8.51E-06	5.53400477	TMEM45A	assignmentsChromaffin_cells.Chrom_SOX2_statusHigh
assignmentsChromaffin_cells.Chrom_SOX2_statusHigh.CALM3	2.01295398	4.92687433	5.4107519	7.42E-07	8.57E-06	5.52092469	CALM3	assignmentsChromaffin_cells.Chrom_SOX2_statusHigh
assignmentsChromaffin_cells.Chrom_SOX2_statusHigh.GLOD4	1.3771042	4.26402766	5.39756571	7.81E-07	8.95E-06	5.51197139	GLOD4	assignmentsChromaffin_cells.Chrom_SOX2_statusHigh
assignmentsChromaffin_cells.Chrom_SOX2_statusHigh.RAB5C	1.37190575	5.27471055	5.40206651	7.67E-07	8.81E-06	5.50956472	RAB5C	assignmentsChromaffin_cells.Chrom_SOX2_statusHigh
assignmentsChromaffin_cells.Chrom_SOX2_statusHigh.RPL35	2.71150175	6.6950586	5.42067568	7.16E-07	8.33E-06	5.50704936	RPL35	assignmentsChromaffin_cells.Chrom_SOX2_statusHigh
assignmentsChromaffin_cells.Chrom_SOX2_statusHigh.PSMC4	1.42946384	4.7824574	5.40046967	7.72E-07	8.86E-06	5.50047158	PSMC4	assignmentsChromaffin_cells.Chrom_SOX2_statusHigh
assignmentsChromaffin_cells.Chrom_SOX2_statusHigh.ARPC5	1.70290611	6.00253597	5.40404631	7.61E-07	8.76E-06	5.49780181	ARPC5	assignmentsChromaffin_cells.Chrom_SOX2_statusHigh
assignmentsChromaffin_cells.Chrom_SOX2_statusHigh.GPR108	1.36944907	3.97375676	5.38561591	8.19E-07	9.30E-06	5.4960004	GPR108	assignmentsChromaffin_cells.Chrom_SOX2_statusHigh
assignmentsChromaffin_cells.Chrom_SOX2_statusHigh.LINC01725	4.02796998	3.71962937	5.39762273	7.85E-07	8.98E-06	5.49245708	LINC01725	assignmentsChromaffin_cells.Chrom_SOX2_statusHigh
assignmentsChromaffin_cells.Chrom_SOX2_statusHigh.PCNA	1.8897163	3.04123583	5.37509482	8.54E-07	9.66E-06	5.48743011	PCNA	assignmentsChromaffin_cells.Chrom_SOX2_statusHigh
assignmentsChromaffin_cells.Chrom_SOX2_statusHigh.GAPDH	3.51295482	7.49696503	5.42956749	6.91E-07	8.07E-06	5.48667604	GAPDH	assignmentsChromaffin_cells.Chrom_SOX2_statusHigh
assignmentsChromaffin_cells.Chrom_SOX2_statusHigh.NRM	1.79002417	1.32103763	5.35306661	9.33E-07	1.03E-05	5.48496145	NRM	assignmentsChromaffin_cells.Chrom_SOX2_statusHigh
assignmentsChromaffin_cells.Chrom_SOX2_statusHigh.TNFRSF12A	3.22932463	3.02890846	5.36385241	8.97E-07	1.01E-05	5.48001363	TNFRSF12A	assignmentsChromaffin_cells.Chrom_SOX2_statusHigh
assignmentsChromaffin_cells.Chrom_SOX2_statusHigh.RELCH	-0.8914347	6.86568389	-5.4043002	7.60E-07	8.76E-06	5.47891551	RELCH	assignmentsChromaffin_cells.Chrom_SOX2_statusHigh

assignmentsChromaffin_cells.Chrom_SOX2_statusHigh.NUP205	-0.9998354	5.58231559	-5.3901334	8.05E-07	9.17E-06	5.47372075	NUP205	assignmentsChromaffin_cells.Chrom_SOX2_statusHigh
assignmentsChromaffin_cells.Chrom_SOX2_statusHigh.LUC7L2	-0.9085733	7.78039615	-5.4124673	7.36E-07	8.52E-06	5.47270988	LUC7L2	assignmentsChromaffin_cells.Chrom_SOX2_statusHigh
assignmentsChromaffin_cells.Chrom_SOX2_statusHigh.CDRT4	-1.8727934	5.46837415	-5.3886165	8.09E-07	9.21E-06	5.46973327	CDRT4	assignmentsChromaffin_cells.Chrom_SOX2_statusHigh
assignmentsChromaffin_cells.Chrom_SOX2_statusHigh.KANK2	2.41037881	3.77409714	5.35826028	9.14E-07	1.02E-05	5.46366144	KANK2	assignmentsChromaffin_cells.Chrom_SOX2_statusHigh
assignmentsChromaffin_cells.Chrom_SOX2_statusHigh.NCOA1	-0.987378	8.56066664	-5.413341	7.33E-07	8.50E-06	5.45861138	NCOA1	assignmentsChromaffin_cells.Chrom_SOX2_statusHigh
assignmentsChromaffin_cells.Chrom_SOX2_statusHigh.CFL2	1.33014185	3.8676649	5.38030656	8.37E-07	9.48E-06	5.4562937	CFL2	assignmentsChromaffin_cells.Chrom_SOX2_statusHigh
assignmentsChromaffin_cells.Chrom_SOX2_statusHigh.C19orf81	2.82355517	1.7005622	5.36774801	8.83E-07	9.93E-06	5.45116399	C19orf81	assignmentsChromaffin_cells.Chrom_SOX2_statusHigh
assignmentsChromaffin_cells.Chrom_SOX2_statusHigh.ADH5	1.22339588	4.66971072	5.38233897	8.30E-07	9.41E-06	5.44055029	ADH5	assignmentsChromaffin_cells.Chrom_SOX2_statusHigh
assignmentsChromaffin_cells.Chrom_SOX2_statusHigh.ZNF439	-3.3433048	4.55269438	-5.3557496	9.28E-07	1.03E-05	5.44011643	ZNF439	assignmentsChromaffin_cells.Chrom_SOX2_statusHigh
assignmentsChromaffin_cells.Chrom_SOX2_statusHigh.KRR1	1.08960748	5.13472329	5.38665537	8.16E-07	9.27E-06	5.43729265	KRR1	assignmentsChromaffin_cells.Chrom_SOX2_statusHigh
assignmentsChromaffin_cells.Chrom_SOX2_statusHigh.C18orf21	1.36437912	2.8603437	5.36212713	9.00E-07	1.01E-05	5.43185493	C18orf21	assignmentsChromaffin_cells.Chrom_SOX2_statusHigh
assignmentsChromaffin_cells.Chrom_SOX2_statusHigh.BSCL2	1.73775182	5.78250638	5.4011916	7.70E-07	8.84E-06	5.43071427	BSCL2	assignmentsChromaffin_cells.Chrom_SOX2_statusHigh
assignmentsChromaffin_cells.Chrom_SOX2_statusHigh.RPL35A	3.52790939	6.56311208	5.40076156	7.75E-07	8.89E-06	5.42668511	RPL35A	assignmentsChromaffin_cells.Chrom_SOX2_statusHigh
assignmentsChromaffin_cells.Chrom_SOX2_statusHigh.EMC9	1.65312935	2.76543408	5.36326719	8.96E-07	1.01E-05	5.41786954	EMC9	assignmentsChromaffin_cells.Chrom_SOX2_statusHigh
assignmentsChromaffin_cells.Chrom_SOX2_statusHigh.MPLKIP	1.21256923	4.58690693	5.37749969	8.46E-07	9.57E-06	5.41684684	MPLKIP	assignmentsChromaffin_cells.Chrom_SOX2_statusHigh
assignmentsChromaffin_cells.Chrom_SOX2_statusHigh.VCP	1.11967994	6.37372503	5.39097727	8.02E-07	9.15E-06	5.41501262	VCP	assignmentsChromaffin_cells.Chrom_SOX2_statusHigh
assignmentsChromaffin_cells.Chrom_SOX2_statusHigh.UHMK1	0.87092319	5.46423644	5.38354791	8.26E-07	9.38E-06	5.41474076	UHMK1	assignmentsChromaffin_cells.Chrom_SOX2_statusHigh
assignmentsChromaffin_cells.Chrom_SOX2_statusHigh.PHF5A	1.42685536	3.39655867	5.36095026	9.04E-07	1.01E-05	5.41395241	PHF5A	assignmentsChromaffin_cells.Chrom_SOX2_statusHigh
assignmentsChromaffin_cells.Chrom_SOX2_statusHigh.FAM168B	-0.9957724	5.68384954	-5.3741448	8.58E-07	9.69E-06	5.41088468	FAM168B	assignmentsChromaffin_cells.Chrom_SOX2_statusHigh
assignmentsChromaffin_cells.Chrom_SOX2_statusHigh.CBFA2T2	-1.1532487	7.21837137	-5.3911398	8.01E-07	9.15E-06	5.41081458	CBFA2T2	assignmentsChromaffin_cells.Chrom_SOX2_statusHigh
assignmentsChromaffin_cells.Chrom_SOX2_statusHigh.TRRAP	-0.8715344	6.99016351	-5.3896688	8.06E-07	9.18E-06	5.40562091	TRRAP	assignmentsChromaffin_cells.Chrom_SOX2_statusHigh
assignmentsChromaffin_cells.Chrom_SOX2_statusHigh.ACAD10	-1.3397536	4.71635298	-5.3612725	9.03E-07	1.01E-05	5.39359935	ACAD10	assignmentsChromaffin_cells.Chrom_SOX2_statusHigh
assignmentsChromaffin_cells.Chrom_SOX2_statusHigh.PLPP5	1.2812234	4.51766215	5.37088652	8.69E-07	9.78E-06	5.39003735	PLPP5	assignmentsChromaffin_cells.Chrom_SOX2_statusHigh
assignmentsChromaffin_cells.Chrom_SOX2_statusHigh.NR2C2	-0.832057	6.30029104	-5.3736139	8.59E-07	9.69E-06	5.38931896	NR2C2	assignmentsChromaffin_cells.Chrom_SOX2_statusHigh
assignmentsChromaffin_cells.Chrom_SOX2_statusHigh.HTATIP2	3.58013482	2.06835804	5.3255507	1.04E-06	1.14E-05	5.38852743	HTATIP2	assignmentsChromaffin_cells.Chrom_SOX2_statusHigh
assignmentsChromaffin_cells.Chrom_SOX2_statusHigh.RITA1	1.39351807	2.25575598	5.34289292	9.71E-07	1.07E-05	5.38528586	RITA1	assignmentsChromaffin_cells.Chrom_SOX2_statusHigh
assignmentsChromaffin_cells.Chrom_SOX2_statusHigh.CDKN2AIP	1.7290283	3.77320554	5.35534907	9.24E-07	1.03E-05	5.38520456	CDKN2AIP	assignmentsChromaffin_cells.Chrom_SOX2_statusHigh
assignmentsChromaffin_cells.Chrom_SOX2_statusHigh.ZNF24	0.92364787	5.43557602	5.37169034	8.66E-07	9.75E-06	5.37547873	ZNF24	assignmentsChromaffin_cells.Chrom_SOX2_statusHigh
assignmentsChromaffin_cells.Chrom_SOX2_statusHigh.HS3ST1	4.01641779	3.44755012	5.35592095	9.27E-07	1.03E-05	5.37370711	HS3ST1	assignmentsChromaffin_cells.Chrom_SOX2_statusHigh
assignmentsChromaffin_cells.Chrom_SOX2_statusHigh.DGUOK	1.06626767	5.8751586	5.37811714	8.44E-07	9.56E-06	5.37225292	DGUOK	assignmentsChromaffin_cells.Chrom_SOX2_statusHigh
assignmentsChromaffin_cells.Chrom_SOX2_statusHigh.TIMM8B	2.25650135	3.44054397	5.3550079	9.27E-07	1.03E-05	5.37186016	TIMM8B	assignmentsChromaffin_cells.Chrom_SOX2_statusHigh
assignmentsChromaffin_cells.Chrom_SOX2_statusHigh.POGZ	-0.7872335	7.68888585	-5.3871572	8.14E-07	9.26E-06	5.37130083	POGZ	assignmentsChromaffin_cells.Chrom_SOX2_statusHigh
assignmentsChromaffin_cells.Chrom_SOX2_statusHigh.TINF2	1.45395147	3.13788111	5.34259917	9.72E-07	1.07E-05	5.36576162	TINF2	assignmentsChromaffin_cells.Chrom_SOX2_statusHigh
assignmentsChromaffin_cells.Chrom_SOX2_statusHigh.IFITM1	4.20362028	2.5935186	5.31804119	1.07E-06	1.16E-05	5.35984102	IFITM1	assignmentsChromaffin_cells.Chrom_SOX2_statusHigh
assignmentsChromaffin_cells.Chrom_SOX2_statusHigh.NAP1L3	1.87573415	2.62363597	5.34950471	9.46E-07	1.04E-05	5.34939117	NAP1L3	assignmentsChromaffin_cells.Chrom_SOX2_statusHigh
assignmentsChromaffin_cells.Chrom_SOX2_statusHigh.CRIP2	2.05474711	4.86477627	5.3599322	9.09E-07	1.02E-05	5.34250597	CRIP2	assignmentsChromaffin_cells.Chrom_SOX2_statusHigh
assignmentsChromaffin_cells.Chrom_SOX2_statusHigh.ZC3H12A	3.16595534	2.12969948	5.31208517	1.10E-06	1.19E-05	5.33971883	ZC3H12A	assignmentsChromaffin_cells.Chrom_SOX2_statusHigh
assignmentsChromaffin_cells.Chrom_SOX2_statusHigh.ATG101	1.76336603	3.52776229	5.34149708	9.77E-07	1.07E-05	5.33754752	ATG101	assignmentsChromaffin_cells.Chrom_SOX2_statusHigh
assignmentsChromaffin_cells.Chrom_SOX2_statusHigh.ASCL1	4.22110457	-0.0243315	5.31171961	1.11E-06	1.19E-05	5.33726233	ASCL1	assignmentsChromaffin_cells.Chrom_SOX2_statusHigh
assignmentsChromaffin_cells.Chrom_SOX2_statusHigh.GSTM4	1.90313025	2.29820602	5.32605702	1.04E-06	1.13E-05	5.33693384	GSTM4	assignmentsChromaffin_cells.Chrom_SOX2_statusHigh
assignmentsChromaffin_cells.Chrom_SOX2_statusHigh.HACD1	-2.0372386	3.03879549	-5.3269421	1.03E-06	1.13E-05	5.33364991	HACD1	assignmentsChromaffin_cells.Chrom_SOX2_statusHigh
assignmentsChromaffin_cells.Chrom_SOX2_statusHigh.STK16	1.4267851	2.40792579	5.33007642	1.02E-06	1.12E-05	5.3334448	STK16	assignmentsChromaffin_cells.Chrom_SOX2_statusHigh
assignmentsChromaffin_cells.Chrom_SOX2_statusHigh.PAN3	-1.1519836	8.07506503	-5.373722	8.59E-07	9.69E-06	5.33098501	PAN3	assignmentsChromaffin_cells.Chrom_SOX2_statusHigh
assignmentsChromaffin_cells.Chrom_SOX2_statusHigh.MED29	1.57148306	4.36663132	5.3475593	9.53E-07	1.05E-05	5.32677865	MED29	assignmentsChromaffin_cells.Chrom_SOX2_statusHigh
assignmentsChromaffin_cells.Chrom_SOX2_statusHigh.CFAP53	1.56344579	1.82749085	5.32485822	1.04E-06	1.14E-05	5.32577656	CFAP53	assignmentsChromaffin_cells.Chrom_SOX2_statusHigh
assignmentsChromaffin_cells.Chrom_SOX2_statusHigh.IFT20	1.34424257	4.37420768	5.34967763	9.45E-07	1.04E-05	5.31968516	IFT20	assignmentsChromaffin_cells.Chrom_SOX2_statusHigh
assignmentsChromaffin_cells.Chrom_SOX2_statusHigh.TUBB	2.28854186	5.43895547	5.35933046	9.12E-07	1.02E-05	5.3181672	TUBB	assignmentsChromaffin_cells.Chrom_SOX2_statusHigh
assignmentsChromaffin_cells.Chrom_SOX2_statusHigh.MYLK3	2.90038543	0.91918031	5.31241399	1.10E-06	1.18E-05	5.31513068	MYLK3	assignmentsChromaffin_cells.Chrom_SOX2_statusHigh
assignmentsChromaffin_cells.Chrom_SOX2_statusHigh.MIF4GD	1.74568004	2.82001199	5.32607508	1.04E-06	1.13E-05	5.30904426	MIF4GD	assignmentsChromaffin_cells.Chrom_SOX2_statusHigh
assignmentsChromaffin_cells.Chrom_SOX2_statusHigh.EPC2	-1.3488306	6.80161423	-5.3539244	9.30E-07	1.03E-05	5.30695632	EPC2	assignmentsChromaffin_cells.Chrom_SOX2_statusHigh
assignmentsChromaffin_cells.Chrom_SOX2_statusHigh.LSM7	2.01750783	4.84478681	5.35302494	9.34E-07	1.04E-05	5.30560201	LSM7	assignmentsChromaffin_cells.Chrom_SOX2_statusHigh
assignmentsChromaffin_cells.Chrom_SOX2_statusHigh.MORC3	-0.8334877	6.50178653	-5.3500854	9.44E-07	1.04E-05	5.29876323	MORC3	assignmentsChromaffin_cells.Chrom_SOX2_statusHigh
assignmentsChromaffin_cells.Chrom_SOX2_statusHigh.MAP4K4	-1.4797365	7.64116964	-5.3504214	9.43E-07	1.04E-05	5.29640324	MAP4K4	assignmentsChromaffin_cells.Chrom_SOX2_statusHigh
assignmentsChromaffin_cells.Chrom_SOX2_statusHigh.RTP4	3.60202489	-0.1049649	5.33527005	1.00E-06	1.10E-05	5.29063459	RTP4	assignmentsChromaffin_cells.Chrom_SOX2_statusHigh
assignmentsChromaffin_cells.Chrom_SOX2_statusHigh.WRN	-0.9151688	6.7009914	-5.3543754	9.28E-07	1.03E-05	5.28445005	WRN	assignmentsChromaffin_cells.Chrom_SOX2_statusHigh
assignmentsChromaffin_cells.Chrom_SOX2_statusHigh.TRIM47	3.63069935	1.46495252	5.30224426	1.14E-06	1.22E-05	5.28403263	TRIM47	assignmentsChromaffin_cells.Chrom_SOX2_statusHigh
assignmentsChromaffin_cells.Chrom_SOX2_statusHigh.NUBP2	1.25666136	3.63131272	5.33085278	1.02E-06	1.12E-05	5.27788464	NUBP2	assignmentsChromaffin_cells.Chrom_SOX2_statusHigh
assignmentsChromaffin_cells.Chrom_SOX2_statusHigh.NDUFA4	2.51164678	5.41292686	5.3537519	9.35E-07	1.04E-05	5.27615776	NDUFA4	assignmentsChromaffin_cells.Chrom_SOX2_statusHigh
assignmentsChromaffin_cells.Chrom_SOX2_statusHigh.PEF1	1.62762449	3.6866526	5.32653299	1.04E-06	1.13E-05	5.2719111	PEF1	assignmentsChromaffin_cells.Chrom_SOX2_statusHigh
assignmentsChromaffin_cells.Chrom_SOX2_statusHigh.AGAP1	-1.9090605	8.18381112	-5.358001	9.16E-07	1.02E-05	5.27017175	AGAP1	assignmentsChromaffin_cells.Chrom_SOX2_statusHigh
assignmentsChromaffin_cells.Chrom_SOX2_statusHigh.PDS5A	-1.0026984	7.58898487	-5.3543811	9.28E-07	1.03E-05	5.26592097	PDS5A	assignmentsChromaffin_cells.Chrom_SOX2_statusHigh
assignmentsChromaffin_cells.Chrom_SOX2_statusHigh.NRG2	-1.7133558	4.47191799	-5.3268895	1.04E-06	1.13E-05	5.25912755	NRG2	assignmentsChromaffin_cells.Chrom_SOX2_statusHigh
assignmentsChromaffin_cells.Chrom_SOX2_statusHigh.RIF1	-0.7285195	6.83401924	-5.3447842	9.64E-07	1.06E-05	5.2526282	RIF1	assignmentsChromaffin_cells.Chrom_SOX2_statusHigh
assignmentsChromaffin_cells.Chrom_SOX2_statusHigh.TMEM101	1.77970167	2.90463012	5.31845979	1.07E-06	1.16E-05	5.24938051	TMEM101	assignmentsChromaffin_cells.Chrom_SOX2_statusHigh

assignmentsChromaffin_cells.Chrom_SOX2_statusHigh.NKIRAS2	1.43700611	3.47387125	5.3156832	1.08E-06	1.17E-05	5.24737751	NKIRAS2	assignmentsChromaffin_cells.Chrom_SOX2_statusHigh
assignmentsChromaffin_cells.Chrom_SOX2_statusHigh.LARS2	-1.1992832	5.21275129	-5.3202317	1.06E-06	1.15E-05	5.23266685	LARS2	assignmentsChromaffin_cells.Chrom_SOX2_statusHigh
assignmentsChromaffin_cells.Chrom_SOX2_statusHigh.ARHGEF19	2.92620157	0.21749908	5.33155375	1.02E-06	1.11E-05	5.22860453	ARHGEF19	assignmentsChromaffin_cells.Chrom_SOX2_statusHigh
assignmentsChromaffin_cells.Chrom_SOX2_statusHigh.APEH	1.48298286	3.01562964	5.30963643	1.11E-06	1.19E-05	5.22714397	APEH	assignmentsChromaffin_cells.Chrom_SOX2_statusHigh
assignmentsChromaffin_cells.Chrom_SOX2_statusHigh.USP34	-0.8426937	8.8205142	-5.3569744	9.18E-07	1.02E-05	5.22552236	USP34	assignmentsChromaffin_cells.Chrom_SOX2_statusHigh
assignmentsChromaffin_cells.Chrom_SOX2_statusHigh.PPIC	2.24104543	2.27963434	5.29171531	1.19E-06	1.27E-05	5.22382868	PPIC	assignmentsChromaffin_cells.Chrom_SOX2_statusHigh
assignmentsChromaffin_cells.Chrom_SOX2_statusHigh.RPS6	3.09639635	7.28320571	5.35318452	9.37E-07	1.04E-05	5.21852778	RPS6	assignmentsChromaffin_cells.Chrom_SOX2_statusHigh
assignmentsChromaffin_cells.Chrom_SOX2_statusHigh.MGARP	3.76736721	0.34730131	5.28242004	1.24E-06	1.31E-05	5.21774188	MGARP	assignmentsChromaffin_cells.Chrom_SOX2_statusHigh
assignmentsChromaffin_cells.Chrom_SOX2_statusHigh.ERC1	-1.2596038	9.14036312	-5.3600609	9.07E-07	1.02E-05	5.21491052	ERC1	assignmentsChromaffin_cells.Chrom_SOX2_statusHigh
assignmentsChromaffin_cells.Chrom_SOX2_statusHigh.NAPA	1.37350516	6.27497501	5.34138918	9.77E-07	1.07E-05	5.20876137	NAPA	assignmentsChromaffin_cells.Chrom_SOX2_statusHigh
assignmentsChromaffin_cells.Chrom_SOX2_statusHigh.ANKS1A	-1.2816632	7.32283587	-5.329562	1.02E-06	1.12E-05	5.20746655	ANKS1A	assignmentsChromaffin_cells.Chrom_SOX2_statusHigh
assignmentsChromaffin_cells.Chrom_SOX2_statusHigh.TRAPPC11	-0.8490558	5.79755092	-5.3232096	1.05E-06	1.14E-05	5.20200621	TRAPPC11	assignmentsChromaffin_cells.Chrom_SOX2_statusHigh
assignmentsChromaffin_cells.Chrom_SOX2_statusHigh.MTMR1	-1.0904388	5.48965008	-5.3195261	1.07E-06	1.16E-05	5.19880938	MTMR1	assignmentsChromaffin_cells.Chrom_SOX2_statusHigh
assignmentsChromaffin_cells.Chrom_SOX2_statusHigh.FAM136A	1.29992915	3.90851259	5.30867584	1.11E-06	1.20E-05	5.19345776	FAM136A	assignmentsChromaffin_cells.Chrom_SOX2_statusHigh
assignmentsChromaffin_cells.Chrom_SOX2_statusHigh.MAPKAPK5-	1.78794608	3.4820833	5.30436508	1.13E-06	1.21E-05	5.18779555	MAPKAPK5-A	assignmentsChromaffin_cells.Chrom_SOX2_statusHigh
assignmentsChromaffin_cells.Chrom_SOX2_statusHigh.HMGN3	1.31362666	6.73622375	5.33830586	9.89E-07	1.09E-05	5.18211892	HMGN3	assignmentsChromaffin_cells.Chrom_SOX2_statusHigh
assignmentsChromaffin_cells.Chrom_SOX2_statusHigh.VPS35	0.98826457	5.86957381	5.3244974	1.04E-06	1.14E-05	5.18200375	VPS35	assignmentsChromaffin_cells.Chrom_SOX2_statusHigh
assignmentsChromaffin_cells.Chrom_SOX2_statusHigh.NAA25	-0.9248895	6.24948678	-5.3208005	1.06E-06	1.15E-05	5.17164098	NAA25	assignmentsChromaffin_cells.Chrom_SOX2_statusHigh
assignmentsChromaffin_cells.Chrom_SOX2_statusHigh.MRFAP1	2.64021799	4.83644216	5.31922426	1.07E-06	1.16E-05	5.16921594	MRFAP1	assignmentsChromaffin_cells.Chrom_SOX2_statusHigh
assignmentsChromaffin_cells.Chrom_SOX2_statusHigh.CNOT7	0.89373163	4.97916172	5.3164273	1.08E-06	1.17E-05	5.16919007	CNOT7	assignmentsChromaffin_cells.Chrom_SOX2_statusHigh
assignmentsChromaffin_cells.Chrom_SOX2_statusHigh.ANAPC1	-1.0218806	5.47426462	-5.3067001	1.12E-06	1.20E-05	5.1612653	ANAPC1	assignmentsChromaffin_cells.Chrom_SOX2_statusHigh
assignmentsChromaffin_cells.Chrom_SOX2_statusHigh.AVL9	-1.0548859	7.09085741	-5.3233866	1.05E-06	1.14E-05	5.15273362	AVL9	assignmentsChromaffin_cells.Chrom_SOX2_statusHigh
assignmentsChromaffin_cells.Chrom_SOX2_statusHigh.ARMCX1	1.6210441	4.032205	5.30493369	1.13E-06	1.21E-05	5.14443774	ARMCX1	assignmentsChromaffin_cells.Chrom_SOX2_statusHigh
assignmentsChromaffin_cells.Chrom_SOX2_statusHigh.PSMA4	1.32648706	5.13283216	5.30937109	1.11E-06	1.19E-05	5.14365124	PSMA4	assignmentsChromaffin_cells.Chrom_SOX2_statusHigh
assignmentsChromaffin_cells.Chrom_SOX2_statusHigh.ARF3	1.06110883	5.02815959	5.30607237	1.12E-06	1.20E-05	5.13914749	ARF3	assignmentsChromaffin_cells.Chrom_SOX2_statusHigh
assignmentsChromaffin_cells.Chrom_SOX2_statusHigh.RPS19	3.08251464	7.36143118	5.33322806	1.01E-06	1.11E-05	5.13782804	RPS19	assignmentsChromaffin_cells.Chrom_SOX2_statusHigh
assignmentsChromaffin_cells.Chrom_SOX2_statusHigh.OAS1	2.76920032	2.72933771	5.27088796	1.30E-06	1.35E-05	5.12733769	OAS1	assignmentsChromaffin_cells.Chrom_SOX2_statusHigh
assignmentsChromaffin_cells.Chrom_SOX2_statusHigh.PSMD4	1.04506464	5.26477533	5.30738059	1.12E-06	1.20E-05	5.12613229	PSMD4	assignmentsChromaffin_cells.Chrom_SOX2_statusHigh
assignmentsChromaffin_cells.Chrom_SOX2_statusHigh.MDM4	-0.6830703	7.09946268	-5.3148392	1.09E-06	1.17E-05	5.12259678	MDM4	assignmentsChromaffin_cells.Chrom_SOX2_statusHigh
assignmentsChromaffin_cells.Chrom_SOX2_statusHigh.CLEC11A	2.38576641	3.37966868	5.28584214	1.22E-06	1.29E-05	5.11174429	CLEC11A	assignmentsChromaffin_cells.Chrom_SOX2_statusHigh
assignmentsChromaffin_cells.Chrom_SOX2_statusHigh.ETV2	3.81335104	-0.0743208	5.24979622	1.41E-06	1.45E-05	5.10666508	ETV2	assignmentsChromaffin_cells.Chrom_SOX2_statusHigh
assignmentsChromaffin_cells.Chrom_SOX2_statusHigh.TAGLN3	2.39079183	2.48067222	5.28922179	1.20E-06	1.28E-05	5.10648865	TAGLN3	assignmentsChromaffin_cells.Chrom_SOX2_statusHigh
assignmentsChromaffin_cells.Chrom_SOX2_statusHigh.BBS10	1.33680179	2.14566473	5.2645808	1.33E-06	1.38E-05	5.10136453	BBS10	assignmentsChromaffin_cells.Chrom_SOX2_statusHigh
assignmentsChromaffin_cells.Chrom_SOX2_statusHigh.CAMLG	1.56887413	5.05939964	5.30141234	1.15E-06	1.22E-05	5.09997787	CAMLG	assignmentsChromaffin_cells.Chrom_SOX2_statusHigh
assignmentsChromaffin_cells.Chrom_SOX2_statusHigh.UTP23	1.26272054	4.71698383	5.29511176	1.17E-06	1.25E-05	5.09855578	UTP23	assignmentsChromaffin_cells.Chrom_SOX2_statusHigh
assignmentsChromaffin_cells.Chrom_SOX2_statusHigh.APEX2	1.40029413	2.2167095	5.26260201	1.34E-06	1.39E-05	5.09775726	APEX2	assignmentsChromaffin_cells.Chrom_SOX2_statusHigh
assignmentsChromaffin_cells.Chrom_SOX2_statusHigh.FEN1	2.14273595	1.54513127	5.25790776	1.36E-06	1.41E-05	5.09542185	FEN1	assignmentsChromaffin_cells.Chrom_SOX2_statusHigh
assignmentsChromaffin_cells.Chrom_SOX2_statusHigh.TLE5	1.77917843	5.71731316	5.30111094	1.15E-06	1.22E-05	5.09070039	TLE5	assignmentsChromaffin_cells.Chrom_SOX2_statusHigh
assignmentsChromaffin_cells.Chrom_SOX2_statusHigh.KLHL5	-1.3633049	7.13363995	-5.3007938	1.15E-06	1.22E-05	5.08233828	KLHL5	assignmentsChromaffin_cells.Chrom_SOX2_statusHigh
assignmentsChromaffin_cells.Chrom_SOX2_statusHigh.ST8SIA2	-4.1298624	2.25732556	-5.2505882	1.41E-06	1.45E-05	5.08160163	ST8SIA2	assignmentsChromaffin_cells.Chrom_SOX2_statusHigh
assignmentsChromaffin_cells.Chrom_SOX2_statusHigh.PHF21A	-1.243283	7.74168429	-5.3060485	1.12E-06	1.20E-05	5.08131839	PHF21A	assignmentsChromaffin_cells.Chrom_SOX2_statusHigh
assignmentsChromaffin_cells.Chrom_SOX2_statusHigh.MRPS36	1.38097677	4.72174058	5.29170009	1.19E-06	1.27E-05	5.08029622	MRPS36	assignmentsChromaffin_cells.Chrom_SOX2_statusHigh
assignmentsChromaffin_cells.Chrom_SOX2_statusHigh.COPS9	2.84111852	3.73155659	5.28328482	1.24E-06	1.31E-05	5.07799636	COPS9	assignmentsChromaffin_cells.Chrom_SOX2_statusHigh
assignmentsChromaffin_cells.Chrom_SOX2_statusHigh.TBL1X	-1.6913108	8.20283669	-5.3141647	1.09E-06	1.18E-05	5.07649057	TBL1X	assignmentsChromaffin_cells.Chrom_SOX2_statusHigh
assignmentsChromaffin_cells.Chrom_SOX2_statusHigh.ICA1L	-1.7305946	5.69328967	-5.293702	1.18E-06	1.26E-05	5.0613203	ICA1L	assignmentsChromaffin_cells.Chrom_SOX2_statusHigh
assignmentsChromaffin_cells.Chrom_SOX2_statusHigh.MRPS18C	1.46285784	4.41592829	5.27968754	1.25E-06	1.31E-05	5.05185521	MRPS18C	assignmentsChromaffin_cells.Chrom_SOX2_statusHigh
assignmentsChromaffin_cells.Chrom_SOX2_statusHigh.RSPRY1	-0.9187257	5.96257594	-5.2859935	1.22E-06	1.29E-05	5.05103984	RSPRY1	assignmentsChromaffin_cells.Chrom_SOX2_statusHigh
assignmentsChromaffin_cells.Chrom_SOX2_statusHigh.IMMP2L	-1.2211264	9.04233374	-5.3095491	1.11E-06	1.19E-05	5.04952001	IMMP2L	assignmentsChromaffin_cells.Chrom_SOX2_statusHigh
assignmentsChromaffin_cells.Chrom_SOX2_statusHigh.TRIM59	1.32816716	2.81190565	5.26034227	1.35E-06	1.40E-05	5.04700714	TRIM59	assignmentsChromaffin_cells.Chrom_SOX2_statusHigh
assignmentsChromaffin_cells.Chrom_SOX2_statusHigh.SMIM7	1.10689236	5.75931475	5.29149546	1.19E-06	1.27E-05	5.04488076	SMIM7	assignmentsChromaffin_cells.Chrom_SOX2_statusHigh
assignmentsChromaffin_cells.Chrom_SOX2_statusHigh.RFLNA	-1.545821	4.34509199	-5.2730357	1.28E-06	1.34E-05	5.04386648	RFLNA	assignmentsChromaffin_cells.Chrom_SOX2_statusHigh
assignmentsChromaffin_cells.Chrom_SOX2_statusHigh.RFXANK	1.70491086	2.94000736	5.25833916	1.36E-06	1.41E-05	5.04335958	RFXANK	assignmentsChromaffin_cells.Chrom_SOX2_statusHigh
assignmentsChromaffin_cells.Chrom_SOX2_statusHigh.SEC61G	2.76836062	4.9742547	5.29040793	1.20E-06	1.28E-05	5.04207072	SEC61G	assignmentsChromaffin_cells.Chrom_SOX2_statusHigh
assignmentsChromaffin_cells.Chrom_SOX2_statusHigh.RPL37	2.87692401	7.06762302	5.30492324	1.14E-06	1.21E-05	5.04118742	RPL37	assignmentsChromaffin_cells.Chrom_SOX2_statusHigh
assignmentsChromaffin_cells.Chrom_SOX2_statusHigh.GNG10	1.81293325	4.83531685	5.27861929	1.25E-06	1.32E-05	5.0348903	GNG10	assignmentsChromaffin_cells.Chrom_SOX2_statusHigh
assignmentsChromaffin_cells.Chrom_SOX2_statusHigh.TRAF3IP2-A	-0.9622538	6.76231497	-5.2869553	1.21E-06	1.28E-05	5.03357601	TRAF3IP2-AS	assignmentsChromaffin_cells.Chrom_SOX2_statusHigh
assignmentsChromaffin_cells.Chrom_SOX2_statusHigh.TUBG1	1.78953359	2.86355029	5.25885861	1.36E-06	1.40E-05	5.03321149	TUBG1	assignmentsChromaffin_cells.Chrom_SOX2_statusHigh
assignmentsChromaffin_cells.Chrom_SOX2_statusHigh.MAPK14	-0.8843299	6.78203429	-5.2844631	1.22E-06	1.29E-05	5.0324459	MAPK14	assignmentsChromaffin_cells.Chrom_SOX2_statusHigh
assignmentsChromaffin_cells.Chrom_SOX2_statusHigh.POLD2	1.46909677	3.99621135	5.27198359	1.29E-06	1.35E-05	5.03167455	POLD2	assignmentsChromaffin_cells.Chrom_SOX2_statusHigh
assignmentsChromaffin_cells.Chrom_SOX2_statusHigh.C1orf109	1.2812212	2.74413137	5.25178343	1.39E-06	1.44E-05	5.02438428	C1orf109	assignmentsChromaffin_cells.Chrom_SOX2_statusHigh
assignmentsChromaffin_cells.Chrom_SOX2_statusHigh.CHCHD10	3.92289517	3.96853403	5.26389726	1.34E-06	1.39E-05	5.02402904	CHCHD10	assignmentsChromaffin_cells.Chrom_SOX2_statusHigh
assignmentsChromaffin_cells.Chrom_SOX2_statusHigh.MICU1	-0.8798321	6.81370058	-5.2846845	1.22E-06	1.29E-05	5.01958977	MICU1	assignmentsChromaffin_cells.Chrom_SOX2_statusHigh
assignmentsChromaffin_cells.Chrom_SOX2_statusHigh.ILVBL	1.67940381	3.58354504	5.26921971	1.30E-06	1.36E-05	5.01907343	ILVBL	assignmentsChromaffin_cells.Chrom_SOX2_statusHigh
assignmentsChromaffin_cells.Chrom_SOX2_statusHigh.CTDSPL2	-0.9087069	6.47111693	-5.2808136	1.24E-06	1.31E-05	5.01797315	CTDSPL2	assignmentsChromaffin_cells.Chrom_SOX2_statusHigh

assignmentsChromaffin_cells.Chrom_SOX2_statusHigh.CHST14	2.15098283	1.18551305	5.22876546	1.53E-06	1.55E-05	5.01694633	CHST14	assignmentsChromaffin_cells.Chrom_SOX2_statusHigh
assignmentsChromaffin_cells.Chrom_SOX2_statusHigh.NUMB	-1.0977817	7.78786917	-5.2884202	1.21E-06	1.28E-05	5.01590607	NUMB	assignmentsChromaffin_cells.Chrom_SOX2_statusHigh
assignmentsChromaffin_cells.Chrom_SOX2_statusHigh.TCF20	-1.334681	6.12728016	-5.273522	1.28E-06	1.34E-05	5.01078792	TCF20	assignmentsChromaffin_cells.Chrom_SOX2_statusHigh
assignmentsChromaffin_cells.Chrom_SOX2_statusHigh.TIMM50	1.13076901	3.78110234	5.26125486	1.34E-06	1.39E-05	5.0046341	TIMM50	assignmentsChromaffin_cells.Chrom_SOX2_statusHigh
assignmentsChromaffin_cells.Chrom_SOX2_statusHigh.AKR1B1	1.87374762	4.58471466	5.26597258	1.32E-06	1.37E-05	4.99870337	AKR1B1	assignmentsChromaffin_cells.Chrom_SOX2_statusHigh
assignmentsChromaffin_cells.Chrom_SOX2_statusHigh.ARL15	-1.5989735	8.7933096	-5.2858149	1.22E-06	1.29E-05	4.99726598	ARL15	assignmentsChromaffin_cells.Chrom_SOX2_statusHigh
assignmentsChromaffin_cells.Chrom_SOX2_statusHigh.RPS14	3.32539795	7.37353396	5.29701673	1.17E-06	1.25E-05	4.99587266	RPS14	assignmentsChromaffin_cells.Chrom_SOX2_statusHigh
assignmentsChromaffin_cells.Chrom_SOX2_statusHigh.TNXB	-2.5922343	3.64671222	-5.2346793	1.49E-06	1.52E-05	4.99521148	TNXB	assignmentsChromaffin_cells.Chrom_SOX2_statusHigh
assignmentsChromaffin_cells.Chrom_SOX2_statusHigh.STK39	-1.3312725	6.95022656	-5.280328	1.25E-06	1.31E-05	4.99305538	STK39	assignmentsChromaffin_cells.Chrom_SOX2_statusHigh
assignmentsChromaffin_cells.Chrom_SOX2_statusHigh.MT1X	5.45714025	2.04303912	5.22018032	1.59E-06	1.59E-05	4.99276961	MT1X	assignmentsChromaffin_cells.Chrom_SOX2_statusHigh
assignmentsChromaffin_cells.Chrom_SOX2_statusHigh.CHURC1	1.012994	5.7289183	5.27312706	1.28E-06	1.34E-05	4.99045257	CHURC1	assignmentsChromaffin_cells.Chrom_SOX2_statusHigh
assignmentsChromaffin_cells.Chrom_SOX2_statusHigh.ANG	2.85409984	0.31684132	5.21583002	1.61E-06	1.61E-05	4.98770265	ANG	assignmentsChromaffin_cells.Chrom_SOX2_statusHigh
assignmentsChromaffin_cells.Chrom_SOX2_statusHigh.NOL7	1.40106474	5.64721226	5.2721672	1.29E-06	1.35E-05	4.98108345	NOL7	assignmentsChromaffin_cells.Chrom_SOX2_statusHigh
assignmentsChromaffin_cells.Chrom_SOX2_statusHigh.GFRA2	-2.3624466	4.67988081	-5.2536307	1.39E-06	1.43E-05	4.97968551	GFRA2	assignmentsChromaffin_cells.Chrom_SOX2_statusHigh
assignmentsChromaffin_cells.Chrom_SOX2_statusHigh.PLEKHH1	-2.0389808	3.49312638	-5.235264	1.49E-06	1.52E-05	4.97179669	PLEKHH1	assignmentsChromaffin_cells.Chrom_SOX2_statusHigh
assignmentsChromaffin_cells.Chrom_SOX2_statusHigh.PIKFYVE	-0.8999526	6.8429094	-5.2697009	1.30E-06	1.36E-05	4.97129799	PIKFYVE	assignmentsChromaffin_cells.Chrom_SOX2_statusHigh
assignmentsChromaffin_cells.Chrom_SOX2_statusHigh.CCDC110	2.05133455	0.84558	5.21726099	1.60E-06	1.60E-05	4.97078951	CCDC110	assignmentsChromaffin_cells.Chrom_SOX2_statusHigh
assignmentsChromaffin_cells.Chrom_SOX2_statusHigh.SRPK1	-0.7031258	5.94358856	-5.2628056	1.33E-06	1.39E-05	4.96562159	SRPK1	assignmentsChromaffin_cells.Chrom_SOX2_statusHigh
assignmentsChromaffin_cells.Chrom_SOX2_statusHigh.ZFAS1	1.91363854	6.32140581	5.27555433	1.27E-06	1.33E-05	4.96219444	ZFAS1	assignmentsChromaffin_cells.Chrom_SOX2_statusHigh
assignmentsChromaffin_cells.Chrom_SOX2_statusHigh.TRIM28	1.35147017	4.37649275	5.25362734	1.38E-06	1.43E-05	4.95982901	TRIM28	assignmentsChromaffin_cells.Chrom_SOX2_statusHigh
assignmentsChromaffin_cells.Chrom_SOX2_statusHigh.MITF	-2.3443339	5.59447797	-5.2335898	1.50E-06	1.52E-05	4.95747219	MITF	assignmentsChromaffin_cells.Chrom_SOX2_statusHigh
assignmentsChromaffin_cells.Chrom_SOX2_statusHigh.SPCS2	1.51860015	6.36827177	5.27477365	1.27E-06	1.34E-05	4.95512275	SPCS2	assignmentsChromaffin_cells.Chrom_SOX2_statusHigh
assignmentsChromaffin_cells.Chrom_SOX2_statusHigh.ADAM23	-2.4435758	5.85813295	-5.2612963	1.35E-06	1.40E-05	4.95507347	ADAM23	assignmentsChromaffin_cells.Chrom_SOX2_statusHigh
assignmentsChromaffin_cells.Chrom_SOX2_statusHigh.TMEM120A	1.5986307	3.72504606	5.24688196	1.42E-06	1.46E-05	4.95211131	TMEM120A	assignmentsChromaffin_cells.Chrom_SOX2_statusHigh
assignmentsChromaffin_cells.Chrom_SOX2_statusHigh.GUK1	2.23782767	5.86578251	5.26859251	1.31E-06	1.37E-05	4.95152285	GUK1	assignmentsChromaffin_cells.Chrom_SOX2_statusHigh
assignmentsChromaffin_cells.Chrom_SOX2_statusHigh.GNPDA1	1.2193668	4.21571286	5.25000055	1.40E-06	1.45E-05	4.95041611	GNPDA1	assignmentsChromaffin_cells.Chrom_SOX2_statusHigh
assignmentsChromaffin_cells.Chrom_SOX2_statusHigh.AGTPBP1	-1.2330676	6.99979617	-5.2631523	1.33E-06	1.39E-05	4.94943595	AGTPBP1	assignmentsChromaffin_cells.Chrom_SOX2_statusHigh
assignmentsChromaffin_cells.Chrom_SOX2_statusHigh.THAP5	1.20696441	4.28442426	5.25253183	1.39E-06	1.43E-05	4.94830781	THAP5	assignmentsChromaffin_cells.Chrom_SOX2_statusHigh
assignmentsChromaffin_cells.Chrom_SOX2_statusHigh.ST6GALNAC	3.3431317	1.76402483	5.21180776	1.64E-06	1.63E-05	4.94766859	ST6GALNAC4	assignmentsChromaffin_cells.Chrom_SOX2_statusHigh
assignmentsChromaffin_cells.Chrom_SOX2_statusHigh.URM1	0.87344855	4.27536056	5.24728479	1.42E-06	1.46E-05	4.94483085	URM1	assignmentsChromaffin_cells.Chrom_SOX2_statusHigh
assignmentsChromaffin_cells.Chrom_SOX2_statusHigh.INO80	-1.0159389	6.68140394	-5.2620457	1.34E-06	1.39E-05	4.94351884	INO80	assignmentsChromaffin_cells.Chrom_SOX2_statusHigh
assignmentsChromaffin_cells.Chrom_SOX2_statusHigh.NFKB2	1.8901788	3.59778932	5.22113227	1.57E-06	1.58E-05	4.94317819	NFKB2	assignmentsChromaffin_cells.Chrom_SOX2_statusHigh
assignmentsChromaffin_cells.Chrom_SOX2_statusHigh.MFAP1	1.11207925	4.52571667	5.24690131	1.42E-06	1.46E-05	4.93652867	MFAP1	assignmentsChromaffin_cells.Chrom_SOX2_statusHigh
assignmentsChromaffin_cells.Chrom_SOX2_statusHigh.SND1	-0.9425512	8.24239453	-5.2780243	1.26E-06	1.32E-05	4.93481999	SND1	assignmentsChromaffin_cells.Chrom_SOX2_statusHigh
assignmentsChromaffin_cells.Chrom_SOX2_statusHigh.CCND2-AS1	-3.1115801	2.91935253	-5.2174663	1.60E-06	1.61E-05	4.93342816	CCND2-AS1	assignmentsChromaffin_cells.Chrom_SOX2_statusHigh
assignmentsChromaffin_cells.Chrom_SOX2_statusHigh.CHIC1	-0.9577326	5.98529198	-5.256948	1.37E-06	1.41E-05	4.93047717	CHIC1	assignmentsChromaffin_cells.Chrom_SOX2_statusHigh
assignmentsChromaffin_cells.Chrom_SOX2_statusHigh.SNRNP27	1.66118946	4.11830048	5.24501406	1.43E-06	1.47E-05	4.93006682	SNRNP27	assignmentsChromaffin_cells.Chrom_SOX2_statusHigh
assignmentsChromaffin_cells.Chrom_SOX2_statusHigh.PARD6A	2.35064707	1.8393469	5.22658399	1.54E-06	1.56E-05	4.92730179	PARD6A	assignmentsChromaffin_cells.Chrom_SOX2_statusHigh
assignmentsChromaffin_cells.Chrom_SOX2_statusHigh.LDAH	-1.1591726	6.02393744	-5.2539322	1.38E-06	1.43E-05	4.92630407	LDAH	assignmentsChromaffin_cells.Chrom_SOX2_statusHigh
assignmentsChromaffin_cells.Chrom_SOX2_statusHigh.BORCS7	1.28579356	4.78059394	5.24720448	1.42E-06	1.46E-05	4.91183026	BORCS7	assignmentsChromaffin_cells.Chrom_SOX2_statusHigh
assignmentsChromaffin_cells.Chrom_SOX2_statusHigh.DDX41	1.38625463	3.09140307	5.22250425	1.56E-06	1.58E-05	4.90625203	DDX41	assignmentsChromaffin_cells.Chrom_SOX2_statusHigh
assignmentsChromaffin_cells.Chrom_SOX2_statusHigh.GDF15	3.40496202	1.27502296	5.19247664	1.76E-06	1.75E-05	4.90311099	GDF15	assignmentsChromaffin_cells.Chrom_SOX2_statusHigh
assignmentsChromaffin_cells.Chrom_SOX2_statusHigh.TRAPPC2B	1.72882288	3.22249069	5.22834453	1.53E-06	1.55E-05	4.90299147	TRAPPC2B	assignmentsChromaffin_cells.Chrom_SOX2_statusHigh
assignmentsChromaffin_cells.Chrom_SOX2_statusHigh.COX7B	2.12370288	5.32283741	5.25047099	1.40E-06	1.45E-05	4.88955304	COX7B	assignmentsChromaffin_cells.Chrom_SOX2_statusHigh
assignmentsChromaffin_cells.Chrom_SOX2_statusHigh.MRPL47	1.67198137	4.07454631	5.23037724	1.52E-06	1.54E-05	4.88635539	MRPL47	assignmentsChromaffin_cells.Chrom_SOX2_statusHigh
assignmentsChromaffin_cells.Chrom_SOX2_statusHigh.TUBB4B	2.45195162	4.6791502	5.23507804	1.49E-06	1.52E-05	4.88554363	TUBB4B	assignmentsChromaffin_cells.Chrom_SOX2_statusHigh
assignmentsChromaffin_cells.Chrom_SOX2_statusHigh.ITSN1	-1.330619	7.23178784	-5.2441827	1.44E-06	1.47E-05	4.87598554	ITSN1	assignmentsChromaffin_cells.Chrom_SOX2_statusHigh
assignmentsChromaffin_cells.Chrom_SOX2_statusHigh.N4BP1	-0.9845642	6.46735547	-5.2376564	1.47E-06	1.50E-05	4.86998754	N4BP1	assignmentsChromaffin_cells.Chrom_SOX2_statusHigh
assignmentsChromaffin_cells.Chrom_SOX2_statusHigh.MCRIP2	1.69582691	2.80109447	5.21863461	1.59E-06	1.60E-05	4.86339982	MCRIP2	assignmentsChromaffin_cells.Chrom_SOX2_statusHigh
assignmentsChromaffin_cells.Chrom_SOX2_statusHigh.MAPK1	-0.993421	7.06560555	-5.2398169	1.46E-06	1.49E-05	4.86081112	MAPK1	assignmentsChromaffin_cells.Chrom_SOX2_statusHigh
assignmentsChromaffin_cells.Chrom_SOX2_statusHigh.MIEF2	1.5552307	1.73009828	5.1990419	1.72E-06	1.70E-05	4.85869806	MIEF2	assignmentsChromaffin_cells.Chrom_SOX2_statusHigh
assignmentsChromaffin_cells.Chrom_SOX2_statusHigh.MTHFSD	-1.1739879	3.90857773	-5.2080919	1.66E-06	1.65E-05	4.8574066	MTHFSD	assignmentsChromaffin_cells.Chrom_SOX2_statusHigh
assignmentsChromaffin_cells.Chrom_SOX2_statusHigh.TVP23C	-1.6664277	6.98716221	-5.2419264	1.45E-06	1.48E-05	4.85132578	TVP23C	assignmentsChromaffin_cells.Chrom_SOX2_statusHigh
assignmentsChromaffin_cells.Chrom_SOX2_statusHigh.NCBP2	1.29488359	4.25446072	5.22065398	1.58E-06	1.59E-05	4.85091288	NCBP2	assignmentsChromaffin_cells.Chrom_SOX2_statusHigh
assignmentsChromaffin_cells.Chrom_SOX2_statusHigh.ARHGAP32	-1.2617299	6.65590391	-5.2373272	1.48E-06	1.50E-05	4.84155855	ARHGAP32	assignmentsChromaffin_cells.Chrom_SOX2_statusHigh
assignmentsChromaffin_cells.Chrom_SOX2_statusHigh.VPS72	1.13661933	3.69391326	5.21430793	1.62E-06	1.62E-05	4.83349435	VPS72	assignmentsChromaffin_cells.Chrom_SOX2_statusHigh
assignmentsChromaffin_cells.Chrom_SOX2_statusHigh.LINC00342	-1.371496	7.22884934	-5.240474	1.46E-06	1.49E-05	4.82980675	LINC00342	assignmentsChromaffin_cells.Chrom_SOX2_statusHigh
assignmentsChromaffin_cells.Chrom_SOX2_statusHigh.RAB12	-1.1083204	5.93988312	-5.2267473	1.54E-06	1.56E-05	4.82898935	RAB12	assignmentsChromaffin_cells.Chrom_SOX2_statusHigh
assignmentsChromaffin_cells.Chrom_SOX2_statusHigh.RPS26	3.2133137	6.4610933	5.24497706	1.44E-06	1.47E-05	4.82721087	RPS26	assignmentsChromaffin_cells.Chrom_SOX2_statusHigh
assignmentsChromaffin_cells.Chrom_SOX2_statusHigh.TNRC6B	-0.9563003	8.58594093	-5.2495105	1.41E-06	1.45E-05	4.82701315	TNRC6B	assignmentsChromaffin_cells.Chrom_SOX2_statusHigh
assignmentsChromaffin_cells.Chrom_SOX2_statusHigh.ARID2	-0.8020264	7.87824024	-5.2420776	1.45E-06	1.48E-05	4.82511111	ARID2	assignmentsChromaffin_cells.Chrom_SOX2_statusHigh
assignmentsChromaffin_cells.Chrom_SOX2_statusHigh.TNRC18	-1.6733658	6.57836912	-5.2241327	1.55E-06	1.57E-05	4.82321826	TNRC18	assignmentsChromaffin_cells.Chrom_SOX2_statusHigh
assignmentsChromaffin_cells.Chrom_SOX2_statusHigh.FES	3.07411743	2.56223566	5.16912736	1.93E-06	1.89E-05	4.81915114	FES	assignmentsChromaffin_cells.Chrom_SOX2_statusHigh
assignmentsChromaffin_cells.Chrom_SOX2_statusHigh.RHOG	1.92433432	3.43431106	5.19158863	1.77E-06	1.75E-05	4.81514603	RHOG	assignmentsChromaffin_cells.Chrom_SOX2_statusHigh

assignmentsChromaffin_cells.Chrom_SOX2_statusHigh.DPP7	1.72745902	5.54508385	5.23072337	1.51E-06	1.54E-05	4.81260749	DPP7	assignmentsChromaffin_cells.Chrom_SOX2_statusHigh
assignmentsChromaffin_cells.Chrom_SOX2_statusHigh.SNHG4	3.49647789	1.76717594	5.16733979	1.95E-06	1.90E-05	4.80767298	SNHG4	assignmentsChromaffin_cells.Chrom_SOX2_statusHigh
assignmentsChromaffin_cells.Chrom_SOX2_statusHigh.TRPT1	2.10693915	2.95270801	5.1960074	1.74E-06	1.72E-05	4.79828095	TRPT1	assignmentsChromaffin_cells.Chrom_SOX2_statusHigh
assignmentsChromaffin_cells.Chrom_SOX2_statusHigh.SYNGR2	2.06282266	3.8202096	5.20329173	1.69E-06	1.68E-05	4.79700204	SYNGR2	assignmentsChromaffin_cells.Chrom_SOX2_statusHigh
assignmentsChromaffin_cells.Chrom_SOX2_statusHigh.MFAP4	4.93919506	0.2949931	5.16521522	1.97E-06	1.92E-05	4.79240369	MFAP4	assignmentsChromaffin_cells.Chrom_SOX2_statusHigh
assignmentsChromaffin_cells.Chrom_SOX2_statusHigh.CCT8	1.29632291	5.80821606	5.22469118	1.55E-06	1.57E-05	4.79070257	CCT8	assignmentsChromaffin_cells.Chrom_SOX2_statusHigh
assignmentsChromaffin_cells.Chrom_SOX2_statusHigh.EMSY	-1.1407852	6.7016095	-5.2221641	1.57E-06	1.58E-05	4.79034775	EMSY	assignmentsChromaffin_cells.Chrom_SOX2_statusHigh
assignmentsChromaffin_cells.Chrom_SOX2_statusHigh.CLIP1	-1.0058572	7.10837721	-5.2266189	1.54E-06	1.56E-05	4.79025165	CLIP1	assignmentsChromaffin_cells.Chrom_SOX2_statusHigh
assignmentsChromaffin_cells.Chrom_SOX2_statusHigh.NKD1	4.71512879	2.55251941	5.17086586	1.93E-06	1.88E-05	4.78556255	NKD1	assignmentsChromaffin_cells.Chrom_SOX2_statusHigh
assignmentsChromaffin_cells.Chrom_SOX2_statusHigh.ADGRA3	-1.556934	5.12137147	-5.205942	1.67E-06	1.66E-05	4.77949288	ADGRA3	assignmentsChromaffin_cells.Chrom_SOX2_statusHigh
assignmentsChromaffin_cells.Chrom_SOX2_statusHigh.RTF2	1.03665016	5.60084285	5.21466845	1.61E-06	1.62E-05	4.77881033	RTF2	assignmentsChromaffin_cells.Chrom_SOX2_statusHigh
assignmentsChromaffin_cells.Chrom_SOX2_statusHigh.THAP7	1.53661365	3.14534352	5.1976273	1.73E-06	1.71E-05	4.77513225	THAP7	assignmentsChromaffin_cells.Chrom_SOX2_statusHigh
assignmentsChromaffin_cells.Chrom_SOX2_statusHigh.ADCYAP1R1	-4.0265821	3.34118502	-5.1785546	1.87E-06	1.84E-05	4.77152655	ADCYAP1R1	assignmentsChromaffin_cells.Chrom_SOX2_statusHigh
assignmentsChromaffin_cells.Chrom_SOX2_statusHigh.AHCYL2	-1.190494	7.18946816	-5.2252882	1.55E-06	1.56E-05	4.77114632	AHCYL2	assignmentsChromaffin_cells.Chrom_SOX2_statusHigh
assignmentsChromaffin_cells.Chrom_SOX2_statusHigh.OTOGL	-3.7726208	1.93361882	-5.1580126	2.02E-06	1.96E-05	4.76810007	OTOGL	assignmentsChromaffin_cells.Chrom_SOX2_statusHigh
assignmentsChromaffin_cells.Chrom_SOX2_statusHigh.EIF4EBP1	1.49707645	3.66090369	5.19485163	1.75E-06	1.73E-05	4.76724008	EIF4EBP1	assignmentsChromaffin_cells.Chrom_SOX2_statusHigh
assignmentsChromaffin_cells.Chrom_SOX2_statusHigh.COQ10A	1.29989159	3.37009361	5.19308235	1.76E-06	1.74E-05	4.75709404	COQ10A	assignmentsChromaffin_cells.Chrom_SOX2_statusHigh
assignmentsChromaffin_cells.Chrom_SOX2_statusHigh.APRT	2.44459198	4.09834515	5.19677357	1.74E-06	1.72E-05	4.7558843	APRT	assignmentsChromaffin_cells.Chrom_SOX2_statusHigh
assignmentsChromaffin_cells.Chrom_SOX2_statusHigh.CSMD2	-3.8858087	3.36084283	-5.1577243	2.03E-06	1.96E-05	4.75349645	CSMD2	assignmentsChromaffin_cells.Chrom_SOX2_statusHigh
assignmentsChromaffin_cells.Chrom_SOX2_statusHigh.MED14	-0.7811903	6.18103285	-5.2027017	1.69E-06	1.68E-05	4.75100245	MED14	assignmentsChromaffin_cells.Chrom_SOX2_statusHigh
assignmentsChromaffin_cells.Chrom_SOX2_statusHigh.MISP3	1.99651973	0.49186382	5.15242266	2.06E-06	1.99E-05	4.74771463	MISP3	assignmentsChromaffin_cells.Chrom_SOX2_statusHigh
assignmentsChromaffin_cells.Chrom_SOX2_statusHigh.TCTA	1.3403541	3.49294672	5.1874948	1.80E-06	1.77E-05	4.74478188	TCTA	assignmentsChromaffin_cells.Chrom_SOX2_statusHigh
assignmentsChromaffin_cells.Chrom_SOX2_statusHigh.EIF5A	2.51610319	4.24406115	5.19274031	1.77E-06	1.75E-05	4.73398868	EIF5A	assignmentsChromaffin_cells.Chrom_SOX2_statusHigh
assignmentsChromaffin_cells.Chrom_SOX2_statusHigh.CALM2	1.96799854	7.94813992	5.2345129	1.50E-06	1.52E-05	4.73164305	CALM2	assignmentsChromaffin_cells.Chrom_SOX2_statusHigh
assignmentsChromaffin_cells.Chrom_SOX2_statusHigh.AMACR	1.27012248	2.5939001	5.16989978	1.92E-06	1.88E-05	4.72976994	AMACR	assignmentsChromaffin_cells.Chrom_SOX2_statusHigh
assignmentsChromaffin_cells.Chrom_SOX2_statusHigh.COG5	-1.1105411	8.33467105	-5.2238855	1.56E-06	1.57E-05	4.72678397	COG5	assignmentsChromaffin_cells.Chrom_SOX2_statusHigh
assignmentsChromaffin_cells.Chrom_SOX2_statusHigh.ANKRD36	-1.8736392	7.81334164	-5.2166203	1.60E-06	1.61E-05	4.7237439	ANKRD36	assignmentsChromaffin_cells.Chrom_SOX2_statusHigh
assignmentsChromaffin_cells.Chrom_SOX2_statusHigh.ENY2	1.57009494	5.63110179	5.2037159	1.69E-06	1.68E-05	4.71723525	ENY2	assignmentsChromaffin_cells.Chrom_SOX2_statusHigh
assignmentsChromaffin_cells.Chrom_SOX2_statusHigh.FARSA	1.23043798	4.05384213	5.18609517	1.81E-06	1.78E-05	4.71439833	FARSA	assignmentsChromaffin_cells.Chrom_SOX2_statusHigh
assignmentsChromaffin_cells.Chrom_SOX2_statusHigh.SEC62	1.63430877	7.04754418	5.21421456	1.62E-06	1.62E-05	4.70599903	SEC62	assignmentsChromaffin_cells.Chrom_SOX2_statusHigh
assignmentsChromaffin_cells.Chrom_SOX2_statusHigh.COMTD1	1.8938066	3.1686443	5.17899614	1.86E-06	1.83E-05	4.70463015	COMTD1	assignmentsChromaffin_cells.Chrom_SOX2_statusHigh
assignmentsChromaffin_cells.Chrom_SOX2_statusHigh.NEO1	-1.7328766	4.96895742	-5.1802679	1.85E-06	1.82E-05	4.69967428	NEO1	assignmentsChromaffin_cells.Chrom_SOX2_statusHigh
assignmentsChromaffin_cells.Chrom_SOX2_statusHigh.NELFA	-0.8382285	5.00902007	-5.1835734	1.82E-06	1.80E-05	4.69553625	NELFA	assignmentsChromaffin_cells.Chrom_SOX2_statusHigh
assignmentsChromaffin_cells.Chrom_SOX2_statusHigh.RPL10	3.15354282	8.24475775	5.22482951	1.56E-06	1.57E-05	4.69202596	RPL10	assignmentsChromaffin_cells.Chrom_SOX2_statusHigh
assignmentsChromaffin_cells.Chrom_SOX2_statusHigh.RPS15A	3.11495223	6.86174115	5.21127183	1.64E-06	1.64E-05	4.69171638	RPS15A	assignmentsChromaffin_cells.Chrom_SOX2_statusHigh
assignmentsChromaffin_cells.Chrom_SOX2_statusHigh.NEU1	1.68259552	4.17449761	5.1817987	1.84E-06	1.81E-05	4.68737303	NEU1	assignmentsChromaffin_cells.Chrom_SOX2_statusHigh
assignmentsChromaffin_cells.Chrom_SOX2_statusHigh.FBXL19	-1.4947587	3.0437957	-5.1535815	2.05E-06	1.99E-05	4.68675996	FBXL19	assignmentsChromaffin_cells.Chrom_SOX2_statusHigh
assignmentsChromaffin_cells.Chrom_SOX2_statusHigh.RPS7	2.90422354	7.0911679	5.21133331	1.64E-06	1.64E-05	4.68401901	RPS7	assignmentsChromaffin_cells.Chrom_SOX2_statusHigh
assignmentsChromaffin_cells.Chrom_SOX2_statusHigh.ZNF570	1.15468722	3.01125279	5.1628457	1.98E-06	1.92E-05	4.68348022	ZNF570	assignmentsChromaffin_cells.Chrom_SOX2_statusHigh
assignmentsChromaffin_cells.Chrom_SOX2_statusHigh.GPATCH4	1.59252277	3.49731942	5.17023923	1.92E-06	1.88E-05	4.68150659	GPATCH4	assignmentsChromaffin_cells.Chrom_SOX2_statusHigh
assignmentsChromaffin_cells.Chrom_SOX2_statusHigh.ZNF408	1.42069502	1.20243972	5.13952293	2.17E-06	2.07E-05	4.6774389	ZNF408	assignmentsChromaffin_cells.Chrom_SOX2_statusHigh
assignmentsChromaffin_cells.Chrom_SOX2_statusHigh.RPL6	2.87050305	7.45863136	5.21321328	1.63E-06	1.63E-05	4.66950397	RPL6	assignmentsChromaffin_cells.Chrom_SOX2_statusHigh
assignmentsChromaffin_cells.Chrom_SOX2_statusHigh.C19orf73	1.90258147	0.46616534	5.13565694	2.20E-06	2.10E-05	4.66711823	C19orf73	assignmentsChromaffin_cells.Chrom_SOX2_statusHigh
assignmentsChromaffin_cells.Chrom_SOX2_statusHigh.KBTBD4	1.4141002	2.16951452	5.14640098	2.11E-06	2.03E-05	4.65832324	KBTBD4	assignmentsChromaffin_cells.Chrom_SOX2_statusHigh
assignmentsChromaffin_cells.Chrom_SOX2_statusHigh.CMTM4	-1.2559548	4.98099869	-5.1727566	1.90E-06	1.87E-05	4.65470208	CMTM4	assignmentsChromaffin_cells.Chrom_SOX2_statusHigh
assignmentsChromaffin_cells.Chrom_SOX2_statusHigh.GOLGA7	1.17608803	4.54062713	5.17134115	1.91E-06	1.88E-05	4.64609848	GOLGA7	assignmentsChromaffin_cells.Chrom_SOX2_statusHigh
assignmentsChromaffin_cells.Chrom_SOX2_statusHigh.HACE1	-1.1553944	5.64017126	-5.1700773	1.92E-06	1.88E-05	4.64325012	HACE1	assignmentsChromaffin_cells.Chrom_SOX2_statusHigh
assignmentsChromaffin_cells.Chrom_SOX2_statusHigh.DEPDC5	-1.0555415	5.1903033	-5.1636957	1.97E-06	1.92E-05	4.64280451	DEPDC5	assignmentsChromaffin_cells.Chrom_SOX2_statusHigh
assignmentsChromaffin_cells.Chrom_SOX2_statusHigh.RAVER2	-1.5135909	4.11888866	-5.1565666	2.03E-06	1.96E-05	4.63697849	RAVER2	assignmentsChromaffin_cells.Chrom_SOX2_statusHigh
assignmentsChromaffin_cells.Chrom_SOX2_statusHigh.TMEM14A	1.60834131	3.81119153	5.16924662	1.93E-06	1.89E-05	4.63385185	TMEM14A	assignmentsChromaffin_cells.Chrom_SOX2_statusHigh
assignmentsChromaffin_cells.Chrom_SOX2_statusHigh.UBC	2.77044213	7.79022076	5.20301448	1.70E-06	1.69E-05	4.62533375	UBC	assignmentsChromaffin_cells.Chrom_SOX2_statusHigh
assignmentsChromaffin_cells.Chrom_SOX2_statusHigh.CTSB	1.59264069	6.14434927	5.1701389	1.92E-06	1.88E-05	4.62357266	CTSB	assignmentsChromaffin_cells.Chrom_SOX2_statusHigh
assignmentsChromaffin_cells.Chrom_SOX2_statusHigh.JMJD4	1.2522809	2.52947977	5.144552	2.13E-06	2.04E-05	4.61413676	JMJD4	assignmentsChromaffin_cells.Chrom_SOX2_statusHigh
assignmentsChromaffin_cells.Chrom_SOX2_statusHigh.ZNF414	1.27824799	2.95410989	5.146338	2.11E-06	2.03E-05	4.61232673	ZNF414	assignmentsChromaffin_cells.Chrom_SOX2_statusHigh
assignmentsChromaffin_cells.Chrom_SOX2_statusHigh.RPL15	3.3562159	6.72377072	5.19003715	1.79E-06	1.76E-05	4.61109925	RPL15	assignmentsChromaffin_cells.Chrom_SOX2_statusHigh
assignmentsChromaffin_cells.Chrom_SOX2_statusHigh.HGF	3.66767261	1.98840069	5.11246067	2.42E-06	2.27E-05	4.61095815	HGF	assignmentsChromaffin_cells.Chrom_SOX2_statusHigh
assignmentsChromaffin_cells.Chrom_SOX2_statusHigh.EMP3	3.3602684	4.31769751	5.14590972	2.12E-06	2.04E-05	4.60765986	EMP3	assignmentsChromaffin_cells.Chrom_SOX2_statusHigh
assignmentsChromaffin_cells.Chrom_SOX2_statusHigh.RARRES2	4.4244312	0.73552127	5.11736183	2.37E-06	2.23E-05	4.60759193	RARRES2	assignmentsChromaffin_cells.Chrom_SOX2_statusHigh
assignmentsChromaffin_cells.Chrom_SOX2_statusHigh.RPL39L	3.03957812	0.42756963	5.13163508	2.24E-06	2.13E-05	4.60692699	RPL39L	assignmentsChromaffin_cells.Chrom_SOX2_statusHigh
assignmentsChromaffin_cells.Chrom_SOX2_statusHigh.WDR70	-1.0402515	7.69201191	-5.1794429	1.85E-06	1.82E-05	4.59609496	WDR70	assignmentsChromaffin_cells.Chrom_SOX2_statusHigh
assignmentsChromaffin_cells.Chrom_SOX2_statusHigh.TAMM41	-1.0655142	4.96054353	-5.1511463	2.07E-06	2.00E-05	4.59541835	TAMM41	assignmentsChromaffin_cells.Chrom_SOX2_statusHigh
assignmentsChromaffin_cells.Chrom_SOX2_statusHigh.MRPL28	1.77327533	3.2993188	5.14567926	2.12E-06	2.03E-05	4.59380604	MRPL28	assignmentsChromaffin_cells.Chrom_SOX2_statusHigh
assignmentsChromaffin_cells.Chrom_SOX2_statusHigh.CCDC7	-1.3824958	6.59038338	-5.165099	1.96E-06	1.91E-05	4.59112941	CCDC7	assignmentsChromaffin_cells.Chrom_SOX2_statusHigh
assignmentsChromaffin_cells.Chrom_SOX2_statusHigh.RPRD1A	-0.8492225	6.76032257	-5.175284	1.88E-06	1.85E-05	4.58929018	RPRD1A	assignmentsChromaffin_cells.Chrom_SOX2_statusHigh

assignmentsChromaffin_cells.Chrom_SOX2_statusHigh.AGFG1	-1.1065654	6.87882951	-5.1662232	1.95E-06	1.90E-05	4.58587603	AGFG1	assignmentsChromaffin_cells.Chrom_SOX2_statusHigh
assignmentsChromaffin_cells.Chrom_SOX2_statusHigh.HILPDA	2.23239728	2.00751822	5.12461844	2.30E-06	2.17E-05	4.58464639	HILPDA	assignmentsChromaffin_cells.Chrom_SOX2_statusHigh
assignmentsChromaffin_cells.Chrom_SOX2_statusHigh.CDK2AP2	2.21883802	3.39534196	5.14153407	2.15E-06	2.06E-05	4.57738649	CDK2AP2	assignmentsChromaffin_cells.Chrom_SOX2_statusHigh
assignmentsChromaffin_cells.Chrom_SOX2_statusHigh.GPANK1	1.346319	3.35717136	5.14076232	2.16E-06	2.07E-05	4.57695718	GPANK1	assignmentsChromaffin_cells.Chrom_SOX2_statusHigh
assignmentsChromaffin_cells.Chrom_SOX2_statusHigh.EBPL	1.44367514	4.22082126	5.15209295	2.06E-06	1.99E-05	4.57591086	EBPL	assignmentsChromaffin_cells.Chrom_SOX2_statusHigh
assignmentsChromaffin_cells.Chrom_SOX2_statusHigh.NR6A1	-1.7567592	6.58372375	-5.1634119	1.97E-06	1.92E-05	4.56949241	NR6A1	assignmentsChromaffin_cells.Chrom_SOX2_statusHigh
assignmentsChromaffin_cells.Chrom_SOX2_statusHigh.SLC3A2	1.57231987	5.76460906	5.16491699	1.96E-06	1.91E-05	4.56708149	SLC3A2	assignmentsChromaffin_cells.Chrom_SOX2_statusHigh
assignmentsChromaffin_cells.Chrom_SOX2_statusHigh.EIF5	1.66510463	6.34344542	5.1661294	1.95E-06	1.90E-05	4.56622763	EIF5	assignmentsChromaffin_cells.Chrom_SOX2_statusHigh
assignmentsChromaffin_cells.Chrom_SOX2_statusHigh.HYAL2	2.25130222	3.08222171	5.12520176	2.29E-06	2.17E-05	4.55946625	HYAL2	assignmentsChromaffin_cells.Chrom_SOX2_statusHigh
assignmentsChromaffin_cells.Chrom_SOX2_statusHigh.KMT5B	-1.0719622	6.44712381	-5.160342	2.00E-06	1.94E-05	4.55942506	KMT5B	assignmentsChromaffin_cells.Chrom_SOX2_statusHigh
assignmentsChromaffin_cells.Chrom_SOX2_statusHigh.CCDC59	1.51650108	4.52266458	5.14834011	2.09E-06	2.02E-05	4.55316276	CCDC59	assignmentsChromaffin_cells.Chrom_SOX2_statusHigh
assignmentsChromaffin_cells.Chrom_SOX2_statusHigh.TMED3	1.55389643	4.59452917	5.15201891	2.06E-06	1.99E-05	4.55312782	TMED3	assignmentsChromaffin_cells.Chrom_SOX2_statusHigh
assignmentsChromaffin_cells.Chrom_SOX2_statusHigh.PRIMPOL	-1.0647708	5.30441234	-5.1465414	2.11E-06	2.03E-05	4.55240384	PRIMPOL	assignmentsChromaffin_cells.Chrom_SOX2_statusHigh
assignmentsChromaffin_cells.Chrom_SOX2_statusHigh.TMEM54	2.75825245	1.34655375	5.11149817	2.42E-06	2.27E-05	4.54671212	TMEM54	assignmentsChromaffin_cells.Chrom_SOX2_statusHigh
assignmentsChromaffin_cells.Chrom_SOX2_statusHigh.SNRPF	1.86370699	4.52404472	5.14836558	2.09E-06	2.02E-05	4.5447125	SNRPF	assignmentsChromaffin_cells.Chrom_SOX2_statusHigh
assignmentsChromaffin_cells.Chrom_SOX2_statusHigh.MVB12A	1.3889555	3.83012299	5.13459717	2.21E-06	2.11E-05	4.54393173	MVB12A	assignmentsChromaffin_cells.Chrom_SOX2_statusHigh
assignmentsChromaffin_cells.Chrom_SOX2_statusHigh.CCDC93	-0.9778809	6.5232483	-5.1528426	2.06E-06	1.99E-05	4.53744529	CCDC93	assignmentsChromaffin_cells.Chrom_SOX2_statusHigh
assignmentsChromaffin_cells.Chrom_SOX2_statusHigh.ENOPH1	0.91353776	4.55876211	5.14115352	2.15E-06	2.06E-05	4.53401413	ENOPH1	assignmentsChromaffin_cells.Chrom_SOX2_statusHigh
assignmentsChromaffin_cells.Chrom_SOX2_statusHigh.PDXDC1	-0.8933183	7.11859532	-5.1608557	1.99E-06	1.94E-05	4.53326457	PDXDC1	assignmentsChromaffin_cells.Chrom_SOX2_statusHigh
assignmentsChromaffin_cells.Chrom_SOX2_statusHigh.VPS9D1	-1.1227433	4.75566404	-5.1322655	2.23E-06	2.12E-05	4.5224845	VPS9D1	assignmentsChromaffin_cells.Chrom_SOX2_statusHigh
assignmentsChromaffin_cells.Chrom_SOX2_statusHigh.FAM66C	-1.6759177	4.32084556	-5.129918	2.25E-06	2.14E-05	4.52157089	FAM66C	assignmentsChromaffin_cells.Chrom_SOX2_statusHigh
assignmentsChromaffin_cells.Chrom_SOX2_statusHigh.FBXO48	2.07840287	1.50668765	5.09066744	2.63E-06	2.44E-05	4.51070837	FBXO48	assignmentsChromaffin_cells.Chrom_SOX2_statusHigh
assignmentsChromaffin_cells.Chrom_SOX2_statusHigh.NFATC4	3.87547108	1.21667353	5.08650098	2.67E-06	2.47E-05	4.51039317	NFATC4	assignmentsChromaffin_cells.Chrom_SOX2_statusHigh
assignmentsChromaffin_cells.Chrom_SOX2_statusHigh.DRAP1	2.32740657	4.7063056	5.13856963	2.18E-06	2.08E-05	4.510168	DRAP1	assignmentsChromaffin_cells.Chrom_SOX2_statusHigh
assignmentsChromaffin_cells.Chrom_SOX2_statusHigh.SRPRB	1.4425623	4.05228363	5.12894037	2.26E-06	2.14E-05	4.5063102	SRPRB	assignmentsChromaffin_cells.Chrom_SOX2_statusHigh
assignmentsChromaffin_cells.Chrom_SOX2_statusHigh.NAA20	1.24122873	5.06454953	5.14123388	2.15E-06	2.06E-05	4.50318441	NAA20	assignmentsChromaffin_cells.Chrom_SOX2_statusHigh
assignmentsChromaffin_cells.Chrom_SOX2_statusHigh.SNRPB2	1.92839678	4.99248017	5.14264008	2.15E-06	2.06E-05	4.50276528	SNRPB2	assignmentsChromaffin_cells.Chrom_SOX2_statusHigh
assignmentsChromaffin_cells.Chrom_SOX2_statusHigh.PPP1CC	0.92003033	5.38593655	5.14005073	2.16E-06	2.07E-05	4.49725412	PPP1CC	assignmentsChromaffin_cells.Chrom_SOX2_statusHigh
assignmentsChromaffin_cells.Chrom_SOX2_statusHigh.ATF4	1.64189735	5.78422656	5.14614212	2.11E-06	2.03E-05	4.49682107	ATF4	assignmentsChromaffin_cells.Chrom_SOX2_statusHigh
assignmentsChromaffin_cells.Chrom_SOX2_statusHigh.RFK	1.34249624	4.1708476	5.1295102	2.26E-06	2.14E-05	4.4913127	RFK	assignmentsChromaffin_cells.Chrom_SOX2_statusHigh
assignmentsChromaffin_cells.Chrom_SOX2_statusHigh.KYAT1	-1.3867649	4.24037235	-5.1171062	2.37E-06	2.23E-05	4.49098108	KYAT1	assignmentsChromaffin_cells.Chrom_SOX2_statusHigh
assignmentsChromaffin_cells.Chrom_SOX2_statusHigh.NR2F6	1.48053402	3.26841609	5.12485219	2.30E-06	2.17E-05	4.48871101	NR2F6	assignmentsChromaffin_cells.Chrom_SOX2_statusHigh
assignmentsChromaffin_cells.Chrom_SOX2_statusHigh.KBTBD6	1.4547586	2.30913	5.1113106	2.42E-06	2.27E-05	4.48779861	KBTBD6	assignmentsChromaffin_cells.Chrom_SOX2_statusHigh
assignmentsChromaffin_cells.Chrom_SOX2_statusHigh.SLC35C1	1.69457043	1.4208594	5.08205343	2.71E-06	2.50E-05	4.47672683	SLC35C1	assignmentsChromaffin_cells.Chrom_SOX2_statusHigh
assignmentsChromaffin_cells.Chrom_SOX2_statusHigh.NUDT2	1.11303038	4.12668714	5.12398877	2.30E-06	2.18E-05	4.47558038	NUDT2	assignmentsChromaffin_cells.Chrom_SOX2_statusHigh
assignmentsChromaffin_cells.Chrom_SOX2_statusHigh.ZNF318	-1.1788105	4.76951326	-5.1162302	2.38E-06	2.23E-05	4.47171839	ZNF318	assignmentsChromaffin_cells.Chrom_SOX2_statusHigh
assignmentsChromaffin_cells.Chrom_SOX2_statusHigh.CRYBA2	4.08426872	-0.383537	5.07406586	2.81E-06	2.58E-05	4.4705399	CRYBA2	assignmentsChromaffin_cells.Chrom_SOX2_statusHigh
assignmentsChromaffin_cells.Chrom_SOX2_statusHigh.COPS7A	1.46420426	4.10694566	5.12400976	2.30E-06	2.18E-05	4.4679152	COPS7A	assignmentsChromaffin_cells.Chrom_SOX2_statusHigh
assignmentsChromaffin_cells.Chrom_SOX2_statusHigh.NFKBIB	1.516242	3.12169527	5.10549877	2.48E-06	2.31E-05	4.46643565	NFKBIB	assignmentsChromaffin_cells.Chrom_SOX2_statusHigh
assignmentsChromaffin_cells.Chrom_SOX2_statusHigh.ASPDH	2.36579585	1.87680086	5.11386633	2.40E-06	2.25E-05	4.46529243	ASPDH	assignmentsChromaffin_cells.Chrom_SOX2_statusHigh
assignmentsChromaffin_cells.Chrom_SOX2_statusHigh.SCAMP3	1.16006756	4.47914014	5.12386068	2.31E-06	2.18E-05	4.45838924	SCAMP3	assignmentsChromaffin_cells.Chrom_SOX2_statusHigh
assignmentsChromaffin_cells.Chrom_SOX2_statusHigh.TRPM7	-1.0922206	7.28373571	-5.1403193	2.16E-06	2.07E-05	4.45690486	TRPM7	assignmentsChromaffin_cells.Chrom_SOX2_statusHigh
assignmentsChromaffin_cells.Chrom_SOX2_statusHigh.C17orf58	1.9360053	1.86188591	5.08976986	2.63E-06	2.44E-05	4.45426779	C17orf58	assignmentsChromaffin_cells.Chrom_SOX2_statusHigh
assignmentsChromaffin_cells.Chrom_SOX2_statusHigh.MRTFB	-1.4471506	6.90320498	-5.1323376	2.23E-06	2.12E-05	4.45076677	MRTFB	assignmentsChromaffin_cells.Chrom_SOX2_statusHigh
assignmentsChromaffin_cells.Chrom_SOX2_statusHigh.LRRN4CL	2.75506621	0.07201854	5.06838431	2.86E-06	2.63E-05	4.44665349	LRRN4CL	assignmentsChromaffin_cells.Chrom_SOX2_statusHigh
assignmentsChromaffin_cells.Chrom_SOX2_statusHigh.SERPINH1	3.30302656	3.92595642	5.08868306	2.66E-06	2.46E-05	4.43037548	SERPINH1	assignmentsChromaffin_cells.Chrom_SOX2_statusHigh
assignmentsChromaffin_cells.Chrom_SOX2_statusHigh.SNN	2.06280725	2.13895518	5.08191783	2.72E-06	2.50E-05	4.43019512	SNN	assignmentsChromaffin_cells.Chrom_SOX2_statusHigh
assignmentsChromaffin_cells.Chrom_SOX2_statusHigh.NUDT15	1.72246376	2.24433341	5.08650658	2.67E-06	2.46E-05	4.42932521	NUDT15	assignmentsChromaffin_cells.Chrom_SOX2_statusHigh
assignmentsChromaffin_cells.Chrom_SOX2_statusHigh.MAP1LC3B	1.93319647	5.90953127	5.12971632	2.26E-06	2.14E-05	4.4280434	MAP1LC3B	assignmentsChromaffin_cells.Chrom_SOX2_statusHigh
assignmentsChromaffin_cells.Chrom_SOX2_statusHigh.RAD54L2	-1.5792629	5.88787306	-5.1155492	2.38E-06	2.24E-05	4.42667328	RAD54L2	assignmentsChromaffin_cells.Chrom_SOX2_statusHigh
assignmentsChromaffin_cells.Chrom_SOX2_statusHigh.MAP2K5	-0.9339554	7.18971916	-5.1319097	2.23E-06	2.12E-05	4.41701886	MAP2K5	assignmentsChromaffin_cells.Chrom_SOX2_statusHigh
assignmentsChromaffin_cells.Chrom_SOX2_statusHigh.PLEKHM3	-1.3512503	6.08239406	-5.119766	2.34E-06	2.21E-05	4.41643792	PLEKHM3	assignmentsChromaffin_cells.Chrom_SOX2_statusHigh
assignmentsChromaffin_cells.Chrom_SOX2_statusHigh.EHBP1	-1.2861736	7.83221495	-5.1351615	2.21E-06	2.10E-05	4.41367636	EHBP1	assignmentsChromaffin_cells.Chrom_SOX2_statusHigh
assignmentsChromaffin_cells.Chrom_SOX2_statusHigh.SPATA5	-0.9992446	6.84287047	-5.1187792	2.35E-06	2.22E-05	4.40334346	SPATA5	assignmentsChromaffin_cells.Chrom_SOX2_statusHigh
assignmentsChromaffin_cells.Chrom_SOX2_statusHigh.UHRF2	-1.0061949	6.39156172	-5.1095447	2.44E-06	2.28E-05	4.40156331	UHRF2	assignmentsChromaffin_cells.Chrom_SOX2_statusHigh
assignmentsChromaffin_cells.Chrom_SOX2_statusHigh.ZNF667-AS:	1.45115452	4.20733303	5.11476177	2.39E-06	2.24E-05	4.40060907	ZNF667-AS1	assignmentsChromaffin_cells.Chrom_SOX2_statusHigh
assignmentsChromaffin_cells.Chrom_SOX2_statusHigh.LINC00324	2.57417939	1.32564678	5.05282561	3.04E-06	2.77E-05	4.3961838	LINC00324	assignmentsChromaffin_cells.Chrom_SOX2_statusHigh
assignmentsChromaffin_cells.Chrom_SOX2_statusHigh.MORF4L1	1.38681229	7.38266695	5.13238841	2.23E-06	2.12E-05	4.39195292	MORF4L1	assignmentsChromaffin_cells.Chrom_SOX2_statusHigh
assignmentsChromaffin_cells.Chrom_SOX2_statusHigh.MYL12A	2.50717153	5.40593595	5.09947853	2.55E-06	2.37E-05	4.38924775	MYL12A	assignmentsChromaffin_cells.Chrom_SOX2_statusHigh
assignmentsChromaffin_cells.Chrom_SOX2_statusHigh.UQCRB	3.10960274	5.93258559	5.12561987	2.30E-06	2.17E-05	4.38699344	UQCRB	assignmentsChromaffin_cells.Chrom_SOX2_statusHigh
assignmentsChromaffin_cells.Chrom_SOX2_statusHigh.MCRIP1	1.58442213	4.53827415	5.10643539	2.47E-06	2.31E-05	4.38547009	MCRIP1	assignmentsChromaffin_cells.Chrom_SOX2_statusHigh
assignmentsChromaffin_cells.Chrom_SOX2_statusHigh.RTTN	-1.0832208	5.63167336	-5.1002156	2.53E-06	2.36E-05	4.38505907	RTTN	assignmentsChromaffin_cells.Chrom_SOX2_statusHigh
assignmentsChromaffin_cells.Chrom_SOX2_statusHigh.RPSA	2.4231821	6.81737796	5.13044834	2.26E-06	2.14E-05	4.38271455	RPSA	assignmentsChromaffin_cells.Chrom_SOX2_statusHigh
assignmentsChromaffin_cells.Chrom_SOX2_statusHigh.PUSL1	1.8140233	1.23219366	5.05837445	2.98E-06	2.72E-05	4.37793272	PUSL1	assignmentsChromaffin_cells.Chrom_SOX2_statusHigh

assignmentsChromaffin_cells.Chrom_SOX2_statusHigh.DNPH1	2.60793705	2.98455683	5.08805168	2.66E-06	2.46E-05	4.37632709	DNPH1	assignmentsChromaffin_cells.Chrom_SOX2_statusHigh
assignmentsChromaffin_cells.Chrom_SOX2_statusHigh.LZIC	1.24450273	4.06627003	5.08964216	2.64E-06	2.44E-05	4.36924319	LZIC	assignmentsChromaffin_cells.Chrom_SOX2_statusHigh
assignmentsChromaffin_cells.Chrom_SOX2_statusHigh.REPIN1	1.27801969	3.40676615	5.09144594	2.62E-06	2.43E-05	4.36916123	REPIN1	assignmentsChromaffin_cells.Chrom_SOX2_statusHigh
assignmentsChromaffin_cells.Chrom_SOX2_statusHigh.RUFY2	-0.8144551	6.3936076	-5.1102441	2.43E-06	2.28E-05	4.36033264	RUFY2	assignmentsChromaffin_cells.Chrom_SOX2_statusHigh
assignmentsChromaffin_cells.Chrom_SOX2_statusHigh.GRINA	1.98568524	4.39648307	5.09382412	2.60E-06	2.41E-05	4.35748913	GRINA	assignmentsChromaffin_cells.Chrom_SOX2_statusHigh
assignmentsChromaffin_cells.Chrom_SOX2_statusHigh.RAC1	1.71572514	6.64462304	5.11358267	2.40E-06	2.25E-05	4.35457453	RAC1	assignmentsChromaffin_cells.Chrom_SOX2_statusHigh
assignmentsChromaffin_cells.Chrom_SOX2_statusHigh.LMTK2	-0.8182197	5.67380794	-5.0981847	2.55E-06	2.38E-05	4.35198714	LMTK2	assignmentsChromaffin_cells.Chrom_SOX2_statusHigh
assignmentsChromaffin_cells.Chrom_SOX2_statusHigh.ATP9A	-1.8221116	5.23284715	-5.0906822	2.62E-06	2.44E-05	4.35004336	ATP9A	assignmentsChromaffin_cells.Chrom_SOX2_statusHigh
assignmentsChromaffin_cells.Chrom_SOX2_statusHigh.NEIL2	1.23570822	2.95206441	5.0765485	2.77E-06	2.55E-05	4.34539724	NEIL2	assignmentsChromaffin_cells.Chrom_SOX2_statusHigh
assignmentsChromaffin_cells.Chrom_SOX2_statusHigh.LDHB	1.69584612	5.77074912	5.10855066	2.45E-06	2.29E-05	4.34372289	LDHB	assignmentsChromaffin_cells.Chrom_SOX2_statusHigh
assignmentsChromaffin_cells.Chrom_SOX2_statusHigh.KIAA1328	-1.1321968	7.15313586	-5.1104517	2.43E-06	2.27E-05	4.33821973	KIAA1328	assignmentsChromaffin_cells.Chrom_SOX2_statusHigh
assignmentsChromaffin_cells.Chrom_SOX2_statusHigh.AMBRA1	-1.2789827	6.89795897	-5.097861	2.55E-06	2.38E-05	4.33214198	AMBRA1	assignmentsChromaffin_cells.Chrom_SOX2_statusHigh
assignmentsChromaffin_cells.Chrom_SOX2_statusHigh.DNAJC8	1.41022702	5.22067155	5.09095073	2.62E-06	2.44E-05	4.32906597	DNAJC8	assignmentsChromaffin_cells.Chrom_SOX2_statusHigh
assignmentsChromaffin_cells.Chrom_SOX2_statusHigh.PIGU	-1.0908365	5.19005201	-5.0828092	2.71E-06	2.50E-05	4.31187516	PIGU	assignmentsChromaffin_cells.Chrom_SOX2_statusHigh
assignmentsChromaffin_cells.Chrom_SOX2_statusHigh.SSNA1	1.82178824	3.59924603	5.07246564	2.82E-06	2.59E-05	4.31119656	SSNA1	assignmentsChromaffin_cells.Chrom_SOX2_statusHigh
assignmentsChromaffin_cells.Chrom_SOX2_statusHigh.PJA1	1.25795974	2.6994624	5.06286332	2.93E-06	2.68E-05	4.30360245	PJA1	assignmentsChromaffin_cells.Chrom_SOX2_statusHigh
assignmentsChromaffin_cells.Chrom_SOX2_statusHigh.NUTM2B-A!	-0.8694338	7.21710258	-5.0978611	2.55E-06	2.38E-05	4.30283431	NUTM2B-AS	assignmentsChromaffin_cells.Chrom_SOX2_statusHigh
assignmentsChromaffin_cells.Chrom_SOX2_statusHigh.RGPD5	-2.1172197	2.52712095	-5.0394635	3.20E-06	2.88E-05	4.30180928	RGPD5	assignmentsChromaffin_cells.Chrom_SOX2_statusHigh
assignmentsChromaffin_cells.Chrom_SOX2_statusHigh.C3orf38	1.14836395	4.39869773	5.0705358	2.84E-06	2.61E-05	4.28178318	C3orf38	assignmentsChromaffin_cells.Chrom_SOX2_statusHigh
assignmentsChromaffin_cells.Chrom_SOX2_statusHigh.MRPL20-AS	1.84467225	1.76336766	5.0312764	3.31E-06	2.96E-05	4.26830792	MRPL20-AS1	assignmentsChromaffin_cells.Chrom_SOX2_statusHigh
assignmentsChromaffin_cells.Chrom_SOX2_statusHigh.PTP4A3	3.96180914	2.54333181	5.02252743	3.44E-06	3.06E-05	4.26579599	PTP4A3	assignmentsChromaffin_cells.Chrom_SOX2_statusHigh
assignmentsChromaffin_cells.Chrom_SOX2_statusHigh.LINC02745	3.98249286	0.41519032	5.02475363	3.41E-06	3.04E-05	4.2654085	LINC02745	assignmentsChromaffin_cells.Chrom_SOX2_statusHigh
assignmentsChromaffin_cells.Chrom_SOX2_statusHigh.PEX10	1.20672755	2.85275164	5.04651954	3.12E-06	2.82E-05	4.26481594	PEX10	assignmentsChromaffin_cells.Chrom_SOX2_statusHigh
assignmentsChromaffin_cells.Chrom_SOX2_statusHigh.SERTAD1	3.19963565	2.93499241	5.04424675	3.16E-06	2.85E-05	4.25989267	SERTAD1	assignmentsChromaffin_cells.Chrom_SOX2_statusHigh
assignmentsChromaffin_cells.Chrom_SOX2_statusHigh.COPB2	1.19726865	6.31205374	5.09009503	2.63E-06	2.44E-05	4.25923234	COPB2	assignmentsChromaffin_cells.Chrom_SOX2_statusHigh
assignmentsChromaffin_cells.Chrom_SOX2_statusHigh.FBXL15	1.42504771	3.78775731	5.06625371	2.89E-06	2.65E-05	4.2585568	FBXL15	assignmentsChromaffin_cells.Chrom_SOX2_statusHigh
assignmentsChromaffin_cells.Chrom_SOX2_statusHigh.ATXN2	-0.7659622	7.68451586	-5.0932882	2.60E-06	2.42E-05	4.25667511	ATXN2	assignmentsChromaffin_cells.Chrom_SOX2_statusHigh
assignmentsChromaffin_cells.Chrom_SOX2_statusHigh.NAGA	1.57244151	2.73569724	5.04396173	3.15E-06	2.84E-05	4.25626715	NAGA	assignmentsChromaffin_cells.Chrom_SOX2_statusHigh
assignmentsChromaffin_cells.Chrom_SOX2_statusHigh.ZNF783	-1.56266	3.9423891	-5.0488294	3.09E-06	2.80E-05	4.25467962	ZNF783	assignmentsChromaffin_cells.Chrom_SOX2_statusHigh
assignmentsChromaffin_cells.Chrom_SOX2_statusHigh.GSTK1	1.48315326	5.45543694	5.07140833	2.83E-06	2.60E-05	4.25183831	GSTK1	assignmentsChromaffin_cells.Chrom_SOX2_statusHigh
assignmentsChromaffin_cells.Chrom_SOX2_statusHigh.B3GALT6	1.78910421	1.91816568	5.03012139	3.32E-06	2.98E-05	4.2460514	B3GALT6	assignmentsChromaffin_cells.Chrom_SOX2_statusHigh
assignmentsChromaffin_cells.Chrom_SOX2_statusHigh.LINC01002	-1.7514596	1.77204969	-5.0182559	3.48E-06	3.10E-05	4.24304396	LINC01002	assignmentsChromaffin_cells.Chrom_SOX2_statusHigh
assignmentsChromaffin_cells.Chrom_SOX2_statusHigh.CCDC107	1.95535917	4.06181802	5.05115757	3.06E-06	2.78E-05	4.23579842	CCDC107	assignmentsChromaffin_cells.Chrom_SOX2_statusHigh
assignmentsChromaffin_cells.Chrom_SOX2_statusHigh.DCAF13	1.0174104	4.64019959	5.06196807	2.94E-06	2.69E-05	4.23400201	DCAF13	assignmentsChromaffin_cells.Chrom_SOX2_statusHigh
assignmentsChromaffin_cells.Chrom_SOX2_statusHigh.SMIM29	1.50998261	3.93032498	5.06004172	2.96E-06	2.70E-05	4.23358228	SMIM29	assignmentsChromaffin_cells.Chrom_SOX2_statusHigh
assignmentsChromaffin_cells.Chrom_SOX2_statusHigh.NKAP	0.98916144	4.76247393	5.06666745	2.88E-06	2.64E-05	4.23202312	NKAP	assignmentsChromaffin_cells.Chrom_SOX2_statusHigh
assignmentsChromaffin_cells.Chrom_SOX2_statusHigh.CTSA	1.50985379	4.40209422	5.05568821	3.01E-06	2.74E-05	4.22055251	CTSA	assignmentsChromaffin_cells.Chrom_SOX2_statusHigh
assignmentsChromaffin_cells.Chrom_SOX2_statusHigh.GPATCH8	-0.763976	8.05249326	-5.0891807	2.64E-06	2.44E-05	4.21935346	GPATCH8	assignmentsChromaffin_cells.Chrom_SOX2_statusHigh
assignmentsChromaffin_cells.Chrom_SOX2_statusHigh.DCAF17	-1.0899307	4.70670661	-5.0450976	3.14E-06	2.83E-05	4.2179131	DCAF17	assignmentsChromaffin_cells.Chrom_SOX2_statusHigh
assignmentsChromaffin_cells.Chrom_SOX2_statusHigh.IPO4	1.48119851	1.62692608	5.02229604	3.43E-06	3.06E-05	4.21565689	IPO4	assignmentsChromaffin_cells.Chrom_SOX2_statusHigh
assignmentsChromaffin_cells.Chrom_SOX2_statusHigh.WFS1	1.60039504	2.77071577	5.03953891	3.20E-06	2.88E-05	4.21473636	WFS1	assignmentsChromaffin_cells.Chrom_SOX2_statusHigh
assignmentsChromaffin_cells.Chrom_SOX2_statusHigh.KAT6A	-0.8979656	7.89447172	-5.0770594	2.77E-06	2.55E-05	4.20737442	KAT6A	assignmentsChromaffin_cells.Chrom_SOX2_statusHigh
assignmentsChromaffin_cells.Chrom_SOX2_statusHigh.PRKCE	-1.0972751	8.50144074	-5.0854148	2.68E-06	2.47E-05	4.20497511	PRKCE	assignmentsChromaffin_cells.Chrom_SOX2_statusHigh
assignmentsChromaffin_cells.Chrom_SOX2_statusHigh.CEP164	-1.0463513	5.75082223	-5.0590921	2.97E-06	2.71E-05	4.19944986	CEP164	assignmentsChromaffin_cells.Chrom_SOX2_statusHigh
assignmentsChromaffin_cells.Chrom_SOX2_statusHigh.SF3B4	1.63237057	3.76738966	5.04674704	3.12E-06	2.82E-05	4.19895324	SF3B4	assignmentsChromaffin_cells.Chrom_SOX2_statusHigh
assignmentsChromaffin_cells.Chrom_SOX2_statusHigh.C17orf75	1.04202638	3.46831846	5.04631065	3.12E-06	2.82E-05	4.19757287	C17orf75	assignmentsChromaffin_cells.Chrom_SOX2_statusHigh
assignmentsChromaffin_cells.Chrom_SOX2_statusHigh.RNF126	1.15494811	4.0817213	5.04890647	3.09E-06	2.80E-05	4.19648077	RNF126	assignmentsChromaffin_cells.Chrom_SOX2_statusHigh
assignmentsChromaffin_cells.Chrom_SOX2_statusHigh.TMEM14C	1.89208995	4.70861991	5.05599171	3.01E-06	2.74E-05	4.19434693	TMEM14C	assignmentsChromaffin_cells.Chrom_SOX2_statusHigh
assignmentsChromaffin_cells.Chrom_SOX2_statusHigh.ADIRF-AS1	3.944018	-0.0748721	5.03484219	3.26E-06	2.93E-05	4.1902932	ADIRF-AS1	assignmentsChromaffin_cells.Chrom_SOX2_statusHigh
assignmentsChromaffin_cells.Chrom_SOX2_statusHigh.NBN	1.10959901	5.46164454	5.05463167	3.02E-06	2.75E-05	4.18551106	NBN	assignmentsChromaffin_cells.Chrom_SOX2_statusHigh
assignmentsChromaffin_cells.Chrom_SOX2_statusHigh.JMJD8	1.27864623	2.65860607	5.02505969	3.39E-06	3.03E-05	4.18480149	JMJD8	assignmentsChromaffin_cells.Chrom_SOX2_statusHigh
assignmentsChromaffin_cells.Chrom_SOX2_statusHigh.AP2A1	1.00728632	5.14533836	5.05248582	3.05E-06	2.77E-05	4.17854245	AP2A1	assignmentsChromaffin_cells.Chrom_SOX2_statusHigh
assignmentsChromaffin_cells.Chrom_SOX2_statusHigh.POLR1D	1.23016743	5.47190868	5.05804526	2.98E-06	2.72E-05	4.17691423	POLR1D	assignmentsChromaffin_cells.Chrom_SOX2_statusHigh
assignmentsChromaffin_cells.Chrom_SOX2_statusHigh.PSMA5	1.27468825	5.24310045	5.04709731	3.11E-06	2.82E-05	4.16998227	PSMA5	assignmentsChromaffin_cells.Chrom_SOX2_statusHigh
assignmentsChromaffin_cells.Chrom_SOX2_statusHigh.DHX35	-1.1878095	4.59668387	-5.0325556	3.29E-06	2.95E-05	4.16987984	DHX35	assignmentsChromaffin_cells.Chrom_SOX2_statusHigh
assignmentsChromaffin_cells.Chrom_SOX2_statusHigh.RGL3	3.09140896	3.38123007	5.01541211	3.53E-06	3.14E-05	4.15697449	RGL3	assignmentsChromaffin_cells.Chrom_SOX2_statusHigh
assignmentsChromaffin_cells.Chrom_SOX2_statusHigh.CMBL	2.72209341	1.9256707	5.01622005	3.52E-06	3.13E-05	4.15296919	CMBL	assignmentsChromaffin_cells.Chrom_SOX2_statusHigh
assignmentsChromaffin_cells.Chrom_SOX2_statusHigh.DUSP28	1.51863459	2.29646165	5.00946075	3.60E-06	3.19E-05	4.15082064	DUSP28	assignmentsChromaffin_cells.Chrom_SOX2_statusHigh
assignmentsChromaffin_cells.Chrom_SOX2_statusHigh.ACSF3	-0.9806728	4.80892022	-5.0283673	3.35E-06	2.99E-05	4.14727692	ACSF3	assignmentsChromaffin_cells.Chrom_SOX2_statusHigh
assignmentsChromaffin_cells.Chrom_SOX2_statusHigh.ZBTB38	1.17516801	6.78000766	5.05529234	3.01E-06	2.74E-05	4.14376109	ZBTB38	assignmentsChromaffin_cells.Chrom_SOX2_statusHigh
assignmentsChromaffin_cells.Chrom_SOX2_statusHigh.C8orf33	1.18425785	3.99414745	5.03258246	3.29E-06	2.95E-05	4.13962713	C8orf33	assignmentsChromaffin_cells.Chrom_SOX2_statusHigh
assignmentsChromaffin_cells.Chrom_SOX2_statusHigh.CPSF6	-0.8925478	7.55253651	-5.0600422	2.96E-06	2.70E-05	4.13542416	CPSF6	assignmentsChromaffin_cells.Chrom_SOX2_statusHigh
assignmentsChromaffin_cells.Chrom_SOX2_statusHigh.NELFE	1.21642869	4.27419023	5.03146284	3.31E-06	2.96E-05	4.1284737	NELFE	assignmentsChromaffin_cells.Chrom_SOX2_statusHigh
assignmentsChromaffin_cells.Chrom_SOX2_statusHigh.B4GALT3	1.16437418	3.71363092	5.02195977	3.43E-06	3.06E-05	4.1268027	B4GALT3	assignmentsChromaffin_cells.Chrom_SOX2_statusHigh

assignmentsChromaffin_cells.Chrom_SOX2_statusHigh.HMGN2	1.39493945	6.07672955	5.04998647	3.08E-06	2.79E-05	4.12355577	HMGN2	assignmentsChromaffin_cells.Chrom_SOX2_statusHigh
assignmentsChromaffin_cells.Chrom_SOX2_statusHigh.SPPL3	-0.9597436	7.61705815	-5.054198	3.03E-06	2.75E-05	4.1234045	SPPL3	assignmentsChromaffin_cells.Chrom_SOX2_statusHigh
assignmentsChromaffin_cells.Chrom_SOX2_statusHigh.DGCR2	-1.0426707	5.74756412	-5.038718	3.21E-06	2.89E-05	4.11999569	DGCR2	assignmentsChromaffin_cells.Chrom_SOX2_statusHigh
assignmentsChromaffin_cells.Chrom_SOX2_statusHigh.RBM3	1.30258645	6.16103325	5.05118929	3.06E-06	2.78E-05	4.11988132	RBM3	assignmentsChromaffin_cells.Chrom_SOX2_statusHigh
assignmentsChromaffin_cells.Chrom_SOX2_statusHigh.IDS	1.10712825	7.25505326	5.06444366	2.91E-06	2.66E-05	4.11486035	IDS	assignmentsChromaffin_cells.Chrom_SOX2_statusHigh
assignmentsChromaffin_cells.Chrom_SOX2_statusHigh.FUNDC2	1.62004818	4.66491414	5.03182882	3.30E-06	2.96E-05	4.11009934	FUNDC2	assignmentsChromaffin_cells.Chrom_SOX2_statusHigh
assignmentsChromaffin_cells.Chrom_SOX2_statusHigh.ATP5F1C	1.06782786	6.20286773	5.04690834	3.11E-06	2.82E-05	4.10523348	ATP5F1C	assignmentsChromaffin_cells.Chrom_SOX2_statusHigh
assignmentsChromaffin_cells.Chrom_SOX2_statusHigh.USE1	1.72622946	3.54491984	5.01304996	3.55E-06	3.15E-05	4.10410238	USE1	assignmentsChromaffin_cells.Chrom_SOX2_statusHigh
assignmentsChromaffin_cells.Chrom_SOX2_statusHigh.DENND6A	-1.0697334	6.1363881	-5.0261028	3.38E-06	3.02E-05	4.0991721	DENND6A	assignmentsChromaffin_cells.Chrom_SOX2_statusHigh
assignmentsChromaffin_cells.Chrom_SOX2_statusHigh.DNAJA1	2.24856645	6.85972789	5.05115082	3.08E-06	2.79E-05	4.09550378	DNAJA1	assignmentsChromaffin_cells.Chrom_SOX2_statusHigh
assignmentsChromaffin_cells.Chrom_SOX2_statusHigh.FAHD1	1.33014306	2.87222269	5.0028354	3.69E-06	3.27E-05	4.07128205	FAHD1	assignmentsChromaffin_cells.Chrom_SOX2_statusHigh
assignmentsChromaffin_cells.Chrom_SOX2_statusHigh.UBXN6	1.19397966	3.84580233	5.01435238	3.53E-06	3.14E-05	4.06966452	UBXN6	assignmentsChromaffin_cells.Chrom_SOX2_statusHigh
assignmentsChromaffin_cells.Chrom_SOX2_statusHigh.ANAPC15	2.0226279	2.27037026	4.98930594	3.89E-06	3.41E-05	4.06573392	ANAPC15	assignmentsChromaffin_cells.Chrom_SOX2_statusHigh
assignmentsChromaffin_cells.Chrom_SOX2_statusHigh.SLC35A4	1.57631856	2.55909393	4.99132072	3.86E-06	3.39E-05	4.06175927	SLC35A4	assignmentsChromaffin_cells.Chrom_SOX2_statusHigh
assignmentsChromaffin_cells.Chrom_SOX2_statusHigh.CDC20	2.7648838	0.17635337	4.95914798	4.37E-06	3.76E-05	4.0569405	CDC20	assignmentsChromaffin_cells.Chrom_SOX2_statusHigh
assignmentsChromaffin_cells.Chrom_SOX2_statusHigh.RAD1	1.01644259	3.45121417	4.99894948	3.75E-06	3.31E-05	4.05614978	RAD1	assignmentsChromaffin_cells.Chrom_SOX2_statusHigh
assignmentsChromaffin_cells.Chrom_SOX2_statusHigh.RPL5	2.84356155	7.17398524	5.04511001	3.15E-06	2.84E-05	4.05581186	RPL5	assignmentsChromaffin_cells.Chrom_SOX2_statusHigh
assignmentsChromaffin_cells.Chrom_SOX2_statusHigh.AKAP10	-1.1477232	6.42557773	-5.0174447	3.49E-06	3.11E-05	4.04349018	AKAP10	assignmentsChromaffin_cells.Chrom_SOX2_statusHigh
assignmentsChromaffin_cells.Chrom_SOX2_statusHigh.KCNG3	-3.1056841	2.30398863	-4.9721983	4.17E-06	3.62E-05	4.03918924	KCNG3	assignmentsChromaffin_cells.Chrom_SOX2_statusHigh
assignmentsChromaffin_cells.Chrom_SOX2_statusHigh.SRPK3	2.89436308	0.33506407	4.96629081	4.26E-06	3.69E-05	4.03903598	SRPK3	assignmentsChromaffin_cells.Chrom_SOX2_statusHigh
assignmentsChromaffin_cells.Chrom_SOX2_statusHigh.TWF1	1.14079532	4.58949058	5.01133744	3.57E-06	3.17E-05	4.03876014	TWF1	assignmentsChromaffin_cells.Chrom_SOX2_statusHigh
assignmentsChromaffin_cells.Chrom_SOX2_statusHigh.SLC39A7	1.60648638	4.43791733	5.01112489	3.58E-06	3.17E-05	4.03282936	SLC39A7	assignmentsChromaffin_cells.Chrom_SOX2_statusHigh
assignmentsChromaffin_cells.Chrom_SOX2_statusHigh.CDKN1B	1.5505071	5.09145327	5.01148735	3.57E-06	3.17E-05	4.0312188	CDKN1B	assignmentsChromaffin_cells.Chrom_SOX2_statusHigh
assignmentsChromaffin_cells.Chrom_SOX2_statusHigh.SFR1	1.68006683	1.91338145	4.96681545	4.25E-06	3.68E-05	4.0304629	SFR1	assignmentsChromaffin_cells.Chrom_SOX2_statusHigh
assignmentsChromaffin_cells.Chrom_SOX2_statusHigh.COL16A1	2.84306082	2.03806203	4.95582773	4.44E-06	3.80E-05	4.02271301	COL16A1	assignmentsChromaffin_cells.Chrom_SOX2_statusHigh
assignmentsChromaffin_cells.Chrom_SOX2_statusHigh.SSU72	1.24802794	4.90177474	5.00455893	3.67E-06	3.25E-05	4.02073278	SSU72	assignmentsChromaffin_cells.Chrom_SOX2_statusHigh
assignmentsChromaffin_cells.Chrom_SOX2_statusHigh.MRPL44	1.17631426	3.34309754	4.98852287	3.90E-06	3.42E-05	4.01648275	MRPL44	assignmentsChromaffin_cells.Chrom_SOX2_statusHigh
assignmentsChromaffin_cells.Chrom_SOX2_statusHigh.RBM19	-0.9458281	5.27311677	-4.9974163	3.77E-06	3.33E-05	4.01336609	RBM19	assignmentsChromaffin_cells.Chrom_SOX2_statusHigh
assignmentsChromaffin_cells.Chrom_SOX2_statusHigh.THG1L	1.48034532	2.34828324	4.97435838	4.12E-06	3.58E-05	4.01311815	THG1L	assignmentsChromaffin_cells.Chrom_SOX2_statusHigh
assignmentsChromaffin_cells.Chrom_SOX2_statusHigh.TKFC	1.49544919	2.3946063	4.97981883	4.04E-06	3.52E-05	4.00855894	TKFC	assignmentsChromaffin_cells.Chrom_SOX2_statusHigh
assignmentsChromaffin_cells.Chrom_SOX2_statusHigh.FAM181B	3.88283447	-0.3739826	4.95187131	4.50E-06	3.85E-05	4.00152641	FAM181B	assignmentsChromaffin_cells.Chrom_SOX2_statusHigh
assignmentsChromaffin_cells.Chrom_SOX2_statusHigh.FER	-1.1264718	8.69117967	-5.0332865	3.28E-06	2.95E-05	3.99334377	FER	assignmentsChromaffin_cells.Chrom_SOX2_statusHigh
assignmentsChromaffin_cells.Chrom_SOX2_statusHigh.ZNF562	-0.9983697	5.33088431	-4.9961272	3.79E-06	3.34E-05	3.98829639	ZNF562	assignmentsChromaffin_cells.Chrom_SOX2_statusHigh
assignmentsChromaffin_cells.Chrom_SOX2_statusHigh.CCNG1	1.18402596	4.2049544	4.99519762	3.80E-06	3.35E-05	3.98784782	CCNG1	assignmentsChromaffin_cells.Chrom_SOX2_statusHigh
assignmentsChromaffin_cells.Chrom_SOX2_statusHigh.MAGED2	1.75915421	5.33358891	5.00997951	3.60E-06	3.19E-05	3.9843766	MAGED2	assignmentsChromaffin_cells.Chrom_SOX2_statusHigh
assignmentsChromaffin_cells.Chrom_SOX2_statusHigh.ZMYM2	-0.8279356	8.54166143	-5.0265496	3.37E-06	3.01E-05	3.97615302	ZMYM2	assignmentsChromaffin_cells.Chrom_SOX2_statusHigh
assignmentsChromaffin_cells.Chrom_SOX2_statusHigh.ANKRD36C	-1.7870233	7.62522734	-5.0161419	3.51E-06	3.13E-05	3.97526201	ANKRD36C	assignmentsChromaffin_cells.Chrom_SOX2_statusHigh
assignmentsChromaffin_cells.Chrom_SOX2_statusHigh.RPE	-0.9334035	5.15659011	-4.9888914	3.90E-06	3.42E-05	3.97445205	RPE	assignmentsChromaffin_cells.Chrom_SOX2_statusHigh
assignmentsChromaffin_cells.Chrom_SOX2_statusHigh.PDSS1	-0.8902431	4.08272846	-4.9722866	4.16E-06	3.61E-05	3.97310594	PDSS1	assignmentsChromaffin_cells.Chrom_SOX2_statusHigh
assignmentsChromaffin_cells.Chrom_SOX2_statusHigh.KIF9-AS1	-1.1280604	5.84354467	-4.9987679	3.75E-06	3.31E-05	3.96117284	KIF9-AS1	assignmentsChromaffin_cells.Chrom_SOX2_statusHigh
assignmentsChromaffin_cells.Chrom_SOX2_statusHigh.RNF5	1.92224987	3.03304769	4.9680298	4.23E-06	3.66E-05	3.9565851	RNF5	assignmentsChromaffin_cells.Chrom_SOX2_statusHigh
assignmentsChromaffin_cells.Chrom_SOX2_statusHigh.MYADM	2.09984211	5.13643587	4.98665705	3.94E-06	3.45E-05	3.95033861	MYADM	assignmentsChromaffin_cells.Chrom_SOX2_statusHigh
assignmentsChromaffin_cells.Chrom_SOX2_statusHigh.TMBIM4	1.13475693	6.53085976	5.0022023	3.70E-06	3.27E-05	3.94693567	TMBIM4	assignmentsChromaffin_cells.Chrom_SOX2_statusHigh
assignmentsChromaffin_cells.Chrom_SOX2_statusHigh.TRMT6	1.5501711	3.82712433	4.98227908	4.00E-06	3.49E-05	3.9441984	TRMT6	assignmentsChromaffin_cells.Chrom_SOX2_statusHigh
assignmentsChromaffin_cells.Chrom_SOX2_statusHigh.CAPNS1	1.8429375	4.50750815	4.98337379	3.98E-06	3.48E-05	3.94382952	CAPNS1	assignmentsChromaffin_cells.Chrom_SOX2_statusHigh
assignmentsChromaffin_cells.Chrom_SOX2_statusHigh.KDELR2	1.65837913	4.99840883	4.9936699	3.83E-06	3.36E-05	3.94265705	KDELR2	assignmentsChromaffin_cells.Chrom_SOX2_statusHigh
assignmentsChromaffin_cells.Chrom_SOX2_statusHigh.CYTH2	0.90391994	4.5749934	4.98201048	4.00E-06	3.50E-05	3.940804	CYTH2	assignmentsChromaffin_cells.Chrom_SOX2_statusHigh
assignmentsChromaffin_cells.Chrom_SOX2_statusHigh.PLPPR2	1.46986695	2.98825616	4.96465233	4.28E-06	3.70E-05	3.93514713	PLPPR2	assignmentsChromaffin_cells.Chrom_SOX2_statusHigh
assignmentsChromaffin_cells.Chrom_SOX2_statusHigh.KIAA0040	3.39426698	3.06773441	4.9261979	4.97E-06	4.18E-05	3.93114732	KIAA0040	assignmentsChromaffin_cells.Chrom_SOX2_statusHigh
assignmentsChromaffin_cells.Chrom_SOX2_statusHigh.FAM110A	1.78248854	2.55150478	4.93724571	4.76E-06	4.04E-05	3.92999463	FAM110A	assignmentsChromaffin_cells.Chrom_SOX2_statusHigh
assignmentsChromaffin_cells.Chrom_SOX2_statusHigh.ARFRP1	1.01894735	4.2247305	4.97585739	4.10E-06	3.57E-05	3.92953219	ARFRP1	assignmentsChromaffin_cells.Chrom_SOX2_statusHigh
assignmentsChromaffin_cells.Chrom_SOX2_statusHigh.LINC00595	2.85717496	0.17049961	4.93010176	4.90E-06	4.13E-05	3.92814314	LINC00595	assignmentsChromaffin_cells.Chrom_SOX2_statusHigh
assignmentsChromaffin_cells.Chrom_SOX2_statusHigh.VTN	2.2782898	0.59401108	4.936174	4.78E-06	4.05E-05	3.92273134	VTN	assignmentsChromaffin_cells.Chrom_SOX2_statusHigh
assignmentsChromaffin_cells.Chrom_SOX2_statusHigh.ASAH1	1.62851741	6.34640988	4.99810627	3.76E-06	3.32E-05	3.92126325	ASAH1	assignmentsChromaffin_cells.Chrom_SOX2_statusHigh
assignmentsChromaffin_cells.Chrom_SOX2_statusHigh.PEX11B	1.11418734	3.70885851	4.96571275	4.26E-06	3.69E-05	3.91962298	PEX11B	assignmentsChromaffin_cells.Chrom_SOX2_statusHigh
assignmentsChromaffin_cells.Chrom_SOX2_statusHigh.CREB3L1	4.24134547	0.19472675	4.92262545	5.04E-06	4.22E-05	3.91497361	CREB3L1	assignmentsChromaffin_cells.Chrom_SOX2_statusHigh
assignmentsChromaffin_cells.Chrom_SOX2_statusHigh.TAF3	-0.8568303	6.97366296	-4.99933126	3.83E-06	3.37E-05	3.91446936	TAF3	assignmentsChromaffin_cells.Chrom_SOX2_statusHigh
assignmentsChromaffin_cells.Chrom_SOX2_statusHigh.EEF2	2.16786426	6.80132885	5.00538279	3.67E-06	3.25E-05	3.91393263	EEF2	assignmentsChromaffin_cells.Chrom_SOX2_statusHigh
assignmentsChromaffin_cells.Chrom_SOX2_statusHigh.ECPAS	-1.0493016	7.32697725	-4.9959311	3.79E-06	3.34E-05	3.91236214	ECPAS	assignmentsChromaffin_cells.Chrom_SOX2_statusHigh
assignmentsChromaffin_cells.Chrom_SOX2_statusHigh.EIF2S2	1.04522666	6.41389832	4.9965541	3.78E-06	3.34E-05	3.91207292	EIF2S2	assignmentsChromaffin_cells.Chrom_SOX2_statusHigh
assignmentsChromaffin_cells.Chrom_SOX2_statusHigh.TSFM	1.08339963	3.63094263	4.96294661	4.31E-06	3.72E-05	3.90156808	TSFM	assignmentsChromaffin_cells.Chrom_SOX2_statusHigh
assignmentsChromaffin_cells.Chrom_SOX2_statusHigh.TAPT1-AS1	-1.8104515	5.93434261	-4.9791784	4.05E-06	3.53E-05	3.89038822	TAPT1-AS1	assignmentsChromaffin_cells.Chrom_SOX2_statusHigh
assignmentsChromaffin_cells.Chrom_SOX2_statusHigh.DDO	3.54449837	0.45625332	4.91028439	5.28E-06	4.39E-05	3.88254884	DDO	assignmentsChromaffin_cells.Chrom_SOX2_statusHigh
assignmentsChromaffin_cells.Chrom_SOX2_statusHigh.CDK13	-0.950438	7.92647286	-4.9937606	3.83E-06	3.36E-05	3.87940645	CDK13	assignmentsChromaffin_cells.Chrom_SOX2_statusHigh

assignmentsChromaffin_cells.Chrom_SOX2_statusHigh.NFIX	3.27951775	2.97189476	4.92564787	5.00E-06	4.20E-05	3.87536394	NFIX	assignmentsChromaffin_cells.Chrom_SOX2_statusHigh
assignmentsChromaffin_cells.Chrom_SOX2_statusHigh.WDR75	-0.7517719	5.32963408	-4.9627208	4.31E-06	3.72E-05	3.87188061	WDR75	assignmentsChromaffin_cells.Chrom_SOX2_statusHigh
assignmentsChromaffin_cells.Chrom_SOX2_statusHigh.SLC22A15	-2.3741312	4.57649441	-4.9434923	4.65E-06	3.96E-05	3.86927458	SLC22A15	assignmentsChromaffin_cells.Chrom_SOX2_statusHigh
assignmentsChromaffin_cells.Chrom_SOX2_statusHigh.SLC25A4	2.11866742	4.37602672	4.97334922	4.15E-06	3.60E-05	3.86399926	SLC25A4	assignmentsChromaffin_cells.Chrom_SOX2_statusHigh
assignmentsChromaffin_cells.Chrom_SOX2_statusHigh.SCG3	2.79564685	4.48337104	4.98417465	3.99E-06	3.49E-05	3.86274272	SCG3	assignmentsChromaffin_cells.Chrom_SOX2_statusHigh
assignmentsChromaffin_cells.Chrom_SOX2_statusHigh.IGFBP4	3.10446687	4.37012471	4.93045558	4.91E-06	4.13E-05	3.86203022	IGFBP4	assignmentsChromaffin_cells.Chrom_SOX2_statusHigh
assignmentsChromaffin_cells.Chrom_SOX2_statusHigh.GHDC	1.82504438	2.21415266	4.93177196	4.86E-06	4.10E-05	3.86197376	GHDC	assignmentsChromaffin_cells.Chrom_SOX2_statusHigh
assignmentsChromaffin_cells.Chrom_SOX2_statusHigh.ZNF511	1.368093	3.93070401	4.95587614	4.43E-06	3.80E-05	3.86078862	ZNF511	assignmentsChromaffin_cells.Chrom_SOX2_statusHigh
assignmentsChromaffin_cells.Chrom_SOX2_statusHigh.NIFK	1.56977045	4.45991457	4.96159836	4.33E-06	3.73E-05	3.86034839	NIFK	assignmentsChromaffin_cells.Chrom_SOX2_statusHigh
assignmentsChromaffin_cells.Chrom_SOX2_statusHigh.RPL11	2.8447175	7.22026653	4.98960813	3.90E-06	3.42E-05	3.8562095	RPL11	assignmentsChromaffin_cells.Chrom_SOX2_statusHigh
assignmentsChromaffin_cells.Chrom_SOX2_statusHigh.ABHD11	1.33220048	2.68832363	4.9394238	4.72E-06	4.01E-05	3.85363539	ABHD11	assignmentsChromaffin_cells.Chrom_SOX2_statusHigh
assignmentsChromaffin_cells.Chrom_SOX2_statusHigh.NUDT16L1	1.6392791	2.77636423	4.94272221	4.66E-06	3.96E-05	3.85328739	NUDT16L1	assignmentsChromaffin_cells.Chrom_SOX2_statusHigh
assignmentsChromaffin_cells.Chrom_SOX2_statusHigh.SCMH1	-1.0515112	7.20600257	-4.9814529	4.01E-06	3.50E-05	3.85035797	SCMH1	assignmentsChromaffin_cells.Chrom_SOX2_statusHigh
assignmentsChromaffin_cells.Chrom_SOX2_statusHigh.MRPS18B	1.38827613	3.83150148	4.95107549	4.51E-06	3.85E-05	3.84714918	MRPS18B	assignmentsChromaffin_cells.Chrom_SOX2_statusHigh
assignmentsChromaffin_cells.Chrom_SOX2_statusHigh.RCN2	1.02419326	5.68696088	4.97525467	4.11E-06	3.57E-05	3.84663492	RCN2	assignmentsChromaffin_cells.Chrom_SOX2_statusHigh
assignmentsChromaffin_cells.Chrom_SOX2_statusHigh.CD59	1.91295194	5.96701489	4.9646475	4.29E-06	3.70E-05	3.84655459	CD59	assignmentsChromaffin_cells.Chrom_SOX2_statusHigh
assignmentsChromaffin_cells.Chrom_SOX2_statusHigh.VAMP3	1.53878086	4.28116392	4.95135866	4.51E-06	3.85E-05	3.84508804	VAMP3	assignmentsChromaffin_cells.Chrom_SOX2_statusHigh
assignmentsChromaffin_cells.Chrom_SOX2_statusHigh.FARP2	-0.9079685	6.01029888	-4.9601742	4.36E-06	3.75E-05	3.84198542	FARP2	assignmentsChromaffin_cells.Chrom_SOX2_statusHigh
assignmentsChromaffin_cells.Chrom_SOX2_statusHigh.TFCP2L1	3.68912093	0.63645174	4.89881407	5.52E-06	4.56E-05	3.84194127	TFCP2L1	assignmentsChromaffin_cells.Chrom_SOX2_statusHigh
assignmentsChromaffin_cells.Chrom_SOX2_statusHigh.CST3	4.02362116	5.62908501	4.96150123	4.35E-06	3.75E-05	3.83860585	CST3	assignmentsChromaffin_cells.Chrom_SOX2_statusHigh
assignmentsChromaffin_cells.Chrom_SOX2_statusHigh.SEC11C	2.35433152	4.77011172	4.97024136	4.21E-06	3.65E-05	3.83339374	SEC11C	assignmentsChromaffin_cells.Chrom_SOX2_statusHigh
assignmentsChromaffin_cells.Chrom_SOX2_statusHigh.MRPS11	1.04867447	4.02248014	4.94647958	4.59E-06	3.92E-05	3.82687539	MRPS11	assignmentsChromaffin_cells.Chrom_SOX2_statusHigh
assignmentsChromaffin_cells.Chrom_SOX2_statusHigh.MT2A	4.27699487	5.18541252	4.93107507	4.89E-06	4.12E-05	3.82531227	MT2A	assignmentsChromaffin_cells.Chrom_SOX2_statusHigh
assignmentsChromaffin_cells.Chrom_SOX2_statusHigh.PRPS1	1.46677604	3.33816314	4.934999	4.80E-06	4.07E-05	3.82446546	PRPS1	assignmentsChromaffin_cells.Chrom_SOX2_statusHigh
assignmentsChromaffin_cells.Chrom_SOX2_statusHigh.TMEFF1	-1.3601035	3.40753279	-4.9307616	4.88E-06	4.12E-05	3.82113075	TMEFF1	assignmentsChromaffin_cells.Chrom_SOX2_statusHigh
assignmentsChromaffin_cells.Chrom_SOX2_statusHigh.EGFLAM	-2.1638891	6.82779318	-4.9754985	4.12E-06	3.58E-05	3.81945667	EGFLAM	assignmentsChromaffin_cells.Chrom_SOX2_statusHigh
assignmentsChromaffin_cells.Chrom_SOX2_statusHigh.MCM3AP	-1.058839	5.80358504	-4.9531469	4.48E-06	3.83E-05	3.81826895	MCM3AP	assignmentsChromaffin_cells.Chrom_SOX2_statusHigh
assignmentsChromaffin_cells.Chrom_SOX2_statusHigh.SMIM30	2.07763026	2.87502397	4.93289105	4.84E-06	4.09E-05	3.81734116	SMIM30	assignmentsChromaffin_cells.Chrom_SOX2_statusHigh
assignmentsChromaffin_cells.Chrom_SOX2_statusHigh.TUBB2A	2.68895174	3.0782328	4.93689972	4.79E-06	4.05E-05	3.81394118	TUBB2A	assignmentsChromaffin_cells.Chrom_SOX2_statusHigh
assignmentsChromaffin_cells.Chrom_SOX2_statusHigh.ZNF292	-0.7360373	7.79484802	-4.9783208	4.06E-06	3.54E-05	3.81294299	ZNF292	assignmentsChromaffin_cells.Chrom_SOX2_statusHigh
assignmentsChromaffin_cells.Chrom_SOX2_statusHigh.ATG3	1.2567632	5.0693966	4.95153172	4.50E-06	3.85E-05	3.81240829	ATG3	assignmentsChromaffin_cells.Chrom_SOX2_statusHigh
assignmentsChromaffin_cells.Chrom_SOX2_statusHigh.KTI12	2.25730659	0.81274067	4.89320977	5.64E-06	4.64E-05	3.80731364	KTI12	assignmentsChromaffin_cells.Chrom_SOX2_statusHigh
assignmentsChromaffin_cells.Chrom_SOX2_statusHigh.PCSK1N	3.76286299	6.51126877	4.99179839	3.87E-06	3.40E-05	3.80448752	PCSK1N	assignmentsChromaffin_cells.Chrom_SOX2_statusHigh
assignmentsChromaffin_cells.Chrom_SOX2_statusHigh.NME3	1.55004428	5.47064155	4.96198347	4.33E-06	3.73E-05	3.80261092	NME3	assignmentsChromaffin_cells.Chrom_SOX2_statusHigh
assignmentsChromaffin_cells.Chrom_SOX2_statusHigh.FBXL6	1.05502955	2.52909528	4.92256637	5.04E-06	4.22E-05	3.80246378	FBXL6	assignmentsChromaffin_cells.Chrom_SOX2_statusHigh
assignmentsChromaffin_cells.Chrom_SOX2_statusHigh.MPHOSPH9	-1.0986199	5.81887926	-4.9530792	4.48E-06	3.83E-05	3.80122235	MPHOSPH9	assignmentsChromaffin_cells.Chrom_SOX2_statusHigh
assignmentsChromaffin_cells.Chrom_SOX2_statusHigh.MRPL27	1.26190788	4.17829606	4.94722148	4.58E-06	3.91E-05	3.80042021	MRPL27	assignmentsChromaffin_cells.Chrom_SOX2_statusHigh
assignmentsChromaffin_cells.Chrom_SOX2_statusHigh.ZNF747	1.1728317	1.74431229	4.90961478	5.29E-06	4.39E-05	3.8002578	ZNF747	assignmentsChromaffin_cells.Chrom_SOX2_statusHigh
assignmentsChromaffin_cells.Chrom_SOX2_statusHigh.TRAPPC6A	1.27133697	3.27942406	4.92705743	4.95E-06	4.16E-05	3.79477001	TRAPPC6A	assignmentsChromaffin_cells.Chrom_SOX2_statusHigh
assignmentsChromaffin_cells.Chrom_SOX2_statusHigh.CKAP4	1.49843167	3.96556748	4.94023734	4.70E-06	4.00E-05	3.79153132	CKAP4	assignmentsChromaffin_cells.Chrom_SOX2_statusHigh
assignmentsChromaffin_cells.Chrom_SOX2_statusHigh.FRS2	-0.7305975	6.62368519	-4.9570265	4.41E-06	3.79E-05	3.79075507	FRS2	assignmentsChromaffin_cells.Chrom_SOX2_statusHigh
assignmentsChromaffin_cells.Chrom_SOX2_statusHigh.RPS3	2.74176568	7.42235282	4.97814738	4.08E-06	3.55E-05	3.78987529	RPS3	assignmentsChromaffin_cells.Chrom_SOX2_statusHigh
assignmentsChromaffin_cells.Chrom_SOX2_statusHigh.CREG1	1.80935975	4.44851983	4.9343696	4.81E-06	4.07E-05	3.7832983	CREG1	assignmentsChromaffin_cells.Chrom_SOX2_statusHigh
assignmentsChromaffin_cells.Chrom_SOX2_statusHigh.FBXW8	-1.0799301	5.91718009	-4.9509176	4.51E-06	3.85E-05	3.78236754	FBXW8	assignmentsChromaffin_cells.Chrom_SOX2_statusHigh
assignmentsChromaffin_cells.Chrom_SOX2_statusHigh.ASF1A	1.26679458	3.5441989	4.93012717	4.89E-06	4.12E-05	3.78201401	ASF1A	assignmentsChromaffin_cells.Chrom_SOX2_statusHigh
assignmentsChromaffin_cells.Chrom_SOX2_statusHigh.PPP1R21	-0.9768806	6.39719438	-4.9517584	4.50E-06	3.85E-05	3.77984967	PPP1R21	assignmentsChromaffin_cells.Chrom_SOX2_statusHigh
assignmentsChromaffin_cells.Chrom_SOX2_statusHigh.MNAT1	-1.0514234	7.47983077	-4.9644657	4.28E-06	3.70E-05	3.77938945	MNAT1	assignmentsChromaffin_cells.Chrom_SOX2_statusHigh
assignmentsChromaffin_cells.Chrom_SOX2_statusHigh.RPL26	3.36548019	6.80117388	4.96826495	4.24E-06	3.67E-05	3.77862679	RPL26	assignmentsChromaffin_cells.Chrom_SOX2_statusHigh
assignmentsChromaffin_cells.Chrom_SOX2_statusHigh.EDA2R	-1.976	3.26228213	-4.9138605	5.21E-06	4.34E-05	3.77351999	EDA2R	assignmentsChromaffin_cells.Chrom_SOX2_statusHigh
assignmentsChromaffin_cells.Chrom_SOX2_statusHigh.MRPL21	1.51853547	4.02603299	4.93449528	4.81E-06	4.07E-05	3.76950923	MRPL21	assignmentsChromaffin_cells.Chrom_SOX2_statusHigh
assignmentsChromaffin_cells.Chrom_SOX2_statusHigh.GSPT2	1.53747668	2.76715896	4.91850203	5.12E-06	4.27E-05	3.75777124	GSPT2	assignmentsChromaffin_cells.Chrom_SOX2_statusHigh
assignmentsChromaffin_cells.Chrom_SOX2_statusHigh.RSL1D1	1.58062857	5.2321926	4.94274319	4.66E-06	3.96E-05	3.75007774	RSL1D1	assignmentsChromaffin_cells.Chrom_SOX2_statusHigh
assignmentsChromaffin_cells.Chrom_SOX2_statusHigh.UQCRC1	1.22558302	4.60897427	4.93197325	4.86E-06	4.10E-05	3.74225192	UQCRC1	assignmentsChromaffin_cells.Chrom_SOX2_statusHigh
assignmentsChromaffin_cells.Chrom_SOX2_statusHigh.COA6	1.41506737	3.47379441	4.91933356	5.10E-06	4.26E-05	3.73618363	COA6	assignmentsChromaffin_cells.Chrom_SOX2_statusHigh
assignmentsChromaffin_cells.Chrom_SOX2_statusHigh.CAMSAP1	-1.0921304	5.15677391	-4.9195372	5.10E-06	4.26E-05	3.73271922	CAMSAP1	assignmentsChromaffin_cells.Chrom_SOX2_statusHigh
assignmentsChromaffin_cells.Chrom_SOX2_statusHigh.INKA1	2.24533565	0.52925311	4.86713638	6.23E-06	5.07E-05	3.730481	INKA1	assignmentsChromaffin_cells.Chrom_SOX2_statusHigh
assignmentsChromaffin_cells.Chrom_SOX2_statusHigh.CCDC34	1.5508751	2.4834811	4.90279789	5.43E-06	4.50E-05	3.72794474	CCDC34	assignmentsChromaffin_cells.Chrom_SOX2_statusHigh
assignmentsChromaffin_cells.Chrom_SOX2_statusHigh.SPOPL	-1.0400078	5.91347315	-4.9282853	4.93E-06	4.15E-05	3.72671022	SPOPL	assignmentsChromaffin_cells.Chrom_SOX2_statusHigh
assignmentsChromaffin_cells.Chrom_SOX2_statusHigh.PROX1-AS1	-2.0845966	4.02840051	-4.9225382	5.04E-06	4.22E-05	3.72601934	PROX1-AS1	assignmentsChromaffin_cells.Chrom_SOX2_statusHigh
assignmentsChromaffin_cells.Chrom_SOX2_statusHigh.RPL32	3.30080975	6.92145403	4.95419848	4.48E-06	3.83E-05	3.72501506	RPL32	assignmentsChromaffin_cells.Chrom_SOX2_statusHigh
assignmentsChromaffin_cells.Chrom_SOX2_statusHigh.NXPH4	1.91296943	2.45954197	4.91357481	5.21E-06	4.34E-05	3.72215297	NXPH4	assignmentsChromaffin_cells.Chrom_SOX2_statusHigh
assignmentsChromaffin_cells.Chrom_SOX2_statusHigh.RPS27A	3.4255209	7.46311928	4.96038604	4.37E-06	3.76E-05	3.717985	RPS27A	assignmentsChromaffin_cells.Chrom_SOX2_statusHigh
assignmentsChromaffin_cells.Chrom_SOX2_statusHigh.RPS2	2.91355552	7.42420439	4.95742309	4.42E-06	3.80E-05	3.71296405	RPS2	assignmentsChromaffin_cells.Chrom_SOX2_statusHigh
assignmentsChromaffin_cells.Chrom_SOX2_statusHigh.SCAMP2	0.99444141	4.86186477	4.92011292	5.08E-06	4.25E-05	3.71273583	SCAMP2	assignmentsChromaffin_cells.Chrom_SOX2_statusHigh

assignmentsChromaffin_cells.Chrom_SOX2_statusHigh.JUNB	3.66017062	7.17451885	4.94176756	4.70E-06	3.99E-05	3.71108511	JUNB	assignmentsChromaffin_cells.Chrom_SOX2_statusHigh
assignmentsChromaffin_cells.Chrom_SOX2_statusHigh.MMGT1	1.45662097	3.58495458	4.91034229	5.28E-06	4.39E-05	3.70977548	MMGT1	assignmentsChromaffin_cells.Chrom_SOX2_statusHigh
assignmentsChromaffin_cells.Chrom_SOX2_statusHigh.KRT80	3.84423405	-0.8471114	4.86931046	6.18E-06	5.03E-05	3.7082989	KRT80	assignmentsChromaffin_cells.Chrom_SOX2_statusHigh
assignmentsChromaffin_cells.Chrom_SOX2_statusHigh.IFI27L2	2.29635695	3.62232319	4.91429064	5.21E-06	4.34E-05	3.70335875	IFI27L2	assignmentsChromaffin_cells.Chrom_SOX2_statusHigh
assignmentsChromaffin_cells.Chrom_SOX2_statusHigh.FAM104B	1.34481882	2.75132039	4.89978415	5.50E-06	4.54E-05	3.7024562	FAM104B	assignmentsChromaffin_cells.Chrom_SOX2_statusHigh
assignmentsChromaffin_cells.Chrom_SOX2_statusHigh.INS	5.29624481	0.91850598	4.87498495	6.07E-06	4.96E-05	3.70055482	INS	assignmentsChromaffin_cells.Chrom_SOX2_statusHigh
assignmentsChromaffin_cells.Chrom_SOX2_statusHigh.IAH1	1.09624167	4.83834443	4.92291839	5.03E-06	4.22E-05	3.69733185	IAH1	assignmentsChromaffin_cells.Chrom_SOX2_statusHigh
assignmentsChromaffin_cells.Chrom_SOX2_statusHigh.ATP5F1A	1.36622693	5.93201947	4.93818483	4.74E-06	4.02E-05	3.69442125	ATP5F1A	assignmentsChromaffin_cells.Chrom_SOX2_statusHigh
assignmentsChromaffin_cells.Chrom_SOX2_statusHigh.ERVK13-1	-1.3367512	5.3518893	-4.9156743	5.17E-06	4.31E-05	3.68624122	ERVK13-1	assignmentsChromaffin_cells.Chrom_SOX2_statusHigh
assignmentsChromaffin_cells.Chrom_SOX2_statusHigh.CACNG2	-1.7436943	4.46723001	-4.9173758	5.14E-06	4.29E-05	3.68245363	CACNG2	assignmentsChromaffin_cells.Chrom_SOX2_statusHigh
assignmentsChromaffin_cells.Chrom_SOX2_statusHigh.CORO1B	1.91896702	3.44474838	4.89310942	5.64E-06	4.64E-05	3.68009457	CORO1B	assignmentsChromaffin_cells.Chrom_SOX2_statusHigh
assignmentsChromaffin_cells.Chrom_SOX2_statusHigh.SCUBE3	-3.1481838	1.29802168	-4.8546969	6.53E-06	5.26E-05	3.67673254	SCUBE3	assignmentsChromaffin_cells.Chrom_SOX2_statusHigh
assignmentsChromaffin_cells.Chrom_SOX2_statusHigh.LINC01250	2.16805947	1.82222983	4.88737656	5.77E-06	4.74E-05	3.66890102	LINC01250	assignmentsChromaffin_cells.Chrom_SOX2_statusHigh
assignmentsChromaffin_cells.Chrom_SOX2_statusHigh.UBAC2	-1.1231468	7.26218049	-4.9235034	5.02E-06	4.21E-05	3.66448923	UBAC2	assignmentsChromaffin_cells.Chrom_SOX2_statusHigh
assignmentsChromaffin_cells.Chrom_SOX2_statusHigh.TBC1D19	-1.1838949	6.48243738	-4.9238926	5.01E-06	4.21E-05	3.65855631	TBC1D19	assignmentsChromaffin_cells.Chrom_SOX2_statusHigh
assignmentsChromaffin_cells.Chrom_SOX2_statusHigh.RORA	-1.9748153	8.96166867	-4.9452217	4.63E-06	3.95E-05	3.65721615	RORA	assignmentsChromaffin_cells.Chrom_SOX2_statusHigh
assignmentsChromaffin_cells.Chrom_SOX2_statusHigh.LINC01422	-1.8848905	3.19131991	-4.859894	6.40E-06	5.18E-05	3.65703152	LINC01422	assignmentsChromaffin_cells.Chrom_SOX2_statusHigh
assignmentsChromaffin_cells.Chrom_SOX2_statusHigh.RPL24	3.13928042	6.65922445	4.93439989	4.83E-06	4.09E-05	3.65519455	RPL24	assignmentsChromaffin_cells.Chrom_SOX2_statusHigh
assignmentsChromaffin_cells.Chrom_SOX2_statusHigh.RAB11B-AS	1.49728614	2.48893166	4.87083994	6.14E-06	5.01E-05	3.64315572	RAB11B-AS1	assignmentsChromaffin_cells.Chrom_SOX2_statusHigh
assignmentsChromaffin_cells.Chrom_SOX2_statusHigh.EMC3	1.04192807	5.10559293	4.90553471	5.38E-06	4.45E-05	3.63774665	EMC3	assignmentsChromaffin_cells.Chrom_SOX2_statusHigh
assignmentsChromaffin_cells.Chrom_SOX2_statusHigh.GM2A	1.51382287	2.72148221	4.8635518	6.32E-06	5.12E-05	3.63571486	GM2A	assignmentsChromaffin_cells.Chrom_SOX2_statusHigh
assignmentsChromaffin_cells.Chrom_SOX2_statusHigh.GTF3A	1.39100349	4.88777168	4.90575771	5.37E-06	4.45E-05	3.63443829	GTF3A	assignmentsChromaffin_cells.Chrom_SOX2_statusHigh
assignmentsChromaffin_cells.Chrom_SOX2_statusHigh.TLNRD1	2.02106839	3.03147512	4.86252422	6.34E-06	5.14E-05	3.6342918	TLNRD1	assignmentsChromaffin_cells.Chrom_SOX2_statusHigh
assignmentsChromaffin_cells.Chrom_SOX2_statusHigh.POLR1E	1.28875887	2.54236227	4.86896069	6.19E-06	5.04E-05	3.62482267	POLR1E	assignmentsChromaffin_cells.Chrom_SOX2_statusHigh
assignmentsChromaffin_cells.Chrom_SOX2_statusHigh.PARP1	1.00624307	5.49092425	4.90728312	5.34E-06	4.43E-05	3.620623	PARP1	assignmentsChromaffin_cells.Chrom_SOX2_statusHigh
assignmentsChromaffin_cells.Chrom_SOX2_statusHigh.EEF1A1	3.28920074	8.86862058	4.94158019	4.70E-06	3.99E-05	3.60483067	EEF1A1	assignmentsChromaffin_cells.Chrom_SOX2_statusHigh
assignmentsChromaffin_cells.Chrom_SOX2_statusHigh.PDE4B	3.0310633	7.18570164	4.91282622	5.25E-06	4.37E-05	3.6040596	PDE4B	assignmentsChromaffin_cells.Chrom_SOX2_statusHigh
assignmentsChromaffin_cells.Chrom_SOX2_statusHigh.DAZAP2	1.18057246	5.41995294	4.89656352	5.57E-06	4.59E-05	3.6013446	DAZAP2	assignmentsChromaffin_cells.Chrom_SOX2_statusHigh
assignmentsChromaffin_cells.Chrom_SOX2_statusHigh.UIMC1	-0.9305911	6.50186661	-4.904301	5.40E-06	4.47E-05	3.60078404	UIMC1	assignmentsChromaffin_cells.Chrom_SOX2_statusHigh
assignmentsChromaffin_cells.Chrom_SOX2_statusHigh.APLP1	2.50471756	4.64970854	4.91246651	5.26E-06	4.37E-05	3.59888113	APLP1	assignmentsChromaffin_cells.Chrom_SOX2_statusHigh
assignmentsChromaffin_cells.Chrom_SOX2_statusHigh.RGL4	-3.2962285	0.34571932	-4.8548657	6.53E-06	5.26E-05	3.59726396	RGL4	assignmentsChromaffin_cells.Chrom_SOX2_statusHigh
assignmentsChromaffin_cells.Chrom_SOX2_statusHigh.TAS2R30	-1.4551619	1.59404391	-4.8361094	7.01E-06	5.59E-05	3.59342554	TAS2R30	assignmentsChromaffin_cells.Chrom_SOX2_statusHigh
assignmentsChromaffin_cells.Chrom_SOX2_statusHigh.FOXN3	-1.4109651	8.8864571	-4.9193414	5.10E-06	4.26E-05	3.59171975	FOXN3	assignmentsChromaffin_cells.Chrom_SOX2_statusHigh
assignmentsChromaffin_cells.Chrom_SOX2_statusHigh.RPL39	3.39658838	6.4817027	4.91462135	5.21E-06	4.34E-05	3.58679226	RPL39	assignmentsChromaffin_cells.Chrom_SOX2_statusHigh
assignmentsChromaffin_cells.Chrom_SOX2_statusHigh.PLEKHJ1	1.44354332	3.55847998	4.87577697	6.03E-06	4.93E-05	3.58207071	PLEKHJ1	assignmentsChromaffin_cells.Chrom_SOX2_statusHigh
assignmentsChromaffin_cells.Chrom_SOX2_statusHigh.UBE2I	0.93686262	5.74928161	4.89922042	5.51E-06	4.55E-05	3.58042753	UBE2I	assignmentsChromaffin_cells.Chrom_SOX2_statusHigh
assignmentsChromaffin_cells.Chrom_SOX2_statusHigh.FRY	-1.4815573	7.71715279	-4.907128	5.34E-06	4.43E-05	3.57053743	FRY	assignmentsChromaffin_cells.Chrom_SOX2_statusHigh
assignmentsChromaffin_cells.Chrom_SOX2_statusHigh.ACP2	1.71004614	2.85790724	4.85976079	6.41E-06	5.18E-05	3.56770513	ACP2	assignmentsChromaffin_cells.Chrom_SOX2_statusHigh
assignmentsChromaffin_cells.Chrom_SOX2_statusHigh.NUBPL	-1.0797999	6.65255998	-4.8938105	5.62E-06	4.63E-05	3.56743358	NUBPL	assignmentsChromaffin_cells.Chrom_SOX2_statusHigh
assignmentsChromaffin_cells.Chrom_SOX2_statusHigh.TIMM17A	1.13857086	4.55598408	4.88225306	5.88E-06	4.82E-05	3.55969355	TIMM17A	assignmentsChromaffin_cells.Chrom_SOX2_statusHigh
assignmentsChromaffin_cells.Chrom_SOX2_statusHigh.MROH8	-1.3224727	3.55154909	-4.8597869	6.41E-06	5.18E-05	3.55921971	MROH8	assignmentsChromaffin_cells.Chrom_SOX2_statusHigh
assignmentsChromaffin_cells.Chrom_SOX2_statusHigh.ZNF586	-1.3332731	5.73170523	-4.8801685	5.93E-06	4.85E-05	3.558822	ZNF586	assignmentsChromaffin_cells.Chrom_SOX2_statusHigh
assignmentsChromaffin_cells.Chrom_SOX2_statusHigh.CTSL	2.12107401	5.42638699	4.88477195	5.83E-06	4.79E-05	3.55845374	CTSL	assignmentsChromaffin_cells.Chrom_SOX2_statusHigh
assignmentsChromaffin_cells.Chrom_SOX2_statusHigh.ZFAND3	-1.1224631	9.54939767	-4.9206632	5.07E-06	4.25E-05	3.55743771	ZFAND3	assignmentsChromaffin_cells.Chrom_SOX2_statusHigh
assignmentsChromaffin_cells.Chrom_SOX2_statusHigh.BCKDK	1.64987911	3.13577228	4.85816053	6.45E-06	5.21E-05	3.55731307	BCKDK	assignmentsChromaffin_cells.Chrom_SOX2_statusHigh
assignmentsChromaffin_cells.Chrom_SOX2_statusHigh.STS	-1.6269986	4.07434718	-4.855168	6.52E-06	5.26E-05	3.55583169	STS	assignmentsChromaffin_cells.Chrom_SOX2_statusHigh
assignmentsChromaffin_cells.Chrom_SOX2_statusHigh.C7orf25	1.31467132	2.3414672	4.85510738	6.52E-06	5.26E-05	3.5543348	C7orf25	assignmentsChromaffin_cells.Chrom_SOX2_statusHigh
assignmentsChromaffin_cells.Chrom_SOX2_statusHigh.RIMS1	-4.3558817	4.41115478	-4.8654712	6.29E-06	5.12E-05	3.5535568	RIMS1	assignmentsChromaffin_cells.Chrom_SOX2_statusHigh
assignmentsChromaffin_cells.Chrom_SOX2_statusHigh.ZNF280C	-1.0122684	4.24574094	-4.864923	6.28E-06	5.11E-05	3.55344861	ZNF280C	assignmentsChromaffin_cells.Chrom_SOX2_statusHigh
assignmentsChromaffin_cells.Chrom_SOX2_statusHigh.SNX12	0.85443685	3.39612479	4.86351866	6.32E-06	5.12E-05	3.55305701	SNX12	assignmentsChromaffin_cells.Chrom_SOX2_statusHigh
assignmentsChromaffin_cells.Chrom_SOX2_statusHigh.HAUS7	2.17629018	1.32931255	4.82960696	7.19E-06	5.71E-05	3.55175684	HAUS7	assignmentsChromaffin_cells.Chrom_SOX2_statusHigh
assignmentsChromaffin_cells.Chrom_SOX2_statusHigh.TAS2R4	-1.3662483	2.51837997	-4.8439651	6.81E-06	5.46E-05	3.55100876	TAS2R4	assignmentsChromaffin_cells.Chrom_SOX2_statusHigh
assignmentsChromaffin_cells.Chrom_SOX2_statusHigh.RAD21	0.85294422	6.20361454	4.89427345	5.61E-06	4.63E-05	3.54987486	RAD21	assignmentsChromaffin_cells.Chrom_SOX2_statusHigh
assignmentsChromaffin_cells.Chrom_SOX2_statusHigh.SEM1	1.20184897	6.67614445	4.90192852	5.45E-06	4.51E-05	3.54978072	SEM1	assignmentsChromaffin_cells.Chrom_SOX2_statusHigh
assignmentsChromaffin_cells.Chrom_SOX2_statusHigh.ARPP21	4.12216842	-0.5813449	4.81395829	7.64E-06	6.02E-05	3.53611917	ARPP21	assignmentsChromaffin_cells.Chrom_SOX2_statusHigh
assignmentsChromaffin_cells.Chrom_SOX2_statusHigh.OCIAD1-AS1	-2.4121189	1.13237844	-4.8127424	7.67E-06	6.03E-05	3.53597962	OCIAD1-AS1	assignmentsChromaffin_cells.Chrom_SOX2_statusHigh
assignmentsChromaffin_cells.Chrom_SOX2_statusHigh.N4BP2	-1.0392457	5.67539082	-4.880144	5.93E-06	4.85E-05	3.53548848	N4BP2	assignmentsChromaffin_cells.Chrom_SOX2_statusHigh
assignmentsChromaffin_cells.Chrom_SOX2_statusHigh.CBX3	1.18252217	6.13781389	4.89039664	5.70E-06	4.69E-05	3.52953633	CBX3	assignmentsChromaffin_cells.Chrom_SOX2_statusHigh
assignmentsChromaffin_cells.Chrom_SOX2_statusHigh.SETD9	1.82741193	2.12484129	4.83645863	7.01E-06	5.59E-05	3.52661587	SETD9	assignmentsChromaffin_cells.Chrom_SOX2_statusHigh
assignmentsChromaffin_cells.Chrom_SOX2_statusHigh.WDPCP	-0.9922371	7.53986148	-4.8963964	5.57E-06	4.59E-05	3.52497308	WDPCP	assignmentsChromaffin_cells.Chrom_SOX2_statusHigh
assignmentsChromaffin_cells.Chrom_SOX2_statusHigh.BCL3	3.18285908	2.88473367	4.8125258	7.69E-06	6.05E-05	3.52428096	BCL3	assignmentsChromaffin_cells.Chrom_SOX2_statusHigh
assignmentsChromaffin_cells.Chrom_SOX2_statusHigh.ZC3H8	-0.8464328	5.54272531	-4.8755537	6.03E-06	4.93E-05	3.52227979	ZC3H8	assignmentsChromaffin_cells.Chrom_SOX2_statusHigh
assignmentsChromaffin_cells.Chrom_SOX2_statusHigh.CHCHD3	-0.9030051	7.65216701	-4.8958257	5.58E-06	4.60E-05	3.51907665	CHCHD3	assignmentsChromaffin_cells.Chrom_SOX2_statusHigh
assignmentsChromaffin_cells.Chrom_SOX2_statusHigh.RAB28	-0.7932342	6.87571581	-4.8894831	5.72E-06	4.70E-05	3.51815431	RAB28	assignmentsChromaffin_cells.Chrom_SOX2_statusHigh

assignmentsChromaffin_cells.Chrom_SOX2_statusHigh.C1orf35	1.11624131	3.92449676	4.86005585	6.40E-06	5.18E-05	3.51171807	C1orf35	assignmentsChromaffin_cells.Chrom_SOX2_statusHigh
assignmentsChromaffin_cells.Chrom_SOX2_statusHigh.KIF13A	-1.2165601	7.10946634	-4.8805159	5.92E-06	4.85E-05	3.51105289	KIF13A	assignmentsChromaffin_cells.Chrom_SOX2_statusHigh
assignmentsChromaffin_cells.Chrom_SOX2_statusHigh.SERINC3	1.0395576	6.08729842	4.8837723	5.85E-06	4.80E-05	3.51090491	SERINC3	assignmentsChromaffin_cells.Chrom_SOX2_statusHigh
assignmentsChromaffin_cells.Chrom_SOX2_statusHigh.GNA11	1.11852157	3.93549702	4.85380859	6.56E-06	5.27E-05	3.49224805	GNA11	assignmentsChromaffin_cells.Chrom_SOX2_statusHigh
assignmentsChromaffin_cells.Chrom_SOX2_statusHigh.C12orf76	1.40435054	3.8662585	4.85477963	6.53E-06	5.26E-05	3.49178062	C12orf76	assignmentsChromaffin_cells.Chrom_SOX2_statusHigh
assignmentsChromaffin_cells.Chrom_SOX2_statusHigh.KCTD11	1.87133229	0.92188886	4.80546755	7.88E-06	6.19E-05	3.48841631	KCTD11	assignmentsChromaffin_cells.Chrom_SOX2_statusHigh
assignmentsChromaffin_cells.Chrom_SOX2_statusHigh.CCNT2-AS1	-1.6362615	3.84798253	-4.8386874	6.95E-06	5.56E-05	3.48461481	CCNT2-AS1	assignmentsChromaffin_cells.Chrom_SOX2_statusHigh
assignmentsChromaffin_cells.Chrom_SOX2_statusHigh.KDM7A	-1.1595003	6.05261318	-4.8580364	6.45E-06	5.21E-05	3.4842241	KDM7A	assignmentsChromaffin_cells.Chrom_SOX2_statusHigh
assignmentsChromaffin_cells.Chrom_SOX2_statusHigh.C3orf14	1.45034506	3.53668171	4.86148158	6.37E-06	5.16E-05	3.48165968	C3orf14	assignmentsChromaffin_cells.Chrom_SOX2_statusHigh
assignmentsChromaffin_cells.Chrom_SOX2_statusHigh.MT1F	4.23170597	-0.0937766	4.80952394	7.77E-06	6.10E-05	3.47668248	MT1F	assignmentsChromaffin_cells.Chrom_SOX2_statusHigh
assignmentsChromaffin_cells.Chrom_SOX2_statusHigh.SUGT1	0.8776135	6.01903731	4.87440614	6.06E-06	4.95E-05	3.47564141	SUGT1	assignmentsChromaffin_cells.Chrom_SOX2_statusHigh
assignmentsChromaffin_cells.Chrom_SOX2_statusHigh.PPP1R12B	-0.9982893	7.72859863	-4.8809028	5.91E-06	4.85E-05	3.46879662	PPP1R12B	assignmentsChromaffin_cells.Chrom_SOX2_statusHigh
assignmentsChromaffin_cells.Chrom_SOX2_statusHigh.C12orf4	-1.0626804	5.21628017	-4.851902	6.60E-06	5.31E-05	3.46498939	C12orf4	assignmentsChromaffin_cells.Chrom_SOX2_statusHigh
assignmentsChromaffin_cells.Chrom_SOX2_statusHigh.RPAIN	1.02012272	5.29631297	4.86437792	6.30E-06	5.12E-05	3.46239652	RPAIN	assignmentsChromaffin_cells.Chrom_SOX2_statusHigh
assignmentsChromaffin_cells.Chrom_SOX2_statusHigh.RARA-AS1	1.93684929	1.38863985	4.80155538	8.00E-06	6.27E-05	3.45964057	RARA-AS1	assignmentsChromaffin_cells.Chrom_SOX2_statusHigh
assignmentsChromaffin_cells.Chrom_SOX2_statusHigh.PSMG1	0.99698166	3.7486244	4.84427947	6.80E-06	5.46E-05	3.4518911	PSMG1	assignmentsChromaffin_cells.Chrom_SOX2_statusHigh
assignmentsChromaffin_cells.Chrom_SOX2_statusHigh.MAD2L1BP	1.63872903	2.58309456	4.82744366	7.25E-06	5.75E-05	3.44901832	MAD2L1BP	assignmentsChromaffin_cells.Chrom_SOX2_statusHigh
assignmentsChromaffin_cells.Chrom_SOX2_statusHigh.UCK1	1.11414043	2.46764934	4.82201502	7.40E-06	5.85E-05	3.44815095	UCK1	assignmentsChromaffin_cells.Chrom_SOX2_statusHigh
assignmentsChromaffin_cells.Chrom_SOX2_statusHigh.ZNF609	-1.3339555	7.83022524	-4.8722919	6.11E-06	4.98E-05	3.44148457	ZNF609	assignmentsChromaffin_cells.Chrom_SOX2_statusHigh
assignmentsChromaffin_cells.Chrom_SOX2_statusHigh.RPL7	3.28111016	5.99302959	4.86894565	6.21E-06	5.06E-05	3.43764617	RPL7	assignmentsChromaffin_cells.Chrom_SOX2_statusHigh
assignmentsChromaffin_cells.Chrom_SOX2_statusHigh.RANGRF	1.69248025	2.90881215	4.82778547	7.24E-06	5.74E-05	3.433225	RANGRF	assignmentsChromaffin_cells.Chrom_SOX2_statusHigh
assignmentsChromaffin_cells.Chrom_SOX2_statusHigh.MZT2A	1.52173449	4.68825441	4.85500812	6.53E-06	5.26E-05	3.43084518	MZT2A	assignmentsChromaffin_cells.Chrom_SOX2_statusHigh
assignmentsChromaffin_cells.Chrom_SOX2_statusHigh.PCGF1	1.50338678	2.82114247	4.82786935	7.24E-06	5.74E-05	3.42851553	PCGF1	assignmentsChromaffin_cells.Chrom_SOX2_statusHigh
assignmentsChromaffin_cells.Chrom_SOX2_statusHigh.BIRC6	-0.8074604	8.87471999	-4.8798142	5.93E-06	4.86E-05	3.41775879	BIRC6	assignmentsChromaffin_cells.Chrom_SOX2_statusHigh
assignmentsChromaffin_cells.Chrom_SOX2_statusHigh.NPM3	1.78330558	1.48811244	4.80097655	8.02E-06	6.28E-05	3.41385	NPM3	assignmentsChromaffin_cells.Chrom_SOX2_statusHigh
assignmentsChromaffin_cells.Chrom_SOX2_statusHigh.RPP25	4.73561659	0.20407702	4.78341518	8.60E-06	6.68E-05	3.41380174	RPP25	assignmentsChromaffin_cells.Chrom_SOX2_statusHigh
assignmentsChromaffin_cells.Chrom_SOX2_statusHigh.RANBP10	-1.2502806	5.25013022	-4.8345332	7.06E-06	5.62E-05	3.40871652	RANBP10	assignmentsChromaffin_cells.Chrom_SOX2_statusHigh
assignmentsChromaffin_cells.Chrom_SOX2_statusHigh.BRAF	-0.9613364	8.45115209	-4.872519	6.10E-06	4.98E-05	3.4069834	BRAF	assignmentsChromaffin_cells.Chrom_SOX2_statusHigh
assignmentsChromaffin_cells.Chrom_SOX2_statusHigh.SVBP	1.66715107	2.8130065	4.8139758	7.63E-06	6.01E-05	3.40516768	SVBP	assignmentsChromaffin_cells.Chrom_SOX2_statusHigh
assignmentsChromaffin_cells.Chrom_SOX2_statusHigh.PGAM2	3.37469523	-0.0855626	4.77296925	8.93E-06	6.91E-05	3.40031914	PGAM2	assignmentsChromaffin_cells.Chrom_SOX2_statusHigh
assignmentsChromaffin_cells.Chrom_SOX2_statusHigh.TBCD	-1.0684858	6.67967784	-4.8471365	6.73E-06	5.40E-05	3.39935124	TBCD	assignmentsChromaffin_cells.Chrom_SOX2_statusHigh
assignmentsChromaffin_cells.Chrom_SOX2_statusHigh.PLAAT3	2.18321818	4.39479567	4.8327679	7.12E-06	5.66E-05	3.39420876	PLAAT3	assignmentsChromaffin_cells.Chrom_SOX2_statusHigh
assignmentsChromaffin_cells.Chrom_SOX2_statusHigh.MYL6	2.25912948	7.29664355	4.8643799	6.32E-06	5.13E-05	3.39008639	MYL6	assignmentsChromaffin_cells.Chrom_SOX2_statusHigh
assignmentsChromaffin_cells.Chrom_SOX2_statusHigh.ATG2B	-1.2872989	6.31371975	-4.8424024	6.85E-06	5.49E-05	3.38536876	ATG2B	assignmentsChromaffin_cells.Chrom_SOX2_statusHigh
assignmentsChromaffin_cells.Chrom_SOX2_statusHigh.NUP214	-0.9307557	6.41922042	-4.8423092	6.85E-06	5.49E-05	3.38275567	NUP214	assignmentsChromaffin_cells.Chrom_SOX2_statusHigh
assignmentsChromaffin_cells.Chrom_SOX2_statusHigh.RPS8	3.1691221	7.49153255	4.8652506	6.30E-06	5.12E-05	3.3799637	RPS8	assignmentsChromaffin_cells.Chrom_SOX2_statusHigh
assignmentsChromaffin_cells.Chrom_SOX2_statusHigh.M6PR	1.08851352	5.57116634	4.83867873	6.95E-06	5.56E-05	3.37133616	M6PR	assignmentsChromaffin_cells.Chrom_SOX2_statusHigh
assignmentsChromaffin_cells.Chrom_SOX2_statusHigh.DDRGK1	1.22733494	4.85707421	4.83618114	7.01E-06	5.59E-05	3.37103735	DDRGK1	assignmentsChromaffin_cells.Chrom_SOX2_statusHigh
assignmentsChromaffin_cells.Chrom_SOX2_statusHigh.PSMA3-AS1	-0.7386017	6.15443333	-4.8356993	7.03E-06	5.60E-05	3.36960877	PSMA3-AS1	assignmentsChromaffin_cells.Chrom_SOX2_statusHigh
assignmentsChromaffin_cells.Chrom_SOX2_statusHigh.FAU	3.36102344	6.77919679	4.85681542	6.51E-06	5.25E-05	3.36926796	FAU	assignmentsChromaffin_cells.Chrom_SOX2_statusHigh
assignmentsChromaffin_cells.Chrom_SOX2_statusHigh.OSTM1	0.91824585	5.70188633	4.84258193	6.84E-06	5.49E-05	3.36918985	OSTM1	assignmentsChromaffin_cells.Chrom_SOX2_statusHigh
assignmentsChromaffin_cells.Chrom_SOX2_statusHigh.UBXN1	1.88344997	5.11223765	4.83701851	7.00E-06	5.59E-05	3.36818125	UBXN1	assignmentsChromaffin_cells.Chrom_SOX2_statusHigh
assignmentsChromaffin_cells.Chrom_SOX2_statusHigh.LINC02334	-3.1469944	1.87153894	-4.7726749	8.94E-06	6.91E-05	3.3661814	LINC02334	assignmentsChromaffin_cells.Chrom_SOX2_statusHigh
assignmentsChromaffin_cells.Chrom_SOX2_statusHigh.FDX2	1.57494119	3.38408946	4.81988256	7.46E-06	5.90E-05	3.3589271	FDX2	assignmentsChromaffin_cells.Chrom_SOX2_statusHigh
assignmentsChromaffin_cells.Chrom_SOX2_statusHigh.PSMB2	1.28766783	4.96600099	4.82743424	7.25E-06	5.75E-05	3.35629978	PSMB2	assignmentsChromaffin_cells.Chrom_SOX2_statusHigh
assignmentsChromaffin_cells.Chrom_SOX2_statusHigh.TNPO3	-0.7404205	6.49180499	-4.838303	6.96E-06	5.56E-05	3.3551991	TNPO3	assignmentsChromaffin_cells.Chrom_SOX2_statusHigh
assignmentsChromaffin_cells.Chrom_SOX2_statusHigh.POLD4	1.81823725	4.32340365	4.81659808	7.56E-06	5.96E-05	3.35515559	POLD4	assignmentsChromaffin_cells.Chrom_SOX2_statusHigh
assignmentsChromaffin_cells.Chrom_SOX2_statusHigh.IWS1	-0.8614058	6.7131729	-4.8370894	6.99E-06	5.58E-05	3.34910433	IWS1	assignmentsChromaffin_cells.Chrom_SOX2_statusHigh
assignmentsChromaffin_cells.Chrom_SOX2_statusHigh.MZT2B	2.52492758	4.84349998	4.83289192	7.13E-06	5.67E-05	3.34662214	MZT2B	assignmentsChromaffin_cells.Chrom_SOX2_statusHigh
assignmentsChromaffin_cells.Chrom_SOX2_statusHigh.AHSA1	1.75062394	4.94371321	4.82797717	7.24E-06	5.74E-05	3.34574038	AHSA1	assignmentsChromaffin_cells.Chrom_SOX2_statusHigh
assignmentsChromaffin_cells.Chrom_SOX2_statusHigh.C6orf47	1.34486603	2.62385361	4.79029412	8.35E-06	6.51E-05	3.34272353	C6orf47	assignmentsChromaffin_cells.Chrom_SOX2_statusHigh
assignmentsChromaffin_cells.Chrom_SOX2_statusHigh.SNRPD1	1.62883825	4.75281226	4.82559968	7.30E-06	5.78E-05	3.33865552	SNRPD1	assignmentsChromaffin_cells.Chrom_SOX2_statusHigh
assignmentsChromaffin_cells.Chrom_SOX2_statusHigh.SELENOM	3.58883999	3.9764267	4.81829803	7.54E-06	5.95E-05	3.3370319	SELENOM	assignmentsChromaffin_cells.Chrom_SOX2_statusHigh
assignmentsChromaffin_cells.Chrom_SOX2_statusHigh.SOD3	3.81253161	1.17950951	4.75319859	9.63E-06	7.34E-05	3.33276164	SOD3	assignmentsChromaffin_cells.Chrom_SOX2_statusHigh
assignmentsChromaffin_cells.Chrom_SOX2_statusHigh.ALYREF	1.88981306	2.68101305	4.79449292	8.22E-06	6.42E-05	3.33255968	ALYREF	assignmentsChromaffin_cells.Chrom_SOX2_statusHigh
assignmentsChromaffin_cells.Chrom_SOX2_statusHigh.RXYLT1	1.22359763	3.93188584	4.81117079	7.71E-06	6.06E-05	3.33131116	RXYLT1	assignmentsChromaffin_cells.Chrom_SOX2_statusHigh
assignmentsChromaffin_cells.Chrom_SOX2_statusHigh.USP37	-1.2050422	6.52047391	-4.8287926	7.21E-06	5.73E-05	3.32594712	USP37	assignmentsChromaffin_cells.Chrom_SOX2_statusHigh
assignmentsChromaffin_cells.Chrom_SOX2_statusHigh.LINC02851	3.34663427	-0.1901629	4.75081321	9.71E-06	7.39E-05	3.32508778	LINC02851	assignmentsChromaffin_cells.Chrom_SOX2_statusHigh
assignmentsChromaffin_cells.Chrom_SOX2_statusHigh.BTRC	-0.9082257	7.18656318	-4.8374906	6.98E-06	5.58E-05	3.31952866	BTRC	assignmentsChromaffin_cells.Chrom_SOX2_statusHigh
assignmentsChromaffin_cells.Chrom_SOX2_statusHigh.MTFP1	2.6091769	1.55542036	4.75744389	9.46E-06	7.24E-05	3.31881951	MTFP1	assignmentsChromaffin_cells.Chrom_SOX2_statusHigh
assignmentsChromaffin_cells.Chrom_SOX2_statusHigh.CUL3	-1.0859396	7.39748983	-4.8301754	7.18E-06	5.71E-05	3.3062079	CUL3	assignmentsChromaffin_cells.Chrom_SOX2_statusHigh
assignmentsChromaffin_cells.Chrom_SOX2_statusHigh.TLK2	-0.98714	6.87231488	-4.82568	7.30E-06	5.78E-05	3.30566541	TLK2	assignmentsChromaffin_cells.Chrom_SOX2_statusHigh
assignmentsChromaffin_cells.Chrom_SOX2_statusHigh.RAF1	-0.9498878	6.6241125	-4.8190802	7.49E-06	5.91E-05	3.29778043	RAF1	assignmentsChromaffin_cells.Chrom_SOX2_statusHigh
assignmentsChromaffin_cells.Chrom_SOX2_statusHigh.C21orf62-A	-1.4247925	4.57281938	-4.8012528	8.01E-06	6.28E-05	3.28905964	C21orf62-AS	assignmentsChromaffin_cells.Chrom_SOX2_statusHigh

assignmentsChromaffin_cells.Chrom_SOX2_statusHigh.LRRTM2	1.51695735	1.4639263	4.77447707	8.87E-06	6.87E-05	3.28901138	LRRTM2	assignmentsChromaffin_cells.Chrom_SOX2_statusHigh
assignmentsChromaffin_cells.Chrom_SOX2_statusHigh.SLC25A1	1.42942266	3.65215327	4.79801238	8.11E-06	6.35E-05	3.28460603	SLC25A1	assignmentsChromaffin_cells.Chrom_SOX2_statusHigh
assignmentsChromaffin_cells.Chrom_SOX2_statusHigh.ARMT1	1.03716576	3.32547996	4.78087725	8.66E-06	6.71E-05	3.27752004	ARMT1	assignmentsChromaffin_cells.Chrom_SOX2_statusHigh
assignmentsChromaffin_cells.Chrom_SOX2_statusHigh.UVRAG	-1.0885526	7.81200779	-4.823827	7.35E-06	5.81E-05	3.26917964	UVRAG	assignmentsChromaffin_cells.Chrom_SOX2_statusHigh
assignmentsChromaffin_cells.Chrom_SOX2_statusHigh.EXOC3L1	2.52788694	0.69921744	4.73435793	1.03E-05	7.79E-05	3.26781465	EXOC3L1	assignmentsChromaffin_cells.Chrom_SOX2_statusHigh
assignmentsChromaffin_cells.Chrom_SOX2_statusHigh.HMGB1	1.4462271	7.93041195	4.83475688	7.05E-06	5.62E-05	3.26549286	HMGB1	assignmentsChromaffin_cells.Chrom_SOX2_statusHigh
assignmentsChromaffin_cells.Chrom_SOX2_statusHigh.VPS13C	-1.0947992	8.94505072	-4.8417656	6.86E-06	5.50E-05	3.26408913	VPS13C	assignmentsChromaffin_cells.Chrom_SOX2_statusHigh
assignmentsChromaffin_cells.Chrom_SOX2_statusHigh.POLE2	-1.649218	2.82715562	-4.7531814	9.61E-06	7.34E-05	3.26393045	POLE2	assignmentsChromaffin_cells.Chrom_SOX2_statusHigh
assignmentsChromaffin_cells.Chrom_SOX2_statusHigh.HSD17B8	1.67807153	2.00117087	4.77290553	8.92E-06	6.91E-05	3.26212391	HSD17B8	assignmentsChromaffin_cells.Chrom_SOX2_statusHigh
assignmentsChromaffin_cells.Chrom_SOX2_statusHigh.TIMMDC1	1.04304307	5.08832812	4.80358434	7.94E-06	6.23E-05	3.25673586	TIMMDC1	assignmentsChromaffin_cells.Chrom_SOX2_statusHigh
assignmentsChromaffin_cells.Chrom_SOX2_statusHigh.BLOC1S4	1.85222767	2.49638226	4.76877524	9.06E-06	6.97E-05	3.25368903	BLOC1S4	assignmentsChromaffin_cells.Chrom_SOX2_statusHigh
assignmentsChromaffin_cells.Chrom_SOX2_statusHigh.USP25	-1.0602747	6.64364231	-4.7969336	8.14E-06	6.37E-05	3.24958511	USP25	assignmentsChromaffin_cells.Chrom_SOX2_statusHigh
assignmentsChromaffin_cells.Chrom_SOX2_statusHigh.WDR5B	1.3294674	1.97449184	4.76068007	9.35E-06	7.17E-05	3.24524218	WDR5B	assignmentsChromaffin_cells.Chrom_SOX2_statusHigh
assignmentsChromaffin_cells.Chrom_SOX2_statusHigh.TBC1D5	-0.96426	8.67320959	-4.8243208	7.34E-06	5.81E-05	3.24252244	TBC1D5	assignmentsChromaffin_cells.Chrom_SOX2_statusHigh
assignmentsChromaffin_cells.Chrom_SOX2_statusHigh.GCSH	1.49284724	3.81377913	4.79127738	8.32E-06	6.49E-05	3.24224412	GCSH	assignmentsChromaffin_cells.Chrom_SOX2_statusHigh
assignmentsChromaffin_cells.Chrom_SOX2_statusHigh.GLRX5	1.93312607	3.42147595	4.7837138	8.57E-06	6.66E-05	3.23986592	GLRX5	assignmentsChromaffin_cells.Chrom_SOX2_statusHigh
assignmentsChromaffin_cells.Chrom_SOX2_statusHigh.RGPD8	-1.4537736	4.15626291	-4.7716161	8.97E-06	6.92E-05	3.23546771	RGPD8	assignmentsChromaffin_cells.Chrom_SOX2_statusHigh
assignmentsChromaffin_cells.Chrom_SOX2_statusHigh.PTOV1	1.3730999	5.1157655	4.8035303	7.94E-06	6.23E-05	3.23291732	PTOV1	assignmentsChromaffin_cells.Chrom_SOX2_statusHigh
assignmentsChromaffin_cells.Chrom_SOX2_statusHigh.LARGE1	-1.8398034	7.8228143	-4.8182921	7.52E-06	5.94E-05	3.23274415	LARGE1	assignmentsChromaffin_cells.Chrom_SOX2_statusHigh
assignmentsChromaffin_cells.Chrom_SOX2_statusHigh.FABP5	3.92658734	3.11392522	4.75201294	9.69E-06	7.38E-05	3.23195923	FABP5	assignmentsChromaffin_cells.Chrom_SOX2_statusHigh
assignmentsChromaffin_cells.Chrom_SOX2_statusHigh.MRPL35	1.22029323	3.51024587	4.77741504	8.77E-06	6.80E-05	3.22886227	MRPL35	assignmentsChromaffin_cells.Chrom_SOX2_statusHigh
assignmentsChromaffin_cells.Chrom_SOX2_statusHigh.TM9SF2	0.69799569	6.82962806	4.81357334	7.64E-06	6.02E-05	3.22816016	TM9SF2	assignmentsChromaffin_cells.Chrom_SOX2_statusHigh
assignmentsChromaffin_cells.Chrom_SOX2_statusHigh.PDXP	1.95633298	1.89124073	4.761053	9.33E-06	7.17E-05	3.22682635	PDXP	assignmentsChromaffin_cells.Chrom_SOX2_statusHigh
assignmentsChromaffin_cells.Chrom_SOX2_statusHigh.UBE2Q1	1.31536412	3.98609619	4.78240565	8.61E-06	6.68E-05	3.22376192	UBE2Q1	assignmentsChromaffin_cells.Chrom_SOX2_statusHigh
assignmentsChromaffin_cells.Chrom_SOX2_statusHigh.STX10	1.43862667	3.63221757	4.77570392	8.83E-06	6.84E-05	3.22199397	STX10	assignmentsChromaffin_cells.Chrom_SOX2_statusHigh
assignmentsChromaffin_cells.Chrom_SOX2_statusHigh.TMEM179B	1.7475444	4.50679661	4.78723372	8.45E-06	6.58E-05	3.2189253	TMEM179B	assignmentsChromaffin_cells.Chrom_SOX2_statusHigh
assignmentsChromaffin_cells.Chrom_SOX2_statusHigh.LDLRAP1	2.77846777	2.0041881	4.71978597	1.09E-05	8.17E-05	3.21785402	LDLRAP1	assignmentsChromaffin_cells.Chrom_SOX2_statusHigh
assignmentsChromaffin_cells.Chrom_SOX2_statusHigh.BLVRB	2.86968514	3.72141578	4.7587829	9.45E-06	7.24E-05	3.21200721	BLVRB	assignmentsChromaffin_cells.Chrom_SOX2_statusHigh
assignmentsChromaffin_cells.Chrom_SOX2_statusHigh.TMSB4X	3.75349064	7.33784304	4.79674068	8.18E-06	6.40E-05	3.20889219	TMSB4X	assignmentsChromaffin_cells.Chrom_SOX2_statusHigh
assignmentsChromaffin_cells.Chrom_SOX2_statusHigh.PTGES2	0.90428904	4.31128027	4.78446899	8.54E-06	6.64E-05	3.20227735	PTGES2	assignmentsChromaffin_cells.Chrom_SOX2_statusHigh
assignmentsChromaffin_cells.Chrom_SOX2_statusHigh.AP2M1	1.78332201	5.16056273	4.79173671	8.31E-06	6.49E-05	3.19920367	AP2M1	assignmentsChromaffin_cells.Chrom_SOX2_statusHigh
assignmentsChromaffin_cells.Chrom_SOX2_statusHigh.CD14	3.45758878	2.65552567	4.72251441	1.08E-05	8.12E-05	3.19612695	CD14	assignmentsChromaffin_cells.Chrom_SOX2_statusHigh
assignmentsChromaffin_cells.Chrom_SOX2_statusHigh.CUX1	-1.365813	8.49921612	-4.8113906	7.71E-06	6.06E-05	3.18754316	CUX1	assignmentsChromaffin_cells.Chrom_SOX2_statusHigh
assignmentsChromaffin_cells.Chrom_SOX2_statusHigh.DNAJB9	2.27838857	4.75017399	4.79305706	8.30E-06	6.47E-05	3.1861325	DNAJB9	assignmentsChromaffin_cells.Chrom_SOX2_statusHigh
assignmentsChromaffin_cells.Chrom_SOX2_statusHigh.TRAK2	-1.0193065	6.47023865	-4.7938712	8.24E-06	6.44E-05	3.18455441	TRAK2	assignmentsChromaffin_cells.Chrom_SOX2_statusHigh
assignmentsChromaffin_cells.Chrom_SOX2_statusHigh.PFDN6	1.58339647	3.26856298	4.76194469	9.30E-06	7.15E-05	3.18293111	PFDN6	assignmentsChromaffin_cells.Chrom_SOX2_statusHigh
assignmentsChromaffin_cells.Chrom_SOX2_statusHigh.HCST	3.14067012	1.21142047	4.70960504	1.13E-05	8.44E-05	3.1828587	HCST	assignmentsChromaffin_cells.Chrom_SOX2_statusHigh
assignmentsChromaffin_cells.Chrom_SOX2_statusHigh.CCDC188	2.5679734	0.21539674	4.72017465	1.09E-05	8.16E-05	3.18145799	CCDC188	assignmentsChromaffin_cells.Chrom_SOX2_statusHigh
assignmentsChromaffin_cells.Chrom_SOX2_statusHigh.DNALI1	1.74161251	1.59339235	4.7380156	1.02E-05	7.71E-05	3.17977024	DNALI1	assignmentsChromaffin_cells.Chrom_SOX2_statusHigh
assignmentsChromaffin_cells.Chrom_SOX2_statusHigh.MRPL50	1.32991032	3.74623495	4.7690112	9.05E-06	6.97E-05	3.17877027	MRPL50	assignmentsChromaffin_cells.Chrom_SOX2_statusHigh
assignmentsChromaffin_cells.Chrom_SOX2_statusHigh.FGFBP2	3.51599187	-0.597813	4.73203786	1.04E-05	7.86E-05	3.17257629	FGFBP2	assignmentsChromaffin_cells.Chrom_SOX2_statusHigh
assignmentsChromaffin_cells.Chrom_SOX2_statusHigh.LINC01023	2.14502986	1.35682275	4.73659144	1.02E-05	7.74E-05	3.1708574	LINC01023	assignmentsChromaffin_cells.Chrom_SOX2_statusHigh
assignmentsChromaffin_cells.Chrom_SOX2_statusHigh.SDHC	0.8886054	5.43829582	4.78440107	8.54E-06	6.64E-05	3.1649917	SDHC	assignmentsChromaffin_cells.Chrom_SOX2_statusHigh
assignmentsChromaffin_cells.Chrom_SOX2_statusHigh.TXN	1.84280347	5.44740495	4.78359888	8.58E-06	6.66E-05	3.16471429	TXN	assignmentsChromaffin_cells.Chrom_SOX2_statusHigh
assignmentsChromaffin_cells.Chrom_SOX2_statusHigh.MRPL38	1.21811683	4.27142544	4.77180338	8.96E-06	6.91E-05	3.16326499	MRPL38	assignmentsChromaffin_cells.Chrom_SOX2_statusHigh
assignmentsChromaffin_cells.Chrom_SOX2_statusHigh.TCIRG1	2.45483558	4.102433	4.75709534	9.50E-06	7.27E-05	3.16306946	TCIRG1	assignmentsChromaffin_cells.Chrom_SOX2_statusHigh
assignmentsChromaffin_cells.Chrom_SOX2_statusHigh.PROKR1	2.77449526	0.07164444	4.71663824	1.11E-05	8.27E-05	3.15590402	PROKR1	assignmentsChromaffin_cells.Chrom_SOX2_statusHigh
assignmentsChromaffin_cells.Chrom_SOX2_statusHigh.INPP5B	-1.357428	5.47635315	-4.7606038	9.35E-06	7.17E-05	3.1521846	INPP5B	assignmentsChromaffin_cells.Chrom_SOX2_statusHigh
assignmentsChromaffin_cells.Chrom_SOX2_statusHigh.CCDC28A	1.38618095	3.83512939	4.75535897	9.54E-06	7.29E-05	3.14685866	CCDC28A	assignmentsChromaffin_cells.Chrom_SOX2_statusHigh
assignmentsChromaffin_cells.Chrom_SOX2_statusHigh.RABGAP1	-1.122078	7.55902862	-4.7873967	8.44E-06	6.58E-05	3.14396655	RABGAP1	assignmentsChromaffin_cells.Chrom_SOX2_statusHigh
assignmentsChromaffin_cells.Chrom_SOX2_statusHigh.MIATNB	-1.1777206	4.90925586	-4.7523683	9.64E-06	7.35E-05	3.14226608	MIATNB	assignmentsChromaffin_cells.Chrom_SOX2_statusHigh
assignmentsChromaffin_cells.Chrom_SOX2_statusHigh.SNF8	1.01601709	4.93111577	4.77204284	8.95E-06	6.91E-05	3.14209007	SNF8	assignmentsChromaffin_cells.Chrom_SOX2_statusHigh
assignmentsChromaffin_cells.Chrom_SOX2_statusHigh.SLC25A16	-0.8156978	5.64026533	-4.7699462	9.02E-06	6.96E-05	3.14001164	SLC25A16	assignmentsChromaffin_cells.Chrom_SOX2_statusHigh
assignmentsChromaffin_cells.Chrom_SOX2_statusHigh.SMOC1	4.53214619	0.8318803	4.71268951	1.12E-05	8.38E-05	3.13885641	SMOC1	assignmentsChromaffin_cells.Chrom_SOX2_statusHigh
assignmentsChromaffin_cells.Chrom_SOX2_statusHigh.EIF1AX	1.76210136	5.94164988	4.78123421	8.65E-06	6.71E-05	3.13201962	EIF1AX	assignmentsChromaffin_cells.Chrom_SOX2_statusHigh
assignmentsChromaffin_cells.Chrom_SOX2_statusHigh.NOX4	-4.0008469	4.13774907	-4.7095521	1.14E-05	8.46E-05	3.13180141	NOX4	assignmentsChromaffin_cells.Chrom_SOX2_statusHigh
assignmentsChromaffin_cells.Chrom_SOX2_statusHigh.FAM200B	1.26791572	4.5929018	4.769102	9.05E-06	6.97E-05	3.12911905	FAM200B	assignmentsChromaffin_cells.Chrom_SOX2_statusHigh
assignmentsChromaffin_cells.Chrom_SOX2_statusHigh.C6orf132	2.36696173	0.14746848	4.69002776	1.22E-05	8.99E-05	3.11482359	C6orf132	assignmentsChromaffin_cells.Chrom_SOX2_statusHigh
assignmentsChromaffin_cells.Chrom_SOX2_statusHigh.TMEM91	-1.1962862	5.1679792	-4.7528139	9.63E-06	7.34E-05	3.1139141	TMEM91	assignmentsChromaffin_cells.Chrom_SOX2_statusHigh
assignmentsChromaffin_cells.Chrom_SOX2_statusHigh.NEK1	-1.1615106	8.0628248	-4.7924247	8.28E-06	6.47E-05	3.1109389	NEK1	assignmentsChromaffin_cells.Chrom_SOX2_statusHigh
assignmentsChromaffin_cells.Chrom_SOX2_statusHigh.P4HA2	1.89557886	4.26046753	4.75885572	9.42E-06	7.22E-05	3.11079662	P4HA2	assignmentsChromaffin_cells.Chrom_SOX2_statusHigh
assignmentsChromaffin_cells.Chrom_SOX2_statusHigh.GAR1	0.91343501	3.75228329	4.74401953	9.95E-06	7.57E-05	3.10500695	GAR1	assignmentsChromaffin_cells.Chrom_SOX2_statusHigh
assignmentsChromaffin_cells.Chrom_SOX2_statusHigh.RRP7A	1.13065849	4.28542533	4.74895272	9.77E-06	7.43E-05	3.10392186	RRP7A	assignmentsChromaffin_cells.Chrom_SOX2_statusHigh
assignmentsChromaffin_cells.Chrom_SOX2_statusHigh.PRPF31	1.36822438	4.08521505	4.75347229	9.60E-06	7.34E-05	3.10080623	PRPF31	assignmentsChromaffin_cells.Chrom_SOX2_statusHigh

assignmentsChromaffin_cells.Chrom_SOX2_statusHigh.EEF1D	1.67835196	6.40493993	4.77254403	8.94E-06	6.91E-05	3.09416729	EEF1D	assignmentsChromaffin_cells.Chrom_SOX2_statusHigh
assignmentsChromaffin_cells.Chrom_SOX2_statusHigh.AGPAT3	-1.0480246	5.97695255	-4.7533853	9.61E-06	7.34E-05	3.09238171	AGPAT3	assignmentsChromaffin_cells.Chrom_SOX2_statusHigh
assignmentsChromaffin_cells.Chrom_SOX2_statusHigh.POLA1	-0.9417599	6.26669931	-4.7596919	9.38E-06	7.19E-05	3.08948371	POLA1	assignmentsChromaffin_cells.Chrom_SOX2_statusHigh
assignmentsChromaffin_cells.Chrom_SOX2_statusHigh.DPCD	1.29570141	2.94115742	4.74126604	1.01E-05	7.63E-05	3.08741256	DPCD	assignmentsChromaffin_cells.Chrom_SOX2_statusHigh
assignmentsChromaffin_cells.Chrom_SOX2_statusHigh.RCOR3	-0.9015113	6.99182169	-4.7724549	8.94E-06	6.91E-05	3.08522534	RCOR3	assignmentsChromaffin_cells.Chrom_SOX2_statusHigh
assignmentsChromaffin_cells.Chrom_SOX2_statusHigh.NUDT14	1.95135142	2.81531414	4.72699007	1.06E-05	7.98E-05	3.08492494	NUDT14	assignmentsChromaffin_cells.Chrom_SOX2_statusHigh
assignmentsChromaffin_cells.Chrom_SOX2_statusHigh.CYB5R3	1.37257933	5.45201891	4.75570797	9.52E-06	7.28E-05	3.07178865	CYB5R3	assignmentsChromaffin_cells.Chrom_SOX2_statusHigh
assignmentsChromaffin_cells.Chrom_SOX2_statusHigh.ANXA2	3.05793117	5.63382238	4.72335213	1.08E-05	8.10E-05	3.07150716	ANXA2	assignmentsChromaffin_cells.Chrom_SOX2_statusHigh
assignmentsChromaffin_cells.Chrom_SOX2_statusHigh.ORC6	1.55027883	2.11426555	4.71184582	1.12E-05	8.38E-05	3.0696942	ORC6	assignmentsChromaffin_cells.Chrom_SOX2_statusHigh
assignmentsChromaffin_cells.Chrom_SOX2_statusHigh.ASMTL-AS1	-1.7064075	3.14733036	-4.708564	1.14E-05	8.46E-05	3.06954846	ASMTL-AS1	assignmentsChromaffin_cells.Chrom_SOX2_statusHigh
assignmentsChromaffin_cells.Chrom_SOX2_statusHigh.RPL36A	2.61464435	6.38488135	4.76985133	9.06E-06	6.97E-05	3.06775513	RPL36A	assignmentsChromaffin_cells.Chrom_SOX2_statusHigh
assignmentsChromaffin_cells.Chrom_SOX2_statusHigh.SYNGAP1	-1.0717163	5.25759426	-4.7495814	9.75E-06	7.42E-05	3.06774261	SYNGAP1	assignmentsChromaffin_cells.Chrom_SOX2_statusHigh
assignmentsChromaffin_cells.Chrom_SOX2_statusHigh.NDUFAF4	1.44043463	3.84392032	4.74050084	1.01E-05	7.65E-05	3.06550702	NDUFAF4	assignmentsChromaffin_cells.Chrom_SOX2_statusHigh
assignmentsChromaffin_cells.Chrom_SOX2_statusHigh.PIGN	-0.9792392	6.07463423	-4.7513843	9.68E-06	7.38E-05	3.06120144	PIGN	assignmentsChromaffin_cells.Chrom_SOX2_statusHigh
assignmentsChromaffin_cells.Chrom_SOX2_statusHigh.ATF5	1.6551083	3.21334109	4.73032369	1.05E-05	7.89E-05	3.05954967	ATF5	assignmentsChromaffin_cells.Chrom_SOX2_statusHigh
assignmentsChromaffin_cells.Chrom_SOX2_statusHigh.NOSIP	1.21365677	4.55542655	4.7382071	1.02E-05	7.71E-05	3.05513602	NOSIP	assignmentsChromaffin_cells.Chrom_SOX2_statusHigh
assignmentsChromaffin_cells.Chrom_SOX2_statusHigh.PRR7	1.71097202	1.58697186	4.70390771	1.16E-05	8.59E-05	3.04185287	PRR7	assignmentsChromaffin_cells.Chrom_SOX2_statusHigh
assignmentsChromaffin_cells.Chrom_SOX2_statusHigh.TET1	-1.829429	4.98472619	-4.7319408	1.04E-05	7.86E-05	3.03986928	TET1	assignmentsChromaffin_cells.Chrom_SOX2_statusHigh
assignmentsChromaffin_cells.Chrom_SOX2_statusHigh.NOS2	3.1100264	-0.2074609	4.67690956	1.28E-05	9.35E-05	3.03936566	NOS2	assignmentsChromaffin_cells.Chrom_SOX2_statusHigh
assignmentsChromaffin_cells.Chrom_SOX2_statusHigh.EPC1	-0.7791316	7.82097942	-4.7634042	9.25E-06	7.11E-05	3.03855166	EPC1	assignmentsChromaffin_cells.Chrom_SOX2_statusHigh
assignmentsChromaffin_cells.Chrom_SOX2_statusHigh.HOXB2	1.58819849	4.05401794	4.735761	1.03E-05	7.76E-05	3.03040374	HOXB2	assignmentsChromaffin_cells.Chrom_SOX2_statusHigh
assignmentsChromaffin_cells.Chrom_SOX2_statusHigh.GMDS-DT	-1.019288	7.66794347	-4.7608271	9.34E-06	7.17E-05	3.02741554	GMDS-DT	assignmentsChromaffin_cells.Chrom_SOX2_statusHigh
assignmentsChromaffin_cells.Chrom_SOX2_statusHigh.RBM15B	1.4216154	2.49077294	4.7032587	1.16E-05	8.60E-05	3.02415145	RBM15B	assignmentsChromaffin_cells.Chrom_SOX2_statusHigh
assignmentsChromaffin_cells.Chrom_SOX2_statusHigh.USP42	-0.6512427	5.75774896	-4.7375278	1.02E-05	7.72E-05	3.02373656	USP42	assignmentsChromaffin_cells.Chrom_SOX2_statusHigh
assignmentsChromaffin_cells.Chrom_SOX2_statusHigh.SSH2	-1.1565272	8.32638519	-4.7604023	9.36E-06	7.18E-05	3.02186978	SSH2	assignmentsChromaffin_cells.Chrom_SOX2_statusHigh
assignmentsChromaffin_cells.Chrom_SOX2_statusHigh.NEFL	3.67697451	3.05219196	4.73643271	1.03E-05	7.76E-05	3.01746464	NEFL	assignmentsChromaffin_cells.Chrom_SOX2_statusHigh
assignmentsChromaffin_cells.Chrom_SOX2_statusHigh.DAXX	1.17476458	3.35135482	4.71062293	1.13E-05	8.41E-05	3.01703627	DAXX	assignmentsChromaffin_cells.Chrom_SOX2_statusHigh
assignmentsChromaffin_cells.Chrom_SOX2_statusHigh.COPS6	1.2743663	5.1795312	4.74367744	9.97E-06	7.57E-05	3.01583596	COPS6	assignmentsChromaffin_cells.Chrom_SOX2_statusHigh
assignmentsChromaffin_cells.Chrom_SOX2_statusHigh.BCL7B	1.29009097	3.80819919	4.71468221	1.11E-05	8.31E-05	3.00121909	BCL7B	assignmentsChromaffin_cells.Chrom_SOX2_statusHigh
assignmentsChromaffin_cells.Chrom_SOX2_statusHigh.FAM104A	0.72430388	4.59698579	4.72515464	1.07E-05	8.02E-05	2.99619064	FAM104A	assignmentsChromaffin_cells.Chrom_SOX2_statusHigh
assignmentsChromaffin_cells.Chrom_SOX2_statusHigh.UFL1	0.90518124	5.86144972	4.73987611	1.01E-05	7.66E-05	2.99548923	UFL1	assignmentsChromaffin_cells.Chrom_SOX2_statusHigh
assignmentsChromaffin_cells.Chrom_SOX2_statusHigh.PHF11	1.61122706	4.77580603	4.69950681	1.18E-05	8.72E-05	2.9874118	PHF11	assignmentsChromaffin_cells.Chrom_SOX2_statusHigh
assignmentsChromaffin_cells.Chrom_SOX2_statusHigh.ZRANB3	-1.2524462	6.1366927	-4.7360032	1.03E-05	7.75E-05	2.98681071	ZRANB3	assignmentsChromaffin_cells.Chrom_SOX2_statusHigh
assignmentsChromaffin_cells.Chrom_SOX2_statusHigh.TPRKB	1.45921029	3.50691944	4.70640438	1.15E-05	8.52E-05	2.98474861	TPRKB	assignmentsChromaffin_cells.Chrom_SOX2_statusHigh
assignmentsChromaffin_cells.Chrom_SOX2_statusHigh.CCDC85B	2.28256116	4.5527758	4.72171045	1.09E-05	8.14E-05	2.98472286	CCDC85B	assignmentsChromaffin_cells.Chrom_SOX2_statusHigh
assignmentsChromaffin_cells.Chrom_SOX2_statusHigh.GNPTG	0.91633462	5.3361125	4.73096055	1.05E-05	7.88E-05	2.97706181	GNPTG	assignmentsChromaffin_cells.Chrom_SOX2_statusHigh
assignmentsChromaffin_cells.Chrom_SOX2_statusHigh.EIF4H	0.90069208	5.6632514	4.72991535	1.05E-05	7.90E-05	2.97691631	EIF4H	assignmentsChromaffin_cells.Chrom_SOX2_statusHigh
assignmentsChromaffin_cells.Chrom_SOX2_statusHigh.GMPPB	1.40891697	2.47440508	4.6922607	1.21E-05	8.92E-05	2.97421158	GMPPB	assignmentsChromaffin_cells.Chrom_SOX2_statusHigh
assignmentsChromaffin_cells.Chrom_SOX2_statusHigh.ZNF154	-2.3271875	2.46839098	-4.6449203	1.45E-05	0.0001036	2.95997856	ZNF154	assignmentsChromaffin_cells.Chrom_SOX2_statusHigh
assignmentsChromaffin_cells.Chrom_SOX2_statusHigh.RPS11	3.09259765	6.56086393	4.74360798	1.00E-05	7.59E-05	2.9584804	RPS11	assignmentsChromaffin_cells.Chrom_SOX2_statusHigh
assignmentsChromaffin_cells.Chrom_SOX2_statusHigh.PPP1R15A	3.03304328	5.87972703	4.72680071	1.07E-05	8.01E-05	2.95630945	PPP1R15A	assignmentsChromaffin_cells.Chrom_SOX2_statusHigh
assignmentsChromaffin_cells.Chrom_SOX2_statusHigh.EP400	-0.8251728	6.69604036	-4.7318864	1.04E-05	7.86E-05	2.95258328	EP400	assignmentsChromaffin_cells.Chrom_SOX2_statusHigh
assignmentsChromaffin_cells.Chrom_SOX2_statusHigh.SCYL2	-0.7657269	6.57380297	-4.7279028	1.06E-05	7.96E-05	2.94904946	SCYL2	assignmentsChromaffin_cells.Chrom_SOX2_statusHigh
assignmentsChromaffin_cells.Chrom_SOX2_statusHigh.NF2	-0.7877867	5.06511803	-4.7062189	1.15E-05	8.52E-05	2.94786665	NF2	assignmentsChromaffin_cells.Chrom_SOX2_statusHigh
assignmentsChromaffin_cells.Chrom_SOX2_statusHigh.PDIA4	1.6828539	4.59012491	4.71438177	1.11E-05	8.31E-05	2.94671384	PDIA4	assignmentsChromaffin_cells.Chrom_SOX2_statusHigh
assignmentsChromaffin_cells.Chrom_SOX2_statusHigh.NCKAP1	-1.1256805	7.19470545	-4.7378584	1.02E-05	7.71E-05	2.94639489	NCKAP1	assignmentsChromaffin_cells.Chrom_SOX2_statusHigh
assignmentsChromaffin_cells.Chrom_SOX2_statusHigh.TPM3	1.34605257	6.71065843	4.72586983	1.07E-05	8.01E-05	2.9398734	TPM3	assignmentsChromaffin_cells.Chrom_SOX2_statusHigh
assignmentsChromaffin_cells.Chrom_SOX2_statusHigh.STOML1	1.11571933	2.23995984	4.68157362	1.26E-05	9.23E-05	2.93541033	STOML1	assignmentsChromaffin_cells.Chrom_SOX2_statusHigh
assignmentsChromaffin_cells.Chrom_SOX2_statusHigh.INO80D	-0.8201339	6.32063803	-4.716897	1.10E-05	8.25E-05	2.93138318	INO80D	assignmentsChromaffin_cells.Chrom_SOX2_statusHigh
assignmentsChromaffin_cells.Chrom_SOX2_statusHigh.HIF1A-AS3	-3.2094415	6.35395624	-4.7094424	1.14E-05	8.46E-05	2.93105692	HIF1A-AS3	assignmentsChromaffin_cells.Chrom_SOX2_statusHigh
assignmentsChromaffin_cells.Chrom_SOX2_statusHigh.S100A13	3.17605878	2.59101417	4.65535891	1.39E-05	0.00010054	2.9301222	S100A13	assignmentsChromaffin_cells.Chrom_SOX2_statusHigh
assignmentsChromaffin_cells.Chrom_SOX2_statusHigh.GGCT	1.46781254	4.32415762	4.70987258	1.13E-05	8.43E-05	2.92882452	GGCT	assignmentsChromaffin_cells.Chrom_SOX2_statusHigh
assignmentsChromaffin_cells.Chrom_SOX2_statusHigh.BOLA1	1.63587511	2.25207268	4.67945963	1.27E-05	9.28E-05	2.92789127	BOLA1	assignmentsChromaffin_cells.Chrom_SOX2_statusHigh
assignmentsChromaffin_cells.Chrom_SOX2_statusHigh.RALGPS1	-1.6420381	5.9644191	-4.7157097	1.11E-05	8.28E-05	2.91730588	RALGPS1	assignmentsChromaffin_cells.Chrom_SOX2_statusHigh
assignmentsChromaffin_cells.Chrom_SOX2_statusHigh.RSL24D1	1.71450601	5.08960019	4.71425017	1.11E-05	8.32E-05	2.9137626	RSL24D1	assignmentsChromaffin_cells.Chrom_SOX2_statusHigh
assignmentsChromaffin_cells.Chrom_SOX2_statusHigh.UBE3C	-1.0668336	7.30946301	-4.7250769	1.07E-05	8.02E-05	2.91273815	UBE3C	assignmentsChromaffin_cells.Chrom_SOX2_statusHigh
assignmentsChromaffin_cells.Chrom_SOX2_statusHigh.C5orf24	1.2527929	4.99737355	4.70404166	1.16E-05	8.59E-05	2.90347424	C5orf24	assignmentsChromaffin_cells.Chrom_SOX2_statusHigh
assignmentsChromaffin_cells.Chrom_SOX2_statusHigh.CRNKL1	0.90273208	4.29269364	4.69346102	1.20E-05	8.89E-05	2.89746047	CRNKL1	assignmentsChromaffin_cells.Chrom_SOX2_statusHigh
assignmentsChromaffin_cells.Chrom_SOX2_statusHigh.MRTO4	1.32827391	3.1149466	4.67517396	1.29E-05	9.40E-05	2.89628644	MRTO4	assignmentsChromaffin_cells.Chrom_SOX2_statusHigh
assignmentsChromaffin_cells.Chrom_SOX2_statusHigh.DERL2	1.02301719	4.29661872	4.6913467	1.21E-05	8.95E-05	2.89542349	DERL2	assignmentsChromaffin_cells.Chrom_SOX2_statusHigh
assignmentsChromaffin_cells.Chrom_SOX2_statusHigh.NEUROD4	4.456366	2.11631362	4.68887765	1.23E-05	9.05E-05	2.89301752	NEUROD4	assignmentsChromaffin_cells.Chrom_SOX2_statusHigh
assignmentsChromaffin_cells.Chrom_SOX2_statusHigh.POLDIP2	1.16324424	3.66811821	4.68775374	1.23E-05	9.05E-05	2.8921129	POLDIP2	assignmentsChromaffin_cells.Chrom_SOX2_statusHigh
assignmentsChromaffin_cells.Chrom_SOX2_statusHigh.SGCB	1.17942579	3.99593904	4.69757647	1.19E-05	8.77E-05	2.89189155	SGCB	assignmentsChromaffin_cells.Chrom_SOX2_statusHigh
assignmentsChromaffin_cells.Chrom_SOX2_statusHigh.TOR1A	1.03307544	3.23555428	4.67478517	1.29E-05	9.41E-05	2.89177782	TOR1A	assignmentsChromaffin_cells.Chrom_SOX2_statusHigh

assignmentsChromaffin_cells.Chrom_SOX2_statusHigh.GPLD1	-1.342305	2.31443315	-4.6662558	1.33E-05	9.68E-05	2.88807911	GPLD1	assignmentsChromaffin_cells.Chrom_SOX2_statusHigh
assignmentsChromaffin_cells.Chrom_SOX2_statusHigh.NEBL-AS1	1.95673891	0.06377004	4.64400891	1.45E-05	0.00010386	2.88689358	NEBL-AS1	assignmentsChromaffin_cells.Chrom_SOX2_statusHigh
assignmentsChromaffin_cells.Chrom_SOX2_statusHigh.ANKRD24	-1.6674752	2.1625093	-4.646751	1.44E-05	0.00010303	2.87738102	ANKRD24	assignmentsChromaffin_cells.Chrom_SOX2_statusHigh
assignmentsChromaffin_cells.Chrom_SOX2_statusHigh.EIF3F	1.39499463	5.18077418	4.6957504	1.19E-05	8.82E-05	2.87589973	EIF3F	assignmentsChromaffin_cells.Chrom_SOX2_statusHigh
assignmentsChromaffin_cells.Chrom_SOX2_statusHigh.PEX5L	-2.7253317	4.57541214	-4.6896348	1.23E-05	9.03E-05	2.87343398	PEX5L	assignmentsChromaffin_cells.Chrom_SOX2_statusHigh
assignmentsChromaffin_cells.Chrom_SOX2_statusHigh.MAPK8IP3	-0.8705234	6.12682264	-4.7025465	1.16E-05	8.62E-05	2.87291903	MAPK8IP3	assignmentsChromaffin_cells.Chrom_SOX2_statusHigh
assignmentsChromaffin_cells.Chrom_SOX2_statusHigh.IGFN1	3.9331251	-0.8763373	4.63286028	1.51E-05	0.00010787	2.8677171	IGFN1	assignmentsChromaffin_cells.Chrom_SOX2_statusHigh
assignmentsChromaffin_cells.Chrom_SOX2_statusHigh.ID1	3.13150368	3.38999897	4.66732159	1.33E-05	9.68E-05	2.86724273	ID1	assignmentsChromaffin_cells.Chrom_SOX2_statusHigh
assignmentsChromaffin_cells.Chrom_SOX2_statusHigh.TSC1	-1.0849349	5.69305161	-4.6956991	1.19E-05	8.82E-05	2.86289676	TSC1	assignmentsChromaffin_cells.Chrom_SOX2_statusHigh
assignmentsChromaffin_cells.Chrom_SOX2_statusHigh.FTX	-1.2453959	10.2404856	-4.7428324	1.00E-05	7.59E-05	2.86221911	FTX	assignmentsChromaffin_cells.Chrom_SOX2_statusHigh
assignmentsChromaffin_cells.Chrom_SOX2_statusHigh.MAP4K3	-1.0545876	7.76915406	-4.7161006	1.11E-05	8.27E-05	2.86028442	MAP4K3	assignmentsChromaffin_cells.Chrom_SOX2_statusHigh
assignmentsChromaffin_cells.Chrom_SOX2_statusHigh.RAB14	0.68401587	6.21584094	4.70332364	1.16E-05	8.60E-05	2.85807116	RAB14	assignmentsChromaffin_cells.Chrom_SOX2_statusHigh
assignmentsChromaffin_cells.Chrom_SOX2_statusHigh.DKC1	1.18206495	4.31097863	4.6800542	1.27E-05	9.27E-05	2.85125386	DKC1	assignmentsChromaffin_cells.Chrom_SOX2_statusHigh
assignmentsChromaffin_cells.Chrom_SOX2_statusHigh.SMCO4	1.42810075	4.06610504	4.67555036	1.29E-05	9.39E-05	2.85101664	SMCO4	assignmentsChromaffin_cells.Chrom_SOX2_statusHigh
assignmentsChromaffin_cells.Chrom_SOX2_statusHigh.SLC39A4	2.98023161	0.94863537	4.61956153	1.59E-05	0.00011261	2.8509245	SLC39A4	assignmentsChromaffin_cells.Chrom_SOX2_statusHigh
assignmentsChromaffin_cells.Chrom_SOX2_statusHigh.SERPINI1	1.8112254	4.17041485	4.68584014	1.24E-05	9.11E-05	2.84872719	SERPINI1	assignmentsChromaffin_cells.Chrom_SOX2_statusHigh
assignmentsChromaffin_cells.Chrom_SOX2_statusHigh.RPUSD1	1.63183229	1.69654328	4.63731385	1.49E-05	0.00010635	2.84350931	RPUSD1	assignmentsChromaffin_cells.Chrom_SOX2_statusHigh
assignmentsChromaffin_cells.Chrom_SOX2_statusHigh.LSM2	1.80948854	3.82732552	4.67271196	1.30E-05	9.47E-05	2.83844601	LSM2	assignmentsChromaffin_cells.Chrom_SOX2_statusHigh
assignmentsChromaffin_cells.Chrom_SOX2_statusHigh.ZNF618	-1.9836673	5.66281248	-4.6692279	1.32E-05	9.60E-05	2.83786477	ZNF618	assignmentsChromaffin_cells.Chrom_SOX2_statusHigh
assignmentsChromaffin_cells.Chrom_SOX2_statusHigh.BTG1	2.25821749	7.22190116	4.70766519	1.15E-05	8.51E-05	2.83098098	BTG1	assignmentsChromaffin_cells.Chrom_SOX2_statusHigh
assignmentsChromaffin_cells.Chrom_SOX2_statusHigh.GPKOW	0.91320824	2.90527029	4.65577418	1.39E-05	0.00010018	2.82929593	GPKOW	assignmentsChromaffin_cells.Chrom_SOX2_statusHigh
assignmentsChromaffin_cells.Chrom_SOX2_statusHigh.DNAJA2	1.14360233	5.04704145	4.6838054	1.25E-05	9.17E-05	2.8275828	DNAJA2	assignmentsChromaffin_cells.Chrom_SOX2_statusHigh
assignmentsChromaffin_cells.Chrom_SOX2_statusHigh.NABP2	1.46668739	2.60486668	4.65606543	1.39E-05	0.00010012	2.82726584	NABP2	assignmentsChromaffin_cells.Chrom_SOX2_statusHigh
assignmentsChromaffin_cells.Chrom_SOX2_statusHigh.CTPS2	-1.0521157	5.48537608	-4.6773622	1.28E-05	9.34E-05	2.82290299	CTPS2	assignmentsChromaffin_cells.Chrom_SOX2_statusHigh
assignmentsChromaffin_cells.Chrom_SOX2_statusHigh.HTRA2	1.51250068	3.07191713	4.65746147	1.38E-05	9.97E-05	2.82091877	HTRA2	assignmentsChromaffin_cells.Chrom_SOX2_statusHigh
assignmentsChromaffin_cells.Chrom_SOX2_statusHigh.CCDC47	1.10198465	6.0322655	4.69490494	1.20E-05	8.84E-05	2.81971879	CCDC47	assignmentsChromaffin_cells.Chrom_SOX2_statusHigh
assignmentsChromaffin_cells.Chrom_SOX2_statusHigh.XPO7	-0.8691412	6.24174654	-4.6869599	1.23E-05	9.07E-05	2.81853094	XPO7	assignmentsChromaffin_cells.Chrom_SOX2_statusHigh
assignmentsChromaffin_cells.Chrom_SOX2_statusHigh.TEX30	1.18479206	2.65801426	4.65290428	1.40E-05	0.00010095	2.81790859	TEX30	assignmentsChromaffin_cells.Chrom_SOX2_statusHigh
assignmentsChromaffin_cells.Chrom_SOX2_statusHigh.CENPV	2.2284221	2.81531411	4.66138168	1.36E-05	9.85E-05	2.81771717	CENPV	assignmentsChromaffin_cells.Chrom_SOX2_statusHigh
assignmentsChromaffin_cells.Chrom_SOX2_statusHigh.INF2	2.78997666	3.1542646	4.61872227	1.60E-05	0.00011289	2.81723119	INF2	assignmentsChromaffin_cells.Chrom_SOX2_statusHigh
assignmentsChromaffin_cells.Chrom_SOX2_statusHigh.EPB41L5	-1.1646895	5.61309472	-4.6825053	1.26E-05	9.20E-05	2.81544211	EPB41L5	assignmentsChromaffin_cells.Chrom_SOX2_statusHigh
assignmentsChromaffin_cells.Chrom_SOX2_statusHigh.DMAC2	0.95078	3.14476565	4.65614172	1.39E-05	0.00010012	2.81535293	DMAC2	assignmentsChromaffin_cells.Chrom_SOX2_statusHigh
assignmentsChromaffin_cells.Chrom_SOX2_statusHigh.CLASP2	-1.1006848	8.46082907	-4.7118377	1.12E-05	8.38E-05	2.80861382	CLASP2	assignmentsChromaffin_cells.Chrom_SOX2_statusHigh
assignmentsChromaffin_cells.Chrom_SOX2_statusHigh.CEBPB	2.70880795	4.95331776	4.67536845	1.29E-05	9.42E-05	2.8068227	CEBPB	assignmentsChromaffin_cells.Chrom_SOX2_statusHigh
assignmentsChromaffin_cells.Chrom_SOX2_statusHigh.CCNB1	1.639197	1.66402537	4.63673931	1.49E-05	0.00010651	2.80590202	CCNB1	assignmentsChromaffin_cells.Chrom_SOX2_statusHigh
assignmentsChromaffin_cells.Chrom_SOX2_statusHigh.VPS13A	-1.3057645	7.44311443	-4.6987735	1.18E-05	8.74E-05	2.80524938	VPS13A	assignmentsChromaffin_cells.Chrom_SOX2_statusHigh
assignmentsChromaffin_cells.Chrom_SOX2_statusHigh.MBOAT2	-1.4508378	5.71505139	-4.6810764	1.26E-05	9.24E-05	2.80344883	MBOAT2	assignmentsChromaffin_cells.Chrom_SOX2_statusHigh
assignmentsChromaffin_cells.Chrom_SOX2_statusHigh.PSMB8	1.94877551	3.68845608	4.6464692	1.44E-05	0.00010309	2.80313812	PSMB8	assignmentsChromaffin_cells.Chrom_SOX2_statusHigh
assignmentsChromaffin_cells.Chrom_SOX2_statusHigh.C11orf80	-1.5345242	5.74072824	-4.6752812	1.29E-05	9.40E-05	2.7925499	C11orf80	assignmentsChromaffin_cells.Chrom_SOX2_statusHigh
assignmentsChromaffin_cells.Chrom_SOX2_statusHigh.SSB	1.3499435	5.74954913	4.68562059	1.24E-05	9.11E-05	2.79252114	SSB	assignmentsChromaffin_cells.Chrom_SOX2_statusHigh
assignmentsChromaffin_cells.Chrom_SOX2_statusHigh.CWF19L1	-0.7071337	5.02909391	-4.6654325	1.34E-05	9.70E-05	2.79060316	CWF19L1	assignmentsChromaffin_cells.Chrom_SOX2_statusHigh
assignmentsChromaffin_cells.Chrom_SOX2_statusHigh.ESD	0.91438751	5.56967252	4.68287565	1.25E-05	9.19E-05	2.78759153	ESD	assignmentsChromaffin_cells.Chrom_SOX2_statusHigh
assignmentsChromaffin_cells.Chrom_SOX2_statusHigh.STX18-AS1	-1.6800302	5.52446712	-4.6711821	1.31E-05	9.52E-05	2.78612706	STX18-AS1	assignmentsChromaffin_cells.Chrom_SOX2_statusHigh
assignmentsChromaffin_cells.Chrom_SOX2_statusHigh.SYCE2	-2.1078491	2.49439704	-4.6256804	1.55E-05	0.0001104	2.78247741	SYCE2	assignmentsChromaffin_cells.Chrom_SOX2_statusHigh
assignmentsChromaffin_cells.Chrom_SOX2_statusHigh.DEXI	2.31771148	3.03447387	4.64576411	1.44E-05	0.00010347	2.7799007	DEXI	assignmentsChromaffin_cells.Chrom_SOX2_statusHigh
assignmentsChromaffin_cells.Chrom_SOX2_statusHigh.YJEFN3	-1.5159662	2.6605481	-4.6247455	1.56E-05	0.00011068	2.77442634	YJEFN3	assignmentsChromaffin_cells.Chrom_SOX2_statusHigh
assignmentsChromaffin_cells.Chrom_SOX2_statusHigh.NKAIN1	2.07483869	0.99616179	4.61968674	1.59E-05	0.0001125	2.76934287	NKAIN1	assignmentsChromaffin_cells.Chrom_SOX2_statusHigh
assignmentsChromaffin_cells.Chrom_SOX2_statusHigh.ALG2	1.24017483	4.0881112	4.66059962	1.36E-05	9.86E-05	2.76846482	ALG2	assignmentsChromaffin_cells.Chrom_SOX2_statusHigh
assignmentsChromaffin_cells.Chrom_SOX2_statusHigh.CREBBP	-0.9667757	7.27532923	-4.6790738	1.27E-05	9.29E-05	2.76660052	CREBBP	assignmentsChromaffin_cells.Chrom_SOX2_statusHigh
assignmentsChromaffin_cells.Chrom_SOX2_statusHigh.CWC15	1.2879393	4.82402116	4.6657052	1.34E-05	9.70E-05	2.76604318	CWC15	assignmentsChromaffin_cells.Chrom_SOX2_statusHigh
assignmentsChromaffin_cells.Chrom_SOX2_statusHigh.AKR1A1	1.35687759	4.84089748	4.66130809	1.36E-05	9.84E-05	2.76565085	AKR1A1	assignmentsChromaffin_cells.Chrom_SOX2_statusHigh
assignmentsChromaffin_cells.Chrom_SOX2_statusHigh.LRP10	1.48164405	4.08819313	4.64914408	1.42E-05	0.00010229	2.76547833	LRP10	assignmentsChromaffin_cells.Chrom_SOX2_statusHigh
assignmentsChromaffin_cells.Chrom_SOX2_statusHigh.SRPK2	-0.8691444	8.26877458	-4.6949163	1.20E-05	8.84E-05	2.76514715	SRPK2	assignmentsChromaffin_cells.Chrom_SOX2_statusHigh
assignmentsChromaffin_cells.Chrom_SOX2_statusHigh.ZDHHC21	-1.0584436	6.72156539	-4.6801343	1.27E-05	9.27E-05	2.76500494	ZDHHC21	assignmentsChromaffin_cells.Chrom_SOX2_statusHigh
assignmentsChromaffin_cells.Chrom_SOX2_statusHigh.RFESD	1.72549795	1.27512758	4.60662421	1.67E-05	0.0001174	2.7641173	RFESD	assignmentsChromaffin_cells.Chrom_SOX2_statusHigh
assignmentsChromaffin_cells.Chrom_SOX2_statusHigh.STEAP3-AS1	-2.0061641	1.12546366	-4.5940594	1.75E-05	0.00012255	2.76350474	STEAP3-AS1	assignmentsChromaffin_cells.Chrom_SOX2_statusHigh
assignmentsChromaffin_cells.Chrom_SOX2_statusHigh.PSPC1	-1.0233535	7.422514	-4.6835139	1.25E-05	9.18E-05	2.75944422	PSPC1	assignmentsChromaffin_cells.Chrom_SOX2_statusHigh
assignmentsChromaffin_cells.Chrom_SOX2_statusHigh.PPP1R16A	1.31494461	3.34212068	4.64813331	1.43E-05	0.00010259	2.75501362	PPP1R16A	assignmentsChromaffin_cells.Chrom_SOX2_statusHigh
assignmentsChromaffin_cells.Chrom_SOX2_statusHigh.ZNF440	-2.4577496	3.60886803	-4.6016429	1.70E-05	0.00011945	2.75490412	ZNF440	assignmentsChromaffin_cells.Chrom_SOX2_statusHigh
assignmentsChromaffin_cells.Chrom_SOX2_statusHigh.TGFB2	2.29567971	3.69304481	4.6112923	1.64E-05	0.00011571	2.75456995	TGFB2	assignmentsChromaffin_cells.Chrom_SOX2_statusHigh
assignmentsChromaffin_cells.Chrom_SOX2_statusHigh.UBAP2	-1.1105175	6.61602882	-4.6687547	1.32E-05	9.60E-05	2.75255834	UBAP2	assignmentsChromaffin_cells.Chrom_SOX2_statusHigh
assignmentsChromaffin_cells.Chrom_SOX2_statusHigh.C1orf216	1.22232293	2.49244227	4.61842826	1.60E-05	0.00011289	2.75247044	C1orf216	assignmentsChromaffin_cells.Chrom_SOX2_statusHigh
assignmentsChromaffin_cells.Chrom_SOX2_statusHigh.DNTTIP2	1.76992846	4.6033968	4.65358937	1.40E-05	0.00010079	2.7510795	DNTTIP2	assignmentsChromaffin_cells.Chrom_SOX2_statusHigh
assignmentsChromaffin_cells.Chrom_SOX2_statusHigh.PLEKHF1	2.57468288	0.5344506	4.58388197	1.82E-05	0.00012656	2.74866276	PLEKHF1	assignmentsChromaffin_cells.Chrom_SOX2_statusHigh

assignmentsChromaffin_cells.Chrom_SOX2_statusHigh.PACS2	-1.0539029	5.54673368	-4.6552579	1.39E-05	0.00010033	2.74716221	PACS2	assignmentsChromaffin_cells.Chrom_SOX2_statusHigh
assignmentsChromaffin_cells.Chrom_SOX2_statusHigh.SPIRE2	-1.3597902	3.33674708	-4.6328109	1.51E-05	0.00010787	2.74505166	SPIRE2	assignmentsChromaffin_cells.Chrom_SOX2_statusHigh
assignmentsChromaffin_cells.Chrom_SOX2_statusHigh.KLHL11	1.10918832	2.71241941	4.63500965	1.50E-05	0.00010713	2.74305843	KLHL11	assignmentsChromaffin_cells.Chrom_SOX2_statusHigh
assignmentsChromaffin_cells.Chrom_SOX2_statusHigh.NF1	-0.8136151	8.55482033	-4.6919066	1.21E-05	8.93E-05	2.73931658	NF1	assignmentsChromaffin_cells.Chrom_SOX2_statusHigh
assignmentsChromaffin_cells.Chrom_SOX2_statusHigh.MRPL23	2.10103254	2.85947823	4.64057383	1.47E-05	0.00010529	2.73696064	MRPL23	assignmentsChromaffin_cells.Chrom_SOX2_statusHigh
assignmentsChromaffin_cells.Chrom_SOX2_statusHigh.CALHM2	1.62294738	2.41507955	4.61124118	1.64E-05	0.00011571	2.73451805	CALHM2	assignmentsChromaffin_cells.Chrom_SOX2_statusHigh
assignmentsChromaffin_cells.Chrom_SOX2_statusHigh.ATXN10	-0.7060842	7.50057451	-4.6777379	1.28E-05	9.33E-05	2.72914916	ATXN10	assignmentsChromaffin_cells.Chrom_SOX2_statusHigh
assignmentsChromaffin_cells.Chrom_SOX2_statusHigh.RGS16	4.73776838	2.98077277	4.58851049	1.79E-05	0.00012519	2.7285502	RGS16	assignmentsChromaffin_cells.Chrom_SOX2_statusHigh
assignmentsChromaffin_cells.Chrom_SOX2_statusHigh.PWWP2B	1.21280429	2.76932256	4.62638648	1.55E-05	0.00011015	2.72488296	PWWP2B	assignmentsChromaffin_cells.Chrom_SOX2_statusHigh
assignmentsChromaffin_cells.Chrom_SOX2_statusHigh.EIF5A2	1.14459835	2.57075537	4.62729677	1.54E-05	0.00010988	2.72477597	EIF5A2	assignmentsChromaffin_cells.Chrom_SOX2_statusHigh
assignmentsChromaffin_cells.Chrom_SOX2_statusHigh.ZNF266	-0.8424536	5.31787297	-4.6481698	1.43E-05	0.00010259	2.72261405	ZNF266	assignmentsChromaffin_cells.Chrom_SOX2_statusHigh
assignmentsChromaffin_cells.Chrom_SOX2_statusHigh.TSR3	1.65233545	3.01568125	4.62935738	1.53E-05	0.00010923	2.71950163	TSR3	assignmentsChromaffin_cells.Chrom_SOX2_statusHigh
assignmentsChromaffin_cells.Chrom_SOX2_statusHigh.MPC1	1.23435977	5.33132705	4.65421163	1.40E-05	0.00010064	2.71252973	MPC1	assignmentsChromaffin_cells.Chrom_SOX2_statusHigh
assignmentsChromaffin_cells.Chrom_SOX2_statusHigh.FGF12-AS1	-3.2298934	0.74338276	-4.571959	1.90E-05	0.00013116	2.7104389	FGF12-AS1	assignmentsChromaffin_cells.Chrom_SOX2_statusHigh
assignmentsChromaffin_cells.Chrom_SOX2_statusHigh.RIN2	-2.6367105	6.44536125	-4.6283358	1.54E-05	0.00010988	2.70581256	RIN2	assignmentsChromaffin_cells.Chrom_SOX2_statusHigh
assignmentsChromaffin_cells.Chrom_SOX2_statusHigh.RPS24	2.75451673	7.68108071	4.6832737	1.26E-05	9.20E-05	2.69875258	RPS24	assignmentsChromaffin_cells.Chrom_SOX2_statusHigh
assignmentsChromaffin_cells.Chrom_SOX2_statusHigh.RHEBL1	2.10366834	1.08786051	4.57671788	1.86E-05	0.00012931	2.69728448	RHEBL1	assignmentsChromaffin_cells.Chrom_SOX2_statusHigh
assignmentsChromaffin_cells.Chrom_SOX2_statusHigh.BST2	3.07608118	3.68875921	4.58793124	1.79E-05	0.00012529	2.69578837	BST2	assignmentsChromaffin_cells.Chrom_SOX2_statusHigh
assignmentsChromaffin_cells.Chrom_SOX2_statusHigh.SMIM19	1.38578008	3.93820926	4.6340364	1.51E-05	0.00010747	2.69535437	SMIM19	assignmentsChromaffin_cells.Chrom_SOX2_statusHigh
assignmentsChromaffin_cells.Chrom_SOX2_statusHigh.RPL34	3.21907098	7.3216515	4.68029642	1.27E-05	9.28E-05	2.69386404	RPL34	assignmentsChromaffin_cells.Chrom_SOX2_statusHigh
assignmentsChromaffin_cells.Chrom_SOX2_statusHigh.LINC00847	2.14997074	2.13851199	4.58489736	1.81E-05	0.00012619	2.6862052	LINC00847	assignmentsChromaffin_cells.Chrom_SOX2_statusHigh
assignmentsChromaffin_cells.Chrom_SOX2_statusHigh.DEGS1	1.15876393	5.27508432	4.64767301	1.43E-05	0.00010272	2.67516432	DEGS1	assignmentsChromaffin_cells.Chrom_SOX2_statusHigh
assignmentsChromaffin_cells.Chrom_SOX2_statusHigh.RPLP2	2.88618086	6.91640843	4.66294304	1.36E-05	9.82E-05	2.67006314	RPLP2	assignmentsChromaffin_cells.Chrom_SOX2_statusHigh
assignmentsChromaffin_cells.Chrom_SOX2_statusHigh.RILP	1.82901152	1.13455203	4.56897083	1.92E-05	0.0001324	2.66747999	RILP	assignmentsChromaffin_cells.Chrom_SOX2_statusHigh
assignmentsChromaffin_cells.Chrom_SOX2_statusHigh.SCAI	-1.3348248	5.67293545	-4.6366952	1.49E-05	0.00010651	2.66731211	SCAI	assignmentsChromaffin_cells.Chrom_SOX2_statusHigh
assignmentsChromaffin_cells.Chrom_SOX2_statusHigh.LRRTM3	-3.425509	2.51965885	-4.5833197	1.83E-05	0.00012719	2.66527046	LRRTM3	assignmentsChromaffin_cells.Chrom_SOX2_statusHigh
assignmentsChromaffin_cells.Chrom_SOX2_statusHigh.SSRP1	1.24940434	4.19412399	4.62483594	1.56E-05	0.00011068	2.6541456	SSRP1	assignmentsChromaffin_cells.Chrom_SOX2_statusHigh
assignmentsChromaffin_cells.Chrom_SOX2_statusHigh.COX7A2L	1.42505037	5.67203298	4.65120444	1.41E-05	0.00010155	2.65343604	COX7A2L	assignmentsChromaffin_cells.Chrom_SOX2_statusHigh
assignmentsChromaffin_cells.Chrom_SOX2_statusHigh.CTXN1	2.02716834	0.3791845	4.56830303	1.92E-05	0.00013262	2.64909799	CTXN1	assignmentsChromaffin_cells.Chrom_SOX2_statusHigh
assignmentsChromaffin_cells.Chrom_SOX2_statusHigh.BABAM1	1.0735303	4.61958793	4.6288232	1.54E-05	0.0001094	2.6452834	BABAM1	assignmentsChromaffin_cells.Chrom_SOX2_statusHigh
assignmentsChromaffin_cells.Chrom_SOX2_statusHigh.LRP6	-1.5468317	6.46790798	-4.6444608	1.45E-05	0.00010373	2.64358903	LRP6	assignmentsChromaffin_cells.Chrom_SOX2_statusHigh
assignmentsChromaffin_cells.Chrom_SOX2_statusHigh.DYNLL2	1.43157021	4.43110006	4.62199849	1.58E-05	0.00011173	2.61842434	DYNLL2	assignmentsChromaffin_cells.Chrom_SOX2_statusHigh
assignmentsChromaffin_cells.Chrom_SOX2_statusHigh.RPL3	3.01886981	7.58947171	4.66121553	1.36E-05	9.87E-05	2.6169607	RPL3	assignmentsChromaffin_cells.Chrom_SOX2_statusHigh
assignmentsChromaffin_cells.Chrom_SOX2_statusHigh.RPL13	2.7092831	8.32186779	4.66731194	1.33E-05	9.68E-05	2.61659882	RPL13	assignmentsChromaffin_cells.Chrom_SOX2_statusHigh
assignmentsChromaffin_cells.Chrom_SOX2_statusHigh.CPTP	1.64468875	1.17642684	4.55636661	2.01E-05	0.0001378	2.61592409	CPTP	assignmentsChromaffin_cells.Chrom_SOX2_statusHigh
assignmentsChromaffin_cells.Chrom_SOX2_statusHigh.ZNF142	-0.7640193	3.97086031	-4.6001809	1.71E-05	0.00011999	2.61178064	ZNF142	assignmentsChromaffin_cells.Chrom_SOX2_statusHigh
assignmentsChromaffin_cells.Chrom_SOX2_statusHigh.HEXIM2	1.68864908	1.5944656	4.58153975	1.83E-05	0.0001275	2.60797024	HEXIM2	assignmentsChromaffin_cells.Chrom_SOX2_statusHigh
assignmentsChromaffin_cells.Chrom_SOX2_statusHigh.FRMPD1	-1.9614845	1.28131427	-4.5599915	1.98E-05	0.00013618	2.60459709	FRMPD1	assignmentsChromaffin_cells.Chrom_SOX2_statusHigh
assignmentsChromaffin_cells.Chrom_SOX2_statusHigh.MAGOH	1.27977184	4.45706517	4.6091375	1.65E-05	0.00011648	2.6017235	MAGOH	assignmentsChromaffin_cells.Chrom_SOX2_statusHigh
assignmentsChromaffin_cells.Chrom_SOX2_statusHigh.RAB11B	1.43041781	5.14400005	4.6264851	1.55E-05	0.00011015	2.60164891	RAB11B	assignmentsChromaffin_cells.Chrom_SOX2_statusHigh
assignmentsChromaffin_cells.Chrom_SOX2_statusHigh.EML5	-2.7013975	7.82667073	-4.6538455	1.40E-05	0.00010095	2.59801873	EML5	assignmentsChromaffin_cells.Chrom_SOX2_statusHigh
assignmentsChromaffin_cells.Chrom_SOX2_statusHigh.B3GALNT1	1.36271686	2.74424857	4.58542465	1.81E-05	0.000126	2.58948786	B3GALNT1	assignmentsChromaffin_cells.Chrom_SOX2_statusHigh
assignmentsChromaffin_cells.Chrom_SOX2_statusHigh.ZFP36L2	2.61002773	6.5853634	4.61550226	1.62E-05	0.0001144	2.58819214	ZFP36L2	assignmentsChromaffin_cells.Chrom_SOX2_statusHigh
assignmentsChromaffin_cells.Chrom_SOX2_statusHigh.LRBA	-1.2660135	8.80757095	-4.6538501	1.40E-05	0.00010073	2.5862119	LRBA	assignmentsChromaffin_cells.Chrom_SOX2_statusHigh
assignmentsChromaffin_cells.Chrom_SOX2_statusHigh.TRIM69	1.19634658	4.06232536	4.60424196	1.68E-05	0.00011839	2.58558922	TRIM69	assignmentsChromaffin_cells.Chrom_SOX2_statusHigh
assignmentsChromaffin_cells.Chrom_SOX2_statusHigh.COPZ2	2.39667535	2.27305469	4.57768452	1.86E-05	0.00012908	2.5824772	COPZ2	assignmentsChromaffin_cells.Chrom_SOX2_statusHigh
assignmentsChromaffin_cells.Chrom_SOX2_statusHigh.TNFSF13	2.51065295	1.62850537	4.53751549	2.16E-05	0.0001464	2.58180003	TNFSF13	assignmentsChromaffin_cells.Chrom_SOX2_statusHigh
assignmentsChromaffin_cells.Chrom_SOX2_statusHigh.VPS29	0.87225083	5.91835107	4.62426152	1.56E-05	0.00011084	2.57607596	VPS29	assignmentsChromaffin_cells.Chrom_SOX2_statusHigh
assignmentsChromaffin_cells.Chrom_SOX2_statusHigh.EGLN2	1.17579096	3.93607551	4.59607737	1.74E-05	0.00012173	2.57442141	EGLN2	assignmentsChromaffin_cells.Chrom_SOX2_statusHigh
assignmentsChromaffin_cells.Chrom_SOX2_statusHigh.RNASET2	1.91507632	5.10150629	4.60927092	1.65E-05	0.00011649	2.56754213	RNASET2	assignmentsChromaffin_cells.Chrom_SOX2_statusHigh
assignmentsChromaffin_cells.Chrom_SOX2_statusHigh.PCBP3	-1.7519656	7.19309448	-4.6385836	1.48E-05	0.00010605	2.56472896	PCBP3	assignmentsChromaffin_cells.Chrom_SOX2_statusHigh
assignmentsChromaffin_cells.Chrom_SOX2_statusHigh.GOLGA8A	-1.7133725	5.83088813	-4.6087024	1.66E-05	0.00011661	2.56188856	GOLGA8A	assignmentsChromaffin_cells.Chrom_SOX2_statusHigh
assignmentsChromaffin_cells.Chrom_SOX2_statusHigh.NVL	-0.9546865	5.76725401	-4.6115762	1.64E-05	0.00011565	2.55790138	NVL	assignmentsChromaffin_cells.Chrom_SOX2_statusHigh
assignmentsChromaffin_cells.Chrom_SOX2_statusHigh.SMARCE1	0.87985605	5.85627528	4.62037495	1.58E-05	0.00011236	2.55434677	SMARCE1	assignmentsChromaffin_cells.Chrom_SOX2_statusHigh
assignmentsChromaffin_cells.Chrom_SOX2_statusHigh.TSSC4	1.44694372	3.10956142	4.57993881	1.84E-05	0.00012802	2.55389179	TSSC4	assignmentsChromaffin_cells.Chrom_SOX2_statusHigh
assignmentsChromaffin_cells.Chrom_SOX2_statusHigh.STAG2	-0.6909203	7.56034171	-4.627398	1.54E-05	0.00010988	2.55387003	STAG2	assignmentsChromaffin_cells.Chrom_SOX2_statusHigh
assignmentsChromaffin_cells.Chrom_SOX2_statusHigh.MRM2	1.19690394	3.05939356	4.57987623	1.84E-05	0.00012802	2.55236764	MRM2	assignmentsChromaffin_cells.Chrom_SOX2_statusHigh
assignmentsChromaffin_cells.Chrom_SOX2_statusHigh.SLC35D3	3.63423701	2.53206257	4.59340883	1.76E-05	0.0001232	2.55024132	SLC35D3	assignmentsChromaffin_cells.Chrom_SOX2_statusHigh
assignmentsChromaffin_cells.Chrom_SOX2_statusHigh.MT1E	4.3656868	1.49598303	4.52480745	2.27E-05	0.00015258	2.54471806	MT1E	assignmentsChromaffin_cells.Chrom_SOX2_statusHigh
assignmentsChromaffin_cells.Chrom_SOX2_statusHigh.EXD3	-1.3242585	5.09318583	-4.5890966	1.78E-05	0.00012462	2.54336717	EXD3	assignmentsChromaffin_cells.Chrom_SOX2_statusHigh
assignmentsChromaffin_cells.Chrom_SOX2_statusHigh.DNAJB11	1.43453278	4.81525134	4.60098028	1.70E-05	0.00011969	2.54152308	DNAJB11	assignmentsChromaffin_cells.Chrom_SOX2_statusHigh
assignmentsChromaffin_cells.Chrom_SOX2_statusHigh.RNF215	-0.9854428	3.99274443	-4.5743614	1.88E-05	0.00013028	2.53483261	RNF215	assignmentsChromaffin_cells.Chrom_SOX2_statusHigh
assignmentsChromaffin_cells.Chrom_SOX2_statusHigh.IRF2BPL	1.38606445	4.02827026	4.59109891	1.77E-05	0.0001238	2.5246661	IRF2BPL	assignmentsChromaffin_cells.Chrom_SOX2_statusHigh
assignmentsChromaffin_cells.Chrom_SOX2_statusHigh.PDCL	0.93144797	4.00670852	4.58062379	1.84E-05	0.00012777	2.51940377	PDCL	assignmentsChromaffin_cells.Chrom_SOX2_statusHigh

assignmentsChromaffin_cells.Chrom_SOX2_statusHigh.POU6F1	-1.1515467	4.06335399	-4.5737698	1.89E-05	0.0001304	2.51916728	POU6F1	assignmentsChromaffin_cells.Chrom_SOX2_statusHigh
assignmentsChromaffin_cells.Chrom_SOX2_statusHigh.SEMA3G	3.11192644	-0.1008629	4.52635387	2.25E-05	0.00015162	2.51831776	SEMA3G	assignmentsChromaffin_cells.Chrom_SOX2_statusHigh
assignmentsChromaffin_cells.Chrom_SOX2_statusHigh.ATP6V1G2	1.4620125	1.87586131	4.55989938	1.99E-05	0.00013618	2.51817002	ATP6V1G2	assignmentsChromaffin_cells.Chrom_SOX2_statusHigh
assignmentsChromaffin_cells.Chrom_SOX2_statusHigh.PIK3C2A	-1.001479	7.26619864	-4.6090836	1.65E-05	0.00011648	2.51253146	PIK3C2A	assignmentsChromaffin_cells.Chrom_SOX2_statusHigh
assignmentsChromaffin_cells.Chrom_SOX2_statusHigh.NR1D1	1.77673908	2.11675895	4.56269116	1.96E-05	0.00013503	2.50852868	NR1D1	assignmentsChromaffin_cells.Chrom_SOX2_statusHigh
assignmentsChromaffin_cells.Chrom_SOX2_statusHigh.QDPR	1.53067685	5.76583001	4.61713909	1.60E-05	0.00011344	2.50515579	QDPR	assignmentsChromaffin_cells.Chrom_SOX2_statusHigh
assignmentsChromaffin_cells.Chrom_SOX2_statusHigh.KCNK15	4.65777234	-0.3463752	4.51144231	2.38E-05	0.00015911	2.50401114	KCNK15	assignmentsChromaffin_cells.Chrom_SOX2_statusHigh
assignmentsChromaffin_cells.Chrom_SOX2_statusHigh.MFAP3L	-1.578867	3.13293881	-4.5586472	1.99E-05	0.0001367	2.50170729	MFAP3L	assignmentsChromaffin_cells.Chrom_SOX2_statusHigh
assignmentsChromaffin_cells.Chrom_SOX2_statusHigh.MYO9A	-0.8561995	7.90730938	-4.619835	1.59E-05	0.0001125	2.49911474	MYO9A	assignmentsChromaffin_cells.Chrom_SOX2_statusHigh
assignmentsChromaffin_cells.Chrom_SOX2_statusHigh.WDFY3	-1.0604485	8.12425312	-4.6196931	1.59E-05	0.0001125	2.49906889	WDFY3	assignmentsChromaffin_cells.Chrom_SOX2_statusHigh
assignmentsChromaffin_cells.Chrom_SOX2_statusHigh.MRPL32	1.17735703	4.366317	4.58439647	1.81E-05	0.00012638	2.49518203	MRPL32	assignmentsChromaffin_cells.Chrom_SOX2_statusHigh
assignmentsChromaffin_cells.Chrom_SOX2_statusHigh.CACNB4	-2.2106916	5.15079083	-4.5818527	1.83E-05	0.00012761	2.49267304	CACNB4	assignmentsChromaffin_cells.Chrom_SOX2_statusHigh
assignmentsChromaffin_cells.Chrom_SOX2_statusHigh.PDCD6	0.94340448	5.93154398	4.60290411	1.69E-05	0.00011893	2.49043919	PDCD6	assignmentsChromaffin_cells.Chrom_SOX2_statusHigh
assignmentsChromaffin_cells.Chrom_SOX2_statusHigh.PLEKHB1	2.13845261	-0.0145109	4.50834237	2.40E-05	0.00016014	2.48868724	PLEKHB1	assignmentsChromaffin_cells.Chrom_SOX2_statusHigh
assignmentsChromaffin_cells.Chrom_SOX2_statusHigh.CNTNAP4	-4.6228075	3.95733749	-4.5480021	2.08E-05	0.00014193	2.48419775	CNTNAP4	assignmentsChromaffin_cells.Chrom_SOX2_statusHigh
assignmentsChromaffin_cells.Chrom_SOX2_statusHigh.LINC01376	-1.3453552	5.34196926	-4.5809961	1.84E-05	0.00012765	2.47752906	LINC01376	assignmentsChromaffin_cells.Chrom_SOX2_statusHigh
assignmentsChromaffin_cells.Chrom_SOX2_statusHigh.BEX1	2.60024833	5.17799036	4.61190247	1.64E-05	0.00011575	2.47467365	BEX1	assignmentsChromaffin_cells.Chrom_SOX2_statusHigh
assignmentsChromaffin_cells.Chrom_SOX2_statusHigh.UBXN8	1.28093034	2.90748293	4.55365132	2.03E-05	0.00013896	2.47211929	UBXN8	assignmentsChromaffin_cells.Chrom_SOX2_statusHigh
assignmentsChromaffin_cells.Chrom_SOX2_statusHigh.CNTN4	-3.6992129	6.0720976	-4.5748418	1.88E-05	0.0001304	2.46913861	CNTN4	assignmentsChromaffin_cells.Chrom_SOX2_statusHigh
assignmentsChromaffin_cells.Chrom_SOX2_statusHigh.POLR3H	1.09209023	3.00787878	4.55263478	2.04E-05	0.00013942	2.46887066	POLR3H	assignmentsChromaffin_cells.Chrom_SOX2_statusHigh
assignmentsChromaffin_cells.Chrom_SOX2_statusHigh.ACSS1	2.65048973	2.90757083	4.5004357	2.47E-05	0.00016412	2.4680261	ACSS1	assignmentsChromaffin_cells.Chrom_SOX2_statusHigh
assignmentsChromaffin_cells.Chrom_SOX2_statusHigh.LINC01035	4.20119979	0.43068292	4.5314426	2.21E-05	0.00014957	2.46797552	LINC01035	assignmentsChromaffin_cells.Chrom_SOX2_statusHigh
assignmentsChromaffin_cells.Chrom_SOX2_statusHigh.ARV1	1.02961209	3.8377957	4.57499126	1.88E-05	0.00013003	2.46712799	ARV1	assignmentsChromaffin_cells.Chrom_SOX2_statusHigh
assignmentsChromaffin_cells.Chrom_SOX2_statusHigh.PRSS36	2.45743626	-0.1587415	4.50320893	2.45E-05	0.0001627	2.46541711	PRSS36	assignmentsChromaffin_cells.Chrom_SOX2_statusHigh
assignmentsChromaffin_cells.Chrom_SOX2_statusHigh.FARSB	-1.011321	5.86948227	-4.5871808	1.79E-05	0.00012529	2.4618471	FARSB	assignmentsChromaffin_cells.Chrom_SOX2_statusHigh
assignmentsChromaffin_cells.Chrom_SOX2_statusHigh.TBC1D1	-1.7854292	7.12956442	-4.57833	1.86E-05	0.00012882	2.45901869	TBC1D1	assignmentsChromaffin_cells.Chrom_SOX2_statusHigh
assignmentsChromaffin_cells.Chrom_SOX2_statusHigh.NOS3	2.16140872	0.64677358	4.49773885	2.50E-05	0.00016546	2.45711383	NOS3	assignmentsChromaffin_cells.Chrom_SOX2_statusHigh
assignmentsChromaffin_cells.Chrom_SOX2_statusHigh.CHM	-0.8001871	6.80671227	-4.5967132	1.73E-05	0.0001215	2.45011988	CHM	assignmentsChromaffin_cells.Chrom_SOX2_statusHigh
assignmentsChromaffin_cells.Chrom_SOX2_statusHigh.EP300	-0.887916	7.00909602	-4.5903243	1.77E-05	0.0001241	2.44976177	EP300	assignmentsChromaffin_cells.Chrom_SOX2_statusHigh
assignmentsChromaffin_cells.Chrom_SOX2_statusHigh.RPL22	2.60812274	6.25270281	4.5964061	1.74E-05	0.00012194	2.44692479	RPL22	assignmentsChromaffin_cells.Chrom_SOX2_statusHigh
assignmentsChromaffin_cells.Chrom_SOX2_statusHigh.CSRP1	1.45106971	4.03635841	4.54913738	2.07E-05	0.00014102	2.44332725	CSRP1	assignmentsChromaffin_cells.Chrom_SOX2_statusHigh
assignmentsChromaffin_cells.Chrom_SOX2_statusHigh.ITGAE	1.47654184	4.02850675	4.5560211	2.01E-05	0.00013792	2.44084833	ITGAE	assignmentsChromaffin_cells.Chrom_SOX2_statusHigh
assignmentsChromaffin_cells.Chrom_SOX2_statusHigh.ARF5	2.26104736	3.83180193	4.56563558	1.95E-05	0.00013406	2.43826545	ARF5	assignmentsChromaffin_cells.Chrom_SOX2_statusHigh
assignmentsChromaffin_cells.Chrom_SOX2_statusHigh.ANKRD44	-1.2722196	7.77476531	-4.5878976	1.79E-05	0.00012512	2.42967734	ANKRD44	assignmentsChromaffin_cells.Chrom_SOX2_statusHigh
assignmentsChromaffin_cells.Chrom_SOX2_statusHigh.SMPDL3A	2.42117048	2.92826491	4.54022113	2.14E-05	0.00014546	2.42739257	SMPDL3A	assignmentsChromaffin_cells.Chrom_SOX2_statusHigh
assignmentsChromaffin_cells.Chrom_SOX2_statusHigh.PCYT2	1.64175121	1.60790353	4.52685697	2.24E-05	0.0001514	2.42634261	PCYT2	assignmentsChromaffin_cells.Chrom_SOX2_statusHigh
assignmentsChromaffin_cells.Chrom_SOX2_statusHigh.RBM5	-0.9703539	6.9742791	-4.5822666	1.83E-05	0.00012722	2.41984048	RBM5	assignmentsChromaffin_cells.Chrom_SOX2_statusHigh
assignmentsChromaffin_cells.Chrom_SOX2_statusHigh.ZXDB	1.02227927	1.84697571	4.52741022	2.24E-05	0.00015115	2.41742013	ZXDB	assignmentsChromaffin_cells.Chrom_SOX2_statusHigh
assignmentsChromaffin_cells.Chrom_SOX2_statusHigh.EID2B	1.30865016	2.96718437	4.54911126	2.07E-05	0.00014102	2.41706005	EID2B	assignmentsChromaffin_cells.Chrom_SOX2_statusHigh
assignmentsChromaffin_cells.Chrom_SOX2_statusHigh.RNF26	2.1082821	1.52944415	4.51931842	2.31E-05	0.00015483	2.41668692	RNF26	assignmentsChromaffin_cells.Chrom_SOX2_statusHigh
assignmentsChromaffin_cells.Chrom_SOX2_statusHigh.INTS7	-1.1369162	5.35764202	-4.5655095	1.94E-05	0.00013383	2.41393987	INTS7	assignmentsChromaffin_cells.Chrom_SOX2_statusHigh
assignmentsChromaffin_cells.Chrom_SOX2_statusHigh.MINDY3	-0.7055524	6.26631538	-4.5754605	1.87E-05	0.00012986	2.41253558	MINDY3	assignmentsChromaffin_cells.Chrom_SOX2_statusHigh
assignmentsChromaffin_cells.Chrom_SOX2_statusHigh.MAFG	1.07271357	4.6344373	4.56571272	1.94E-05	0.00013378	2.40974784	MAFG	assignmentsChromaffin_cells.Chrom_SOX2_statusHigh
assignmentsChromaffin_cells.Chrom_SOX2_statusHigh.DUS2	-1.2936933	3.57377054	-4.5290012	2.23E-05	0.00015039	2.40930324	DUS2	assignmentsChromaffin_cells.Chrom_SOX2_statusHigh
assignmentsChromaffin_cells.Chrom_SOX2_statusHigh.CALY	4.41617327	1.68706258	4.52586576	2.26E-05	0.00015218	2.40927533	CALY	assignmentsChromaffin_cells.Chrom_SOX2_statusHigh
assignmentsChromaffin_cells.Chrom_SOX2_statusHigh.IREB2	-0.7924265	6.20135156	-4.5711509	1.90E-05	0.0001315	2.4071329	IREB2	assignmentsChromaffin_cells.Chrom_SOX2_statusHigh
assignmentsChromaffin_cells.Chrom_SOX2_statusHigh.CEP19	1.41630377	1.60658606	4.51487422	2.35E-05	0.00015694	2.40461127	CEP19	assignmentsChromaffin_cells.Chrom_SOX2_statusHigh
assignmentsChromaffin_cells.Chrom_SOX2_statusHigh.MYO1B	2.67846013	5.11020962	4.55238685	2.05E-05	0.00013994	2.40187742	MYO1B	assignmentsChromaffin_cells.Chrom_SOX2_statusHigh
assignmentsChromaffin_cells.Chrom_SOX2_statusHigh.CRTAP	1.29584485	4.7553084	4.55486742	2.02E-05	0.00013845	2.39852196	CRTAP	assignmentsChromaffin_cells.Chrom_SOX2_statusHigh
assignmentsChromaffin_cells.Chrom_SOX2_statusHigh.TET2	-1.1425984	7.85476887	-4.586929	1.80E-05	0.00012535	2.39686403	TET2	assignmentsChromaffin_cells.Chrom_SOX2_statusHigh
assignmentsChromaffin_cells.Chrom_SOX2_statusHigh.FBXO36	-2.9968396	3.19416383	-4.5133223	2.37E-05	0.0001582	2.39626344	FBXO36	assignmentsChromaffin_cells.Chrom_SOX2_statusHigh
assignmentsChromaffin_cells.Chrom_SOX2_statusHigh.B3GALT5	2.48104216	0.65155864	4.50725314	2.41E-05	0.00016077	2.39580566	B3GALT5	assignmentsChromaffin_cells.Chrom_SOX2_statusHigh
assignmentsChromaffin_cells.Chrom_SOX2_statusHigh.SIKE1	0.84509983	3.95221969	4.54151695	2.13E-05	0.00014468	2.39295125	SIKE1	assignmentsChromaffin_cells.Chrom_SOX2_statusHigh
assignmentsChromaffin_cells.Chrom_SOX2_statusHigh.TPPP3	3.50249979	1.22315202	4.48063752	2.67E-05	0.00017481	2.38775856	TPPP3	assignmentsChromaffin_cells.Chrom_SOX2_statusHigh
assignmentsChromaffin_cells.Chrom_SOX2_statusHigh.FAM217B	1.12216844	3.65680156	4.53938191	2.14E-05	0.00014558	2.38472369	FAM217B	assignmentsChromaffin_cells.Chrom_SOX2_statusHigh
assignmentsChromaffin_cells.Chrom_SOX2_statusHigh.PABPC5	1.18773806	1.97159769	4.52546272	2.26E-05	0.00015199	2.37593033	PABPC5	assignmentsChromaffin_cells.Chrom_SOX2_statusHigh
assignmentsChromaffin_cells.Chrom_SOX2_statusHigh.ANAPC7	-0.7687204	5.24490378	-4.5513838	2.05E-05	0.00013995	2.37552659	ANAPC7	assignmentsChromaffin_cells.Chrom_SOX2_statusHigh
assignmentsChromaffin_cells.Chrom_SOX2_statusHigh.CC2D2A	-1.5411132	5.12069025	-4.5488898	2.07E-05	0.00014107	2.37520435	CC2D2A	assignmentsChromaffin_cells.Chrom_SOX2_statusHigh
assignmentsChromaffin_cells.Chrom_SOX2_statusHigh.NSA2	1.76710491	5.15149871	4.56342742	1.96E-05	0.00013488	2.37416601	NSA2	assignmentsChromaffin_cells.Chrom_SOX2_statusHigh
assignmentsChromaffin_cells.Chrom_SOX2_statusHigh.ACTRT3	2.88675503	-0.1199535	4.47199786	2.75E-05	0.00017962	2.37320906	ACTRT3	assignmentsChromaffin_cells.Chrom_SOX2_statusHigh
assignmentsChromaffin_cells.Chrom_SOX2_statusHigh.MDM2	-0.9274649	6.48113314	-4.562639	1.97E-05	0.00013503	2.37219734	MDM2	assignmentsChromaffin_cells.Chrom_SOX2_statusHigh
assignmentsChromaffin_cells.Chrom_SOX2_statusHigh.KIAA0232	-0.8920853	7.3714077	-4.5776359	1.86E-05	0.00012898	2.37063367	KIAA0232	assignmentsChromaffin_cells.Chrom_SOX2_statusHigh
assignmentsChromaffin_cells.Chrom_SOX2_statusHigh.FAM110B	-3.7967488	4.69066366	-4.4956172	2.53E-05	0.00016693	2.36988484	FAM110B	assignmentsChromaffin_cells.Chrom_SOX2_statusHigh
assignmentsChromaffin_cells.Chrom_SOX2_statusHigh.RAPGEFL1	-1.0989546	2.4927031	-4.5120492	2.37E-05	0.00015839	2.36909148	RAPGEFL1	assignmentsChromaffin_cells.Chrom_SOX2_statusHigh

assignmentsChromaffin_cells.Chrom_SOX2_statusHigh.HECTD1	-0.8448027	7.47454026	-4.5738347	1.88E-05	0.0001304	2.36731482	HECTD1	assignmentsChromaffin_cells.Chrom_SOX2_statusHigh
assignmentsChromaffin_cells.Chrom_SOX2_statusHigh.INVS	-1.2194282	7.5200186	-4.5733052	1.89E-05	0.00013057	2.35957319	INVS	assignmentsChromaffin_cells.Chrom_SOX2_statusHigh
assignmentsChromaffin_cells.Chrom_SOX2_statusHigh.DCLRE1B	1.88038104	0.86228112	4.47106097	2.76E-05	0.00018012	2.35801799	DCLRE1B	assignmentsChromaffin_cells.Chrom_SOX2_statusHigh
assignmentsChromaffin_cells.Chrom_SOX2_statusHigh.MRPS10	1.04879934	4.22417561	4.54189803	2.12E-05	0.00014453	2.35521403	MRPS10	assignmentsChromaffin_cells.Chrom_SOX2_statusHigh
assignmentsChromaffin_cells.Chrom_SOX2_statusHigh.CFDP1	-0.654657	7.148856	-4.569247	1.92E-05	0.00013232	2.35289491	CFDP1	assignmentsChromaffin_cells.Chrom_SOX2_statusHigh
assignmentsChromaffin_cells.Chrom_SOX2_statusHigh.PCDHB3	2.14518862	1.18530276	4.49761913	2.50E-05	0.00016547	2.35254621	PCDHB3	assignmentsChromaffin_cells.Chrom_SOX2_statusHigh
assignmentsChromaffin_cells.Chrom_SOX2_statusHigh.ZNF710	-1.5571505	5.44064132	-4.5132494	2.36E-05	0.00015782	2.34862902	ZNF710	assignmentsChromaffin_cells.Chrom_SOX2_statusHigh
assignmentsChromaffin_cells.Chrom_SOX2_statusHigh.HNRNPK	1.18100939	6.90554164	4.56971331	1.91E-05	0.00013215	2.34292306	HNRNPK	assignmentsChromaffin_cells.Chrom_SOX2_statusHigh
assignmentsChromaffin_cells.Chrom_SOX2_statusHigh.ARMCX4	-1.0032237	6.49501167	-4.5599028	1.99E-05	0.00013618	2.3419295	ARMCX4	assignmentsChromaffin_cells.Chrom_SOX2_statusHigh
assignmentsChromaffin_cells.Chrom_SOX2_statusHigh.CNP	1.08983263	3.35343187	4.51913876	2.31E-05	0.00015487	2.34112341	CNP	assignmentsChromaffin_cells.Chrom_SOX2_statusHigh
assignmentsChromaffin_cells.Chrom_SOX2_statusHigh.IMPDH2	1.55870851	3.9424652	4.53489345	2.18E-05	0.00014764	2.33871476	IMPDH2	assignmentsChromaffin_cells.Chrom_SOX2_statusHigh
assignmentsChromaffin_cells.Chrom_SOX2_statusHigh.OR6A2	-4.4512621	-0.4432573	-4.4979956	2.50E-05	0.00016538	2.33633907	OR6A2	assignmentsChromaffin_cells.Chrom_SOX2_statusHigh
assignmentsChromaffin_cells.Chrom_SOX2_statusHigh.COP1	-0.7556821	8.01922365	-4.5662359	1.94E-05	0.00013358	2.33175545	COP1	assignmentsChromaffin_cells.Chrom_SOX2_statusHigh
assignmentsChromaffin_cells.Chrom_SOX2_statusHigh.G6PD	1.37020906	2.92981101	4.5098332	2.39E-05	0.00015942	2.3294103	G6PD	assignmentsChromaffin_cells.Chrom_SOX2_statusHigh
assignmentsChromaffin_cells.Chrom_SOX2_statusHigh.GABBR1	-1.1013074	5.38443231	-4.5385933	2.15E-05	0.00014594	2.32719434	GABBR1	assignmentsChromaffin_cells.Chrom_SOX2_statusHigh
assignmentsChromaffin_cells.Chrom_SOX2_statusHigh.FAM193B	-0.8816928	5.6739219	-4.5407859	2.13E-05	0.00014501	2.3250015	FAM193B	assignmentsChromaffin_cells.Chrom_SOX2_statusHigh
assignmentsChromaffin_cells.Chrom_SOX2_statusHigh.LMBR1	-0.9746213	7.40329373	-4.5621284	1.97E-05	0.00013523	2.32220564	LMBR1	assignmentsChromaffin_cells.Chrom_SOX2_statusHigh
assignmentsChromaffin_cells.Chrom_SOX2_statusHigh.AKT1	1.04648455	4.20128658	4.52477725	2.26E-05	0.00015225	2.31593993	AKT1	assignmentsChromaffin_cells.Chrom_SOX2_statusHigh
assignmentsChromaffin_cells.Chrom_SOX2_statusHigh.LZTS1	2.15617545	3.10442666	4.50353753	2.45E-05	0.0001627	2.31511619	LZTS1	assignmentsChromaffin_cells.Chrom_SOX2_statusHigh
assignmentsChromaffin_cells.Chrom_SOX2_statusHigh.MMAB	0.87198444	4.18420364	4.53654861	2.16E-05	0.00014687	2.31471538	MMAB	assignmentsChromaffin_cells.Chrom_SOX2_statusHigh
assignmentsChromaffin_cells.Chrom_SOX2_statusHigh.RNLS	3.16718339	4.73179635	4.53266109	2.20E-05	0.00014903	2.31432783	RNLS	assignmentsChromaffin_cells.Chrom_SOX2_statusHigh
assignmentsChromaffin_cells.Chrom_SOX2_statusHigh.SLC43A1	2.36811356	1.46048698	4.45770179	2.90E-05	0.00018847	2.31045158	SLC43A1	assignmentsChromaffin_cells.Chrom_SOX2_statusHigh
assignmentsChromaffin_cells.Chrom_SOX2_statusHigh.ANKMY1	-1.3122614	5.34784701	-4.5232301	2.27E-05	0.00015287	2.30988561	ANKMY1	assignmentsChromaffin_cells.Chrom_SOX2_statusHigh
assignmentsChromaffin_cells.Chrom_SOX2_statusHigh.MON2	-0.8471405	8.16132699	-4.5686508	1.92E-05	0.0001325	2.30930633	MON2	assignmentsChromaffin_cells.Chrom_SOX2_statusHigh
assignmentsChromaffin_cells.Chrom_SOX2_statusHigh.MED30	1.05038928	3.93950601	4.52341983	2.27E-05	0.00015282	2.30732041	MED30	assignmentsChromaffin_cells.Chrom_SOX2_statusHigh
assignmentsChromaffin_cells.Chrom_SOX2_statusHigh.B4GALT4	0.8677013	4.26182557	4.52413223	2.27E-05	0.00015255	2.29839706	B4GALT4	assignmentsChromaffin_cells.Chrom_SOX2_statusHigh
assignmentsChromaffin_cells.Chrom_SOX2_statusHigh.KIDINS220	-0.7221233	7.45758348	-4.5596308	1.99E-05	0.00013626	2.29740742	KIDINS220	assignmentsChromaffin_cells.Chrom_SOX2_statusHigh
assignmentsChromaffin_cells.Chrom_SOX2_statusHigh.SLC25A38	1.01379326	3.41608316	4.5160549	2.34E-05	0.00015652	2.29111327	SLC25A38	assignmentsChromaffin_cells.Chrom_SOX2_statusHigh
assignmentsChromaffin_cells.Chrom_SOX2_statusHigh.ARFGEF2	-0.8350821	6.86842392	-4.5466548	2.09E-05	0.00014213	2.28946891	ARFGEF2	assignmentsChromaffin_cells.Chrom_SOX2_statusHigh
assignmentsChromaffin_cells.Chrom_SOX2_statusHigh.STAM2	-0.9330235	5.93156619	-4.5326079	2.20E-05	0.00014865	2.28851338	STAM2	assignmentsChromaffin_cells.Chrom_SOX2_statusHigh
assignmentsChromaffin_cells.Chrom_SOX2_statusHigh.N4BP2L2-IT	-1.1675496	3.38397346	-4.4931556	2.54E-05	0.00016767	2.2871495	N4BP2L2-IT2	assignmentsChromaffin_cells.Chrom_SOX2_statusHigh
assignmentsChromaffin_cells.Chrom_SOX2_statusHigh.TMEM230	1.47914567	5.43247993	4.53777194	2.16E-05	0.00014633	2.28168357	TMEM230	assignmentsChromaffin_cells.Chrom_SOX2_statusHigh
assignmentsChromaffin_cells.Chrom_SOX2_statusHigh.HLCS	-0.9620616	6.60734866	-4.5404952	2.13E-05	0.0001451	2.27483345	HLCS	assignmentsChromaffin_cells.Chrom_SOX2_statusHigh
assignmentsChromaffin_cells.Chrom_SOX2_statusHigh.SPRYD7	0.87930843	4.25999251	4.52309335	2.28E-05	0.00015288	2.26952786	SPRYD7	assignmentsChromaffin_cells.Chrom_SOX2_statusHigh
assignmentsChromaffin_cells.Chrom_SOX2_statusHigh.FBXL5	-0.7673221	7.06999692	-4.5426417	2.12E-05	0.0001442	2.26410318	FBXL5	assignmentsChromaffin_cells.Chrom_SOX2_statusHigh
assignmentsChromaffin_cells.Chrom_SOX2_statusHigh.CHPF2	0.90880144	3.58259148	4.50872726	2.40E-05	0.00016001	2.26009001	CHPF2	assignmentsChromaffin_cells.Chrom_SOX2_statusHigh
assignmentsChromaffin_cells.Chrom_SOX2_statusHigh.XPO6	-0.8910834	6.32390287	-4.5288686	2.23E-05	0.0001504	2.25986721	XPO6	assignmentsChromaffin_cells.Chrom_SOX2_statusHigh
assignmentsChromaffin_cells.Chrom_SOX2_statusHigh.ALKBH6	1.25750083	2.88902143	4.49701987	2.51E-05	0.00016577	2.25866346	ALKBH6	assignmentsChromaffin_cells.Chrom_SOX2_statusHigh
assignmentsChromaffin_cells.Chrom_SOX2_statusHigh.RNF121	-0.7496013	5.07683698	-4.5154671	2.34E-05	0.00015669	2.25478448	RNF121	assignmentsChromaffin_cells.Chrom_SOX2_statusHigh
assignmentsChromaffin_cells.Chrom_SOX2_statusHigh.CCT2	1.32806223	5.72744153	4.53338675	2.19E-05	0.00014828	2.25471878	CCT2	assignmentsChromaffin_cells.Chrom_SOX2_statusHigh
assignmentsChromaffin_cells.Chrom_SOX2_statusHigh.MACROD2	-1.7483773	7.8054693	-4.5552337	2.02E-05	0.00013845	2.25216932	MACROD2	assignmentsChromaffin_cells.Chrom_SOX2_statusHigh
assignmentsChromaffin_cells.Chrom_SOX2_statusHigh.MAPK7	1.37930816	2.19503381	4.48009607	2.67E-05	0.00017481	2.25048618	MAPK7	assignmentsChromaffin_cells.Chrom_SOX2_statusHigh
assignmentsChromaffin_cells.Chrom_SOX2_statusHigh.ADI1	1.83480754	3.82263696	4.50761346	2.41E-05	0.00016059	2.2503316	ADI1	assignmentsChromaffin_cells.Chrom_SOX2_statusHigh
assignmentsChromaffin_cells.Chrom_SOX2_statusHigh.XBP1	1.52808607	5.93657751	4.53422095	2.18E-05	0.00014795	2.24489008	XBP1	assignmentsChromaffin_cells.Chrom_SOX2_statusHigh
assignmentsChromaffin_cells.Chrom_SOX2_statusHigh.UQCRH	2.02727268	5.66943909	4.53225604	2.21E-05	0.0001491	2.24293062	UQCRH	assignmentsChromaffin_cells.Chrom_SOX2_statusHigh
assignmentsChromaffin_cells.Chrom_SOX2_statusHigh.PCMT1	0.84224206	5.97487181	4.53394389	2.19E-05	0.00014804	2.2412304	PCMT1	assignmentsChromaffin_cells.Chrom_SOX2_statusHigh
assignmentsChromaffin_cells.Chrom_SOX2_statusHigh.PUS3	1.38132337	2.82898303	4.49359087	2.54E-05	0.00016753	2.23813438	PUS3	assignmentsChromaffin_cells.Chrom_SOX2_statusHigh
assignmentsChromaffin_cells.Chrom_SOX2_statusHigh.TSPYL1	1.693614	4.59147862	4.51752027	2.32E-05	0.00015586	2.23588888	TSPYL1	assignmentsChromaffin_cells.Chrom_SOX2_statusHigh
assignmentsChromaffin_cells.Chrom_SOX2_statusHigh.C1orf50	0.89836821	3.47354693	4.48997013	2.57E-05	0.00016936	2.23522037	C1orf50	assignmentsChromaffin_cells.Chrom_SOX2_statusHigh
assignmentsChromaffin_cells.Chrom_SOX2_statusHigh.LGALSL-DT	-1.6460378	3.01010707	-4.4730001	2.74E-05	0.00017904	2.23496029	LGALSL-DT	assignmentsChromaffin_cells.Chrom_SOX2_statusHigh
assignmentsChromaffin_cells.Chrom_SOX2_statusHigh.MCAT	1.19152296	2.08282848	4.4802861	2.66E-05	0.0001748	2.23309395	MCAT	assignmentsChromaffin_cells.Chrom_SOX2_statusHigh
assignmentsChromaffin_cells.Chrom_SOX2_statusHigh.LANCL1-AS1	-2.5971465	1.78373857	-4.4457685	3.03E-05	0.00019581	2.22419925	LANCL1-AS1	assignmentsChromaffin_cells.Chrom_SOX2_statusHigh
assignmentsChromaffin_cells.Chrom_SOX2_statusHigh.BET1L	1.25487137	3.67908018	4.48608684	2.61E-05	0.00017131	2.21938234	BET1L	assignmentsChromaffin_cells.Chrom_SOX2_statusHigh
assignmentsChromaffin_cells.Chrom_SOX2_statusHigh.SCAF1	0.96089094	4.07172621	4.50204492	2.46E-05	0.00016333	2.21729133	SCAF1	assignmentsChromaffin_cells.Chrom_SOX2_statusHigh
assignmentsChromaffin_cells.Chrom_SOX2_statusHigh.CFAP298	0.99410165	4.89342214	4.51006287	2.39E-05	0.00015936	2.21374877	CFAP298	assignmentsChromaffin_cells.Chrom_SOX2_statusHigh
assignmentsChromaffin_cells.Chrom_SOX2_statusHigh.ADCY6	-1.196403	4.16250502	-4.4866066	2.60E-05	0.00017106	2.20556137	ADCY6	assignmentsChromaffin_cells.Chrom_SOX2_statusHigh
assignmentsChromaffin_cells.Chrom_SOX2_statusHigh.CNPPD1	1.4097872	3.11565019	4.47524593	2.71E-05	0.00017764	2.20339935	CNPPD1	assignmentsChromaffin_cells.Chrom_SOX2_statusHigh
assignmentsChromaffin_cells.Chrom_SOX2_statusHigh.TAS2R31	-1.6174077	0.869354	-4.4271174	3.24E-05	0.00020772	2.20306493	TAS2R31	assignmentsChromaffin_cells.Chrom_SOX2_statusHigh
assignmentsChromaffin_cells.Chrom_SOX2_statusHigh.XAB2	1.28452171	3.27243162	4.48692742	2.60E-05	0.00017105	2.20256551	XAB2	assignmentsChromaffin_cells.Chrom_SOX2_statusHigh
assignmentsChromaffin_cells.Chrom_SOX2_statusHigh.FASTKD1	-0.8009361	5.69542671	-4.5107746	2.38E-05	0.00015907	2.19729233	FASTKD1	assignmentsChromaffin_cells.Chrom_SOX2_statusHigh
assignmentsChromaffin_cells.Chrom_SOX2_statusHigh.AREL1	-0.9792478	5.27551231	-4.4950488	2.52E-05	0.00016684	2.19715453	AREL1	assignmentsChromaffin_cells.Chrom_SOX2_statusHigh
assignmentsChromaffin_cells.Chrom_SOX2_statusHigh.CLP1	1.76660998	1.63327728	4.4479941	3.00E-05	0.0001942	2.19665451	CLP1	assignmentsChromaffin_cells.Chrom_SOX2_statusHigh
assignmentsChromaffin_cells.Chrom_SOX2_statusHigh.POP5	1.17238787	4.41449932	4.50058167	2.47E-05	0.00016412	2.18971412	POP5	assignmentsChromaffin_cells.Chrom_SOX2_statusHigh
assignmentsChromaffin_cells.Chrom_SOX2_statusHigh.SETD2	-0.8826546	7.83284618	-4.5260485	2.25E-05	0.00015173	2.18963082	SETD2	assignmentsChromaffin_cells.Chrom_SOX2_statusHigh

assignmentsChromaffin_cells.Chrom_SOX2_statusHigh.B3GAT3	1.26654189	4.30552526	4.49980119	2.48E-05	0.00016441	2.18925619	B3GAT3	assignmentsChromaffin_cells.Chrom_SOX2_statusHigh
assignmentsChromaffin_cells.Chrom_SOX2_statusHigh.CAMKMT	-1.108516	7.48787885	-4.5202097	2.30E-05	0.00015445	2.18800386	CAMKMT	assignmentsChromaffin_cells.Chrom_SOX2_statusHigh
assignmentsChromaffin_cells.Chrom_SOX2_statusHigh.CDC37L1-DT	1.2055025	1.38044239	4.45103374	2.97E-05	0.00019236	2.17924327	CDC37L1-DT	assignmentsChromaffin_cells.Chrom_SOX2_statusHigh
assignmentsChromaffin_cells.Chrom_SOX2_statusHigh.SAT2	1.60717708	5.05736853	4.50556324	2.43E-05	0.00016146	2.17721183	SAT2	assignmentsChromaffin_cells.Chrom_SOX2_statusHigh
assignmentsChromaffin_cells.Chrom_SOX2_statusHigh.NLGN4X	-2.4251619	4.89362588	-4.4928698	2.55E-05	0.00016822	2.17546112	NLGN4X	assignmentsChromaffin_cells.Chrom_SOX2_statusHigh
assignmentsChromaffin_cells.Chrom_SOX2_statusHigh.LEXM	-3.5253418	-0.3699111	-4.428028	3.23E-05	0.00020711	2.17443188	LEXM	assignmentsChromaffin_cells.Chrom_SOX2_statusHigh
assignmentsChromaffin_cells.Chrom_SOX2_statusHigh.RRP9	1.42454674	2.36040361	4.46010624	2.87E-05	0.00018696	2.17413803	RRP9	assignmentsChromaffin_cells.Chrom_SOX2_statusHigh
assignmentsChromaffin_cells.Chrom_SOX2_statusHigh.CDKN3	1.36649735	2.00652876	4.45970489	2.87E-05	0.00018716	2.17150221	CDKN3	assignmentsChromaffin_cells.Chrom_SOX2_statusHigh
assignmentsChromaffin_cells.Chrom_SOX2_statusHigh.UBN2	-1.0229221	6.77719334	-4.5154108	2.34E-05	0.00015669	2.16986471	UBN2	assignmentsChromaffin_cells.Chrom_SOX2_statusHigh
assignmentsChromaffin_cells.Chrom_SOX2_statusHigh.MBP	2.46866131	5.51989867	4.48385379	2.64E-05	0.00017319	2.16859035	MBP	assignmentsChromaffin_cells.Chrom_SOX2_statusHigh
assignmentsChromaffin_cells.Chrom_SOX2_statusHigh.TSN	1.0621731	4.46404357	4.49396771	2.53E-05	0.00016737	2.16838972	TSN	assignmentsChromaffin_cells.Chrom_SOX2_statusHigh
assignmentsChromaffin_cells.Chrom_SOX2_statusHigh.ZFYVE16	-1.0505935	7.43099707	-4.5159407	2.34E-05	0.00015652	2.16567291	ZFYVE16	assignmentsChromaffin_cells.Chrom_SOX2_statusHigh
assignmentsChromaffin_cells.Chrom_SOX2_statusHigh.RHOBTB3	2.64097732	5.22114498	4.49349315	2.55E-05	0.0001679	2.15751466	RHOBTB3	assignmentsChromaffin_cells.Chrom_SOX2_statusHigh
assignmentsChromaffin_cells.Chrom_SOX2_statusHigh.UFM1	0.93563498	5.94474229	4.5082822	2.40E-05	0.00016014	2.15442726	UFM1	assignmentsChromaffin_cells.Chrom_SOX2_statusHigh
assignmentsChromaffin_cells.Chrom_SOX2_statusHigh.SLC9A7	-1.4819629	5.80783577	-4.5056172	2.43E-05	0.00016146	2.15258974	SLC9A7	assignmentsChromaffin_cells.Chrom_SOX2_statusHigh
assignmentsChromaffin_cells.Chrom_SOX2_statusHigh.TBC1D22A	-0.9565132	7.78028374	-4.5061879	2.42E-05	0.00016118	2.14172123	TBC1D22A	assignmentsChromaffin_cells.Chrom_SOX2_statusHigh
assignmentsChromaffin_cells.Chrom_SOX2_statusHigh.ORAI2	0.94801359	4.65295163	4.48683919	2.60E-05	0.00017105	2.13811038	ORAI2	assignmentsChromaffin_cells.Chrom_SOX2_statusHigh
assignmentsChromaffin_cells.Chrom_SOX2_statusHigh.IFT57	1.19024523	5.06544792	4.49527561	2.52E-05	0.00016677	2.13776174	IFT57	assignmentsChromaffin_cells.Chrom_SOX2_statusHigh
assignmentsChromaffin_cells.Chrom_SOX2_statusHigh.RIOX1	2.45862381	0.79738798	4.4008176	3.57E-05	0.00022579	2.13214362	RIOX1	assignmentsChromaffin_cells.Chrom_SOX2_statusHigh
assignmentsChromaffin_cells.Chrom_SOX2_statusHigh.REM2	2.62745026	-0.14098	4.40276994	3.54E-05	0.00022436	2.12932916	REM2	assignmentsChromaffin_cells.Chrom_SOX2_statusHigh
assignmentsChromaffin_cells.Chrom_SOX2_statusHigh.RPL17	2.69894256	7.25076715	4.52020776	2.31E-05	0.00015483	2.12915404	RPL17	assignmentsChromaffin_cells.Chrom_SOX2_statusHigh
assignmentsChromaffin_cells.Chrom_SOX2_statusHigh.ATP1B1	1.7680435	7.88168393	4.53720118	2.17E-05	0.00014692	2.12351792	ATP1B1	assignmentsChromaffin_cells.Chrom_SOX2_statusHigh
assignmentsChromaffin_cells.Chrom_SOX2_statusHigh.SELENOS	1.61784089	5.85543051	4.50064391	2.47E-05	0.00016412	2.12157324	SELENOS	assignmentsChromaffin_cells.Chrom_SOX2_statusHigh
assignmentsChromaffin_cells.Chrom_SOX2_statusHigh.IFI35	1.92040975	3.13693003	4.44330719	3.05E-05	0.00019702	2.1178058	IFI35	assignmentsChromaffin_cells.Chrom_SOX2_statusHigh
assignmentsChromaffin_cells.Chrom_SOX2_statusHigh.PYCR3	1.37844216	1.29125751	4.43003525	3.20E-05	0.00020592	2.11449656	PYCR3	assignmentsChromaffin_cells.Chrom_SOX2_statusHigh
assignmentsChromaffin_cells.Chrom_SOX2_statusHigh.FN3KRP	0.94418324	3.9706443	4.46738925	2.79E-05	0.0001824	2.11152236	FN3KRP	assignmentsChromaffin_cells.Chrom_SOX2_statusHigh
assignmentsChromaffin_cells.Chrom_SOX2_statusHigh.PHF23	1.42688707	3.08069541	4.45151353	2.96E-05	0.00019215	2.10881844	PHF23	assignmentsChromaffin_cells.Chrom_SOX2_statusHigh
assignmentsChromaffin_cells.Chrom_SOX2_statusHigh.UBE2T	1.48902788	2.27029378	4.45226338	2.95E-05	0.00019173	2.10680187	UBE2T	assignmentsChromaffin_cells.Chrom_SOX2_statusHigh
assignmentsChromaffin_cells.Chrom_SOX2_statusHigh.MYO7B	2.69406086	-0.0549859	4.39428804	3.65E-05	0.00023032	2.10585657	MYO7B	assignmentsChromaffin_cells.Chrom_SOX2_statusHigh
assignmentsChromaffin_cells.Chrom_SOX2_statusHigh.RASA1	-0.8422217	7.5411087	-4.4983718	2.49E-05	0.00016522	2.10316178	RASA1	assignmentsChromaffin_cells.Chrom_SOX2_statusHigh
assignmentsChromaffin_cells.Chrom_SOX2_statusHigh.ZNF579	1.47553154	2.01154395	4.4383175	3.11E-05	0.00020025	2.1031429	ZNF579	assignmentsChromaffin_cells.Chrom_SOX2_statusHigh
assignmentsChromaffin_cells.Chrom_SOX2_statusHigh.THADA	-0.9448143	7.25702974	-4.49314	2.54E-05	0.00016767	2.10282963	THADA	assignmentsChromaffin_cells.Chrom_SOX2_statusHigh
assignmentsChromaffin_cells.Chrom_SOX2_statusHigh.ENOX1	-3.3276289	5.46794865	-4.4624326	2.85E-05	0.00018601	2.10132574	ENOX1	assignmentsChromaffin_cells.Chrom_SOX2_statusHigh
assignmentsChromaffin_cells.Chrom_SOX2_statusHigh.PSMC1	0.92712575	5.09532864	4.48004628	2.67E-05	0.00017481	2.09845363	PSMC1	assignmentsChromaffin_cells.Chrom_SOX2_statusHigh
assignmentsChromaffin_cells.Chrom_SOX2_statusHigh.RIC8A	1.23308604	3.45565103	4.44824323	3.00E-05	0.00019411	2.09632952	RIC8A	assignmentsChromaffin_cells.Chrom_SOX2_statusHigh
assignmentsChromaffin_cells.Chrom_SOX2_statusHigh.AQP1	2.30518552	3.0398975	4.43393317	3.16E-05	0.00020342	2.08686916	AQP1	assignmentsChromaffin_cells.Chrom_SOX2_statusHigh
assignmentsChromaffin_cells.Chrom_SOX2_statusHigh.RWDD2A	1.22174049	2.33552954	4.44395767	3.05E-05	0.00019678	2.08364921	RWDD2A	assignmentsChromaffin_cells.Chrom_SOX2_statusHigh
assignmentsChromaffin_cells.Chrom_SOX2_statusHigh.NPPC	3.46991724	0.01968697	4.39417477	3.66E-05	0.00023086	2.08226146	NPPC	assignmentsChromaffin_cells.Chrom_SOX2_statusHigh
assignmentsChromaffin_cells.Chrom_SOX2_statusHigh.TNIP2	1.26033252	3.79364086	4.44182399	3.07E-05	0.00019793	2.07938039	TNIP2	assignmentsChromaffin_cells.Chrom_SOX2_statusHigh
assignmentsChromaffin_cells.Chrom_SOX2_statusHigh.SUSD2	2.3318753	0.19267221	4.38382509	3.79E-05	0.00023768	2.07878391	SUSD2	assignmentsChromaffin_cells.Chrom_SOX2_statusHigh
assignmentsChromaffin_cells.Chrom_SOX2_statusHigh.ATM	-1.1094898	7.52814776	-4.4909997	2.56E-05	0.00016879	2.07716323	ATM	assignmentsChromaffin_cells.Chrom_SOX2_statusHigh
assignmentsChromaffin_cells.Chrom_SOX2_statusHigh.TMEM135	-0.9943275	7.16678754	-4.488679	2.58E-05	0.00017003	2.07503502	TMEM135	assignmentsChromaffin_cells.Chrom_SOX2_statusHigh
assignmentsChromaffin_cells.Chrom_SOX2_statusHigh.PHKB	-0.9263098	7.48454542	-4.4890817	2.58E-05	0.00016985	2.07167202	PHKB	assignmentsChromaffin_cells.Chrom_SOX2_statusHigh
assignmentsChromaffin_cells.Chrom_SOX2_statusHigh.NDUFA6-DT	-1.0991813	4.10897697	-4.4434664	3.05E-05	0.00019702	2.06521046	NDUFA6-DT	assignmentsChromaffin_cells.Chrom_SOX2_statusHigh
assignmentsChromaffin_cells.Chrom_SOX2_statusHigh.LYPD1	3.10829632	0.92704474	4.38540587	3.78E-05	0.00023688	2.06116066	LYPD1	assignmentsChromaffin_cells.Chrom_SOX2_statusHigh
assignmentsChromaffin_cells.Chrom_SOX2_statusHigh.CBX5	0.76350722	5.8369248	4.47845931	2.68E-05	0.00017576	2.05780828	CBX5	assignmentsChromaffin_cells.Chrom_SOX2_statusHigh
assignmentsChromaffin_cells.Chrom_SOX2_statusHigh.WBP2	1.11531714	5.10919487	4.46922587	2.78E-05	0.00018124	2.05378997	WBP2	assignmentsChromaffin_cells.Chrom_SOX2_statusHigh
assignmentsChromaffin_cells.Chrom_SOX2_statusHigh.CKB	2.63188465	1.66443876	4.42252889	3.30E-05	0.00021148	2.05242037	CKB	assignmentsChromaffin_cells.Chrom_SOX2_statusHigh
assignmentsChromaffin_cells.Chrom_SOX2_statusHigh.TMEM126A	1.359379	3.32206606	4.44417191	3.04E-05	0.00019671	2.05145594	TMEM126A	assignmentsChromaffin_cells.Chrom_SOX2_statusHigh
assignmentsChromaffin_cells.Chrom_SOX2_statusHigh.LAX1	2.47771453	0.2858741	4.3796916	3.85E-05	0.00024044	2.04677454	LAX1	assignmentsChromaffin_cells.Chrom_SOX2_statusHigh
assignmentsChromaffin_cells.Chrom_SOX2_statusHigh.PREB	1.24965351	3.61811349	4.44306636	3.06E-05	0.00019711	2.04667455	PREB	assignmentsChromaffin_cells.Chrom_SOX2_statusHigh
assignmentsChromaffin_cells.Chrom_SOX2_statusHigh.PYCR1	1.66523321	2.04041318	4.43567092	3.14E-05	0.00020204	2.04577412	PYCR1	assignmentsChromaffin_cells.Chrom_SOX2_statusHigh
assignmentsChromaffin_cells.Chrom_SOX2_statusHigh.NR2C2AP	2.41938676	1.78489851	4.4095767	3.46E-05	0.00021961	2.04342609	NR2C2AP	assignmentsChromaffin_cells.Chrom_SOX2_statusHigh
assignmentsChromaffin_cells.Chrom_SOX2_statusHigh.CNIH1	0.93049893	4.80861394	4.46257844	2.84E-05	0.0001855	2.04137011	CNIH1	assignmentsChromaffin_cells.Chrom_SOX2_statusHigh
assignmentsChromaffin_cells.Chrom_SOX2_statusHigh.BTBD7	-0.9135602	6.85764811	-4.4710307	2.76E-05	0.00018012	2.04110345	BTBD7	assignmentsChromaffin_cells.Chrom_SOX2_statusHigh
assignmentsChromaffin_cells.Chrom_SOX2_statusHigh.MIS18A	1.13310065	2.13072214	4.40968626	3.45E-05	0.00021946	2.02517748	MIS18A	assignmentsChromaffin_cells.Chrom_SOX2_statusHigh
assignmentsChromaffin_cells.Chrom_SOX2_statusHigh.NPTX2	3.98471509	1.1669974	4.37119525	3.98E-05	0.00024705	2.02410926	NPTX2	assignmentsChromaffin_cells.Chrom_SOX2_statusHigh
assignmentsChromaffin_cells.Chrom_SOX2_statusHigh.SUB1	1.23572266	6.5199141	4.47570811	2.71E-05	0.00017741	2.0180979	SUB1	assignmentsChromaffin_cells.Chrom_SOX2_statusHigh
assignmentsChromaffin_cells.Chrom_SOX2_statusHigh.SLIRP	1.04201993	5.44572039	4.46239383	2.85E-05	0.00018555	2.01652815	SLIRP	assignmentsChromaffin_cells.Chrom_SOX2_statusHigh
assignmentsChromaffin_cells.Chrom_SOX2_statusHigh.SLC25A21-A	1.34970816	1.27599274	4.41025654	3.45E-05	0.00021917	2.01477506	SLC25A21-AS	assignmentsChromaffin_cells.Chrom_SOX2_statusHigh
assignmentsChromaffin_cells.Chrom_SOX2_statusHigh.ZNF559	-1.0707262	5.48919191	-4.4557565	2.92E-05	0.00018967	2.0143469	ZNF559	assignmentsChromaffin_cells.Chrom_SOX2_statusHigh
assignmentsChromaffin_cells.Chrom_SOX2_statusHigh.TFAM	1.07177311	4.51304732	4.44602827	3.02E-05	0.00019553	2.00652757	TFAM	assignmentsChromaffin_cells.Chrom_SOX2_statusHigh
assignmentsChromaffin_cells.Chrom_SOX2_statusHigh.C2CD3	-0.8348263	5.69808894	-4.4505021	2.97E-05	0.00019266	2.00417889	C2CD3	assignmentsChromaffin_cells.Chrom_SOX2_statusHigh
assignmentsChromaffin_cells.Chrom_SOX2_statusHigh.TCF12	-0.956333	9.30129675	-4.4866002	2.60E-05	0.00017106	2.00263011	TCF12	assignmentsChromaffin_cells.Chrom_SOX2_statusHigh

assignmentsChromaffin_cells.Chrom_SOX2_statusHigh.FBXL4	-0.8703933	6.02948247	-4.4528476	2.95E-05	0.00019147	1.99331819	FBXL4	assignmentsChromaffin_cells.Chrom_SOX2_statusHigh
assignmentsChromaffin_cells.Chrom_SOX2_statusHigh.ITGA11	3.48910664	3.03207017	4.412302	3.43E-05	0.00021826	1.99187151	ITGA11	assignmentsChromaffin_cells.Chrom_SOX2_statusHigh
assignmentsChromaffin_cells.Chrom_SOX2_statusHigh.DENND11	-1.0564776	5.15654548	-4.4296161	3.21E-05	0.00020616	1.99053831	DENND11	assignmentsChromaffin_cells.Chrom_SOX2_statusHigh
assignmentsChromaffin_cells.Chrom_SOX2_statusHigh.GRIK3	-4.0117892	3.55436538	-4.3936327	3.67E-05	0.00023126	1.98887027	GRIK3	assignmentsChromaffin_cells.Chrom_SOX2_statusHigh
assignmentsChromaffin_cells.Chrom_SOX2_statusHigh.ERAL1	1.14935537	2.82165561	4.41800532	3.35E-05	0.00021399	1.98050784	ERAL1	assignmentsChromaffin_cells.Chrom_SOX2_statusHigh
assignmentsChromaffin_cells.Chrom_SOX2_statusHigh.ZCCHC7	-0.8868093	8.39416586	-4.4775658	2.69E-05	0.00017627	1.97783939	ZCCHC7	assignmentsChromaffin_cells.Chrom_SOX2_statusHigh
assignmentsChromaffin_cells.Chrom_SOX2_statusHigh.BLK	-3.2503202	-0.1201197	-4.3776651	3.88E-05	0.00024194	1.97692163	BLK	assignmentsChromaffin_cells.Chrom_SOX2_statusHigh
assignmentsChromaffin_cells.Chrom_SOX2_statusHigh.PTBP2	-0.9504629	7.28519456	-4.4550091	2.92E-05	0.00019011	1.97098026	PTBP2	assignmentsChromaffin_cells.Chrom_SOX2_statusHigh
assignmentsChromaffin_cells.Chrom_SOX2_statusHigh.DGKQ	-1.5773226	2.53091025	-4.3822764	3.82E-05	0.00023856	1.96814012	DGKQ	assignmentsChromaffin_cells.Chrom_SOX2_statusHigh
assignmentsChromaffin_cells.Chrom_SOX2_statusHigh.MLH3	-0.9891916	5.74016486	-4.4414606	3.07E-05	0.00019811	1.9681334	MLH3	assignmentsChromaffin_cells.Chrom_SOX2_statusHigh
assignmentsChromaffin_cells.Chrom_SOX2_statusHigh.SESTD1	-1.0983175	6.4966288	-4.4433519	3.05E-05	0.00019702	1.96619055	SESTD1	assignmentsChromaffin_cells.Chrom_SOX2_statusHigh
assignmentsChromaffin_cells.Chrom_SOX2_statusHigh.ZHX3	-1.230493	5.98643865	-4.4378315	3.11E-05	0.00020053	1.9589712	ZHX3	assignmentsChromaffin_cells.Chrom_SOX2_statusHigh
assignmentsChromaffin_cells.Chrom_SOX2_statusHigh.GRK3	-1.7594526	7.01507767	-4.4528076	2.95E-05	0.00019166	1.95642384	GRK3	assignmentsChromaffin_cells.Chrom_SOX2_statusHigh
assignmentsChromaffin_cells.Chrom_SOX2_statusHigh.SRBD1	-0.9208454	6.60657871	-4.4392412	3.10E-05	0.00019965	1.95320533	SRBD1	assignmentsChromaffin_cells.Chrom_SOX2_statusHigh
assignmentsChromaffin_cells.Chrom_SOX2_statusHigh.DDX49	1.08645809	3.44176686	4.4150109	3.39E-05	0.00021583	1.95255686	DDX49	assignmentsChromaffin_cells.Chrom_SOX2_statusHigh
assignmentsChromaffin_cells.Chrom_SOX2_statusHigh.TMEM41A	1.11955101	3.25213647	4.40887632	3.46E-05	0.00021985	1.95079394	TMEM41A	assignmentsChromaffin_cells.Chrom_SOX2_statusHigh
assignmentsChromaffin_cells.Chrom_SOX2_statusHigh.UBE2G2	-0.8399765	6.67041065	-4.4493041	2.99E-05	0.00019343	1.95072884	UBE2G2	assignmentsChromaffin_cells.Chrom_SOX2_statusHigh
assignmentsChromaffin_cells.Chrom_SOX2_statusHigh.TTC28-AS1	-0.9114856	5.37850661	-4.4349874	3.15E-05	0.00020246	1.94758859	TTC28-AS1	assignmentsChromaffin_cells.Chrom_SOX2_statusHigh
assignmentsChromaffin_cells.Chrom_SOX2_statusHigh.NUP50-DT	-0.9966031	4.78551146	-4.4162975	3.37E-05	0.00021507	1.9461524	NUP50-DT	assignmentsChromaffin_cells.Chrom_SOX2_statusHigh
assignmentsChromaffin_cells.Chrom_SOX2_statusHigh.HDAC11	3.35411692	1.89547167	4.37240196	3.97E-05	0.00024635	1.94285029	HDAC11	assignmentsChromaffin_cells.Chrom_SOX2_statusHigh
assignmentsChromaffin_cells.Chrom_SOX2_statusHigh.SIRT5	-1.0005013	4.36018653	-4.4119898	3.42E-05	0.00021805	1.93754092	SIRT5	assignmentsChromaffin_cells.Chrom_SOX2_statusHigh
assignmentsChromaffin_cells.Chrom_SOX2_statusHigh.ETV7	2.35983524	0.18356872	4.33953823	4.46E-05	0.00027179	1.93351241	ETV7	assignmentsChromaffin_cells.Chrom_SOX2_statusHigh
assignmentsChromaffin_cells.Chrom_SOX2_statusHigh.NUCB2	1.27194642	6.35348706	4.45715361	2.90E-05	0.00018877	1.93314217	NUCB2	assignmentsChromaffin_cells.Chrom_SOX2_statusHigh
assignmentsChromaffin_cells.Chrom_SOX2_statusHigh.CHD9	-0.9798563	8.60893078	-4.4645912	2.82E-05	0.00018421	1.93207925	CHD9	assignmentsChromaffin_cells.Chrom_SOX2_statusHigh
assignmentsChromaffin_cells.Chrom_SOX2_statusHigh.RGN	3.11604814	0.87165848	4.34381844	4.39E-05	0.00026855	1.92957205	RGN	assignmentsChromaffin_cells.Chrom_SOX2_statusHigh
assignmentsChromaffin_cells.Chrom_SOX2_statusHigh.GLT8D1	1.08623961	4.18758416	4.42269732	3.29E-05	0.00021094	1.925269	GLT8D1	assignmentsChromaffin_cells.Chrom_SOX2_statusHigh
assignmentsChromaffin_cells.Chrom_SOX2_statusHigh.TAPBPL	1.18693651	3.19225481	4.39880676	3.59E-05	0.00022718	1.92399147	TAPBPL	assignmentsChromaffin_cells.Chrom_SOX2_statusHigh
assignmentsChromaffin_cells.Chrom_SOX2_statusHigh.NCOR1	-0.780177	8.0949701	-4.4535454	2.94E-05	0.00019106	1.9194826	NCOR1	assignmentsChromaffin_cells.Chrom_SOX2_statusHigh
assignmentsChromaffin_cells.Chrom_SOX2_statusHigh.ACBD6	-0.8503463	7.48137987	-4.4514459	2.96E-05	0.00019215	1.91387331	ACBD6	assignmentsChromaffin_cells.Chrom_SOX2_statusHigh
assignmentsChromaffin_cells.Chrom_SOX2_statusHigh.SH3TC1	2.84160516	3.61204283	4.37214758	3.97E-05	0.00024649	1.91357946	SH3TC1	assignmentsChromaffin_cells.Chrom_SOX2_statusHigh
assignmentsChromaffin_cells.Chrom_SOX2_statusHigh.STK36	-1.0971316	3.40538489	-4.3955193	3.64E-05	0.00022938	1.91199738	STK36	assignmentsChromaffin_cells.Chrom_SOX2_statusHigh
assignmentsChromaffin_cells.Chrom_SOX2_statusHigh.CNTFR	3.17569297	-0.0712416	4.33386956	4.55E-05	0.00027613	1.91178031	CNTFR	assignmentsChromaffin_cells.Chrom_SOX2_statusHigh
assignmentsChromaffin_cells.Chrom_SOX2_statusHigh.VEZF1	0.80155731	5.36712939	4.4294456	3.21E-05	0.0002062	1.90977445	VEZF1	assignmentsChromaffin_cells.Chrom_SOX2_statusHigh
assignmentsChromaffin_cells.Chrom_SOX2_statusHigh.ZNF544	-1.0166228	5.79569035	-4.4284735	3.22E-05	0.00020685	1.90499136	ZNF544	assignmentsChromaffin_cells.Chrom_SOX2_statusHigh
assignmentsChromaffin_cells.Chrom_SOX2_statusHigh.LGALS1	3.99563448	4.06128783	4.37708868	3.90E-05	0.00024294	1.9039708	LGALS1	assignmentsChromaffin_cells.Chrom_SOX2_statusHigh
assignmentsChromaffin_cells.Chrom_SOX2_statusHigh.TARBP2	1.16235908	2.53486833	4.39266893	3.67E-05	0.00023132	1.90174283	TARBP2	assignmentsChromaffin_cells.Chrom_SOX2_statusHigh
assignmentsChromaffin_cells.Chrom_SOX2_statusHigh.IL3RA	-2.9735208	4.61895397	-4.3820782	3.83E-05	0.00023932	1.9003103	IL3RA	assignmentsChromaffin_cells.Chrom_SOX2_statusHigh
assignmentsChromaffin_cells.Chrom_SOX2_statusHigh.EXOC6	-1.0918759	6.8479522	-4.4308569	3.20E-05	0.00020539	1.89394799	EXOC6	assignmentsChromaffin_cells.Chrom_SOX2_statusHigh
assignmentsChromaffin_cells.Chrom_SOX2_statusHigh.PAIP1	0.88779596	5.10370376	4.41991936	3.33E-05	0.00021263	1.88902987	PAIP1	assignmentsChromaffin_cells.Chrom_SOX2_statusHigh
assignmentsChromaffin_cells.Chrom_SOX2_statusHigh.KCTD16	-2.5854323	5.16411133	-4.4171782	3.37E-05	0.00021507	1.88842664	KCTD16	assignmentsChromaffin_cells.Chrom_SOX2_statusHigh
assignmentsChromaffin_cells.Chrom_SOX2_statusHigh.ANKRD13C	-0.9559022	6.16663331	-4.4166665	3.37E-05	0.00021495	1.88340316	ANKRD13C	assignmentsChromaffin_cells.Chrom_SOX2_statusHigh
assignmentsChromaffin_cells.Chrom_SOX2_statusHigh.VMA21	1.63555157	4.25331884	4.41348718	3.41E-05	0.00021699	1.88267433	VMA21	assignmentsChromaffin_cells.Chrom_SOX2_statusHigh
assignmentsChromaffin_cells.Chrom_SOX2_statusHigh.METTL27	2.43154796	1.28347785	4.3545326	4.22E-05	0.00026024	1.8808133	METTL27	assignmentsChromaffin_cells.Chrom_SOX2_statusHigh
assignmentsChromaffin_cells.Chrom_SOX2_statusHigh.CHN1	-1.4242822	6.16675846	-4.4257738	3.26E-05	0.00020866	1.88032023	CHN1	assignmentsChromaffin_cells.Chrom_SOX2_statusHigh
assignmentsChromaffin_cells.Chrom_SOX2_statusHigh.CTSC	2.12857044	5.28125857	4.38835478	3.74E-05	0.0002348	1.87935018	CTSC	assignmentsChromaffin_cells.Chrom_SOX2_statusHigh
assignmentsChromaffin_cells.Chrom_SOX2_statusHigh.PSMD6	0.94583677	4.71498039	4.40949305	3.45E-05	0.00021953	1.87616706	PSMD6	assignmentsChromaffin_cells.Chrom_SOX2_statusHigh
assignmentsChromaffin_cells.Chrom_SOX2_statusHigh.SELENOT	1.23493656	5.46929639	4.42000374	3.32E-05	0.00021263	1.8714625	SELENOT	assignmentsChromaffin_cells.Chrom_SOX2_statusHigh
assignmentsChromaffin_cells.Chrom_SOX2_statusHigh.NUP93	-0.7365468	5.21204259	-4.4017353	3.55E-05	0.00022512	1.8707769	NUP93	assignmentsChromaffin_cells.Chrom_SOX2_statusHigh
assignmentsChromaffin_cells.Chrom_SOX2_statusHigh.AFAP1	-1.7289145	5.39577442	-4.3873596	3.75E-05	0.00023529	1.8685993	AFAP1	assignmentsChromaffin_cells.Chrom_SOX2_statusHigh
assignmentsChromaffin_cells.Chrom_SOX2_statusHigh.LYSMD2	1.4790907	3.91816044	4.39836197	3.60E-05	0.00022746	1.86321472	LYSMD2	assignmentsChromaffin_cells.Chrom_SOX2_statusHigh
assignmentsChromaffin_cells.Chrom_SOX2_statusHigh.PSMD12	0.80229495	5.85800103	4.42135151	3.31E-05	0.00021181	1.86317047	PSMD12	assignmentsChromaffin_cells.Chrom_SOX2_statusHigh
assignmentsChromaffin_cells.Chrom_SOX2_statusHigh.DNASE1L1	1.12182813	3.59278453	4.38951424	3.72E-05	0.00023372	1.8622638	DNASE1L1	assignmentsChromaffin_cells.Chrom_SOX2_statusHigh
assignmentsChromaffin_cells.Chrom_SOX2_statusHigh.PPP2R2D	-0.8557781	6.42344554	-4.4198723	3.33E-05	0.00021263	1.86213413	PPP2R2D	assignmentsChromaffin_cells.Chrom_SOX2_statusHigh
assignmentsChromaffin_cells.Chrom_SOX2_statusHigh.FKBP7	1.4525308	2.98306367	4.38364046	3.80E-05	0.00023775	1.8522285	FKBP7	assignmentsChromaffin_cells.Chrom_SOX2_statusHigh
assignmentsChromaffin_cells.Chrom_SOX2_statusHigh.MIA2	-0.9003766	6.65412081	-4.4198613	3.33E-05	0.00021263	1.84893946	MIA2	assignmentsChromaffin_cells.Chrom_SOX2_statusHigh
assignmentsChromaffin_cells.Chrom_SOX2_statusHigh.ZNF506	-1.1210262	6.14581976	-4.4098418	3.45E-05	0.00021942	1.84604946	ZNF506	assignmentsChromaffin_cells.Chrom_SOX2_statusHigh
assignmentsChromaffin_cells.Chrom_SOX2_statusHigh.ARHGEF26-	-3.0601119	1.56312589	-4.3219338	4.75E-05	0.00028625	1.84442432	ARHGEF26-A	assignmentsChromaffin_cells.Chrom_SOX2_statusHigh
assignmentsChromaffin_cells.Chrom_SOX2_statusHigh.THAP3	1.03905412	2.64090137	4.36709058	4.03E-05	0.00024977	1.83579204	THAP3	assignmentsChromaffin_cells.Chrom_SOX2_statusHigh
assignmentsChromaffin_cells.Chrom_SOX2_statusHigh.GAK	-0.919622	6.24801963	-4.4050786	3.51E-05	0.00022274	1.83559922	GAK	assignmentsChromaffin_cells.Chrom_SOX2_statusHigh
assignmentsChromaffin_cells.Chrom_SOX2_statusHigh.INTS5	1.77991743	1.55558121	4.34893276	4.31E-05	0.00026415	1.83178676	INTS5	assignmentsChromaffin_cells.Chrom_SOX2_statusHigh
assignmentsChromaffin_cells.Chrom_SOX2_statusHigh.TRUB2	1.02761497	3.24288317	4.37858671	3.87E-05	0.00024122	1.82918959	TRUB2	assignmentsChromaffin_cells.Chrom_SOX2_statusHigh
assignmentsChromaffin_cells.Chrom_SOX2_statusHigh.CDCA5	1.83132002	0.45519318	4.31918665	4.80E-05	0.00028851	1.828681	CDCA5	assignmentsChromaffin_cells.Chrom_SOX2_statusHigh
assignmentsChromaffin_cells.Chrom_SOX2_statusHigh.CHRNA5	1.55974736	2.71784856	4.37709398	3.89E-05	0.00024235	1.82180191	CHRNA5	assignmentsChromaffin_cells.Chrom_SOX2_statusHigh
assignmentsChromaffin_cells.Chrom_SOX2_statusHigh.ENC1	3.07428564	2.91834292	4.33693791	4.51E-05	0.00027402	1.81756274	ENC1	assignmentsChromaffin_cells.Chrom_SOX2_statusHigh

assignmentsChromaffin_cells.Chrom_SOX2_statusHigh.CLYBL	-1.36545	5.23305443	-4.3849472	3.78E-05	0.0002369	1.81671507	CLYBL	assignmentsChromaffin_cells.Chrom_SOX2_statusHigh
assignmentsChromaffin_cells.Chrom_SOX2_statusHigh.IFI27	2.36094595	3.35874079	4.34775712	4.33E-05	0.00026542	1.81655011	IFI27	assignmentsChromaffin_cells.Chrom_SOX2_statusHigh
assignmentsChromaffin_cells.Chrom_SOX2_statusHigh.PTHLH	2.96812249	0.75559493	4.31619052	4.85E-05	0.00029094	1.81525673	PTHLH	assignmentsChromaffin_cells.Chrom_SOX2_statusHigh
assignmentsChromaffin_cells.Chrom_SOX2_statusHigh.TMEM176B	4.10746302	3.03237359	4.33975889	4.46E-05	0.00027203	1.81521384	TMEM176B	assignmentsChromaffin_cells.Chrom_SOX2_statusHigh
assignmentsChromaffin_cells.Chrom_SOX2_statusHigh.CDK2	1.25843986	2.33492188	4.3343999	4.54E-05	0.00027559	1.81496019	CDK2	assignmentsChromaffin_cells.Chrom_SOX2_statusHigh
assignmentsChromaffin_cells.Chrom_SOX2_statusHigh.FAM241B	1.57451443	1.74201719	4.36310758	4.09E-05	0.00025303	1.80012233	FAM241B	assignmentsChromaffin_cells.Chrom_SOX2_statusHigh
assignmentsChromaffin_cells.Chrom_SOX2_statusHigh.RB1	-0.7708482	8.13222239	-4.4153915	3.38E-05	0.00021561	1.79947679	RB1	assignmentsChromaffin_cells.Chrom_SOX2_statusHigh
assignmentsChromaffin_cells.Chrom_SOX2_statusHigh.ZNF76	-0.7746468	4.92564495	-4.382922	3.81E-05	0.00023809	1.79912095	ZNF76	assignmentsChromaffin_cells.Chrom_SOX2_statusHigh
assignmentsChromaffin_cells.Chrom_SOX2_statusHigh.KCTD14	1.55107521	1.39542985	4.34466786	4.37E-05	0.00026755	1.79793976	KCTD14	assignmentsChromaffin_cells.Chrom_SOX2_statusHigh
assignmentsChromaffin_cells.Chrom_SOX2_statusHigh.TMEM185B	1.61241698	2.21739175	4.33999977	4.45E-05	0.00027149	1.79421062	TMEM185B	assignmentsChromaffin_cells.Chrom_SOX2_statusHigh
assignmentsChromaffin_cells.Chrom_SOX2_statusHigh.SCARB2	0.74145982	6.58476776	4.4116897	3.43E-05	0.0002182	1.79408108	SCARB2	assignmentsChromaffin_cells.Chrom_SOX2_statusHigh
assignmentsChromaffin_cells.Chrom_SOX2_statusHigh.BNIP3	1.46322365	4.57668761	4.39584311	3.63E-05	0.0002292	1.79287677	BNIP3	assignmentsChromaffin_cells.Chrom_SOX2_statusHigh
assignmentsChromaffin_cells.Chrom_SOX2_statusHigh.KLC1	-0.8729701	7.27680519	-4.4065531	3.49E-05	0.00022163	1.78970017	KLC1	assignmentsChromaffin_cells.Chrom_SOX2_statusHigh
assignmentsChromaffin_cells.Chrom_SOX2_statusHigh.NR2F2-AS1	-4.0749169	4.90119207	-4.326412	4.69E-05	0.00028294	1.7888254	NR2F2-AS1	assignmentsChromaffin_cells.Chrom_SOX2_statusHigh
assignmentsChromaffin_cells.Chrom_SOX2_statusHigh.GAN	-0.8814608	5.44619672	-4.3861534	3.76E-05	0.00023614	1.78862317	GAN	assignmentsChromaffin_cells.Chrom_SOX2_statusHigh
assignmentsChromaffin_cells.Chrom_SOX2_statusHigh.CBR4	-0.8327208	6.56081645	-4.3962761	3.63E-05	0.00022892	1.77617175	CBR4	assignmentsChromaffin_cells.Chrom_SOX2_statusHigh
assignmentsChromaffin_cells.Chrom_SOX2_statusHigh.LIN7B	1.28845655	3.19759161	4.37457825	3.92E-05	0.00024429	1.77597348	LIN7B	assignmentsChromaffin_cells.Chrom_SOX2_statusHigh
assignmentsChromaffin_cells.Chrom_SOX2_statusHigh.MFSD14B	-0.8734243	5.85739133	-4.3843988	3.79E-05	0.00023728	1.77482758	MFSD14B	assignmentsChromaffin_cells.Chrom_SOX2_statusHigh
assignmentsChromaffin_cells.Chrom_SOX2_statusHigh.MED27	-0.9823312	6.95881656	-4.4031548	3.54E-05	0.00022422	1.77354321	MED27	assignmentsChromaffin_cells.Chrom_SOX2_statusHigh
assignmentsChromaffin_cells.Chrom_SOX2_statusHigh.ZDHHC24	1.29390708	3.62222864	4.35956767	4.14E-05	0.0002562	1.77178566	ZDHHC24	assignmentsChromaffin_cells.Chrom_SOX2_statusHigh
assignmentsChromaffin_cells.Chrom_SOX2_statusHigh.TXNDC12	1.26907346	4.58391018	4.37177045	3.96E-05	0.00024632	1.76071039	TXNDC12	assignmentsChromaffin_cells.Chrom_SOX2_statusHigh
assignmentsChromaffin_cells.Chrom_SOX2_statusHigh.RPL13A	2.87292537	7.49617094	4.41665133	3.38E-05	0.00021533	1.75892043	RPL13A	assignmentsChromaffin_cells.Chrom_SOX2_statusHigh
assignmentsChromaffin_cells.Chrom_SOX2_statusHigh.LFNG	2.82194971	2.0395328	4.2887283	5.35E-05	0.0003165	1.75880002	LFNG	assignmentsChromaffin_cells.Chrom_SOX2_statusHigh
assignmentsChromaffin_cells.Chrom_SOX2_statusHigh.MPP1	1.51439811	4.17560904	4.35930204	4.15E-05	0.00025626	1.75651633	MPP1	assignmentsChromaffin_cells.Chrom_SOX2_statusHigh
assignmentsChromaffin_cells.Chrom_SOX2_statusHigh.LSM8	0.80172122	5.64784835	4.38978609	3.71E-05	0.00023358	1.75521013	LSM8	assignmentsChromaffin_cells.Chrom_SOX2_statusHigh
assignmentsChromaffin_cells.Chrom_SOX2_statusHigh.GLMN	-0.8683548	5.50296763	-4.3721787	3.96E-05	0.00024605	1.75485195	GLMN	assignmentsChromaffin_cells.Chrom_SOX2_statusHigh
assignmentsChromaffin_cells.Chrom_SOX2_statusHigh.PIGG	-0.8470785	5.23528109	-4.3704763	3.98E-05	0.00024705	1.75326937	PIGG	assignmentsChromaffin_cells.Chrom_SOX2_statusHigh
assignmentsChromaffin_cells.Chrom_SOX2_statusHigh.MARCKSL1	2.55587839	2.51687175	4.30070134	5.02E-05	0.00029919	1.74707938	MARCKSL1	assignmentsChromaffin_cells.Chrom_SOX2_statusHigh
assignmentsChromaffin_cells.Chrom_SOX2_statusHigh.TMLHE-AS1	-2.5414278	3.35865253	-4.3280966	4.65E-05	0.0002812	1.7465048	TMLHE-AS1	assignmentsChromaffin_cells.Chrom_SOX2_statusHigh
assignmentsChromaffin_cells.Chrom_SOX2_statusHigh.GATAD2B	-0.8557014	7.45335019	-4.3973645	3.61E-05	0.00022811	1.74581189	GATAD2B	assignmentsChromaffin_cells.Chrom_SOX2_statusHigh
assignmentsChromaffin_cells.Chrom_SOX2_statusHigh.KDM4C	-0.9244884	7.80286732	-4.4030159	3.54E-05	0.00022425	1.74370061	KDM4C	assignmentsChromaffin_cells.Chrom_SOX2_statusHigh
assignmentsChromaffin_cells.Chrom_SOX2_statusHigh.NR2F1	2.60678907	2.25646978	4.32995345	4.62E-05	0.00027962	1.74219855	NR2F1	assignmentsChromaffin_cells.Chrom_SOX2_statusHigh
assignmentsChromaffin_cells.Chrom_SOX2_statusHigh.REEP4	1.50627482	2.30138847	4.32482893	4.70E-05	0.00028358	1.7367579	REEP4	assignmentsChromaffin_cells.Chrom_SOX2_statusHigh
assignmentsChromaffin_cells.Chrom_SOX2_statusHigh.PAXIP1	-1.0635298	4.36887066	-4.3526784	4.25E-05	0.00026138	1.73532615	PAXIP1	assignmentsChromaffin_cells.Chrom_SOX2_statusHigh
assignmentsChromaffin_cells.Chrom_SOX2_statusHigh.HELZ	-0.6366863	7.65369361	-4.3997954	3.58E-05	0.00022645	1.73507091	HELZ	assignmentsChromaffin_cells.Chrom_SOX2_statusHigh
assignmentsChromaffin_cells.Chrom_SOX2_statusHigh.MAP1S	1.01586184	3.52485946	4.34739456	4.33E-05	0.00026542	1.73416272	MAP1S	assignmentsChromaffin_cells.Chrom_SOX2_statusHigh
assignmentsChromaffin_cells.Chrom_SOX2_statusHigh.FBXW7	-0.9198762	8.14140156	-4.4003518	3.57E-05	0.00022608	1.73185449	FBXW7	assignmentsChromaffin_cells.Chrom_SOX2_statusHigh
assignmentsChromaffin_cells.Chrom_SOX2_statusHigh.TMEM182	-1.4604404	4.18342628	-4.3556758	4.20E-05	0.00025935	1.73164455	TMEM182	assignmentsChromaffin_cells.Chrom_SOX2_statusHigh
assignmentsChromaffin_cells.Chrom_SOX2_statusHigh.FAM111A-D	1.99685763	1.40610382	4.29998606	5.14E-05	0.00030583	1.73005204	FAM111A-D1	assignmentsChromaffin_cells.Chrom_SOX2_statusHigh
assignmentsChromaffin_cells.Chrom_SOX2_statusHigh.ODC1	1.50769482	4.38886455	4.36557947	4.05E-05	0.00025096	1.73002482	ODC1	assignmentsChromaffin_cells.Chrom_SOX2_statusHigh
assignmentsChromaffin_cells.Chrom_SOX2_statusHigh.RNGTT	-0.7499529	7.1290297	-4.389325	3.72E-05	0.00023379	1.72955862	RNGTT	assignmentsChromaffin_cells.Chrom_SOX2_statusHigh
assignmentsChromaffin_cells.Chrom_SOX2_statusHigh.MRPL49	1.31772823	2.38954633	4.33937784	4.46E-05	0.00027179	1.72868487	MRPL49	assignmentsChromaffin_cells.Chrom_SOX2_statusHigh
assignmentsChromaffin_cells.Chrom_SOX2_statusHigh.ARMCX2	2.61112956	3.26990822	4.35285951	4.26E-05	0.00026183	1.72604712	ARMCX2	assignmentsChromaffin_cells.Chrom_SOX2_statusHigh
assignmentsChromaffin_cells.Chrom_SOX2_statusHigh.ZC2HC1B	-2.999665	1.11757963	-4.2738552	5.65E-05	0.00033158	1.72143949	ZC2HC1B	assignmentsChromaffin_cells.Chrom_SOX2_statusHigh
assignmentsChromaffin_cells.Chrom_SOX2_statusHigh.NPRL3	-0.8835403	5.10006937	-4.3663607	4.04E-05	0.00025034	1.7204793	NPRL3	assignmentsChromaffin_cells.Chrom_SOX2_statusHigh
assignmentsChromaffin_cells.Chrom_SOX2_statusHigh.TENM2	-3.6286939	4.90444666	-4.349696	4.31E-05	0.00026415	1.71948152	TENM2	assignmentsChromaffin_cells.Chrom_SOX2_statusHigh
assignmentsChromaffin_cells.Chrom_SOX2_statusHigh.ITFG2	-0.9546641	5.736081	-4.3724851	3.95E-05	0.00024587	1.71876647	ITFG2	assignmentsChromaffin_cells.Chrom_SOX2_statusHigh
assignmentsChromaffin_cells.Chrom_SOX2_statusHigh.REPS1	-1.1405873	6.86850294	-4.3832503	3.80E-05	0.0002379	1.71871875	REPS1	assignmentsChromaffin_cells.Chrom_SOX2_statusHigh
assignmentsChromaffin_cells.Chrom_SOX2_statusHigh.SPECC1	-2.2484684	6.19400321	-4.3555708	4.22E-05	0.00025994	1.71784316	SPECC1	assignmentsChromaffin_cells.Chrom_SOX2_statusHigh
assignmentsChromaffin_cells.Chrom_SOX2_statusHigh.UQCC2	1.07681388	4.8151943	4.37280041	3.95E-05	0.00024578	1.71653018	UQCC2	assignmentsChromaffin_cells.Chrom_SOX2_statusHigh
assignmentsChromaffin_cells.Chrom_SOX2_statusHigh.ZC3H14	-0.9226072	6.42388655	-4.3798747	3.85E-05	0.00024037	1.71169472	ZC3H14	assignmentsChromaffin_cells.Chrom_SOX2_statusHigh
assignmentsChromaffin_cells.Chrom_SOX2_statusHigh.BICC1	-2.8033937	5.41108224	-4.3500005	4.30E-05	0.00026406	1.70823259	BICC1	assignmentsChromaffin_cells.Chrom_SOX2_statusHigh
assignmentsChromaffin_cells.Chrom_SOX2_statusHigh.BOP1	1.38911181	2.79157583	4.33171225	4.58E-05	0.00027755	1.7078446	BOP1	assignmentsChromaffin_cells.Chrom_SOX2_statusHigh
assignmentsChromaffin_cells.Chrom_SOX2_statusHigh.TYW3	0.93505887	4.54316179	4.3592968	4.15E-05	0.00025626	1.70391966	TYW3	assignmentsChromaffin_cells.Chrom_SOX2_statusHigh
assignmentsChromaffin_cells.Chrom_SOX2_statusHigh.DNAJC11	-0.8695135	4.95660847	-4.350915	4.28E-05	0.00026286	1.70304358	DNAJC11	assignmentsChromaffin_cells.Chrom_SOX2_statusHigh
assignmentsChromaffin_cells.Chrom_SOX2_statusHigh.MICAL3	-1.2172442	6.58555049	-4.3789399	3.86E-05	0.00024101	1.70105862	MICAL3	assignmentsChromaffin_cells.Chrom_SOX2_statusHigh
assignmentsChromaffin_cells.Chrom_SOX2_statusHigh.DIP2C	-1.046318	7.63690122	-4.3903244	3.71E-05	0.00023321	1.70038154	DIP2C	assignmentsChromaffin_cells.Chrom_SOX2_statusHigh
assignmentsChromaffin_cells.Chrom_SOX2_statusHigh.KANSL1-AS:	1.43497707	1.36558264	4.29687372	5.20E-05	0.0003086	1.69901111	KANSL1-AS1	assignmentsChromaffin_cells.Chrom_SOX2_statusHigh
assignmentsChromaffin_cells.Chrom_SOX2_statusHigh.XPR1	-1.0039574	7.53029735	-4.3868779	3.75E-05	0.00023561	1.6969029	XPR1	assignmentsChromaffin_cells.Chrom_SOX2_statusHigh
assignmentsChromaffin_cells.Chrom_SOX2_statusHigh.GCN1	-0.7301396	5.18169555	-4.3569798	4.18E-05	0.00025822	1.69654537	GCN1	assignmentsChromaffin_cells.Chrom_SOX2_statusHigh
assignmentsChromaffin_cells.Chrom_SOX2_statusHigh.ZFYVE26	-1.0896053	5.07917105	-4.3503073	4.29E-05	0.00026324	1.69623988	ZFYVE26	assignmentsChromaffin_cells.Chrom_SOX2_statusHigh
assignmentsChromaffin_cells.Chrom_SOX2_statusHigh.SIRT7	0.88312603	3.79211646	4.34190028	4.42E-05	0.00026994	1.6958718	SIRT7	assignmentsChromaffin_cells.Chrom_SOX2_statusHigh
assignmentsChromaffin_cells.Chrom_SOX2_statusHigh.NUP35	-0.7221141	4.25136053	-4.3408695	4.43E-05	0.00027084	1.69148817	NUP35	assignmentsChromaffin_cells.Chrom_SOX2_statusHigh
assignmentsChromaffin_cells.Chrom_SOX2_statusHigh.RASD1	4.96856397	3.19196852	4.31469703	4.89E-05	0.00029265	1.69006675	RASD1	assignmentsChromaffin_cells.Chrom_SOX2_statusHigh

assignmentsChromaffin_cells.Chrom_SOX2_statusHigh.ZNF526	0.99124715	2.24731775	4.31647756	4.84E-05	0.00029042	1.68708921	ZNF526	assignmentsChromaffin_cells.Chrom_SOX2_statusHigh
assignmentsChromaffin_cells.Chrom_SOX2_statusHigh.WFDC1	3.5090529	1.93650661	4.30893025	4.99E-05	0.00029802	1.6866379	WFDC1	assignmentsChromaffin_cells.Chrom_SOX2_statusHigh
assignmentsChromaffin_cells.Chrom_SOX2_statusHigh.CAMSAP2	-0.7947585	7.30076448	-4.3851313	3.78E-05	0.00023688	1.68631647	CAMSAP2	assignmentsChromaffin_cells.Chrom_SOX2_statusHigh
assignmentsChromaffin_cells.Chrom_SOX2_statusHigh.RHNO1	1.30153343	1.68340392	4.31375521	4.89E-05	0.00029274	1.68598471	RHNO1	assignmentsChromaffin_cells.Chrom_SOX2_statusHigh
assignmentsChromaffin_cells.Chrom_SOX2_statusHigh.SNX14	-0.7037784	7.3149414	-4.3802959	3.84E-05	0.0002401	1.68597096	SNX14	assignmentsChromaffin_cells.Chrom_SOX2_statusHigh
assignmentsChromaffin_cells.Chrom_SOX2_statusHigh.MORC2	-0.929607	4.8721451	-4.346728	4.34E-05	0.00026596	1.68312661	MORC2	assignmentsChromaffin_cells.Chrom_SOX2_statusHigh
assignmentsChromaffin_cells.Chrom_SOX2_statusHigh.MGAT4B	1.31292284	3.1982159	4.33269815	4.57E-05	0.00027687	1.68255195	MGAT4B	assignmentsChromaffin_cells.Chrom_SOX2_statusHigh
assignmentsChromaffin_cells.Chrom_SOX2_statusHigh.FUBP3	-0.9322301	6.0357412	-4.3634717	4.09E-05	0.00025279	1.68254448	FUBP3	assignmentsChromaffin_cells.Chrom_SOX2_statusHigh
assignmentsChromaffin_cells.Chrom_SOX2_statusHigh.CDC42BPB	-0.9605541	6.74931198	-4.3710138	3.97E-05	0.00024671	1.68147083	CDC42BPB	assignmentsChromaffin_cells.Chrom_SOX2_statusHigh
assignmentsChromaffin_cells.Chrom_SOX2_statusHigh.PHF13	1.21467735	3.04658393	4.32378057	4.72E-05	0.00028434	1.68100511	PHF13	assignmentsChromaffin_cells.Chrom_SOX2_statusHigh
assignmentsChromaffin_cells.Chrom_SOX2_statusHigh.MED7	1.16065328	3.28999695	4.33701906	4.50E-05	0.0002733	1.68097093	MED7	assignmentsChromaffin_cells.Chrom_SOX2_statusHigh
assignmentsChromaffin_cells.Chrom_SOX2_statusHigh.RPS3A	2.92935423	7.32534084	4.39406026	3.67E-05	0.00023099	1.68020852	RPS3A	assignmentsChromaffin_cells.Chrom_SOX2_statusHigh
assignmentsChromaffin_cells.Chrom_SOX2_statusHigh.TMEM74B	-1.7567029	2.06539673	-4.3003901	5.13E-05	0.0003055	1.68013973	TMEM74B	assignmentsChromaffin_cells.Chrom_SOX2_statusHigh
assignmentsChromaffin_cells.Chrom_SOX2_statusHigh.TRIM24	-1.7347954	6.84673193	-4.370482	3.99E-05	0.0002472	1.68003995	TRIM24	assignmentsChromaffin_cells.Chrom_SOX2_statusHigh
assignmentsChromaffin_cells.Chrom_SOX2_statusHigh.LRRC47	0.95934999	3.49391528	4.33318325	4.56E-05	0.00027649	1.67562301	LRRC47	assignmentsChromaffin_cells.Chrom_SOX2_statusHigh
assignmentsChromaffin_cells.Chrom_SOX2_statusHigh.ABHD16A	1.09712922	4.52409173	4.35038644	4.28E-05	0.00026324	1.6742173	ABHD16A	assignmentsChromaffin_cells.Chrom_SOX2_statusHigh
assignmentsChromaffin_cells.Chrom_SOX2_statusHigh.MAT2B	1.07521141	3.99212782	4.33364934	4.55E-05	0.00027613	1.67107236	MAT2B	assignmentsChromaffin_cells.Chrom_SOX2_statusHigh
assignmentsChromaffin_cells.Chrom_SOX2_statusHigh.ID3	3.1875615	4.35809473	4.30267637	5.11E-05	0.00030414	1.66957066	ID3	assignmentsChromaffin_cells.Chrom_SOX2_statusHigh
assignmentsChromaffin_cells.Chrom_SOX2_statusHigh.COMMD3	1.33821016	4.56716946	4.35349197	4.24E-05	0.00026087	1.66899457	COMMD3	assignmentsChromaffin_cells.Chrom_SOX2_statusHigh
assignmentsChromaffin_cells.Chrom_SOX2_statusHigh.ZBTB6	0.90931437	2.57899463	4.31749552	4.83E-05	0.00028979	1.66482441	ZBTB6	assignmentsChromaffin_cells.Chrom_SOX2_statusHigh
assignmentsChromaffin_cells.Chrom_SOX2_statusHigh.ARIH2OS	1.62201571	1.57932828	4.29893456	5.16E-05	0.00030665	1.6633577	ARIH2OS	assignmentsChromaffin_cells.Chrom_SOX2_statusHigh
assignmentsChromaffin_cells.Chrom_SOX2_statusHigh.SMARCAL1	-0.9288118	4.89601669	-4.3373183	4.49E-05	0.00027327	1.66281432	SMARCAL1	assignmentsChromaffin_cells.Chrom_SOX2_statusHigh
assignmentsChromaffin_cells.Chrom_SOX2_statusHigh.CAHM	1.58683875	1.65937801	4.29720183	5.19E-05	0.00030835	1.65992841	CAHM	assignmentsChromaffin_cells.Chrom_SOX2_statusHigh
assignmentsChromaffin_cells.Chrom_SOX2_statusHigh.PCSK5	-2.3916055	5.71774018	-4.315431	4.87E-05	0.00029189	1.65965604	PCSK5	assignmentsChromaffin_cells.Chrom_SOX2_statusHigh
assignmentsChromaffin_cells.Chrom_SOX2_statusHigh.RAB1B	1.37150916	4.08032428	4.33844488	4.47E-05	0.0002724	1.65928199	RAB1B	assignmentsChromaffin_cells.Chrom_SOX2_statusHigh
assignmentsChromaffin_cells.Chrom_SOX2_statusHigh.RANBP9	-1.0078462	6.68515759	-4.3574368	4.18E-05	0.00025789	1.65901095	RANBP9	assignmentsChromaffin_cells.Chrom_SOX2_statusHigh
assignmentsChromaffin_cells.Chrom_SOX2_statusHigh.FANCC	-1.1250845	5.74582509	-4.3492803	4.30E-05	0.00026406	1.65547765	FANCC	assignmentsChromaffin_cells.Chrom_SOX2_statusHigh
assignmentsChromaffin_cells.Chrom_SOX2_statusHigh.GPHN	-1.2228676	9.20739131	-4.3979425	3.60E-05	0.00022772	1.65477657	GPHN	assignmentsChromaffin_cells.Chrom_SOX2_statusHigh
assignmentsChromaffin_cells.Chrom_SOX2_statusHigh.PIGS	0.90015914	3.70763037	4.33125405	4.59E-05	0.0002779	1.65117446	PIGS	assignmentsChromaffin_cells.Chrom_SOX2_statusHigh
assignmentsChromaffin_cells.Chrom_SOX2_statusHigh.LINC02615	-1.1704163	4.65942137	-4.3389281	4.47E-05	0.00027203	1.65030392	LINC02615	assignmentsChromaffin_cells.Chrom_SOX2_statusHigh
assignmentsChromaffin_cells.Chrom_SOX2_statusHigh.SHLD3	1.20932365	1.10861933	4.28838585	5.36E-05	0.00031671	1.64771434	SHLD3	assignmentsChromaffin_cells.Chrom_SOX2_statusHigh
assignmentsChromaffin_cells.Chrom_SOX2_statusHigh.EPHX2	3.7367736	2.5478488	4.31977455	4.80E-05	0.00028851	1.64593082	EPHX2	assignmentsChromaffin_cells.Chrom_SOX2_statusHigh
assignmentsChromaffin_cells.Chrom_SOX2_statusHigh.LY6G5B	-1.1807379	2.24981404	-4.2869525	5.39E-05	0.00031795	1.64331754	LY6G5B	assignmentsChromaffin_cells.Chrom_SOX2_statusHigh
assignmentsChromaffin_cells.Chrom_SOX2_statusHigh.FAF1	-0.9953947	8.26683447	-4.3726743	3.95E-05	0.0002458	1.63749465	FAF1	assignmentsChromaffin_cells.Chrom_SOX2_statusHigh
assignmentsChromaffin_cells.Chrom_SOX2_statusHigh.WWOX	-0.9787732	9.11030859	-4.3835136	3.80E-05	0.00023777	1.63717583	WWOX	assignmentsChromaffin_cells.Chrom_SOX2_statusHigh
assignmentsChromaffin_cells.Chrom_SOX2_statusHigh.DIRAS1	1.81240319	1.64593827	4.31119662	4.94E-05	0.00029513	1.63610174	DIRAS1	assignmentsChromaffin_cells.Chrom_SOX2_statusHigh
assignmentsChromaffin_cells.Chrom_SOX2_statusHigh.TMEM106C	1.15804984	3.492452	4.33454823	4.54E-05	0.00027554	1.63609553	TMEM106C	assignmentsChromaffin_cells.Chrom_SOX2_statusHigh
assignmentsChromaffin_cells.Chrom_SOX2_statusHigh.ZNF281	1.02050239	4.12402441	4.3260895	4.68E-05	0.00028261	1.63344609	ZNF281	assignmentsChromaffin_cells.Chrom_SOX2_statusHigh
assignmentsChromaffin_cells.Chrom_SOX2_statusHigh.ECHDC3	3.11035466	1.44838119	4.26484237	5.85E-05	0.00034166	1.6329479	ECHDC3	assignmentsChromaffin_cells.Chrom_SOX2_statusHigh
assignmentsChromaffin_cells.Chrom_SOX2_statusHigh.IP6K1	-0.958157	5.98171573	-4.3454539	4.36E-05	0.00026699	1.6317584	IP6K1	assignmentsChromaffin_cells.Chrom_SOX2_statusHigh
assignmentsChromaffin_cells.Chrom_SOX2_statusHigh.SNHG26	2.52413289	1.9750904	4.26733645	5.79E-05	0.0003387	1.62952365	SNHG26	assignmentsChromaffin_cells.Chrom_SOX2_statusHigh
assignmentsChromaffin_cells.Chrom_SOX2_statusHigh.PQBP1	1.21003344	4.2739266	4.33725881	4.49E-05	0.00027327	1.62468166	PQBP1	assignmentsChromaffin_cells.Chrom_SOX2_statusHigh
assignmentsChromaffin_cells.Chrom_SOX2_statusHigh.APBB2	-1.5647703	7.41809883	-4.3546831	4.22E-05	0.00026017	1.62372746	APBB2	assignmentsChromaffin_cells.Chrom_SOX2_statusHigh
assignmentsChromaffin_cells.Chrom_SOX2_statusHigh.NFX1	-0.8396591	6.71790442	-4.3533222	4.24E-05	0.00026087	1.61414721	NFX1	assignmentsChromaffin_cells.Chrom_SOX2_statusHigh
assignmentsChromaffin_cells.Chrom_SOX2_statusHigh.ASH1L	-0.6649576	8.72553683	-4.3752825	3.91E-05	0.00024376	1.61036547	ASH1L	assignmentsChromaffin_cells.Chrom_SOX2_statusHigh
assignmentsChromaffin_cells.Chrom_SOX2_statusHigh.CCDC171	-1.6965174	5.95293474	-4.3383827	4.48E-05	0.00027258	1.60648554	CCDC171	assignmentsChromaffin_cells.Chrom_SOX2_statusHigh
assignmentsChromaffin_cells.Chrom_SOX2_statusHigh.ABL1	-1.2779417	7.01541998	-4.3403198	4.44E-05	0.00027128	1.60509191	ABL1	assignmentsChromaffin_cells.Chrom_SOX2_statusHigh
assignmentsChromaffin_cells.Chrom_SOX2_statusHigh.PPP1R11	1.13120665	4.32581404	4.32437982	4.71E-05	0.00028383	1.5961393	PPP1R11	assignmentsChromaffin_cells.Chrom_SOX2_statusHigh
assignmentsChromaffin_cells.Chrom_SOX2_statusHigh.SLX4IP	-1.042538	6.30335715	-4.3394089	4.46E-05	0.00027179	1.59482523	SLX4IP	assignmentsChromaffin_cells.Chrom_SOX2_statusHigh
assignmentsChromaffin_cells.Chrom_SOX2_statusHigh.TTBK2	-0.8908424	6.99961637	-4.3533428	4.24E-05	0.00026087	1.59480282	TTBK2	assignmentsChromaffin_cells.Chrom_SOX2_statusHigh
assignmentsChromaffin_cells.Chrom_SOX2_statusHigh.XPO4	-0.8930396	6.4238234	-4.3429818	4.40E-05	0.00026898	1.59474846	XPO4	assignmentsChromaffin_cells.Chrom_SOX2_statusHigh
assignmentsChromaffin_cells.Chrom_SOX2_statusHigh.NORAD	1.6416211	6.2353036	4.35534282	4.21E-05	0.00025986	1.59449701	NORAD	assignmentsChromaffin_cells.Chrom_SOX2_statusHigh
assignmentsChromaffin_cells.Chrom_SOX2_statusHigh.GPR35	-2.1250336	1.11485669	-4.2354626	6.48E-05	0.00037343	1.59435514	GPR35	assignmentsChromaffin_cells.Chrom_SOX2_statusHigh
assignmentsChromaffin_cells.Chrom_SOX2_statusHigh.POLH	-0.8681569	4.91711122	-4.3234218	4.72E-05	0.0002846	1.59411719	POLH	assignmentsChromaffin_cells.Chrom_SOX2_statusHigh
assignmentsChromaffin_cells.Chrom_SOX2_statusHigh.ELOVL7	-1.7699082	4.5526366	-4.3231783	4.73E-05	0.00028497	1.58715736	ELOVL7	assignmentsChromaffin_cells.Chrom_SOX2_statusHigh
assignmentsChromaffin_cells.Chrom_SOX2_statusHigh.PHTF2	-1.0043817	6.88434424	-4.3447225	4.37E-05	0.00026755	1.58532308	PHTF2	assignmentsChromaffin_cells.Chrom_SOX2_statusHigh
assignmentsChromaffin_cells.Chrom_SOX2_statusHigh.CTIF	-1.0681514	6.68706407	-4.3462565	4.35E-05	0.00026632	1.576488	CTIF	assignmentsChromaffin_cells.Chrom_SOX2_statusHigh
assignmentsChromaffin_cells.Chrom_SOX2_statusHigh.MTRF1L	0.95024507	4.42829613	4.31777258	4.82E-05	0.00028971	1.57269805	MTRF1L	assignmentsChromaffin_cells.Chrom_SOX2_statusHigh
assignmentsChromaffin_cells.Chrom_SOX2_statusHigh.TAF4	-0.9527496	4.68862902	-4.3076762	5.00E-05	0.00029835	1.56878777	TAF4	assignmentsChromaffin_cells.Chrom_SOX2_statusHigh
assignmentsChromaffin_cells.Chrom_SOX2_statusHigh.RPL41	3.18602745	7.95479556	4.36982887	4.00E-05	0.00024809	1.5678448	RPL41	assignmentsChromaffin_cells.Chrom_SOX2_statusHigh
assignmentsChromaffin_cells.Chrom_SOX2_statusHigh.LEPROT	1.35015205	4.91419818	4.31656652	4.84E-05	0.00029042	1.56502745	LEPROT	assignmentsChromaffin_cells.Chrom_SOX2_statusHigh
assignmentsChromaffin_cells.Chrom_SOX2_statusHigh.LRFN3	1.01646468	2.05870443	4.28328207	5.46E-05	0.00032158	1.56415759	LRFN3	assignmentsChromaffin_cells.Chrom_SOX2_statusHigh
assignmentsChromaffin_cells.Chrom_SOX2_statusHigh.ELOVL6	-1.2342255	4.72404509	-4.3097762	4.96E-05	0.00029654	1.55775706	ELOVL6	assignmentsChromaffin_cells.Chrom_SOX2_statusHigh
assignmentsChromaffin_cells.Chrom_SOX2_statusHigh.RND2	1.27537312	1.34476809	4.27968021	5.53E-05	0.00032566	1.55483838	RND2	assignmentsChromaffin_cells.Chrom_SOX2_statusHigh

assignmentsChromaffin_cells.Chrom_SOX2_statusHigh.KCNJ2-AS1	3.04156122	-0.3604195	4.22418625	6.75E-05	0.0003863	1.5505384	KCNJ2-AS1	assignmentsChromaffin_cells.Chrom_SOX2_statusHigh
assignmentsChromaffin_cells.Chrom_SOX2_statusHigh.BRMS1	1.30492165	2.97559126	4.28884582	5.35E-05	0.00031649	1.55024904	BRMS1	assignmentsChromaffin_cells.Chrom_SOX2_statusHigh
assignmentsChromaffin_cells.Chrom_SOX2_statusHigh.PSRC1	1.83992796	1.18085656	4.25247681	6.10E-05	0.00035393	1.54455396	PSRC1	assignmentsChromaffin_cells.Chrom_SOX2_statusHigh
assignmentsChromaffin_cells.Chrom_SOX2_statusHigh.SERPINA3	3.87206244	0.02815871	4.22160542	6.83E-05	0.00039033	1.54365834	SERPINA3	assignmentsChromaffin_cells.Chrom_SOX2_statusHigh
assignmentsChromaffin_cells.Chrom_SOX2_statusHigh.ASXL2	-0.7554606	6.88963518	-4.3318117	4.58E-05	0.00027755	1.54185772	ASXL2	assignmentsChromaffin_cells.Chrom_SOX2_statusHigh
assignmentsChromaffin_cells.Chrom_SOX2_statusHigh.SNRPD3	1.55226144	4.88968692	4.32043677	4.78E-05	0.00028737	1.54108738	SNRPD3	assignmentsChromaffin_cells.Chrom_SOX2_statusHigh
assignmentsChromaffin_cells.Chrom_SOX2_statusHigh.MAPK8	-0.7661533	7.5367869	-4.337029	4.50E-05	0.0002733	1.53776241	MAPK8	assignmentsChromaffin_cells.Chrom_SOX2_statusHigh
assignmentsChromaffin_cells.Chrom_SOX2_statusHigh.ZC3H7B	-0.6502824	4.9988939	-4.3078669	5.00E-05	0.00029826	1.53672234	ZC3H7B	assignmentsChromaffin_cells.Chrom_SOX2_statusHigh
assignmentsChromaffin_cells.Chrom_SOX2_statusHigh.MCAM	2.54662839	3.83183644	4.26322907	5.88E-05	0.00034309	1.53547615	MCAM	assignmentsChromaffin_cells.Chrom_SOX2_statusHigh
assignmentsChromaffin_cells.Chrom_SOX2_statusHigh.BATF3	2.42073652	1.28455011	4.21351908	7.01E-05	0.00039859	1.52431952	BATF3	assignmentsChromaffin_cells.Chrom_SOX2_statusHigh
assignmentsChromaffin_cells.Chrom_SOX2_statusHigh.FUT8	-1.4413046	7.43005459	-4.3320651	4.58E-05	0.00027744	1.52055138	FUT8	assignmentsChromaffin_cells.Chrom_SOX2_statusHigh
assignmentsChromaffin_cells.Chrom_SOX2_statusHigh.RPS17	2.90574136	5.71385831	4.32524612	4.71E-05	0.00028382	1.51913672	RPS17	assignmentsChromaffin_cells.Chrom_SOX2_statusHigh
assignmentsChromaffin_cells.Chrom_SOX2_statusHigh.SF3B3	-0.6941427	5.83590389	-4.3125687	4.91E-05	0.00029378	1.51412663	SF3B3	assignmentsChromaffin_cells.Chrom_SOX2_statusHigh
assignmentsChromaffin_cells.Chrom_SOX2_statusHigh.FSCN1	2.65532475	2.32682309	4.21907333	6.87E-05	0.00039279	1.51207809	FSCN1	assignmentsChromaffin_cells.Chrom_SOX2_statusHigh
assignmentsChromaffin_cells.Chrom_SOX2_statusHigh.TMED10	0.94130991	6.52755065	4.33019104	4.61E-05	0.00027887	1.51194138	TMED10	assignmentsChromaffin_cells.Chrom_SOX2_statusHigh
assignmentsChromaffin_cells.Chrom_SOX2_statusHigh.MBTD1	-0.7519999	6.35314482	-4.317633	4.82E-05	0.00028975	1.50974167	MBTD1	assignmentsChromaffin_cells.Chrom_SOX2_statusHigh
assignmentsChromaffin_cells.Chrom_SOX2_statusHigh.LRIG2	-0.8666481	5.23340607	-4.2981189	5.18E-05	0.00030744	1.50895225	LRIG2	assignmentsChromaffin_cells.Chrom_SOX2_statusHigh
assignmentsChromaffin_cells.Chrom_SOX2_statusHigh.RAB8A	1.3470471	4.0862527	4.29026978	5.32E-05	0.00031498	1.50716756	RAB8A	assignmentsChromaffin_cells.Chrom_SOX2_statusHigh
assignmentsChromaffin_cells.Chrom_SOX2_statusHigh.LINC01679	3.9929867	-0.0507789	4.21737564	6.92E-05	0.00039443	1.50601134	LINC01679	assignmentsChromaffin_cells.Chrom_SOX2_statusHigh
assignmentsChromaffin_cells.Chrom_SOX2_statusHigh.ARPC4	1.25398882	4.94748192	4.29905801	5.16E-05	0.00030663	1.50446316	ARPC4	assignmentsChromaffin_cells.Chrom_SOX2_statusHigh
assignmentsChromaffin_cells.Chrom_SOX2_statusHigh.NEFM	4.22146716	3.65900181	4.3141183	4.90E-05	0.00029304	1.50126587	NEFM	assignmentsChromaffin_cells.Chrom_SOX2_statusHigh
assignmentsChromaffin_cells.Chrom_SOX2_statusHigh.PDCD2	1.0060594	4.62413362	4.30041297	5.13E-05	0.0003055	1.49862729	PDCD2	assignmentsChromaffin_cells.Chrom_SOX2_statusHigh
assignmentsChromaffin_cells.Chrom_SOX2_statusHigh.HNRNPA1	1.62990891	6.77932579	4.32922805	4.63E-05	0.00027995	1.49594856	HNRNPA1	assignmentsChromaffin_cells.Chrom_SOX2_statusHigh
assignmentsChromaffin_cells.Chrom_SOX2_statusHigh.HSPB7	3.73920551	0.32175118	4.20915245	7.14E-05	0.00040409	1.49540845	HSPB7	assignmentsChromaffin_cells.Chrom_SOX2_statusHigh
assignmentsChromaffin_cells.Chrom_SOX2_statusHigh.EIF4A1	1.81634071	6.89537849	4.32604703	4.69E-05	0.00028302	1.4948658	EIF4A1	assignmentsChromaffin_cells.Chrom_SOX2_statusHigh
assignmentsChromaffin_cells.Chrom_SOX2_statusHigh.RPRD1B	-0.6897953	5.73026623	-4.3034661	5.08E-05	0.00030269	1.48931623	RPRD1B	assignmentsChromaffin_cells.Chrom_SOX2_statusHigh
assignmentsChromaffin_cells.Chrom_SOX2_statusHigh.KLHDC8B	1.45036059	3.64375009	4.28398731	5.45E-05	0.00032101	1.48210392	KLHDC8B	assignmentsChromaffin_cells.Chrom_SOX2_statusHigh
assignmentsChromaffin_cells.Chrom_SOX2_statusHigh.MAP2K4	-0.8393754	7.03612562	-4.3169887	4.84E-05	0.00029021	1.47586287	MAP2K4	assignmentsChromaffin_cells.Chrom_SOX2_statusHigh
assignmentsChromaffin_cells.Chrom_SOX2_statusHigh.PGR	2.87637227	0.63165752	4.19806365	7.41E-05	0.00041659	1.47298462	PGR	assignmentsChromaffin_cells.Chrom_SOX2_statusHigh
assignmentsChromaffin_cells.Chrom_SOX2_statusHigh.CCNB2	1.9795674	0.83170629	4.2179797	6.90E-05	0.00039383	1.47280139	CCNB2	assignmentsChromaffin_cells.Chrom_SOX2_statusHigh
assignmentsChromaffin_cells.Chrom_SOX2_statusHigh.ZNF337-AS1	-1.4359848	4.64689575	-4.2866662	5.39E-05	0.00031816	1.47054026	ZNF337-AS1	assignmentsChromaffin_cells.Chrom_SOX2_statusHigh
assignmentsChromaffin_cells.Chrom_SOX2_statusHigh.CXXC5	1.91293308	4.26288402	4.29585794	5.22E-05	0.00030976	1.47036001	CXXC5	assignmentsChromaffin_cells.Chrom_SOX2_statusHigh
assignmentsChromaffin_cells.Chrom_SOX2_statusHigh.PCIF1	0.80003238	3.49187059	4.27418107	5.64E-05	0.00033131	1.46733965	PCIF1	assignmentsChromaffin_cells.Chrom_SOX2_statusHigh
assignmentsChromaffin_cells.Chrom_SOX2_statusHigh.TRAM2-AS1	1.33175189	2.15108356	4.2544473	6.06E-05	0.00035216	1.46677079	TRAM2-AS1	assignmentsChromaffin_cells.Chrom_SOX2_statusHigh
assignmentsChromaffin_cells.Chrom_SOX2_statusHigh.PDLIM1	2.95393888	4.08602361	4.24483794	6.28E-05	0.00036359	1.46591737	PDLIM1	assignmentsChromaffin_cells.Chrom_SOX2_statusHigh
assignmentsChromaffin_cells.Chrom_SOX2_statusHigh.SLC9A1	1.40974161	4.06890113	4.27493324	5.63E-05	0.00033054	1.46559689	SLC9A1	assignmentsChromaffin_cells.Chrom_SOX2_statusHigh
assignmentsChromaffin_cells.Chrom_SOX2_statusHigh.PIAS2	-0.6898615	6.64324627	-4.3094611	4.97E-05	0.00029676	1.46477091	PIAS2	assignmentsChromaffin_cells.Chrom_SOX2_statusHigh
assignmentsChromaffin_cells.Chrom_SOX2_statusHigh.EEF1G	2.19235721	6.54105223	4.31759484	4.84E-05	0.00029025	1.46280154	EEF1G	assignmentsChromaffin_cells.Chrom_SOX2_statusHigh
assignmentsChromaffin_cells.Chrom_SOX2_statusHigh.TUBA1A	1.85753987	6.59685835	4.31977425	4.80E-05	0.00028851	1.45915054	TUBA1A	assignmentsChromaffin_cells.Chrom_SOX2_statusHigh
assignmentsChromaffin_cells.Chrom_SOX2_statusHigh.ERCC1	1.44093646	5.40131674	4.29283143	5.28E-05	0.00031243	1.45343853	ERCC1	assignmentsChromaffin_cells.Chrom_SOX2_statusHigh
assignmentsChromaffin_cells.Chrom_SOX2_statusHigh.CAVIN3	2.51140106	1.68423633	4.19890499	7.39E-05	0.00041549	1.45284255	CAVIN3	assignmentsChromaffin_cells.Chrom_SOX2_statusHigh
assignmentsChromaffin_cells.Chrom_SOX2_statusHigh.CISD2	1.19746527	4.86505605	4.29439216	5.25E-05	0.00031091	1.4516587	CISD2	assignmentsChromaffin_cells.Chrom_SOX2_statusHigh
assignmentsChromaffin_cells.Chrom_SOX2_statusHigh.TMEM100	3.47021688	1.15113044	4.22263346	6.80E-05	0.00038914	1.45116763	TMEM100	assignmentsChromaffin_cells.Chrom_SOX2_statusHigh
assignmentsChromaffin_cells.Chrom_SOX2_statusHigh.DUSP14	1.43856873	3.66972685	4.27535797	5.62E-05	0.00033028	1.45110351	DUSP14	assignmentsChromaffin_cells.Chrom_SOX2_statusHigh
assignmentsChromaffin_cells.Chrom_SOX2_statusHigh.AARS2	-0.950597	3.66562445	-4.2592219	5.95E-05	0.00034706	1.4502972	AARS2	assignmentsChromaffin_cells.Chrom_SOX2_statusHigh
assignmentsChromaffin_cells.Chrom_SOX2_statusHigh.BGN	2.6100067	2.91843164	4.22108514	6.83E-05	0.00039045	1.44811237	BGN	assignmentsChromaffin_cells.Chrom_SOX2_statusHigh
assignmentsChromaffin_cells.Chrom_SOX2_statusHigh.PRR14L	-0.6872676	6.14028642	-4.2935928	5.26E-05	0.00031169	1.44755589	PRR14L	assignmentsChromaffin_cells.Chrom_SOX2_statusHigh
assignmentsChromaffin_cells.Chrom_SOX2_statusHigh.NUDT9	1.00153826	5.05260495	4.29577795	5.22E-05	0.00030959	1.44326295	NUDT9	assignmentsChromaffin_cells.Chrom_SOX2_statusHigh
assignmentsChromaffin_cells.Chrom_SOX2_statusHigh.PRDX6	1.60481298	5.71463595	4.29928595	5.16E-05	0.00030663	1.44325582	PRDX6	assignmentsChromaffin_cells.Chrom_SOX2_statusHigh
assignmentsChromaffin_cells.Chrom_SOX2_statusHigh.TMEM161B	-0.8248774	5.63940738	-4.2913635	5.30E-05	0.00031398	1.44224588	TMEM161B	assignmentsChromaffin_cells.Chrom_SOX2_statusHigh
assignmentsChromaffin_cells.Chrom_SOX2_statusHigh.LMF1	-0.9600015	5.31654317	-4.2762007	5.60E-05	0.0003294	1.44201077	LMF1	assignmentsChromaffin_cells.Chrom_SOX2_statusHigh
assignmentsChromaffin_cells.Chrom_SOX2_statusHigh.RIC1	-0.8405373	7.11009205	-4.3014735	5.11E-05	0.00030454	1.44187404	RIC1	assignmentsChromaffin_cells.Chrom_SOX2_statusHigh
assignmentsChromaffin_cells.Chrom_SOX2_statusHigh.ARL4A	1.80468624	3.56185387	4.25584898	6.03E-05	0.00035078	1.43895393	ARL4A	assignmentsChromaffin_cells.Chrom_SOX2_statusHigh
assignmentsChromaffin_cells.Chrom_SOX2_statusHigh.TH	6.4196355	4.72848339	4.31475172	4.89E-05	0.00029265	1.43759076	TH	assignmentsChromaffin_cells.Chrom_SOX2_statusHigh
assignmentsChromaffin_cells.Chrom_SOX2_statusHigh.CHTF8	1.9945154	0.64023448	4.21006847	7.10E-05	0.00040225	1.43723546	CHTF8	assignmentsChromaffin_cells.Chrom_SOX2_statusHigh
assignmentsChromaffin_cells.Chrom_SOX2_statusHigh.RALGAPB	-0.9338932	6.77226433	-4.3028421	5.09E-05	0.00030326	1.43506425	RALGAPB	assignmentsChromaffin_cells.Chrom_SOX2_statusHigh
assignmentsChromaffin_cells.Chrom_SOX2_statusHigh.ZNF718	-1.0726095	5.60378574	-4.2835781	5.45E-05	0.00032136	1.43430172	ZNF718	assignmentsChromaffin_cells.Chrom_SOX2_statusHigh
assignmentsChromaffin_cells.Chrom_SOX2_statusHigh.ZSCAN30	-0.8766694	5.43227344	-4.2872136	5.38E-05	0.00031777	1.43325051	ZSCAN30	assignmentsChromaffin_cells.Chrom_SOX2_statusHigh
assignmentsChromaffin_cells.Chrom_SOX2_statusHigh.EI24	1.2901807	4.04092726	4.27499646	5.63E-05	0.00033054	1.42995751	EI24	assignmentsChromaffin_cells.Chrom_SOX2_statusHigh
assignmentsChromaffin_cells.Chrom_SOX2_statusHigh.CNPY4	1.09862393	2.82591075	4.25830116	5.97E-05	0.00034808	1.42911638	CNPY4	assignmentsChromaffin_cells.Chrom_SOX2_statusHigh
assignmentsChromaffin_cells.Chrom_SOX2_statusHigh.EIF4E3	-1.6647441	4.16439347	-4.2535875	6.07E-05	0.00035299	1.42607396	EIF4E3	assignmentsChromaffin_cells.Chrom_SOX2_statusHigh
assignmentsChromaffin_cells.Chrom_SOX2_statusHigh.FLNC	2.85586763	0.21077397	4.18374629	7.80E-05	0.00043456	1.42575551	FLNC	assignmentsChromaffin_cells.Chrom_SOX2_statusHigh
assignmentsChromaffin_cells.Chrom_SOX2_statusHigh.HAGH	0.95939849	5.42803838	4.29600851	5.22E-05	0.00030945	1.42559693	HAGH	assignmentsChromaffin_cells.Chrom_SOX2_statusHigh
assignmentsChromaffin_cells.Chrom_SOX2_statusHigh.CLHC1	-1.2376956	5.13079285	-4.2778276	5.57E-05	0.0003276	1.42448375	CLHC1	assignmentsChromaffin_cells.Chrom_SOX2_statusHigh

assignmentsChromaffin_cells.Chrom_SOX2_statusHigh.CD58	2.41566171	5.48622132	4.26223558	5.90E-05	0.00034447	1.42321038	CD58	assignmentsChromaffin_cells.Chrom_SOX2_statusHigh
assignmentsChromaffin_cells.Chrom_SOX2_statusHigh.MSANTD2	-1.0706096	5.23126377	-4.2725658	5.67E-05	0.000333	1.42269146	MSANTD2	assignmentsChromaffin_cells.Chrom_SOX2_statusHigh
assignmentsChromaffin_cells.Chrom_SOX2_statusHigh.FOXJ3	-0.7327867	7.02672274	-4.2909836	5.31E-05	0.00031429	1.41500802	FOXJ3	assignmentsChromaffin_cells.Chrom_SOX2_statusHigh
assignmentsChromaffin_cells.Chrom_SOX2_statusHigh.MAGIX	1.97004656	1.01594941	4.21315205	7.02E-05	0.00039897	1.41377309	MAGIX	assignmentsChromaffin_cells.Chrom_SOX2_statusHigh
assignmentsChromaffin_cells.Chrom_SOX2_statusHigh.FAM222B	-1.0409759	6.53134607	-4.2878531	5.37E-05	0.00031715	1.40318006	FAM222B	assignmentsChromaffin_cells.Chrom_SOX2_statusHigh
assignmentsChromaffin_cells.Chrom_SOX2_statusHigh.IFIT1	2.04265402	3.52811836	4.25660548	6.02E-05	0.00035033	1.40190057	IFIT1	assignmentsChromaffin_cells.Chrom_SOX2_statusHigh
assignmentsChromaffin_cells.Chrom_SOX2_statusHigh.HMMR	1.53721583	1.07451196	4.20317447	7.27E-05	0.00040994	1.40091746	HMMR	assignmentsChromaffin_cells.Chrom_SOX2_statusHigh
assignmentsChromaffin_cells.Chrom_SOX2_statusHigh.FAM162A	1.30852832	4.61658156	4.27229164	5.68E-05	0.00033321	1.40087568	FAM162A	assignmentsChromaffin_cells.Chrom_SOX2_statusHigh
assignmentsChromaffin_cells.Chrom_SOX2_statusHigh.ARHGAP5-A	1.84949387	1.04117104	4.21615363	6.95E-05	0.0003957	1.40055198	ARHGAP5-AS	assignmentsChromaffin_cells.Chrom_SOX2_statusHigh
assignmentsChromaffin_cells.Chrom_SOX2_statusHigh.TYW5	-0.8629294	4.27467597	-4.2547236	6.05E-05	0.00035194	1.39980536	TYW5	assignmentsChromaffin_cells.Chrom_SOX2_statusHigh
assignmentsChromaffin_cells.Chrom_SOX2_statusHigh.DHTKD1	-1.1435276	4.27157747	-4.2542396	6.06E-05	0.0003523	1.39866811	DHTKD1	assignmentsChromaffin_cells.Chrom_SOX2_statusHigh
assignmentsChromaffin_cells.Chrom_SOX2_statusHigh.SAC3D1	1.64466533	1.98645113	4.23235175	6.55E-05	0.00037707	1.39766794	SAC3D1	assignmentsChromaffin_cells.Chrom_SOX2_statusHigh
assignmentsChromaffin_cells.Chrom_SOX2_statusHigh.ALKBH1	-1.0146432	4.66419934	-4.2532827	6.08E-05	0.00035325	1.39395485	ALKBH1	assignmentsChromaffin_cells.Chrom_SOX2_statusHigh
assignmentsChromaffin_cells.Chrom_SOX2_statusHigh.ZNF552	-1.0246461	3.70230353	-4.2401006	6.38E-05	0.00036821	1.39144014	ZNF552	assignmentsChromaffin_cells.Chrom_SOX2_statusHigh
assignmentsChromaffin_cells.Chrom_SOX2_statusHigh.WWC2	-2.5790098	5.93596408	-4.236316	6.48E-05	0.00037337	1.38424408	WWC2	assignmentsChromaffin_cells.Chrom_SOX2_statusHigh
assignmentsChromaffin_cells.Chrom_SOX2_statusHigh.QSOX1	0.95062594	4.70398144	4.2643451	5.84E-05	0.00034166	1.38269224	QSOX1	assignmentsChromaffin_cells.Chrom_SOX2_statusHigh
assignmentsChromaffin_cells.Chrom_SOX2_statusHigh.BCL10	1.48587813	3.50933779	4.23770605	6.43E-05	0.00037097	1.38151825	BCL10	assignmentsChromaffin_cells.Chrom_SOX2_statusHigh
assignmentsChromaffin_cells.Chrom_SOX2_statusHigh.TICAM2	1.17967509	3.28165867	4.23474713	6.50E-05	0.00037412	1.38096029	TICAM2	assignmentsChromaffin_cells.Chrom_SOX2_statusHigh
assignmentsChromaffin_cells.Chrom_SOX2_statusHigh.YY1AP1	-0.6576088	6.17081018	-4.2780832	5.56E-05	0.00032742	1.37222626	YY1AP1	assignmentsChromaffin_cells.Chrom_SOX2_statusHigh
assignmentsChromaffin_cells.Chrom_SOX2_statusHigh.PMS1	-1.0268574	6.01408142	-4.2709873	5.71E-05	0.00033453	1.37104806	PMS1	assignmentsChromaffin_cells.Chrom_SOX2_statusHigh
assignmentsChromaffin_cells.Chrom_SOX2_statusHigh.DEPP1	3.45255872	2.36050469	4.20354036	7.28E-05	0.00041027	1.36763319	DEPP1	assignmentsChromaffin_cells.Chrom_SOX2_statusHigh
assignmentsChromaffin_cells.Chrom_SOX2_statusHigh.HAGLR	2.52992262	1.12488908	4.20467175	7.24E-05	0.00040847	1.36669295	HAGLR	assignmentsChromaffin_cells.Chrom_SOX2_statusHigh
assignmentsChromaffin_cells.Chrom_SOX2_statusHigh.ARPC1B	1.86829692	5.47074184	4.2408367	6.36E-05	0.00036764	1.35005938	ARPC1B	assignmentsChromaffin_cells.Chrom_SOX2_statusHigh
assignmentsChromaffin_cells.Chrom_SOX2_statusHigh.TBCA	0.86371822	7.03642347	4.28833879	5.36E-05	0.00031671	1.34898241	TBCA	assignmentsChromaffin_cells.Chrom_SOX2_statusHigh
assignmentsChromaffin_cells.Chrom_SOX2_statusHigh.CYYR1-AS1	-2.0150807	2.31397844	-4.2107915	7.08E-05	0.00040181	1.34811023	CYYR1-AS1	assignmentsChromaffin_cells.Chrom_SOX2_statusHigh
assignmentsChromaffin_cells.Chrom_SOX2_statusHigh.SOX4	2.30044948	5.22226503	4.26373972	5.87E-05	0.00034299	1.34094967	SOX4	assignmentsChromaffin_cells.Chrom_SOX2_statusHigh
assignmentsChromaffin_cells.Chrom_SOX2_statusHigh.ZNF347	-1.1012775	4.10589497	-4.2294723	6.62E-05	0.00038042	1.34066529	ZNF347	assignmentsChromaffin_cells.Chrom_SOX2_statusHigh
assignmentsChromaffin_cells.Chrom_SOX2_statusHigh.KATNBL1	-0.7057029	6.69698494	-4.2693179	5.74E-05	0.00033642	1.33572007	KATNBL1	assignmentsChromaffin_cells.Chrom_SOX2_statusHigh
assignmentsChromaffin_cells.Chrom_SOX2_statusHigh.KIAA1671	-1.435211	5.27519812	-4.2414619	6.34E-05	0.00036668	1.3349449	KIAA1671	assignmentsChromaffin_cells.Chrom_SOX2_statusHigh
assignmentsChromaffin_cells.Chrom_SOX2_statusHigh.SNHG32	1.94789739	4.50887651	4.25746754	6.00E-05	0.00034962	1.33442614	SNHG32	assignmentsChromaffin_cells.Chrom_SOX2_statusHigh
assignmentsChromaffin_cells.Chrom_SOX2_statusHigh.TMEM126B	1.07630553	4.23218679	4.24857316	6.18E-05	0.00035849	1.3323497	TMEM126B	assignmentsChromaffin_cells.Chrom_SOX2_statusHigh
assignmentsChromaffin_cells.Chrom_SOX2_statusHigh.ARHGAP31-	2.23865072	0.51926698	4.15373866	8.67E-05	0.00047709	1.33021831	ARHGAP31-A	assignmentsChromaffin_cells.Chrom_SOX2_statusHigh
assignmentsChromaffin_cells.Chrom_SOX2_statusHigh.ZNF230	1.1529993	2.13549681	4.21288778	7.03E-05	0.00039921	1.32995544	ZNF230	assignmentsChromaffin_cells.Chrom_SOX2_statusHigh
assignmentsChromaffin_cells.Chrom_SOX2_statusHigh.CLIP1-AS1	-2.705822	0.09133719	-4.1540594	8.66E-05	0.00047671	1.32696154	CLIP1-AS1	assignmentsChromaffin_cells.Chrom_SOX2_statusHigh
assignmentsChromaffin_cells.Chrom_SOX2_statusHigh.SLC31A2	1.52972801	3.13254565	4.21875261	6.88E-05	0.00039303	1.32584999	SLC31A2	assignmentsChromaffin_cells.Chrom_SOX2_statusHigh
assignmentsChromaffin_cells.Chrom_SOX2_statusHigh.MRPL37	0.95869482	3.25135481	4.2238715	6.76E-05	0.00038659	1.31966773	MRPL37	assignmentsChromaffin_cells.Chrom_SOX2_statusHigh
assignmentsChromaffin_cells.Chrom_SOX2_statusHigh.CD81-AS1	-2.250249	2.43342359	-4.1836714	7.80E-05	0.00043463	1.31325304	CD81-AS1	assignmentsChromaffin_cells.Chrom_SOX2_statusHigh
assignmentsChromaffin_cells.Chrom_SOX2_statusHigh.CLOCK	-0.595052	6.8218479	-4.2711442	5.70E-05	0.00033446	1.31257773	CLOCK	assignmentsChromaffin_cells.Chrom_SOX2_statusHigh
assignmentsChromaffin_cells.Chrom_SOX2_statusHigh.HDAC2	0.80348942	5.75428991	4.26008112	5.93E-05	0.00034612	1.31025153	HDAC2	assignmentsChromaffin_cells.Chrom_SOX2_statusHigh
assignmentsChromaffin_cells.Chrom_SOX2_statusHigh.SLC25A13	-0.8046642	7.22234295	-4.2686504	5.75E-05	0.0003371	1.30788074	SLC25A13	assignmentsChromaffin_cells.Chrom_SOX2_statusHigh
assignmentsChromaffin_cells.Chrom_SOX2_statusHigh.HIVEP3	-1.3844373	6.70549955	-4.247753	6.20E-05	0.00035915	1.30637555	HIVEP3	assignmentsChromaffin_cells.Chrom_SOX2_statusHigh
assignmentsChromaffin_cells.Chrom_SOX2_statusHigh.TENM3-AS1	2.69806683	1.63517902	4.20879575	7.15E-05	0.0004045	1.30539515	TENM3-AS1	assignmentsChromaffin_cells.Chrom_SOX2_statusHigh
assignmentsChromaffin_cells.Chrom_SOX2_statusHigh.SLC16A14	-3.5481343	1.27269844	-4.1435353	9.00E-05	0.0004916	1.30367987	SLC16A14	assignmentsChromaffin_cells.Chrom_SOX2_statusHigh
assignmentsChromaffin_cells.Chrom_SOX2_statusHigh.RNF216	-0.6127881	7.21890655	-4.2684724	5.76E-05	0.00033719	1.301402	RNF216	assignmentsChromaffin_cells.Chrom_SOX2_statusHigh
assignmentsChromaffin_cells.Chrom_SOX2_statusHigh.PFKFB3	-1.8922001	6.45785965	-4.2140155	7.00E-05	0.00039831	1.2953668	PFKFB3	assignmentsChromaffin_cells.Chrom_SOX2_statusHigh
assignmentsChromaffin_cells.Chrom_SOX2_statusHigh.TPT1	3.17990973	7.98175111	4.28483769	5.44E-05	0.00032099	1.29463304	TPT1	assignmentsChromaffin_cells.Chrom_SOX2_statusHigh
assignmentsChromaffin_cells.Chrom_SOX2_statusHigh.ATXN7	-1.0936155	6.6289617	-4.2480805	6.20E-05	0.00035887	1.2940412	ATXN7	assignmentsChromaffin_cells.Chrom_SOX2_statusHigh
assignmentsChromaffin_cells.Chrom_SOX2_statusHigh.CMPK2	1.62405187	2.54448196	4.19512931	7.49E-05	0.00042009	1.2928691	CMPK2	assignmentsChromaffin_cells.Chrom_SOX2_statusHigh
assignmentsChromaffin_cells.Chrom_SOX2_statusHigh.UNC13B	-1.1878086	6.3057061	-4.2508807	6.13E-05	0.00035579	1.28721265	UNC13B	assignmentsChromaffin_cells.Chrom_SOX2_statusHigh
assignmentsChromaffin_cells.Chrom_SOX2_statusHigh.PIGB	-1.0814755	4.55208632	-4.2251602	6.73E-05	0.00038523	1.28652245	PIGB	assignmentsChromaffin_cells.Chrom_SOX2_statusHigh
assignmentsChromaffin_cells.Chrom_SOX2_statusHigh.ACVRL1	1.97033843	1.79949524	4.14383591	8.98E-05	0.00049097	1.28618299	ACVRL1	assignmentsChromaffin_cells.Chrom_SOX2_statusHigh
assignmentsChromaffin_cells.Chrom_SOX2_statusHigh.VGF	2.8106401	1.8906133	4.21928734	6.89E-05	0.00039312	1.28614499	VGF	assignmentsChromaffin_cells.Chrom_SOX2_statusHigh
assignmentsChromaffin_cells.Chrom_SOX2_statusHigh.USP32	-0.8120342	7.46660983	-4.2652957	5.82E-05	0.00034082	1.28500932	USP32	assignmentsChromaffin_cells.Chrom_SOX2_statusHigh
assignmentsChromaffin_cells.Chrom_SOX2_statusHigh.ZNF573	-0.9380146	4.51762517	-4.2269964	6.68E-05	0.00038312	1.28470734	ZNF573	assignmentsChromaffin_cells.Chrom_SOX2_statusHigh
assignmentsChromaffin_cells.Chrom_SOX2_statusHigh.OGT	-0.8958795	7.09985946	-4.2552187	6.04E-05	0.00035144	1.28242432	OGT	assignmentsChromaffin_cells.Chrom_SOX2_statusHigh
assignmentsChromaffin_cells.Chrom_SOX2_statusHigh.L2HGDH	-0.9191961	4.39974799	-4.2291501	6.63E-05	0.00038072	1.28134315	L2HGDH	assignmentsChromaffin_cells.Chrom_SOX2_statusHigh
assignmentsChromaffin_cells.Chrom_SOX2_statusHigh.RC3H2	-0.8775866	6.48560966	-4.2480772	6.20E-05	0.00035887	1.28023331	RC3H2	assignmentsChromaffin_cells.Chrom_SOX2_statusHigh
assignmentsChromaffin_cells.Chrom_SOX2_statusHigh.ITPK1-AS1	-2.6778764	0.28181666	-4.1362394	9.23E-05	0.00050341	1.27984168	ITPK1-AS1	assignmentsChromaffin_cells.Chrom_SOX2_statusHigh
assignmentsChromaffin_cells.Chrom_SOX2_statusHigh.CLIP3	1.01782361	3.32175061	4.23062415	6.60E-05	0.00037899	1.27954807	CLIP3	assignmentsChromaffin_cells.Chrom_SOX2_statusHigh
assignmentsChromaffin_cells.Chrom_SOX2_statusHigh.HMGB3	1.99178719	1.02038289	4.16435233	8.35E-05	0.00046121	1.27773247	HMGB3	assignmentsChromaffin_cells.Chrom_SOX2_statusHigh
assignmentsChromaffin_cells.Chrom_SOX2_statusHigh.CNOT4	-0.6909143	7.54305957	-4.2634298	5.86E-05	0.00034261	1.27009498	CNOT4	assignmentsChromaffin_cells.Chrom_SOX2_statusHigh
assignmentsChromaffin_cells.Chrom_SOX2_statusHigh.CHD6	-0.7455367	7.6100605	-4.2647588	5.84E-05	0.00034135	1.2688555	CHD6	assignmentsChromaffin_cells.Chrom_SOX2_statusHigh
assignmentsChromaffin_cells.Chrom_SOX2_statusHigh.DHRSX	-0.989843	6.92230182	-4.2507333	6.14E-05	0.00035585	1.268391	DHRSX	assignmentsChromaffin_cells.Chrom_SOX2_statusHigh
assignmentsChromaffin_cells.Chrom_SOX2_statusHigh.KANTR	-0.7730683	6.45821038	-4.2531468	6.08E-05	0.0003533	1.26288286	KANTR	assignmentsChromaffin_cells.Chrom_SOX2_statusHigh

assignmentsChromaffin_cells.Chrom_SOX2_statusHigh.ATP6V1D	0.84802603	5.58146986	4.24697587	6.22E-05	0.00036003	1.26093252	ATP6V1D	assignmentsChromaffin_cells.Chrom_SOX2_statusHigh
assignmentsChromaffin_cells.Chrom_SOX2_statusHigh.CDK18	3.08145929	1.8251516	4.14828317	8.85E-05	0.00048535	1.2603143	CDK18	assignmentsChromaffin_cells.Chrom_SOX2_statusHigh
assignmentsChromaffin_cells.Chrom_SOX2_statusHigh.ANKIB1	-0.7139582	7.60292377	-4.2627616	5.88E-05	0.00034309	1.25958602	ANKIB1	assignmentsChromaffin_cells.Chrom_SOX2_statusHigh
assignmentsChromaffin_cells.Chrom_SOX2_statusHigh.ULBP2	2.48086391	-0.0390789	4.12986461	9.44E-05	0.00051278	1.25351095	ULBP2	assignmentsChromaffin_cells.Chrom_SOX2_statusHigh
assignmentsChromaffin_cells.Chrom_SOX2_statusHigh.TMEM161A	0.99536624	3.61550505	4.21441931	6.99E-05	0.00039802	1.2513864	TMEM161A	assignmentsChromaffin_cells.Chrom_SOX2_statusHigh
assignmentsChromaffin_cells.Chrom_SOX2_statusHigh.EXOSC1	0.79167956	4.68395587	4.2306445	6.59E-05	0.00037899	1.25100076	EXOSC1	assignmentsChromaffin_cells.Chrom_SOX2_statusHigh
assignmentsChromaffin_cells.Chrom_SOX2_statusHigh.MCHR1	2.6292366	-0.0787536	4.14617372	8.91E-05	0.00048769	1.24881986	MCHR1	assignmentsChromaffin_cells.Chrom_SOX2_statusHigh
assignmentsChromaffin_cells.Chrom_SOX2_statusHigh.COLEC12	-3.5350519	4.24348589	-4.1835512	7.82E-05	0.00043532	1.24725553	COLEC12	assignmentsChromaffin_cells.Chrom_SOX2_statusHigh
assignmentsChromaffin_cells.Chrom_SOX2_statusHigh.RNASEH2C	1.37220435	4.50405813	4.22536366	6.72E-05	0.00038509	1.24471065	RNASEH2C	assignmentsChromaffin_cells.Chrom_SOX2_statusHigh
assignmentsChromaffin_cells.Chrom_SOX2_statusHigh.VPS50	-0.9157905	6.3859524	-4.2420213	6.33E-05	0.00036608	1.2438793	VPS50	assignmentsChromaffin_cells.Chrom_SOX2_statusHigh
assignmentsChromaffin_cells.Chrom_SOX2_statusHigh.OCIAD2	1.67178581	4.32759036	4.23923863	6.40E-05	0.00036943	1.24291515	OCIAD2	assignmentsChromaffin_cells.Chrom_SOX2_statusHigh
assignmentsChromaffin_cells.Chrom_SOX2_statusHigh.CYREN	1.21074011	4.22839711	4.20488392	7.23E-05	0.00040817	1.24076248	CYREN	assignmentsChromaffin_cells.Chrom_SOX2_statusHigh
assignmentsChromaffin_cells.Chrom_SOX2_statusHigh.FBXO28	-0.6634493	6.00381417	-4.2357429	6.48E-05	0.00037332	1.24058937	FBXO28	assignmentsChromaffin_cells.Chrom_SOX2_statusHigh
assignmentsChromaffin_cells.Chrom_SOX2_statusHigh.MC1R	-1.4834112	3.43429473	-4.1953543	7.48E-05	0.0004199	1.23924498	MC1R	assignmentsChromaffin_cells.Chrom_SOX2_statusHigh
assignmentsChromaffin_cells.Chrom_SOX2_statusHigh.ZNF44	-1.2576898	5.8356271	-4.2242902	6.75E-05	0.00038629	1.23862689	ZNF44	assignmentsChromaffin_cells.Chrom_SOX2_statusHigh
assignmentsChromaffin_cells.Chrom_SOX2_statusHigh.CAP1	1.25623074	6.0175745	4.22666384	6.69E-05	0.00038344	1.23758586	CAP1	assignmentsChromaffin_cells.Chrom_SOX2_statusHigh
assignmentsChromaffin_cells.Chrom_SOX2_statusHigh.SNHG12	1.35898865	4.21222507	4.20766809	7.16E-05	0.00040472	1.23709061	SNHG12	assignmentsChromaffin_cells.Chrom_SOX2_statusHigh
assignmentsChromaffin_cells.Chrom_SOX2_statusHigh.ELP5	1.29959932	3.61228397	4.20765867	7.16E-05	0.00040472	1.23583957	ELP5	assignmentsChromaffin_cells.Chrom_SOX2_statusHigh
assignmentsChromaffin_cells.Chrom_SOX2_statusHigh.CETN3	0.99205131	3.52575013	4.21140077	7.06E-05	0.00040091	1.23488384	CETN3	assignmentsChromaffin_cells.Chrom_SOX2_statusHigh
assignmentsChromaffin_cells.Chrom_SOX2_statusHigh.MUL1	0.95404572	2.63879774	4.18363985	7.80E-05	0.00043457	1.23212605	MUL1	assignmentsChromaffin_cells.Chrom_SOX2_statusHigh
assignmentsChromaffin_cells.Chrom_SOX2_statusHigh.PODXL2	2.3651559	2.94731622	4.21344977	7.03E-05	0.00039926	1.22768171	PODXL2	assignmentsChromaffin_cells.Chrom_SOX2_statusHigh
assignmentsChromaffin_cells.Chrom_SOX2_statusHigh.LINC00511	1.9848568	2.86617648	4.1822045	7.84E-05	0.00043648	1.22746484	LINC00511	assignmentsChromaffin_cells.Chrom_SOX2_statusHigh
assignmentsChromaffin_cells.Chrom_SOX2_statusHigh.RERE	-1.0737561	8.65146448	-4.2524434	6.10E-05	0.00035393	1.21939852	RERE	assignmentsChromaffin_cells.Chrom_SOX2_statusHigh
assignmentsChromaffin_cells.Chrom_SOX2_statusHigh.RBL1	-1.0997205	4.82745772	-4.2088364	7.13E-05	0.00040373	1.21907606	RBL1	assignmentsChromaffin_cells.Chrom_SOX2_statusHigh
assignmentsChromaffin_cells.Chrom_SOX2_statusHigh.AUH	-0.9614825	6.51193506	-4.2313189	6.58E-05	0.00037833	1.2188775	AUH	assignmentsChromaffin_cells.Chrom_SOX2_statusHigh
assignmentsChromaffin_cells.Chrom_SOX2_statusHigh.MED31	1.21110005	3.84640832	4.21045651	7.09E-05	0.00040197	1.21617752	MED31	assignmentsChromaffin_cells.Chrom_SOX2_statusHigh
assignmentsChromaffin_cells.Chrom_SOX2_statusHigh.ZFPM2-AS1	-2.2889744	3.66561483	-4.1695195	8.21E-05	0.00045439	1.21402626	ZFPM2-AS1	assignmentsChromaffin_cells.Chrom_SOX2_statusHigh
assignmentsChromaffin_cells.Chrom_SOX2_statusHigh.CIAO2A	0.7624705	4.54783492	4.21617195	6.94E-05	0.0003957	1.21249628	CIAO2A	assignmentsChromaffin_cells.Chrom_SOX2_statusHigh
assignmentsChromaffin_cells.Chrom_SOX2_statusHigh.ATP2B1-AS1	1.41968147	3.71809667	4.2052882	7.22E-05	0.00040772	1.20978619	ATP2B1-AS1	assignmentsChromaffin_cells.Chrom_SOX2_statusHigh
assignmentsChromaffin_cells.Chrom_SOX2_statusHigh.MAP1A	1.32280964	5.17448027	4.23533363	6.49E-05	0.00037347	1.20875972	MAP1A	assignmentsChromaffin_cells.Chrom_SOX2_statusHigh
assignmentsChromaffin_cells.Chrom_SOX2_statusHigh.PANO1	1.63251782	0.58585264	4.14642614	8.90E-05	0.00048709	1.20868806	PANO1	assignmentsChromaffin_cells.Chrom_SOX2_statusHigh
assignmentsChromaffin_cells.Chrom_SOX2_statusHigh.SCAF8	-0.9478145	7.27302668	-4.2367218	6.45E-05	0.00037215	1.2082211	SCAF8	assignmentsChromaffin_cells.Chrom_SOX2_statusHigh
assignmentsChromaffin_cells.Chrom_SOX2_statusHigh.SFMBT1	-0.910848	5.70974425	-4.2175686	6.91E-05	0.00039413	1.20682077	SFMBT1	assignmentsChromaffin_cells.Chrom_SOX2_statusHigh
assignmentsChromaffin_cells.Chrom_SOX2_statusHigh.AAK1	-0.8344954	7.92556111	-4.2425215	6.32E-05	0.00036555	1.20118925	AAK1	assignmentsChromaffin_cells.Chrom_SOX2_statusHigh
assignmentsChromaffin_cells.Chrom_SOX2_statusHigh.NEMP2	-2.3677798	2.66604164	-4.1191863	9.80E-05	0.00053031	1.19825099	NEMP2	assignmentsChromaffin_cells.Chrom_SOX2_statusHigh
assignmentsChromaffin_cells.Chrom_SOX2_statusHigh.DNAJC3	0.75998523	6.9000988	4.23955319	6.39E-05	0.00036879	1.19545856	DNAJC3	assignmentsChromaffin_cells.Chrom_SOX2_statusHigh
assignmentsChromaffin_cells.Chrom_SOX2_statusHigh.EBF4	-1.3745216	4.44745936	-4.1993364	7.37E-05	0.000415	1.19298842	EBF4	assignmentsChromaffin_cells.Chrom_SOX2_statusHigh
assignmentsChromaffin_cells.Chrom_SOX2_statusHigh.PAM16	1.0014875	4.69020073	4.21140274	7.06E-05	0.00040091	1.19146773	PAM16	assignmentsChromaffin_cells.Chrom_SOX2_statusHigh
assignmentsChromaffin_cells.Chrom_SOX2_statusHigh.NOLC1	1.20637983	4.63505532	4.21018923	7.09E-05	0.00040221	1.19123111	NOLC1	assignmentsChromaffin_cells.Chrom_SOX2_statusHigh
assignmentsChromaffin_cells.Chrom_SOX2_statusHigh.DYRK1B	0.95107102	2.88585912	4.19006336	7.62E-05	0.00042624	1.18761151	DYRK1B	assignmentsChromaffin_cells.Chrom_SOX2_statusHigh
assignmentsChromaffin_cells.Chrom_SOX2_statusHigh.CASK	-0.9696598	7.22264656	-4.2287416	6.64E-05	0.00038114	1.18540566	CASK	assignmentsChromaffin_cells.Chrom_SOX2_statusHigh
assignmentsChromaffin_cells.Chrom_SOX2_statusHigh.FAM149A	-1.5304801	2.70648742	-4.1577767	8.55E-05	0.00047079	1.18222301	FAM149A	assignmentsChromaffin_cells.Chrom_SOX2_statusHigh
assignmentsChromaffin_cells.Chrom_SOX2_statusHigh.SOCS1	3.07786634	1.3865552	4.10658182	0.00010249	0.00055194	1.17834925	SOCS1	assignmentsChromaffin_cells.Chrom_SOX2_statusHigh
assignmentsChromaffin_cells.Chrom_SOX2_statusHigh.SLFNL1-AS1	-1.4516508	2.68585318	-4.1625131	8.41E-05	0.00046376	1.17685917	SLFNL1-AS1	assignmentsChromaffin_cells.Chrom_SOX2_statusHigh
assignmentsChromaffin_cells.Chrom_SOX2_statusHigh.PLB1	2.12466962	2.24625715	4.1470691	8.88E-05	0.00048652	1.17637851	PLB1	assignmentsChromaffin_cells.Chrom_SOX2_statusHigh
assignmentsChromaffin_cells.Chrom_SOX2_statusHigh.PARN	-0.8598825	6.24383229	-4.2140254	7.00E-05	0.0003983	1.17409309	PARN	assignmentsChromaffin_cells.Chrom_SOX2_statusHigh
assignmentsChromaffin_cells.Chrom_SOX2_statusHigh.MRPL2	1.08360746	3.34930389	4.18744693	7.69E-05	0.00042977	1.17234792	MRPL2	assignmentsChromaffin_cells.Chrom_SOX2_statusHigh
assignmentsChromaffin_cells.Chrom_SOX2_statusHigh.DTWD2	-1.3927671	4.71485977	-4.1920365	7.57E-05	0.00042429	1.16444001	DTWD2	assignmentsChromaffin_cells.Chrom_SOX2_statusHigh
assignmentsChromaffin_cells.Chrom_SOX2_statusHigh.SDC4	2.17778165	2.52490006	4.16254008	8.41E-05	0.00046401	1.16348523	SDC4	assignmentsChromaffin_cells.Chrom_SOX2_statusHigh
assignmentsChromaffin_cells.Chrom_SOX2_statusHigh.GHET1	2.09002385	-0.2037607	4.10443987	0.0001032	0.0005554	1.16268651	GHET1	assignmentsChromaffin_cells.Chrom_SOX2_statusHigh
assignmentsChromaffin_cells.Chrom_SOX2_statusHigh.ALMS1	-0.9031061	6.38061163	-4.2177251	6.91E-05	0.00039405	1.16157066	ALMS1	assignmentsChromaffin_cells.Chrom_SOX2_statusHigh
assignmentsChromaffin_cells.Chrom_SOX2_statusHigh.LAMB4	2.09615859	0.89034839	4.13495084	9.27E-05	0.00050554	1.15974783	LAMB4	assignmentsChromaffin_cells.Chrom_SOX2_statusHigh
assignmentsChromaffin_cells.Chrom_SOX2_statusHigh.ACTR3B	-0.764416	5.04859631	-4.2045982	7.24E-05	0.00040829	1.15890063	ACTR3B	assignmentsChromaffin_cells.Chrom_SOX2_statusHigh
assignmentsChromaffin_cells.Chrom_SOX2_statusHigh.WDR83	1.0185851	2.96856593	4.17945427	7.92E-05	0.00044017	1.15741642	WDR83	assignmentsChromaffin_cells.Chrom_SOX2_statusHigh
assignmentsChromaffin_cells.Chrom_SOX2_statusHigh.ZFAT	-0.8792333	5.11057246	-4.1915772	7.58E-05	0.00042484	1.15709821	ZFAT	assignmentsChromaffin_cells.Chrom_SOX2_statusHigh
assignmentsChromaffin_cells.Chrom_SOX2_statusHigh.CHMP4A	0.9821583	4.74792753	4.19622615	7.46E-05	0.00041889	1.15537344	CHMP4A	assignmentsChromaffin_cells.Chrom_SOX2_statusHigh
assignmentsChromaffin_cells.Chrom_SOX2_statusHigh.ARHGEF12	-0.9446038	7.76111953	-4.2281626	6.65E-05	0.00038166	1.15451604	ARHGEF12	assignmentsChromaffin_cells.Chrom_SOX2_statusHigh
assignmentsChromaffin_cells.Chrom_SOX2_statusHigh.GPN1	1.06925098	3.71593512	4.18332934	7.81E-05	0.00043475	1.15331931	GPN1	assignmentsChromaffin_cells.Chrom_SOX2_statusHigh
assignmentsChromaffin_cells.Chrom_SOX2_statusHigh.SMURF1	-0.9829139	6.90114593	-4.2080051	7.15E-05	0.0004045	1.14978858	SMURF1	assignmentsChromaffin_cells.Chrom_SOX2_statusHigh
assignmentsChromaffin_cells.Chrom_SOX2_statusHigh.KMT2A	-0.5938236	7.71649453	-4.2323481	6.55E-05	0.00037707	1.14915981	KMT2A	assignmentsChromaffin_cells.Chrom_SOX2_statusHigh
assignmentsChromaffin_cells.Chrom_SOX2_statusHigh.MAOB	3.27392312	0.35120884	4.09649215	0.0001062	0.00057021	1.14808314	MAOB	assignmentsChromaffin_cells.Chrom_SOX2_statusHigh
assignmentsChromaffin_cells.Chrom_SOX2_statusHigh.NAA15	-0.7308577	6.73142358	-4.2138659	7.00E-05	0.00039831	1.13555131	NAA15	assignmentsChromaffin_cells.Chrom_SOX2_statusHigh
assignmentsChromaffin_cells.Chrom_SOX2_statusHigh.ADK	-0.9433879	8.20273496	-4.2283832	6.65E-05	0.0003815	1.13114968	ADK	assignmentsChromaffin_cells.Chrom_SOX2_statusHigh
assignmentsChromaffin_cells.Chrom_SOX2_statusHigh.METTL5	1.00068233	4.63676095	4.19362687	7.53E-05	0.00042219	1.1268544	METTL5	assignmentsChromaffin_cells.Chrom_SOX2_statusHigh

assignmentsChromaffin_cells.Chrom_SOX2_statusHigh.LDHA	2.08320092	6.24344572	4.21495521	6.99E-05	0.00039811	1.12660968	LDHA	assignmentsChromaffin_cells.Chrom_SOX2_statusHigh
assignmentsChromaffin_cells.Chrom_SOX2_statusHigh.ZNF781	-1.8272797	1.77454307	-4.1116786	0.0001006	0.00054289	1.12568956	ZNF781	assignmentsChromaffin_cells.Chrom_SOX2_statusHigh
assignmentsChromaffin_cells.Chrom_SOX2_statusHigh.TRIM33	-0.9661227	6.87718359	-4.205561	7.21E-05	0.00040747	1.12462114	TRIM33	assignmentsChromaffin_cells.Chrom_SOX2_statusHigh
assignmentsChromaffin_cells.Chrom_SOX2_statusHigh.ZMIZ1-AS1	-1.3406225	3.16728627	-4.1523056	8.72E-05	0.00047902	1.11730665	ZMIZ1-AS1	assignmentsChromaffin_cells.Chrom_SOX2_statusHigh
assignmentsChromaffin_cells.Chrom_SOX2_statusHigh.DTNBP1	-0.9225216	6.22540665	-4.1957845	7.47E-05	0.0004194	1.1157116	DTNBP1	assignmentsChromaffin_cells.Chrom_SOX2_statusHigh
assignmentsChromaffin_cells.Chrom_SOX2_statusHigh.TFPT	1.44152515	2.38568511	4.1486479	8.83E-05	0.0004846	1.11428356	TFPT	assignmentsChromaffin_cells.Chrom_SOX2_statusHigh
assignmentsChromaffin_cells.Chrom_SOX2_statusHigh.PEX2	0.94461939	4.73753572	4.19099819	7.60E-05	0.00042528	1.11229932	PEX2	assignmentsChromaffin_cells.Chrom_SOX2_statusHigh
assignmentsChromaffin_cells.Chrom_SOX2_statusHigh.FUT9	-2.7672531	3.96742162	-4.1756883	8.04E-05	0.0004461	1.11213388	FUT9	assignmentsChromaffin_cells.Chrom_SOX2_statusHigh
assignmentsChromaffin_cells.Chrom_SOX2_statusHigh.TTC13	-1.2090242	5.79949659	-4.1858799	7.74E-05	0.00043173	1.11019185	TTC13	assignmentsChromaffin_cells.Chrom_SOX2_statusHigh
assignmentsChromaffin_cells.Chrom_SOX2_statusHigh.ANP32E	1.2659337	4.9208475	4.19319442	7.54E-05	0.00042269	1.10909711	ANP32E	assignmentsChromaffin_cells.Chrom_SOX2_statusHigh
assignmentsChromaffin_cells.Chrom_SOX2_statusHigh.LYSMD3	0.9571243	4.9149389	4.19078368	7.60E-05	0.00042544	1.10670522	LYSMD3	assignmentsChromaffin_cells.Chrom_SOX2_statusHigh
assignmentsChromaffin_cells.Chrom_SOX2_statusHigh.PTPN2	-0.9647086	6.66703086	-4.1978222	7.41E-05	0.00041681	1.10446062	PTPN2	assignmentsChromaffin_cells.Chrom_SOX2_statusHigh
assignmentsChromaffin_cells.Chrom_SOX2_statusHigh.CNTNAP3C	-3.0544328	0.66858501	-4.0869453	0.00010983	0.00058619	1.10216444	CNTNAP3C	assignmentsChromaffin_cells.Chrom_SOX2_statusHigh
assignmentsChromaffin_cells.Chrom_SOX2_statusHigh.WBP11	1.02130292	5.67708212	4.20097889	7.33E-05	0.00041287	1.10158777	WBP11	assignmentsChromaffin_cells.Chrom_SOX2_statusHigh
assignmentsChromaffin_cells.Chrom_SOX2_statusHigh.CLPTM1	0.77738303	4.88197597	4.18594799	7.73E-05	0.00043173	1.09959647	CLPTM1	assignmentsChromaffin_cells.Chrom_SOX2_statusHigh
assignmentsChromaffin_cells.Chrom_SOX2_statusHigh.PAXBP1	-1.0869355	6.92890376	-4.2046571	7.24E-05	0.00040829	1.09939845	PAXBP1	assignmentsChromaffin_cells.Chrom_SOX2_statusHigh
assignmentsChromaffin_cells.Chrom_SOX2_statusHigh.DIRC3-AS1	2.76869266	1.44081776	4.09146604	0.00010809	0.00057899	1.09934712	DIRC3-AS1	assignmentsChromaffin_cells.Chrom_SOX2_statusHigh
assignmentsChromaffin_cells.Chrom_SOX2_statusHigh.GNE	-0.9289092	4.63562387	-4.1738784	8.07E-05	0.00044758	1.09816604	GNE	assignmentsChromaffin_cells.Chrom_SOX2_statusHigh
assignmentsChromaffin_cells.Chrom_SOX2_statusHigh.SNRPC	1.16299603	4.17422283	4.17214987	8.12E-05	0.00045018	1.08984869	SNRPC	assignmentsChromaffin_cells.Chrom_SOX2_statusHigh
assignmentsChromaffin_cells.Chrom_SOX2_statusHigh.KIAA1191	0.8556597	4.10001472	4.17139927	8.14E-05	0.00045122	1.08753773	KIAA1191	assignmentsChromaffin_cells.Chrom_SOX2_statusHigh
assignmentsChromaffin_cells.Chrom_SOX2_statusHigh.TOM1L2	-0.8255944	6.5118303	-4.1976582	7.42E-05	0.0004169	1.0852746	TOM1L2	assignmentsChromaffin_cells.Chrom_SOX2_statusHigh
assignmentsChromaffin_cells.Chrom_SOX2_statusHigh.CISH	1.8524295	0.93756581	4.08380651	0.00011096	0.00059064	1.08420476	CISH	assignmentsChromaffin_cells.Chrom_SOX2_statusHigh
assignmentsChromaffin_cells.Chrom_SOX2_statusHigh.FKBP4	2.199156	3.89000514	4.16916019	8.23E-05	0.00045545	1.08111559	FKBP4	assignmentsChromaffin_cells.Chrom_SOX2_statusHigh
assignmentsChromaffin_cells.Chrom_SOX2_statusHigh.CAMKK2	-1.0923213	4.56528524	-4.151053	8.75E-05	0.00048098	1.08023406	CAMKK2	assignmentsChromaffin_cells.Chrom_SOX2_statusHigh
assignmentsChromaffin_cells.Chrom_SOX2_statusHigh.NDUFA5	1.11016333	5.92105537	4.20415146	7.25E-05	0.00040866	1.07816	NDUFA5	assignmentsChromaffin_cells.Chrom_SOX2_statusHigh
assignmentsChromaffin_cells.Chrom_SOX2_statusHigh.ANAPC10	-0.7385745	6.19179318	-4.1909913	7.60E-05	0.00042528	1.07446684	ANAPC10	assignmentsChromaffin_cells.Chrom_SOX2_statusHigh
assignmentsChromaffin_cells.Chrom_SOX2_statusHigh.NUP153	-0.9875739	6.73734849	-4.1910511	7.60E-05	0.00042528	1.07257467	NUP153	assignmentsChromaffin_cells.Chrom_SOX2_statusHigh
assignmentsChromaffin_cells.Chrom_SOX2_statusHigh.KCNMA1-AS	-2.0982344	0.97756564	-4.0827249	0.00011138	0.0005921	1.07001931	KCNMA1-AS	assignmentsChromaffin_cells.Chrom_SOX2_statusHigh
assignmentsChromaffin_cells.Chrom_SOX2_statusHigh.DNAI1	-1.4379214	1.97956735	-4.1320414	9.36E-05	0.00050972	1.07001222	DNAI1	assignmentsChromaffin_cells.Chrom_SOX2_statusHigh
assignmentsChromaffin_cells.Chrom_SOX2_statusHigh.VPS41	-0.8790758	7.00130623	-4.19992	7.36E-05	0.00041428	1.06940392	VPS41	assignmentsChromaffin_cells.Chrom_SOX2_statusHigh
assignmentsChromaffin_cells.Chrom_SOX2_statusHigh.SMCHD1	-0.9025471	8.5473613	-4.206906	7.18E-05	0.00040566	1.06872689	SMCHD1	assignmentsChromaffin_cells.Chrom_SOX2_statusHigh
assignmentsChromaffin_cells.Chrom_SOX2_statusHigh.ITFG1	-0.6701144	8.14037877	-4.2098168	7.10E-05	0.00040247	1.06447633	ITFG1	assignmentsChromaffin_cells.Chrom_SOX2_statusHigh
assignmentsChromaffin_cells.Chrom_SOX2_statusHigh.YLPM1	-1.0035395	6.75433029	-4.1902584	7.62E-05	0.00042609	1.05918082	YLPM1	assignmentsChromaffin_cells.Chrom_SOX2_statusHigh
assignmentsChromaffin_cells.Chrom_SOX2_statusHigh.SDCBP	1.56963076	6.51543622	4.18779254	7.69E-05	0.0004297	1.05817125	SDCBP	assignmentsChromaffin_cells.Chrom_SOX2_statusHigh
assignmentsChromaffin_cells.Chrom_SOX2_statusHigh.PSMA6	0.79598054	5.9992665	4.18806783	7.68E-05	0.00042913	1.05775079	PSMA6	assignmentsChromaffin_cells.Chrom_SOX2_statusHigh
assignmentsChromaffin_cells.Chrom_SOX2_statusHigh.LINC02731	2.03681214	1.46974465	4.1199934	9.78E-05	0.00052929	1.05674736	LINC02731	assignmentsChromaffin_cells.Chrom_SOX2_statusHigh
assignmentsChromaffin_cells.Chrom_SOX2_statusHigh.APOL1	2.27643047	1.75343828	4.0685754	0.00011705	0.00061893	1.05519958	APOL1	assignmentsChromaffin_cells.Chrom_SOX2_statusHigh
assignmentsChromaffin_cells.Chrom_SOX2_statusHigh.WDR44	-0.7356047	6.06573543	-4.1750265	8.04E-05	0.0004461	1.05475853	WDR44	assignmentsChromaffin_cells.Chrom_SOX2_statusHigh
assignmentsChromaffin_cells.Chrom_SOX2_statusHigh.TMEM128	0.85375791	3.90303021	4.1609754	8.45E-05	0.00046597	1.05129466	TMEM128	assignmentsChromaffin_cells.Chrom_SOX2_statusHigh
assignmentsChromaffin_cells.Chrom_SOX2_statusHigh.TM2D3	0.66382217	5.2957388	4.1801085	7.90E-05	0.0004393	1.05015738	TM2D3	assignmentsChromaffin_cells.Chrom_SOX2_statusHigh
assignmentsChromaffin_cells.Chrom_SOX2_statusHigh.ARFIP1	-0.6438512	6.6023272	-4.1848728	7.76E-05	0.00043304	1.04596676	ARFIP1	assignmentsChromaffin_cells.Chrom_SOX2_statusHigh
assignmentsChromaffin_cells.Chrom_SOX2_statusHigh.SRSF2	1.09180399	6.3038864	4.18641226	7.72E-05	0.00043121	1.04453177	SRSF2	assignmentsChromaffin_cells.Chrom_SOX2_statusHigh
assignmentsChromaffin_cells.Chrom_SOX2_statusHigh.MAK	-2.1773456	4.05340886	-4.1349721	9.28E-05	0.00050589	1.04317017	MAK	assignmentsChromaffin_cells.Chrom_SOX2_statusHigh
assignmentsChromaffin_cells.Chrom_SOX2_statusHigh.LARS2-AS1	-3.0648434	0.20978817	-4.060133	0.00012056	0.00063459	1.03607699	LARS2-AS1	assignmentsChromaffin_cells.Chrom_SOX2_statusHigh
assignmentsChromaffin_cells.Chrom_SOX2_statusHigh.ADAMTS13	-1.5267577	2.37137639	-4.1201642	9.76E-05	0.00052885	1.03561078	ADAMTS13	assignmentsChromaffin_cells.Chrom_SOX2_statusHigh
assignmentsChromaffin_cells.Chrom_SOX2_statusHigh.PCGF3	-0.9085433	5.32603527	-4.1564186	8.59E-05	0.0004729	1.03427107	PCGF3	assignmentsChromaffin_cells.Chrom_SOX2_statusHigh
assignmentsChromaffin_cells.Chrom_SOX2_statusHigh.SGPP1	-1.1038395	5.2894822	-4.1481285	8.85E-05	0.00048526	1.03138599	SGPP1	assignmentsChromaffin_cells.Chrom_SOX2_statusHigh
assignmentsChromaffin_cells.Chrom_SOX2_statusHigh.EEF1A2	2.24087334	4.29876494	4.18551594	7.77E-05	0.00043304	1.02928283	EEF1A2	assignmentsChromaffin_cells.Chrom_SOX2_statusHigh
assignmentsChromaffin_cells.Chrom_SOX2_statusHigh.CRAMP1	-0.9315488	5.36406775	-4.1580764	8.54E-05	0.00047046	1.02779324	CRAMP1	assignmentsChromaffin_cells.Chrom_SOX2_statusHigh
assignmentsChromaffin_cells.Chrom_SOX2_statusHigh.CERS5	-1.0082373	5.40101492	-4.1598144	8.49E-05	0.00046773	1.02406896	CERS5	assignmentsChromaffin_cells.Chrom_SOX2_statusHigh
assignmentsChromaffin_cells.Chrom_SOX2_statusHigh.POLR3B	-0.7978923	4.55405495	-4.146798	8.89E-05	0.00048662	1.02232191	POLR3B	assignmentsChromaffin_cells.Chrom_SOX2_statusHigh
assignmentsChromaffin_cells.Chrom_SOX2_statusHigh.BCL2L13	-0.7962563	6.0652934	-4.1754476	8.03E-05	0.00044574	1.02194489	BCL2L13	assignmentsChromaffin_cells.Chrom_SOX2_statusHigh
assignmentsChromaffin_cells.Chrom_SOX2_statusHigh.PIN4	0.95922653	5.06033133	4.16625031	8.30E-05	0.00045859	1.01763397	PIN4	assignmentsChromaffin_cells.Chrom_SOX2_statusHigh
assignmentsChromaffin_cells.Chrom_SOX2_statusHigh.MCM2	1.31226425	0.83320696	4.08825344	0.00010924	0.00058401	1.0168497	MCM2	assignmentsChromaffin_cells.Chrom_SOX2_statusHigh
assignmentsChromaffin_cells.Chrom_SOX2_statusHigh.FBXO34	-1.4232339	7.17626684	-4.1762753	8.01E-05	0.00044486	1.01468631	FBXO34	assignmentsChromaffin_cells.Chrom_SOX2_statusHigh
assignmentsChromaffin_cells.Chrom_SOX2_statusHigh.IRF9	1.42550429	5.0920797	4.1534581	8.68E-05	0.0004774	1.01398849	IRF9	assignmentsChromaffin_cells.Chrom_SOX2_statusHigh
assignmentsChromaffin_cells.Chrom_SOX2_statusHigh.DNAAF2	0.92185243	3.58406659	4.14028859	9.09E-05	0.00049659	1.01329323	DNAAF2	assignmentsChromaffin_cells.Chrom_SOX2_statusHigh
assignmentsChromaffin_cells.Chrom_SOX2_statusHigh.TSGA10	-1.169175	4.7113339	-4.1487737	8.83E-05	0.00048455	1.01087364	TSGA10	assignmentsChromaffin_cells.Chrom_SOX2_statusHigh
assignmentsChromaffin_cells.Chrom_SOX2_statusHigh.KDELR3	1.90675837	1.04191038	4.07656136	0.00011382	0.00060364	1.00769571	KDELR3	assignmentsChromaffin_cells.Chrom_SOX2_statusHigh
assignmentsChromaffin_cells.Chrom_SOX2_statusHigh.DTX3L	1.19509407	4.26798442	4.13142653	9.38E-05	0.00051031	1.00398444	DTX3L	assignmentsChromaffin_cells.Chrom_SOX2_statusHigh
assignmentsChromaffin_cells.Chrom_SOX2_statusHigh.ERCC6L2	-1.0677315	6.95759748	-4.1768544	7.99E-05	0.0004441	1.00352317	ERCC6L2	assignmentsChromaffin_cells.Chrom_SOX2_statusHigh
assignmentsChromaffin_cells.Chrom_SOX2_statusHigh.TRMT61A	1.23580786	1.76355127	4.10048844	0.00010464	0.00056243	0.99947475	TRMT61A	assignmentsChromaffin_cells.Chrom_SOX2_statusHigh
assignmentsChromaffin_cells.Chrom_SOX2_statusHigh.SNX29	-1.0789799	7.53383513	-4.1671274	8.27E-05	0.00045732	0.99790175	SNX29	assignmentsChromaffin_cells.Chrom_SOX2_statusHigh
assignmentsChromaffin_cells.Chrom_SOX2_statusHigh.SCARF2	-1.7037777	1.95273545	-4.0613507	0.00012005	0.0006327	0.99767686	SCARF2	assignmentsChromaffin_cells.Chrom_SOX2_statusHigh

assignmentsChromaffin_cells.Chrom_SOX2_statusHigh.ERVK3-1	1.00474803	5.48488244	4.16250518	8.41E-05	0.00046376	0.99472656	ERVK3-1	assignmentsChromaffin_cells.Chrom_SOX2_statusHigh
assignmentsChromaffin_cells.Chrom_SOX2_statusHigh.RER1	1.19215637	4.90473942	4.14801409	8.85E-05	0.00048526	0.99360022	RER1	assignmentsChromaffin_cells.Chrom_SOX2_statusHigh
assignmentsChromaffin_cells.Chrom_SOX2_statusHigh.IGSF10	2.64759717	0.66592065	4.08983969	0.00010883	0.00058218	0.99180786	IGSF10	assignmentsChromaffin_cells.Chrom_SOX2_statusHigh
assignmentsChromaffin_cells.Chrom_SOX2_statusHigh.RICTOR	-0.777078	7.34357968	-4.1754238	8.03E-05	0.00044574	0.98509788	RICTOR	assignmentsChromaffin_cells.Chrom_SOX2_statusHigh
assignmentsChromaffin_cells.Chrom_SOX2_statusHigh.ABL2	-1.2304922	7.33775083	-4.1693001	8.21E-05	0.00045439	0.98438926	ABL2	assignmentsChromaffin_cells.Chrom_SOX2_statusHigh
assignmentsChromaffin_cells.Chrom_SOX2_statusHigh.NPRL2	1.04750135	3.19684743	4.12574644	9.57E-05	0.00051959	0.98343682	NPRL2	assignmentsChromaffin_cells.Chrom_SOX2_statusHigh
assignmentsChromaffin_cells.Chrom_SOX2_statusHigh.PISD	-0.7464932	5.21893219	-4.1479724	8.85E-05	0.00048526	0.98187634	PISD	assignmentsChromaffin_cells.Chrom_SOX2_statusHigh
assignmentsChromaffin_cells.Chrom_SOX2_statusHigh.PIK3R2	1.12231704	4.05328155	4.14978053	8.79E-05	0.00048299	0.98099807	PIK3R2	assignmentsChromaffin_cells.Chrom_SOX2_statusHigh
assignmentsChromaffin_cells.Chrom_SOX2_statusHigh.LMAN1	1.25303998	6.23284262	4.1741876	8.06E-05	0.00044724	0.97578659	LMAN1	assignmentsChromaffin_cells.Chrom_SOX2_statusHigh
assignmentsChromaffin_cells.Chrom_SOX2_statusHigh.MAGEE1	1.54694711	1.04363514	4.09484072	0.00010674	0.00057275	0.9707198	MAGEE1	assignmentsChromaffin_cells.Chrom_SOX2_statusHigh
assignmentsChromaffin_cells.Chrom_SOX2_statusHigh.TIAL1	-0.5417907	6.79241797	-4.1676514	8.25E-05	0.00045663	0.96721156	TIAL1	assignmentsChromaffin_cells.Chrom_SOX2_statusHigh
assignmentsChromaffin_cells.Chrom_SOX2_statusHigh.RPL10A	2.50088014	6.76062151	4.17673672	8.01E-05	0.00044505	0.96198739	RPL10A	assignmentsChromaffin_cells.Chrom_SOX2_statusHigh
assignmentsChromaffin_cells.Chrom_SOX2_statusHigh.DNAJB4	2.05257442	4.17499262	4.13194034	9.38E-05	0.00051031	0.9605185	DNAJB4	assignmentsChromaffin_cells.Chrom_SOX2_statusHigh
assignmentsChromaffin_cells.Chrom_SOX2_statusHigh.SCLY	-0.7662581	4.69967881	-4.1341843	9.29E-05	0.00050656	0.9604295	SCLY	assignmentsChromaffin_cells.Chrom_SOX2_statusHigh
assignmentsChromaffin_cells.Chrom_SOX2_statusHigh.CSRP2	2.13985036	2.2233734	4.08558225	0.00011034	0.00058793	0.95894393	CSRP2	assignmentsChromaffin_cells.Chrom_SOX2_statusHigh
assignmentsChromaffin_cells.Chrom_SOX2_statusHigh.PBRM1	-0.7520216	7.33421888	-4.1643855	8.35E-05	0.00046121	0.95631239	PBRM1	assignmentsChromaffin_cells.Chrom_SOX2_statusHigh
assignmentsChromaffin_cells.Chrom_SOX2_statusHigh.RAD51B	-1.113786	7.87264247	-4.1636808	8.37E-05	0.00046215	0.95596822	RAD51B	assignmentsChromaffin_cells.Chrom_SOX2_statusHigh
assignmentsChromaffin_cells.Chrom_SOX2_statusHigh.LINC00174	-0.9966574	5.56277775	-4.1438345	8.98E-05	0.00049097	0.9551019	LINC00174	assignmentsChromaffin_cells.Chrom_SOX2_statusHigh
assignmentsChromaffin_cells.Chrom_SOX2_statusHigh.ANKRD39	1.02752836	3.08246292	4.1168448	9.88E-05	0.00053453	0.95499085	ANKRD39	assignmentsChromaffin_cells.Chrom_SOX2_statusHigh
assignmentsChromaffin_cells.Chrom_SOX2_statusHigh.HOXC-AS2	3.17549846	-0.3845031	4.04794321	0.00012601	0.0006583	0.95252754	HOXC-AS2	assignmentsChromaffin_cells.Chrom_SOX2_statusHigh
assignmentsChromaffin_cells.Chrom_SOX2_statusHigh.DHFR2	1.30609699	2.0724511	4.09513018	0.00010663	0.00057236	0.95237619	DHFR2	assignmentsChromaffin_cells.Chrom_SOX2_statusHigh
assignmentsChromaffin_cells.Chrom_SOX2_statusHigh.GCGR	2.72600419	2.2998093	4.12428212	9.65E-05	0.00052314	0.94855345	GCGR	assignmentsChromaffin_cells.Chrom_SOX2_statusHigh
assignmentsChromaffin_cells.Chrom_SOX2_statusHigh.SGCA	2.99623672	-0.4253198	4.03929248	0.00012966	0.00067474	0.94373387	SGCA	assignmentsChromaffin_cells.Chrom_SOX2_statusHigh
assignmentsChromaffin_cells.Chrom_SOX2_statusHigh.HSPA6	5.46650463	3.02632601	4.08635368	0.00011022	0.00058768	0.94359592	HSPA6	assignmentsChromaffin_cells.Chrom_SOX2_statusHigh
assignmentsChromaffin_cells.Chrom_SOX2_statusHigh.E2F3	-1.2656398	5.68395561	-4.1257742	9.57E-05	0.00051959	0.94114486	E2F3	assignmentsChromaffin_cells.Chrom_SOX2_statusHigh
assignmentsChromaffin_cells.Chrom_SOX2_statusHigh.UBE2M	2.17574571	3.51208339	4.1283387	9.51E-05	0.00051658	0.93979931	UBE2M	assignmentsChromaffin_cells.Chrom_SOX2_statusHigh
assignmentsChromaffin_cells.Chrom_SOX2_statusHigh.ANAPC4	-0.8286631	5.45565112	-4.1330898	9.33E-05	0.00050835	0.93671129	ANAPC4	assignmentsChromaffin_cells.Chrom_SOX2_statusHigh
assignmentsChromaffin_cells.Chrom_SOX2_statusHigh.COPS8	1.08636348	5.01347294	4.14228655	9.03E-05	0.00049327	0.93038838	COPS8	assignmentsChromaffin_cells.Chrom_SOX2_statusHigh
assignmentsChromaffin_cells.Chrom_SOX2_statusHigh.CWC22	-0.5999243	5.65143446	-4.1384399	9.15E-05	0.00049968	0.9270871	CWC22	assignmentsChromaffin_cells.Chrom_SOX2_statusHigh
assignmentsChromaffin_cells.Chrom_SOX2_statusHigh.LRRC4B	-1.3565548	4.57188237	-4.1321859	9.36E-05	0.00050963	0.92515831	LRRC4B	assignmentsChromaffin_cells.Chrom_SOX2_statusHigh
assignmentsChromaffin_cells.Chrom_SOX2_statusHigh.MAML2	-2.0032628	9.07808003	-4.1538316	8.69E-05	0.00047774	0.92103376	MAML2	assignmentsChromaffin_cells.Chrom_SOX2_statusHigh
assignmentsChromaffin_cells.Chrom_SOX2_statusHigh.TRA2A	-0.7235609	7.92681105	-4.1652725	8.32E-05	0.00046003	0.91906443	TRA2A	assignmentsChromaffin_cells.Chrom_SOX2_statusHigh
assignmentsChromaffin_cells.Chrom_SOX2_statusHigh.DEDD2	1.64051521	3.7934182	4.1216323	9.72E-05	0.00052677	0.9147713	DEDD2	assignmentsChromaffin_cells.Chrom_SOX2_statusHigh
assignmentsChromaffin_cells.Chrom_SOX2_statusHigh.UNC50	0.86280762	4.8194808	4.1327234	9.34E-05	0.00050884	0.91454833	UNC50	assignmentsChromaffin_cells.Chrom_SOX2_statusHigh
assignmentsChromaffin_cells.Chrom_SOX2_statusHigh.RNASE4	2.46184133	0.68811768	4.03574439	0.00013128	0.00068204	0.91257667	RNASE4	assignmentsChromaffin_cells.Chrom_SOX2_statusHigh
assignmentsChromaffin_cells.Chrom_SOX2_statusHigh.SEPTIN9	2.76610714	5.72273782	4.1033984	0.00010381	0.00055834	0.91223682	SEPTIN9	assignmentsChromaffin_cells.Chrom_SOX2_statusHigh
assignmentsChromaffin_cells.Chrom_SOX2_statusHigh.UBAP1	-1.0015723	6.86061629	-4.1437959	8.98E-05	0.00049097	0.9050782	UBAP1	assignmentsChromaffin_cells.Chrom_SOX2_statusHigh
assignmentsChromaffin_cells.Chrom_SOX2_statusHigh.IPPK	-0.7499845	4.36387353	-4.1071342	0.00010222	0.00055072	0.90233502	IPPK	assignmentsChromaffin_cells.Chrom_SOX2_statusHigh
assignmentsChromaffin_cells.Chrom_SOX2_statusHigh.MRTFA-AS1	-3.1992262	0.71874032	-4.0165778	0.00014034	0.00072187	0.90204782	MRTFA-AS1	assignmentsChromaffin_cells.Chrom_SOX2_statusHigh
assignmentsChromaffin_cells.Chrom_SOX2_statusHigh.FLYWCH2	1.13673287	3.91798717	4.11660673	9.89E-05	0.0005348	0.89758147	FLYWCH2	assignmentsChromaffin_cells.Chrom_SOX2_statusHigh
assignmentsChromaffin_cells.Chrom_SOX2_statusHigh.BIRC7	3.2177188	-1.007617	4.02396745	0.00013678	0.00070558	0.89556032	BIRC7	assignmentsChromaffin_cells.Chrom_SOX2_statusHigh
assignmentsChromaffin_cells.Chrom_SOX2_statusHigh.RNASEH2A	2.24900379	0.68014929	4.04186343	0.00012851	0.00066937	0.88777837	RNASEH2A	assignmentsChromaffin_cells.Chrom_SOX2_statusHigh
assignmentsChromaffin_cells.Chrom_SOX2_statusHigh.DNAJC22	2.59733367	-0.3306133	4.01156757	0.00014281	0.00073128	0.88669019	DNAJC22	assignmentsChromaffin_cells.Chrom_SOX2_statusHigh
assignmentsChromaffin_cells.Chrom_SOX2_statusHigh.ACSS2	-0.9872569	5.0286973	-4.1050207	0.00010299	0.00055446	0.88459419	ACSS2	assignmentsChromaffin_cells.Chrom_SOX2_statusHigh
assignmentsChromaffin_cells.Chrom_SOX2_statusHigh.SERTM2	3.25342145	-0.0496829	4.02898469	0.0001347	0.0006962	0.87940405	SERTM2	assignmentsChromaffin_cells.Chrom_SOX2_statusHigh
assignmentsChromaffin_cells.Chrom_SOX2_statusHigh.MX1	2.7677943	4.35249422	4.06954726	0.00011691	0.00061861	0.87722432	MX1	assignmentsChromaffin_cells.Chrom_SOX2_statusHigh
assignmentsChromaffin_cells.Chrom_SOX2_statusHigh.SHOC1	-2.6965472	3.85564367	-4.0577353	0.00012184	0.00063964	0.87395178	SHOC1	assignmentsChromaffin_cells.Chrom_SOX2_statusHigh
assignmentsChromaffin_cells.Chrom_SOX2_statusHigh.ALKBH2	1.28414061	2.90565648	4.09035452	0.00010844	0.00058068	0.87241179	ALKBH2	assignmentsChromaffin_cells.Chrom_SOX2_statusHigh
assignmentsChromaffin_cells.Chrom_SOX2_statusHigh.TST	1.48795759	1.05901234	4.02923383	0.00013429	0.00069477	0.86577757	TST	assignmentsChromaffin_cells.Chrom_SOX2_statusHigh
assignmentsChromaffin_cells.Chrom_SOX2_statusHigh.EIF3D	1.00431376	5.23317842	4.12057002	9.75E-05	0.00052827	0.86559247	EIF3D	assignmentsChromaffin_cells.Chrom_SOX2_statusHigh
assignmentsChromaffin_cells.Chrom_SOX2_statusHigh.MMP24OS	1.03889986	5.53682132	4.12184167	9.71E-05	0.00052626	0.86456571	MMP24OS	assignmentsChromaffin_cells.Chrom_SOX2_statusHigh
assignmentsChromaffin_cells.Chrom_SOX2_statusHigh.PIGX	1.1281302	4.27192232	4.10007027	0.0001048	0.00056306	0.86212986	PIGX	assignmentsChromaffin_cells.Chrom_SOX2_statusHigh
assignmentsChromaffin_cells.Chrom_SOX2_statusHigh.B4GALT1-A:	2.27807418	-0.2955503	4.00029392	0.00014851	0.00075589	0.85634824	B4GALT1-AS	assignmentsChromaffin_cells.Chrom_SOX2_statusHigh
assignmentsChromaffin_cells.Chrom_SOX2_statusHigh.PSEN1	-0.9089117	6.29356951	-4.1152754	9.93E-05	0.00053677	0.85477417	PSEN1	assignmentsChromaffin_cells.Chrom_SOX2_statusHigh
assignmentsChromaffin_cells.Chrom_SOX2_statusHigh.HMGB2	1.51175645	4.76157727	4.1132487	0.00010006	0.00054013	0.85449531	HMGB2	assignmentsChromaffin_cells.Chrom_SOX2_statusHigh
assignmentsChromaffin_cells.Chrom_SOX2_statusHigh.ZNF691	1.15928655	2.04324394	4.05322201	0.00012351	0.00064669	0.85019376	ZNF691	assignmentsChromaffin_cells.Chrom_SOX2_statusHigh
assignmentsChromaffin_cells.Chrom_SOX2_statusHigh.PCDHB10	1.50607613	1.85139036	4.06880707	0.00011695	0.00061863	0.84978701	PCDHB10	assignmentsChromaffin_cells.Chrom_SOX2_statusHigh
assignmentsChromaffin_cells.Chrom_SOX2_statusHigh.PARD6G	-1.9405122	3.06981439	-4.0533723	0.00012344	0.00064665	0.84762843	PARD6G	assignmentsChromaffin_cells.Chrom_SOX2_statusHigh
assignmentsChromaffin_cells.Chrom_SOX2_statusHigh.ALAS1	1.11603109	3.81665446	4.08969038	0.00010869	0.00058165	0.84718819	ALAS1	assignmentsChromaffin_cells.Chrom_SOX2_statusHigh
assignmentsChromaffin_cells.Chrom_SOX2_statusHigh.MMP11	2.41844568	0.43391258	3.99685781	0.00015029	0.00076399	0.84582175	MMP11	assignmentsChromaffin_cells.Chrom_SOX2_statusHigh
assignmentsChromaffin_cells.Chrom_SOX2_statusHigh.PIP5K1A	-0.7481297	6.76731817	-4.1314526	9.38E-05	0.00051031	0.84557098	PIP5K1A	assignmentsChromaffin_cells.Chrom_SOX2_statusHigh
assignmentsChromaffin_cells.Chrom_SOX2_statusHigh.PRPF40B	-0.8237696	5.2018863	-4.1089734	0.00010157	0.00054753	0.84397507	PRPF40B	assignmentsChromaffin_cells.Chrom_SOX2_statusHigh
assignmentsChromaffin_cells.Chrom_SOX2_statusHigh.TTC27	-0.6652741	5.72746225	-4.1114638	0.00010068	0.00054311	0.84327039	TTC27	assignmentsChromaffin_cells.Chrom_SOX2_statusHigh
assignmentsChromaffin_cells.Chrom_SOX2_statusHigh.SUN1	-0.6854209	6.26135205	-4.1267894	9.54E-05	0.00051803	0.84308452	SUN1	assignmentsChromaffin_cells.Chrom_SOX2_statusHigh

assignmentsChromaffin_cells.Chrom_SOX2_statusHigh.HEATR5B	-0.7761855	6.49649497	-4.1239665	9.63E-05	0.00052268	0.84286781	HEATR5B	assignmentsChromaffin_cells.Chrom_SOX2_statusHigh
assignmentsChromaffin_cells.Chrom_SOX2_statusHigh.INA	1.68488834	3.51588558	4.11568742	9.93E-05	0.00053669	0.83909894	INA	assignmentsChromaffin_cells.Chrom_SOX2_statusHigh
assignmentsChromaffin_cells.Chrom_SOX2_statusHigh.BATF2	2.38806076	-0.047985	3.99434658	0.00015161	0.00076969	0.83818173	BATF2	assignmentsChromaffin_cells.Chrom_SOX2_statusHigh
assignmentsChromaffin_cells.Chrom_SOX2_statusHigh.PRCP	1.20985979	5.21031062	4.10788514	0.00010196	0.00054945	0.83092787	PRCP	assignmentsChromaffin_cells.Chrom_SOX2_statusHigh
assignmentsChromaffin_cells.Chrom_SOX2_statusHigh.EXD2	-0.9002176	4.23683853	-4.085473	0.00011031	0.00058793	0.82761874	EXD2	assignmentsChromaffin_cells.Chrom_SOX2_statusHigh
assignmentsChromaffin_cells.Chrom_SOX2_statusHigh.S100A10	3.06988526	4.85091208	4.08254267	0.0001117	0.00059322	0.82160984	S100A10	assignmentsChromaffin_cells.Chrom_SOX2_statusHigh
assignmentsChromaffin_cells.Chrom_SOX2_statusHigh.TMEM216	1.2348626	2.34519177	4.05895486	0.00012106	0.00063656	0.82141927	TMEM216	assignmentsChromaffin_cells.Chrom_SOX2_statusHigh
assignmentsChromaffin_cells.Chrom_SOX2_statusHigh.SLCO3A1	-1.7104408	7.27505259	-4.1037921	0.00010353	0.00055702	0.81887891	SLCO3A1	assignmentsChromaffin_cells.Chrom_SOX2_statusHigh
assignmentsChromaffin_cells.Chrom_SOX2_statusHigh.ATF7	-0.7700341	6.72509632	-4.1144012	9.96E-05	0.00053825	0.8116297	ATF7	assignmentsChromaffin_cells.Chrom_SOX2_statusHigh
assignmentsChromaffin_cells.Chrom_SOX2_statusHigh.CARF	-1.0573624	4.83686659	-4.08571	0.00011022	0.00058768	0.80991122	CARF	assignmentsChromaffin_cells.Chrom_SOX2_statusHigh
assignmentsChromaffin_cells.Chrom_SOX2_statusHigh.TMEM223	1.25170143	3.45016926	4.07779146	0.00011333	0.00060124	0.80963769	TMEM223	assignmentsChromaffin_cells.Chrom_SOX2_statusHigh
assignmentsChromaffin_cells.Chrom_SOX2_statusHigh.MLST8	1.29129753	2.96479548	4.07442728	0.00011467	0.00060777	0.80960364	MLST8	assignmentsChromaffin_cells.Chrom_SOX2_statusHigh
assignmentsChromaffin_cells.Chrom_SOX2_statusHigh.DBH	6.58781199	5.41879997	4.13069332	9.43E-05	0.00051265	0.80804839	DBH	assignmentsChromaffin_cells.Chrom_SOX2_statusHigh
assignmentsChromaffin_cells.Chrom_SOX2_statusHigh.ZWINT	1.83812159	0.90139498	4.02651542	0.00013557	0.00070027	0.80741867	ZWINT	assignmentsChromaffin_cells.Chrom_SOX2_statusHigh
assignmentsChromaffin_cells.Chrom_SOX2_statusHigh.RETREG2	1.05066711	3.9311306	4.08846992	0.00010916	0.00058376	0.80356218	RETREG2	assignmentsChromaffin_cells.Chrom_SOX2_statusHigh
assignmentsChromaffin_cells.Chrom_SOX2_statusHigh.EAF1	1.16103817	4.02909983	4.08234501	0.00011153	0.00059249	0.79968432	EAF1	assignmentsChromaffin_cells.Chrom_SOX2_statusHigh
assignmentsChromaffin_cells.Chrom_SOX2_statusHigh.ZNF274	-0.9517207	5.08434098	-4.0860849	0.00011008	0.0005873	0.79680495	ZNF274	assignmentsChromaffin_cells.Chrom_SOX2_statusHigh
assignmentsChromaffin_cells.Chrom_SOX2_statusHigh.RNF212	3.20663122	0.61554707	3.98776709	0.00015524	0.00078515	0.79679763	RNF212	assignmentsChromaffin_cells.Chrom_SOX2_statusHigh
assignmentsChromaffin_cells.Chrom_SOX2_statusHigh.PIK3C3	-0.6412634	6.71871429	-4.1141982	9.97E-05	0.00053845	0.7936752	PIK3C3	assignmentsChromaffin_cells.Chrom_SOX2_statusHigh
assignmentsChromaffin_cells.Chrom_SOX2_statusHigh.SLBP	1.09527276	4.89670503	4.0924805	0.00010763	0.00057694	0.79154953	SLBP	assignmentsChromaffin_cells.Chrom_SOX2_statusHigh
assignmentsChromaffin_cells.Chrom_SOX2_statusHigh.RAB34	3.57873116	2.42076419	4.02712285	0.00013557	0.00070027	0.78954245	RAB34	assignmentsChromaffin_cells.Chrom_SOX2_statusHigh
assignmentsChromaffin_cells.Chrom_SOX2_statusHigh.GPR156	1.86354845	0.9768557	4.01208852	0.00014255	0.00073054	0.7861198	GPR156	assignmentsChromaffin_cells.Chrom_SOX2_statusHigh
assignmentsChromaffin_cells.Chrom_SOX2_statusHigh.HSP90AB1	2.48413286	8.51652006	4.14786909	8.87E-05	0.00048626	0.78530659	HSP90AB1	assignmentsChromaffin_cells.Chrom_SOX2_statusHigh
assignmentsChromaffin_cells.Chrom_SOX2_statusHigh.PRICKLE3	2.14101987	1.31176048	3.9842452	0.00015701	0.00079285	0.78413041	PRICKLE3	assignmentsChromaffin_cells.Chrom_SOX2_statusHigh
assignmentsChromaffin_cells.Chrom_SOX2_statusHigh.VPS54	-0.9747385	7.26097137	-4.1154608	9.93E-05	0.00053669	0.78080879	VPS54	assignmentsChromaffin_cells.Chrom_SOX2_statusHigh
assignmentsChromaffin_cells.Chrom_SOX2_statusHigh.LINC01786	-1.4355606	0.94006603	-4.003458	0.00014689	0.00074858	0.77837713	LINC01786	assignmentsChromaffin_cells.Chrom_SOX2_statusHigh
assignmentsChromaffin_cells.Chrom_SOX2_statusHigh.HOPX	3.43655299	0.73195423	3.97480587	0.00016232	0.00081684	0.77645558	HOPX	assignmentsChromaffin_cells.Chrom_SOX2_statusHigh
assignmentsChromaffin_cells.Chrom_SOX2_statusHigh.C1QL2	4.38044018	-1.0261474	3.97424196	0.00016284	0.00081916	0.77452114	C1QL2	assignmentsChromaffin_cells.Chrom_SOX2_statusHigh
assignmentsChromaffin_cells.Chrom_SOX2_statusHigh.HOXC10	3.15160756	0.98472412	4.03415043	0.0001323	0.00068577	0.7730125	HOXC10	assignmentsChromaffin_cells.Chrom_SOX2_statusHigh
assignmentsChromaffin_cells.Chrom_SOX2_statusHigh.RBM22	0.96745931	4.87007497	4.08417087	0.00011082	0.00059009	0.7706662	RBM22	assignmentsChromaffin_cells.Chrom_SOX2_statusHigh
assignmentsChromaffin_cells.Chrom_SOX2_statusHigh.ARMC9	-1.2604419	6.12315322	-4.1005068	0.00010464	0.00056243	0.76957105	ARMC9	assignmentsChromaffin_cells.Chrom_SOX2_statusHigh
assignmentsChromaffin_cells.Chrom_SOX2_statusHigh.KPNA2	1.37900449	4.69228228	4.09022195	0.00010849	0.00058076	0.76923077	KPNA2	assignmentsChromaffin_cells.Chrom_SOX2_statusHigh
assignmentsChromaffin_cells.Chrom_SOX2_statusHigh.PARG	-0.7661306	6.05122732	-4.0971466	0.00010588	0.00056869	0.7666074	PARG	assignmentsChromaffin_cells.Chrom_SOX2_statusHigh
assignmentsChromaffin_cells.Chrom_SOX2_statusHigh.RTL6	1.34922792	1.61598632	4.03413765	0.00013202	0.00068476	0.76514716	RTL6	assignmentsChromaffin_cells.Chrom_SOX2_statusHigh
assignmentsChromaffin_cells.Chrom_SOX2_statusHigh.GAP43	2.65309209	0.85468636	4.0195208	0.00013915	0.00071668	0.7609327	GAP43	assignmentsChromaffin_cells.Chrom_SOX2_statusHigh
assignmentsChromaffin_cells.Chrom_SOX2_statusHigh.ESCO1	-0.6141822	6.01271467	-4.094351	0.00010693	0.00057335	0.7602456	ESCO1	assignmentsChromaffin_cells.Chrom_SOX2_statusHigh
assignmentsChromaffin_cells.Chrom_SOX2_statusHigh.ST6GALNAC	-3.4361009	5.76815463	-4.0126501	0.00014257	0.00073054	0.75995766	ST6GALNAC3	assignmentsChromaffin_cells.Chrom_SOX2_statusHigh
assignmentsChromaffin_cells.Chrom_SOX2_statusHigh.LARGE-AS1	-3.0745968	1.26030114	-3.9807639	0.00015905	0.00080242	0.75079374	LARGE-AS1	assignmentsChromaffin_cells.Chrom_SOX2_statusHigh
assignmentsChromaffin_cells.Chrom_SOX2_statusHigh.TCEA3	2.96439584	0.97538107	3.97894427	0.00016005	0.00080721	0.75054139	TCEA3	assignmentsChromaffin_cells.Chrom_SOX2_statusHigh
assignmentsChromaffin_cells.Chrom_SOX2_statusHigh.KCNMA1-AS	-2.2095883	1.58978558	-4.0153111	0.00014105	0.00072455	0.75043086	KCNMA1-AS1	assignmentsChromaffin_cells.Chrom_SOX2_statusHigh
assignmentsChromaffin_cells.Chrom_SOX2_statusHigh.SSTR2	2.38800562	3.49571555	4.07548299	0.0001145	0.00060708	0.74613011	SSTR2	assignmentsChromaffin_cells.Chrom_SOX2_statusHigh
assignmentsChromaffin_cells.Chrom_SOX2_statusHigh.TIGD6	0.97461063	1.98936946	4.03614404	0.0001311	0.00068131	0.7444081	TIGD6	assignmentsChromaffin_cells.Chrom_SOX2_statusHigh
assignmentsChromaffin_cells.Chrom_SOX2_statusHigh.SLC16A1-AS	-1.1343424	5.86984659	-4.082772	0.00011136	0.0005921	0.74318472	SLC16A1-AS1	assignmentsChromaffin_cells.Chrom_SOX2_statusHigh
assignmentsChromaffin_cells.Chrom_SOX2_statusHigh.CAMK2N1	2.98516608	3.32125395	4.06339746	0.00011945	0.00062978	0.73931455	CAMK2N1	assignmentsChromaffin_cells.Chrom_SOX2_statusHigh
assignmentsChromaffin_cells.Chrom_SOX2_statusHigh.CD99L2	-0.7791555	5.10819841	-4.0718544	0.00011571	0.00061287	0.73803711	CD99L2	assignmentsChromaffin_cells.Chrom_SOX2_statusHigh
assignmentsChromaffin_cells.Chrom_SOX2_statusHigh.CEP41	0.83675481	3.1788121	4.05420176	0.00012309	0.00064532	0.73519915	CEP41	assignmentsChromaffin_cells.Chrom_SOX2_statusHigh
assignmentsChromaffin_cells.Chrom_SOX2_statusHigh.SEC14L2	-1.4696187	3.17493282	-4.0352286	0.00013152	0.00068292	0.73325958	SEC14L2	assignmentsChromaffin_cells.Chrom_SOX2_statusHigh
assignmentsChromaffin_cells.Chrom_SOX2_statusHigh.TCEAL1	1.17261402	4.06294282	4.07153749	0.00011584	0.00061335	0.72677773	TCEAL1	assignmentsChromaffin_cells.Chrom_SOX2_statusHigh
assignmentsChromaffin_cells.Chrom_SOX2_statusHigh.HSD17B12	1.15146514	5.27413862	4.08416435	0.00011082	0.00059009	0.72229471	HSD17B12	assignmentsChromaffin_cells.Chrom_SOX2_statusHigh
assignmentsChromaffin_cells.Chrom_SOX2_statusHigh.WNT9B	2.49597685	-0.6000614	3.95722302	0.00017238	0.00085857	0.72054068	WNT9B	assignmentsChromaffin_cells.Chrom_SOX2_statusHigh
assignmentsChromaffin_cells.Chrom_SOX2_statusHigh.RPS18	2.88530345	7.32064247	4.11074597	0.00010117	0.00054556	0.71815119	RPS18	assignmentsChromaffin_cells.Chrom_SOX2_statusHigh
assignmentsChromaffin_cells.Chrom_SOX2_statusHigh.SH3D19	-1.1417622	6.39593329	-4.0836921	0.00011101	0.00059068	0.71671486	SH3D19	assignmentsChromaffin_cells.Chrom_SOX2_statusHigh
assignmentsChromaffin_cells.Chrom_SOX2_statusHigh.SMC4	0.89525293	5.82686717	4.07973165	0.00011256	0.00059741	0.71337455	SMC4	assignmentsChromaffin_cells.Chrom_SOX2_statusHigh
assignmentsChromaffin_cells.Chrom_SOX2_statusHigh.WDR33	-0.7670071	6.88812438	-4.087613	0.00010949	0.00058474	0.70994744	WDR33	assignmentsChromaffin_cells.Chrom_SOX2_statusHigh
assignmentsChromaffin_cells.Chrom_SOX2_statusHigh.ZNF346	-0.9195834	5.04185539	-4.0644094	0.00011877	0.00062678	0.70758491	ZNF346	assignmentsChromaffin_cells.Chrom_SOX2_statusHigh
assignmentsChromaffin_cells.Chrom_SOX2_statusHigh.ABCB7	-0.6783468	6.08430116	-4.079706	0.00011257	0.00059741	0.70485436	ABCB7	assignmentsChromaffin_cells.Chrom_SOX2_statusHigh
assignmentsChromaffin_cells.Chrom_SOX2_statusHigh.MRPL14	1.40797442	4.26761186	4.06461826	0.00011868	0.00062653	0.7045462	MRPL14	assignmentsChromaffin_cells.Chrom_SOX2_statusHigh
assignmentsChromaffin_cells.Chrom_SOX2_statusHigh.ACMSD	-3.1117302	0.4914432	-3.9534327	0.00017474	0.00086872	0.70448937	ACMSD	assignmentsChromaffin_cells.Chrom_SOX2_statusHigh
assignmentsChromaffin_cells.Chrom_SOX2_statusHigh.METTL8	-0.7586915	5.03006922	-4.0533355	0.00012346	0.00064665	0.70324186	METTL8	assignmentsChromaffin_cells.Chrom_SOX2_statusHigh
assignmentsChromaffin_cells.Chrom_SOX2_statusHigh.SPG7	-0.925049	6.67679886	-4.0825695	0.00011144	0.00059222	0.70279652	SPG7	assignmentsChromaffin_cells.Chrom_SOX2_statusHigh
assignmentsChromaffin_cells.Chrom_SOX2_statusHigh.METTL24	3.07028644	1.3404084	3.97768098	0.00016093	0.00081088	0.70070429	METTL24	assignmentsChromaffin_cells.Chrom_SOX2_statusHigh
assignmentsChromaffin_cells.Chrom_SOX2_statusHigh.C18orf25	-0.7686017	5.66481163	-4.0670164	0.00011769	0.00062211	0.70047286	C18orf25	assignmentsChromaffin_cells.Chrom_SOX2_statusHigh
assignmentsChromaffin_cells.Chrom_SOX2_statusHigh.TRPC4AP	-0.7481761	7.49072543	-4.0920797	0.00010778	0.00057756	0.70019973	TRPC4AP	assignmentsChromaffin_cells.Chrom_SOX2_statusHigh
assignmentsChromaffin_cells.Chrom_SOX2_statusHigh.C2CD4D-AS1	2.95724386	-0.133029	3.94932061	0.00017714	0.00087901	0.69966165	C2CD4D-AS1	assignmentsChromaffin_cells.Chrom_SOX2_statusHigh

assignmentsChromaffin_cells.Chrom_SOX2_statusHigh.SEC61A2	-0.9240052	4.60848024	-4.0565385	0.00012208	0.00064049	0.69755744	SEC61A2	assignmentsChromaffin_cells.Chrom_SOX2_statusHigh
assignmentsChromaffin_cells.Chrom_SOX2_statusHigh.MSX2	4.06636637	-0.4646468	3.94923381	0.00017734	0.00087975	0.69467586	MSX2	assignmentsChromaffin_cells.Chrom_SOX2_statusHigh
assignmentsChromaffin_cells.Chrom_SOX2_statusHigh.ANTXR1	-1.7858502	5.63374922	-4.0465349	0.00012653	0.00066013	0.69070226	ANTXR1	assignmentsChromaffin_cells.Chrom_SOX2_statusHigh
assignmentsChromaffin_cells.Chrom_SOX2_statusHigh.RPS29	2.36392615	6.48650375	4.08830896	0.00010947	0.00058474	0.68751947	RPS29	assignmentsChromaffin_cells.Chrom_SOX2_statusHigh
assignmentsChromaffin_cells.Chrom_SOX2_statusHigh.FUCA2	2.3896729	3.07627654	3.99824777	0.0001497	0.00076122	0.68610492	FUCA2	assignmentsChromaffin_cells.Chrom_SOX2_statusHigh
assignmentsChromaffin_cells.Chrom_SOX2_statusHigh.MFSD8	-0.9021329	5.35853019	-4.0640089	0.00011893	0.00062746	0.68561682	MFSD8	assignmentsChromaffin_cells.Chrom_SOX2_statusHigh
assignmentsChromaffin_cells.Chrom_SOX2_statusHigh.RAC3	1.75900611	1.03595667	4.00850565	0.00014434	0.00073745	0.68554752	RAC3	assignmentsChromaffin_cells.Chrom_SOX2_statusHigh
assignmentsChromaffin_cells.Chrom_SOX2_statusHigh.TSPAN9	-1.6705251	5.05523912	-4.0402832	0.00012927	0.00067313	0.68353987	TSPAN9	assignmentsChromaffin_cells.Chrom_SOX2_statusHigh
assignmentsChromaffin_cells.Chrom_SOX2_statusHigh.RNF138	-0.8443256	5.77290652	-4.0608121	0.00012027	0.00063368	0.68335344	RNF138	assignmentsChromaffin_cells.Chrom_SOX2_statusHigh
assignmentsChromaffin_cells.Chrom_SOX2_statusHigh.DCLK2	-1.194079	5.75736155	-4.0653389	0.00011838	0.00062557	0.68179544	DCLK2	assignmentsChromaffin_cells.Chrom_SOX2_statusHigh
assignmentsChromaffin_cells.Chrom_SOX2_statusHigh.HEBP1	2.48761754	3.15185492	4.01562453	0.00014111	0.00072455	0.6817434	HEBP1	assignmentsChromaffin_cells.Chrom_SOX2_statusHigh
assignmentsChromaffin_cells.Chrom_SOX2_statusHigh.PGRMC2	0.8943412	5.30344453	4.07368361	0.00011497	0.00060915	0.68054392	PGRMC2	assignmentsChromaffin_cells.Chrom_SOX2_statusHigh
assignmentsChromaffin_cells.Chrom_SOX2_statusHigh.TIMM8A	1.32698523	1.90406069	4.01688448	0.00014019	0.00072133	0.67649414	TIMM8A	assignmentsChromaffin_cells.Chrom_SOX2_statusHigh
assignmentsChromaffin_cells.Chrom_SOX2_statusHigh.ZNF41	-1.044169	4.15696633	-4.0338706	0.00013214	0.00068518	0.67570479	ZNF41	assignmentsChromaffin_cells.Chrom_SOX2_statusHigh
assignmentsChromaffin_cells.Chrom_SOX2_statusHigh.NR1H2	0.88912672	4.66924659	4.05738355	0.00012172	0.00063923	0.67508153	NR1H2	assignmentsChromaffin_cells.Chrom_SOX2_statusHigh
assignmentsChromaffin_cells.Chrom_SOX2_statusHigh.ST13	1.49235752	6.44844682	4.08734333	0.00010968	0.00058557	0.67481811	ST13	assignmentsChromaffin_cells.Chrom_SOX2_statusHigh
assignmentsChromaffin_cells.Chrom_SOX2_statusHigh.METRN	1.67512803	3.76215574	4.05119838	0.00012446	0.00065149	0.66805228	METRN	assignmentsChromaffin_cells.Chrom_SOX2_statusHigh
assignmentsChromaffin_cells.Chrom_SOX2_statusHigh.POT1	-0.7385534	6.01159908	-4.069296	0.00011675	0.00061798	0.66498067	POT1	assignmentsChromaffin_cells.Chrom_SOX2_statusHigh
assignmentsChromaffin_cells.Chrom_SOX2_statusHigh.SLC38A4	-2.4142989	0.90414308	-3.955572	0.00017336	0.00086268	0.66300623	SLC38A4	assignmentsChromaffin_cells.Chrom_SOX2_statusHigh
assignmentsChromaffin_cells.Chrom_SOX2_statusHigh.LINC01776	3.04349148	1.02415724	4.01183961	0.00014298	0.00073167	0.65816076	LINC01776	assignmentsChromaffin_cells.Chrom_SOX2_statusHigh
assignmentsChromaffin_cells.Chrom_SOX2_statusHigh.ATG9B	2.23542992	0.664032	3.97218216	0.00016376	0.00082279	0.65773528	ATG9B	assignmentsChromaffin_cells.Chrom_SOX2_statusHigh
assignmentsChromaffin_cells.Chrom_SOX2_statusHigh.SCAF4	-0.7932577	6.31751588	-4.0591672	0.00012097	0.00063629	0.65424995	SCAF4	assignmentsChromaffin_cells.Chrom_SOX2_statusHigh
assignmentsChromaffin_cells.Chrom_SOX2_statusHigh.APOL6	1.73337859	3.93964133	4.01430259	0.00014146	0.00072543	0.65413991	APOL6	assignmentsChromaffin_cells.Chrom_SOX2_statusHigh
assignmentsChromaffin_cells.Chrom_SOX2_statusHigh.MLX	1.16208116	4.20785093	4.03966656	0.0001295	0.00067408	0.65095473	MLX	assignmentsChromaffin_cells.Chrom_SOX2_statusHigh
assignmentsChromaffin_cells.Chrom_SOX2_statusHigh.ATRX	-0.5416647	8.63154823	-4.094391	0.00010691	0.00057335	0.650178	ATRX	assignmentsChromaffin_cells.Chrom_SOX2_statusHigh
assignmentsChromaffin_cells.Chrom_SOX2_statusHigh.MRPS16	0.84922313	4.62286523	4.0501912	0.00012482	0.00065294	0.64650769	MRPS16	assignmentsChromaffin_cells.Chrom_SOX2_statusHigh
assignmentsChromaffin_cells.Chrom_SOX2_statusHigh.NEPRO	0.7614703	4.71979495	4.0423638	0.00012828	0.00066842	0.64474455	NEPRO	assignmentsChromaffin_cells.Chrom_SOX2_statusHigh
assignmentsChromaffin_cells.Chrom_SOX2_statusHigh.HOXB6	3.85837115	1.33282893	3.97151802	0.00016441	0.00082556	0.63979605	HOXB6	assignmentsChromaffin_cells.Chrom_SOX2_statusHigh
assignmentsChromaffin_cells.Chrom_SOX2_statusHigh.SELENOK	2.23196812	5.51214519	4.06567146	0.00011851	0.00062602	0.63714674	SELENOK	assignmentsChromaffin_cells.Chrom_SOX2_statusHigh
assignmentsChromaffin_cells.Chrom_SOX2_statusHigh.PHYHIP	2.42899602	1.49309389	3.99121227	0.0001535	0.0007776	0.63507839	PHYHIP	assignmentsChromaffin_cells.Chrom_SOX2_statusHigh
assignmentsChromaffin_cells.Chrom_SOX2_statusHigh.ANO10	-0.8843823	6.5579698	-4.0601203	0.00012056	0.00063459	0.63369401	ANO10	assignmentsChromaffin_cells.Chrom_SOX2_statusHigh
assignmentsChromaffin_cells.Chrom_SOX2_statusHigh.VPS13B	-0.7726434	8.58438465	-4.0834835	0.00011109	0.00059091	0.63274124	VPS13B	assignmentsChromaffin_cells.Chrom_SOX2_statusHigh
assignmentsChromaffin_cells.Chrom_SOX2_statusHigh.VPS35L	-0.8176827	6.38119334	-4.0573977	0.00012172	0.00063923	0.6309888	VPS35L	assignmentsChromaffin_cells.Chrom_SOX2_statusHigh
assignmentsChromaffin_cells.Chrom_SOX2_statusHigh.SERPINB8	1.67893984	2.06455065	3.98635478	0.00015586	0.00078782	0.62908362	SERPINB8	assignmentsChromaffin_cells.Chrom_SOX2_statusHigh
assignmentsChromaffin_cells.Chrom_SOX2_statusHigh.C8orf82	1.93159664	2.51280789	4.00790726	0.00014474	0.00073928	0.6275208	C8orf82	assignmentsChromaffin_cells.Chrom_SOX2_statusHigh
assignmentsChromaffin_cells.Chrom_SOX2_statusHigh.ATP1A3	1.47191944	4.75127052	4.06361703	0.00011917	0.00062851	0.62698009	ATP1A3	assignmentsChromaffin_cells.Chrom_SOX2_statusHigh
assignmentsChromaffin_cells.Chrom_SOX2_statusHigh.ATG7	-1.0374967	7.59780347	-4.0604211	0.00012044	0.00063434	0.62610448	ATG7	assignmentsChromaffin_cells.Chrom_SOX2_statusHigh
assignmentsChromaffin_cells.Chrom_SOX2_statusHigh.SMC3	0.75702952	5.85150256	4.06002461	0.0001206	0.0006346	0.6233513	SMC3	assignmentsChromaffin_cells.Chrom_SOX2_statusHigh
assignmentsChromaffin_cells.Chrom_SOX2_statusHigh.SLC46A1	1.03334523	2.24999436	4.00569049	0.00014575	0.00074328	0.6170812	SLC46A1	assignmentsChromaffin_cells.Chrom_SOX2_statusHigh
assignmentsChromaffin_cells.Chrom_SOX2_statusHigh.PHF19	1.67622011	3.26718748	4.00599014	0.0001456	0.00074298	0.61643606	PHF19	assignmentsChromaffin_cells.Chrom_SOX2_statusHigh
assignmentsChromaffin_cells.Chrom_SOX2_statusHigh.TLL1	-3.0743905	4.9806251	-4.0326556	0.00013299	0.00068913	0.61452837	TLL1	assignmentsChromaffin_cells.Chrom_SOX2_statusHigh
assignmentsChromaffin_cells.Chrom_SOX2_statusHigh.WDR26	-0.8370074	6.99095819	-4.0584126	0.00012129	0.00063756	0.61408919	WDR26	assignmentsChromaffin_cells.Chrom_SOX2_statusHigh
assignmentsChromaffin_cells.Chrom_SOX2_statusHigh.SPG11	-0.8556786	6.81478581	-4.0544264	0.00012299	0.00064503	0.61320729	SPG11	assignmentsChromaffin_cells.Chrom_SOX2_statusHigh
assignmentsChromaffin_cells.Chrom_SOX2_statusHigh.CMC4	2.19604428	1.64002218	3.98301636	0.00015781	0.00079642	0.61220259	CMC4	assignmentsChromaffin_cells.Chrom_SOX2_statusHigh
assignmentsChromaffin_cells.Chrom_SOX2_statusHigh.DNASE1	-0.9580127	5.04422782	-4.0344948	0.00013185	0.00068435	0.61057991	DNASE1	assignmentsChromaffin_cells.Chrom_SOX2_statusHigh
assignmentsChromaffin_cells.Chrom_SOX2_statusHigh.TCAIM	-0.9758979	5.933718	-4.0466572	0.00012637	0.00065954	0.60959388	TCAIM	assignmentsChromaffin_cells.Chrom_SOX2_statusHigh
assignmentsChromaffin_cells.Chrom_SOX2_statusHigh.SMIM27	1.320966	2.72658714	4.0117592	0.00014271	0.00073102	0.60952252	SMIM27	assignmentsChromaffin_cells.Chrom_SOX2_statusHigh
assignmentsChromaffin_cells.Chrom_SOX2_statusHigh.SHLD2	-0.7861735	6.24169147	-4.0468828	0.00012627	0.00065924	0.60897304	SHLD2	assignmentsChromaffin_cells.Chrom_SOX2_statusHigh
assignmentsChromaffin_cells.Chrom_SOX2_statusHigh.AK6	0.90259939	3.99176234	4.02925591	0.00013428	0.00069477	0.60763601	AK6	assignmentsChromaffin_cells.Chrom_SOX2_statusHigh
assignmentsChromaffin_cells.Chrom_SOX2_statusHigh.DGCR8	-1.1111824	4.70695824	-4.0244004	0.00013657	0.00070475	0.60759329	DGCR8	assignmentsChromaffin_cells.Chrom_SOX2_statusHigh
assignmentsChromaffin_cells.Chrom_SOX2_statusHigh.ZNF654	-0.7742498	6.50934294	-4.0501891	0.00012482	0.00065294	0.60742658	ZNF654	assignmentsChromaffin_cells.Chrom_SOX2_statusHigh
assignmentsChromaffin_cells.Chrom_SOX2_statusHigh.UBE2S	2.24685481	4.04332762	4.03215555	0.00013322	0.00069011	0.6049085	UBE2S	assignmentsChromaffin_cells.Chrom_SOX2_statusHigh
assignmentsChromaffin_cells.Chrom_SOX2_statusHigh.TRADD	1.05257112	3.37035649	3.99954751	0.0001489	0.00075737	0.60367961	TRADD	assignmentsChromaffin_cells.Chrom_SOX2_statusHigh
assignmentsChromaffin_cells.Chrom_SOX2_statusHigh.ADNP	-0.7537711	7.07010996	-4.0576063	0.00012163	0.00063915	0.60140462	ADNP	assignmentsChromaffin_cells.Chrom_SOX2_statusHigh
assignmentsChromaffin_cells.Chrom_SOX2_statusHigh.PPP3CC	-0.6985514	6.92877876	-4.0499452	0.00012493	0.00065307	0.60039836	PPP3CC	assignmentsChromaffin_cells.Chrom_SOX2_statusHigh
assignmentsChromaffin_cells.Chrom_SOX2_statusHigh.PDLIM3	3.38790814	4.34344954	4.02260779	0.00013772	0.00071022	0.59991504	PDLIM3	assignmentsChromaffin_cells.Chrom_SOX2_statusHigh
assignmentsChromaffin_cells.Chrom_SOX2_statusHigh.JMJD1C	-0.8821696	9.85951676	-4.0879003	0.00010938	0.00058454	0.5994728	JMJD1C	assignmentsChromaffin_cells.Chrom_SOX2_statusHigh
assignmentsChromaffin_cells.Chrom_SOX2_statusHigh.TCEAL6	4.53661372	-0.2951926	3.92624883	0.00019215	0.00094272	0.5985641	TCEAL6	assignmentsChromaffin_cells.Chrom_SOX2_statusHigh
assignmentsChromaffin_cells.Chrom_SOX2_statusHigh.HMOX1	2.98344711	2.88545443	3.97835229	0.00016057	0.00080958	0.59721902	HMOX1	assignmentsChromaffin_cells.Chrom_SOX2_statusHigh
assignmentsChromaffin_cells.Chrom_SOX2_statusHigh.EIF3E	1.16677215	6.87639161	4.06481589	0.0001186	0.0006263	0.59687889	EIF3E	assignmentsChromaffin_cells.Chrom_SOX2_statusHigh
assignmentsChromaffin_cells.Chrom_SOX2_statusHigh.LINC00886	2.62310986	2.76047491	3.96784242	0.00016645	0.00083266	0.59678374	LINC00886	assignmentsChromaffin_cells.Chrom_SOX2_statusHigh
assignmentsChromaffin_cells.Chrom_SOX2_statusHigh.RNF38	-0.9783922	7.01505505	-4.0473435	0.00012607	0.00065839	0.59074732	RNF38	assignmentsChromaffin_cells.Chrom_SOX2_statusHigh
assignmentsChromaffin_cells.Chrom_SOX2_statusHigh.EIF3J-DT	0.99152902	4.28558704	4.02640168	0.00013562	0.0007003	0.5886579	EIF3J-DT	assignmentsChromaffin_cells.Chrom_SOX2_statusHigh
assignmentsChromaffin_cells.Chrom_SOX2_statusHigh.PLPP6	1.18886645	1.35462895	3.96832891	0.00016589	0.00083118	0.58363399	PLPP6	assignmentsChromaffin_cells.Chrom_SOX2_statusHigh

assignmentsChromaffin_cells.Chrom_SOX2_statusHigh.DDX10	-0.8660255	6.72114534	-4.0443872	0.00012738	0.00066393	0.57992839	DDX10	assignmentsChromaffin_cells.Chrom_SOX2_statusHigh
assignmentsChromaffin_cells.Chrom_SOX2_statusHigh.NOM1	-0.9705578	4.98842901	-4.0154846	0.00014088	0.00072416	0.57992153	NOM1	assignmentsChromaffin_cells.Chrom_SOX2_statusHigh
assignmentsChromaffin_cells.Chrom_SOX2_statusHigh.COASY	0.83197232	3.4024386	4.01070288	0.00014324	0.00073277	0.57612458	COASY	assignmentsChromaffin_cells.Chrom_SOX2_statusHigh
assignmentsChromaffin_cells.Chrom_SOX2_statusHigh.CYP4X1	2.92383788	-0.033586	3.9078302	0.00020428	0.00099402	0.57235722	CYP4X1	assignmentsChromaffin_cells.Chrom_SOX2_statusHigh
assignmentsChromaffin_cells.Chrom_SOX2_statusHigh.IDH3B	1.03503396	3.81333084	4.00856042	0.00014431	0.00073745	0.57109099	IDH3B	assignmentsChromaffin_cells.Chrom_SOX2_statusHigh
assignmentsChromaffin_cells.Chrom_SOX2_statusHigh.ITM2C	2.36756817	5.08008628	4.04637522	0.00012677	0.00066121	0.56949589	ITM2C	assignmentsChromaffin_cells.Chrom_SOX2_statusHigh
assignmentsChromaffin_cells.Chrom_SOX2_statusHigh.RAB3GAP2	-0.6504637	7.44365972	-4.0569258	0.00012192	0.00063983	0.56765986	RAB3GAP2	assignmentsChromaffin_cells.Chrom_SOX2_statusHigh
assignmentsChromaffin_cells.Chrom_SOX2_statusHigh.VARS2	-1.1245986	2.5374329	-3.9677628	0.00016622	0.00083203	0.56678977	VARS2	assignmentsChromaffin_cells.Chrom_SOX2_statusHigh
assignmentsChromaffin_cells.Chrom_SOX2_statusHigh.SS18	-0.5668187	6.37348214	-4.0378489	0.00013032	0.00067757	0.56654703	SS18	assignmentsChromaffin_cells.Chrom_SOX2_statusHigh
assignmentsChromaffin_cells.Chrom_SOX2_statusHigh.LINC01574	4.46258756	-1.1693488	3.90687531	0.00020524	0.00099805	0.5637816	LINC01574	assignmentsChromaffin_cells.Chrom_SOX2_statusHigh
assignmentsChromaffin_cells.Chrom_SOX2_statusHigh.SEZ6L2	1.67133623	5.33275186	4.05383512	0.00012345	0.00064665	0.56307177	SEZ6L2	assignmentsChromaffin_cells.Chrom_SOX2_statusHigh
assignmentsChromaffin_cells.Chrom_SOX2_statusHigh.BSN	-1.2635385	3.78753611	-4.0157442	0.00014075	0.00072374	0.56058435	BSN	assignmentsChromaffin_cells.Chrom_SOX2_statusHigh
assignmentsChromaffin_cells.Chrom_SOX2_statusHigh.PNPLA2	1.32475651	4.05923766	4.00568934	0.00014575	0.00074328	0.55876531	PNPLA2	assignmentsChromaffin_cells.Chrom_SOX2_statusHigh
assignmentsChromaffin_cells.Chrom_SOX2_statusHigh.TSTD1	2.47916252	2.92622779	4.00459193	0.00014662	0.00074745	0.55722306	TSTD1	assignmentsChromaffin_cells.Chrom_SOX2_statusHigh
assignmentsChromaffin_cells.Chrom_SOX2_statusHigh.SNAPC4	-1.050661	3.74892622	-3.990759	0.0001535	0.0007776	0.55664745	SNAPC4	assignmentsChromaffin_cells.Chrom_SOX2_statusHigh
assignmentsChromaffin_cells.Chrom_SOX2_statusHigh.IMPACT	0.95165063	3.96968071	4.01424158	0.00014149	0.00072543	0.55460988	IMPACT	assignmentsChromaffin_cells.Chrom_SOX2_statusHigh
assignmentsChromaffin_cells.Chrom_SOX2_statusHigh.GBF1	-0.6877424	7.48546166	-4.0498133	0.00012499	0.00065316	0.55096006	GBF1	assignmentsChromaffin_cells.Chrom_SOX2_statusHigh
assignmentsChromaffin_cells.Chrom_SOX2_statusHigh.ACTR6	0.81327921	4.52418919	4.02059372	0.00013839	0.000713	0.5500289	ACTR6	assignmentsChromaffin_cells.Chrom_SOX2_statusHigh
assignmentsChromaffin_cells.Chrom_SOX2_statusHigh.ZBED4	-0.9102625	4.81552438	-4.0013941	0.00014794	0.00075349	0.5492605	ZBED4	assignmentsChromaffin_cells.Chrom_SOX2_statusHigh
assignmentsChromaffin_cells.Chrom_SOX2_statusHigh.PLOD3	0.83291801	4.80363181	4.01773851	0.00013978	0.00071966	0.54863374	PLOD3	assignmentsChromaffin_cells.Chrom_SOX2_statusHigh
assignmentsChromaffin_cells.Chrom_SOX2_statusHigh.ABI1	-0.8620565	7.016632	-4.0288653	0.00013447	0.00069544	0.54747202	ABI1	assignmentsChromaffin_cells.Chrom_SOX2_statusHigh
assignmentsChromaffin_cells.Chrom_SOX2_statusHigh.ZNF587	-0.831131	4.55902089	-4.0014595	0.00014791	0.00075349	0.54429566	ZNF587	assignmentsChromaffin_cells.Chrom_SOX2_statusHigh
assignmentsChromaffin_cells.Chrom_SOX2_statusHigh.DROSHA	-0.8044527	5.78316314	-4.0245176	0.00013652	0.00070468	0.54322648	DROSHA	assignmentsChromaffin_cells.Chrom_SOX2_statusHigh
assignmentsChromaffin_cells.Chrom_SOX2_statusHigh.COA7	1.18890598	1.93269203	3.96001556	0.00017073	0.00085166	0.54307009	COA7	assignmentsChromaffin_cells.Chrom_SOX2_statusHigh
assignmentsChromaffin_cells.Chrom_SOX2_statusHigh.ROGDI	1.24847998	2.74455535	3.9963693	0.00015055	0.0007648	0.53959939	ROGDI	assignmentsChromaffin_cells.Chrom_SOX2_statusHigh
assignmentsChromaffin_cells.Chrom_SOX2_statusHigh.RBPMS	-2.0097644	6.33216532	-4.0002682	0.00014876	0.00075691	0.5345593	RBPMS	assignmentsChromaffin_cells.Chrom_SOX2_statusHigh
assignmentsChromaffin_cells.Chrom_SOX2_statusHigh.IL6R-AS1	2.75552837	-0.2236475	3.89411041	0.0002141	0.00103525	0.53225138	IL6R-AS1	assignmentsChromaffin_cells.Chrom_SOX2_statusHigh
assignmentsChromaffin_cells.Chrom_SOX2_statusHigh.PRKCA	-2.0758593	8.19527714	-4.0348166	0.00013199	0.00068476	0.52911479	PRKCA	assignmentsChromaffin_cells.Chrom_SOX2_statusHigh
assignmentsChromaffin_cells.Chrom_SOX2_statusHigh.LEPROTL1	1.01269221	5.3223354	4.01460013	0.00014131	0.00072499	0.52505998	LEPROTL1	assignmentsChromaffin_cells.Chrom_SOX2_statusHigh
assignmentsChromaffin_cells.Chrom_SOX2_statusHigh.LINC00393	1.65455512	0.37671682	3.9116859	0.0002016	0.00098276	0.52477372	LINC00393	assignmentsChromaffin_cells.Chrom_SOX2_statusHigh
assignmentsChromaffin_cells.Chrom_SOX2_statusHigh.PTGS1	2.99834751	1.87667754	3.9241652	0.00019352	0.00094854	0.52404919	PTGS1	assignmentsChromaffin_cells.Chrom_SOX2_statusHigh
assignmentsChromaffin_cells.Chrom_SOX2_statusHigh.LINC02193	-1.3707411	1.81821313	-3.9287911	0.00019011	0.00093382	0.52384905	LINC02193	assignmentsChromaffin_cells.Chrom_SOX2_statusHigh
assignmentsChromaffin_cells.Chrom_SOX2_statusHigh.THOC2	-0.5269495	7.35636207	-4.0389158	0.00012983	0.00067541	0.5198919	THOC2	assignmentsChromaffin_cells.Chrom_SOX2_statusHigh
assignmentsChromaffin_cells.Chrom_SOX2_statusHigh.PDCL3	1.6894427	2.85662401	3.97255966	0.00016349	0.00082168	0.51912172	PDCL3	assignmentsChromaffin_cells.Chrom_SOX2_statusHigh
assignmentsChromaffin_cells.Chrom_SOX2_statusHigh.SCAPER	-0.8431497	8.0514091	-4.0500837	0.00012487	0.00065297	0.5167385	SCAPER	assignmentsChromaffin_cells.Chrom_SOX2_statusHigh
assignmentsChromaffin_cells.Chrom_SOX2_statusHigh.TOGARAM1	-0.7897204	6.51625268	-4.0295803	0.00013413	0.0006946	0.5158343	TOGARAM1	assignmentsChromaffin_cells.Chrom_SOX2_statusHigh
assignmentsChromaffin_cells.Chrom_SOX2_statusHigh.LINC01353	2.08503454	0.91008939	3.93594862	0.00018553	0.00091442	0.51350436	LINC01353	assignmentsChromaffin_cells.Chrom_SOX2_statusHigh
assignmentsChromaffin_cells.Chrom_SOX2_statusHigh.CAMK2N2	1.78056882	1.23613021	3.96804337	0.0001661	0.00083168	0.51317901	CAMK2N2	assignmentsChromaffin_cells.Chrom_SOX2_statusHigh
assignmentsChromaffin_cells.Chrom_SOX2_statusHigh.MRPS22	1.3456568	4.759579	4.01524994	0.00014099	0.00072452	0.50080648	MRPS22	assignmentsChromaffin_cells.Chrom_SOX2_statusHigh
assignmentsChromaffin_cells.Chrom_SOX2_statusHigh.EPN1	0.81979713	4.80054965	4.00671724	0.00014524	0.00074157	0.49796076	EPN1	assignmentsChromaffin_cells.Chrom_SOX2_statusHigh
assignmentsChromaffin_cells.Chrom_SOX2_statusHigh.RHOA	1.08270664	7.10621756	4.02860177	0.00013459	0.00069585	0.49397292	RHOA	assignmentsChromaffin_cells.Chrom_SOX2_statusHigh
assignmentsChromaffin_cells.Chrom_SOX2_statusHigh.DCAF1	-0.9378043	5.14976435	-3.9920146	0.00015284	0.00077514	0.49310322	DCAF1	assignmentsChromaffin_cells.Chrom_SOX2_statusHigh
assignmentsChromaffin_cells.Chrom_SOX2_statusHigh.NR3C2	-1.0617148	7.05692313	-4.0292648	0.00013428	0.00069477	0.4910533	NR3C2	assignmentsChromaffin_cells.Chrom_SOX2_statusHigh
assignmentsChromaffin_cells.Chrom_SOX2_statusHigh.DUSP5	2.80846897	3.63703623	3.9520262	0.00017585	0.00087315	0.48305761	DUSP5	assignmentsChromaffin_cells.Chrom_SOX2_statusHigh
assignmentsChromaffin_cells.Chrom_SOX2_statusHigh.PENK	6.40457314	2.15012536	3.94581339	0.00017965	0.00089011	0.48204272	PENK	assignmentsChromaffin_cells.Chrom_SOX2_statusHigh
assignmentsChromaffin_cells.Chrom_SOX2_statusHigh.RPL27A	2.82654648	6.04872592	4.01774958	0.00014007	0.00072093	0.48117133	RPL27A	assignmentsChromaffin_cells.Chrom_SOX2_statusHigh
assignmentsChromaffin_cells.Chrom_SOX2_statusHigh.METTL1	1.27370834	1.79546686	3.95209196	0.00017546	0.00087174	0.47986773	METTL1	assignmentsChromaffin_cells.Chrom_SOX2_statusHigh
assignmentsChromaffin_cells.Chrom_SOX2_statusHigh.HLA-C	2.23975674	6.80829259	4.01557406	0.00014113	0.00072455	0.47940191	HLA-C	assignmentsChromaffin_cells.Chrom_SOX2_statusHigh
assignmentsChromaffin_cells.Chrom_SOX2_statusHigh.KCNMA1	-1.4508476	8.7458105	-4.0457181	0.0001269	0.00066167	0.47597268	KCNMA1	assignmentsChromaffin_cells.Chrom_SOX2_statusHigh
assignmentsChromaffin_cells.Chrom_SOX2_statusHigh.DNMT3A	-0.9223071	6.23294612	-4.011433	0.00014287	0.00073138	0.4729676	DNMT3A	assignmentsChromaffin_cells.Chrom_SOX2_statusHigh
assignmentsChromaffin_cells.Chrom_SOX2_statusHigh.DIAPH2	-1.2407274	8.82218764	-4.0378157	0.00013033	0.00067757	0.468887	DIAPH2	assignmentsChromaffin_cells.Chrom_SOX2_statusHigh
assignmentsChromaffin_cells.Chrom_SOX2_statusHigh.RAP2B	1.67397155	3.666584	3.9499419	0.00017676	0.00087741	0.46466104	RAP2B	assignmentsChromaffin_cells.Chrom_SOX2_statusHigh
assignmentsChromaffin_cells.Chrom_SOX2_statusHigh.TMEM44-A	1.44177993	3.00416297	3.96932337	0.00016532	0.00082885	0.46451698	TMEM44-AS	assignmentsChromaffin_cells.Chrom_SOX2_statusHigh
assignmentsChromaffin_cells.Chrom_SOX2_statusHigh.ANKRD12	-0.7103164	8.69043345	-4.0351875	0.00013153	0.00068292	0.46393611	ANKRD12	assignmentsChromaffin_cells.Chrom_SOX2_statusHigh
assignmentsChromaffin_cells.Chrom_SOX2_statusHigh.ZNF14	-1.6466898	4.3787564	-3.9633307	0.00016878	0.00084329	0.46157579	ZNF14	assignmentsChromaffin_cells.Chrom_SOX2_statusHigh
assignmentsChromaffin_cells.Chrom_SOX2_statusHigh.ZNF358	1.11264803	3.47774867	3.97564648	0.00016175	0.00081423	0.45931147	ZNF358	assignmentsChromaffin_cells.Chrom_SOX2_statusHigh
assignmentsChromaffin_cells.Chrom_SOX2_statusHigh.HNRNPAB	1.60995818	4.9642167	3.98823153	0.00015495	0.00078417	0.45901622	HNRNPAB	assignmentsChromaffin_cells.Chrom_SOX2_statusHigh
assignmentsChromaffin_cells.Chrom_SOX2_statusHigh.SPIN2A	-3.38743	0.81078243	-3.8695991	0.0002329	0.00111203	0.4581938	SPIN2A	assignmentsChromaffin_cells.Chrom_SOX2_statusHigh
assignmentsChromaffin_cells.Chrom_SOX2_statusHigh.TMSB15B-A	-1.5841197	3.76108853	-3.9586919	0.00017151	0.0008553	0.45633663	TMSB15B-AS	assignmentsChromaffin_cells.Chrom_SOX2_statusHigh
assignmentsChromaffin_cells.Chrom_SOX2_statusHigh.NUTM2G	-3.0463827	0.07240357	-3.8688473	0.00023338	0.00111303	0.45612667	NUTM2G	assignmentsChromaffin_cells.Chrom_SOX2_statusHigh
assignmentsChromaffin_cells.Chrom_SOX2_statusHigh.PIK3CB	-0.9176181	6.1677256	-3.9958465	0.00015082	0.00076594	0.45336166	PIK3CB	assignmentsChromaffin_cells.Chrom_SOX2_statusHigh
assignmentsChromaffin_cells.Chrom_SOX2_statusHigh.PLAAT4	2.72760997	1.32864917	3.873051	0.00023006	0.00110077	0.45268895	PLAAT4	assignmentsChromaffin_cells.Chrom_SOX2_statusHigh
assignmentsChromaffin_cells.Chrom_SOX2_statusHigh.PDE8A	-0.9280011	8.39443437	-4.0217136	0.00013786	0.00071068	0.44887892	PDE8A	assignmentsChromaffin_cells.Chrom_SOX2_statusHigh
assignmentsChromaffin_cells.Chrom_SOX2_statusHigh.DUSP7	1.59262755	1.88752658	3.92931876	0.00018976	0.00093248	0.44835271	DUSP7	assignmentsChromaffin_cells.Chrom_SOX2_statusHigh

assignmentsChromaffin_cells.Chrom_SOX2_statusHigh.CDC37	1.13674583	6.69378774	4.01028977	0.00014344	0.00073359	0.44338906	CDC37	assignmentsChromaffin_cells.Chrom_SOX2_statusHigh
assignmentsChromaffin_cells.Chrom_SOX2_statusHigh.CEP350	-0.5865242	7.90762125	-4.0212255	0.00013809	0.00071166	0.44303725	CEP350	assignmentsChromaffin_cells.Chrom_SOX2_statusHigh
assignmentsChromaffin_cells.Chrom_SOX2_statusHigh.BCAM	2.37324198	2.94649528	3.93020928	0.00018946	0.00093151	0.44080864	BCAM	assignmentsChromaffin_cells.Chrom_SOX2_statusHigh
assignmentsChromaffin_cells.Chrom_SOX2_statusHigh.SVOP	-1.4815848	3.09617338	-3.9623536	0.00016935	0.00084561	0.43702809	SVOP	assignmentsChromaffin_cells.Chrom_SOX2_statusHigh
assignmentsChromaffin_cells.Chrom_SOX2_statusHigh.AASDHPPT	0.87926861	5.19254251	3.99672793	0.00015036	0.00076409	0.43622896	AASDHPPT	assignmentsChromaffin_cells.Chrom_SOX2_statusHigh
assignmentsChromaffin_cells.Chrom_SOX2_statusHigh.LRRK1	2.72807032	4.52174555	3.95464854	0.00017427	0.00086691	0.43298952	LRRK1	assignmentsChromaffin_cells.Chrom_SOX2_statusHigh
assignmentsChromaffin_cells.Chrom_SOX2_statusHigh.SNHG17	0.88361489	3.87703953	3.96991671	0.00016499	0.00082767	0.43252588	SNHG17	assignmentsChromaffin_cells.Chrom_SOX2_statusHigh
assignmentsChromaffin_cells.Chrom_SOX2_statusHigh.AHCTF1	-0.6872466	6.9737227	-4.0060241	0.00014559	0.00074298	0.42977631	AHCTF1	assignmentsChromaffin_cells.Chrom_SOX2_statusHigh
assignmentsChromaffin_cells.Chrom_SOX2_statusHigh.RNFT1-DT	1.61745856	0.81799987	3.89502322	0.00021344	0.00103354	0.42520398	RNFT1-DT	assignmentsChromaffin_cells.Chrom_SOX2_statusHigh
assignmentsChromaffin_cells.Chrom_SOX2_statusHigh.CACNG7	-1.9244171	3.31637756	-3.960835	0.00017039	0.00085025	0.42495172	CACNG7	assignmentsChromaffin_cells.Chrom_SOX2_statusHigh
assignmentsChromaffin_cells.Chrom_SOX2_statusHigh.SMURF2	-0.9510309	7.11047735	-3.9901769	0.00015381	0.00077868	0.42224665	SMURF2	assignmentsChromaffin_cells.Chrom_SOX2_statusHigh
assignmentsChromaffin_cells.Chrom_SOX2_statusHigh.TMEM168	-0.7432072	5.99216509	-3.9919476	0.00015287	0.00077514	0.41980041	TMEM168	assignmentsChromaffin_cells.Chrom_SOX2_statusHigh
assignmentsChromaffin_cells.Chrom_SOX2_statusHigh.FKBP3	0.90548582	5.01733007	3.98731226	0.00015535	0.00078546	0.41847044	FKBP3	assignmentsChromaffin_cells.Chrom_SOX2_statusHigh
assignmentsChromaffin_cells.Chrom_SOX2_statusHigh.MTPN	1.08914789	6.28834759	4.00046043	0.00014842	0.00075569	0.41654096	MTPN	assignmentsChromaffin_cells.Chrom_SOX2_statusHigh
assignmentsChromaffin_cells.Chrom_SOX2_statusHigh.PIAS1	-0.6014616	7.90595421	-4.0091414	0.00014402	0.00073629	0.41534954	PIAS1	assignmentsChromaffin_cells.Chrom_SOX2_statusHigh
assignmentsChromaffin_cells.Chrom_SOX2_statusHigh.EXT2	-0.8762945	5.92133771	-3.976012	0.00016155	0.00081347	0.41476157	EXT2	assignmentsChromaffin_cells.Chrom_SOX2_statusHigh
assignmentsChromaffin_cells.Chrom_SOX2_statusHigh.GARNL3	-1.3596067	6.05926465	-3.9925368	0.00015256	0.00077405	0.41310605	GARNL3	assignmentsChromaffin_cells.Chrom_SOX2_statusHigh
assignmentsChromaffin_cells.Chrom_SOX2_statusHigh.FGFBP3	1.24925695	1.61720382	3.9285264	0.00019028	0.00093439	0.41264328	FGFBP3	assignmentsChromaffin_cells.Chrom_SOX2_statusHigh
assignmentsChromaffin_cells.Chrom_SOX2_statusHigh.SDSL	1.26908869	2.51443226	3.936914	0.00018487	0.00091286	0.41237313	SDSL	assignmentsChromaffin_cells.Chrom_SOX2_statusHigh
assignmentsChromaffin_cells.Chrom_SOX2_statusHigh.ELP4	-0.9176585	6.64155707	-3.9875512	0.00015522	0.00078515	0.41216338	ELP4	assignmentsChromaffin_cells.Chrom_SOX2_statusHigh
assignmentsChromaffin_cells.Chrom_SOX2_statusHigh.PSMG3	1.34699703	2.65513482	3.94218999	0.00018154	0.00089809	0.40983756	PSMG3	assignmentsChromaffin_cells.Chrom_SOX2_statusHigh
assignmentsChromaffin_cells.Chrom_SOX2_statusHigh.POU3F1	3.82251382	-0.360731	3.85600352	0.00024394	0.00115409	0.40943759	POU3F1	assignmentsChromaffin_cells.Chrom_SOX2_statusHigh
assignmentsChromaffin_cells.Chrom_SOX2_statusHigh.CIB2	1.92168653	0.93185486	3.90051524	0.00020946	0.00101707	0.40814893	CIB2	assignmentsChromaffin_cells.Chrom_SOX2_statusHigh
assignmentsChromaffin_cells.Chrom_SOX2_statusHigh.SLC17A9	2.84641327	0.78311123	3.85261333	0.00024664	0.00116478	0.40775832	SLC17A9	assignmentsChromaffin_cells.Chrom_SOX2_statusHigh
assignmentsChromaffin_cells.Chrom_SOX2_statusHigh.JKAMP	0.82801037	5.40068424	3.99020002	0.0001538	0.00077868	0.40748345	JKAMP	assignmentsChromaffin_cells.Chrom_SOX2_statusHigh
assignmentsChromaffin_cells.Chrom_SOX2_statusHigh.TRAPPC10	-0.8974139	6.62530296	-3.9861146	0.00015599	0.00078823	0.40719002	TRAPPC10	assignmentsChromaffin_cells.Chrom_SOX2_statusHigh
assignmentsChromaffin_cells.Chrom_SOX2_statusHigh.ATG10	-0.7321545	6.5836395	-3.9939255	0.00015183	0.00077058	0.40641809	ATG10	assignmentsChromaffin_cells.Chrom_SOX2_statusHigh
assignmentsChromaffin_cells.Chrom_SOX2_statusHigh.FAM172A	-0.671659	8.36944954	-4.0147211	0.00014125	0.00072492	0.40614193	FAM172A	assignmentsChromaffin_cells.Chrom_SOX2_statusHigh
assignmentsChromaffin_cells.Chrom_SOX2_statusHigh.ZNF521	-4.169696	4.88975198	-3.8996508	0.00021049	0.00102159	0.40467449	ZNF521	assignmentsChromaffin_cells.Chrom_SOX2_statusHigh
assignmentsChromaffin_cells.Chrom_SOX2_statusHigh.PACSIN3	2.36734504	0.84609637	3.89927966	0.00021052	0.00102159	0.40325045	PACSIN3	assignmentsChromaffin_cells.Chrom_SOX2_statusHigh
assignmentsChromaffin_cells.Chrom_SOX2_statusHigh.C2CD4B	4.57180645	1.03368829	3.87454096	0.00022933	0.00109906	0.40070074	C2CD4B	assignmentsChromaffin_cells.Chrom_SOX2_statusHigh
assignmentsChromaffin_cells.Chrom_SOX2_statusHigh.CES4A	2.63820372	0.8891334	3.87410282	0.00022937	0.00109906	0.39905336	CES4A	assignmentsChromaffin_cells.Chrom_SOX2_statusHigh
assignmentsChromaffin_cells.Chrom_SOX2_statusHigh.PHKG2	0.83611614	3.83302777	3.95743326	0.00017225	0.00085822	0.39643902	PHKG2	assignmentsChromaffin_cells.Chrom_SOX2_statusHigh
assignmentsChromaffin_cells.Chrom_SOX2_statusHigh.PTAR1	-0.9591083	6.57555819	-3.9847451	0.00015674	0.00079173	0.38072189	PTAR1	assignmentsChromaffin_cells.Chrom_SOX2_statusHigh
assignmentsChromaffin_cells.Chrom_SOX2_statusHigh.TOMM34	0.80833839	3.54099089	3.94401448	0.00018041	0.00089302	0.37930445	TOMM34	assignmentsChromaffin_cells.Chrom_SOX2_statusHigh
assignmentsChromaffin_cells.Chrom_SOX2_statusHigh.ABCA12	-2.3031904	1.05037229	-3.882468	0.00022291	0.00107231	0.37838301	ABCA12	assignmentsChromaffin_cells.Chrom_SOX2_statusHigh
assignmentsChromaffin_cells.Chrom_SOX2_statusHigh.EEF1B2	2.36755548	6.11450953	3.99146783	0.00015345	0.0007776	0.37831786	EEF1B2	assignmentsChromaffin_cells.Chrom_SOX2_statusHigh
assignmentsChromaffin_cells.Chrom_SOX2_statusHigh.ACVR1	-1.0061279	6.06409986	-3.9736301	0.00016288	0.00081916	0.37299962	ACVR1	assignmentsChromaffin_cells.Chrom_SOX2_statusHigh
assignmentsChromaffin_cells.Chrom_SOX2_statusHigh.PDCD4-AS1	1.60888332	0.87216122	3.8915586	0.00021598	0.00104334	0.37289651	PDCD4-AS1	assignmentsChromaffin_cells.Chrom_SOX2_statusHigh
assignmentsChromaffin_cells.Chrom_SOX2_statusHigh.FAM234A	-0.7553952	4.81783459	-3.9557981	0.00017323	0.00086227	0.37251449	FAM234A	assignmentsChromaffin_cells.Chrom_SOX2_statusHigh
assignmentsChromaffin_cells.Chrom_SOX2_statusHigh.TOP1MT	1.46673414	2.46890748	3.91627508	0.00019845	0.00097036	0.37227292	TOP1MT	assignmentsChromaffin_cells.Chrom_SOX2_statusHigh
assignmentsChromaffin_cells.Chrom_SOX2_statusHigh.ZNF821	-0.8228554	4.85289519	-3.9584596	0.00017164	0.00085572	0.36719418	ZNF821	assignmentsChromaffin_cells.Chrom_SOX2_statusHigh
assignmentsChromaffin_cells.Chrom_SOX2_statusHigh.TAF10	1.79352743	3.58762819	3.93627657	0.00018539	0.00091399	0.36554374	TAF10	assignmentsChromaffin_cells.Chrom_SOX2_statusHigh
assignmentsChromaffin_cells.Chrom_SOX2_statusHigh.CENPW	1.99106744	1.38999856	3.90348542	0.00020745	0.00100822	0.36373321	CENPW	assignmentsChromaffin_cells.Chrom_SOX2_statusHigh
assignmentsChromaffin_cells.Chrom_SOX2_statusHigh.PRDM15	-0.9658618	3.40810872	-3.9141455	0.00019991	0.00097599	0.36280201	PRDM15	assignmentsChromaffin_cells.Chrom_SOX2_statusHigh
assignmentsChromaffin_cells.Chrom_SOX2_statusHigh.COPS5	0.81837991	5.03162266	3.96540428	0.00016758	0.00083779	0.35860232	COPS5	assignmentsChromaffin_cells.Chrom_SOX2_statusHigh
assignmentsChromaffin_cells.Chrom_SOX2_statusHigh.SPCS3	1.07342245	5.81548208	3.97600707	0.00016155	0.00081347	0.35767211	SPCS3	assignmentsChromaffin_cells.Chrom_SOX2_statusHigh
assignmentsChromaffin_cells.Chrom_SOX2_statusHigh.FBXO42	-1.028189	6.35408881	-3.968104	0.00016602	0.00083157	0.35554938	FBXO42	assignmentsChromaffin_cells.Chrom_SOX2_statusHigh
assignmentsChromaffin_cells.Chrom_SOX2_statusHigh.PPAN	0.93221064	3.63004912	3.94008526	0.00018286	0.00090406	0.34854258	PPAN	assignmentsChromaffin_cells.Chrom_SOX2_statusHigh
assignmentsChromaffin_cells.Chrom_SOX2_statusHigh.ATAT1	-0.8501747	3.88457043	-3.9354835	0.00018578	0.00091511	0.34733498	ATAT1	assignmentsChromaffin_cells.Chrom_SOX2_statusHigh
assignmentsChromaffin_cells.Chrom_SOX2_statusHigh.PNRC1	2.11138514	6.72507437	3.98443364	0.0001572	0.00079373	0.34583435	PNRC1	assignmentsChromaffin_cells.Chrom_SOX2_statusHigh
assignmentsChromaffin_cells.Chrom_SOX2_statusHigh.BCOR	0.99420799	4.44966745	3.93654251	0.00018511	0.00091346	0.34573354	BCOR	assignmentsChromaffin_cells.Chrom_SOX2_statusHigh
assignmentsChromaffin_cells.Chrom_SOX2_statusHigh.NUP62	0.753921	4.08658771	3.94474749	0.00017995	0.00089124	0.34537025	NUP62	assignmentsChromaffin_cells.Chrom_SOX2_statusHigh
assignmentsChromaffin_cells.Chrom_SOX2_statusHigh.LINC00243	2.10395071	1.02345055	3.83797746	0.0002592	0.0012165	0.34278857	LINC00243	assignmentsChromaffin_cells.Chrom_SOX2_statusHigh
assignmentsChromaffin_cells.Chrom_SOX2_statusHigh.TSTD2	-1.0504187	5.79714694	-3.9628909	0.00016904	0.0008443	0.34067499	TSTD2	assignmentsChromaffin_cells.Chrom_SOX2_statusHigh
assignmentsChromaffin_cells.Chrom_SOX2_statusHigh.PGF	2.70326245	2.68485338	3.89466264	0.00021411	0.00103525	0.33924487	PGF	assignmentsChromaffin_cells.Chrom_SOX2_statusHigh
assignmentsChromaffin_cells.Chrom_SOX2_statusHigh.CENPP	-1.2678906	6.59815494	-3.9665971	0.00016689	0.0008346	0.33877164	CENPP	assignmentsChromaffin_cells.Chrom_SOX2_statusHigh
assignmentsChromaffin_cells.Chrom_SOX2_statusHigh.VDAC3	0.92948017	5.57820923	3.97133532	0.00016418	0.00082465	0.33687174	VDAC3	assignmentsChromaffin_cells.Chrom_SOX2_statusHigh
assignmentsChromaffin_cells.Chrom_SOX2_statusHigh.ACTC1	2.83914263	-0.3803609	3.83305217	0.00026357	0.00123311	0.33586628	ACTC1	assignmentsChromaffin_cells.Chrom_SOX2_statusHigh
assignmentsChromaffin_cells.Chrom_SOX2_statusHigh.SORBS3	1.26573245	4.17024474	3.93632898	0.00018524	0.00091385	0.33559114	SORBS3	assignmentsChromaffin_cells.Chrom_SOX2_statusHigh
assignmentsChromaffin_cells.Chrom_SOX2_statusHigh.FRMD5	-2.4016863	6.34043546	-3.9693448	0.00016565	0.00083023	0.33500885	FRMD5	assignmentsChromaffin_cells.Chrom_SOX2_statusHigh
assignmentsChromaffin_cells.Chrom_SOX2_statusHigh.AKT3-IT1	-1.5062106	1.73547516	-3.8721949	0.00023074	0.00110332	0.3273354	AKT3-IT1	assignmentsChromaffin_cells.Chrom_SOX2_statusHigh
assignmentsChromaffin_cells.Chrom_SOX2_statusHigh.MCM3AP-A	-1.1501742	3.28980006	-3.9187396	0.00019678	0.00096366	0.31873754	MCM3AP-AS	assignmentsChromaffin_cells.Chrom_SOX2_statusHigh
assignmentsChromaffin_cells.Chrom_SOX2_statusHigh.ARL3	0.90231293	5.47563719	3.96753876	0.00016635	0.00083241	0.3163409	ARL3	assignmentsChromaffin_cells.Chrom_SOX2_statusHigh

assignmentsChromaffin_cells.Chrom_SOX2_statusHigh.ELMOD1	-2.6935839	1.68007825	-3.8639219	0.00023766	0.00112905	0.31387458	ELMOD1	assignmentsChromaffin_cells.Chrom_SOX2_statusHigh
assignmentsChromaffin_cells.Chrom_SOX2_statusHigh.SEMA4G	-1.8956349	1.50758591	-3.8662623	0.00023545	0.00112066	0.31353309	SEMA4G	assignmentsChromaffin_cells.Chrom_SOX2_statusHigh
assignmentsChromaffin_cells.Chrom_SOX2_statusHigh.DR1	0.93276396	4.5595062	3.9344001	0.00018648	0.00091796	0.31333148	DR1	assignmentsChromaffin_cells.Chrom_SOX2_statusHigh
assignmentsChromaffin_cells.Chrom_SOX2_statusHigh.CSTF3	-0.8780639	5.75736766	-3.9479619	0.00017797	0.00088205	0.30957972	CSTF3	assignmentsChromaffin_cells.Chrom_SOX2_statusHigh
assignmentsChromaffin_cells.Chrom_SOX2_statusHigh.KLHDC8A	3.41797678	0.36148015	3.81928534	0.00027626	0.00128314	0.30783445	KLHDC8A	assignmentsChromaffin_cells.Chrom_SOX2_statusHigh
assignmentsChromaffin_cells.Chrom_SOX2_statusHigh.NDUFA12	1.3110262	5.24702945	3.96148812	0.00016986	0.00084788	0.30694427	NDUFA12	assignmentsChromaffin_cells.Chrom_SOX2_statusHigh
assignmentsChromaffin_cells.Chrom_SOX2_statusHigh.ZEB2	2.3144024	7.43555557	3.92475468	0.00019314	0.00094728	0.30598044	ZEB2	assignmentsChromaffin_cells.Chrom_SOX2_statusHigh
assignmentsChromaffin_cells.Chrom_SOX2_statusHigh.OLA1	-0.6464229	7.18012489	-3.9733177	0.00016306	0.00081979	0.30345128	OLA1	assignmentsChromaffin_cells.Chrom_SOX2_statusHigh
assignmentsChromaffin_cells.Chrom_SOX2_statusHigh.IER5L	3.09644961	3.18429368	3.87492788	0.00022903	0.00109811	0.30190171	IER5L	assignmentsChromaffin_cells.Chrom_SOX2_statusHigh
assignmentsChromaffin_cells.Chrom_SOX2_statusHigh.MEIS2	-2.1044635	7.14043831	-3.9703623	0.00016507	0.00082783	0.30053195	MEIS2	assignmentsChromaffin_cells.Chrom_SOX2_statusHigh
assignmentsChromaffin_cells.Chrom_SOX2_statusHigh.CYYR1	-2.5359838	3.76993356	-3.8869502	0.00021983	0.00105969	0.29959291	CYYR1	assignmentsChromaffin_cells.Chrom_SOX2_statusHigh
assignmentsChromaffin_cells.Chrom_SOX2_statusHigh.ZNF215	-2.5254226	1.18227119	-3.8385317	0.00025883	0.00121522	0.29796431	ZNF215	assignmentsChromaffin_cells.Chrom_SOX2_statusHigh
assignmentsChromaffin_cells.Chrom_SOX2_statusHigh.UBE2K	-0.847553	7.61749266	-3.9708171	0.00016447	0.00082561	0.29526118	UBE2K	assignmentsChromaffin_cells.Chrom_SOX2_statusHigh
assignmentsChromaffin_cells.Chrom_SOX2_statusHigh.CBFB	-0.9221388	6.6039982	-3.9517673	0.00017565	0.00087244	0.2929166	CBFB	assignmentsChromaffin_cells.Chrom_SOX2_statusHigh
assignmentsChromaffin_cells.Chrom_SOX2_statusHigh.TTC17	-0.9763084	7.81734535	-3.970029	0.00016492	0.0008276	0.29192332	TTC17	assignmentsChromaffin_cells.Chrom_SOX2_statusHigh
assignmentsChromaffin_cells.Chrom_SOX2_statusHigh.ZNF674	-0.968861	3.45335785	-3.9066425	0.00020512	0.00099777	0.29166343	ZNF674	assignmentsChromaffin_cells.Chrom_SOX2_statusHigh
assignmentsChromaffin_cells.Chrom_SOX2_statusHigh.ARL4D	2.61414753	2.3488345	3.90256844	0.0002084	0.00101221	0.29116846	ARL4D	assignmentsChromaffin_cells.Chrom_SOX2_statusHigh
assignmentsChromaffin_cells.Chrom_SOX2_statusHigh.C1orf159	-0.8700177	4.080897	-3.9123009	0.00020118	0.00098158	0.29105563	C1orf159	assignmentsChromaffin_cells.Chrom_SOX2_statusHigh
assignmentsChromaffin_cells.Chrom_SOX2_statusHigh.MPHOSPH1	0.74949516	5.16793724	3.94833952	0.00017774	0.00088117	0.28949957	MPHOSPH10	assignmentsChromaffin_cells.Chrom_SOX2_statusHigh
assignmentsChromaffin_cells.Chrom_SOX2_statusHigh.PTPN12	-1.0452125	7.39151397	-3.9565549	0.00017278	0.00086029	0.2890821	PTPN12	assignmentsChromaffin_cells.Chrom_SOX2_statusHigh
assignmentsChromaffin_cells.Chrom_SOX2_statusHigh.MYOZ1	2.90799094	-0.3911911	3.81323919	0.00028184	0.00130454	0.28901845	MYOZ1	assignmentsChromaffin_cells.Chrom_SOX2_statusHigh
assignmentsChromaffin_cells.Chrom_SOX2_statusHigh.ARNT2	-1.5118321	4.00743818	-3.9282379	0.00019047	0.00093503	0.28844023	ARNT2	assignmentsChromaffin_cells.Chrom_SOX2_statusHigh
assignmentsChromaffin_cells.Chrom_SOX2_statusHigh.TEX15	-5.8632781	0.45793296	-3.8152307	0.00028046	0.00129962	0.28673089	TEX15	assignmentsChromaffin_cells.Chrom_SOX2_statusHigh
assignmentsChromaffin_cells.Chrom_SOX2_statusHigh.PIGW	1.31940294	1.34829347	3.87334247	0.00022984	0.00110033	0.28666729	PIGW	assignmentsChromaffin_cells.Chrom_SOX2_statusHigh
assignmentsChromaffin_cells.Chrom_SOX2_statusHigh.RAB2A	0.90796587	7.91344315	3.97744277	0.00016075	0.00081021	0.27841741	RAB2A	assignmentsChromaffin_cells.Chrom_SOX2_statusHigh
assignmentsChromaffin_cells.Chrom_SOX2_statusHigh.EDNRB-AS1	2.39484788	0.12990651	3.83127891	0.00026516	0.0012391	0.27490986	EDNRB-AS1	assignmentsChromaffin_cells.Chrom_SOX2_statusHigh
assignmentsChromaffin_cells.Chrom_SOX2_statusHigh.TMSB15B	-1.2603491	1.94487663	-3.8725604	0.00023045	0.00110228	0.27076697	TMSB15B	assignmentsChromaffin_cells.Chrom_SOX2_statusHigh
assignmentsChromaffin_cells.Chrom_SOX2_statusHigh.PRKX	-2.5055809	5.21296776	-3.8594124	0.00024127	0.00114246	0.26998351	PRKX	assignmentsChromaffin_cells.Chrom_SOX2_statusHigh
assignmentsChromaffin_cells.Chrom_SOX2_statusHigh.CCAR1	-0.5852957	6.87145741	-3.9526754	0.0001751	0.00087025	0.26646171	CCAR1	assignmentsChromaffin_cells.Chrom_SOX2_statusHigh
assignmentsChromaffin_cells.Chrom_SOX2_statusHigh.METAP1D	-0.975021	4.29117419	-3.9154926	0.00019899	0.00097268	0.26497333	METAP1D	assignmentsChromaffin_cells.Chrom_SOX2_statusHigh
assignmentsChromaffin_cells.Chrom_SOX2_statusHigh.HEATR5A	-0.9516995	5.87585828	-3.9292994	0.00018978	0.00093248	0.26334649	HEATR5A	assignmentsChromaffin_cells.Chrom_SOX2_statusHigh
assignmentsChromaffin_cells.Chrom_SOX2_statusHigh.TAX1BP3	1.69841433	2.68628855	3.88039346	0.00022437	0.0010774	0.26321125	TAX1BP3	assignmentsChromaffin_cells.Chrom_SOX2_statusHigh
assignmentsChromaffin_cells.Chrom_SOX2_statusHigh.PITHD1	1.20459576	4.11978265	3.91675803	0.00019812	0.00096905	0.2628892	PITHD1	assignmentsChromaffin_cells.Chrom_SOX2_statusHigh
assignmentsChromaffin_cells.Chrom_SOX2_statusHigh.SRA1	0.95853848	5.32625503	3.93790451	0.00018424	0.00091031	0.25701304	SRA1	assignmentsChromaffin_cells.Chrom_SOX2_statusHigh
assignmentsChromaffin_cells.Chrom_SOX2_statusHigh.TMEM187	1.0121052	2.05379247	3.88111205	0.00022382	0.0010754	0.25644842	TMEM187	assignmentsChromaffin_cells.Chrom_SOX2_statusHigh
assignmentsChromaffin_cells.Chrom_SOX2_statusHigh.TAB1	-0.9953293	3.92227935	-3.8977209	0.00021148	0.00102548	0.25640021	TAB1	assignmentsChromaffin_cells.Chrom_SOX2_statusHigh
assignmentsChromaffin_cells.Chrom_SOX2_statusHigh.ACACA	-0.9460771	6.81969292	-3.9539089	0.00017436	0.0008671	0.25249251	ACACA	assignmentsChromaffin_cells.Chrom_SOX2_statusHigh
assignmentsChromaffin_cells.Chrom_SOX2_statusHigh.ZNF277	-0.6591576	6.22557717	-3.9354818	0.00018578	0.00091511	0.25057224	ZNF277	assignmentsChromaffin_cells.Chrom_SOX2_statusHigh
assignmentsChromaffin_cells.Chrom_SOX2_statusHigh.CNTN4-AS2	-3.5803686	-0.0179979	-3.8000427	0.00029484	0.00135606	0.25005412	CNTN4-AS2	assignmentsChromaffin_cells.Chrom_SOX2_statusHigh
assignmentsChromaffin_cells.Chrom_SOX2_statusHigh.FH	0.8961143	4.23545601	3.9225051	0.00019426	0.00095187	0.25001615	FH	assignmentsChromaffin_cells.Chrom_SOX2_statusHigh
assignmentsChromaffin_cells.Chrom_SOX2_statusHigh.ANKS3	-0.7666787	4.8402081	-3.9202384	0.00019577	0.00095901	0.24980901	ANKS3	assignmentsChromaffin_cells.Chrom_SOX2_statusHigh
assignmentsChromaffin_cells.Chrom_SOX2_statusHigh.SYNDIG1	3.59619076	2.48560246	3.86965406	0.00023318	0.00111303	0.24934551	SYNDIG1	assignmentsChromaffin_cells.Chrom_SOX2_statusHigh
assignmentsChromaffin_cells.Chrom_SOX2_statusHigh.CRTC3	-0.8549884	6.74695567	-3.9376761	0.00018439	0.00091075	0.2478267	CRTC3	assignmentsChromaffin_cells.Chrom_SOX2_statusHigh
assignmentsChromaffin_cells.Chrom_SOX2_statusHigh.FAR1	-0.874594	6.94554688	-3.941236	0.00018214	0.00090077	0.24654269	FAR1	assignmentsChromaffin_cells.Chrom_SOX2_statusHigh
assignmentsChromaffin_cells.Chrom_SOX2_statusHigh.RPL9	2.75607377	7.01591833	3.96480034	0.00016827	0.00084099	0.24616077	RPL9	assignmentsChromaffin_cells.Chrom_SOX2_statusHigh
assignmentsChromaffin_cells.Chrom_SOX2_statusHigh.MFSD3	1.39290722	2.03755977	3.8883413	0.00021837	0.00105392	0.24476218	MFSD3	assignmentsChromaffin_cells.Chrom_SOX2_statusHigh
assignmentsChromaffin_cells.Chrom_SOX2_statusHigh.DGKA	-1.303122	4.9243868	-3.8930532	0.00021488	0.00103865	0.24153039	DGKA	assignmentsChromaffin_cells.Chrom_SOX2_statusHigh
assignmentsChromaffin_cells.Chrom_SOX2_statusHigh.SRXN1	1.63175919	1.9260671	3.87784002	0.00022634	0.00108585	0.24108027	SRXN1	assignmentsChromaffin_cells.Chrom_SOX2_statusHigh
assignmentsChromaffin_cells.Chrom_SOX2_statusHigh.ZNF181	1.07253674	3.34020832	3.90398868	0.00020699	0.00100627	0.23619476	ZNF181	assignmentsChromaffin_cells.Chrom_SOX2_statusHigh
assignmentsChromaffin_cells.Chrom_SOX2_statusHigh.YBX1	1.84611775	6.17039568	3.93571213	0.00018601	0.00091593	0.23278072	YBX1	assignmentsChromaffin_cells.Chrom_SOX2_statusHigh
assignmentsChromaffin_cells.Chrom_SOX2_statusHigh.C1orf74	1.23298161	0.72716798	3.835875	0.00026106	0.00122352	0.23037243	C1orf74	assignmentsChromaffin_cells.Chrom_SOX2_statusHigh
assignmentsChromaffin_cells.Chrom_SOX2_statusHigh.WDR11	-0.7035453	6.04337882	-3.9332491	0.00018722	0.00092132	0.2296562	WDR11	assignmentsChromaffin_cells.Chrom_SOX2_statusHigh
assignmentsChromaffin_cells.Chrom_SOX2_statusHigh.TTLL5	-0.8662819	6.36573029	-3.9279189	0.00019068	0.00093577	0.22616565	TTLL5	assignmentsChromaffin_cells.Chrom_SOX2_statusHigh
assignmentsChromaffin_cells.Chrom_SOX2_statusHigh.PALB2	-0.8923156	3.51673308	-3.8827364	0.00022259	0.00107106	0.22571684	PALB2	assignmentsChromaffin_cells.Chrom_SOX2_statusHigh
assignmentsChromaffin_cells.Chrom_SOX2_statusHigh.MYL9	2.19591511	3.05694352	3.83593079	0.00026113	0.00122352	0.2216189	MYL9	assignmentsChromaffin_cells.Chrom_SOX2_statusHigh
assignmentsChromaffin_cells.Chrom_SOX2_statusHigh.LAMP2	0.95541364	5.98572034	3.9384864	0.00018387	0.00090877	0.22161445	LAMP2	assignmentsChromaffin_cells.Chrom_SOX2_statusHigh
assignmentsChromaffin_cells.Chrom_SOX2_statusHigh.IKBKE	2.09637204	1.72570967	3.81389637	0.00028122	0.00130219	0.22014128	IKBKE	assignmentsChromaffin_cells.Chrom_SOX2_statusHigh
assignmentsChromaffin_cells.Chrom_SOX2_statusHigh.SUPT16H	0.75294567	5.95562217	3.93680704	0.00018494	0.00091291	0.21774926	SUPT16H	assignmentsChromaffin_cells.Chrom_SOX2_statusHigh
assignmentsChromaffin_cells.Chrom_SOX2_statusHigh.CNOT6	-0.9895073	5.44321249	-3.9152348	0.00019916	0.00097294	0.21610755	CNOT6	assignmentsChromaffin_cells.Chrom_SOX2_statusHigh
assignmentsChromaffin_cells.Chrom_SOX2_statusHigh.ZFC3H1	-0.7956876	7.51268563	-3.9429096	0.0001811	0.00089615	0.21512709	ZFC3H1	assignmentsChromaffin_cells.Chrom_SOX2_statusHigh
assignmentsChromaffin_cells.Chrom_SOX2_statusHigh.LINC00523	3.66911095	-1.2500945	3.78760337	0.00030746	0.00140487	0.21397282	LINC00523	assignmentsChromaffin_cells.Chrom_SOX2_statusHigh
assignmentsChromaffin_cells.Chrom_SOX2_statusHigh.CASP4	2.25552784	3.82917845	3.80501214	0.00028978	0.00133704	0.21316179	CASP4	assignmentsChromaffin_cells.Chrom_SOX2_statusHigh
assignmentsChromaffin_cells.Chrom_SOX2_statusHigh.PPP1R32	1.08374842	1.35149698	3.84753965	0.00025093	0.00118329	0.21086269	PPP1R32	assignmentsChromaffin_cells.Chrom_SOX2_statusHigh
assignmentsChromaffin_cells.Chrom_SOX2_statusHigh.CRTAC1	4.32051027	1.34728253	3.82906864	0.00026763	0.00124777	0.20930409	CRTAC1	assignmentsChromaffin_cells.Chrom_SOX2_statusHigh

assignmentsChromaffin_cells.Chrom_SOX2_statusHigh.PABPC1L	-1.300362	4.17915923	-3.8870986	0.0002193	0.00105745	0.20826962	PABPC1L	assignmentsChromaffin_cells.Chrom_SOX2_statusHigh
assignmentsChromaffin_cells.Chrom_SOX2_statusHigh.RPL37A	2.24754905	7.51914583	3.95885879	0.00017176	0.00085601	0.20356086	RPL37A	assignmentsChromaffin_cells.Chrom_SOX2_statusHigh
assignmentsChromaffin_cells.Chrom_SOX2_statusHigh.GLRX	2.24533905	4.06231701	3.8905538	0.00021714	0.00104862	0.19978447	GLRX	assignmentsChromaffin_cells.Chrom_SOX2_statusHigh
assignmentsChromaffin_cells.Chrom_SOX2_statusHigh.HEY1	1.98954154	2.24104428	3.84741131	0.00025115	0.001184	0.19852689	HEY1	assignmentsChromaffin_cells.Chrom_SOX2_statusHigh
assignmentsChromaffin_cells.Chrom_SOX2_statusHigh.EML6	-1.8314916	6.93919848	-3.9452644	0.00017999	0.00089124	0.19436394	EML6	assignmentsChromaffin_cells.Chrom_SOX2_statusHigh
assignmentsChromaffin_cells.Chrom_SOX2_statusHigh.TCAP	1.88137966	0.41189839	3.8172937	0.00027801	0.00128976	0.19265675	TCAP	assignmentsChromaffin_cells.Chrom_SOX2_statusHigh
assignmentsChromaffin_cells.Chrom_SOX2_statusHigh.FNIP1	-0.7932798	8.17124281	-3.9487955	0.00017746	0.00088006	0.18732387	FNIP1	assignmentsChromaffin_cells.Chrom_SOX2_statusHigh
assignmentsChromaffin_cells.Chrom_SOX2_statusHigh.ZNF433-AS	-1.1935125	5.46353371	-3.8959643	0.00021275	0.00103053	0.1838304	ZNF433-AS1	assignmentsChromaffin_cells.Chrom_SOX2_statusHigh
assignmentsChromaffin_cells.Chrom_SOX2_statusHigh.B3GNT9	1.68678927	0.49920368	3.80273279	0.00029202	0.00134698	0.18243789	B3GNT9	assignmentsChromaffin_cells.Chrom_SOX2_statusHigh
assignmentsChromaffin_cells.Chrom_SOX2_statusHigh.TXNRD2	-0.7185124	4.08219389	-3.8850609	0.00022083	0.00106323	0.17806655	TXNRD2	assignmentsChromaffin_cells.Chrom_SOX2_statusHigh
assignmentsChromaffin_cells.Chrom_SOX2_statusHigh.PRXL2B	1.38461424	1.45196831	3.83047008	0.00026588	0.00124177	0.17627294	PRXL2B	assignmentsChromaffin_cells.Chrom_SOX2_statusHigh
assignmentsChromaffin_cells.Chrom_SOX2_statusHigh.PGPEP1	0.88482127	3.91955663	3.88593149	0.00022017	0.00106103	0.1758083	PGPEP1	assignmentsChromaffin_cells.Chrom_SOX2_statusHigh
assignmentsChromaffin_cells.Chrom_SOX2_statusHigh.PCOLCE	2.67553128	2.77044862	3.85302508	0.00024675	0.00116497	0.17251697	PCOLCE	assignmentsChromaffin_cells.Chrom_SOX2_statusHigh
assignmentsChromaffin_cells.Chrom_SOX2_statusHigh.PPP3CB	-0.8465783	6.65549066	-3.9238426	0.00019337	0.00094809	0.17093611	PPP3CB	assignmentsChromaffin_cells.Chrom_SOX2_statusHigh
assignmentsChromaffin_cells.Chrom_SOX2_statusHigh.NRIP2	-2.6105718	0.97850649	-3.7743118	0.00032134	0.00145749	0.16740725	NRIP2	assignmentsChromaffin_cells.Chrom_SOX2_statusHigh
assignmentsChromaffin_cells.Chrom_SOX2_statusHigh.BICRA-AS1	-3.265595	1.294673	-3.7795932	0.00031592	0.001439	0.16421673	BICRA-AS1	assignmentsChromaffin_cells.Chrom_SOX2_statusHigh
assignmentsChromaffin_cells.Chrom_SOX2_statusHigh.CLDND1	1.03922122	5.80336022	3.9115702	0.00020168	0.00098285	0.16071778	CLDND1	assignmentsChromaffin_cells.Chrom_SOX2_statusHigh
assignmentsChromaffin_cells.Chrom_SOX2_statusHigh.FBXO11	-0.8183294	8.12258534	-3.9327497	0.00018754	0.00092262	0.15973473	FBXO11	assignmentsChromaffin_cells.Chrom_SOX2_statusHigh
assignmentsChromaffin_cells.Chrom_SOX2_statusHigh.TANC2	-1.3768206	8.30681033	-3.9363612	0.00018533	0.00091399	0.15924589	TANC2	assignmentsChromaffin_cells.Chrom_SOX2_statusHigh
assignmentsChromaffin_cells.Chrom_SOX2_statusHigh.KIFC2	-1.0846392	3.35581481	-3.8704304	0.00023213	0.00110932	0.15922601	KIFC2	assignmentsChromaffin_cells.Chrom_SOX2_statusHigh
assignmentsChromaffin_cells.Chrom_SOX2_statusHigh.UBE3A	-0.515758	7.47729741	-3.9307179	0.00018885	0.0009288	0.15873464	UBE3A	assignmentsChromaffin_cells.Chrom_SOX2_statusHigh
assignmentsChromaffin_cells.Chrom_SOX2_statusHigh.ALDH4A1	1.47392385	1.29813383	3.81674091	0.00027853	0.0012918	0.1535366	ALDH4A1	assignmentsChromaffin_cells.Chrom_SOX2_statusHigh
assignmentsChromaffin_cells.Chrom_SOX2_statusHigh.SYDE2	-2.456556	1.96855875	-3.7925147	0.0003024	0.00138488	0.14784275	SYDE2	assignmentsChromaffin_cells.Chrom_SOX2_statusHigh
assignmentsChromaffin_cells.Chrom_SOX2_statusHigh.PLEKHO2	1.40578806	3.72916825	3.82895031	0.00026726	0.00124673	0.14731541	PLEKHO2	assignmentsChromaffin_cells.Chrom_SOX2_statusHigh
assignmentsChromaffin_cells.Chrom_SOX2_statusHigh.PPM1B	-0.7044735	6.7483661	-3.9120732	0.00020133	0.00098205	0.14639487	PPM1B	assignmentsChromaffin_cells.Chrom_SOX2_statusHigh
assignmentsChromaffin_cells.Chrom_SOX2_statusHigh.SEC11A	1.02838302	6.36497133	3.91837273	0.00019703	0.00096399	0.14604373	SEC11A	assignmentsChromaffin_cells.Chrom_SOX2_statusHigh
assignmentsChromaffin_cells.Chrom_SOX2_statusHigh.ATP11C	-1.6424233	6.03228105	-3.8809173	0.0002241	0.00107639	0.14558828	ATP11C	assignmentsChromaffin_cells.Chrom_SOX2_statusHigh
assignmentsChromaffin_cells.Chrom_SOX2_statusHigh.BLOC1S2	0.94487973	4.51785565	3.89432648	0.00021395	0.00103507	0.14444097	BLOC1S2	assignmentsChromaffin_cells.Chrom_SOX2_statusHigh
assignmentsChromaffin_cells.Chrom_SOX2_statusHigh.PYGO2	0.9299909	3.13054138	3.86846933	0.00023368	0.00111344	0.14417604	PYGO2	assignmentsChromaffin_cells.Chrom_SOX2_statusHigh
assignmentsChromaffin_cells.Chrom_SOX2_statusHigh.MTOR	-0.9221586	5.69764589	-3.888196	0.00021848	0.00105413	0.14402207	MTOR	assignmentsChromaffin_cells.Chrom_SOX2_statusHigh
assignmentsChromaffin_cells.Chrom_SOX2_statusHigh.PTPRU	2.24251615	1.58255209	3.82930403	0.00026714	0.00124655	0.14281024	PTPRU	assignmentsChromaffin_cells.Chrom_SOX2_statusHigh
assignmentsChromaffin_cells.Chrom_SOX2_statusHigh.POLR1A	-0.7177121	5.14655446	-3.8920846	0.00021559	0.00104178	0.14219111	POLR1A	assignmentsChromaffin_cells.Chrom_SOX2_statusHigh
assignmentsChromaffin_cells.Chrom_SOX2_statusHigh.LINC01460	2.27865434	0.03006726	3.79524184	0.00029954	0.00137299	0.13904172	LINC01460	assignmentsChromaffin_cells.Chrom_SOX2_statusHigh
assignmentsChromaffin_cells.Chrom_SOX2_statusHigh.FKBP2	2.70331566	2.85608508	3.8737439	0.00022996	0.00110057	0.13767813	FKBP2	assignmentsChromaffin_cells.Chrom_SOX2_statusHigh
assignmentsChromaffin_cells.Chrom_SOX2_statusHigh.FGF12	-2.1740323	6.08330211	-3.9098187	0.00020329	0.00098979	0.12976804	FGF12	assignmentsChromaffin_cells.Chrom_SOX2_statusHigh
assignmentsChromaffin_cells.Chrom_SOX2_statusHigh.TMEM186	1.12501021	1.769421	3.84386925	0.00025407	0.00119707	0.12899844	TMEM186	assignmentsChromaffin_cells.Chrom_SOX2_statusHigh
assignmentsChromaffin_cells.Chrom_SOX2_statusHigh.DDIT4	1.98971592	5.83108773	3.89659017	0.00021271	0.00103053	0.12885193	DDIT4	assignmentsChromaffin_cells.Chrom_SOX2_statusHigh
assignmentsChromaffin_cells.Chrom_SOX2_statusHigh.RPUSD3	0.93149718	3.53804452	3.86642368	0.00023532	0.00112056	0.12791769	RPUSD3	assignmentsChromaffin_cells.Chrom_SOX2_statusHigh
assignmentsChromaffin_cells.Chrom_SOX2_statusHigh.CFAP36	0.81269116	5.67917079	3.91105206	0.00020204	0.00098401	0.12629962	CFAP36	assignmentsChromaffin_cells.Chrom_SOX2_statusHigh
assignmentsChromaffin_cells.Chrom_SOX2_statusHigh.CUL9	-0.8275483	4.51383015	-3.8714245	0.00023134	0.0011059	0.12185232	CUL9	assignmentsChromaffin_cells.Chrom_SOX2_statusHigh
assignmentsChromaffin_cells.Chrom_SOX2_statusHigh.RAP1GDS1	-0.5757154	7.33782703	-3.913143	0.0002006	0.00097905	0.11881887	RAP1GDS1	assignmentsChromaffin_cells.Chrom_SOX2_statusHigh
assignmentsChromaffin_cells.Chrom_SOX2_statusHigh.FNBP1	-1.2811229	8.01741496	-3.9110502	0.00020204	0.00098401	0.1184005	FNBP1	assignmentsChromaffin_cells.Chrom_SOX2_statusHigh
assignmentsChromaffin_cells.Chrom_SOX2_statusHigh.TBCK	-0.8095594	7.51024526	-3.9185083	0.00019694	0.00096384	0.1181472	TBCK	assignmentsChromaffin_cells.Chrom_SOX2_statusHigh
assignmentsChromaffin_cells.Chrom_SOX2_statusHigh.ZBED3	1.3608888	3.17388062	3.86570339	0.0002359	0.00112164	0.11734221	ZBED3	assignmentsChromaffin_cells.Chrom_SOX2_statusHigh
assignmentsChromaffin_cells.Chrom_SOX2_statusHigh.SMC5	-0.9165672	7.86914001	-3.9185599	0.0001969	0.00096384	0.11698202	SMC5	assignmentsChromaffin_cells.Chrom_SOX2_statusHigh
assignmentsChromaffin_cells.Chrom_SOX2_statusHigh.GALNT11	-0.7898893	6.67275729	-3.9082541	0.00020399	0.00099288	0.11540184	GALNT11	assignmentsChromaffin_cells.Chrom_SOX2_statusHigh
assignmentsChromaffin_cells.Chrom_SOX2_statusHigh.RPS6KC1	-0.6493483	7.28486216	-3.9149822	0.00019934	0.00097349	0.11402929	RPS6KC1	assignmentsChromaffin_cells.Chrom_SOX2_statusHigh
assignmentsChromaffin_cells.Chrom_SOX2_statusHigh.ADCK2	1.06665308	2.55856648	3.85435438	0.00024518	0.00115927	0.11322436	ADCK2	assignmentsChromaffin_cells.Chrom_SOX2_statusHigh
assignmentsChromaffin_cells.Chrom_SOX2_statusHigh.PSMC2	0.62599073	5.36311906	3.89705459	0.00021196	0.00102732	0.11213861	PSMC2	assignmentsChromaffin_cells.Chrom_SOX2_statusHigh
assignmentsChromaffin_cells.Chrom_SOX2_statusHigh.SUPT7L	0.5705562	4.56617936	3.88189373	0.00022323	0.00107318	0.11090135	SUPT7L	assignmentsChromaffin_cells.Chrom_SOX2_statusHigh
assignmentsChromaffin_cells.Chrom_SOX2_statusHigh.SMC2	1.01415285	3.91714175	3.86764065	0.00023434	0.00111625	0.1098119	SMC2	assignmentsChromaffin_cells.Chrom_SOX2_statusHigh
assignmentsChromaffin_cells.Chrom_SOX2_statusHigh.DMXL1	-0.7369243	7.39117841	-3.9118861	0.00020146	0.00098238	0.10672415	DMXL1	assignmentsChromaffin_cells.Chrom_SOX2_statusHigh
assignmentsChromaffin_cells.Chrom_SOX2_statusHigh.UBP1	-0.818308	5.73966721	-3.8813999	0.0002236	0.00107467	0.10667446	UBP1	assignmentsChromaffin_cells.Chrom_SOX2_statusHigh
assignmentsChromaffin_cells.Chrom_SOX2_statusHigh.ZNF276	-0.7275399	4.65922192	-3.8605666	0.00024006	0.00113945	0.10599459	ZNF276	assignmentsChromaffin_cells.Chrom_SOX2_statusHigh
assignmentsChromaffin_cells.Chrom_SOX2_statusHigh.MALINC1	1.2510099	1.19778422	3.80745434	0.00028741	0.0013276	0.10499916	MALINC1	assignmentsChromaffin_cells.Chrom_SOX2_statusHigh
assignmentsChromaffin_cells.Chrom_SOX2_statusHigh.ADO	1.19103501	1.92858269	3.82624881	0.00026971	0.00125599	0.10408733	ADO	assignmentsChromaffin_cells.Chrom_SOX2_statusHigh
assignmentsChromaffin_cells.Chrom_SOX2_statusHigh.LIN52	-0.9712449	5.49318809	-3.8794822	0.00022507	0.00108043	0.10204398	LIN52	assignmentsChromaffin_cells.Chrom_SOX2_statusHigh
assignmentsChromaffin_cells.Chrom_SOX2_statusHigh.ANXA11	2.2040956	4.68076765	3.80953737	0.00028558	0.00131993	0.10128725	ANXA11	assignmentsChromaffin_cells.Chrom_SOX2_statusHigh
assignmentsChromaffin_cells.Chrom_SOX2_statusHigh.ALOX15B	3.18969184	-0.0669698	3.74953133	0.00034934	0.00156925	0.09804083	ALOX15B	assignmentsChromaffin_cells.Chrom_SOX2_statusHigh
assignmentsChromaffin_cells.Chrom_SOX2_statusHigh.YEATS4	0.71836969	3.82920629	3.86527328	0.00023624	0.00112295	0.09682575	YEATS4	assignmentsChromaffin_cells.Chrom_SOX2_statusHigh
assignmentsChromaffin_cells.Chrom_SOX2_statusHigh.KRBOX4	-0.7250186	5.15858484	-3.8736544	0.00022959	0.00109949	0.09596516	KRBOX4	assignmentsChromaffin_cells.Chrom_SOX2_statusHigh
assignmentsChromaffin_cells.Chrom_SOX2_statusHigh.TP53RK	0.89997386	3.15779199	3.85420973	0.0002453	0.0011595	0.0944239	TP53RK	assignmentsChromaffin_cells.Chrom_SOX2_statusHigh
assignmentsChromaffin_cells.Chrom_SOX2_statusHigh.TBL3	1.29644751	2.46634064	3.84227259	0.00025545	0.00120146	0.09335468	TBL3	assignmentsChromaffin_cells.Chrom_SOX2_statusHigh
assignmentsChromaffin_cells.Chrom_SOX2_statusHigh.FNTB	-0.880644	4.82331469	-3.869485	0.00023288	0.00111203	0.09311992	FNTB	assignmentsChromaffin_cells.Chrom_SOX2_statusHigh

assignmentsChromaffin_cells.Chrom_SOX2_statusHigh.FAM156A	-0.9280084	3.32804481	-3.8436838	0.00025423	0.00119727	0.09292848	FAM156A	assignmentsChromaffin_cells.Chrom_SOX2_statusHigh
assignmentsChromaffin_cells.Chrom_SOX2_statusHigh.DOP1A	-0.9703502	6.95429151	-3.902999	0.00020769	0.00100907	0.09019434	DOP1A	assignmentsChromaffin_cells.Chrom_SOX2_statusHigh
assignmentsChromaffin_cells.Chrom_SOX2_statusHigh.RGS9	2.36921202	3.46794619	3.86640832	0.00023577	0.00112139	0.08976459	RGS9	assignmentsChromaffin_cells.Chrom_SOX2_statusHigh
assignmentsChromaffin_cells.Chrom_SOX2_statusHigh.NRBF2	1.07749873	4.17197463	3.85991925	0.00024059	0.00114023	0.07745011	NRBF2	assignmentsChromaffin_cells.Chrom_SOX2_statusHigh
assignmentsChromaffin_cells.Chrom_SOX2_statusHigh.NIPBL	-0.6617903	8.53958599	-3.9153396	0.00019909	0.00097289	0.07656263	NIPBL	assignmentsChromaffin_cells.Chrom_SOX2_statusHigh
assignmentsChromaffin_cells.Chrom_SOX2_statusHigh.ANTKMT	1.24114915	4.41286177	3.87887402	0.00022554	0.00108235	0.07413706	ANTKMT	assignmentsChromaffin_cells.Chrom_SOX2_statusHigh
assignmentsChromaffin_cells.Chrom_SOX2_statusHigh.ZC3H6	-0.7433134	6.0453432	-3.8889987	0.00021788	0.00105187	0.0716736	ZC3H6	assignmentsChromaffin_cells.Chrom_SOX2_statusHigh
assignmentsChromaffin_cells.Chrom_SOX2_statusHigh.ACOX3	-0.734615	4.60987162	-3.8585296	0.00024173	0.00114428	0.07091704	ACOX3	assignmentsChromaffin_cells.Chrom_SOX2_statusHigh
assignmentsChromaffin_cells.Chrom_SOX2_statusHigh.NUAK1	3.29221931	3.87294975	3.82318949	0.00027301	0.00126989	0.07061868	NUAK1	assignmentsChromaffin_cells.Chrom_SOX2_statusHigh
assignmentsChromaffin_cells.Chrom_SOX2_statusHigh.PURA	0.72197475	6.26838332	3.89811668	0.00021119	0.00102452	0.06909074	PURA	assignmentsChromaffin_cells.Chrom_SOX2_statusHigh
assignmentsChromaffin_cells.Chrom_SOX2_statusHigh.LOXL1	2.8453367	0.77420469	3.73990174	0.00036061	0.00161459	0.06847293	LOXL1	assignmentsChromaffin_cells.Chrom_SOX2_statusHigh
assignmentsChromaffin_cells.Chrom_SOX2_statusHigh.PPT1	1.02324447	4.48002302	3.85910166	0.00024126	0.00114246	0.06694272	PPT1	assignmentsChromaffin_cells.Chrom_SOX2_statusHigh
assignmentsChromaffin_cells.Chrom_SOX2_statusHigh.CDH20	2.45697816	1.22765366	3.80196088	0.00029319	0.00135083	0.06530341	CDH20	assignmentsChromaffin_cells.Chrom_SOX2_statusHigh
assignmentsChromaffin_cells.Chrom_SOX2_statusHigh.AGA	1.2717483	2.99412589	3.84070286	0.00025682	0.0012061	0.06451765	AGA	assignmentsChromaffin_cells.Chrom_SOX2_statusHigh
assignmentsChromaffin_cells.Chrom_SOX2_statusHigh.MTIF3	0.74483907	5.65361076	3.88532483	0.00022063	0.0010626	0.06152992	MTIF3	assignmentsChromaffin_cells.Chrom_SOX2_statusHigh
assignmentsChromaffin_cells.Chrom_SOX2_statusHigh.IRF3	0.92843458	5.10617862	3.86517408	0.00023632	0.001123	0.0595849	IRF3	assignmentsChromaffin_cells.Chrom_SOX2_statusHigh
assignmentsChromaffin_cells.Chrom_SOX2_statusHigh.NPEPL1	1.62770786	3.31669378	3.78624394	0.00030871	0.00141015	0.05940511	NPEPL1	assignmentsChromaffin_cells.Chrom_SOX2_statusHigh
assignmentsChromaffin_cells.Chrom_SOX2_statusHigh.UTP11	1.26356839	3.65492997	3.84343808	0.00025445	0.00119742	0.0558652	UTP11	assignmentsChromaffin_cells.Chrom_SOX2_statusHigh
assignmentsChromaffin_cells.Chrom_SOX2_statusHigh.C12orf43	0.91900905	3.05560692	3.84134593	0.00025626	0.00120431	0.05452181	C12orf43	assignmentsChromaffin_cells.Chrom_SOX2_statusHigh
assignmentsChromaffin_cells.Chrom_SOX2_statusHigh.ARMC1	0.72121208	4.76715278	3.86877293	0.00023344	0.00111303	0.04889682	ARMC1	assignmentsChromaffin_cells.Chrom_SOX2_statusHigh
assignmentsChromaffin_cells.Chrom_SOX2_statusHigh.TCEAL9	1.78436035	5.16392357	3.88434337	0.00022179	0.00106756	0.04877081	TCEAL9	assignmentsChromaffin_cells.Chrom_SOX2_statusHigh
assignmentsChromaffin_cells.Chrom_SOX2_statusHigh.ZNF689	1.00276898	1.71955794	3.80978524	0.00028515	0.00131833	0.04838417	ZNF689	assignmentsChromaffin_cells.Chrom_SOX2_statusHigh
assignmentsChromaffin_cells.Chrom_SOX2_statusHigh.PDE12	0.88580563	3.47884505	3.84122739	0.00025636	0.00120431	0.0482897	PDE12	assignmentsChromaffin_cells.Chrom_SOX2_statusHigh
assignmentsChromaffin_cells.Chrom_SOX2_statusHigh.VPS4A	0.71194036	4.83165576	3.86055503	0.00024007	0.00113945	0.04387849	VPS4A	assignmentsChromaffin_cells.Chrom_SOX2_statusHigh
assignmentsChromaffin_cells.Chrom_SOX2_statusHigh.DYNC2H1	-1.1787496	7.12207619	-3.8943351	0.00021394	0.00103507	0.04164045	DYNC2H1	assignmentsChromaffin_cells.Chrom_SOX2_statusHigh
assignmentsChromaffin_cells.Chrom_SOX2_statusHigh.CCDC71L	2.11647455	2.10159933	3.79684949	0.00029807	0.00136819	0.0369607	CCDC71L	assignmentsChromaffin_cells.Chrom_SOX2_statusHigh
assignmentsChromaffin_cells.Chrom_SOX2_statusHigh.TRIM45	1.30727492	1.13205663	3.78887382	0.00030599	0.00139931	0.03546963	TRIM45	assignmentsChromaffin_cells.Chrom_SOX2_statusHigh
assignmentsChromaffin_cells.Chrom_SOX2_statusHigh.ARHGAP17	-0.8256411	6.5661676	-3.8687503	0.00023346	0.00111303	0.03385296	ARHGAP17	assignmentsChromaffin_cells.Chrom_SOX2_statusHigh
assignmentsChromaffin_cells.Chrom_SOX2_statusHigh.PPP1R12A	-0.7319025	8.3863872	-3.8976652	0.00021152	0.00102548	0.03322423	PPP1R12A	assignmentsChromaffin_cells.Chrom_SOX2_statusHigh
assignmentsChromaffin_cells.Chrom_SOX2_statusHigh.AUP1	1.38705526	4.68574431	3.86009919	0.00024044	0.00113987	0.02990745	AUP1	assignmentsChromaffin_cells.Chrom_SOX2_statusHigh
assignmentsChromaffin_cells.Chrom_SOX2_statusHigh.EFEMP2	2.09992003	1.87577154	3.74943966	0.00034929	0.00156925	0.02887688	EFEMP2	assignmentsChromaffin_cells.Chrom_SOX2_statusHigh
assignmentsChromaffin_cells.Chrom_SOX2_statusHigh.SNX3	1.39912827	6.21438338	3.88214188	0.00022316	0.00107318	0.02871279	SNX3	assignmentsChromaffin_cells.Chrom_SOX2_statusHigh
assignmentsChromaffin_cells.Chrom_SOX2_statusHigh.SEPTIN10	-0.8156806	5.39436946	-3.8526298	0.00024662	0.00116478	0.0233539	SEPTIN10	assignmentsChromaffin_cells.Chrom_SOX2_statusHigh
assignmentsChromaffin_cells.Chrom_SOX2_statusHigh.CKS2	2.01259254	3.22569088	3.83513873	0.0002621	0.00122695	0.01700764	CKS2	assignmentsChromaffin_cells.Chrom_SOX2_statusHigh
assignmentsChromaffin_cells.Chrom_SOX2_statusHigh.SLC25A21	-1.4553161	3.2806171	-3.8230019	0.00027269	0.00126877	0.01659356	SLC25A21	assignmentsChromaffin_cells.Chrom_SOX2_statusHigh
assignmentsChromaffin_cells.Chrom_SOX2_statusHigh.MAP2K2	1.0523433	4.9865866	3.86162853	0.00023919	0.00113596	0.01469927	MAP2K2	assignmentsChromaffin_cells.Chrom_SOX2_statusHigh
assignmentsChromaffin_cells.Chrom_SOX2_statusHigh.GPN2	0.79283663	3.31519891	3.81833247	0.00027703	0.00128598	0.01437613	GPN2	assignmentsChromaffin_cells.Chrom_SOX2_statusHigh
assignmentsChromaffin_cells.Chrom_SOX2_statusHigh.ZNF337	-0.8944733	5.48659105	-3.8591389	0.00024123	0.00114246	0.01352531	ZNF337	assignmentsChromaffin_cells.Chrom_SOX2_statusHigh
assignmentsChromaffin_cells.Chrom_SOX2_statusHigh.PKNOX1	-0.9597472	5.58862863	-3.8519894	0.00024716	0.00116656	0.01276482	PKNOX1	assignmentsChromaffin_cells.Chrom_SOX2_statusHigh
assignmentsChromaffin_cells.Chrom_SOX2_statusHigh.PTPN21	-1.0590737	4.50437332	-3.8366457	0.00026038	0.00122103	0.00878806	PTPN21	assignmentsChromaffin_cells.Chrom_SOX2_statusHigh
assignmentsChromaffin_cells.Chrom_SOX2_statusHigh.CCSER2	-0.8781557	7.8128034	-3.8855345	0.00022047	0.00106215	0.00682393	CCSER2	assignmentsChromaffin_cells.Chrom_SOX2_statusHigh
assignmentsChromaffin_cells.Chrom_SOX2_statusHigh.NOC4L	1.21432013	2.44676416	3.8115876	0.00028342	0.00131108	0.00660832	NOC4L	assignmentsChromaffin_cells.Chrom_SOX2_statusHigh
assignmentsChromaffin_cells.Chrom_SOX2_statusHigh.VPS45	-0.8685969	6.64215365	-3.8738551	0.00022943	0.00109906	0.00476517	VPS45	assignmentsChromaffin_cells.Chrom_SOX2_statusHigh
assignmentsChromaffin_cells.Chrom_SOX2_statusHigh.TASOR	-0.6807242	6.42205728	-3.8661359	0.00023555	0.00112066	0.00218538	TASOR	assignmentsChromaffin_cells.Chrom_SOX2_statusHigh
assignmentsChromaffin_cells.Chrom_SOX2_statusHigh.DEAF1	-1.0033808	4.75669251	-3.8301769	0.00026615	0.00124264	0.00172233	DEAF1	assignmentsChromaffin_cells.Chrom_SOX2_statusHigh
assignmentsChromaffin_cells.Chrom_SOX2_statusHigh.CYB5A	1.29864003	5.12900635	3.86979905	0.00023263	0.00111138	0.00123777	CYB5A	assignmentsChromaffin_cells.Chrom_SOX2_statusHigh
assignmentsChromaffin_cells.Chrom_SOX2_statusHigh.PAXX	1.97542739	4.19369069	3.83324365	0.00026379	0.00123377	-9.06E-05	PAXX	assignmentsChromaffin_cells.Chrom_SOX2_statusHigh
assignmentsChromaffin_cells.Chrom_SOX2_statusHigh.CAPRIN2	-0.6642022	5.42359835	-3.8540325	0.00024545	0.00115986	-0.000749	CAPRIN2	assignmentsChromaffin_cells.Chrom_SOX2_statusHigh
assignmentsChromaffin_cells.Chrom_SOX2_statusHigh.ARSA	0.9408884	3.60748711	3.82722653	0.00026882	0.00125257	-0.0017653	ARSA	assignmentsChromaffin_cells.Chrom_SOX2_statusHigh
assignmentsChromaffin_cells.Chrom_SOX2_statusHigh.IER5	3.09410316	3.60082367	3.81854226	0.00027734	0.00128701	-0.0025286	IER5	assignmentsChromaffin_cells.Chrom_SOX2_statusHigh
assignmentsChromaffin_cells.Chrom_SOX2_statusHigh.EFHD2	1.75972375	4.27817698	3.83327689	0.00026357	0.00123311	-0.0032119	EFHD2	assignmentsChromaffin_cells.Chrom_SOX2_statusHigh
assignmentsChromaffin_cells.Chrom_SOX2_statusHigh.NIPSNAP1	2.12206566	3.16414926	3.82497927	0.00027136	0.00126296	-0.0033706	NIPSNAP1	assignmentsChromaffin_cells.Chrom_SOX2_statusHigh
assignmentsChromaffin_cells.Chrom_SOX2_statusHigh.SOX9	2.98453687	-0.0907692	3.72116674	0.00038406	0.00170672	-0.0053555	SOX9	assignmentsChromaffin_cells.Chrom_SOX2_statusHigh
assignmentsChromaffin_cells.Chrom_SOX2_statusHigh.S100P	2.7314788	-0.7969135	3.71275775	0.00039477	0.00174563	-0.0071314	S100P	assignmentsChromaffin_cells.Chrom_SOX2_statusHigh
assignmentsChromaffin_cells.Chrom_SOX2_statusHigh.THUMPD3	0.85085831	4.25508987	3.84175528	0.0002559	0.00120321	-0.0083265	THUMPD3	assignmentsChromaffin_cells.Chrom_SOX2_statusHigh
assignmentsChromaffin_cells.Chrom_SOX2_statusHigh.RPL21	2.74326109	7.02593027	3.88866145	0.00021855	0.00105415	-0.0085597	RPL21	assignmentsChromaffin_cells.Chrom_SOX2_statusHigh
assignmentsChromaffin_cells.Chrom_SOX2_statusHigh.PLSCR3	1.3279547	2.69668279	3.79674454	0.00029798	0.00136816	-0.0099288	PLSCR3	assignmentsChromaffin_cells.Chrom_SOX2_statusHigh
assignmentsChromaffin_cells.Chrom_SOX2_statusHigh.DDIT4L	3.85991238	-0.2464712	3.71839467	0.00038808	0.00172075	-0.012489	DDIT4L	assignmentsChromaffin_cells.Chrom_SOX2_statusHigh
assignmentsChromaffin_cells.Chrom_SOX2_statusHigh.DIP2B	-0.9261887	7.70735876	-3.8755153	0.00022814	0.00109417	-0.0141747	DIP2B	assignmentsChromaffin_cells.Chrom_SOX2_statusHigh
assignmentsChromaffin_cells.Chrom_SOX2_statusHigh.SPDYE3	-1.9247413	2.28554883	-3.7836563	0.00031156	0.00142198	-0.019654	SPDYE3	assignmentsChromaffin_cells.Chrom_SOX2_statusHigh
assignmentsChromaffin_cells.Chrom_SOX2_statusHigh.MED11	1.44048117	2.74273913	3.79865423	0.00029607	0.00136023	-0.0209258	MED11	assignmentsChromaffin_cells.Chrom_SOX2_statusHigh
assignmentsChromaffin_cells.Chrom_SOX2_statusHigh.FBXW9	1.09138895	1.24238858	3.78263139	0.00031248	0.00142536	-0.0212961	FBXW9	assignmentsChromaffin_cells.Chrom_SOX2_statusHigh
assignmentsChromaffin_cells.Chrom_SOX2_statusHigh.NEK4	-0.7809119	4.7310062	-3.8310755	0.00026534	0.00123959	-0.0217901	NEK4	assignmentsChromaffin_cells.Chrom_SOX2_statusHigh
assignmentsChromaffin_cells.Chrom_SOX2_statusHigh.NIT1	0.86696714	3.83115469	3.82644225	0.00026954	0.00125554	-0.0225572	NIT1	assignmentsChromaffin_cells.Chrom_SOX2_statusHigh

assignmentsChromaffin_cells.Chrom_SOX2_statusHigh.LRRC10B	4.3066545	-0.8466936	3.7064087	0.00040373	0.0017823	-0.0241447	LRRC10B	assignmentsChromaffin_cells.Chrom_SOX2_statusHigh
assignmentsChromaffin_cells.Chrom_SOX2_statusHigh.SMG7	-0.7046344	6.72515869	-3.860418	0.00024018	0.00113964	-0.0247401	SMG7	assignmentsChromaffin_cells.Chrom_SOX2_statusHigh
assignmentsChromaffin_cells.Chrom_SOX2_statusHigh.CCNYL1	-1.3832143	5.13116537	-3.8210639	0.00027449	0.001276	-0.0256009	CCNYL1	assignmentsChromaffin_cells.Chrom_SOX2_statusHigh
assignmentsChromaffin_cells.Chrom_SOX2_statusHigh.DNAJC17	-0.6435622	4.78067703	-3.8286306	0.00026755	0.00124772	-0.0266246	DNAJC17	assignmentsChromaffin_cells.Chrom_SOX2_statusHigh
assignmentsChromaffin_cells.Chrom_SOX2_statusHigh.B3GLCT	-1.187826	4.62686875	-3.814837	0.00028033	0.00129939	-0.0289189	B3GLCT	assignmentsChromaffin_cells.Chrom_SOX2_statusHigh
assignmentsChromaffin_cells.Chrom_SOX2_statusHigh.RFX7	-0.8548913	7.25624518	-3.8688995	0.00023334	0.00111303	-0.0342061	RFX7	assignmentsChromaffin_cells.Chrom_SOX2_statusHigh
assignmentsChromaffin_cells.Chrom_SOX2_statusHigh.FCHSD1	1.96684701	1.98641877	3.7231525	0.00038134	0.00169605	-0.0365481	FCHSD1	assignmentsChromaffin_cells.Chrom_SOX2_statusHigh
assignmentsChromaffin_cells.Chrom_SOX2_statusHigh.SMG6	-1.0743208	7.15685696	-3.8571991	0.00024282	0.00114913	-0.0401515	SMG6	assignmentsChromaffin_cells.Chrom_SOX2_statusHigh
assignmentsChromaffin_cells.Chrom_SOX2_statusHigh.APLP2	1.3056887	6.86137009	3.86631761	0.0002355	0.00112066	-0.042994	APLP2	assignmentsChromaffin_cells.Chrom_SOX2_statusHigh
assignmentsChromaffin_cells.Chrom_SOX2_statusHigh.IER3IP1	1.24966713	5.58117144	3.86029966	0.00024028	0.00113976	-0.0479035	IER3IP1	assignmentsChromaffin_cells.Chrom_SOX2_statusHigh
assignmentsChromaffin_cells.Chrom_SOX2_statusHigh.PC	-1.0303755	3.48251983	-3.8020181	0.00029273	0.00134946	-0.0499479	PC	assignmentsChromaffin_cells.Chrom_SOX2_statusHigh
assignmentsChromaffin_cells.Chrom_SOX2_statusHigh.GDF10	4.20232996	-0.9429269	3.6961172	0.0004177	0.00183305	-0.0539055	GDF10	assignmentsChromaffin_cells.Chrom_SOX2_statusHigh
assignmentsChromaffin_cells.Chrom_SOX2_statusHigh.KIRREL2	2.07679273	0.13661342	3.75227137	0.00034614	0.00155851	-0.0563455	KIRREL2	assignmentsChromaffin_cells.Chrom_SOX2_statusHigh
assignmentsChromaffin_cells.Chrom_SOX2_statusHigh.AKR1C1	2.71692188	1.85523122	3.76611483	0.00033088	0.00149482	-0.0568564	AKR1C1	assignmentsChromaffin_cells.Chrom_SOX2_statusHigh
assignmentsChromaffin_cells.Chrom_SOX2_statusHigh.AGPAT1	1.10115356	3.63993155	3.81499926	0.00028017	0.00129905	-0.0585538	AGPAT1	assignmentsChromaffin_cells.Chrom_SOX2_statusHigh
assignmentsChromaffin_cells.Chrom_SOX2_statusHigh.ZNF599	1.22188912	2.05493891	3.7902094	0.00030461	0.00139462	-0.0596684	ZNF599	assignmentsChromaffin_cells.Chrom_SOX2_statusHigh
assignmentsChromaffin_cells.Chrom_SOX2_statusHigh.EYA3	-0.779179	5.81931103	-3.8247975	0.00027104	0.00126181	-0.0636395	EYA3	assignmentsChromaffin_cells.Chrom_SOX2_statusHigh
assignmentsChromaffin_cells.Chrom_SOX2_statusHigh.ZNF282	-0.9443307	3.53898222	-3.7958044	0.00029892	0.00137093	-0.0676454	ZNF282	assignmentsChromaffin_cells.Chrom_SOX2_statusHigh
assignmentsChromaffin_cells.Chrom_SOX2_statusHigh.AUTS2	-1.2066885	9.78556114	-3.8946985	0.00021379	0.00103495	-0.0697706	AUTS2	assignmentsChromaffin_cells.Chrom_SOX2_statusHigh
assignmentsChromaffin_cells.Chrom_SOX2_statusHigh.KISS1R	3.16634048	-0.6882713	3.69091931	0.00042464	0.00185975	-0.0711172	KISS1R	assignmentsChromaffin_cells.Chrom_SOX2_statusHigh
assignmentsChromaffin_cells.Chrom_SOX2_statusHigh.MGAT5	-1.1912331	8.2784684	-3.8601309	0.00024041	0.00113987	-0.0716615	MGAT5	assignmentsChromaffin_cells.Chrom_SOX2_statusHigh
assignmentsChromaffin_cells.Chrom_SOX2_statusHigh.LMO4	1.20536155	4.88378493	3.81272314	0.00028234	0.00130644	-0.0740545	LMO4	assignmentsChromaffin_cells.Chrom_SOX2_statusHigh
assignmentsChromaffin_cells.Chrom_SOX2_statusHigh.CNOT9	-0.9053666	5.85084703	-3.8357866	0.00026114	0.00122352	-0.0748166	CNOT9	assignmentsChromaffin_cells.Chrom_SOX2_statusHigh
assignmentsChromaffin_cells.Chrom_SOX2_statusHigh.HTR2B	-2.2120923	1.2369315	-3.7054713	0.00040446	0.00178451	-0.0767843	HTR2B	assignmentsChromaffin_cells.Chrom_SOX2_statusHigh
assignmentsChromaffin_cells.Chrom_SOX2_statusHigh.ZMYM4	-0.7656843	7.03576109	-3.8457043	0.0002525	0.00118999	-0.0774719	ZMYM4	assignmentsChromaffin_cells.Chrom_SOX2_statusHigh
assignmentsChromaffin_cells.Chrom_SOX2_statusHigh.POM121	-0.6770844	6.35562861	-3.8412649	0.00025633	0.00120431	-0.0779115	POM121	assignmentsChromaffin_cells.Chrom_SOX2_statusHigh
assignmentsChromaffin_cells.Chrom_SOX2_statusHigh.CEBPG	1.03988366	4.80831308	3.83215755	0.00026437	0.00123613	-0.0805363	CEBPG	assignmentsChromaffin_cells.Chrom_SOX2_statusHigh
assignmentsChromaffin_cells.Chrom_SOX2_statusHigh.ZFAND2A	2.0001262	3.8091449	3.81125069	0.00028425	0.00131455	-0.0841183	ZFAND2A	assignmentsChromaffin_cells.Chrom_SOX2_statusHigh
assignmentsChromaffin_cells.Chrom_SOX2_statusHigh.TAF1	-0.6685612	6.61208829	-3.8428725	0.00025493	0.00119936	-0.0842821	TAF1	assignmentsChromaffin_cells.Chrom_SOX2_statusHigh
assignmentsChromaffin_cells.Chrom_SOX2_statusHigh.ZHX2	-1.2513114	7.4826334	-3.8434671	0.00025442	0.00119742	-0.0845121	ZHX2	assignmentsChromaffin_cells.Chrom_SOX2_statusHigh
assignmentsChromaffin_cells.Chrom_SOX2_statusHigh.LINC02254	1.85668733	0.04694805	3.70876765	0.00040005	0.00176749	-0.0849131	LINC02254	assignmentsChromaffin_cells.Chrom_SOX2_statusHigh
assignmentsChromaffin_cells.Chrom_SOX2_statusHigh.TUBA1C	1.80184772	4.53480382	3.80607891	0.00028896	0.00133362	-0.0879098	TUBA1C	assignmentsChromaffin_cells.Chrom_SOX2_statusHigh
assignmentsChromaffin_cells.Chrom_SOX2_statusHigh.OXA1L	0.97005782	4.65267997	3.82111138	0.00027444	0.001276	-0.0884109	OXA1L	assignmentsChromaffin_cells.Chrom_SOX2_statusHigh
assignmentsChromaffin_cells.Chrom_SOX2_statusHigh.REXO2	1.24908258	4.89501487	3.81870496	0.00027669	0.00128474	-0.0908172	REXO2	assignmentsChromaffin_cells.Chrom_SOX2_statusHigh
assignmentsChromaffin_cells.Chrom_SOX2_statusHigh.COA5	0.9296082	4.07546066	3.81415936	0.00028097	0.00130162	-0.0962141	COA5	assignmentsChromaffin_cells.Chrom_SOX2_statusHigh
assignmentsChromaffin_cells.Chrom_SOX2_statusHigh.DHX15	-0.6534078	6.77811619	-3.837914	0.00025926	0.0012165	-0.0991923	DHX15	assignmentsChromaffin_cells.Chrom_SOX2_statusHigh
assignmentsChromaffin_cells.Chrom_SOX2_statusHigh.STK32A	-1.6807486	5.42123237	-3.8357956	0.00026149	0.0012248	-0.1020193	STK32A	assignmentsChromaffin_cells.Chrom_SOX2_statusHigh
assignmentsChromaffin_cells.Chrom_SOX2_statusHigh.RASAL1	2.89221704	-0.6254403	3.67923443	0.00044124	0.00192506	-0.1021059	RASAL1	assignmentsChromaffin_cells.Chrom_SOX2_statusHigh
assignmentsChromaffin_cells.Chrom_SOX2_statusHigh.NEK2	2.28824733	-0.219711	3.69297796	0.00042159	0.0018474	-0.1023479	NEK2	assignmentsChromaffin_cells.Chrom_SOX2_statusHigh
assignmentsChromaffin_cells.Chrom_SOX2_statusHigh.OTUD3	-1.1008523	4.40279457	-3.7962675	0.00029846	0.00136947	-0.102842	OTUD3	assignmentsChromaffin_cells.Chrom_SOX2_statusHigh
assignmentsChromaffin_cells.Chrom_SOX2_statusHigh.GAS5	1.32574394	6.24564305	3.84377969	0.00025427	0.00119727	-0.1074088	GAS5	assignmentsChromaffin_cells.Chrom_SOX2_statusHigh
assignmentsChromaffin_cells.Chrom_SOX2_statusHigh.PTGES3	1.35409096	7.1815992	3.8515217	0.00024769	0.00116871	-0.1093045	PTGES3	assignmentsChromaffin_cells.Chrom_SOX2_statusHigh
assignmentsChromaffin_cells.Chrom_SOX2_statusHigh.PHKA2	-0.6261703	4.95910021	-3.806745	0.00028809	0.0013304	-0.1111774	PHKA2	assignmentsChromaffin_cells.Chrom_SOX2_statusHigh
assignmentsChromaffin_cells.Chrom_SOX2_statusHigh.KDM7A-DT	1.82770733	0.48461455	3.71629225	0.00039016	0.00172903	-0.1195552	KDM7A-DT	assignmentsChromaffin_cells.Chrom_SOX2_statusHigh
assignmentsChromaffin_cells.Chrom_SOX2_statusHigh.EBP	1.21825647	2.84845337	3.78491257	0.00031009	0.00141567	-0.1201168	EBP	assignmentsChromaffin_cells.Chrom_SOX2_statusHigh
assignmentsChromaffin_cells.Chrom_SOX2_statusHigh.SOS2	-0.889747	7.27675145	-3.8350741	0.00026177	0.00122576	-0.1209953	SOS2	assignmentsChromaffin_cells.Chrom_SOX2_statusHigh
assignmentsChromaffin_cells.Chrom_SOX2_statusHigh.ZNF446	1.00830156	2.40018363	3.77825655	0.00031711	0.00144238	-0.1240962	ZNF446	assignmentsChromaffin_cells.Chrom_SOX2_statusHigh
assignmentsChromaffin_cells.Chrom_SOX2_statusHigh.BABAM2	-0.6760654	8.20529217	-3.8509276	0.00024806	0.00117009	-0.1246134	BABAM2	assignmentsChromaffin_cells.Chrom_SOX2_statusHigh
assignmentsChromaffin_cells.Chrom_SOX2_statusHigh.INTS1	-0.9708941	4.59421347	-3.7962056	0.00029852	0.00136947	-0.129943	INTS1	assignmentsChromaffin_cells.Chrom_SOX2_statusHigh
assignmentsChromaffin_cells.Chrom_SOX2_statusHigh.NKAPL	2.93264644	0.98982275	3.70543904	0.0004051	0.00178684	-0.1331821	NKAPL	assignmentsChromaffin_cells.Chrom_SOX2_statusHigh
assignmentsChromaffin_cells.Chrom_SOX2_statusHigh.SRSF10	0.70629274	6.68133001	3.8273727	0.00026869	0.00125231	-0.1353157	SRSF10	assignmentsChromaffin_cells.Chrom_SOX2_statusHigh
assignmentsChromaffin_cells.Chrom_SOX2_statusHigh.HSP90AA1	2.49838452	8.91928942	3.86914026	0.00023359	0.00111332	-0.1355099	HSP90AA1	assignmentsChromaffin_cells.Chrom_SOX2_statusHigh
assignmentsChromaffin_cells.Chrom_SOX2_statusHigh.DMXL2	-1.0749938	6.85812142	-3.8315846	0.00026488	0.00123817	-0.1358526	DMXL2	assignmentsChromaffin_cells.Chrom_SOX2_statusHigh
assignmentsChromaffin_cells.Chrom_SOX2_statusHigh.TRAF3IP1	-0.9916224	4.96846585	-3.7991962	0.00029553	0.00135845	-0.1407236	TRAF3IP1	assignmentsChromaffin_cells.Chrom_SOX2_statusHigh
assignmentsChromaffin_cells.Chrom_SOX2_statusHigh.LINC01068	1.97464323	-0.3316506	3.67032653	0.00045443	0.00197458	-0.1423406	LINC01068	assignmentsChromaffin_cells.Chrom_SOX2_statusHigh
assignmentsChromaffin_cells.Chrom_SOX2_statusHigh.IGF2	3.90427652	6.11824523	3.85605705	0.00024422	0.00115507	-0.1463142	IGF2	assignmentsChromaffin_cells.Chrom_SOX2_statusHigh
assignmentsChromaffin_cells.Chrom_SOX2_statusHigh.CASZ1	2.2582718	2.68604082	3.75831195	0.00033964	0.00153227	-0.148776	CASZ1	assignmentsChromaffin_cells.Chrom_SOX2_statusHigh
assignmentsChromaffin_cells.Chrom_SOX2_statusHigh.ATG4B	-0.7119303	5.91628322	-3.8138572	0.00028126	0.00130219	-0.1533068	ATG4B	assignmentsChromaffin_cells.Chrom_SOX2_statusHigh
assignmentsChromaffin_cells.Chrom_SOX2_statusHigh.ROR2	-3.9632296	4.63514995	-3.7622259	0.00033522	0.00151316	-0.1535075	ROR2	assignmentsChromaffin_cells.Chrom_SOX2_statusHigh
assignmentsChromaffin_cells.Chrom_SOX2_statusHigh.XXYLT1	-0.9856237	4.96906412	-3.7896059	0.00030523	0.00139657	-0.1551168	XXYLT1	assignmentsChromaffin_cells.Chrom_SOX2_statusHigh
assignmentsChromaffin_cells.Chrom_SOX2_statusHigh.IMPA1	0.69212822	4.77284598	3.80037722	0.00029435	0.001355	-0.1554851	IMPA1	assignmentsChromaffin_cells.Chrom_SOX2_statusHigh
assignmentsChromaffin_cells.Chrom_SOX2_statusHigh.GTF2I	-0.9073864	7.70695923	-3.8371011	0.00025997	0.0012195	-0.1572964	GTF2I	assignmentsChromaffin_cells.Chrom_SOX2_statusHigh
assignmentsChromaffin_cells.Chrom_SOX2_statusHigh.ZFP36L1	2.34787196	5.77736073	3.73434123	0.00036785	0.00164198	-0.1592106	ZFP36L1	assignmentsChromaffin_cells.Chrom_SOX2_statusHigh
assignmentsChromaffin_cells.Chrom_SOX2_statusHigh.ZNF891	-0.859416	5.10385483	-3.8015877	0.00029315	0.00135083	-0.1597728	ZNF891	assignmentsChromaffin_cells.Chrom_SOX2_statusHigh

assignmentsChromaffin_cells.Chrom_SOX2_statusHigh.ZNF316	-0.8237935	4.04777452	-3.7785624	0.00031679	0.00144202	-0.1602678	ZNF316	assignmentsChromaffin_cells.Chrom_SOX2_statusHigh
assignmentsChromaffin_cells.Chrom_SOX2_statusHigh.TJAP1	-0.6684338	4.23856635	-3.776301	0.0003192	0.00145025	-0.1607734	TJAP1	assignmentsChromaffin_cells.Chrom_SOX2_statusHigh
assignmentsChromaffin_cells.Chrom_SOX2_statusHigh.COG3	-0.6572614	5.85974325	-3.8085156	0.00028638	0.00132323	-0.1621402	COG3	assignmentsChromaffin_cells.Chrom_SOX2_statusHigh
assignmentsChromaffin_cells.Chrom_SOX2_statusHigh.CCDC137	0.85315668	3.9405643	3.77550934	0.00032005	0.00145287	-0.1628617	CCDC137	assignmentsChromaffin_cells.Chrom_SOX2_statusHigh
assignmentsChromaffin_cells.Chrom_SOX2_statusHigh.CDC42-IT1	-2.0565742	0.86884317	-3.6660224	0.00046094	0.00199853	-0.1653824	CDC42-IT1	assignmentsChromaffin_cells.Chrom_SOX2_statusHigh
assignmentsChromaffin_cells.Chrom_SOX2_statusHigh.GRID1	-1.7042317	6.7259513	-3.8304299	0.0002664	0.00124348	-0.1655809	GRID1	assignmentsChromaffin_cells.Chrom_SOX2_statusHigh
assignmentsChromaffin_cells.Chrom_SOX2_statusHigh.FAM8A1	0.76162351	3.79581971	3.78200622	0.00031314	0.00142795	-0.1660492	FAM8A1	assignmentsChromaffin_cells.Chrom_SOX2_statusHigh
assignmentsChromaffin_cells.Chrom_SOX2_statusHigh.GPAM	-1.1052092	4.84333722	-3.7995792	0.00029514	0.00135709	-0.1728579	GPAM	assignmentsChromaffin_cells.Chrom_SOX2_statusHigh
assignmentsChromaffin_cells.Chrom_SOX2_statusHigh.AP4S1	-0.9196555	4.55633423	-3.7689127	0.00032722	0.00148123	-0.1767818	AP4S1	assignmentsChromaffin_cells.Chrom_SOX2_statusHigh
assignmentsChromaffin_cells.Chrom_SOX2_statusHigh.SPA17	1.18784269	1.97833492	3.74197589	0.00035812	0.00160434	-0.1781279	SPA17	assignmentsChromaffin_cells.Chrom_SOX2_statusHigh
assignmentsChromaffin_cells.Chrom_SOX2_statusHigh.ZNF480	0.6435967	4.20393769	3.77835347	0.00031701	0.00144232	-0.180869	ZNF480	assignmentsChromaffin_cells.Chrom_SOX2_statusHigh
assignmentsChromaffin_cells.Chrom_SOX2_statusHigh.PPIH	0.88207979	3.96514139	3.76893927	0.00032719	0.00148123	-0.1829446	PPIH	assignmentsChromaffin_cells.Chrom_SOX2_statusHigh
assignmentsChromaffin_cells.Chrom_SOX2_statusHigh.FAM50B	2.93271293	0.50259922	3.67100139	0.00045372	0.00197201	-0.1831406	FAM50B	assignmentsChromaffin_cells.Chrom_SOX2_statusHigh
assignmentsChromaffin_cells.Chrom_SOX2_statusHigh.STIM1	-1.1098113	6.89811941	-3.8023364	0.00029241	0.00134839	-0.1839426	STIM1	assignmentsChromaffin_cells.Chrom_SOX2_statusHigh
assignmentsChromaffin_cells.Chrom_SOX2_statusHigh.ELN	3.36704033	2.79129645	3.70521937	0.00040546	0.00178745	-0.1854685	ELN	assignmentsChromaffin_cells.Chrom_SOX2_statusHigh
assignmentsChromaffin_cells.Chrom_SOX2_statusHigh.MBLAC2	1.03443327	2.57524145	3.75484881	0.00034302	0.00154577	-0.1865429	MBLAC2	assignmentsChromaffin_cells.Chrom_SOX2_statusHigh
assignmentsChromaffin_cells.Chrom_SOX2_statusHigh.ATG16L1	-0.7583441	4.68841985	-3.7668721	0.00032947	0.00149014	-0.1866683	ATG16L1	assignmentsChromaffin_cells.Chrom_SOX2_statusHigh
assignmentsChromaffin_cells.Chrom_SOX2_statusHigh.ADAM32	1.45829115	3.1012479	3.74715087	0.00035198	0.0015799	-0.1870545	ADAM32	assignmentsChromaffin_cells.Chrom_SOX2_statusHigh
assignmentsChromaffin_cells.Chrom_SOX2_statusHigh.UBXN11	1.05298872	2.94154419	3.74979344	0.00034888	0.00156863	-0.1894162	UBXN11	assignmentsChromaffin_cells.Chrom_SOX2_statusHigh
assignmentsChromaffin_cells.Chrom_SOX2_statusHigh.TEX52	-3.8870855	0.30201886	-3.6488774	0.00048808	0.00209523	-0.189422	TEX52	assignmentsChromaffin_cells.Chrom_SOX2_statusHigh
assignmentsChromaffin_cells.Chrom_SOX2_statusHigh.FAM120AO	0.76242628	4.98211811	3.78968609	0.00030515	0.00139657	-0.1896323	FAM120AOS	assignmentsChromaffin_cells.Chrom_SOX2_statusHigh
assignmentsChromaffin_cells.Chrom_SOX2_statusHigh.INAFM1	1.78761521	2.39278657	3.73408951	0.00036778	0.00164198	-0.1896768	INAFM1	assignmentsChromaffin_cells.Chrom_SOX2_statusHigh
assignmentsChromaffin_cells.Chrom_SOX2_statusHigh.LRRC57	0.75402262	3.67507786	3.76322237	0.00033353	0.00150637	-0.1926875	LRRC57	assignmentsChromaffin_cells.Chrom_SOX2_statusHigh
assignmentsChromaffin_cells.Chrom_SOX2_statusHigh.SP3	-0.7109826	6.64835248	-3.8001301	0.0002946	0.00135574	-0.1955708	SP3	assignmentsChromaffin_cells.Chrom_SOX2_statusHigh
assignmentsChromaffin_cells.Chrom_SOX2_statusHigh.LPIN3	2.2764554	1.19151629	3.67414272	0.00044873	0.00195353	-0.1958577	LPIN3	assignmentsChromaffin_cells.Chrom_SOX2_statusHigh
assignmentsChromaffin_cells.Chrom_SOX2_statusHigh.ZNF792	1.34175903	0.74862979	3.68337891	0.00043522	0.00190038	-0.2000252	ZNF792	assignmentsChromaffin_cells.Chrom_SOX2_statusHigh
assignmentsChromaffin_cells.Chrom_SOX2_statusHigh.STIP1	1.29164654	5.55350491	3.80086777	0.00029387	0.00135315	-0.200125	STIP1	assignmentsChromaffin_cells.Chrom_SOX2_statusHigh
assignmentsChromaffin_cells.Chrom_SOX2_statusHigh.TOMM40	0.97981668	4.20012602	3.78185209	0.0003133	0.00142829	-0.200916	TOMM40	assignmentsChromaffin_cells.Chrom_SOX2_statusHigh
assignmentsChromaffin_cells.Chrom_SOX2_statusHigh.BCL2L12	1.3492305	1.78178531	3.70193735	0.00040924	0.00180262	-0.2015072	BCL2L12	assignmentsChromaffin_cells.Chrom_SOX2_statusHigh
assignmentsChromaffin_cells.Chrom_SOX2_statusHigh.COX10	-1.0488614	5.26866425	-3.7788941	0.00031643	0.00144093	-0.2018717	COX10	assignmentsChromaffin_cells.Chrom_SOX2_statusHigh
assignmentsChromaffin_cells.Chrom_SOX2_statusHigh.ZNF69	-2.6741239	3.71845635	-3.6819209	0.00043764	0.00191042	-0.2041492	ZNF69	assignmentsChromaffin_cells.Chrom_SOX2_statusHigh
assignmentsChromaffin_cells.Chrom_SOX2_statusHigh.PYGO1	-1.0249487	4.09527791	-3.7750814	0.00032051	0.00145455	-0.2088862	PYGO1	assignmentsChromaffin_cells.Chrom_SOX2_statusHigh
assignmentsChromaffin_cells.Chrom_SOX2_statusHigh.SLMAP	-0.779649	7.12415299	-3.8062386	0.00028859	0.00133229	-0.2089237	SLMAP	assignmentsChromaffin_cells.Chrom_SOX2_statusHigh
assignmentsChromaffin_cells.Chrom_SOX2_statusHigh.FAM53B-AS	-2.506073	0.92817752	-3.6440959	0.0004955	0.00211973	-0.2122381	FAM53B-AS1	assignmentsChromaffin_cells.Chrom_SOX2_statusHigh
assignmentsChromaffin_cells.Chrom_SOX2_statusHigh.PIGV	1.18211187	1.57334279	3.71576686	0.00039084	0.00173158	-0.2128129	PIGV	assignmentsChromaffin_cells.Chrom_SOX2_statusHigh
assignmentsChromaffin_cells.Chrom_SOX2_statusHigh.MSH3	-0.7113152	6.57921073	-3.8012618	0.00029347	0.00135174	-0.2133108	MSH3	assignmentsChromaffin_cells.Chrom_SOX2_statusHigh
assignmentsChromaffin_cells.Chrom_SOX2_statusHigh.TIMM22	1.10996316	3.01707997	3.75552947	0.00034224	0.00154268	-0.214273	TIMM22	assignmentsChromaffin_cells.Chrom_SOX2_statusHigh
assignmentsChromaffin_cells.Chrom_SOX2_statusHigh.AGK	-0.7729617	5.54851852	-3.7856396	0.00030933	0.00141261	-0.2143498	AGK	assignmentsChromaffin_cells.Chrom_SOX2_statusHigh
assignmentsChromaffin_cells.Chrom_SOX2_statusHigh.ZNF791	0.6211878	5.537695	3.79507279	0.00029966	0.00137314	-0.2143996	ZNF791	assignmentsChromaffin_cells.Chrom_SOX2_statusHigh
assignmentsChromaffin_cells.Chrom_SOX2_statusHigh.R3HDM2	-1.0792042	7.98035879	-3.8197759	0.00027568	0.00128083	-0.2176302	R3HDM2	assignmentsChromaffin_cells.Chrom_SOX2_statusHigh
assignmentsChromaffin_cells.Chrom_SOX2_statusHigh.TLN1	1.08478064	6.56711157	3.79863966	0.00029608	0.00136023	-0.2188032	TLN1	assignmentsChromaffin_cells.Chrom_SOX2_statusHigh
assignmentsChromaffin_cells.Chrom_SOX2_statusHigh.COMT	0.96628814	5.68253483	3.79568663	0.00029904	0.00137109	-0.2191062	COMT	assignmentsChromaffin_cells.Chrom_SOX2_statusHigh
assignmentsChromaffin_cells.Chrom_SOX2_statusHigh.RGS14	2.32343569	0.69521457	3.64822405	0.00048881	0.00209726	-0.2204809	RGS14	assignmentsChromaffin_cells.Chrom_SOX2_statusHigh
assignmentsChromaffin_cells.Chrom_SOX2_statusHigh.SPG21	0.6001241	5.04488062	3.78097168	0.00031423	0.00143212	-0.2228669	SPG21	assignmentsChromaffin_cells.Chrom_SOX2_statusHigh
assignmentsChromaffin_cells.Chrom_SOX2_statusHigh.PRELP	3.1279401	1.02454604	3.66299371	0.00046623	0.00201928	-0.2247716	PRELP	assignmentsChromaffin_cells.Chrom_SOX2_statusHigh
assignmentsChromaffin_cells.Chrom_SOX2_statusHigh.IRAK4	0.85657244	4.99437187	3.77077083	0.00032519	0.0014741	-0.225885	IRAK4	assignmentsChromaffin_cells.Chrom_SOX2_statusHigh
assignmentsChromaffin_cells.Chrom_SOX2_statusHigh.LEMD3	-0.597741	6.23427724	-3.7875441	0.00030736	0.00140479	-0.2271374	LEMD3	assignmentsChromaffin_cells.Chrom_SOX2_statusHigh
assignmentsChromaffin_cells.Chrom_SOX2_statusHigh.VPS39	-0.8170933	5.67474545	-3.7798695	0.0003154	0.00143703	-0.2288395	VPS39	assignmentsChromaffin_cells.Chrom_SOX2_statusHigh
assignmentsChromaffin_cells.Chrom_SOX2_statusHigh.RNF157	-1.3895163	4.50662727	-3.7656794	0.00033079	0.00149482	-0.2289072	RNF157	assignmentsChromaffin_cells.Chrom_SOX2_statusHigh
assignmentsChromaffin_cells.Chrom_SOX2_statusHigh.AOPEP	-0.8434792	8.21887074	-3.820835	0.0002747	0.00127662	-0.2293644	AOPEP	assignmentsChromaffin_cells.Chrom_SOX2_statusHigh
assignmentsChromaffin_cells.Chrom_SOX2_statusHigh.RAPH1	-1.2277447	6.51517798	-3.7771817	0.00031826	0.00144678	-0.2302696	RAPH1	assignmentsChromaffin_cells.Chrom_SOX2_statusHigh
assignmentsChromaffin_cells.Chrom_SOX2_statusHigh.CUBN	-1.4797557	4.31696264	-3.7421204	0.00035795	0.00160434	-0.2336712	CUBN	assignmentsChromaffin_cells.Chrom_SOX2_statusHigh
assignmentsChromaffin_cells.Chrom_SOX2_statusHigh.TBC1D12	-0.7709379	6.04199169	-3.7784992	0.00031685	0.00144202	-0.2377217	TBC1D12	assignmentsChromaffin_cells.Chrom_SOX2_statusHigh
assignmentsChromaffin_cells.Chrom_SOX2_statusHigh.LTBR	2.21584934	4.03910189	3.74001421	0.00036108	0.00161625	-0.2378128	LTBR	assignmentsChromaffin_cells.Chrom_SOX2_statusHigh
assignmentsChromaffin_cells.Chrom_SOX2_statusHigh.MUC20	-1.4946835	3.23412203	-3.7369909	0.00036413	0.0016281	-0.2422217	MUC20	assignmentsChromaffin_cells.Chrom_SOX2_statusHigh
assignmentsChromaffin_cells.Chrom_SOX2_statusHigh.USP51	2.75709972	1.45905502	3.68878529	0.00042818	0.00187269	-0.2423031	USP51	assignmentsChromaffin_cells.Chrom_SOX2_statusHigh
assignmentsChromaffin_cells.Chrom_SOX2_statusHigh.COA1	-0.7772138	7.10026751	-3.8000136	0.00029471	0.00135588	-0.2493886	COA1	assignmentsChromaffin_cells.Chrom_SOX2_statusHigh
assignmentsChromaffin_cells.Chrom_SOX2_statusHigh.KDM2A	-0.853787	7.59638328	-3.7969972	0.00029773	0.00136739	-0.2503334	KDM2A	assignmentsChromaffin_cells.Chrom_SOX2_statusHigh
assignmentsChromaffin_cells.Chrom_SOX2_statusHigh.ELP3	-0.6637508	5.61492289	-3.7736923	0.00032201	0.00146012	-0.2531185	ELP3	assignmentsChromaffin_cells.Chrom_SOX2_statusHigh
assignmentsChromaffin_cells.Chrom_SOX2_statusHigh.PRDM8	2.98354033	0.48494844	3.63445756	0.00051169	0.00218141	-0.2532254	PRDM8	assignmentsChromaffin_cells.Chrom_SOX2_statusHigh
assignmentsChromaffin_cells.Chrom_SOX2_statusHigh.DGLUCY	-1.1525346	6.08139877	-3.7702984	0.0003257	0.00147602	-0.2538693	DGLUCY	assignmentsChromaffin_cells.Chrom_SOX2_statusHigh
assignmentsChromaffin_cells.Chrom_SOX2_statusHigh.WWP2	-0.9633847	5.87504811	-3.7660407	0.00033039	0.00149388	-0.254082	WWP2	assignmentsChromaffin_cells.Chrom_SOX2_statusHigh
assignmentsChromaffin_cells.Chrom_SOX2_statusHigh.FAM217A	-2.8511753	1.07941181	-3.6370586	0.00050733	0.00216514	-0.2543056	FAM217A	assignmentsChromaffin_cells.Chrom_SOX2_statusHigh
assignmentsChromaffin_cells.Chrom_SOX2_statusHigh.PEAK1	-1.096458	8.09494944	-3.7879892	0.0003069	0.00140309	-0.2556879	PEAK1	assignmentsChromaffin_cells.Chrom_SOX2_statusHigh

assignmentsChromaffin_cells.Chrom_SOX2_statusHigh.NBEAL1	-0.8537978	7.67698238	-3.7939451	0.0003008	0.00137797	-0.2580811	NBEAL1	assignmentsChromaffin_cells.Chrom_SOX2_statusHigh
assignmentsChromaffin_cells.Chrom_SOX2_statusHigh.WWC3	-0.8697006	5.23178538	-3.7420292	0.00035806	0.00160434	-0.2603774	WWC3	assignmentsChromaffin_cells.Chrom_SOX2_statusHigh
assignmentsChromaffin_cells.Chrom_SOX2_statusHigh.GLYR1	-0.5346017	6.32110443	-3.7828935	0.00031221	0.00142451	-0.2610647	GLYR1	assignmentsChromaffin_cells.Chrom_SOX2_statusHigh
assignmentsChromaffin_cells.Chrom_SOX2_statusHigh.FKBP10	2.64048032	4.38701826	3.77044954	0.0003261	0.0014774	-0.2634725	FKBP10	assignmentsChromaffin_cells.Chrom_SOX2_statusHigh
assignmentsChromaffin_cells.Chrom_SOX2_statusHigh.PAIP2	0.82528573	6.24750219	3.78954037	0.0003053	0.00139657	-0.2663426	PAIP2	assignmentsChromaffin_cells.Chrom_SOX2_statusHigh
assignmentsChromaffin_cells.Chrom_SOX2_statusHigh.TOMM70	0.73553483	5.25378909	3.77580713	0.00031973	0.00145183	-0.2722328	TOMM70	assignmentsChromaffin_cells.Chrom_SOX2_statusHigh
assignmentsChromaffin_cells.Chrom_SOX2_statusHigh.UNK	-0.6448759	5.99633598	-3.7774788	0.00031794	0.00144575	-0.2723169	UNK	assignmentsChromaffin_cells.Chrom_SOX2_statusHigh
assignmentsChromaffin_cells.Chrom_SOX2_statusHigh.CREB1	-0.7126526	6.69777496	-3.775945	0.00031958	0.00145157	-0.2730188	CREB1	assignmentsChromaffin_cells.Chrom_SOX2_statusHigh
assignmentsChromaffin_cells.Chrom_SOX2_statusHigh.LAP3	1.11462078	4.85574946	3.74933924	0.00034941	0.00156925	-0.2755239	LAP3	assignmentsChromaffin_cells.Chrom_SOX2_statusHigh
assignmentsChromaffin_cells.Chrom_SOX2_statusHigh.SEPTIN8	0.64136833	3.94866213	3.74954349	0.00034917	0.00156925	-0.2763217	SEPTIN8	assignmentsChromaffin_cells.Chrom_SOX2_statusHigh
assignmentsChromaffin_cells.Chrom_SOX2_statusHigh.ZNF578	-1.5168174	2.71424311	-3.7198431	0.00038557	0.00171106	-0.2767353	ZNF578	assignmentsChromaffin_cells.Chrom_SOX2_statusHigh
assignmentsChromaffin_cells.Chrom_SOX2_statusHigh.SFRP1	3.50810214	1.73947754	3.68088152	0.00043954	0.00191817	-0.2767857	SFRP1	assignmentsChromaffin_cells.Chrom_SOX2_statusHigh
assignmentsChromaffin_cells.Chrom_SOX2_statusHigh.BTBD10	-0.8126017	6.07915321	-3.7676291	0.00032863	0.00148678	-0.2772199	BTBD10	assignmentsChromaffin_cells.Chrom_SOX2_statusHigh
assignmentsChromaffin_cells.Chrom_SOX2_statusHigh.ARMC8	-0.664328	6.51236247	-3.7766609	0.00031882	0.0014489	-0.2783043	ARMC8	assignmentsChromaffin_cells.Chrom_SOX2_statusHigh
assignmentsChromaffin_cells.Chrom_SOX2_statusHigh.SLC38A11	3.35469441	2.53373902	3.66972218	0.00045606	0.00198111	-0.2787179	SLC38A11	assignmentsChromaffin_cells.Chrom_SOX2_statusHigh
assignmentsChromaffin_cells.Chrom_SOX2_statusHigh.ZNF461	-0.8750124	3.83731317	-3.7327353	0.00036935	0.00164773	-0.2846977	ZNF461	assignmentsChromaffin_cells.Chrom_SOX2_statusHigh
assignmentsChromaffin_cells.Chrom_SOX2_statusHigh.DNMBP-AS:	-2.4271825	1.27668291	-3.6209623	0.00053463	0.00226717	-0.292759	DNMBP-AS1	assignmentsChromaffin_cells.Chrom_SOX2_statusHigh
assignmentsChromaffin_cells.Chrom_SOX2_statusHigh.BRF1	-0.9338406	5.07675466	-3.7500483	0.00034858	0.00156773	-0.2963637	BRF1	assignmentsChromaffin_cells.Chrom_SOX2_statusHigh
assignmentsChromaffin_cells.Chrom_SOX2_statusHigh.MXRA7	1.49790347	5.01924997	3.76968184	0.00032659	0.0014792	-0.2999252	MXRA7	assignmentsChromaffin_cells.Chrom_SOX2_statusHigh
assignmentsChromaffin_cells.Chrom_SOX2_statusHigh.TTC7B	-1.2504418	6.2901441	-3.7566323	0.00034098	0.00153742	-0.3051377	TTC7B	assignmentsChromaffin_cells.Chrom_SOX2_statusHigh
assignmentsChromaffin_cells.Chrom_SOX2_statusHigh.GET1	0.74985094	4.59898321	3.75306379	0.00034508	0.00155416	-0.3074698	GET1	assignmentsChromaffin_cells.Chrom_SOX2_statusHigh
assignmentsChromaffin_cells.Chrom_SOX2_statusHigh.PRPF39	-0.8128758	5.6985369	-3.757258	0.00034026	0.00153463	-0.312244	PRPF39	assignmentsChromaffin_cells.Chrom_SOX2_statusHigh
assignmentsChromaffin_cells.Chrom_SOX2_statusHigh.ABCB10	-1.0288972	4.61980507	-3.7282766	0.00037488	0.00166964	-0.3131086	ABCB10	assignmentsChromaffin_cells.Chrom_SOX2_statusHigh
assignmentsChromaffin_cells.Chrom_SOX2_statusHigh.SOCS3	3.33586911	3.66583971	3.65938015	0.0004719	0.00203997	-0.315548	SOCS3	assignmentsChromaffin_cells.Chrom_SOX2_statusHigh
assignmentsChromaffin_cells.Chrom_SOX2_statusHigh.AVPI1	1.55877102	2.69465166	3.70641335	0.00040319	0.0017804	-0.3155836	AVPI1	assignmentsChromaffin_cells.Chrom_SOX2_statusHigh
assignmentsChromaffin_cells.Chrom_SOX2_statusHigh.ING2	1.57512461	2.68211647	3.7133623	0.00039398	0.00174309	-0.3166185	ING2	assignmentsChromaffin_cells.Chrom_SOX2_statusHigh
assignmentsChromaffin_cells.Chrom_SOX2_statusHigh.MTMR2	-0.7689327	5.75495028	-3.7506523	0.00034788	0.00156588	-0.3188643	MTMR2	assignmentsChromaffin_cells.Chrom_SOX2_statusHigh
assignmentsChromaffin_cells.Chrom_SOX2_statusHigh.ABCC10	-0.817089	4.1241855	-3.7207394	0.00038442	0.00170752	-0.3189454	ABCC10	assignmentsChromaffin_cells.Chrom_SOX2_statusHigh
assignmentsChromaffin_cells.Chrom_SOX2_statusHigh.HR	2.12469971	0.63549776	3.65325055	0.00048094	0.0020719	-0.3194049	HR	assignmentsChromaffin_cells.Chrom_SOX2_statusHigh
assignmentsChromaffin_cells.Chrom_SOX2_statusHigh.DNLZ	2.82753043	1.56409136	3.66830635	0.0004582	0.00198819	-0.3243843	DNLZ	assignmentsChromaffin_cells.Chrom_SOX2_statusHigh
assignmentsChromaffin_cells.Chrom_SOX2_statusHigh.WDR54	1.21648517	2.86276308	3.72545571	0.00037843	0.00168402	-0.3247606	WDR54	assignmentsChromaffin_cells.Chrom_SOX2_statusHigh
assignmentsChromaffin_cells.Chrom_SOX2_statusHigh.DDAH2	1.55688681	3.6738902	3.69324029	0.00042122	0.00184629	-0.3266755	DDAH2	assignmentsChromaffin_cells.Chrom_SOX2_statusHigh
assignmentsChromaffin_cells.Chrom_SOX2_statusHigh.LTBP4	1.93025651	3.9805075	3.71421558	0.00039325	0.00174081	-0.3308967	LTBP4	assignmentsChromaffin_cells.Chrom_SOX2_statusHigh
assignmentsChromaffin_cells.Chrom_SOX2_statusHigh.RFC4	0.91299	2.72435638	3.70472795	0.00040546	0.00178745	-0.3317692	RFC4	assignmentsChromaffin_cells.Chrom_SOX2_statusHigh
assignmentsChromaffin_cells.Chrom_SOX2_statusHigh.PRORP	-0.7933886	5.87872257	-3.7418094	0.00035832	0.00160478	-0.3343871	PRORP	assignmentsChromaffin_cells.Chrom_SOX2_statusHigh
assignmentsChromaffin_cells.Chrom_SOX2_statusHigh.MRPL16	1.18646153	4.1382624	3.73299549	0.00036903	0.00164675	-0.3361628	MRPL16	assignmentsChromaffin_cells.Chrom_SOX2_statusHigh
assignmentsChromaffin_cells.Chrom_SOX2_statusHigh.HPCAL1	2.10417548	5.00075534	3.69900637	0.00041377	0.00181896	-0.337816	HPCAL1	assignmentsChromaffin_cells.Chrom_SOX2_statusHigh
assignmentsChromaffin_cells.Chrom_SOX2_statusHigh.CYBC1	0.93090102	4.37656337	3.72071556	0.00038445	0.00170752	-0.3410893	CYBC1	assignmentsChromaffin_cells.Chrom_SOX2_statusHigh
assignmentsChromaffin_cells.Chrom_SOX2_statusHigh.KLHDC1	-0.9380093	5.20977041	-3.7391779	0.00036148	0.0016176	-0.3420877	KLHDC1	assignmentsChromaffin_cells.Chrom_SOX2_statusHigh
assignmentsChromaffin_cells.Chrom_SOX2_statusHigh.ZNF468	1.11522974	1.77146454	3.67136674	0.00045287	0.00196993	-0.3472493	ZNF468	assignmentsChromaffin_cells.Chrom_SOX2_statusHigh
assignmentsChromaffin_cells.Chrom_SOX2_statusHigh.PHF14	-0.7716384	7.6489543	-3.7745794	0.00032105	0.0014566	-0.3496456	PHF14	assignmentsChromaffin_cells.Chrom_SOX2_statusHigh
assignmentsChromaffin_cells.Chrom_SOX2_statusHigh.SIL1	-0.6728155	6.95510927	-3.7659328	0.00033051	0.001494	-0.351851	SIL1	assignmentsChromaffin_cells.Chrom_SOX2_statusHigh
assignmentsChromaffin_cells.Chrom_SOX2_statusHigh.MTX2	-0.656235	6.37557247	-3.762517	0.00033432	0.00150952	-0.353431	MTX2	assignmentsChromaffin_cells.Chrom_SOX2_statusHigh
assignmentsChromaffin_cells.Chrom_SOX2_statusHigh.SPN	2.52833999	1.00004295	3.60046037	0.00057175	0.00240424	-0.355636	SPN	assignmentsChromaffin_cells.Chrom_SOX2_statusHigh
assignmentsChromaffin_cells.Chrom_SOX2_statusHigh.TM7SF2	1.50508709	2.59219333	3.71788484	0.0003882	0.00172081	-0.3601907	TM7SF2	assignmentsChromaffin_cells.Chrom_SOX2_statusHigh
assignmentsChromaffin_cells.Chrom_SOX2_statusHigh.ISCA1	0.83852329	4.78898542	3.73656769	0.00036465	0.00162995	-0.3627425	ISCA1	assignmentsChromaffin_cells.Chrom_SOX2_statusHigh
assignmentsChromaffin_cells.Chrom_SOX2_statusHigh.PKP4-AS1	-1.9179434	0.96322197	-3.6195731	0.00053707	0.00227572	-0.3635198	PKP4-AS1	assignmentsChromaffin_cells.Chrom_SOX2_statusHigh
assignmentsChromaffin_cells.Chrom_SOX2_statusHigh.RNF185	-0.7638397	5.10121843	-3.7292582	0.00037366	0.00166464	-0.3639925	RNF185	assignmentsChromaffin_cells.Chrom_SOX2_statusHigh
assignmentsChromaffin_cells.Chrom_SOX2_statusHigh.CRLF3	-1.2185138	5.91763616	-3.7317059	0.00037062	0.00165202	-0.3731791	CRLF3	assignmentsChromaffin_cells.Chrom_SOX2_statusHigh
assignmentsChromaffin_cells.Chrom_SOX2_statusHigh.CD99	1.24925195	6.81210661	3.75037393	0.0003482	0.0015669	-0.3734318	CD99	assignmentsChromaffin_cells.Chrom_SOX2_statusHigh
assignmentsChromaffin_cells.Chrom_SOX2_statusHigh.TMLHE	-0.9906258	5.78149366	-3.7371487	0.00036394	0.0016281	-0.3765615	TMLHE	assignmentsChromaffin_cells.Chrom_SOX2_statusHigh
assignmentsChromaffin_cells.Chrom_SOX2_statusHigh.FHIT	-1.2549864	8.29875436	-3.7685414	0.00032777	0.00148329	-0.3806865	FHIT	assignmentsChromaffin_cells.Chrom_SOX2_statusHigh
assignmentsChromaffin_cells.Chrom_SOX2_statusHigh.PLA2R1	2.56803981	2.67082809	3.65187803	0.00048371	0.00207937	-0.3807957	PLA2R1	assignmentsChromaffin_cells.Chrom_SOX2_statusHigh
assignmentsChromaffin_cells.Chrom_SOX2_statusHigh.TMEM121	1.22611957	0.99666458	3.65809468	0.00047316	0.00204324	-0.381646	TMEM121	assignmentsChromaffin_cells.Chrom_SOX2_statusHigh
assignmentsChromaffin_cells.Chrom_SOX2_statusHigh.SOX7	2.28312044	1.63110565	3.65248395	0.00048235	0.00207454	-0.3826688	SOX7	assignmentsChromaffin_cells.Chrom_SOX2_statusHigh
assignmentsChromaffin_cells.Chrom_SOX2_statusHigh.SLC39A13	1.11757375	3.30174421	3.69638708	0.00041685	0.00183062	-0.3876328	SLC39A13	assignmentsChromaffin_cells.Chrom_SOX2_statusHigh
assignmentsChromaffin_cells.Chrom_SOX2_statusHigh.SRRM1	0.77555373	6.82460039	3.75017722	0.00034843	0.00156749	-0.3892446	SRRM1	assignmentsChromaffin_cells.Chrom_SOX2_statusHigh
assignmentsChromaffin_cells.Chrom_SOX2_statusHigh.ZNF140	-0.600821	4.81050604	-3.7244073	0.00037975	0.00168944	-0.3892468	ZNF140	assignmentsChromaffin_cells.Chrom_SOX2_statusHigh
assignmentsChromaffin_cells.Chrom_SOX2_statusHigh.ABHD15	1.19244346	1.85356811	3.64438199	0.00049504	0.0021183	-0.3969919	ABHD15	assignmentsChromaffin_cells.Chrom_SOX2_statusHigh
assignmentsChromaffin_cells.Chrom_SOX2_statusHigh.UPP1	1.5654627	5.24095031	3.71523759	0.00039173	0.00173454	-0.3981669	UPP1	assignmentsChromaffin_cells.Chrom_SOX2_statusHigh
assignmentsChromaffin_cells.Chrom_SOX2_statusHigh.RRAS	1.73205235	2.51456431	3.67119573	0.00045329	0.00197121	-0.3990286	RRAS	assignmentsChromaffin_cells.Chrom_SOX2_statusHigh
assignmentsChromaffin_cells.Chrom_SOX2_statusHigh.EIF1AD	0.96621599	2.96315163	3.68640422	0.00043088	0.00188347	-0.401542	EIF1AD	assignmentsChromaffin_cells.Chrom_SOX2_statusHigh
assignmentsChromaffin_cells.Chrom_SOX2_statusHigh.JUN	2.67609038	7.01963103	3.74410643	0.00035618	0.00159698	-0.40406	JUN	assignmentsChromaffin_cells.Chrom_SOX2_statusHigh
assignmentsChromaffin_cells.Chrom_SOX2_statusHigh.THOC7	0.83341404	4.76554999	3.72108746	0.00038398	0.00170672	-0.4076654	THOC7	assignmentsChromaffin_cells.Chrom_SOX2_statusHigh

assignmentsChromaffin_cells.Chrom_SOX2_statusHigh.KHK	1.25933356	1.11328266	3.6599648	0.00047025	0.00203395	-0.407761	KHK	assignmentsChromaffin_cells.Chrom_SOX2_statusHigh
assignmentsChromaffin_cells.Chrom_SOX2_statusHigh.GABRA1	-3.2263982	-0.1882456	-3.5791238	0.00061335	0.00254973	-0.4085246	GABRA1	assignmentsChromaffin_cells.Chrom_SOX2_statusHigh
assignmentsChromaffin_cells.Chrom_SOX2_statusHigh.PIBF1	-0.7215957	7.54551483	-3.7533266	0.00034477	0.00155323	-0.4093321	PIBF1	assignmentsChromaffin_cells.Chrom_SOX2_statusHigh
assignmentsChromaffin_cells.Chrom_SOX2_statusHigh.ZNF592	-0.8107313	5.63054774	-3.7229923	0.00038155	0.00169649	-0.4100743	ZNF592	assignmentsChromaffin_cells.Chrom_SOX2_statusHigh
assignmentsChromaffin_cells.Chrom_SOX2_statusHigh.PKN2	-0.6578654	7.50346858	-3.7449993	0.00035452	0.00159087	-0.4115651	PKN2	assignmentsChromaffin_cells.Chrom_SOX2_statusHigh
assignmentsChromaffin_cells.Chrom_SOX2_statusHigh.MLLT3	-1.0405944	6.92094621	-3.7446016	0.00035499	0.00159254	-0.4119089	MLLT3	assignmentsChromaffin_cells.Chrom_SOX2_statusHigh
assignmentsChromaffin_cells.Chrom_SOX2_statusHigh.LBH	2.55770284	3.84546796	3.63400664	0.00051297	0.00218629	-0.4173571	LBH	assignmentsChromaffin_cells.Chrom_SOX2_statusHigh
assignmentsChromaffin_cells.Chrom_SOX2_statusHigh.PAWR	-1.6146482	5.39043446	-3.7134486	0.00039414	0.0017433	-0.4195806	PAWR	assignmentsChromaffin_cells.Chrom_SOX2_statusHigh
assignmentsChromaffin_cells.Chrom_SOX2_statusHigh.ZNF793	-0.9825686	4.14594058	-3.6985595	0.00041385	0.00181896	-0.4221335	ZNF793	assignmentsChromaffin_cells.Chrom_SOX2_statusHigh
assignmentsChromaffin_cells.Chrom_SOX2_statusHigh.CDC40	0.7043326	5.63301791	3.72606276	0.00037766	0.00168154	-0.4240209	CDC40	assignmentsChromaffin_cells.Chrom_SOX2_statusHigh
assignmentsChromaffin_cells.Chrom_SOX2_statusHigh.MAP4K3-DT	-0.7829371	6.15291871	-3.7354106	0.00036606	0.00163554	-0.4247887	MAP4K3-DT	assignmentsChromaffin_cells.Chrom_SOX2_statusHigh
assignmentsChromaffin_cells.Chrom_SOX2_statusHigh.GAPVD1	-0.7483898	6.79332531	-3.7353752	0.00036611	0.00163554	-0.4251578	GAPVD1	assignmentsChromaffin_cells.Chrom_SOX2_statusHigh
assignmentsChromaffin_cells.Chrom_SOX2_statusHigh.TTC39C-AS1	1.819198	0.12247954	3.6119316	0.00055069	0.00232788	-0.4272257	TTC39C-AS1	assignmentsChromaffin_cells.Chrom_SOX2_statusHigh
assignmentsChromaffin_cells.Chrom_SOX2_statusHigh.C11orf71	1.14671274	2.40542046	3.67468891	0.00044792	0.00195052	-0.4275532	C11orf71	assignmentsChromaffin_cells.Chrom_SOX2_statusHigh
assignmentsChromaffin_cells.Chrom_SOX2_statusHigh.PDP1	1.27461038	4.03222099	3.6994508	0.00041263	0.00181557	-0.4302001	PDP1	assignmentsChromaffin_cells.Chrom_SOX2_statusHigh
assignmentsChromaffin_cells.Chrom_SOX2_statusHigh.ZER1	-0.990678	5.38208159	-3.7155458	0.00039113	0.00173237	-0.4310025	ZER1	assignmentsChromaffin_cells.Chrom_SOX2_statusHigh
assignmentsChromaffin_cells.Chrom_SOX2_statusHigh.DCTN2	0.70745841	5.9912924	3.73699152	0.00036413	0.0016281	-0.4316496	DCTN2	assignmentsChromaffin_cells.Chrom_SOX2_statusHigh
assignmentsChromaffin_cells.Chrom_SOX2_statusHigh.GRAMD1A	0.66901377	5.35776492	3.7134678	0.00039384	0.00174295	-0.4317915	GRAMD1A	assignmentsChromaffin_cells.Chrom_SOX2_statusHigh
assignmentsChromaffin_cells.Chrom_SOX2_statusHigh.TMEM51-AS	-1.5679507	3.24800422	-3.6732081	0.00045012	0.00195904	-0.432499	TMEM51-AS	assignmentsChromaffin_cells.Chrom_SOX2_statusHigh
assignmentsChromaffin_cells.Chrom_SOX2_statusHigh.NANOS3	1.74571623	0.67117492	3.6085705	0.00055678	0.00235054	-0.4339738	NANOS3	assignmentsChromaffin_cells.Chrom_SOX2_statusHigh
assignmentsChromaffin_cells.Chrom_SOX2_statusHigh.IFITM3	2.31392101	4.59360558	3.65314822	0.00048169	0.00207337	-0.434273	IFITM3	assignmentsChromaffin_cells.Chrom_SOX2_statusHigh
assignmentsChromaffin_cells.Chrom_SOX2_statusHigh.EEF1AKMT4	1.17946697	1.64353982	3.6568703	0.00047508	0.0020493	-0.43559	EEF1AKMT4	assignmentsChromaffin_cells.Chrom_SOX2_statusHigh
assignmentsChromaffin_cells.Chrom_SOX2_statusHigh.TBC1D30	-1.6183323	5.12181739	-3.6999123	0.00041225	0.00181439	-0.437182	TBC1D30	assignmentsChromaffin_cells.Chrom_SOX2_statusHigh
assignmentsChromaffin_cells.Chrom_SOX2_statusHigh.CELF1	-0.9221193	7.36670432	-3.7324955	0.00036964	0.00164813	-0.4377935	CELF1	assignmentsChromaffin_cells.Chrom_SOX2_statusHigh
assignmentsChromaffin_cells.Chrom_SOX2_statusHigh.ST3GAL3	-0.9031692	6.30958696	-3.720373	0.00038489	0.00170852	-0.4387627	ST3GAL3	assignmentsChromaffin_cells.Chrom_SOX2_statusHigh
assignmentsChromaffin_cells.Chrom_SOX2_statusHigh.USP33	-0.5698228	6.83892175	-3.7304862	0.00037213	0.00165829	-0.4397692	USP33	assignmentsChromaffin_cells.Chrom_SOX2_statusHigh
assignmentsChromaffin_cells.Chrom_SOX2_statusHigh.RRAGC	0.80656787	3.8688544	3.68832553	0.00042815	0.00187269	-0.4406485	RRAGC	assignmentsChromaffin_cells.Chrom_SOX2_statusHigh
assignmentsChromaffin_cells.Chrom_SOX2_statusHigh.MPZ	2.05045647	1.62886767	3.60506199	0.00056321	0.00237269	-0.4425005	MPZ	assignmentsChromaffin_cells.Chrom_SOX2_statusHigh
assignmentsChromaffin_cells.Chrom_SOX2_statusHigh.MAP4	-0.9179883	8.26222315	-3.7473909	0.00035169	0.00157907	-0.4426336	MAP4	assignmentsChromaffin_cells.Chrom_SOX2_statusHigh
assignmentsChromaffin_cells.Chrom_SOX2_statusHigh.CYSTM1	0.90595545	6.78234041	3.74396	0.00035575	0.00159551	-0.4429313	CYSTM1	assignmentsChromaffin_cells.Chrom_SOX2_statusHigh
assignmentsChromaffin_cells.Chrom_SOX2_statusHigh.CSNK1G1	-0.7922244	6.59353371	-3.7254599	0.00037842	0.00168402	-0.4430622	CSNK1G1	assignmentsChromaffin_cells.Chrom_SOX2_statusHigh
assignmentsChromaffin_cells.Chrom_SOX2_statusHigh.AMZ2	1.2620125	4.67128638	3.7008273	0.00041075	0.00180828	-0.4438192	AMZ2	assignmentsChromaffin_cells.Chrom_SOX2_statusHigh
assignmentsChromaffin_cells.Chrom_SOX2_statusHigh.ARID1B	-0.8382349	9.13339829	-3.7579807	0.00033944	0.00153178	-0.4458652	ARID1B	assignmentsChromaffin_cells.Chrom_SOX2_statusHigh
assignmentsChromaffin_cells.Chrom_SOX2_statusHigh.SYT11	0.79276794	5.63492862	3.73251467	0.00036962	0.00164813	-0.4470708	SYT11	assignmentsChromaffin_cells.Chrom_SOX2_statusHigh
assignmentsChromaffin_cells.Chrom_SOX2_statusHigh.BHMT2	2.48771425	1.13239287	3.58699146	0.00059771	0.00249637	-0.4518875	BHMT2	assignmentsChromaffin_cells.Chrom_SOX2_statusHigh
assignmentsChromaffin_cells.Chrom_SOX2_statusHigh.PRRT3	0.94750009	1.76027545	3.65233212	0.00048224	0.00207454	-0.4518948	PRRT3	assignmentsChromaffin_cells.Chrom_SOX2_statusHigh
assignmentsChromaffin_cells.Chrom_SOX2_statusHigh.MROH6	-2.1755771	1.0810778	-3.5793605	0.0006125	0.00254754	-0.4525475	MROH6	assignmentsChromaffin_cells.Chrom_SOX2_statusHigh
assignmentsChromaffin_cells.Chrom_SOX2_statusHigh.LINC00327	2.46332725	0.40498188	3.57117972	0.00062903	0.00260347	-0.4553148	LINC00327	assignmentsChromaffin_cells.Chrom_SOX2_statusHigh
assignmentsChromaffin_cells.Chrom_SOX2_statusHigh.CFAP157	1.81802618	-0.0922583	3.59274273	0.00058635	0.00245788	-0.4573707	CFAP157	assignmentsChromaffin_cells.Chrom_SOX2_statusHigh
assignmentsChromaffin_cells.Chrom_SOX2_statusHigh.RNASEH1	0.61026783	4.35470002	3.69348431	0.00042088	0.0018458	-0.4611641	RNASEH1	assignmentsChromaffin_cells.Chrom_SOX2_statusHigh
assignmentsChromaffin_cells.Chrom_SOX2_statusHigh.ZNF782	-0.8178135	5.45068848	-3.7030876	0.00040768	0.00179623	-0.4630792	ZNF782	assignmentsChromaffin_cells.Chrom_SOX2_statusHigh
assignmentsChromaffin_cells.Chrom_SOX2_statusHigh.PRSS48	-2.5461778	0.84587938	-3.5772182	0.00061705	0.00256313	-0.4643351	PRSS48	assignmentsChromaffin_cells.Chrom_SOX2_statusHigh
assignmentsChromaffin_cells.Chrom_SOX2_statusHigh.SLC43A3	2.13415285	2.53293106	3.55614303	0.00066052	0.00270656	-0.4663279	SLC43A3	assignmentsChromaffin_cells.Chrom_SOX2_statusHigh
assignmentsChromaffin_cells.Chrom_SOX2_statusHigh.GALNT7	-1.0445152	5.8815789	-3.6992047	0.00041297	0.00181606	-0.4666216	GALNT7	assignmentsChromaffin_cells.Chrom_SOX2_statusHigh
assignmentsChromaffin_cells.Chrom_SOX2_statusHigh.JAKMIP2-AS	-2.1665222	2.16923434	-3.6366123	0.00050828	0.00216805	-0.4670169	JAKMIP2-AS1	assignmentsChromaffin_cells.Chrom_SOX2_statusHigh
assignmentsChromaffin_cells.Chrom_SOX2_statusHigh.CENPB	1.35897408	2.73457084	3.66077307	0.000469	0.00202963	-0.4672029	CENPB	assignmentsChromaffin_cells.Chrom_SOX2_statusHigh
assignmentsChromaffin_cells.Chrom_SOX2_statusHigh.SENP1	-0.5963872	5.08330677	-3.6934028	0.000421	0.0018458	-0.4707176	SENP1	assignmentsChromaffin_cells.Chrom_SOX2_statusHigh
assignmentsChromaffin_cells.Chrom_SOX2_statusHigh.TRIM37	-0.6756058	6.06889551	-3.7188144	0.00038689	0.00171599	-0.4722628	TRIM37	assignmentsChromaffin_cells.Chrom_SOX2_statusHigh
assignmentsChromaffin_cells.Chrom_SOX2_statusHigh.CLDN12	-0.8162409	5.41358477	-3.7188438	0.00038686	0.00171599	-0.4727599	CLDN12	assignmentsChromaffin_cells.Chrom_SOX2_statusHigh
assignmentsChromaffin_cells.Chrom_SOX2_statusHigh.ARPP19	0.84123524	5.91918965	3.72058508	0.00038462	0.00170779	-0.4730639	ARPP19	assignmentsChromaffin_cells.Chrom_SOX2_statusHigh
assignmentsChromaffin_cells.Chrom_SOX2_statusHigh.TSPO	2.98555674	3.31044173	3.64298149	0.00049809	0.00212909	-0.4749386	TSPO	assignmentsChromaffin_cells.Chrom_SOX2_statusHigh
assignmentsChromaffin_cells.Chrom_SOX2_statusHigh.NAA80	1.20109751	1.57166003	3.63117443	0.00051701	0.00220117	-0.4775714	NAA80	assignmentsChromaffin_cells.Chrom_SOX2_statusHigh
assignmentsChromaffin_cells.Chrom_SOX2_statusHigh.RAB11FIP3	-0.8770591	6.06234791	-3.7094602	0.00039913	0.00176391	-0.4784023	RAB11FIP3	assignmentsChromaffin_cells.Chrom_SOX2_statusHigh
assignmentsChromaffin_cells.Chrom_SOX2_statusHigh.FUZ	1.03356405	2.36043004	3.6610905	0.00046851	0.00202805	-0.4798555	FUZ	assignmentsChromaffin_cells.Chrom_SOX2_statusHigh
assignmentsChromaffin_cells.Chrom_SOX2_statusHigh.LRRC45	1.11412156	1.49503157	3.62877086	0.00052111	0.00221744	-0.4799368	LRRC45	assignmentsChromaffin_cells.Chrom_SOX2_statusHigh
assignmentsChromaffin_cells.Chrom_SOX2_statusHigh.CRELD1	0.90611542	4.25368249	3.69609102	0.00041726	0.00183192	-0.4806913	CRELD1	assignmentsChromaffin_cells.Chrom_SOX2_statusHigh
assignmentsChromaffin_cells.Chrom_SOX2_statusHigh.BCAS3	-0.7138536	8.39817367	-3.7350236	0.00036654	0.00163701	-0.4835247	BCAS3	assignmentsChromaffin_cells.Chrom_SOX2_statusHigh
assignmentsChromaffin_cells.Chrom_SOX2_statusHigh.PHETA2	2.63813283	0.38036909	3.55379829	0.00066557	0.00272445	-0.4847093	PHETA2	assignmentsChromaffin_cells.Chrom_SOX2_statusHigh
assignmentsChromaffin_cells.Chrom_SOX2_statusHigh.C2orf42	-0.8766591	3.87664877	-3.6634919	0.00046481	0.00201367	-0.4864937	C2orf42	assignmentsChromaffin_cells.Chrom_SOX2_statusHigh
assignmentsChromaffin_cells.Chrom_SOX2_statusHigh.MTUS2	-2.246306	3.12455594	-3.6536085	0.00048096	0.0020719	-0.4875942	MTUS2	assignmentsChromaffin_cells.Chrom_SOX2_statusHigh
assignmentsChromaffin_cells.Chrom_SOX2_statusHigh.SFTPD-AS1	-2.6201044	0.6180404	-3.5662644	0.00063944	0.00263699	-0.4942835	SFTPD-AS1	assignmentsChromaffin_cells.Chrom_SOX2_statusHigh
assignmentsChromaffin_cells.Chrom_SOX2_statusHigh.LIN28B-AS1	-2.4664277	0.98382065	-3.5895823	0.00059279	0.00247906	-0.4946228	LIN28B-AS1	assignmentsChromaffin_cells.Chrom_SOX2_statusHigh
assignmentsChromaffin_cells.Chrom_SOX2_statusHigh.SHB	2.73329592	3.10845381	3.58901207	0.00059425	0.00248451	-0.4996712	SHB	assignmentsChromaffin_cells.Chrom_SOX2_statusHigh
assignmentsChromaffin_cells.Chrom_SOX2_statusHigh.JRKL	1.01698287	3.54462254	3.66844628	0.00045726	0.00198473	-0.5059858	JRKL	assignmentsChromaffin_cells.Chrom_SOX2_statusHigh

assignmentsChromaffin_cells.Chrom_SOX2_statusHigh.MAPK3	1.10721155	3.2341587	3.65693459	0.00047498	0.0020493	-0.5060755	MAPK3	assignmentsChromaffin_cells.Chrom_SOX2_statusHigh
assignmentsChromaffin_cells.Chrom_SOX2_statusHigh.LRWD1	1.27841562	1.94517878	3.63819612	0.00050521	0.00215668	-0.5066213	LRWD1	assignmentsChromaffin_cells.Chrom_SOX2_statusHigh
assignmentsChromaffin_cells.Chrom_SOX2_statusHigh.HRAS	1.35544784	2.85014673	3.65319655	0.00048087	0.0020719	-0.5066219	HRAS	assignmentsChromaffin_cells.Chrom_SOX2_statusHigh
assignmentsChromaffin_cells.Chrom_SOX2_statusHigh.DHX58	1.90288343	2.0181337	3.60289008	0.00056723	0.00238772	-0.5093953	DHX58	assignmentsChromaffin_cells.Chrom_SOX2_statusHigh
assignmentsChromaffin_cells.Chrom_SOX2_statusHigh.WASHC4	-0.6015517	6.99620413	-3.7097273	0.00039878	0.00176283	-0.5099319	WASHC4	assignmentsChromaffin_cells.Chrom_SOX2_statusHigh
assignmentsChromaffin_cells.Chrom_SOX2_statusHigh.BEX5	2.83327069	1.46193576	3.64130354	0.00050085	0.00214031	-0.510139	BEX5	assignmentsChromaffin_cells.Chrom_SOX2_statusHigh
assignmentsChromaffin_cells.Chrom_SOX2_statusHigh.ECRG4	3.22890928	0.09183228	3.54590023	0.00068323	0.00278466	-0.5106247	ECRG4	assignmentsChromaffin_cells.Chrom_SOX2_statusHigh
assignmentsChromaffin_cells.Chrom_SOX2_statusHigh.STAP2	1.7289488	2.16122464	3.64918568	0.0004875	0.0020933	-0.5135048	STAP2	assignmentsChromaffin_cells.Chrom_SOX2_statusHigh
assignmentsChromaffin_cells.Chrom_SOX2_statusHigh.CDC42EP3	1.83669618	4.74021941	3.67615175	0.00044633	0.00194414	-0.5159149	CDC42EP3	assignmentsChromaffin_cells.Chrom_SOX2_statusHigh
assignmentsChromaffin_cells.Chrom_SOX2_statusHigh.MRPL22	0.76905443	4.44392396	3.68374101	0.0004347	0.00189862	-0.5165557	MRPL22	assignmentsChromaffin_cells.Chrom_SOX2_statusHigh
assignmentsChromaffin_cells.Chrom_SOX2_statusHigh.FRAT2	1.83790577	1.08558242	3.573461	0.00062438	0.00258621	-0.5182346	FRAT2	assignmentsChromaffin_cells.Chrom_SOX2_statusHigh
assignmentsChromaffin_cells.Chrom_SOX2_statusHigh.LRRC37B	-0.9412932	5.06304694	-3.6765599	0.00044516	0.00193954	-0.5203393	LRRC37B	assignmentsChromaffin_cells.Chrom_SOX2_statusHigh
assignmentsChromaffin_cells.Chrom_SOX2_statusHigh.ZNF385B	-2.5391536	1.71181532	-3.5900942	0.00059209	0.00247677	-0.5228915	ZNF385B	assignmentsChromaffin_cells.Chrom_SOX2_statusHigh
assignmentsChromaffin_cells.Chrom_SOX2_statusHigh.RBMS2	-1.0928676	5.83444673	-3.671871	0.00045212	0.00196718	-0.5229589	RBMS2	assignmentsChromaffin_cells.Chrom_SOX2_statusHigh
assignmentsChromaffin_cells.Chrom_SOX2_statusHigh.PUM1	-0.7427835	7.44707088	-3.7085573	0.00040033	0.00176824	-0.5230941	PUM1	assignmentsChromaffin_cells.Chrom_SOX2_statusHigh
assignmentsChromaffin_cells.Chrom_SOX2_statusHigh.SNX30	-1.0500079	4.56402119	-3.6530144	0.00048116	0.00207218	-0.5244296	SNX30	assignmentsChromaffin_cells.Chrom_SOX2_statusHigh
assignmentsChromaffin_cells.Chrom_SOX2_statusHigh.MAP3K7	-0.5984441	6.35498719	-3.6993299	0.0004128	0.0018158	-0.525736	MAP3K7	assignmentsChromaffin_cells.Chrom_SOX2_statusHigh
assignmentsChromaffin_cells.Chrom_SOX2_statusHigh.AP3S2	-0.7759387	5.88781097	-3.6883559	0.0004281	0.00187269	-0.5264146	AP3S2	assignmentsChromaffin_cells.Chrom_SOX2_statusHigh
assignmentsChromaffin_cells.Chrom_SOX2_statusHigh.TNIK	-1.4085853	6.35762563	-3.6876603	0.00042927	0.00187696	-0.5265341	TNIK	assignmentsChromaffin_cells.Chrom_SOX2_statusHigh
assignmentsChromaffin_cells.Chrom_SOX2_statusHigh.TXNIP	2.43187899	7.15493068	3.69859675	0.00041447	0.00182118	-0.5267228	TXNIP	assignmentsChromaffin_cells.Chrom_SOX2_statusHigh
assignmentsChromaffin_cells.Chrom_SOX2_statusHigh.SRP19	0.78786287	5.96594912	3.70412635	0.00040627	0.00179054	-0.5273277	SRP19	assignmentsChromaffin_cells.Chrom_SOX2_statusHigh
assignmentsChromaffin_cells.Chrom_SOX2_statusHigh.JAKMIP3	-1.8646528	3.07384921	-3.6486756	0.00048841	0.0020961	-0.5287836	JAKMIP3	assignmentsChromaffin_cells.Chrom_SOX2_statusHigh
assignmentsChromaffin_cells.Chrom_SOX2_statusHigh.CCDC90B	0.83410072	4.91084506	3.68485156	0.0004331	0.00189216	-0.5290843	CCDC90B	assignmentsChromaffin_cells.Chrom_SOX2_statusHigh
assignmentsChromaffin_cells.Chrom_SOX2_statusHigh.ENDOD1	1.24523826	4.20537319	3.66117767	0.00046837	0.00202802	-0.5291328	ENDOD1	assignmentsChromaffin_cells.Chrom_SOX2_statusHigh
assignmentsChromaffin_cells.Chrom_SOX2_statusHigh.GCC1	0.95274509	2.33496115	3.6332137	0.00051356	0.00218821	-0.5320896	GCC1	assignmentsChromaffin_cells.Chrom_SOX2_statusHigh
assignmentsChromaffin_cells.Chrom_SOX2_statusHigh.DDX54	0.80523405	3.71019143	3.65724286	0.00047449	0.00204788	-0.5331076	DDX54	assignmentsChromaffin_cells.Chrom_SOX2_statusHigh
assignmentsChromaffin_cells.Chrom_SOX2_statusHigh.CEND1	1.42461131	2.35739285	3.66063716	0.00046921	0.00203	-0.5335625	CEND1	assignmentsChromaffin_cells.Chrom_SOX2_statusHigh
assignmentsChromaffin_cells.Chrom_SOX2_statusHigh.ACD	1.33898235	2.71806975	3.64990139	0.00048612	0.00208794	-0.5364805	ACD	assignmentsChromaffin_cells.Chrom_SOX2_statusHigh
assignmentsChromaffin_cells.Chrom_SOX2_statusHigh.TMPRSS11D	-2.7983335	0.67166837	-3.5401499	0.00069594	0.00282999	-0.5365806	TMPRSS11D	assignmentsChromaffin_cells.Chrom_SOX2_statusHigh
assignmentsChromaffin_cells.Chrom_SOX2_statusHigh.LINC01431	1.67776647	0.20123462	3.57945747	0.00061231	0.0025474	-0.5373208	LINC01431	assignmentsChromaffin_cells.Chrom_SOX2_statusHigh
assignmentsChromaffin_cells.Chrom_SOX2_statusHigh.CARD8-AS1	1.67784927	0.96706458	3.55042987	0.00067288	0.00275015	-0.5383761	CARD8-AS1	assignmentsChromaffin_cells.Chrom_SOX2_statusHigh
assignmentsChromaffin_cells.Chrom_SOX2_statusHigh.NPIPB11	-1.4067429	2.28332906	-3.5944769	0.00058304	0.00244528	-0.5388554	NPIPB11	assignmentsChromaffin_cells.Chrom_SOX2_statusHigh
assignmentsChromaffin_cells.Chrom_SOX2_statusHigh.C20orf203	-1.4091322	1.29262656	-3.5753203	0.00062061	0.00257393	-0.5388805	C20orf203	assignmentsChromaffin_cells.Chrom_SOX2_statusHigh
assignmentsChromaffin_cells.Chrom_SOX2_statusHigh.GRIK4	-1.8163414	4.69490424	-3.6779596	0.00044379	0.00193411	-0.540743	GRIK4	assignmentsChromaffin_cells.Chrom_SOX2_statusHigh
assignmentsChromaffin_cells.Chrom_SOX2_statusHigh.TCTN1	0.9730445	4.33958108	3.6855574	0.00043209	0.00188825	-0.5412485	TCTN1	assignmentsChromaffin_cells.Chrom_SOX2_statusHigh
assignmentsChromaffin_cells.Chrom_SOX2_statusHigh.BMPR1A	-0.8636871	6.61782128	-3.7015564	0.00040975	0.0018044	-0.5437694	BMPR1A	assignmentsChromaffin_cells.Chrom_SOX2_statusHigh
assignmentsChromaffin_cells.Chrom_SOX2_statusHigh.LINC00937	-2.1482963	2.71541215	-3.5992248	0.00057442	0.00241293	-0.543895	LINC00937	assignmentsChromaffin_cells.Chrom_SOX2_statusHigh
assignmentsChromaffin_cells.Chrom_SOX2_statusHigh.ARAP2	-3.2392687	5.42467105	-3.6076504	0.0005593	0.00235929	-0.545407	ARAP2	assignmentsChromaffin_cells.Chrom_SOX2_statusHigh
assignmentsChromaffin_cells.Chrom_SOX2_statusHigh.TMEM176A	4.40064331	1.15791154	3.55284395	0.00066858	0.00273397	-0.5508515	TMEM176A	assignmentsChromaffin_cells.Chrom_SOX2_statusHigh
assignmentsChromaffin_cells.Chrom_SOX2_statusHigh.ORMDL1	0.75655507	6.32164936	3.69491086	0.0004189	0.0018376	-0.552576	ORMDL1	assignmentsChromaffin_cells.Chrom_SOX2_statusHigh
assignmentsChromaffin_cells.Chrom_SOX2_statusHigh.SNHG30	0.91213207	3.73901363	3.65269939	0.00048166	0.00207337	-0.5539237	SNHG30	assignmentsChromaffin_cells.Chrom_SOX2_statusHigh
assignmentsChromaffin_cells.Chrom_SOX2_statusHigh.DNER	-3.5811405	5.23224137	-3.6690097	0.00045713	0.00198471	-0.5549355	DNER	assignmentsChromaffin_cells.Chrom_SOX2_statusHigh
assignmentsChromaffin_cells.Chrom_SOX2_statusHigh.CNNM2	-1.1987122	6.48729854	-3.6899602	0.00042583	0.00186394	-0.5607474	CNNM2	assignmentsChromaffin_cells.Chrom_SOX2_statusHigh
assignmentsChromaffin_cells.Chrom_SOX2_statusHigh.IER2	2.14613041	5.78087376	3.65879597	0.00047281	0.0020428	-0.5607955	IER2	assignmentsChromaffin_cells.Chrom_SOX2_statusHigh
assignmentsChromaffin_cells.Chrom_SOX2_statusHigh.ADAM10	-0.7220963	7.44817025	-3.6914915	0.00042367	0.00185602	-0.5629562	ADAM10	assignmentsChromaffin_cells.Chrom_SOX2_statusHigh
assignmentsChromaffin_cells.Chrom_SOX2_statusHigh.ZNF133	-1.1341073	5.25572435	-3.6690228	0.00045639	0.00198203	-0.5632844	ZNF133	assignmentsChromaffin_cells.Chrom_SOX2_statusHigh
assignmentsChromaffin_cells.Chrom_SOX2_statusHigh.ZCCHC8	-0.5755001	5.82053503	-3.6775513	0.0004437	0.00193411	-0.5642536	ZCCHC8	assignmentsChromaffin_cells.Chrom_SOX2_statusHigh
assignmentsChromaffin_cells.Chrom_SOX2_statusHigh.DIP2A	-0.9696121	6.99680413	-3.6902155	0.00042547	0.00186287	-0.5646005	DIP2A	assignmentsChromaffin_cells.Chrom_SOX2_statusHigh
assignmentsChromaffin_cells.Chrom_SOX2_statusHigh.MMS19	-0.7109948	5.6077819	-3.6779398	0.00044313	0.0019328	-0.5668577	MMS19	assignmentsChromaffin_cells.Chrom_SOX2_statusHigh
assignmentsChromaffin_cells.Chrom_SOX2_statusHigh.CASC15	2.03014137	4.68705211	3.62418852	0.0005296	0.00224942	-0.5686017	CASC15	assignmentsChromaffin_cells.Chrom_SOX2_statusHigh
assignmentsChromaffin_cells.Chrom_SOX2_statusHigh.CENPL	1.14380043	2.19411734	3.6140095	0.00054696	0.00231331	-0.5739363	CENPL	assignmentsChromaffin_cells.Chrom_SOX2_statusHigh
assignmentsChromaffin_cells.Chrom_SOX2_statusHigh.PHLDA3	1.58010433	3.10282717	3.65153674	0.00048373	0.00207937	-0.5747795	PHLDA3	assignmentsChromaffin_cells.Chrom_SOX2_statusHigh
assignmentsChromaffin_cells.Chrom_SOX2_statusHigh.LINC00240	-1.7054851	2.00420794	-3.6068137	0.00056003	0.00236175	-0.575914	LINC00240	assignmentsChromaffin_cells.Chrom_SOX2_statusHigh
assignmentsChromaffin_cells.Chrom_SOX2_statusHigh.TEX10	-0.6841609	5.07396912	-3.65673	0.0004753	0.0020497	-0.5894165	TEX10	assignmentsChromaffin_cells.Chrom_SOX2_statusHigh
assignmentsChromaffin_cells.Chrom_SOX2_statusHigh.OXSM	1.04226402	2.79428054	3.62810467	0.00052225	0.00222111	-0.5895571	OXSM	assignmentsChromaffin_cells.Chrom_SOX2_statusHigh
assignmentsChromaffin_cells.Chrom_SOX2_statusHigh.VAT1L	-1.8875417	5.12241458	-3.6783387	0.00044325	0.0019328	-0.5901235	VAT1L	assignmentsChromaffin_cells.Chrom_SOX2_statusHigh
assignmentsChromaffin_cells.Chrom_SOX2_statusHigh.A2ML1-AS1	-1.6380285	2.92677682	-3.5948823	0.00058227	0.00244333	-0.5915343	A2ML1-AS1	assignmentsChromaffin_cells.Chrom_SOX2_statusHigh
assignmentsChromaffin_cells.Chrom_SOX2_statusHigh.LINC01570	-3.7902154	-0.7963041	-3.5056118	0.00077799	0.00311158	-0.5935227	LINC01570	assignmentsChromaffin_cells.Chrom_SOX2_statusHigh
assignmentsChromaffin_cells.Chrom_SOX2_statusHigh.CLUL1	2.06357449	0.35740586	3.5789686	0.00061356	0.00254995	-0.5937568	CLUL1	assignmentsChromaffin_cells.Chrom_SOX2_statusHigh
assignmentsChromaffin_cells.Chrom_SOX2_statusHigh.MUC12	2.60276617	-0.0874681	3.52531741	0.00073007	0.00294934	-0.5940011	MUC12	assignmentsChromaffin_cells.Chrom_SOX2_statusHigh
assignmentsChromaffin_cells.Chrom_SOX2_statusHigh.TEAD1	-1.1106318	7.93344332	-3.7057162	0.00040413	0.00178356	-0.5959116	TEAD1	assignmentsChromaffin_cells.Chrom_SOX2_statusHigh
assignmentsChromaffin_cells.Chrom_SOX2_statusHigh.ETNK2	1.41312066	1.18961901	3.5955466	0.00058101	0.00243867	-0.5977146	ETNK2	assignmentsChromaffin_cells.Chrom_SOX2_statusHigh
assignmentsChromaffin_cells.Chrom_SOX2_statusHigh.RET	3.72166735	1.10006854	3.57494948	0.00062226	0.00257944	-0.5988242	RET	assignmentsChromaffin_cells.Chrom_SOX2_statusHigh
assignmentsChromaffin_cells.Chrom_SOX2_statusHigh.PCDH11X	-1.8410968	0.83548265	-3.5345317	0.00070842	0.00287635	-0.6018537	PCDH11X	assignmentsChromaffin_cells.Chrom_SOX2_statusHigh

assignmentsChromaffin_cells.Chrom_SOX2_statusHigh.LRSAM1	-0.8824431	5.32054114	-3.6581794	0.00047303	0.00204322	-0.6037297	LRSAM1	assignmentsChromaffin_cells.Chrom_SOX2_statusHigh
assignmentsChromaffin_cells.Chrom_SOX2_statusHigh.EFNA1	2.00181898	1.43936804	3.52496389	0.00073065	0.00295038	-0.6056066	EFNA1	assignmentsChromaffin_cells.Chrom_SOX2_statusHigh
assignmentsChromaffin_cells.Chrom_SOX2_statusHigh.NUP160	-0.8093854	5.95865699	-3.6595395	0.00047091	0.00203626	-0.6079322	NUP160	assignmentsChromaffin_cells.Chrom_SOX2_statusHigh
assignmentsChromaffin_cells.Chrom_SOX2_statusHigh.SLC6A12	2.00678973	-0.0142961	3.52255782	0.00073635	0.00297171	-0.6165596	SLC6A12	assignmentsChromaffin_cells.Chrom_SOX2_statusHigh
assignmentsChromaffin_cells.Chrom_SOX2_statusHigh.FKBP1A	1.15930282	5.76518955	3.65847953	0.00047256	0.00204229	-0.6185606	FKBP1A	assignmentsChromaffin_cells.Chrom_SOX2_statusHigh
assignmentsChromaffin_cells.Chrom_SOX2_statusHigh.TMEM254-A	-1.2139734	2.07361663	-3.5807143	0.00060981	0.00253854	-0.6195007	TMEM254-AS	assignmentsChromaffin_cells.Chrom_SOX2_statusHigh
assignmentsChromaffin_cells.Chrom_SOX2_statusHigh.EPN2-AS1	-2.2551087	0.75387803	-3.5092243	0.00076868	0.00307664	-0.6195909	EPN2-AS1	assignmentsChromaffin_cells.Chrom_SOX2_statusHigh
assignmentsChromaffin_cells.Chrom_SOX2_statusHigh.CCN4	2.32899883	0.69120659	3.50006919	0.00079166	0.00315364	-0.6198157	CCN4	assignmentsChromaffin_cells.Chrom_SOX2_statusHigh
assignmentsChromaffin_cells.Chrom_SOX2_statusHigh.RELB	1.29048615	4.48461903	3.62542768	0.00052686	0.00223894	-0.6200113	RELB	assignmentsChromaffin_cells.Chrom_SOX2_statusHigh
assignmentsChromaffin_cells.Chrom_SOX2_statusHigh.TENT5A	2.18788753	7.21230433	3.69788076	0.00041546	0.00182501	-0.6203263	TENT5A	assignmentsChromaffin_cells.Chrom_SOX2_statusHigh
assignmentsChromaffin_cells.Chrom_SOX2_statusHigh.ASCC3	-0.7043836	8.11022886	-3.69574	0.00041774	0.00183305	-0.6205819	ASCC3	assignmentsChromaffin_cells.Chrom_SOX2_statusHigh
assignmentsChromaffin_cells.Chrom_SOX2_statusHigh.NIPBL-DT	0.70188451	3.73368229	3.63653951	0.00050797	0.0021673	-0.6206302	NIPBL-DT	assignmentsChromaffin_cells.Chrom_SOX2_statusHigh
assignmentsChromaffin_cells.Chrom_SOX2_statusHigh.TBCEL	-0.8388235	5.53246342	-3.6655699	0.00046163	0.00200098	-0.621271	TBCEL	assignmentsChromaffin_cells.Chrom_SOX2_statusHigh
assignmentsChromaffin_cells.Chrom_SOX2_statusHigh.G0S2	4.0348934	0.86713621	3.50805632	0.00077262	0.00309089	-0.6254658	G0S2	assignmentsChromaffin_cells.Chrom_SOX2_statusHigh
assignmentsChromaffin_cells.Chrom_SOX2_statusHigh.CCDC125	-0.8362714	4.89549093	-3.6446142	0.00049466	0.00211724	-0.6291056	CCDC125	assignmentsChromaffin_cells.Chrom_SOX2_statusHigh
assignmentsChromaffin_cells.Chrom_SOX2_statusHigh.MRRF	-0.8542599	5.45003102	-3.6502164	0.00048561	0.00208633	-0.6315824	MRRF	assignmentsChromaffin_cells.Chrom_SOX2_statusHigh
assignmentsChromaffin_cells.Chrom_SOX2_statusHigh.C11orf98	2.69169834	0.89540079	3.54506965	0.00068553	0.00279262	-0.6343749	C11orf98	assignmentsChromaffin_cells.Chrom_SOX2_statusHigh
assignmentsChromaffin_cells.Chrom_SOX2_statusHigh.CCDC86	1.32244945	1.80780339	3.58620826	0.00059898	0.00249974	-0.6408257	CCDC86	assignmentsChromaffin_cells.Chrom_SOX2_statusHigh
assignmentsChromaffin_cells.Chrom_SOX2_statusHigh.WDR46	0.97521582	3.74784708	3.62501765	0.00052757	0.00224136	-0.6432011	WDR46	assignmentsChromaffin_cells.Chrom_SOX2_statusHigh
assignmentsChromaffin_cells.Chrom_SOX2_statusHigh.NEK9	-0.7682135	5.88391998	-3.6472077	0.00049045	0.00210148	-0.6481029	NEK9	assignmentsChromaffin_cells.Chrom_SOX2_statusHigh
assignmentsChromaffin_cells.Chrom_SOX2_statusHigh.MYO5B	-2.1603874	3.19869739	-3.5923398	0.00058795	0.00246265	-0.6497025	MYO5B	assignmentsChromaffin_cells.Chrom_SOX2_statusHigh
assignmentsChromaffin_cells.Chrom_SOX2_statusHigh.DDX56	0.74521205	4.30760236	3.63462044	0.00051119	0.00217986	-0.6503882	DDX56	assignmentsChromaffin_cells.Chrom_SOX2_statusHigh
assignmentsChromaffin_cells.Chrom_SOX2_statusHigh.ELP1	-0.9094425	6.26983916	-3.6649761	0.00046254	0.00200436	-0.6526749	ELP1	assignmentsChromaffin_cells.Chrom_SOX2_statusHigh
assignmentsChromaffin_cells.Chrom_SOX2_statusHigh.PPID	0.74560251	4.99294097	3.64645495	0.00049167	0.00210523	-0.6527988	PPID	assignmentsChromaffin_cells.Chrom_SOX2_statusHigh
assignmentsChromaffin_cells.Chrom_SOX2_statusHigh.BCLAF3	-0.7366189	5.27870429	-3.638898	0.00050405	0.00215342	-0.6535494	BCLAF3	assignmentsChromaffin_cells.Chrom_SOX2_statusHigh
assignmentsChromaffin_cells.Chrom_SOX2_statusHigh.INSRR	2.82886494	0.83922903	3.57116012	0.00062998	0.00260671	-0.6582719	INSRR	assignmentsChromaffin_cells.Chrom_SOX2_statusHigh
assignmentsChromaffin_cells.Chrom_SOX2_statusHigh.CAMKV	3.07700256	-0.3084405	3.53206405	0.00071508	0.00289827	-0.6585755	CAMKV	assignmentsChromaffin_cells.Chrom_SOX2_statusHigh
assignmentsChromaffin_cells.Chrom_SOX2_statusHigh.CBX8	1.42340812	1.86617536	3.58523029	0.0006009	0.00250707	-0.6633809	CBX8	assignmentsChromaffin_cells.Chrom_SOX2_statusHigh
assignmentsChromaffin_cells.Chrom_SOX2_statusHigh.SCFD2	-0.8054973	7.41342106	-3.6674345	0.00045879	0.00198976	-0.663746	SCFD2	assignmentsChromaffin_cells.Chrom_SOX2_statusHigh
assignmentsChromaffin_cells.Chrom_SOX2_statusHigh.FLCN	-0.8780929	4.90804273	-3.6331046	0.00051374	0.00218842	-0.6645216	FLCN	assignmentsChromaffin_cells.Chrom_SOX2_statusHigh
assignmentsChromaffin_cells.Chrom_SOX2_statusHigh.CDC73	-0.5168296	7.26012194	-3.6677558	0.00045831	0.00198819	-0.6681154	CDC73	assignmentsChromaffin_cells.Chrom_SOX2_statusHigh
assignmentsChromaffin_cells.Chrom_SOX2_statusHigh.RAB30-DT	-0.9429935	5.81006055	-3.6467798	0.00049114	0.00210389	-0.6683855	RAB30-DT	assignmentsChromaffin_cells.Chrom_SOX2_statusHigh
assignmentsChromaffin_cells.Chrom_SOX2_statusHigh.MEPCE	1.11167735	2.72276739	3.58621228	0.00059898	0.00249974	-0.6692269	MEPCE	assignmentsChromaffin_cells.Chrom_SOX2_statusHigh
assignmentsChromaffin_cells.Chrom_SOX2_statusHigh.HBEGF	1.86279897	4.16140406	3.58719324	0.00059743	0.00249585	-0.6717531	HBEGF	assignmentsChromaffin_cells.Chrom_SOX2_statusHigh
assignmentsChromaffin_cells.Chrom_SOX2_statusHigh.TRAM1L1	1.44407203	1.07857624	3.58068587	0.00060986	0.00253854	-0.6719884	TRAM1L1	assignmentsChromaffin_cells.Chrom_SOX2_statusHigh
assignmentsChromaffin_cells.Chrom_SOX2_statusHigh.SLX4	-0.8994473	3.03593261	-3.5862095	0.00059898	0.00249974	-0.6719985	SLX4	assignmentsChromaffin_cells.Chrom_SOX2_statusHigh
assignmentsChromaffin_cells.Chrom_SOX2_statusHigh.ZCCHC4	-0.7191478	3.95521496	-3.6103978	0.00055346	0.00233837	-0.6724374	ZCCHC4	assignmentsChromaffin_cells.Chrom_SOX2_statusHigh
assignmentsChromaffin_cells.Chrom_SOX2_statusHigh.NAA35	-0.7739263	6.70131155	-3.6575671	0.00047399	0.00204625	-0.6732872	NAA35	assignmentsChromaffin_cells.Chrom_SOX2_statusHigh
assignmentsChromaffin_cells.Chrom_SOX2_statusHigh.HEATR3	-0.930307	4.88746701	-3.6088283	0.00055632	0.00234918	-0.676804	HEATR3	assignmentsChromaffin_cells.Chrom_SOX2_statusHigh
assignmentsChromaffin_cells.Chrom_SOX2_statusHigh.NR2C1	-0.8082347	6.09334942	-3.6476132	0.0004898	0.0020998	-0.6794672	NR2C1	assignmentsChromaffin_cells.Chrom_SOX2_statusHigh
assignmentsChromaffin_cells.Chrom_SOX2_statusHigh.RPTOR	-0.8856965	6.89428736	-3.6561952	0.00047614	0.00205276	-0.6894553	RPTOR	assignmentsChromaffin_cells.Chrom_SOX2_statusHigh
assignmentsChromaffin_cells.Chrom_SOX2_statusHigh.DDX19B	-0.6640653	4.65303266	-3.6151585	0.0005449	0.00230645	-0.6898441	DDX19B	assignmentsChromaffin_cells.Chrom_SOX2_statusHigh
assignmentsChromaffin_cells.Chrom_SOX2_statusHigh.PRKCD	1.79445242	2.64941939	3.53175952	0.00071479	0.00289783	-0.6913493	PRKCD	assignmentsChromaffin_cells.Chrom_SOX2_statusHigh
assignmentsChromaffin_cells.Chrom_SOX2_statusHigh.TPRA1	0.92624296	3.65593289	3.60182202	0.00056921	0.00239481	-0.6919733	TPRA1	assignmentsChromaffin_cells.Chrom_SOX2_statusHigh
assignmentsChromaffin_cells.Chrom_SOX2_statusHigh.PKHD1	-2.1581831	1.07239755	-3.5287956	0.00072196	0.00292155	-0.6937119	PKHD1	assignmentsChromaffin_cells.Chrom_SOX2_statusHigh
assignmentsChromaffin_cells.Chrom_SOX2_statusHigh.RNF135	1.89556822	3.31370511	3.56610868	0.00063982	0.00263723	-0.6950842	RNF135	assignmentsChromaffin_cells.Chrom_SOX2_statusHigh
assignmentsChromaffin_cells.Chrom_SOX2_statusHigh.FAM72A	1.90390212	0.48258163	3.48623546	0.00082761	0.00326925	-0.6964487	FAM72A	assignmentsChromaffin_cells.Chrom_SOX2_statusHigh
assignmentsChromaffin_cells.Chrom_SOX2_statusHigh.USP28	-0.8853073	4.76294612	-3.6102915	0.00055366	0.00233857	-0.69891	USP28	assignmentsChromaffin_cells.Chrom_SOX2_statusHigh
assignmentsChromaffin_cells.Chrom_SOX2_statusHigh.SOCS5	0.91565539	3.79062409	3.60489173	0.00056353	0.00237338	-0.7009859	SOCS5	assignmentsChromaffin_cells.Chrom_SOX2_statusHigh
assignmentsChromaffin_cells.Chrom_SOX2_statusHigh.KCTD1	-0.9672058	4.99310827	-3.6222749	0.00053234	0.00225922	-0.7012184	KCTD1	assignmentsChromaffin_cells.Chrom_SOX2_statusHigh
assignmentsChromaffin_cells.Chrom_SOX2_statusHigh.AQR	-0.511124	6.34021377	-3.6433666	0.00049669	0.00212369	-0.701907	AQR	assignmentsChromaffin_cells.Chrom_SOX2_statusHigh
assignmentsChromaffin_cells.Chrom_SOX2_statusHigh.RPS20	2.6188782	6.1338949	3.65159994	0.00048416	0.00208063	-0.7025324	RPS20	assignmentsChromaffin_cells.Chrom_SOX2_statusHigh
assignmentsChromaffin_cells.Chrom_SOX2_statusHigh.ADIRF	3.25761453	2.43041647	3.51708212	0.00075049	0.00301741	-0.7036637	ADIRF	assignmentsChromaffin_cells.Chrom_SOX2_statusHigh
assignmentsChromaffin_cells.Chrom_SOX2_statusHigh.SHMT2	1.19242556	3.8118852	3.62241871	0.00053209	0.00225876	-0.7043151	SHMT2	assignmentsChromaffin_cells.Chrom_SOX2_statusHigh
assignmentsChromaffin_cells.Chrom_SOX2_statusHigh.MYO16	1.6507171	0.9584385	3.52877907	0.00072171	0.00292155	-0.7043972	MYO16	assignmentsChromaffin_cells.Chrom_SOX2_statusHigh
assignmentsChromaffin_cells.Chrom_SOX2_statusHigh.TMEM106B	0.69540943	5.53255193	3.64343922	0.00049657	0.00212369	-0.7078702	TMEM106B	assignmentsChromaffin_cells.Chrom_SOX2_statusHigh
assignmentsChromaffin_cells.Chrom_SOX2_statusHigh.RGS22	2.84607599	0.40303032	3.504383	0.00078161	0.00311904	-0.7089597	RGS22	assignmentsChromaffin_cells.Chrom_SOX2_statusHigh
assignmentsChromaffin_cells.Chrom_SOX2_statusHigh.GAB2	-0.937736	8.40794078	-3.6709356	0.00045352	0.00197167	-0.709081	GAB2	assignmentsChromaffin_cells.Chrom_SOX2_statusHigh
assignmentsChromaffin_cells.Chrom_SOX2_statusHigh.LIN28B	-3.2456782	3.03623139	-3.5602683	0.00065267	0.00268054	-0.7112897	LIN28B	assignmentsChromaffin_cells.Chrom_SOX2_statusHigh
assignmentsChromaffin_cells.Chrom_SOX2_statusHigh.LAT	-1.3552232	2.62819806	-3.5284124	0.00072256	0.00292195	-0.7117512	LAT	assignmentsChromaffin_cells.Chrom_SOX2_statusHigh
assignmentsChromaffin_cells.Chrom_SOX2_statusHigh.SLC26A2	1.14744804	4.63541882	3.62121372	0.00053419	0.0022659	-0.7128624	SLC26A2	assignmentsChromaffin_cells.Chrom_SOX2_statusHigh
assignmentsChromaffin_cells.Chrom_SOX2_statusHigh.SOS1	-0.7751911	7.73226625	-3.6524569	0.00048204	0.00207432	-0.7167094	SOS1	assignmentsChromaffin_cells.Chrom_SOX2_statusHigh
assignmentsChromaffin_cells.Chrom_SOX2_statusHigh.UBE2C	2.28996978	0.84130969	3.5278081	0.00072439	0.00292862	-0.7168562	UBE2C	assignmentsChromaffin_cells.Chrom_SOX2_statusHigh
assignmentsChromaffin_cells.Chrom_SOX2_statusHigh.LINC01004	-1.1548122	6.23583338	-3.6234946	0.00053021	0.00225139	-0.7169394	LINC01004	assignmentsChromaffin_cells.Chrom_SOX2_statusHigh

assignmentsChromaffin_cells.Chrom_SOX2_statusHigh.GABPA	-0.6390776	4.89988541	-3.6104361	0.00055339	0.00233837	-0.7180789	GABPA	assignmentsChromaffin_cells.Chrom_SOX2_statusHigh
assignmentsChromaffin_cells.Chrom_SOX2_statusHigh.C5	-1.2302051	3.8082127	-3.5755784	0.00062009	0.0025731	-0.7204562	C5	assignmentsChromaffin_cells.Chrom_SOX2_statusHigh
assignmentsChromaffin_cells.Chrom_SOX2_statusHigh.SYN3	-3.6501719	5.3065084	-3.5831314	0.0006059	0.00252665	-0.7240251	SYN3	assignmentsChromaffin_cells.Chrom_SOX2_statusHigh
assignmentsChromaffin_cells.Chrom_SOX2_statusHigh.STARD3	0.97226557	4.79489827	3.61587854	0.00054362	0.00230162	-0.7257306	STARD3	assignmentsChromaffin_cells.Chrom_SOX2_statusHigh
assignmentsChromaffin_cells.Chrom_SOX2_statusHigh.PORCN	0.82853336	2.16981957	3.5619479	0.00064819	0.00266694	-0.7269866	PORCN	assignmentsChromaffin_cells.Chrom_SOX2_statusHigh
assignmentsChromaffin_cells.Chrom_SOX2_statusHigh.PHRF1	-0.8967856	4.67775071	-3.5922466	0.0005873	0.00246122	-0.727761	PHRF1	assignmentsChromaffin_cells.Chrom_SOX2_statusHigh
assignmentsChromaffin_cells.Chrom_SOX2_statusHigh.HENMT1	2.46954365	0.70806213	3.45734113	0.00090774	0.00354046	-0.7301206	HENMT1	assignmentsChromaffin_cells.Chrom_SOX2_statusHigh
assignmentsChromaffin_cells.Chrom_SOX2_statusHigh.LINC00920	2.25765673	-0.1000491	3.46180847	0.00089489	0.00349799	-0.7301394	LINC00920	assignmentsChromaffin_cells.Chrom_SOX2_statusHigh
assignmentsChromaffin_cells.Chrom_SOX2_statusHigh.L3MBTL4-A	-2.8524385	-0.1554347	-3.4556678	0.0009126	0.00355768	-0.7303863	L3MBTL4-AS	assignmentsChromaffin_cells.Chrom_SOX2_statusHigh
assignmentsChromaffin_cells.Chrom_SOX2_statusHigh.KIF20A	1.92650874	-0.0643141	3.47209737	0.00086594	0.00339809	-0.7362135	KIF20A	assignmentsChromaffin_cells.Chrom_SOX2_statusHigh
assignmentsChromaffin_cells.Chrom_SOX2_statusHigh.STARD10	1.3378564	3.17529674	3.60251718	0.00056792	0.00239001	-0.7403903	STARD10	assignmentsChromaffin_cells.Chrom_SOX2_statusHigh
assignmentsChromaffin_cells.Chrom_SOX2_statusHigh.RBM26	-0.8650038	7.56785764	-3.6479267	0.00048929	0.00209876	-0.7427377	RBM26	assignmentsChromaffin_cells.Chrom_SOX2_statusHigh
assignmentsChromaffin_cells.Chrom_SOX2_statusHigh.DNAJC12	1.41644224	3.73628874	3.61708443	0.00054171	0.00229475	-0.7428057	DNAJC12	assignmentsChromaffin_cells.Chrom_SOX2_statusHigh
assignmentsChromaffin_cells.Chrom_SOX2_statusHigh.CYSLTR2	3.12485107	3.72042566	3.59982751	0.00057378	0.00241089	-0.7473365	CYSLTR2	assignmentsChromaffin_cells.Chrom_SOX2_statusHigh
assignmentsChromaffin_cells.Chrom_SOX2_statusHigh.ZNF436	1.07312889	1.8305877	3.53226827	0.00071362	0.0028938	-0.7485977	ZNF436	assignmentsChromaffin_cells.Chrom_SOX2_statusHigh
assignmentsChromaffin_cells.Chrom_SOX2_statusHigh.ARL8A	1.00230678	4.34834594	3.60116845	0.00057043	0.00239931	-0.7494339	ARL8A	assignmentsChromaffin_cells.Chrom_SOX2_statusHigh
assignmentsChromaffin_cells.Chrom_SOX2_statusHigh.GPR171	-2.8594496	0.60298972	-3.4468051	0.00093875	0.00364015	-0.7527589	GPR171	assignmentsChromaffin_cells.Chrom_SOX2_statusHigh
assignmentsChromaffin_cells.Chrom_SOX2_statusHigh.VEZT	-0.7848545	7.40539361	-3.6472436	0.00049039	0.00210148	-0.7534086	VEZT	assignmentsChromaffin_cells.Chrom_SOX2_statusHigh
assignmentsChromaffin_cells.Chrom_SOX2_statusHigh.COMMD1	-0.7049606	7.29446534	-3.6385498	0.00050463	0.00215474	-0.7542507	COMMD1	assignmentsChromaffin_cells.Chrom_SOX2_statusHigh
assignmentsChromaffin_cells.Chrom_SOX2_statusHigh.CWC27	-0.64002	6.73730127	-3.6326775	0.00051446	0.00219091	-0.7546359	CWC27	assignmentsChromaffin_cells.Chrom_SOX2_statusHigh
assignmentsChromaffin_cells.Chrom_SOX2_statusHigh.ZNF672	0.82698921	3.11001003	3.57404471	0.0006232	0.00258264	-0.7548737	ZNF672	assignmentsChromaffin_cells.Chrom_SOX2_statusHigh
assignmentsChromaffin_cells.Chrom_SOX2_statusHigh.IFRD2	1.03660332	3.41440056	3.58046467	0.0006103	0.00253971	-0.7564422	IFRD2	assignmentsChromaffin_cells.Chrom_SOX2_statusHigh
assignmentsChromaffin_cells.Chrom_SOX2_statusHigh.LINC01515	-2.6003467	5.55482638	-3.6152916	0.00054548	0.0023083	-0.759691	LINC01515	assignmentsChromaffin_cells.Chrom_SOX2_statusHigh
assignmentsChromaffin_cells.Chrom_SOX2_statusHigh.RAPGEF2	-0.8897093	8.23786225	-3.6385675	0.0005046	0.00215474	-0.7597509	RAPGEF2	assignmentsChromaffin_cells.Chrom_SOX2_statusHigh
assignmentsChromaffin_cells.Chrom_SOX2_statusHigh.NMT2	-0.8365655	4.9959582	-3.5958059	0.00058051	0.00243724	-0.7601422	NMT2	assignmentsChromaffin_cells.Chrom_SOX2_statusHigh
assignmentsChromaffin_cells.Chrom_SOX2_statusHigh.FAM135A	-0.8765218	6.68088752	-3.6281129	0.00052223	0.00222111	-0.7609763	FAM135A	assignmentsChromaffin_cells.Chrom_SOX2_statusHigh
assignmentsChromaffin_cells.Chrom_SOX2_statusHigh.SCG5	2.48764954	5.43773959	3.64812325	0.00048973	0.0020998	-0.7616128	SCG5	assignmentsChromaffin_cells.Chrom_SOX2_statusHigh
assignmentsChromaffin_cells.Chrom_SOX2_statusHigh.AAMP	1.0610779	4.31236368	3.5998269	0.00057294	0.00240859	-0.7634317	AAMP	assignmentsChromaffin_cells.Chrom_SOX2_statusHigh
assignmentsChromaffin_cells.Chrom_SOX2_statusHigh.LGALSL	1.16513101	2.76297599	3.56425679	0.00064335	0.00264905	-0.7638116	LGALSL	assignmentsChromaffin_cells.Chrom_SOX2_statusHigh
assignmentsChromaffin_cells.Chrom_SOX2_statusHigh.PDK4	3.18021755	5.46915077	3.57288236	0.00062646	0.00259416	-0.764195	PDK4	assignmentsChromaffin_cells.Chrom_SOX2_statusHigh
assignmentsChromaffin_cells.Chrom_SOX2_statusHigh.TMEM30A	1.0180834	5.97547288	3.62783395	0.00052271	0.0022225	-0.7646649	TMEM30A	assignmentsChromaffin_cells.Chrom_SOX2_statusHigh
assignmentsChromaffin_cells.Chrom_SOX2_statusHigh.PET117	1.69319515	1.89698622	3.55531602	0.00066257	0.00271287	-0.7688847	PET117	assignmentsChromaffin_cells.Chrom_SOX2_statusHigh
assignmentsChromaffin_cells.Chrom_SOX2_statusHigh.G2E3-AS1	-2.0601087	1.5908425	-3.4879515	0.00082307	0.00325704	-0.7689538	G2E3-AS1	assignmentsChromaffin_cells.Chrom_SOX2_statusHigh
assignmentsChromaffin_cells.Chrom_SOX2_statusHigh.GTPBP10	-0.7278244	5.67667944	-3.614107	0.00054678	0.00231319	-0.7692723	GTPBP10	assignmentsChromaffin_cells.Chrom_SOX2_statusHigh
assignmentsChromaffin_cells.Chrom_SOX2_statusHigh.GATB	-0.8518305	3.62861266	-3.5759052	0.00061943	0.0025717	-0.7700966	GATB	assignmentsChromaffin_cells.Chrom_SOX2_statusHigh
assignmentsChromaffin_cells.Chrom_SOX2_statusHigh.DOT1L	-0.9126436	4.97951703	-3.5916346	0.00058847	0.00246357	-0.7725247	DOT1L	assignmentsChromaffin_cells.Chrom_SOX2_statusHigh
assignmentsChromaffin_cells.Chrom_SOX2_statusHigh.DHX32	-0.6452373	5.44701867	-3.6030011	0.00056702	0.00238748	-0.7726524	DHX32	assignmentsChromaffin_cells.Chrom_SOX2_statusHigh
assignmentsChromaffin_cells.Chrom_SOX2_statusHigh.CCDC71	1.26342982	2.11522602	3.54966214	0.00067455	0.0027542	-0.7734596	CCDC71	assignmentsChromaffin_cells.Chrom_SOX2_statusHigh
assignmentsChromaffin_cells.Chrom_SOX2_statusHigh.HOXA6	2.69661322	1.27173087	3.55037829	0.00067394	0.0027531	-0.7752004	HOXA6	assignmentsChromaffin_cells.Chrom_SOX2_statusHigh
assignmentsChromaffin_cells.Chrom_SOX2_statusHigh.SERPING1	2.41713866	2.73915542	3.49854209	0.00079601	0.00316942	-0.7759494	SERPING1	assignmentsChromaffin_cells.Chrom_SOX2_statusHigh
assignmentsChromaffin_cells.Chrom_SOX2_statusHigh.GLS	-1.0302029	7.95882718	-3.6261354	0.00052564	0.00223433	-0.7766101	GLS	assignmentsChromaffin_cells.Chrom_SOX2_statusHigh
assignmentsChromaffin_cells.Chrom_SOX2_statusHigh.THSD8	1.76339388	0.39634977	3.4954814	0.00080341	0.00319175	-0.7766188	THSD8	assignmentsChromaffin_cells.Chrom_SOX2_statusHigh
assignmentsChromaffin_cells.Chrom_SOX2_statusHigh.DDX39A	0.97443268	4.69997066	3.58990058	0.00059181	0.00247626	-0.7882321	DDX39A	assignmentsChromaffin_cells.Chrom_SOX2_statusHigh
assignmentsChromaffin_cells.Chrom_SOX2_statusHigh.SOBP	-1.3925361	7.58521712	-3.6466832	0.00049172	0.00210523	-0.7884175	SOBP	assignmentsChromaffin_cells.Chrom_SOX2_statusHigh
assignmentsChromaffin_cells.Chrom_SOX2_statusHigh.METTL2B	-0.5177401	5.09686895	-3.5995542	0.00057345	0.0024101	-0.7918947	METTL2B	assignmentsChromaffin_cells.Chrom_SOX2_statusHigh
assignmentsChromaffin_cells.Chrom_SOX2_statusHigh.FOXN2	-1.0613515	6.19240229	-3.5963377	0.00057951	0.00243365	-0.7921625	FOXN2	assignmentsChromaffin_cells.Chrom_SOX2_statusHigh
assignmentsChromaffin_cells.Chrom_SOX2_statusHigh.TEP1	-0.9606012	4.32823807	-3.5500766	0.00067365	0.0027526	-0.7937482	TEP1	assignmentsChromaffin_cells.Chrom_SOX2_statusHigh
assignmentsChromaffin_cells.Chrom_SOX2_statusHigh.NSG1	2.15672832	2.62754772	3.58135171	0.00060943	0.00253804	-0.7945062	NSG1	assignmentsChromaffin_cells.Chrom_SOX2_statusHigh
assignmentsChromaffin_cells.Chrom_SOX2_statusHigh.RNF169	-0.8941409	6.19207744	-3.6058361	0.00056179	0.00236756	-0.7945076	RNF169	assignmentsChromaffin_cells.Chrom_SOX2_statusHigh
assignmentsChromaffin_cells.Chrom_SOX2_statusHigh.PMAIP1	2.60227829	2.14235058	3.52904041	0.0007221	0.00292155	-0.7957355	PMAIP1	assignmentsChromaffin_cells.Chrom_SOX2_statusHigh
assignmentsChromaffin_cells.Chrom_SOX2_statusHigh.RAB37	2.21434926	1.11434256	3.48452139	0.00083249	0.0032861	-0.7982779	RAB37	assignmentsChromaffin_cells.Chrom_SOX2_statusHigh
assignmentsChromaffin_cells.Chrom_SOX2_statusHigh.PSME4	-0.7863712	7.54122677	-3.6218872	0.00053301	0.0022615	-0.7997788	PSME4	assignmentsChromaffin_cells.Chrom_SOX2_statusHigh
assignmentsChromaffin_cells.Chrom_SOX2_statusHigh.C3orf20	1.78387988	1.29668083	3.50890042	0.00076948	0.00307909	-0.8015413	C3orf20	assignmentsChromaffin_cells.Chrom_SOX2_statusHigh
assignmentsChromaffin_cells.Chrom_SOX2_statusHigh.USP24	-0.7811801	6.83723295	-3.6072492	0.0005592	0.00235929	-0.8022783	USP24	assignmentsChromaffin_cells.Chrom_SOX2_statusHigh
assignmentsChromaffin_cells.Chrom_SOX2_statusHigh.CCNY	-0.896858	8.11086423	-3.6299081	0.00051916	0.00220976	-0.8025891	CCNY	assignmentsChromaffin_cells.Chrom_SOX2_statusHigh
assignmentsChromaffin_cells.Chrom_SOX2_statusHigh.CDKN1C	3.45374931	1.78578227	3.47358138	0.00086297	0.0033881	-0.8026528	CDKN1C	assignmentsChromaffin_cells.Chrom_SOX2_statusHigh
assignmentsChromaffin_cells.Chrom_SOX2_statusHigh.OAS2	2.25627986	2.17052828	3.44185502	0.00095366	0.0036926	-0.8029171	OAS2	assignmentsChromaffin_cells.Chrom_SOX2_statusHigh
assignmentsChromaffin_cells.Chrom_SOX2_statusHigh.FAM13B	-0.6366254	7.40903877	-3.6206428	0.00053519	0.00226895	-0.8075016	FAM13B	assignmentsChromaffin_cells.Chrom_SOX2_statusHigh
assignmentsChromaffin_cells.Chrom_SOX2_statusHigh.IFI27L1	0.85873703	3.59286418	3.56690593	0.00063783	0.00263174	-0.8079234	IFI27L1	assignmentsChromaffin_cells.Chrom_SOX2_statusHigh
assignmentsChromaffin_cells.Chrom_SOX2_statusHigh.TSPAN6	2.03046155	1.26858673	3.49139262	0.0008143	0.00322939	-0.808841	TSPAN6	assignmentsChromaffin_cells.Chrom_SOX2_statusHigh
assignmentsChromaffin_cells.Chrom_SOX2_statusHigh.RAB27B	-3.264043	1.7290002	-3.4535673	0.00091992	0.00357925	-0.8098789	RAB27B	assignmentsChromaffin_cells.Chrom_SOX2_statusHigh
assignmentsChromaffin_cells.Chrom_SOX2_statusHigh.TMEM42	1.49110082	2.2518914	3.54320818	0.00068881	0.00280525	-0.8099018	TMEM42	assignmentsChromaffin_cells.Chrom_SOX2_statusHigh
assignmentsChromaffin_cells.Chrom_SOX2_statusHigh.ZNF473	0.82636184	2.95650166	3.55916593	0.00065408	0.00268357	-0.8103675	ZNF473	assignmentsChromaffin_cells.Chrom_SOX2_statusHigh
assignmentsChromaffin_cells.Chrom_SOX2_statusHigh.IL17RE	1.30767805	1.48403178	3.51234401	0.000761	0.00305198	-0.811525	IL17RE	assignmentsChromaffin_cells.Chrom_SOX2_statusHigh

assignmentsChromaffin_cells.Chrom_SOX2_statusHigh.TMEM218	1.04088096	3.43911251	3.55961419	0.00065312	0.00268172	-0.8164131	TMEM218	assignmentsChromaffin_cells.Chrom_SOX2_statusHigh
assignmentsChromaffin_cells.Chrom_SOX2_statusHigh.SF3B2	1.16827655	6.12093115	3.61249985	0.00054967	0.00232417	-0.8177458	SF3B2	assignmentsChromaffin_cells.Chrom_SOX2_statusHigh
assignmentsChromaffin_cells.Chrom_SOX2_statusHigh.ABHD17A	1.19272716	4.64891929	3.58243666	0.00060639	0.00252803	-0.8206261	ABHD17A	assignmentsChromaffin_cells.Chrom_SOX2_statusHigh
assignmentsChromaffin_cells.Chrom_SOX2_statusHigh.MED13	-0.6969152	7.62182019	-3.6204924	0.00053546	0.00226947	-0.8207467	MED13	assignmentsChromaffin_cells.Chrom_SOX2_statusHigh
assignmentsChromaffin_cells.Chrom_SOX2_statusHigh.CTSF	2.45315033	3.24037081	3.56029431	0.00065261	0.00268054	-0.821927	CTSF	assignmentsChromaffin_cells.Chrom_SOX2_statusHigh
assignmentsChromaffin_cells.Chrom_SOX2_statusHigh.SLC25A10	0.98927028	1.08062947	3.51970392	0.00074316	0.00299016	-0.8222662	SLC25A10	assignmentsChromaffin_cells.Chrom_SOX2_statusHigh
assignmentsChromaffin_cells.Chrom_SOX2_statusHigh.ZNF551	-1.040981	4.07902093	-3.5590485	0.00065432	0.0026839	-0.8227668	ZNF551	assignmentsChromaffin_cells.Chrom_SOX2_statusHigh
assignmentsChromaffin_cells.Chrom_SOX2_statusHigh.MEI1	-1.6985101	1.61902698	-3.4649802	0.00088587	0.00346696	-0.8230149	MEI1	assignmentsChromaffin_cells.Chrom_SOX2_statusHigh
assignmentsChromaffin_cells.Chrom_SOX2_statusHigh.LINC02714	4.13137535	-0.4103265	3.42490323	0.00100769	0.0038729	-0.823018	LINC02714	assignmentsChromaffin_cells.Chrom_SOX2_statusHigh
assignmentsChromaffin_cells.Chrom_SOX2_statusHigh.CLCN6	-0.7901204	4.92735295	-3.5704685	0.00063049	0.00260747	-0.8256784	CLCN6	assignmentsChromaffin_cells.Chrom_SOX2_statusHigh
assignmentsChromaffin_cells.Chrom_SOX2_statusHigh.STN1	1.12204669	4.13030472	3.56020234	0.00065188	0.00267935	-0.8325148	STN1	assignmentsChromaffin_cells.Chrom_SOX2_statusHigh
assignmentsChromaffin_cells.Chrom_SOX2_statusHigh.ANKRD60	2.14228544	0.03121772	3.49089339	0.00081574	0.00323432	-0.8328311	ANKRD60	assignmentsChromaffin_cells.Chrom_SOX2_statusHigh
assignmentsChromaffin_cells.Chrom_SOX2_statusHigh.EIF4ENIF1	-0.5306724	4.88046276	-3.5738112	0.00062367	0.00258393	-0.837926	EIF4ENIF1	assignmentsChromaffin_cells.Chrom_SOX2_statusHigh
assignmentsChromaffin_cells.Chrom_SOX2_statusHigh.APBA1	-1.3757707	7.03876467	-3.6164975	0.00054286	0.00229901	-0.8384769	APBA1	assignmentsChromaffin_cells.Chrom_SOX2_statusHigh
assignmentsChromaffin_cells.Chrom_SOX2_statusHigh.ANKRD35	2.13663324	0.74886517	3.45993452	0.00090026	0.00351471	-0.8387755	ANKRD35	assignmentsChromaffin_cells.Chrom_SOX2_statusHigh
assignmentsChromaffin_cells.Chrom_SOX2_statusHigh.SPARC	1.77239678	5.71530903	3.55951611	0.00065389	0.00268349	-0.8418943	SPARC	assignmentsChromaffin_cells.Chrom_SOX2_statusHigh
assignmentsChromaffin_cells.Chrom_SOX2_statusHigh.NEDD4L	-1.8862034	5.97288152	-3.5758595	0.00062042	0.00257381	-0.8422894	NEDD4L	assignmentsChromaffin_cells.Chrom_SOX2_statusHigh
assignmentsChromaffin_cells.Chrom_SOX2_statusHigh.RNF115	-0.7364703	7.25827206	-3.6079637	0.00055789	0.0023546	-0.8434774	RNF115	assignmentsChromaffin_cells.Chrom_SOX2_statusHigh
assignmentsChromaffin_cells.Chrom_SOX2_statusHigh.CNKSR3	-3.8573743	4.5252422	-3.520578	0.00074209	0.00298734	-0.8435655	CNKSR3	assignmentsChromaffin_cells.Chrom_SOX2_statusHigh
assignmentsChromaffin_cells.Chrom_SOX2_statusHigh.TSPAN5	-1.1755017	6.38215624	-3.574995	0.00062127	0.00257599	-0.8466912	TSPAN5	assignmentsChromaffin_cells.Chrom_SOX2_statusHigh
assignmentsChromaffin_cells.Chrom_SOX2_statusHigh.ANKRD26	-0.7108455	6.89887938	-3.6063693	0.00056081	0.00236442	-0.8494108	ANKRD26	assignmentsChromaffin_cells.Chrom_SOX2_statusHigh
assignmentsChromaffin_cells.Chrom_SOX2_statusHigh.WBP1L	-0.844977	5.88822115	-3.5705116	0.0006304	0.00260747	-0.851874	WBP1L	assignmentsChromaffin_cells.Chrom_SOX2_statusHigh
assignmentsChromaffin_cells.Chrom_SOX2_statusHigh.DNAJC4	1.23877888	3.21741417	3.54685606	0.00068072	0.00277794	-0.8523533	DNAJC4	assignmentsChromaffin_cells.Chrom_SOX2_statusHigh
assignmentsChromaffin_cells.Chrom_SOX2_statusHigh.PIGH	0.71722364	3.95447429	3.56440123	0.00064305	0.00264849	-0.8538025	PIGH	assignmentsChromaffin_cells.Chrom_SOX2_statusHigh
assignmentsChromaffin_cells.Chrom_SOX2_statusHigh.PRMT7	-0.8740348	4.80052881	-3.562952	0.00064608	0.00265894	-0.8550631	PRMT7	assignmentsChromaffin_cells.Chrom_SOX2_statusHigh
assignmentsChromaffin_cells.Chrom_SOX2_statusHigh.ABHD10	0.8330686	3.77487197	3.56050042	0.00065125	0.00267783	-0.8555011	ABHD10	assignmentsChromaffin_cells.Chrom_SOX2_statusHigh
assignmentsChromaffin_cells.Chrom_SOX2_statusHigh.GOLIM4	1.00582227	6.82863446	3.6058035	0.00056185	0.00236756	-0.8567749	GOLIM4	assignmentsChromaffin_cells.Chrom_SOX2_statusHigh
assignmentsChromaffin_cells.Chrom_SOX2_statusHigh.TMEM98	2.5036547	0.73337523	3.42494305	0.00100631	0.00386937	-0.8572718	TMEM98	assignmentsChromaffin_cells.Chrom_SOX2_statusHigh
assignmentsChromaffin_cells.Chrom_SOX2_statusHigh.RFWD3	-0.7129429	4.67759117	-3.5556699	0.00066154	0.00270934	-0.8580444	RFWD3	assignmentsChromaffin_cells.Chrom_SOX2_statusHigh
assignmentsChromaffin_cells.Chrom_SOX2_statusHigh.MAP3K3	-0.8184043	6.25647552	-3.5816868	0.00060788	0.0025329	-0.8580947	MAP3K3	assignmentsChromaffin_cells.Chrom_SOX2_statusHigh
assignmentsChromaffin_cells.Chrom_SOX2_statusHigh.ZDHHC7	-0.6145917	4.99427645	-3.5563887	0.00066	0.0027051	-0.8621602	ZDHHC7	assignmentsChromaffin_cells.Chrom_SOX2_statusHigh
assignmentsChromaffin_cells.Chrom_SOX2_statusHigh.CBX4	1.41963552	3.71381373	3.54754619	0.0006792	0.00277245	-0.8678957	CBX4	assignmentsChromaffin_cells.Chrom_SOX2_statusHigh
assignmentsChromaffin_cells.Chrom_SOX2_statusHigh.FAM98C	1.02059655	2.75344358	3.53330198	0.00071124	0.00288634	-0.8691018	FAM98C	assignmentsChromaffin_cells.Chrom_SOX2_statusHigh
assignmentsChromaffin_cells.Chrom_SOX2_statusHigh.COQ3	0.9016925	2.20777602	3.529158	0.00072083	0.00291934	-0.8698126	COQ3	assignmentsChromaffin_cells.Chrom_SOX2_statusHigh
assignmentsChromaffin_cells.Chrom_SOX2_statusHigh.HOXC9	2.93780261	2.34058304	3.54661274	0.00068221	0.00278334	-0.870042	HOXC9	assignmentsChromaffin_cells.Chrom_SOX2_statusHigh
assignmentsChromaffin_cells.Chrom_SOX2_statusHigh.MET	2.52171158	1.06078628	3.41882411	0.00102602	0.00393386	-0.8724366	MET	assignmentsChromaffin_cells.Chrom_SOX2_statusHigh
assignmentsChromaffin_cells.Chrom_SOX2_statusHigh.MAU2	-0.6067449	5.7083739	-3.5791859	0.00061285	0.00254833	-0.8745449	MAU2	assignmentsChromaffin_cells.Chrom_SOX2_statusHigh
assignmentsChromaffin_cells.Chrom_SOX2_statusHigh.DTNB	-0.812161	6.60417578	-3.5919638	0.00058784	0.00246265	-0.8774028	DTNB	assignmentsChromaffin_cells.Chrom_SOX2_statusHigh
assignmentsChromaffin_cells.Chrom_SOX2_statusHigh.MIDN	1.37464456	5.69120913	3.58143631	0.00060863	0.0025354	-0.8792721	MIDN	assignmentsChromaffin_cells.Chrom_SOX2_statusHigh
assignmentsChromaffin_cells.Chrom_SOX2_statusHigh.EDC3	-0.6094537	4.30002213	-3.551056	0.00067151	0.00274528	-0.8797049	EDC3	assignmentsChromaffin_cells.Chrom_SOX2_statusHigh
assignmentsChromaffin_cells.Chrom_SOX2_statusHigh.SETX	-0.7856521	7.67943516	-3.5947961	0.00058243	0.00244338	-0.8841893	SETX	assignmentsChromaffin_cells.Chrom_SOX2_statusHigh
assignmentsChromaffin_cells.Chrom_SOX2_statusHigh.RTN4IP1	-0.8153074	3.88964131	-3.5418079	0.00069193	0.00281728	-0.8866384	RTN4IP1	assignmentsChromaffin_cells.Chrom_SOX2_statusHigh
assignmentsChromaffin_cells.Chrom_SOX2_statusHigh.ENAH	-1.4358457	6.13441726	-3.5758437	0.0006199	0.00257299	-0.8912727	ENAH	assignmentsChromaffin_cells.Chrom_SOX2_statusHigh
assignmentsChromaffin_cells.Chrom_SOX2_statusHigh.SLC5A9	-2.6906915	0.3216296	-3.39485	0.00110683	0.00418962	-0.8929671	SLC5A9	assignmentsChromaffin_cells.Chrom_SOX2_statusHigh
assignmentsChromaffin_cells.Chrom_SOX2_statusHigh.MTCP1	-1.6701904	2.30506952	-3.4842866	0.00083279	0.0032865	-0.8930387	MTCP1	assignmentsChromaffin_cells.Chrom_SOX2_statusHigh
assignmentsChromaffin_cells.Chrom_SOX2_statusHigh.CCT4	0.97871272	6.36334611	3.59172398	0.0005883	0.00246349	-0.8948244	CCT4	assignmentsChromaffin_cells.Chrom_SOX2_statusHigh
assignmentsChromaffin_cells.Chrom_SOX2_statusHigh.MMP21	-2.3358516	0.86478729	-3.4108034	0.00105242	0.00402222	-0.8971101	MMP21	assignmentsChromaffin_cells.Chrom_SOX2_statusHigh
assignmentsChromaffin_cells.Chrom_SOX2_statusHigh.ANXA5	1.34954519	6.81063154	3.58843381	0.00059498	0.0024869	-0.898569	ANXA5	assignmentsChromaffin_cells.Chrom_SOX2_statusHigh
assignmentsChromaffin_cells.Chrom_SOX2_statusHigh.POLR2F	1.1507556	4.89870772	3.56594843	0.00063982	0.00263723	-0.8992249	POLR2F	assignmentsChromaffin_cells.Chrom_SOX2_statusHigh
assignmentsChromaffin_cells.Chrom_SOX2_statusHigh.TIMM10B	0.91976053	3.81200003	3.5384062	0.00069959	0.00284413	-0.8995258	TIMM10B	assignmentsChromaffin_cells.Chrom_SOX2_statusHigh
assignmentsChromaffin_cells.Chrom_SOX2_statusHigh.SAP30L	0.70835787	3.80665904	3.54021053	0.00069552	0.00282972	-0.9013656	SAP30L	assignmentsChromaffin_cells.Chrom_SOX2_statusHigh
assignmentsChromaffin_cells.Chrom_SOX2_statusHigh.HOXD-AS2	2.20749449	2.10094588	3.51532812	0.00075475	0.00302995	-0.9016144	HOXD-AS2	assignmentsChromaffin_cells.Chrom_SOX2_statusHigh
assignmentsChromaffin_cells.Chrom_SOX2_statusHigh.PHYH	0.97149206	4.16923473	3.55344268	0.00066634	0.00272689	-0.902578	PHYH	assignmentsChromaffin_cells.Chrom_SOX2_statusHigh
assignmentsChromaffin_cells.Chrom_SOX2_statusHigh.ZNF451	-0.6031161	7.05300747	-3.5871662	0.00059712	0.00249519	-0.9084597	ZNF451	assignmentsChromaffin_cells.Chrom_SOX2_statusHigh
assignmentsChromaffin_cells.Chrom_SOX2_statusHigh.KLHL7	-0.6553079	5.79133809	-3.569018	0.00063347	0.00261539	-0.9091806	KLHL7	assignmentsChromaffin_cells.Chrom_SOX2_statusHigh
assignmentsChromaffin_cells.Chrom_SOX2_statusHigh.FAM171A1	-1.1995668	4.75074117	-3.541053	0.00069363	0.00282345	-0.9095009	FAM171A1	assignmentsChromaffin_cells.Chrom_SOX2_statusHigh
assignmentsChromaffin_cells.Chrom_SOX2_statusHigh.PARP6	-0.6650642	5.61113625	-3.5663489	0.00063899	0.00263584	-0.9126466	PARP6	assignmentsChromaffin_cells.Chrom_SOX2_statusHigh
assignmentsChromaffin_cells.Chrom_SOX2_statusHigh.FAM50A	0.98763647	5.2741714	3.56956319	0.00063235	0.00261314	-0.9133237	FAM50A	assignmentsChromaffin_cells.Chrom_SOX2_statusHigh
assignmentsChromaffin_cells.Chrom_SOX2_statusHigh.DENND5A	-1.1560035	6.6973752	-3.5600406	0.00065222	0.00268007	-0.9167539	DENND5A	assignmentsChromaffin_cells.Chrom_SOX2_statusHigh
assignmentsChromaffin_cells.Chrom_SOX2_statusHigh.PTTG1IP	1.01251638	5.9249876	3.56898165	0.00063354	0.00261539	-0.9186666	PTTG1IP	assignmentsChromaffin_cells.Chrom_SOX2_statusHigh
assignmentsChromaffin_cells.Chrom_SOX2_statusHigh.NSMCE2	-0.7747549	8.16026059	-3.5942398	0.00058349	0.00244654	-0.9233047	NSMCE2	assignmentsChromaffin_cells.Chrom_SOX2_statusHigh
assignmentsChromaffin_cells.Chrom_SOX2_statusHigh.EIF2AK1	0.60655048	5.59800222	3.572083	0.00062719	0.00259649	-0.9253706	EIF2AK1	assignmentsChromaffin_cells.Chrom_SOX2_statusHigh
assignmentsChromaffin_cells.Chrom_SOX2_statusHigh.DHCR7	1.28114474	2.0673331	3.5043596	0.00078081	0.0031174	-0.9258874	DHCR7	assignmentsChromaffin_cells.Chrom_SOX2_statusHigh
assignmentsChromaffin_cells.Chrom_SOX2_statusHigh.PIRT	2.71133941	-0.262317	3.41197629	0.00104906	0.00401344	-0.9276292	PIRT	assignmentsChromaffin_cells.Chrom_SOX2_statusHigh

assignmentsChromaffin_cells.Chrom_SOX2_statusHigh.EIF4G1	0.98825099	5.69065279	3.56531952	0.00064113	0.00264195	-0.9280514	EIF4G1	assignmentsChromaffin_cells.Chrom_SOX2_statusHigh
assignmentsChromaffin_cells.Chrom_SOX2_statusHigh.ZBTB22	1.00049537	1.7094364	3.49258533	0.00081092	0.00321918	-0.928273	ZBTB22	assignmentsChromaffin_cells.Chrom_SOX2_statusHigh
assignmentsChromaffin_cells.Chrom_SOX2_statusHigh.CNOT2	-0.6350432	7.5135923	-3.5819791	0.0006073	0.00253115	-0.9295382	CNOT2	assignmentsChromaffin_cells.Chrom_SOX2_statusHigh
assignmentsChromaffin_cells.Chrom_SOX2_statusHigh.COL9A2	2.12644021	0.54847745	3.39656496	0.00110086	0.00416995	-0.9324387	COL9A2	assignmentsChromaffin_cells.Chrom_SOX2_statusHigh
assignmentsChromaffin_cells.Chrom_SOX2_statusHigh.HSH2D	1.80705805	1.19829586	3.41215221	0.00104793	0.00401115	-0.9330488	HSH2D	assignmentsChromaffin_cells.Chrom_SOX2_statusHigh
assignmentsChromaffin_cells.Chrom_SOX2_statusHigh.AP5S1	1.03425041	2.13008774	3.48664128	0.00082653	0.0032658	-0.9342771	AP5S1	assignmentsChromaffin_cells.Chrom_SOX2_statusHigh
assignmentsChromaffin_cells.Chrom_SOX2_statusHigh.RAD9A	-0.9201614	5.37751987	-3.5457791	0.00068309	0.00278466	-0.9353401	RAD9A	assignmentsChromaffin_cells.Chrom_SOX2_statusHigh
assignmentsChromaffin_cells.Chrom_SOX2_statusHigh.PODNL1	1.55498467	-4.97E-05	3.40583169	0.0010691	0.00407466	-0.9377945	PODNL1	assignmentsChromaffin_cells.Chrom_SOX2_statusHigh
assignmentsChromaffin_cells.Chrom_SOX2_statusHigh.VWA8	-0.9946359	7.56735623	-3.5850738	0.0006012	0.0025077	-0.9379214	VWA8	assignmentsChromaffin_cells.Chrom_SOX2_statusHigh
assignmentsChromaffin_cells.Chrom_SOX2_statusHigh.USP47	-0.6791812	7.70575948	-3.577909	0.0006154	0.00255696	-0.9396185	USP47	assignmentsChromaffin_cells.Chrom_SOX2_statusHigh
assignmentsChromaffin_cells.Chrom_SOX2_statusHigh.EIF4E1B	-2.4421997	0.10535186	-3.3962765	0.00110186	0.00417277	-0.9406614	EIF4E1B	assignmentsChromaffin_cells.Chrom_SOX2_statusHigh
assignmentsChromaffin_cells.Chrom_SOX2_statusHigh.BTLA	1.71316758	0.08331646	3.38980452	0.00112459	0.00424622	-0.9408612	BTLA	assignmentsChromaffin_cells.Chrom_SOX2_statusHigh
assignmentsChromaffin_cells.Chrom_SOX2_statusHigh.PIP4K2C	0.73086492	3.30788127	3.5215869	0.00073866	0.00297914	-0.9416821	PIP4K2C	assignmentsChromaffin_cells.Chrom_SOX2_statusHigh
assignmentsChromaffin_cells.Chrom_SOX2_statusHigh.GINM1	0.75494889	5.4983408	3.55743097	0.00065777	0.00269665	-0.9492692	GINM1	assignmentsChromaffin_cells.Chrom_SOX2_statusHigh
assignmentsChromaffin_cells.Chrom_SOX2_statusHigh.TMEM134	1.26403353	3.36595466	3.51970398	0.00074316	0.00299016	-0.9499665	TMEM134	assignmentsChromaffin_cells.Chrom_SOX2_statusHigh
assignmentsChromaffin_cells.Chrom_SOX2_statusHigh.SLC5A4-AS1	-5.6497469	2.11979851	-3.3811074	0.00115722	0.00434647	-0.9500087	SLC5A4-AS1	assignmentsChromaffin_cells.Chrom_SOX2_statusHigh
assignmentsChromaffin_cells.Chrom_SOX2_statusHigh.ARHGAP36	5.24538353	-0.4422157	3.39015709	0.0011247	0.00424622	-0.9505206	ARHGAP36	assignmentsChromaffin_cells.Chrom_SOX2_statusHigh
assignmentsChromaffin_cells.Chrom_SOX2_statusHigh.TMEM266	-1.987151	1.64471322	-3.4302814	0.0009894	0.00381167	-0.95162	TMEM266	assignmentsChromaffin_cells.Chrom_SOX2_statusHigh
assignmentsChromaffin_cells.Chrom_SOX2_statusHigh.ATP8A2	-2.9573904	3.76842063	-3.5161881	0.00075266	0.00302384	-0.9537098	ATP8A2	assignmentsChromaffin_cells.Chrom_SOX2_statusHigh
assignmentsChromaffin_cells.Chrom_SOX2_statusHigh.MRTFA	-0.9877125	7.90417501	-3.5694744	0.00063253	0.00261314	-0.9541366	MRTFA	assignmentsChromaffin_cells.Chrom_SOX2_statusHigh
assignmentsChromaffin_cells.Chrom_SOX2_statusHigh.DDX31	-0.9581164	4.96652951	-3.532617	0.00071281	0.0028916	-0.9542894	DDX31	assignmentsChromaffin_cells.Chrom_SOX2_statusHigh
assignmentsChromaffin_cells.Chrom_SOX2_statusHigh.PLCXD2	1.52791047	1.30355611	3.45865933	0.00090393	0.00352817	-0.9591225	PLCXD2	assignmentsChromaffin_cells.Chrom_SOX2_statusHigh
assignmentsChromaffin_cells.Chrom_SOX2_statusHigh.LINC01374	-2.6859523	2.81410661	-3.4465515	0.00094071	0.00364687	-0.9595418	LINC01374	assignmentsChromaffin_cells.Chrom_SOX2_statusHigh
assignmentsChromaffin_cells.Chrom_SOX2_statusHigh.ZNF671	-1.4160963	2.25636046	-3.458302	0.00090496	0.00353134	-0.9595722	ZNF671	assignmentsChromaffin_cells.Chrom_SOX2_statusHigh
assignmentsChromaffin_cells.Chrom_SOX2_statusHigh.MTMR12	-0.6296337	5.41313383	-3.5378485	0.00070086	0.00284782	-0.9597518	MTMR12	assignmentsChromaffin_cells.Chrom_SOX2_statusHigh
assignmentsChromaffin_cells.Chrom_SOX2_statusHigh.SREBF2	-1.1645596	6.91308253	-3.5616586	0.0006488	0.00266877	-0.9638162	SREBF2	assignmentsChromaffin_cells.Chrom_SOX2_statusHigh
assignmentsChromaffin_cells.Chrom_SOX2_statusHigh.PTMS	1.59055451	5.99996286	3.56984731	0.00063267	0.00261314	-0.9642669	PTMS	assignmentsChromaffin_cells.Chrom_SOX2_statusHigh
assignmentsChromaffin_cells.Chrom_SOX2_statusHigh.MBD4	0.98846831	5.12021641	3.54027747	0.00069537	0.00282972	-0.9647533	MBD4	assignmentsChromaffin_cells.Chrom_SOX2_statusHigh
assignmentsChromaffin_cells.Chrom_SOX2_statusHigh.U2AF1L4	1.08958055	3.37578019	3.52154968	0.00073875	0.00297914	-0.9652357	U2AF1L4	assignmentsChromaffin_cells.Chrom_SOX2_statusHigh
assignmentsChromaffin_cells.Chrom_SOX2_statusHigh.CPLANE1	-0.9398107	7.00998439	-3.5703697	0.00063069	0.00260764	-0.9654558	CPLANE1	assignmentsChromaffin_cells.Chrom_SOX2_statusHigh
assignmentsChromaffin_cells.Chrom_SOX2_statusHigh.CERS6	-1.0131996	7.21164858	-3.5669948	0.00063765	0.00263166	-0.9666587	CERS6	assignmentsChromaffin_cells.Chrom_SOX2_statusHigh
assignmentsChromaffin_cells.Chrom_SOX2_statusHigh.IGSF6	-2.0651763	2.53594955	-3.4391135	0.00096237	0.00372274	-0.9699042	IGSF6	assignmentsChromaffin_cells.Chrom_SOX2_statusHigh
assignmentsChromaffin_cells.Chrom_SOX2_statusHigh.ASTN2-AS1	-1.6748659	1.16739228	-3.4480584	0.00093501	0.0036274	-0.9699087	ASTN2-AS1	assignmentsChromaffin_cells.Chrom_SOX2_statusHigh
assignmentsChromaffin_cells.Chrom_SOX2_statusHigh.USP15	-0.6064402	8.17332838	-3.5765775	0.00061808	0.00256674	-0.9702267	USP15	assignmentsChromaffin_cells.Chrom_SOX2_statusHigh
assignmentsChromaffin_cells.Chrom_SOX2_statusHigh.FBXW5	1.02584465	4.04588187	3.52055322	0.00074113	0.00298648	-0.9705237	FBXW5	assignmentsChromaffin_cells.Chrom_SOX2_statusHigh
assignmentsChromaffin_cells.Chrom_SOX2_statusHigh.BLOC1S6	0.78731249	5.73746902	3.55581747	0.00066122	0.00270873	-0.9716303	BLOC1S6	assignmentsChromaffin_cells.Chrom_SOX2_statusHigh
assignmentsChromaffin_cells.Chrom_SOX2_statusHigh.MON1B	0.57384239	3.92954505	3.51555604	0.00075316	0.00302458	-0.974237	MON1B	assignmentsChromaffin_cells.Chrom_SOX2_statusHigh
assignmentsChromaffin_cells.Chrom_SOX2_statusHigh.ETNK1	-0.6564584	7.26367481	-3.5648015	0.00064221	0.00264572	-0.9757758	ETNK1	assignmentsChromaffin_cells.Chrom_SOX2_statusHigh
assignmentsChromaffin_cells.Chrom_SOX2_statusHigh.TRIM34	2.22172606	2.29037756	3.42997708	0.00099082	0.00381623	-0.975915	TRIM34	assignmentsChromaffin_cells.Chrom_SOX2_statusHigh
assignmentsChromaffin_cells.Chrom_SOX2_statusHigh.IL6R	2.11949722	4.1503956	3.47476783	0.00085949	0.00337624	-0.9774858	IL6R	assignmentsChromaffin_cells.Chrom_SOX2_statusHigh
assignmentsChromaffin_cells.Chrom_SOX2_statusHigh.TMEM129	1.07939208	3.01450961	3.51069864	0.00076504	0.00306437	-0.9781311	TMEM129	assignmentsChromaffin_cells.Chrom_SOX2_statusHigh
assignmentsChromaffin_cells.Chrom_SOX2_statusHigh.PRRT2	-1.173958	4.79059318	-3.5370083	0.00070276	0.00285485	-0.9792723	PRRT2	assignmentsChromaffin_cells.Chrom_SOX2_statusHigh
assignmentsChromaffin_cells.Chrom_SOX2_statusHigh.ZNF34	0.87367036	3.12345473	3.4996959	0.00079261	0.00315665	-0.9805615	ZNF34	assignmentsChromaffin_cells.Chrom_SOX2_statusHigh
assignmentsChromaffin_cells.Chrom_SOX2_statusHigh.EPS8L2	1.7377437	0.99639102	3.44956507	0.00093053	0.00361528	-0.9812442	EPS8L2	assignmentsChromaffin_cells.Chrom_SOX2_statusHigh
assignmentsChromaffin_cells.Chrom_SOX2_statusHigh.MDN1	-0.7409007	6.4283987	-3.5496692	0.00067454	0.0027542	-0.9817382	MDN1	assignmentsChromaffin_cells.Chrom_SOX2_statusHigh
assignmentsChromaffin_cells.Chrom_SOX2_statusHigh.MICA	2.106845	2.60452492	3.40812386	0.00106138	0.00405102	-0.9860125	MICA	assignmentsChromaffin_cells.Chrom_SOX2_statusHigh
assignmentsChromaffin_cells.Chrom_SOX2_statusHigh.KIAA2013	1.09952648	2.95462052	3.4880676	0.00082276	0.00325704	-0.9878344	KIAA2013	assignmentsChromaffin_cells.Chrom_SOX2_statusHigh
assignmentsChromaffin_cells.Chrom_SOX2_statusHigh.MCOLN1	1.05456719	4.56322195	3.52662568	0.00072674	0.00293738	-0.9880068	MCOLN1	assignmentsChromaffin_cells.Chrom_SOX2_statusHigh
assignmentsChromaffin_cells.Chrom_SOX2_statusHigh.MYL3	2.08311994	-0.273852	3.3620394	0.00122721	0.00456551	-0.9880815	MYL3	assignmentsChromaffin_cells.Chrom_SOX2_statusHigh
assignmentsChromaffin_cells.Chrom_SOX2_statusHigh.NGFR	2.41541001	0.72962845	3.37126644	0.00119216	0.00445582	-0.9896468	NGFR	assignmentsChromaffin_cells.Chrom_SOX2_statusHigh
assignmentsChromaffin_cells.Chrom_SOX2_statusHigh.AKIP1	0.85320937	3.65341408	3.50464958	0.00078008	0.00311683	-0.9904669	AKIP1	assignmentsChromaffin_cells.Chrom_SOX2_statusHigh
assignmentsChromaffin_cells.Chrom_SOX2_statusHigh.RIOX2	0.71048356	3.59112753	3.51114003	0.00076395	0.00306154	-0.9915025	RIOX2	assignmentsChromaffin_cells.Chrom_SOX2_statusHigh
assignmentsChromaffin_cells.Chrom_SOX2_statusHigh.POLRMT	0.80851662	3.12074498	3.49804726	0.00079682	0.00317075	-0.991799	POLRMT	assignmentsChromaffin_cells.Chrom_SOX2_statusHigh
assignmentsChromaffin_cells.Chrom_SOX2_statusHigh.HMG20A	-0.6205392	5.87488142	-3.5400927	0.00069579	0.00282999	-0.9957704	HMG20A	assignmentsChromaffin_cells.Chrom_SOX2_statusHigh
assignmentsChromaffin_cells.Chrom_SOX2_statusHigh.ABHD15-AS	-1.5337656	3.73361298	-3.4743434	0.00085974	0.00337624	-0.9971426	ABHD15-AS1	assignmentsChromaffin_cells.Chrom_SOX2_statusHigh
assignmentsChromaffin_cells.Chrom_SOX2_statusHigh.ZNF717	-0.8606153	4.9043292	-3.5235952	0.00073389	0.00296252	-0.9989355	ZNF717	assignmentsChromaffin_cells.Chrom_SOX2_statusHigh
assignmentsChromaffin_cells.Chrom_SOX2_statusHigh.CHRM2	-4.4195353	0.4497292	-3.3582457	0.00124335	0.00461701	-0.9991542	CHRM2	assignmentsChromaffin_cells.Chrom_SOX2_statusHigh
assignmentsChromaffin_cells.Chrom_SOX2_statusHigh.RECQL	0.88629181	4.99388344	3.52037365	0.00074156	0.00298665	-0.9992815	RECQL	assignmentsChromaffin_cells.Chrom_SOX2_statusHigh
assignmentsChromaffin_cells.Chrom_SOX2_statusHigh.BTAF1	-0.7310167	7.41671034	-3.5604559	0.00065134	0.00267783	-0.9998564	BTAF1	assignmentsChromaffin_cells.Chrom_SOX2_statusHigh
assignmentsChromaffin_cells.Chrom_SOX2_statusHigh.SDCBP2-AS:	-1.1209381	4.60814296	-3.5130479	0.00075927	0.0030466	-1.0005516	SDCBP2-AS1	assignmentsChromaffin_cells.Chrom_SOX2_statusHigh
assignmentsChromaffin_cells.Chrom_SOX2_statusHigh.GGNBP2	0.47287953	6.70435928	3.55833392	0.00065584	0.00268945	-1.0007216	GGNBP2	assignmentsChromaffin_cells.Chrom_SOX2_statusHigh
assignmentsChromaffin_cells.Chrom_SOX2_statusHigh.SYNGR4	2.33381417	-0.7689393	3.36533863	0.00121457	0.00452856	-1.0008698	SYNGR4	assignmentsChromaffin_cells.Chrom_SOX2_statusHigh
assignmentsChromaffin_cells.Chrom_SOX2_statusHigh.GLG1	-0.5406821	7.61572119	-3.5635668	0.00064479	0.00265431	-1.0028868	GLG1	assignmentsChromaffin_cells.Chrom_SOX2_statusHigh
assignmentsChromaffin_cells.Chrom_SOX2_statusHigh.COL28A1	-2.0178991	2.54623759	-3.4367797	0.0009696	0.00374438	-1.0051713	COL28A1	assignmentsChromaffin_cells.Chrom_SOX2_statusHigh

assignmentsChromaffin_cells.Chrom_SOX2_statusHigh.LAMP1	1.02383183	5.92114308	3.54574344	0.00068317	0.00278466	-1.0071211	LAMP1	assignmentsChromaffin_cells.Chrom_SOX2_statusHigh
assignmentsChromaffin_cells.Chrom_SOX2_statusHigh.C15orf39	1.1746423	2.70952826	3.45519424	0.00091398	0.00356046	-1.0077805	C15orf39	assignmentsChromaffin_cells.Chrom_SOX2_statusHigh
assignmentsChromaffin_cells.Chrom_SOX2_statusHigh.GCNT1	-1.4474598	4.4414199	-3.4887865	0.00082086	0.00325143	-1.0084806	GCNT1	assignmentsChromaffin_cells.Chrom_SOX2_statusHigh
assignmentsChromaffin_cells.Chrom_SOX2_statusHigh.GALNT13	-1.9051732	6.03158294	-3.5598127	0.00065363	0.00268313	-1.009477	GALNT13	assignmentsChromaffin_cells.Chrom_SOX2_statusHigh
assignmentsChromaffin_cells.Chrom_SOX2_statusHigh.PI4K2B	-0.7438208	4.87628094	-3.5037907	0.00078224	0.00312033	-1.0099862	PI4K2B	assignmentsChromaffin_cells.Chrom_SOX2_statusHigh
assignmentsChromaffin_cells.Chrom_SOX2_statusHigh.WDR13	0.88558264	4.20306064	3.52105488	0.00073993	0.00298315	-1.013388	WDR13	assignmentsChromaffin_cells.Chrom_SOX2_statusHigh
assignmentsChromaffin_cells.Chrom_SOX2_statusHigh.ZNF732	-2.1963257	0.35672934	-3.3634168	0.00122191	0.00455005	-1.0134035	ZNF732	assignmentsChromaffin_cells.Chrom_SOX2_statusHigh
assignmentsChromaffin_cells.Chrom_SOX2_statusHigh.CYP51A1-AS	1.17753129	1.06371108	3.45155688	0.00092464	0.00359501	-1.015361	CYP51A1-AS:	assignmentsChromaffin_cells.Chrom_SOX2_statusHigh
assignmentsChromaffin_cells.Chrom_SOX2_statusHigh.SLC41A3	0.93706834	3.37108238	3.49248535	0.00081118	0.00321942	-1.0172213	SLC41A3	assignmentsChromaffin_cells.Chrom_SOX2_statusHigh
assignmentsChromaffin_cells.Chrom_SOX2_statusHigh.NUDCD3	-0.5799153	6.44009088	-3.5448147	0.00068523	0.00279211	-1.0172597	NUDCD3	assignmentsChromaffin_cells.Chrom_SOX2_statusHigh
assignmentsChromaffin_cells.Chrom_SOX2_statusHigh.MLF2	1.32549818	5.47505214	3.545946	0.00068302	0.00278466	-1.0188175	MLF2	assignmentsChromaffin_cells.Chrom_SOX2_statusHigh
assignmentsChromaffin_cells.Chrom_SOX2_statusHigh.MIGA2	-0.8511071	3.9638758	-3.4878207	0.00082341	0.00325704	-1.0190276	MIGA2	assignmentsChromaffin_cells.Chrom_SOX2_statusHigh
assignmentsChromaffin_cells.Chrom_SOX2_statusHigh.ADGRF3	1.11718856	2.30059685	3.46916257	0.00087411	0.00342594	-1.0249368	ADGRF3	assignmentsChromaffin_cells.Chrom_SOX2_statusHigh
assignmentsChromaffin_cells.Chrom_SOX2_statusHigh.STARD7-AS	-0.9214268	3.02371202	-3.4609347	0.00089739	0.00350605	-1.0254917	STARD7-AS1	assignmentsChromaffin_cells.Chrom_SOX2_statusHigh
assignmentsChromaffin_cells.Chrom_SOX2_statusHigh.GSAP	-1.2282222	5.97078429	-3.5249463	0.0007307	0.00295038	-1.0265142	GSAP	assignmentsChromaffin_cells.Chrom_SOX2_statusHigh
assignmentsChromaffin_cells.Chrom_SOX2_statusHigh.COG7	-0.6502734	5.48737814	-3.5284786	0.00072241	0.00292195	-1.0277023	COG7	assignmentsChromaffin_cells.Chrom_SOX2_statusHigh
assignmentsChromaffin_cells.Chrom_SOX2_statusHigh.BEND5	1.2902932	1.61178173	3.46373304	0.00088941	0.00347995	-1.0284574	BEND5	assignmentsChromaffin_cells.Chrom_SOX2_statusHigh
assignmentsChromaffin_cells.Chrom_SOX2_statusHigh.ADAM33	2.83353758	0.00289806	3.34956363	0.00127613	0.00472234	-1.0307408	ADAM33	assignmentsChromaffin_cells.Chrom_SOX2_statusHigh
assignmentsChromaffin_cells.Chrom_SOX2_statusHigh.GID8	0.86459387	4.34493306	3.51280592	0.00075987	0.00304821	-1.0309337	GID8	assignmentsChromaffin_cells.Chrom_SOX2_statusHigh
assignmentsChromaffin_cells.Chrom_SOX2_statusHigh.RCOR1	-1.0201554	6.85053702	-3.5255696	0.00072923	0.00294667	-1.0331949	RCOR1	assignmentsChromaffin_cells.Chrom_SOX2_statusHigh
assignmentsChromaffin_cells.Chrom_SOX2_statusHigh.SH3RF1	-1.0034245	6.8006858	-3.5351817	0.00070693	0.00287103	-1.0352151	SH3RF1	assignmentsChromaffin_cells.Chrom_SOX2_statusHigh
assignmentsChromaffin_cells.Chrom_SOX2_statusHigh.GSTP1	2.82248531	4.93973252	3.51823419	0.00074771	0.00300773	-1.035414	GSTP1	assignmentsChromaffin_cells.Chrom_SOX2_statusHigh
assignmentsChromaffin_cells.Chrom_SOX2_statusHigh.SS18L2	1.11977184	4.008871	3.51222663	0.00076129	0.00305238	-1.0385454	SS18L2	assignmentsChromaffin_cells.Chrom_SOX2_statusHigh
assignmentsChromaffin_cells.Chrom_SOX2_statusHigh.JARID2	-1.1465522	8.0081884	-3.5340489	0.00070952	0.00288011	-1.0393581	JARID2	assignmentsChromaffin_cells.Chrom_SOX2_statusHigh
assignmentsChromaffin_cells.Chrom_SOX2_statusHigh.SDHAF4	0.89164056	3.18254297	3.4921137	0.00081215	0.00322246	-1.0397095	SDHAF4	assignmentsChromaffin_cells.Chrom_SOX2_statusHigh
assignmentsChromaffin_cells.Chrom_SOX2_statusHigh.STMN4	2.26581503	1.80733967	3.4832806	0.00083659	0.00329825	-1.0399998	STMN4	assignmentsChromaffin_cells.Chrom_SOX2_statusHigh
assignmentsChromaffin_cells.Chrom_SOX2_statusHigh.HIBADH	-0.7331772	6.51409916	-3.5378715	0.00070081	0.00284782	-1.0401197	HIBADH	assignmentsChromaffin_cells.Chrom_SOX2_statusHigh
assignmentsChromaffin_cells.Chrom_SOX2_statusHigh.YWHAQ	0.87627513	7.20683556	3.55288473	0.00066754	0.00273044	-1.0408131	YWHAQ	assignmentsChromaffin_cells.Chrom_SOX2_statusHigh
assignmentsChromaffin_cells.Chrom_SOX2_statusHigh.CPA4	2.1231789	0.15311467	3.41760801	0.00103052	0.00395016	-1.0423669	CPA4	assignmentsChromaffin_cells.Chrom_SOX2_statusHigh
assignmentsChromaffin_cells.Chrom_SOX2_statusHigh.CBX1	0.67568226	4.91596734	3.52202919	0.00073761	0.00297603	-1.0450996	CBX1	assignmentsChromaffin_cells.Chrom_SOX2_statusHigh
assignmentsChromaffin_cells.Chrom_SOX2_statusHigh.MIEF1	0.69481808	3.35455315	3.48451597	0.00083218	0.00328571	-1.0470601	MIEF1	assignmentsChromaffin_cells.Chrom_SOX2_statusHigh
assignmentsChromaffin_cells.Chrom_SOX2_statusHigh.LINC00682	1.73927504	3.88133523	3.53301302	0.00071289	0.0028916	-1.0500278	LINC00682	assignmentsChromaffin_cells.Chrom_SOX2_statusHigh
assignmentsChromaffin_cells.Chrom_SOX2_statusHigh.ZNF764	0.78704712	2.5874612	3.46163077	0.0008954	0.00349912	-1.05071	ZNF764	assignmentsChromaffin_cells.Chrom_SOX2_statusHigh
assignmentsChromaffin_cells.Chrom_SOX2_statusHigh.EEF2KMT	0.96578942	1.87752727	3.44871016	0.00093307	0.0036225	-1.052218	EEF2KMT	assignmentsChromaffin_cells.Chrom_SOX2_statusHigh
assignmentsChromaffin_cells.Chrom_SOX2_statusHigh.MFAP2	2.99726271	1.07555736	3.41110375	0.00105272	0.00402222	-1.0547277	MFAP2	assignmentsChromaffin_cells.Chrom_SOX2_statusHigh
assignmentsChromaffin_cells.Chrom_SOX2_statusHigh.RINT1	-0.645203	4.7807703	-3.5044086	0.00078069	0.0031174	-1.0549563	RINT1	assignmentsChromaffin_cells.Chrom_SOX2_statusHigh
assignmentsChromaffin_cells.Chrom_SOX2_statusHigh.NAIF1	0.97326626	1.31986352	3.42394573	0.00100949	0.0038779	-1.0556935	NAIF1	assignmentsChromaffin_cells.Chrom_SOX2_statusHigh
assignmentsChromaffin_cells.Chrom_SOX2_statusHigh.KCTD17	0.99150842	2.33857466	3.46277745	0.00089213	0.00348974	-1.0560125	KCTD17	assignmentsChromaffin_cells.Chrom_SOX2_statusHigh
assignmentsChromaffin_cells.Chrom_SOX2_statusHigh.NUCKS1	0.95300683	7.30200566	3.55301117	0.00066727	0.00273001	-1.0564229	NUCKS1	assignmentsChromaffin_cells.Chrom_SOX2_statusHigh
assignmentsChromaffin_cells.Chrom_SOX2_statusHigh.PON3	2.33693896	0.2150436	3.35217409	0.00126574	0.00469056	-1.0581963	PON3	assignmentsChromaffin_cells.Chrom_SOX2_statusHigh
assignmentsChromaffin_cells.Chrom_SOX2_statusHigh.CCDC174	0.71220131	5.056008	3.51326505	0.00075874	0.00304523	-1.0655147	CCDC174	assignmentsChromaffin_cells.Chrom_SOX2_statusHigh
assignmentsChromaffin_cells.Chrom_SOX2_statusHigh.ANK2	-1.2174837	8.57931837	-3.5699351	0.00063196	0.00261223	-1.0658516	ANK2	assignmentsChromaffin_cells.Chrom_SOX2_statusHigh
assignmentsChromaffin_cells.Chrom_SOX2_statusHigh.JMJD6	1.13761275	4.66856143	3.50062863	0.00079023	0.00315032	-1.0667756	JMJD6	assignmentsChromaffin_cells.Chrom_SOX2_statusHigh
assignmentsChromaffin_cells.Chrom_SOX2_statusHigh.VAC14	-0.733027	4.73723775	-3.4837293	0.00083428	0.00329076	-1.0689162	VAC14	assignmentsChromaffin_cells.Chrom_SOX2_statusHigh
assignmentsChromaffin_cells.Chrom_SOX2_statusHigh.CSRNP3	-1.310615	6.00815902	-3.528843	0.00072185	0.00292155	-1.0699685	CSRNP3	assignmentsChromaffin_cells.Chrom_SOX2_statusHigh
assignmentsChromaffin_cells.Chrom_SOX2_statusHigh.URGCP	-0.8355407	5.85323506	-3.5203834	0.00074153	0.00298665	-1.0714265	URGCP	assignmentsChromaffin_cells.Chrom_SOX2_statusHigh
assignmentsChromaffin_cells.Chrom_SOX2_statusHigh.DES	3.50353338	-0.6128382	3.32766883	0.00136702	0.00499285	-1.0738242	DES	assignmentsChromaffin_cells.Chrom_SOX2_statusHigh
assignmentsChromaffin_cells.Chrom_SOX2_statusHigh.GNAS	1.44573977	10.6339977	3.59197277	0.00058869	0.00246383	-1.0761072	GNAS	assignmentsChromaffin_cells.Chrom_SOX2_statusHigh
assignmentsChromaffin_cells.Chrom_SOX2_statusHigh.IFI16	1.71882087	6.79036936	3.50490639	0.00078049	0.0031174	-1.0801125	IFI16	assignmentsChromaffin_cells.Chrom_SOX2_statusHigh
assignmentsChromaffin_cells.Chrom_SOX2_statusHigh.PCNT	-0.9955909	5.62284233	-3.4979996	0.00079694	0.00317075	-1.0811225	PCNT	assignmentsChromaffin_cells.Chrom_SOX2_statusHigh
assignmentsChromaffin_cells.Chrom_SOX2_statusHigh.DXO	1.03425862	3.00434019	3.47750827	0.00085108	0.00334794	-1.082759	DXO	assignmentsChromaffin_cells.Chrom_SOX2_statusHigh
assignmentsChromaffin_cells.Chrom_SOX2_statusHigh.TMEM117	-0.9137188	7.22317453	-3.5294776	0.00072008	0.00291707	-1.0855183	TMEM117	assignmentsChromaffin_cells.Chrom_SOX2_statusHigh
assignmentsChromaffin_cells.Chrom_SOX2_statusHigh.NRDE2	-0.7569566	5.07139603	-3.4934999	0.00080854	0.00321054	-1.0859183	NRDE2	assignmentsChromaffin_cells.Chrom_SOX2_statusHigh
assignmentsChromaffin_cells.Chrom_SOX2_statusHigh.KDM3B	-0.729375	6.45633636	-3.5155319	0.00075322	0.00302458	-1.0863001	KDM3B	assignmentsChromaffin_cells.Chrom_SOX2_statusHigh
assignmentsChromaffin_cells.Chrom_SOX2_statusHigh.MIR194-2H(	2.37036905	-0.7951975	3.32499703	0.00137789	0.00502356	-1.0870692	MIR194-2HG	assignmentsChromaffin_cells.Chrom_SOX2_statusHigh
assignmentsChromaffin_cells.Chrom_SOX2_statusHigh.FAM120A	-0.6834204	6.86418352	-3.5206073	0.000741	0.00298648	-1.0874822	FAM120A	assignmentsChromaffin_cells.Chrom_SOX2_statusHigh
assignmentsChromaffin_cells.Chrom_SOX2_statusHigh.ZNF398	-0.7366121	5.00151401	-3.4964785	0.00080084	0.00318312	-1.087805	ZNF398	assignmentsChromaffin_cells.Chrom_SOX2_statusHigh
assignmentsChromaffin_cells.Chrom_SOX2_statusHigh.CITED4	2.78210679	0.54111687	3.33712092	0.00132719	0.00487059	-1.0890485	CITED4	assignmentsChromaffin_cells.Chrom_SOX2_statusHigh
assignmentsChromaffin_cells.Chrom_SOX2_statusHigh.INE1	-1.0921387	1.30506265	-3.3732199	0.00118487	0.00443474	-1.0903354	INE1	assignmentsChromaffin_cells.Chrom_SOX2_statusHigh
assignmentsChromaffin_cells.Chrom_SOX2_statusHigh.RHBDD1	-0.7249894	6.15714144	-3.5102258	0.00076621	0.00306827	-1.0905438	RHBDD1	assignmentsChromaffin_cells.Chrom_SOX2_statusHigh
assignmentsChromaffin_cells.Chrom_SOX2_statusHigh.CDK5RAP2	-0.9001566	6.51074848	-3.5160635	0.00075193	0.00302243	-1.0918314	CDK5RAP2	assignmentsChromaffin_cells.Chrom_SOX2_statusHigh
assignmentsChromaffin_cells.Chrom_SOX2_statusHigh.PINCR	-3.1557091	-0.3198474	-3.3272846	0.00136869	0.00499599	-1.0935726	PINCR	assignmentsChromaffin_cells.Chrom_SOX2_statusHigh
assignmentsChromaffin_cells.Chrom_SOX2_statusHigh.SLC4A1AP	0.64084387	5.11140123	3.50484917	0.00077958	0.00311629	-1.0950878	SLC4A1AP	assignmentsChromaffin_cells.Chrom_SOX2_statusHigh
assignmentsChromaffin_cells.Chrom_SOX2_statusHigh.FAM72B	2.07089427	0.1191341	3.33146909	0.00135037	0.00494005	-1.0961966	FAM72B	assignmentsChromaffin_cells.Chrom_SOX2_statusHigh

assignmentsChromaffin_cells.Chrom_SOX2_statusHigh.COPG1	0.79580554	4.93704654	3.49720804	0.00079897	0.00317725	-1.0992452	COPG1	assignmentsChromaffin_cells.Chrom_SOX2_statusHigh
assignmentsChromaffin_cells.Chrom_SOX2_statusHigh.MACROD2-A	-2.1443179	0.8547299	-3.3764751	0.00117321	0.00439931	-1.100069	MACROD2-A	assignmentsChromaffin_cells.Chrom_SOX2_statusHigh
assignmentsChromaffin_cells.Chrom_SOX2_statusHigh.TAOK1	-0.6579654	7.56771865	-3.5310885	0.00071634	0.00290266	-1.1004464	TAOK1	assignmentsChromaffin_cells.Chrom_SOX2_statusHigh
assignmentsChromaffin_cells.Chrom_SOX2_statusHigh.PWP1	0.64013357	5.06972125	3.500108	0.00079156	0.00315364	-1.1018065	PWP1	assignmentsChromaffin_cells.Chrom_SOX2_statusHigh
assignmentsChromaffin_cells.Chrom_SOX2_statusHigh.GPR107	-0.771202	6.06571237	-3.5041795	0.00078126	0.00311843	-1.1028676	GPR107	assignmentsChromaffin_cells.Chrom_SOX2_statusHigh
assignmentsChromaffin_cells.Chrom_SOX2_statusHigh.SLC66A3	1.2618516	3.1669555	3.43201077	0.00098398	0.00379286	-1.1035875	SLC66A3	assignmentsChromaffin_cells.Chrom_SOX2_statusHigh
assignmentsChromaffin_cells.Chrom_SOX2_statusHigh.WASHC5-AS	-2.3460405	-0.0722515	-3.3170996	0.0014122	0.00511721	-1.1074391	WASHC5-AS1	assignmentsChromaffin_cells.Chrom_SOX2_statusHigh
assignmentsChromaffin_cells.Chrom_SOX2_statusHigh.GSEC	1.13823924	0.9434344	3.39300854	0.00111328	0.00420906	-1.1080921	GSEC	assignmentsChromaffin_cells.Chrom_SOX2_statusHigh
assignmentsChromaffin_cells.Chrom_SOX2_statusHigh.GAD1	-2.0519922	3.72340427	-3.477842	0.00085129	0.00334796	-1.1086788	GAD1	assignmentsChromaffin_cells.Chrom_SOX2_statusHigh
assignmentsChromaffin_cells.Chrom_SOX2_statusHigh.LINC00865	2.34169431	-0.3734554	3.3131372	0.00142971	0.00517016	-1.1098692	LINC00865	assignmentsChromaffin_cells.Chrom_SOX2_statusHigh
assignmentsChromaffin_cells.Chrom_SOX2_statusHigh.CALHM6	2.54545629	0.52658682	3.32345143	0.00138454	0.00504322	-1.1111639	CALHM6	assignmentsChromaffin_cells.Chrom_SOX2_statusHigh
assignmentsChromaffin_cells.Chrom_SOX2_statusHigh.SLC35B2	1.35651283	2.65127928	3.44409092	0.0009469	0.00366908	-1.1112592	SLC35B2	assignmentsChromaffin_cells.Chrom_SOX2_statusHigh
assignmentsChromaffin_cells.Chrom_SOX2_statusHigh.ALOX12	-2.3386275	0.39383995	-3.3271274	0.00136877	0.00499599	-1.112652	ALOX12	assignmentsChromaffin_cells.Chrom_SOX2_statusHigh
assignmentsChromaffin_cells.Chrom_SOX2_statusHigh.MT-ATP8	2.73933553	5.78061785	3.50996043	0.00076791	0.00307431	-1.1135658	MT-ATP8	assignmentsChromaffin_cells.Chrom_SOX2_statusHigh
assignmentsChromaffin_cells.Chrom_SOX2_statusHigh.SENP7	-0.7279339	7.23431296	-3.5158004	0.00075257	0.00302384	-1.1150652	SENP7	assignmentsChromaffin_cells.Chrom_SOX2_statusHigh
assignmentsChromaffin_cells.Chrom_SOX2_statusHigh.GAB1	-1.1659391	6.60138033	-3.4962311	0.00080148	0.00318487	-1.1174194	GAB1	assignmentsChromaffin_cells.Chrom_SOX2_statusHigh
assignmentsChromaffin_cells.Chrom_SOX2_statusHigh.COL4A5	2.81747682	3.88372714	3.49741807	0.0007995	0.00317859	-1.1174502	COL4A5	assignmentsChromaffin_cells.Chrom_SOX2_statusHigh
assignmentsChromaffin_cells.Chrom_SOX2_statusHigh.ALPL	2.36547882	0.09699292	3.31853141	0.00140592	0.00510023	-1.1177613	ALPL	assignmentsChromaffin_cells.Chrom_SOX2_statusHigh
assignmentsChromaffin_cells.Chrom_SOX2_statusHigh.CEBPD	2.467707	5.85000756	3.49817171	0.00079757	0.00317247	-1.1188858	CEBPD	assignmentsChromaffin_cells.Chrom_SOX2_statusHigh
assignmentsChromaffin_cells.Chrom_SOX2_statusHigh.AHNAK2	2.57314828	1.33751124	3.40704288	0.00106632	0.00406699	-1.1217838	AHNAK2	assignmentsChromaffin_cells.Chrom_SOX2_statusHigh
assignmentsChromaffin_cells.Chrom_SOX2_statusHigh.MAPKAPK5	-0.5594623	5.21985851	-3.4869555	0.0008257	0.00326332	-1.1223577	MAPKAPK5	assignmentsChromaffin_cells.Chrom_SOX2_statusHigh
assignmentsChromaffin_cells.Chrom_SOX2_statusHigh.RALGPS2	-1.5576465	5.73116983	-3.481068	0.0008419	0.00331589	-1.1223975	RALGPS2	assignmentsChromaffin_cells.Chrom_SOX2_statusHigh
assignmentsChromaffin_cells.Chrom_SOX2_statusHigh.RANBP3L	-2.4807031	2.23703546	-3.3937199	0.00111214	0.00420672	-1.1226584	RANBP3L	assignmentsChromaffin_cells.Chrom_SOX2_statusHigh
assignmentsChromaffin_cells.Chrom_SOX2_statusHigh.SCD5	-1.3957545	4.95457178	-3.4764756	0.00085418	0.00335766	-1.1230314	SCD5	assignmentsChromaffin_cells.Chrom_SOX2_statusHigh
assignmentsChromaffin_cells.Chrom_SOX2_statusHigh.EMX2	3.52080109	-0.8698076	3.30945851	0.00144682	0.00522029	-1.1231975	EMX2	assignmentsChromaffin_cells.Chrom_SOX2_statusHigh
assignmentsChromaffin_cells.Chrom_SOX2_statusHigh.SLC8A1	-1.921872	7.74022447	-3.5116273	0.0007638	0.00306154	-1.1268624	SLC8A1	assignmentsChromaffin_cells.Chrom_SOX2_statusHigh
assignmentsChromaffin_cells.Chrom_SOX2_statusHigh.USP11	0.78023359	5.83340012	3.51074724	0.00076492	0.00306437	-1.1270074	USP11	assignmentsChromaffin_cells.Chrom_SOX2_statusHigh
assignmentsChromaffin_cells.Chrom_SOX2_statusHigh.GUSB	0.70417317	4.93202195	3.48778647	0.0008235	0.00325704	-1.1287571	GUSB	assignmentsChromaffin_cells.Chrom_SOX2_statusHigh
assignmentsChromaffin_cells.Chrom_SOX2_statusHigh.PTPN9	-0.9748934	6.35243858	-3.4878971	0.00082321	0.00325704	-1.1325662	PTPN9	assignmentsChromaffin_cells.Chrom_SOX2_statusHigh
assignmentsChromaffin_cells.Chrom_SOX2_statusHigh.UBE3D	-0.8270236	6.23562666	-3.5049272	0.00077938	0.00311629	-1.1326526	UBE3D	assignmentsChromaffin_cells.Chrom_SOX2_statusHigh
assignmentsChromaffin_cells.Chrom_SOX2_statusHigh.AKR1C2	2.51806221	1.00080609	3.38819178	0.00113169	0.00426959	-1.1326683	AKR1C2	assignmentsChromaffin_cells.Chrom_SOX2_statusHigh
assignmentsChromaffin_cells.Chrom_SOX2_statusHigh.RNH1	1.20639449	5.01681184	3.4852994	0.00083009	0.00327827	-1.1335316	RNH1	assignmentsChromaffin_cells.Chrom_SOX2_statusHigh
assignmentsChromaffin_cells.Chrom_SOX2_statusHigh.TOPORS	0.71420404	4.91189156	3.48721933	0.000825	0.00326136	-1.1337538	TOPORS	assignmentsChromaffin_cells.Chrom_SOX2_statusHigh
assignmentsChromaffin_cells.Chrom_SOX2_statusHigh.TDRD3	-0.7894877	6.77045156	-3.5037586	0.00078232	0.00312033	-1.1395297	TDRD3	assignmentsChromaffin_cells.Chrom_SOX2_statusHigh
assignmentsChromaffin_cells.Chrom_SOX2_statusHigh.ME3	-0.9141122	5.74459625	-3.4902473	0.00081702	0.00323782	-1.1397677	ME3	assignmentsChromaffin_cells.Chrom_SOX2_statusHigh
assignmentsChromaffin_cells.Chrom_SOX2_statusHigh.HPCA	2.53512334	-0.4626948	3.32577287	0.00137495	0.00501584	-1.14005	HPCA	assignmentsChromaffin_cells.Chrom_SOX2_statusHigh
assignmentsChromaffin_cells.Chrom_SOX2_statusHigh.C19orf12	0.6097012	4.66352058	3.48306187	0.00083607	0.003297	-1.1401919	C19orf12	assignmentsChromaffin_cells.Chrom_SOX2_statusHigh
assignmentsChromaffin_cells.Chrom_SOX2_statusHigh.CRYZL1	-0.6445017	5.92035735	-3.494073	0.00080705	0.00320543	-1.1424291	CRYZL1	assignmentsChromaffin_cells.Chrom_SOX2_statusHigh
assignmentsChromaffin_cells.Chrom_SOX2_statusHigh.DHH	2.43465515	0.43193748	3.34160044	0.00130876	0.00481972	-1.1425373	DHH	assignmentsChromaffin_cells.Chrom_SOX2_statusHigh
assignmentsChromaffin_cells.Chrom_SOX2_statusHigh.SFI1	-0.7424304	5.1853697	-3.4701728	0.00087129	0.0034174	-1.1444211	SFI1	assignmentsChromaffin_cells.Chrom_SOX2_statusHigh
assignmentsChromaffin_cells.Chrom_SOX2_statusHigh.LINC01226	2.12332542	-0.1149852	3.32001783	0.00139943	0.00508473	-1.1448338	LINC01226	assignmentsChromaffin_cells.Chrom_SOX2_statusHigh
assignmentsChromaffin_cells.Chrom_SOX2_statusHigh.OASL	2.16877216	0.89134622	3.31255671	0.00143229	0.00517833	-1.1449374	OASL	assignmentsChromaffin_cells.Chrom_SOX2_statusHigh
assignmentsChromaffin_cells.Chrom_SOX2_statusHigh.SPDYE1	-2.1594223	1.88159588	-3.3877623	0.00113244	0.00427142	-1.1454881	SPDYE1	assignmentsChromaffin_cells.Chrom_SOX2_statusHigh
assignmentsChromaffin_cells.Chrom_SOX2_statusHigh.USP20	-0.8721603	4.65821779	-3.4605934	0.00089837	0.00350902	-1.1459722	USP20	assignmentsChromaffin_cells.Chrom_SOX2_statusHigh
assignmentsChromaffin_cells.Chrom_SOX2_statusHigh.ALOX12-AS1	-0.8672542	5.71195571	-3.4804635	0.00084306	0.00331966	-1.1460387	ALOX12-AS1	assignmentsChromaffin_cells.Chrom_SOX2_statusHigh
assignmentsChromaffin_cells.Chrom_SOX2_statusHigh.SCAMP1-AS	1.04662106	2.34073985	3.43191333	0.00098428	0.00379287	-1.1471958	SCAMP1-AS1	assignmentsChromaffin_cells.Chrom_SOX2_statusHigh
assignmentsChromaffin_cells.Chrom_SOX2_statusHigh.CAMK1	0.91552357	3.5291285	3.45519941	0.00091397	0.00356046	-1.1477562	CAMK1	assignmentsChromaffin_cells.Chrom_SOX2_statusHigh
assignmentsChromaffin_cells.Chrom_SOX2_statusHigh.SETD3	-0.6309271	6.25442849	-3.489303	0.0008195	0.00324684	-1.1482503	SETD3	assignmentsChromaffin_cells.Chrom_SOX2_statusHigh
assignmentsChromaffin_cells.Chrom_SOX2_statusHigh.NDRG1	2.25932767	5.54760375	3.47797645	0.00085092	0.00334794	-1.148724	NDRG1	assignmentsChromaffin_cells.Chrom_SOX2_statusHigh
assignmentsChromaffin_cells.Chrom_SOX2_statusHigh.PDLIM2	2.08769013	3.82561401	3.44890951	0.00093367	0.00362397	-1.1490483	PDLIM2	assignmentsChromaffin_cells.Chrom_SOX2_statusHigh
assignmentsChromaffin_cells.Chrom_SOX2_statusHigh.GSK3A	1.0478902	3.5371895	3.45521387	0.00091392	0.00356046	-1.1505692	GSK3A	assignmentsChromaffin_cells.Chrom_SOX2_statusHigh
assignmentsChromaffin_cells.Chrom_SOX2_statusHigh.CCDC39	-2.2951082	1.00244683	-3.3283886	0.00136341	0.00498207	-1.1526412	CCDC39	assignmentsChromaffin_cells.Chrom_SOX2_statusHigh
assignmentsChromaffin_cells.Chrom_SOX2_statusHigh.LARP7	0.64076961	5.64194538	3.49132452	0.00081421	0.00322939	-1.1581492	LARP7	assignmentsChromaffin_cells.Chrom_SOX2_statusHigh
assignmentsChromaffin_cells.Chrom_SOX2_statusHigh.FAM3A	0.84481647	3.48595681	3.45250029	0.00092187	0.00358595	-1.1583584	FAM3A	assignmentsChromaffin_cells.Chrom_SOX2_statusHigh
assignmentsChromaffin_cells.Chrom_SOX2_statusHigh.C19orf25	0.70742941	4.80143999	3.4796609	0.00084523	0.00332739	-1.1595137	C19orf25	assignmentsChromaffin_cells.Chrom_SOX2_statusHigh
assignmentsChromaffin_cells.Chrom_SOX2_statusHigh.CDK2AP1	1.36750221	4.662763	3.47743077	0.00085161	0.00334841	-1.1595501	CDK2AP1	assignmentsChromaffin_cells.Chrom_SOX2_statusHigh
assignmentsChromaffin_cells.Chrom_SOX2_statusHigh.HMGN4	0.69969135	4.74676366	3.47493268	0.00085812	0.00337235	-1.1600053	HMGN4	assignmentsChromaffin_cells.Chrom_SOX2_statusHigh
assignmentsChromaffin_cells.Chrom_SOX2_statusHigh.TNFAIP3	2.49200455	5.59609449	3.42776693	0.00099858	0.00384243	-1.1616518	TNFAIP3	assignmentsChromaffin_cells.Chrom_SOX2_statusHigh
assignmentsChromaffin_cells.Chrom_SOX2_statusHigh.NPY	5.86972975	2.9335591	3.44326943	0.00095059	0.00368249	-1.1631662	NPY	assignmentsChromaffin_cells.Chrom_SOX2_statusHigh
assignmentsChromaffin_cells.Chrom_SOX2_statusHigh.MYCBP2	-0.6738841	8.50719288	-3.5203017	0.00074173	0.00298665	-1.163269	MYCBP2	assignmentsChromaffin_cells.Chrom_SOX2_statusHigh
assignmentsChromaffin_cells.Chrom_SOX2_statusHigh.OCIAD1	0.62617171	7.12986467	3.51668792	0.00075042	0.00301741	-1.1635466	OCIAD1	assignmentsChromaffin_cells.Chrom_SOX2_statusHigh
assignmentsChromaffin_cells.Chrom_SOX2_statusHigh.UBA7	1.85874644	2.35270941	3.3446017	0.0012961	0.00477748	-1.1648581	UBA7	assignmentsChromaffin_cells.Chrom_SOX2_statusHigh
assignmentsChromaffin_cells.Chrom_SOX2_statusHigh.FBH1	-0.6374351	5.66471493	-3.478076	0.00084953	0.0033435	-1.1661927	FBH1	assignmentsChromaffin_cells.Chrom_SOX2_statusHigh
assignmentsChromaffin_cells.Chrom_SOX2_statusHigh.PI15	2.48122307	1.55495879	3.41064859	0.00105423	0.00402662	-1.1663054	PI15	assignmentsChromaffin_cells.Chrom_SOX2_statusHigh

assignmentsChromaffin_cells.Chrom_SOX2_statusHigh.SLC44A1	-0.8084229	6.1236723	-3.4827697	0.00083685	0.00329846	-1.1663116	SLC44A1	assignmentsChromaffin_cells.Chrom_SOX2_statusHigh
assignmentsChromaffin_cells.Chrom_SOX2_statusHigh.CACNA1C-A	-2.7396718	0.94972803	-3.3199768	0.00140027	0.00508571	-1.1663524	CACNA1C-AS	assignmentsChromaffin_cells.Chrom_SOX2_statusHigh
assignmentsChromaffin_cells.Chrom_SOX2_statusHigh.DBP	1.12633269	2.79442684	3.44684031	0.00093864	0.00364015	-1.1663752	DBP	assignmentsChromaffin_cells.Chrom_SOX2_statusHigh
assignmentsChromaffin_cells.Chrom_SOX2_statusHigh.SERPIND1	-2.2278901	0.99526586	-3.3182376	0.0014072	0.00510143	-1.1691509	SERPIND1	assignmentsChromaffin_cells.Chrom_SOX2_statusHigh
assignmentsChromaffin_cells.Chrom_SOX2_statusHigh.MN1	2.42131458	1.51226386	3.31434608	0.00142435	0.00515309	-1.1733227	MN1	assignmentsChromaffin_cells.Chrom_SOX2_statusHigh
assignmentsChromaffin_cells.Chrom_SOX2_statusHigh.OTUB1	1.16325071	4.6362641	3.47002305	0.00087171	0.0034182	-1.1751536	OTUB1	assignmentsChromaffin_cells.Chrom_SOX2_statusHigh
assignmentsChromaffin_cells.Chrom_SOX2_statusHigh.RPL23	2.44676237	6.30367881	3.49857079	0.00079655	0.00317075	-1.1773926	RPL23	assignmentsChromaffin_cells.Chrom_SOX2_statusHigh
assignmentsChromaffin_cells.Chrom_SOX2_statusHigh.GCFC2	-0.8089469	5.89390972	-3.4839507	0.00083369	0.00328923	-1.1791138	GCFC2	assignmentsChromaffin_cells.Chrom_SOX2_statusHigh
assignmentsChromaffin_cells.Chrom_SOX2_statusHigh.THOC6	1.22043341	2.55525713	3.42406586	0.00100911	0.00387736	-1.179539	THOC6	assignmentsChromaffin_cells.Chrom_SOX2_statusHigh
assignmentsChromaffin_cells.Chrom_SOX2_statusHigh.ASF1B	1.31354713	1.10996086	3.37416452	0.00118135	0.00442366	-1.1797674	ASF1B	assignmentsChromaffin_cells.Chrom_SOX2_statusHigh
assignmentsChromaffin_cells.Chrom_SOX2_statusHigh.KPNB1	0.54109195	6.87556616	3.50248573	0.00078553	0.00313235	-1.1803389	KPNB1	assignmentsChromaffin_cells.Chrom_SOX2_statusHigh
assignmentsChromaffin_cells.Chrom_SOX2_statusHigh.LINC00957	0.98562814	1.26112777	3.40679231	0.00106585	0.00406617	-1.1808919	LINC00957	assignmentsChromaffin_cells.Chrom_SOX2_statusHigh
assignmentsChromaffin_cells.Chrom_SOX2_statusHigh.ZNF605	-0.8809557	4.34953288	-3.45118	0.00092575	0.00359846	-1.181139	ZNF605	assignmentsChromaffin_cells.Chrom_SOX2_statusHigh
assignmentsChromaffin_cells.Chrom_SOX2_statusHigh.TBL1XR1	-0.9261199	7.8781955	-3.500159	0.00079143	0.00315364	-1.181595	TBL1XR1	assignmentsChromaffin_cells.Chrom_SOX2_statusHigh
assignmentsChromaffin_cells.Chrom_SOX2_statusHigh.CHPF	1.78083197	4.13355214	3.48179435	0.00084058	0.00331154	-1.1829608	CHPF	assignmentsChromaffin_cells.Chrom_SOX2_statusHigh
assignmentsChromaffin_cells.Chrom_SOX2_statusHigh.NLE1	1.00623123	1.41393586	3.3847345	0.0011427	0.00430406	-1.1858426	NLE1	assignmentsChromaffin_cells.Chrom_SOX2_statusHigh
assignmentsChromaffin_cells.Chrom_SOX2_statusHigh.SRSF12	-1.0941432	2.84568921	-3.4108602	0.00105223	0.00402222	-1.1877923	SRSF12	assignmentsChromaffin_cells.Chrom_SOX2_statusHigh
assignmentsChromaffin_cells.Chrom_SOX2_statusHigh.ANKRD6	-1.0304227	5.44724381	-3.4544826	0.00091606	0.00356682	-1.1888904	ANKRD6	assignmentsChromaffin_cells.Chrom_SOX2_statusHigh
assignmentsChromaffin_cells.Chrom_SOX2_statusHigh.ZFP64	-0.8766752	4.83017501	-3.4545633	0.00091582	0.00356677	-1.189619	ZFP64	assignmentsChromaffin_cells.Chrom_SOX2_statusHigh
assignmentsChromaffin_cells.Chrom_SOX2_statusHigh.CD274	1.31728383	1.78973171	3.37897736	0.0011636	0.00436637	-1.1897512	CD274	assignmentsChromaffin_cells.Chrom_SOX2_statusHigh
assignmentsChromaffin_cells.Chrom_SOX2_statusHigh.TAF6	0.80531402	3.21844543	3.44159067	0.00095446	0.00369482	-1.1899359	TAF6	assignmentsChromaffin_cells.Chrom_SOX2_statusHigh
assignmentsChromaffin_cells.Chrom_SOX2_statusHigh.CYP27A1	2.0313399	2.95653449	3.39808239	0.00109656	0.00415761	-1.1921562	CYP27A1	assignmentsChromaffin_cells.Chrom_SOX2_statusHigh
assignmentsChromaffin_cells.Chrom_SOX2_statusHigh.WDR17	-1.1764733	5.47779239	-3.4876663	0.00082382	0.0032575	-1.193197	WDR17	assignmentsChromaffin_cells.Chrom_SOX2_statusHigh
assignmentsChromaffin_cells.Chrom_SOX2_statusHigh.DERL1	0.86078304	5.01376918	3.46956459	0.00087299	0.00342238	-1.1954137	DERL1	assignmentsChromaffin_cells.Chrom_SOX2_statusHigh
assignmentsChromaffin_cells.Chrom_SOX2_statusHigh.FAM193A	-1.0190415	7.11775889	-3.4886384	0.00082125	0.00325216	-1.1958974	FAM193A	assignmentsChromaffin_cells.Chrom_SOX2_statusHigh
assignmentsChromaffin_cells.Chrom_SOX2_statusHigh.MRM3	1.05132508	2.4278467	3.41174581	0.00104928	0.00401344	-1.2001594	MRM3	assignmentsChromaffin_cells.Chrom_SOX2_statusHigh
assignmentsChromaffin_cells.Chrom_SOX2_statusHigh.LINGO1	-1.9036503	3.52232255	-3.4239488	0.00101055	0.00388104	-1.201693	LINGO1	assignmentsChromaffin_cells.Chrom_SOX2_statusHigh
assignmentsChromaffin_cells.Chrom_SOX2_statusHigh.ABHD8	0.81839052	3.56593888	3.44833809	0.00093418	0.00362504	-1.2027128	ABHD8	assignmentsChromaffin_cells.Chrom_SOX2_statusHigh
assignmentsChromaffin_cells.Chrom_SOX2_statusHigh.CMYA5	1.82320254	2.30647739	3.37207918	0.00118947	0.0044499	-1.2031499	CMYA5	assignmentsChromaffin_cells.Chrom_SOX2_statusHigh
assignmentsChromaffin_cells.Chrom_SOX2_statusHigh.PCSK7	-0.7787217	5.90016859	-3.4601867	0.00089954	0.00351273	-1.2048659	PCSK7	assignmentsChromaffin_cells.Chrom_SOX2_statusHigh
assignmentsChromaffin_cells.Chrom_SOX2_statusHigh.TBRG4	0.77649272	3.63851934	3.43594498	0.00097175	0.0037509	-1.204901	TBRG4	assignmentsChromaffin_cells.Chrom_SOX2_statusHigh
assignmentsChromaffin_cells.Chrom_SOX2_statusHigh.AMN1	-0.706989	5.09389149	-3.4539484	0.00091762	0.00357117	-1.2080112	AMN1	assignmentsChromaffin_cells.Chrom_SOX2_statusHigh
assignmentsChromaffin_cells.Chrom_SOX2_statusHigh.RPS27L	1.32133652	6.04357957	3.48244919	0.00083811	0.00330261	-1.2090424	RPS27L	assignmentsChromaffin_cells.Chrom_SOX2_statusHigh
assignmentsChromaffin_cells.Chrom_SOX2_statusHigh.BPGM	1.30285776	3.15476973	3.41422557	0.00104108	0.00398872	-1.2097043	BPGM	assignmentsChromaffin_cells.Chrom_SOX2_statusHigh
assignmentsChromaffin_cells.Chrom_SOX2_statusHigh.ZNF81	-0.6888807	5.09839418	-3.4492169	0.00093156	0.00361753	-1.2139857	ZNF81	assignmentsChromaffin_cells.Chrom_SOX2_statusHigh
assignmentsChromaffin_cells.Chrom_SOX2_statusHigh.WDR64	-3.107544	0.74548156	-3.2847052	0.0015622	0.00556519	-1.2149135	WDR64	assignmentsChromaffin_cells.Chrom_SOX2_statusHigh
assignmentsChromaffin_cells.Chrom_SOX2_statusHigh.C6	1.91262146	0.06845543	3.3105505	0.00144125	0.00520603	-1.2166223	C6	assignmentsChromaffin_cells.Chrom_SOX2_statusHigh
assignmentsChromaffin_cells.Chrom_SOX2_statusHigh.STC2	2.24224906	1.05027108	3.29704056	0.00150297	0.00538891	-1.2168357	STC2	assignmentsChromaffin_cells.Chrom_SOX2_statusHigh
assignmentsChromaffin_cells.Chrom_SOX2_statusHigh.KLF16	1.43311417	2.01404352	3.38303696	0.00114883	0.00432104	-1.2201242	KLF16	assignmentsChromaffin_cells.Chrom_SOX2_statusHigh
assignmentsChromaffin_cells.Chrom_SOX2_statusHigh.MIR193BHG	-2.3677192	2.58689485	-3.4014049	0.00108549	0.00412049	-1.2220853	MIR193BHG	assignmentsChromaffin_cells.Chrom_SOX2_statusHigh
assignmentsChromaffin_cells.Chrom_SOX2_statusHigh.GADD45G	4.50054634	1.53919534	3.29088651	0.00153358	0.00547907	-1.2231014	GADD45G	assignmentsChromaffin_cells.Chrom_SOX2_statusHigh
assignmentsChromaffin_cells.Chrom_SOX2_statusHigh.KDM3A	-0.7903101	6.37094774	-3.4711607	0.00086854	0.00340745	-1.2236051	KDM3A	assignmentsChromaffin_cells.Chrom_SOX2_statusHigh
assignmentsChromaffin_cells.Chrom_SOX2_statusHigh.ZNF607	-1.2743527	3.02369082	-3.404319	0.00107422	0.00409031	-1.2237335	ZNF607	assignmentsChromaffin_cells.Chrom_SOX2_statusHigh
assignmentsChromaffin_cells.Chrom_SOX2_statusHigh.CABIN1	-0.730797	6.22251662	-3.4688522	0.00087498	0.0034285	-1.2244277	CABIN1	assignmentsChromaffin_cells.Chrom_SOX2_statusHigh
assignmentsChromaffin_cells.Chrom_SOX2_statusHigh.HSPE1	2.04983039	6.51896529	3.4908785	0.00081646	0.00323638	-1.227894	HSPE1	assignmentsChromaffin_cells.Chrom_SOX2_statusHigh
assignmentsChromaffin_cells.Chrom_SOX2_statusHigh.TRMT10A	0.82202228	2.67722053	3.42157333	0.00101712	0.00390345	-1.2287065	TRMT10A	assignmentsChromaffin_cells.Chrom_SOX2_statusHigh
assignmentsChromaffin_cells.Chrom_SOX2_statusHigh.ZNF222	0.74664331	1.89320873	3.39685078	0.00109987	0.00416816	-1.2293263	ZNF222	assignmentsChromaffin_cells.Chrom_SOX2_statusHigh
assignmentsChromaffin_cells.Chrom_SOX2_statusHigh.TMEM151A	1.81887703	1.18216825	3.4034559	0.00107775	0.00410082	-1.2311599	TMEM151A	assignmentsChromaffin_cells.Chrom_SOX2_statusHigh
assignmentsChromaffin_cells.Chrom_SOX2_statusHigh.ADRB2	2.74354939	1.54074909	3.30034398	0.00148834	0.00534244	-1.2325975	ADRB2	assignmentsChromaffin_cells.Chrom_SOX2_statusHigh
assignmentsChromaffin_cells.Chrom_SOX2_statusHigh.SLC8A1-AS1	-2.3924433	3.12652365	-3.3645602	0.00121897	0.00454064	-1.2335819	SLC8A1-AS1	assignmentsChromaffin_cells.Chrom_SOX2_statusHigh
assignmentsChromaffin_cells.Chrom_SOX2_statusHigh.ENOSF1	-0.8679952	6.52938313	-3.4661776	0.00088249	0.00345456	-1.235135	ENOSF1	assignmentsChromaffin_cells.Chrom_SOX2_statusHigh
assignmentsChromaffin_cells.Chrom_SOX2_statusHigh.NPAT	-0.632484	5.77967908	-3.4521868	0.00092279	0.00358867	-1.2354494	NPAT	assignmentsChromaffin_cells.Chrom_SOX2_statusHigh
assignmentsChromaffin_cells.Chrom_SOX2_statusHigh.TOE1	0.82879923	2.05866103	3.3792126	0.00116274	0.00436416	-1.2355792	TOE1	assignmentsChromaffin_cells.Chrom_SOX2_statusHigh
assignmentsChromaffin_cells.Chrom_SOX2_statusHigh.IST1	-0.4477165	6.45665397	-3.4680163	0.00087732	0.00343684	-1.2356334	IST1	assignmentsChromaffin_cells.Chrom_SOX2_statusHigh
assignmentsChromaffin_cells.Chrom_SOX2_statusHigh.POMZP3	-1.003159	3.67452291	-3.413922	0.00104208	0.0039916	-1.2367207	POMZP3	assignmentsChromaffin_cells.Chrom_SOX2_statusHigh
assignmentsChromaffin_cells.Chrom_SOX2_statusHigh.LRRC58	0.74955745	4.89892017	3.45007348	0.00092902	0.0036103	-1.2378223	LRRC58	assignmentsChromaffin_cells.Chrom_SOX2_statusHigh
assignmentsChromaffin_cells.Chrom_SOX2_statusHigh.SCNM1	1.1417866	3.82253978	3.43198923	0.00098404	0.00379286	-1.2387264	SCNM1	assignmentsChromaffin_cells.Chrom_SOX2_statusHigh
assignmentsChromaffin_cells.Chrom_SOX2_statusHigh.HOXA5	2.57870302	1.66309543	3.39328919	0.00111365	0.00420946	-1.2395059	HOXA5	assignmentsChromaffin_cells.Chrom_SOX2_statusHigh
assignmentsChromaffin_cells.Chrom_SOX2_statusHigh.CCDC3	-1.7694744	4.32191	-3.395371	0.00110558	0.00418585	-1.2418701	CCDC3	assignmentsChromaffin_cells.Chrom_SOX2_statusHigh
assignmentsChromaffin_cells.Chrom_SOX2_statusHigh.AKIRIN2	0.76124685	5.30966589	3.44942253	0.00093095	0.00361604	-1.2430737	AKIRIN2	assignmentsChromaffin_cells.Chrom_SOX2_statusHigh
assignmentsChromaffin_cells.Chrom_SOX2_statusHigh.GAS2	-1.3456562	3.57379693	-3.4117664	0.00104921	0.00401344	-1.2435711	GAS2	assignmentsChromaffin_cells.Chrom_SOX2_statusHigh
assignmentsChromaffin_cells.Chrom_SOX2_statusHigh.ATRN	-0.7140651	6.5921657	-3.4625868	0.00089267	0.00349101	-1.2438674	ATRN	assignmentsChromaffin_cells.Chrom_SOX2_statusHigh
assignmentsChromaffin_cells.Chrom_SOX2_statusHigh.RAB32	1.21501753	3.11210723	3.40365066	0.00107649	0.00409799	-1.244168	RAB32	assignmentsChromaffin_cells.Chrom_SOX2_statusHigh
assignmentsChromaffin_cells.Chrom_SOX2_statusHigh.ZFYVE21	1.00605123	3.96291343	3.43949461	0.00096085	0.00371774	-1.2448014	ZFYVE21	assignmentsChromaffin_cells.Chrom_SOX2_statusHigh

assignmentsChromaffin_cells.Chrom_SOX2_statusHigh.APPBP2	-0.6044392	6.82660183	-3.4722714	0.00086546	0.00339703	-1.2458869	APPBP2	assignmentsChromaffin_cells.Chrom_SOX2_statusHigh
assignmentsChromaffin_cells.Chrom_SOX2_statusHigh.TRIM62	0.97664619	3.58846567	3.42450369	0.00100771	0.0038729	-1.2464122	TRIM62	assignmentsChromaffin_cells.Chrom_SOX2_statusHigh
assignmentsChromaffin_cells.Chrom_SOX2_statusHigh.RALYL	-3.2266326	8.64641613	-3.5052018	0.00077975	0.00311629	-1.2471211	RALYL	assignmentsChromaffin_cells.Chrom_SOX2_statusHigh
assignmentsChromaffin_cells.Chrom_SOX2_statusHigh.SLC25A22	1.21970141	2.17020296	3.40109625	0.00108522	0.00412044	-1.2483414	SLC25A22	assignmentsChromaffin_cells.Chrom_SOX2_statusHigh
assignmentsChromaffin_cells.Chrom_SOX2_statusHigh.FMNL2	-1.212144	8.10806372	-3.4744668	0.00085969	0.00337624	-1.2487012	FMNL2	assignmentsChromaffin_cells.Chrom_SOX2_statusHigh
assignmentsChromaffin_cells.Chrom_SOX2_statusHigh.RILPL1	-0.8471744	5.11317838	-3.436958	0.00096863	0.00374244	-1.2489878	RILPL1	assignmentsChromaffin_cells.Chrom_SOX2_statusHigh
assignmentsChromaffin_cells.Chrom_SOX2_statusHigh.HOXC-AS1	2.40604801	0.32165034	3.3511207	0.00127141	0.00470811	-1.2503796	HOXC-AS1	assignmentsChromaffin_cells.Chrom_SOX2_statusHigh
assignmentsChromaffin_cells.Chrom_SOX2_statusHigh.INTS3	-0.7608132	5.69973435	-3.456319	0.00091071	0.00355116	-1.250626	INTS3	assignmentsChromaffin_cells.Chrom_SOX2_statusHigh
assignmentsChromaffin_cells.Chrom_SOX2_statusHigh.INTS4	-0.839945	5.54365322	-3.4427747	0.00095087	0.0036827	-1.251041	INTS4	assignmentsChromaffin_cells.Chrom_SOX2_statusHigh
assignmentsChromaffin_cells.Chrom_SOX2_statusHigh.SCIMP	-2.4006087	1.92755282	-3.300221	0.0014887	0.00534254	-1.2552303	SCIMP	assignmentsChromaffin_cells.Chrom_SOX2_statusHigh
assignmentsChromaffin_cells.Chrom_SOX2_statusHigh.DPP10	-3.9445098	3.04222351	-3.3595985	0.00123809	0.00459854	-1.2601241	DPP10	assignmentsChromaffin_cells.Chrom_SOX2_statusHigh
assignmentsChromaffin_cells.Chrom_SOX2_statusHigh.DZIP1L	-1.061927	4.16352234	-3.407793	0.00106249	0.00405429	-1.2636678	DZIP1L	assignmentsChromaffin_cells.Chrom_SOX2_statusHigh
assignmentsChromaffin_cells.Chrom_SOX2_statusHigh.PSMC3IP	1.05760232	1.45805927	3.35978171	0.00123593	0.00459157	-1.265207	PSMC3IP	assignmentsChromaffin_cells.Chrom_SOX2_statusHigh
assignmentsChromaffin_cells.Chrom_SOX2_statusHigh.VPS53	-0.7505088	7.05424195	-3.4662116	0.00088239	0.00345456	-1.2660507	VPS53	assignmentsChromaffin_cells.Chrom_SOX2_statusHigh
assignmentsChromaffin_cells.Chrom_SOX2_statusHigh.AMIGO2	3.00413847	2.01354211	3.33975132	0.00131741	0.00484269	-1.2674671	AMIGO2	assignmentsChromaffin_cells.Chrom_SOX2_statusHigh
assignmentsChromaffin_cells.Chrom_SOX2_statusHigh.SMPD2	1.00679423	2.36643772	3.3823841	0.00115119	0.00432689	-1.2686959	SMPD2	assignmentsChromaffin_cells.Chrom_SOX2_statusHigh
assignmentsChromaffin_cells.Chrom_SOX2_statusHigh.SOGA1	-1.0489459	5.37512424	-3.4280446	0.00099645	0.00383523	-1.269616	SOGA1	assignmentsChromaffin_cells.Chrom_SOX2_statusHigh
assignmentsChromaffin_cells.Chrom_SOX2_statusHigh.KDM2B-DT	-1.3928951	2.78597402	-3.3824127	0.00115109	0.00432689	-1.2701933	KDM2B-DT	assignmentsChromaffin_cells.Chrom_SOX2_statusHigh
assignmentsChromaffin_cells.Chrom_SOX2_statusHigh.MAP7D3	0.93298686	4.15938803	3.41067888	0.00105283	0.00402222	-1.2735024	MAP7D3	assignmentsChromaffin_cells.Chrom_SOX2_statusHigh
assignmentsChromaffin_cells.Chrom_SOX2_statusHigh.COL6A1	1.77901213	3.49676296	3.36176521	0.00122868	0.00456888	-1.2748286	COL6A1	assignmentsChromaffin_cells.Chrom_SOX2_statusHigh
assignmentsChromaffin_cells.Chrom_SOX2_statusHigh.EMC2	0.7991128	5.6211612	3.45403968	0.00091735	0.00357099	-1.2760371	EMC2	assignmentsChromaffin_cells.Chrom_SOX2_statusHigh
assignmentsChromaffin_cells.Chrom_SOX2_statusHigh.TMCC1	-0.9176385	7.61439962	-3.4667948	0.00088075	0.00344944	-1.2772081	TMCC1	assignmentsChromaffin_cells.Chrom_SOX2_statusHigh
assignmentsChromaffin_cells.Chrom_SOX2_statusHigh.TMX1	0.987412	5.06496692	3.44054751	0.00095763	0.00370621	-1.2773943	TMX1	assignmentsChromaffin_cells.Chrom_SOX2_statusHigh
assignmentsChromaffin_cells.Chrom_SOX2_statusHigh.CRYAB	2.99286374	2.2482691	3.32240672	0.00139063	0.00506079	-1.2788853	CRYAB	assignmentsChromaffin_cells.Chrom_SOX2_statusHigh
assignmentsChromaffin_cells.Chrom_SOX2_statusHigh.SYTL2	-1.5239719	4.32463591	-3.3695016	0.00119879	0.00447643	-1.2791789	SYTL2	assignmentsChromaffin_cells.Chrom_SOX2_statusHigh
assignmentsChromaffin_cells.Chrom_SOX2_statusHigh.SYNGR3	1.34011484	1.11829971	3.38282789	0.00114959	0.00432287	-1.2836451	SYNGR3	assignmentsChromaffin_cells.Chrom_SOX2_statusHigh
assignmentsChromaffin_cells.Chrom_SOX2_statusHigh.CLDN20	-1.8416771	0.8875008	-3.2967354	0.00150439	0.0053916	-1.2854699	CLDN20	assignmentsChromaffin_cells.Chrom_SOX2_statusHigh
assignmentsChromaffin_cells.Chrom_SOX2_statusHigh.LINC02740	2.93662937	0.75971888	3.34895616	0.00128005	0.00473246	-1.2870802	LINC02740	assignmentsChromaffin_cells.Chrom_SOX2_statusHigh
assignmentsChromaffin_cells.Chrom_SOX2_statusHigh.CEP250	-0.8968159	5.12807317	-3.4340345	0.00097767	0.00377011	-1.2880074	CEP250	assignmentsChromaffin_cells.Chrom_SOX2_statusHigh
assignmentsChromaffin_cells.Chrom_SOX2_statusHigh.TMEM248	0.6552029	5.64106415	3.44561519	0.00094231	0.0036522	-1.289911	TMEM248	assignmentsChromaffin_cells.Chrom_SOX2_statusHigh
assignmentsChromaffin_cells.Chrom_SOX2_statusHigh.FGF18	1.72647094	0.86820375	3.32034607	0.001398	0.00508069	-1.2901063	FGF18	assignmentsChromaffin_cells.Chrom_SOX2_statusHigh
assignmentsChromaffin_cells.Chrom_SOX2_statusHigh.SEH1L	-0.7903698	4.68761047	-3.4122101	0.00104774	0.00401115	-1.2904426	SEH1L	assignmentsChromaffin_cells.Chrom_SOX2_statusHigh
assignmentsChromaffin_cells.Chrom_SOX2_statusHigh.MYLK4	-1.2413197	2.24618256	-3.3472989	0.00128521	0.00474607	-1.2912792	MYLK4	assignmentsChromaffin_cells.Chrom_SOX2_statusHigh
assignmentsChromaffin_cells.Chrom_SOX2_statusHigh.HDGF	0.91338811	4.86365547	3.43432175	0.00097678	0.00376758	-1.2924828	HDGF	assignmentsChromaffin_cells.Chrom_SOX2_statusHigh
assignmentsChromaffin_cells.Chrom_SOX2_statusHigh.VIM	2.22993276	7.59194769	3.4361944	0.00097221	0.00375177	-1.2943224	VIM	assignmentsChromaffin_cells.Chrom_SOX2_statusHigh
assignmentsChromaffin_cells.Chrom_SOX2_statusHigh.HOXB4	2.0329707	2.6322482	3.37094851	0.00119471	0.00446326	-1.3007572	HOXB4	assignmentsChromaffin_cells.Chrom_SOX2_statusHigh
assignmentsChromaffin_cells.Chrom_SOX2_statusHigh.BYSL	1.06629792	1.92267471	3.36228945	0.00122624	0.00456299	-1.3030723	BYSL	assignmentsChromaffin_cells.Chrom_SOX2_statusHigh
assignmentsChromaffin_cells.Chrom_SOX2_statusHigh.UBOX5-AS1	-2.7146369	1.99213577	-3.3032453	0.00147596	0.00530273	-1.3032752	UBOX5-AS1	assignmentsChromaffin_cells.Chrom_SOX2_statusHigh
assignmentsChromaffin_cells.Chrom_SOX2_statusHigh.LINC02208	-1.8564641	1.64970881	-3.3212828	0.00139406	0.00507097	-1.3038203	LINC02208	assignmentsChromaffin_cells.Chrom_SOX2_statusHigh
assignmentsChromaffin_cells.Chrom_SOX2_statusHigh.SERP1	1.00491016	6.43763832	3.45797187	0.00090592	0.0035342	-1.3041695	SERP1	assignmentsChromaffin_cells.Chrom_SOX2_statusHigh
assignmentsChromaffin_cells.Chrom_SOX2_statusHigh.NADK2	-0.8110479	4.94848653	-3.4205432	0.00102044	0.00391435	-1.3042983	NADK2	assignmentsChromaffin_cells.Chrom_SOX2_statusHigh
assignmentsChromaffin_cells.Chrom_SOX2_statusHigh.RHOB	1.99754749	6.33051679	3.4375841	0.00096793	0.00374064	-1.3071806	RHOB	assignmentsChromaffin_cells.Chrom_SOX2_statusHigh
assignmentsChromaffin_cells.Chrom_SOX2_statusHigh.DPYSL5	-1.5100199	3.35643264	-3.4057677	0.00106978	0.00407534	-1.3073563	DPYSL5	assignmentsChromaffin_cells.Chrom_SOX2_statusHigh
assignmentsChromaffin_cells.Chrom_SOX2_statusHigh.ANKRD10-IT	-1.4932453	2.75859163	-3.3324231	0.00134635	0.00492776	-1.3096282	ANKRD10-IT:	assignmentsChromaffin_cells.Chrom_SOX2_statusHigh
assignmentsChromaffin_cells.Chrom_SOX2_statusHigh.CCDC15-DT	1.63771376	0.05356793	3.27933802	0.00158761	0.00564568	-1.3135432	CCDC15-DT	assignmentsChromaffin_cells.Chrom_SOX2_statusHigh
assignmentsChromaffin_cells.Chrom_SOX2_statusHigh.CERS4	-0.8159006	5.30879903	-3.4253855	0.00100489	0.00386486	-1.3168576	CERS4	assignmentsChromaffin_cells.Chrom_SOX2_statusHigh
assignmentsChromaffin_cells.Chrom_SOX2_statusHigh.NT5C	1.15819486	4.53380365	3.42070445	0.00101992	0.00391328	-1.3178486	NT5C	assignmentsChromaffin_cells.Chrom_SOX2_statusHigh
assignmentsChromaffin_cells.Chrom_SOX2_statusHigh.KDSR	0.78825743	5.40900589	3.43646267	0.00097015	0.00374563	-1.3181784	KDSR	assignmentsChromaffin_cells.Chrom_SOX2_statusHigh
assignmentsChromaffin_cells.Chrom_SOX2_statusHigh.DOLPP1	0.99559985	1.26891104	3.34938253	0.00127686	0.00472392	-1.318419	DOLPP1	assignmentsChromaffin_cells.Chrom_SOX2_statusHigh
assignmentsChromaffin_cells.Chrom_SOX2_statusHigh.TGFBRAP1	-0.7897105	4.20987747	-3.394386	0.00110846	0.00419477	-1.3196506	TGFBRAP1	assignmentsChromaffin_cells.Chrom_SOX2_statusHigh
assignmentsChromaffin_cells.Chrom_SOX2_statusHigh.SOX10	-3.3452304	0.15868726	-3.2329371	0.00183173	0.00638915	-1.3200735	SOX10	assignmentsChromaffin_cells.Chrom_SOX2_statusHigh
assignmentsChromaffin_cells.Chrom_SOX2_statusHigh.BUB3	0.77578028	5.69556874	3.43774033	0.00096622	0.00373584	-1.3216112	BUB3	assignmentsChromaffin_cells.Chrom_SOX2_statusHigh
assignmentsChromaffin_cells.Chrom_SOX2_statusHigh.TIFA	1.2764909	2.05195425	3.35208867	0.00126608	0.00469056	-1.3217934	TIFA	assignmentsChromaffin_cells.Chrom_SOX2_statusHigh
assignmentsChromaffin_cells.Chrom_SOX2_statusHigh.ALKBH4	0.91997232	2.65009259	3.37969711	0.00116097	0.00435853	-1.3224725	ALKBH4	assignmentsChromaffin_cells.Chrom_SOX2_statusHigh
assignmentsChromaffin_cells.Chrom_SOX2_statusHigh.COL11A2	-1.8428787	1.41623844	-3.3029797	0.00147554	0.00530241	-1.3234429	COL11A2	assignmentsChromaffin_cells.Chrom_SOX2_statusHigh
assignmentsChromaffin_cells.Chrom_SOX2_statusHigh.GCDH	0.76432847	3.870973	3.40150069	0.00108383	0.00412044	-1.3239008	GCDH	assignmentsChromaffin_cells.Chrom_SOX2_statusHigh
assignmentsChromaffin_cells.Chrom_SOX2_statusHigh.WDFY3-AS1	-1.8973304	1.20129734	-3.2968484	0.00150387	0.00539093	-1.3243703	WDFY3-AS1	assignmentsChromaffin_cells.Chrom_SOX2_statusHigh
assignmentsChromaffin_cells.Chrom_SOX2_statusHigh.MROH7	-1.2860106	4.03927271	-3.4011226	0.00108513	0.00412044	-1.3246646	MROH7	assignmentsChromaffin_cells.Chrom_SOX2_statusHigh
assignmentsChromaffin_cells.Chrom_SOX2_statusHigh.TECTA	-0.9469621	2.79144013	-3.3797612	0.00116074	0.00435853	-1.3254056	TECTA	assignmentsChromaffin_cells.Chrom_SOX2_statusHigh
assignmentsChromaffin_cells.Chrom_SOX2_statusHigh.WDFY2	-1.1232453	8.02447208	-3.4619598	0.00089466	0.00349795	-1.3267947	WDFY2	assignmentsChromaffin_cells.Chrom_SOX2_statusHigh
assignmentsChromaffin_cells.Chrom_SOX2_statusHigh.PPP1R18	1.24231136	3.97200683	3.37453039	0.00117999	0.00441961	-1.3312335	PPP1R18	assignmentsChromaffin_cells.Chrom_SOX2_statusHigh
assignmentsChromaffin_cells.Chrom_SOX2_statusHigh.AFG3L2	-0.6160278	5.80447295	-3.4268979	0.00100008	0.00384728	-1.3318999	AFG3L2	assignmentsChromaffin_cells.Chrom_SOX2_statusHigh
assignmentsChromaffin_cells.Chrom_SOX2_statusHigh.LINC01362	1.44980625	0.85636452	3.32305801	0.00138624	0.00504597	-1.3328131	LINC01362	assignmentsChromaffin_cells.Chrom_SOX2_statusHigh
assignmentsChromaffin_cells.Chrom_SOX2_statusHigh.WDCP	0.86307766	2.4714259	3.36258439	0.00122511	0.00455983	-1.3332009	WDCP	assignmentsChromaffin_cells.Chrom_SOX2_statusHigh

assignmentsChromaffin_cells.Chrom_SOX2_statusHigh.MRPL45	0.8999761	3.85476367	3.40329891	0.00107769	0.00410082	-1.3332191	MRPL45	assignmentsChromaffin_cells.Chrom_SOX2_statusHigh
assignmentsChromaffin_cells.Chrom_SOX2_statusHigh.TLN2	-1.4036504	6.29298566	-3.4381789	0.00096539	0.00373352	-1.3335798	TLN2	assignmentsChromaffin_cells.Chrom_SOX2_statusHigh
assignmentsChromaffin_cells.Chrom_SOX2_statusHigh.PSME3	0.73855913	4.20590144	3.4012445	0.00108471	0.00412044	-1.3356499	PSME3	assignmentsChromaffin_cells.Chrom_SOX2_statusHigh
assignmentsChromaffin_cells.Chrom_SOX2_statusHigh.ZBED5	-0.638489	5.42149252	-3.4150094	0.00103849	0.00397978	-1.3361857	ZBED5	assignmentsChromaffin_cells.Chrom_SOX2_statusHigh
assignmentsChromaffin_cells.Chrom_SOX2_statusHigh.LYG2	-1.8369144	0.77847097	-3.2774607	0.00159684	0.00567109	-1.338287	LYG2	assignmentsChromaffin_cells.Chrom_SOX2_statusHigh
assignmentsChromaffin_cells.Chrom_SOX2_statusHigh.ZFPL1	1.10606091	3.2260137	3.38524921	0.00114085	0.00429995	-1.3411368	ZFPL1	assignmentsChromaffin_cells.Chrom_SOX2_statusHigh
assignmentsChromaffin_cells.Chrom_SOX2_statusHigh.MXRA8	1.9174933	2.09946217	3.31450067	0.00142412	0.00515309	-1.3428813	MXRA8	assignmentsChromaffin_cells.Chrom_SOX2_statusHigh
assignmentsChromaffin_cells.Chrom_SOX2_statusHigh.MKX	-3.7520848	2.33178813	-3.3187843	0.0014064	0.00510081	-1.3439658	MKX	assignmentsChromaffin_cells.Chrom_SOX2_statusHigh
assignmentsChromaffin_cells.Chrom_SOX2_statusHigh.IP6K3	2.89362625	0.02274834	3.26891009	0.00164126	0.00580302	-1.3458222	IP6K3	assignmentsChromaffin_cells.Chrom_SOX2_statusHigh
assignmentsChromaffin_cells.Chrom_SOX2_statusHigh.DMAP1	0.71433162	3.85339108	3.38962656	0.00112522	0.00424719	-1.3461181	DMAP1	assignmentsChromaffin_cells.Chrom_SOX2_statusHigh
assignmentsChromaffin_cells.Chrom_SOX2_statusHigh.KIAA0319	-1.4735172	3.96781194	-3.4017682	0.00108341	0.00412041	-1.3472772	KIAA0319	assignmentsChromaffin_cells.Chrom_SOX2_statusHigh
assignmentsChromaffin_cells.Chrom_SOX2_statusHigh.NRSN2-AS1	-0.9587093	4.12599511	-3.3930636	0.00111309	0.00420906	-1.3483717	NRSN2-AS1	assignmentsChromaffin_cells.Chrom_SOX2_statusHigh
assignmentsChromaffin_cells.Chrom_SOX2_statusHigh.BACE1	-0.7167464	4.29699532	-3.3912486	0.00111948	0.0042295	-1.3491628	BACE1	assignmentsChromaffin_cells.Chrom_SOX2_statusHigh
assignmentsChromaffin_cells.Chrom_SOX2_statusHigh.PHC3	-0.7837962	7.20676929	-3.4367035	0.00096941	0.00374438	-1.3511198	PHC3	assignmentsChromaffin_cells.Chrom_SOX2_statusHigh
assignmentsChromaffin_cells.Chrom_SOX2_statusHigh.BRD1	-1.1464686	5.84826571	-3.4082675	0.00106089	0.00405013	-1.351516	BRD1	assignmentsChromaffin_cells.Chrom_SOX2_statusHigh
assignmentsChromaffin_cells.Chrom_SOX2_statusHigh.FGD5-AS1	1.00183171	4.85793709	3.41001395	0.00105505	0.00402878	-1.352751	FGD5-AS1	assignmentsChromaffin_cells.Chrom_SOX2_statusHigh
assignmentsChromaffin_cells.Chrom_SOX2_statusHigh.MATN2	-1.8188189	3.60962184	-3.3515531	0.00126883	0.00469964	-1.3534144	MATN2	assignmentsChromaffin_cells.Chrom_SOX2_statusHigh
assignmentsChromaffin_cells.Chrom_SOX2_statusHigh.RPH3A	2.06999302	0.50436936	3.31380955	0.00142739	0.00516295	-1.353598	RPH3A	assignmentsChromaffin_cells.Chrom_SOX2_statusHigh
assignmentsChromaffin_cells.Chrom_SOX2_statusHigh.LCA5	0.71916701	4.32219861	3.40622148	0.00106778	0.00407158	-1.3539045	LCA5	assignmentsChromaffin_cells.Chrom_SOX2_statusHigh
assignmentsChromaffin_cells.Chrom_SOX2_statusHigh.SCN8A	-1.3778572	5.61518905	-3.4357179	0.00097297	0.00375379	-1.35456	SCN8A	assignmentsChromaffin_cells.Chrom_SOX2_statusHigh
assignmentsChromaffin_cells.Chrom_SOX2_statusHigh.LRRCC1	1.32312476	3.24513147	3.36970998	0.00119801	0.00447453	-1.3580087	LRRCC1	assignmentsChromaffin_cells.Chrom_SOX2_statusHigh
assignmentsChromaffin_cells.Chrom_SOX2_statusHigh.MGRN1	-0.5799114	5.65954097	-3.4110562	0.00105157	0.00402125	-1.3595619	MGRN1	assignmentsChromaffin_cells.Chrom_SOX2_statusHigh
assignmentsChromaffin_cells.Chrom_SOX2_statusHigh.PTGDS	2.76304749	0.3014375	3.22575694	0.00187187	0.0065037	-1.3597732	PTGDS	assignmentsChromaffin_cells.Chrom_SOX2_statusHigh
assignmentsChromaffin_cells.Chrom_SOX2_statusHigh.TKT	1.14448874	5.41444346	3.4220783	0.00101549	0.00389908	-1.3612048	TKT	assignmentsChromaffin_cells.Chrom_SOX2_statusHigh
assignmentsChromaffin_cells.Chrom_SOX2_statusHigh.C8orf88	1.0972063	3.08706919	3.38601294	0.00113811	0.0042918	-1.3625408	C8orf88	assignmentsChromaffin_cells.Chrom_SOX2_statusHigh
assignmentsChromaffin_cells.Chrom_SOX2_statusHigh.HMGA1	1.35578759	2.76257639	3.37274072	0.00118665	0.0044404	-1.3626626	HMGA1	assignmentsChromaffin_cells.Chrom_SOX2_statusHigh
assignmentsChromaffin_cells.Chrom_SOX2_statusHigh.ZDHHC15	-0.9666814	4.19594553	-3.3940298	0.0011097	0.00419849	-1.3633818	ZDHHC15	assignmentsChromaffin_cells.Chrom_SOX2_statusHigh
assignmentsChromaffin_cells.Chrom_SOX2_statusHigh.DNAJB1	3.16393033	5.80455823	3.42843356	0.00099647	0.00383523	-1.3637207	DNAJB1	assignmentsChromaffin_cells.Chrom_SOX2_statusHigh
assignmentsChromaffin_cells.Chrom_SOX2_statusHigh.BAMBI	2.62063414	0.67637128	3.23507911	0.00181967	0.00635123	-1.3643441	BAMBI	assignmentsChromaffin_cells.Chrom_SOX2_statusHigh
assignmentsChromaffin_cells.Chrom_SOX2_statusHigh.LMNTD1	1.9014206	0.52467263	3.31446506	0.0014243	0.00515309	-1.3650222	LMNTD1	assignmentsChromaffin_cells.Chrom_SOX2_statusHigh
assignmentsChromaffin_cells.Chrom_SOX2_statusHigh.FOXP1-AS1	-2.3263475	0.81068589	-3.2303542	0.00184572	0.00642677	-1.3680734	FOXP1-AS1	assignmentsChromaffin_cells.Chrom_SOX2_statusHigh
assignmentsChromaffin_cells.Chrom_SOX2_statusHigh.FKTN	-0.9186933	5.66540857	-3.4133231	0.00104405	0.00399822	-1.3728403	FKTN	assignmentsChromaffin_cells.Chrom_SOX2_statusHigh
assignmentsChromaffin_cells.Chrom_SOX2_statusHigh.ALDH1A3	2.77799795	1.47959564	3.27780381	0.00159687	0.00567109	-1.3736277	ALDH1A3	assignmentsChromaffin_cells.Chrom_SOX2_statusHigh
assignmentsChromaffin_cells.Chrom_SOX2_statusHigh.CHMP4C	2.54901205	-0.3967193	3.22676843	0.00186609	0.00648782	-1.3759517	CHMP4C	assignmentsChromaffin_cells.Chrom_SOX2_statusHigh
assignmentsChromaffin_cells.Chrom_SOX2_statusHigh.HEXIM1	1.6817999	3.8386552	3.38526486	0.00114183	0.00430176	-1.3762976	HEXIM1	assignmentsChromaffin_cells.Chrom_SOX2_statusHigh
assignmentsChromaffin_cells.Chrom_SOX2_statusHigh.SNAI3	-2.6549108	1.08275334	-3.230525	0.00184526	0.00642657	-1.3775199	SNAI3	assignmentsChromaffin_cells.Chrom_SOX2_statusHigh
assignmentsChromaffin_cells.Chrom_SOX2_statusHigh.NKX6-1	3.00183553	-0.2731486	3.25060121	0.00173642	0.00610048	-1.3777577	NKX6-1	assignmentsChromaffin_cells.Chrom_SOX2_statusHigh
assignmentsChromaffin_cells.Chrom_SOX2_statusHigh.DPY30	1.05116603	4.49155644	3.39835697	0.00109465	0.00415231	-1.3835692	DPY30	assignmentsChromaffin_cells.Chrom_SOX2_statusHigh
assignmentsChromaffin_cells.Chrom_SOX2_statusHigh.LTB4R	-1.5230958	3.34956172	-3.3427126	0.00130389	0.00480508	-1.3858422	LTB4R	assignmentsChromaffin_cells.Chrom_SOX2_statusHigh
assignmentsChromaffin_cells.Chrom_SOX2_statusHigh.LINC02520	2.21747278	-0.8178915	3.21545739	0.00193171	0.00667977	-1.3887145	LINC02520	assignmentsChromaffin_cells.Chrom_SOX2_statusHigh
assignmentsChromaffin_cells.Chrom_SOX2_statusHigh.MCM5	1.14406884	2.34581559	3.31454134	0.00142348	0.00515309	-1.3889765	MCM5	assignmentsChromaffin_cells.Chrom_SOX2_statusHigh
assignmentsChromaffin_cells.Chrom_SOX2_statusHigh.ZNF322	0.69455457	4.86969571	3.39948247	0.00109076	0.00413856	-1.3904582	ZNF322	assignmentsChromaffin_cells.Chrom_SOX2_statusHigh
assignmentsChromaffin_cells.Chrom_SOX2_statusHigh.COMMD2	0.8857554	4.47188604	3.384271	0.00114437	0.00430832	-1.390482	COMMD2	assignmentsChromaffin_cells.Chrom_SOX2_statusHigh
assignmentsChromaffin_cells.Chrom_SOX2_statusHigh.ARFIP2	0.97567661	3.5026293	3.36920872	0.0011999	0.0044795	-1.3908687	ARFIP2	assignmentsChromaffin_cells.Chrom_SOX2_statusHigh
assignmentsChromaffin_cells.Chrom_SOX2_statusHigh.ALG5	0.59384079	5.13821704	3.40587999	0.00106893	0.00407466	-1.3914287	ALG5	assignmentsChromaffin_cells.Chrom_SOX2_statusHigh
assignmentsChromaffin_cells.Chrom_SOX2_statusHigh.GALNS	-0.8997297	3.91870704	-3.3601547	0.00123448	0.00458832	-1.3961716	GALNS	assignmentsChromaffin_cells.Chrom_SOX2_statusHigh
assignmentsChromaffin_cells.Chrom_SOX2_statusHigh.UFD1	0.89725674	5.25745774	3.40571204	0.0010695	0.00407524	-1.3966451	UFD1	assignmentsChromaffin_cells.Chrom_SOX2_statusHigh
assignmentsChromaffin_cells.Chrom_SOX2_statusHigh.FAS	-1.4766073	4.32532103	-3.3412519	0.00130974	0.00482224	-1.3974369	FAS	assignmentsChromaffin_cells.Chrom_SOX2_statusHigh
assignmentsChromaffin_cells.Chrom_SOX2_statusHigh.TOB1-AS1	1.16599136	2.46924389	3.35367381	0.00125981	0.0046752	-1.398021	TOB1-AS1	assignmentsChromaffin_cells.Chrom_SOX2_statusHigh
assignmentsChromaffin_cells.Chrom_SOX2_statusHigh.SPATC1L	1.56623135	2.08302179	3.35226364	0.00126582	0.00469056	-1.4025432	SPATC1L	assignmentsChromaffin_cells.Chrom_SOX2_statusHigh
assignmentsChromaffin_cells.Chrom_SOX2_statusHigh.TSEN2	-0.9007598	4.87555361	-3.3814143	0.00115471	0.00433809	-1.4062144	TSEN2	assignmentsChromaffin_cells.Chrom_SOX2_statusHigh
assignmentsChromaffin_cells.Chrom_SOX2_statusHigh.SF3A3	0.68438976	4.73106036	3.37474147	0.00117921	0.0044177	-1.4094004	SF3A3	assignmentsChromaffin_cells.Chrom_SOX2_statusHigh
assignmentsChromaffin_cells.Chrom_SOX2_statusHigh.CYP39A1	1.89462018	1.88525654	3.32472289	0.00137978	0.00502817	-1.4119373	CYP39A1	assignmentsChromaffin_cells.Chrom_SOX2_statusHigh
assignmentsChromaffin_cells.Chrom_SOX2_statusHigh.ALG1L9P	-1.4085452	3.40966714	-3.3461339	0.0012899	0.00475902	-1.4154993	ALG1L9P	assignmentsChromaffin_cells.Chrom_SOX2_statusHigh
assignmentsChromaffin_cells.Chrom_SOX2_statusHigh.SNHG1	1.21458764	4.42305015	3.3837834	0.00114613	0.00431394	-1.4164439	SNHG1	assignmentsChromaffin_cells.Chrom_SOX2_statusHigh
assignmentsChromaffin_cells.Chrom_SOX2_statusHigh.RBP2	-2.0160548	0.5248896	-3.2492244	0.00174195	0.00611857	-1.4167595	RBP2	assignmentsChromaffin_cells.Chrom_SOX2_statusHigh
assignmentsChromaffin_cells.Chrom_SOX2_statusHigh.TOR2A	0.94006381	1.97080538	3.3194408	0.00140194	0.00509042	-1.4207116	TOR2A	assignmentsChromaffin_cells.Chrom_SOX2_statusHigh
assignmentsChromaffin_cells.Chrom_SOX2_statusHigh.SLC4A5	-1.1242106	4.65958398	-3.3737195	0.00118301	0.00442882	-1.4209563	SLC4A5	assignmentsChromaffin_cells.Chrom_SOX2_statusHigh
assignmentsChromaffin_cells.Chrom_SOX2_statusHigh.BBS12	0.82679641	1.44224262	3.31900707	0.00140384	0.00509597	-1.4209727	BBS12	assignmentsChromaffin_cells.Chrom_SOX2_statusHigh
assignmentsChromaffin_cells.Chrom_SOX2_statusHigh.BCL2L14	-2.0512323	3.6330998	-3.3401467	0.00131579	0.00483783	-1.4209836	BCL2L14	assignmentsChromaffin_cells.Chrom_SOX2_statusHigh
assignmentsChromaffin_cells.Chrom_SOX2_statusHigh.FLOT1	0.84567717	5.96552203	3.40138916	0.00108421	0.00412044	-1.4212117	FLOT1	assignmentsChromaffin_cells.Chrom_SOX2_statusHigh
assignmentsChromaffin_cells.Chrom_SOX2_statusHigh.UBAP2L	-0.5107863	6.35276863	-3.4045711	0.00107337	0.00408802	-1.4231407	UBAP2L	assignmentsChromaffin_cells.Chrom_SOX2_statusHigh
assignmentsChromaffin_cells.Chrom_SOX2_statusHigh.RBM25	-0.8202657	8.22766295	-3.4294319	0.00099207	0.00382012	-1.4252417	RBM25	assignmentsChromaffin_cells.Chrom_SOX2_statusHigh
assignmentsChromaffin_cells.Chrom_SOX2_statusHigh.HSD17B1	1.27646087	1.02461695	3.30864463	0.00144981	0.0052287	-1.4265403	HSD17B1	assignmentsChromaffin_cells.Chrom_SOX2_statusHigh

assignmentsChromaffin_cells.Chrom_SOX2_statusHigh.CARMN	-2.1290289	4.45013438	-3.3374385	0.00132696	0.00487059	-1.4266198	CARMN	assignmentsChromaffin_cells.Chrom_SOX2_statusHigh
assignmentsChromaffin_cells.Chrom_SOX2_statusHigh.ADRA2A	2.41999988	0.05651197	3.22916842	0.00185298	0.00644925	-1.4270996	ADRA2A	assignmentsChromaffin_cells.Chrom_SOX2_statusHigh
assignmentsChromaffin_cells.Chrom_SOX2_statusHigh.ZNF329	-1.2285755	3.98006977	-3.3446093	0.00129607	0.00477748	-1.4280021	ZNF329	assignmentsChromaffin_cells.Chrom_SOX2_statusHigh
assignmentsChromaffin_cells.Chrom_SOX2_statusHigh.ELF4	1.99262023	2.62978447	3.26693374	0.00165003	0.00582376	-1.4293225	ELF4	assignmentsChromaffin_cells.Chrom_SOX2_statusHigh
assignmentsChromaffin_cells.Chrom_SOX2_statusHigh.RAB40A	-1.2771621	1.9555879	-3.3019744	0.00148015	0.0053154	-1.429427	RAB40A	assignmentsChromaffin_cells.Chrom_SOX2_statusHigh
assignmentsChromaffin_cells.Chrom_SOX2_statusHigh.CLCN7	-0.780651	5.19390174	-3.3818193	0.00115324	0.00433357	-1.4296199	CLCN7	assignmentsChromaffin_cells.Chrom_SOX2_statusHigh
assignmentsChromaffin_cells.Chrom_SOX2_statusHigh.SCFD1	-0.6060321	7.52720576	-3.4216552	0.00101685	0.00390337	-1.4304196	SCFD1	assignmentsChromaffin_cells.Chrom_SOX2_statusHigh
assignmentsChromaffin_cells.Chrom_SOX2_statusHigh.RTN4	0.83595471	8.36017907	3.43721395	0.00096784	0.00374064	-1.4305771	RTN4	assignmentsChromaffin_cells.Chrom_SOX2_statusHigh
assignmentsChromaffin_cells.Chrom_SOX2_statusHigh.HOXD8	1.79044687	2.55820886	3.33936945	0.00131837	0.00484512	-1.4306689	HOXD8	assignmentsChromaffin_cells.Chrom_SOX2_statusHigh
assignmentsChromaffin_cells.Chrom_SOX2_statusHigh.SLF2	-0.8499416	6.52954576	-3.4027994	0.00107939	0.0041061	-1.4315667	SLF2	assignmentsChromaffin_cells.Chrom_SOX2_statusHigh
assignmentsChromaffin_cells.Chrom_SOX2_statusHigh.UGDH-AS1	-0.7583135	4.47274904	-3.3751351	0.00117775	0.00441327	-1.4328709	UGDH-AS1	assignmentsChromaffin_cells.Chrom_SOX2_statusHigh
assignmentsChromaffin_cells.Chrom_SOX2_statusHigh.KRT18	2.83100782	-0.068112	3.18867704	0.0020958	0.00715277	-1.433959	KRT18	assignmentsChromaffin_cells.Chrom_SOX2_statusHigh
assignmentsChromaffin_cells.Chrom_SOX2_statusHigh.DIRC3	2.06209109	5.11774886	3.3281285	0.00136606	0.00499064	-1.4343242	DIRC3	assignmentsChromaffin_cells.Chrom_SOX2_statusHigh
assignmentsChromaffin_cells.Chrom_SOX2_statusHigh.PLPPR4	-2.3966404	2.67679491	-3.3250583	0.00137919	0.00502716	-1.4349436	PLPPR4	assignmentsChromaffin_cells.Chrom_SOX2_statusHigh
assignmentsChromaffin_cells.Chrom_SOX2_statusHigh.ST7L	-0.8015323	5.70084839	-3.3787722	0.00116436	0.00436817	-1.4355576	ST7L	assignmentsChromaffin_cells.Chrom_SOX2_statusHigh
assignmentsChromaffin_cells.Chrom_SOX2_statusHigh.STX19	-2.1041022	0.73822098	-3.2224343	0.00189098	0.00655873	-1.4360334	STX19	assignmentsChromaffin_cells.Chrom_SOX2_statusHigh
assignmentsChromaffin_cells.Chrom_SOX2_statusHigh.TRPV1	-1.2178254	2.90172082	-3.3121116	0.00143427	0.00518433	-1.4367016	TRPV1	assignmentsChromaffin_cells.Chrom_SOX2_statusHigh
assignmentsChromaffin_cells.Chrom_SOX2_statusHigh.ZNF207	-0.4956972	7.76249999	-3.4189847	0.0010255	0.00393279	-1.4442747	ZNF207	assignmentsChromaffin_cells.Chrom_SOX2_statusHigh
assignmentsChromaffin_cells.Chrom_SOX2_statusHigh.GCNT7	-1.9957942	1.05026966	-3.2245714	0.00187867	0.00652307	-1.4468045	GCNT7	assignmentsChromaffin_cells.Chrom_SOX2_statusHigh
assignmentsChromaffin_cells.Chrom_SOX2_statusHigh.GABRB1	-4.7774708	2.02500324	-3.2313589	0.00184195	0.00641782	-1.4470842	GABRB1	assignmentsChromaffin_cells.Chrom_SOX2_statusHigh
assignmentsChromaffin_cells.Chrom_SOX2_statusHigh.PPIL4	0.75365091	6.12624933	3.39789855	0.00109623	0.00415734	-1.4471669	PPIL4	assignmentsChromaffin_cells.Chrom_SOX2_statusHigh
assignmentsChromaffin_cells.Chrom_SOX2_statusHigh.MAD1L1	-0.8651123	5.9144254	-3.3717879	0.00119021	0.00445163	-1.4479828	MAD1L1	assignmentsChromaffin_cells.Chrom_SOX2_statusHigh
assignmentsChromaffin_cells.Chrom_SOX2_statusHigh.MTHFD1L	-1.1460116	7.05922837	-3.4012998	0.00108452	0.00412044	-1.4503105	MTHFD1L	assignmentsChromaffin_cells.Chrom_SOX2_statusHigh
assignmentsChromaffin_cells.Chrom_SOX2_statusHigh.SREBF1	2.20037036	3.66898878	3.30485375	0.00146861	0.00528225	-1.451771	SREBF1	assignmentsChromaffin_cells.Chrom_SOX2_statusHigh
assignmentsChromaffin_cells.Chrom_SOX2_statusHigh.COX16	0.75117848	5.18367433	3.3879192	0.00113129	0.0042691	-1.4529667	COX16	assignmentsChromaffin_cells.Chrom_SOX2_statusHigh
assignmentsChromaffin_cells.Chrom_SOX2_statusHigh.KRT19	3.93291636	-0.4196692	3.21544988	0.00193371	0.00668525	-1.4531337	KRT19	assignmentsChromaffin_cells.Chrom_SOX2_statusHigh
assignmentsChromaffin_cells.Chrom_SOX2_statusHigh.ZNF644	-0.7461438	7.5740532	-3.3998881	0.00108937	0.00413424	-1.4578137	ZNF644	assignmentsChromaffin_cells.Chrom_SOX2_statusHigh
assignmentsChromaffin_cells.Chrom_SOX2_statusHigh.ABRACL	1.82041175	2.94381642	3.30505086	0.00146676	0.00527798	-1.4579635	ABRACL	assignmentsChromaffin_cells.Chrom_SOX2_statusHigh
assignmentsChromaffin_cells.Chrom_SOX2_statusHigh.HSPA14	1.09069652	3.24372336	3.34034942	0.00131344	0.00483211	-1.4583211	HSPA14	assignmentsChromaffin_cells.Chrom_SOX2_statusHigh
assignmentsChromaffin_cells.Chrom_SOX2_statusHigh.HS3ST3A1	-4.1822236	1.77529075	-3.1817245	0.00214258	0.00727869	-1.4602392	HS3ST3A1	assignmentsChromaffin_cells.Chrom_SOX2_statusHigh
assignmentsChromaffin_cells.Chrom_SOX2_statusHigh.CCDC80	1.77355506	2.63825928	3.30994245	0.00144465	0.00521596	-1.4604329	CCDC80	assignmentsChromaffin_cells.Chrom_SOX2_statusHigh
assignmentsChromaffin_cells.Chrom_SOX2_statusHigh.C3orf49	-2.4943389	0.4889055	-3.1964905	0.00204663	0.00702114	-1.4620659	C3orf49	assignmentsChromaffin_cells.Chrom_SOX2_statusHigh
assignmentsChromaffin_cells.Chrom_SOX2_statusHigh.AQP2	4.32993796	-0.575452	3.19982711	0.00202798	0.00696759	-1.4667744	AQP2	assignmentsChromaffin_cells.Chrom_SOX2_statusHigh
assignmentsChromaffin_cells.Chrom_SOX2_statusHigh.CYP3A5	-1.6758557	1.56596163	-3.2701657	0.0016332	0.0057809	-1.4675521	CYP3A5	assignmentsChromaffin_cells.Chrom_SOX2_statusHigh
assignmentsChromaffin_cells.Chrom_SOX2_statusHigh.SCML1	0.80273176	5.75170887	3.38303896	0.00114882	0.00432104	-1.4713139	SCML1	assignmentsChromaffin_cells.Chrom_SOX2_statusHigh
assignmentsChromaffin_cells.Chrom_SOX2_statusHigh.RGS12	-0.758752	5.68281059	-3.3667813	0.00120908	0.00451272	-1.4752284	RGS12	assignmentsChromaffin_cells.Chrom_SOX2_statusHigh
assignmentsChromaffin_cells.Chrom_SOX2_statusHigh.EXOG	-0.7671933	5.17314653	-3.365197	0.00121511	0.00452856	-1.4757599	EXOG	assignmentsChromaffin_cells.Chrom_SOX2_statusHigh
assignmentsChromaffin_cells.Chrom_SOX2_statusHigh.PPP1R16B	-1.4816302	5.33676781	-3.3477286	0.00128401	0.00474275	-1.4759906	PPP1R16B	assignmentsChromaffin_cells.Chrom_SOX2_statusHigh
assignmentsChromaffin_cells.Chrom_SOX2_statusHigh.SMCR5	-2.5949482	1.02776324	-3.213898	0.00194175	0.0067058	-1.4776483	SMCR5	assignmentsChromaffin_cells.Chrom_SOX2_statusHigh
assignmentsChromaffin_cells.Chrom_SOX2_statusHigh.EPOR	1.54000028	3.54150366	3.34849614	0.00128103	0.00473389	-1.4793372	EPOR	assignmentsChromaffin_cells.Chrom_SOX2_statusHigh
assignmentsChromaffin_cells.Chrom_SOX2_statusHigh.ARFGEF1	-0.5543736	7.66908693	-3.401404	0.00108416	0.00412044	-1.4843472	ARFGEF1	assignmentsChromaffin_cells.Chrom_SOX2_statusHigh
assignmentsChromaffin_cells.Chrom_SOX2_statusHigh.KCNJ9	-3.1840163	-0.3479049	-3.1688603	0.00222605	0.00750661	-1.4844775	KCNJ9	assignmentsChromaffin_cells.Chrom_SOX2_statusHigh
assignmentsChromaffin_cells.Chrom_SOX2_statusHigh.NTNG2	-1.5483851	4.73244582	-3.3654055	0.00121529	0.00452856	-1.4845941	NTNG2	assignmentsChromaffin_cells.Chrom_SOX2_statusHigh
assignmentsChromaffin_cells.Chrom_SOX2_statusHigh.GAPLINC	1.69400783	0.03509496	3.17032388	0.00221569	0.00747709	-1.4850213	GAPLINC	assignmentsChromaffin_cells.Chrom_SOX2_statusHigh
assignmentsChromaffin_cells.Chrom_SOX2_statusHigh.FAM186A	-1.9756963	0.55170967	-3.2065859	0.00198469	0.00683642	-1.4853015	FAM186A	assignmentsChromaffin_cells.Chrom_SOX2_statusHigh
assignmentsChromaffin_cells.Chrom_SOX2_statusHigh.EEFSEC	-0.8353425	5.90887982	-3.3660476	0.00121187	0.00452103	-1.4858962	EEFSEC	assignmentsChromaffin_cells.Chrom_SOX2_statusHigh
assignmentsChromaffin_cells.Chrom_SOX2_statusHigh.PITPNC1	-2.2360659	7.90500271	-3.3339674	0.00134142	0.00491478	-1.4863755	PITPNC1	assignmentsChromaffin_cells.Chrom_SOX2_statusHigh
assignmentsChromaffin_cells.Chrom_SOX2_statusHigh.KLHL8	-0.7635351	5.29024056	-3.3641505	0.0012191	0.00454064	-1.4864321	KLHL8	assignmentsChromaffin_cells.Chrom_SOX2_statusHigh
assignmentsChromaffin_cells.Chrom_SOX2_statusHigh.CTNND1	-0.7733422	7.6293268	-3.3973615	0.00109809	0.00416243	-1.4926083	CTNND1	assignmentsChromaffin_cells.Chrom_SOX2_statusHigh
assignmentsChromaffin_cells.Chrom_SOX2_statusHigh.TCEAL2	1.96881205	3.51907004	3.36563494	0.00121487	0.00452856	-1.4945292	TCEAL2	assignmentsChromaffin_cells.Chrom_SOX2_statusHigh
assignmentsChromaffin_cells.Chrom_SOX2_statusHigh.MYO5A	-0.8721893	7.48631598	-3.3856658	0.00113936	0.00429548	-1.4961166	MYO5A	assignmentsChromaffin_cells.Chrom_SOX2_statusHigh
assignmentsChromaffin_cells.Chrom_SOX2_statusHigh.BRI3	1.20774155	5.97111861	3.3852887	0.00114108	0.00429995	-1.497319	BRI3	assignmentsChromaffin_cells.Chrom_SOX2_statusHigh
assignmentsChromaffin_cells.Chrom_SOX2_statusHigh.TENT5C	2.22810565	2.00770802	3.23752372	0.00180641	0.00631856	-1.498836	TENT5C	assignmentsChromaffin_cells.Chrom_SOX2_statusHigh
assignmentsChromaffin_cells.Chrom_SOX2_statusHigh.EVI5L	-0.8006658	3.82645444	-3.3370469	0.00132706	0.00487059	-1.5002397	EVI5L	assignmentsChromaffin_cells.Chrom_SOX2_statusHigh
assignmentsChromaffin_cells.Chrom_SOX2_statusHigh.ULBP3	1.7482176	-0.5368478	3.16698334	0.00223819	0.00753962	-1.5024697	ULBP3	assignmentsChromaffin_cells.Chrom_SOX2_statusHigh
assignmentsChromaffin_cells.Chrom_SOX2_statusHigh.METTL15	-0.7778399	7.04593508	-3.3832396	0.0011481	0.00432031	-1.5026928	METTL15	assignmentsChromaffin_cells.Chrom_SOX2_statusHigh
assignmentsChromaffin_cells.Chrom_SOX2_statusHigh.LONP2	-0.7108189	7.62643012	-3.3967034	0.00110038	0.00416912	-1.5067317	LONP2	assignmentsChromaffin_cells.Chrom_SOX2_statusHigh
assignmentsChromaffin_cells.Chrom_SOX2_statusHigh.ZNF404	-1.1654346	2.14965442	-3.2999219	0.0014896	0.00534457	-1.5078728	ZNF404	assignmentsChromaffin_cells.Chrom_SOX2_statusHigh
assignmentsChromaffin_cells.Chrom_SOX2_statusHigh.NOL10	-0.6455228	6.16431904	-3.3713554	0.00119183	0.00445561	-1.5090274	NOL10	assignmentsChromaffin_cells.Chrom_SOX2_statusHigh
assignmentsChromaffin_cells.Chrom_SOX2_statusHigh.NPIPA9	-1.1401398	1.71962499	-3.2692308	0.00163792	0.00579504	-1.509097	NPIPA9	assignmentsChromaffin_cells.Chrom_SOX2_statusHigh
assignmentsChromaffin_cells.Chrom_SOX2_statusHigh.FLYWCH1	-0.7587651	4.63680505	-3.3411469	0.00131017	0.00482271	-1.5107397	FLYWCH1	assignmentsChromaffin_cells.Chrom_SOX2_statusHigh
assignmentsChromaffin_cells.Chrom_SOX2_statusHigh.PANK1	-1.2636015	3.29939105	-3.3084125	0.00145085	0.0052313	-1.5162214	PANK1	assignmentsChromaffin_cells.Chrom_SOX2_statusHigh
assignmentsChromaffin_cells.Chrom_SOX2_statusHigh.CBX2	1.91398002	0.29467621	3.22595731	0.00187072	0.00650112	-1.5178854	CBX2	assignmentsChromaffin_cells.Chrom_SOX2_statusHigh
assignmentsChromaffin_cells.Chrom_SOX2_statusHigh.CCDC92	-0.6451402	5.76809734	-3.36171	0.00122847	0.00456888	-1.518805	CCDC92	assignmentsChromaffin_cells.Chrom_SOX2_statusHigh

assignmentsChromaffin_cells.Chrom_SOX2_statusHigh.TRIM52-AS1	1.11953359	2.36641313	3.31194936	0.001435	0.00518578	-1.5214281	TRIM52-AS1	assignmentsChromaffin_cells.Chrom_SOX2_statusHigh
assignmentsChromaffin_cells.Chrom_SOX2_statusHigh.IGF2BP1	2.88432733	-0.3185387	3.19529486	0.00205573	0.00704635	-1.523139	IGF2BP1	assignmentsChromaffin_cells.Chrom_SOX2_statusHigh
assignmentsChromaffin_cells.Chrom_SOX2_statusHigh.SLC4A8	-1.0122161	5.80661454	-3.3715668	0.00119104	0.00445369	-1.5233896	SLC4A8	assignmentsChromaffin_cells.Chrom_SOX2_statusHigh
assignmentsChromaffin_cells.Chrom_SOX2_statusHigh.WASF3	-1.7564097	3.76600996	-3.3097808	0.00144567	0.00521728	-1.5245593	WASF3	assignmentsChromaffin_cells.Chrom_SOX2_statusHigh
assignmentsChromaffin_cells.Chrom_SOX2_statusHigh.MOCOS	1.59765463	1.88865234	3.28734573	0.0015492	0.00552578	-1.5245672	MOCOS	assignmentsChromaffin_cells.Chrom_SOX2_statusHigh
assignmentsChromaffin_cells.Chrom_SOX2_statusHigh.WAC	-0.6799027	8.09025795	-3.3919697	0.00111694	0.00422089	-1.525935	WAC	assignmentsChromaffin_cells.Chrom_SOX2_statusHigh
assignmentsChromaffin_cells.Chrom_SOX2_statusHigh.NQO1	1.24400397	2.0129466	3.26810977	0.00164359	0.00580778	-1.5269931	NQO1	assignmentsChromaffin_cells.Chrom_SOX2_statusHigh
assignmentsChromaffin_cells.Chrom_SOX2_statusHigh.ZNF426	0.60722881	3.98394467	3.33591133	0.00133177	0.00488429	-1.5281552	ZNF426	assignmentsChromaffin_cells.Chrom_SOX2_statusHigh
assignmentsChromaffin_cells.Chrom_SOX2_statusHigh.SLC25A45	-1.2126636	4.19990769	-3.3231163	0.00138599	0.00504597	-1.5284942	SLC25A45	assignmentsChromaffin_cells.Chrom_SOX2_statusHigh
assignmentsChromaffin_cells.Chrom_SOX2_statusHigh.ZBTB49	-0.8337594	3.49295832	-3.309122	0.00144766	0.00522213	-1.5317676	ZBTB49	assignmentsChromaffin_cells.Chrom_SOX2_statusHigh
assignmentsChromaffin_cells.Chrom_SOX2_statusHigh.ZBTB46-AS1	-3.2588619	0.80546944	-3.1639041	0.00226042	0.00760589	-1.5320092	ZBTB46-AS1	assignmentsChromaffin_cells.Chrom_SOX2_statusHigh
assignmentsChromaffin_cells.Chrom_SOX2_statusHigh.PRLHR	3.62510806	2.56564762	3.33816298	0.00132396	0.00486232	-1.532153	PRLHR	assignmentsChromaffin_cells.Chrom_SOX2_statusHigh
assignmentsChromaffin_cells.Chrom_SOX2_statusHigh.GACAT2	1.40107065	0.79755576	3.27361297	0.00161592	0.00572986	-1.5334796	GACAT2	assignmentsChromaffin_cells.Chrom_SOX2_statusHigh
assignmentsChromaffin_cells.Chrom_SOX2_statusHigh.DNTTIP1	0.67259038	4.22210487	3.3326794	0.00134528	0.00492593	-1.5374744	DNTTIP1	assignmentsChromaffin_cells.Chrom_SOX2_statusHigh
assignmentsChromaffin_cells.Chrom_SOX2_statusHigh.MIAT	-1.6835153	7.04195664	-3.3914504	0.00112013	0.00423094	-1.5393401	MIAT	assignmentsChromaffin_cells.Chrom_SOX2_statusHigh
assignmentsChromaffin_cells.Chrom_SOX2_statusHigh.KIAA2026	-0.7486782	7.28187562	-3.3758996	0.00117492	0.00440473	-1.5401418	KIAA2026	assignmentsChromaffin_cells.Chrom_SOX2_statusHigh
assignmentsChromaffin_cells.Chrom_SOX2_statusHigh.STX2	-0.5863892	5.36309996	-3.3425111	0.0013046	0.0048066	-1.5428257	STX2	assignmentsChromaffin_cells.Chrom_SOX2_statusHigh
assignmentsChromaffin_cells.Chrom_SOX2_statusHigh.GSDMD	2.02803831	2.76405258	3.18114157	0.00214427	0.00728254	-1.5429727	GSDMD	assignmentsChromaffin_cells.Chrom_SOX2_statusHigh
assignmentsChromaffin_cells.Chrom_SOX2_statusHigh.PINK1	1.0427012	3.93052842	3.31829218	0.00140696	0.00510143	-1.5442813	PINK1	assignmentsChromaffin_cells.Chrom_SOX2_statusHigh
assignmentsChromaffin_cells.Chrom_SOX2_statusHigh.MTHFD2	1.13966792	5.09756396	3.35264452	0.00126388	0.00468782	-1.5446072	MTHFD2	assignmentsChromaffin_cells.Chrom_SOX2_statusHigh
assignmentsChromaffin_cells.Chrom_SOX2_statusHigh.SYT16	-2.4941003	1.64755734	-3.2346297	0.0018236	0.00636355	-1.5451747	SYT16	assignmentsChromaffin_cells.Chrom_SOX2_statusHigh
assignmentsChromaffin_cells.Chrom_SOX2_statusHigh.FSTL1	1.99209852	4.17704245	3.29141991	0.00153105	0.00547125	-1.5452687	FSTL1	assignmentsChromaffin_cells.Chrom_SOX2_statusHigh
assignmentsChromaffin_cells.Chrom_SOX2_statusHigh.SPDL1	0.7104545	3.10244719	3.30359452	0.00147272	0.00529586	-1.5483061	SPDL1	assignmentsChromaffin_cells.Chrom_SOX2_statusHigh
assignmentsChromaffin_cells.Chrom_SOX2_statusHigh.GSTZ1	0.94407067	2.58287105	3.29400161	0.00151719	0.0054314	-1.548322	GSTZ1	assignmentsChromaffin_cells.Chrom_SOX2_statusHigh
assignmentsChromaffin_cells.Chrom_SOX2_statusHigh.PICALM	-0.9746489	8.50787917	-3.3708859	0.00119359	0.00446011	-1.5509092	PICALM	assignmentsChromaffin_cells.Chrom_SOX2_statusHigh
assignmentsChromaffin_cells.Chrom_SOX2_statusHigh.MEF2B	-1.2082021	3.77208364	-3.2946481	0.00151415	0.00542174	-1.5530122	MEF2B	assignmentsChromaffin_cells.Chrom_SOX2_statusHigh
assignmentsChromaffin_cells.Chrom_SOX2_statusHigh.CCDC167	1.16137714	2.94806511	3.30672892	0.00145846	0.005254	-1.5530766	CCDC167	assignmentsChromaffin_cells.Chrom_SOX2_statusHigh
assignmentsChromaffin_cells.Chrom_SOX2_statusHigh.PTMA	2.07849996	7.93880008	3.38490914	0.00114345	0.00430586	-1.5531361	PTMA	assignmentsChromaffin_cells.Chrom_SOX2_statusHigh
assignmentsChromaffin_cells.Chrom_SOX2_statusHigh.TMEM69	0.74665195	2.86416265	3.29275632	0.00152305	0.00544876	-1.5540162	TMEM69	assignmentsChromaffin_cells.Chrom_SOX2_statusHigh
assignmentsChromaffin_cells.Chrom_SOX2_statusHigh.GGA2	-0.5501914	6.0985551	-3.3536159	0.00126004	0.0046752	-1.5542892	GGA2	assignmentsChromaffin_cells.Chrom_SOX2_statusHigh
assignmentsChromaffin_cells.Chrom_SOX2_statusHigh.FAM167B	1.76735275	0.3640643	3.16807914	0.00223079	0.00751941	-1.5554071	FAM167B	assignmentsChromaffin_cells.Chrom_SOX2_statusHigh
assignmentsChromaffin_cells.Chrom_SOX2_statusHigh.CCDC66	-0.6896811	6.38786435	-3.3599819	0.00123515	0.00458975	-1.5555779	CCDC66	assignmentsChromaffin_cells.Chrom_SOX2_statusHigh
assignmentsChromaffin_cells.Chrom_SOX2_statusHigh.PLEKHF2	0.84208272	4.06314884	3.3046867	0.00146774	0.0052803	-1.5563257	PLEKHF2	assignmentsChromaffin_cells.Chrom_SOX2_statusHigh
assignmentsChromaffin_cells.Chrom_SOX2_statusHigh.ZBTB1	-0.821283	6.43763573	-3.348069	0.00128211	0.00473682	-1.5564865	ZBTB1	assignmentsChromaffin_cells.Chrom_SOX2_statusHigh
assignmentsChromaffin_cells.Chrom_SOX2_statusHigh.SSTR1	2.90753807	-0.8794558	3.15251302	0.0023388	0.00782755	-1.5576039	SSTR1	assignmentsChromaffin_cells.Chrom_SOX2_statusHigh
assignmentsChromaffin_cells.Chrom_SOX2_statusHigh.CTNNA3	-2.5711591	5.20904067	-3.2866241	0.00155394	0.00553823	-1.562105	CTNNA3	assignmentsChromaffin_cells.Chrom_SOX2_statusHigh
assignmentsChromaffin_cells.Chrom_SOX2_statusHigh.RIPOR2	-1.9196487	6.30541354	-3.3411828	0.00131154	0.0048257	-1.5622257	RIPOR2	assignmentsChromaffin_cells.Chrom_SOX2_statusHigh
assignmentsChromaffin_cells.Chrom_SOX2_statusHigh.PDCD10	0.63328212	5.96764043	3.35056481	0.00127214	0.00470973	-1.5628055	PDCD10	assignmentsChromaffin_cells.Chrom_SOX2_statusHigh
assignmentsChromaffin_cells.Chrom_SOX2_statusHigh.KLHL2	-0.7575407	5.49688239	-3.3285232	0.00136283	0.00498207	-1.5640501	KLHL2	assignmentsChromaffin_cells.Chrom_SOX2_statusHigh
assignmentsChromaffin_cells.Chrom_SOX2_statusHigh.RPS6KA2	-1.4667509	6.46920781	-3.3448713	0.00129571	0.00477748	-1.5643046	RPS6KA2	assignmentsChromaffin_cells.Chrom_SOX2_statusHigh
assignmentsChromaffin_cells.Chrom_SOX2_statusHigh.APPL2	-0.7979708	6.43254065	-3.3489942	0.00127841	0.00472749	-1.5645617	APPL2	assignmentsChromaffin_cells.Chrom_SOX2_statusHigh
assignmentsChromaffin_cells.Chrom_SOX2_statusHigh.FREM3	-1.913684	0.32338272	-3.1821197	0.00213792	0.00727056	-1.5680017	FREM3	assignmentsChromaffin_cells.Chrom_SOX2_statusHigh
assignmentsChromaffin_cells.Chrom_SOX2_statusHigh.EPHA10	-1.2944994	1.13360693	-3.2548589	0.00171204	0.00602406	-1.5712517	EPHA10	assignmentsChromaffin_cells.Chrom_SOX2_statusHigh
assignmentsChromaffin_cells.Chrom_SOX2_statusHigh.CGA	4.49758704	-0.2055194	3.17828275	0.00216504	0.00733791	-1.571602	CGA	assignmentsChromaffin_cells.Chrom_SOX2_statusHigh
assignmentsChromaffin_cells.Chrom_SOX2_statusHigh.JAK3	1.69770634	2.91063353	3.17472364	0.00218638	0.00739927	-1.5746634	JAK3	assignmentsChromaffin_cells.Chrom_SOX2_statusHigh
assignmentsChromaffin_cells.Chrom_SOX2_statusHigh.CHMP3	0.90379432	5.98260232	3.3535791	0.00126019	0.0046752	-1.5763527	CHMP3	assignmentsChromaffin_cells.Chrom_SOX2_statusHigh
assignmentsChromaffin_cells.Chrom_SOX2_statusHigh.CXCL2	3.77552693	2.15818169	3.19176635	0.00207828	0.00711147	-1.577816	CXCL2	assignmentsChromaffin_cells.Chrom_SOX2_statusHigh
assignmentsChromaffin_cells.Chrom_SOX2_statusHigh.MOK	-1.0335026	5.19249909	-3.3408015	0.00131159	0.0048257	-1.5780719	MOK	assignmentsChromaffin_cells.Chrom_SOX2_statusHigh
assignmentsChromaffin_cells.Chrom_SOX2_statusHigh.DPAGT1	1.13950749	2.90394766	3.29197205	0.00152676	0.00545835	-1.5784038	DPAGT1	assignmentsChromaffin_cells.Chrom_SOX2_statusHigh
assignmentsChromaffin_cells.Chrom_SOX2_statusHigh.UBALD1	0.92974248	3.06842626	3.29652788	0.00150536	0.00539387	-1.5789946	UBALD1	assignmentsChromaffin_cells.Chrom_SOX2_statusHigh
assignmentsChromaffin_cells.Chrom_SOX2_statusHigh.CDKN2B	1.31146354	3.35943797	3.29884217	0.0014946	0.00536081	-1.5796301	CDKN2B	assignmentsChromaffin_cells.Chrom_SOX2_statusHigh
assignmentsChromaffin_cells.Chrom_SOX2_statusHigh.TNF	2.97843893	0.29007894	3.13173032	0.00248902	0.00826391	-1.5797657	TNF	assignmentsChromaffin_cells.Chrom_SOX2_statusHigh
assignmentsChromaffin_cells.Chrom_SOX2_statusHigh.NDUFB1	0.9490548	5.26891848	3.34629517	0.00128925	0.00475777	-1.5822849	NDUFB1	assignmentsChromaffin_cells.Chrom_SOX2_statusHigh
assignmentsChromaffin_cells.Chrom_SOX2_statusHigh.ZHX1	0.69256313	5.47049201	3.34499675	0.0012945	0.00477487	-1.5828266	ZHX1	assignmentsChromaffin_cells.Chrom_SOX2_statusHigh
assignmentsChromaffin_cells.Chrom_SOX2_statusHigh.KDM6A	-0.861054	7.78541566	-3.3651473	0.0012153	0.00452856	-1.5835097	KDM6A	assignmentsChromaffin_cells.Chrom_SOX2_statusHigh
assignmentsChromaffin_cells.Chrom_SOX2_statusHigh.BCAN	2.34570323	0.24768216	3.20739757	0.00198098	0.00682804	-1.584873	BCAN	assignmentsChromaffin_cells.Chrom_SOX2_statusHigh
assignmentsChromaffin_cells.Chrom_SOX2_statusHigh.DENND4C	-0.670184	7.60013265	-3.36277	0.0012244	0.00455823	-1.5862586	DENND4C	assignmentsChromaffin_cells.Chrom_SOX2_statusHigh
assignmentsChromaffin_cells.Chrom_SOX2_statusHigh.HSPA1B	3.81219407	5.65093509	3.35246179	0.00126608	0.00469056	-1.5897669	HSPA1B	assignmentsChromaffin_cells.Chrom_SOX2_statusHigh
assignmentsChromaffin_cells.Chrom_SOX2_statusHigh.UNC5B	2.01076468	5.09598183	3.33332174	0.00134412	0.00492281	-1.5909511	UNC5B	assignmentsChromaffin_cells.Chrom_SOX2_statusHigh
assignmentsChromaffin_cells.Chrom_SOX2_statusHigh.SIK3	-0.9380868	8.98885428	-3.3756002	0.00117603	0.00440785	-1.592507	SIK3	assignmentsChromaffin_cells.Chrom_SOX2_statusHigh
assignmentsChromaffin_cells.Chrom_SOX2_statusHigh.RMST	-3.5943462	2.14608718	-3.2404719	0.00179124	0.00627659	-1.5925322	RMST	assignmentsChromaffin_cells.Chrom_SOX2_statusHigh
assignmentsChromaffin_cells.Chrom_SOX2_statusHigh.CEP95	-0.6116765	6.5022849	-3.3501372	0.00127384	0.00471495	-1.5936799	CEP95	assignmentsChromaffin_cells.Chrom_SOX2_statusHigh
assignmentsChromaffin_cells.Chrom_SOX2_statusHigh.SLC29A2	-1.1867882	1.88321945	-3.2627841	0.00167078	0.00589309	-1.5960743	SLC29A2	assignmentsChromaffin_cells.Chrom_SOX2_statusHigh
assignmentsChromaffin_cells.Chrom_SOX2_statusHigh.CYB561D1	1.05185104	2.66782051	3.27054702	0.00163128	0.00577666	-1.5975705	CYB561D1	assignmentsChromaffin_cells.Chrom_SOX2_statusHigh

assignmentsChromaffin_cells.Chrom_SOX2_statusHigh.TMEM163	-2.8060913	5.13741309	-3.2965202	0.00150705	0.00539874	-1.5986221	TMEM163	assignmentsChromaffin_cells.Chrom_SOX2_statusHigh
assignmentsChromaffin_cells.Chrom_SOX2_statusHigh.MAFA	2.65567238	-0.6717466	3.1225338	0.00255864	0.00845221	-1.600231	MAFA	assignmentsChromaffin_cells.Chrom_SOX2_statusHigh
assignmentsChromaffin_cells.Chrom_SOX2_statusHigh.NT5DC2	1.34899731	5.26624359	3.35550952	0.00125322	0.00465258	-1.6008206	NT5DC2	assignmentsChromaffin_cells.Chrom_SOX2_statusHigh
assignmentsChromaffin_cells.Chrom_SOX2_statusHigh.GIGYF1	-0.7647348	5.00906295	-3.3160769	0.0014167	0.0051312	-1.6016289	GIGYF1	assignmentsChromaffin_cells.Chrom_SOX2_statusHigh
assignmentsChromaffin_cells.Chrom_SOX2_statusHigh.DIDO1	-0.4904353	6.35906105	-3.3418118	0.00130745	0.00481601	-1.6028239	DIDO1	assignmentsChromaffin_cells.Chrom_SOX2_statusHigh
assignmentsChromaffin_cells.Chrom_SOX2_statusHigh.SLC13A4	-2.4056193	1.55387875	-3.1882367	0.00209954	0.00716131	-1.6031804	SLC13A4	assignmentsChromaffin_cells.Chrom_SOX2_statusHigh
assignmentsChromaffin_cells.Chrom_SOX2_statusHigh.OSER1	0.68423999	4.53710295	3.31981057	0.00140033	0.00508571	-1.6051019	OSER1	assignmentsChromaffin_cells.Chrom_SOX2_statusHigh
assignmentsChromaffin_cells.Chrom_SOX2_statusHigh.OGN	-2.0051034	1.42464585	-3.1874484	0.00210395	0.00717087	-1.6080188	OGN	assignmentsChromaffin_cells.Chrom_SOX2_statusHigh
assignmentsChromaffin_cells.Chrom_SOX2_statusHigh.ISYNA1	1.73003628	2.61594478	3.2354649	0.0018176	0.00634677	-1.6084234	ISYNA1	assignmentsChromaffin_cells.Chrom_SOX2_statusHigh
assignmentsChromaffin_cells.Chrom_SOX2_statusHigh.EGOT	-2.343435	1.30571206	-3.1703902	0.0022159	0.00747709	-1.6085888	EGOT	assignmentsChromaffin_cells.Chrom_SOX2_statusHigh
assignmentsChromaffin_cells.Chrom_SOX2_statusHigh.MRPL3	0.68764435	5.40905617	3.33355161	0.00134162	0.00491478	-1.6091598	MRPL3	assignmentsChromaffin_cells.Chrom_SOX2_statusHigh
assignmentsChromaffin_cells.Chrom_SOX2_statusHigh.CTBP2	-0.8751565	7.34198287	-3.3467696	0.00128734	0.00475284	-1.6098927	CTBP2	assignmentsChromaffin_cells.Chrom_SOX2_statusHigh
assignmentsChromaffin_cells.Chrom_SOX2_statusHigh.ALDOA	2.27666885	3.74072613	3.3179193	0.00141019	0.00511108	-1.6108635	ALDOA	assignmentsChromaffin_cells.Chrom_SOX2_statusHigh
assignmentsChromaffin_cells.Chrom_SOX2_statusHigh.RCN1	1.11562284	4.64491755	3.31650925	0.00141479	0.00512546	-1.6114224	RCN1	assignmentsChromaffin_cells.Chrom_SOX2_statusHigh
assignmentsChromaffin_cells.Chrom_SOX2_statusHigh.CD163L1	1.8162105	4.78159194	3.32093395	0.00139702	0.00507943	-1.6116926	CD163L1	assignmentsChromaffin_cells.Chrom_SOX2_statusHigh
assignmentsChromaffin_cells.Chrom_SOX2_statusHigh.CFLAR-AS1	-2.4186685	2.55729734	-3.172781	0.00220017	0.0074381	-1.6126136	CFLAR-AS1	assignmentsChromaffin_cells.Chrom_SOX2_statusHigh
assignmentsChromaffin_cells.Chrom_SOX2_statusHigh.ORC5	-0.7920065	5.45076767	-3.3204418	0.00139758	0.00508033	-1.6159472	ORC5	assignmentsChromaffin_cells.Chrom_SOX2_statusHigh
assignmentsChromaffin_cells.Chrom_SOX2_statusHigh.ZBTB24	0.93364187	3.81385572	3.28719431	0.00154951	0.00552578	-1.6167801	ZBTB24	assignmentsChromaffin_cells.Chrom_SOX2_statusHigh
assignmentsChromaffin_cells.Chrom_SOX2_statusHigh.SPDYE5	-1.6970668	2.17088816	-3.2389383	0.00179836	0.00629739	-1.617106	SPDYE5	assignmentsChromaffin_cells.Chrom_SOX2_statusHigh
assignmentsChromaffin_cells.Chrom_SOX2_statusHigh.PARP8	-0.9803142	6.92545932	-3.3303994	0.00135488	0.00495543	-1.6177726	PARP8	assignmentsChromaffin_cells.Chrom_SOX2_statusHigh
assignmentsChromaffin_cells.Chrom_SOX2_statusHigh.CNIH2	1.2702658	1.59509442	3.27076111	0.0016302	0.00577412	-1.6223283	CNIH2	assignmentsChromaffin_cells.Chrom_SOX2_statusHigh
assignmentsChromaffin_cells.Chrom_SOX2_statusHigh.SMARCD3	-1.1655727	4.14366779	-3.2920671	0.00152631	0.00545796	-1.623811	SMARCD3	assignmentsChromaffin_cells.Chrom_SOX2_statusHigh
assignmentsChromaffin_cells.Chrom_SOX2_statusHigh.CNTRL	-0.8473106	6.16884644	-3.3153106	0.00142008	0.00514229	-1.6243534	CNTRL	assignmentsChromaffin_cells.Chrom_SOX2_statusHigh
assignmentsChromaffin_cells.Chrom_SOX2_statusHigh.DGKG	1.87121763	4.60452406	3.31901691	0.00140538	0.00509942	-1.6257244	DGKG	assignmentsChromaffin_cells.Chrom_SOX2_statusHigh
assignmentsChromaffin_cells.Chrom_SOX2_statusHigh.TRHDE	2.87580637	1.62641028	3.26908367	0.00164042	0.00580132	-1.6260951	TRHDE	assignmentsChromaffin_cells.Chrom_SOX2_statusHigh
assignmentsChromaffin_cells.Chrom_SOX2_statusHigh.MYO1H	-2.303753	0.38977584	-3.1243351	0.00254486	0.00841709	-1.6305273	MYO1H	assignmentsChromaffin_cells.Chrom_SOX2_statusHigh
assignmentsChromaffin_cells.Chrom_SOX2_statusHigh.NDST3	-3.6925547	1.55153458	-3.1715453	0.00220966	0.00746387	-1.6317427	NDST3	assignmentsChromaffin_cells.Chrom_SOX2_statusHigh
assignmentsChromaffin_cells.Chrom_SOX2_statusHigh.PDE1C	2.60741157	4.45986531	3.25164794	0.00173084	0.00608356	-1.6318327	PDE1C	assignmentsChromaffin_cells.Chrom_SOX2_statusHigh
assignmentsChromaffin_cells.Chrom_SOX2_statusHigh.MRM1	1.12474248	1.48125618	3.22303626	0.0018875	0.00654974	-1.6326287	MRM1	assignmentsChromaffin_cells.Chrom_SOX2_statusHigh
assignmentsChromaffin_cells.Chrom_SOX2_statusHigh.NONO	0.57706236	6.09649712	3.33695714	0.00132743	0.00487059	-1.634259	NONO	assignmentsChromaffin_cells.Chrom_SOX2_statusHigh
assignmentsChromaffin_cells.Chrom_SOX2_statusHigh.ZC3H7A	-0.6318053	6.7045878	-3.3365624	0.00132907	0.00487549	-1.634366	ZC3H7A	assignmentsChromaffin_cells.Chrom_SOX2_statusHigh
assignmentsChromaffin_cells.Chrom_SOX2_statusHigh.KAT2B	-1.0353049	5.74207882	-3.2927555	0.00152306	0.00544876	-1.6359916	KAT2B	assignmentsChromaffin_cells.Chrom_SOX2_statusHigh
assignmentsChromaffin_cells.Chrom_SOX2_statusHigh.FLRT1	1.85036294	1.59500422	3.262995	0.00167114	0.00589309	-1.6381553	FLRT1	assignmentsChromaffin_cells.Chrom_SOX2_statusHigh
assignmentsChromaffin_cells.Chrom_SOX2_statusHigh.RIOK2	0.63800271	4.30635924	3.30281037	0.00147631	0.00530282	-1.6382059	RIOK2	assignmentsChromaffin_cells.Chrom_SOX2_statusHigh
assignmentsChromaffin_cells.Chrom_SOX2_statusHigh.NMNAT2	-1.3528646	5.65332111	-3.3401267	0.00131501	0.00483606	-1.638879	NMNAT2	assignmentsChromaffin_cells.Chrom_SOX2_statusHigh
assignmentsChromaffin_cells.Chrom_SOX2_statusHigh.ZNF75A	-0.7509331	5.15047622	-3.3112897	0.00143794	0.00519525	-1.6390352	ZNF75A	assignmentsChromaffin_cells.Chrom_SOX2_statusHigh
assignmentsChromaffin_cells.Chrom_SOX2_statusHigh.BROX	0.79327242	4.75223665	3.30816103	0.00145199	0.00523421	-1.643155	BROX	assignmentsChromaffin_cells.Chrom_SOX2_statusHigh
assignmentsChromaffin_cells.Chrom_SOX2_statusHigh.KCNJ8	-1.9932065	3.40498543	-3.2612249	0.00168061	0.00592258	-1.6467356	KCNJ8	assignmentsChromaffin_cells.Chrom_SOX2_statusHigh
assignmentsChromaffin_cells.Chrom_SOX2_statusHigh.DDX17	-0.8192043	9.14310056	-3.3661239	0.00121158	0.004521	-1.6484324	DDX17	assignmentsChromaffin_cells.Chrom_SOX2_statusHigh
assignmentsChromaffin_cells.Chrom_SOX2_statusHigh.ZZEF1	-0.7818133	6.77826875	-3.3274752	0.00136729	0.00499285	-1.6486717	ZZEF1	assignmentsChromaffin_cells.Chrom_SOX2_statusHigh
assignmentsChromaffin_cells.Chrom_SOX2_statusHigh.FIGN	-1.5180782	5.94652989	-3.3234324	0.0013859	0.00504597	-1.6521113	FIGN	assignmentsChromaffin_cells.Chrom_SOX2_statusHigh
assignmentsChromaffin_cells.Chrom_SOX2_statusHigh.CUL5	-0.6009998	6.90584228	-3.3340823	0.0013394	0.00490906	-1.6537152	CUL5	assignmentsChromaffin_cells.Chrom_SOX2_statusHigh
assignmentsChromaffin_cells.Chrom_SOX2_statusHigh.TMED8	-0.6128436	4.90290554	-3.3012726	0.00148337	0.00532579	-1.6537511	TMED8	assignmentsChromaffin_cells.Chrom_SOX2_statusHigh
assignmentsChromaffin_cells.Chrom_SOX2_statusHigh.RBX1	1.09609034	5.67590608	3.32410348	0.00138173	0.00503413	-1.6540996	RBX1	assignmentsChromaffin_cells.Chrom_SOX2_statusHigh
assignmentsChromaffin_cells.Chrom_SOX2_statusHigh.SERTAD4-A!	1.24754496	1.26931951	3.23875563	0.00179882	0.00629754	-1.6551913	SERTAD4-AS	assignmentsChromaffin_cells.Chrom_SOX2_statusHigh
assignmentsChromaffin_cells.Chrom_SOX2_statusHigh.PPIE	0.8194919	4.16057577	3.2913137	0.00152987	0.00546827	-1.65566	PPIE	assignmentsChromaffin_cells.Chrom_SOX2_statusHigh
assignmentsChromaffin_cells.Chrom_SOX2_statusHigh.ZMYND8	-0.9098184	7.43368278	-3.346291	0.00128927	0.00475777	-1.65611	ZMYND8	assignmentsChromaffin_cells.Chrom_SOX2_statusHigh
assignmentsChromaffin_cells.Chrom_SOX2_statusHigh.WDR74	1.11297919	3.35293125	3.26745111	0.00164693	0.00581666	-1.658152	WDR74	assignmentsChromaffin_cells.Chrom_SOX2_statusHigh
assignmentsChromaffin_cells.Chrom_SOX2_statusHigh.ZDHHC20	-0.730923	6.94722307	-3.3293892	0.00135916	0.00496994	-1.6599102	ZDHHC20	assignmentsChromaffin_cells.Chrom_SOX2_statusHigh
assignmentsChromaffin_cells.Chrom_SOX2_statusHigh.B4GALT2	0.8727382	1.94130423	3.24364597	0.00177204	0.0062161	-1.6604361	B4GALT2	assignmentsChromaffin_cells.Chrom_SOX2_statusHigh
assignmentsChromaffin_cells.Chrom_SOX2_statusHigh.ASXL3	-3.7859434	1.66281304	-3.1444002	0.00239825	0.00800315	-1.6617751	ASXL3	assignmentsChromaffin_cells.Chrom_SOX2_statusHigh
assignmentsChromaffin_cells.Chrom_SOX2_statusHigh.LATS1	-0.6915283	6.27590374	-3.318945	0.00140411	0.00509597	-1.6618728	LATS1	assignmentsChromaffin_cells.Chrom_SOX2_statusHigh
assignmentsChromaffin_cells.Chrom_SOX2_statusHigh.HTR7	2.55081323	0.53324951	3.1268747	0.00252623	0.00837273	-1.6626804	HTR7	assignmentsChromaffin_cells.Chrom_SOX2_statusHigh
assignmentsChromaffin_cells.Chrom_SOX2_statusHigh.ARGLU1	-0.8264227	8.51407584	-3.3491834	0.00127765	0.00472578	-1.6648533	ARGLU1	assignmentsChromaffin_cells.Chrom_SOX2_statusHigh
assignmentsChromaffin_cells.Chrom_SOX2_statusHigh.RAPGEF6	-0.5309901	7.57154326	-3.338123	0.00132261	0.00485956	-1.6654967	RAPGEF6	assignmentsChromaffin_cells.Chrom_SOX2_statusHigh
assignmentsChromaffin_cells.Chrom_SOX2_statusHigh.TRIM44	-0.764417	7.18483014	-3.3270361	0.00136916	0.00499628	-1.6671879	TRIM44	assignmentsChromaffin_cells.Chrom_SOX2_statusHigh
assignmentsChromaffin_cells.Chrom_SOX2_statusHigh.LRRC8D	-1.2672243	5.54944392	-3.2882591	0.00154441	0.00551163	-1.6677929	LRRC8D	assignmentsChromaffin_cells.Chrom_SOX2_statusHigh
assignmentsChromaffin_cells.Chrom_SOX2_statusHigh.SZT2	-0.8448176	4.96722697	-3.279071	0.00158892	0.00564909	-1.6681787	SZT2	assignmentsChromaffin_cells.Chrom_SOX2_statusHigh
assignmentsChromaffin_cells.Chrom_SOX2_statusHigh.TUT4	-0.5850997	7.66934368	-3.3379815	0.00132319	0.0048606	-1.6684167	TUT4	assignmentsChromaffin_cells.Chrom_SOX2_statusHigh
assignmentsChromaffin_cells.Chrom_SOX2_statusHigh.CDK17	-0.8900992	7.80590991	-3.3283886	0.0013634	0.00498207	-1.6699367	CDK17	assignmentsChromaffin_cells.Chrom_SOX2_statusHigh
assignmentsChromaffin_cells.Chrom_SOX2_statusHigh.FBXL20	-0.8607768	6.91927861	-3.3251861	0.00137708	0.00502174	-1.6725731	FBXL20	assignmentsChromaffin_cells.Chrom_SOX2_statusHigh
assignmentsChromaffin_cells.Chrom_SOX2_statusHigh.MYSM1	-0.6354278	6.04675413	-3.305797	0.00146269	0.00526667	-1.6725804	MYSM1	assignmentsChromaffin_cells.Chrom_SOX2_statusHigh
assignmentsChromaffin_cells.Chrom_SOX2_statusHigh.BORCS5	-0.8372245	6.17439527	-3.3077822	0.0014537	0.00523919	-1.6733014	BORCS5	assignmentsChromaffin_cells.Chrom_SOX2_statusHigh
assignmentsChromaffin_cells.Chrom_SOX2_statusHigh.AP4E1	-0.679487	5.20501693	-3.2898347	0.0015369	0.00548847	-1.6744513	AP4E1	assignmentsChromaffin_cells.Chrom_SOX2_statusHigh

assignmentsChromaffin_cells.Chrom_SOX2_statusHigh.WWC3-AS1	-1.9476632	0.42847288	-3.1112532	0.00264652	0.00869415	-1.6754051	WWC3-AS1	assignmentsChromaffin_cells.Chrom_SOX2_statusHigh
assignmentsChromaffin_cells.Chrom_SOX2_statusHigh.ADARB2	-3.7633728	2.52318182	-3.1945495	0.00206078	0.00706063	-1.6760835	ADARB2	assignmentsChromaffin_cells.Chrom_SOX2_statusHigh
assignmentsChromaffin_cells.Chrom_SOX2_statusHigh.VCPIP1	0.64715711	4.62460744	3.2859809	0.00155533	0.00554198	-1.6769508	VCPIP1	assignmentsChromaffin_cells.Chrom_SOX2_statusHigh
assignmentsChromaffin_cells.Chrom_SOX2_statusHigh.SREK1IP1	0.5734152	5.43031217	3.30984543	0.00144441	0.00521596	-1.6770878	SREK1IP1	assignmentsChromaffin_cells.Chrom_SOX2_statusHigh
assignmentsChromaffin_cells.Chrom_SOX2_statusHigh.POLR3E	-0.6529585	4.78193723	-3.2831583	0.00156897	0.00558684	-1.6775325	POLR3E	assignmentsChromaffin_cells.Chrom_SOX2_statusHigh
assignmentsChromaffin_cells.Chrom_SOX2_statusHigh.THOC3	1.27154509	2.46635008	3.24053045	0.00178906	0.00627168	-1.6778003	THOC3	assignmentsChromaffin_cells.Chrom_SOX2_statusHigh
assignmentsChromaffin_cells.Chrom_SOX2_statusHigh.MDC1-AS1	-1.7344746	0.67626187	-3.1457949	0.00238596	0.0079671	-1.67785	MDC1-AS1	assignmentsChromaffin_cells.Chrom_SOX2_statusHigh
assignmentsChromaffin_cells.Chrom_SOX2_statusHigh.ELF2	-0.7446398	8.06191715	-3.3355526	0.00133327	0.00488865	-1.6810749	ELF2	assignmentsChromaffin_cells.Chrom_SOX2_statusHigh
assignmentsChromaffin_cells.Chrom_SOX2_statusHigh.CEBPZOS	0.69265044	5.18307197	3.30632357	0.0014603	0.00525944	-1.6817607	CEBPZOS	assignmentsChromaffin_cells.Chrom_SOX2_statusHigh
assignmentsChromaffin_cells.Chrom_SOX2_statusHigh.TUBGCP5	-0.7229794	5.12452074	-3.292674	0.00152344	0.00544892	-1.6817992	TUBGCP5	assignmentsChromaffin_cells.Chrom_SOX2_statusHigh
assignmentsChromaffin_cells.Chrom_SOX2_statusHigh.DLEU2	-0.8955994	8.99041158	-3.3484773	0.00128048	0.00473296	-1.6846499	DLEU2	assignmentsChromaffin_cells.Chrom_SOX2_statusHigh
assignmentsChromaffin_cells.Chrom_SOX2_statusHigh.GNB3	-1.1627381	1.77400322	-3.2308163	0.00184311	0.00642047	-1.6855195	GNB3	assignmentsChromaffin_cells.Chrom_SOX2_statusHigh
assignmentsChromaffin_cells.Chrom_SOX2_statusHigh.TMEM14B	-0.7034685	7.05123505	-3.3256366	0.00137515	0.00501584	-1.6878485	TMEM14B	assignmentsChromaffin_cells.Chrom_SOX2_statusHigh
assignmentsChromaffin_cells.Chrom_SOX2_statusHigh.OGA	-0.5776226	7.67430677	-3.3324139	0.00134639	0.00492776	-1.6892361	OGA	assignmentsChromaffin_cells.Chrom_SOX2_statusHigh
assignmentsChromaffin_cells.Chrom_SOX2_statusHigh.NT5C2	-0.7172856	7.24811877	-3.3218178	0.00139161	0.0050632	-1.6893509	NT5C2	assignmentsChromaffin_cells.Chrom_SOX2_statusHigh
assignmentsChromaffin_cells.Chrom_SOX2_statusHigh.THY1	2.58089669	2.61092309	3.18738119	0.00210613	0.00717616	-1.6906291	THY1	assignmentsChromaffin_cells.Chrom_SOX2_statusHigh
assignmentsChromaffin_cells.Chrom_SOX2_statusHigh.COL4A3	-2.524005	1.29039256	-3.1209136	0.00257178	0.00848514	-1.6917008	COL4A3	assignmentsChromaffin_cells.Chrom_SOX2_statusHigh
assignmentsChromaffin_cells.Chrom_SOX2_statusHigh.CCL28	2.17120909	0.46681207	3.1250251	0.00253961	0.00840318	-1.693203	CCL28	assignmentsChromaffin_cells.Chrom_SOX2_statusHigh
assignmentsChromaffin_cells.Chrom_SOX2_statusHigh.SCGB2B2	-0.8112214	5.15463309	-3.2960004	0.00150782	0.00540028	-1.695866	SCGB2B2	assignmentsChromaffin_cells.Chrom_SOX2_statusHigh
assignmentsChromaffin_cells.Chrom_SOX2_statusHigh.STX1B	-1.9804573	1.12732109	-3.1633132	0.00226374	0.00760969	-1.6985521	STX1B	assignmentsChromaffin_cells.Chrom_SOX2_statusHigh
assignmentsChromaffin_cells.Chrom_SOX2_statusHigh.CREB3L4	0.96805709	2.0692933	3.23233819	0.00183454	0.00639618	-1.6987324	CREB3L4	assignmentsChromaffin_cells.Chrom_SOX2_statusHigh
assignmentsChromaffin_cells.Chrom_SOX2_statusHigh.SIMC1	-0.798023	5.6432598	-3.298799	0.0014948	0.00536081	-1.6996496	SIMC1	assignmentsChromaffin_cells.Chrom_SOX2_statusHigh
assignmentsChromaffin_cells.Chrom_SOX2_statusHigh.MTR	-0.6113938	7.06050617	-3.3208674	0.00139573	0.0050759	-1.7020579	MTR	assignmentsChromaffin_cells.Chrom_SOX2_statusHigh
assignmentsChromaffin_cells.Chrom_SOX2_statusHigh.UBAC2-AS1	1.1220936	1.13663534	3.19764757	0.00203944	0.00700095	-1.7029965	UBAC2-AS1	assignmentsChromaffin_cells.Chrom_SOX2_statusHigh
assignmentsChromaffin_cells.Chrom_SOX2_statusHigh.CYP2E1	-1.5620822	1.27190588	-3.167329	0.00223585	0.00753491	-1.705073	CYP2E1	assignmentsChromaffin_cells.Chrom_SOX2_statusHigh
assignmentsChromaffin_cells.Chrom_SOX2_statusHigh.PRNP	1.49193219	5.64254058	3.30600421	0.00146307	0.00526667	-1.7075166	PRNP	assignmentsChromaffin_cells.Chrom_SOX2_statusHigh
assignmentsChromaffin_cells.Chrom_SOX2_statusHigh.SKOR1	-1.2678432	0.58465323	-3.1710179	0.00221104	0.00746542	-1.707637	SKOR1	assignmentsChromaffin_cells.Chrom_SOX2_statusHigh
assignmentsChromaffin_cells.Chrom_SOX2_statusHigh.SKIDA1	1.23303099	0.65455985	3.18202087	0.00213856	0.0072712	-1.7081174	SKIDA1	assignmentsChromaffin_cells.Chrom_SOX2_statusHigh
assignmentsChromaffin_cells.Chrom_SOX2_statusHigh.CHGB	3.26352548	9.47575235	3.37907531	0.00116464	0.00436821	-1.7084323	CHGB	assignmentsChromaffin_cells.Chrom_SOX2_statusHigh
assignmentsChromaffin_cells.Chrom_SOX2_statusHigh.TFAP2E	-1.1928016	1.98267913	-3.1990147	0.00203097	0.00697638	-1.7086178	TFAP2E	assignmentsChromaffin_cells.Chrom_SOX2_statusHigh
assignmentsChromaffin_cells.Chrom_SOX2_statusHigh.CACNA1C	-1.6650436	9.0793918	-3.3524643	0.00126607	0.00469056	-1.7093384	CACNA1C	assignmentsChromaffin_cells.Chrom_SOX2_statusHigh
assignmentsChromaffin_cells.Chrom_SOX2_statusHigh.HERC4	-0.6323077	8.20402042	-3.3340709	0.00133945	0.00490906	-1.7101236	HERC4	assignmentsChromaffin_cells.Chrom_SOX2_statusHigh
assignmentsChromaffin_cells.Chrom_SOX2_statusHigh.AASS	-1.0792257	5.5373651	-3.2874268	0.00154839	0.00552461	-1.7108228	AASS	assignmentsChromaffin_cells.Chrom_SOX2_statusHigh
assignmentsChromaffin_cells.Chrom_SOX2_statusHigh.TERF2IP	0.69254652	7.26179566	3.3316756	0.0013495	0.004938	-1.7123005	TERF2IP	assignmentsChromaffin_cells.Chrom_SOX2_statusHigh
assignmentsChromaffin_cells.Chrom_SOX2_statusHigh.CDCA2	1.35147149	1.55025717	3.20798495	0.00197624	0.00681466	-1.7123592	CDCA2	assignmentsChromaffin_cells.Chrom_SOX2_statusHigh
assignmentsChromaffin_cells.Chrom_SOX2_statusHigh.PDK1	-0.9380166	4.71008794	-3.2650404	0.00165921	0.00585359	-1.713878	PDK1	assignmentsChromaffin_cells.Chrom_SOX2_statusHigh
assignmentsChromaffin_cells.Chrom_SOX2_statusHigh.AS3MT	-1.1408343	3.1237707	-3.2449659	0.00176488	0.00619367	-1.7140288	AS3MT	assignmentsChromaffin_cells.Chrom_SOX2_statusHigh
assignmentsChromaffin_cells.Chrom_SOX2_statusHigh.MED25	0.71707415	4.31371973	3.27855038	0.00159147	0.00565567	-1.7145598	MED25	assignmentsChromaffin_cells.Chrom_SOX2_statusHigh
assignmentsChromaffin_cells.Chrom_SOX2_statusHigh.KEL	-1.7509357	0.43411845	-3.1107821	0.00265025	0.00870285	-1.7159347	KEL	assignmentsChromaffin_cells.Chrom_SOX2_statusHigh
assignmentsChromaffin_cells.Chrom_SOX2_statusHigh.PDZD4	-0.954471	2.93088006	-3.244016	0.00177003	0.0062104	-1.7167329	PDZD4	assignmentsChromaffin_cells.Chrom_SOX2_statusHigh
assignmentsChromaffin_cells.Chrom_SOX2_statusHigh.MRPS18A	0.93335699	3.4237311	3.2543831	0.00171454	0.00603024	-1.7194655	MRPS18A	assignmentsChromaffin_cells.Chrom_SOX2_statusHigh
assignmentsChromaffin_cells.Chrom_SOX2_statusHigh.MSRA	-0.9046663	6.13804436	-3.2713341	0.00162733	0.00576774	-1.726433	MSRA	assignmentsChromaffin_cells.Chrom_SOX2_statusHigh
assignmentsChromaffin_cells.Chrom_SOX2_statusHigh.PNP	1.33493551	3.91052966	3.25157384	0.00172955	0.00608034	-1.7267467	PNP	assignmentsChromaffin_cells.Chrom_SOX2_statusHigh
assignmentsChromaffin_cells.Chrom_SOX2_statusHigh.ADARB1	-1.0681852	5.83548258	-3.2661675	0.00165346	0.00583458	-1.7268354	ADARB1	assignmentsChromaffin_cells.Chrom_SOX2_statusHigh
assignmentsChromaffin_cells.Chrom_SOX2_statusHigh.CEBPA	2.61505838	0.0236542	3.09860135	0.00274915	0.00897339	-1.7295968	CEBPA	assignmentsChromaffin_cells.Chrom_SOX2_statusHigh
assignmentsChromaffin_cells.Chrom_SOX2_statusHigh.PIK3C2B	-1.291428	4.13058678	-3.2358951	0.00181466	0.00633956	-1.7323142	PIK3C2B	assignmentsChromaffin_cells.Chrom_SOX2_statusHigh
assignmentsChromaffin_cells.Chrom_SOX2_statusHigh.AEBP2	-0.7789379	7.11167248	-3.3067936	0.00145817	0.005254	-1.7340132	AEBP2	assignmentsChromaffin_cells.Chrom_SOX2_statusHigh
assignmentsChromaffin_cells.Chrom_SOX2_statusHigh.HERPUD1	1.2299779	6.61837691	3.30314029	0.00147528	0.00530241	-1.7347382	HERPUD1	assignmentsChromaffin_cells.Chrom_SOX2_statusHigh
assignmentsChromaffin_cells.Chrom_SOX2_statusHigh.DLK1	4.24347405	8.62895379	3.36112231	0.00123219	0.00458087	-1.74167	DLK1	assignmentsChromaffin_cells.Chrom_SOX2_statusHigh
assignmentsChromaffin_cells.Chrom_SOX2_statusHigh.FSD1L	-0.9242481	5.57975344	-3.2871426	0.00154975	0.00552578	-1.7436603	FSD1L	assignmentsChromaffin_cells.Chrom_SOX2_statusHigh
assignmentsChromaffin_cells.Chrom_SOX2_statusHigh.CRY2	-1.0430537	5.31190294	-3.2680548	0.00164387	0.00580778	-1.7439524	CRY2	assignmentsChromaffin_cells.Chrom_SOX2_statusHigh
assignmentsChromaffin_cells.Chrom_SOX2_statusHigh.MLLT6	-0.6417156	6.0433872	-3.2903675	0.00153436	0.00548065	-1.7440014	MLLT6	assignmentsChromaffin_cells.Chrom_SOX2_statusHigh
assignmentsChromaffin_cells.Chrom_SOX2_statusHigh.RABGEF1	-0.7960259	7.39749222	-3.3030075	0.00147541	0.00530241	-1.7464721	RABGEF1	assignmentsChromaffin_cells.Chrom_SOX2_statusHigh
assignmentsChromaffin_cells.Chrom_SOX2_statusHigh.TMTC4	-1.2159382	5.18158537	-3.2766541	0.00160106	0.0056822	-1.748297	TMTC4	assignmentsChromaffin_cells.Chrom_SOX2_statusHigh
assignmentsChromaffin_cells.Chrom_SOX2_statusHigh.TPK1	-0.9868887	5.58774252	-3.239557	0.00179441	0.00628631	-1.7486969	TPK1	assignmentsChromaffin_cells.Chrom_SOX2_statusHigh
assignmentsChromaffin_cells.Chrom_SOX2_statusHigh.ATF1	-0.7039393	5.88986957	-3.2829108	0.00157017	0.00558988	-1.7506832	ATF1	assignmentsChromaffin_cells.Chrom_SOX2_statusHigh
assignmentsChromaffin_cells.Chrom_SOX2_statusHigh.TP53I11	2.08289929	2.34933451	3.13833869	0.00244076	0.00813146	-1.7529201	TP53I11	assignmentsChromaffin_cells.Chrom_SOX2_statusHigh
assignmentsChromaffin_cells.Chrom_SOX2_statusHigh.SAR1B	0.60385284	5.6561259	3.28850296	0.00154324	0.00550869	-1.7572302	SAR1B	assignmentsChromaffin_cells.Chrom_SOX2_statusHigh
assignmentsChromaffin_cells.Chrom_SOX2_statusHigh.HOXA9	4.45107578	2.5208998	3.21476417	0.00193776	0.00669493	-1.7592052	HOXA9	assignmentsChromaffin_cells.Chrom_SOX2_statusHigh
assignmentsChromaffin_cells.Chrom_SOX2_statusHigh.KDM1A	-0.6384135	5.6747338	-3.2708339	0.00162984	0.0057741	-1.7609938	KDM1A	assignmentsChromaffin_cells.Chrom_SOX2_statusHigh
assignmentsChromaffin_cells.Chrom_SOX2_statusHigh.FLVCR1	-0.7259927	4.98397245	-3.2680181	0.00164405	0.00580778	-1.7612964	FLVCR1	assignmentsChromaffin_cells.Chrom_SOX2_statusHigh
assignmentsChromaffin_cells.Chrom_SOX2_statusHigh.PCAT6	1.46537587	0.87898236	3.18020492	0.00215037	0.00729897	-1.7624981	PCAT6	assignmentsChromaffin_cells.Chrom_SOX2_statusHigh
assignmentsChromaffin_cells.Chrom_SOX2_statusHigh.TPCN2	-1.0340708	3.85089108	-3.225407	0.00187387	0.00650924	-1.7635307	TPCN2	assignmentsChromaffin_cells.Chrom_SOX2_statusHigh
assignmentsChromaffin_cells.Chrom_SOX2_statusHigh.C1orf112	-0.954682	3.89752118	-3.2207571	0.0019007	0.00658673	-1.7645819	C1orf112	assignmentsChromaffin_cells.Chrom_SOX2_statusHigh

assignmentsChromaffin_cells.Chrom_SOX2_statusHigh.SMARCC1	-0.7533279	6.96635933	-3.2866628	0.00155206	0.00553276	-1.7651302	SMARCC1	assignmentsChromaffin_cells.Chrom_SOX2_statusHigh
assignmentsChromaffin_cells.Chrom_SOX2_statusHigh.RGPD3	-1.5582042	0.78140384	-3.1095289	0.0026602	0.00873015	-1.766615	RGPD3	assignmentsChromaffin_cells.Chrom_SOX2_statusHigh
assignmentsChromaffin_cells.Chrom_SOX2_statusHigh.KNSTRN	0.84501447	2.35895199	3.21493044	0.00193482	0.00668764	-1.7674148	KNSTRN	assignmentsChromaffin_cells.Chrom_SOX2_statusHigh
assignmentsChromaffin_cells.Chrom_SOX2_statusHigh.ANKFY1	-0.7909536	5.90213997	-3.2683266	0.00164249	0.00580609	-1.7675501	ANKFY1	assignmentsChromaffin_cells.Chrom_SOX2_statusHigh
assignmentsChromaffin_cells.Chrom_SOX2_statusHigh.RPS6KB1	-0.4963771	6.05452403	-3.2801258	0.00158375	0.00563445	-1.7699398	RPS6KB1	assignmentsChromaffin_cells.Chrom_SOX2_statusHigh
assignmentsChromaffin_cells.Chrom_SOX2_statusHigh.CDC42EP2	1.59218841	1.4106528	3.11036121	0.00265359	0.00871202	-1.7765273	CDC42EP2	assignmentsChromaffin_cells.Chrom_SOX2_statusHigh
assignmentsChromaffin_cells.Chrom_SOX2_statusHigh.LSM6	0.88067771	4.33002334	3.24877344	0.00174436	0.00612436	-1.7783859	LSM6	assignmentsChromaffin_cells.Chrom_SOX2_statusHigh
assignmentsChromaffin_cells.Chrom_SOX2_statusHigh.MOSMO	-0.6925131	6.24211898	-3.2789146	0.00158968	0.00565057	-1.7786406	MOSMO	assignmentsChromaffin_cells.Chrom_SOX2_statusHigh
assignmentsChromaffin_cells.Chrom_SOX2_statusHigh.KCNG4	3.03068812	0.98864082	3.19293399	0.00207092	0.0070878	-1.7790737	KCNG4	assignmentsChromaffin_cells.Chrom_SOX2_statusHigh
assignmentsChromaffin_cells.Chrom_SOX2_statusHigh.SDCCAG8	-0.6988796	8.04967208	-3.3056639	0.00146329	0.00526667	-1.7805775	SDCCAG8	assignmentsChromaffin_cells.Chrom_SOX2_statusHigh
assignmentsChromaffin_cells.Chrom_SOX2_statusHigh.SNHG21	0.85237461	2.4498252	3.22067423	0.00190118	0.00658697	-1.7811607	SNHG21	assignmentsChromaffin_cells.Chrom_SOX2_statusHigh
assignmentsChromaffin_cells.Chrom_SOX2_statusHigh.ATP6V1E1	0.77412564	5.4879322	3.27619564	0.00160309	0.00568813	-1.7817662	ATP6V1E1	assignmentsChromaffin_cells.Chrom_SOX2_statusHigh
assignmentsChromaffin_cells.Chrom_SOX2_statusHigh.UNC79	-1.4375235	7.01176067	-3.3100764	0.00144499	0.00521601	-1.7835935	UNC79	assignmentsChromaffin_cells.Chrom_SOX2_statusHigh
assignmentsChromaffin_cells.Chrom_SOX2_statusHigh.RPA2	1.11802669	3.89350103	3.22348816	0.0018849	0.00654329	-1.7849016	RPA2	assignmentsChromaffin_cells.Chrom_SOX2_statusHigh
assignmentsChromaffin_cells.Chrom_SOX2_statusHigh.LMO7	-0.9004399	5.79358102	-3.2767885	0.00160016	0.00568024	-1.7862188	LMO7	assignmentsChromaffin_cells.Chrom_SOX2_statusHigh
assignmentsChromaffin_cells.Chrom_SOX2_statusHigh.CPSF4	-0.7818417	4.0682529	-3.233109	0.00183022	0.00638526	-1.7886375	CPSF4	assignmentsChromaffin_cells.Chrom_SOX2_statusHigh
assignmentsChromaffin_cells.Chrom_SOX2_statusHigh.ZBTB8A	-1.0033744	3.86992327	-3.2209089	0.00189982	0.0065851	-1.7914667	ZBTB8A	assignmentsChromaffin_cells.Chrom_SOX2_statusHigh
assignmentsChromaffin_cells.Chrom_SOX2_statusHigh.ARHGEF11	-0.8774038	6.33916486	-3.2778312	0.00159501	0.005667	-1.7924821	ARHGEF11	assignmentsChromaffin_cells.Chrom_SOX2_statusHigh
assignmentsChromaffin_cells.Chrom_SOX2_statusHigh.TRNP1	1.77077522	1.00189296	3.14206001	0.00241309	0.00804765	-1.7934129	TRNP1	assignmentsChromaffin_cells.Chrom_SOX2_statusHigh
assignmentsChromaffin_cells.Chrom_SOX2_statusHigh.CD2AP	-0.56113	6.5957687	-3.2716028	0.00162598	0.00576424	-1.7942547	CD2AP	assignmentsChromaffin_cells.Chrom_SOX2_statusHigh
assignmentsChromaffin_cells.Chrom_SOX2_statusHigh.NUTM2A-A	-0.79735	7.10317378	-3.2840182	0.0015648	0.00557325	-1.7943332	NUTM2A-AS	assignmentsChromaffin_cells.Chrom_SOX2_statusHigh
assignmentsChromaffin_cells.Chrom_SOX2_statusHigh.MLH1	-0.5634225	5.1494593	-3.2537449	0.00171791	0.00604076	-1.7945903	MLH1	assignmentsChromaffin_cells.Chrom_SOX2_statusHigh
assignmentsChromaffin_cells.Chrom_SOX2_statusHigh.SRPRA	1.16904728	5.66085467	3.27583169	0.00160532	0.00569478	-1.7962946	SRPRA	assignmentsChromaffin_cells.Chrom_SOX2_statusHigh
assignmentsChromaffin_cells.Chrom_SOX2_statusHigh.ACBD3-AS1	-1.6454583	0.30652409	-3.099216	0.00274339	0.008961	-1.7985475	ACBD3-AS1	assignmentsChromaffin_cells.Chrom_SOX2_statusHigh
assignmentsChromaffin_cells.Chrom_SOX2_statusHigh.NLRC3	-2.1225817	1.3978218	-3.0589291	0.00309218	0.0099583	-1.8005042	NLRC3	assignmentsChromaffin_cells.Chrom_SOX2_statusHigh
assignmentsChromaffin_cells.Chrom_SOX2_statusHigh.BAX	1.26391939	4.60465432	3.24898404	0.00174369	0.00612332	-1.8006799	BAX	assignmentsChromaffin_cells.Chrom_SOX2_statusHigh
assignmentsChromaffin_cells.Chrom_SOX2_statusHigh.ITPRID1	1.54441031	-0.0221381	3.06373526	0.0030485	0.00984929	-1.8018179	ITPRID1	assignmentsChromaffin_cells.Chrom_SOX2_statusHigh
assignmentsChromaffin_cells.Chrom_SOX2_statusHigh.SIPA1	1.69839108	2.99480728	3.11876078	0.00258772	0.00852546	-1.8056917	SIPA1	assignmentsChromaffin_cells.Chrom_SOX2_statusHigh
assignmentsChromaffin_cells.Chrom_SOX2_statusHigh.FAM120B	-0.7157961	7.30378114	-3.2816896	0.00157611	0.00560978	-1.8094238	FAM120B	assignmentsChromaffin_cells.Chrom_SOX2_statusHigh
assignmentsChromaffin_cells.Chrom_SOX2_statusHigh.OSCP1	0.9503953	2.13779105	3.18955366	0.00209023	0.00714017	-1.8102909	OSCP1	assignmentsChromaffin_cells.Chrom_SOX2_statusHigh
assignmentsChromaffin_cells.Chrom_SOX2_statusHigh.VPS8	-0.7282608	7.08133603	-3.2772863	0.0015977	0.00567277	-1.8110484	VPS8	assignmentsChromaffin_cells.Chrom_SOX2_statusHigh
assignmentsChromaffin_cells.Chrom_SOX2_statusHigh.LTBP1	-2.5832543	4.86489983	-3.1841031	0.00212718	0.00723865	-1.8149679	LTBP1	assignmentsChromaffin_cells.Chrom_SOX2_statusHigh
assignmentsChromaffin_cells.Chrom_SOX2_statusHigh.ELMO1	-1.4610306	8.51839864	-3.2811538	0.00158044	0.00562393	-1.8150725	ELMO1	assignmentsChromaffin_cells.Chrom_SOX2_statusHigh
assignmentsChromaffin_cells.Chrom_SOX2_statusHigh.CES2	0.53337649	3.73956894	3.22981384	0.00184878	0.00643601	-1.816048	CES2	assignmentsChromaffin_cells.Chrom_SOX2_statusHigh
assignmentsChromaffin_cells.Chrom_SOX2_statusHigh.OGDH	-0.670058	6.43133441	-3.2690935	0.00163861	0.00579621	-1.817585	OGDH	assignmentsChromaffin_cells.Chrom_SOX2_statusHigh
assignmentsChromaffin_cells.Chrom_SOX2_statusHigh.KL	2.4250388	3.47942685	3.23758566	0.00180716	0.00631856	-1.8180808	KL	assignmentsChromaffin_cells.Chrom_SOX2_statusHigh
assignmentsChromaffin_cells.Chrom_SOX2_statusHigh.RENBP	2.01216163	1.82119448	3.09004402	0.00281942	0.00918128	-1.8187961	RENBP	assignmentsChromaffin_cells.Chrom_SOX2_statusHigh
assignmentsChromaffin_cells.Chrom_SOX2_statusHigh.NMUR2	2.8579534	0.18629821	3.11163888	0.00264587	0.0086938	-1.8212822	NMUR2	assignmentsChromaffin_cells.Chrom_SOX2_statusHigh
assignmentsChromaffin_cells.Chrom_SOX2_statusHigh.NRXN1	-2.6114	6.93271946	-3.2799083	0.00158653	0.0056431	-1.8240806	NRXN1	assignmentsChromaffin_cells.Chrom_SOX2_statusHigh
assignmentsChromaffin_cells.Chrom_SOX2_statusHigh.GYG1	0.9308634	3.94171689	3.2068765	0.00198293	0.00683331	-1.8265762	GYG1	assignmentsChromaffin_cells.Chrom_SOX2_statusHigh
assignmentsChromaffin_cells.Chrom_SOX2_statusHigh.BCR	-0.9771188	5.32990776	-3.2327584	0.00183219	0.00638934	-1.8291807	BCR	assignmentsChromaffin_cells.Chrom_SOX2_statusHigh
assignmentsChromaffin_cells.Chrom_SOX2_statusHigh.MMP24	-0.9787901	3.91778944	-3.2193909	0.00190865	0.00660857	-1.8306635	MMP24	assignmentsChromaffin_cells.Chrom_SOX2_statusHigh
assignmentsChromaffin_cells.Chrom_SOX2_statusHigh.YOD1	0.84341284	2.82718533	3.19470164	0.00205779	0.00705188	-1.8313502	YOD1	assignmentsChromaffin_cells.Chrom_SOX2_statusHigh
assignmentsChromaffin_cells.Chrom_SOX2_statusHigh.HMBS	1.09468666	2.25692987	3.1963911	0.00204724	0.00702175	-1.8324517	HMBS	assignmentsChromaffin_cells.Chrom_SOX2_statusHigh
assignmentsChromaffin_cells.Chrom_SOX2_statusHigh.RAE1	0.46861039	4.46436373	3.23212444	0.00183575	0.00639898	-1.8330516	RAE1	assignmentsChromaffin_cells.Chrom_SOX2_statusHigh
assignmentsChromaffin_cells.Chrom_SOX2_statusHigh.DGUOK-AS	-1.6008891	1.69664946	-3.1191119	0.002585	0.0085235	-1.8345107	DGUOK-AS1	assignmentsChromaffin_cells.Chrom_SOX2_statusHigh
assignmentsChromaffin_cells.Chrom_SOX2_statusHigh.RBBP7	0.72866426	5.46924867	3.25445048	0.00171419	0.00603024	-1.8354459	RBBP7	assignmentsChromaffin_cells.Chrom_SOX2_statusHigh
assignmentsChromaffin_cells.Chrom_SOX2_statusHigh.SNX21	0.75953274	2.92940635	3.19535297	0.00205372	0.00704094	-1.8355675	SNX21	assignmentsChromaffin_cells.Chrom_SOX2_statusHigh
assignmentsChromaffin_cells.Chrom_SOX2_statusHigh.BRSK1	-0.8803291	3.88599325	-3.2317314	0.00183796	0.00640529	-1.8360714	BRSK1	assignmentsChromaffin_cells.Chrom_SOX2_statusHigh
assignmentsChromaffin_cells.Chrom_SOX2_statusHigh.GZMB	2.45628012	-0.1994618	3.03879228	0.00328158	0.01045992	-1.8367746	GZMB	assignmentsChromaffin_cells.Chrom_SOX2_statusHigh
assignmentsChromaffin_cells.Chrom_SOX2_statusHigh.BPTF	-0.5889572	8.26162979	-3.2932611	0.00152067	0.00544266	-1.8378538	BPTF	assignmentsChromaffin_cells.Chrom_SOX2_statusHigh
assignmentsChromaffin_cells.Chrom_SOX2_statusHigh.SFT2D2	0.72377412	5.06452612	3.23666884	0.00181037	0.00632839	-1.8378911	SFT2D2	assignmentsChromaffin_cells.Chrom_SOX2_statusHigh
assignmentsChromaffin_cells.Chrom_SOX2_statusHigh.PAQR6	-1.3896573	2.4283203	-3.1877777	0.00210153	0.00716505	-1.8385056	PAQR6	assignmentsChromaffin_cells.Chrom_SOX2_statusHigh
assignmentsChromaffin_cells.Chrom_SOX2_statusHigh.ZNF341	-0.939592	2.60161844	-3.1713194	0.00220903	0.00746332	-1.8389353	ZNF341	assignmentsChromaffin_cells.Chrom_SOX2_statusHigh
assignmentsChromaffin_cells.Chrom_SOX2_statusHigh.FBXL17	-0.6528859	8.27499342	-3.2896382	0.00153783	0.00549059	-1.8396944	FBXL17	assignmentsChromaffin_cells.Chrom_SOX2_statusHigh
assignmentsChromaffin_cells.Chrom_SOX2_statusHigh.PCNP	0.79913126	6.32650388	3.26966426	0.00163573	0.00578857	-1.8427246	PCNP	assignmentsChromaffin_cells.Chrom_SOX2_statusHigh
assignmentsChromaffin_cells.Chrom_SOX2_statusHigh.SLC20A2	-0.6524849	6.55798479	-3.2562354	0.0017048	0.00600124	-1.8433632	SLC20A2	assignmentsChromaffin_cells.Chrom_SOX2_statusHigh
assignmentsChromaffin_cells.Chrom_SOX2_statusHigh.ZXDA	1.97460037	1.44118677	3.15658972	0.00231122	0.00775144	-1.8444469	ZXDA	assignmentsChromaffin_cells.Chrom_SOX2_statusHigh
assignmentsChromaffin_cells.Chrom_SOX2_statusHigh.ZNF32	0.84212992	3.67012179	3.22217251	0.00189249	0.0065622	-1.8471727	ZNF32	assignmentsChromaffin_cells.Chrom_SOX2_statusHigh
assignmentsChromaffin_cells.Chrom_SOX2_statusHigh.WDR27	-0.901023	5.98527914	-3.239059	0.00179715	0.00629454	-1.8477661	WDR27	assignmentsChromaffin_cells.Chrom_SOX2_statusHigh
assignmentsChromaffin_cells.Chrom_SOX2_statusHigh.TIGD5	1.08951207	1.16673665	3.15618366	0.0023124	0.00775378	-1.8507984	TIGD5	assignmentsChromaffin_cells.Chrom_SOX2_statusHigh
assignmentsChromaffin_cells.Chrom_SOX2_statusHigh.ZNF454	-1.4425114	2.49385459	-3.1635597	0.00226148	0.00760589	-1.8512892	ZNF454	assignmentsChromaffin_cells.Chrom_SOX2_statusHigh
assignmentsChromaffin_cells.Chrom_SOX2_statusHigh.ARSD	2.3771738	2.4593126	3.13740734	0.00244921	0.00815455	-1.8524733	ARSD	assignmentsChromaffin_cells.Chrom_SOX2_statusHigh
assignmentsChromaffin_cells.Chrom_SOX2_statusHigh.STRAP	0.99852425	6.26136255	3.26724583	0.00164797	0.00581778	-1.8532531	STRAP	assignmentsChromaffin_cells.Chrom_SOX2_statusHigh

assignmentsChromaffin_cells.Chrom_SOX2_statusHigh.NDUFA10	-0.4971929	6.42009309	-3.2597342	0.00168655	0.00594219	-1.8548482	NDUFA10	assignmentsChromaffin_cells.Chrom_SOX2_statusHigh
assignmentsChromaffin_cells.Chrom_SOX2_statusHigh.YTHDC2	-0.6002719	7.1822649	-3.2672608	0.0016479	0.00581778	-1.8552894	YTHDC2	assignmentsChromaffin_cells.Chrom_SOX2_statusHigh
assignmentsChromaffin_cells.Chrom_SOX2_statusHigh.SPESP1	-1.2095998	3.06282293	-3.1815566	0.00214158	0.00727836	-1.8556224	SPESP1	assignmentsChromaffin_cells.Chrom_SOX2_statusHigh
assignmentsChromaffin_cells.Chrom_SOX2_statusHigh.CHRM3-AS2	-3.6464058	1.05545145	-3.0336531	0.0033344	0.01060002	-1.8567004	CHRM3-AS2	assignmentsChromaffin_cells.Chrom_SOX2_statusHigh
assignmentsChromaffin_cells.Chrom_SOX2_statusHigh.R3HDM4	0.82179211	3.52331795	3.19595084	0.00204999	0.00702965	-1.859608	R3HDM4	assignmentsChromaffin_cells.Chrom_SOX2_statusHigh
assignmentsChromaffin_cells.Chrom_SOX2_statusHigh.SSC4D	1.67011485	-0.1417524	3.03400256	0.00332819	0.01058659	-1.8619749	SSC4D	assignmentsChromaffin_cells.Chrom_SOX2_statusHigh
assignmentsChromaffin_cells.Chrom_SOX2_statusHigh.PPFIA1	-0.6974838	6.69366206	-3.2505206	0.00173502	0.00609691	-1.8628419	PPFIA1	assignmentsChromaffin_cells.Chrom_SOX2_statusHigh
assignmentsChromaffin_cells.Chrom_SOX2_statusHigh.FGFRL1	-2.1872474	0.60950161	-3.0361487	0.00330723	0.01052409	-1.8637315	FGFRL1	assignmentsChromaffin_cells.Chrom_SOX2_statusHigh
assignmentsChromaffin_cells.Chrom_SOX2_statusHigh.TRMT2B	-1.0142499	4.06579245	-3.1885522	0.0020966	0.00715277	-1.8638326	TRMT2B	assignmentsChromaffin_cells.Chrom_SOX2_statusHigh
assignmentsChromaffin_cells.Chrom_SOX2_statusHigh.FBXL2	-0.8282145	5.86207308	-3.2568247	0.00170172	0.00599169	-1.8651432	FBXL2	assignmentsChromaffin_cells.Chrom_SOX2_statusHigh
assignmentsChromaffin_cells.Chrom_SOX2_statusHigh.CYP4B1	2.26068398	-0.4720934	3.03349974	0.00333312	0.01059806	-1.8655427	CYP4B1	assignmentsChromaffin_cells.Chrom_SOX2_statusHigh
assignmentsChromaffin_cells.Chrom_SOX2_statusHigh.ZNF768	0.77704006	2.28350816	3.17667681	0.00217348	0.00736184	-1.8662413	ZNF768	assignmentsChromaffin_cells.Chrom_SOX2_statusHigh
assignmentsChromaffin_cells.Chrom_SOX2_statusHigh.KDM2B	-1.1822404	5.61778946	-3.2221202	0.0018928	0.0065622	-1.8697103	KDM2B	assignmentsChromaffin_cells.Chrom_SOX2_statusHigh
assignmentsChromaffin_cells.Chrom_SOX2_statusHigh.EFCAB6	-0.9736846	3.97820433	-3.2109484	0.00195847	0.00675772	-1.8714571	EFCAB6	assignmentsChromaffin_cells.Chrom_SOX2_statusHigh
assignmentsChromaffin_cells.Chrom_SOX2_statusHigh.LARP1B	-0.6925482	6.63620869	-3.2612425	0.00167874	0.00591857	-1.873535	LARP1B	assignmentsChromaffin_cells.Chrom_SOX2_statusHigh
assignmentsChromaffin_cells.Chrom_SOX2_statusHigh.SHTN1	-1.067207	6.84204145	-3.2703318	0.00163257	0.00577995	-1.8735852	SHTN1	assignmentsChromaffin_cells.Chrom_SOX2_statusHigh
assignmentsChromaffin_cells.Chrom_SOX2_statusHigh.CGAS	1.84218877	3.30946939	3.09778951	0.00275514	0.00899024	-1.8748088	CGAS	assignmentsChromaffin_cells.Chrom_SOX2_statusHigh
assignmentsChromaffin_cells.Chrom_SOX2_statusHigh.AFDN	-0.8797259	6.50568931	-3.2555945	0.00170817	0.00601177	-1.8749332	AFDN	assignmentsChromaffin_cells.Chrom_SOX2_statusHigh
assignmentsChromaffin_cells.Chrom_SOX2_statusHigh.PNMA8C	1.24111043	0.57141447	3.14426984	0.00239694	0.00800043	-1.8749956	PNMA8C	assignmentsChromaffin_cells.Chrom_SOX2_statusHigh
assignmentsChromaffin_cells.Chrom_SOX2_statusHigh.FIP1L1	-0.4249379	6.72979877	-3.2569148	0.00170125	0.00599134	-1.8761447	FIP1L1	assignmentsChromaffin_cells.Chrom_SOX2_statusHigh
assignmentsChromaffin_cells.Chrom_SOX2_statusHigh.CTBP1-AS	-2.4452228	0.49941986	-3.0362276	0.00330672	0.01052409	-1.8769489	CTBP1-AS	assignmentsChromaffin_cells.Chrom_SOX2_statusHigh
assignmentsChromaffin_cells.Chrom_SOX2_statusHigh.PAQR7	1.9686242	1.34847801	3.07825073	0.00292005	0.0094667	-1.8779435	PAQR7	assignmentsChromaffin_cells.Chrom_SOX2_statusHigh
assignmentsChromaffin_cells.Chrom_SOX2_statusHigh.SLC22A23	-1.7466523	5.11900874	-3.1742799	0.00219059	0.00740883	-1.8787723	SLC22A23	assignmentsChromaffin_cells.Chrom_SOX2_statusHigh
assignmentsChromaffin_cells.Chrom_SOX2_statusHigh.DNAJC3-DT	0.90027975	3.66119334	3.21623419	0.00192713	0.00666682	-1.8833832	DNAJC3-DT	assignmentsChromaffin_cells.Chrom_SOX2_statusHigh
assignmentsChromaffin_cells.Chrom_SOX2_statusHigh.PLEKHA7	-1.2350591	5.6165054	-3.2373722	0.00180701	0.00631856	-1.8839844	PLEKHA7	assignmentsChromaffin_cells.Chrom_SOX2_statusHigh
assignmentsChromaffin_cells.Chrom_SOX2_statusHigh.NECTIN1	1.31043156	3.14335393	3.19079982	0.00208234	0.00712079	-1.8839905	NECTIN1	assignmentsChromaffin_cells.Chrom_SOX2_statusHigh
assignmentsChromaffin_cells.Chrom_SOX2_statusHigh.GTPBP8	0.88369332	3.45579475	3.19023991	0.00208588	0.00712834	-1.884639	GTPBP8	assignmentsChromaffin_cells.Chrom_SOX2_statusHigh
assignmentsChromaffin_cells.Chrom_SOX2_statusHigh.NPLOC4	-0.6539174	6.11229647	-3.2384112	0.00180072	0.00630018	-1.884648	NPLOC4	assignmentsChromaffin_cells.Chrom_SOX2_statusHigh
assignmentsChromaffin_cells.Chrom_SOX2_statusHigh.EFCAB14	0.72363496	5.46954676	3.22602787	0.00187032	0.00650112	-1.8846728	EFCAB14	assignmentsChromaffin_cells.Chrom_SOX2_statusHigh
assignmentsChromaffin_cells.Chrom_SOX2_statusHigh.LZTFL1	0.82753172	3.39376702	3.18860454	0.00209626	0.00715277	-1.8852377	LZTFL1	assignmentsChromaffin_cells.Chrom_SOX2_statusHigh
assignmentsChromaffin_cells.Chrom_SOX2_statusHigh.POGLUT1	0.69175412	4.53954444	3.2026833	0.00200842	0.0069067	-1.8895239	POGLUT1	assignmentsChromaffin_cells.Chrom_SOX2_statusHigh
assignmentsChromaffin_cells.Chrom_SOX2_statusHigh.SH3GL2	-2.4438526	1.27319555	-3.1139819	0.0026274	0.00864373	-1.8901372	SH3GL2	assignmentsChromaffin_cells.Chrom_SOX2_statusHigh
assignmentsChromaffin_cells.Chrom_SOX2_statusHigh.SYT9	-1.8427655	4.47502329	-3.21985	0.00190791	0.00660746	-1.8902768	SYT9	assignmentsChromaffin_cells.Chrom_SOX2_statusHigh
assignmentsChromaffin_cells.Chrom_SOX2_statusHigh.NRP2	2.17278515	5.0326614	3.13713395	0.00245123	0.00815956	-1.8917288	NRP2	assignmentsChromaffin_cells.Chrom_SOX2_statusHigh
assignmentsChromaffin_cells.Chrom_SOX2_statusHigh.SAP25	-1.3997899	2.02594898	-3.0972623	0.00275942	0.00900236	-1.893214	SAP25	assignmentsChromaffin_cells.Chrom_SOX2_statusHigh
assignmentsChromaffin_cells.Chrom_SOX2_statusHigh.GPX7	2.59340613	1.17628772	3.06309768	0.00305623	0.00986906	-1.8979239	GPX7	assignmentsChromaffin_cells.Chrom_SOX2_statusHigh
assignmentsChromaffin_cells.Chrom_SOX2_statusHigh.PPP3CA	-0.9745738	8.76107977	-3.2739616	0.00161419	0.00572497	-1.8991729	PPP3CA	assignmentsChromaffin_cells.Chrom_SOX2_statusHigh
assignmentsChromaffin_cells.Chrom_SOX2_statusHigh.MCM3	0.75547918	3.46597414	3.18172562	0.00214048	0.00727617	-1.9017472	MCM3	assignmentsChromaffin_cells.Chrom_SOX2_statusHigh
assignmentsChromaffin_cells.Chrom_SOX2_statusHigh.DNAAF3	1.67080228	0.1550725	3.08436761	0.00286744	0.00931875	-1.9031329	DNAAF3	assignmentsChromaffin_cells.Chrom_SOX2_statusHigh
assignmentsChromaffin_cells.Chrom_SOX2_statusHigh.PMM2	-0.6375411	6.25443616	-3.2356422	0.00181607	0.0063428	-1.9040454	PMM2	assignmentsChromaffin_cells.Chrom_SOX2_statusHigh
assignmentsChromaffin_cells.Chrom_SOX2_statusHigh.WDR19	-0.7266059	5.63996591	-3.2351356	0.00181889	0.00634989	-1.9043029	WDR19	assignmentsChromaffin_cells.Chrom_SOX2_statusHigh
assignmentsChromaffin_cells.Chrom_SOX2_statusHigh.R3HCC1L	-0.5668397	6.13552765	-3.2269509	0.00186504	0.0064856	-1.9062137	R3HCC1L	assignmentsChromaffin_cells.Chrom_SOX2_statusHigh
assignmentsChromaffin_cells.Chrom_SOX2_statusHigh.ARMC6	0.61026445	3.10402994	3.18070651	0.0021471	0.00728942	-1.9066373	ARMC6	assignmentsChromaffin_cells.Chrom_SOX2_statusHigh
assignmentsChromaffin_cells.Chrom_SOX2_statusHigh.BEX2	1.86019352	4.28205592	3.24105737	0.00178803	0.00626945	-1.9070101	BEX2	assignmentsChromaffin_cells.Chrom_SOX2_statusHigh
assignmentsChromaffin_cells.Chrom_SOX2_statusHigh.MGA	-0.5705976	6.53705512	-3.2386903	0.00179918	0.00629754	-1.9077958	MGA	assignmentsChromaffin_cells.Chrom_SOX2_statusHigh
assignmentsChromaffin_cells.Chrom_SOX2_statusHigh.QRICH1	-0.7416907	5.72212131	-3.2170975	0.00192206	0.00665071	-1.9095585	QRICH1	assignmentsChromaffin_cells.Chrom_SOX2_statusHigh
assignmentsChromaffin_cells.Chrom_SOX2_statusHigh.URB1	-0.8017735	5.020166	-3.2026647	0.00200853	0.0069067	-1.9114476	URB1	assignmentsChromaffin_cells.Chrom_SOX2_statusHigh
assignmentsChromaffin_cells.Chrom_SOX2_statusHigh.CRYZ	0.91561801	3.65112873	3.18881679	0.00209491	0.00715159	-1.9117343	CRYZ	assignmentsChromaffin_cells.Chrom_SOX2_statusHigh
assignmentsChromaffin_cells.Chrom_SOX2_statusHigh.CASC17	-2.5793203	0.43582504	-3.0506486	0.00317029	0.01016089	-1.9118333	CASC17	assignmentsChromaffin_cells.Chrom_SOX2_statusHigh
assignmentsChromaffin_cells.Chrom_SOX2_statusHigh.ERCC3	-0.5198588	4.9493142	-3.2030198	0.00200636	0.0069022	-1.9134106	ERCC3	assignmentsChromaffin_cells.Chrom_SOX2_statusHigh
assignmentsChromaffin_cells.Chrom_SOX2_statusHigh.DUSP15	0.94029497	1.64512862	3.15641218	0.00231081	0.00775144	-1.913657	DUSP15	assignmentsChromaffin_cells.Chrom_SOX2_statusHigh
assignmentsChromaffin_cells.Chrom_SOX2_statusHigh.PEX14	-0.7473297	5.58625587	-3.2067086	0.00198394	0.00683534	-1.9143399	PEX14	assignmentsChromaffin_cells.Chrom_SOX2_statusHigh
assignmentsChromaffin_cells.Chrom_SOX2_statusHigh.TRIB2	2.14656045	3.02354212	3.11548557	0.00261536	0.00860943	-1.9144619	TRIB2	assignmentsChromaffin_cells.Chrom_SOX2_statusHigh
assignmentsChromaffin_cells.Chrom_SOX2_statusHigh.SFSWAP	-0.555248	6.7731875	-3.2412545	0.00178509	0.0062605	-1.9146541	SFSWAP	assignmentsChromaffin_cells.Chrom_SOX2_statusHigh
assignmentsChromaffin_cells.Chrom_SOX2_statusHigh.DUSP16	-0.9271308	7.07911053	-3.2385576	0.00179992	0.00629872	-1.9174068	DUSP16	assignmentsChromaffin_cells.Chrom_SOX2_statusHigh
assignmentsChromaffin_cells.Chrom_SOX2_statusHigh.MICU2	-0.785538	7.56481168	-3.2608847	0.00168059	0.00592258	-1.9179366	MICU2	assignmentsChromaffin_cells.Chrom_SOX2_statusHigh
assignmentsChromaffin_cells.Chrom_SOX2_statusHigh.ZNF26	-0.674998	4.98244367	-3.2052381	0.00199285	0.00686161	-1.9182725	ZNF26	assignmentsChromaffin_cells.Chrom_SOX2_statusHigh
assignmentsChromaffin_cells.Chrom_SOX2_statusHigh.CARD10	1.95718865	0.12074389	3.0041774	0.00363256	0.01138761	-1.9199825	CARD10	assignmentsChromaffin_cells.Chrom_SOX2_statusHigh
assignmentsChromaffin_cells.Chrom_SOX2_statusHigh.SIN3A	-0.5652584	5.75956323	-3.2147216	0.00193605	0.00669046	-1.9202517	SIN3A	assignmentsChromaffin_cells.Chrom_SOX2_statusHigh
assignmentsChromaffin_cells.Chrom_SOX2_statusHigh.TMPRSS11A	-2.2339638	0.28574503	-3.0294034	0.00337353	0.01070533	-1.9212389	TMPRSS11A	assignmentsChromaffin_cells.Chrom_SOX2_statusHigh
assignmentsChromaffin_cells.Chrom_SOX2_statusHigh.CCDC154	-1.1835357	2.24731147	-3.1465	0.0023809	0.00795517	-1.9215919	CCDC154	assignmentsChromaffin_cells.Chrom_SOX2_statusHigh
assignmentsChromaffin_cells.Chrom_SOX2_statusHigh.BEND7	-0.7872354	5.2696098	-3.2228361	0.00188866	0.00655209	-1.9222137	BEND7	assignmentsChromaffin_cells.Chrom_SOX2_statusHigh
assignmentsChromaffin_cells.Chrom_SOX2_statusHigh.CYP51A1	0.79530305	5.72544491	3.23588042	0.00181475	0.00633956	-1.9247829	CYP51A1	assignmentsChromaffin_cells.Chrom_SOX2_statusHigh
assignmentsChromaffin_cells.Chrom_SOX2_statusHigh.AGAP5	-0.8023169	3.08479823	-3.1524263	0.00233876	0.00782755	-1.9281424	AGAP5	assignmentsChromaffin_cells.Chrom_SOX2_statusHigh

assignmentsChromaffin_cells.Chrom_SOX2_statusHigh.TDRKH-AS1	1.16445362	1.11203044	3.13727945	0.00244787	0.00815177	-1.9321712	TDRKH-AS1	assignmentsChromaffin_cells.Chrom_SOX2_statusHigh
assignmentsChromaffin_cells.Chrom_SOX2_statusHigh.CPT1B	-1.0484363	3.00479484	-3.1544081	0.00232482	0.00778891	-1.9328735	CPT1B	assignmentsChromaffin_cells.Chrom_SOX2_statusHigh
assignmentsChromaffin_cells.Chrom_SOX2_statusHigh.CTNNA2	-1.9914805	8.06027348	-3.2713717	0.00162888	0.005772	-1.9330367	CTNNA2	assignmentsChromaffin_cells.Chrom_SOX2_statusHigh
assignmentsChromaffin_cells.Chrom_SOX2_statusHigh.IFIT2	1.69425008	2.96741762	3.11891595	0.00258721	0.00852546	-1.9348532	IFIT2	assignmentsChromaffin_cells.Chrom_SOX2_statusHigh
assignmentsChromaffin_cells.Chrom_SOX2_statusHigh.LINC00364	2.53289554	-0.9885352	3.01066356	0.00356497	0.01121517	-1.9354484	LINC00364	assignmentsChromaffin_cells.Chrom_SOX2_statusHigh
assignmentsChromaffin_cells.Chrom_SOX2_statusHigh.DCAF16	-0.5138276	5.35821925	-3.2171328	0.00192185	0.00665071	-1.9356741	DCAF16	assignmentsChromaffin_cells.Chrom_SOX2_statusHigh
assignmentsChromaffin_cells.Chrom_SOX2_statusHigh.KANSL1	-0.8742648	8.61860047	-3.2588363	0.00169122	0.00595733	-1.9360601	KANSL1	assignmentsChromaffin_cells.Chrom_SOX2_statusHigh
assignmentsChromaffin_cells.Chrom_SOX2_statusHigh.TRIM56	0.7963264	5.5610468	3.21151209	0.0019551	0.00674757	-1.9361233	TRIM56	assignmentsChromaffin_cells.Chrom_SOX2_statusHigh
assignmentsChromaffin_cells.Chrom_SOX2_statusHigh.LRRC7	-2.3558543	3.04813805	-3.1278423	0.00252056	0.00835567	-1.9367302	LRRC7	assignmentsChromaffin_cells.Chrom_SOX2_statusHigh
assignmentsChromaffin_cells.Chrom_SOX2_statusHigh.TSPAN13	1.50225123	3.50487271	3.19877298	0.00203368	0.00698417	-1.9382859	TSPAN13	assignmentsChromaffin_cells.Chrom_SOX2_statusHigh
assignmentsChromaffin_cells.Chrom_SOX2_statusHigh.MARF1	-0.4913401	5.76225862	-3.2099057	0.0019647	0.00677779	-1.9400742	MARF1	assignmentsChromaffin_cells.Chrom_SOX2_statusHigh
assignmentsChromaffin_cells.Chrom_SOX2_statusHigh.IRF1	1.66669908	4.95577338	3.17273085	0.00220121	0.00744004	-1.9414101	IRF1	assignmentsChromaffin_cells.Chrom_SOX2_statusHigh
assignmentsChromaffin_cells.Chrom_SOX2_statusHigh.PPP1R37	0.90846418	2.98260757	3.17102519	0.002211	0.00746542	-1.9417058	PPP1R37	assignmentsChromaffin_cells.Chrom_SOX2_statusHigh
assignmentsChromaffin_cells.Chrom_SOX2_statusHigh.PPP2R5D	-0.7232574	3.62118775	-3.1635327	0.00226166	0.00760589	-1.9422126	PPP2R5D	assignmentsChromaffin_cells.Chrom_SOX2_statusHigh
assignmentsChromaffin_cells.Chrom_SOX2_statusHigh.CINP	0.70357554	3.36953289	3.17832004	0.00216269	0.00733148	-1.9424494	CINP	assignmentsChromaffin_cells.Chrom_SOX2_statusHigh
assignmentsChromaffin_cells.Chrom_SOX2_statusHigh.RABIF	0.8730277	3.49807244	3.17424853	0.00218953	0.0074068	-1.9431155	RABIF	assignmentsChromaffin_cells.Chrom_SOX2_statusHigh
assignmentsChromaffin_cells.Chrom_SOX2_statusHigh.NAP1L2	1.16175682	1.79172889	3.15776672	0.00230138	0.00772167	-1.9432867	NAP1L2	assignmentsChromaffin_cells.Chrom_SOX2_statusHigh
assignmentsChromaffin_cells.Chrom_SOX2_statusHigh.TXLNA	0.64338687	4.7286714	3.19084317	0.00208206	0.00712079	-1.9443551	TXLNA	assignmentsChromaffin_cells.Chrom_SOX2_statusHigh
assignmentsChromaffin_cells.Chrom_SOX2_statusHigh.CLDND2	1.60011903	1.49684643	3.10620207	0.00268678	0.00880657	-1.9457123	CLDND2	assignmentsChromaffin_cells.Chrom_SOX2_statusHigh
assignmentsChromaffin_cells.Chrom_SOX2_statusHigh.C3orf52	1.97592682	0.58902222	3.00992406	0.00357198	0.01123503	-1.9463708	C3orf52	assignmentsChromaffin_cells.Chrom_SOX2_statusHigh
assignmentsChromaffin_cells.Chrom_SOX2_statusHigh.ZNF382	-0.9467798	3.97072379	-3.1866887	0.00210849	0.00718266	-1.9472692	ZNF382	assignmentsChromaffin_cells.Chrom_SOX2_statusHigh
assignmentsChromaffin_cells.Chrom_SOX2_statusHigh.PIGO	0.59455717	2.83483259	3.15587019	0.00231459	0.00775949	-1.9490565	PIGO	assignmentsChromaffin_cells.Chrom_SOX2_statusHigh
assignmentsChromaffin_cells.Chrom_SOX2_statusHigh.CSRNP1	1.6812772	4.4356786	3.17941309	0.00215755	0.00731869	-1.9502216	CSRNP1	assignmentsChromaffin_cells.Chrom_SOX2_statusHigh
assignmentsChromaffin_cells.Chrom_SOX2_statusHigh.DCBLD2	0.85276658	5.42407836	3.21602064	0.00192839	0.00666973	-1.9546726	DCBLD2	assignmentsChromaffin_cells.Chrom_SOX2_statusHigh
assignmentsChromaffin_cells.Chrom_SOX2_statusHigh.PRPF18	-0.7826187	5.98141207	-3.2129297	0.00194667	0.00672113	-1.9570703	PRPF18	assignmentsChromaffin_cells.Chrom_SOX2_statusHigh
assignmentsChromaffin_cells.Chrom_SOX2_statusHigh.FBXO38	-0.6982794	5.84842777	-3.2048273	0.00199535	0.00686725	-1.957133	FBXO38	assignmentsChromaffin_cells.Chrom_SOX2_statusHigh
assignmentsChromaffin_cells.Chrom_SOX2_statusHigh.LDHC	2.45442062	0.50381838	3.0377643	0.00329269	0.01048822	-1.9576099	LDHC	assignmentsChromaffin_cells.Chrom_SOX2_statusHigh
assignmentsChromaffin_cells.Chrom_SOX2_statusHigh.CSNK1G3	-0.5176002	6.72402686	-3.2230242	0.00188757	0.00654974	-1.96074	CSNK1G3	assignmentsChromaffin_cells.Chrom_SOX2_statusHigh
assignmentsChromaffin_cells.Chrom_SOX2_statusHigh.SNAP29	0.65760687	4.35578366	3.18470794	0.0021212	0.00722136	-1.9618726	SNAP29	assignmentsChromaffin_cells.Chrom_SOX2_statusHigh
assignmentsChromaffin_cells.Chrom_SOX2_statusHigh.ST3GAL4	1.02702379	3.98063824	3.16358952	0.00226127	0.00760589	-1.9623158	ST3GAL4	assignmentsChromaffin_cells.Chrom_SOX2_statusHigh
assignmentsChromaffin_cells.Chrom_SOX2_statusHigh.ARHGAP44	-1.1838569	5.30445453	-3.2075427	0.0019794	0.00682407	-1.9626905	ARHGAP44	assignmentsChromaffin_cells.Chrom_SOX2_statusHigh
assignmentsChromaffin_cells.Chrom_SOX2_statusHigh.F10	3.00649295	1.65812194	3.11918872	0.00258678	0.00852546	-1.9631585	F10	assignmentsChromaffin_cells.Chrom_SOX2_statusHigh
assignmentsChromaffin_cells.Chrom_SOX2_statusHigh.LGALS9	1.60282599	2.59083222	3.05073045	0.00316803	0.01015569	-1.9654316	LGALS9	assignmentsChromaffin_cells.Chrom_SOX2_statusHigh
assignmentsChromaffin_cells.Chrom_SOX2_statusHigh.TMEM86A	1.21038115	0.75699621	3.03876471	0.00328185	0.01045992	-1.9659799	TMEM86A	assignmentsChromaffin_cells.Chrom_SOX2_statusHigh
assignmentsChromaffin_cells.Chrom_SOX2_statusHigh.OGFOD2	0.85768806	2.3576871	3.14734965	0.00237481	0.00793814	-1.9679791	OGFOD2	assignmentsChromaffin_cells.Chrom_SOX2_statusHigh
assignmentsChromaffin_cells.Chrom_SOX2_statusHigh.LRRC40	-0.5985671	4.82490679	-3.1744503	0.00218819	0.00740384	-1.9710002	LRRC40	assignmentsChromaffin_cells.Chrom_SOX2_statusHigh
assignmentsChromaffin_cells.Chrom_SOX2_statusHigh.SNX25	-0.7625806	6.45359805	-3.2063916	0.00198586	0.006839	-1.9747924	SNX25	assignmentsChromaffin_cells.Chrom_SOX2_statusHigh
assignmentsChromaffin_cells.Chrom_SOX2_statusHigh.RALGAPA2	-0.9763234	7.18990335	-3.2141278	0.00193956	0.0066997	-1.9762061	RALGAPA2	assignmentsChromaffin_cells.Chrom_SOX2_statusHigh
assignmentsChromaffin_cells.Chrom_SOX2_statusHigh.TSBP1	-2.2529008	-0.2136818	-2.9664878	0.00405413	0.01250627	-1.9777078	TSBP1	assignmentsChromaffin_cells.Chrom_SOX2_statusHigh
assignmentsChromaffin_cells.Chrom_SOX2_statusHigh.CARNMT1	-0.6900246	4.98544712	-3.181475	0.00214211	0.00727862	-1.978035	CARNMT1	assignmentsChromaffin_cells.Chrom_SOX2_statusHigh
assignmentsChromaffin_cells.Chrom_SOX2_statusHigh.FAM89B	1.63189438	2.5239749	3.12960311	0.00250593	0.00831578	-1.9785134	FAM89B	assignmentsChromaffin_cells.Chrom_SOX2_statusHigh
assignmentsChromaffin_cells.Chrom_SOX2_statusHigh.SLC7A6	-0.6833878	5.3442878	-3.1849407	0.0021197	0.0072178	-1.9791926	SLC7A6	assignmentsChromaffin_cells.Chrom_SOX2_statusHigh
assignmentsChromaffin_cells.Chrom_SOX2_statusHigh.SLC9A2	-1.5766533	1.83776246	-3.1209402	0.00257157	0.00848514	-1.9795586	SLC9A2	assignmentsChromaffin_cells.Chrom_SOX2_statusHigh
assignmentsChromaffin_cells.Chrom_SOX2_statusHigh.INO80-AS1	-2.5152037	0.41053017	-3.0087605	0.00358526	0.01127458	-1.9797942	INO80-AS1	assignmentsChromaffin_cells.Chrom_SOX2_statusHigh
assignmentsChromaffin_cells.Chrom_SOX2_statusHigh.MVB12B	-1.2764434	6.15638841	-3.190526	0.00208466	0.0071257	-1.9798605	MVB12B	assignmentsChromaffin_cells.Chrom_SOX2_statusHigh
assignmentsChromaffin_cells.Chrom_SOX2_statusHigh.ANAPC16	0.61360273	6.68734093	3.22840689	0.00185676	0.00646098	-1.9801556	ANAPC16	assignmentsChromaffin_cells.Chrom_SOX2_statusHigh
assignmentsChromaffin_cells.Chrom_SOX2_statusHigh.NEMF	-0.5298955	7.35256631	-3.2273747	0.00186263	0.0064786	-1.9802083	NEMF	assignmentsChromaffin_cells.Chrom_SOX2_statusHigh
assignmentsChromaffin_cells.Chrom_SOX2_statusHigh.CMAS	0.84317029	4.65579081	3.19684077	0.00204445	0.00701665	-1.9828585	CMAS	assignmentsChromaffin_cells.Chrom_SOX2_statusHigh
assignmentsChromaffin_cells.Chrom_SOX2_statusHigh.PPIL1	0.87524738	2.82524777	3.14662306	0.00238001	0.00795388	-1.984599	PPIL1	assignmentsChromaffin_cells.Chrom_SOX2_statusHigh
assignmentsChromaffin_cells.Chrom_SOX2_statusHigh.ZNF177	-0.8628578	3.28866724	-3.1514309	0.00234579	0.00784603	-1.984702	ZNF177	assignmentsChromaffin_cells.Chrom_SOX2_statusHigh
assignmentsChromaffin_cells.Chrom_SOX2_statusHigh.EVI2B	-2.4443561	2.94476718	-3.0621231	0.00306572	0.00989098	-1.9847852	EVI2B	assignmentsChromaffin_cells.Chrom_SOX2_statusHigh
assignmentsChromaffin_cells.Chrom_SOX2_statusHigh.MIA3	0.59173249	6.39498978	3.219141	0.0019101	0.00661219	-1.9848419	MIA3	assignmentsChromaffin_cells.Chrom_SOX2_statusHigh
assignmentsChromaffin_cells.Chrom_SOX2_statusHigh.BCAP29	0.59690167	6.16261739	3.2128698	0.00194702	0.00672113	-1.9857985	BCAP29	assignmentsChromaffin_cells.Chrom_SOX2_statusHigh
assignmentsChromaffin_cells.Chrom_SOX2_statusHigh.DUSP2	3.4085216	2.15363019	3.03289895	0.0033418	0.01061514	-1.987447	DUSP2	assignmentsChromaffin_cells.Chrom_SOX2_statusHigh
assignmentsChromaffin_cells.Chrom_SOX2_statusHigh.IGBP1	0.75768441	5.09488084	3.1900556	0.00208704	0.00713081	-1.9888663	IGBP1	assignmentsChromaffin_cells.Chrom_SOX2_statusHigh
assignmentsChromaffin_cells.Chrom_SOX2_statusHigh.LINC02649	-2.214543	3.41423715	-3.0031858	0.00364312	0.01141406	-1.9891464	LINC02649	assignmentsChromaffin_cells.Chrom_SOX2_statusHigh
assignmentsChromaffin_cells.Chrom_SOX2_statusHigh.PNPLA7	-1.1375596	3.90818998	-3.157833	0.00230092	0.00772167	-1.9892041	PNPLA7	assignmentsChromaffin_cells.Chrom_SOX2_statusHigh
assignmentsChromaffin_cells.Chrom_SOX2_statusHigh.LIMS2	1.54192004	2.40240991	3.04333481	0.00323794	0.01035288	-1.9911678	LIMS2	assignmentsChromaffin_cells.Chrom_SOX2_statusHigh
assignmentsChromaffin_cells.Chrom_SOX2_statusHigh.LDLRAD4	-1.126854	9.01456722	-3.2403877	0.00179054	0.0062755	-1.9940879	LDLRAD4	assignmentsChromaffin_cells.Chrom_SOX2_statusHigh
assignmentsChromaffin_cells.Chrom_SOX2_statusHigh.CNGA4	1.83212674	0.11661357	2.99155553	0.00376899	0.01174184	-1.9946082	CNGA4	assignmentsChromaffin_cells.Chrom_SOX2_statusHigh
assignmentsChromaffin_cells.Chrom_SOX2_statusHigh.LIFR-AS1	1.09673831	1.67063725	3.12225835	0.00256075	0.0084557	-1.9951646	LIFR-AS1	assignmentsChromaffin_cells.Chrom_SOX2_statusHigh
assignmentsChromaffin_cells.Chrom_SOX2_statusHigh.LRRTM1	-1.746535	0.35572126	-3.0290203	0.00337733	0.01071315	-1.9961655	LRRTM1	assignmentsChromaffin_cells.Chrom_SOX2_statusHigh
assignmentsChromaffin_cells.Chrom_SOX2_statusHigh.VSIG10	-1.08271	4.13868771	-3.1540217	0.00232753	0.00779636	-1.9975062	VSIG10	assignmentsChromaffin_cells.Chrom_SOX2_statusHigh
assignmentsChromaffin_cells.Chrom_SOX2_statusHigh.BIVM	-0.8602681	4.75309522	-3.1797132	0.00215358	0.00730831	-1.999713	BIVM	assignmentsChromaffin_cells.Chrom_SOX2_statusHigh

assignmentsChromaffin_cells.Chrom_SOX2_statusHigh.LYRM9	0.62626818	3.37210649	3.16336427	0.00226281	0.00760816	-2.0009319	LYRM9	assignmentsChromaffin_cells.Chrom_SOX2_statusHigh
assignmentsChromaffin_cells.Chrom_SOX2_statusHigh.TIMP2	2.03255168	5.74271128	3.1893979	0.00209328	0.00714755	-2.0017071	TIMP2	assignmentsChromaffin_cells.Chrom_SOX2_statusHigh
assignmentsChromaffin_cells.Chrom_SOX2_statusHigh.EGFL7	2.46175232	3.44565757	3.10569008	0.00269331	0.00882439	-2.004225	EGFL7	assignmentsChromaffin_cells.Chrom_SOX2_statusHigh
assignmentsChromaffin_cells.Chrom_SOX2_statusHigh.HSPA13	1.05818332	4.90848999	3.19671495	0.00204523	0.00701784	-2.0046482	HSPA13	assignmentsChromaffin_cells.Chrom_SOX2_statusHigh
assignmentsChromaffin_cells.Chrom_SOX2_statusHigh.DDX24	0.95900665	7.7601319	3.23592015	0.00181453	0.00633956	-2.0047807	DDX24	assignmentsChromaffin_cells.Chrom_SOX2_statusHigh
assignmentsChromaffin_cells.Chrom_SOX2_statusHigh.ZNF343	-0.8076005	3.74216737	-3.1462864	0.00238243	0.00795863	-2.0056385	ZNF343	assignmentsChromaffin_cells.Chrom_SOX2_statusHigh
assignmentsChromaffin_cells.Chrom_SOX2_statusHigh.SET	1.35645779	5.60844142	3.1942143	0.00206169	0.00706225	-2.0062791	SET	assignmentsChromaffin_cells.Chrom_SOX2_statusHigh
assignmentsChromaffin_cells.Chrom_SOX2_statusHigh.LINC00685	-1.2093927	3.1620272	-3.1252915	0.00253757	0.00839992	-2.006329	LINC00685	assignmentsChromaffin_cells.Chrom_SOX2_statusHigh
assignmentsChromaffin_cells.Chrom_SOX2_statusHigh.TTC21B-AS1	-2.0162166	0.68643934	-3.0226819	0.00344109	0.01088738	-2.0069762	TTC21B-AS1	assignmentsChromaffin_cells.Chrom_SOX2_statusHigh
assignmentsChromaffin_cells.Chrom_SOX2_statusHigh.KRBA1	-0.7321829	2.69741876	-3.126541	0.00252808	0.00837715	-2.0077681	KRBA1	assignmentsChromaffin_cells.Chrom_SOX2_statusHigh
assignmentsChromaffin_cells.Chrom_SOX2_statusHigh.NKTR	-0.6280233	8.1807947	-3.2280193	0.00185896	0.00646724	-2.0077727	NKTR	assignmentsChromaffin_cells.Chrom_SOX2_statusHigh
assignmentsChromaffin_cells.Chrom_SOX2_statusHigh.ALG13	-0.6170769	7.64110586	-3.2203915	0.00190282	0.00659124	-2.0099862	ALG13	assignmentsChromaffin_cells.Chrom_SOX2_statusHigh
assignmentsChromaffin_cells.Chrom_SOX2_statusHigh.EIF2B5	0.72796871	4.48816042	3.17512401	0.00218373	0.00739187	-2.0105513	EIF2B5	assignmentsChromaffin_cells.Chrom_SOX2_statusHigh
assignmentsChromaffin_cells.Chrom_SOX2_statusHigh.LIPI	-2.5271835	-0.200507	-2.9617653	0.00411003	0.01264953	-2.0132239	LIPI	assignmentsChromaffin_cells.Chrom_SOX2_statusHigh
assignmentsChromaffin_cells.Chrom_SOX2_statusHigh.GNAI2	1.26661385	5.8030487	3.18233207	0.00213716	0.00726951	-2.0139063	GNAI2	assignmentsChromaffin_cells.Chrom_SOX2_statusHigh
assignmentsChromaffin_cells.Chrom_SOX2_statusHigh.SNRK	-0.7240086	6.86193245	-3.1909507	0.00208138	0.00712055	-2.0139484	SNRK	assignmentsChromaffin_cells.Chrom_SOX2_statusHigh
assignmentsChromaffin_cells.Chrom_SOX2_statusHigh.MIR497HG	1.72172544	0.3199734	2.9800721	0.00389722	0.01208733	-2.0144099	MIR497HG	assignmentsChromaffin_cells.Chrom_SOX2_statusHigh
assignmentsChromaffin_cells.Chrom_SOX2_statusHigh.ZNF530	0.81437139	1.64741113	3.10077384	0.00273067	0.00892672	-2.0157345	ZNF530	assignmentsChromaffin_cells.Chrom_SOX2_statusHigh
assignmentsChromaffin_cells.Chrom_SOX2_statusHigh.SMYD2	-1.058222	5.64223903	-3.1928958	0.00206911	0.00708312	-2.0158996	SMYD2	assignmentsChromaffin_cells.Chrom_SOX2_statusHigh
assignmentsChromaffin_cells.Chrom_SOX2_statusHigh.ANKEF1	1.14410794	1.43110794	3.12351872	0.0025511	0.00843424	-2.0162205	ANKEF1	assignmentsChromaffin_cells.Chrom_SOX2_statusHigh
assignmentsChromaffin_cells.Chrom_SOX2_statusHigh.TMEM260	-0.7522486	5.8067779	-3.1862663	0.00211119	0.00719035	-2.0182752	TMEM260	assignmentsChromaffin_cells.Chrom_SOX2_statusHigh
assignmentsChromaffin_cells.Chrom_SOX2_statusHigh.MSI2	-0.7724345	8.96539788	-3.2482934	0.00174694	0.00613206	-2.0200696	MSI2	assignmentsChromaffin_cells.Chrom_SOX2_statusHigh
assignmentsChromaffin_cells.Chrom_SOX2_statusHigh.HSD11B1L	0.98396332	2.51533438	3.14329098	0.00240401	0.00802069	-2.0208247	HSD11B1L	assignmentsChromaffin_cells.Chrom_SOX2_statusHigh
assignmentsChromaffin_cells.Chrom_SOX2_statusHigh.DCAF6	-0.5537146	7.14558997	-3.2089778	0.00197027	0.00679553	-2.0234113	DCAF6	assignmentsChromaffin_cells.Chrom_SOX2_statusHigh
assignmentsChromaffin_cells.Chrom_SOX2_statusHigh.PIP4P1	0.8598648	4.29778475	3.16671596	0.00224	0.00754413	-2.0244546	PIP4P1	assignmentsChromaffin_cells.Chrom_SOX2_statusHigh
assignmentsChromaffin_cells.Chrom_SOX2_statusHigh.ZNF793-AS1	1.00118051	0.88390842	3.08598215	0.0028537	0.00928009	-2.0245297	ZNF793-AS1	assignmentsChromaffin_cells.Chrom_SOX2_statusHigh
assignmentsChromaffin_cells.Chrom_SOX2_statusHigh.IDH3A	0.58685527	4.85798073	3.18108775	0.00214462	0.00728254	-2.0260468	IDH3A	assignmentsChromaffin_cells.Chrom_SOX2_statusHigh
assignmentsChromaffin_cells.Chrom_SOX2_statusHigh.TMEM30B	1.95784492	-0.5653699	2.94567908	0.00430586	0.01317637	-2.0264073	TMEM30B	assignmentsChromaffin_cells.Chrom_SOX2_statusHigh
assignmentsChromaffin_cells.Chrom_SOX2_statusHigh.CAPN10	-0.6990236	3.49433984	-3.1328304	0.00248081	0.00824606	-2.0266058	CAPN10	assignmentsChromaffin_cells.Chrom_SOX2_statusHigh
assignmentsChromaffin_cells.Chrom_SOX2_statusHigh.PHIP	-0.5586226	8.15924902	-3.2249721	0.00187637	0.00651649	-2.0296045	PHIP	assignmentsChromaffin_cells.Chrom_SOX2_statusHigh
assignmentsChromaffin_cells.Chrom_SOX2_statusHigh.PLK3	1.22666517	3.56514332	3.08278477	0.00288097	0.00936081	-2.0299621	PLK3	assignmentsChromaffin_cells.Chrom_SOX2_statusHigh
assignmentsChromaffin_cells.Chrom_SOX2_statusHigh.POLR3D	0.77021343	4.1037435	3.15201104	0.00234169	0.00783559	-2.0348181	POLR3D	assignmentsChromaffin_cells.Chrom_SOX2_statusHigh
assignmentsChromaffin_cells.Chrom_SOX2_statusHigh.PPM1D	-0.7229995	6.16489485	-3.1794366	0.00215538	0.00731289	-2.0362135	PPM1D	assignmentsChromaffin_cells.Chrom_SOX2_statusHigh
assignmentsChromaffin_cells.Chrom_SOX2_statusHigh.PRKAR1B	-1.0233248	4.71411662	-3.1534297	0.00233169	0.00780866	-2.0370571	PRKAR1B	assignmentsChromaffin_cells.Chrom_SOX2_statusHigh
assignmentsChromaffin_cells.Chrom_SOX2_statusHigh.BRSK2	-1.0722718	2.58528297	-3.1260094	0.00253212	0.00838359	-2.0397259	BRSK2	assignmentsChromaffin_cells.Chrom_SOX2_statusHigh
assignmentsChromaffin_cells.Chrom_SOX2_statusHigh.USP18	1.95398667	1.38491525	3.01423987	0.00352712	0.01111136	-2.0398912	USP18	assignmentsChromaffin_cells.Chrom_SOX2_statusHigh
assignmentsChromaffin_cells.Chrom_SOX2_statusHigh.PHF2	-0.6923684	5.16122076	-3.1600143	0.00228582	0.00767705	-2.0400361	PHF2	assignmentsChromaffin_cells.Chrom_SOX2_statusHigh
assignmentsChromaffin_cells.Chrom_SOX2_statusHigh.HEPACAM2	2.29012688	-0.6691274	2.99301095	0.00375388	0.01170616	-2.0401191	HEPACAM2	assignmentsChromaffin_cells.Chrom_SOX2_statusHigh
assignmentsChromaffin_cells.Chrom_SOX2_statusHigh.PTP4A2	1.35315134	5.28322562	3.17101671	0.00221195	0.0074669	-2.0404526	PTP4A2	assignmentsChromaffin_cells.Chrom_SOX2_statusHigh
assignmentsChromaffin_cells.Chrom_SOX2_statusHigh.TMEM250	0.89249047	2.09288564	3.09876589	0.00274708	0.00897121	-2.0418656	TMEM250	assignmentsChromaffin_cells.Chrom_SOX2_statusHigh
assignmentsChromaffin_cells.Chrom_SOX2_statusHigh.C21orf62	-1.6269232	1.14514411	-3.0473619	0.0031997	0.0102449	-2.0424247	C21orf62	assignmentsChromaffin_cells.Chrom_SOX2_statusHigh
assignmentsChromaffin_cells.Chrom_SOX2_statusHigh.NBDY	0.56560124	6.62717078	3.20218111	0.00201149	0.00691539	-2.0432532	NBDY	assignmentsChromaffin_cells.Chrom_SOX2_statusHigh
assignmentsChromaffin_cells.Chrom_SOX2_statusHigh.NOA1	0.79278879	3.37504724	3.1327148	0.00248167	0.00824721	-2.0475067	NOA1	assignmentsChromaffin_cells.Chrom_SOX2_statusHigh
assignmentsChromaffin_cells.Chrom_SOX2_statusHigh.EIF3H	0.75722117	7.00088074	3.20437843	0.00199808	0.00687517	-2.0480331	EIF3H	assignmentsChromaffin_cells.Chrom_SOX2_statusHigh
assignmentsChromaffin_cells.Chrom_SOX2_statusHigh.PSMA1	0.80250221	6.66081537	3.19388879	0.00206288	0.0070648	-2.0483791	PSMA1	assignmentsChromaffin_cells.Chrom_SOX2_statusHigh
assignmentsChromaffin_cells.Chrom_SOX2_statusHigh.TAC4	-2.3015461	0.83335998	-2.9846325	0.0038467	0.01195375	-2.0529698	TAC4	assignmentsChromaffin_cells.Chrom_SOX2_statusHigh
assignmentsChromaffin_cells.Chrom_SOX2_statusHigh.OSBPL6	-1.10666	5.85105805	-3.1892201	0.00209268	0.00714701	-2.0546696	OSBPL6	assignmentsChromaffin_cells.Chrom_SOX2_statusHigh
assignmentsChromaffin_cells.Chrom_SOX2_statusHigh.HNRNPR	0.51771858	6.66358834	3.19048346	0.00208433	0.0071257	-2.0560006	HNRNPR	assignmentsChromaffin_cells.Chrom_SOX2_statusHigh
assignmentsChromaffin_cells.Chrom_SOX2_statusHigh.FDPS	0.65608062	5.27735445	3.17181962	0.00220569	0.0074536	-2.056526	FDPS	assignmentsChromaffin_cells.Chrom_SOX2_statusHigh
assignmentsChromaffin_cells.Chrom_SOX2_statusHigh.MT-ND4L	2.85753962	6.91608043	3.20535431	0.00199415	0.00686459	-2.0605553	MT-ND4L	assignmentsChromaffin_cells.Chrom_SOX2_statusHigh
assignmentsChromaffin_cells.Chrom_SOX2_statusHigh.UBALD2	1.47514283	3.56159327	3.10187565	0.00272236	0.00890318	-2.0606325	UBALD2	assignmentsChromaffin_cells.Chrom_SOX2_statusHigh
assignmentsChromaffin_cells.Chrom_SOX2_statusHigh.ESR2	-1.2152006	3.15751854	-3.0936392	0.00278938	0.00909456	-2.060742	ESR2	assignmentsChromaffin_cells.Chrom_SOX2_statusHigh
assignmentsChromaffin_cells.Chrom_SOX2_statusHigh.SAMD9L	2.20913766	4.47373194	3.06822713	0.00301081	0.00973534	-2.061806	SAMD9L	assignmentsChromaffin_cells.Chrom_SOX2_statusHigh
assignmentsChromaffin_cells.Chrom_SOX2_statusHigh.PRRG2	1.44230464	0.80611887	3.02481591	0.00341932	0.01083134	-2.0618152	PRRG2	assignmentsChromaffin_cells.Chrom_SOX2_statusHigh
assignmentsChromaffin_cells.Chrom_SOX2_statusHigh.RAMP2	1.52213571	1.91416489	3.04310206	0.00324016	0.01035586	-2.0626434	RAMP2	assignmentsChromaffin_cells.Chrom_SOX2_statusHigh
assignmentsChromaffin_cells.Chrom_SOX2_statusHigh.CCNI	0.99308646	7.05233038	3.20068805	0.00202066	0.00694393	-2.0640931	CCNI	assignmentsChromaffin_cells.Chrom_SOX2_statusHigh
assignmentsChromaffin_cells.Chrom_SOX2_statusHigh.TWIST2	2.66940489	0.27318472	2.94157106	0.00435724	0.01331045	-2.0646138	TWIST2	assignmentsChromaffin_cells.Chrom_SOX2_statusHigh
assignmentsChromaffin_cells.Chrom_SOX2_statusHigh.PRPF4B	-0.6355171	7.39339347	-3.1931051	0.0020678	0.00708013	-2.0658128	PRPF4B	assignmentsChromaffin_cells.Chrom_SOX2_statusHigh
assignmentsChromaffin_cells.Chrom_SOX2_statusHigh.TMEM33	0.58051308	5.25572983	3.16930024	0.00222256	0.00749642	-2.0658259	TMEM33	assignmentsChromaffin_cells.Chrom_SOX2_statusHigh
assignmentsChromaffin_cells.Chrom_SOX2_statusHigh.PIGF	0.59339487	5.21703967	3.16607638	0.00224434	0.00755715	-2.0668608	PIGF	assignmentsChromaffin_cells.Chrom_SOX2_statusHigh
assignmentsChromaffin_cells.Chrom_SOX2_statusHigh.HGSNAT	-0.6655138	6.97474008	-3.1879069	0.00210071	0.00716376	-2.0675311	HGSNAT	assignmentsChromaffin_cells.Chrom_SOX2_statusHigh
assignmentsChromaffin_cells.Chrom_SOX2_statusHigh.GP6	-2.0195529	2.11150326	-3.0510558	0.00316648	0.01015477	-2.0681757	GP6	assignmentsChromaffin_cells.Chrom_SOX2_statusHigh
assignmentsChromaffin_cells.Chrom_SOX2_statusHigh.CSMD3	-4.317268	1.13872167	-2.9677239	0.00404274	0.01248314	-2.0683559	CSMD3	assignmentsChromaffin_cells.Chrom_SOX2_statusHigh
assignmentsChromaffin_cells.Chrom_SOX2_statusHigh.HMGN1	0.63565574	6.42364059	3.18736964	0.00210414	0.00717087	-2.0690325	HMGN1	assignmentsChromaffin_cells.Chrom_SOX2_statusHigh

assignmentsChromaffin_cells.Chrom_SOX2_statusHigh.CCDC146	-1.0288439	5.39835777	-3.1319258	0.00248756	0.00826164	-2.0690491	CCDC146	assignmentsChromaffin_cells.Chrom_SOX2_statusHigh
assignmentsChromaffin_cells.Chrom_SOX2_statusHigh.KIF9	1.16968569	2.8996776	3.11313585	0.00263166	0.0086542	-2.0700782	KIF9	assignmentsChromaffin_cells.Chrom_SOX2_statusHigh
assignmentsChromaffin_cells.Chrom_SOX2_statusHigh.MB	2.04835717	-0.1699127	3.0080938	0.00359201	0.01128697	-2.0713247	MB	assignmentsChromaffin_cells.Chrom_SOX2_statusHigh
assignmentsChromaffin_cells.Chrom_SOX2_statusHigh.SCAT2	-2.1706486	0.64695761	-2.9785361	0.00391498	0.01213537	-2.0720885	SCAT2	assignmentsChromaffin_cells.Chrom_SOX2_statusHigh
assignmentsChromaffin_cells.Chrom_SOX2_statusHigh.COX6A2	3.23022481	-1.2357533	2.93144674	0.00448753	0.01364044	-2.0728608	COX6A2	assignmentsChromaffin_cells.Chrom_SOX2_statusHigh
assignmentsChromaffin_cells.Chrom_SOX2_statusHigh.ACADL	-2.5938298	0.05900943	-2.9523804	0.00422418	0.01295362	-2.0740436	ACADL	assignmentsChromaffin_cells.Chrom_SOX2_statusHigh
assignmentsChromaffin_cells.Chrom_SOX2_statusHigh.SPAST	-0.6325178	5.68386155	-3.1597703	0.00228751	0.00767993	-2.0772096	SPAST	assignmentsChromaffin_cells.Chrom_SOX2_statusHigh
assignmentsChromaffin_cells.Chrom_SOX2_statusHigh.CNBD2	1.10257181	2.53018654	3.10316572	0.00271125	0.00887046	-2.0780619	CNBD2	assignmentsChromaffin_cells.Chrom_SOX2_statusHigh
assignmentsChromaffin_cells.Chrom_SOX2_statusHigh.PTGER4	2.09108826	4.16196205	3.13232665	0.00248687	0.00826107	-2.0827224	PTGER4	assignmentsChromaffin_cells.Chrom_SOX2_statusHigh
assignmentsChromaffin_cells.Chrom_SOX2_statusHigh.CUL1	-0.4610329	6.95501414	-3.1838453	0.00212676	0.00723865	-2.0832542	CUL1	assignmentsChromaffin_cells.Chrom_SOX2_statusHigh
assignmentsChromaffin_cells.Chrom_SOX2_statusHigh.ZNF205	0.86268995	2.03800204	3.09970986	0.00273935	0.00894963	-2.0834028	ZNF205	assignmentsChromaffin_cells.Chrom_SOX2_statusHigh
assignmentsChromaffin_cells.Chrom_SOX2_statusHigh.TARBP1	-0.9807678	5.97703839	-3.1682286	0.00222978	0.0075176	-2.0850868	TARBP1	assignmentsChromaffin_cells.Chrom_SOX2_statusHigh
assignmentsChromaffin_cells.Chrom_SOX2_statusHigh.CBR1	1.6353649	2.8153724	3.10393621	0.00270611	0.00885727	-2.0874096	CBR1	assignmentsChromaffin_cells.Chrom_SOX2_statusHigh
assignmentsChromaffin_cells.Chrom_SOX2_statusHigh.PTDSS1	0.70928296	4.48605702	3.12754037	0.00252052	0.00835567	-2.0877683	PTDSS1	assignmentsChromaffin_cells.Chrom_SOX2_statusHigh
assignmentsChromaffin_cells.Chrom_SOX2_statusHigh.RNPS1	0.45046155	6.00604019	3.17731341	0.0021693	0.00735076	-2.0878907	RNPS1	assignmentsChromaffin_cells.Chrom_SOX2_statusHigh
assignmentsChromaffin_cells.Chrom_SOX2_statusHigh.CATSPER2	-0.7945108	5.26134796	-3.1657406	0.00224662	0.00756323	-2.0885538	CATSPER2	assignmentsChromaffin_cells.Chrom_SOX2_statusHigh
assignmentsChromaffin_cells.Chrom_SOX2_statusHigh.CHST15	-2.8839494	4.65581715	-3.0229737	0.00344071	0.01088738	-2.0891344	CHST15	assignmentsChromaffin_cells.Chrom_SOX2_statusHigh
assignmentsChromaffin_cells.Chrom_SOX2_statusHigh.TRAF1	1.67439148	3.06974746	3.03179756	0.00334986	0.01063653	-2.0901805	TRAF1	assignmentsChromaffin_cells.Chrom_SOX2_statusHigh
assignmentsChromaffin_cells.Chrom_SOX2_statusHigh.ZNF503	1.70326626	2.40252163	3.07651089	0.00293625	0.00951345	-2.0903664	ZNF503	assignmentsChromaffin_cells.Chrom_SOX2_statusHigh
assignmentsChromaffin_cells.Chrom_SOX2_statusHigh.WDR18	1.0134219	3.17649381	3.12521836	0.00253813	0.00840003	-2.0928462	WDR18	assignmentsChromaffin_cells.Chrom_SOX2_statusHigh
assignmentsChromaffin_cells.Chrom_SOX2_statusHigh.QRICH2	-1.3559921	2.40778591	-3.0621656	0.0030627	0.00988322	-2.0930868	QRICH2	assignmentsChromaffin_cells.Chrom_SOX2_statusHigh
assignmentsChromaffin_cells.Chrom_SOX2_statusHigh.MIPOL1	-0.9499939	6.18389913	-3.1783732	0.00216234	0.00733148	-2.0937285	MIPOL1	assignmentsChromaffin_cells.Chrom_SOX2_statusHigh
assignmentsChromaffin_cells.Chrom_SOX2_statusHigh.DLG1	-0.753328	8.06924903	-3.1984794	0.00203428	0.00698476	-2.0941974	DLG1	assignmentsChromaffin_cells.Chrom_SOX2_statusHigh
assignmentsChromaffin_cells.Chrom_SOX2_statusHigh.NUP210	-1.1503165	4.52479455	-3.1378052	0.00244401	0.0081406	-2.0948093	NUP210	assignmentsChromaffin_cells.Chrom_SOX2_statusHigh
assignmentsChromaffin_cells.Chrom_SOX2_statusHigh.SMIM12	0.89156352	4.36091362	3.12642739	0.00252894	0.00837827	-2.0950226	SMIM12	assignmentsChromaffin_cells.Chrom_SOX2_statusHigh
assignmentsChromaffin_cells.Chrom_SOX2_statusHigh.POLE4	1.47901764	2.89137683	3.06670908	0.00302177	0.00976685	-2.0964736	POLE4	assignmentsChromaffin_cells.Chrom_SOX2_statusHigh
assignmentsChromaffin_cells.Chrom_SOX2_statusHigh.ZRANB1	-0.6792453	5.85754403	-3.158596	0.00229563	0.00770559	-2.0966351	ZRANB1	assignmentsChromaffin_cells.Chrom_SOX2_statusHigh
assignmentsChromaffin_cells.Chrom_SOX2_statusHigh.KLF3-AS1	-1.4569984	3.90497635	-3.1087814	0.00266692	0.00875043	-2.097358	KLF3-AS1	assignmentsChromaffin_cells.Chrom_SOX2_statusHigh
assignmentsChromaffin_cells.Chrom_SOX2_statusHigh.SEC23IP	-0.4154788	5.63600557	-3.1599637	0.00228617	0.00767705	-2.097816	SEC23IP	assignmentsChromaffin_cells.Chrom_SOX2_statusHigh
assignmentsChromaffin_cells.Chrom_SOX2_statusHigh.HHIP	-1.6059083	1.7881435	-3.0561685	0.00311819	0.01002998	-2.0987681	HHIP	assignmentsChromaffin_cells.Chrom_SOX2_statusHigh
assignmentsChromaffin_cells.Chrom_SOX2_statusHigh.TNKS	-0.6248761	8.11602601	-3.2018755	0.00201336	0.00692035	-2.1002679	TNKS	assignmentsChromaffin_cells.Chrom_SOX2_statusHigh
assignmentsChromaffin_cells.Chrom_SOX2_statusHigh.ADPRH	2.07387763	1.25518508	2.92953873	0.00451099	0.01369012	-2.1009876	ADPRH	assignmentsChromaffin_cells.Chrom_SOX2_statusHigh
assignmentsChromaffin_cells.Chrom_SOX2_statusHigh.THSD7A	2.54056306	5.37890962	3.17717722	0.00217231	0.00735941	-2.1022429	THSD7A	assignmentsChromaffin_cells.Chrom_SOX2_statusHigh
assignmentsChromaffin_cells.Chrom_SOX2_statusHigh.MAP7D1	0.79665106	4.65962627	3.12788385	0.00251792	0.00835037	-2.1044847	MAP7D1	assignmentsChromaffin_cells.Chrom_SOX2_statusHigh
assignmentsChromaffin_cells.Chrom_SOX2_statusHigh.SRP72	0.6908021	6.05809673	3.17341474	0.00219506	0.00742239	-2.1062388	SRP72	assignmentsChromaffin_cells.Chrom_SOX2_statusHigh
assignmentsChromaffin_cells.Chrom_SOX2_statusHigh.ZNF724	1.04190642	1.77954762	3.05391284	0.00313839	0.0100808	-2.1074013	ZNF724	assignmentsChromaffin_cells.Chrom_SOX2_statusHigh
assignmentsChromaffin_cells.Chrom_SOX2_statusHigh.NFATC3	-0.8217767	6.78649285	-3.1626345	0.0022678	0.00762176	-2.1093105	NFATC3	assignmentsChromaffin_cells.Chrom_SOX2_statusHigh
assignmentsChromaffin_cells.Chrom_SOX2_statusHigh.WARS2-IT1	-1.8322956	-0.1168415	-2.9255543	0.004563	0.01382346	-2.1095687	WARS2-IT1	assignmentsChromaffin_cells.Chrom_SOX2_statusHigh
assignmentsChromaffin_cells.Chrom_SOX2_statusHigh.LINC02814	1.54318941	-0.0369283	2.96514761	0.00406992	0.01254775	-2.1107102	LINC02814	assignmentsChromaffin_cells.Chrom_SOX2_statusHigh
assignmentsChromaffin_cells.Chrom_SOX2_statusHigh.POLK	-0.5344901	7.15623605	-3.1786604	0.00216046	0.00732701	-2.1111649	POLK	assignmentsChromaffin_cells.Chrom_SOX2_statusHigh
assignmentsChromaffin_cells.Chrom_SOX2_statusHigh.PLCXD3	2.93737671	4.75532342	3.16997358	0.00222019	0.00748997	-2.1125058	PLCXD3	assignmentsChromaffin_cells.Chrom_SOX2_statusHigh
assignmentsChromaffin_cells.Chrom_SOX2_statusHigh.CTDNEP1	1.03648433	3.95795961	3.12201589	0.00256261	0.00846011	-2.1141569	CTDNEP1	assignmentsChromaffin_cells.Chrom_SOX2_statusHigh
assignmentsChromaffin_cells.Chrom_SOX2_statusHigh.CYB5D1	1.22548967	1.88256027	3.05695201	0.00311031	0.01001068	-2.1162717	CYB5D1	assignmentsChromaffin_cells.Chrom_SOX2_statusHigh
assignmentsChromaffin_cells.Chrom_SOX2_statusHigh.TMIGD3	1.83303402	0.70587582	2.94522014	0.00431157	0.01319133	-2.1166961	TMIGD3	assignmentsChromaffin_cells.Chrom_SOX2_statusHigh
assignmentsChromaffin_cells.Chrom_SOX2_statusHigh.EIF4G2	1.03955968	7.04943148	3.1755938	0.00218063	0.00738449	-2.1175645	EIF4G2	assignmentsChromaffin_cells.Chrom_SOX2_statusHigh
assignmentsChromaffin_cells.Chrom_SOX2_statusHigh.KCNJ13	-1.4649272	1.62973045	-3.0329926	0.0033381	0.01060968	-2.1177155	KCNJ13	assignmentsChromaffin_cells.Chrom_SOX2_statusHigh
assignmentsChromaffin_cells.Chrom_SOX2_statusHigh.ITIH4	-1.402806	2.2955954	-3.0224161	0.00344351	0.01088859	-2.1207081	ITIH4	assignmentsChromaffin_cells.Chrom_SOX2_statusHigh
assignmentsChromaffin_cells.Chrom_SOX2_statusHigh.FGL1	2.11471838	-0.4350023	2.94199815	0.00435187	0.0132994	-2.121082	FGL1	assignmentsChromaffin_cells.Chrom_SOX2_statusHigh
assignmentsChromaffin_cells.Chrom_SOX2_statusHigh.OTUD4	-0.5629725	5.97968215	-3.146165	0.0023833	0.00795988	-2.1213281	OTUD4	assignmentsChromaffin_cells.Chrom_SOX2_statusHigh
assignmentsChromaffin_cells.Chrom_SOX2_statusHigh.TIPRL	0.53332271	5.48537539	3.15332109	0.00233246	0.00780959	-2.1237179	TIPRL	assignmentsChromaffin_cells.Chrom_SOX2_statusHigh
assignmentsChromaffin_cells.Chrom_SOX2_statusHigh.TMUB2	0.67085189	4.45241	3.13373097	0.00247411	0.00823061	-2.12373	TMUB2	assignmentsChromaffin_cells.Chrom_SOX2_statusHigh
assignmentsChromaffin_cells.Chrom_SOX2_statusHigh.RAB3B	2.23322601	1.98397344	3.10243861	0.00271958	0.00889591	-2.1240171	RAB3B	assignmentsChromaffin_cells.Chrom_SOX2_statusHigh
assignmentsChromaffin_cells.Chrom_SOX2_statusHigh.HOXD9	2.19144101	1.07898149	3.0179487	0.00349127	0.01101357	-2.1266155	HOXD9	assignmentsChromaffin_cells.Chrom_SOX2_statusHigh
assignmentsChromaffin_cells.Chrom_SOX2_statusHigh.RHBDF1	1.73240431	1.33166914	2.9441461	0.00432496	0.01322222	-2.1271856	RHBDF1	assignmentsChromaffin_cells.Chrom_SOX2_statusHigh
assignmentsChromaffin_cells.Chrom_SOX2_statusHigh.LINGO2	-2.7775356	6.4794286	-3.1642949	0.00225862	0.00760206	-2.12729	LINGO2	assignmentsChromaffin_cells.Chrom_SOX2_statusHigh
assignmentsChromaffin_cells.Chrom_SOX2_statusHigh.GIT2	-0.6380133	6.28236084	-3.1455212	0.00238792	0.007972	-2.1287434	GIT2	assignmentsChromaffin_cells.Chrom_SOX2_statusHigh
assignmentsChromaffin_cells.Chrom_SOX2_statusHigh.SLC16A2	-0.9195199	4.15686822	-3.1200774	0.00257754	0.00850064	-2.1288336	SLC16A2	assignmentsChromaffin_cells.Chrom_SOX2_statusHigh
assignmentsChromaffin_cells.Chrom_SOX2_statusHigh.TRERF1	-1.2604406	6.23026625	-3.1498012	0.00235817	0.00788581	-2.1289402	TRERF1	assignmentsChromaffin_cells.Chrom_SOX2_statusHigh
assignmentsChromaffin_cells.Chrom_SOX2_statusHigh.DOCK7	-0.832113	6.40841566	-3.1545469	0.00232385	0.00778727	-2.129878	DOCK7	assignmentsChromaffin_cells.Chrom_SOX2_statusHigh
assignmentsChromaffin_cells.Chrom_SOX2_statusHigh.CDR2	-0.6491469	6.58927385	-3.151922	0.00234232	0.00783606	-2.1304204	CDR2	assignmentsChromaffin_cells.Chrom_SOX2_statusHigh
assignmentsChromaffin_cells.Chrom_SOX2_statusHigh.CCDC196	-1.6777672	0.97737399	-2.9972504	0.00370686	0.01157977	-2.1357389	CCDC196	assignmentsChromaffin_cells.Chrom_SOX2_statusHigh
assignmentsChromaffin_cells.Chrom_SOX2_statusHigh.PLD6	-1.0232097	1.66905669	-3.0541303	0.00313637	0.01007634	-2.136072	PLD6	assignmentsChromaffin_cells.Chrom_SOX2_statusHigh
assignmentsChromaffin_cells.Chrom_SOX2_statusHigh.WNK1	-0.6675449	8.17102921	-3.1826517	0.00213447	0.00726192	-2.136622	WNK1	assignmentsChromaffin_cells.Chrom_SOX2_statusHigh
assignmentsChromaffin_cells.Chrom_SOX2_statusHigh.RARS2	-0.5210608	6.47343619	-3.1557129	0.00231569	0.00776155	-2.1372785	RARS2	assignmentsChromaffin_cells.Chrom_SOX2_statusHigh

assignmentsChromaffin_cells.Chrom_SOX2_statusHigh.TECPR2	-0.9010858	5.2522139	-3.1224526	0.00255926	0.00845252	-2.1374578	TECPR2	assignmentsChromaffin_cells.Chrom_SOX2_statusHigh
assignmentsChromaffin_cells.Chrom_SOX2_statusHigh.TMEM183A	0.62465329	4.13231935	3.11575148	0.00261114	0.00859732	-2.1379615	TMEM183A	assignmentsChromaffin_cells.Chrom_SOX2_statusHigh
assignmentsChromaffin_cells.Chrom_SOX2_statusHigh.NEXMIF	-1.3478825	5.96113362	-3.1672943	0.00223778	0.00753962	-2.1382493	NEXMIF	assignmentsChromaffin_cells.Chrom_SOX2_statusHigh
assignmentsChromaffin_cells.Chrom_SOX2_statusHigh.RABEP1	-0.6648145	7.0874086	-3.1619202	0.0022727	0.00763502	-2.1388893	RABEP1	assignmentsChromaffin_cells.Chrom_SOX2_statusHigh
assignmentsChromaffin_cells.Chrom_SOX2_statusHigh.GOLGA7B	1.09727569	2.40736181	3.10714083	0.00267925	0.00878549	-2.1406315	GOLGA7B	assignmentsChromaffin_cells.Chrom_SOX2_statusHigh
assignmentsChromaffin_cells.Chrom_SOX2_statusHigh.ZNF438	-0.7235489	6.28566567	-3.1351369	0.00246368	0.00819762	-2.1411089	ZNF438	assignmentsChromaffin_cells.Chrom_SOX2_statusHigh
assignmentsChromaffin_cells.Chrom_SOX2_statusHigh.NBR2	-0.8518595	4.5189276	-3.1187874	0.00258752	0.00852546	-2.1425558	NBR2	assignmentsChromaffin_cells.Chrom_SOX2_statusHigh
assignmentsChromaffin_cells.Chrom_SOX2_statusHigh.ADCK1	-0.9166857	3.57033222	-3.082502	0.00288339	0.00936679	-2.1433683	ADCK1	assignmentsChromaffin_cells.Chrom_SOX2_statusHigh
assignmentsChromaffin_cells.Chrom_SOX2_statusHigh.DENND6A-A	-1.60031	3.10894682	-3.0573644	0.00310733	0.0100031	-2.1437143	DENND6A-AS	assignmentsChromaffin_cells.Chrom_SOX2_statusHigh
assignmentsChromaffin_cells.Chrom_SOX2_statusHigh.DRD2	2.39125639	2.24394335	3.10535473	0.00269601	0.00883142	-2.1438304	DRD2	assignmentsChromaffin_cells.Chrom_SOX2_statusHigh
assignmentsChromaffin_cells.Chrom_SOX2_statusHigh.MALT1	-0.8681619	6.43903755	-3.1328826	0.00248042	0.00824606	-2.1440829	MALT1	assignmentsChromaffin_cells.Chrom_SOX2_statusHigh
assignmentsChromaffin_cells.Chrom_SOX2_statusHigh.ZNF814	-0.7960836	5.01246566	-3.1182503	0.00259168	0.00853675	-2.1461584	ZNF814	assignmentsChromaffin_cells.Chrom_SOX2_statusHigh
assignmentsChromaffin_cells.Chrom_SOX2_statusHigh.CHRM3	-3.8206254	4.56391364	-3.0604174	0.00308124	0.00993104	-2.1464937	CHRM3	assignmentsChromaffin_cells.Chrom_SOX2_statusHigh
assignmentsChromaffin_cells.Chrom_SOX2_statusHigh.GPRASP2	0.85100993	2.40649968	3.09974722	0.00273905	0.00894963	-2.1475454	GPRASP2	assignmentsChromaffin_cells.Chrom_SOX2_statusHigh
assignmentsChromaffin_cells.Chrom_SOX2_statusHigh.SMG9	-0.6775709	4.60082035	-3.1060218	0.00268822	0.0088095	-2.1503534	SMG9	assignmentsChromaffin_cells.Chrom_SOX2_statusHigh
assignmentsChromaffin_cells.Chrom_SOX2_statusHigh.PRCD	1.06059646	3.41952902	3.12472138	0.00254192	0.00840909	-2.1513142	PRCD	assignmentsChromaffin_cells.Chrom_SOX2_statusHigh
assignmentsChromaffin_cells.Chrom_SOX2_statusHigh.ZNF577	-0.8137379	5.05814911	-3.1241646	0.00254616	0.00841966	-2.1527975	ZNF577	assignmentsChromaffin_cells.Chrom_SOX2_statusHigh
assignmentsChromaffin_cells.Chrom_SOX2_statusHigh.ITGB1-DT	0.89741585	3.13855723	3.07385225	0.00295843	0.00957371	-2.1534276	ITGB1-DT	assignmentsChromaffin_cells.Chrom_SOX2_statusHigh
assignmentsChromaffin_cells.Chrom_SOX2_statusHigh.HSPB8	2.97408651	2.8334518	3.07941755	0.0029125	0.00944603	-2.1535563	HSPB8	assignmentsChromaffin_cells.Chrom_SOX2_statusHigh
assignmentsChromaffin_cells.Chrom_SOX2_statusHigh.PRKX-AS1	-2.8069529	0.34501378	-2.8938545	0.00499697	0.0149235	-2.1543727	PRKX-AS1	assignmentsChromaffin_cells.Chrom_SOX2_statusHigh
assignmentsChromaffin_cells.Chrom_SOX2_statusHigh.SBK1	-1.0968409	0.97867621	-3.0121008	0.00354928	0.01117458	-2.1557635	SBK1	assignmentsChromaffin_cells.Chrom_SOX2_statusHigh
assignmentsChromaffin_cells.Chrom_SOX2_statusHigh.GOLGA8B	-1.3269491	6.06455384	-3.1492338	0.00236255	0.0078988	-2.1575416	GOLGA8B	assignmentsChromaffin_cells.Chrom_SOX2_statusHigh
assignmentsChromaffin_cells.Chrom_SOX2_statusHigh.PPP1R7	0.74546346	4.66661007	3.12159845	0.00256582	0.00846895	-2.162484	PPP1R7	assignmentsChromaffin_cells.Chrom_SOX2_statusHigh
assignmentsChromaffin_cells.Chrom_SOX2_statusHigh.CCL2	2.86150706	2.08916076	2.93073379	0.004498	0.01366526	-2.163091	CCL2	assignmentsChromaffin_cells.Chrom_SOX2_statusHigh
assignmentsChromaffin_cells.Chrom_SOX2_statusHigh.RIPPLY2	1.15920489	2.0146698	3.09851922	0.0027491	0.00897339	-2.1640566	RIPPLY2	assignmentsChromaffin_cells.Chrom_SOX2_statusHigh
assignmentsChromaffin_cells.Chrom_SOX2_statusHigh.P3H4	0.86343671	3.43415891	3.10381321	0.00270601	0.00885727	-2.166867	P3H4	assignmentsChromaffin_cells.Chrom_SOX2_statusHigh
assignmentsChromaffin_cells.Chrom_SOX2_statusHigh.DCAF11	0.5651212	3.79244597	3.08953536	0.00282369	0.00919332	-2.1675939	DCAF11	assignmentsChromaffin_cells.Chrom_SOX2_statusHigh
assignmentsChromaffin_cells.Chrom_SOX2_statusHigh.LBX2-AS1	1.76939521	0.31371498	2.9314851	0.00448578	0.01363848	-2.1709494	LBX2-AS1	assignmentsChromaffin_cells.Chrom_SOX2_statusHigh
assignmentsChromaffin_cells.Chrom_SOX2_statusHigh.RASGRP1	-2.0019579	2.42612078	-2.9944644	0.00373838	0.01166235	-2.1710982	RASGRP1	assignmentsChromaffin_cells.Chrom_SOX2_statusHigh
assignmentsChromaffin_cells.Chrom_SOX2_statusHigh.GPER1	-2.148387	0.65021377	-2.9200964	0.00463515	0.01402084	-2.1726781	GPER1	assignmentsChromaffin_cells.Chrom_SOX2_statusHigh
assignmentsChromaffin_cells.Chrom_SOX2_statusHigh.PARD3	-1.0081861	7.84979196	-3.1752499	0.00218325	0.00739181	-2.1736244	PARD3	assignmentsChromaffin_cells.Chrom_SOX2_statusHigh
assignmentsChromaffin_cells.Chrom_SOX2_statusHigh.EGR4	1.99595935	0.97759901	3.05702484	0.00311231	0.01001508	-2.1745118	EGR4	assignmentsChromaffin_cells.Chrom_SOX2_statusHigh
assignmentsChromaffin_cells.Chrom_SOX2_statusHigh.LINC01637	1.29979555	0.98121602	2.97647443	0.00393822	0.01219798	-2.1746848	LINC01637	assignmentsChromaffin_cells.Chrom_SOX2_statusHigh
assignmentsChromaffin_cells.Chrom_SOX2_statusHigh.IPO8	-0.5028512	5.55619534	-3.1260655	0.00253169	0.00838359	-2.1762962	IPO8	assignmentsChromaffin_cells.Chrom_SOX2_statusHigh
assignmentsChromaffin_cells.Chrom_SOX2_statusHigh.SEC13	0.58256738	5.34142072	3.12916061	0.00250829	0.00832188	-2.1771495	SEC13	assignmentsChromaffin_cells.Chrom_SOX2_statusHigh
assignmentsChromaffin_cells.Chrom_SOX2_statusHigh.PIGC	0.92274487	4.48557087	3.1121088	0.00263976	0.00867905	-2.1791135	PIGC	assignmentsChromaffin_cells.Chrom_SOX2_statusHigh
assignmentsChromaffin_cells.Chrom_SOX2_statusHigh.RNF149	-0.7861394	7.4829901	-3.1334015	0.00247656	0.00823705	-2.1794356	RNF149	assignmentsChromaffin_cells.Chrom_SOX2_statusHigh
assignmentsChromaffin_cells.Chrom_SOX2_statusHigh.JAK2	-0.8254418	6.69783797	-3.1331356	0.00247854	0.00824192	-2.1801349	JAK2	assignmentsChromaffin_cells.Chrom_SOX2_statusHigh
assignmentsChromaffin_cells.Chrom_SOX2_statusHigh.PBX4	1.92964498	1.96415139	2.99606982	0.0037209	0.01161461	-2.1808828	PBX4	assignmentsChromaffin_cells.Chrom_SOX2_statusHigh
assignmentsChromaffin_cells.Chrom_SOX2_statusHigh.SLC9A8	-0.6858288	5.33734908	-3.1044378	0.00270097	0.00884405	-2.1820653	SLC9A8	assignmentsChromaffin_cells.Chrom_SOX2_statusHigh
assignmentsChromaffin_cells.Chrom_SOX2_statusHigh.ZNF706	0.87181611	6.04945159	3.13943128	0.00243208	0.00810425	-2.1852597	ZNF706	assignmentsChromaffin_cells.Chrom_SOX2_statusHigh
assignmentsChromaffin_cells.Chrom_SOX2_statusHigh.C4orf46	1.10314588	1.91204743	3.05286505	0.00314812	0.01010676	-2.1865518	C4orf46	assignmentsChromaffin_cells.Chrom_SOX2_statusHigh
assignmentsChromaffin_cells.Chrom_SOX2_statusHigh.KLKB1	-1.4215593	2.00285489	-3.0031839	0.00364313	0.01141406	-2.1878042	KLKB1	assignmentsChromaffin_cells.Chrom_SOX2_statusHigh
assignmentsChromaffin_cells.Chrom_SOX2_statusHigh.ETV5	1.6443705	5.55629769	3.13610096	0.00245884	0.00818322	-2.1878658	ETV5	assignmentsChromaffin_cells.Chrom_SOX2_statusHigh
assignmentsChromaffin_cells.Chrom_SOX2_statusHigh.SNAP47	0.58625952	3.87500713	3.10747637	0.00267657	0.00878027	-2.1879477	SNAP47	assignmentsChromaffin_cells.Chrom_SOX2_statusHigh
assignmentsChromaffin_cells.Chrom_SOX2_statusHigh.ACIN1	-0.6796702	7.04424483	-3.1424179	0.00241033	0.00804012	-2.1897753	ACIN1	assignmentsChromaffin_cells.Chrom_SOX2_statusHigh
assignmentsChromaffin_cells.Chrom_SOX2_statusHigh.TBPL1	0.84121976	4.31383422	3.11023804	0.00265456	0.00871345	-2.1923452	TBPL1	assignmentsChromaffin_cells.Chrom_SOX2_statusHigh
assignmentsChromaffin_cells.Chrom_SOX2_statusHigh.CTNNBL1	-0.5138044	5.96010658	-3.1231164	0.00255418	0.00844094	-2.1927996	CTNNBL1	assignmentsChromaffin_cells.Chrom_SOX2_statusHigh
assignmentsChromaffin_cells.Chrom_SOX2_statusHigh.RABGGTA	0.9900103	2.37947891	3.06142866	0.00306939	0.00990082	-2.1940543	RABGGTA	assignmentsChromaffin_cells.Chrom_SOX2_statusHigh
assignmentsChromaffin_cells.Chrom_SOX2_statusHigh.THEGL	1.49907287	1.7825303	3.05987623	0.0030844	0.00993754	-2.1948439	THEGL	assignmentsChromaffin_cells.Chrom_SOX2_statusHigh
assignmentsChromaffin_cells.Chrom_SOX2_statusHigh.LINC01891	2.12046551	-0.4215547	2.88503541	0.00512429	0.01523326	-2.195472	LINC01891	assignmentsChromaffin_cells.Chrom_SOX2_statusHigh
assignmentsChromaffin_cells.Chrom_SOX2_statusHigh.AADAT	-1.0079895	2.93805973	-3.0753849	0.002945	0.0095341	-2.1961668	AADAT	assignmentsChromaffin_cells.Chrom_SOX2_statusHigh
assignmentsChromaffin_cells.Chrom_SOX2_statusHigh.LINC02798	1.67095902	2.58118172	2.99350523	0.00374786	0.01168964	-2.1971991	LINC02798	assignmentsChromaffin_cells.Chrom_SOX2_statusHigh
assignmentsChromaffin_cells.Chrom_SOX2_statusHigh.MYO19	-0.603594	5.16034517	-3.1072259	0.00267857	0.00878505	-2.1974819	MYO19	assignmentsChromaffin_cells.Chrom_SOX2_statusHigh
assignmentsChromaffin_cells.Chrom_SOX2_statusHigh.NPIPB4	-1.0595889	4.78655996	-3.0799854	0.00290504	0.00942756	-2.197981	NPIPB4	assignmentsChromaffin_cells.Chrom_SOX2_statusHigh
assignmentsChromaffin_cells.Chrom_SOX2_statusHigh.CACNA2D3-	-2.9839202	0.20696121	-2.9275446	0.00454031	0.01376512	-2.1991748	CACNA2D3-A	assignmentsChromaffin_cells.Chrom_SOX2_statusHigh
assignmentsChromaffin_cells.Chrom_SOX2_statusHigh.SLC35F4	-3.1896742	3.02719542	-3.0326713	0.00334404	0.01062015	-2.1998161	SLC35F4	assignmentsChromaffin_cells.Chrom_SOX2_statusHigh
assignmentsChromaffin_cells.Chrom_SOX2_statusHigh.NOP53-AS1	-2.1236636	0.31871202	-2.899917	0.00491113	0.01470589	-2.203352	NOP53-AS1	assignmentsChromaffin_cells.Chrom_SOX2_statusHigh
assignmentsChromaffin_cells.Chrom_SOX2_statusHigh.HULC	-1.02703	5.25767647	-3.1133367	0.00263008	0.00865078	-2.20387	HULC	assignmentsChromaffin_cells.Chrom_SOX2_statusHigh
assignmentsChromaffin_cells.Chrom_SOX2_statusHigh.CASP8AP2	-0.5361069	5.46434952	-3.1111643	0.00264722	0.00869469	-2.2045614	CASP8AP2	assignmentsChromaffin_cells.Chrom_SOX2_statusHigh
assignmentsChromaffin_cells.Chrom_SOX2_statusHigh.TRAPPC12	-0.5633033	6.19384977	-3.1262844	0.00253003	0.00838013	-2.2052108	TRAPPC12	assignmentsChromaffin_cells.Chrom_SOX2_statusHigh
assignmentsChromaffin_cells.Chrom_SOX2_statusHigh.GSS	0.61596135	4.01794127	3.09131849	0.00280873	0.00915021	-2.2052614	GSS	assignmentsChromaffin_cells.Chrom_SOX2_statusHigh
assignmentsChromaffin_cells.Chrom_SOX2_statusHigh.SNX27	-0.508991	5.85378229	-3.1227054	0.00255732	0.0084496	-2.2072137	SNX27	assignmentsChromaffin_cells.Chrom_SOX2_statusHigh
assignmentsChromaffin_cells.Chrom_SOX2_statusHigh.DFFA	0.66894063	3.90903226	3.08746458	0.00284114	0.00924265	-2.2074368	DFFA	assignmentsChromaffin_cells.Chrom_SOX2_statusHigh

assignmentsChromaffin_cells.Chrom_SOX2_statusHigh.APOL3	2.18250014	2.70744517	2.91798229	0.00466369	0.01409383	-2.209991	APOL3	assignmentsChromaffin_cells.Chrom_SOX2_statusHigh
assignmentsChromaffin_cells.Chrom_SOX2_statusHigh.LINC00265	-1.0358123	3.51070443	-3.0482052	0.00319174	0.0102276	-2.21046	LINC00265	assignmentsChromaffin_cells.Chrom_SOX2_statusHigh
assignmentsChromaffin_cells.Chrom_SOX2_statusHigh.PTPRD	-2.6015276	6.65861136	-3.1411334	0.00242193	0.00807546	-2.2154451	PTPRD	assignmentsChromaffin_cells.Chrom_SOX2_statusHigh
assignmentsChromaffin_cells.Chrom_SOX2_statusHigh.RFX4	-1.7612479	0.02641146	-2.8886936	0.00507112	0.01509757	-2.2157646	RFX4	assignmentsChromaffin_cells.Chrom_SOX2_statusHigh
assignmentsChromaffin_cells.Chrom_SOX2_statusHigh.OARD1	0.68624195	4.30515834	3.09030945	0.00281719	0.00917588	-2.2163843	OARD1	assignmentsChromaffin_cells.Chrom_SOX2_statusHigh
assignmentsChromaffin_cells.Chrom_SOX2_statusHigh.C11orf68	1.19465736	2.18417249	3.04103688	0.00325995	0.01040459	-2.2179823	C11orf68	assignmentsChromaffin_cells.Chrom_SOX2_statusHigh
assignmentsChromaffin_cells.Chrom_SOX2_statusHigh.GPM6A	-4.188999	4.3341918	-3.0470396	0.00320545	0.01025924	-2.2199666	GPM6A	assignmentsChromaffin_cells.Chrom_SOX2_statusHigh
assignmentsChromaffin_cells.Chrom_SOX2_statusHigh.FAHD2A	-0.9216002	3.32580921	-3.0564005	0.00311539	0.01002299	-2.2204383	FAHD2A	assignmentsChromaffin_cells.Chrom_SOX2_statusHigh
assignmentsChromaffin_cells.Chrom_SOX2_statusHigh.RCCD1	0.87211261	2.72998543	3.05823008	0.00309858	0.00997691	-2.2213308	RCCD1	assignmentsChromaffin_cells.Chrom_SOX2_statusHigh
assignmentsChromaffin_cells.Chrom_SOX2_statusHigh.TMEM164	-0.7138924	5.80890149	-3.1113868	0.00264546	0.0086938	-2.2224726	TMEM164	assignmentsChromaffin_cells.Chrom_SOX2_statusHigh
assignmentsChromaffin_cells.Chrom_SOX2_statusHigh.ACER3	-0.854681	6.86194687	-3.116161	0.00260795	0.00858855	-2.2224959	ACER3	assignmentsChromaffin_cells.Chrom_SOX2_statusHigh
assignmentsChromaffin_cells.Chrom_SOX2_statusHigh.HLF	-1.0970732	4.10615128	-3.0942899	0.00278398	0.00907879	-2.2230231	HLF	assignmentsChromaffin_cells.Chrom_SOX2_statusHigh
assignmentsChromaffin_cells.Chrom_SOX2_statusHigh.NDUFS1	-0.5019909	6.51082165	-3.1316962	0.00248927	0.00826391	-2.2274678	NDUFS1	assignmentsChromaffin_cells.Chrom_SOX2_statusHigh
assignmentsChromaffin_cells.Chrom_SOX2_statusHigh.LRTM1	-2.9240077	0.66066895	-2.9608351	0.00412429	0.01268855	-2.2284389	LRTM1	assignmentsChromaffin_cells.Chrom_SOX2_statusHigh
assignmentsChromaffin_cells.Chrom_SOX2_statusHigh.LINC02608	2.28884077	0.0979626	2.90181479	0.00488532	0.01465049	-2.2321512	LINC02608	assignmentsChromaffin_cells.Chrom_SOX2_statusHigh
assignmentsChromaffin_cells.Chrom_SOX2_statusHigh.PDE7B	1.86822068	6.10670719	3.0893058	0.00282812	0.00920589	-2.2326518	PDE7B	assignmentsChromaffin_cells.Chrom_SOX2_statusHigh
assignmentsChromaffin_cells.Chrom_SOX2_statusHigh.JAZF1	-1.0010782	8.68031688	-3.1625546	0.00226889	0.00762382	-2.2329764	JAZF1	assignmentsChromaffin_cells.Chrom_SOX2_statusHigh
assignmentsChromaffin_cells.Chrom_SOX2_statusHigh.RAB38	2.05539911	1.02018073	2.87002692	0.00534789	0.01577223	-2.2331628	RAB38	assignmentsChromaffin_cells.Chrom_SOX2_statusHigh
assignmentsChromaffin_cells.Chrom_SOX2_statusHigh.WAPL	-0.5133424	7.04336519	-3.1288343	0.00251075	0.00832831	-2.2345893	WAPL	assignmentsChromaffin_cells.Chrom_SOX2_statusHigh
assignmentsChromaffin_cells.Chrom_SOX2_statusHigh.USP27X	1.0179428	1.68459114	3.03445377	0.00332378	0.01057464	-2.2347973	USP27X	assignmentsChromaffin_cells.Chrom_SOX2_statusHigh
assignmentsChromaffin_cells.Chrom_SOX2_statusHigh.ZBTB46	-1.0323898	5.68488246	-3.0887327	0.00283044	0.00921157	-2.2352353	ZBTB46	assignmentsChromaffin_cells.Chrom_SOX2_statusHigh
assignmentsChromaffin_cells.Chrom_SOX2_statusHigh.SLC2A1	1.20299759	1.99186553	3.01902912	0.00347791	0.01098222	-2.2365751	SLC2A1	assignmentsChromaffin_cells.Chrom_SOX2_statusHigh
assignmentsChromaffin_cells.Chrom_SOX2_statusHigh.BHMT	1.34183508	-0.0971262	2.95143097	0.00423487	0.0129839	-2.2368125	BHMT	assignmentsChromaffin_cells.Chrom_SOX2_statusHigh
assignmentsChromaffin_cells.Chrom_SOX2_statusHigh.C9orf24	1.37934102	1.6512636	3.03290766	0.00333894	0.01061025	-2.2384502	C9orf24	assignmentsChromaffin_cells.Chrom_SOX2_statusHigh
assignmentsChromaffin_cells.Chrom_SOX2_statusHigh.HDAC8	-0.5832912	7.60899633	-3.1404761	0.00242445	0.00808218	-2.2386629	HDAC8	assignmentsChromaffin_cells.Chrom_SOX2_statusHigh
assignmentsChromaffin_cells.Chrom_SOX2_statusHigh.CTDSPL	-1.0595386	6.08329836	-3.1065532	0.00268396	0.00879912	-2.2390274	CTDSPL	assignmentsChromaffin_cells.Chrom_SOX2_statusHigh
assignmentsChromaffin_cells.Chrom_SOX2_statusHigh.PRPF3	-0.697652	6.09643846	-3.1142249	0.0026231	0.00863136	-2.2391969	PRPF3	assignmentsChromaffin_cells.Chrom_SOX2_statusHigh
assignmentsChromaffin_cells.Chrom_SOX2_statusHigh.NETO2	-1.7124117	4.006696	-3.0384008	0.00328727	0.0104751	-2.2395868	NETO2	assignmentsChromaffin_cells.Chrom_SOX2_statusHigh
assignmentsChromaffin_cells.Chrom_SOX2_statusHigh.TMEM35A	1.85242635	1.53608863	3.04745726	0.0032015	0.01024865	-2.2412013	TMEM35A	assignmentsChromaffin_cells.Chrom_SOX2_statusHigh
assignmentsChromaffin_cells.Chrom_SOX2_statusHigh.MPV17	0.60005999	4.81446681	3.10008003	0.00273633	0.0089434	-2.2423509	MPV17	assignmentsChromaffin_cells.Chrom_SOX2_statusHigh
assignmentsChromaffin_cells.Chrom_SOX2_statusHigh.MANEAL	0.90330977	1.6054338	3.04066134	0.00326356	0.01041404	-2.2431977	MANEAL	assignmentsChromaffin_cells.Chrom_SOX2_statusHigh
assignmentsChromaffin_cells.Chrom_SOX2_statusHigh.SCARA3	2.22795284	0.62447769	2.89780684	0.0049412	0.01477937	-2.2466393	SCARA3	assignmentsChromaffin_cells.Chrom_SOX2_statusHigh
assignmentsChromaffin_cells.Chrom_SOX2_statusHigh.GGT5	2.01502331	1.56481591	2.90207222	0.00488095	0.01464011	-2.2484218	GGT5	assignmentsChromaffin_cells.Chrom_SOX2_statusHigh
assignmentsChromaffin_cells.Chrom_SOX2_statusHigh.HSPA1A	4.23358439	5.67458115	3.09204969	0.00280512	0.00914028	-2.2486757	HSPA1A	assignmentsChromaffin_cells.Chrom_SOX2_statusHigh
assignmentsChromaffin_cells.Chrom_SOX2_statusHigh.TIAM1	-1.9777808	6.03131482	-3.0987799	0.00274942	0.00897339	-2.2490681	TIAM1	assignmentsChromaffin_cells.Chrom_SOX2_statusHigh
assignmentsChromaffin_cells.Chrom_SOX2_statusHigh.TMEM243	0.70404086	4.94807806	3.08112493	0.00289522	0.0094014	-2.251193	TMEM243	assignmentsChromaffin_cells.Chrom_SOX2_statusHigh
assignmentsChromaffin_cells.Chrom_SOX2_statusHigh.GABRQ	2.42508552	0.37363073	2.96373984	0.00408972	0.01259911	-2.2538921	GABRQ	assignmentsChromaffin_cells.Chrom_SOX2_statusHigh
assignmentsChromaffin_cells.Chrom_SOX2_statusHigh.DUSP26	1.12265273	3.33355787	3.09644895	0.00276613	0.0090224	-2.2560913	DUSP26	assignmentsChromaffin_cells.Chrom_SOX2_statusHigh
assignmentsChromaffin_cells.Chrom_SOX2_statusHigh.FGD5	-2.3196349	3.17831045	-2.92298	0.00459832	0.01392256	-2.2564472	FGD5	assignmentsChromaffin_cells.Chrom_SOX2_statusHigh
assignmentsChromaffin_cells.Chrom_SOX2_statusHigh.TSNARE1	-0.7935198	4.41366715	-3.0704971	0.00298802	0.00966362	-2.2583933	TSNARE1	assignmentsChromaffin_cells.Chrom_SOX2_statusHigh
assignmentsChromaffin_cells.Chrom_SOX2_statusHigh.OAZ2	0.94829339	4.96001762	3.08787949	0.00283764	0.00923312	-2.2606716	OAZ2	assignmentsChromaffin_cells.Chrom_SOX2_statusHigh
assignmentsChromaffin_cells.Chrom_SOX2_statusHigh.SS18L1	-0.5702611	4.79293205	-3.0773418	0.00292794	0.00948844	-2.2622288	SS18L1	assignmentsChromaffin_cells.Chrom_SOX2_statusHigh
assignmentsChromaffin_cells.Chrom_SOX2_statusHigh.SLC22A1	1.35608719	1.44311069	2.95759254	0.00416002	0.01278376	-2.2626205	SLC22A1	assignmentsChromaffin_cells.Chrom_SOX2_statusHigh
assignmentsChromaffin_cells.Chrom_SOX2_statusHigh.ERICH6-AS1	0.8998603	2.25850632	3.0167724	0.00350101	0.01104212	-2.2647902	ERICH6-AS1	assignmentsChromaffin_cells.Chrom_SOX2_statusHigh
assignmentsChromaffin_cells.Chrom_SOX2_statusHigh.DPP3	0.9315239	2.48210986	3.02382884	0.00342925	0.01086065	-2.2660224	DPP3	assignmentsChromaffin_cells.Chrom_SOX2_statusHigh
assignmentsChromaffin_cells.Chrom_SOX2_statusHigh.LINC01707	-2.6513207	0.59299402	-2.9111085	0.00475935	0.01432898	-2.266758	LINC01707	assignmentsChromaffin_cells.Chrom_SOX2_statusHigh
assignmentsChromaffin_cells.Chrom_SOX2_statusHigh.SETD5	-0.5655794	8.29494849	-3.140058	0.0024275	0.00809067	-2.2708733	SETD5	assignmentsChromaffin_cells.Chrom_SOX2_statusHigh
assignmentsChromaffin_cells.Chrom_SOX2_statusHigh.KCNQ5	-2.6110129	7.56050345	-3.1317095	0.00249148	0.00826954	-2.2719287	KCNQ5	assignmentsChromaffin_cells.Chrom_SOX2_statusHigh
assignmentsChromaffin_cells.Chrom_SOX2_statusHigh.NNT	-0.6617937	6.49718506	-3.1144623	0.00262124	0.008627	-2.2735054	NNT	assignmentsChromaffin_cells.Chrom_SOX2_statusHigh
assignmentsChromaffin_cells.Chrom_SOX2_statusHigh.TAFA2	-1.3556267	5.02637683	-3.0764028	0.00293719	0.00951406	-2.2736178	TAFA2	assignmentsChromaffin_cells.Chrom_SOX2_statusHigh
assignmentsChromaffin_cells.Chrom_SOX2_statusHigh.ATP6V0D1	0.76859406	5.53076909	3.10120575	0.00272715	0.00891704	-2.2739133	ATP6V0D1	assignmentsChromaffin_cells.Chrom_SOX2_statusHigh
assignmentsChromaffin_cells.Chrom_SOX2_statusHigh.NFASC	-1.2828353	5.72996182	-3.1050405	0.00269713	0.00883328	-2.2739691	NFASC	assignmentsChromaffin_cells.Chrom_SOX2_statusHigh
assignmentsChromaffin_cells.Chrom_SOX2_statusHigh.LARP6	0.85455246	3.36380345	3.0719788	0.00297492	0.00962319	-2.2764649	LARP6	assignmentsChromaffin_cells.Chrom_SOX2_statusHigh
assignmentsChromaffin_cells.Chrom_SOX2_statusHigh.MAP1LC3B2	2.15259606	0.47394754	2.90236751	0.00487776	0.01463329	-2.27789	MAP1LC3B2	assignmentsChromaffin_cells.Chrom_SOX2_statusHigh
assignmentsChromaffin_cells.Chrom_SOX2_statusHigh.TMED5	0.70144094	5.49942658	3.08227151	0.00288537	0.00937131	-2.2790385	TMED5	assignmentsChromaffin_cells.Chrom_SOX2_statusHigh
assignmentsChromaffin_cells.Chrom_SOX2_statusHigh.KCNH3	-1.5027511	1.65811077	-2.9850069	0.00384163	0.01194264	-2.2792008	KCNH3	assignmentsChromaffin_cells.Chrom_SOX2_statusHigh
assignmentsChromaffin_cells.Chrom_SOX2_statusHigh.B4GALT5	-0.8053595	6.32371597	-3.0859696	0.00285381	0.00928009	-2.2807853	B4GALT5	assignmentsChromaffin_cells.Chrom_SOX2_statusHigh
assignmentsChromaffin_cells.Chrom_SOX2_statusHigh.ARID3B	-1.020805	4.47189225	-3.0419106	0.00325156	0.01038609	-2.2827976	ARID3B	assignmentsChromaffin_cells.Chrom_SOX2_statusHigh
assignmentsChromaffin_cells.Chrom_SOX2_statusHigh.HOXA4	1.63431494	0.89409586	2.97302637	0.00397865	0.01229946	-2.2829718	HOXA4	assignmentsChromaffin_cells.Chrom_SOX2_statusHigh
assignmentsChromaffin_cells.Chrom_SOX2_statusHigh.ADD1	-0.5747735	7.15895795	-3.1114408	0.00264503	0.0086938	-2.2865541	ADD1	assignmentsChromaffin_cells.Chrom_SOX2_statusHigh
assignmentsChromaffin_cells.Chrom_SOX2_statusHigh.SIAH2-AS1	1.16595989	1.58437382	2.9990456	0.00368747	0.01153255	-2.2895049	SIAH2-AS1	assignmentsChromaffin_cells.Chrom_SOX2_statusHigh
assignmentsChromaffin_cells.Chrom_SOX2_statusHigh.GON4L	-0.4721708	6.85406167	-3.103514	0.00270843	0.00886305	-2.2906941	GON4L	assignmentsChromaffin_cells.Chrom_SOX2_statusHigh
assignmentsChromaffin_cells.Chrom_SOX2_statusHigh.AMOTL2	2.00839719	0.76210515	2.85764794	0.00553903	0.01624315	-2.2920362	AMOTL2	assignmentsChromaffin_cells.Chrom_SOX2_statusHigh
assignmentsChromaffin_cells.Chrom_SOX2_statusHigh.TROAP	1.77157821	0.34782507	2.94091483	0.00436613	0.01333028	-2.2927956	TROAP	assignmentsChromaffin_cells.Chrom_SOX2_statusHigh

assignmentsChromaffin_cells.Chrom_SOX2_statusHigh.FAM169A	-2.7004791	3.15504372	-3.0080017	0.00359505	0.01129431	-2.2945468	FAM169A	assignmentsChromaffin_cells.Chrom_SOX2_statusHigh
assignmentsChromaffin_cells.Chrom_SOX2_statusHigh.CDC42BPA	-0.8037425	7.98514108	-3.1320587	0.00248657	0.00826107	-2.2949493	CDC42BPA	assignmentsChromaffin_cells.Chrom_SOX2_statusHigh
assignmentsChromaffin_cells.Chrom_SOX2_statusHigh.HAUS4	1.4299341	3.4189919	3.03128499	0.00335576	0.01065316	-2.2956736	HAUS4	assignmentsChromaffin_cells.Chrom_SOX2_statusHigh
assignmentsChromaffin_cells.Chrom_SOX2_statusHigh.AKIRIN1	0.70762629	5.33932885	3.07887364	0.00291465	0.0094511	-2.2959547	AKIRIN1	assignmentsChromaffin_cells.Chrom_SOX2_statusHigh
assignmentsChromaffin_cells.Chrom_SOX2_statusHigh.NAA38	0.94712051	5.49802842	3.09256434	0.00279833	0.00912188	-2.2975515	NAA38	assignmentsChromaffin_cells.Chrom_SOX2_statusHigh
assignmentsChromaffin_cells.Chrom_SOX2_statusHigh.S100A6	3.23851392	4.93321451	3.04339254	0.00324012	0.01035586	-2.2976358	S100A6	assignmentsChromaffin_cells.Chrom_SOX2_statusHigh
assignmentsChromaffin_cells.Chrom_SOX2_statusHigh.TMEM120B	-0.6877689	4.91511561	-3.072989	0.00296602	0.00959632	-2.2976681	TMEM120B	assignmentsChromaffin_cells.Chrom_SOX2_statusHigh
assignmentsChromaffin_cells.Chrom_SOX2_statusHigh.MANEA	0.89117445	3.78787612	3.05479367	0.00313023	0.01005862	-2.3003554	MANEA	assignmentsChromaffin_cells.Chrom_SOX2_statusHigh
assignmentsChromaffin_cells.Chrom_SOX2_statusHigh.STPG1	-1.2636859	2.3356813	-2.9820616	0.00387472	0.01202917	-2.3023887	STPG1	assignmentsChromaffin_cells.Chrom_SOX2_statusHigh
assignmentsChromaffin_cells.Chrom_SOX2_statusHigh.CDC25C	1.14309497	1.10133908	2.96729988	0.00404458	0.01248644	-2.3024294	CDC25C	assignmentsChromaffin_cells.Chrom_SOX2_statusHigh
assignmentsChromaffin_cells.Chrom_SOX2_statusHigh.SLC5A4	-3.4712149	1.45226266	-2.861969	0.00547536	0.01608311	-2.3027056	SLC5A4	assignmentsChromaffin_cells.Chrom_SOX2_statusHigh
assignmentsChromaffin_cells.Chrom_SOX2_statusHigh.ZNF662	2.76883914	2.28111471	3.00564936	0.00361988	0.01135007	-2.3030948	ZNF662	assignmentsChromaffin_cells.Chrom_SOX2_statusHigh
assignmentsChromaffin_cells.Chrom_SOX2_statusHigh.CPEB2	-0.9834449	6.35417983	-3.085397	0.00285867	0.00929403	-2.3048708	CPEB2	assignmentsChromaffin_cells.Chrom_SOX2_statusHigh
assignmentsChromaffin_cells.Chrom_SOX2_statusHigh.LINC02470	2.90296569	-0.6456424	2.83915262	0.00583751	0.01696972	-2.3051091	LINC02470	assignmentsChromaffin_cells.Chrom_SOX2_statusHigh
assignmentsChromaffin_cells.Chrom_SOX2_statusHigh.LINC01602	-3.4305621	0.70318417	-2.8771392	0.00524449	0.0155158	-2.3065186	LINC01602	assignmentsChromaffin_cells.Chrom_SOX2_statusHigh
assignmentsChromaffin_cells.Chrom_SOX2_statusHigh.LINC01237	-1.267438	4.6369677	-3.0508186	0.00316791	0.01015569	-2.3067932	LINC01237	assignmentsChromaffin_cells.Chrom_SOX2_statusHigh
assignmentsChromaffin_cells.Chrom_SOX2_statusHigh.ZKSCAN2-D	-0.925352	2.54266953	-3.0056943	0.00361648	0.01134385	-2.3082474	ZKSCAN2-DT	assignmentsChromaffin_cells.Chrom_SOX2_statusHigh
assignmentsChromaffin_cells.Chrom_SOX2_statusHigh.DNPEP	0.68220216	4.37887678	3.06039165	0.00307883	0.00992726	-2.3090215	DNPEP	assignmentsChromaffin_cells.Chrom_SOX2_statusHigh
assignmentsChromaffin_cells.Chrom_SOX2_statusHigh.CAD	-0.8967836	3.31283195	-3.0185316	0.00348299	0.01099393	-2.3092268	CAD	assignmentsChromaffin_cells.Chrom_SOX2_statusHigh
assignmentsChromaffin_cells.Chrom_SOX2_statusHigh.ERLIN2	0.63933614	3.71222426	3.04805122	0.00319319	0.01023021	-2.3095087	ERLIN2	assignmentsChromaffin_cells.Chrom_SOX2_statusHigh
assignmentsChromaffin_cells.Chrom_SOX2_statusHigh.ZNF639	0.67576724	3.90377022	3.04612154	0.00321143	0.01027427	-2.3096165	ZNF639	assignmentsChromaffin_cells.Chrom_SOX2_statusHigh
assignmentsChromaffin_cells.Chrom_SOX2_statusHigh.TCF4-AS2	-1.7517905	1.26203843	-2.9332477	0.00446347	0.01357321	-2.3118661	TCF4-AS2	assignmentsChromaffin_cells.Chrom_SOX2_statusHigh
assignmentsChromaffin_cells.Chrom_SOX2_statusHigh.MAPRE1	0.81510234	5.41055532	3.08087019	0.00289741	0.00940661	-2.3130247	MAPRE1	assignmentsChromaffin_cells.Chrom_SOX2_statusHigh
assignmentsChromaffin_cells.Chrom_SOX2_statusHigh.N4BP3	2.19263371	0.7423005	2.93935144	0.0043879	0.0133756	-2.3144623	N4BP3	assignmentsChromaffin_cells.Chrom_SOX2_statusHigh
assignmentsChromaffin_cells.Chrom_SOX2_statusHigh.PP2D1	-1.4754533	1.12990059	-2.9194845	0.0046433	0.01404021	-2.3151836	PP2D1	assignmentsChromaffin_cells.Chrom_SOX2_statusHigh
assignmentsChromaffin_cells.Chrom_SOX2_statusHigh.TMEM62	-0.739973	5.18506112	-3.0609533	0.00307371	0.00991277	-2.3159926	TMEM62	assignmentsChromaffin_cells.Chrom_SOX2_statusHigh
assignmentsChromaffin_cells.Chrom_SOX2_statusHigh.TK1	1.59197243	0.62143793	2.90496243	0.00484074	0.01454399	-2.3177495	TK1	assignmentsChromaffin_cells.Chrom_SOX2_statusHigh
assignmentsChromaffin_cells.Chrom_SOX2_statusHigh.GADD45A	1.75803723	3.76828087	3.04152628	0.00325799	0.0104004	-2.321431	GADD45A	assignmentsChromaffin_cells.Chrom_SOX2_statusHigh
assignmentsChromaffin_cells.Chrom_SOX2_statusHigh.RPF1	0.88050578	3.8198691	3.03275128	0.00334047	0.01061302	-2.3230949	RPF1	assignmentsChromaffin_cells.Chrom_SOX2_statusHigh
assignmentsChromaffin_cells.Chrom_SOX2_statusHigh.GJD2	1.82785584	0.38702045	2.96316846	0.00409467	0.0126095	-2.3260742	GJD2	assignmentsChromaffin_cells.Chrom_SOX2_statusHigh
assignmentsChromaffin_cells.Chrom_SOX2_statusHigh.CCDC91	-0.9400077	8.30956401	-3.1203708	0.00257528	0.00849492	-2.3296974	CCDC91	assignmentsChromaffin_cells.Chrom_SOX2_statusHigh
assignmentsChromaffin_cells.Chrom_SOX2_statusHigh.SLC38A9	-0.7014379	5.72980995	-3.0597703	0.00308449	0.00993754	-2.331854	SLC38A9	assignmentsChromaffin_cells.Chrom_SOX2_statusHigh
assignmentsChromaffin_cells.Chrom_SOX2_statusHigh.C8orf76	0.63431748	4.85726825	3.06285375	0.00305647	0.00986906	-2.3338521	C8orf76	assignmentsChromaffin_cells.Chrom_SOX2_statusHigh
assignmentsChromaffin_cells.Chrom_SOX2_statusHigh.SRD5A3	1.01381824	3.00496246	3.00825754	0.00358945	0.01128113	-2.3340041	SRD5A3	assignmentsChromaffin_cells.Chrom_SOX2_statusHigh
assignmentsChromaffin_cells.Chrom_SOX2_statusHigh.PEA15	1.17547824	4.74975966	3.05507899	0.003128	0.0100535	-2.3346056	PEA15	assignmentsChromaffin_cells.Chrom_SOX2_statusHigh
assignmentsChromaffin_cells.Chrom_SOX2_statusHigh.RALGAPA1	-0.7721831	8.72884913	-3.123415	0.00255189	0.00843512	-2.334889	RALGAPA1	assignmentsChromaffin_cells.Chrom_SOX2_statusHigh
assignmentsChromaffin_cells.Chrom_SOX2_statusHigh.RTN4RL2	1.8828439	-0.137472	2.82864001	0.00601184	0.01742597	-2.3369607	RTN4RL2	assignmentsChromaffin_cells.Chrom_SOX2_statusHigh
assignmentsChromaffin_cells.Chrom_SOX2_statusHigh.MIR4527HG	-2.6973445	-0.3447823	-2.8283823	0.00601719	0.01743517	-2.337179	MIR4527HG	assignmentsChromaffin_cells.Chrom_SOX2_statusHigh
assignmentsChromaffin_cells.Chrom_SOX2_statusHigh.INO80B	0.63807335	3.11371069	3.01804188	0.003488	0.01100541	-2.3374995	INO80B	assignmentsChromaffin_cells.Chrom_SOX2_statusHigh
assignmentsChromaffin_cells.Chrom_SOX2_statusHigh.BTG3	0.96295576	5.49187622	3.07526778	0.00294603	0.00953549	-2.3388587	BTG3	assignmentsChromaffin_cells.Chrom_SOX2_statusHigh
assignmentsChromaffin_cells.Chrom_SOX2_statusHigh.CCR7	2.37173207	0.44775723	2.84123993	0.00580209	0.01687289	-2.3399579	CCR7	assignmentsChromaffin_cells.Chrom_SOX2_statusHigh
assignmentsChromaffin_cells.Chrom_SOX2_statusHigh.DNHD1	-0.8543108	4.80282897	-3.0380978	0.0032883	0.01047632	-2.3407226	DNHD1	assignmentsChromaffin_cells.Chrom_SOX2_statusHigh
assignmentsChromaffin_cells.Chrom_SOX2_statusHigh.ZBTB20-AS	-1.5144747	2.4717172	-2.9647728	0.00407472	0.01256012	-2.3416127	ZBTB20-AS1	assignmentsChromaffin_cells.Chrom_SOX2_statusHigh
assignmentsChromaffin_cells.Chrom_SOX2_statusHigh.SSBP4	1.12262011	4.90661073	3.0672057	0.00301763	0.00975545	-2.3418169	SSBP4	assignmentsChromaffin_cells.Chrom_SOX2_statusHigh
assignmentsChromaffin_cells.Chrom_SOX2_statusHigh.DDR2	1.38588161	4.38578227	3.03070943	0.00336164	0.01066971	-2.3428869	DDR2	assignmentsChromaffin_cells.Chrom_SOX2_statusHigh
assignmentsChromaffin_cells.Chrom_SOX2_statusHigh.COIL	0.6197319	4.44211665	3.05528216	0.00312571	0.01004813	-2.3504832	COIL	assignmentsChromaffin_cells.Chrom_SOX2_statusHigh
assignmentsChromaffin_cells.Chrom_SOX2_statusHigh.C2orf81	0.84520502	2.28670283	3.00630235	0.00361005	0.01133033	-2.3513665	C2orf81	assignmentsChromaffin_cells.Chrom_SOX2_statusHigh
assignmentsChromaffin_cells.Chrom_SOX2_statusHigh.CEPT1	-0.7214838	5.16624234	-3.0393016	0.00327666	0.01044753	-2.3514528	CEPT1	assignmentsChromaffin_cells.Chrom_SOX2_statusHigh
assignmentsChromaffin_cells.Chrom_SOX2_statusHigh.PRKAR2B	-1.4214228	3.9186015	-3.0112832	0.00355868	0.0111976	-2.3522178	PRKAR2B	assignmentsChromaffin_cells.Chrom_SOX2_statusHigh
assignmentsChromaffin_cells.Chrom_SOX2_statusHigh.EVL	-0.7337574	7.14314434	-3.0852653	0.0028598	0.00929578	-2.3533455	EVL	assignmentsChromaffin_cells.Chrom_SOX2_statusHigh
assignmentsChromaffin_cells.Chrom_SOX2_statusHigh.DET1	-0.790734	4.12699577	-3.0363526	0.00330525	0.01052404	-2.3547094	DET1	assignmentsChromaffin_cells.Chrom_SOX2_statusHigh
assignmentsChromaffin_cells.Chrom_SOX2_statusHigh.PARP12	2.00545957	4.36985056	2.98831756	0.00380776	0.01184193	-2.3596013	PARP12	assignmentsChromaffin_cells.Chrom_SOX2_statusHigh
assignmentsChromaffin_cells.Chrom_SOX2_statusHigh.MCRS1	0.60801621	3.70522222	3.02614081	0.00340604	0.01079779	-2.3603555	MCRS1	assignmentsChromaffin_cells.Chrom_SOX2_statusHigh
assignmentsChromaffin_cells.Chrom_SOX2_statusHigh.LIN7C	0.80373613	3.99625787	3.02833234	0.00338417	0.01073271	-2.3605471	LIN7C	assignmentsChromaffin_cells.Chrom_SOX2_statusHigh
assignmentsChromaffin_cells.Chrom_SOX2_statusHigh.INTS9	-0.5240652	5.38813581	-3.0464221	0.00320858	0.01026721	-2.3645913	INTS9	assignmentsChromaffin_cells.Chrom_SOX2_statusHigh
assignmentsChromaffin_cells.Chrom_SOX2_statusHigh.CLN5	0.66495988	4.69975354	3.05277413	0.00314896	0.01010676	-2.3662165	CLN5	assignmentsChromaffin_cells.Chrom_SOX2_statusHigh
assignmentsChromaffin_cells.Chrom_SOX2_statusHigh.ADCY5	-1.8866343	2.53330307	-2.9686492	0.00403158	0.01245108	-2.3666825	ADCY5	assignmentsChromaffin_cells.Chrom_SOX2_statusHigh
assignmentsChromaffin_cells.Chrom_SOX2_statusHigh.TMEM234	0.72033631	3.24866185	3.00838017	0.00358816	0.01127929	-2.3667074	TMEM234	assignmentsChromaffin_cells.Chrom_SOX2_statusHigh
assignmentsChromaffin_cells.Chrom_SOX2_statusHigh.ZMPSTE24	0.68643486	4.3460978	3.0250368	0.0034171	0.01082646	-2.3672927	ZMPSTE24	assignmentsChromaffin_cells.Chrom_SOX2_statusHigh
assignmentsChromaffin_cells.Chrom_SOX2_statusHigh.GMPR	1.70773349	2.52285046	2.97635942	0.00394115	0.01220233	-2.3676695	GMPR	assignmentsChromaffin_cells.Chrom_SOX2_statusHigh
assignmentsChromaffin_cells.Chrom_SOX2_statusHigh.EIF2B2	0.63369962	3.94461447	3.02242585	0.00344341	0.01088859	-2.3709842	EIF2B2	assignmentsChromaffin_cells.Chrom_SOX2_statusHigh
assignmentsChromaffin_cells.Chrom_SOX2_statusHigh.ARL5C	2.20128475	-0.4183839	2.81159676	0.00630666	0.01814933	-2.3713637	ARL5C	assignmentsChromaffin_cells.Chrom_SOX2_statusHigh
assignmentsChromaffin_cells.Chrom_SOX2_statusHigh.SDHA	0.487424	5.6822197	3.06227767	0.00306169	0.00988193	-2.3729998	SDHA	assignmentsChromaffin_cells.Chrom_SOX2_statusHigh
assignmentsChromaffin_cells.Chrom_SOX2_statusHigh.RAI14	-2.0289969	4.57721245	-2.936357	0.00442594	0.01347186	-2.3733826	RAI14	assignmentsChromaffin_cells.Chrom_SOX2_statusHigh

assignmentsChromaffin_cells.Chrom_SOX2_statusHigh.ST8SIA6-AS	-1.7466664	0.03177614	-2.8171495	0.00620918	0.01790092	-2.3737983	ST8SIA6-AS1	assignmentsChromaffin_cells.Chrom_SOX2_statusHigh
assignmentsChromaffin_cells.Chrom_SOX2_statusHigh.LINC02485	-2.5085172	0.47919058	-2.8806573	0.00519209	0.01539769	-2.3747131	LINC02485	assignmentsChromaffin_cells.Chrom_SOX2_statusHigh
assignmentsChromaffin_cells.Chrom_SOX2_statusHigh.SLC66A1	0.75974412	2.25714278	2.98483939	0.0038435	0.01194614	-2.3749866	SLC66A1	assignmentsChromaffin_cells.Chrom_SOX2_statusHigh
assignmentsChromaffin_cells.Chrom_SOX2_statusHigh.TENT2	-0.7305832	7.51738131	-3.0795729	0.0029086	0.00943721	-2.3774008	TENT2	assignmentsChromaffin_cells.Chrom_SOX2_statusHigh
assignmentsChromaffin_cells.Chrom_SOX2_statusHigh.ARRDC4	0.92363156	3.90487688	3.01625839	0.00350629	0.01105442	-2.3777511	ARRDC4	assignmentsChromaffin_cells.Chrom_SOX2_statusHigh
assignmentsChromaffin_cells.Chrom_SOX2_statusHigh.PCBD2	-0.8025378	5.12887955	-3.0374552	0.00329453	0.010492	-2.3777966	PCBD2	assignmentsChromaffin_cells.Chrom_SOX2_statusHigh
assignmentsChromaffin_cells.Chrom_SOX2_statusHigh.SERHL2	-1.1570762	1.58008212	-2.9150245	0.00470313	0.01419436	-2.3787595	SERHL2	assignmentsChromaffin_cells.Chrom_SOX2_statusHigh
assignmentsChromaffin_cells.Chrom_SOX2_statusHigh.EIF3M	0.75972207	5.61633256	3.05596926	0.00311937	0.01003176	-2.3827065	EIF3M	assignmentsChromaffin_cells.Chrom_SOX2_statusHigh
assignmentsChromaffin_cells.Chrom_SOX2_statusHigh.POLE	-0.8354759	3.65350089	-2.9969377	0.00371024	0.01158358	-2.3836577	POLE	assignmentsChromaffin_cells.Chrom_SOX2_statusHigh
assignmentsChromaffin_cells.Chrom_SOX2_statusHigh.CCDC88A	-0.7151636	7.08099721	-3.0780363	0.00292191	0.00947081	-2.3837248	CCDC88A	assignmentsChromaffin_cells.Chrom_SOX2_statusHigh
assignmentsChromaffin_cells.Chrom_SOX2_statusHigh.AGO4	-0.9198529	5.69243857	-3.0281407	0.00338608	0.01073664	-2.3845808	AGO4	assignmentsChromaffin_cells.Chrom_SOX2_statusHigh
assignmentsChromaffin_cells.Chrom_SOX2_statusHigh.TCERG1	-0.6348906	7.10063769	-3.080202	0.00290317	0.0094234	-2.3848555	TCERG1	assignmentsChromaffin_cells.Chrom_SOX2_statusHigh
assignmentsChromaffin_cells.Chrom_SOX2_statusHigh.SDHAF3	1.44549599	3.62841079	3.01368519	0.00353405	0.01113099	-2.3862063	SDHAF3	assignmentsChromaffin_cells.Chrom_SOX2_statusHigh
assignmentsChromaffin_cells.Chrom_SOX2_statusHigh.CCDC180	-1.0774921	3.68865726	-2.9984466	0.00369393	0.01154838	-2.3865876	CCDC180	assignmentsChromaffin_cells.Chrom_SOX2_statusHigh
assignmentsChromaffin_cells.Chrom_SOX2_statusHigh.MAGOHB	0.74539393	4.23467918	3.03371801	0.00333098	0.01059336	-2.3898861	MAGOHB	assignmentsChromaffin_cells.Chrom_SOX2_statusHigh
assignmentsChromaffin_cells.Chrom_SOX2_statusHigh.PAN2	-0.7692057	4.49157136	-3.022116	0.00344654	0.0108939	-2.3935126	PAN2	assignmentsChromaffin_cells.Chrom_SOX2_statusHigh
assignmentsChromaffin_cells.Chrom_SOX2_statusHigh.KHSRP	0.6691163	4.7702969	3.03962012	0.00327359	0.01043981	-2.3964351	KHSRP	assignmentsChromaffin_cells.Chrom_SOX2_statusHigh
assignmentsChromaffin_cells.Chrom_SOX2_statusHigh.CNTN5	-3.0597513	2.83684615	-2.9587045	0.00414982	0.01275731	-2.3968466	CNTN5	assignmentsChromaffin_cells.Chrom_SOX2_statusHigh
assignmentsChromaffin_cells.Chrom_SOX2_statusHigh.RBMS1	-1.0120676	8.93815218	-3.0922588	0.0028015	0.00913035	-2.3977145	RBMS1	assignmentsChromaffin_cells.Chrom_SOX2_statusHigh
assignmentsChromaffin_cells.Chrom_SOX2_statusHigh.GSTA4	-0.6147986	4.64353976	-3.0416299	0.00325426	0.01039055	-2.3982917	GSTA4	assignmentsChromaffin_cells.Chrom_SOX2_statusHigh
assignmentsChromaffin_cells.Chrom_SOX2_statusHigh.LMAN2L	-0.6822926	4.25990787	-3.0182663	0.0034857	0.01100033	-2.3983817	LMAN2L	assignmentsChromaffin_cells.Chrom_SOX2_statusHigh
assignmentsChromaffin_cells.Chrom_SOX2_statusHigh.KRT222	2.10713962	1.70395587	2.99860529	0.00369518	0.01155003	-2.3988351	KRT222	assignmentsChromaffin_cells.Chrom_SOX2_statusHigh
assignmentsChromaffin_cells.Chrom_SOX2_statusHigh.CARD14	-2.1479162	0.83926005	-2.8387869	0.00584275	0.0169788	-2.3997059	CARD14	assignmentsChromaffin_cells.Chrom_SOX2_statusHigh
assignmentsChromaffin_cells.Chrom_SOX2_statusHigh.KDM4B	-0.7457197	6.22287513	-3.0515435	0.00316043	0.01014146	-2.3999395	KDM4B	assignmentsChromaffin_cells.Chrom_SOX2_statusHigh
assignmentsChromaffin_cells.Chrom_SOX2_statusHigh.PDIK1L	0.76890945	2.56113497	2.97445882	0.00396136	0.01225546	-2.4002543	PDIK1L	assignmentsChromaffin_cells.Chrom_SOX2_statusHigh
assignmentsChromaffin_cells.Chrom_SOX2_statusHigh.ZNF677	0.83726331	3.84119624	3.02339241	0.00343365	0.01087028	-2.4008465	ZNF677	assignmentsChromaffin_cells.Chrom_SOX2_statusHigh
assignmentsChromaffin_cells.Chrom_SOX2_statusHigh.ZSCAN23	-0.9960359	3.18691985	-2.9995851	0.00368166	0.01152126	-2.4015966	ZSCAN23	assignmentsChromaffin_cells.Chrom_SOX2_statusHigh
assignmentsChromaffin_cells.Chrom_SOX2_statusHigh.POLR2H	0.72785605	4.26696416	3.02560232	0.00341143	0.01081276	-2.4018873	POLR2H	assignmentsChromaffin_cells.Chrom_SOX2_statusHigh
assignmentsChromaffin_cells.Chrom_SOX2_statusHigh.PLA1A	1.86152869	0.28948741	2.79296851	0.00664404	0.01892666	-2.4035085	PLA1A	assignmentsChromaffin_cells.Chrom_SOX2_statusHigh
assignmentsChromaffin_cells.Chrom_SOX2_statusHigh.LMF2	0.88890958	4.14986297	3.01893829	0.00347884	0.01098299	-2.4045099	LMF2	assignmentsChromaffin_cells.Chrom_SOX2_statusHigh
assignmentsChromaffin_cells.Chrom_SOX2_statusHigh.KIF5B	0.53456711	7.13967866	3.07578443	0.00294151	0.00952472	-2.4063676	KIF5B	assignmentsChromaffin_cells.Chrom_SOX2_statusHigh
assignmentsChromaffin_cells.Chrom_SOX2_statusHigh.MICU3	-0.786642	6.48022165	-3.0649653	0.00303742	0.00981545	-2.406515	MICU3	assignmentsChromaffin_cells.Chrom_SOX2_statusHigh
assignmentsChromaffin_cells.Chrom_SOX2_statusHigh.PILRA	-1.2053499	3.84252735	-2.9824638	0.00387018	0.01201975	-2.4065208	PILRA	assignmentsChromaffin_cells.Chrom_SOX2_statusHigh
assignmentsChromaffin_cells.Chrom_SOX2_statusHigh.LY6G6C	2.06569686	-0.5945859	2.79243638	0.00665391	0.0189447	-2.4083168	LY6G6C	assignmentsChromaffin_cells.Chrom_SOX2_statusHigh
assignmentsChromaffin_cells.Chrom_SOX2_statusHigh.ACACB	-0.7410419	6.23321728	-3.0601882	0.00308068	0.00993104	-2.4085403	ACACB	assignmentsChromaffin_cells.Chrom_SOX2_statusHigh
assignmentsChromaffin_cells.Chrom_SOX2_statusHigh.ITSN2	-0.4367316	7.79855233	-3.0762298	0.00293762	0.00951406	-2.4092114	ITSN2	assignmentsChromaffin_cells.Chrom_SOX2_statusHigh
assignmentsChromaffin_cells.Chrom_SOX2_statusHigh.SNHG9	1.4712131	3.19786403	3.00552219	0.00361956	0.01135007	-2.4107175	SNHG9	assignmentsChromaffin_cells.Chrom_SOX2_statusHigh
assignmentsChromaffin_cells.Chrom_SOX2_statusHigh.NUDT22	1.0587105	3.47389377	2.99715077	0.00370793	0.01158031	-2.4109571	NUDT22	assignmentsChromaffin_cells.Chrom_SOX2_statusHigh
assignmentsChromaffin_cells.Chrom_SOX2_statusHigh.KCNC1	-1.3471223	2.99629302	-2.9889612	0.00379848	0.01181766	-2.4117735	KCNC1	assignmentsChromaffin_cells.Chrom_SOX2_statusHigh
assignmentsChromaffin_cells.Chrom_SOX2_statusHigh.MTCH1	0.89155695	6.30774114	3.06232352	0.00306127	0.00988193	-2.4121404	MTCH1	assignmentsChromaffin_cells.Chrom_SOX2_statusHigh
assignmentsChromaffin_cells.Chrom_SOX2_statusHigh.C14orf39	-1.7643467	4.5510627	-3.0402705	0.00327007	0.01043065	-2.4132195	C14orf39	assignmentsChromaffin_cells.Chrom_SOX2_statusHigh
assignmentsChromaffin_cells.Chrom_SOX2_statusHigh.ADD3	-0.7166938	7.00462789	-3.0536982	0.00314038	0.01008518	-2.414114	ADD3	assignmentsChromaffin_cells.Chrom_SOX2_statusHigh
assignmentsChromaffin_cells.Chrom_SOX2_statusHigh.DUSP4	2.18704932	3.90459939	3.00270905	0.00365113	0.01143689	-2.4150824	DUSP4	assignmentsChromaffin_cells.Chrom_SOX2_statusHigh
assignmentsChromaffin_cells.Chrom_SOX2_statusHigh.IGSF8	0.8090931	3.69014102	3.02248231	0.00344284	0.01088859	-2.4156256	IGSF8	assignmentsChromaffin_cells.Chrom_SOX2_statusHigh
assignmentsChromaffin_cells.Chrom_SOX2_statusHigh.MAPK6	-0.6938587	6.20408835	-3.0473804	0.00319952	0.0102449	-2.4156956	MAPK6	assignmentsChromaffin_cells.Chrom_SOX2_statusHigh
assignmentsChromaffin_cells.Chrom_SOX2_statusHigh.WASF3-AS1	-2.668178	-0.186134	-2.7955864	0.00659676	0.01880868	-2.416696	WASF3-AS1	assignmentsChromaffin_cells.Chrom_SOX2_statusHigh
assignmentsChromaffin_cells.Chrom_SOX2_statusHigh.CASC6	2.27187256	-0.753319	2.8218705	0.00612817	0.01770238	-2.4169226	CASC6	assignmentsChromaffin_cells.Chrom_SOX2_statusHigh
assignmentsChromaffin_cells.Chrom_SOX2_statusHigh.FCGRT	1.77245221	4.63989871	2.97759699	0.00392846	0.0121701	-2.4173896	FCGRT	assignmentsChromaffin_cells.Chrom_SOX2_statusHigh
assignmentsChromaffin_cells.Chrom_SOX2_statusHigh.AGFG2	-0.9096452	3.66892469	-2.9908902	0.00377631	0.01176204	-2.4183388	AGFG2	assignmentsChromaffin_cells.Chrom_SOX2_statusHigh
assignmentsChromaffin_cells.Chrom_SOX2_statusHigh.CD27-AS1	0.69456566	4.07850021	3.02095346	0.00345832	0.01092468	-2.4186453	CD27-AS1	assignmentsChromaffin_cells.Chrom_SOX2_statusHigh
assignmentsChromaffin_cells.Chrom_SOX2_statusHigh.ETHE1	1.05053586	3.83192143	3.001693	0.00365905	0.01145944	-2.4188067	ETHE1	assignmentsChromaffin_cells.Chrom_SOX2_statusHigh
assignmentsChromaffin_cells.Chrom_SOX2_statusHigh.GNG12-AS1	-1.3409202	3.29059863	-2.9540274	0.00420318	0.01289662	-2.4191218	GNG12-AS1	assignmentsChromaffin_cells.Chrom_SOX2_statusHigh
assignmentsChromaffin_cells.Chrom_SOX2_statusHigh.HOXB7	2.27192648	2.05025693	2.96724517	0.00404836	0.01249328	-2.4211818	HOXB7	assignmentsChromaffin_cells.Chrom_SOX2_statusHigh
assignmentsChromaffin_cells.Chrom_SOX2_statusHigh.NOX3	2.03259483	-0.4114084	2.83217219	0.00595235	0.01727227	-2.4214863	NOX3	assignmentsChromaffin_cells.Chrom_SOX2_statusHigh
assignmentsChromaffin_cells.Chrom_SOX2_statusHigh.KIAA0586	-0.5983052	6.04232035	-3.0436495	0.00323493	0.01034534	-2.4236233	KIAA0586	assignmentsChromaffin_cells.Chrom_SOX2_statusHigh
assignmentsChromaffin_cells.Chrom_SOX2_statusHigh.SLC44A2	-0.8772934	5.16884504	-2.9888833	0.00379847	0.01181766	-2.4259555	SLC44A2	assignmentsChromaffin_cells.Chrom_SOX2_statusHigh
assignmentsChromaffin_cells.Chrom_SOX2_statusHigh.GAS1	2.28750962	-0.2831154	2.78870489	0.00672355	0.01909888	-2.4272888	GAS1	assignmentsChromaffin_cells.Chrom_SOX2_statusHigh
assignmentsChromaffin_cells.Chrom_SOX2_statusHigh.IFITM2	2.10510157	4.22441438	2.92275166	0.0046033	0.0139324	-2.4273512	IFITM2	assignmentsChromaffin_cells.Chrom_SOX2_statusHigh
assignmentsChromaffin_cells.Chrom_SOX2_statusHigh.MELTF	2.54796576	1.50290508	2.91501244	0.00470673	0.01419719	-2.4282556	MELTF	assignmentsChromaffin_cells.Chrom_SOX2_statusHigh
assignmentsChromaffin_cells.Chrom_SOX2_statusHigh.IL6	2.70505604	0.81206329	2.83902065	0.00584243	0.0169788	-2.4306855	IL6	assignmentsChromaffin_cells.Chrom_SOX2_statusHigh
assignmentsChromaffin_cells.Chrom_SOX2_statusHigh.ITGB1	1.00244185	8.24693682	3.07933349	0.0029113	0.00944406	-2.4316554	ITGB1	assignmentsChromaffin_cells.Chrom_SOX2_statusHigh
assignmentsChromaffin_cells.Chrom_SOX2_statusHigh.FZD2	2.06525801	-0.205139	2.78591225	0.0067761	0.01922433	-2.4342007	FZD2	assignmentsChromaffin_cells.Chrom_SOX2_statusHigh
assignmentsChromaffin_cells.Chrom_SOX2_statusHigh.KLHL12	-0.675485	4.50835014	-3.0038739	0.00363579	0.0113955	-2.4362149	KLHL12	assignmentsChromaffin_cells.Chrom_SOX2_statusHigh
assignmentsChromaffin_cells.Chrom_SOX2_statusHigh.KCNK3	3.09912953	3.13042316	2.99795399	0.00370221	0.01156977	-2.4362973	KCNK3	assignmentsChromaffin_cells.Chrom_SOX2_statusHigh

assignmentsChromaffin_cells.Chrom_SOX2_statusHigh.KHDRBS1	0.71170257	6.80520529	3.05104134	0.00316512	0.01015245	-2.4373752	KHDRBS1	assignmentsChromaffin_cells.Chrom_SOX2_statusHigh
assignmentsChromaffin_cells.Chrom_SOX2_statusHigh.HEG1	-1.3570019	5.27846398	-2.9629445	0.00409653	0.01261283	-2.4376674	HEG1	assignmentsChromaffin_cells.Chrom_SOX2_statusHigh
assignmentsChromaffin_cells.Chrom_SOX2_statusHigh.TTI1	-0.6342625	4.99817675	-3.0128315	0.00354169	0.01115287	-2.441114	TTI1	assignmentsChromaffin_cells.Chrom_SOX2_statusHigh
assignmentsChromaffin_cells.Chrom_SOX2_statusHigh.ELOVL4	0.76738879	3.5307558	3.01492553	0.00352002	0.01109335	-2.4422656	ELOVL4	assignmentsChromaffin_cells.Chrom_SOX2_statusHigh
assignmentsChromaffin_cells.Chrom_SOX2_statusHigh.LINC01031	1.06775414	1.21636956	2.91789436	0.00466455	0.01409383	-2.4423451	LINC01031	assignmentsChromaffin_cells.Chrom_SOX2_statusHigh
assignmentsChromaffin_cells.Chrom_SOX2_statusHigh.BTG2	1.90358379	6.84933999	3.05940156	0.00309051	0.00995493	-2.4427996	BTG2	assignmentsChromaffin_cells.Chrom_SOX2_statusHigh
assignmentsChromaffin_cells.Chrom_SOX2_statusHigh.UBR1	-0.5967546	7.67189456	-3.0629493	0.0030556	0.00986906	-2.4436614	UBR1	assignmentsChromaffin_cells.Chrom_SOX2_statusHigh
assignmentsChromaffin_cells.Chrom_SOX2_statusHigh.MYLK-AS1	-1.0417332	2.87364097	-2.9448213	0.00431654	0.01320402	-2.4451623	MYLK-AS1	assignmentsChromaffin_cells.Chrom_SOX2_statusHigh
assignmentsChromaffin_cells.Chrom_SOX2_statusHigh.MTRES1	0.68204469	3.64426952	2.99728196	0.00370651	0.01157977	-2.4470214	MTRES1	assignmentsChromaffin_cells.Chrom_SOX2_statusHigh
assignmentsChromaffin_cells.Chrom_SOX2_statusHigh.TSPAN15	1.76131515	3.09579901	2.88887266	0.00506957	0.01509575	-2.4483236	TSPAN15	assignmentsChromaffin_cells.Chrom_SOX2_statusHigh
assignmentsChromaffin_cells.Chrom_SOX2_statusHigh.KLHL32	-1.2747914	2.8335559	-2.9795844	0.00390327	0.01210376	-2.4496523	KLHL32	assignmentsChromaffin_cells.Chrom_SOX2_statusHigh
assignmentsChromaffin_cells.Chrom_SOX2_statusHigh.LINC00880	1.18575029	0.58217642	2.8584224	0.00552689	0.01621073	-2.4505705	LINC00880	assignmentsChromaffin_cells.Chrom_SOX2_statusHigh
assignmentsChromaffin_cells.Chrom_SOX2_statusHigh.NIPA2	0.4288258	5.1454581	3.02326091	0.00343497	0.01087234	-2.4525904	NIPA2	assignmentsChromaffin_cells.Chrom_SOX2_statusHigh
assignmentsChromaffin_cells.Chrom_SOX2_statusHigh.CENPN	0.84764273	2.12714684	2.93951633	0.00438314	0.01336696	-2.4550339	CENPN	assignmentsChromaffin_cells.Chrom_SOX2_statusHigh
assignmentsChromaffin_cells.Chrom_SOX2_statusHigh.LINC01090	-1.6651937	2.3467186	-2.9360532	0.00442852	0.01347715	-2.4550642	LINC01090	assignmentsChromaffin_cells.Chrom_SOX2_statusHigh
assignmentsChromaffin_cells.Chrom_SOX2_statusHigh.SUPT20H	-0.6069495	6.37986923	-3.0420268	0.00325045	0.0103846	-2.4560724	SUPT20H	assignmentsChromaffin_cells.Chrom_SOX2_statusHigh
assignmentsChromaffin_cells.Chrom_SOX2_statusHigh.SRD5A1	0.74742519	4.28970411	3.01428808	0.0035266	0.01111136	-2.4566023	SRD5A1	assignmentsChromaffin_cells.Chrom_SOX2_statusHigh
assignmentsChromaffin_cells.Chrom_SOX2_statusHigh.DCAF5	-0.5580962	7.01649489	-3.0476821	0.00319667	0.01023932	-2.4570802	DCAF5	assignmentsChromaffin_cells.Chrom_SOX2_statusHigh
assignmentsChromaffin_cells.Chrom_SOX2_statusHigh.SCN1A-AS1	-1.4700224	3.12552526	-2.9806158	0.00389234	0.01207687	-2.4588094	SCN1A-AS1	assignmentsChromaffin_cells.Chrom_SOX2_statusHigh
assignmentsChromaffin_cells.Chrom_SOX2_statusHigh.CRABP1	2.9583126	-0.7764313	2.77734731	0.00694144	0.01963435	-2.4597423	CRABP1	assignmentsChromaffin_cells.Chrom_SOX2_statusHigh
assignmentsChromaffin_cells.Chrom_SOX2_statusHigh.ARMC7	0.86766116	2.33701723	2.92653324	0.00455017	0.01378981	-2.460949	ARMC7	assignmentsChromaffin_cells.Chrom_SOX2_statusHigh
assignmentsChromaffin_cells.Chrom_SOX2_statusHigh.TMEM242	0.72093675	4.59984755	3.00745974	0.00359784	0.01130086	-2.4619327	TMEM242	assignmentsChromaffin_cells.Chrom_SOX2_statusHigh
assignmentsChromaffin_cells.Chrom_SOX2_statusHigh.NPR3	2.16003102	1.67064785	2.91425239	0.004717	0.01421481	-2.46547	NPR3	assignmentsChromaffin_cells.Chrom_SOX2_statusHigh
assignmentsChromaffin_cells.Chrom_SOX2_statusHigh.GOLGA6L7	-2.751563	0.07106591	-2.803894	0.00644728	0.01846783	-2.4656574	GOLGA6L7	assignmentsChromaffin_cells.Chrom_SOX2_statusHigh
assignmentsChromaffin_cells.Chrom_SOX2_statusHigh.DICER1-AS1	-0.756391	3.31944889	-2.9787019	0.00391279	0.01213092	-2.4661615	DICER1-AS1	assignmentsChromaffin_cells.Chrom_SOX2_statusHigh
assignmentsChromaffin_cells.Chrom_SOX2_statusHigh.CCT6B	0.79023184	2.52202927	2.95835709	0.00415082	0.01275792	-2.4664033	CCT6B	assignmentsChromaffin_cells.Chrom_SOX2_statusHigh
assignmentsChromaffin_cells.Chrom_SOX2_statusHigh.CD33	2.51985801	0.49571145	2.79628311	0.00658431	0.01878321	-2.4668516	CD33	assignmentsChromaffin_cells.Chrom_SOX2_statusHigh
assignmentsChromaffin_cells.Chrom_SOX2_statusHigh.VASN	1.44059643	2.11250115	2.93049494	0.00449917	0.01366623	-2.46703	VASN	assignmentsChromaffin_cells.Chrom_SOX2_statusHigh
assignmentsChromaffin_cells.Chrom_SOX2_statusHigh.MIF	2.86689982	5.89156439	3.04061005	0.0032668	0.01042229	-2.4677924	MIF	assignmentsChromaffin_cells.Chrom_SOX2_statusHigh
assignmentsChromaffin_cells.Chrom_SOX2_statusHigh.MPHOSPH8	0.50543467	7.20278281	3.05535269	0.00312506	0.01004805	-2.4687275	MPHOSPH8	assignmentsChromaffin_cells.Chrom_SOX2_statusHigh
assignmentsChromaffin_cells.Chrom_SOX2_statusHigh.GAS7	-2.2987106	6.5231207	-3.0070171	0.00360542	0.01131802	-2.4691738	GAS7	assignmentsChromaffin_cells.Chrom_SOX2_statusHigh
assignmentsChromaffin_cells.Chrom_SOX2_statusHigh.PBX1	-1.3803204	7.04625085	-3.0457982	0.00321721	0.01029072	-2.4733231	PBX1	assignmentsChromaffin_cells.Chrom_SOX2_statusHigh
assignmentsChromaffin_cells.Chrom_SOX2_statusHigh.RAB23	-1.0244098	3.66088343	-2.9639073	0.00408459	0.01258675	-2.4745087	RAB23	assignmentsChromaffin_cells.Chrom_SOX2_statusHigh
assignmentsChromaffin_cells.Chrom_SOX2_statusHigh.SCYL1	0.71556796	4.37412072	2.99968781	0.00368055	0.01152005	-2.4759651	SCYL1	assignmentsChromaffin_cells.Chrom_SOX2_statusHigh
assignmentsChromaffin_cells.Chrom_SOX2_statusHigh.ZNF547	0.75384487	2.36582129	2.95289059	0.00421703	0.01293415	-2.4774863	ZNF547	assignmentsChromaffin_cells.Chrom_SOX2_statusHigh
assignmentsChromaffin_cells.Chrom_SOX2_statusHigh.GINS4	1.06056853	1.2981694	2.90619693	0.00482366	0.01450354	-2.4793072	GINS4	assignmentsChromaffin_cells.Chrom_SOX2_statusHigh
assignmentsChromaffin_cells.Chrom_SOX2_statusHigh.MARVELD2	-1.9614827	0.60550911	-2.8218869	0.00612711	0.01770238	-2.4797415	MARVELD2	assignmentsChromaffin_cells.Chrom_SOX2_statusHigh
assignmentsChromaffin_cells.Chrom_SOX2_statusHigh.EVX1-AS	3.3127495	-0.8237722	2.78057792	0.00688181	0.01951043	-2.4816778	EVX1-AS	assignmentsChromaffin_cells.Chrom_SOX2_statusHigh
assignmentsChromaffin_cells.Chrom_SOX2_statusHigh.NOB1	0.87405722	3.17716272	2.96689474	0.00404934	0.01249391	-2.4831001	NOB1	assignmentsChromaffin_cells.Chrom_SOX2_statusHigh
assignmentsChromaffin_cells.Chrom_SOX2_statusHigh.MORC1	-2.2908819	0.48014031	-2.8178123	0.00619933	0.01787894	-2.4857757	MORC1	assignmentsChromaffin_cells.Chrom_SOX2_statusHigh
assignmentsChromaffin_cells.Chrom_SOX2_statusHigh.DSEL	1.67390237	2.89258459	2.96158776	0.00411517	0.01266292	-2.4858498	DSEL	assignmentsChromaffin_cells.Chrom_SOX2_statusHigh
assignmentsChromaffin_cells.Chrom_SOX2_statusHigh.ELF3-AS1	-1.920056	0.44509913	-2.8050023	0.00642425	0.01840846	-2.4863044	ELF3-AS1	assignmentsChromaffin_cells.Chrom_SOX2_statusHigh
assignmentsChromaffin_cells.Chrom_SOX2_statusHigh.THRA	0.64929678	4.4690325	3.00844506	0.00358748	0.01127929	-2.486699	THRA	assignmentsChromaffin_cells.Chrom_SOX2_statusHigh
assignmentsChromaffin_cells.Chrom_SOX2_statusHigh.FAXC	-1.0679061	3.56763887	-2.9952233	0.00372886	0.01163492	-2.4873198	FAXC	assignmentsChromaffin_cells.Chrom_SOX2_statusHigh
assignmentsChromaffin_cells.Chrom_SOX2_statusHigh.FLT3LG	1.16710604	2.40512635	2.91609977	0.00468864	0.01416129	-2.4877912	FLT3LG	assignmentsChromaffin_cells.Chrom_SOX2_statusHigh
assignmentsChromaffin_cells.Chrom_SOX2_statusHigh.DCDC2B	-1.9170959	0.48895403	-2.7950922	0.00660476	0.01882813	-2.4898013	DCDC2B	assignmentsChromaffin_cells.Chrom_SOX2_statusHigh
assignmentsChromaffin_cells.Chrom_SOX2_statusHigh.PRXL2C	0.79451917	4.54250755	2.99921314	0.00368566	0.01153154	-2.4914567	PRXL2C	assignmentsChromaffin_cells.Chrom_SOX2_statusHigh
assignmentsChromaffin_cells.Chrom_SOX2_statusHigh.SIAH2	0.95907855	5.25509401	3.01545453	0.00351456	0.01107833	-2.4914638	SIAH2	assignmentsChromaffin_cells.Chrom_SOX2_statusHigh
assignmentsChromaffin_cells.Chrom_SOX2_statusHigh.DNM1L	-0.4469615	7.11444903	-3.0417298	0.0032533	0.01038956	-2.49472	DNM1L	assignmentsChromaffin_cells.Chrom_SOX2_statusHigh
assignmentsChromaffin_cells.Chrom_SOX2_statusHigh.LZTS3	-0.7654077	2.34086288	-2.9490797	0.00426376	0.01306249	-2.4954154	LZTS3	assignmentsChromaffin_cells.Chrom_SOX2_statusHigh
assignmentsChromaffin_cells.Chrom_SOX2_statusHigh.LINC01238	-1.2881098	0.86498561	-2.8679527	0.00537948	0.01584503	-2.4955928	LINC01238	assignmentsChromaffin_cells.Chrom_SOX2_statusHigh
assignmentsChromaffin_cells.Chrom_SOX2_statusHigh.TPP2	-0.4978011	6.75257031	-3.0216173	0.00345159	0.01090556	-2.4964834	TPP2	assignmentsChromaffin_cells.Chrom_SOX2_statusHigh
assignmentsChromaffin_cells.Chrom_SOX2_statusHigh.LINC01322	-1.6647443	1.45866835	-2.8751275	0.00527182	0.01559376	-2.4965918	LINC01322	assignmentsChromaffin_cells.Chrom_SOX2_statusHigh
assignmentsChromaffin_cells.Chrom_SOX2_statusHigh.LRP2BP	-1.1434723	5.06119469	-3.0060425	0.00361352	0.01133899	-2.4966689	LRP2BP	assignmentsChromaffin_cells.Chrom_SOX2_statusHigh
assignmentsChromaffin_cells.Chrom_SOX2_statusHigh.SOWAHC	1.00800246	1.18413692	2.90148048	0.00488922	0.01465943	-2.4984007	SOWAHC	assignmentsChromaffin_cells.Chrom_SOX2_statusHigh
assignmentsChromaffin_cells.Chrom_SOX2_statusHigh.DGKI	-1.8945444	5.20782101	-2.9910392	0.00377767	0.01176204	-2.4989503	DGKI	assignmentsChromaffin_cells.Chrom_SOX2_statusHigh
assignmentsChromaffin_cells.Chrom_SOX2_statusHigh.RSBN1L	-0.4563074	7.1375886	-3.0426996	0.00324401	0.01036609	-2.5003648	RSBN1L	assignmentsChromaffin_cells.Chrom_SOX2_statusHigh
assignmentsChromaffin_cells.Chrom_SOX2_statusHigh.SHANK3	-1.0958392	3.96405947	-2.9541144	0.00420212	0.01289662	-2.5029762	SHANK3	assignmentsChromaffin_cells.Chrom_SOX2_statusHigh
assignmentsChromaffin_cells.Chrom_SOX2_statusHigh.ARL4C	1.57146554	5.18331635	2.99583816	0.00372515	0.0116256	-2.5049096	ARL4C	assignmentsChromaffin_cells.Chrom_SOX2_statusHigh
assignmentsChromaffin_cells.Chrom_SOX2_statusHigh.PNKP	0.61649829	5.10990875	3.00009194	0.00367621	0.0115087	-2.5052135	PNKP	assignmentsChromaffin_cells.Chrom_SOX2_statusHigh
assignmentsChromaffin_cells.Chrom_SOX2_statusHigh.MSANTD4	0.59946439	4.57797978	3.01169463	0.0035535	0.01118569	-2.5056236	MSANTD4	assignmentsChromaffin_cells.Chrom_SOX2_statusHigh
assignmentsChromaffin_cells.Chrom_SOX2_statusHigh.RHOU	1.27613571	4.20327929	2.99090932	0.00377722	0.01176204	-2.5064817	RHOU	assignmentsChromaffin_cells.Chrom_SOX2_statusHigh
assignmentsChromaffin_cells.Chrom_SOX2_statusHigh.FAM177A1	0.7714678	6.37534672	3.02518811	0.00341559	0.01082378	-2.507536	FAM177A1	assignmentsChromaffin_cells.Chrom_SOX2_statusHigh
assignmentsChromaffin_cells.Chrom_SOX2_statusHigh.RNASEL	0.61743164	3.44527571	2.96610002	0.00405869	0.01251553	-2.507942	RNASEL	assignmentsChromaffin_cells.Chrom_SOX2_statusHigh

assignmentsChromaffin_cells.Chrom_SOX2_statusHigh.SENP6	-0.5237415	8.20813416	-3.0512329	0.00316333	0.01014874	-2.5106457	SENP6	assignmentsChromaffin_cells.Chrom_SOX2_statusHigh
assignmentsChromaffin_cells.Chrom_SOX2_statusHigh.AGGF1	0.55938258	5.28841767	3.00582262	0.00361512	0.01134181	-2.5120228	AGGF1	assignmentsChromaffin_cells.Chrom_SOX2_statusHigh
assignmentsChromaffin_cells.Chrom_SOX2_statusHigh.MAK16	0.71560899	2.9719701	2.9459594	0.00430237	0.01316822	-2.5142461	MAK16	assignmentsChromaffin_cells.Chrom_SOX2_statusHigh
assignmentsChromaffin_cells.Chrom_SOX2_statusHigh.EIF2A	0.71492882	5.83623736	3.01657647	0.00350302	0.01104629	-2.5150102	EIF2A	assignmentsChromaffin_cells.Chrom_SOX2_statusHigh
assignmentsChromaffin_cells.Chrom_SOX2_statusHigh.FAM219A	-0.8598829	6.1944886	-3.0217469	0.00345028	0.01090356	-2.5159284	FAM219A	assignmentsChromaffin_cells.Chrom_SOX2_statusHigh
assignmentsChromaffin_cells.Chrom_SOX2_statusHigh.NDUFS2	0.917867	5.81431211	3.01943096	0.00347381	0.01097144	-2.5160134	NDUFS2	assignmentsChromaffin_cells.Chrom_SOX2_statusHigh
assignmentsChromaffin_cells.Chrom_SOX2_statusHigh.ZCCHC12	2.21186902	0.19022998	2.85461468	0.00559045	0.01637542	-2.516674	ZCCHC12	assignmentsChromaffin_cells.Chrom_SOX2_statusHigh
assignmentsChromaffin_cells.Chrom_SOX2_statusHigh.HCG25	-0.8649737	2.54597081	-2.9197408	0.00463988	0.01403252	-2.5181935	HCG25	assignmentsChromaffin_cells.Chrom_SOX2_statusHigh
assignmentsChromaffin_cells.Chrom_SOX2_statusHigh.C16orf89	-2.0270317	0.32828857	-2.763496	0.00721177	0.02027031	-2.5203934	C16orf89	assignmentsChromaffin_cells.Chrom_SOX2_statusHigh
assignmentsChromaffin_cells.Chrom_SOX2_statusHigh.CMTM6	0.93132869	5.12623187	2.9670469	0.00404755	0.0124932	-2.5206829	CMTM6	assignmentsChromaffin_cells.Chrom_SOX2_statusHigh
assignmentsChromaffin_cells.Chrom_SOX2_statusHigh.CD52	2.42294066	1.03075328	2.77387184	0.00700829	0.01977462	-2.5213875	CD52	assignmentsChromaffin_cells.Chrom_SOX2_statusHigh
assignmentsChromaffin_cells.Chrom_SOX2_statusHigh.SEC16A	-0.6317301	5.55131582	-2.990447	0.00378119	0.01177072	-2.5231959	SEC16A	assignmentsChromaffin_cells.Chrom_SOX2_statusHigh
assignmentsChromaffin_cells.Chrom_SOX2_statusHigh.PEX11A	0.85206101	2.08498178	2.92918929	0.00451553	0.01369801	-2.5233071	PEX11A	assignmentsChromaffin_cells.Chrom_SOX2_statusHigh
assignmentsChromaffin_cells.Chrom_SOX2_statusHigh.SH3YL1	-0.6436065	5.67187308	-3.0069593	0.00360312	0.01131521	-2.5239688	SH3YL1	assignmentsChromaffin_cells.Chrom_SOX2_statusHigh
assignmentsChromaffin_cells.Chrom_SOX2_statusHigh.TBXA2R	1.30934448	0.47869748	2.77387071	0.00700704	0.01977458	-2.5253685	TBXA2R	assignmentsChromaffin_cells.Chrom_SOX2_statusHigh
assignmentsChromaffin_cells.Chrom_SOX2_statusHigh.EPS15L1	-0.5253942	6.09219776	-3.0068811	0.00360394	0.01131559	-2.5287272	EPS15L1	assignmentsChromaffin_cells.Chrom_SOX2_statusHigh
assignmentsChromaffin_cells.Chrom_SOX2_statusHigh.IPO9	-0.4656237	6.50489424	-3.0223477	0.0034442	0.01088863	-2.528755	IPO9	assignmentsChromaffin_cells.Chrom_SOX2_statusHigh
assignmentsChromaffin_cells.Chrom_SOX2_statusHigh.RGS11	-1.622531	3.6129791	-2.9543096	0.00420281	0.01289662	-2.529167	RGS11	assignmentsChromaffin_cells.Chrom_SOX2_statusHigh
assignmentsChromaffin_cells.Chrom_SOX2_statusHigh.SELENBP1	1.89233733	-0.0163778	2.73262872	0.00785371	0.02181879	-2.5309053	SELENBP1	assignmentsChromaffin_cells.Chrom_SOX2_statusHigh
assignmentsChromaffin_cells.Chrom_SOX2_statusHigh.CYP20A1	-0.6041511	6.20559856	-2.9971012	0.00370847	0.01158031	-2.5310691	CYP20A1	assignmentsChromaffin_cells.Chrom_SOX2_statusHigh
assignmentsChromaffin_cells.Chrom_SOX2_statusHigh.ADGRG2	-1.9843875	1.42152321	-2.8071545	0.00638666	0.01834168	-2.5310983	ADGRG2	assignmentsChromaffin_cells.Chrom_SOX2_statusHigh
assignmentsChromaffin_cells.Chrom_SOX2_statusHigh.SLC27A5	1.22263061	2.94098449	2.955386	0.00418699	0.01285679	-2.5323514	SLC27A5	assignmentsChromaffin_cells.Chrom_SOX2_statusHigh
assignmentsChromaffin_cells.Chrom_SOX2_statusHigh.TUBB2B	1.42682366	1.48776699	2.92246335	0.00460471	0.01393403	-2.5328172	TUBB2B	assignmentsChromaffin_cells.Chrom_SOX2_statusHigh
assignmentsChromaffin_cells.Chrom_SOX2_statusHigh.MEG8	-2.8515459	8.49498277	-3.0530598	0.00314899	0.01010676	-2.5341526	MEG8	assignmentsChromaffin_cells.Chrom_SOX2_statusHigh
assignmentsChromaffin_cells.Chrom_SOX2_statusHigh.PXK	-0.6501818	5.63689002	-2.9833914	0.00385974	0.01198965	-2.5357241	PXK	assignmentsChromaffin_cells.Chrom_SOX2_statusHigh
assignmentsChromaffin_cells.Chrom_SOX2_statusHigh.EED	-0.5962935	5.20643611	-2.9754299	0.0039502	0.01222563	-2.5385767	EED	assignmentsChromaffin_cells.Chrom_SOX2_statusHigh
assignmentsChromaffin_cells.Chrom_SOX2_statusHigh.GPR137C	-1.1731787	5.35656232	-2.9866364	0.00382431	0.01189109	-2.541437	GPR137C	assignmentsChromaffin_cells.Chrom_SOX2_statusHigh
assignmentsChromaffin_cells.Chrom_SOX2_statusHigh.ZNF157	-1.2323238	1.24615477	-2.8801524	0.00519608	0.01540382	-2.5436116	ZNF157	assignmentsChromaffin_cells.Chrom_SOX2_statusHigh
assignmentsChromaffin_cells.Chrom_SOX2_statusHigh.CLCN3	-0.6616577	7.22303056	-3.0292917	0.00337464	0.01070672	-2.5450002	CLCN3	assignmentsChromaffin_cells.Chrom_SOX2_statusHigh
assignmentsChromaffin_cells.Chrom_SOX2_statusHigh.RWDD2B	0.82116081	2.4245576	2.92397345	0.00458379	0.01388382	-2.5465693	RWDD2B	assignmentsChromaffin_cells.Chrom_SOX2_statusHigh
assignmentsChromaffin_cells.Chrom_SOX2_statusHigh.UNC93B1	1.65519145	1.82108572	2.77438524	0.00699703	0.01975527	-2.5471531	UNC93B1	assignmentsChromaffin_cells.Chrom_SOX2_statusHigh
assignmentsChromaffin_cells.Chrom_SOX2_statusHigh.LINC02125	-2.6826291	-0.628033	-2.7225946	0.00807338	0.02234356	-2.5478399	LINC02125	assignmentsChromaffin_cells.Chrom_SOX2_statusHigh
assignmentsChromaffin_cells.Chrom_SOX2_statusHigh.WRNIP1	-0.611322	5.1135973	-2.9740596	0.00396596	0.01226496	-2.5479464	WRNIP1	assignmentsChromaffin_cells.Chrom_SOX2_statusHigh
assignmentsChromaffin_cells.Chrom_SOX2_statusHigh.CACUL1	-0.6316225	7.17119227	-3.0234604	0.00343296	0.01087026	-2.5489508	CACUL1	assignmentsChromaffin_cells.Chrom_SOX2_statusHigh
assignmentsChromaffin_cells.Chrom_SOX2_statusHigh.TMUB1	1.01708757	3.73944799	2.96876378	0.00402744	0.01244068	-2.5505342	TMUB1	assignmentsChromaffin_cells.Chrom_SOX2_statusHigh
assignmentsChromaffin_cells.Chrom_SOX2_statusHigh.NACC2	-0.9719761	4.37739549	-2.9372162	0.00441231	0.01343549	-2.5516814	NACC2	assignmentsChromaffin_cells.Chrom_SOX2_statusHigh
assignmentsChromaffin_cells.Chrom_SOX2_statusHigh.DCP1A	-0.6521285	6.37224994	-2.9990498	0.00368742	0.01153255	-2.5526979	DCP1A	assignmentsChromaffin_cells.Chrom_SOX2_statusHigh
assignmentsChromaffin_cells.Chrom_SOX2_statusHigh.SPSB2	0.91957982	1.7759618	2.89539186	0.00497507	0.01486404	-2.5527934	SPSB2	assignmentsChromaffin_cells.Chrom_SOX2_statusHigh
assignmentsChromaffin_cells.Chrom_SOX2_statusHigh.SLC24A5	-1.7301521	0.01044045	-2.7970288	0.00656912	0.01875395	-2.5534124	SLC24A5	assignmentsChromaffin_cells.Chrom_SOX2_statusHigh
assignmentsChromaffin_cells.Chrom_SOX2_statusHigh.PPP1R26-AS	-1.0717659	1.85844023	-2.8891845	0.00506402	0.01508477	-2.5536714	PPP1R26-AS	assignmentsChromaffin_cells.Chrom_SOX2_statusHigh
assignmentsChromaffin_cells.Chrom_SOX2_statusHigh.GNA12	-0.7871918	6.7222528	-3.0011782	0.00366456	0.01147446	-2.554121	GNA12	assignmentsChromaffin_cells.Chrom_SOX2_statusHigh
assignmentsChromaffin_cells.Chrom_SOX2_statusHigh.SNX31	2.08573442	0.91253232	2.82000934	0.0061612	0.01778177	-2.5543658	SNX31	assignmentsChromaffin_cells.Chrom_SOX2_statusHigh
assignmentsChromaffin_cells.Chrom_SOX2_statusHigh.GDPD5	1.58879245	3.88463147	2.95920284	0.00414384	0.0127438	-2.5558857	GDPD5	assignmentsChromaffin_cells.Chrom_SOX2_statusHigh
assignmentsChromaffin_cells.Chrom_SOX2_statusHigh.C9orf85	-0.7503047	5.52800111	-2.9763564	0.00393957	0.01219981	-2.5559816	C9orf85	assignmentsChromaffin_cells.Chrom_SOX2_statusHigh
assignmentsChromaffin_cells.Chrom_SOX2_statusHigh.PNMA2	0.95511	3.46389646	2.97768703	0.00392435	0.01215974	-2.5594804	PNMA2	assignmentsChromaffin_cells.Chrom_SOX2_statusHigh
assignmentsChromaffin_cells.Chrom_SOX2_statusHigh.DRD4	-1.4511678	0.62083798	-2.8070668	0.00638722	0.01834168	-2.5628922	DRD4	assignmentsChromaffin_cells.Chrom_SOX2_statusHigh
assignmentsChromaffin_cells.Chrom_SOX2_statusHigh.SDCBP2	1.49520232	1.57763006	2.85758847	0.00553997	0.01624315	-2.5632158	SDCBP2	assignmentsChromaffin_cells.Chrom_SOX2_statusHigh
assignmentsChromaffin_cells.Chrom_SOX2_statusHigh.AMY2B	-1.0086896	3.61624241	-2.9225286	0.00460287	0.0139324	-2.5639792	AMY2B	assignmentsChromaffin_cells.Chrom_SOX2_statusHigh
assignmentsChromaffin_cells.Chrom_SOX2_statusHigh.TBC1D2B	-0.7108925	6.17374659	-2.9661835	0.00405771	0.01251491	-2.564991	TBC1D2B	assignmentsChromaffin_cells.Chrom_SOX2_statusHigh
assignmentsChromaffin_cells.Chrom_SOX2_statusHigh.SLC41A1	0.8075982	4.20325015	2.95913163	0.00414152	0.0127391	-2.5678768	SLC41A1	assignmentsChromaffin_cells.Chrom_SOX2_statusHigh
assignmentsChromaffin_cells.Chrom_SOX2_statusHigh.FJX1	1.74825727	0.49401934	2.74524134	0.00758535	0.02117559	-2.5687038	FJX1	assignmentsChromaffin_cells.Chrom_SOX2_statusHigh
assignmentsChromaffin_cells.Chrom_SOX2_statusHigh.SERBP1	0.89427979	6.0951915	2.99166005	0.00376784	0.01174054	-2.5736044	SERBP1	assignmentsChromaffin_cells.Chrom_SOX2_statusHigh
assignmentsChromaffin_cells.Chrom_SOX2_statusHigh.SURF4	0.64881933	5.9722254	2.99898277	0.00368814	0.01153255	-2.5745971	SURF4	assignmentsChromaffin_cells.Chrom_SOX2_statusHigh
assignmentsChromaffin_cells.Chrom_SOX2_statusHigh.NPIPA1	-1.0915124	3.9395551	-2.9404487	0.00437137	0.0133412	-2.576745	NPIPA1	assignmentsChromaffin_cells.Chrom_SOX2_statusHigh
assignmentsChromaffin_cells.Chrom_SOX2_statusHigh.ASNSD1	0.89392839	3.2642347	2.93793879	0.00440313	0.01341007	-2.5768254	ASNSD1	assignmentsChromaffin_cells.Chrom_SOX2_statusHigh
assignmentsChromaffin_cells.Chrom_SOX2_statusHigh.CFLAR	-0.8176788	7.13756999	-2.9704422	0.00400786	0.01238259	-2.5783419	CFLAR	assignmentsChromaffin_cells.Chrom_SOX2_statusHigh
assignmentsChromaffin_cells.Chrom_SOX2_statusHigh.DNMBP	-1.1288249	5.35561313	-2.9336874	0.00445742	0.01355996	-2.5794891	DNMBP	assignmentsChromaffin_cells.Chrom_SOX2_statusHigh
assignmentsChromaffin_cells.Chrom_SOX2_statusHigh.LINC01970	1.39994416	0.53224278	2.80688593	0.00639046	0.01834566	-2.5796322	LINC01970	assignmentsChromaffin_cells.Chrom_SOX2_statusHigh
assignmentsChromaffin_cells.Chrom_SOX2_statusHigh.GPX8	1.85643947	0.92337403	2.75385141	0.00740699	0.02075718	-2.5824899	GPX8	assignmentsChromaffin_cells.Chrom_SOX2_statusHigh
assignmentsChromaffin_cells.Chrom_SOX2_statusHigh.UNC5D	-4.0363624	4.10350689	-2.9138745	0.00472211	0.01422755	-2.5825867	UNC5D	assignmentsChromaffin_cells.Chrom_SOX2_statusHigh
assignmentsChromaffin_cells.Chrom_SOX2_statusHigh.VSNL1	2.61501691	2.7131059	2.9570662	0.00416956	0.01280775	-2.5849114	VSNL1	assignmentsChromaffin_cells.Chrom_SOX2_statusHigh
assignmentsChromaffin_cells.Chrom_SOX2_statusHigh.PTPRN2	-1.1821033	7.98049501	-3.0364406	0.00330664	0.01052409	-2.5881446	PTPRN2	assignmentsChromaffin_cells.Chrom_SOX2_statusHigh
assignmentsChromaffin_cells.Chrom_SOX2_statusHigh.EXOC1	-0.4812562	6.45372636	-2.9917799	0.00376652	0.01173872	-2.5891598	EXOC1	assignmentsChromaffin_cells.Chrom_SOX2_statusHigh
assignmentsChromaffin_cells.Chrom_SOX2_statusHigh.TCF4-AS1	-1.3739333	3.25189832	-2.901237	0.0048935	0.01466953	-2.5911426	TCF4-AS1	assignmentsChromaffin_cells.Chrom_SOX2_statusHigh

assignmentsChromaffin_cells.Chrom_SOX2_statusHigh.SQLE	0.85766602	4.82766003	2.97111526	0.00400003	0.01236079	-2.5928755	SQLE	assignmentsChromaffin_cells.Chrom_SOX2_statusHigh
assignmentsChromaffin_cells.Chrom_SOX2_statusHigh.RUNDC1	0.50375435	3.80903047	2.94967764	0.00425639	0.01304491	-2.593004	RUNDC1	assignmentsChromaffin_cells.Chrom_SOX2_statusHigh
assignmentsChromaffin_cells.Chrom_SOX2_statusHigh.DENR	0.4230206	5.76067868	2.98097461	0.003887	0.01206496	-2.5932223	DENR	assignmentsChromaffin_cells.Chrom_SOX2_statusHigh
assignmentsChromaffin_cells.Chrom_SOX2_statusHigh.ZNF883	-1.4554356	2.08921917	-2.8665192	0.00540169	0.01589876	-2.5936657	ZNF883	assignmentsChromaffin_cells.Chrom_SOX2_statusHigh
assignmentsChromaffin_cells.Chrom_SOX2_statusHigh.IL18BP	1.04944915	2.95607093	2.89764731	0.00494311	0.01478231	-2.5938345	IL18BP	assignmentsChromaffin_cells.Chrom_SOX2_statusHigh
assignmentsChromaffin_cells.Chrom_SOX2_statusHigh.KAT6B	-0.7213495	7.59538876	-3.0113537	0.00355705	0.01119467	-2.5946691	KAT6B	assignmentsChromaffin_cells.Chrom_SOX2_statusHigh
assignmentsChromaffin_cells.Chrom_SOX2_statusHigh.CCDC22	0.80469684	2.43296433	2.89197868	0.0050238	0.01498451	-2.5966313	CCDC22	assignmentsChromaffin_cells.Chrom_SOX2_statusHigh
assignmentsChromaffin_cells.Chrom_SOX2_statusHigh.YPEL5	0.93133165	6.11039835	2.98978748	0.00378847	0.01179109	-2.5971194	YPEL5	assignmentsChromaffin_cells.Chrom_SOX2_statusHigh
assignmentsChromaffin_cells.Chrom_SOX2_statusHigh.SCO1	0.78173538	4.23340603	2.94318261	0.00433701	0.01325653	-2.6001241	SCO1	assignmentsChromaffin_cells.Chrom_SOX2_statusHigh
assignmentsChromaffin_cells.Chrom_SOX2_statusHigh.SETD7	-0.6435158	5.66606744	-2.9712103	0.00399893	0.01235976	-2.6013719	SETD7	assignmentsChromaffin_cells.Chrom_SOX2_statusHigh
assignmentsChromaffin_cells.Chrom_SOX2_statusHigh.PSMA3	0.47893748	5.94004898	2.98415006	0.00385123	0.01196551	-2.604268	PSMA3	assignmentsChromaffin_cells.Chrom_SOX2_statusHigh
assignmentsChromaffin_cells.Chrom_SOX2_statusHigh.ATP8A1	-0.9601001	6.49108959	-2.9882708	0.00380526	0.01183646	-2.6048642	ATP8A1	assignmentsChromaffin_cells.Chrom_SOX2_statusHigh
assignmentsChromaffin_cells.Chrom_SOX2_statusHigh.ZC3HC1	-0.5879895	4.33773729	-2.9479829	0.00427729	0.01309395	-2.6049721	ZC3HC1	assignmentsChromaffin_cells.Chrom_SOX2_statusHigh
assignmentsChromaffin_cells.Chrom_SOX2_statusHigh.MIR3936HG	-0.8661018	5.18522922	-2.9801496	0.00389634	0.01208694	-2.6062419	MIR3936HG	assignmentsChromaffin_cells.Chrom_SOX2_statusHigh
assignmentsChromaffin_cells.Chrom_SOX2_statusHigh.WDFY3-AS2	0.70832533	2.98807021	2.92798935	0.00453115	0.01373995	-2.606262	WDFY3-AS2	assignmentsChromaffin_cells.Chrom_SOX2_statusHigh
assignmentsChromaffin_cells.Chrom_SOX2_statusHigh.HAAO	-1.8129803	1.87653403	-2.765186	0.00717805	0.02018409	-2.6067233	HAAO	assignmentsChromaffin_cells.Chrom_SOX2_statusHigh
assignmentsChromaffin_cells.Chrom_SOX2_statusHigh.NUDT12	0.66385345	3.36650867	2.93500737	0.0044405	0.01351104	-2.6071217	NUDT12	assignmentsChromaffin_cells.Chrom_SOX2_statusHigh
assignmentsChromaffin_cells.Chrom_SOX2_statusHigh.HYI	1.58622912	2.90070122	2.89685122	0.00495604	0.01481269	-2.6100246	HYI	assignmentsChromaffin_cells.Chrom_SOX2_statusHigh
assignmentsChromaffin_cells.Chrom_SOX2_statusHigh.GK-AS1	-1.9386253	0.92154701	-2.7602604	0.00727674	0.02043501	-2.6111201	GK-AS1	assignmentsChromaffin_cells.Chrom_SOX2_statusHigh
assignmentsChromaffin_cells.Chrom_SOX2_statusHigh.ARHGAP31	1.90871998	5.21326711	2.84545555	0.00573632	0.0167119	-2.6127319	ARHGAP31	assignmentsChromaffin_cells.Chrom_SOX2_statusHigh
assignmentsChromaffin_cells.Chrom_SOX2_statusHigh.PSTPIP2	-1.2511391	3.98623808	-2.8974915	0.00494531	0.01478616	-2.6146327	PSTPIP2	assignmentsChromaffin_cells.Chrom_SOX2_statusHigh
assignmentsChromaffin_cells.Chrom_SOX2_statusHigh.LTN1	-0.4288943	6.2157759	-2.9777405	0.00392374	0.01215974	-2.6146347	LTN1	assignmentsChromaffin_cells.Chrom_SOX2_statusHigh
assignmentsChromaffin_cells.Chrom_SOX2_statusHigh.ALKBH5	0.87150003	5.12356201	2.96387858	0.00408493	0.01258675	-2.6180411	ALKBH5	assignmentsChromaffin_cells.Chrom_SOX2_statusHigh
assignmentsChromaffin_cells.Chrom_SOX2_statusHigh.GOLGA8R	-1.1461908	1.11053866	-2.8149811	0.00624708	0.01799674	-2.6182662	GOLGA8R	assignmentsChromaffin_cells.Chrom_SOX2_statusHigh
assignmentsChromaffin_cells.Chrom_SOX2_statusHigh.BCDIN3D	0.74194282	2.43694831	2.90306406	0.00486712	0.01460682	-2.6188192	BCDIN3D	assignmentsChromaffin_cells.Chrom_SOX2_statusHigh
assignmentsChromaffin_cells.Chrom_SOX2_statusHigh.WBP4	0.65340228	5.51849638	2.97563752	0.00394781	0.01222061	-2.6196239	WBP4	assignmentsChromaffin_cells.Chrom_SOX2_statusHigh
assignmentsChromaffin_cells.Chrom_SOX2_statusHigh.GPATCH3	0.66842038	2.43826136	2.88067689	0.00518832	0.01539023	-2.6204789	GPATCH3	assignmentsChromaffin_cells.Chrom_SOX2_statusHigh
assignmentsChromaffin_cells.Chrom_SOX2_statusHigh.KLHL20	-0.464831	6.15444305	-2.9741789	0.00396459	0.01226307	-2.6251993	KLHL20	assignmentsChromaffin_cells.Chrom_SOX2_statusHigh
assignmentsChromaffin_cells.Chrom_SOX2_statusHigh.FBXW2	-0.5291558	5.46434986	-2.9480672	0.00427625	0.01309327	-2.625396	FBXW2	assignmentsChromaffin_cells.Chrom_SOX2_statusHigh
assignmentsChromaffin_cells.Chrom_SOX2_statusHigh.NECAP2	0.86490674	4.21785886	2.91033031	0.00476687	0.01434624	-2.6290503	NECAP2	assignmentsChromaffin_cells.Chrom_SOX2_statusHigh
assignmentsChromaffin_cells.Chrom_SOX2_statusHigh.COL13A1	2.29982193	1.07561443	2.72155086	0.00809736	0.02239832	-2.6294308	COL13A1	assignmentsChromaffin_cells.Chrom_SOX2_statusHigh
assignmentsChromaffin_cells.Chrom_SOX2_statusHigh.AGPAT2	2.09757938	2.28077319	2.83405999	0.0059247	0.01720137	-2.6306519	AGPAT2	assignmentsChromaffin_cells.Chrom_SOX2_statusHigh
assignmentsChromaffin_cells.Chrom_SOX2_statusHigh.STEAP2	-1.228203	3.60539055	-2.929961	0.00450648	0.01368065	-2.6311649	STEAP2	assignmentsChromaffin_cells.Chrom_SOX2_statusHigh
assignmentsChromaffin_cells.Chrom_SOX2_statusHigh.GATA1	-2.0867483	-0.7752118	-2.6863022	0.00891555	0.02424911	-2.6313012	GATA1	assignmentsChromaffin_cells.Chrom_SOX2_statusHigh
assignmentsChromaffin_cells.Chrom_SOX2_statusHigh.NUPR1	2.22128484	2.04922619	2.79631571	0.00658561	0.01878357	-2.6317117	NUPR1	assignmentsChromaffin_cells.Chrom_SOX2_statusHigh
assignmentsChromaffin_cells.Chrom_SOX2_statusHigh.CLTCL1	-0.8653301	3.9790914	-2.9187286	0.00465339	0.01406541	-2.63173	CLTCL1	assignmentsChromaffin_cells.Chrom_SOX2_statusHigh
assignmentsChromaffin_cells.Chrom_SOX2_statusHigh.SPRED2	-0.7671341	6.82173755	-2.9730099	0.00397808	0.01229946	-2.632289	SPRED2	assignmentsChromaffin_cells.Chrom_SOX2_statusHigh
assignmentsChromaffin_cells.Chrom_SOX2_statusHigh.LINC02381	1.32299999	2.67967389	2.88984933	0.00505523	0.01506705	-2.6340534	LINC02381	assignmentsChromaffin_cells.Chrom_SOX2_statusHigh
assignmentsChromaffin_cells.Chrom_SOX2_statusHigh.DGKD	-0.8132456	6.70678516	-2.9567466	0.00417022	0.01280775	-2.6351268	DGKD	assignmentsChromaffin_cells.Chrom_SOX2_statusHigh
assignmentsChromaffin_cells.Chrom_SOX2_statusHigh.RMDN2-AS1	-2.0656111	1.08560612	-2.7501863	0.00748355	0.02094243	-2.6362962	RMDN2-AS1	assignmentsChromaffin_cells.Chrom_SOX2_statusHigh
assignmentsChromaffin_cells.Chrom_SOX2_statusHigh.GBGT1	1.66337222	1.26387836	2.70096488	0.0085661	0.02347627	-2.638416	GBGT1	assignmentsChromaffin_cells.Chrom_SOX2_statusHigh
assignmentsChromaffin_cells.Chrom_SOX2_statusHigh.IFIT3	1.58321336	3.42602747	2.87472821	0.00527844	0.01561047	-2.6399977	IFIT3	assignmentsChromaffin_cells.Chrom_SOX2_statusHigh
assignmentsChromaffin_cells.Chrom_SOX2_statusHigh.SYPL1	0.78536808	4.85158373	2.94470541	0.00431799	0.01320592	-2.6403122	SYPL1	assignmentsChromaffin_cells.Chrom_SOX2_statusHigh
assignmentsChromaffin_cells.Chrom_SOX2_statusHigh.CDHR3	-1.0794202	5.60899469	-2.9632445	0.00409322	0.01260747	-2.6413276	CDHR3	assignmentsChromaffin_cells.Chrom_SOX2_statusHigh
assignmentsChromaffin_cells.Chrom_SOX2_statusHigh.LDLRAD3	-1.3569921	6.30385589	-2.9399947	0.00437873	0.01335604	-2.6424323	LDLRAD3	assignmentsChromaffin_cells.Chrom_SOX2_statusHigh
assignmentsChromaffin_cells.Chrom_SOX2_statusHigh.ZNF518B	1.41173519	3.37254031	2.90290089	0.00487086	0.01461531	-2.6426887	ZNF518B	assignmentsChromaffin_cells.Chrom_SOX2_statusHigh
assignmentsChromaffin_cells.Chrom_SOX2_statusHigh.TSPAN2	2.71631874	0.71703774	2.70892399	0.00838369	0.02305515	-2.6437864	TSPAN2	assignmentsChromaffin_cells.Chrom_SOX2_statusHigh
assignmentsChromaffin_cells.Chrom_SOX2_statusHigh.LRRFIP2	-0.5164255	7.27904396	-2.9808304	0.00388863	0.01206769	-2.6443259	LRRFIP2	assignmentsChromaffin_cells.Chrom_SOX2_statusHigh
assignmentsChromaffin_cells.Chrom_SOX2_statusHigh.ZBED5-AS1	0.86606928	2.37095352	2.88098722	0.00518374	0.01538146	-2.6450881	ZBED5-AS1	assignmentsChromaffin_cells.Chrom_SOX2_statusHigh
assignmentsChromaffin_cells.Chrom_SOX2_statusHigh.YPEL3	1.21858111	4.37519947	2.9304113	0.00450064	0.01366811	-2.645174	YPEL3	assignmentsChromaffin_cells.Chrom_SOX2_statusHigh
assignmentsChromaffin_cells.Chrom_SOX2_statusHigh.USP4	-0.6381779	5.93009994	-2.9393954	0.00438467	0.01336908	-2.6453414	USP4	assignmentsChromaffin_cells.Chrom_SOX2_statusHigh
assignmentsChromaffin_cells.Chrom_SOX2_statusHigh.TREM1	2.76283784	0.27895106	2.6973207	0.00865358	0.02367557	-2.6456099	TREM1	assignmentsChromaffin_cells.Chrom_SOX2_statusHigh
assignmentsChromaffin_cells.Chrom_SOX2_statusHigh.MCM7	0.97511508	3.59355108	2.89830396	0.00493384	0.0147601	-2.6480013	MCM7	assignmentsChromaffin_cells.Chrom_SOX2_statusHigh
assignmentsChromaffin_cells.Chrom_SOX2_statusHigh.TTF2	0.75151003	4.23738037	2.92085328	0.00462508	0.01399302	-2.6482258	TTF2	assignmentsChromaffin_cells.Chrom_SOX2_statusHigh
assignmentsChromaffin_cells.Chrom_SOX2_statusHigh.RMDN2	-0.9406011	3.8216058	-2.8900661	0.0050513	0.01505813	-2.6491394	RMDN2	assignmentsChromaffin_cells.Chrom_SOX2_statusHigh
assignmentsChromaffin_cells.Chrom_SOX2_statusHigh.TPM2	1.55603512	3.58669326	2.84022427	0.00581987	0.01692151	-2.6514295	TPM2	assignmentsChromaffin_cells.Chrom_SOX2_statusHigh
assignmentsChromaffin_cells.Chrom_SOX2_statusHigh.LINC01505	-1.2128921	5.16090933	-2.9400595	0.00437728	0.01335416	-2.6517693	LINC01505	assignmentsChromaffin_cells.Chrom_SOX2_statusHigh
assignmentsChromaffin_cells.Chrom_SOX2_statusHigh.PABPC1	1.01579324	7.85202169	2.99190546	0.00376601	0.01173872	-2.6525191	PABPC1	assignmentsChromaffin_cells.Chrom_SOX2_statusHigh
assignmentsChromaffin_cells.Chrom_SOX2_statusHigh.SWT1	-0.4832656	5.36976154	-2.9407411	0.00436768	0.01333249	-2.6538531	SWT1	assignmentsChromaffin_cells.Chrom_SOX2_statusHigh
assignmentsChromaffin_cells.Chrom_SOX2_statusHigh.MTPAP	-0.5645355	4.16349355	-2.9148977	0.00470484	0.01419685	-2.6545053	MTPAP	assignmentsChromaffin_cells.Chrom_SOX2_statusHigh
assignmentsChromaffin_cells.Chrom_SOX2_statusHigh.MBD2	-0.7125234	6.24508106	-2.9481177	0.00427563	0.01309327	-2.6568699	MBD2	assignmentsChromaffin_cells.Chrom_SOX2_statusHigh
assignmentsChromaffin_cells.Chrom_SOX2_statusHigh.DHRS4	0.84039498	3.12367265	2.89128415	0.00503377	0.01501146	-2.6603352	DHRS4	assignmentsChromaffin_cells.Chrom_SOX2_statusHigh
assignmentsChromaffin_cells.Chrom_SOX2_statusHigh.SLC41A2	-0.7573067	5.92583698	-2.9618512	0.00410901	0.01264881	-2.6615626	SLC41A2	assignmentsChromaffin_cells.Chrom_SOX2_statusHigh
assignmentsChromaffin_cells.Chrom_SOX2_statusHigh.MIR133A1H	-2.2054918	0.17707916	-2.7099907	0.00835726	0.02299034	-2.6615862	MIR133A1HG	assignmentsChromaffin_cells.Chrom_SOX2_statusHigh

assignmentsChromaffin_cells.Chrom_SOX2_statusHigh.BCL9	-0.8149805	4.31907842	-2.9259433	0.0045579	0.01381062	-2.661703	BCL9	assignmentsChromaffin_cells.Chrom_SOX2_statusHigh
assignmentsChromaffin_cells.Chrom_SOX2_statusHigh.AIM2	1.6588227	1.67504485	2.80506036	0.00642423	0.01840846	-2.6621936	AIM2	assignmentsChromaffin_cells.Chrom_SOX2_statusHigh
assignmentsChromaffin_cells.Chrom_SOX2_statusHigh.ZGRF1	-0.8222597	4.12306955	-2.9152114	0.00470061	0.01418942	-2.6636277	ZGRF1	assignmentsChromaffin_cells.Chrom_SOX2_statusHigh
assignmentsChromaffin_cells.Chrom_SOX2_statusHigh.PYM1	0.609548	3.82109846	2.90754083	0.00480513	0.01445324	-2.6637933	PYM1	assignmentsChromaffin_cells.Chrom_SOX2_statusHigh
assignmentsChromaffin_cells.Chrom_SOX2_statusHigh.PPM1A	-0.5697948	6.19958292	-2.958747	0.00414613	0.01274841	-2.664444	PPM1A	assignmentsChromaffin_cells.Chrom_SOX2_statusHigh
assignmentsChromaffin_cells.Chrom_SOX2_statusHigh.PXMP4	0.6295509	2.8995001	2.89922138	0.00492091	0.01472967	-2.6655747	PXMP4	assignmentsChromaffin_cells.Chrom_SOX2_statusHigh
assignmentsChromaffin_cells.Chrom_SOX2_statusHigh.CD300C	2.04617636	-0.6971589	2.6644565	0.00946033	0.02545718	-2.6658155	CD300C	assignmentsChromaffin_cells.Chrom_SOX2_statusHigh
assignmentsChromaffin_cells.Chrom_SOX2_statusHigh.EIF3C	-0.7621066	2.78970168	-2.8613342	0.00548146	0.0160981	-2.6667485	EIF3C	assignmentsChromaffin_cells.Chrom_SOX2_statusHigh
assignmentsChromaffin_cells.Chrom_SOX2_statusHigh.TIGD2	1.03162826	1.19006286	2.82753798	0.00603051	0.01746789	-2.666927	TIGD2	assignmentsChromaffin_cells.Chrom_SOX2_statusHigh
assignmentsChromaffin_cells.Chrom_SOX2_statusHigh.PPP1R14A	2.01760317	1.05915563	2.72076472	0.00811404	0.02243286	-2.6671595	PPP1R14A	assignmentsChromaffin_cells.Chrom_SOX2_statusHigh
assignmentsChromaffin_cells.Chrom_SOX2_statusHigh.YIPF6	0.5355726	5.15753695	2.9505875	0.00424521	0.01301312	-2.6672528	YIPF6	assignmentsChromaffin_cells.Chrom_SOX2_statusHigh
assignmentsChromaffin_cells.Chrom_SOX2_statusHigh.TRABD2B	-2.4281299	2.75785365	-2.7929287	0.00664897	0.01893735	-2.6682142	TRABD2B	assignmentsChromaffin_cells.Chrom_SOX2_statusHigh
assignmentsChromaffin_cells.Chrom_SOX2_statusHigh.ALG13-AS1	-1.9495955	2.42123688	-2.8477194	0.00570071	0.01663233	-2.6690498	ALG13-AS1	assignmentsChromaffin_cells.Chrom_SOX2_statusHigh
assignmentsChromaffin_cells.Chrom_SOX2_statusHigh.TMSB15A	2.27829058	-0.0138708	2.74113608	0.00767367	0.02138124	-2.6699699	TMSB15A	assignmentsChromaffin_cells.Chrom_SOX2_statusHigh
assignmentsChromaffin_cells.Chrom_SOX2_statusHigh.GFOD2	-0.6910461	4.68776258	-2.9146336	0.00470841	0.0141996	-2.6717177	GFOD2	assignmentsChromaffin_cells.Chrom_SOX2_statusHigh
assignmentsChromaffin_cells.Chrom_SOX2_statusHigh.FAM89A	1.34126836	3.12627142	2.89378034	0.00499911	0.0149235	-2.6721371	FAM89A	assignmentsChromaffin_cells.Chrom_SOX2_statusHigh
assignmentsChromaffin_cells.Chrom_SOX2_statusHigh.CSE1L	-0.4481468	5.29111434	-2.9313035	0.00448813	0.01364044	-2.6763232	CSE1L	assignmentsChromaffin_cells.Chrom_SOX2_statusHigh
assignmentsChromaffin_cells.Chrom_SOX2_statusHigh.MFN1	-0.5648227	5.72403102	-2.9382956	0.0043986	0.01339882	-2.6775162	MFN1	assignmentsChromaffin_cells.Chrom_SOX2_statusHigh
assignmentsChromaffin_cells.Chrom_SOX2_statusHigh.HCAR2	2.44691438	-1.1475609	2.6563714	0.00966952	0.02592873	-2.6782367	HCAR2	assignmentsChromaffin_cells.Chrom_SOX2_statusHigh
assignmentsChromaffin_cells.Chrom_SOX2_statusHigh.EIF4G3	-0.8940118	8.74777669	-2.9926118	0.00375739	0.01171483	-2.6784111	EIF4G3	assignmentsChromaffin_cells.Chrom_SOX2_statusHigh
assignmentsChromaffin_cells.Chrom_SOX2_statusHigh.BHLHE41	1.85648283	2.95842886	2.91551249	0.00469998	0.01418942	-2.6785927	BHLHE41	assignmentsChromaffin_cells.Chrom_SOX2_statusHigh
assignmentsChromaffin_cells.Chrom_SOX2_statusHigh.KCNJ2	1.73506617	1.74436396	2.78890438	0.00672103	0.0190985	-2.6787984	KCNJ2	assignmentsChromaffin_cells.Chrom_SOX2_statusHigh
assignmentsChromaffin_cells.Chrom_SOX2_statusHigh.KMT2D	-0.5366011	5.25789619	-2.9267896	0.00454682	0.01378225	-2.6804181	KMT2D	assignmentsChromaffin_cells.Chrom_SOX2_statusHigh
assignmentsChromaffin_cells.Chrom_SOX2_statusHigh.UGGT2	-0.8012097	7.65968533	-2.9820911	0.00387438	0.01202917	-2.6810773	UGGT2	assignmentsChromaffin_cells.Chrom_SOX2_statusHigh
assignmentsChromaffin_cells.Chrom_SOX2_statusHigh.PTENP1-AS	-1.9057565	1.36367424	-2.7901783	0.00669774	0.01904581	-2.6838728	PTENP1-AS	assignmentsChromaffin_cells.Chrom_SOX2_statusHigh
assignmentsChromaffin_cells.Chrom_SOX2_statusHigh.MICALL1	-0.8415988	3.16397075	-2.8567699	0.00555283	0.0162749	-2.6841543	MICALL1	assignmentsChromaffin_cells.Chrom_SOX2_statusHigh
assignmentsChromaffin_cells.Chrom_SOX2_statusHigh.NRAS	0.82850692	3.37448325	2.86888707	0.00536523	0.01580885	-2.6862925	NRAS	assignmentsChromaffin_cells.Chrom_SOX2_statusHigh
assignmentsChromaffin_cells.Chrom_SOX2_statusHigh.OOEP	1.58957758	1.24667646	2.8068837	0.00639151	0.01834566	-2.6865448	OOEP	assignmentsChromaffin_cells.Chrom_SOX2_statusHigh
assignmentsChromaffin_cells.Chrom_SOX2_statusHigh.MIR9-3HG	1.80549016	0.92389043	2.85727887	0.00554861	0.0162655	-2.6866649	MIR9-3HG	assignmentsChromaffin_cells.Chrom_SOX2_statusHigh
assignmentsChromaffin_cells.Chrom_SOX2_statusHigh.KIF26B-AS1	-2.3464451	0.38211019	-2.7293421	0.00792699	0.02199535	-2.6875275	KIF26B-AS1	assignmentsChromaffin_cells.Chrom_SOX2_statusHigh
assignmentsChromaffin_cells.Chrom_SOX2_statusHigh.FAM83D	1.44323366	1.18384205	2.78908117	0.0067165	0.01908899	-2.6876459	FAM83D	assignmentsChromaffin_cells.Chrom_SOX2_statusHigh
assignmentsChromaffin_cells.Chrom_SOX2_statusHigh.TPRG1	1.42364668	4.1890314	2.85303829	0.0056129	0.01642696	-2.6876573	TPRG1	assignmentsChromaffin_cells.Chrom_SOX2_statusHigh
assignmentsChromaffin_cells.Chrom_SOX2_statusHigh.NLGN2	-0.6944189	3.39635296	-2.889121	0.00506494	0.01508477	-2.688869	NLGN2	assignmentsChromaffin_cells.Chrom_SOX2_statusHigh
assignmentsChromaffin_cells.Chrom_SOX2_statusHigh.SF3A1	0.60471889	4.37055437	2.91223802	0.00474087	0.01427871	-2.6891508	SF3A1	assignmentsChromaffin_cells.Chrom_SOX2_statusHigh
assignmentsChromaffin_cells.Chrom_SOX2_statusHigh.ZNF445	-0.7284463	5.22184728	-2.9291829	0.00451561	0.01369801	-2.6891995	ZNF445	assignmentsChromaffin_cells.Chrom_SOX2_statusHigh
assignmentsChromaffin_cells.Chrom_SOX2_statusHigh.ZNF583	0.64995926	3.74528695	2.91142309	0.00475926	0.01430943	-2.6894214	ZNF583	assignmentsChromaffin_cells.Chrom_SOX2_statusHigh
assignmentsChromaffin_cells.Chrom_SOX2_statusHigh.OR2A7	2.03901344	0.80177412	2.80339334	0.00645719	0.01848964	-2.6902978	OR2A7	assignmentsChromaffin_cells.Chrom_SOX2_statusHigh
assignmentsChromaffin_cells.Chrom_SOX2_statusHigh.SEMA3E	-2.3426001	0.72500109	-2.7178462	0.00818125	0.02258362	-2.6911285	SEMA3E	assignmentsChromaffin_cells.Chrom_SOX2_statusHigh
assignmentsChromaffin_cells.Chrom_SOX2_statusHigh.GPC1	-1.0610046	3.28946077	-2.8815371	0.00517563	0.01536308	-2.692003	GPC1	assignmentsChromaffin_cells.Chrom_SOX2_statusHigh
assignmentsChromaffin_cells.Chrom_SOX2_statusHigh.DPYS	1.70908675	1.14031595	2.80614483	0.00640527	0.01837117	-2.6920096	DPYS	assignmentsChromaffin_cells.Chrom_SOX2_statusHigh
assignmentsChromaffin_cells.Chrom_SOX2_statusHigh.KCNC3	-0.9521585	3.04197752	-2.8791778	0.00521051	0.01544091	-2.6920431	KCNC3	assignmentsChromaffin_cells.Chrom_SOX2_statusHigh
assignmentsChromaffin_cells.Chrom_SOX2_statusHigh.HEBP2	1.47162182	4.83289835	2.94177279	0.00435797	0.01331045	-2.6924517	HEBP2	assignmentsChromaffin_cells.Chrom_SOX2_statusHigh
assignmentsChromaffin_cells.Chrom_SOX2_statusHigh.ACY1	0.90674367	2.33829993	2.86371395	0.00544459	0.01601118	-2.6931349	ACY1	assignmentsChromaffin_cells.Chrom_SOX2_statusHigh
assignmentsChromaffin_cells.Chrom_SOX2_statusHigh.PIGL	-0.8268565	5.82532312	-2.9301351	0.00450325	0.01367345	-2.6932416	PIGL	assignmentsChromaffin_cells.Chrom_SOX2_statusHigh
assignmentsChromaffin_cells.Chrom_SOX2_statusHigh.JUP	1.33387914	3.07502951	2.85134447	0.00563895	0.01649115	-2.6951672	JUP	assignmentsChromaffin_cells.Chrom_SOX2_statusHigh
assignmentsChromaffin_cells.Chrom_SOX2_statusHigh.ANKRD18B	-2.8264366	-0.1430945	-2.6901282	0.00882652	0.02405433	-2.695649	ANKRD18B	assignmentsChromaffin_cells.Chrom_SOX2_statusHigh
assignmentsChromaffin_cells.Chrom_SOX2_statusHigh.TSC22D3	1.76347106	6.35048078	2.92853479	0.0045274	0.01373116	-2.6997631	TSC22D3	assignmentsChromaffin_cells.Chrom_SOX2_statusHigh
assignmentsChromaffin_cells.Chrom_SOX2_statusHigh.MACROD1	-0.7481383	4.10492786	-2.900108	0.00490845	0.01470061	-2.7020607	MACROD1	assignmentsChromaffin_cells.Chrom_SOX2_statusHigh
assignmentsChromaffin_cells.Chrom_SOX2_statusHigh.LRIG2-DT	-1.0075319	2.82974094	-2.861131	0.00548462	0.01610169	-2.7022403	LRIG2-DT	assignmentsChromaffin_cells.Chrom_SOX2_statusHigh
assignmentsChromaffin_cells.Chrom_SOX2_statusHigh.A4GALT	1.93523249	0.82568065	2.6946292	0.00871554	0.02381384	-2.7049813	A4GALT	assignmentsChromaffin_cells.Chrom_SOX2_statusHigh
assignmentsChromaffin_cells.Chrom_SOX2_statusHigh.DUS1L	0.52850184	4.10377297	2.90072383	0.00489981	0.01468296	-2.7052203	DUS1L	assignmentsChromaffin_cells.Chrom_SOX2_statusHigh
assignmentsChromaffin_cells.Chrom_SOX2_statusHigh.ETV6	-0.9510947	8.09006487	-2.9335705	0.00445892	0.01356195	-2.7053039	ETV6	assignmentsChromaffin_cells.Chrom_SOX2_statusHigh
assignmentsChromaffin_cells.Chrom_SOX2_statusHigh.CHTF18	-1.3427257	2.01323311	-2.8219689	0.0061257	0.01770161	-2.7080949	CHTF18	assignmentsChromaffin_cells.Chrom_SOX2_statusHigh
assignmentsChromaffin_cells.Chrom_SOX2_statusHigh.WAKMAR2	1.6936026	3.26355883	2.80845939	0.00636407	0.01830141	-2.7093916	WAKMAR2	assignmentsChromaffin_cells.Chrom_SOX2_statusHigh
assignmentsChromaffin_cells.Chrom_SOX2_statusHigh.HGD	1.32330884	0.0947072	2.72838913	0.00794585	0.02204004	-2.7120338	HGD	assignmentsChromaffin_cells.Chrom_SOX2_statusHigh
assignmentsChromaffin_cells.Chrom_SOX2_statusHigh.STX16	1.42904663	5.99813773	2.90847488	0.0047951	0.01442849	-2.7170671	STX16	assignmentsChromaffin_cells.Chrom_SOX2_statusHigh
assignmentsChromaffin_cells.Chrom_SOX2_statusHigh.DUSP18	0.75012623	3.70168863	2.87252812	0.00531001	0.01568522	-2.7196802	DUSP18	assignmentsChromaffin_cells.Chrom_SOX2_statusHigh
assignmentsChromaffin_cells.Chrom_SOX2_statusHigh.GRB10	-1.510783	6.91408144	-2.9297724	0.0045113	0.01369012	-2.7209768	GRB10	assignmentsChromaffin_cells.Chrom_SOX2_statusHigh
assignmentsChromaffin_cells.Chrom_SOX2_statusHigh.GTPBP2	-0.699853	4.72623759	-2.9036065	0.00485957	0.01458689	-2.7213158	GTPBP2	assignmentsChromaffin_cells.Chrom_SOX2_statusHigh
assignmentsChromaffin_cells.Chrom_SOX2_statusHigh.PKD1	-0.8299188	5.62245507	-2.9367632	0.00441808	0.01345049	-2.7217642	PKD1	assignmentsChromaffin_cells.Chrom_SOX2_statusHigh
assignmentsChromaffin_cells.Chrom_SOX2_statusHigh.AKTIP	0.60420443	3.78261489	2.89385845	0.00499691	0.0149235	-2.7261249	AKTIP	assignmentsChromaffin_cells.Chrom_SOX2_statusHigh
assignmentsChromaffin_cells.Chrom_SOX2_statusHigh.TENT5B	2.06493801	-0.6449346	2.64337025	0.01001473	0.02665388	-2.7267927	TENT5B	assignmentsChromaffin_cells.Chrom_SOX2_statusHigh
assignmentsChromaffin_cells.Chrom_SOX2_statusHigh.FCHSD2	-0.9129699	8.03316898	-2.9410297	0.00436405	0.01332646	-2.7300671	FCHSD2	assignmentsChromaffin_cells.Chrom_SOX2_statusHigh
assignmentsChromaffin_cells.Chrom_SOX2_statusHigh.ULK2	-0.8703269	5.3462187	-2.9041024	0.00485267	0.01457165	-2.7308736	ULK2	assignmentsChromaffin_cells.Chrom_SOX2_statusHigh

assignmentsChromaffin_cells.Chrom_SOX2_statusHigh.SDE2	0.76437611	4.14217857	2.88967555	0.00505693	0.01506932	-2.7310073	SDE2	assignmentsChromaffin_cells.Chrom_SOX2_statusHigh
assignmentsChromaffin_cells.Chrom_SOX2_statusHigh.IER3	2.39144883	3.72483778	2.74062637	0.00768711	0.02141494	-2.7343501	IER3	assignmentsChromaffin_cells.Chrom_SOX2_statusHigh
assignmentsChromaffin_cells.Chrom_SOX2_statusHigh.FOXP1	-0.8098611	8.82921143	-2.9544935	0.00419751	0.01288662	-2.7375572	FOXP1	assignmentsChromaffin_cells.Chrom_SOX2_statusHigh
assignmentsChromaffin_cells.Chrom_SOX2_statusHigh.NME9	-1.2775199	2.84441824	-2.8450309	0.00574032	0.01671748	-2.7377025	NME9	assignmentsChromaffin_cells.Chrom_SOX2_statusHigh
assignmentsChromaffin_cells.Chrom_SOX2_statusHigh.LRCH3	-0.6134655	7.09922876	-2.9401356	0.00437532	0.01335072	-2.7382798	LRCH3	assignmentsChromaffin_cells.Chrom_SOX2_statusHigh
assignmentsChromaffin_cells.Chrom_SOX2_statusHigh.ERCC8	-0.6217348	5.50849454	-2.9157199	0.00469376	0.01417407	-2.739734	ERCC8	assignmentsChromaffin_cells.Chrom_SOX2_statusHigh
assignmentsChromaffin_cells.Chrom_SOX2_statusHigh.RAB40B	0.64857823	3.5255289	2.89355814	0.0050012	0.01492541	-2.7399554	RAB40B	assignmentsChromaffin_cells.Chrom_SOX2_statusHigh
assignmentsChromaffin_cells.Chrom_SOX2_statusHigh.PCDHGA2	1.59943967	0.70938932	2.79814401	0.0065499	0.01871834	-2.7407722	PCDHGA2	assignmentsChromaffin_cells.Chrom_SOX2_statusHigh
assignmentsChromaffin_cells.Chrom_SOX2_statusHigh.ETFRF1	0.72516517	4.51362144	2.91413164	0.0047152	0.01421205	-2.7412725	ETFRF1	assignmentsChromaffin_cells.Chrom_SOX2_statusHigh
assignmentsChromaffin_cells.Chrom_SOX2_statusHigh.MAP2K3	1.27968838	5.43843803	2.91432948	0.004714	0.0142111	-2.7420287	MAP2K3	assignmentsChromaffin_cells.Chrom_SOX2_statusHigh
assignmentsChromaffin_cells.Chrom_SOX2_statusHigh.PDE4A	1.55322357	3.89456237	2.84834053	0.005689	0.01661026	-2.743016	PDE4A	assignmentsChromaffin_cells.Chrom_SOX2_statusHigh
assignmentsChromaffin_cells.Chrom_SOX2_statusHigh.DHDH	1.35708027	0.86180237	2.79701476	0.00656938	0.01875395	-2.7433232	DHDH	assignmentsChromaffin_cells.Chrom_SOX2_statusHigh
assignmentsChromaffin_cells.Chrom_SOX2_statusHigh.NEDD9	-1.6009287	6.74205136	-2.8595021	0.00551312	0.0161733	-2.7459531	NEDD9	assignmentsChromaffin_cells.Chrom_SOX2_statusHigh
assignmentsChromaffin_cells.Chrom_SOX2_statusHigh.ENPP4	0.98703448	4.01326943	2.90108713	0.00489472	0.01467045	-2.7461575	ENPP4	assignmentsChromaffin_cells.Chrom_SOX2_statusHigh
assignmentsChromaffin_cells.Chrom_SOX2_statusHigh.SNX15	0.79473249	2.58438096	2.84706485	0.00570741	0.01664887	-2.7470245	SNX15	assignmentsChromaffin_cells.Chrom_SOX2_statusHigh
assignmentsChromaffin_cells.Chrom_SOX2_statusHigh.RBM14	-0.559299	5.32225691	-2.9044429	0.00484795	0.01456291	-2.7507775	RBM14	assignmentsChromaffin_cells.Chrom_SOX2_statusHigh
assignmentsChromaffin_cells.Chrom_SOX2_statusHigh.HSF1	0.57698077	5.24463874	2.90774327	0.00480234	0.01444757	-2.7514986	HSF1	assignmentsChromaffin_cells.Chrom_SOX2_statusHigh
assignmentsChromaffin_cells.Chrom_SOX2_statusHigh.ONECUT2	-0.8845401	2.68184303	-2.8773041	0.00523837	0.01550914	-2.7516671	ONECUT2	assignmentsChromaffin_cells.Chrom_SOX2_statusHigh
assignmentsChromaffin_cells.Chrom_SOX2_statusHigh.SGSM2	-0.8315906	4.96231946	-2.8937842	0.00499797	0.0149235	-2.751986	SGSM2	assignmentsChromaffin_cells.Chrom_SOX2_statusHigh
assignmentsChromaffin_cells.Chrom_SOX2_statusHigh.DOK4	-1.0205846	2.65904924	-2.8483943	0.00568601	0.01660691	-2.7522116	DOK4	assignmentsChromaffin_cells.Chrom_SOX2_statusHigh
assignmentsChromaffin_cells.Chrom_SOX2_statusHigh.ZEB1-AS1	0.8643887	3.49970668	2.8508688	0.00564636	0.01650679	-2.7525205	ZEB1-AS1	assignmentsChromaffin_cells.Chrom_SOX2_statusHigh
assignmentsChromaffin_cells.Chrom_SOX2_statusHigh.WNT4	1.59056817	0.07455482	2.70083786	0.00856907	0.0234804	-2.7557373	WNT4	assignmentsChromaffin_cells.Chrom_SOX2_statusHigh
assignmentsChromaffin_cells.Chrom_SOX2_statusHigh.CLPTM1L	0.77619645	4.49150016	2.89217893	0.00502093	0.01497873	-2.7571741	CLPTM1L	assignmentsChromaffin_cells.Chrom_SOX2_statusHigh
assignmentsChromaffin_cells.Chrom_SOX2_statusHigh.ZC3H18	-0.5617317	5.61996017	-2.9005774	0.00490187	0.01468637	-2.7581043	ZC3H18	assignmentsChromaffin_cells.Chrom_SOX2_statusHigh
assignmentsChromaffin_cells.Chrom_SOX2_statusHigh.RPA4	-1.1412697	0.98408695	-2.7410456	0.00767367	0.02138124	-2.7593191	RPA4	assignmentsChromaffin_cells.Chrom_SOX2_statusHigh
assignmentsChromaffin_cells.Chrom_SOX2_statusHigh.TOP3B	-0.8228482	3.29829418	-2.8499727	0.00566069	0.01653964	-2.7600603	TOP3B	assignmentsChromaffin_cells.Chrom_SOX2_statusHigh
assignmentsChromaffin_cells.Chrom_SOX2_statusHigh.PAFAH1B1	-0.5310466	8.16936479	-2.9529796	0.00421594	0.01293329	-2.7616451	PAFAH1B1	assignmentsChromaffin_cells.Chrom_SOX2_statusHigh
assignmentsChromaffin_cells.Chrom_SOX2_statusHigh.HHLA3	0.727213	2.1515952	2.82497296	0.00607418	0.01757809	-2.7629973	HHLA3	assignmentsChromaffin_cells.Chrom_SOX2_statusHigh
assignmentsChromaffin_cells.Chrom_SOX2_statusHigh.TTC8	-1.142297	6.21299277	-2.9390717	0.00439015	0.0133756	-2.7644192	TTC8	assignmentsChromaffin_cells.Chrom_SOX2_statusHigh
assignmentsChromaffin_cells.Chrom_SOX2_statusHigh.SATB2	-1.8347996	4.39721093	-2.8712069	0.00533368	0.01574192	-2.7650064	SATB2	assignmentsChromaffin_cells.Chrom_SOX2_statusHigh
assignmentsChromaffin_cells.Chrom_SOX2_statusHigh.TFR2	-1.2456896	0.86553747	-2.7397252	0.00770167	0.02144805	-2.7653004	TFR2	assignmentsChromaffin_cells.Chrom_SOX2_statusHigh
assignmentsChromaffin_cells.Chrom_SOX2_statusHigh.BEAN1	-2.2235657	1.13247454	-2.6790051	0.00909571	0.02463722	-2.7665167	BEAN1	assignmentsChromaffin_cells.Chrom_SOX2_statusHigh
assignmentsChromaffin_cells.Chrom_SOX2_statusHigh.DPP8	-0.4793242	6.14892292	-2.9170886	0.00467535	0.01412381	-2.7671189	DPP8	assignmentsChromaffin_cells.Chrom_SOX2_statusHigh
assignmentsChromaffin_cells.Chrom_SOX2_statusHigh.STXBP6	-2.4759719	3.35573032	-2.8151582	0.00624803	0.01799674	-2.7674148	STXBP6	assignmentsChromaffin_cells.Chrom_SOX2_statusHigh
assignmentsChromaffin_cells.Chrom_SOX2_statusHigh.CLCA4-AS1	1.17966434	1.81814362	2.78861923	0.00672516	0.01910006	-2.7677285	CLCA4-AS1	assignmentsChromaffin_cells.Chrom_SOX2_statusHigh
assignmentsChromaffin_cells.Chrom_SOX2_statusHigh.ZNF324B	0.73341748	2.12192405	2.8255455	0.00606441	0.01755614	-2.7678812	ZNF324B	assignmentsChromaffin_cells.Chrom_SOX2_statusHigh
assignmentsChromaffin_cells.Chrom_SOX2_statusHigh.ZBTB20-AS:	-2.4861108	1.51660851	-2.7244562	0.00803687	0.02225403	-2.7690525	ZBTB20-AS2	assignmentsChromaffin_cells.Chrom_SOX2_statusHigh
assignmentsChromaffin_cells.Chrom_SOX2_statusHigh.TSPYL4	0.94402836	4.79773227	2.91922137	0.00464681	0.01404817	-2.7691449	TSPYL4	assignmentsChromaffin_cells.Chrom_SOX2_statusHigh
assignmentsChromaffin_cells.Chrom_SOX2_statusHigh.RBM48	0.61536178	4.02298065	2.88006141	0.00519742	0.01540496	-2.7701788	RBM48	assignmentsChromaffin_cells.Chrom_SOX2_statusHigh
assignmentsChromaffin_cells.Chrom_SOX2_statusHigh.DSTN	1.21893957	6.91354082	2.94196846	0.00435469	0.0133055	-2.7701866	DSTN	assignmentsChromaffin_cells.Chrom_SOX2_statusHigh
assignmentsChromaffin_cells.Chrom_SOX2_statusHigh.GPATCH11	0.71259083	3.98387159	2.87380499	0.00529077	0.01564406	-2.7720365	GPATCH11	assignmentsChromaffin_cells.Chrom_SOX2_statusHigh
assignmentsChromaffin_cells.Chrom_SOX2_statusHigh.DOCK3	-1.2519575	7.35146207	-2.9570724	0.00416948	0.01280775	-2.7726447	DOCK3	assignmentsChromaffin_cells.Chrom_SOX2_statusHigh
assignmentsChromaffin_cells.Chrom_SOX2_statusHigh.USP30	-0.5624981	4.20927276	-2.8812226	0.00518027	0.015374	-2.7750719	USP30	assignmentsChromaffin_cells.Chrom_SOX2_statusHigh
assignmentsChromaffin_cells.Chrom_SOX2_statusHigh.ZFYVE28	-0.9249939	5.94174045	-2.905982	0.00482663	0.01450975	-2.775516	ZFYVE28	assignmentsChromaffin_cells.Chrom_SOX2_statusHigh
assignmentsChromaffin_cells.Chrom_SOX2_statusHigh.SYNPO	2.01909239	3.78558252	2.87032195	0.00534711	0.01577223	-2.7762514	SYNPO	assignmentsChromaffin_cells.Chrom_SOX2_statusHigh
assignmentsChromaffin_cells.Chrom_SOX2_statusHigh.C15orf40	0.60448742	4.19831354	2.88708516	0.00509444	0.01516417	-2.7768916	C15orf40	assignmentsChromaffin_cells.Chrom_SOX2_statusHigh
assignmentsChromaffin_cells.Chrom_SOX2_statusHigh.RFX5	0.69673562	3.10550651	2.84633115	0.00571926	0.01667433	-2.7784378	RFX5	assignmentsChromaffin_cells.Chrom_SOX2_statusHigh
assignmentsChromaffin_cells.Chrom_SOX2_statusHigh.CDC26	1.11648044	3.75749206	2.87843679	0.00522154	0.01546408	-2.7788481	CDC26	assignmentsChromaffin_cells.Chrom_SOX2_statusHigh
assignmentsChromaffin_cells.Chrom_SOX2_statusHigh.NSMCE1	0.64459485	4.72036434	2.88380316	0.00514232	0.01527553	-2.7793085	NSMCE1	assignmentsChromaffin_cells.Chrom_SOX2_statusHigh
assignmentsChromaffin_cells.Chrom_SOX2_statusHigh.PHACTR4	-0.71037	6.69474203	-2.9054237	0.00483435	0.01453024	-2.7795545	PHACTR4	assignmentsChromaffin_cells.Chrom_SOX2_statusHigh
assignmentsChromaffin_cells.Chrom_SOX2_statusHigh.RNF8	-0.4749623	5.06346785	-2.893479	0.00500233	0.01492601	-2.7800525	RNF8	assignmentsChromaffin_cells.Chrom_SOX2_statusHigh
assignmentsChromaffin_cells.Chrom_SOX2_statusHigh.RIC8B	-0.5016017	5.92246613	-2.9072696	0.00480886	0.01446176	-2.7821459	RIC8B	assignmentsChromaffin_cells.Chrom_SOX2_statusHigh
assignmentsChromaffin_cells.Chrom_SOX2_statusHigh.UBR5	-0.6569708	8.42598774	-2.949431	0.00425943	0.01305172	-2.7840416	UBR5	assignmentsChromaffin_cells.Chrom_SOX2_statusHigh
assignmentsChromaffin_cells.Chrom_SOX2_statusHigh.PCNX2	-1.0530287	7.35971606	-2.9443146	0.00432408	0.01322204	-2.7842404	PCNX2	assignmentsChromaffin_cells.Chrom_SOX2_statusHigh
assignmentsChromaffin_cells.Chrom_SOX2_statusHigh.ARAF	0.68661675	4.05775924	2.87249181	0.00531056	0.01568522	-2.7844024	ARAF	assignmentsChromaffin_cells.Chrom_SOX2_statusHigh
assignmentsChromaffin_cells.Chrom_SOX2_statusHigh.LINC00571	-1.26751	5.12621568	-2.8868316	0.00509963	0.015174	-2.784708	LINC00571	assignmentsChromaffin_cells.Chrom_SOX2_statusHigh
assignmentsChromaffin_cells.Chrom_SOX2_statusHigh.LAMTOR5-A	-1.0930965	4.29623739	-2.8438456	0.00575956	0.01676135	-2.7851868	LAMTOR5-AS	assignmentsChromaffin_cells.Chrom_SOX2_statusHigh
assignmentsChromaffin_cells.Chrom_SOX2_statusHigh.SPATA21	-1.8685754	0.66798647	-2.6710744	0.00929218	0.02507055	-2.7860461	SPATA21	assignmentsChromaffin_cells.Chrom_SOX2_statusHigh
assignmentsChromaffin_cells.Chrom_SOX2_statusHigh.MAP2K6	-1.7428343	4.08450623	-2.8310705	0.00597479	0.01733111	-2.7874072	MAP2K6	assignmentsChromaffin_cells.Chrom_SOX2_statusHigh
assignmentsChromaffin_cells.Chrom_SOX2_statusHigh.SPTB	-1.0338169	3.64073765	-2.8729621	0.00530346	0.01567004	-2.7882699	SPTB	assignmentsChromaffin_cells.Chrom_SOX2_statusHigh
assignmentsChromaffin_cells.Chrom_SOX2_statusHigh.MT-CYB	2.95887322	8.88061474	2.97509486	0.00395713	0.01224474	-2.7894837	MT-CYB	assignmentsChromaffin_cells.Chrom_SOX2_statusHigh
assignmentsChromaffin_cells.Chrom_SOX2_statusHigh.RANBP17	-1.0216458	7.25814728	-2.9390591	0.00438988	0.0133756	-2.7913787	RANBP17	assignmentsChromaffin_cells.Chrom_SOX2_statusHigh
assignmentsChromaffin_cells.Chrom_SOX2_statusHigh.SVIP	0.79434963	4.61930605	2.88925702	0.00506297	0.01508452	-2.7935162	SVIP	assignmentsChromaffin_cells.Chrom_SOX2_statusHigh
assignmentsChromaffin_cells.Chrom_SOX2_statusHigh.MPRIP	-0.8564623	6.84157716	-2.9042989	0.00484995	0.01456618	-2.7953674	MPRIP	assignmentsChromaffin_cells.Chrom_SOX2_statusHigh

assignmentsChromaffin_cells.Chrom_SOX2_statusHigh.MPRIP-AS1	-1.7231019	0.70057315	-2.6776083	0.00912884	0.02470193	-2.7962157	MPRIP-AS1	assignmentsChromaffin_cells.Chrom_SOX2_statusHigh
assignmentsChromaffin_cells.Chrom_SOX2_statusHigh.BRPF1	0.69260294	3.11172576	2.83454604	0.00591267	0.01716954	-2.7970905	BRPF1	assignmentsChromaffin_cells.Chrom_SOX2_statusHigh
assignmentsChromaffin_cells.Chrom_SOX2_statusHigh.CFAP97	0.57003686	5.45597739	2.89935569	0.00491902	0.01472677	-2.7986773	CFAP97	assignmentsChromaffin_cells.Chrom_SOX2_statusHigh
assignmentsChromaffin_cells.Chrom_SOX2_statusHigh.RHOD	1.88738566	-0.129539	2.64530463	0.00996267	0.02655057	-2.7995864	RHOD	assignmentsChromaffin_cells.Chrom_SOX2_statusHigh
assignmentsChromaffin_cells.Chrom_SOX2_statusHigh.CTU1	1.02294103	1.9917796	2.8067602	0.00639271	0.01834566	-2.7996079	CTU1	assignmentsChromaffin_cells.Chrom_SOX2_statusHigh
assignmentsChromaffin_cells.Chrom_SOX2_statusHigh.SREK1	-0.4513907	6.95171794	-2.9233801	0.00459162	0.0139049	-2.8001009	SREK1	assignmentsChromaffin_cells.Chrom_SOX2_statusHigh
assignmentsChromaffin_cells.Chrom_SOX2_statusHigh.HLA-DPA1	1.78991561	4.06339279	2.80297815	0.00646488	0.01850173	-2.8003143	HLA-DPA1	assignmentsChromaffin_cells.Chrom_SOX2_statusHigh
assignmentsChromaffin_cells.Chrom_SOX2_statusHigh.PNO1	0.77801857	4.19354401	2.87149828	0.00532558	0.01572378	-2.8012168	PNO1	assignmentsChromaffin_cells.Chrom_SOX2_statusHigh
assignmentsChromaffin_cells.Chrom_SOX2_statusHigh.S100PBP	-0.7374891	5.95611714	-2.8864886	0.00510311	0.01518154	-2.8016429	S100PBP	assignmentsChromaffin_cells.Chrom_SOX2_statusHigh
assignmentsChromaffin_cells.Chrom_SOX2_statusHigh.FKBP14	0.69360102	4.59934163	2.89681166	0.00495493	0.01481214	-2.8024847	FKBP14	assignmentsChromaffin_cells.Chrom_SOX2_statusHigh
assignmentsChromaffin_cells.Chrom_SOX2_statusHigh.GALK2	-0.6544485	6.44911916	-2.9106792	0.00476211	0.01433459	-2.803053	GALK2	assignmentsChromaffin_cells.Chrom_SOX2_statusHigh
assignmentsChromaffin_cells.Chrom_SOX2_statusHigh.HS3ST4	-4.2394116	1.22531051	-2.6521105	0.00978664	0.02617278	-2.8034966	HS3ST4	assignmentsChromaffin_cells.Chrom_SOX2_statusHigh
assignmentsChromaffin_cells.Chrom_SOX2_statusHigh.ZFYVE1	-0.5973608	4.80975487	-2.8674063	0.00538784	0.0158638	-2.804256	ZFYVE1	assignmentsChromaffin_cells.Chrom_SOX2_statusHigh
assignmentsChromaffin_cells.Chrom_SOX2_statusHigh.NOL3	0.80939307	4.59432636	2.9001609	0.00490771	0.01470061	-2.804354	NOL3	assignmentsChromaffin_cells.Chrom_SOX2_statusHigh
assignmentsChromaffin_cells.Chrom_SOX2_statusHigh.MAP3K1	-0.9332107	6.09162361	-2.8624889	0.00546355	0.01606018	-2.8044885	MAP3K1	assignmentsChromaffin_cells.Chrom_SOX2_statusHigh
assignmentsChromaffin_cells.Chrom_SOX2_statusHigh.NGDN	0.60804956	4.67431869	2.88182666	0.00517136	0.01535325	-2.8046729	NGDN	assignmentsChromaffin_cells.Chrom_SOX2_statusHigh
assignmentsChromaffin_cells.Chrom_SOX2_statusHigh.NDUFAF1	0.58255097	3.31785129	2.84834401	0.00568681	0.01660691	-2.8074654	NDUFAF1	assignmentsChromaffin_cells.Chrom_SOX2_statusHigh
assignmentsChromaffin_cells.Chrom_SOX2_statusHigh.GSDMB	-0.7545015	3.17940212	-2.8181004	0.00619262	0.01786283	-2.8076685	GSDMB	assignmentsChromaffin_cells.Chrom_SOX2_statusHigh
assignmentsChromaffin_cells.Chrom_SOX2_statusHigh.SNX24	-0.796785	5.66453078	-2.8840276	0.00513904	0.01527117	-2.8079396	SNX24	assignmentsChromaffin_cells.Chrom_SOX2_statusHigh
assignmentsChromaffin_cells.Chrom_SOX2_statusHigh.NTRK2	-3.445594	2.79590094	-2.6850431	0.00895111	0.02430713	-2.8085381	NTRK2	assignmentsChromaffin_cells.Chrom_SOX2_statusHigh
assignmentsChromaffin_cells.Chrom_SOX2_statusHigh.RBBP9	0.78045242	2.26931024	2.8095724	0.00634254	0.01824605	-2.8085411	RBBP9	assignmentsChromaffin_cells.Chrom_SOX2_statusHigh
assignmentsChromaffin_cells.Chrom_SOX2_statusHigh.FLAD1	0.69802789	3.74358824	2.8623601	0.00546554	0.0160631	-2.8089494	FLAD1	assignmentsChromaffin_cells.Chrom_SOX2_statusHigh
assignmentsChromaffin_cells.Chrom_SOX2_statusHigh.CHST9	-2.469786	1.08428506	-2.6431683	0.01002215	0.02666342	-2.80952	CHST9	assignmentsChromaffin_cells.Chrom_SOX2_statusHigh
assignmentsChromaffin_cells.Chrom_SOX2_statusHigh.MCUR1	0.92155829	4.00718484	2.86112643	0.00548469	0.01610169	-2.8102078	MCUR1	assignmentsChromaffin_cells.Chrom_SOX2_statusHigh
assignmentsChromaffin_cells.Chrom_SOX2_statusHigh.GALM	1.64627302	3.90774151	2.79121314	0.00667848	0.01900788	-2.810288	GALM	assignmentsChromaffin_cells.Chrom_SOX2_statusHigh
assignmentsChromaffin_cells.Chrom_SOX2_statusHigh.ADAM29	-2.7580728	0.10234753	-2.6613294	0.00954588	0.02564006	-2.8119438	ADAM29	assignmentsChromaffin_cells.Chrom_SOX2_statusHigh
assignmentsChromaffin_cells.Chrom_SOX2_statusHigh.HCFC2	-0.5420843	5.46641306	-2.8936681	0.00499962	0.0149235	-2.8123756	HCFC2	assignmentsChromaffin_cells.Chrom_SOX2_statusHigh
assignmentsChromaffin_cells.Chrom_SOX2_statusHigh.NFKBIE	1.4231569	2.14721506	2.7566838	0.00734916	0.02062395	-2.8127706	NFKBIE	assignmentsChromaffin_cells.Chrom_SOX2_statusHigh
assignmentsChromaffin_cells.Chrom_SOX2_statusHigh.TRPV2	2.10934678	3.46328485	2.79420077	0.00662541	0.0188803	-2.8138757	TRPV2	assignmentsChromaffin_cells.Chrom_SOX2_statusHigh
assignmentsChromaffin_cells.Chrom_SOX2_statusHigh.PWP2	1.84893419	1.80346808	2.77879701	0.00691555	0.01957495	-2.8141618	PWP2	assignmentsChromaffin_cells.Chrom_SOX2_statusHigh
assignmentsChromaffin_cells.Chrom_SOX2_statusHigh.NIFK-AS1	-0.6142426	4.42013135	-2.862105	0.0054695	0.01606884	-2.8144079	NIFK-AS1	assignmentsChromaffin_cells.Chrom_SOX2_statusHigh
assignmentsChromaffin_cells.Chrom_SOX2_statusHigh.DCP1B	-0.8500666	5.20575741	-2.8839682	0.0051399	0.01527117	-2.814936	DCP1B	assignmentsChromaffin_cells.Chrom_SOX2_statusHigh
assignmentsChromaffin_cells.Chrom_SOX2_statusHigh.NUP98	-0.6323383	7.02444927	-2.9037785	0.00485717	0.01458243	-2.8153813	NUP98	assignmentsChromaffin_cells.Chrom_SOX2_statusHigh
assignmentsChromaffin_cells.Chrom_SOX2_statusHigh.CSF1	1.67660825	2.49257785	2.73063458	0.00789807	0.02192646	-2.8161937	CSF1	assignmentsChromaffin_cells.Chrom_SOX2_statusHigh
assignmentsChromaffin_cells.Chrom_SOX2_statusHigh.RAI1	-0.7669948	5.67894463	-2.8783915	0.00522219	0.01546408	-2.8162596	RAI1	assignmentsChromaffin_cells.Chrom_SOX2_statusHigh
assignmentsChromaffin_cells.Chrom_SOX2_statusHigh.SAMD11	2.17522523	3.10761498	2.87377715	0.00529487	0.01565329	-2.8164779	SAMD11	assignmentsChromaffin_cells.Chrom_SOX2_statusHigh
assignmentsChromaffin_cells.Chrom_SOX2_statusHigh.FKBP15	-0.6327354	5.67843024	-2.8803068	0.00519379	0.0153999	-2.8173927	FKBP15	assignmentsChromaffin_cells.Chrom_SOX2_statusHigh
assignmentsChromaffin_cells.Chrom_SOX2_statusHigh.TWSG1	-0.9009516	5.18774309	-2.8649815	0.00542505	0.01595872	-2.8180959	TWSG1	assignmentsChromaffin_cells.Chrom_SOX2_statusHigh
assignmentsChromaffin_cells.Chrom_SOX2_statusHigh.POMT1	-0.8156409	4.10607653	-2.8476102	0.00569862	0.01662928	-2.8183485	POMT1	assignmentsChromaffin_cells.Chrom_SOX2_statusHigh
assignmentsChromaffin_cells.Chrom_SOX2_statusHigh.LRRC19	-1.5775483	0.56557399	-2.7063182	0.00844166	0.02319071	-2.8184603	LRRC19	assignmentsChromaffin_cells.Chrom_SOX2_statusHigh
assignmentsChromaffin_cells.Chrom_SOX2_statusHigh.CCDC163	1.43706876	0.58295095	2.72291682	0.00806624	0.02232763	-2.8188377	CCDC163	assignmentsChromaffin_cells.Chrom_SOX2_statusHigh
assignmentsChromaffin_cells.Chrom_SOX2_statusHigh.COA6-AS1	1.06613278	2.72581713	2.84513153	0.00573868	0.01671575	-2.8219275	COA6-AS1	assignmentsChromaffin_cells.Chrom_SOX2_statusHigh
assignmentsChromaffin_cells.Chrom_SOX2_statusHigh.TUB	-1.00652	3.31449643	-2.8441074	0.0057553	0.016752	-2.8221555	TUB	assignmentsChromaffin_cells.Chrom_SOX2_statusHigh
assignmentsChromaffin_cells.Chrom_SOX2_statusHigh.DGCR11	-0.9574577	1.52400515	-2.745719	0.00757535	0.02115137	-2.823297	DGCR11	assignmentsChromaffin_cells.Chrom_SOX2_statusHigh
assignmentsChromaffin_cells.Chrom_SOX2_statusHigh.SILC1	-2.1108339	-0.452873	-2.6061683	0.01106529	0.028955	-2.8236071	SILC1	assignmentsChromaffin_cells.Chrom_SOX2_statusHigh
assignmentsChromaffin_cells.Chrom_SOX2_statusHigh.FAM120C	-0.6105322	4.70266029	-2.8663135	0.00540458	0.01590433	-2.8238555	FAM120C	assignmentsChromaffin_cells.Chrom_SOX2_statusHigh
assignmentsChromaffin_cells.Chrom_SOX2_statusHigh.SH3BGR	1.06063452	2.78508302	2.85040743	0.00565373	0.01652534	-2.8252102	SH3BGR	assignmentsChromaffin_cells.Chrom_SOX2_statusHigh
assignmentsChromaffin_cells.Chrom_SOX2_statusHigh.GRK5	-1.0659628	7.67822791	-2.9143908	0.00471275	0.01421003	-2.8265783	GRK5	assignmentsChromaffin_cells.Chrom_SOX2_statusHigh
assignmentsChromaffin_cells.Chrom_SOX2_statusHigh.USP7	-0.5492349	6.02804383	-2.8843017	0.00513502	0.01526232	-2.8269507	USP7	assignmentsChromaffin_cells.Chrom_SOX2_statusHigh
assignmentsChromaffin_cells.Chrom_SOX2_statusHigh.C7orf50	0.61131258	5.67621231	2.89904691	0.00492337	0.01473427	-2.8280458	C7orf50	assignmentsChromaffin_cells.Chrom_SOX2_statusHigh
assignmentsChromaffin_cells.Chrom_SOX2_statusHigh.ERBIN	-0.68756	7.80852509	-2.9049677	0.00484067	0.01454399	-2.829373	ERBIN	assignmentsChromaffin_cells.Chrom_SOX2_statusHigh
assignmentsChromaffin_cells.Chrom_SOX2_statusHigh.BAG4	0.46360341	4.63234657	2.86369539	0.00544488	0.01601118	-2.8294018	BAG4	assignmentsChromaffin_cells.Chrom_SOX2_statusHigh
assignmentsChromaffin_cells.Chrom_SOX2_statusHigh.COL10A1	-1.8703586	1.91973109	-2.7497839	0.00749386	0.02096398	-2.8295547	COL10A1	assignmentsChromaffin_cells.Chrom_SOX2_statusHigh
assignmentsChromaffin_cells.Chrom_SOX2_statusHigh.CEP78	-0.770507	4.63901195	-2.8540426	0.00559587	0.01638609	-2.8295718	CEP78	assignmentsChromaffin_cells.Chrom_SOX2_statusHigh
assignmentsChromaffin_cells.Chrom_SOX2_statusHigh.LYRM7	0.52404603	3.97717406	2.85433734	0.00559121	0.01637542	-2.8296159	LYRM7	assignmentsChromaffin_cells.Chrom_SOX2_statusHigh
assignmentsChromaffin_cells.Chrom_SOX2_statusHigh.LINC02231	-2.9427921	0.10663789	-2.6187728	0.01070273	0.02815327	-2.8315201	LINC02231	assignmentsChromaffin_cells.Chrom_SOX2_statusHigh
assignmentsChromaffin_cells.Chrom_SOX2_statusHigh.FASTKD5	0.77530503	2.27833511	2.80729288	0.00638318	0.01834168	-2.8322121	FASTKD5	assignmentsChromaffin_cells.Chrom_SOX2_statusHigh
assignmentsChromaffin_cells.Chrom_SOX2_statusHigh.RORA-AS1	-1.0590828	4.65452588	-2.8511974	0.00564111	0.01649446	-2.8325578	RORA-AS1	assignmentsChromaffin_cells.Chrom_SOX2_statusHigh
assignmentsChromaffin_cells.Chrom_SOX2_statusHigh.DANT2	-1.0980267	7.55297574	-2.9391239	0.00438976	0.0133756	-2.8329213	DANT2	assignmentsChromaffin_cells.Chrom_SOX2_statusHigh
assignmentsChromaffin_cells.Chrom_SOX2_statusHigh.LINC00526	1.19841506	1.02465334	2.79075301	0.00668525	0.01902288	-2.8345647	LINC00526	assignmentsChromaffin_cells.Chrom_SOX2_statusHigh
assignmentsChromaffin_cells.Chrom_SOX2_statusHigh.GRIP2	2.07569	1.31494838	2.72596953	0.00800265	0.02216694	-2.8358249	GRIP2	assignmentsChromaffin_cells.Chrom_SOX2_statusHigh
assignmentsChromaffin_cells.Chrom_SOX2_statusHigh.QSER1	-0.7126775	6.08654434	-2.8835672	0.00514578	0.01528297	-2.8364303	QSER1	assignmentsChromaffin_cells.Chrom_SOX2_statusHigh
assignmentsChromaffin_cells.Chrom_SOX2_statusHigh.ING1	1.21455098	3.18928991	2.80757752	0.0063781	0.01833219	-2.8371172	ING1	assignmentsChromaffin_cells.Chrom_SOX2_statusHigh
assignmentsChromaffin_cells.Chrom_SOX2_statusHigh.RNF139	0.91735894	4.00241673	2.85618135	0.00556209	0.01629907	-2.8374304	RNF139	assignmentsChromaffin_cells.Chrom_SOX2_statusHigh

assignmentsChromaffin_cells.Chrom_SOX2_statusHigh.GTSE1	1.12972862	1.46304672	2.77923843	0.00690322	0.01955729	-2.8383079	GTSE1	assignmentsChromaffin_cells.Chrom_SOX2_statusHigh
assignmentsChromaffin_cells.Chrom_SOX2_statusHigh.LINC00630	-0.8817221	5.46770587	-2.8827484	0.0051578	0.01531583	-2.8390777	LINC00630	assignmentsChromaffin_cells.Chrom_SOX2_statusHigh
assignmentsChromaffin_cells.Chrom_SOX2_statusHigh.NRXN2	-1.7250838	3.0154751	-2.8023865	0.00647559	0.01852246	-2.8400489	NRXN2	assignmentsChromaffin_cells.Chrom_SOX2_statusHigh
assignmentsChromaffin_cells.Chrom_SOX2_statusHigh.CADM3	2.62601133	1.54231946	2.80308026	0.00646303	0.01849975	-2.8410387	CADM3	assignmentsChromaffin_cells.Chrom_SOX2_statusHigh
assignmentsChromaffin_cells.Chrom_SOX2_statusHigh.ARL11	1.80332796	0.04964059	2.60190684	0.0111918	0.02923358	-2.8417001	ARL11	assignmentsChromaffin_cells.Chrom_SOX2_statusHigh
assignmentsChromaffin_cells.Chrom_SOX2_statusHigh.PIP4P2	0.68339979	5.41016909	2.89559811	0.00497214	0.01485805	-2.8435508	PIP4P2	assignmentsChromaffin_cells.Chrom_SOX2_statusHigh
assignmentsChromaffin_cells.Chrom_SOX2_statusHigh.SLC2A4RG	0.74410479	3.74149236	2.84475203	0.00574483	0.0167276	-2.8442196	SLC2A4RG	assignmentsChromaffin_cells.Chrom_SOX2_statusHigh
assignmentsChromaffin_cells.Chrom_SOX2_statusHigh.MALRD1	-1.8688105	2.25694769	-2.7208831	0.00811337	0.02243286	-2.8443075	MALRD1	assignmentsChromaffin_cells.Chrom_SOX2_statusHigh
assignmentsChromaffin_cells.Chrom_SOX2_statusHigh.MT-ND5	2.46183902	7.86898693	2.93106733	0.00449452	0.01365728	-2.8446809	MT-ND5	assignmentsChromaffin_cells.Chrom_SOX2_statusHigh
assignmentsChromaffin_cells.Chrom_SOX2_statusHigh.PLBD1-AS1	1.79007475	0.38527821	2.60151764	0.01120342	0.02925916	-2.8447475	PLBD1-AS1	assignmentsChromaffin_cells.Chrom_SOX2_statusHigh
assignmentsChromaffin_cells.Chrom_SOX2_statusHigh.MED21	0.79721368	4.73346686	2.8712485	0.00532936	0.01573205	-2.8459569	MED21	assignmentsChromaffin_cells.Chrom_SOX2_statusHigh
assignmentsChromaffin_cells.Chrom_SOX2_statusHigh.SEC23A	-0.6428704	6.66941013	-2.8989041	0.00492538	0.01473754	-2.8461478	SEC23A	assignmentsChromaffin_cells.Chrom_SOX2_statusHigh
assignmentsChromaffin_cells.Chrom_SOX2_statusHigh.TAFA5	-3.9956273	3.31174515	-2.7431765	0.00763323	0.02129075	-2.8529815	TAFA5	assignmentsChromaffin_cells.Chrom_SOX2_statusHigh
assignmentsChromaffin_cells.Chrom_SOX2_statusHigh.STIMATE	-0.6612814	4.48954818	-2.8228826	0.00610999	0.01766257	-2.8535464	STIMATE	assignmentsChromaffin_cells.Chrom_SOX2_statusHigh
assignmentsChromaffin_cells.Chrom_SOX2_statusHigh.KIAA0319L	-0.6289707	6.60065187	-2.8870075	0.00509557	0.01516472	-2.8538527	KIAA0319L	assignmentsChromaffin_cells.Chrom_SOX2_statusHigh
assignmentsChromaffin_cells.Chrom_SOX2_statusHigh.HUWE1	-0.4130098	7.74921389	-2.9148304	0.00470575	0.01419693	-2.8555609	HUWE1	assignmentsChromaffin_cells.Chrom_SOX2_statusHigh
assignmentsChromaffin_cells.Chrom_SOX2_statusHigh.NBPF12	-0.9698008	5.0139216	-2.8515281	0.00563584	0.01648806	-2.8556282	NBPF12	assignmentsChromaffin_cells.Chrom_SOX2_statusHigh
assignmentsChromaffin_cells.Chrom_SOX2_statusHigh.ETS2	1.5641493	5.8685357	2.8774409	0.00523999	0.01551107	-2.8565029	ETS2	assignmentsChromaffin_cells.Chrom_SOX2_statusHigh
assignmentsChromaffin_cells.Chrom_SOX2_statusHigh.DNAJC14	0.62818623	2.89408702	2.80605799	0.00640529	0.01837117	-2.8572795	DNAJC14	assignmentsChromaffin_cells.Chrom_SOX2_statusHigh
assignmentsChromaffin_cells.Chrom_SOX2_statusHigh.MXD4	1.05400501	5.30406228	2.87707375	0.00524263	0.01551511	-2.8573311	MXD4	assignmentsChromaffin_cells.Chrom_SOX2_statusHigh
assignmentsChromaffin_cells.Chrom_SOX2_statusHigh.HYOU1	1.11858852	4.69747789	2.87062182	0.00533992	0.01575745	-2.8573641	HYOU1	assignmentsChromaffin_cells.Chrom_SOX2_statusHigh
assignmentsChromaffin_cells.Chrom_SOX2_statusHigh.CORO6	1.91640721	-0.0460806	2.61409229	0.01083349	0.02843158	-2.8578573	CORO6	assignmentsChromaffin_cells.Chrom_SOX2_statusHigh
assignmentsChromaffin_cells.Chrom_SOX2_statusHigh.CERNA1	1.06599062	1.16297092	2.77217888	0.00704006	0.01985378	-2.8593256	CERNA1	assignmentsChromaffin_cells.Chrom_SOX2_statusHigh
assignmentsChromaffin_cells.Chrom_SOX2_statusHigh.MYO18A	-0.7720231	4.8224047	-2.8372563	0.00586766	0.01704812	-2.8595517	MYO18A	assignmentsChromaffin_cells.Chrom_SOX2_statusHigh
assignmentsChromaffin_cells.Chrom_SOX2_statusHigh.CHD8	-0.6011236	6.20939718	-2.8806572	0.00518861	0.01539023	-2.8611145	CHD8	assignmentsChromaffin_cells.Chrom_SOX2_statusHigh
assignmentsChromaffin_cells.Chrom_SOX2_statusHigh.LINC01473	-0.7361059	7.71527457	-2.9123564	0.00473927	0.01427655	-2.8630807	LINC01473	assignmentsChromaffin_cells.Chrom_SOX2_statusHigh
assignmentsChromaffin_cells.Chrom_SOX2_statusHigh.FASTK	1.01343614	4.20884808	2.85953324	0.00550952	0.01616759	-2.8633459	FASTK	assignmentsChromaffin_cells.Chrom_SOX2_statusHigh
assignmentsChromaffin_cells.Chrom_SOX2_statusHigh.B9D2	1.16134037	1.59748711	2.75695772	0.00734359	0.02061193	-2.8669556	B9D2	assignmentsChromaffin_cells.Chrom_SOX2_statusHigh
assignmentsChromaffin_cells.Chrom_SOX2_statusHigh.ASTE1	0.73098468	2.92809678	2.79789866	0.00655317	0.01872435	-2.8679372	ASTE1	assignmentsChromaffin_cells.Chrom_SOX2_statusHigh
assignmentsChromaffin_cells.Chrom_SOX2_statusHigh.DPP6	-1.8682336	8.97412902	-2.9485386	0.00427367	0.01309035	-2.8687335	DPP6	assignmentsChromaffin_cells.Chrom_SOX2_statusHigh
assignmentsChromaffin_cells.Chrom_SOX2_statusHigh.SCP2	0.74563855	5.7534693	2.87221125	0.0053148	0.01569484	-2.8690725	SCP2	assignmentsChromaffin_cells.Chrom_SOX2_statusHigh
assignmentsChromaffin_cells.Chrom_SOX2_statusHigh.COL18A1-AS	2.07463585	-0.7389919	2.57940931	0.01188167	0.03073024	-2.8694113	COL18A1-AS	assignmentsChromaffin_cells.Chrom_SOX2_statusHigh
assignmentsChromaffin_cells.Chrom_SOX2_statusHigh.TRIT1	-0.6437801	4.71853902	-2.8445191	0.00574862	0.01673558	-2.8704368	TRIT1	assignmentsChromaffin_cells.Chrom_SOX2_statusHigh
assignmentsChromaffin_cells.Chrom_SOX2_statusHigh.LINC00954	2.09072201	0.6418674	2.67927549	0.00908973	0.02462934	-2.872074	LINC00954	assignmentsChromaffin_cells.Chrom_SOX2_statusHigh
assignmentsChromaffin_cells.Chrom_SOX2_statusHigh.PPFIBP2	-1.232404	5.76403826	-2.8660159	0.00541071	0.01591945	-2.8728609	PPFIBP2	assignmentsChromaffin_cells.Chrom_SOX2_statusHigh
assignmentsChromaffin_cells.Chrom_SOX2_statusHigh.DLX5	2.07362482	0.10731104	2.58255982	0.0117828	0.03052869	-2.8729911	DLX5	assignmentsChromaffin_cells.Chrom_SOX2_statusHigh
assignmentsChromaffin_cells.Chrom_SOX2_statusHigh.DIAPH2-AS1	-1.5183211	4.0235634	-2.8158748	0.00623472	0.01796809	-2.8780303	DIAPH2-AS1	assignmentsChromaffin_cells.Chrom_SOX2_statusHigh
assignmentsChromaffin_cells.Chrom_SOX2_statusHigh.EDRF1-AS1	-1.3583132	1.11032516	-2.6949592	0.00870769	0.02379925	-2.8792596	EDRF1-AS1	assignmentsChromaffin_cells.Chrom_SOX2_statusHigh
assignmentsChromaffin_cells.Chrom_SOX2_statusHigh.FAM117B	-1.3046482	5.10748604	-2.8276274	0.00603066	0.01746789	-2.8793405	FAM117B	assignmentsChromaffin_cells.Chrom_SOX2_statusHigh
assignmentsChromaffin_cells.Chrom_SOX2_statusHigh.PIK3CA	-0.6393067	6.53309113	-2.8680145	0.00537854	0.01584503	-2.8797613	PIK3CA	assignmentsChromaffin_cells.Chrom_SOX2_statusHigh
assignmentsChromaffin_cells.Chrom_SOX2_statusHigh.TRPC1	-0.6538867	6.37441797	-2.8904939	0.00504514	0.01504255	-2.8807593	TRPC1	assignmentsChromaffin_cells.Chrom_SOX2_statusHigh
assignmentsChromaffin_cells.Chrom_SOX2_statusHigh.EBLN2	-0.8000116	2.11563191	-2.7467072	0.00755471	0.02110169	-2.8815724	EBLN2	assignmentsChromaffin_cells.Chrom_SOX2_statusHigh
assignmentsChromaffin_cells.Chrom_SOX2_statusHigh.XDH	-3.0333912	-0.4806696	-2.5672956	0.01227104	0.03156706	-2.8818266	XDH	assignmentsChromaffin_cells.Chrom_SOX2_statusHigh
assignmentsChromaffin_cells.Chrom_SOX2_statusHigh.ZNF516	-0.8038547	6.42394366	-2.8769744	0.00524329	0.01551511	-2.8825309	ZNF516	assignmentsChromaffin_cells.Chrom_SOX2_statusHigh
assignmentsChromaffin_cells.Chrom_SOX2_statusHigh.ASIC1	-1.3283793	1.17078143	-2.7152784	0.00823706	0.02271815	-2.8834676	ASIC1	assignmentsChromaffin_cells.Chrom_SOX2_statusHigh
assignmentsChromaffin_cells.Chrom_SOX2_statusHigh.COPG2	-0.7624007	5.68819863	-2.8733981	0.0052969	0.0156564	-2.8852326	COPG2	assignmentsChromaffin_cells.Chrom_SOX2_statusHigh
assignmentsChromaffin_cells.Chrom_SOX2_statusHigh.RMI2	1.00773751	1.00649314	2.70069204	0.00857249	0.02348173	-2.8860464	RMI2	assignmentsChromaffin_cells.Chrom_SOX2_statusHigh
assignmentsChromaffin_cells.Chrom_SOX2_statusHigh.TEX261	0.69284511	3.40193841	2.81510329	0.00624494	0.01799431	-2.8870864	TEX261	assignmentsChromaffin_cells.Chrom_SOX2_statusHigh
assignmentsChromaffin_cells.Chrom_SOX2_statusHigh.ZNF589	-0.8142083	4.03737633	-2.8145135	0.00625528	0.0180144	-2.8871026	ZNF589	assignmentsChromaffin_cells.Chrom_SOX2_statusHigh
assignmentsChromaffin_cells.Chrom_SOX2_statusHigh.ZNF138	-0.7879455	5.10558848	-2.8494817	0.00566855	0.0165596	-2.8878635	ZNF138	assignmentsChromaffin_cells.Chrom_SOX2_statusHigh
assignmentsChromaffin_cells.Chrom_SOX2_statusHigh.C2orf92	-0.7561008	3.68314061	-2.7958621	0.00659057	0.01879437	-2.8892621	C2orf92	assignmentsChromaffin_cells.Chrom_SOX2_statusHigh
assignmentsChromaffin_cells.Chrom_SOX2_statusHigh.PHF20	-0.695788	7.47567799	-2.8852013	0.00512187	0.01523166	-2.8895435	PHF20	assignmentsChromaffin_cells.Chrom_SOX2_statusHigh
assignmentsChromaffin_cells.Chrom_SOX2_statusHigh.PTPRT	-1.5380648	1.24363141	-2.6726711	0.00925202	0.02498887	-2.8911681	PTPRT	assignmentsChromaffin_cells.Chrom_SOX2_statusHigh
assignmentsChromaffin_cells.Chrom_SOX2_statusHigh.SCAP	-0.5677192	4.97500727	-2.8464535	0.00571728	0.01667433	-2.8914273	SCAP	assignmentsChromaffin_cells.Chrom_SOX2_statusHigh
assignmentsChromaffin_cells.Chrom_SOX2_statusHigh.COL1A1	2.20629548	3.50829497	2.73119226	0.00788944	0.02190631	-2.891775	COL1A1	assignmentsChromaffin_cells.Chrom_SOX2_statusHigh
assignmentsChromaffin_cells.Chrom_SOX2_statusHigh.SAT1	1.26840118	8.28097924	2.87057935	0.00534227	0.01576146	-2.89205	SAT1	assignmentsChromaffin_cells.Chrom_SOX2_statusHigh
assignmentsChromaffin_cells.Chrom_SOX2_statusHigh.IRF6	1.50513663	0.55660788	2.74751387	0.0075392	0.02107611	-2.8922108	IRF6	assignmentsChromaffin_cells.Chrom_SOX2_statusHigh
assignmentsChromaffin_cells.Chrom_SOX2_statusHigh.WDR25	-0.6998914	5.05564692	-2.8415167	0.00579756	0.01686887	-2.8940336	WDR25	assignmentsChromaffin_cells.Chrom_SOX2_statusHigh
assignmentsChromaffin_cells.Chrom_SOX2_statusHigh.SLC36A1	-1.1936679	5.27331337	-2.8454862	0.00573431	0.01670909	-2.8951631	SLC36A1	assignmentsChromaffin_cells.Chrom_SOX2_statusHigh
assignmentsChromaffin_cells.Chrom_SOX2_statusHigh.TNK2	-0.8543719	4.93028988	-2.8296878	0.00599414	0.01737779	-2.8972453	TNK2	assignmentsChromaffin_cells.Chrom_SOX2_statusHigh
assignmentsChromaffin_cells.Chrom_SOX2_statusHigh.FAM199X	0.38373646	5.08634787	2.85365471	0.00560202	0.0164011	-2.8983813	FAM199X	assignmentsChromaffin_cells.Chrom_SOX2_statusHigh
assignmentsChromaffin_cells.Chrom_SOX2_statusHigh.HOXA10	2.98396938	1.17216991	2.71027831	0.00835544	0.02298929	-2.8998555	HOXA10	assignmentsChromaffin_cells.Chrom_SOX2_statusHigh
assignmentsChromaffin_cells.Chrom_SOX2_statusHigh.KCTD3	-0.7595617	5.68858481	-2.8633432	0.00545032	0.01602425	-2.9001256	KCTD3	assignmentsChromaffin_cells.Chrom_SOX2_statusHigh
assignmentsChromaffin_cells.Chrom_SOX2_statusHigh.ST7	-0.6284324	5.78148951	-2.8690729	0.0053624	0.01580341	-2.9009887	ST7	assignmentsChromaffin_cells.Chrom_SOX2_statusHigh

assignmentsChromaffin_cells.Chrom_SOX2_statusHigh.POMC	2.57683697	0.42494773	2.61231249	0.01088846	0.02856217	-2.9016647	POMC	assignmentsChromaffin_cells.Chrom_SOX2_statusHigh
assignmentsChromaffin_cells.Chrom_SOX2_statusHigh.SOCS2-AS1	-1.1903935	4.07071268	-2.8052057	0.00642125	0.01840644	-2.9047823	SOCS2-AS1	assignmentsChromaffin_cells.Chrom_SOX2_statusHigh
assignmentsChromaffin_cells.Chrom_SOX2_statusHigh.ANKRD54	0.52790752	3.46059153	2.81611885	0.00622717	0.01794955	-2.90525	ANKRD54	assignmentsChromaffin_cells.Chrom_SOX2_statusHigh
assignmentsChromaffin_cells.Chrom_SOX2_statusHigh.ARMH1	-1.0519036	2.81221766	-2.7699827	0.00708313	0.0199542	-2.9064911	ARMH1	assignmentsChromaffin_cells.Chrom_SOX2_statusHigh
assignmentsChromaffin_cells.Chrom_SOX2_statusHigh.PYY	-1.6156435	0.33761635	-2.6341281	0.01026691	0.02723464	-2.9067907	PYY	assignmentsChromaffin_cells.Chrom_SOX2_statusHigh
assignmentsChromaffin_cells.Chrom_SOX2_statusHigh.SETDB2	-0.6355609	5.15793548	-2.8250762	0.00607242	0.01757615	-2.9068961	SETDB2	assignmentsChromaffin_cells.Chrom_SOX2_statusHigh
assignmentsChromaffin_cells.Chrom_SOX2_statusHigh.WNT9A	2.20394936	0.35207049	2.62846106	0.01042638	0.02755286	-2.9072904	WNT9A	assignmentsChromaffin_cells.Chrom_SOX2_statusHigh
assignmentsChromaffin_cells.Chrom_SOX2_statusHigh.MKKS	0.71055685	3.4640168	2.82355333	0.00609848	0.01763566	-2.9087066	MKKS	assignmentsChromaffin_cells.Chrom_SOX2_statusHigh
assignmentsChromaffin_cells.Chrom_SOX2_statusHigh.SNHG15	1.16914993	4.54850257	2.82977393	0.00599383	0.01737779	-2.9129767	SNHG15	assignmentsChromaffin_cells.Chrom_SOX2_statusHigh
assignmentsChromaffin_cells.Chrom_SOX2_statusHigh.LINC01140	1.87124846	-0.1323963	2.5729076	0.01208809	0.03118364	-2.9137596	LINC01140	assignmentsChromaffin_cells.Chrom_SOX2_statusHigh
assignmentsChromaffin_cells.Chrom_SOX2_statusHigh.GDI1	0.80644403	5.63969692	2.86909079	0.00536213	0.01580341	-2.914819	GDI1	assignmentsChromaffin_cells.Chrom_SOX2_statusHigh
assignmentsChromaffin_cells.Chrom_SOX2_statusHigh.KLF7-IT1	-1.219199	0.86446847	-2.6429296	0.01002663	0.02666342	-2.9153792	KLF7-IT1	assignmentsChromaffin_cells.Chrom_SOX2_statusHigh
assignmentsChromaffin_cells.Chrom_SOX2_statusHigh.RPL31	1.60928285	6.45340319	2.87879467	0.00521985	0.01546286	-2.9169794	RPL31	assignmentsChromaffin_cells.Chrom_SOX2_statusHigh
assignmentsChromaffin_cells.Chrom_SOX2_statusHigh.RNF125	-2.1727183	2.80490864	-2.6141856	0.0108324	0.02843158	-2.9204838	RNF125	assignmentsChromaffin_cells.Chrom_SOX2_statusHigh
assignmentsChromaffin_cells.Chrom_SOX2_statusHigh.LEKR1	1.34243812	2.81235885	2.7768717	0.00695007	0.01965183	-2.920925	LEKR1	assignmentsChromaffin_cells.Chrom_SOX2_statusHigh
assignmentsChromaffin_cells.Chrom_SOX2_statusHigh.RNF145	-0.5512832	7.37813117	-2.8785744	0.00521947	0.01546286	-2.9221229	RNF145	assignmentsChromaffin_cells.Chrom_SOX2_statusHigh
assignmentsChromaffin_cells.Chrom_SOX2_statusHigh.MREG	-1.0317978	3.53865559	-2.8126232	0.00628853	0.01810366	-2.9231652	MREG	assignmentsChromaffin_cells.Chrom_SOX2_statusHigh
assignmentsChromaffin_cells.Chrom_SOX2_statusHigh.ST14	1.60152022	0.60705978	2.60404149	0.01112827	0.02909605	-2.9234707	ST14	assignmentsChromaffin_cells.Chrom_SOX2_statusHigh
assignmentsChromaffin_cells.Chrom_SOX2_statusHigh.RINL	1.26392779	2.15160367	2.77563769	0.00697351	0.01970769	-2.9248865	RINL	assignmentsChromaffin_cells.Chrom_SOX2_statusHigh
assignmentsChromaffin_cells.Chrom_SOX2_statusHigh.ME2	-0.5636319	6.32555705	-2.8622884	0.00546665	0.01606342	-2.925634	ME2	assignmentsChromaffin_cells.Chrom_SOX2_statusHigh
assignmentsChromaffin_cells.Chrom_SOX2_statusHigh.ADA2	1.87870156	2.45968737	2.68531626	0.00894158	0.02429929	-2.9257407	ADA2	assignmentsChromaffin_cells.Chrom_SOX2_statusHigh
assignmentsChromaffin_cells.Chrom_SOX2_statusHigh.LINC02422	1.29595661	1.58136634	2.68627813	0.00891614	0.02424911	-2.9274479	LINC02422	assignmentsChromaffin_cells.Chrom_SOX2_statusHigh
assignmentsChromaffin_cells.Chrom_SOX2_statusHigh.SELENOF	0.68071949	5.98078766	2.85479648	0.00558394	0.01636012	-2.9280486	SELENOF	assignmentsChromaffin_cells.Chrom_SOX2_statusHigh
assignmentsChromaffin_cells.Chrom_SOX2_statusHigh.CSTF1	0.65015915	3.40530434	2.80134646	0.00649031	0.01855795	-2.9286714	CSTF1	assignmentsChromaffin_cells.Chrom_SOX2_statusHigh
assignmentsChromaffin_cells.Chrom_SOX2_statusHigh.NDFIP1	0.60832058	7.32214616	2.88635291	0.00510508	0.0151846	-2.9292491	NDFIP1	assignmentsChromaffin_cells.Chrom_SOX2_statusHigh
assignmentsChromaffin_cells.Chrom_SOX2_statusHigh.QRFPR	2.02245473	0.01422028	2.59913244	0.01127488	0.02940269	-2.9295676	QRFPR	assignmentsChromaffin_cells.Chrom_SOX2_statusHigh
assignmentsChromaffin_cells.Chrom_SOX2_statusHigh.FAM226B	-2.8150093	0.29055721	-2.6237636	0.01056196	0.02785149	-2.929619	FAM226B	assignmentsChromaffin_cells.Chrom_SOX2_statusHigh
assignmentsChromaffin_cells.Chrom_SOX2_statusHigh.ADORA2B	1.68710553	0.28078513	2.61417656	0.01083105	0.02843158	-2.9304116	ADORA2B	assignmentsChromaffin_cells.Chrom_SOX2_statusHigh
assignmentsChromaffin_cells.Chrom_SOX2_statusHigh.MCPH1-AS1	-0.8271096	4.03635877	-2.8057797	0.00641028	0.01838157	-2.9308971	MCPH1-AS1	assignmentsChromaffin_cells.Chrom_SOX2_statusHigh
assignmentsChromaffin_cells.Chrom_SOX2_statusHigh.UTP25	0.63042681	3.63762802	2.80336106	0.00645383	0.0184833	-2.9322967	UTP25	assignmentsChromaffin_cells.Chrom_SOX2_statusHigh
assignmentsChromaffin_cells.Chrom_SOX2_statusHigh.DCTN4	-0.5278396	6.8485446	-2.8697423	0.00535221	0.01577919	-2.934215	DCTN4	assignmentsChromaffin_cells.Chrom_SOX2_statusHigh
assignmentsChromaffin_cells.Chrom_SOX2_statusHigh.NT5DC3	1.3584696	2.60932675	2.76110364	0.00726103	0.02039803	-2.9357613	NT5DC3	assignmentsChromaffin_cells.Chrom_SOX2_statusHigh
assignmentsChromaffin_cells.Chrom_SOX2_statusHigh.GALE	0.78181882	2.7928823	2.7683679	0.00711496	0.02002276	-2.9361553	GALE	assignmentsChromaffin_cells.Chrom_SOX2_statusHigh
assignmentsChromaffin_cells.Chrom_SOX2_statusHigh.FRG1-DT	-0.863471	3.60746364	-2.785281	0.00678803	0.01925137	-2.9363344	FRG1-DT	assignmentsChromaffin_cells.Chrom_SOX2_statusHigh
assignmentsChromaffin_cells.Chrom_SOX2_statusHigh.NRBP1	0.50208941	5.50601208	2.8478467	0.00569482	0.01662119	-2.9365953	NRBP1	assignmentsChromaffin_cells.Chrom_SOX2_statusHigh
assignmentsChromaffin_cells.Chrom_SOX2_statusHigh.TRIL	1.86815267	-0.4885487	2.54645873	0.01296161	0.03303326	-2.9371826	TRIL	assignmentsChromaffin_cells.Chrom_SOX2_statusHigh
assignmentsChromaffin_cells.Chrom_SOX2_statusHigh.PTGER3	-2.4572831	3.29235378	-2.7588029	0.0073106	0.02052291	-2.9372634	PTGER3	assignmentsChromaffin_cells.Chrom_SOX2_statusHigh
assignmentsChromaffin_cells.Chrom_SOX2_statusHigh.APBA2	-2.3176827	2.66932793	-2.7058226	0.00845791	0.02322342	-2.9384741	APBA2	assignmentsChromaffin_cells.Chrom_SOX2_statusHigh
assignmentsChromaffin_cells.Chrom_SOX2_statusHigh.MED15	-0.5090059	6.37858447	-2.8501617	0.00565766	0.01653381	-2.9387494	MED15	assignmentsChromaffin_cells.Chrom_SOX2_statusHigh
assignmentsChromaffin_cells.Chrom_SOX2_statusHigh.DLX1	1.76883557	1.65488008	2.77309021	0.00702659	0.01981927	-2.9395267	DLX1	assignmentsChromaffin_cells.Chrom_SOX2_statusHigh
assignmentsChromaffin_cells.Chrom_SOX2_statusHigh.GABBR2	-2.3789784	1.83176624	-2.7356747	0.0077927	0.02167945	-2.9400654	GABBR2	assignmentsChromaffin_cells.Chrom_SOX2_statusHigh
assignmentsChromaffin_cells.Chrom_SOX2_statusHigh.PPP2R5C	-0.740085	7.75905289	-2.8730844	0.00530162	0.01566747	-2.940294	PPP2R5C	assignmentsChromaffin_cells.Chrom_SOX2_statusHigh
assignmentsChromaffin_cells.Chrom_SOX2_statusHigh.GRPEL2	0.74045385	2.78952318	2.77103271	0.00706251	0.01990659	-2.9409826	GRPEL2	assignmentsChromaffin_cells.Chrom_SOX2_statusHigh
assignmentsChromaffin_cells.Chrom_SOX2_statusHigh.DALRD3	0.87291621	3.66458829	2.79633927	0.00658179	0.01877935	-2.9451669	DALRD3	assignmentsChromaffin_cells.Chrom_SOX2_statusHigh
assignmentsChromaffin_cells.Chrom_SOX2_statusHigh.DLG3	-0.8835791	3.96335009	-2.8023485	0.00647214	0.01851591	-2.9459965	DLG3	assignmentsChromaffin_cells.Chrom_SOX2_statusHigh
assignmentsChromaffin_cells.Chrom_SOX2_statusHigh.LINC00159	-0.8592477	2.28323971	-2.7273934	0.00796763	0.02208518	-2.9461774	LINC00159	assignmentsChromaffin_cells.Chrom_SOX2_statusHigh
assignmentsChromaffin_cells.Chrom_SOX2_statusHigh.C7orf31	1.36048013	2.0211315	2.72017772	0.00812713	0.02245571	-2.9464941	C7orf31	assignmentsChromaffin_cells.Chrom_SOX2_statusHigh
assignmentsChromaffin_cells.Chrom_SOX2_statusHigh.PRX	2.36144382	0.60954811	2.58319694	0.01176467	0.03049073	-2.9469164	PRX	assignmentsChromaffin_cells.Chrom_SOX2_statusHigh
assignmentsChromaffin_cells.Chrom_SOX2_statusHigh.GNG3	1.52379725	1.3260413	2.77072388	0.00707156	0.01992861	-2.9473379	GNG3	assignmentsChromaffin_cells.Chrom_SOX2_statusHigh
assignmentsChromaffin_cells.Chrom_SOX2_statusHigh.METTL16	-0.5855734	5.84507	-2.841326	0.00580068	0.01687183	-2.9481344	METTL16	assignmentsChromaffin_cells.Chrom_SOX2_statusHigh
assignmentsChromaffin_cells.Chrom_SOX2_statusHigh.RP9	0.62336551	3.9024179	2.8071797	0.0063852	0.01834168	-2.9496465	RP9	assignmentsChromaffin_cells.Chrom_SOX2_statusHigh
assignmentsChromaffin_cells.Chrom_SOX2_statusHigh.TASOR2	-0.4698212	7.41835339	-2.8697802	0.00535164	0.01577919	-2.9497131	TASOR2	assignmentsChromaffin_cells.Chrom_SOX2_statusHigh
assignmentsChromaffin_cells.Chrom_SOX2_statusHigh.ARMCX3	0.96081216	6.31301987	2.86675374	0.00539834	0.0158918	-2.9530193	ARMCX3	assignmentsChromaffin_cells.Chrom_SOX2_statusHigh
assignmentsChromaffin_cells.Chrom_SOX2_statusHigh.TULP3	-0.5633465	5.43174155	-2.8312998	0.00596699	0.01731161	-2.9553513	TULP3	assignmentsChromaffin_cells.Chrom_SOX2_statusHigh
assignmentsChromaffin_cells.Chrom_SOX2_statusHigh.SIRT6	0.68227218	3.4349625	2.79459248	0.00661398	0.01885108	-2.9555643	SIRT6	assignmentsChromaffin_cells.Chrom_SOX2_statusHigh
assignmentsChromaffin_cells.Chrom_SOX2_statusHigh.DPF1	0.93566766	0.67368227	2.72014239	0.00812791	0.02245571	-2.9561223	DPF1	assignmentsChromaffin_cells.Chrom_SOX2_statusHigh
assignmentsChromaffin_cells.Chrom_SOX2_statusHigh.DDC-AS1	-1.4742231	0.93369039	-2.7143011	0.00826042	0.02277474	-2.9565666	DDC-AS1	assignmentsChromaffin_cells.Chrom_SOX2_statusHigh
assignmentsChromaffin_cells.Chrom_SOX2_statusHigh.EPS15	-0.7153181	7.42319561	-2.8514028	0.00563783	0.0164909	-2.9567601	EPS15	assignmentsChromaffin_cells.Chrom_SOX2_statusHigh
assignmentsChromaffin_cells.Chrom_SOX2_statusHigh.PCSK1	2.51487576	4.06799662	2.84829706	0.00569141	0.01661427	-2.9572167	PCSK1	assignmentsChromaffin_cells.Chrom_SOX2_statusHigh
assignmentsChromaffin_cells.Chrom_SOX2_statusHigh.NCOA7-AS1	-1.7709835	1.62047342	-2.7071743	0.00842501	0.02315677	-2.9596324	NCOA7-AS1	assignmentsChromaffin_cells.Chrom_SOX2_statusHigh
assignmentsChromaffin_cells.Chrom_SOX2_statusHigh.DCT	-1.681975	0.65006289	-2.647101	0.00991458	0.02644437	-2.9609792	DCT	assignmentsChromaffin_cells.Chrom_SOX2_statusHigh
assignmentsChromaffin_cells.Chrom_SOX2_statusHigh.MAN1A1	2.05027038	6.5717541	2.82157426	0.0061365	0.01772006	-2.9618656	MAN1A1	assignmentsChromaffin_cells.Chrom_SOX2_statusHigh
assignmentsChromaffin_cells.Chrom_SOX2_statusHigh.MAP3K5	-0.8508691	7.36247134	-2.8600046	0.00550216	0.01615002	-2.9661819	MAP3K5	assignmentsChromaffin_cells.Chrom_SOX2_statusHigh
assignmentsChromaffin_cells.Chrom_SOX2_statusHigh.CNTN6	2.22159042	1.25686176	2.73332932	0.00784318	0.02179674	-2.9662437	CNTN6	assignmentsChromaffin_cells.Chrom_SOX2_statusHigh

assignmentsChromaffin_cells.Chrom_SOX2_statusHigh.MFSD1	0.81089855	5.43040115	2.82008403	0.00615822	0.01777638	-2.9663838	MFSD1	assignmentsChromaffin_cells.Chrom_SOX2_statusHigh
assignmentsChromaffin_cells.Chrom_SOX2_statusHigh.RPL7L1	0.65413209	5.20910012	2.83269301	0.00594362	0.01725005	-2.9666486	RPL7L1	assignmentsChromaffin_cells.Chrom_SOX2_statusHigh
assignmentsChromaffin_cells.Chrom_SOX2_statusHigh.NKAPD1	0.60805236	3.79467895	2.79068342	0.00668655	0.01902288	-2.9686231	NKAPD1	assignmentsChromaffin_cells.Chrom_SOX2_statusHigh
assignmentsChromaffin_cells.Chrom_SOX2_statusHigh.TTC22	-1.8024388	0.89661311	-2.6945173	0.00872112	0.02382119	-2.9703517	TTC22	assignmentsChromaffin_cells.Chrom_SOX2_statusHigh
assignmentsChromaffin_cells.Chrom_SOX2_statusHigh.ABLIM2	-1.4300023	4.5189848	-2.7772852	0.00694322	0.01963593	-2.9712036	ABLIM2	assignmentsChromaffin_cells.Chrom_SOX2_statusHigh
assignmentsChromaffin_cells.Chrom_SOX2_statusHigh.ZNF585B	-0.6345393	4.70873535	-2.8023808	0.00647155	0.01851591	-2.971972	ZNF585B	assignmentsChromaffin_cells.Chrom_SOX2_statusHigh
assignmentsChromaffin_cells.Chrom_SOX2_statusHigh.CHRNE	-1.2141203	2.32659684	-2.6865567	0.00890938	0.02423895	-2.9727043	CHRNE	assignmentsChromaffin_cells.Chrom_SOX2_statusHigh
assignmentsChromaffin_cells.Chrom_SOX2_statusHigh.CADPS	-2.2825021	5.87621283	-2.8416272	0.00579963	0.01687183	-2.9761594	CADPS	assignmentsChromaffin_cells.Chrom_SOX2_statusHigh
assignmentsChromaffin_cells.Chrom_SOX2_statusHigh.RRP1B	-0.5265966	5.34784751	-2.817175	0.00620873	0.01790092	-2.9777827	RRP1B	assignmentsChromaffin_cells.Chrom_SOX2_statusHigh
assignmentsChromaffin_cells.Chrom_SOX2_statusHigh.MADD	-0.6086125	5.57765991	-2.8236893	0.00609615	0.0176321	-2.9781427	MADD	assignmentsChromaffin_cells.Chrom_SOX2_statusHigh
assignmentsChromaffin_cells.Chrom_SOX2_statusHigh.CLGN	1.44759328	3.62477802	2.83017831	0.00598982	0.01737155	-2.9796922	CLGN	assignmentsChromaffin_cells.Chrom_SOX2_statusHigh
assignmentsChromaffin_cells.Chrom_SOX2_statusHigh.SSPN	1.24317834	2.08798533	2.68205559	0.00901916	0.02446707	-2.9800379	SSPN	assignmentsChromaffin_cells.Chrom_SOX2_statusHigh
assignmentsChromaffin_cells.Chrom_SOX2_statusHigh.ABCB9	-0.7406942	3.52136295	-2.7790812	0.00690624	0.01956239	-2.9822365	ABCB9	assignmentsChromaffin_cells.Chrom_SOX2_statusHigh
assignmentsChromaffin_cells.Chrom_SOX2_statusHigh.DNAH1	-1.0947499	3.400246	-2.7119925	0.00831156	0.0228882	-2.9832324	DNAH1	assignmentsChromaffin_cells.Chrom_SOX2_statusHigh
assignmentsChromaffin_cells.Chrom_SOX2_statusHigh.AXIN1	-0.6085183	4.43822217	-2.7757465	0.0069706	0.01970293	-2.9838978	AXIN1	assignmentsChromaffin_cells.Chrom_SOX2_statusHigh
assignmentsChromaffin_cells.Chrom_SOX2_statusHigh.RBM18	0.5162123	4.03942354	2.78960528	0.00670669	0.01906449	-2.983984	RBM18	assignmentsChromaffin_cells.Chrom_SOX2_statusHigh
assignmentsChromaffin_cells.Chrom_SOX2_statusHigh.PLEKHG1	-2.5217985	4.98605935	-2.6148133	0.0108181	0.02841479	-2.9844449	PLEKHG1	assignmentsChromaffin_cells.Chrom_SOX2_statusHigh
assignmentsChromaffin_cells.Chrom_SOX2_statusHigh.GUCD1	0.68044785	2.72555654	2.75279665	0.00742863	0.02080692	-2.985128	GUCD1	assignmentsChromaffin_cells.Chrom_SOX2_statusHigh
assignmentsChromaffin_cells.Chrom_SOX2_statusHigh.HLA-B	1.84246898	7.36257646	2.83661495	0.0058822	0.01708415	-2.9883864	HLA-B	assignmentsChromaffin_cells.Chrom_SOX2_statusHigh
assignmentsChromaffin_cells.Chrom_SOX2_statusHigh.KATNB1	0.62348965	2.46689037	2.75523985	0.00737859	0.02069011	-2.9885381	KATNB1	assignmentsChromaffin_cells.Chrom_SOX2_statusHigh
assignmentsChromaffin_cells.Chrom_SOX2_statusHigh.SMAD1-AS2	-1.8517766	-0.3316692	-2.5519829	0.01277459	0.03261365	-2.9918555	SMAD1-AS2	assignmentsChromaffin_cells.Chrom_SOX2_statusHigh
assignmentsChromaffin_cells.Chrom_SOX2_statusHigh.PATJ	-0.936566	6.57616544	-2.8533196	0.00560737	0.01641376	-2.9920894	PATJ	assignmentsChromaffin_cells.Chrom_SOX2_statusHigh
assignmentsChromaffin_cells.Chrom_SOX2_statusHigh.EIF4EBP2	0.72211673	4.54945161	2.7868879	0.0067577	0.0191823	-2.9927835	EIF4EBP2	assignmentsChromaffin_cells.Chrom_SOX2_statusHigh
assignmentsChromaffin_cells.Chrom_SOX2_statusHigh.ZNF692	-0.7777976	4.00175815	-2.7861891	0.00677088	0.0192129	-2.9930908	ZNF692	assignmentsChromaffin_cells.Chrom_SOX2_statusHigh
assignmentsChromaffin_cells.Chrom_SOX2_statusHigh.TRHDE-AS1	2.6724011	0.16652942	2.65698747	0.00965859	0.0259081	-2.9938694	TRHDE-AS1	assignmentsChromaffin_cells.Chrom_SOX2_statusHigh
assignmentsChromaffin_cells.Chrom_SOX2_statusHigh.ABCB1	0.99722963	5.75741707	2.8522235	0.00562586	0.01646188	-2.9941175	ABCB1	assignmentsChromaffin_cells.Chrom_SOX2_statusHigh
assignmentsChromaffin_cells.Chrom_SOX2_statusHigh.SDR39U1	0.90368437	4.55268409	2.809462	0.00634451	0.01824842	-2.9943448	SDR39U1	assignmentsChromaffin_cells.Chrom_SOX2_statusHigh
assignmentsChromaffin_cells.Chrom_SOX2_statusHigh.RPA1	-0.6526732	5.16358767	-2.8051907	0.00642086	0.01840644	-2.9944029	RPA1	assignmentsChromaffin_cells.Chrom_SOX2_statusHigh
assignmentsChromaffin_cells.Chrom_SOX2_statusHigh.LYSMD1	0.68186574	1.87411479	2.74145094	0.0076651	0.0213722	-2.9948396	LYSMD1	assignmentsChromaffin_cells.Chrom_SOX2_statusHigh
assignmentsChromaffin_cells.Chrom_SOX2_statusHigh.DHX38	-0.4910759	4.89472989	-2.7966131	0.00657676	0.01877167	-2.9949057	DHX38	assignmentsChromaffin_cells.Chrom_SOX2_statusHigh
assignmentsChromaffin_cells.Chrom_SOX2_statusHigh.SH2B1	-0.6591676	4.66199945	-2.7926286	0.00665035	0.0189379	-2.9951682	SH2B1	assignmentsChromaffin_cells.Chrom_SOX2_statusHigh
assignmentsChromaffin_cells.Chrom_SOX2_statusHigh.CAST	0.85249598	7.78297545	2.85949184	0.00551017	0.01616759	-2.9963848	CAST	assignmentsChromaffin_cells.Chrom_SOX2_statusHigh
assignmentsChromaffin_cells.Chrom_SOX2_statusHigh.WDR55	0.53439706	3.9166398	2.77732425	0.00694008	0.01963397	-2.9974398	WDR55	assignmentsChromaffin_cells.Chrom_SOX2_statusHigh
assignmentsChromaffin_cells.Chrom_SOX2_statusHigh.LINC01341	-1.4267491	1.6944297	-2.6913746	0.0087937	0.02396897	-2.9979885	LINC01341	assignmentsChromaffin_cells.Chrom_SOX2_statusHigh
assignmentsChromaffin_cells.Chrom_SOX2_statusHigh.C1orf174	0.81185717	3.0711485	2.7465933	0.00755708	0.02110404	-3.000792	C1orf174	assignmentsChromaffin_cells.Chrom_SOX2_statusHigh
assignmentsChromaffin_cells.Chrom_SOX2_statusHigh.ZFHX2-AS1	-0.8649125	2.88682823	-2.7372953	0.00775342	0.02157719	-3.0024133	ZFHX2-AS1	assignmentsChromaffin_cells.Chrom_SOX2_statusHigh
assignmentsChromaffin_cells.Chrom_SOX2_statusHigh.HSF2BP	-1.1283931	2.35675236	-2.7156616	0.00822842	0.0227021	-3.0027307	HSF2BP	assignmentsChromaffin_cells.Chrom_SOX2_statusHigh
assignmentsChromaffin_cells.Chrom_SOX2_statusHigh.GPR18	-2.3462879	0.54879481	-2.5529681	0.01274325	0.03254486	-3.0042141	GPR18	assignmentsChromaffin_cells.Chrom_SOX2_statusHigh
assignmentsChromaffin_cells.Chrom_SOX2_statusHigh.LIMCH1	-1.296341	6.86666597	-2.8458664	0.00573063	0.01670141	-3.004321	LIMCH1	assignmentsChromaffin_cells.Chrom_SOX2_statusHigh
assignmentsChromaffin_cells.Chrom_SOX2_statusHigh.NOC3L	0.65725173	4.76168665	2.79725958	0.00656489	0.0187478	-3.0053748	NOC3L	assignmentsChromaffin_cells.Chrom_SOX2_statusHigh
assignmentsChromaffin_cells.Chrom_SOX2_statusHigh.FCGR2A	1.72439478	2.64373408	2.61946302	0.01067935	0.02810559	-3.0065083	FCGR2A	assignmentsChromaffin_cells.Chrom_SOX2_statusHigh
assignmentsChromaffin_cells.Chrom_SOX2_statusHigh.LHFPL4	-0.9722418	4.50606896	-2.818739	0.00618154	0.01783405	-3.0077177	LHFPL4	assignmentsChromaffin_cells.Chrom_SOX2_statusHigh
assignmentsChromaffin_cells.Chrom_SOX2_statusHigh.PCDHA9	-1.2131619	1.24884878	-2.6958072	0.00868757	0.02375235	-3.008667	PCDHA9	assignmentsChromaffin_cells.Chrom_SOX2_statusHigh
assignmentsChromaffin_cells.Chrom_SOX2_statusHigh.MEIS1-AS2	-1.7373157	0.6487334	-2.6177655	0.01072818	0.02820171	-3.0090479	MEIS1-AS2	assignmentsChromaffin_cells.Chrom_SOX2_statusHigh
assignmentsChromaffin_cells.Chrom_SOX2_statusHigh.UBE2E3	-0.6446675	6.71454217	-2.8270601	0.00603863	0.01748781	-3.0090664	UBE2E3	assignmentsChromaffin_cells.Chrom_SOX2_statusHigh
assignmentsChromaffin_cells.Chrom_SOX2_statusHigh.THNSL2	2.19247429	1.74984196	2.68159548	0.00903543	0.02450706	-3.0122644	THNSL2	assignmentsChromaffin_cells.Chrom_SOX2_statusHigh
assignmentsChromaffin_cells.Chrom_SOX2_statusHigh.SF3A2	0.65020223	4.34771669	2.79640309	0.00658062	0.01877934	-3.0135896	SF3A2	assignmentsChromaffin_cells.Chrom_SOX2_statusHigh
assignmentsChromaffin_cells.Chrom_SOX2_statusHigh.SYNJ2BP	0.58586694	4.71728461	2.80757134	0.0063782	0.01833219	-3.0140388	SYNJ2BP	assignmentsChromaffin_cells.Chrom_SOX2_statusHigh
assignmentsChromaffin_cells.Chrom_SOX2_statusHigh.PHKA1	-0.8329539	4.91506523	-2.8189476	0.00617791	0.0178268	-3.0146463	PHKA1	assignmentsChromaffin_cells.Chrom_SOX2_statusHigh
assignmentsChromaffin_cells.Chrom_SOX2_statusHigh.NOTCH2NLB	2.94631664	0.48011516	2.5916693	0.01150676	0.02988589	-3.0155885	NOTCH2NLB	assignmentsChromaffin_cells.Chrom_SOX2_statusHigh
assignmentsChromaffin_cells.Chrom_SOX2_statusHigh.ATF3	1.90375351	6.37013229	2.82275489	0.00611618	0.0176773	-3.0167166	ATF3	assignmentsChromaffin_cells.Chrom_SOX2_statusHigh
assignmentsChromaffin_cells.Chrom_SOX2_statusHigh.FECH	-0.6202463	3.85090461	-2.7684056	0.00711421	0.02002276	-3.0174655	FECH	assignmentsChromaffin_cells.Chrom_SOX2_statusHigh
assignmentsChromaffin_cells.Chrom_SOX2_statusHigh.HTRA3	2.22008651	0.20509841	2.52233069	0.01380776	0.03474797	-3.0194589	HTRA3	assignmentsChromaffin_cells.Chrom_SOX2_statusHigh
assignmentsChromaffin_cells.Chrom_SOX2_statusHigh.VPS37C	-0.6365975	3.98185062	-2.7552106	0.00737919	0.02069011	-3.0198887	VPS37C	assignmentsChromaffin_cells.Chrom_SOX2_statusHigh
assignmentsChromaffin_cells.Chrom_SOX2_statusHigh.CAPN15	-0.7569462	3.83342803	-2.7477971	0.007532	0.02106333	-3.0211188	CAPN15	assignmentsChromaffin_cells.Chrom_SOX2_statusHigh
assignmentsChromaffin_cells.Chrom_SOX2_statusHigh.NUP58	-0.5346496	6.81728927	-2.8364971	0.00588024	0.01708156	-3.022625	NUP58	assignmentsChromaffin_cells.Chrom_SOX2_statusHigh
assignmentsChromaffin_cells.Chrom_SOX2_statusHigh.SYMPK	-0.375499	5.80778536	-2.8113169	0.00631161	0.01816031	-3.0243606	SYMPK	assignmentsChromaffin_cells.Chrom_SOX2_statusHigh
assignmentsChromaffin_cells.Chrom_SOX2_statusHigh.VPS13D	-0.902409	7.47661362	-2.83316	0.00593581	0.0172305	-3.0248201	VPS13D	assignmentsChromaffin_cells.Chrom_SOX2_statusHigh
assignmentsChromaffin_cells.Chrom_SOX2_statusHigh.SHFL	1.37855939	4.87033	2.77474289	0.00699305	0.01974899	-3.0254915	SHFL	assignmentsChromaffin_cells.Chrom_SOX2_statusHigh
assignmentsChromaffin_cells.Chrom_SOX2_statusHigh.DHX40	0.44271948	5.00822396	2.80709741	0.00638667	0.01834168	-3.0255404	DHX40	assignmentsChromaffin_cells.Chrom_SOX2_statusHigh
assignmentsChromaffin_cells.Chrom_SOX2_statusHigh.TRIM7	1.50162295	0.51221014	2.64722578	0.00991122	0.02644437	-3.0268954	TRIM7	assignmentsChromaffin_cells.Chrom_SOX2_statusHigh
assignmentsChromaffin_cells.Chrom_SOX2_statusHigh.PSD3	1.53992797	6.06631587	2.78794821	0.00674198	0.01914446	-3.0289168	PSD3	assignmentsChromaffin_cells.Chrom_SOX2_statusHigh
assignmentsChromaffin_cells.Chrom_SOX2_statusHigh.ZNF212	-0.7473085	3.45922546	-2.7477148	0.00753371	0.02106445	-3.0299081	ZNF212	assignmentsChromaffin_cells.Chrom_SOX2_statusHigh
assignmentsChromaffin_cells.Chrom_SOX2_statusHigh.MMD	0.79748511	5.15770305	2.82441652	0.0060837	0.01760244	-3.0332288	MMD	assignmentsChromaffin_cells.Chrom_SOX2_statusHigh

assignmentsChromaffin_cells.Chrom_SOX2_statusHigh.TP53INP2	0.69521914	3.29180737	2.75595026	0.0073641	0.02065864	-3.0333944	TP53INP2	assignmentsChromaffin_cells.Chrom_SOX2_statusHigh
assignmentsChromaffin_cells.Chrom_SOX2_statusHigh.SLC6A6	1.20510845	4.28481497	2.75653061	0.00735357	0.0206327	-3.033911	SLC6A6	assignmentsChromaffin_cells.Chrom_SOX2_statusHigh
assignmentsChromaffin_cells.Chrom_SOX2_statusHigh.TNFAIP1	0.6961552	4.29686644	2.77504033	0.0069843	0.01973122	-3.0342425	TNFAIP1	assignmentsChromaffin_cells.Chrom_SOX2_statusHigh
assignmentsChromaffin_cells.Chrom_SOX2_statusHigh.FYCO1	-0.6613897	4.30265067	-2.7379691	0.00773904	0.0215409	-3.0402291	FYCO1	assignmentsChromaffin_cells.Chrom_SOX2_statusHigh
assignmentsChromaffin_cells.Chrom_SOX2_statusHigh.ZCWPW2	-0.5856774	5.19702284	-2.7901234	0.006697	0.01904581	-3.0409779	ZCWPW2	assignmentsChromaffin_cells.Chrom_SOX2_statusHigh
assignmentsChromaffin_cells.Chrom_SOX2_statusHigh.SCRN2	0.78056052	3.6274516	2.77467423	0.00699141	0.01974783	-3.0416455	SCRN2	assignmentsChromaffin_cells.Chrom_SOX2_statusHigh
assignmentsChromaffin_cells.Chrom_SOX2_statusHigh.TNPO1	-0.4769353	7.65672081	-2.8461215	0.00572265	0.01668118	-3.0424156	TNPO1	assignmentsChromaffin_cells.Chrom_SOX2_statusHigh
assignmentsChromaffin_cells.Chrom_SOX2_statusHigh.ZBP1	2.08381243	-0.0167785	2.50532575	0.01443367	0.03609091	-3.042975	ZBP1	assignmentsChromaffin_cells.Chrom_SOX2_statusHigh
assignmentsChromaffin_cells.Chrom_SOX2_statusHigh.MT-CO3	2.5942359	9.58149613	2.88538451	0.00512281	0.01523166	-3.0449403	MT-CO3	assignmentsChromaffin_cells.Chrom_SOX2_statusHigh
assignmentsChromaffin_cells.Chrom_SOX2_statusHigh.PYROXD2	2.0486437	1.80172604	2.59834121	0.01130101	0.02945168	-3.0453727	PYROXD2	assignmentsChromaffin_cells.Chrom_SOX2_statusHigh
assignmentsChromaffin_cells.Chrom_SOX2_statusHigh.BIRC5	1.55626388	0.95428195	2.64717757	0.00991379	0.02644437	-3.0470307	BIRC5	assignmentsChromaffin_cells.Chrom_SOX2_statusHigh
assignmentsChromaffin_cells.Chrom_SOX2_statusHigh.UBTF	0.6445473	4.97770378	2.78875236	0.00672266	0.01909888	-3.0472417	UBTF	assignmentsChromaffin_cells.Chrom_SOX2_statusHigh
assignmentsChromaffin_cells.Chrom_SOX2_statusHigh.ZNF777	-0.7229679	3.00075357	-2.7267495	0.00798175	0.02212048	-3.0472547	ZNF777	assignmentsChromaffin_cells.Chrom_SOX2_statusHigh
assignmentsChromaffin_cells.Chrom_SOX2_statusHigh.MAFTRR	1.88377639	-0.3162353	2.50707445	0.01436814	0.03596072	-3.0478791	MAFTRR	assignmentsChromaffin_cells.Chrom_SOX2_statusHigh
assignmentsChromaffin_cells.Chrom_SOX2_statusHigh.ZNF771	0.71213972	2.71356017	2.74124859	0.00766938	0.0213767	-3.048444	ZNF771	assignmentsChromaffin_cells.Chrom_SOX2_statusHigh
assignmentsChromaffin_cells.Chrom_SOX2_statusHigh.OVOL2	-1.5937106	1.48616912	-2.6243989	0.01053885	0.02779511	-3.0487096	OVOL2	assignmentsChromaffin_cells.Chrom_SOX2_statusHigh
assignmentsChromaffin_cells.Chrom_SOX2_statusHigh.PPP4R3B	-0.4258862	7.00134016	-2.8258995	0.00605838	0.01754184	-3.0488052	PPP4R3B	assignmentsChromaffin_cells.Chrom_SOX2_statusHigh
assignmentsChromaffin_cells.Chrom_SOX2_statusHigh.CD47	0.66340648	6.62394384	2.82134428	0.00613646	0.01772006	-3.0497327	CD47	assignmentsChromaffin_cells.Chrom_SOX2_statusHigh
assignmentsChromaffin_cells.Chrom_SOX2_statusHigh.C1orf226	-1.8580794	1.9838901	-2.7071005	0.00842841	0.02315827	-3.0515926	C1orf226	assignmentsChromaffin_cells.Chrom_SOX2_statusHigh
assignmentsChromaffin_cells.Chrom_SOX2_statusHigh.VSTM4	1.84253674	2.62166673	2.70089866	0.00857249	0.02348173	-3.0532423	VSTM4	assignmentsChromaffin_cells.Chrom_SOX2_statusHigh
assignmentsChromaffin_cells.Chrom_SOX2_statusHigh.ZNF491	-1.414654	2.36043143	-2.6811533	0.00904256	0.02451809	-3.0540263	ZNF491	assignmentsChromaffin_cells.Chrom_SOX2_statusHigh
assignmentsChromaffin_cells.Chrom_SOX2_statusHigh.TANC1	-0.9142116	6.77727029	-2.8121588	0.00629673	0.018124	-3.0541503	TANC1	assignmentsChromaffin_cells.Chrom_SOX2_statusHigh
assignmentsChromaffin_cells.Chrom_SOX2_statusHigh.PDPR	-0.6209734	5.09145055	-2.7797256	0.00689387	0.01953425	-3.0543312	PDPR	assignmentsChromaffin_cells.Chrom_SOX2_statusHigh
assignmentsChromaffin_cells.Chrom_SOX2_statusHigh.FBXO10	-0.6634299	5.01684791	-2.801481	0.00648786	0.01855427	-3.0545852	FBXO10	assignmentsChromaffin_cells.Chrom_SOX2_statusHigh
assignmentsChromaffin_cells.Chrom_SOX2_statusHigh.DAP	0.75522904	5.6408889	2.78707727	0.00675413	0.01917557	-3.0549989	DAP	assignmentsChromaffin_cells.Chrom_SOX2_statusHigh
assignmentsChromaffin_cells.Chrom_SOX2_statusHigh.ERC2	-2.5256723	4.54955339	-2.7689163	0.0071085	0.02001511	-3.0562353	ERC2	assignmentsChromaffin_cells.Chrom_SOX2_statusHigh
assignmentsChromaffin_cells.Chrom_SOX2_statusHigh.CARTPT	4.86438064	6.68643459	2.8677579	0.00538618	0.01586183	-3.0563314	CARTPT	assignmentsChromaffin_cells.Chrom_SOX2_statusHigh
assignmentsChromaffin_cells.Chrom_SOX2_statusHigh.LINC01535	-1.4928883	2.96193618	-2.7283468	0.00794997	0.02204663	-3.0575817	LINC01535	assignmentsChromaffin_cells.Chrom_SOX2_statusHigh
assignmentsChromaffin_cells.Chrom_SOX2_statusHigh.RPRML	2.30129238	-0.0252307	2.58791725	0.01162148	0.03014968	-3.0581705	RPRML	assignmentsChromaffin_cells.Chrom_SOX2_statusHigh
assignmentsChromaffin_cells.Chrom_SOX2_statusHigh.HPGD	-3.2319666	2.27716441	-2.6487177	0.00987663	0.02637826	-3.0603875	HPGD	assignmentsChromaffin_cells.Chrom_SOX2_statusHigh
assignmentsChromaffin_cells.Chrom_SOX2_statusHigh.SYTL5	2.4535565	0.0298044	2.56536733	0.01233679	0.03170284	-3.0609103	SYTL5	assignmentsChromaffin_cells.Chrom_SOX2_statusHigh
assignmentsChromaffin_cells.Chrom_SOX2_statusHigh.PAXBP1-AS	-0.6690758	3.92648348	-2.7607266	0.00726734	0.02041219	-3.0633942	PAXBP1-AS1	assignmentsChromaffin_cells.Chrom_SOX2_statusHigh
assignmentsChromaffin_cells.Chrom_SOX2_statusHigh.LRRC4	-1.7895378	1.52354317	-2.6477693	0.00989959	0.02643077	-3.0650069	LRRC4	assignmentsChromaffin_cells.Chrom_SOX2_statusHigh
assignmentsChromaffin_cells.Chrom_SOX2_statusHigh.NECTIN2	1.42623872	5.44887649	2.77803415	0.00693069	0.01961086	-3.0655373	NECTIN2	assignmentsChromaffin_cells.Chrom_SOX2_statusHigh
assignmentsChromaffin_cells.Chrom_SOX2_statusHigh.DDX20	0.50442047	2.88023802	2.71127754	0.00832785	0.02292302	-3.0659304	DDX20	assignmentsChromaffin_cells.Chrom_SOX2_statusHigh
assignmentsChromaffin_cells.Chrom_SOX2_statusHigh.EPHA5-AS1	3.33782549	0.80606311	2.65765246	0.00964125	0.02587456	-3.0660332	EPHA5-AS1	assignmentsChromaffin_cells.Chrom_SOX2_statusHigh
assignmentsChromaffin_cells.Chrom_SOX2_statusHigh.FBXW11	-0.4974158	7.54463489	-2.8231596	0.00610523	0.01765201	-3.0681443	FBXW11	assignmentsChromaffin_cells.Chrom_SOX2_statusHigh
assignmentsChromaffin_cells.Chrom_SOX2_statusHigh.MARS2	0.98299674	0.77280816	2.64381843	0.01000265	0.02663056	-3.0692067	MARS2	assignmentsChromaffin_cells.Chrom_SOX2_statusHigh
assignmentsChromaffin_cells.Chrom_SOX2_statusHigh.OPN4	2.17344897	-0.9759363	2.47694823	0.0155352	0.03854479	-3.0716835	OPN4	assignmentsChromaffin_cells.Chrom_SOX2_statusHigh
assignmentsChromaffin_cells.Chrom_SOX2_statusHigh.JPX	-0.716082	7.55761168	-2.8285396	0.00601354	0.01742775	-3.0720608	JPX	assignmentsChromaffin_cells.Chrom_SOX2_statusHigh
assignmentsChromaffin_cells.Chrom_SOX2_statusHigh.CCNL2	-0.6435131	6.51750882	-2.7983483	0.00654494	0.0187075	-3.0725987	CCNL2	assignmentsChromaffin_cells.Chrom_SOX2_statusHigh
assignmentsChromaffin_cells.Chrom_SOX2_statusHigh.GRK7	1.55382954	0.79446615	2.560362	0.01249557	0.03205382	-3.0734734	GRK7	assignmentsChromaffin_cells.Chrom_SOX2_statusHigh
assignmentsChromaffin_cells.Chrom_SOX2_statusHigh.ATP8B4	-1.7487639	3.93859516	-2.6028837	0.0111643	0.02916649	-3.0736475	ATP8B4	assignmentsChromaffin_cells.Chrom_SOX2_statusHigh
assignmentsChromaffin_cells.Chrom_SOX2_statusHigh.ZNF625	-0.7329592	3.9323627	-2.7471267	0.00754596	0.02108766	-3.0744232	ZNF625	assignmentsChromaffin_cells.Chrom_SOX2_statusHigh
assignmentsChromaffin_cells.Chrom_SOX2_statusHigh.CXCL8	3.63028077	1.12420839	2.57134882	0.01214387	0.03129734	-3.0747047	CXCL8	assignmentsChromaffin_cells.Chrom_SOX2_statusHigh
assignmentsChromaffin_cells.Chrom_SOX2_statusHigh.ELOVL2	-1.2420645	3.26776588	-2.7239425	0.00804479	0.02227211	-3.0755314	ELOVL2	assignmentsChromaffin_cells.Chrom_SOX2_statusHigh
assignmentsChromaffin_cells.Chrom_SOX2_statusHigh.C11orf52	1.3712934	-0.113915	2.51168752	0.01419658	0.03558685	-3.0766808	C11orf52	assignmentsChromaffin_cells.Chrom_SOX2_statusHigh
assignmentsChromaffin_cells.Chrom_SOX2_statusHigh.ALG9	-0.5838391	5.08712058	-2.7763532	0.00695885	0.01967319	-3.0767519	ALG9	assignmentsChromaffin_cells.Chrom_SOX2_statusHigh
assignmentsChromaffin_cells.Chrom_SOX2_statusHigh.DKK3	2.32866618	3.99693493	2.75021783	0.00748627	0.02094642	-3.0785498	DKK3	assignmentsChromaffin_cells.Chrom_SOX2_statusHigh
assignmentsChromaffin_cells.Chrom_SOX2_statusHigh.DLEC1	-1.3648888	0.82419596	-2.5209324	0.01385828	0.03485872	-3.0785685	DLEC1	assignmentsChromaffin_cells.Chrom_SOX2_statusHigh
assignmentsChromaffin_cells.Chrom_SOX2_statusHigh.PRKAA2	-1.3532371	3.0983543	-2.7303561	0.00790492	0.02194168	-3.079278	PRKAA2	assignmentsChromaffin_cells.Chrom_SOX2_statusHigh
assignmentsChromaffin_cells.Chrom_SOX2_statusHigh.VTI1B	0.52274905	5.1093607	2.78639574	0.00676698	0.01920523	-3.0794798	VTI1B	assignmentsChromaffin_cells.Chrom_SOX2_statusHigh
assignmentsChromaffin_cells.Chrom_SOX2_statusHigh.FAM13A-AS	-0.7605905	3.21673003	-2.6917202	0.00878494	0.02394916	-3.0798308	FAM13A-AS1	assignmentsChromaffin_cells.Chrom_SOX2_statusHigh
assignmentsChromaffin_cells.Chrom_SOX2_statusHigh.SAMD8	-0.5989703	6.03231164	-2.7857816	0.00677857	0.01922793	-3.0809956	SAMD8	assignmentsChromaffin_cells.Chrom_SOX2_statusHigh
assignmentsChromaffin_cells.Chrom_SOX2_statusHigh.DMRTC1B	-2.2045159	-0.5755343	-2.5048512	0.0144518	0.03613063	-3.0868771	DMRTC1B	assignmentsChromaffin_cells.Chrom_SOX2_statusHigh
assignmentsChromaffin_cells.Chrom_SOX2_statusHigh.TAB2	-0.6523725	6.87602952	-2.7975178	0.00656015	0.01873761	-3.0869696	TAB2	assignmentsChromaffin_cells.Chrom_SOX2_statusHigh
assignmentsChromaffin_cells.Chrom_SOX2_statusHigh.SLC12A1	-1.3461649	0.24532272	-2.5788614	0.01189894	0.03076995	-3.0872122	SLC12A1	assignmentsChromaffin_cells.Chrom_SOX2_statusHigh
assignmentsChromaffin_cells.Chrom_SOX2_statusHigh.EDDM13	-1.8772868	1.68269544	-2.6421958	0.01005074	0.02671425	-3.0874907	EDDM13	assignmentsChromaffin_cells.Chrom_SOX2_statusHigh
assignmentsChromaffin_cells.Chrom_SOX2_statusHigh.PACRGL	-0.5914784	5.40541878	-2.7799437	0.00688968	0.01952585	-3.0881064	PACRGL	assignmentsChromaffin_cells.Chrom_SOX2_statusHigh
assignmentsChromaffin_cells.Chrom_SOX2_statusHigh.FEZ1	1.08794295	3.50270124	2.74685575	0.00755161	0.02109979	-3.0917419	FEZ1	assignmentsChromaffin_cells.Chrom_SOX2_statusHigh
assignmentsChromaffin_cells.Chrom_SOX2_statusHigh.MEF2A	-0.6097546	8.42475543	-2.8208238	0.00614544	0.01774267	-3.0931252	MEF2A	assignmentsChromaffin_cells.Chrom_SOX2_statusHigh
assignmentsChromaffin_cells.Chrom_SOX2_statusHigh.SIK1	2.28920657	4.05950572	2.7517933	0.00745375	0.02087362	-3.0934136	SIK1	assignmentsChromaffin_cells.Chrom_SOX2_statusHigh
assignmentsChromaffin_cells.Chrom_SOX2_statusHigh.ZNF678	-0.6987039	5.32836991	-2.7645566	0.00719059	0.02021433	-3.0945814	ZNF678	assignmentsChromaffin_cells.Chrom_SOX2_statusHigh
assignmentsChromaffin_cells.Chrom_SOX2_statusHigh.PER3	-0.6994119	6.74471551	-2.8077738	0.00637459	0.01832836	-3.0953079	PER3	assignmentsChromaffin_cells.Chrom_SOX2_statusHigh

assignmentsChromaffin_cells.Chrom_SOX2_statusHigh.RERE-AS1	-1.5138657	0.63506308	-2.5412706	0.0131395	0.0334017	-3.0954349	RERE-AS1	assignmentsChromaffin_cells.Chrom_SOX2_statusHigh
assignmentsChromaffin_cells.Chrom_SOX2_statusHigh.ARC	2.93058904	3.19946719	2.74337336	0.00762909	0.02128289	-3.0973472	ARC	assignmentsChromaffin_cells.Chrom_SOX2_statusHigh
assignmentsChromaffin_cells.Chrom_SOX2_statusHigh.UPP2	-1.3804395	3.41767279	-2.7196161	0.00814162	0.02248437	-3.09869	UPP2	assignmentsChromaffin_cells.Chrom_SOX2_statusHigh
assignmentsChromaffin_cells.Chrom_SOX2_statusHigh.FAM185A	-0.7835338	4.91499041	-2.7671345	0.00713935	0.02008436	-3.1000397	FAM185A	assignmentsChromaffin_cells.Chrom_SOX2_statusHigh
assignmentsChromaffin_cells.Chrom_SOX2_statusHigh.ACTN4	0.76906795	6.89447761	2.79323535	0.00663909	0.01891592	-3.1024732	ACTN4	assignmentsChromaffin_cells.Chrom_SOX2_statusHigh
assignmentsChromaffin_cells.Chrom_SOX2_statusHigh.DNAJB12	0.44245102	4.96326874	2.76821497	0.00711798	0.02002774	-3.1025527	DNAJB12	assignmentsChromaffin_cells.Chrom_SOX2_statusHigh
assignmentsChromaffin_cells.Chrom_SOX2_statusHigh.ECI1	1.0338635	3.71233106	2.75111291	0.0074633	0.02088944	-3.10312	ECI1	assignmentsChromaffin_cells.Chrom_SOX2_statusHigh
assignmentsChromaffin_cells.Chrom_SOX2_statusHigh.PCYOX1L	0.67030546	2.96306095	2.71777757	0.00818081	0.02258362	-3.1042717	PCYOX1L	assignmentsChromaffin_cells.Chrom_SOX2_statusHigh
assignmentsChromaffin_cells.Chrom_SOX2_statusHigh.LRRC69	0.93963329	3.79767893	2.71530384	0.00823649	0.02271815	-3.1044077	LRRC69	assignmentsChromaffin_cells.Chrom_SOX2_statusHigh
assignmentsChromaffin_cells.Chrom_SOX2_statusHigh.ADAT3	1.82054162	-0.4151625	2.52529632	0.01370114	0.03455557	-3.1061094	ADAT3	assignmentsChromaffin_cells.Chrom_SOX2_statusHigh
assignmentsChromaffin_cells.Chrom_SOX2_statusHigh.FAM24B	0.76390101	2.95152032	2.72815465	0.00795097	0.02204663	-3.1072731	FAM24B	assignmentsChromaffin_cells.Chrom_SOX2_statusHigh
assignmentsChromaffin_cells.Chrom_SOX2_statusHigh.SPDYE16	-1.5927919	0.32886568	-2.5668796	0.01228235	0.03157812	-3.1073534	SPDYE16	assignmentsChromaffin_cells.Chrom_SOX2_statusHigh
assignmentsChromaffin_cells.Chrom_SOX2_statusHigh.TERF1	0.46208367	5.39439633	2.77865425	0.00691445	0.01957495	-3.1082126	TERF1	assignmentsChromaffin_cells.Chrom_SOX2_statusHigh
assignmentsChromaffin_cells.Chrom_SOX2_statusHigh.PTCH2	1.1761389	2.41118118	2.66513317	0.00944301	0.02542764	-3.108293	PTCH2	assignmentsChromaffin_cells.Chrom_SOX2_statusHigh
assignmentsChromaffin_cells.Chrom_SOX2_statusHigh.SPON2	1.82099209	2.06082421	2.59314735	0.01145824	0.02977499	-3.1088727	SPON2	assignmentsChromaffin_cells.Chrom_SOX2_statusHigh
assignmentsChromaffin_cells.Chrom_SOX2_statusHigh.RPRM	2.61528025	-0.0861754	2.55995879	0.01251477	0.03208311	-3.1090882	RPRM	assignmentsChromaffin_cells.Chrom_SOX2_statusHigh
assignmentsChromaffin_cells.Chrom_SOX2_statusHigh.LYG1	-0.9338772	1.49838934	-2.6155416	0.01079158	0.02836371	-3.1112702	LYG1	assignmentsChromaffin_cells.Chrom_SOX2_statusHigh
assignmentsChromaffin_cells.Chrom_SOX2_statusHigh.C1RL-AS1	2.0609847	2.39499053	2.6594881	0.00959353	0.02575942	-3.1130543	C1RL-AS1	assignmentsChromaffin_cells.Chrom_SOX2_statusHigh
assignmentsChromaffin_cells.Chrom_SOX2_statusHigh.NRBP2	-0.7424933	4.02954357	-2.713537	0.00827647	0.02280331	-3.1136552	NRBP2	assignmentsChromaffin_cells.Chrom_SOX2_statusHigh
assignmentsChromaffin_cells.Chrom_SOX2_statusHigh.ZSWIM8	-0.4144596	5.43238299	-2.7742854	0.00699897	0.01975527	-3.1138815	ZSWIM8	assignmentsChromaffin_cells.Chrom_SOX2_statusHigh
assignmentsChromaffin_cells.Chrom_SOX2_statusHigh.GPR146	1.20448043	1.1252146	2.63003921	0.0103803	0.0274673	-3.1156398	GPR146	assignmentsChromaffin_cells.Chrom_SOX2_statusHigh
assignmentsChromaffin_cells.Chrom_SOX2_statusHigh.ZNF664	0.67419261	4.11967566	2.75122958	0.00746089	0.02088633	-3.1158099	ZNF664	assignmentsChromaffin_cells.Chrom_SOX2_statusHigh
assignmentsChromaffin_cells.Chrom_SOX2_statusHigh.STAG1	-0.7149671	8.36229969	-2.8060457	0.00640551	0.01837117	-3.1169469	STAG1	assignmentsChromaffin_cells.Chrom_SOX2_statusHigh
assignmentsChromaffin_cells.Chrom_SOX2_statusHigh.NCL	1.25710672	7.09352772	2.80696304	0.00639318	0.01834566	-3.1173958	NCL	assignmentsChromaffin_cells.Chrom_SOX2_statusHigh
assignmentsChromaffin_cells.Chrom_SOX2_statusHigh.HDAC4	-0.9977044	7.38153784	-2.8007322	0.00650266	0.01858997	-3.1175519	HDAC4	assignmentsChromaffin_cells.Chrom_SOX2_statusHigh
assignmentsChromaffin_cells.Chrom_SOX2_statusHigh.LINC02803	0.95441863	1.5552668	2.6459614	0.00994505	0.02651822	-3.11836	LINC02803	assignmentsChromaffin_cells.Chrom_SOX2_statusHigh
assignmentsChromaffin_cells.Chrom_SOX2_statusHigh.ARHGAP28	-2.0251575	2.62745747	-2.6568064	0.00966332	0.02591644	-3.1194604	ARHGAP28	assignmentsChromaffin_cells.Chrom_SOX2_statusHigh
assignmentsChromaffin_cells.Chrom_SOX2_statusHigh.ACYP2	-0.7057425	7.50654027	-2.8037371	0.00644704	0.01846783	-3.1205475	ACYP2	assignmentsChromaffin_cells.Chrom_SOX2_statusHigh
assignmentsChromaffin_cells.Chrom_SOX2_statusHigh.STOM	1.83998008	4.99631	2.66806496	0.00937338	0.0252656	-3.12094	STOM	assignmentsChromaffin_cells.Chrom_SOX2_statusHigh
assignmentsChromaffin_cells.Chrom_SOX2_statusHigh.ZNF564	-0.6594265	4.01969649	-2.7194893	0.00814249	0.02248437	-3.1210277	ZNF564	assignmentsChromaffin_cells.Chrom_SOX2_statusHigh
assignmentsChromaffin_cells.Chrom_SOX2_statusHigh.METAP1	-0.4618311	4.72531782	-2.7326227	0.00785384	0.02181879	-3.1211777	METAP1	assignmentsChromaffin_cells.Chrom_SOX2_statusHigh
assignmentsChromaffin_cells.Chrom_SOX2_statusHigh.EXTL2	0.75694248	4.05335113	2.7262108	0.00799357	0.02214943	-3.122928	EXTL2	assignmentsChromaffin_cells.Chrom_SOX2_statusHigh
assignmentsChromaffin_cells.Chrom_SOX2_statusHigh.PPP2R5E	-0.85226	7.80887983	-2.8132819	0.00627693	0.01807351	-3.1230126	PPP2R5E	assignmentsChromaffin_cells.Chrom_SOX2_statusHigh
assignmentsChromaffin_cells.Chrom_SOX2_statusHigh.KCNE4	1.34171676	2.37077033	2.64193501	0.01005353	0.02671723	-3.1232949	KCNE4	assignmentsChromaffin_cells.Chrom_SOX2_statusHigh
assignmentsChromaffin_cells.Chrom_SOX2_statusHigh.PEX19	0.57499499	3.654145	2.73955564	0.00770527	0.02145435	-3.1260868	PEX19	assignmentsChromaffin_cells.Chrom_SOX2_statusHigh
assignmentsChromaffin_cells.Chrom_SOX2_statusHigh.CARD8	-0.7830443	5.87107177	-2.7354532	0.00779287	0.02167945	-3.1263302	CARD8	assignmentsChromaffin_cells.Chrom_SOX2_statusHigh
assignmentsChromaffin_cells.Chrom_SOX2_statusHigh.FAM111A	1.1882949	4.64205088	2.69198535	0.0087786	0.02394001	-3.1270166	FAM111A	assignmentsChromaffin_cells.Chrom_SOX2_statusHigh
assignmentsChromaffin_cells.Chrom_SOX2_statusHigh.C1orf21	-1.5222359	6.72783331	-2.7717071	0.00705363	0.01988507	-3.1270881	C1orf21	assignmentsChromaffin_cells.Chrom_SOX2_statusHigh
assignmentsChromaffin_cells.Chrom_SOX2_statusHigh.CCDC181	0.89948918	3.0776066	2.74714938	0.00754549	0.02108766	-3.1279067	CCDC181	assignmentsChromaffin_cells.Chrom_SOX2_statusHigh
assignmentsChromaffin_cells.Chrom_SOX2_statusHigh.GPR173	-0.7621706	5.27818427	-2.7742989	0.00699871	0.01975527	-3.1284908	GPR173	assignmentsChromaffin_cells.Chrom_SOX2_statusHigh
assignmentsChromaffin_cells.Chrom_SOX2_statusHigh.MBD6	-0.7028256	4.46483771	-2.7413592	0.00766704	0.02137389	-3.1293132	MBD6	assignmentsChromaffin_cells.Chrom_SOX2_statusHigh
assignmentsChromaffin_cells.Chrom_SOX2_statusHigh.PAPOLA	-0.4562719	7.44078973	-2.802948	0.00646129	0.01849808	-3.1296231	PAPOLA	assignmentsChromaffin_cells.Chrom_SOX2_statusHigh
assignmentsChromaffin_cells.Chrom_SOX2_statusHigh.WDR37	-0.4824696	6.58373589	-2.7828587	0.00683399	0.01937829	-3.1303237	WDR37	assignmentsChromaffin_cells.Chrom_SOX2_statusHigh
assignmentsChromaffin_cells.Chrom_SOX2_statusHigh.SLC50A1	0.80817252	3.13392195	2.69617549	0.00867885	0.02373648	-3.1306445	SLC50A1	assignmentsChromaffin_cells.Chrom_SOX2_statusHigh
assignmentsChromaffin_cells.Chrom_SOX2_statusHigh.GALNTL6	-2.0929702	1.34741199	-2.5670936	0.01227943	0.03157568	-3.1317478	GALNTL6	assignmentsChromaffin_cells.Chrom_SOX2_statusHigh
assignmentsChromaffin_cells.Chrom_SOX2_statusHigh.SHPK	-0.8057812	3.44260787	-2.6943715	0.00872166	0.02382119	-3.1322226	SHPK	assignmentsChromaffin_cells.Chrom_SOX2_statusHigh
assignmentsChromaffin_cells.Chrom_SOX2_statusHigh.THUMPD1	0.58086411	4.9964536	2.7543471	0.00739684	0.02073236	-3.1324135	THUMPD1	assignmentsChromaffin_cells.Chrom_SOX2_statusHigh
assignmentsChromaffin_cells.Chrom_SOX2_statusHigh.SLC22A10	1.80973456	-0.5386864	2.53227411	0.01345321	0.03408571	-3.1326273	SLC22A10	assignmentsChromaffin_cells.Chrom_SOX2_statusHigh
assignmentsChromaffin_cells.Chrom_SOX2_statusHigh.C12orf75	1.32404995	2.55569992	2.67554603	0.00918156	0.02481947	-3.1331857	C12orf75	assignmentsChromaffin_cells.Chrom_SOX2_statusHigh
assignmentsChromaffin_cells.Chrom_SOX2_statusHigh.RASGEF1B	1.58311858	5.44311778	2.7261069	0.00800051	0.02216483	-3.1334467	RASGEF1B	assignmentsChromaffin_cells.Chrom_SOX2_statusHigh
assignmentsChromaffin_cells.Chrom_SOX2_statusHigh.SNX19	-0.5389703	5.80917703	-2.767037	0.00714129	0.02008627	-3.1336679	SNX19	assignmentsChromaffin_cells.Chrom_SOX2_statusHigh
assignmentsChromaffin_cells.Chrom_SOX2_statusHigh.DISC1FP1	1.72202119	2.26965131	2.66695094	0.00940172	0.02533346	-3.1338242	DISC1FP1	assignmentsChromaffin_cells.Chrom_SOX2_statusHigh
assignmentsChromaffin_cells.Chrom_SOX2_statusHigh.ACKR4	-1.4254083	1.32601483	-2.5980367	0.01130783	0.02946466	-3.1338299	ACKR4	assignmentsChromaffin_cells.Chrom_SOX2_statusHigh
assignmentsChromaffin_cells.Chrom_SOX2_statusHigh.SLC7A5	2.70480891	4.99549304	2.74341899	0.00762813	0.02128289	-3.1338936	SLC7A5	assignmentsChromaffin_cells.Chrom_SOX2_statusHigh
assignmentsChromaffin_cells.Chrom_SOX2_statusHigh.POU5F2	-0.6144149	2.78664232	-2.6826002	0.00900581	0.02443499	-3.1339968	POU5F2	assignmentsChromaffin_cells.Chrom_SOX2_statusHigh
assignmentsChromaffin_cells.Chrom_SOX2_statusHigh.GPR162	-1.1636964	2.95554512	-2.7070037	0.00842642	0.02315677	-3.1352648	GPR162	assignmentsChromaffin_cells.Chrom_SOX2_statusHigh
assignmentsChromaffin_cells.Chrom_SOX2_statusHigh.PURPL	-2.2909332	2.85752775	-2.6992333	0.00861156	0.02358109	-3.1373185	PURPL	assignmentsChromaffin_cells.Chrom_SOX2_statusHigh
assignmentsChromaffin_cells.Chrom_SOX2_statusHigh.SDHAF2	0.53247425	4.94879493	2.75462057	0.00739125	0.0207203	-3.1381596	SDHAF2	assignmentsChromaffin_cells.Chrom_SOX2_statusHigh
assignmentsChromaffin_cells.Chrom_SOX2_statusHigh.ITGA9-AS1	-0.9808264	4.69041488	-2.7314013	0.00788028	0.02188467	-3.1392861	ITGA9-AS1	assignmentsChromaffin_cells.Chrom_SOX2_statusHigh
assignmentsChromaffin_cells.Chrom_SOX2_statusHigh.ACP6	-0.8214374	3.97804544	-2.7322357	0.00786221	0.02183826	-3.1402738	ACP6	assignmentsChromaffin_cells.Chrom_SOX2_statusHigh
assignmentsChromaffin_cells.Chrom_SOX2_statusHigh.RBPJ	-0.8370463	7.68448429	-2.7915031	0.00667127	0.01899074	-3.1410281	RBPJ	assignmentsChromaffin_cells.Chrom_SOX2_statusHigh
assignmentsChromaffin_cells.Chrom_SOX2_statusHigh.FAT2	-2.1021999	0.52339718	-2.5385158	0.0132374	0.03360743	-3.1421976	FAT2	assignmentsChromaffin_cells.Chrom_SOX2_statusHigh
assignmentsChromaffin_cells.Chrom_SOX2_statusHigh.TNFRSF6B	-0.8241282	2.15319707	-2.6581791	0.00962239	0.02583258	-3.1422968	TNFRSF6B	assignmentsChromaffin_cells.Chrom_SOX2_statusHigh
assignmentsChromaffin_cells.Chrom_SOX2_statusHigh.HOXD4	1.59425234	1.41728669	2.65300951	0.00976061	0.02610751	-3.1493621	HOXD4	assignmentsChromaffin_cells.Chrom_SOX2_statusHigh

assignmentsChromaffin_cells.Chrom_SOX2_statusHigh.RASL11A	1.69332287	1.46001576	2.62968238	0.01039322	0.02749242	-3.1495983	RASL11A	assignmentsChromaffin_cells.Chrom_SOX2_statusHigh
assignmentsChromaffin_cells.Chrom_SOX2_statusHigh.MYOCOS	1.33152563	0.64231277	2.59035834	0.01154126	0.0299658	-3.1511346	MYOCOS	assignmentsChromaffin_cells.Chrom_SOX2_statusHigh
assignmentsChromaffin_cells.Chrom_SOX2_statusHigh.PDLIM5	-0.816806	7.83852497	-2.7906427	0.0066873	0.01902288	-3.1517425	PDLIM5	assignmentsChromaffin_cells.Chrom_SOX2_statusHigh
assignmentsChromaffin_cells.Chrom_SOX2_statusHigh.BACH1	-0.8612263	7.2661059	-2.7493681	0.00749938	0.02097576	-3.1524572	BACH1	assignmentsChromaffin_cells.Chrom_SOX2_statusHigh
assignmentsChromaffin_cells.Chrom_SOX2_statusHigh.PSMD9	0.6882736	4.65265587	2.74316962	0.00762884	0.02128289	-3.1524951	PSMD9	assignmentsChromaffin_cells.Chrom_SOX2_statusHigh
assignmentsChromaffin_cells.Chrom_SOX2_statusHigh.MERTK	-1.4072521	5.55729485	-2.6927123	0.00876332	0.02390646	-3.1537003	MERTK	assignmentsChromaffin_cells.Chrom_SOX2_statusHigh
assignmentsChromaffin_cells.Chrom_SOX2_statusHigh.STK11IP	-0.7338706	3.04833077	-2.6803711	0.00906056	0.02456276	-3.1547381	STK11IP	assignmentsChromaffin_cells.Chrom_SOX2_statusHigh
assignmentsChromaffin_cells.Chrom_SOX2_statusHigh.SARDH	1.67469448	0.72033111	2.5384126	0.01323844	0.03360743	-3.1574556	SARDH	assignmentsChromaffin_cells.Chrom_SOX2_statusHigh
assignmentsChromaffin_cells.Chrom_SOX2_statusHigh.LSG1	0.52648396	4.73406212	2.73373439	0.00782984	0.02176343	-3.1575651	LSG1	assignmentsChromaffin_cells.Chrom_SOX2_statusHigh
assignmentsChromaffin_cells.Chrom_SOX2_statusHigh.C12orf40	-1.4662078	2.11055982	-2.6221022	0.01060523	0.02794722	-3.1591012	C12orf40	assignmentsChromaffin_cells.Chrom_SOX2_statusHigh
assignmentsChromaffin_cells.Chrom_SOX2_statusHigh.IL2RB	1.36357024	0.29607003	2.46240926	0.01612831	0.03977638	-3.160351	IL2RB	assignmentsChromaffin_cells.Chrom_SOX2_statusHigh
assignmentsChromaffin_cells.Chrom_SOX2_statusHigh.PMFBP1	0.85548328	1.80517706	2.67200467	0.00926876	0.02502566	-3.1604397	PMFBP1	assignmentsChromaffin_cells.Chrom_SOX2_statusHigh
assignmentsChromaffin_cells.Chrom_SOX2_statusHigh.LINC02100	1.8525387	1.29432439	2.51425186	0.01410201	0.03538853	-3.1610485	LINC02100	assignmentsChromaffin_cells.Chrom_SOX2_statusHigh
assignmentsChromaffin_cells.Chrom_SOX2_statusHigh.LINC02646	-1.1746304	1.76981742	-2.6272842	0.01045734	0.02763013	-3.1613898	LINC02646	assignmentsChromaffin_cells.Chrom_SOX2_statusHigh
assignmentsChromaffin_cells.Chrom_SOX2_statusHigh.SLC8B1	1.20954486	4.04820495	2.65386908	0.00973512	0.02606106	-3.1621835	SLC8B1	assignmentsChromaffin_cells.Chrom_SOX2_statusHigh
assignmentsChromaffin_cells.Chrom_SOX2_statusHigh.ZNF320	-0.9797979	3.52977132	-2.6961146	0.00868029	0.02373648	-3.1625888	ZNF320	assignmentsChromaffin_cells.Chrom_SOX2_statusHigh
assignmentsChromaffin_cells.Chrom_SOX2_statusHigh.FBXL18	-0.9228757	4.14758539	-2.7047078	0.00847892	0.02327456	-3.1667795	FBXL18	assignmentsChromaffin_cells.Chrom_SOX2_statusHigh
assignmentsChromaffin_cells.Chrom_SOX2_statusHigh.ZEB1	-0.6935295	8.70281922	-2.7977692	0.00655554	0.01872779	-3.1686389	ZEB1	assignmentsChromaffin_cells.Chrom_SOX2_statusHigh
assignmentsChromaffin_cells.Chrom_SOX2_statusHigh.MAZ	0.75979632	4.45480094	2.7350042	0.00780251	0.02169875	-3.1689552	MAZ	assignmentsChromaffin_cells.Chrom_SOX2_statusHigh
assignmentsChromaffin_cells.Chrom_SOX2_statusHigh.PNLDC1	1.33675326	0.25310937	2.55328565	0.01273084	0.03252266	-3.1696212	PNLDC1	assignmentsChromaffin_cells.Chrom_SOX2_statusHigh
assignmentsChromaffin_cells.Chrom_SOX2_statusHigh.MAPKAPK3	0.70828754	3.21183545	2.6888694	0.00885345	0.02411951	-3.169913	MAPKAPK3	assignmentsChromaffin_cells.Chrom_SOX2_statusHigh
assignmentsChromaffin_cells.Chrom_SOX2_statusHigh.DCAF15	0.59939517	3.27553893	2.69381215	0.00873498	0.0238462	-3.1703056	DCAF15	assignmentsChromaffin_cells.Chrom_SOX2_statusHigh
assignmentsChromaffin_cells.Chrom_SOX2_statusHigh.TBC1D32	-0.6868782	6.53373788	-2.7779106	0.00692877	0.01960889	-3.1705989	TBC1D32	assignmentsChromaffin_cells.Chrom_SOX2_statusHigh
assignmentsChromaffin_cells.Chrom_SOX2_statusHigh.SYPL2	1.599278	0.29054137	2.47178632	0.0157435	0.03897116	-3.1710874	SYPL2	assignmentsChromaffin_cells.Chrom_SOX2_statusHigh
assignmentsChromaffin_cells.Chrom_SOX2_statusHigh.CCDC113	0.69155859	2.2201017	2.69352546	0.00874181	0.02385996	-3.1720617	CCDC113	assignmentsChromaffin_cells.Chrom_SOX2_statusHigh
assignmentsChromaffin_cells.Chrom_SOX2_statusHigh.PLXND1	1.73229325	5.00856404	2.63204177	0.01032896	0.02736757	-3.1724565	PLXND1	assignmentsChromaffin_cells.Chrom_SOX2_statusHigh
assignmentsChromaffin_cells.Chrom_SOX2_statusHigh.MEG3	-3.4011264	9.75336568	-2.8240722	0.00609358	0.01762786	-3.1727309	MEG3	assignmentsChromaffin_cells.Chrom_SOX2_statusHigh
assignmentsChromaffin_cells.Chrom_SOX2_statusHigh.ANGPTL1	-1.5369099	1.76924829	-2.598802	0.01128642	0.029428	-3.1744285	ANGPTL1	assignmentsChromaffin_cells.Chrom_SOX2_statusHigh
assignmentsChromaffin_cells.Chrom_SOX2_statusHigh.MT-CO1	2.49164911	10.9345884	2.84662135	0.00571842	0.01667433	-3.1773449	MT-CO1	assignmentsChromaffin_cells.Chrom_SOX2_statusHigh
assignmentsChromaffin_cells.Chrom_SOX2_statusHigh.ROR1	2.46647464	4.01417238	2.62552316	0.01051226	0.02775095	-3.1784083	ROR1	assignmentsChromaffin_cells.Chrom_SOX2_statusHigh
assignmentsChromaffin_cells.Chrom_SOX2_statusHigh.SPATS2	-0.5053867	6.96238274	-2.7799435	0.00688969	0.01952585	-3.178689	SPATS2	assignmentsChromaffin_cells.Chrom_SOX2_statusHigh
assignmentsChromaffin_cells.Chrom_SOX2_statusHigh.MED4	0.45238615	6.01112373	2.76303155	0.00722106	0.02029287	-3.1804389	MED4	assignmentsChromaffin_cells.Chrom_SOX2_statusHigh
assignmentsChromaffin_cells.Chrom_SOX2_statusHigh.HSPA4	0.69344895	6.49645173	2.77183698	0.00704675	0.01986916	-3.1817521	HSPA4	assignmentsChromaffin_cells.Chrom_SOX2_statusHigh
assignmentsChromaffin_cells.Chrom_SOX2_statusHigh.C1RL	1.54894326	4.21939856	2.70840872	0.0083983	0.02309136	-3.1819383	C1RL	assignmentsChromaffin_cells.Chrom_SOX2_statusHigh
assignmentsChromaffin_cells.Chrom_SOX2_statusHigh.MPDU1	0.9791671	3.30945784	2.68490928	0.00894942	0.02430665	-3.1824428	MPDU1	assignmentsChromaffin_cells.Chrom_SOX2_statusHigh
assignmentsChromaffin_cells.Chrom_SOX2_statusHigh.AVPR1A	1.69560157	-0.360408	2.43255439	0.01741028	0.04239691	-3.1825513	AVPR1A	assignmentsChromaffin_cells.Chrom_SOX2_statusHigh
assignmentsChromaffin_cells.Chrom_SOX2_statusHigh.RTEL1	-0.66095	4.06694814	-2.7020001	0.00854191	0.02341396	-3.1836682	RTEL1	assignmentsChromaffin_cells.Chrom_SOX2_statusHigh
assignmentsChromaffin_cells.Chrom_SOX2_statusHigh.ZFP36	2.40684892	6.6274899	2.66906374	0.00934805	0.02520579	-3.1847911	ZFP36	assignmentsChromaffin_cells.Chrom_SOX2_statusHigh
assignmentsChromaffin_cells.Chrom_SOX2_statusHigh.RHOT1	-0.4651774	6.58625679	-2.7651595	0.00717858	0.02018409	-3.1854764	RHOT1	assignmentsChromaffin_cells.Chrom_SOX2_statusHigh
assignmentsChromaffin_cells.Chrom_SOX2_statusHigh.KBTBD11-O	1.6958851	0.63305566	2.63280636	0.01030777	0.02732045	-3.18854	KBTBD11-OT	assignmentsChromaffin_cells.Chrom_SOX2_statusHigh
assignmentsChromaffin_cells.Chrom_SOX2_statusHigh.NUP133	-0.46052	5.63437991	-2.7441246	0.00760876	0.02123727	-3.1886386	NUP133	assignmentsChromaffin_cells.Chrom_SOX2_statusHigh
assignmentsChromaffin_cells.Chrom_SOX2_statusHigh.PFKM	0.60771018	4.07791938	2.73468974	0.00780927	0.02171378	-3.1889222	PFKM	assignmentsChromaffin_cells.Chrom_SOX2_statusHigh
assignmentsChromaffin_cells.Chrom_SOX2_statusHigh.ST8SIA5	1.36789689	0.81765913	2.60009929	0.01124587	0.02934089	-3.1892654	ST8SIA5	assignmentsChromaffin_cells.Chrom_SOX2_statusHigh
assignmentsChromaffin_cells.Chrom_SOX2_statusHigh.NOC2L	0.5971865	4.35349073	2.71955547	0.00814101	0.02248437	-3.1898286	NOC2L	assignmentsChromaffin_cells.Chrom_SOX2_statusHigh
assignmentsChromaffin_cells.Chrom_SOX2_statusHigh.CYCS	1.40271359	6.09709235	2.77555646	0.0069786	0.01971859	-3.1903767	CYCS	assignmentsChromaffin_cells.Chrom_SOX2_statusHigh
assignmentsChromaffin_cells.Chrom_SOX2_statusHigh.DLST	-0.4457541	5.3902108	-2.7278027	0.00795867	0.02206415	-3.1908534	DLST	assignmentsChromaffin_cells.Chrom_SOX2_statusHigh
assignmentsChromaffin_cells.Chrom_SOX2_statusHigh.RFC2	0.63744517	3.32906006	2.68376285	0.00897738	0.02436608	-3.1911049	RFC2	assignmentsChromaffin_cells.Chrom_SOX2_statusHigh
assignmentsChromaffin_cells.Chrom_SOX2_statusHigh.CFAP206	0.8045033	1.53226336	2.64720237	0.00991183	0.02644437	-3.1916706	CFAP206	assignmentsChromaffin_cells.Chrom_SOX2_statusHigh
assignmentsChromaffin_cells.Chrom_SOX2_statusHigh.MAP3K20-A	-1.394186	1.23107829	-2.5597015	0.01251736	0.03208461	-3.1921607	MAP3K20-AS	assignmentsChromaffin_cells.Chrom_SOX2_statusHigh
assignmentsChromaffin_cells.Chrom_SOX2_statusHigh.LY6E-DT	-1.9400564	1.40695017	-2.5695386	0.01220068	0.03141347	-3.1924449	LY6E-DT	assignmentsChromaffin_cells.Chrom_SOX2_statusHigh
assignmentsChromaffin_cells.Chrom_SOX2_statusHigh.UBE2V2	0.55442571	5.44733056	2.75130754	0.00745928	0.02088547	-3.1925667	UBE2V2	assignmentsChromaffin_cells.Chrom_SOX2_statusHigh
assignmentsChromaffin_cells.Chrom_SOX2_statusHigh.LINC02088	2.55049878	-1.261919	2.42477469	0.01776084	0.04311312	-3.1942922	LINC02088	assignmentsChromaffin_cells.Chrom_SOX2_statusHigh
assignmentsChromaffin_cells.Chrom_SOX2_statusHigh.UBASH3B	-1.8053349	5.71680018	-2.6551454	0.00970678	0.02601194	-3.1942985	UBASH3B	assignmentsChromaffin_cells.Chrom_SOX2_statusHigh
assignmentsChromaffin_cells.Chrom_SOX2_statusHigh.AGO3	-0.5443132	7.41036349	-2.7692299	0.00709795	0.01999244	-3.1945528	AGO3	assignmentsChromaffin_cells.Chrom_SOX2_statusHigh
assignmentsChromaffin_cells.Chrom_SOX2_statusHigh.STT3B	-0.6002413	6.93847361	-2.7552853	0.00737766	0.02069011	-3.1945634	STT3B	assignmentsChromaffin_cells.Chrom_SOX2_statusHigh
assignmentsChromaffin_cells.Chrom_SOX2_statusHigh.PIM2	0.97580785	2.02296855	2.64276052	0.01003119	0.02667114	-3.1953594	PIM2	assignmentsChromaffin_cells.Chrom_SOX2_statusHigh
assignmentsChromaffin_cells.Chrom_SOX2_statusHigh.KCNQ4	1.34036295	0.24178783	2.49666043	0.01476233	0.03683234	-3.1967376	KCNQ4	assignmentsChromaffin_cells.Chrom_SOX2_statusHigh
assignmentsChromaffin_cells.Chrom_SOX2_statusHigh.G2E3	-0.4980063	5.64472493	-2.7337445	0.00782962	0.02176343	-3.1977588	G2E3	assignmentsChromaffin_cells.Chrom_SOX2_statusHigh
assignmentsChromaffin_cells.Chrom_SOX2_statusHigh.OTUD1	1.31936529	2.39569163	2.63022659	0.01037602	0.02746049	-3.1978504	OTUD1	assignmentsChromaffin_cells.Chrom_SOX2_statusHigh
assignmentsChromaffin_cells.Chrom_SOX2_statusHigh.LRCH2	-0.658819	4.78017574	-2.7401882	0.00769184	0.0214244	-3.1978826	LRCH2	assignmentsChromaffin_cells.Chrom_SOX2_statusHigh
assignmentsChromaffin_cells.Chrom_SOX2_statusHigh.PLSCR2	1.52943173	1.10710442	2.5833426	0.01175988	0.03048407	-3.1990988	PLSCR2	assignmentsChromaffin_cells.Chrom_SOX2_statusHigh
assignmentsChromaffin_cells.Chrom_SOX2_statusHigh.SPIN2B	0.91786952	2.43851899	2.67094757	0.00929538	0.0250709	-3.2018619	SPIN2B	assignmentsChromaffin_cells.Chrom_SOX2_statusHigh
assignmentsChromaffin_cells.Chrom_SOX2_statusHigh.TNNI3	1.82265417	-0.2151792	2.52489459	0.01371733	0.0345696	-3.2026943	TNNI3	assignmentsChromaffin_cells.Chrom_SOX2_statusHigh
assignmentsChromaffin_cells.Chrom_SOX2_statusHigh.CAMTA1	-0.8501968	8.13596642	-2.7896624	0.00670562	0.01906449	-3.2028033	CAMTA1	assignmentsChromaffin_cells.Chrom_SOX2_statusHigh

assignmentsChromaffin_cells.Chrom_SOX2_statusHigh.SYCP2L	-1.4237102	3.40321832	-2.6535009	0.00974697	0.02608843	-3.2044126	SYCP2L	assignmentsChromaffin_cells.Chrom_SOX2_statusHigh
assignmentsChromaffin_cells.Chrom_SOX2_statusHigh.FANCM	-0.7451102	4.31194345	-2.6937995	0.00873528	0.0238462	-3.2064091	FANCM	assignmentsChromaffin_cells.Chrom_SOX2_statusHigh
assignmentsChromaffin_cells.Chrom_SOX2_statusHigh.CYGB	1.7746867	2.98891875	2.68750526	0.00889133	0.02419396	-3.2066605	CYGB	assignmentsChromaffin_cells.Chrom_SOX2_statusHigh
assignmentsChromaffin_cells.Chrom_SOX2_statusHigh.ZNF202	-0.7437558	2.87017619	-2.6544588	0.00971961	0.02603669	-3.2075039	ZNF202	assignmentsChromaffin_cells.Chrom_SOX2_statusHigh
assignmentsChromaffin_cells.Chrom_SOX2_statusHigh.MSC-AS1	-3.0620435	4.85571152	-2.6335975	0.01028689	0.027276	-3.2076823	MSC-AS1	assignmentsChromaffin_cells.Chrom_SOX2_statusHigh
assignmentsChromaffin_cells.Chrom_SOX2_statusHigh.FAH	0.7271338	2.12923217	2.64298839	0.01002504	0.02666342	-3.2079228	FAH	assignmentsChromaffin_cells.Chrom_SOX2_statusHigh
assignmentsChromaffin_cells.Chrom_SOX2_statusHigh.HERC2	-0.8427194	7.26062006	-2.7683988	0.00711435	0.02002276	-3.2085354	HERC2	assignmentsChromaffin_cells.Chrom_SOX2_statusHigh
assignmentsChromaffin_cells.Chrom_SOX2_statusHigh.CHST2	1.6685691	1.53115131	2.55903935	0.01254098	0.03213488	-3.2097958	CHST2	assignmentsChromaffin_cells.Chrom_SOX2_statusHigh
assignmentsChromaffin_cells.Chrom_SOX2_statusHigh.GPSM1	-0.8430441	2.93250388	-2.6569897	0.00965338	0.02590277	-3.2114693	GPSM1	assignmentsChromaffin_cells.Chrom_SOX2_statusHigh
assignmentsChromaffin_cells.Chrom_SOX2_statusHigh.ARSI	1.70270132	-0.8021916	2.41211257	0.01833988	0.04428411	-3.2129474	ARSI	assignmentsChromaffin_cells.Chrom_SOX2_statusHigh
assignmentsChromaffin_cells.Chrom_SOX2_statusHigh.PLCB1	-1.5366156	8.56320632	-2.7790527	0.00691108	0.01957264	-3.2133361	PLCB1	assignmentsChromaffin_cells.Chrom_SOX2_statusHigh
assignmentsChromaffin_cells.Chrom_SOX2_statusHigh.SNRPN	1.01178842	6.03298897	2.77043391	0.00707589	0.0199373	-3.2147368	SNRPN	assignmentsChromaffin_cells.Chrom_SOX2_statusHigh
assignmentsChromaffin_cells.Chrom_SOX2_statusHigh.HOXB5	2.05829011	0.3519905	2.52616407	0.01367377	0.03451207	-3.2159169	HOXB5	assignmentsChromaffin_cells.Chrom_SOX2_statusHigh
assignmentsChromaffin_cells.Chrom_SOX2_statusHigh.RUNX2	-1.5001792	5.06611749	-2.6247737	0.01053027	0.02778161	-3.2175591	RUNX2	assignmentsChromaffin_cells.Chrom_SOX2_statusHigh
assignmentsChromaffin_cells.Chrom_SOX2_statusHigh.DENND2A	-1.4973928	4.8108738	-2.6852862	0.00894519	0.02429929	-3.217902	DENND2A	assignmentsChromaffin_cells.Chrom_SOX2_statusHigh
assignmentsChromaffin_cells.Chrom_SOX2_statusHigh.TBC1D17	0.60255245	4.07846601	2.70898943	0.00838019	0.02304946	-3.2180288	TBC1D17	assignmentsChromaffin_cells.Chrom_SOX2_statusHigh
assignmentsChromaffin_cells.Chrom_SOX2_statusHigh.USP2	1.93601592	0.28758912	2.4697683	0.01582562	0.0391382	-3.2182374	USP2	assignmentsChromaffin_cells.Chrom_SOX2_statusHigh
assignmentsChromaffin_cells.Chrom_SOX2_statusHigh.HSD17B14	-0.6944622	4.37613013	-2.7112493	0.0083285	0.02292302	-3.2201633	HSD17B14	assignmentsChromaffin_cells.Chrom_SOX2_statusHigh
assignmentsChromaffin_cells.Chrom_SOX2_statusHigh.PPP1R9A	-1.133654	7.05585235	-2.7788717	0.00691375	0.01957495	-3.2202224	PPP1R9A	assignmentsChromaffin_cells.Chrom_SOX2_statusHigh
assignmentsChromaffin_cells.Chrom_SOX2_statusHigh.LANCL2	-0.5699711	4.77726347	-2.7215685	0.00809615	0.02239832	-3.2205207	LANCL2	assignmentsChromaffin_cells.Chrom_SOX2_statusHigh
assignmentsChromaffin_cells.Chrom_SOX2_statusHigh.DRAIC	-1.1596282	1.87646372	-2.6101311	0.01094881	0.02869702	-3.2216319	DRAIC	assignmentsChromaffin_cells.Chrom_SOX2_statusHigh
assignmentsChromaffin_cells.Chrom_SOX2_statusHigh.MX2	1.9012017	3.91911599	2.54062862	0.01316423	0.03345926	-3.2226458	MX2	assignmentsChromaffin_cells.Chrom_SOX2_statusHigh
assignmentsChromaffin_cells.Chrom_SOX2_statusHigh.GPR158	-1.655594	6.47506033	-2.7693851	0.00709925	0.01999259	-3.2230322	GPR158	assignmentsChromaffin_cells.Chrom_SOX2_statusHigh
assignmentsChromaffin_cells.Chrom_SOX2_statusHigh.LYL1	1.47450151	1.25418767	2.46561915	0.01599564	0.03951604	-3.2233449	LYL1	assignmentsChromaffin_cells.Chrom_SOX2_statusHigh
assignmentsChromaffin_cells.Chrom_SOX2_statusHigh.PILRB	-0.6387549	6.00916114	-2.7391505	0.00771388	0.0214746	-3.2241736	PILRB	assignmentsChromaffin_cells.Chrom_SOX2_statusHigh
assignmentsChromaffin_cells.Chrom_SOX2_statusHigh.GDE1	0.72323412	3.66478815	2.68756278	0.00888501	0.02418495	-3.2273575	GDE1	assignmentsChromaffin_cells.Chrom_SOX2_statusHigh
assignmentsChromaffin_cells.Chrom_SOX2_statusHigh.DDX51	-0.5369523	3.38567359	-2.6709046	0.00929646	0.0250709	-3.2306616	DDX51	assignmentsChromaffin_cells.Chrom_SOX2_statusHigh
assignmentsChromaffin_cells.Chrom_SOX2_statusHigh.C11orf65	-1.0749686	3.24478976	-2.6532307	0.00975191	0.02609294	-3.2306655	C11orf65	assignmentsChromaffin_cells.Chrom_SOX2_statusHigh
assignmentsChromaffin_cells.Chrom_SOX2_statusHigh.PRPF19	0.54319908	4.02701381	2.69900187	0.00861215	0.02358109	-3.2310767	PRPF19	assignmentsChromaffin_cells.Chrom_SOX2_statusHigh
assignmentsChromaffin_cells.Chrom_SOX2_statusHigh.ZNF451-AS:	-1.3078286	3.53949515	-2.6643228	0.0094659	0.02546789	-3.2313115	ZNF451-AS1	assignmentsChromaffin_cells.Chrom_SOX2_statusHigh
assignmentsChromaffin_cells.Chrom_SOX2_statusHigh.MIER2	-0.5256742	3.88334615	-2.672133	0.00926554	0.02502117	-3.232054	MIER2	assignmentsChromaffin_cells.Chrom_SOX2_statusHigh
assignmentsChromaffin_cells.Chrom_SOX2_statusHigh.SST	2.92408852	-0.5008153	2.48663244	0.01515757	0.03768947	-3.2321063	SST	assignmentsChromaffin_cells.Chrom_SOX2_statusHigh
assignmentsChromaffin_cells.Chrom_SOX2_statusHigh.MAP4K5	-0.6303467	7.5399684	-2.7596935	0.00728817	0.02046353	-3.2343791	MAP4K5	assignmentsChromaffin_cells.Chrom_SOX2_statusHigh
assignmentsChromaffin_cells.Chrom_SOX2_statusHigh.RHD	-2.2469884	0.4565937	-2.4429164	0.01695733	0.04152721	-3.2359268	RHD	assignmentsChromaffin_cells.Chrom_SOX2_statusHigh
assignmentsChromaffin_cells.Chrom_SOX2_statusHigh.ZNF79	-0.7694092	2.69720849	-2.6323195	0.01031692	0.02734019	-3.2370371	ZNF79	assignmentsChromaffin_cells.Chrom_SOX2_statusHigh
assignmentsChromaffin_cells.Chrom_SOX2_statusHigh.WDR24	0.79946008	1.7973505	2.62945027	0.01039673	0.02749319	-3.2382886	WDR24	assignmentsChromaffin_cells.Chrom_SOX2_statusHigh
assignmentsChromaffin_cells.Chrom_SOX2_statusHigh.MAMDC2-A	-1.0655524	3.61402479	-2.6604033	0.00956468	0.02568626	-3.2394905	MAMDC2-AS	assignmentsChromaffin_cells.Chrom_SOX2_statusHigh
assignmentsChromaffin_cells.Chrom_SOX2_statusHigh.PMEL	1.21798824	0.69906919	2.53675081	0.01329627	0.03373071	-3.2399283	PMEL	assignmentsChromaffin_cells.Chrom_SOX2_statusHigh
assignmentsChromaffin_cells.Chrom_SOX2_statusHigh.NPHS1	1.80857816	0.88977223	2.59625332	0.01136729	0.02957656	-3.2404239	NPHS1	assignmentsChromaffin_cells.Chrom_SOX2_statusHigh
assignmentsChromaffin_cells.Chrom_SOX2_statusHigh.ST3GAL6	-0.9206272	6.94058094	-2.7536054	0.00741256	0.02076916	-3.2405458	ST3GAL6	assignmentsChromaffin_cells.Chrom_SOX2_statusHigh
assignmentsChromaffin_cells.Chrom_SOX2_statusHigh.CEP295	-0.7259901	5.58590562	-2.7202921	0.00812457	0.02245422	-3.2420574	CEP295	assignmentsChromaffin_cells.Chrom_SOX2_statusHigh
assignmentsChromaffin_cells.Chrom_SOX2_statusHigh.C17orf107	1.71247892	0.29213314	2.44220885	0.01698608	0.04156587	-3.2422684	C17orf107	assignmentsChromaffin_cells.Chrom_SOX2_statusHigh
assignmentsChromaffin_cells.Chrom_SOX2_statusHigh.APOL2	0.76007347	4.14742072	2.68029519	0.00906243	0.02456368	-3.2501201	APOL2	assignmentsChromaffin_cells.Chrom_SOX2_statusHigh
assignmentsChromaffin_cells.Chrom_SOX2_statusHigh.STX17	-0.620268	6.12159921	-2.7257162	0.00800444	0.02216806	-3.2511999	STX17	assignmentsChromaffin_cells.Chrom_SOX2_statusHigh
assignmentsChromaffin_cells.Chrom_SOX2_statusHigh.PFKFB4	-0.8035351	3.25537409	-2.6545067	0.00971836	0.02603669	-3.2512224	PFKFB4	assignmentsChromaffin_cells.Chrom_SOX2_statusHigh
assignmentsChromaffin_cells.Chrom_SOX2_statusHigh.HS2ST1	-0.6550987	6.75744634	-2.7290009	0.00793249	0.02200679	-3.2516138	HS2ST1	assignmentsChromaffin_cells.Chrom_SOX2_statusHigh
assignmentsChromaffin_cells.Chrom_SOX2_statusHigh.STMN3	0.74362023	4.1343521	2.71307772	0.00828689	0.02282417	-3.2525769	STMN3	assignmentsChromaffin_cells.Chrom_SOX2_statusHigh
assignmentsChromaffin_cells.Chrom_SOX2_statusHigh.GPR84	1.90072382	-0.8112432	2.38291936	0.01974432	0.0470939	-3.2525904	GPR84	assignmentsChromaffin_cells.Chrom_SOX2_statusHigh
assignmentsChromaffin_cells.Chrom_SOX2_statusHigh.INTS6L	1.85448626	4.98318791	2.67144816	0.00928782	0.02506445	-3.2527743	INTS6L	assignmentsChromaffin_cells.Chrom_SOX2_statusHigh
assignmentsChromaffin_cells.Chrom_SOX2_statusHigh.IGFLR1	1.18707786	1.45946709	2.54489771	0.01301491	0.03313289	-3.2529159	IGFLR1	assignmentsChromaffin_cells.Chrom_SOX2_statusHigh
assignmentsChromaffin_cells.Chrom_SOX2_statusHigh.MT-ND1	2.39125281	7.90066306	2.77360619	0.00701652	0.01979437	-3.2533098	MT-ND1	assignmentsChromaffin_cells.Chrom_SOX2_statusHigh
assignmentsChromaffin_cells.Chrom_SOX2_statusHigh.PKM	0.8051135	6.94342222	2.76143862	0.00725302	0.02037909	-3.2536635	PKM	assignmentsChromaffin_cells.Chrom_SOX2_statusHigh
assignmentsChromaffin_cells.Chrom_SOX2_statusHigh.SNIP1	0.49797595	4.09763843	2.67494263	0.00919516	0.02484388	-3.254425	SNIP1	assignmentsChromaffin_cells.Chrom_SOX2_statusHigh
assignmentsChromaffin_cells.Chrom_SOX2_statusHigh.SEC23B	-0.5393329	5.60376833	-2.7176877	0.00818283	0.0225841	-3.2549917	SEC23B	assignmentsChromaffin_cells.Chrom_SOX2_statusHigh
assignmentsChromaffin_cells.Chrom_SOX2_statusHigh.LYPLAL1-DT	-1.0029115	2.54778742	-2.6430066	0.01002455	0.02666342	-3.2564693	LYPLAL1-DT	assignmentsChromaffin_cells.Chrom_SOX2_statusHigh
assignmentsChromaffin_cells.Chrom_SOX2_statusHigh.ANKDD1B	0.99742506	2.6594951	2.64079661	0.01008438	0.02678591	-3.257285	ANKDD1B	assignmentsChromaffin_cells.Chrom_SOX2_statusHigh
assignmentsChromaffin_cells.Chrom_SOX2_statusHigh.EIF4A3	1.35530036	5.04154863	2.71040714	0.0083525	0.02298513	-3.2574359	EIF4A3	assignmentsChromaffin_cells.Chrom_SOX2_statusHigh
assignmentsChromaffin_cells.Chrom_SOX2_statusHigh.CDAN1	-0.6580642	2.73315909	-2.6333789	0.0102876	0.027276	-3.257634	CDAN1	assignmentsChromaffin_cells.Chrom_SOX2_statusHigh
assignmentsChromaffin_cells.Chrom_SOX2_statusHigh.RAB11FIP1	0.91743618	4.43956174	2.67838368	0.00910963	0.0246666	-3.2593058	RAB11FIP1	assignmentsChromaffin_cells.Chrom_SOX2_statusHigh
assignmentsChromaffin_cells.Chrom_SOX2_statusHigh.SGPL1	-0.8870329	6.35091054	-2.7350759	0.00780098	0.02169824	-3.2604961	SGPL1	assignmentsChromaffin_cells.Chrom_SOX2_statusHigh
assignmentsChromaffin_cells.Chrom_SOX2_statusHigh.PCDHAC2	1.37152561	0.52537917	2.59428422	0.01142287	0.02969687	-3.2613255	PCDHAC2	assignmentsChromaffin_cells.Chrom_SOX2_statusHigh
assignmentsChromaffin_cells.Chrom_SOX2_statusHigh.PKHD1L1	2.57907404	0.80333315	2.55786818	0.01258395	0.03221273	-3.263644	PKHD1L1	assignmentsChromaffin_cells.Chrom_SOX2_statusHigh
assignmentsChromaffin_cells.Chrom_SOX2_statusHigh.DENND5B	-0.6900665	7.11250735	-2.7532803	0.0074187	0.02078273	-3.263652	DENND5B	assignmentsChromaffin_cells.Chrom_SOX2_statusHigh
assignmentsChromaffin_cells.Chrom_SOX2_statusHigh.RSPO1	2.85334033	-0.1780199	2.48042359	0.0154029	0.03822836	-3.2645357	RSPO1	assignmentsChromaffin_cells.Chrom_SOX2_statusHigh

assignmentsChromaffin_cells.Chrom_SOX2_statusHigh.MTFR1L	0.64788614	3.71378086	2.67723881	0.00913801	0.02471839	-3.2649717	MTFR1L	assignmentsChromaffin_cells.Chrom_SOX2_statusHigh
assignmentsChromaffin_cells.Chrom_SOX2_statusHigh.DFFB	-0.9230176	2.91426517	-2.6080866	0.01100877	0.02883532	-3.2674615	DFFB	assignmentsChromaffin_cells.Chrom_SOX2_statusHigh
assignmentsChromaffin_cells.Chrom_SOX2_statusHigh.GLUL	1.40304911	5.59625157	2.67996801	0.00907538	0.02459461	-3.2677324	GLUL	assignmentsChromaffin_cells.Chrom_SOX2_statusHigh
assignmentsChromaffin_cells.Chrom_SOX2_statusHigh.PSKH1	-0.7114339	3.58893223	-2.6560423	0.00967812	0.02594746	-3.2678747	PSKH1	assignmentsChromaffin_cells.Chrom_SOX2_statusHigh
assignmentsChromaffin_cells.Chrom_SOX2_statusHigh.FOXO4	0.7889641	1.11862331	2.54974363	0.01285011	0.03277492	-3.2704283	FOXO4	assignmentsChromaffin_cells.Chrom_SOX2_statusHigh
assignmentsChromaffin_cells.Chrom_SOX2_statusHigh.KCTD20	-0.6744792	6.31608426	-2.711334	0.00832657	0.02292302	-3.2713292	KCTD20	assignmentsChromaffin_cells.Chrom_SOX2_statusHigh
assignmentsChromaffin_cells.Chrom_SOX2_statusHigh.ATP6V1E2	0.90158483	3.10998925	2.66464793	0.00945543	0.02545253	-3.2731401	ATP6V1E2	assignmentsChromaffin_cells.Chrom_SOX2_statusHigh
assignmentsChromaffin_cells.Chrom_SOX2_statusHigh.ANKRD44-IT	-1.0295779	0.55247672	-2.4874795	0.01511789	0.03760246	-3.2738778	ANKRD44-IT:	assignmentsChromaffin_cells.Chrom_SOX2_statusHigh
assignmentsChromaffin_cells.Chrom_SOX2_statusHigh.CDS1	-0.9395236	3.47009672	-2.6718857	0.00927176	0.02502953	-3.2758021	CDS1	assignmentsChromaffin_cells.Chrom_SOX2_statusHigh
assignmentsChromaffin_cells.Chrom_SOX2_statusHigh.DOCK9	-0.9351408	6.84173311	-2.7139232	0.00826772	0.02278703	-3.2784791	DOCK9	assignmentsChromaffin_cells.Chrom_SOX2_statusHigh
assignmentsChromaffin_cells.Chrom_SOX2_statusHigh.CRACR2A	-1.0824581	3.25772412	-2.5607279	0.01248351	0.03203368	-3.2796307	CRACR2A	assignmentsChromaffin_cells.Chrom_SOX2_statusHigh
assignmentsChromaffin_cells.Chrom_SOX2_statusHigh.TADA2A	-0.559947	4.9189371	-2.6835441	0.00898272	0.02437646	-3.2800702	TADA2A	assignmentsChromaffin_cells.Chrom_SOX2_statusHigh
assignmentsChromaffin_cells.Chrom_SOX2_statusHigh.PHLDA1	1.43423344	4.7104989	2.65007206	0.00983961	0.02628814	-3.2802758	PHLDA1	assignmentsChromaffin_cells.Chrom_SOX2_statusHigh
assignmentsChromaffin_cells.Chrom_SOX2_statusHigh.SBF2-AS1	-0.8161624	3.47419011	-2.6472734	0.00990994	0.02644437	-3.280964	SBF2-AS1	assignmentsChromaffin_cells.Chrom_SOX2_statusHigh
assignmentsChromaffin_cells.Chrom_SOX2_statusHigh.NES	1.58790151	2.22719334	2.48392613	0.01525755	0.03792045	-3.2813979	NES	assignmentsChromaffin_cells.Chrom_SOX2_statusHigh
assignmentsChromaffin_cells.Chrom_SOX2_statusHigh.HMGXB4	-0.465092	5.4558824	-2.7038175	0.00849958	0.02332187	-3.2823002	HMGXB4	assignmentsChromaffin_cells.Chrom_SOX2_statusHigh
assignmentsChromaffin_cells.Chrom_SOX2_statusHigh.EHD1	1.22177067	3.24984134	2.59338262	0.01144881	0.02975949	-3.2826506	EHD1	assignmentsChromaffin_cells.Chrom_SOX2_statusHigh
assignmentsChromaffin_cells.Chrom_SOX2_statusHigh.GIPC2	1.65920466	2.65733105	2.59321509	0.01145849	0.02977499	-3.2830085	GIPC2	assignmentsChromaffin_cells.Chrom_SOX2_statusHigh
assignmentsChromaffin_cells.Chrom_SOX2_statusHigh.DDI2	-0.7990262	4.96209658	-2.6506936	0.00981893	0.02624162	-3.2844619	DDI2	assignmentsChromaffin_cells.Chrom_SOX2_statusHigh
assignmentsChromaffin_cells.Chrom_SOX2_statusHigh.FAM66B	-1.1800467	3.17099392	-2.6457921	0.00995101	0.0265283	-3.2856402	FAM66B	assignmentsChromaffin_cells.Chrom_SOX2_statusHigh
assignmentsChromaffin_cells.Chrom_SOX2_statusHigh.FMR1	-0.3814815	5.35036415	-2.6877434	0.00888064	0.02417716	-3.2861784	FMR1	assignmentsChromaffin_cells.Chrom_SOX2_statusHigh
assignmentsChromaffin_cells.Chrom_SOX2_statusHigh.LINC01811	-1.6041882	1.79968416	-2.6072097	0.01103907	0.0288958	-3.2866356	LINC01811	assignmentsChromaffin_cells.Chrom_SOX2_statusHigh
assignmentsChromaffin_cells.Chrom_SOX2_statusHigh.MDFI	1.72872398	0.00034239	2.38553106	0.01961488	0.04684787	-3.2881463	MDFI	assignmentsChromaffin_cells.Chrom_SOX2_statusHigh
assignmentsChromaffin_cells.Chrom_SOX2_statusHigh.ZSWIM6	-0.9053644	8.93825705	-2.741871	0.00765702	0.02135338	-3.2890859	ZSWIM6	assignmentsChromaffin_cells.Chrom_SOX2_statusHigh
assignmentsChromaffin_cells.Chrom_SOX2_statusHigh.RCAN3AS	-1.1528884	0.99979225	-2.497821	0.01471793	0.03675013	-3.2901884	RCAN3AS	assignmentsChromaffin_cells.Chrom_SOX2_statusHigh
assignmentsChromaffin_cells.Chrom_SOX2_statusHigh.CCAR2	-0.4382385	4.30339168	-2.6615677	0.0095346	0.02562263	-3.29032	CCAR2	assignmentsChromaffin_cells.Chrom_SOX2_statusHigh
assignmentsChromaffin_cells.Chrom_SOX2_statusHigh.ASCL2	2.11127391	-1.005775	2.36748168	0.02052508	0.04868105	-3.2918017	ASCL2	assignmentsChromaffin_cells.Chrom_SOX2_statusHigh
assignmentsChromaffin_cells.Chrom_SOX2_statusHigh.TSTD3	-0.9105109	5.29597786	-2.6989584	0.00861317	0.02358109	-3.2921542	TSTD3	assignmentsChromaffin_cells.Chrom_SOX2_statusHigh
assignmentsChromaffin_cells.Chrom_SOX2_statusHigh.TXNDC9	0.55943057	4.32383939	2.67809514	0.00911677	0.02468177	-3.2930707	TXNDC9	assignmentsChromaffin_cells.Chrom_SOX2_statusHigh
assignmentsChromaffin_cells.Chrom_SOX2_statusHigh.PHF12	-0.542221	5.53364368	-2.6920214	0.00877773	0.02394001	-3.2932437	PHF12	assignmentsChromaffin_cells.Chrom_SOX2_statusHigh
assignmentsChromaffin_cells.Chrom_SOX2_statusHigh.KLHL28	-0.5129263	5.79361635	-2.7059647	0.00844983	0.02320519	-3.2936216	KLHL28	assignmentsChromaffin_cells.Chrom_SOX2_statusHigh
assignmentsChromaffin_cells.Chrom_SOX2_statusHigh.GTPBP3	0.72989945	2.91594226	2.63292091	0.01030027	0.02730507	-3.2954781	GTPBP3	assignmentsChromaffin_cells.Chrom_SOX2_statusHigh
assignmentsChromaffin_cells.Chrom_SOX2_statusHigh.ZMYM6	-0.5563447	5.69145586	-2.687894	0.008877	0.02417546	-3.2965577	ZMYM6	assignmentsChromaffin_cells.Chrom_SOX2_statusHigh
assignmentsChromaffin_cells.Chrom_SOX2_statusHigh.ANP32B	0.76671366	5.92791607	2.7140892	0.00826396	0.02278056	-3.2977811	ANP32B	assignmentsChromaffin_cells.Chrom_SOX2_statusHigh
assignmentsChromaffin_cells.Chrom_SOX2_statusHigh.PPARA	-0.6074297	5.07594812	-2.6777994	0.0091241	0.02469744	-3.2994252	PPARA	assignmentsChromaffin_cells.Chrom_SOX2_statusHigh
assignmentsChromaffin_cells.Chrom_SOX2_statusHigh.SIX5	1.09954166	0.75359013	2.4942713	0.01485413	0.03702681	-3.3005312	SIX5	assignmentsChromaffin_cells.Chrom_SOX2_statusHigh
assignmentsChromaffin_cells.Chrom_SOX2_statusHigh.GABPB2	-0.8350526	4.95306768	-2.6649714	0.00944715	0.02543451	-3.3024603	GABPB2	assignmentsChromaffin_cells.Chrom_SOX2_statusHigh
assignmentsChromaffin_cells.Chrom_SOX2_statusHigh.B3GALNT2	-0.723104	6.28409161	-2.7148955	0.00824571	0.0227381	-3.305154	B3GALNT2	assignmentsChromaffin_cells.Chrom_SOX2_statusHigh
assignmentsChromaffin_cells.Chrom_SOX2_statusHigh.ACTR1A	0.48826045	4.94054239	2.68116588	0.00904101	0.02451803	-3.305288	ACTR1A	assignmentsChromaffin_cells.Chrom_SOX2_statusHigh
assignmentsChromaffin_cells.Chrom_SOX2_statusHigh.ARHGEF7	-0.9328545	7.95160732	-2.7221237	0.0080847	0.02237102	-3.3059267	ARHGEF7	assignmentsChromaffin_cells.Chrom_SOX2_statusHigh
assignmentsChromaffin_cells.Chrom_SOX2_statusHigh.NME6	0.56586223	3.29382617	2.64733547	0.00990828	0.02644437	-3.3067418	NME6	assignmentsChromaffin_cells.Chrom_SOX2_statusHigh
assignmentsChromaffin_cells.Chrom_SOX2_statusHigh.LINC00461	1.91078186	1.68931305	2.62737803	0.01046009	0.02763033	-3.3067838	LINC00461	assignmentsChromaffin_cells.Chrom_SOX2_statusHigh
assignmentsChromaffin_cells.Chrom_SOX2_statusHigh.MAML1	-0.611056	4.9483463	-2.6611306	0.00954588	0.02564006	-3.3071333	MAML1	assignmentsChromaffin_cells.Chrom_SOX2_statusHigh
assignmentsChromaffin_cells.Chrom_SOX2_statusHigh.DDTL	0.95464021	0.71007703	2.52598051	0.01367665	0.03451207	-3.3073368	DDTL	assignmentsChromaffin_cells.Chrom_SOX2_statusHigh
assignmentsChromaffin_cells.Chrom_SOX2_statusHigh.CCDC28B	0.8170088	2.11290078	2.60369139	0.01113867	0.02910426	-3.3082701	CCDC28B	assignmentsChromaffin_cells.Chrom_SOX2_statusHigh
assignmentsChromaffin_cells.Chrom_SOX2_statusHigh.EMD	1.33887089	5.13590422	2.70272881	0.00852974	0.02338461	-3.3083113	EMD	assignmentsChromaffin_cells.Chrom_SOX2_statusHigh
assignmentsChromaffin_cells.Chrom_SOX2_statusHigh.DSCAM	-1.3783906	1.09215984	-2.4472722	0.01676732	0.04112467	-3.3092126	DSCAM	assignmentsChromaffin_cells.Chrom_SOX2_statusHigh
assignmentsChromaffin_cells.Chrom_SOX2_statusHigh.PPM1L	-0.9683768	7.45008678	-2.7294575	0.00792388	0.0219905	-3.3099903	PPM1L	assignmentsChromaffin_cells.Chrom_SOX2_statusHigh
assignmentsChromaffin_cells.Chrom_SOX2_statusHigh.EAPP	0.53367714	5.35276639	2.69730393	0.00865216	0.02367557	-3.3103212	EAPP	assignmentsChromaffin_cells.Chrom_SOX2_statusHigh
assignmentsChromaffin_cells.Chrom_SOX2_statusHigh.TTLL3	-0.5767899	5.18383502	-2.6716454	0.0092778	0.02504164	-3.3106223	TTLL3	assignmentsChromaffin_cells.Chrom_SOX2_statusHigh
assignmentsChromaffin_cells.Chrom_SOX2_statusHigh.MAML3	-0.8132434	8.71931502	-2.7466966	0.00755493	0.02110169	-3.3109656	MAML3	assignmentsChromaffin_cells.Chrom_SOX2_statusHigh
assignmentsChromaffin_cells.Chrom_SOX2_statusHigh.SLC12A7	1.93902096	3.53897112	2.57384777	0.01206383	0.0311311	-3.312827	SLC12A7	assignmentsChromaffin_cells.Chrom_SOX2_statusHigh
assignmentsChromaffin_cells.Chrom_SOX2_statusHigh.HMCES	0.72348839	4.14483709	2.65754653	0.00963886	0.02587246	-3.3133712	HMCES	assignmentsChromaffin_cells.Chrom_SOX2_statusHigh
assignmentsChromaffin_cells.Chrom_SOX2_statusHigh.ZNF248	-0.6645047	6.34118692	-2.7205522	0.00811877	0.02244206	-3.3140341	ZNF248	assignmentsChromaffin_cells.Chrom_SOX2_statusHigh
assignmentsChromaffin_cells.Chrom_SOX2_statusHigh.LGALS8	0.74306447	4.73450888	2.65330342	0.00974999	0.02609217	-3.3147123	LGALS8	assignmentsChromaffin_cells.Chrom_SOX2_statusHigh
assignmentsChromaffin_cells.Chrom_SOX2_statusHigh.KCMF1	-0.6278473	6.61226214	-2.7071536	0.00842239	0.02315364	-3.3169236	KCMF1	assignmentsChromaffin_cells.Chrom_SOX2_statusHigh
assignmentsChromaffin_cells.Chrom_SOX2_statusHigh.KCNH7	-2.4353224	5.5793815	-2.7043382	0.00849231	0.02330589	-3.3173274	KCNH7	assignmentsChromaffin_cells.Chrom_SOX2_statusHigh
assignmentsChromaffin_cells.Chrom_SOX2_statusHigh.IL13RA2	2.60983149	1.31247723	2.59956799	0.01126739	0.02938793	-3.318482	IL13RA2	assignmentsChromaffin_cells.Chrom_SOX2_statusHigh
assignmentsChromaffin_cells.Chrom_SOX2_statusHigh.FOXK2	-0.6820673	6.80336795	-2.7061303	0.008446	0.02319866	-3.318663	FOXK2	assignmentsChromaffin_cells.Chrom_SOX2_statusHigh
assignmentsChromaffin_cells.Chrom_SOX2_statusHigh.CDH6	-2.0018492	2.94626142	-2.4578439	0.01632151	0.04019997	-3.3199239	CDH6	assignmentsChromaffin_cells.Chrom_SOX2_statusHigh
assignmentsChromaffin_cells.Chrom_SOX2_statusHigh.NUDT17	1.00744756	1.41371837	2.57986869	0.01186721	0.03070274	-3.3208927	NUDT17	assignmentsChromaffin_cells.Chrom_SOX2_statusHigh
assignmentsChromaffin_cells.Chrom_SOX2_statusHigh.SH3GLB2	0.60847349	4.08737445	2.67103381	0.00929321	0.02507055	-3.3216078	SH3GLB2	assignmentsChromaffin_cells.Chrom_SOX2_statusHigh
assignmentsChromaffin_cells.Chrom_SOX2_statusHigh.TXNDC11	-0.5972463	5.92706962	-2.6878251	0.00887866	0.02417589	-3.3218803	TXNDC11	assignmentsChromaffin_cells.Chrom_SOX2_statusHigh
assignmentsChromaffin_cells.Chrom_SOX2_statusHigh.LRIG3	-2.3420264	2.19950165	-2.4511628	0.01660765	0.0408017	-3.3240507	LRIG3	assignmentsChromaffin_cells.Chrom_SOX2_statusHigh

assignmentsChromaffin_cells.Chrom_SOX2_statusHigh.ANKRD20A1	-1.5754599	2.05283693	-2.5805725	0.01184903	0.03066357	-3.3240853	ANKRD20A1	assignmentsChromaffin_cells.Chrom_SOX2_statusHigh
assignmentsChromaffin_cells.Chrom_SOX2_statusHigh.RSAD2	1.46236594	3.44268867	2.61849816	0.01071083	0.02816705	-3.3244351	RSAD2	assignmentsChromaffin_cells.Chrom_SOX2_statusHigh
assignmentsChromaffin_cells.Chrom_SOX2_statusHigh.BCL11A	-1.4425272	3.80221489	-2.6510565	0.00981452	0.02623858	-3.3245513	BCL11A	assignmentsChromaffin_cells.Chrom_SOX2_statusHigh
assignmentsChromaffin_cells.Chrom_SOX2_statusHigh.METTL7A	0.9646566	5.12558706	2.70280035	0.00852444	0.02337635	-3.3251852	METTL7A	assignmentsChromaffin_cells.Chrom_SOX2_statusHigh
assignmentsChromaffin_cells.Chrom_SOX2_statusHigh.METTL25	-0.7163633	5.41280145	-2.6758995	0.0091713	0.02479592	-3.3251941	METTL25	assignmentsChromaffin_cells.Chrom_SOX2_statusHigh
assignmentsChromaffin_cells.Chrom_SOX2_statusHigh.SMARCC2	0.5802209	5.47376688	2.69179396	0.00878317	0.02394841	-3.3260376	SMARCC2	assignmentsChromaffin_cells.Chrom_SOX2_statusHigh
assignmentsChromaffin_cells.Chrom_SOX2_statusHigh.PFDN1	0.41729077	5.60070165	2.6880376	0.00887353	0.02417011	-3.3263885	PFDN1	assignmentsChromaffin_cells.Chrom_SOX2_statusHigh
assignmentsChromaffin_cells.Chrom_SOX2_statusHigh.ZFPM2	-1.833488	6.48542611	-2.6856032	0.00893748	0.02429892	-3.3271158	ZFPM2	assignmentsChromaffin_cells.Chrom_SOX2_statusHigh
assignmentsChromaffin_cells.Chrom_SOX2_statusHigh.PLA2G15	0.73561153	3.1730547	2.62439498	0.0105387	0.02779511	-3.3284505	PLA2G15	assignmentsChromaffin_cells.Chrom_SOX2_statusHigh
assignmentsChromaffin_cells.Chrom_SOX2_statusHigh.SMNDC1	0.56479287	5.302896	2.68433337	0.00896345	0.02433652	-3.3288944	SMNDC1	assignmentsChromaffin_cells.Chrom_SOX2_statusHigh
assignmentsChromaffin_cells.Chrom_SOX2_statusHigh.GRB2	0.52034811	6.93182748	2.70468535	0.00847944	0.02327456	-3.3295209	GRB2	assignmentsChromaffin_cells.Chrom_SOX2_statusHigh
assignmentsChromaffin_cells.Chrom_SOX2_statusHigh.PLPP4	1.53172931	-0.4635704	2.40272079	0.01878165	0.04515367	-3.3301005	PLPP4	assignmentsChromaffin_cells.Chrom_SOX2_statusHigh
assignmentsChromaffin_cells.Chrom_SOX2_statusHigh.CRADD	-0.6165103	7.4056319	-2.721303	0.00810206	0.02240744	-3.3302767	CRADD	assignmentsChromaffin_cells.Chrom_SOX2_statusHigh
assignmentsChromaffin_cells.Chrom_SOX2_statusHigh.HTR4	1.92698892	0.54734039	2.51163325	0.01420369	0.03559912	-3.3311728	HTR4	assignmentsChromaffin_cells.Chrom_SOX2_statusHigh
assignmentsChromaffin_cells.Chrom_SOX2_statusHigh.SGMS2	1.65158326	3.53483561	2.62014733	0.01066478	0.02807185	-3.3347309	SGMS2	assignmentsChromaffin_cells.Chrom_SOX2_statusHigh
assignmentsChromaffin_cells.Chrom_SOX2_statusHigh.DBNDD1	0.8902137	1.8393892	2.62866823	0.01041858	0.02753677	-3.3348004	DBNDD1	assignmentsChromaffin_cells.Chrom_SOX2_statusHigh
assignmentsChromaffin_cells.Chrom_SOX2_statusHigh.LINC00960	-1.3999233	2.85848547	-2.6077802	0.01102016	0.02885572	-3.3348801	LINC00960	assignmentsChromaffin_cells.Chrom_SOX2_statusHigh
assignmentsChromaffin_cells.Chrom_SOX2_statusHigh.APOBEC3C	1.48930934	1.84602738	2.5592742	0.01253395	0.03212201	-3.3362957	APOBEC3C	assignmentsChromaffin_cells.Chrom_SOX2_statusHigh
assignmentsChromaffin_cells.Chrom_SOX2_statusHigh.LINC01239	-2.4490931	1.08099912	-2.4403011	0.01707603	0.04174141	-3.3372164	LINC01239	assignmentsChromaffin_cells.Chrom_SOX2_statusHigh
assignmentsChromaffin_cells.Chrom_SOX2_statusHigh.ADAM21	-1.6810973	0.55289493	-2.4614164	0.01617003	0.03987314	-3.3373502	ADAM21	assignmentsChromaffin_cells.Chrom_SOX2_statusHigh
assignmentsChromaffin_cells.Chrom_SOX2_statusHigh.C11orf58	0.58736723	6.88018651	2.71566326	0.00822838	0.0227021	-3.3374753	C11orf58	assignmentsChromaffin_cells.Chrom_SOX2_statusHigh
assignmentsChromaffin_cells.Chrom_SOX2_statusHigh.MIIP	0.91079277	2.89430101	2.58160379	0.01181272	0.03059223	-3.338035	MIIP	assignmentsChromaffin_cells.Chrom_SOX2_statusHigh
assignmentsChromaffin_cells.Chrom_SOX2_statusHigh.KSR2	-2.2917569	4.15984361	-2.6544539	0.00972492	0.02603812	-3.3385283	KSR2	assignmentsChromaffin_cells.Chrom_SOX2_statusHigh
assignmentsChromaffin_cells.Chrom_SOX2_statusHigh.MEX3B	1.09516152	1.32701698	2.52109744	0.01385231	0.03484916	-3.3401746	MEX3B	assignmentsChromaffin_cells.Chrom_SOX2_statusHigh
assignmentsChromaffin_cells.Chrom_SOX2_statusHigh.UBE4B	-0.7298803	6.3845207	-2.6852496	0.00894113	0.02429929	-3.3407561	UBE4B	assignmentsChromaffin_cells.Chrom_SOX2_statusHigh
assignmentsChromaffin_cells.Chrom_SOX2_statusHigh.PCBP1-AS1	-0.7202578	6.27894437	-2.6838795	0.00897453	0.02436247	-3.3416969	PCBP1-AS1	assignmentsChromaffin_cells.Chrom_SOX2_statusHigh
assignmentsChromaffin_cells.Chrom_SOX2_statusHigh.SLC28A1	1.56478362	0.6933972	2.53178585	0.01347271	0.03412972	-3.3418046	SLC28A1	assignmentsChromaffin_cells.Chrom_SOX2_statusHigh
assignmentsChromaffin_cells.Chrom_SOX2_statusHigh.MT-ND6	1.10185259	4.34160737	2.66192313	0.00952694	0.02560633	-3.3428836	MT-ND6	assignmentsChromaffin_cells.Chrom_SOX2_statusHigh
assignmentsChromaffin_cells.Chrom_SOX2_statusHigh.ZNHIT6	-0.5181389	5.52606855	-2.6550545	0.00970399	0.02601194	-3.3432853	ZNHIT6	assignmentsChromaffin_cells.Chrom_SOX2_statusHigh
assignmentsChromaffin_cells.Chrom_SOX2_statusHigh.AMMECR1	-0.7407019	4.61209054	-2.6371843	0.01018289	0.02703412	-3.3434572	AMMECR1	assignmentsChromaffin_cells.Chrom_SOX2_statusHigh
assignmentsChromaffin_cells.Chrom_SOX2_statusHigh.B3GNT2	0.85101632	5.2777029	2.67629678	0.00916141	0.02477753	-3.3443861	B3GNT2	assignmentsChromaffin_cells.Chrom_SOX2_statusHigh
assignmentsChromaffin_cells.Chrom_SOX2_statusHigh.RABL2A	-0.7053737	3.58215	-2.6225585	0.01059071	0.02791811	-3.3449452	RABL2A	assignmentsChromaffin_cells.Chrom_SOX2_statusHigh
assignmentsChromaffin_cells.Chrom_SOX2_statusHigh.SLC30A1	1.00078399	2.72659392	2.59625383	0.01136165	0.02957126	-3.3459867	SLC30A1	assignmentsChromaffin_cells.Chrom_SOX2_statusHigh
assignmentsChromaffin_cells.Chrom_SOX2_statusHigh.ECHDC1	0.42620919	5.94501794	2.69299037	0.00875457	0.02388667	-3.3477464	ECHDC1	assignmentsChromaffin_cells.Chrom_SOX2_statusHigh
assignmentsChromaffin_cells.Chrom_SOX2_statusHigh.AKAP8L	-0.4273863	6.25710412	-2.698339	0.00862775	0.02361294	-3.3477712	AKAP8L	assignmentsChromaffin_cells.Chrom_SOX2_statusHigh
assignmentsChromaffin_cells.Chrom_SOX2_statusHigh.ZBTB5	-0.4827331	3.8832977	-2.6315338	0.01033872	0.02738891	-3.3494942	ZBTB5	assignmentsChromaffin_cells.Chrom_SOX2_statusHigh
assignmentsChromaffin_cells.Chrom_SOX2_statusHigh.ALKBH8	-0.667845	3.78626715	-2.6144586	0.01082289	0.0284227	-3.3518452	ALKBH8	assignmentsChromaffin_cells.Chrom_SOX2_statusHigh
assignmentsChromaffin_cells.Chrom_SOX2_statusHigh.CD74	1.86198013	5.74899872	2.60683284	0.01105122	0.0289229	-3.3525979	CD74	assignmentsChromaffin_cells.Chrom_SOX2_statusHigh
assignmentsChromaffin_cells.Chrom_SOX2_statusHigh.IL1RAPL1	-2.2417095	3.42537787	-2.484573	0.01523855	0.03787909	-3.3527698	IL1RAPL1	assignmentsChromaffin_cells.Chrom_SOX2_statusHigh
assignmentsChromaffin_cells.Chrom_SOX2_statusHigh.TMEM259	0.6435852	5.8932457	2.69550217	0.0086948	0.02376807	-3.3536515	TMEM259	assignmentsChromaffin_cells.Chrom_SOX2_statusHigh
assignmentsChromaffin_cells.Chrom_SOX2_statusHigh.LINC02653	-2.2305378	0.22491627	-2.4118925	0.01835311	0.04430936	-3.3538191	LINC02653	assignmentsChromaffin_cells.Chrom_SOX2_statusHigh
assignmentsChromaffin_cells.Chrom_SOX2_statusHigh.FANCL	-0.7855307	6.45669328	-2.7031738	0.00851455	0.02335495	-3.3538581	FANCL	assignmentsChromaffin_cells.Chrom_SOX2_statusHigh
assignmentsChromaffin_cells.Chrom_SOX2_statusHigh.UBA6	-0.4246543	6.93629019	-2.7035243	0.0085064	0.02333658	-3.3552003	UBA6	assignmentsChromaffin_cells.Chrom_SOX2_statusHigh
assignmentsChromaffin_cells.Chrom_SOX2_statusHigh.MRPS5	-0.7482796	6.4793996	-2.6946095	0.008716	0.02381384	-3.357208	MRPS5	assignmentsChromaffin_cells.Chrom_SOX2_statusHigh
assignmentsChromaffin_cells.Chrom_SOX2_statusHigh.SPSB3	0.90494557	4.62398623	2.65041204	0.00982639	0.02625719	-3.3576342	SPSB3	assignmentsChromaffin_cells.Chrom_SOX2_statusHigh
assignmentsChromaffin_cells.Chrom_SOX2_statusHigh.LINC02603	-1.6092195	1.16118638	-2.4747564	0.01562483	0.03870729	-3.358171	LINC02603	assignmentsChromaffin_cells.Chrom_SOX2_statusHigh
assignmentsChromaffin_cells.Chrom_SOX2_statusHigh.TMEM53	0.84728103	2.55276144	2.60374255	0.01113715	0.02910426	-3.3583154	TMEM53	assignmentsChromaffin_cells.Chrom_SOX2_statusHigh
assignmentsChromaffin_cells.Chrom_SOX2_statusHigh.TMEM51	-1.0471439	5.04730183	-2.6422217	0.01004632	0.02670693	-3.3597045	TMEM51	assignmentsChromaffin_cells.Chrom_SOX2_statusHigh
assignmentsChromaffin_cells.Chrom_SOX2_statusHigh.ATF7IP2	-1.4900062	6.10623813	-2.6305487	0.01037147	0.02745298	-3.360833	ATF7IP2	assignmentsChromaffin_cells.Chrom_SOX2_statusHigh
assignmentsChromaffin_cells.Chrom_SOX2_statusHigh.AFF2	-2.243676	2.09740502	-2.3976251	0.01902833	0.04567824	-3.3614247	AFF2	assignmentsChromaffin_cells.Chrom_SOX2_statusHigh
assignmentsChromaffin_cells.Chrom_SOX2_statusHigh.TREM2	2.26847431	0.08144408	2.36778097	0.02051185	0.04865686	-3.3620103	TREM2	assignmentsChromaffin_cells.Chrom_SOX2_statusHigh
assignmentsChromaffin_cells.Chrom_SOX2_statusHigh.ADPRM	0.69714326	2.70181294	2.59166696	0.01150117	0.02987622	-3.362489	ADPRM	assignmentsChromaffin_cells.Chrom_SOX2_statusHigh
assignmentsChromaffin_cells.Chrom_SOX2_statusHigh.INPP4A	-0.8979454	7.36837207	-2.6971285	0.00865631	0.02367899	-3.3651865	INPP4A	assignmentsChromaffin_cells.Chrom_SOX2_statusHigh
assignmentsChromaffin_cells.Chrom_SOX2_statusHigh.LAMB3	1.61117852	-0.0666978	2.38294274	0.01974316	0.0470939	-3.3661922	LAMB3	assignmentsChromaffin_cells.Chrom_SOX2_statusHigh
assignmentsChromaffin_cells.Chrom_SOX2_statusHigh.PLA2G4B	-0.894786	2.46132228	-2.5694405	0.01219948	0.03141347	-3.3664917	PLA2G4B	assignmentsChromaffin_cells.Chrom_SOX2_statusHigh
assignmentsChromaffin_cells.Chrom_SOX2_statusHigh.NFKBIL1	0.74151156	3.68979512	2.64350894	0.01001099	0.02664835	-3.3667623	NFKBIL1	assignmentsChromaffin_cells.Chrom_SOX2_statusHigh
assignmentsChromaffin_cells.Chrom_SOX2_statusHigh.PBX2	-0.556279	4.28063963	-2.6252893	0.01051345	0.02775095	-3.3684662	PBX2	assignmentsChromaffin_cells.Chrom_SOX2_statusHigh
assignmentsChromaffin_cells.Chrom_SOX2_statusHigh.GREB1L	-1.2826304	5.39087358	-2.6900308	0.00883039	0.0240608	-3.3697937	GREB1L	assignmentsChromaffin_cells.Chrom_SOX2_statusHigh
assignmentsChromaffin_cells.Chrom_SOX2_statusHigh.ZNF676	-2.4844812	4.08393734	-2.6152009	0.01080689	0.02839465	-3.3701752	ZNF676	assignmentsChromaffin_cells.Chrom_SOX2_statusHigh
assignmentsChromaffin_cells.Chrom_SOX2_statusHigh.GTF3C2	-0.6116933	4.79884311	-2.6494985	0.00985064	0.02631322	-3.3706119	GTF3C2	assignmentsChromaffin_cells.Chrom_SOX2_statusHigh
assignmentsChromaffin_cells.Chrom_SOX2_statusHigh.PTPN20	3.10899504	1.22812964	2.52507481	0.01371526	0.0345696	-3.3738243	PTPN20	assignmentsChromaffin_cells.Chrom_SOX2_statusHigh
assignmentsChromaffin_cells.Chrom_SOX2_statusHigh.MATN1-AS1	1.33169318	0.8939977	2.4289278	0.01757206	0.04273248	-3.3739357	MATN1-AS1	assignmentsChromaffin_cells.Chrom_SOX2_statusHigh
assignmentsChromaffin_cells.Chrom_SOX2_statusHigh.ZNF197	-0.6174608	4.88617744	-2.6543372	0.00972281	0.0260368	-3.3740146	ZNF197	assignmentsChromaffin_cells.Chrom_SOX2_statusHigh
assignmentsChromaffin_cells.Chrom_SOX2_statusHigh.TBC1D4	-1.384467	7.7244153	-2.7139586	0.00827166	0.02279396	-3.3743652	TBC1D4	assignmentsChromaffin_cells.Chrom_SOX2_statusHigh

assignmentsChromaffin_cells.Chrom_SOX2_statusHigh.ZC4H2	-0.9717799	5.34801747	-2.6631711	0.00949356	0.02552091	-3.3749486	ZC4H2	assignmentsChromaffin_cells.Chrom_SOX2_statusHigh
assignmentsChromaffin_cells.Chrom_SOX2_statusHigh.SLC25A29	-0.7355001	4.79577212	-2.6478814	0.0098937	0.02641945	-3.3754245	SLC25A29	assignmentsChromaffin_cells.Chrom_SOX2_statusHigh
assignmentsChromaffin_cells.Chrom_SOX2_statusHigh.LINC01273	2.01411702	0.19409123	2.4397379	0.01709664	0.04177904	-3.3764151	LINC01273	assignmentsChromaffin_cells.Chrom_SOX2_statusHigh
assignmentsChromaffin_cells.Chrom_SOX2_statusHigh.LGALS3	1.87302966	3.51982746	2.46500034	0.01602394	0.03956765	-3.377756	LGALS3	assignmentsChromaffin_cells.Chrom_SOX2_statusHigh
assignmentsChromaffin_cells.Chrom_SOX2_statusHigh.AP2A2	-0.6873284	6.76848834	-2.6773835	0.00913442	0.02471285	-3.3778326	AP2A2	assignmentsChromaffin_cells.Chrom_SOX2_statusHigh
assignmentsChromaffin_cells.Chrom_SOX2_statusHigh.ZBTB39	0.72163133	1.8797794	2.56014983	0.01250256	0.03206206	-3.378006	ZBTB39	assignmentsChromaffin_cells.Chrom_SOX2_statusHigh
assignmentsChromaffin_cells.Chrom_SOX2_statusHigh.PRKCA-AS1	-1.997391	2.54704443	-2.5201464	0.01389299	0.03492957	-3.3790383	PRKCA-AS1	assignmentsChromaffin_cells.Chrom_SOX2_statusHigh
assignmentsChromaffin_cells.Chrom_SOX2_statusHigh.TMEM127	0.57972796	3.93257853	2.61529494	0.0107987	0.02837778	-3.379939	TMEM127	assignmentsChromaffin_cells.Chrom_SOX2_statusHigh
assignmentsChromaffin_cells.Chrom_SOX2_statusHigh.BEGAIN	-1.046996	4.49395667	-2.6663944	0.0094123	0.02535772	-3.3806112	BEGAIN	assignmentsChromaffin_cells.Chrom_SOX2_statusHigh
assignmentsChromaffin_cells.Chrom_SOX2_statusHigh.ARF4	0.86989346	6.21918364	2.68509648	0.00894486	0.02429929	-3.3808995	ARF4	assignmentsChromaffin_cells.Chrom_SOX2_statusHigh
assignmentsChromaffin_cells.Chrom_SOX2_statusHigh.VWA5B1	1.91464092	0.67857093	2.54308083	0.01308322	0.03328501	-3.3809667	VWA5B1	assignmentsChromaffin_cells.Chrom_SOX2_statusHigh
assignmentsChromaffin_cells.Chrom_SOX2_statusHigh.PPIL6	0.80327481	2.69412345	2.59858972	0.01129119	0.02943085	-3.3814059	PPIL6	assignmentsChromaffin_cells.Chrom_SOX2_statusHigh
assignmentsChromaffin_cells.Chrom_SOX2_statusHigh.STPG2-AS1	-1.5363743	0.08023313	-2.4329725	0.01739172	0.04237101	-3.3839029	STPG2-AS1	assignmentsChromaffin_cells.Chrom_SOX2_statusHigh
assignmentsChromaffin_cells.Chrom_SOX2_statusHigh.LIG4	0.50404101	3.79514833	2.63358018	0.01028204	0.02727027	-3.3840934	LIG4	assignmentsChromaffin_cells.Chrom_SOX2_statusHigh
assignmentsChromaffin_cells.Chrom_SOX2_statusHigh.NSMAF	-0.5524686	6.04374353	-2.6644806	0.00945971	0.02545718	-3.3846424	NSMAF	assignmentsChromaffin_cells.Chrom_SOX2_statusHigh
assignmentsChromaffin_cells.Chrom_SOX2_statusHigh.OMG	-1.3198869	2.99721429	-2.5775547	0.01194238	0.03086066	-3.3851925	OMG	assignmentsChromaffin_cells.Chrom_SOX2_statusHigh
assignmentsChromaffin_cells.Chrom_SOX2_statusHigh.PPP2R3B	-0.7440394	3.42821147	-2.6048213	0.01110514	0.02904979	-3.3866886	PPP2R3B	assignmentsChromaffin_cells.Chrom_SOX2_statusHigh
assignmentsChromaffin_cells.Chrom_SOX2_statusHigh.SHROOM4	-2.0161084	3.25063832	-2.4101733	0.018433	0.04446208	-3.3907294	SHROOM4	assignmentsChromaffin_cells.Chrom_SOX2_statusHigh
assignmentsChromaffin_cells.Chrom_SOX2_statusHigh.MIB1	-0.4517703	7.06348259	-2.6939398	0.00873194	0.02384519	-3.3910005	MIB1	assignmentsChromaffin_cells.Chrom_SOX2_statusHigh
assignmentsChromaffin_cells.Chrom_SOX2_statusHigh.SLC30A6	-0.5328285	5.38507118	-2.6568373	0.00965735	0.0259081	-3.391277	SLC30A6	assignmentsChromaffin_cells.Chrom_SOX2_statusHigh
assignmentsChromaffin_cells.Chrom_SOX2_statusHigh.ARL6IP6	-0.5743713	5.27767636	-2.6442741	0.00999038	0.02661114	-3.3917524	ARL6IP6	assignmentsChromaffin_cells.Chrom_SOX2_statusHigh
assignmentsChromaffin_cells.Chrom_SOX2_statusHigh.ARHGEF38	-1.4172775	1.0413092	-2.4585111	0.01629075	0.0401338	-3.3958555	ARHGEF38	assignmentsChromaffin_cells.Chrom_SOX2_statusHigh
assignmentsChromaffin_cells.Chrom_SOX2_statusHigh.HSD17B3	2.2267712	0.28311225	2.37204989	0.02029401	0.04823286	-3.397166	HSD17B3	assignmentsChromaffin_cells.Chrom_SOX2_statusHigh
assignmentsChromaffin_cells.Chrom_SOX2_statusHigh.TSPYL5	1.69139965	1.82957885	2.55434727	0.01270124	0.03246256	-3.397863	TSPYL5	assignmentsChromaffin_cells.Chrom_SOX2_statusHigh
assignmentsChromaffin_cells.Chrom_SOX2_statusHigh.PAGR1	-0.6112838	4.76815363	-2.6440059	0.0099976	0.02662153	-3.3984743	PAGR1	assignmentsChromaffin_cells.Chrom_SOX2_statusHigh
assignmentsChromaffin_cells.Chrom_SOX2_statusHigh.SGCE	-0.6719479	6.44122273	-2.6933259	0.00874657	0.02386889	-3.4001638	SGCE	assignmentsChromaffin_cells.Chrom_SOX2_statusHigh
assignmentsChromaffin_cells.Chrom_SOX2_statusHigh.UBIAD1	0.64915275	2.59103625	2.56719422	0.01227214	0.03156706	-3.4011351	UBIAD1	assignmentsChromaffin_cells.Chrom_SOX2_statusHigh
assignmentsChromaffin_cells.Chrom_SOX2_statusHigh.ZNRF1	-0.7444069	5.50885903	-2.6447524	0.00997751	0.02658569	-3.4038723	ZNRF1	assignmentsChromaffin_cells.Chrom_SOX2_statusHigh
assignmentsChromaffin_cells.Chrom_SOX2_statusHigh.TAC3	1.50781253	-0.1863473	2.45913135	0.01626481	0.04008221	-3.4041161	TAC3	assignmentsChromaffin_cells.Chrom_SOX2_statusHigh
assignmentsChromaffin_cells.Chrom_SOX2_statusHigh.OLFM1	1.05893852	4.40775213	2.6661179	0.00941976	0.02537356	-3.4042239	OLFM1	assignmentsChromaffin_cells.Chrom_SOX2_statusHigh
assignmentsChromaffin_cells.Chrom_SOX2_statusHigh.RBFOX1	-3.0052946	6.25235084	-2.6740056	0.00922361	0.02491634	-3.407389	RBFOX1	assignmentsChromaffin_cells.Chrom_SOX2_statusHigh
assignmentsChromaffin_cells.Chrom_SOX2_statusHigh.IGFBP5	1.89014082	4.50736138	2.56229166	0.01243798	0.03193218	-3.4074912	IGFBP5	assignmentsChromaffin_cells.Chrom_SOX2_statusHigh
assignmentsChromaffin_cells.Chrom_SOX2_statusHigh.JAZF1-AS1	-0.8954822	1.81765919	-2.5533458	0.01272883	0.03252266	-3.4096323	JAZF1-AS1	assignmentsChromaffin_cells.Chrom_SOX2_statusHigh
assignmentsChromaffin_cells.Chrom_SOX2_statusHigh.CIB4	2.33084555	0.87336962	2.51608337	0.01404106	0.0352466	-3.4100461	CIB4	assignmentsChromaffin_cells.Chrom_SOX2_statusHigh
assignmentsChromaffin_cells.Chrom_SOX2_statusHigh.ZSCAN16	0.57969425	1.91675069	2.54563843	0.01298959	0.03308354	-3.4113616	ZSCAN16	assignmentsChromaffin_cells.Chrom_SOX2_statusHigh
assignmentsChromaffin_cells.Chrom_SOX2_statusHigh.GALR1	2.75772941	2.91602999	2.61422512	0.01083513	0.02843158	-3.411386	GALR1	assignmentsChromaffin_cells.Chrom_SOX2_statusHigh
assignmentsChromaffin_cells.Chrom_SOX2_statusHigh.PDGFRL	1.25513593	1.9519213	2.56929601	0.01220583	0.03142168	-3.4122362	PDGFRL	assignmentsChromaffin_cells.Chrom_SOX2_statusHigh
assignmentsChromaffin_cells.Chrom_SOX2_statusHigh.PSD4	1.4862943	1.2720588	2.42938346	0.01755166	0.04270145	-3.4134647	PSD4	assignmentsChromaffin_cells.Chrom_SOX2_statusHigh
assignmentsChromaffin_cells.Chrom_SOX2_statusHigh.RBP1	2.56749772	2.28117823	2.54506623	0.01301517	0.03313289	-3.4148691	RBP1	assignmentsChromaffin_cells.Chrom_SOX2_statusHigh
assignmentsChromaffin_cells.Chrom_SOX2_statusHigh.DCUN1D4	-0.4540034	6.52842953	-2.6776986	0.0091266	0.02470004	-3.4153787	DCUN1D4	assignmentsChromaffin_cells.Chrom_SOX2_statusHigh
assignmentsChromaffin_cells.Chrom_SOX2_statusHigh.ZNF629	0.60750053	2.7484806	2.58463975	0.01171794	0.03038026	-3.4168739	ZNF629	assignmentsChromaffin_cells.Chrom_SOX2_statusHigh
assignmentsChromaffin_cells.Chrom_SOX2_statusHigh.CCDC159	0.58317896	3.73075788	2.60078389	0.01122536	0.02929737	-3.4173724	CCDC159	assignmentsChromaffin_cells.Chrom_SOX2_statusHigh
assignmentsChromaffin_cells.Chrom_SOX2_statusHigh.LIPC	1.77528892	0.65381453	2.35818403	0.02100846	0.04964398	-3.4177543	LIPC	assignmentsChromaffin_cells.Chrom_SOX2_statusHigh
assignmentsChromaffin_cells.Chrom_SOX2_statusHigh.RAD51-AS1	-0.7647567	4.25730348	-2.6104326	0.01094	0.0286786	-3.4178088	RAD51-AS1	assignmentsChromaffin_cells.Chrom_SOX2_statusHigh
assignmentsChromaffin_cells.Chrom_SOX2_statusHigh.OXTR	1.41464798	0.7177873	2.44502749	0.01686399	0.04131754	-3.420773	OXTR	assignmentsChromaffin_cells.Chrom_SOX2_statusHigh
assignmentsChromaffin_cells.Chrom_SOX2_statusHigh.ZNF410	-0.5531836	5.52683424	-2.6367809	0.01019394	0.02705899	-3.4208063	ZNF410	assignmentsChromaffin_cells.Chrom_SOX2_statusHigh
assignmentsChromaffin_cells.Chrom_SOX2_statusHigh.LRRC4C	-1.8275375	5.5460462	-2.6551355	0.00970704	0.02601194	-3.4237806	LRRC4C	assignmentsChromaffin_cells.Chrom_SOX2_statusHigh
assignmentsChromaffin_cells.Chrom_SOX2_statusHigh.EME2	-0.8762461	2.7724882	-2.5641372	0.01237166	0.03178227	-3.4248199	EME2	assignmentsChromaffin_cells.Chrom_SOX2_statusHigh
assignmentsChromaffin_cells.Chrom_SOX2_statusHigh.TSNAX	0.41634278	5.79365759	2.66348723	0.00948519	0.02550268	-3.4251533	TSNAX	assignmentsChromaffin_cells.Chrom_SOX2_statusHigh
assignmentsChromaffin_cells.Chrom_SOX2_statusHigh.TRIM27	-0.5266226	5.54469161	-2.6306185	0.01036417	0.02743818	-3.4253549	TRIM27	assignmentsChromaffin_cells.Chrom_SOX2_statusHigh
assignmentsChromaffin_cells.Chrom_SOX2_statusHigh.LRRC1	-0.9534453	4.47298567	-2.6074574	0.01102728	0.02886966	-3.4262124	LRRC1	assignmentsChromaffin_cells.Chrom_SOX2_statusHigh
assignmentsChromaffin_cells.Chrom_SOX2_statusHigh.CTCF	-0.5878753	5.74867018	-2.6350482	0.01024155	0.02717636	-3.4268933	CTCF	assignmentsChromaffin_cells.Chrom_SOX2_statusHigh
assignmentsChromaffin_cells.Chrom_SOX2_statusHigh.MYH7B	-0.9411583	1.3053697	-2.4757708	0.01558249	0.03863822	-3.4270222	MYH7B	assignmentsChromaffin_cells.Chrom_SOX2_statusHigh
assignmentsChromaffin_cells.Chrom_SOX2_statusHigh.TRIM8	0.79512726	4.69207654	2.62510689	0.0105186	0.02775996	-3.4273104	TRIM8	assignmentsChromaffin_cells.Chrom_SOX2_statusHigh
assignmentsChromaffin_cells.Chrom_SOX2_statusHigh.DNAJC13	-0.6669662	6.34373275	-2.65072	0.00981823	0.02624162	-3.4279329	DNAJC13	assignmentsChromaffin_cells.Chrom_SOX2_statusHigh
assignmentsChromaffin_cells.Chrom_SOX2_statusHigh.IKZF2	-1.2525623	4.91980866	-2.5804793	0.01185016	0.03066357	-3.4283737	IKZF2	assignmentsChromaffin_cells.Chrom_SOX2_statusHigh
assignmentsChromaffin_cells.Chrom_SOX2_statusHigh.HSPD1	1.30233102	7.66150139	2.6987031	0.00862403	0.02360679	-3.4285298	HSPD1	assignmentsChromaffin_cells.Chrom_SOX2_statusHigh
assignmentsChromaffin_cells.Chrom_SOX2_statusHigh.SLC12A5	0.9372425	2.38796919	2.58794433	0.01161555	0.03014403	-3.4308424	SLC12A5	assignmentsChromaffin_cells.Chrom_SOX2_statusHigh
assignmentsChromaffin_cells.Chrom_SOX2_statusHigh.RGS7	-1.8233536	6.12227678	-2.6786587	0.00910782	0.02466587	-3.4309987	RGS7	assignmentsChromaffin_cells.Chrom_SOX2_statusHigh
assignmentsChromaffin_cells.Chrom_SOX2_statusHigh.FBXO33	-1.00029	6.58078071	-2.6636224	0.00948328	0.02550182	-3.4329111	FBXO33	assignmentsChromaffin_cells.Chrom_SOX2_statusHigh
assignmentsChromaffin_cells.Chrom_SOX2_statusHigh.MPDZ	-0.8391934	6.75821461	-2.6752941	0.00918639	0.02482833	-3.4338585	MPDZ	assignmentsChromaffin_cells.Chrom_SOX2_statusHigh
assignmentsChromaffin_cells.Chrom_SOX2_statusHigh.PKIA-AS1	-1.3404476	2.89806785	-2.587541	0.0116314	0.03017053	-3.4343962	PKIA-AS1	assignmentsChromaffin_cells.Chrom_SOX2_statusHigh
assignmentsChromaffin_cells.Chrom_SOX2_statusHigh.ANKRD37	1.34218934	3.53669004	2.5962038	0.01136737	0.02957656	-3.4344637	ANKRD37	assignmentsChromaffin_cells.Chrom_SOX2_statusHigh
assignmentsChromaffin_cells.Chrom_SOX2_statusHigh.SCNN1D	-0.9883997	1.21176095	-2.4888471	0.01506444	0.03749279	-3.4353433	SCNN1D	assignmentsChromaffin_cells.Chrom_SOX2_statusHigh

assignmentsChromaffin_cells.Chrom_SOX2_statusHigh.STK32A-AS1	-0.9744666	1.74123443	-2.5533427	0.01272893	0.03252266	-3.4357751	STK32A-AS1	assignmentsChromaffin_cells.Chrom_SOX2_statusHigh
assignmentsChromaffin_cells.Chrom_SOX2_statusHigh.ANKRD11	-0.7090219	8.19197171	-2.6851347	0.00894393	0.02429929	-3.4362453	ANKRD11	assignmentsChromaffin_cells.Chrom_SOX2_statusHigh
assignmentsChromaffin_cells.Chrom_SOX2_statusHigh.PRTG	1.47844211	3.85054767	2.62513585	0.01052318	0.02776749	-3.4365057	PRTG	assignmentsChromaffin_cells.Chrom_SOX2_statusHigh
assignmentsChromaffin_cells.Chrom_SOX2_statusHigh.GRIP1	-1.4064883	6.6113079	-2.686166	0.0089238	0.02426585	-3.4366435	GRIP1	assignmentsChromaffin_cells.Chrom_SOX2_statusHigh
assignmentsChromaffin_cells.Chrom_SOX2_statusHigh.ZNF610	-1.0726938	2.77326911	-2.5502322	0.01283359	0.03274864	-3.4377183	ZNF610	assignmentsChromaffin_cells.Chrom_SOX2_statusHigh
assignmentsChromaffin_cells.Chrom_SOX2_statusHigh.ASIC3	-0.931337	1.5202689	-2.538559	0.01323335	0.03360743	-3.4379144	ASIC3	assignmentsChromaffin_cells.Chrom_SOX2_statusHigh
assignmentsChromaffin_cells.Chrom_SOX2_statusHigh.HOXC5	1.72762903	0.39376471	2.49281007	0.01491561	0.03716851	-3.4382691	HOXC5	assignmentsChromaffin_cells.Chrom_SOX2_statusHigh
assignmentsChromaffin_cells.Chrom_SOX2_statusHigh.TMEM150A	0.69478424	3.06417415	2.58249204	0.01178492	0.03052925	-3.4384499	TMEM150A	assignmentsChromaffin_cells.Chrom_SOX2_statusHigh
assignmentsChromaffin_cells.Chrom_SOX2_statusHigh.LINC01470	-1.6311401	2.54797082	-2.5614503	0.01246563	0.03199804	-3.4387766	LINC01470	assignmentsChromaffin_cells.Chrom_SOX2_statusHigh
assignmentsChromaffin_cells.Chrom_SOX2_statusHigh.KLF6	1.62065103	7.33977367	2.65459788	0.00972114	0.02603669	-3.4391612	KLF6	assignmentsChromaffin_cells.Chrom_SOX2_statusHigh
assignmentsChromaffin_cells.Chrom_SOX2_statusHigh.RPS6KA6	-0.9648553	4.85860404	-2.6531241	0.009756	0.02609955	-3.4392599	RPS6KA6	assignmentsChromaffin_cells.Chrom_SOX2_statusHigh
assignmentsChromaffin_cells.Chrom_SOX2_statusHigh.VEPH1	-2.0106289	0.94318496	-2.4032697	0.01875858	0.04511173	-3.4403135	VEPH1	assignmentsChromaffin_cells.Chrom_SOX2_statusHigh
assignmentsChromaffin_cells.Chrom_SOX2_statusHigh.NDUFAF5	-0.5773117	5.02401917	-2.6287794	0.01041547	0.02753309	-3.4412225	NDUFAF5	assignmentsChromaffin_cells.Chrom_SOX2_statusHigh
assignmentsChromaffin_cells.Chrom_SOX2_statusHigh.STRADA	-0.5464126	5.52551778	-2.6309558	0.01035478	0.02742239	-3.4413988	STRADA	assignmentsChromaffin_cells.Chrom_SOX2_statusHigh
assignmentsChromaffin_cells.Chrom_SOX2_statusHigh.MMADHC	0.5924722	4.94974815	2.6297267	0.01038901	0.02748582	-3.4417692	MMADHC	assignmentsChromaffin_cells.Chrom_SOX2_statusHigh
assignmentsChromaffin_cells.Chrom_SOX2_statusHigh.HEYL	1.60753154	2.28728978	2.44791381	0.01674175	0.04106824	-3.4424682	HEYL	assignmentsChromaffin_cells.Chrom_SOX2_statusHigh
assignmentsChromaffin_cells.Chrom_SOX2_statusHigh.MND1	1.17456012	1.47057996	2.52978543	0.01354117	0.03423826	-3.4425017	MND1	assignmentsChromaffin_cells.Chrom_SOX2_statusHigh
assignmentsChromaffin_cells.Chrom_SOX2_statusHigh.ARCN1	0.53663562	5.56504128	2.64531951	0.00996227	0.02655057	-3.4433408	ARCN1	assignmentsChromaffin_cells.Chrom_SOX2_statusHigh
assignmentsChromaffin_cells.Chrom_SOX2_statusHigh.ADAM12	1.89975182	3.10700357	2.47538287	0.01560469	0.03867226	-3.4443156	ADAM12	assignmentsChromaffin_cells.Chrom_SOX2_statusHigh
assignmentsChromaffin_cells.Chrom_SOX2_statusHigh.CCDC18-AS1	-0.6677472	7.02403269	-2.6635904	0.00948254	0.02550182	-3.44459	CCDC18-AS1	assignmentsChromaffin_cells.Chrom_SOX2_statusHigh
assignmentsChromaffin_cells.Chrom_SOX2_statusHigh.MT-ND3	2.88972907	6.8572919	2.67514073	0.00919524	0.02484388	-3.4454918	MT-ND3	assignmentsChromaffin_cells.Chrom_SOX2_statusHigh
assignmentsChromaffin_cells.Chrom_SOX2_statusHigh.FUT2	-1.8229484	0.18807546	-2.3827636	0.01975381	0.04710953	-3.4455236	FUT2	assignmentsChromaffin_cells.Chrom_SOX2_statusHigh
assignmentsChromaffin_cells.Chrom_SOX2_statusHigh.MIR4300HG	1.54580543	1.49782062	2.52729424	0.01363397	0.03442949	-3.446063	MIR4300HG	assignmentsChromaffin_cells.Chrom_SOX2_statusHigh
assignmentsChromaffin_cells.Chrom_SOX2_statusHigh.TMEM178B	-1.3064067	7.06982824	-2.6875442	0.00889039	0.02419396	-3.4465654	TMEM178B	assignmentsChromaffin_cells.Chrom_SOX2_statusHigh
assignmentsChromaffin_cells.Chrom_SOX2_statusHigh.CKMT2-AS1	-0.6323578	5.66592945	-2.6402322	0.01009972	0.0268222	-3.4471757	CKMT2-AS1	assignmentsChromaffin_cells.Chrom_SOX2_statusHigh
assignmentsChromaffin_cells.Chrom_SOX2_statusHigh.CORO1A	1.37754201	2.06244255	2.42830877	0.01759981	0.0427805	-3.4471857	CORO1A	assignmentsChromaffin_cells.Chrom_SOX2_statusHigh
assignmentsChromaffin_cells.Chrom_SOX2_statusHigh.TMC7	-1.1117445	2.46183028	-2.5165327	0.01401837	0.03520065	-3.4472376	TMC7	assignmentsChromaffin_cells.Chrom_SOX2_statusHigh
assignmentsChromaffin_cells.Chrom_SOX2_statusHigh.BBS9	-0.7868761	7.79241879	-2.6760196	0.00916831	0.02479201	-3.4478299	BBS9	assignmentsChromaffin_cells.Chrom_SOX2_statusHigh
assignmentsChromaffin_cells.Chrom_SOX2_statusHigh.ZNF462	-0.9029969	5.89199879	-2.64169	0.01006016	0.02673043	-3.44796	ZNF462	assignmentsChromaffin_cells.Chrom_SOX2_statusHigh
assignmentsChromaffin_cells.Chrom_SOX2_statusHigh.BAIAP2L1	-1.5951063	2.30074152	-2.4700407	0.01581698	0.03912287	-3.4479952	BAIAP2L1	assignmentsChromaffin_cells.Chrom_SOX2_statusHigh
assignmentsChromaffin_cells.Chrom_SOX2_statusHigh.TEKT3	-1.2514587	1.30059359	-2.499358	0.01465931	0.03662658	-3.4482437	TEKT3	assignmentsChromaffin_cells.Chrom_SOX2_statusHigh
assignmentsChromaffin_cells.Chrom_SOX2_statusHigh.STARD9	-0.8232108	5.6243595	-2.6120263	0.0108935	0.02857073	-3.4487615	STARD9	assignmentsChromaffin_cells.Chrom_SOX2_statusHigh
assignmentsChromaffin_cells.Chrom_SOX2_statusHigh.LINC02267	-1.2439597	0.11430365	-2.3821342	0.01978338	0.04715902	-3.4489753	LINC02267	assignmentsChromaffin_cells.Chrom_SOX2_statusHigh
assignmentsChromaffin_cells.Chrom_SOX2_statusHigh.FGF14-AS1	-2.4538995	0.33716523	-2.4108209	0.0184072	0.04441597	-3.4503711	FGF14-AS1	assignmentsChromaffin_cells.Chrom_SOX2_statusHigh
assignmentsChromaffin_cells.Chrom_SOX2_statusHigh.GABRB2	-1.8146386	1.04175016	-2.357132	0.0210638	0.0497381	-3.4505042	GABRB2	assignmentsChromaffin_cells.Chrom_SOX2_statusHigh
assignmentsChromaffin_cells.Chrom_SOX2_statusHigh.TAB3	-0.4869864	4.98863283	-2.618325	0.01071149	0.02816705	-3.4506118	TAB3	assignmentsChromaffin_cells.Chrom_SOX2_statusHigh
assignmentsChromaffin_cells.Chrom_SOX2_statusHigh.TRMT13	-0.520023	5.03089867	-2.6131603	0.01086053	0.02849356	-3.4521628	TRMT13	assignmentsChromaffin_cells.Chrom_SOX2_statusHigh
assignmentsChromaffin_cells.Chrom_SOX2_statusHigh.NPAS3	-1.9162466	6.41251812	-2.631064	0.01035713	0.02742408	-3.4529457	NPAS3	assignmentsChromaffin_cells.Chrom_SOX2_statusHigh
assignmentsChromaffin_cells.Chrom_SOX2_statusHigh.SPTBN4	-0.9363498	5.34621875	-2.6512187	0.00980606	0.02622034	-3.4530772	SPTBN4	assignmentsChromaffin_cells.Chrom_SOX2_statusHigh
assignmentsChromaffin_cells.Chrom_SOX2_statusHigh.CNOT3	0.50703051	3.97504124	2.60429391	0.01112078	0.02908595	-3.4531511	CNOT3	assignmentsChromaffin_cells.Chrom_SOX2_statusHigh
assignmentsChromaffin_cells.Chrom_SOX2_statusHigh.MSH5	-0.7850888	3.79196381	-2.5779742	0.01192696	0.03083245	-3.453186	MSH5	assignmentsChromaffin_cells.Chrom_SOX2_statusHigh
assignmentsChromaffin_cells.Chrom_SOX2_statusHigh.BMP2K	-0.7747074	6.78300987	-2.6219679	0.01060748	0.02794855	-3.4553958	BMP2K	assignmentsChromaffin_cells.Chrom_SOX2_statusHigh
assignmentsChromaffin_cells.Chrom_SOX2_statusHigh.CTU2	0.76772025	2.43417868	2.55505858	0.01267152	0.03239693	-3.4559217	CTU2	assignmentsChromaffin_cells.Chrom_SOX2_statusHigh
assignmentsChromaffin_cells.Chrom_SOX2_statusHigh.GLIPR1L1	-1.5138628	2.12993051	-2.5236895	0.01376251	0.03466131	-3.4572298	GLIPR1L1	assignmentsChromaffin_cells.Chrom_SOX2_statusHigh
assignmentsChromaffin_cells.Chrom_SOX2_statusHigh.LINC02008	1.51633627	0.08082297	2.38419069	0.01968122	0.04697133	-3.4578325	LINC02008	assignmentsChromaffin_cells.Chrom_SOX2_statusHigh
assignmentsChromaffin_cells.Chrom_SOX2_statusHigh.CASTOR1	1.06808574	1.765053	2.47912893	0.01544795	0.03833423	-3.4584365	CASTOR1	assignmentsChromaffin_cells.Chrom_SOX2_statusHigh
assignmentsChromaffin_cells.Chrom_SOX2_statusHigh.GINS2	1.16074461	0.80017138	2.49456828	0.01484269	0.03700405	-3.4594755	GINS2	assignmentsChromaffin_cells.Chrom_SOX2_statusHigh
assignmentsChromaffin_cells.Chrom_SOX2_statusHigh.SMIM13	-0.4916864	4.411198	-2.5969836	0.01133959	0.02952344	-3.4605966	SMIM13	assignmentsChromaffin_cells.Chrom_SOX2_statusHigh
assignmentsChromaffin_cells.Chrom_SOX2_statusHigh.NTPCR	0.58803494	5.23380232	2.64406496	0.00999601	0.02662153	-3.4612425	NTPCR	assignmentsChromaffin_cells.Chrom_SOX2_statusHigh
assignmentsChromaffin_cells.Chrom_SOX2_statusHigh.RASD2	1.69707686	1.57038384	2.53172314	0.01347876	0.03413427	-3.4618517	RASD2	assignmentsChromaffin_cells.Chrom_SOX2_statusHigh
assignmentsChromaffin_cells.Chrom_SOX2_statusHigh.CSMD1	-2.7379246	2.76326075	-2.4470639	0.01678308	0.04115075	-3.4624876	CSMD1	assignmentsChromaffin_cells.Chrom_SOX2_statusHigh
assignmentsChromaffin_cells.Chrom_SOX2_statusHigh.PUS7	-0.7183113	4.84428684	-2.6013411	0.0112087	0.02926817	-3.4642022	PUS7	assignmentsChromaffin_cells.Chrom_SOX2_statusHigh
assignmentsChromaffin_cells.Chrom_SOX2_statusHigh.TXNDC16	-0.7073393	5.40905782	-2.6181911	0.01071533	0.02817253	-3.4655963	TXNDC16	assignmentsChromaffin_cells.Chrom_SOX2_statusHigh
assignmentsChromaffin_cells.Chrom_SOX2_statusHigh.LINC02693	0.89870176	4.10479288	2.6212774	0.01062713	0.02799111	-3.4664925	LINC02693	assignmentsChromaffin_cells.Chrom_SOX2_statusHigh
assignmentsChromaffin_cells.Chrom_SOX2_statusHigh.CDH13	-2.1564245	5.19244326	-2.4926764	0.01492214	0.03717337	-3.4666822	CDH13	assignmentsChromaffin_cells.Chrom_SOX2_statusHigh
assignmentsChromaffin_cells.Chrom_SOX2_statusHigh.TINAGL1	1.70522964	1.66033265	2.38242662	0.01976887	0.04713845	-3.4708301	TINAGL1	assignmentsChromaffin_cells.Chrom_SOX2_statusHigh
assignmentsChromaffin_cells.Chrom_SOX2_statusHigh.PGAP1	-1.1069226	7.32901898	-2.6683609	0.0093648	0.02524671	-3.4709497	PGAP1	assignmentsChromaffin_cells.Chrom_SOX2_statusHigh
assignmentsChromaffin_cells.Chrom_SOX2_statusHigh.ABCA7	-0.899363	4.26734133	-2.5950421	0.01139836	0.02964275	-3.471791	ABCA7	assignmentsChromaffin_cells.Chrom_SOX2_statusHigh
assignmentsChromaffin_cells.Chrom_SOX2_statusHigh.NPIPB6	-1.4832387	0.66553443	-2.4169078	0.01811794	0.04388513	-3.4724867	NPIPB6	assignmentsChromaffin_cells.Chrom_SOX2_statusHigh
assignmentsChromaffin_cells.Chrom_SOX2_statusHigh.IGFBPL1	1.12431703	2.69524534	2.60830396	0.01100464	0.02883374	-3.4725804	IGFBPL1	assignmentsChromaffin_cells.Chrom_SOX2_statusHigh
assignmentsChromaffin_cells.Chrom_SOX2_statusHigh.DNAH7	1.20534519	3.05715285	2.58795104	0.01161772	0.0301448	-3.4729752	DNAH7	assignmentsChromaffin_cells.Chrom_SOX2_statusHigh
assignmentsChromaffin_cells.Chrom_SOX2_statusHigh.EDNRB	1.69222555	4.40361927	2.5334646	0.01341742	0.03400578	-3.4731077	EDNRB	assignmentsChromaffin_cells.Chrom_SOX2_statusHigh
assignmentsChromaffin_cells.Chrom_SOX2_statusHigh.TLE2	-0.6144248	4.57706168	-2.6089084	0.01098463	0.0287862	-3.4738952	TLE2	assignmentsChromaffin_cells.Chrom_SOX2_statusHigh
assignmentsChromaffin_cells.Chrom_SOX2_statusHigh.NISCH	-0.5750394	5.89038067	-2.6344337	0.01025848	0.02721678	-3.4749034	NISCH	assignmentsChromaffin_cells.Chrom_SOX2_statusHigh

assignmentsChromaffin_cells.Chrom_SOX2_statusHigh.RBMS3	-1.050925	9.94090846	-2.7133014	0.00828656	0.02282417	-3.4755266	RBMS3	assignmentsChromaffin_cells.Chrom_SOX2_statusHigh
assignmentsChromaffin_cells.Chrom_SOX2_statusHigh.OTUD7B	-0.6022665	5.64683542	-2.6294425	0.01039695	0.02749319	-3.4766584	OTUD7B	assignmentsChromaffin_cells.Chrom_SOX2_statusHigh
assignmentsChromaffin_cells.Chrom_SOX2_statusHigh.OCRL	-0.6378426	4.87420628	-2.6191291	0.01068845	0.02812492	-3.4768654	OCRL	assignmentsChromaffin_cells.Chrom_SOX2_statusHigh
assignmentsChromaffin_cells.Chrom_SOX2_statusHigh.ANXA9	1.23692204	-0.0702647	2.4166198	0.01813119	0.04389915	-3.4769353	ANXA9	assignmentsChromaffin_cells.Chrom_SOX2_statusHigh
assignmentsChromaffin_cells.Chrom_SOX2_statusHigh.TPP1	0.78418229	4.80934595	2.60411196	0.01112618	0.02909533	-3.4770699	TPP1	assignmentsChromaffin_cells.Chrom_SOX2_statusHigh
assignmentsChromaffin_cells.Chrom_SOX2_statusHigh.EFNB3	-1.3807699	1.76367815	-2.517126	0.01399922	0.03515807	-3.4774379	EFNB3	assignmentsChromaffin_cells.Chrom_SOX2_statusHigh
assignmentsChromaffin_cells.Chrom_SOX2_statusHigh.SLC35A5	0.54584421	4.15625423	2.58159345	0.01181305	0.03059223	-3.4829722	SLC35A5	assignmentsChromaffin_cells.Chrom_SOX2_statusHigh
assignmentsChromaffin_cells.Chrom_SOX2_statusHigh.C4orf45	-1.4278247	1.95236316	-2.4574758	0.01633573	0.0402199	-3.4830195	C4orf45	assignmentsChromaffin_cells.Chrom_SOX2_statusHigh
assignmentsChromaffin_cells.Chrom_SOX2_statusHigh.EPN2	-0.705892	6.22177135	-2.6259334	0.01049531	0.02771674	-3.4841955	EPN2	assignmentsChromaffin_cells.Chrom_SOX2_statusHigh
assignmentsChromaffin_cells.Chrom_SOX2_statusHigh.CALN1	0.9724523	2.24395872	2.53087868	0.01350246	0.0341727	-3.4848869	CALN1	assignmentsChromaffin_cells.Chrom_SOX2_statusHigh
assignmentsChromaffin_cells.Chrom_SOX2_statusHigh.NID2	-2.1919685	2.84809643	-2.4086009	0.0185111	0.04461692	-3.4872748	NID2	assignmentsChromaffin_cells.Chrom_SOX2_statusHigh
assignmentsChromaffin_cells.Chrom_SOX2_statusHigh.ZBTB34	-0.7089823	2.93303669	-2.5304012	0.01351935	0.03421005	-3.4877914	ZBTB34	assignmentsChromaffin_cells.Chrom_SOX2_statusHigh
assignmentsChromaffin_cells.Chrom_SOX2_statusHigh.GPS2	0.71241089	5.16167553	2.61127922	0.01091528	0.02861847	-3.4881732	GPS2	assignmentsChromaffin_cells.Chrom_SOX2_statusHigh
assignmentsChromaffin_cells.Chrom_SOX2_statusHigh.NAALAD2	1.73766375	2.18516461	2.52962818	0.01355288	0.03425707	-3.4885556	NAALAD2	assignmentsChromaffin_cells.Chrom_SOX2_statusHigh
assignmentsChromaffin_cells.Chrom_SOX2_statusHigh.ZNF92	0.68838055	3.821825	2.56431421	0.01236588	0.03177251	-3.4888927	ZNF92	assignmentsChromaffin_cells.Chrom_SOX2_statusHigh
assignmentsChromaffin_cells.Chrom_SOX2_statusHigh.TRMT5	0.65465372	2.93435883	2.56065084	0.01248605	0.03203507	-3.4889512	TRMT5	assignmentsChromaffin_cells.Chrom_SOX2_statusHigh
assignmentsChromaffin_cells.Chrom_SOX2_statusHigh.BARD1	-0.7956812	5.14432985	-2.5952464	0.01139216	0.02963625	-3.4922883	BARD1	assignmentsChromaffin_cells.Chrom_SOX2_statusHigh
assignmentsChromaffin_cells.Chrom_SOX2_statusHigh.MTDH	0.51222572	7.74324244	2.66569102	0.00942876	0.02539352	-3.4924442	MTDH	assignmentsChromaffin_cells.Chrom_SOX2_statusHigh
assignmentsChromaffin_cells.Chrom_SOX2_statusHigh.FAM215B	-1.470011	1.68161056	-2.4829698	0.01529807	0.03800937	-3.4936834	FAM215B	assignmentsChromaffin_cells.Chrom_SOX2_statusHigh
assignmentsChromaffin_cells.Chrom_SOX2_statusHigh.KLC4	-0.6522463	3.80461016	-2.5765409	0.01197234	0.03092485	-3.4943513	KLC4	assignmentsChromaffin_cells.Chrom_SOX2_statusHigh
assignmentsChromaffin_cells.Chrom_SOX2_statusHigh.PRXL2A	2.04363866	4.52243629	2.57762898	0.01194364	0.03086066	-3.4944185	PRXL2A	assignmentsChromaffin_cells.Chrom_SOX2_statusHigh
assignmentsChromaffin_cells.Chrom_SOX2_statusHigh.MSL3	0.69738992	4.27502179	2.55823393	0.01256591	0.03218333	-3.4956631	MSL3	assignmentsChromaffin_cells.Chrom_SOX2_statusHigh
assignmentsChromaffin_cells.Chrom_SOX2_statusHigh.PPP3CB-AS1	-0.7540006	2.73127837	-2.5197263	0.013902	0.0349442	-3.4966506	PPP3CB-AS1	assignmentsChromaffin_cells.Chrom_SOX2_statusHigh
assignmentsChromaffin_cells.Chrom_SOX2_statusHigh.ZFP2	-0.8815209	2.6851303	-2.5311115	0.01349423	0.03416265	-3.4988922	ZFP2	assignmentsChromaffin_cells.Chrom_SOX2_statusHigh
assignmentsChromaffin_cells.Chrom_SOX2_statusHigh.NDC1	-0.6188798	3.78749816	-2.5383879	0.01323929	0.03360743	-3.4993245	NDC1	assignmentsChromaffin_cells.Chrom_SOX2_statusHigh
assignmentsChromaffin_cells.Chrom_SOX2_statusHigh.GRIK1	-2.5458597	3.67621189	-2.5263014	0.01367135	0.03451207	-3.4997281	GRIK1	assignmentsChromaffin_cells.Chrom_SOX2_statusHigh
assignmentsChromaffin_cells.Chrom_SOX2_statusHigh.COG1	0.5783945	4.39645518	2.60082441	0.01122415	0.02929737	-3.5003495	COG1	assignmentsChromaffin_cells.Chrom_SOX2_statusHigh
assignmentsChromaffin_cells.Chrom_SOX2_statusHigh.PDE1A	-1.5145482	3.16252616	-2.3902777	0.01938157	0.04636659	-3.5004654	PDE1A	assignmentsChromaffin_cells.Chrom_SOX2_statusHigh
assignmentsChromaffin_cells.Chrom_SOX2_statusHigh.UNC80	-1.252446	6.64612949	-2.6643425	0.00946836	0.02547024	-3.5009409	UNC80	assignmentsChromaffin_cells.Chrom_SOX2_statusHigh
assignmentsChromaffin_cells.Chrom_SOX2_statusHigh.FASTKD2	-0.4973295	5.37042362	-2.6140629	0.01083435	0.02843158	-3.5023834	FASTKD2	assignmentsChromaffin_cells.Chrom_SOX2_statusHigh
assignmentsChromaffin_cells.Chrom_SOX2_statusHigh.ABHD17C	-0.8694283	2.74338291	-2.5224785	0.01380243	0.03474546	-3.5028107	ABHD17C	assignmentsChromaffin_cells.Chrom_SOX2_statusHigh
assignmentsChromaffin_cells.Chrom_SOX2_statusHigh.VMAC	0.73312255	2.48507953	2.53161168	0.01347656	0.0341341	-3.5028918	VMAC	assignmentsChromaffin_cells.Chrom_SOX2_statusHigh
assignmentsChromaffin_cells.Chrom_SOX2_statusHigh.DCTN1	-0.4407122	5.71322543	-2.6271588	0.01046086	0.02763033	-3.5031597	DCTN1	assignmentsChromaffin_cells.Chrom_SOX2_statusHigh
assignmentsChromaffin_cells.Chrom_SOX2_statusHigh.ABCA3	-0.7104612	4.46247061	-2.5923062	0.01148164	0.02983031	-3.5063768	ABCA3	assignmentsChromaffin_cells.Chrom_SOX2_statusHigh
assignmentsChromaffin_cells.Chrom_SOX2_statusHigh.IMPDH1	0.92122222	2.53944953	2.49651061	0.01476808	0.03684094	-3.5069863	IMPDH1	assignmentsChromaffin_cells.Chrom_SOX2_statusHigh
assignmentsChromaffin_cells.Chrom_SOX2_statusHigh.CAPN7	-0.6129603	7.27208857	-2.6459419	0.00994557	0.02651822	-3.5095364	CAPN7	assignmentsChromaffin_cells.Chrom_SOX2_statusHigh
assignmentsChromaffin_cells.Chrom_SOX2_statusHigh.MTSS1	1.48309013	7.21586407	2.64163391	0.01006695	0.02674404	-3.51173	MTSS1	assignmentsChromaffin_cells.Chrom_SOX2_statusHigh
assignmentsChromaffin_cells.Chrom_SOX2_statusHigh.GTF2H2	-0.9336944	4.35874104	-2.572043	0.01211578	0.03124001	-3.5138355	GTF2H2	assignmentsChromaffin_cells.Chrom_SOX2_statusHigh
assignmentsChromaffin_cells.Chrom_SOX2_statusHigh.P2RX5	1.60124422	0.19950021	2.35752604	0.02104305	0.04969642	-3.5139025	P2RX5	assignmentsChromaffin_cells.Chrom_SOX2_statusHigh
assignmentsChromaffin_cells.Chrom_SOX2_statusHigh.CCDC13	1.07179052	1.52520602	2.48046828	0.01539458	0.03821362	-3.514882	CCDC13	assignmentsChromaffin_cells.Chrom_SOX2_statusHigh
assignmentsChromaffin_cells.Chrom_SOX2_statusHigh.FBXO6	0.7681502	1.41007143	2.4488682	0.01669889	0.04097564	-3.515079	FBXO6	assignmentsChromaffin_cells.Chrom_SOX2_statusHigh
assignmentsChromaffin_cells.Chrom_SOX2_statusHigh.BTBD9	-0.7105137	8.62678058	-2.6671304	0.00939206	0.0253117	-3.5155927	BTBD9	assignmentsChromaffin_cells.Chrom_SOX2_statusHigh
assignmentsChromaffin_cells.Chrom_SOX2_statusHigh.CACNA2D3	-2.3498494	6.32887088	-2.6311704	0.01035417	0.02742239	-3.5172781	CACNA2D3	assignmentsChromaffin_cells.Chrom_SOX2_statusHigh
assignmentsChromaffin_cells.Chrom_SOX2_statusHigh.NPEPPS	-0.5250256	7.32662405	-2.6446108	0.00998132	0.02659142	-3.5189284	NPEPPS	assignmentsChromaffin_cells.Chrom_SOX2_statusHigh
assignmentsChromaffin_cells.Chrom_SOX2_statusHigh.RGS5	1.174194	9.09529378	2.70292059	0.00852527	0.02337635	-3.5201258	RGS5	assignmentsChromaffin_cells.Chrom_SOX2_statusHigh
assignmentsChromaffin_cells.Chrom_SOX2_statusHigh.FAM157C	-1.1627203	2.37948378	-2.4881353	0.01509224	0.03755031	-3.5215998	FAM157C	assignmentsChromaffin_cells.Chrom_SOX2_statusHigh
assignmentsChromaffin_cells.Chrom_SOX2_statusHigh.PRH1	-1.1173146	6.00040799	-2.6190139	0.01069477	0.02813692	-3.5247388	PRH1	assignmentsChromaffin_cells.Chrom_SOX2_statusHigh
assignmentsChromaffin_cells.Chrom_SOX2_statusHigh.ACHE	1.4276835	2.10934986	2.54986257	0.01285207	0.03277492	-3.5259029	ACHE	assignmentsChromaffin_cells.Chrom_SOX2_statusHigh
assignmentsChromaffin_cells.Chrom_SOX2_statusHigh.NHLRC2	-0.4521992	6.14215734	-2.6201214	0.01066009	0.0280641	-3.5273341	NHLRC2	assignmentsChromaffin_cells.Chrom_SOX2_statusHigh
assignmentsChromaffin_cells.Chrom_SOX2_statusHigh.SLC30A9	-0.3834978	6.90047194	-2.6360891	0.01021292	0.02710489	-3.5296808	SLC30A9	assignmentsChromaffin_cells.Chrom_SOX2_statusHigh
assignmentsChromaffin_cells.Chrom_SOX2_statusHigh.PPP2R1A	0.69847367	5.7952546	2.62270979	0.01058641	0.02791138	-3.5304704	PPP2R1A	assignmentsChromaffin_cells.Chrom_SOX2_statusHigh
assignmentsChromaffin_cells.Chrom_SOX2_statusHigh.GPR143	-1.3080767	0.730928	-2.4088685	0.01849142	0.04457618	-3.5306426	GPR143	assignmentsChromaffin_cells.Chrom_SOX2_statusHigh
assignmentsChromaffin_cells.Chrom_SOX2_statusHigh.C4orf48	1.36460527	4.5597323	2.59801523	0.01131409	0.02947617	-3.530734	C4orf48	assignmentsChromaffin_cells.Chrom_SOX2_statusHigh
assignmentsChromaffin_cells.Chrom_SOX2_statusHigh.MKRN2OS	-1.4069127	1.61532917	-2.4339215	0.0173508	0.04228417	-3.5317815	MKRN2OS	assignmentsChromaffin_cells.Chrom_SOX2_statusHigh
assignmentsChromaffin_cells.Chrom_SOX2_statusHigh.ZKSCAN8	-0.5002404	4.69039325	-2.5806256	0.01184341	0.03065602	-3.5321372	ZKSCAN8	assignmentsChromaffin_cells.Chrom_SOX2_statusHigh
assignmentsChromaffin_cells.Chrom_SOX2_statusHigh.NPIPB12	-1.2110555	1.53554184	-2.4345482	0.01732191	0.04222817	-3.5326383	NPIPB12	assignmentsChromaffin_cells.Chrom_SOX2_statusHigh
assignmentsChromaffin_cells.Chrom_SOX2_statusHigh.BDP1	-0.6534965	7.60201628	-2.6429674	0.01002561	0.02666342	-3.533368	BDP1	assignmentsChromaffin_cells.Chrom_SOX2_statusHigh
assignmentsChromaffin_cells.Chrom_SOX2_statusHigh.STK33	-1.4752241	5.78521241	-2.6293218	0.01040568	0.02751176	-3.534635	STK33	assignmentsChromaffin_cells.Chrom_SOX2_statusHigh
assignmentsChromaffin_cells.Chrom_SOX2_statusHigh.APPL1	0.4776695	5.9719449	2.61468829	0.01081624	0.02841455	-3.5357752	APPL1	assignmentsChromaffin_cells.Chrom_SOX2_statusHigh
assignmentsChromaffin_cells.Chrom_SOX2_statusHigh.GOLGA6L9	-1.0127456	2.45447952	-2.5112099	0.01421425	0.03562003	-3.5374902	GOLGA6L9	assignmentsChromaffin_cells.Chrom_SOX2_statusHigh
assignmentsChromaffin_cells.Chrom_SOX2_statusHigh.SCYL3	-0.5405555	4.43313959	-2.5584589	0.01255845	0.03216949	-3.5408838	SCYL3	assignmentsChromaffin_cells.Chrom_SOX2_statusHigh
assignmentsChromaffin_cells.Chrom_SOX2_statusHigh.MB21D2	-1.0742779	5.80065037	-2.5432345	0.01307263	0.03326862	-3.5411118	MB21D2	assignmentsChromaffin_cells.Chrom_SOX2_statusHigh
assignmentsChromaffin_cells.Chrom_SOX2_statusHigh.DUSP10	1.44316936	4.77453238	2.58551694	0.01169638	0.03033418	-3.541324	DUSP10	assignmentsChromaffin_cells.Chrom_SOX2_statusHigh
assignmentsChromaffin_cells.Chrom_SOX2_statusHigh.SMUG1	0.62319514	3.40051655	2.55591002	0.01264313	0.03233979	-3.5420631	SMUG1	assignmentsChromaffin_cells.Chrom_SOX2_statusHigh

assignmentsChromaffin_cells.Chrom_SOX2_statusHigh.PCDHA6	-1.1906638	0.2144982	-2.3725727	0.02026462	0.04817014	-3.542229	PCDHA6	assignmentsChromaffin_cells.Chrom_SOX2_statusHigh
assignmentsChromaffin_cells.Chrom_SOX2_statusHigh.UVSSA	-0.6583712	5.53156435	-2.596479	0.01135484	0.02955834	-3.5455167	UVSSA	assignmentsChromaffin_cells.Chrom_SOX2_statusHigh
assignmentsChromaffin_cells.Chrom_SOX2_statusHigh.ARHGAP6	-1.6266837	5.33623874	-2.3960505	0.01910328	0.04581017	-3.5458399	ARHGAP6	assignmentsChromaffin_cells.Chrom_SOX2_statusHigh
assignmentsChromaffin_cells.Chrom_SOX2_statusHigh.C1QC	2.16801015	2.48744868	2.43621399	0.01725528	0.04210899	-3.5461167	C1QC	assignmentsChromaffin_cells.Chrom_SOX2_statusHigh
assignmentsChromaffin_cells.Chrom_SOX2_statusHigh.UMAD1	-0.5802283	7.52912774	-2.6386807	0.01014198	0.02692997	-3.5520089	UMAD1	assignmentsChromaffin_cells.Chrom_SOX2_statusHigh
assignmentsChromaffin_cells.Chrom_SOX2_statusHigh.RMND5B	-0.5443016	4.78755092	-2.5769675	0.01195881	0.03089489	-3.5523722	RMND5B	assignmentsChromaffin_cells.Chrom_SOX2_statusHigh
assignmentsChromaffin_cells.Chrom_SOX2_statusHigh.CASC3	-0.3758986	5.88007147	-2.5973926	0.01132725	0.02950046	-3.55363	CASC3	assignmentsChromaffin_cells.Chrom_SOX2_statusHigh
assignmentsChromaffin_cells.Chrom_SOX2_statusHigh.CPSF7	-0.5175054	5.87839098	-2.5951534	0.01139498	0.02963878	-3.5541142	CPSF7	assignmentsChromaffin_cells.Chrom_SOX2_statusHigh
assignmentsChromaffin_cells.Chrom_SOX2_statusHigh.CC2D2B	-1.2722083	4.11816903	-2.4814885	0.01535593	0.03813539	-3.554226	CC2D2B	assignmentsChromaffin_cells.Chrom_SOX2_statusHigh
assignmentsChromaffin_cells.Chrom_SOX2_statusHigh.ALDH3A2	0.63929697	4.51240388	2.56710883	0.01227491	0.03156912	-3.5549024	ALDH3A2	assignmentsChromaffin_cells.Chrom_SOX2_statusHigh
assignmentsChromaffin_cells.Chrom_SOX2_statusHigh.GNAO1	-0.8523598	5.75430033	-2.6213582	0.01062483	0.02798966	-3.556026	GNAO1	assignmentsChromaffin_cells.Chrom_SOX2_statusHigh
assignmentsChromaffin_cells.Chrom_SOX2_statusHigh.OPTN	0.53970089	6.09145309	2.61176542	0.0109011	0.02858598	-3.5565268	OPTN	assignmentsChromaffin_cells.Chrom_SOX2_statusHigh
assignmentsChromaffin_cells.Chrom_SOX2_statusHigh.WIPI2	0.51959898	5.43277349	2.5946685	0.0114097	0.02966743	-3.5576457	WIPI2	assignmentsChromaffin_cells.Chrom_SOX2_statusHigh
assignmentsChromaffin_cells.Chrom_SOX2_statusHigh.DEPDC4	-0.8589345	1.86225115	-2.470307	0.01580365	0.03909593	-3.5599424	DEPDC4	assignmentsChromaffin_cells.Chrom_SOX2_statusHigh
assignmentsChromaffin_cells.Chrom_SOX2_statusHigh.TOP3A	-0.5030641	4.84300183	-2.5584576	0.0125585	0.03216949	-3.5636127	TOP3A	assignmentsChromaffin_cells.Chrom_SOX2_statusHigh
assignmentsChromaffin_cells.Chrom_SOX2_statusHigh.ARHGEF35	1.85269166	1.41445974	2.48129609	0.01536821	0.03815998	-3.5650492	ARHGEF35	assignmentsChromaffin_cells.Chrom_SOX2_statusHigh
assignmentsChromaffin_cells.Chrom_SOX2_statusHigh.CDK14	-0.901328	7.11768188	-2.6212604	0.01062896	0.02799134	-3.5653603	CDK14	assignmentsChromaffin_cells.Chrom_SOX2_statusHigh
assignmentsChromaffin_cells.Chrom_SOX2_statusHigh.INSR	-0.8922228	7.77378267	-2.62214	0.01060393	0.02794722	-3.5653715	INSR	assignmentsChromaffin_cells.Chrom_SOX2_statusHigh
assignmentsChromaffin_cells.Chrom_SOX2_statusHigh.SLC37A3	-0.5705859	5.02719192	-2.5732872	0.01207595	0.03115734	-3.5665813	SLC37A3	assignmentsChromaffin_cells.Chrom_SOX2_statusHigh
assignmentsChromaffin_cells.Chrom_SOX2_statusHigh.KCNIP2-AS1	-1.297164	1.51983887	-2.4420386	0.01699381	0.04157845	-3.5684441	KCNIP2-AS1	assignmentsChromaffin_cells.Chrom_SOX2_statusHigh
assignmentsChromaffin_cells.Chrom_SOX2_statusHigh.ECM2	-1.3301284	2.86832727	-2.451104	0.01660526	0.0408017	-3.5686533	ECM2	assignmentsChromaffin_cells.Chrom_SOX2_statusHigh
assignmentsChromaffin_cells.Chrom_SOX2_statusHigh.HEXD	-0.5356912	4.84722393	-2.5668047	0.01228478	0.03157932	-3.5689302	HEXD	assignmentsChromaffin_cells.Chrom_SOX2_statusHigh
assignmentsChromaffin_cells.Chrom_SOX2_statusHigh.DDIAS	1.01080285	0.66738124	2.39433205	0.01918425	0.04599058	-3.5704104	DDIAS	assignmentsChromaffin_cells.Chrom_SOX2_statusHigh
assignmentsChromaffin_cells.Chrom_SOX2_statusHigh.ZNF652	-0.5430536	6.43839917	-2.6037942	0.01113561	0.02910426	-3.5707364	ZNF652	assignmentsChromaffin_cells.Chrom_SOX2_statusHigh
assignmentsChromaffin_cells.Chrom_SOX2_statusHigh.ROCK2	-0.7565248	7.23588015	-2.5986334	0.01128987	0.02943085	-3.5716569	ROCK2	assignmentsChromaffin_cells.Chrom_SOX2_statusHigh
assignmentsChromaffin_cells.Chrom_SOX2_statusHigh.STAT1	1.06483991	6.54604925	2.59758245	0.01132387	0.02949688	-3.5724442	STAT1	assignmentsChromaffin_cells.Chrom_SOX2_statusHigh
assignmentsChromaffin_cells.Chrom_SOX2_statusHigh.ZNF529	-0.4326581	5.49553547	-2.5901133	0.01154878	0.02998048	-3.5731119	ZNF529	assignmentsChromaffin_cells.Chrom_SOX2_statusHigh
assignmentsChromaffin_cells.Chrom_SOX2_statusHigh.ABCD3	-0.462066	5.80657209	-2.5750233	0.01202056	0.03103941	-3.5765867	ABCD3	assignmentsChromaffin_cells.Chrom_SOX2_statusHigh
assignmentsChromaffin_cells.Chrom_SOX2_statusHigh.CASP7	0.53266789	4.04910258	2.53023413	0.01352526	0.03421422	-3.5772466	CASP7	assignmentsChromaffin_cells.Chrom_SOX2_statusHigh
assignmentsChromaffin_cells.Chrom_SOX2_statusHigh.YWHAE	0.76746477	7.06868429	2.62025897	0.01065616	0.02805836	-3.5780051	YWHAE	assignmentsChromaffin_cells.Chrom_SOX2_statusHigh
assignmentsChromaffin_cells.Chrom_SOX2_statusHigh.SLC29A3	1.13354705	2.19019748	2.41468007	0.01822074	0.0440694	-3.5781308	SLC29A3	assignmentsChromaffin_cells.Chrom_SOX2_statusHigh
assignmentsChromaffin_cells.Chrom_SOX2_statusHigh.NME7	-0.5975747	7.04441352	-2.6254579	0.0105087	0.02774754	-3.5800722	NME7	assignmentsChromaffin_cells.Chrom_SOX2_statusHigh
assignmentsChromaffin_cells.Chrom_SOX2_statusHigh.LRRC37A2	-0.8615847	4.89238984	-2.559976	0.0125083	0.03207164	-3.5802991	LRRC37A2	assignmentsChromaffin_cells.Chrom_SOX2_statusHigh
assignmentsChromaffin_cells.Chrom_SOX2_statusHigh.TMEM123	0.7918746	5.67648964	2.57622628	0.01198232	0.03094565	-3.5807175	TMEM123	assignmentsChromaffin_cells.Chrom_SOX2_statusHigh
assignmentsChromaffin_cells.Chrom_SOX2_statusHigh.RAB40C	-0.6163997	4.05060312	-2.5335685	0.01340765	0.0339864	-3.5812213	RAB40C	assignmentsChromaffin_cells.Chrom_SOX2_statusHigh
assignmentsChromaffin_cells.Chrom_SOX2_statusHigh.MIER3	-0.4704587	4.56065876	-2.5542715	0.01269783	0.032459	-3.5841591	MIER3	assignmentsChromaffin_cells.Chrom_SOX2_statusHigh
assignmentsChromaffin_cells.Chrom_SOX2_statusHigh.GTPBP4	0.49314621	5.13641741	2.5774471	0.01194363	0.03086066	-3.5844212	GTPBP4	assignmentsChromaffin_cells.Chrom_SOX2_statusHigh
assignmentsChromaffin_cells.Chrom_SOX2_statusHigh.CRPPA	-0.8884176	5.0505084	-2.5636466	0.0123877	0.03181838	-3.5849346	CRPPA	assignmentsChromaffin_cells.Chrom_SOX2_statusHigh
assignmentsChromaffin_cells.Chrom_SOX2_statusHigh.ZNF471	-0.6506786	4.15535583	-2.5529056	0.01274359	0.03254486	-3.5854375	ZNF471	assignmentsChromaffin_cells.Chrom_SOX2_statusHigh
assignmentsChromaffin_cells.Chrom_SOX2_statusHigh.ARRB2	0.87903504	4.22452636	2.52899223	0.0135693	0.03429318	-3.5867902	ARRB2	assignmentsChromaffin_cells.Chrom_SOX2_statusHigh
assignmentsChromaffin_cells.Chrom_SOX2_statusHigh.ZBTB26	-0.6456323	3.51249353	-2.5223432	0.01380731	0.03474797	-3.5879195	ZBTB26	assignmentsChromaffin_cells.Chrom_SOX2_statusHigh
assignmentsChromaffin_cells.Chrom_SOX2_statusHigh.GTF3C3	-0.4774504	5.28578372	-2.5720637	0.01211511	0.03124001	-3.5903726	GTF3C3	assignmentsChromaffin_cells.Chrom_SOX2_statusHigh
assignmentsChromaffin_cells.Chrom_SOX2_statusHigh.CLNS1A	0.50573497	5.79564474	2.59132909	0.01151151	0.0298934	-3.5916994	CLNS1A	assignmentsChromaffin_cells.Chrom_SOX2_statusHigh
assignmentsChromaffin_cells.Chrom_SOX2_statusHigh.GBP2	1.5990723	4.26945737	2.41822992	0.01806018	0.04376026	-3.5925631	GBP2	assignmentsChromaffin_cells.Chrom_SOX2_statusHigh
assignmentsChromaffin_cells.Chrom_SOX2_statusHigh.MINK1	-0.8207988	5.28419932	-2.5421594	0.01310887	0.03333969	-3.5934199	MINK1	assignmentsChromaffin_cells.Chrom_SOX2_statusHigh
assignmentsChromaffin_cells.Chrom_SOX2_statusHigh.GOLPH3	-0.5312239	6.76375135	-2.5973369	0.01132893	0.02950046	-3.5934995	GOLPH3	assignmentsChromaffin_cells.Chrom_SOX2_statusHigh
assignmentsChromaffin_cells.Chrom_SOX2_statusHigh.NBPF9	-0.9786011	4.36098913	-2.5343568	0.01337999	0.03393237	-3.5949025	NBPF9	assignmentsChromaffin_cells.Chrom_SOX2_statusHigh
assignmentsChromaffin_cells.Chrom_SOX2_statusHigh.LRFN1	1.26946302	0.75728247	2.40193507	0.01881904	0.04522322	-3.595519	LRFN1	assignmentsChromaffin_cells.Chrom_SOX2_statusHigh
assignmentsChromaffin_cells.Chrom_SOX2_statusHigh.CHGA	3.2627269	9.06362631	2.67922474	0.00909383	0.02463629	-3.5958108	CHGA	assignmentsChromaffin_cells.Chrom_SOX2_statusHigh
assignmentsChromaffin_cells.Chrom_SOX2_statusHigh.EEA1	-0.4265927	7.29838873	-2.6051459	0.01109553	0.02902937	-3.5962229	EEA1	assignmentsChromaffin_cells.Chrom_SOX2_statusHigh
assignmentsChromaffin_cells.Chrom_SOX2_statusHigh.ZNF394	0.48360856	4.91806356	2.55725227	0.01259847	0.03224099	-3.5967272	ZNF394	assignmentsChromaffin_cells.Chrom_SOX2_statusHigh
assignmentsChromaffin_cells.Chrom_SOX2_statusHigh.CRB1	-1.4744215	2.81494878	-2.4888308	0.01507075	0.03750266	-3.5977005	CRB1	assignmentsChromaffin_cells.Chrom_SOX2_statusHigh
assignmentsChromaffin_cells.Chrom_SOX2_statusHigh.CLSTN1	0.54340195	5.7976292	2.58492236	0.01170915	0.03036238	-3.5978284	CLSTN1	assignmentsChromaffin_cells.Chrom_SOX2_statusHigh
assignmentsChromaffin_cells.Chrom_SOX2_statusHigh.SFTPD	-1.274838	0.91646465	-2.4329016	0.0173952	0.04237305	-3.599766	SFTPD	assignmentsChromaffin_cells.Chrom_SOX2_statusHigh
assignmentsChromaffin_cells.Chrom_SOX2_statusHigh.ZNF433	-0.6747844	3.44093933	-2.5128654	0.01415306	0.03549997	-3.6003172	ZNF433	assignmentsChromaffin_cells.Chrom_SOX2_statusHigh
assignmentsChromaffin_cells.Chrom_SOX2_statusHigh.U2AF1	2.1815046	5.048441	2.57079642	0.01216162	0.03133807	-3.6004392	U2AF1	assignmentsChromaffin_cells.Chrom_SOX2_statusHigh
assignmentsChromaffin_cells.Chrom_SOX2_statusHigh.ZKSCAN7-A	-0.8723569	4.06114626	-2.5418781	0.01311856	0.03335904	-3.6004956	ZKSCAN7-AS	assignmentsChromaffin_cells.Chrom_SOX2_statusHigh
assignmentsChromaffin_cells.Chrom_SOX2_statusHigh.TMEM238	1.52145294	0.89698094	2.43909951	0.01712492	0.04183542	-3.6019932	TMEM238	assignmentsChromaffin_cells.Chrom_SOX2_statusHigh
assignmentsChromaffin_cells.Chrom_SOX2_statusHigh.SUGP2	-0.5538491	6.83679043	-2.6000438	0.01124752	0.02934089	-3.603617	SUGP2	assignmentsChromaffin_cells.Chrom_SOX2_statusHigh
assignmentsChromaffin_cells.Chrom_SOX2_statusHigh.CCZ1B	-0.5846969	4.63220475	-2.5452589	0.01300256	0.0331113	-3.6049084	CCZ1B	assignmentsChromaffin_cells.Chrom_SOX2_statusHigh
assignmentsChromaffin_cells.Chrom_SOX2_statusHigh.ENTR1	-0.6606596	3.97416351	-2.5078653	0.0143386	0.03589819	-3.6068774	ENTR1	assignmentsChromaffin_cells.Chrom_SOX2_statusHigh
assignmentsChromaffin_cells.Chrom_SOX2_statusHigh.HIP1R	-0.9640521	2.88155429	-2.4631803	0.01609635	0.03971589	-3.6074985	HIP1R	assignmentsChromaffin_cells.Chrom_SOX2_statusHigh
assignmentsChromaffin_cells.Chrom_SOX2_statusHigh.JUND	1.76066985	7.02399063	2.60835635	0.01100637	0.02883374	-3.607569	JUND	assignmentsChromaffin_cells.Chrom_SOX2_statusHigh
assignmentsChromaffin_cells.Chrom_SOX2_statusHigh.POMGNT1	0.52496458	3.26400536	2.51484817	0.01408009	0.03533905	-3.6094107	POMGNT1	assignmentsChromaffin_cells.Chrom_SOX2_statusHigh

assignmentsChromaffin_cells.Chrom_SOX2_statusHigh.ZNF839	-0.7599797	3.9307834	-2.5239345	0.01375	0.03463524	-3.609596	ZNF839	assignmentsChromaffin_cells.Chrom_SOX2_statusHigh
assignmentsChromaffin_cells.Chrom_SOX2_statusHigh.MCCC1	-0.5924326	5.47577036	-2.5687919	0.01222042	0.0314542	-3.6102486	MCCC1	assignmentsChromaffin_cells.Chrom_SOX2_statusHigh
assignmentsChromaffin_cells.Chrom_SOX2_statusHigh.EVA1C	-0.9563647	6.49626434	-2.5798435	0.01186966	0.03070414	-3.6105932	EVA1C	assignmentsChromaffin_cells.Chrom_SOX2_statusHigh
assignmentsChromaffin_cells.Chrom_SOX2_statusHigh.TTF1	-0.4300616	5.01107767	-2.5457425	0.01298604	0.03307975	-3.6111685	TTF1	assignmentsChromaffin_cells.Chrom_SOX2_statusHigh
assignmentsChromaffin_cells.Chrom_SOX2_statusHigh.SCCPDH	0.60845625	4.96012928	2.5722121	0.01211036	0.03123607	-3.6117142	SCCPDH	assignmentsChromaffin_cells.Chrom_SOX2_statusHigh
assignmentsChromaffin_cells.Chrom_SOX2_statusHigh.LRRC49	-0.986037	5.5227046	-2.5813761	0.01182154	0.03060802	-3.6122182	LRRC49	assignmentsChromaffin_cells.Chrom_SOX2_statusHigh
assignmentsChromaffin_cells.Chrom_SOX2_statusHigh.ELAVL2	-1.2596063	3.86271043	-2.547168	0.01294234	0.03299462	-3.6123397	ELAVL2	assignmentsChromaffin_cells.Chrom_SOX2_statusHigh
assignmentsChromaffin_cells.Chrom_SOX2_statusHigh.MIR3681HG	1.24412604	1.86258424	2.44227756	0.01698402	0.04156587	-3.613058	MIR3681HG	assignmentsChromaffin_cells.Chrom_SOX2_statusHigh
assignmentsChromaffin_cells.Chrom_SOX2_statusHigh.HCFC1	0.52307613	4.12922493	2.53925487	0.01320921	0.03355762	-3.6134639	HCFC1	assignmentsChromaffin_cells.Chrom_SOX2_statusHigh
assignmentsChromaffin_cells.Chrom_SOX2_statusHigh.PTPN18	-0.5939395	4.7850606	-2.5244197	0.01373258	0.03460221	-3.6134877	PTPN18	assignmentsChromaffin_cells.Chrom_SOX2_statusHigh
assignmentsChromaffin_cells.Chrom_SOX2_statusHigh.MAP3K9	-0.8326823	3.97789751	-2.5458861	0.01298114	0.03307252	-3.6143159	MAP3K9	assignmentsChromaffin_cells.Chrom_SOX2_statusHigh
assignmentsChromaffin_cells.Chrom_SOX2_statusHigh.MFSD10	0.922764	4.25165675	2.54146773	0.0131327	0.03338971	-3.6154028	MFSD10	assignmentsChromaffin_cells.Chrom_SOX2_statusHigh
assignmentsChromaffin_cells.Chrom_SOX2_statusHigh.CTSO	0.84397076	3.25983451	2.48796315	0.01509897	0.03756123	-3.6154948	CTSO	assignmentsChromaffin_cells.Chrom_SOX2_statusHigh
assignmentsChromaffin_cells.Chrom_SOX2_statusHigh.NEURL2	1.23421886	1.81630013	2.44923029	0.01668493	0.04095634	-3.6161771	NEURL2	assignmentsChromaffin_cells.Chrom_SOX2_statusHigh
assignmentsChromaffin_cells.Chrom_SOX2_statusHigh.MT-CO2	2.36152537	9.7823695	2.661385	0.00954444	0.02564006	-3.6171473	MT-CO2	assignmentsChromaffin_cells.Chrom_SOX2_statusHigh
assignmentsChromaffin_cells.Chrom_SOX2_statusHigh.CHMP2B	0.71746989	4.82736534	2.55546068	0.01265811	0.03236778	-3.6175619	CHMP2B	assignmentsChromaffin_cells.Chrom_SOX2_statusHigh
assignmentsChromaffin_cells.Chrom_SOX2_statusHigh.PASK	-0.9843096	2.33154587	-2.4091662	0.01847747	0.04455595	-3.6176887	PASK	assignmentsChromaffin_cells.Chrom_SOX2_statusHigh
assignmentsChromaffin_cells.Chrom_SOX2_statusHigh.MIS12	0.60763249	3.93456937	2.52790792	0.01360786	0.03436896	-3.6182551	MIS12	assignmentsChromaffin_cells.Chrom_SOX2_statusHigh
assignmentsChromaffin_cells.Chrom_SOX2_statusHigh.TNFRSF1A	0.85601141	5.39996236	2.54629312	0.01296726	0.0330424	-3.6192348	TNFRSF1A	assignmentsChromaffin_cells.Chrom_SOX2_statusHigh
assignmentsChromaffin_cells.Chrom_SOX2_statusHigh.MFHAS1	-0.8833463	5.49364811	-2.5309643	0.01349943	0.03417043	-3.6200914	MFHAS1	assignmentsChromaffin_cells.Chrom_SOX2_statusHigh
assignmentsChromaffin_cells.Chrom_SOX2_statusHigh.ZMAT1	-0.5545959	5.72755734	-2.5740867	0.01205041	0.03110147	-3.620601	ZMAT1	assignmentsChromaffin_cells.Chrom_SOX2_statusHigh
assignmentsChromaffin_cells.Chrom_SOX2_statusHigh.PCK2	0.7217899	2.98276729	2.50672785	0.01438111	0.03598194	-3.6225017	PCK2	assignmentsChromaffin_cells.Chrom_SOX2_statusHigh
assignmentsChromaffin_cells.Chrom_SOX2_statusHigh.EDRF1	-0.4204344	5.63744937	-2.5624256	0.0124277	0.03191089	-3.6244193	EDRF1	assignmentsChromaffin_cells.Chrom_SOX2_statusHigh
assignmentsChromaffin_cells.Chrom_SOX2_statusHigh.AACS	-0.6064412	4.17639663	-2.5280661	0.01360223	0.03436896	-3.629601	AACS	assignmentsChromaffin_cells.Chrom_SOX2_statusHigh
assignmentsChromaffin_cells.Chrom_SOX2_statusHigh.GTF3C1	-0.5368824	5.93077982	-2.5695578	0.01219569	0.03141072	-3.6323726	GTF3C1	assignmentsChromaffin_cells.Chrom_SOX2_statusHigh
assignmentsChromaffin_cells.Chrom_SOX2_statusHigh.LINC01655	1.64631103	0.06937671	2.37747678	0.0200196	0.04764422	-3.6340501	LINC01655	assignmentsChromaffin_cells.Chrom_SOX2_statusHigh
assignmentsChromaffin_cells.Chrom_SOX2_statusHigh.DUS3L	0.67742005	3.38310696	2.51631145	0.01402646	0.03521546	-3.6347795	DUS3L	assignmentsChromaffin_cells.Chrom_SOX2_statusHigh
assignmentsChromaffin_cells.Chrom_SOX2_statusHigh.FIZ1	0.53226847	2.30329163	2.47519001	0.01560587	0.03867226	-3.6348395	FIZ1	assignmentsChromaffin_cells.Chrom_SOX2_statusHigh
assignmentsChromaffin_cells.Chrom_SOX2_statusHigh.RBL2	-0.4562264	6.26779232	-2.5677292	0.0122548	0.03153763	-3.6353307	RBL2	assignmentsChromaffin_cells.Chrom_SOX2_statusHigh
assignmentsChromaffin_cells.Chrom_SOX2_statusHigh.LINC02175	-0.8732613	2.87514965	-2.4515013	0.01658654	0.04078108	-3.6359421	LINC02175	assignmentsChromaffin_cells.Chrom_SOX2_statusHigh
assignmentsChromaffin_cells.Chrom_SOX2_statusHigh.RUNDC3A	0.88548341	3.54647348	2.55764572	0.01258542	0.03221273	-3.6360358	RUNDC3A	assignmentsChromaffin_cells.Chrom_SOX2_statusHigh
assignmentsChromaffin_cells.Chrom_SOX2_statusHigh.LSM12	0.53152967	4.89261439	2.54694538	0.01294504	0.03299627	-3.6382799	LSM12	assignmentsChromaffin_cells.Chrom_SOX2_statusHigh
assignmentsChromaffin_cells.Chrom_SOX2_statusHigh.TET3	-0.7462745	6.38829862	-2.5577218	0.01258289	0.03221273	-3.6387417	TET3	assignmentsChromaffin_cells.Chrom_SOX2_statusHigh
assignmentsChromaffin_cells.Chrom_SOX2_statusHigh.CDC42SE2	-0.6538095	6.95350543	-2.5576498	0.01258528	0.03221273	-3.6396813	CDC42SE2	assignmentsChromaffin_cells.Chrom_SOX2_statusHigh
assignmentsChromaffin_cells.Chrom_SOX2_statusHigh.BEX4	1.45463677	4.66438884	2.56127812	0.01247129	0.03200746	-3.6398829	BEX4	assignmentsChromaffin_cells.Chrom_SOX2_statusHigh
assignmentsChromaffin_cells.Chrom_SOX2_statusHigh.YEATS2	-0.7396587	6.71864665	-2.5744749	0.01203803	0.03107452	-3.6409737	YEATS2	assignmentsChromaffin_cells.Chrom_SOX2_statusHigh
assignmentsChromaffin_cells.Chrom_SOX2_statusHigh.MRPS27	-0.4883766	5.51073516	-2.5498773	0.01284559	0.03276881	-3.6411364	MRPS27	assignmentsChromaffin_cells.Chrom_SOX2_statusHigh
assignmentsChromaffin_cells.Chrom_SOX2_statusHigh.DCAF10	-0.5274164	6.63344911	-2.5812772	0.01182296	0.03060802	-3.6417596	DCAF10	assignmentsChromaffin_cells.Chrom_SOX2_statusHigh
assignmentsChromaffin_cells.Chrom_SOX2_statusHigh.ZNF84	-0.471373	5.61005691	-2.5603079	0.01249735	0.03205382	-3.6433763	ZNF84	assignmentsChromaffin_cells.Chrom_SOX2_statusHigh
assignmentsChromaffin_cells.Chrom_SOX2_statusHigh.NPL	-1.3829743	4.36349313	-2.4238386	0.01780397	0.04320474	-3.6438274	NPL	assignmentsChromaffin_cells.Chrom_SOX2_statusHigh
assignmentsChromaffin_cells.Chrom_SOX2_statusHigh.OIP5-AS1	-0.455934	6.15567654	-2.5675174	0.01226166	0.03155023	-3.6440457	OIP5-AS1	assignmentsChromaffin_cells.Chrom_SOX2_statusHigh
assignmentsChromaffin_cells.Chrom_SOX2_statusHigh.RERGL	1.82098582	2.0937687	2.50814968	0.01433431	0.03589819	-3.6443043	RERGL	assignmentsChromaffin_cells.Chrom_SOX2_statusHigh
assignmentsChromaffin_cells.Chrom_SOX2_statusHigh.GOLGA1	-0.611551	5.46550049	-2.5430966	0.01307664	0.03327355	-3.6458703	GOLGA1	assignmentsChromaffin_cells.Chrom_SOX2_statusHigh
assignmentsChromaffin_cells.Chrom_SOX2_statusHigh.ST3GAL2	-0.8203933	4.95579689	-2.5196678	0.01390413	0.0349442	-3.6464	ST3GAL2	assignmentsChromaffin_cells.Chrom_SOX2_statusHigh
assignmentsChromaffin_cells.Chrom_SOX2_statusHigh.TMEM214	0.52878735	4.1731633	2.52485133	0.01371709	0.0345696	-3.6497706	TMEM214	assignmentsChromaffin_cells.Chrom_SOX2_statusHigh
assignmentsChromaffin_cells.Chrom_SOX2_statusHigh.TRAP1	-0.5058238	5.35376567	-2.5510508	0.01280598	0.0326855	-3.649772	TRAP1	assignmentsChromaffin_cells.Chrom_SOX2_statusHigh
assignmentsChromaffin_cells.Chrom_SOX2_statusHigh.SNHG19	0.80682199	3.32862466	2.51359011	0.01412635	0.03543852	-3.6503397	SNHG19	assignmentsChromaffin_cells.Chrom_SOX2_statusHigh
assignmentsChromaffin_cells.Chrom_SOX2_statusHigh.CDC23	0.39402389	4.09815147	2.52484048	0.01371748	0.0345696	-3.6504522	CDC23	assignmentsChromaffin_cells.Chrom_SOX2_statusHigh
assignmentsChromaffin_cells.Chrom_SOX2_statusHigh.DDR1	1.02394017	2.50050416	2.49238366	0.01492703	0.03717386	-3.6509146	DDR1	assignmentsChromaffin_cells.Chrom_SOX2_statusHigh
assignmentsChromaffin_cells.Chrom_SOX2_statusHigh.PUDP	-0.7142881	5.50350887	-2.5378366	0.01325845	0.03365074	-3.6514637	PUDP	assignmentsChromaffin_cells.Chrom_SOX2_statusHigh
assignmentsChromaffin_cells.Chrom_SOX2_statusHigh.NSD3	-0.4544302	7.95315138	-2.6001947	0.011243	0.02933863	-3.653025	NSD3	assignmentsChromaffin_cells.Chrom_SOX2_statusHigh
assignmentsChromaffin_cells.Chrom_SOX2_statusHigh.HSPA9	0.76743369	7.24182313	2.60123124	0.01121198	0.02927198	-3.6533111	HSPA9	assignmentsChromaffin_cells.Chrom_SOX2_statusHigh
assignmentsChromaffin_cells.Chrom_SOX2_statusHigh.PDE4C	1.71526797	1.64595879	2.3880813	0.01949515	0.04660354	-3.6548871	PDE4C	assignmentsChromaffin_cells.Chrom_SOX2_statusHigh
assignmentsChromaffin_cells.Chrom_SOX2_statusHigh.ARL9	1.35079776	1.33411009	2.4262598	0.01769455	0.04298036	-3.6574092	ARL9	assignmentsChromaffin_cells.Chrom_SOX2_statusHigh
assignmentsChromaffin_cells.Chrom_SOX2_statusHigh.AGBL4	-1.5376159	7.33764487	-2.6039718	0.0111359	0.02910426	-3.6590552	AGBL4	assignmentsChromaffin_cells.Chrom_SOX2_statusHigh
assignmentsChromaffin_cells.Chrom_SOX2_statusHigh.MAP3K2	-0.64112	7.50388464	-2.5783411	0.01191536	0.03080745	-3.6595104	MAP3K2	assignmentsChromaffin_cells.Chrom_SOX2_statusHigh
assignmentsChromaffin_cells.Chrom_SOX2_statusHigh.ZNF687	0.5987005	2.49092481	2.46338544	0.01608786	0.03970388	-3.6599083	ZNF687	assignmentsChromaffin_cells.Chrom_SOX2_statusHigh
assignmentsChromaffin_cells.Chrom_SOX2_statusHigh.SETDB1	-0.5664576	5.2854577	-2.5354257	0.01334255	0.03384276	-3.6608538	SETDB1	assignmentsChromaffin_cells.Chrom_SOX2_statusHigh
assignmentsChromaffin_cells.Chrom_SOX2_statusHigh.ACAA1	0.76030387	4.11959917	2.50777718	0.01434189	0.03590061	-3.661313	ACAA1	assignmentsChromaffin_cells.Chrom_SOX2_statusHigh
assignmentsChromaffin_cells.Chrom_SOX2_statusHigh.CEBPZ	0.67373028	6.19466996	2.56971314	0.01219069	0.03140286	-3.6618078	CEBPZ	assignmentsChromaffin_cells.Chrom_SOX2_statusHigh
assignmentsChromaffin_cells.Chrom_SOX2_statusHigh.ACAD11	-0.596178	5.71708942	-2.5471257	0.0129389	0.03299111	-3.6619576	ACAD11	assignmentsChromaffin_cells.Chrom_SOX2_statusHigh
assignmentsChromaffin_cells.Chrom_SOX2_statusHigh.UBR4	-0.70821	6.81413425	-2.5564176	0.01262622	0.0323017	-3.663461	UBR4	assignmentsChromaffin_cells.Chrom_SOX2_statusHigh
assignmentsChromaffin_cells.Chrom_SOX2_statusHigh.ZNF804A	-2.2926426	5.15143418	-2.5281056	0.01360699	0.03436896	-3.6642836	ZNF804A	assignmentsChromaffin_cells.Chrom_SOX2_statusHigh
assignmentsChromaffin_cells.Chrom_SOX2_statusHigh.DUSP19	0.89259917	1.08359728	2.40325006	0.01875651	0.04511173	-3.6649705	DUSP19	assignmentsChromaffin_cells.Chrom_SOX2_statusHigh

assignmentsChromaffin_cells.Chrom_SOX2_statusHigh.DACH2	2.28468778	1.65939832	2.4716628	0.01575512	0.03899393	-3.6670543	DACH2	assignmentsChromaffin_cells.Chrom_SOX2_statusHigh
assignmentsChromaffin_cells.Chrom_SOX2_statusHigh.MEAK7	-0.6191287	3.98706587	-2.4924969	0.01492264	0.03717337	-3.669425	MEAK7	assignmentsChromaffin_cells.Chrom_SOX2_statusHigh
assignmentsChromaffin_cells.Chrom_SOX2_statusHigh.TRIM68	0.83374155	1.46861391	2.39340314	0.0192293	0.04605039	-3.6700958	TRIM68	assignmentsChromaffin_cells.Chrom_SOX2_statusHigh
assignmentsChromaffin_cells.Chrom_SOX2_statusHigh.ATP7A	0.83700025	4.82099095	2.53684529	0.01329297	0.03372768	-3.6728228	ATP7A	assignmentsChromaffin_cells.Chrom_SOX2_statusHigh
assignmentsChromaffin_cells.Chrom_SOX2_statusHigh.CBL	-0.7525919	6.26162115	-2.5217268	0.01382956	0.03479738	-3.6734543	CBL	assignmentsChromaffin_cells.Chrom_SOX2_statusHigh
assignmentsChromaffin_cells.Chrom_SOX2_statusHigh.PSIP1	0.5105242	6.76014146	2.58308956	0.01176625	0.03049073	-3.674751	PSIP1	assignmentsChromaffin_cells.Chrom_SOX2_statusHigh
assignmentsChromaffin_cells.Chrom_SOX2_statusHigh.PPP2R2A	-0.4984758	7.42609992	-2.571462	0.01213442	0.03128304	-3.6773521	PPP2R2A	assignmentsChromaffin_cells.Chrom_SOX2_statusHigh
assignmentsChromaffin_cells.Chrom_SOX2_statusHigh.EDEM2	0.72070703	4.07290819	2.4850773	0.01521218	0.0378194	-3.6775762	EDEM2	assignmentsChromaffin_cells.Chrom_SOX2_statusHigh
assignmentsChromaffin_cells.Chrom_SOX2_statusHigh.BZW1	0.92286097	5.79283302	2.54415541	0.01304131	0.03319419	-3.677961	BZW1	assignmentsChromaffin_cells.Chrom_SOX2_statusHigh
assignmentsChromaffin_cells.Chrom_SOX2_statusHigh.SP110	1.31603772	5.16201271	2.46972112	0.01583137	0.0391464	-3.6799335	SP110	assignmentsChromaffin_cells.Chrom_SOX2_statusHigh
assignmentsChromaffin_cells.Chrom_SOX2_statusHigh.EVI5	-0.6474252	7.39698015	-2.5653056	0.01233354	0.03169957	-3.681491	EVI5	assignmentsChromaffin_cells.Chrom_SOX2_statusHigh
assignmentsChromaffin_cells.Chrom_SOX2_statusHigh.ASCC1	-0.5510724	5.59489097	-2.5234404	0.01376777	0.03466911	-3.6816436	ASCC1	assignmentsChromaffin_cells.Chrom_SOX2_statusHigh
assignmentsChromaffin_cells.Chrom_SOX2_statusHigh.PPP1R2	0.73299801	5.88091936	2.53974931	0.01319208	0.033522	-3.6819392	PPP1R2	assignmentsChromaffin_cells.Chrom_SOX2_statusHigh
assignmentsChromaffin_cells.Chrom_SOX2_statusHigh.TNNI3K	-1.0253169	3.90372577	-2.4805171	0.01539264	0.03821362	-3.6839588	TNNI3K	assignmentsChromaffin_cells.Chrom_SOX2_statusHigh
assignmentsChromaffin_cells.Chrom_SOX2_statusHigh.RASSF4	1.27542345	4.77019937	2.4923246	0.01493413	0.03718578	-3.6864457	RASSF4	assignmentsChromaffin_cells.Chrom_SOX2_statusHigh
assignmentsChromaffin_cells.Chrom_SOX2_statusHigh.SHOX2	2.04699235	0.94369553	2.39719286	0.01905332	0.04572455	-3.6872763	SHOX2	assignmentsChromaffin_cells.Chrom_SOX2_statusHigh
assignmentsChromaffin_cells.Chrom_SOX2_statusHigh.RHCE	0.82164336	1.24247137	2.41283045	0.0183065	0.04422214	-3.687325	RHCE	assignmentsChromaffin_cells.Chrom_SOX2_statusHigh
assignmentsChromaffin_cells.Chrom_SOX2_statusHigh.ZNF500	-0.5827036	3.06955283	-2.4589142	0.01627388	0.0400984	-3.6883979	ZNF500	assignmentsChromaffin_cells.Chrom_SOX2_statusHigh
assignmentsChromaffin_cells.Chrom_SOX2_statusHigh.SMS	0.79982351	5.75706577	2.53971989	0.0131931	0.033522	-3.689704	SMS	assignmentsChromaffin_cells.Chrom_SOX2_statusHigh
assignmentsChromaffin_cells.Chrom_SOX2_statusHigh.ADAM22	-0.8168793	5.41165077	-2.5500886	0.01283845	0.03275581	-3.6912976	ADAM22	assignmentsChromaffin_cells.Chrom_SOX2_statusHigh
assignmentsChromaffin_cells.Chrom_SOX2_statusHigh.ASB3	-0.625522	6.65817971	-2.5564586	0.01262486	0.0323017	-3.6914855	ASB3	assignmentsChromaffin_cells.Chrom_SOX2_statusHigh
assignmentsChromaffin_cells.Chrom_SOX2_statusHigh.AGAP6	2.45008386	2.26220508	2.42610397	0.01770595	0.04299939	-3.6915242	AGAP6	assignmentsChromaffin_cells.Chrom_SOX2_statusHigh
assignmentsChromaffin_cells.Chrom_SOX2_statusHigh.DDX25	0.90950432	1.57578747	2.45446315	0.01646098	0.04050961	-3.6926666	DDX25	assignmentsChromaffin_cells.Chrom_SOX2_statusHigh
assignmentsChromaffin_cells.Chrom_SOX2_statusHigh.FNBP4	-0.618537	7.59547494	-2.5630411	0.01240752	0.03186419	-3.6967554	FNBP4	assignmentsChromaffin_cells.Chrom_SOX2_statusHigh
assignmentsChromaffin_cells.Chrom_SOX2_statusHigh.NRSN2	0.60674544	3.51540205	2.50554079	0.01442559	0.03607636	-3.6984709	NRSN2	assignmentsChromaffin_cells.Chrom_SOX2_statusHigh
assignmentsChromaffin_cells.Chrom_SOX2_statusHigh.HPS1	2.04991013	3.39373489	2.37181269	0.02031075	0.0482655	-3.6997271	HPS1	assignmentsChromaffin_cells.Chrom_SOX2_statusHigh
assignmentsChromaffin_cells.Chrom_SOX2_statusHigh.CITED2	1.92501559	4.75687648	2.5204301	0.0138827	0.03490917	-3.7019978	CITED2	assignmentsChromaffin_cells.Chrom_SOX2_statusHigh
assignmentsChromaffin_cells.Chrom_SOX2_statusHigh.CCDC24	0.64732077	2.6065208	2.46123074	0.01617726	0.03988485	-3.7039591	CCDC24	assignmentsChromaffin_cells.Chrom_SOX2_statusHigh
assignmentsChromaffin_cells.Chrom_SOX2_statusHigh.IRGM	-1.1724992	2.07724927	-2.4201435	0.01797047	0.04356921	-3.7044074	IRGM	assignmentsChromaffin_cells.Chrom_SOX2_statusHigh
assignmentsChromaffin_cells.Chrom_SOX2_statusHigh.SIAE	0.67474257	3.25712575	2.474785	0.01562219	0.03870673	-3.7071175	SIAE	assignmentsChromaffin_cells.Chrom_SOX2_statusHigh
assignmentsChromaffin_cells.Chrom_SOX2_statusHigh.PCAT1	-1.7946202	4.20929314	-2.4253975	0.01773783	0.0430703	-3.7080155	PCAT1	assignmentsChromaffin_cells.Chrom_SOX2_statusHigh
assignmentsChromaffin_cells.Chrom_SOX2_statusHigh.INTS10	-0.5169394	6.24847373	-2.5426975	0.01309036	0.03329788	-3.7096372	INTS10	assignmentsChromaffin_cells.Chrom_SOX2_statusHigh
assignmentsChromaffin_cells.Chrom_SOX2_statusHigh.ASB6	0.6688203	2.3546284	2.43259226	0.0174086	0.04239691	-3.7097397	ASB6	assignmentsChromaffin_cells.Chrom_SOX2_statusHigh
assignmentsChromaffin_cells.Chrom_SOX2_statusHigh.ACAT1	0.58154227	5.15058089	2.53299944	0.01342766	0.03402635	-3.7107302	ACAT1	assignmentsChromaffin_cells.Chrom_SOX2_statusHigh
assignmentsChromaffin_cells.Chrom_SOX2_statusHigh.PLCG1-AS1	-0.7102438	2.94604882	-2.412827	0.01830666	0.04422214	-3.7114659	PLCG1-AS1	assignmentsChromaffin_cells.Chrom_SOX2_statusHigh
assignmentsChromaffin_cells.Chrom_SOX2_statusHigh.STMP1	0.5256731	5.55881794	2.53741179	0.01327324	0.03368294	-3.7139685	STMP1	assignmentsChromaffin_cells.Chrom_SOX2_statusHigh
assignmentsChromaffin_cells.Chrom_SOX2_statusHigh.PTBP3	-0.7716752	7.31727908	-2.5522983	0.01276399	0.03259176	-3.7150248	PTBP3	assignmentsChromaffin_cells.Chrom_SOX2_statusHigh
assignmentsChromaffin_cells.Chrom_SOX2_statusHigh.CYFIP2	-0.8157104	5.16790006	-2.5203752	0.01387847	0.03490401	-3.7158482	CYFIP2	assignmentsChromaffin_cells.Chrom_SOX2_statusHigh
assignmentsChromaffin_cells.Chrom_SOX2_statusHigh.LONRF3	2.30112212	3.26264786	2.4048969	0.01868562	0.04495649	-3.7160656	LONRF3	assignmentsChromaffin_cells.Chrom_SOX2_statusHigh
assignmentsChromaffin_cells.Chrom_SOX2_statusHigh.MAMSTR	0.91980015	2.02531833	2.43483695	0.01730914	0.04221472	-3.7160952	MAMSTR	assignmentsChromaffin_cells.Chrom_SOX2_statusHigh
assignmentsChromaffin_cells.Chrom_SOX2_statusHigh.ZNF235	-0.610465	5.688814	-2.5279658	0.0136058	0.03436896	-3.7178202	ZNF235	assignmentsChromaffin_cells.Chrom_SOX2_statusHigh
assignmentsChromaffin_cells.Chrom_SOX2_statusHigh.MGME1	0.61672154	4.28717922	2.47657603	0.01555013	0.03857588	-3.7186112	MGME1	assignmentsChromaffin_cells.Chrom_SOX2_statusHigh
assignmentsChromaffin_cells.Chrom_SOX2_statusHigh.PAPOLG	-0.557833	5.46404166	-2.49977	0.01464362	0.03659309	-3.7192902	PAPOLG	assignmentsChromaffin_cells.Chrom_SOX2_statusHigh
assignmentsChromaffin_cells.Chrom_SOX2_statusHigh.OMD	-1.4289084	1.79334506	-2.3616278	0.02083058	0.04926719	-3.7196735	OMD	assignmentsChromaffin_cells.Chrom_SOX2_statusHigh
assignmentsChromaffin_cells.Chrom_SOX2_statusHigh.OAT	0.78675721	5.36284752	2.52999276	0.01353381	0.03422714	-3.7197393	OAT	assignmentsChromaffin_cells.Chrom_SOX2_statusHigh
assignmentsChromaffin_cells.Chrom_SOX2_statusHigh.TMEM170B	-0.6545137	3.96886486	-2.4753333	0.0156001	0.03867226	-3.7200477	TMEM170B	assignmentsChromaffin_cells.Chrom_SOX2_statusHigh
assignmentsChromaffin_cells.Chrom_SOX2_statusHigh.HIF1A	-1.0635823	8.27687783	-2.5556936	0.01265475	0.03236435	-3.7208281	HIF1A	assignmentsChromaffin_cells.Chrom_SOX2_statusHigh
assignmentsChromaffin_cells.Chrom_SOX2_statusHigh.UBA5	0.51147087	5.27799167	2.52694356	0.01364224	0.03444494	-3.7208525	UBA5	assignmentsChromaffin_cells.Chrom_SOX2_statusHigh
assignmentsChromaffin_cells.Chrom_SOX2_statusHigh.COL5A2	-1.1372659	5.01849505	-2.4404698	0.01706363	0.04171746	-3.7218248	COL5A2	assignmentsChromaffin_cells.Chrom_SOX2_statusHigh
assignmentsChromaffin_cells.Chrom_SOX2_statusHigh.UCHL1	1.40961882	6.65124905	2.58923027	0.0115816	0.03006079	-3.7219418	UCHL1	assignmentsChromaffin_cells.Chrom_SOX2_statusHigh
assignmentsChromaffin_cells.Chrom_SOX2_statusHigh.MAP3K10	-0.4681204	3.67953207	-2.4666996	0.01595121	0.03941233	-3.7223605	MAP3K10	assignmentsChromaffin_cells.Chrom_SOX2_statusHigh
assignmentsChromaffin_cells.Chrom_SOX2_statusHigh.LINC00641	0.59749828	2.89762946	2.45375742	0.01649082	0.04057682	-3.722631	LINC00641	assignmentsChromaffin_cells.Chrom_SOX2_statusHigh
assignmentsChromaffin_cells.Chrom_SOX2_statusHigh.NANS	0.73940377	4.53922651	2.49723064	0.0147405	0.03678932	-3.723291	NANS	assignmentsChromaffin_cells.Chrom_SOX2_statusHigh
assignmentsChromaffin_cells.Chrom_SOX2_statusHigh.UBFD1	0.45324194	4.13277173	2.49772494	0.0147216	0.03675358	-3.7233457	UBFD1	assignmentsChromaffin_cells.Chrom_SOX2_statusHigh
assignmentsChromaffin_cells.Chrom_SOX2_statusHigh.EFNB2	1.25291743	3.49844402	2.41691118	0.01811992	0.04388513	-3.723569	EFNB2	assignmentsChromaffin_cells.Chrom_SOX2_statusHigh
assignmentsChromaffin_cells.Chrom_SOX2_statusHigh.ARID4B	-0.3955558	8.56073069	-2.5744942	0.01203742	0.03107452	-3.7342395	ARID4B	assignmentsChromaffin_cells.Chrom_SOX2_statusHigh
assignmentsChromaffin_cells.Chrom_SOX2_statusHigh.KMT2E	-0.5032813	8.87436537	-2.5712827	0.01214018	0.03129286	-3.7354863	KMT2E	assignmentsChromaffin_cells.Chrom_SOX2_statusHigh
assignmentsChromaffin_cells.Chrom_SOX2_statusHigh.SRRM2	-0.7351164	8.53087745	-2.5702682	0.0121728	0.03136183	-3.7357362	SRRM2	assignmentsChromaffin_cells.Chrom_SOX2_statusHigh
assignmentsChromaffin_cells.Chrom_SOX2_statusHigh.EXOSC8	0.52201876	5.07902384	2.50600125	0.01440832	0.03603878	-3.7362792	EXOSC8	assignmentsChromaffin_cells.Chrom_SOX2_statusHigh
assignmentsChromaffin_cells.Chrom_SOX2_statusHigh.KHDRBS2	-2.4438872	3.08099299	-2.3896998	0.01941713	0.04643089	-3.7364549	KHDRBS2	assignmentsChromaffin_cells.Chrom_SOX2_statusHigh
assignmentsChromaffin_cells.Chrom_SOX2_statusHigh.KCTD18	-0.5705587	3.40604294	-2.4378096	0.01717821	0.04193366	-3.7364788	KCTD18	assignmentsChromaffin_cells.Chrom_SOX2_statusHigh
assignmentsChromaffin_cells.Chrom_SOX2_statusHigh.PHOSPHO2	1.00910715	2.43021296	2.45759204	0.01632927	0.04021016	-3.7367604	PHOSPHO2	assignmentsChromaffin_cells.Chrom_SOX2_statusHigh
assignmentsChromaffin_cells.Chrom_SOX2_statusHigh.THUMPD2	-0.499662	5.51562958	-2.5103439	0.01424636	0.03568933	-3.7383056	THUMPD2	assignmentsChromaffin_cells.Chrom_SOX2_statusHigh
assignmentsChromaffin_cells.Chrom_SOX2_statusHigh.RNF114	0.35534226	5.74073076	2.51957439	0.01390752	0.0349442	-3.7405049	RNF114	assignmentsChromaffin_cells.Chrom_SOX2_statusHigh

assignmentsChromaffin_cells.Chrom_SOX2_statusHigh.TBC1D14	-0.7004862	6.1222149	-2.5119434	0.01418711	0.03557981	-3.7405205	TBC1D14	assignmentsChromaffin_cells.Chrom_SOX2_statusHigh
assignmentsChromaffin_cells.Chrom_SOX2_statusHigh.IGSF9B	-0.8401027	5.36147313	-2.5303039	0.0135228	0.03421339	-3.7409096	IGSF9B	assignmentsChromaffin_cells.Chrom_SOX2_statusHigh
assignmentsChromaffin_cells.Chrom_SOX2_statusHigh.PRMT5	0.58087249	2.92810204	2.44238348	0.01697849	0.04155998	-3.7418425	PRMT5	assignmentsChromaffin_cells.Chrom_SOX2_statusHigh
assignmentsChromaffin_cells.Chrom_SOX2_statusHigh.TENT4A	-0.5405691	6.13133558	-2.5175712	0.01398043	0.03511639	-3.7424215	TENT4A	assignmentsChromaffin_cells.Chrom_SOX2_statusHigh
assignmentsChromaffin_cells.Chrom_SOX2_statusHigh.STK38L	-0.6799436	6.33538194	-2.5027094	0.0145322	0.03632031	-3.7444755	STK38L	assignmentsChromaffin_cells.Chrom_SOX2_statusHigh
assignmentsChromaffin_cells.Chrom_SOX2_statusHigh.ZNF285	-0.8418737	1.83024394	-2.402721	0.01878164	0.04515367	-3.7446853	ZNF285	assignmentsChromaffin_cells.Chrom_SOX2_statusHigh
assignmentsChromaffin_cells.Chrom_SOX2_statusHigh.PRR16	-2.242172	6.09393817	-2.4948436	0.01483852	0.0369994	-3.7452642	PRR16	assignmentsChromaffin_cells.Chrom_SOX2_statusHigh
assignmentsChromaffin_cells.Chrom_SOX2_statusHigh.LHFPL5	-1.0059235	2.27725632	-2.4082484	0.01852051	0.0446329	-3.7481342	LHFPL5	assignmentsChromaffin_cells.Chrom_SOX2_statusHigh
assignmentsChromaffin_cells.Chrom_SOX2_statusHigh.HDAC9	-1.3515768	9.81827787	-2.608091	0.01101417	0.02884475	-3.7484887	HDAC9	assignmentsChromaffin_cells.Chrom_SOX2_statusHigh
assignmentsChromaffin_cells.Chrom_SOX2_statusHigh.SP100	1.5088291	6.22873729	2.40504386	0.01867858	0.04495305	-3.7501768	SP100	assignmentsChromaffin_cells.Chrom_SOX2_statusHigh
assignmentsChromaffin_cells.Chrom_SOX2_statusHigh.RABL3	0.64181524	3.89450125	2.4746364	0.01562818	0.03870961	-3.7510665	RABL3	assignmentsChromaffin_cells.Chrom_SOX2_statusHigh
assignmentsChromaffin_cells.Chrom_SOX2_statusHigh.MAST2	-0.7029625	6.48682493	-2.5226342	0.01379681	0.03473678	-3.7519268	MAST2	assignmentsChromaffin_cells.Chrom_SOX2_statusHigh
assignmentsChromaffin_cells.Chrom_SOX2_statusHigh.LONRF1	-0.7592489	5.70367742	-2.4988447	0.01467886	0.03666399	-3.7519932	LONRF1	assignmentsChromaffin_cells.Chrom_SOX2_statusHigh
assignmentsChromaffin_cells.Chrom_SOX2_statusHigh.DHRS2	1.67096522	0.87963332	2.41593089	0.01817002	0.04397989	-3.7524946	DHRS2	assignmentsChromaffin_cells.Chrom_SOX2_statusHigh
assignmentsChromaffin_cells.Chrom_SOX2_statusHigh.IRF2BP2	0.85935624	6.10933175	2.52996999	0.01353464	0.03422714	-3.7527529	IRF2BP2	assignmentsChromaffin_cells.Chrom_SOX2_statusHigh
assignmentsChromaffin_cells.Chrom_SOX2_statusHigh.PINLYP	1.1193239	1.75747341	2.37287711	0.02024914	0.04814379	-3.7540263	PINLYP	assignmentsChromaffin_cells.Chrom_SOX2_statusHigh
assignmentsChromaffin_cells.Chrom_SOX2_statusHigh.PCED1A	-0.8345432	4.28178618	-2.462498	0.01612463	0.03977342	-3.7552031	PCED1A	assignmentsChromaffin_cells.Chrom_SOX2_statusHigh
assignmentsChromaffin_cells.Chrom_SOX2_statusHigh.VPS26C	-0.4879067	5.6102977	-2.5068112	0.01437799	0.03597975	-3.7558401	VPS26C	assignmentsChromaffin_cells.Chrom_SOX2_statusHigh
assignmentsChromaffin_cells.Chrom_SOX2_statusHigh.LYRM1	0.44761216	4.43540799	2.49140466	0.01496495	0.03725094	-3.7567241	LYRM1	assignmentsChromaffin_cells.Chrom_SOX2_statusHigh
assignmentsChromaffin_cells.Chrom_SOX2_statusHigh.ZNF528	-0.8032561	4.57841528	-2.4751924	0.01560577	0.03867226	-3.7596714	ZNF528	assignmentsChromaffin_cells.Chrom_SOX2_statusHigh
assignmentsChromaffin_cells.Chrom_SOX2_statusHigh.DLL1	0.67346575	3.27980129	2.42209891	0.01788035	0.04337695	-3.7597415	DLL1	assignmentsChromaffin_cells.Chrom_SOX2_statusHigh
assignmentsChromaffin_cells.Chrom_SOX2_statusHigh.THNSL1	0.65471416	1.96448965	2.41110942	0.01838662	0.0443836	-3.7603167	THNSL1	assignmentsChromaffin_cells.Chrom_SOX2_statusHigh
assignmentsChromaffin_cells.Chrom_SOX2_statusHigh.WIPF3	1.63831354	3.1083049	2.45259365	0.0165469	0.0406961	-3.7616276	WIPF3	assignmentsChromaffin_cells.Chrom_SOX2_statusHigh
assignmentsChromaffin_cells.Chrom_SOX2_statusHigh.FBXO2	1.37125103	2.13683355	2.42986016	0.01753599	0.04267711	-3.7659302	FBXO2	assignmentsChromaffin_cells.Chrom_SOX2_statusHigh
assignmentsChromaffin_cells.Chrom_SOX2_statusHigh.ZBTB25	-0.5668211	5.08190648	-2.482792	0.01530237	0.03801416	-3.7690515	ZBTB25	assignmentsChromaffin_cells.Chrom_SOX2_statusHigh
assignmentsChromaffin_cells.Chrom_SOX2_statusHigh.GJC1	-1.1475904	3.41939388	-2.3669649	0.0205517	0.04872614	-3.7692345	GJC1	assignmentsChromaffin_cells.Chrom_SOX2_statusHigh
assignmentsChromaffin_cells.Chrom_SOX2_statusHigh.ABCC1	-0.800662	6.18965257	-2.4872888	0.01512536	0.0376152	-3.769788	ABCC1	assignmentsChromaffin_cells.Chrom_SOX2_statusHigh
assignmentsChromaffin_cells.Chrom_SOX2_statusHigh.MRPL42	0.52916274	5.40547017	2.51188164	0.0141894	0.03557998	-3.7727802	MRPL42	assignmentsChromaffin_cells.Chrom_SOX2_statusHigh
assignmentsChromaffin_cells.Chrom_SOX2_statusHigh.CHST8	2.23818883	2.01720528	2.41293579	0.0183087	0.04422214	-3.7728244	CHST8	assignmentsChromaffin_cells.Chrom_SOX2_statusHigh
assignmentsChromaffin_cells.Chrom_SOX2_statusHigh.DYNC1I2	-0.4585088	6.74360558	-2.5255839	0.01369084	0.03453503	-3.7732427	DYNC1I2	assignmentsChromaffin_cells.Chrom_SOX2_statusHigh
assignmentsChromaffin_cells.Chrom_SOX2_statusHigh.ATG13	-0.5466422	5.09316407	-2.4732966	0.01568229	0.03882566	-3.7734461	ATG13	assignmentsChromaffin_cells.Chrom_SOX2_statusHigh
assignmentsChromaffin_cells.Chrom_SOX2_statusHigh.GPR176	1.78738117	3.36001825	2.36826354	0.02049236	0.04862329	-3.7740919	GPR176	assignmentsChromaffin_cells.Chrom_SOX2_statusHigh
assignmentsChromaffin_cells.Chrom_SOX2_statusHigh.ZNF83	-0.5306493	6.69275544	-2.5191749	0.01392203	0.03497518	-3.7754201	ZNF83	assignmentsChromaffin_cells.Chrom_SOX2_statusHigh
assignmentsChromaffin_cells.Chrom_SOX2_statusHigh.ZNF33A	-0.492682	7.0239844	-2.534254	0.01338359	0.03393612	-3.7775634	ZNF33A	assignmentsChromaffin_cells.Chrom_SOX2_statusHigh
assignmentsChromaffin_cells.Chrom_SOX2_statusHigh.ZNF253	-0.7166372	4.37942501	-2.4558259	0.01640349	0.04037434	-3.7802111	ZNF253	assignmentsChromaffin_cells.Chrom_SOX2_statusHigh
assignmentsChromaffin_cells.Chrom_SOX2_statusHigh.SRSF7	0.76749662	7.11968124	2.52393808	0.01374988	0.03463524	-3.78091	SRSF7	assignmentsChromaffin_cells.Chrom_SOX2_statusHigh
assignmentsChromaffin_cells.Chrom_SOX2_statusHigh.TOX4	0.45641753	5.78274682	2.51109682	0.01421844	0.03562496	-3.7834918	TOX4	assignmentsChromaffin_cells.Chrom_SOX2_statusHigh
assignmentsChromaffin_cells.Chrom_SOX2_statusHigh.NID1	1.64961938	4.15795849	2.36821859	0.02049467	0.04862329	-3.7841254	NID1	assignmentsChromaffin_cells.Chrom_SOX2_statusHigh
assignmentsChromaffin_cells.Chrom_SOX2_statusHigh.ZNF646	0.66464685	2.20108091	2.38448498	0.01966664	0.04695749	-3.7867018	ZNF646	assignmentsChromaffin_cells.Chrom_SOX2_statusHigh
assignmentsChromaffin_cells.Chrom_SOX2_statusHigh.RFX3	-0.6395817	8.12281869	-2.5388232	0.01322418	0.03359034	-3.788765	RFX3	assignmentsChromaffin_cells.Chrom_SOX2_statusHigh
assignmentsChromaffin_cells.Chrom_SOX2_statusHigh.VKORC1L1	-0.5164532	5.73047386	-2.4916304	0.01495619	0.03723493	-3.7892646	VKORC1L1	assignmentsChromaffin_cells.Chrom_SOX2_statusHigh
assignmentsChromaffin_cells.Chrom_SOX2_statusHigh.ZNF519	-0.8384536	3.9144189	-2.4338356	0.01735344	0.04228419	-3.7917034	ZNF519	assignmentsChromaffin_cells.Chrom_SOX2_statusHigh
assignmentsChromaffin_cells.Chrom_SOX2_statusHigh.TUG1	-0.3882065	6.18083213	-2.5064368	0.014392	0.03600358	-3.7941819	TUG1	assignmentsChromaffin_cells.Chrom_SOX2_statusHigh
assignmentsChromaffin_cells.Chrom_SOX2_statusHigh.RAMP1	1.89898518	5.54437738	2.5315531	0.01348476	0.03414408	-3.7942476	RAMP1	assignmentsChromaffin_cells.Chrom_SOX2_statusHigh
assignmentsChromaffin_cells.Chrom_SOX2_statusHigh.MEG9	-2.3214881	4.03302186	-2.449533	0.01667726	0.04094762	-3.795226	MEG9	assignmentsChromaffin_cells.Chrom_SOX2_statusHigh
assignmentsChromaffin_cells.Chrom_SOX2_statusHigh.AKAP9	-0.4449783	8.40781844	-2.551026	0.01280681	0.0326855	-3.7974269	AKAP9	assignmentsChromaffin_cells.Chrom_SOX2_statusHigh
assignmentsChromaffin_cells.Chrom_SOX2_statusHigh.LY6E	1.68519694	3.33385334	2.39672338	0.01907594	0.04577197	-3.7978522	LY6E	assignmentsChromaffin_cells.Chrom_SOX2_statusHigh
assignmentsChromaffin_cells.Chrom_SOX2_statusHigh.CEP170	-0.514652	7.64106121	-2.5248561	0.01371692	0.0345696	-3.7997659	CEP170	assignmentsChromaffin_cells.Chrom_SOX2_statusHigh
assignmentsChromaffin_cells.Chrom_SOX2_statusHigh.GCC2-AS1	-0.7717509	3.09578944	-2.3937889	0.01921058	0.0460316	-3.7998958	GCC2-AS1	assignmentsChromaffin_cells.Chrom_SOX2_statusHigh
assignmentsChromaffin_cells.Chrom_SOX2_statusHigh.AIMP2	0.61141675	3.85406162	2.45117262	0.01660053	0.0408017	-3.8021303	AIMP2	assignmentsChromaffin_cells.Chrom_SOX2_statusHigh
assignmentsChromaffin_cells.Chrom_SOX2_statusHigh.VAMP4	0.39669615	4.95580677	2.48248103	0.01531468	0.03803886	-3.8049677	VAMP4	assignmentsChromaffin_cells.Chrom_SOX2_statusHigh
assignmentsChromaffin_cells.Chrom_SOX2_statusHigh.ANKRD23	-0.8451202	3.30719275	-2.4134012	0.01828	0.04419296	-3.8063763	ANKRD23	assignmentsChromaffin_cells.Chrom_SOX2_statusHigh
assignmentsChromaffin_cells.Chrom_SOX2_statusHigh.RHEB	0.64278677	6.97295462	2.52583005	0.01368203	0.03451823	-3.8065703	RHEB	assignmentsChromaffin_cells.Chrom_SOX2_statusHigh
assignmentsChromaffin_cells.Chrom_SOX2_statusHigh.MARCKS	1.47051995	4.71363124	2.46947642	0.01584415	0.03917195	-3.8067763	MARCKS	assignmentsChromaffin_cells.Chrom_SOX2_statusHigh
assignmentsChromaffin_cells.Chrom_SOX2_statusHigh.PTCH1	-0.912786	3.6590838	-2.4211508	0.01792353	0.04346856	-3.8080915	PTCH1	assignmentsChromaffin_cells.Chrom_SOX2_statusHigh
assignmentsChromaffin_cells.Chrom_SOX2_statusHigh.QSOX2	-0.8382368	3.79172259	-2.4275491	0.01763391	0.04285041	-3.8093797	QSOX2	assignmentsChromaffin_cells.Chrom_SOX2_statusHigh
assignmentsChromaffin_cells.Chrom_SOX2_statusHigh.TDP1	-0.6396296	4.45586074	-2.4397074	0.01709509	0.04177904	-3.811678	TDP1	assignmentsChromaffin_cells.Chrom_SOX2_statusHigh
assignmentsChromaffin_cells.Chrom_SOX2_statusHigh.UBXN2B	-0.6479686	5.09813096	-2.4671802	0.01593148	0.03937572	-3.8117981	UBXN2B	assignmentsChromaffin_cells.Chrom_SOX2_statusHigh
assignmentsChromaffin_cells.Chrom_SOX2_statusHigh.ATP11B	-0.5374346	6.90996606	-2.497875	0.01471586	0.03675013	-3.8120692	ATP11B	assignmentsChromaffin_cells.Chrom_SOX2_statusHigh
assignmentsChromaffin_cells.Chrom_SOX2_statusHigh.ANKS1B	-1.0656273	6.96536811	-2.5297139	0.01354925	0.03425328	-3.8127929	ANKS1B	assignmentsChromaffin_cells.Chrom_SOX2_statusHigh
assignmentsChromaffin_cells.Chrom_SOX2_statusHigh.TTC31	-0.4659793	4.08139939	-2.4405529	0.01705817	0.04171046	-3.8142988	TTC31	assignmentsChromaffin_cells.Chrom_SOX2_statusHigh
assignmentsChromaffin_cells.Chrom_SOX2_statusHigh.LNPK	-0.6220556	6.23476703	-2.5082967	0.0143225	0.03587449	-3.8148894	LNPK	assignmentsChromaffin_cells.Chrom_SOX2_statusHigh
assignmentsChromaffin_cells.Chrom_SOX2_statusHigh.SYT5	1.42729864	4.49436034	2.5080324	0.01433868	0.03589819	-3.8153953	SYT5	assignmentsChromaffin_cells.Chrom_SOX2_statusHigh
assignmentsChromaffin_cells.Chrom_SOX2_statusHigh.CNNM4	-0.8514248	4.03502901	-2.4200098	0.01797562	0.04357513	-3.8196367	CNNM4	assignmentsChromaffin_cells.Chrom_SOX2_statusHigh

assignmentsChromaffin_cells.Chrom_SOX2_statusHigh.ARHGAP27	-1.0958188	3.72091105	-2.3669087	0.02055459	0.04872614	-3.8203077	ARHGAP27	assignmentsChromaffin_cells.Chrom_SOX2_statusHigh
assignmentsChromaffin_cells.Chrom_SOX2_statusHigh.CYB561A3	0.78383269	2.84393944	2.3962547	0.0190913	0.04580199	-3.8210198	CYB561A3	assignmentsChromaffin_cells.Chrom_SOX2_statusHigh
assignmentsChromaffin_cells.Chrom_SOX2_statusHigh.RASEF	1.74414781	2.11509786	2.40272842	0.01878847	0.0451633	-3.8211563	RASEF	assignmentsChromaffin_cells.Chrom_SOX2_statusHigh
assignmentsChromaffin_cells.Chrom_SOX2_statusHigh.GSTCD	-0.5086613	5.07093184	-2.4630467	0.01610189	0.03972343	-3.8249053	GSTCD	assignmentsChromaffin_cells.Chrom_SOX2_statusHigh
assignmentsChromaffin_cells.Chrom_SOX2_statusHigh.MTO1	-0.4902677	4.54285491	-2.4410937	0.01703459	0.04166552	-3.825416	MTO1	assignmentsChromaffin_cells.Chrom_SOX2_statusHigh
assignmentsChromaffin_cells.Chrom_SOX2_statusHigh.SPRED1	-0.9813999	6.56571704	-2.4292952	0.01755562	0.04270145	-3.825453	SPRED1	assignmentsChromaffin_cells.Chrom_SOX2_statusHigh
assignmentsChromaffin_cells.Chrom_SOX2_statusHigh.DECR1	0.55258236	6.48070458	2.49677411	0.01475798	0.03682721	-3.8254542	DECR1	assignmentsChromaffin_cells.Chrom_SOX2_statusHigh
assignmentsChromaffin_cells.Chrom_SOX2_statusHigh.SPOP	-0.4742982	6.4758413	-2.4924484	0.01492451	0.03717337	-3.8256353	SPOP	assignmentsChromaffin_cells.Chrom_SOX2_statusHigh
assignmentsChromaffin_cells.Chrom_SOX2_statusHigh.POPDC3	1.61522385	0.85912452	2.36589434	0.02061439	0.04883509	-3.8257686	POPDC3	assignmentsChromaffin_cells.Chrom_SOX2_statusHigh
assignmentsChromaffin_cells.Chrom_SOX2_statusHigh.BNIP2	0.45230607	5.70143549	2.47429365	0.01564201	0.03873788	-3.826494	BNIP2	assignmentsChromaffin_cells.Chrom_SOX2_statusHigh
assignmentsChromaffin_cells.Chrom_SOX2_statusHigh.LNX2	-0.4542979	4.77875338	-2.4466145	0.01679559	0.04116883	-3.8289933	LNX2	assignmentsChromaffin_cells.Chrom_SOX2_statusHigh
assignmentsChromaffin_cells.Chrom_SOX2_statusHigh.ZNF761	1.06637148	3.91361181	2.42886796	0.01757679	0.04273749	-3.8315692	ZNF761	assignmentsChromaffin_cells.Chrom_SOX2_statusHigh
assignmentsChromaffin_cells.Chrom_SOX2_statusHigh.DCUN1D2	-0.5679798	5.22946865	-2.4710328	0.01577411	0.0390349	-3.831605	DCUN1D2	assignmentsChromaffin_cells.Chrom_SOX2_statusHigh
assignmentsChromaffin_cells.Chrom_SOX2_statusHigh.BMI1	0.61424126	4.80044132	2.46889864	0.01586111	0.03920785	-3.8324835	BMI1	assignmentsChromaffin_cells.Chrom_SOX2_statusHigh
assignmentsChromaffin_cells.Chrom_SOX2_statusHigh.DENND4B	-0.6728015	4.92529466	-2.446347	0.0168071	0.04119076	-3.8330672	DENND4B	assignmentsChromaffin_cells.Chrom_SOX2_statusHigh
assignmentsChromaffin_cells.Chrom_SOX2_statusHigh.PPP3R1	-0.6501091	7.31562133	-2.4974498	0.01473212	0.03677412	-3.833528	PPP3R1	assignmentsChromaffin_cells.Chrom_SOX2_statusHigh
assignmentsChromaffin_cells.Chrom_SOX2_statusHigh.TFIP11	0.49514166	3.54556423	2.41764335	0.01808409	0.04381157	-3.8355547	TFIP11	assignmentsChromaffin_cells.Chrom_SOX2_statusHigh
assignmentsChromaffin_cells.Chrom_SOX2_statusHigh.DLGAP3	-0.9118363	2.06784308	-2.374475	0.02016805	0.04797615	-3.8367419	DLGAP3	assignmentsChromaffin_cells.Chrom_SOX2_statusHigh
assignmentsChromaffin_cells.Chrom_SOX2_statusHigh.FAM118B	-0.5434663	4.17858032	-2.4166641	0.01812915	0.04389915	-3.8379115	FAM118B	assignmentsChromaffin_cells.Chrom_SOX2_statusHigh
assignmentsChromaffin_cells.Chrom_SOX2_statusHigh.POLR3A	-0.5406597	5.32811456	-2.4669421	0.01594125	0.0393938	-3.8403328	POLR3A	assignmentsChromaffin_cells.Chrom_SOX2_statusHigh
assignmentsChromaffin_cells.Chrom_SOX2_statusHigh.PPARD	-0.6816437	6.0451117	-2.4535366	0.01650017	0.0405936	-3.840733	PPARD	assignmentsChromaffin_cells.Chrom_SOX2_statusHigh
assignmentsChromaffin_cells.Chrom_SOX2_statusHigh.EID2	1.18407026	2.27346381	2.38585535	0.01960148	0.04682284	-3.8411994	EID2	assignmentsChromaffin_cells.Chrom_SOX2_statusHigh
assignmentsChromaffin_cells.Chrom_SOX2_statusHigh.GNL2	0.65620915	4.81992673	2.44119418	0.01703022	0.04166117	-3.8416501	GNL2	assignmentsChromaffin_cells.Chrom_SOX2_statusHigh
assignmentsChromaffin_cells.Chrom_SOX2_statusHigh.XYLB	-0.6906112	3.9330386	-2.4296359	0.01754037	0.04268129	-3.8434523	XYLB	assignmentsChromaffin_cells.Chrom_SOX2_statusHigh
assignmentsChromaffin_cells.Chrom_SOX2_statusHigh.YBEY	0.6318551	3.64689255	2.43457426	0.01732075	0.04222817	-3.8448717	YBEY	assignmentsChromaffin_cells.Chrom_SOX2_statusHigh
assignmentsChromaffin_cells.Chrom_SOX2_statusHigh.MT-ND2	2.47313856	8.07431618	2.53379108	0.01340595	0.0339864	-3.8449343	MT-ND2	assignmentsChromaffin_cells.Chrom_SOX2_statusHigh
assignmentsChromaffin_cells.Chrom_SOX2_statusHigh.NAGK	0.52788345	5.01118737	2.45024148	0.01664021	0.04086292	-3.8450885	NAGK	assignmentsChromaffin_cells.Chrom_SOX2_statusHigh
assignmentsChromaffin_cells.Chrom_SOX2_statusHigh.XKR6	-0.8771588	7.47531009	-2.5196768	0.0139057	0.0349442	-3.8466911	XKR6	assignmentsChromaffin_cells.Chrom_SOX2_statusHigh
assignmentsChromaffin_cells.Chrom_SOX2_statusHigh.SPRTN	0.53287964	4.05583593	2.43214042	0.01742868	0.04242883	-3.8478125	SPRTN	assignmentsChromaffin_cells.Chrom_SOX2_statusHigh
assignmentsChromaffin_cells.Chrom_SOX2_statusHigh.MGAT1	0.73162079	5.21371931	2.42863188	0.01758532	0.04275176	-3.8479106	MGAT1	assignmentsChromaffin_cells.Chrom_SOX2_statusHigh
assignmentsChromaffin_cells.Chrom_SOX2_statusHigh.CCNE2	0.8173572	2.51668428	2.36975804	0.02040826	0.04845292	-3.852046	CCNE2	assignmentsChromaffin_cells.Chrom_SOX2_statusHigh
assignmentsChromaffin_cells.Chrom_SOX2_statusHigh.NGLY1	-0.5736318	6.1499971	-2.4652953	0.01600898	0.0395429	-3.8534745	NGLY1	assignmentsChromaffin_cells.Chrom_SOX2_statusHigh
assignmentsChromaffin_cells.Chrom_SOX2_statusHigh.NUDCD1	0.5201629	4.79919972	2.44705331	0.01677672	0.04114145	-3.8568963	NUDCD1	assignmentsChromaffin_cells.Chrom_SOX2_statusHigh
assignmentsChromaffin_cells.Chrom_SOX2_statusHigh.KANSL1L	-0.7592355	6.49451253	-2.4738208	0.0156611	0.03877917	-3.857285	KANSL1L	assignmentsChromaffin_cells.Chrom_SOX2_statusHigh
assignmentsChromaffin_cells.Chrom_SOX2_statusHigh.KCTD2	0.49911825	3.81683917	2.43032278	0.01750967	0.04261954	-3.8586969	KCTD2	assignmentsChromaffin_cells.Chrom_SOX2_statusHigh
assignmentsChromaffin_cells.Chrom_SOX2_statusHigh.FMN2	-1.294382	6.18162913	-2.5047742	0.01446073	0.03614732	-3.8592564	FMN2	assignmentsChromaffin_cells.Chrom_SOX2_statusHigh
assignmentsChromaffin_cells.Chrom_SOX2_statusHigh.YWHAG	0.74482919	6.57984133	2.49281755	0.01491023	0.03716089	-3.8598072	YWHAG	assignmentsChromaffin_cells.Chrom_SOX2_statusHigh
assignmentsChromaffin_cells.Chrom_SOX2_statusHigh.UBOX5	-0.6823443	3.52400433	-2.391936	0.01930064	0.04618677	-3.8618788	UBOX5	assignmentsChromaffin_cells.Chrom_SOX2_statusHigh
assignmentsChromaffin_cells.Chrom_SOX2_statusHigh.FYN	-0.8076537	8.204172	-2.4961188	0.01478311	0.03687271	-3.8623508	FYN	assignmentsChromaffin_cells.Chrom_SOX2_statusHigh
assignmentsChromaffin_cells.Chrom_SOX2_statusHigh.RNF41	0.6185955	4.39255412	2.43896135	0.01712772	0.04183588	-3.8632666	RNF41	assignmentsChromaffin_cells.Chrom_SOX2_statusHigh
assignmentsChromaffin_cells.Chrom_SOX2_statusHigh.RBMS3-AS2	-0.9029617	4.8474732	-2.4490447	0.01669135	0.04096341	-3.8635693	RBMS3-AS2	assignmentsChromaffin_cells.Chrom_SOX2_statusHigh
assignmentsChromaffin_cells.Chrom_SOX2_statusHigh.XPO1	-0.538169	7.32431927	-2.4950924	0.01482252	0.03696526	-3.8640689	XPO1	assignmentsChromaffin_cells.Chrom_SOX2_statusHigh
assignmentsChromaffin_cells.Chrom_SOX2_statusHigh.PES1	0.69928208	3.87103427	2.40235823	0.0187989	0.04518158	-3.8676028	PES1	assignmentsChromaffin_cells.Chrom_SOX2_statusHigh
assignmentsChromaffin_cells.Chrom_SOX2_statusHigh.BICDL1	-0.8948357	5.50676455	-2.4760842	0.01557166	0.03862329	-3.8706302	BICDL1	assignmentsChromaffin_cells.Chrom_SOX2_statusHigh
assignmentsChromaffin_cells.Chrom_SOX2_statusHigh.ZFR	-0.4087472	7.58222505	-2.4991451	0.01466741	0.03664111	-3.8729324	ZFR	assignmentsChromaffin_cells.Chrom_SOX2_statusHigh
assignmentsChromaffin_cells.Chrom_SOX2_statusHigh.L3MBTL1	-0.562986	4.99472831	-2.4519883	0.01656584	0.04073641	-3.8735288	L3MBTL1	assignmentsChromaffin_cells.Chrom_SOX2_statusHigh
assignmentsChromaffin_cells.Chrom_SOX2_statusHigh.LRIG1	1.26696433	4.21310809	2.37767451	0.02001005	0.04763293	-3.8737534	LRIG1	assignmentsChromaffin_cells.Chrom_SOX2_statusHigh
assignmentsChromaffin_cells.Chrom_SOX2_statusHigh.ARMC10	0.40006421	5.03895542	2.45047965	0.01663005	0.04084422	-3.8745379	ARMC10	assignmentsChromaffin_cells.Chrom_SOX2_statusHigh
assignmentsChromaffin_cells.Chrom_SOX2_statusHigh.UNKL	-0.5760965	4.00394539	-2.4002263	0.01890058	0.04539875	-3.8758925	UNKL	assignmentsChromaffin_cells.Chrom_SOX2_statusHigh
assignmentsChromaffin_cells.Chrom_SOX2_statusHigh.KDM1B	-0.5154726	4.53104985	-2.4062087	0.01861649	0.04485073	-3.8759469	KDM1B	assignmentsChromaffin_cells.Chrom_SOX2_statusHigh
assignmentsChromaffin_cells.Chrom_SOX2_statusHigh.SLC38A6	-0.6891222	6.09074573	-2.4606924	0.01619967	0.03993395	-3.8790115	SLC38A6	assignmentsChromaffin_cells.Chrom_SOX2_statusHigh
assignmentsChromaffin_cells.Chrom_SOX2_statusHigh.HIVEP1	-0.7439216	7.31865039	-2.4757988	0.01558137	0.03863822	-3.8799633	HIVEP1	assignmentsChromaffin_cells.Chrom_SOX2_statusHigh
assignmentsChromaffin_cells.Chrom_SOX2_statusHigh.HOXC6	2.13371214	2.94230594	2.42351104	0.01782321	0.04324489	-3.8828932	HOXC6	assignmentsChromaffin_cells.Chrom_SOX2_statusHigh
assignmentsChromaffin_cells.Chrom_SOX2_statusHigh.MCTS1	0.49800329	5.09077071	2.4531994	0.01651445	0.0406225	-3.8839274	MCTS1	assignmentsChromaffin_cells.Chrom_SOX2_statusHigh
assignmentsChromaffin_cells.Chrom_SOX2_statusHigh.IMMP1L	-0.6280126	5.7619261	-2.4577503	0.01632262	0.04019997	-3.8844584	IMMP1L	assignmentsChromaffin_cells.Chrom_SOX2_statusHigh
assignmentsChromaffin_cells.Chrom_SOX2_statusHigh.SERP2	0.68337762	3.4427308	2.42407063	0.01779084	0.04317941	-3.8864541	SERP2	assignmentsChromaffin_cells.Chrom_SOX2_statusHigh
assignmentsChromaffin_cells.Chrom_SOX2_statusHigh.PPP1R14B	1.35234894	3.68827569	2.40600983	0.01863303	0.04488383	-3.887428	PPP1R14B	assignmentsChromaffin_cells.Chrom_SOX2_statusHigh
assignmentsChromaffin_cells.Chrom_SOX2_statusHigh.SLC27A1	-0.6836832	4.38185134	-2.4003209	0.01889605	0.04539468	-3.8897886	SLC27A1	assignmentsChromaffin_cells.Chrom_SOX2_statusHigh
assignmentsChromaffin_cells.Chrom_SOX2_statusHigh.MRGBP	0.57083914	2.65487829	2.36305861	0.02075379	0.04912179	-3.8919023	MRGBP	assignmentsChromaffin_cells.Chrom_SOX2_statusHigh
assignmentsChromaffin_cells.Chrom_SOX2_statusHigh.OXSR1	-0.5982548	6.3485755	-2.451371	0.01659208	0.04078845	-3.8930034	OXSR1	assignmentsChromaffin_cells.Chrom_SOX2_statusHigh
assignmentsChromaffin_cells.Chrom_SOX2_statusHigh.TTC3	-0.4979734	8.80356066	-2.5259585	0.01367744	0.03451207	-3.8953283	TTC3	assignmentsChromaffin_cells.Chrom_SOX2_statusHigh
assignmentsChromaffin_cells.Chrom_SOX2_statusHigh.ZNF875	-0.4924551	5.46884361	-2.4449298	0.0168682	0.04132156	-3.8959864	ZNF875	assignmentsChromaffin_cells.Chrom_SOX2_statusHigh
assignmentsChromaffin_cells.Chrom_SOX2_statusHigh.BBS2	-0.5588512	4.64057218	-2.4129135	0.01830264	0.04422214	-3.8974666	BBS2	assignmentsChromaffin_cells.Chrom_SOX2_statusHigh
assignmentsChromaffin_cells.Chrom_SOX2_statusHigh.POU2F1	-0.4587883	7.38129806	-2.4837096	0.0152661	0.03793581	-3.8981234	POU2F1	assignmentsChromaffin_cells.Chrom_SOX2_statusHigh

assignmentsChromaffin_cells.Chrom_SOX2_statusHigh.MYO1C	0.622446	4.51734252	2.40578536	0.01863647	0.04488537	-3.8982381	MYO1C	assignmentsChromaffin_cells.Chrom_SOX2_statusHigh
assignmentsChromaffin_cells.Chrom_SOX2_statusHigh.PHF8	-0.4090522	5.61910016	-2.439515	0.0171035	0.04178945	-3.9024853	PHF8	assignmentsChromaffin_cells.Chrom_SOX2_statusHigh
assignmentsChromaffin_cells.Chrom_SOX2_statusHigh.KCNB1	-1.0312844	4.78328004	-2.4386962	0.01714222	0.04186288	-3.9028974	KCNB1	assignmentsChromaffin_cells.Chrom_SOX2_statusHigh
assignmentsChromaffin_cells.Chrom_SOX2_statusHigh.FOSL2	1.48490139	6.97874744	2.47098725	0.01578258	0.03904984	-3.9037099	FOSL2	assignmentsChromaffin_cells.Chrom_SOX2_statusHigh
assignmentsChromaffin_cells.Chrom_SOX2_statusHigh.BCKDHB	-0.6608483	6.45043427	-2.4640401	0.01606078	0.03965253	-3.9041987	BCKDHB	assignmentsChromaffin_cells.Chrom_SOX2_statusHigh
assignmentsChromaffin_cells.Chrom_SOX2_statusHigh.AMMECR1L	-0.5400093	4.53286605	-2.4067279	0.01859202	0.0447985	-3.9050093	AMMECR1L	assignmentsChromaffin_cells.Chrom_SOX2_statusHigh
assignmentsChromaffin_cells.Chrom_SOX2_statusHigh.LINC00910	1.02150849	3.71322946	2.39378215	0.01921284	0.0460316	-3.9054534	LINC00910	assignmentsChromaffin_cells.Chrom_SOX2_statusHigh
assignmentsChromaffin_cells.Chrom_SOX2_statusHigh.RUVBL1	0.61610315	4.51454975	2.41306316	0.01829569	0.04421734	-3.9058619	RUVBL1	assignmentsChromaffin_cells.Chrom_SOX2_statusHigh
assignmentsChromaffin_cells.Chrom_SOX2_statusHigh.NUDT5	0.32187172	5.26985656	2.44248277	0.01697418	0.04155998	-3.9070407	NUDT5	assignmentsChromaffin_cells.Chrom_SOX2_statusHigh
assignmentsChromaffin_cells.Chrom_SOX2_statusHigh.TTC7A	-0.7792946	5.6636777	-2.4093531	0.01846871	0.04454152	-3.9073976	TTC7A	assignmentsChromaffin_cells.Chrom_SOX2_statusHigh
assignmentsChromaffin_cells.Chrom_SOX2_statusHigh.C1orf52	0.43489261	5.21026542	2.42032167	0.01796137	0.04355374	-3.9089047	C1orf52	assignmentsChromaffin_cells.Chrom_SOX2_statusHigh
assignmentsChromaffin_cells.Chrom_SOX2_statusHigh.PPP2R2C	-1.0233876	4.73614827	-2.4492381	0.01668592	0.04095634	-3.9090887	PPP2R2C	assignmentsChromaffin_cells.Chrom_SOX2_statusHigh
assignmentsChromaffin_cells.Chrom_SOX2_statusHigh.IFNLR1	-0.8111684	2.42171186	-2.3620477	0.02080637	0.04922446	-3.9096914	IFNLR1	assignmentsChromaffin_cells.Chrom_SOX2_statusHigh
assignmentsChromaffin_cells.Chrom_SOX2_statusHigh.WDR31	-0.6891059	2.99946252	-2.3814886	0.01981555	0.04722166	-3.9098133	WDR31	assignmentsChromaffin_cells.Chrom_SOX2_statusHigh
assignmentsChromaffin_cells.Chrom_SOX2_statusHigh.SCRN3	0.56013977	4.52023509	2.43222091	0.0174251	0.04242656	-3.9100589	SCRN3	assignmentsChromaffin_cells.Chrom_SOX2_statusHigh
assignmentsChromaffin_cells.Chrom_SOX2_statusHigh.GSE1	-0.9068197	6.92239227	-2.4652206	0.01601402	0.03954925	-3.9109632	GSE1	assignmentsChromaffin_cells.Chrom_SOX2_statusHigh
assignmentsChromaffin_cells.Chrom_SOX2_statusHigh.WLS	-1.3678414	4.95029192	-2.3656515	0.02062693	0.04885759	-3.9153568	WLS	assignmentsChromaffin_cells.Chrom_SOX2_statusHigh
assignmentsChromaffin_cells.Chrom_SOX2_statusHigh.NCK1-DT	-0.9267779	3.83334468	-2.3570056	0.02107044	0.04974647	-3.9164165	NCK1-DT	assignmentsChromaffin_cells.Chrom_SOX2_statusHigh
assignmentsChromaffin_cells.Chrom_SOX2_statusHigh.TUBGCP3	-0.6605238	6.15291702	-2.4484345	0.01671746	0.04101494	-3.9173228	TUBGCP3	assignmentsChromaffin_cells.Chrom_SOX2_statusHigh
assignmentsChromaffin_cells.Chrom_SOX2_statusHigh.PPP1R13L	-0.8743559	3.42570305	-2.3594398	0.02094259	0.04950291	-3.9174832	PPP1R13L	assignmentsChromaffin_cells.Chrom_SOX2_statusHigh
assignmentsChromaffin_cells.Chrom_SOX2_statusHigh.ACSL1	-0.8992242	5.82707341	-2.3934661	0.01922624	0.04604995	-3.9211003	ACSL1	assignmentsChromaffin_cells.Chrom_SOX2_statusHigh
assignmentsChromaffin_cells.Chrom_SOX2_statusHigh.CEP290	-0.4714557	6.57325668	-2.4633577	0.01608901	0.03970388	-3.9227313	CEP290	assignmentsChromaffin_cells.Chrom_SOX2_statusHigh
assignmentsChromaffin_cells.Chrom_SOX2_statusHigh.AP1G2	-1.0131816	4.13252143	-2.3910808	0.01934444	0.04628467	-3.9228907	AP1G2	assignmentsChromaffin_cells.Chrom_SOX2_statusHigh
assignmentsChromaffin_cells.Chrom_SOX2_statusHigh.ANKRD42	0.5219106	4.14827505	2.39500892	0.01915148	0.04591889	-3.9271286	ANKRD42	assignmentsChromaffin_cells.Chrom_SOX2_statusHigh
assignmentsChromaffin_cells.Chrom_SOX2_statusHigh.SPANXA2-O	-0.89371	3.22185092	-2.3697697	0.02040766	0.04845292	-3.9309176	SPANXA2-OT	assignmentsChromaffin_cells.Chrom_SOX2_statusHigh
assignmentsChromaffin_cells.Chrom_SOX2_statusHigh.NECTIN3	0.88726983	4.56259059	2.41857003	0.01804155	0.04372834	-3.9311187	NECTIN3	assignmentsChromaffin_cells.Chrom_SOX2_statusHigh
assignmentsChromaffin_cells.Chrom_SOX2_statusHigh.KCTD9	-0.4076867	5.19091869	-2.4049032	0.01867816	0.04495305	-3.9325826	KCTD9	assignmentsChromaffin_cells.Chrom_SOX2_statusHigh
assignmentsChromaffin_cells.Chrom_SOX2_statusHigh.TOP2B	-0.4856455	6.66821081	-2.4468167	0.01678689	0.0411538	-3.9330779	TOP2B	assignmentsChromaffin_cells.Chrom_SOX2_statusHigh
assignmentsChromaffin_cells.Chrom_SOX2_statusHigh.TAF9B	0.59675125	2.74119569	2.35783578	0.02102676	0.0496799	-3.9347626	TAF9B	assignmentsChromaffin_cells.Chrom_SOX2_statusHigh
assignmentsChromaffin_cells.Chrom_SOX2_statusHigh.TGS1	0.44937848	5.62221253	2.4405617	0.01705779	0.04171046	-3.9362967	TGS1	assignmentsChromaffin_cells.Chrom_SOX2_statusHigh
assignmentsChromaffin_cells.Chrom_SOX2_statusHigh.SUFU	-0.6984218	5.25098758	-2.3780248	0.01998895	0.04759245	-3.936319	SUFU	assignmentsChromaffin_cells.Chrom_SOX2_statusHigh
assignmentsChromaffin_cells.Chrom_SOX2_statusHigh.CDC27	-0.3665149	7.16285452	-2.4601088	0.01622399	0.03998776	-3.9392276	CDC27	assignmentsChromaffin_cells.Chrom_SOX2_statusHigh
assignmentsChromaffin_cells.Chrom_SOX2_statusHigh.HS3ST2	2.53875629	6.00456716	2.45090062	0.0166189	0.04082308	-3.940032	HS3ST2	assignmentsChromaffin_cells.Chrom_SOX2_statusHigh
assignmentsChromaffin_cells.Chrom_SOX2_statusHigh.CSAD	-0.4419479	5.6608492	-2.4156018	0.01817813	0.04398802	-3.9420203	CSAD	assignmentsChromaffin_cells.Chrom_SOX2_statusHigh
assignmentsChromaffin_cells.Chrom_SOX2_statusHigh.ILK	0.67416583	5.45303597	2.40478	0.01868399	0.04495649	-3.9426927	ILK	assignmentsChromaffin_cells.Chrom_SOX2_statusHigh
assignmentsChromaffin_cells.Chrom_SOX2_statusHigh.ZNF714	-0.7927989	4.42533128	-2.3792961	0.01992515	0.04746169	-3.94463	ZNF714	assignmentsChromaffin_cells.Chrom_SOX2_statusHigh
assignmentsChromaffin_cells.Chrom_SOX2_statusHigh.KCNB2	2.88259005	4.77294924	2.46384257	0.01607563	0.03968307	-3.9477597	KCNB2	assignmentsChromaffin_cells.Chrom_SOX2_statusHigh
assignmentsChromaffin_cells.Chrom_SOX2_statusHigh.YTHDF2	0.54063496	5.30106734	2.41013584	0.01843209	0.04446208	-3.9488538	YTHDF2	assignmentsChromaffin_cells.Chrom_SOX2_statusHigh
assignmentsChromaffin_cells.Chrom_SOX2_statusHigh.SCG2	2.3245024	7.66318965	2.51435793	0.01410437	0.03538891	-3.9489264	SCG2	assignmentsChromaffin_cells.Chrom_SOX2_statusHigh
assignmentsChromaffin_cells.Chrom_SOX2_statusHigh.CLPB	-0.429146	4.98347608	-2.4133982	0.01828014	0.04419296	-3.950319	CLPB	assignmentsChromaffin_cells.Chrom_SOX2_statusHigh
assignmentsChromaffin_cells.Chrom_SOX2_statusHigh.ABHD3	1.10845475	6.13156652	2.42939845	0.01755664	0.04270145	-3.9520154	ABHD3	assignmentsChromaffin_cells.Chrom_SOX2_statusHigh
assignmentsChromaffin_cells.Chrom_SOX2_statusHigh.ERGIC2	0.52070224	5.73744877	2.43858976	0.017144	0.04186288	-3.9522695	ERGIC2	assignmentsChromaffin_cells.Chrom_SOX2_statusHigh
assignmentsChromaffin_cells.Chrom_SOX2_statusHigh.VRK2	-0.4368625	6.48620557	-2.438276	0.01715775	0.04189009	-3.952918	VRK2	assignmentsChromaffin_cells.Chrom_SOX2_statusHigh
assignmentsChromaffin_cells.Chrom_SOX2_statusHigh.NUDT21	0.33571812	5.05188209	2.41440022	0.01823369	0.04409408	-3.9583652	NUDT21	assignmentsChromaffin_cells.Chrom_SOX2_statusHigh
assignmentsChromaffin_cells.Chrom_SOX2_statusHigh.NSG2	1.30242546	4.58542466	2.44391462	0.01691894	0.04143951	-3.9604197	NSG2	assignmentsChromaffin_cells.Chrom_SOX2_statusHigh
assignmentsChromaffin_cells.Chrom_SOX2_statusHigh.KIAA0930	1.14670851	4.00527091	2.37592438	0.02009812	0.04782398	-3.9612116	KIAA0930	assignmentsChromaffin_cells.Chrom_SOX2_statusHigh
assignmentsChromaffin_cells.Chrom_SOX2_statusHigh.SLC35B4	0.43331425	4.6772372	2.38956343	0.01941652	0.04643089	-3.9668745	SLC35B4	assignmentsChromaffin_cells.Chrom_SOX2_statusHigh
assignmentsChromaffin_cells.Chrom_SOX2_statusHigh.RAVER1	0.42526595	3.45927927	2.3611088	0.02085532	0.04931844	-3.9687579	RAVER1	assignmentsChromaffin_cells.Chrom_SOX2_statusHigh
assignmentsChromaffin_cells.Chrom_SOX2_statusHigh.PALLD	-0.9621636	6.78140436	-2.4358122	0.01726898	0.04212959	-3.9695652	PALLD	assignmentsChromaffin_cells.Chrom_SOX2_statusHigh
assignmentsChromaffin_cells.Chrom_SOX2_statusHigh.NCALD	-1.2544232	7.1299515	-2.4425659	0.01697742	0.04155998	-3.9698201	NCALD	assignmentsChromaffin_cells.Chrom_SOX2_statusHigh
assignmentsChromaffin_cells.Chrom_SOX2_statusHigh.SERPINB9	2.26244905	5.7354479	2.37874533	0.01996015	0.04753094	-3.9705694	SERPINB9	assignmentsChromaffin_cells.Chrom_SOX2_statusHigh
assignmentsChromaffin_cells.Chrom_SOX2_statusHigh.SLAIN1	-0.9144247	4.82325483	-2.4109696	0.01839504	0.04439725	-3.972907	SLAIN1	assignmentsChromaffin_cells.Chrom_SOX2_statusHigh
assignmentsChromaffin_cells.Chrom_SOX2_statusHigh.ANKRD10	-0.5621642	7.41532195	-2.4356246	0.01727436	0.0421363	-3.9743889	ANKRD10	assignmentsChromaffin_cells.Chrom_SOX2_statusHigh
assignmentsChromaffin_cells.Chrom_SOX2_statusHigh.ARID1A	-0.6919219	6.59249672	-2.4106441	0.01840834	0.04441597	-3.9744754	ARID1A	assignmentsChromaffin_cells.Chrom_SOX2_statusHigh
assignmentsChromaffin_cells.Chrom_SOX2_statusHigh.TRMU	-0.4417291	4.98608363	-2.3961762	0.01909509	0.04580422	-3.9761119	TRMU	assignmentsChromaffin_cells.Chrom_SOX2_statusHigh
assignmentsChromaffin_cells.Chrom_SOX2_statusHigh.RSF1	-0.382867	7.73902972	-2.4560756	0.01639298	0.04035466	-3.9792504	RSF1	assignmentsChromaffin_cells.Chrom_SOX2_statusHigh
assignmentsChromaffin_cells.Chrom_SOX2_statusHigh.GAA	0.68657156	4.31129977	2.38362029	0.01970951	0.04703185	-3.980598	GAA	assignmentsChromaffin_cells.Chrom_SOX2_statusHigh
assignmentsChromaffin_cells.Chrom_SOX2_statusHigh.FBXO16	-0.7338646	4.80663848	-2.4154726	0.0181841	0.04399406	-3.982705	FBXO16	assignmentsChromaffin_cells.Chrom_SOX2_statusHigh
assignmentsChromaffin_cells.Chrom_SOX2_statusHigh.ECE1	0.99413878	6.36491795	2.42481492	0.01776009	0.04311312	-3.9847145	ECE1	assignmentsChromaffin_cells.Chrom_SOX2_statusHigh
assignmentsChromaffin_cells.Chrom_SOX2_statusHigh.SAMM50	0.47989093	4.02967527	2.37026724	0.0203822	0.04842094	-3.9867862	SAMM50	assignmentsChromaffin_cells.Chrom_SOX2_statusHigh
assignmentsChromaffin_cells.Chrom_SOX2_statusHigh.SNHG5	1.10793256	6.08634427	2.42633729	0.01769543	0.04298036	-3.9869852	SNHG5	assignmentsChromaffin_cells.Chrom_SOX2_statusHigh
assignmentsChromaffin_cells.Chrom_SOX2_statusHigh.PSMD5	-0.6770408	5.22370683	-2.3833433	0.01972326	0.04705767	-3.9873446	PSMD5	assignmentsChromaffin_cells.Chrom_SOX2_statusHigh
assignmentsChromaffin_cells.Chrom_SOX2_statusHigh.FAM222A	0.81901221	4.20937461	2.398922	0.01896302	0.04552829	-3.9893891	FAM222A	assignmentsChromaffin_cells.Chrom_SOX2_statusHigh
assignmentsChromaffin_cells.Chrom_SOX2_statusHigh.PGM1	0.51256196	4.18606541	2.37045189	0.02037276	0.04840569	-3.990927	PGM1	assignmentsChromaffin_cells.Chrom_SOX2_statusHigh

assignmentsChromaffin_cells.Chrom_SOX2_statusHigh.WDR41	-0.6828049	5.81272314	-2.3972989	0.01904099	0.0457018	-3.9928076	WDR41	assignmentsChromaffin_cells.Chrom_SOX2_statusHigh
assignmentsChromaffin_cells.Chrom_SOX2_statusHigh.PPP1R15B	0.76767386	5.15050394	2.39228863	0.01928347	0.04615257	-3.9956847	PPP1R15B	assignmentsChromaffin_cells.Chrom_SOX2_statusHigh
assignmentsChromaffin_cells.Chrom_SOX2_statusHigh.CHD2	-0.5251091	8.19728235	-2.4456175	0.01683853	0.04126147	-4.0005949	CHD2	assignmentsChromaffin_cells.Chrom_SOX2_statusHigh
assignmentsChromaffin_cells.Chrom_SOX2_statusHigh.MARK2	-0.6018131	5.70865155	-2.3799874	0.01989054	0.04738628	-4.0012824	MARK2	assignmentsChromaffin_cells.Chrom_SOX2_statusHigh
assignmentsChromaffin_cells.Chrom_SOX2_statusHigh.NDUFV2	0.57612509	6.47974756	2.42812071	0.01760825	0.04279452	-4.0020317	NDUFV2	assignmentsChromaffin_cells.Chrom_SOX2_statusHigh
assignmentsChromaffin_cells.Chrom_SOX2_statusHigh.ATR	-0.6609548	6.71330025	-2.4126033	0.01831706	0.04423565	-4.0064457	ATR	assignmentsChromaffin_cells.Chrom_SOX2_statusHigh
assignmentsChromaffin_cells.Chrom_SOX2_statusHigh.HSD17B4	-0.3979808	6.28730109	-2.4133398	0.01828285	0.04419296	-4.0068542	HSD17B4	assignmentsChromaffin_cells.Chrom_SOX2_statusHigh
assignmentsChromaffin_cells.Chrom_SOX2_statusHigh.PWWP2A	-0.5306035	6.19539889	-2.4089373	0.01848819	0.04457511	-4.0077681	PWWP2A	assignmentsChromaffin_cells.Chrom_SOX2_statusHigh
assignmentsChromaffin_cells.Chrom_SOX2_statusHigh.MYEF2	-0.9877616	6.4522479	-2.4366367	0.01723416	0.04206383	-4.0081317	MYEF2	assignmentsChromaffin_cells.Chrom_SOX2_statusHigh
assignmentsChromaffin_cells.Chrom_SOX2_statusHigh.LIX1L	0.61026599	4.39671683	2.36056072	0.02088394	0.04937157	-4.0082948	LIX1L	assignmentsChromaffin_cells.Chrom_SOX2_statusHigh
assignmentsChromaffin_cells.Chrom_SOX2_statusHigh.ERGIC1	0.58619857	6.11155443	2.39913773	0.01895268	0.04551027	-4.0096893	ERGIC1	assignmentsChromaffin_cells.Chrom_SOX2_statusHigh
assignmentsChromaffin_cells.Chrom_SOX2_statusHigh.NFKBIA	1.55164823	6.46131018	2.40562062	0.01865141	0.04490786	-4.0109459	NFKBIA	assignmentsChromaffin_cells.Chrom_SOX2_statusHigh
assignmentsChromaffin_cells.Chrom_SOX2_statusHigh.DBN1	0.58849638	3.92644968	2.3642375	0.02069261	0.04899868	-4.0131529	DBN1	assignmentsChromaffin_cells.Chrom_SOX2_statusHigh
assignmentsChromaffin_cells.Chrom_SOX2_statusHigh.PRMT9	-1.0563502	6.69973582	-2.4149586	0.01821345	0.04405841	-4.0163726	PRMT9	assignmentsChromaffin_cells.Chrom_SOX2_statusHigh
assignmentsChromaffin_cells.Chrom_SOX2_statusHigh.EPB41	-0.801102	6.8931591	-2.4265282	0.01767989	0.04295562	-4.0171576	EPB41	assignmentsChromaffin_cells.Chrom_SOX2_statusHigh
assignmentsChromaffin_cells.Chrom_SOX2_statusHigh.LANCL1	-0.4726024	5.48191425	-2.394217	0.01918982	0.04599706	-4.0199982	LANCL1	assignmentsChromaffin_cells.Chrom_SOX2_statusHigh
assignmentsChromaffin_cells.Chrom_SOX2_statusHigh.SLC16A10	-1.3612833	6.13471727	-2.3668581	0.02056467	0.04873891	-4.020101	SLC16A10	assignmentsChromaffin_cells.Chrom_SOX2_statusHigh
assignmentsChromaffin_cells.Chrom_SOX2_statusHigh.PKN1	0.50927247	5.18640062	2.38216434	0.01978188	0.04715902	-4.0203551	PKN1	assignmentsChromaffin_cells.Chrom_SOX2_statusHigh
assignmentsChromaffin_cells.Chrom_SOX2_statusHigh.GLO1	0.54810335	5.43042751	2.40120032	0.01885406	0.04530059	-4.022083	GLO1	assignmentsChromaffin_cells.Chrom_SOX2_statusHigh
assignmentsChromaffin_cells.Chrom_SOX2_statusHigh.STXBP4	-0.7042407	6.46218893	-2.418291	0.01805434	0.04375273	-4.0238029	STXBP4	assignmentsChromaffin_cells.Chrom_SOX2_statusHigh
assignmentsChromaffin_cells.Chrom_SOX2_statusHigh.SGPP2	-1.053291	5.60416923	-2.4213377	0.01792082	0.04346856	-4.0240228	SGPP2	assignmentsChromaffin_cells.Chrom_SOX2_statusHigh
assignmentsChromaffin_cells.Chrom_SOX2_statusHigh.IGF1R	-0.8009289	7.65056343	-2.4359149	0.01726312	0.0421217	-4.0243181	IGF1R	assignmentsChromaffin_cells.Chrom_SOX2_statusHigh
assignmentsChromaffin_cells.Chrom_SOX2_statusHigh.SARM1	-0.5498373	4.79450729	-2.3816826	0.01980588	0.04720563	-4.0261592	SARM1	assignmentsChromaffin_cells.Chrom_SOX2_statusHigh
assignmentsChromaffin_cells.Chrom_SOX2_statusHigh.ADM	1.35142725	4.57935788	2.3943074	0.01919269	0.04599707	-4.0278841	ADM	assignmentsChromaffin_cells.Chrom_SOX2_statusHigh
assignmentsChromaffin_cells.Chrom_SOX2_statusHigh.MYO6	-0.5327017	6.76003717	-2.4155861	0.01817886	0.04398802	-4.0285408	MYO6	assignmentsChromaffin_cells.Chrom_SOX2_statusHigh
assignmentsChromaffin_cells.Chrom_SOX2_statusHigh.DPYD	-0.618701	9.01713663	-2.4510458	0.01660593	0.0408017	-4.0286456	DPYD	assignmentsChromaffin_cells.Chrom_SOX2_statusHigh
assignmentsChromaffin_cells.Chrom_SOX2_statusHigh.MALAT1	-0.8171752	15.4810788	-2.511809	0.01419557	0.03558685	-4.0286887	MALAT1	assignmentsChromaffin_cells.Chrom_SOX2_statusHigh
assignmentsChromaffin_cells.Chrom_SOX2_statusHigh.LDB1	-0.4631072	4.79113335	-2.3666254	0.02056918	0.04874239	-4.0325601	LDB1	assignmentsChromaffin_cells.Chrom_SOX2_statusHigh
assignmentsChromaffin_cells.Chrom_SOX2_statusHigh.PRRC2A	-0.4560813	5.55985009	-2.3812226	0.01982882	0.04724626	-4.0334505	PRRC2A	assignmentsChromaffin_cells.Chrom_SOX2_statusHigh
assignmentsChromaffin_cells.Chrom_SOX2_statusHigh.PLCD4	-1.0333753	4.46306197	-2.3757429	0.02010702	0.04783807	-4.0363602	PLCD4	assignmentsChromaffin_cells.Chrom_SOX2_statusHigh
assignmentsChromaffin_cells.Chrom_SOX2_statusHigh.ORC3	-0.4803595	5.88948846	-2.3960677	0.01910032	0.04580993	-4.0379126	ORC3	assignmentsChromaffin_cells.Chrom_SOX2_statusHigh
assignmentsChromaffin_cells.Chrom_SOX2_statusHigh.EFR3B	-0.8105499	5.11216467	-2.3897357	0.01940809	0.04642312	-4.0396339	EFR3B	assignmentsChromaffin_cells.Chrom_SOX2_statusHigh
assignmentsChromaffin_cells.Chrom_SOX2_statusHigh.DENND4A	-0.6399503	8.20322244	-2.434474	0.01732519	0.04222817	-4.0458686	DENND4A	assignmentsChromaffin_cells.Chrom_SOX2_statusHigh
assignmentsChromaffin_cells.Chrom_SOX2_statusHigh.PPIP5K1	-0.6352803	4.97368332	-2.3791888	0.01993053	0.04746746	-4.0488087	PPIP5K1	assignmentsChromaffin_cells.Chrom_SOX2_statusHigh
assignmentsChromaffin_cells.Chrom_SOX2_statusHigh.CPNE8	1.56864772	6.77720937	2.4057108	0.01864715	0.04490435	-4.0494122	CPNE8	assignmentsChromaffin_cells.Chrom_SOX2_statusHigh
assignmentsChromaffin_cells.Chrom_SOX2_statusHigh.CKAP2	0.64875445	4.63970913	2.37011885	0.02038979	0.04842517	-4.0498794	CKAP2	assignmentsChromaffin_cells.Chrom_SOX2_statusHigh
assignmentsChromaffin_cells.Chrom_SOX2_statusHigh.BBS4	-0.4813506	4.90989299	-2.3554741	0.02115124	0.04992253	-4.0543995	BBS4	assignmentsChromaffin_cells.Chrom_SOX2_statusHigh
assignmentsChromaffin_cells.Chrom_SOX2_statusHigh.IRF2	-0.5439043	6.04864631	-2.3607508	0.02087402	0.04935537	-4.0556982	IRF2	assignmentsChromaffin_cells.Chrom_SOX2_statusHigh
assignmentsChromaffin_cells.Chrom_SOX2_statusHigh.ARPC1A	0.52316863	5.56402667	2.38460496	0.01966069	0.0469503	-4.0558658	ARPC1A	assignmentsChromaffin_cells.Chrom_SOX2_statusHigh
assignmentsChromaffin_cells.Chrom_SOX2_statusHigh.SOD2	0.93652866	6.34294742	2.38647108	0.01957121	0.04676447	-4.0562869	SOD2	assignmentsChromaffin_cells.Chrom_SOX2_statusHigh
assignmentsChromaffin_cells.Chrom_SOX2_statusHigh.SCARB1	-1.0301819	5.73213139	-2.3844256	0.01967364	0.04696305	-4.058195	SCARB1	assignmentsChromaffin_cells.Chrom_SOX2_statusHigh
assignmentsChromaffin_cells.Chrom_SOX2_statusHigh.ANKRD36B	-1.0197164	6.74766888	-2.4053926	0.01866038	0.04492273	-4.0582738	ANKRD36B	assignmentsChromaffin_cells.Chrom_SOX2_statusHigh
assignmentsChromaffin_cells.Chrom_SOX2_statusHigh.POR	0.76944876	5.7440255	2.38738415	0.0195235	0.04665741	-4.0616158	POR	assignmentsChromaffin_cells.Chrom_SOX2_statusHigh
assignmentsChromaffin_cells.Chrom_SOX2_statusHigh.SLC4A10	-2.2445293	4.8552577	-2.3566743	0.02109546	0.04979821	-4.0667411	SLC4A10	assignmentsChromaffin_cells.Chrom_SOX2_statusHigh
assignmentsChromaffin_cells.Chrom_SOX2_statusHigh.TARDBP	0.40223524	5.49269496	2.3619516	0.02081138	0.04922904	-4.0696032	TARDBP	assignmentsChromaffin_cells.Chrom_SOX2_statusHigh
assignmentsChromaffin_cells.Chrom_SOX2_statusHigh.UBE2W	-0.4669643	6.94272841	-2.3880712	0.01948972	0.04659751	-4.0715105	UBE2W	assignmentsChromaffin_cells.Chrom_SOX2_statusHigh
assignmentsChromaffin_cells.Chrom_SOX2_statusHigh.UBE2E1	-0.4929066	7.12063063	-2.3878799	0.01949912	0.04660609	-4.0746503	UBE2E1	assignmentsChromaffin_cells.Chrom_SOX2_statusHigh
assignmentsChromaffin_cells.Chrom_SOX2_statusHigh.PPA2	-0.4289731	7.16375975	-2.4035655	0.01874153	0.04508425	-4.0776691	PPA2	assignmentsChromaffin_cells.Chrom_SOX2_statusHigh
assignmentsChromaffin_cells.Chrom_SOX2_statusHigh.SACM1L	-0.4529228	6.18745044	-2.3775686	0.02001189	0.04763293	-4.0794669	SACM1L	assignmentsChromaffin_cells.Chrom_SOX2_statusHigh
assignmentsChromaffin_cells.Chrom_SOX2_statusHigh.SEC22B	0.48421003	5.50654594	2.36396349	0.02070681	0.04902508	-4.0835464	SEC22B	assignmentsChromaffin_cells.Chrom_SOX2_statusHigh
assignmentsChromaffin_cells.Chrom_SOX2_statusHigh.GFM1	0.49243313	5.42906294	2.36235528	0.02079036	0.04919384	-4.0841815	GFM1	assignmentsChromaffin_cells.Chrom_SOX2_statusHigh
assignmentsChromaffin_cells.Chrom_SOX2_statusHigh.STRADB	-0.6863514	5.18974542	-2.3682177	0.02048724	0.04862007	-4.0855587	STRADB	assignmentsChromaffin_cells.Chrom_SOX2_statusHigh
assignmentsChromaffin_cells.Chrom_SOX2_statusHigh.KLHDC10	-0.5048602	6.80936085	-2.3991423	0.01895246	0.04551027	-4.0859675	KLHDC10	assignmentsChromaffin_cells.Chrom_SOX2_statusHigh
assignmentsChromaffin_cells.Chrom_SOX2_statusHigh.ZSCAN16-A[illegible]	0.57419202	4.80179587	2.35754664	0.02104197	0.04969642	-4.0872396	ZSCAN16-AS	assignmentsChromaffin_cells.Chrom_SOX2_statusHigh
assignmentsChromaffin_cells.Chrom_SOX2_statusHigh.USP10	-0.3704854	6.31727939	-2.3671349	0.02054293	0.04871619	-4.0923633	USP10	assignmentsChromaffin_cells.Chrom_SOX2_statusHigh
assignmentsChromaffin_cells.Chrom_SOX2_statusHigh.DNAJC6	-0.8249794	5.18607768	-2.3685028	0.02047262	0.04859256	-4.0957494	DNAJC6	assignmentsChromaffin_cells.Chrom_SOX2_statusHigh
assignmentsChromaffin_cells.Chrom_SOX2_statusHigh.MT-ND4	2.53080338	8.67915912	2.43465079	0.01732429	0.04222817	-4.0963399	MT-ND4	assignmentsChromaffin_cells.Chrom_SOX2_statusHigh
assignmentsChromaffin_cells.Chrom_SOX2_statusHigh.USP9X	-0.4351827	7.76959364	-2.3925998	0.01926833	0.04613009	-4.1038347	USP9X	assignmentsChromaffin_cells.Chrom_SOX2_statusHigh
assignmentsChromaffin_cells.Chrom_SOX2_statusHigh.NAP1L4	-0.4629952	6.72601865	-2.3643196	0.02068835	0.04899584	-4.1084255	NAP1L4	assignmentsChromaffin_cells.Chrom_SOX2_statusHigh
assignmentsChromaffin_cells.Chrom_SOX2_statusHigh.PKP4	-0.7797855	7.76953872	-2.3937058	0.01921587	0.04603197	-4.1226697	PKP4	assignmentsChromaffin_cells.Chrom_SOX2_statusHigh
assignmentsChromaffin_cells.Chrom_SOX2_statusHigh.SPIDR	-0.4272101	8.41615871	-2.3924416	0.01927603	0.04614164	-4.1227153	SPIDR	assignmentsChromaffin_cells.Chrom_SOX2_statusHigh
assignmentsChromaffin_cells.Chrom_SOX2_statusHigh.ARHGEF28	-1.3847027	5.99965827	-2.3628324	0.02077306	0.04916015	-4.1270461	ARHGEF28	assignmentsChromaffin_cells.Chrom_SOX2_statusHigh
assignmentsChromaffin_cells.Chrom_SOX2_statusHigh.DST	-0.6329118	8.97367185	-2.4161506	0.01815281	0.04394488	-4.1310167	DST	assignmentsChromaffin_cells.Chrom_SOX2_statusHigh
assignmentsChromaffin_cells.Chrom_SOX2_statusHigh.MACF1	-0.7243382	8.72716588	-2.3741904	0.02018248	0.04800334	-4.1337072	MACF1	assignmentsChromaffin_cells.Chrom_SOX2_statusHigh

assignmentsChromaffin_cells.Chrom_SOX2_statusHigh.NCOA2	-0.5902823	8.15440369	-2.3860246	0.0195905	0.04680359	-4.1433444	NCOA2	assignmentsChromaffin_cells.Chrom_SOX2_statusHigh
assignmentsChromaffin_cells.Chrom_SOX2_statusHigh.SYN2	-1.2038047	6.07250273	-2.373209	0.0202397	0.04813232	-4.1443768	SYN2	assignmentsChromaffin_cells.Chrom_SOX2_statusHigh
assignmentsChromaffin_cells.Chrom_SOX2_statusHigh.RUFY3	-0.3946564	7.82961925	-2.3931305	0.01924254	0.04607523	-4.150713	RUFY3	assignmentsChromaffin_cells.Chrom_SOX2_statusHigh
assignmentsChromaffin_cells.Chrom_SOX2_statusHigh.GEM	2.16652197	6.39864875	2.3729959	0.02025053	0.04814379	-4.1553081	GEM	assignmentsChromaffin_cells.Chrom_SOX2_statusHigh
assignmentsChromaffin_cells.Chrom_SOX2_statusHigh.PRRC2B	-0.5093453	7.27703596	-2.3585424	0.02098964	0.04960683	-4.1572989	PRRC2B	assignmentsChromaffin_cells.Chrom_SOX2_statusHigh
assignmentsChromaffin_cells.Chrom_SOX2_statusHigh.HNRNPDL	0.56263812	8.11893708	2.38431984	0.01967482	0.04696305	-4.1643875	HNRNPDL	assignmentsChromaffin_cells.Chrom_SOX2_statusHigh
assignmentsChromaffin_cells.Chrom_SOX2_statusHigh.TCEA1	0.6328421	6.84488183	2.36687643	0.02055625	0.04872614	-4.1647961	TCEA1	assignmentsChromaffin_cells.Chrom_SOX2_statusHigh
assignmentsChromaffin_cells.Chrom_SOX2_statusHigh.LARP4	-0.4790535	7.02638418	-2.3576116	0.02103855	0.04969642	-4.1735494	LARP4	assignmentsChromaffin_cells.Chrom_SOX2_statusHigh
assignmentsChromaffin_cells.Chrom_SOX2_statusHigh.GSK3B	-0.5181618	7.97516946	-2.3663276	0.02058454	0.04877159	-4.1741573	GSK3B	assignmentsChromaffin_cells.Chrom_SOX2_statusHigh
assignmentsChromaffin_cells.Chrom_SOX2_statusHigh.SNAP91	-1.316572	6.26781084	-2.3637291	0.02072648	0.04906439	-4.186968	SNAP91	assignmentsChromaffin_cells.Chrom_SOX2_statusHigh
assignmentsChromaffin_cells.Chrom_SOX2_statusHigh.TRIO	-0.6355796	8.84683881	-2.3701144	0.02039002	0.04842517	-4.2109604	TRIO	assignmentsChromaffin_cells.Chrom_SOX2_statusHigh
assignmentsChromaffin_cells.Chrom_SOX2_statusHigh.RBFOX2	-0.5613179	7.9735491	-2.3549112	0.02118101	0.04998543	-4.2294081	RBFOX2	assignmentsChromaffin_cells.Chrom_SOX2_statusHigh
assignmentsChromaffin_cells.Chrom_SOX2_statusHigh.SNHG14	-0.8221704	8.81073002	-2.3697699	0.02041077	0.04845292	-4.2554674	SNHG14	assignmentsChromaffin_cells.Chrom_SOX2_statusHigh
